# Supplementary material for: Universal fragment descriptors for predicting properties of inorganic crystals
Source: Nat Commun. 2017 Jun 5;8:15679. doi: 10.1038/ncomms15679 (PMC5465371; doi:10.1038/ncomms15679)
Supplement: Supplementary Information — Supplementary Notes, Supplementary Tables and Supplementary References [file ncomms15679-s1.pdf]

CONTENTS

Supplementary Note 1. Feature importance 2

Supplementary Note 2. Five-fold cross validation predictions 3
- Metal/insulator classification 3
- Band gap energy 302
- Bulk modulus 441
- Shear modulus 470
- Debye temperature 499
- Heat capacity at constant pressure 528
- Heat capacity at constant volume 557
- Thermal expansion coefficient 586

Supplementary Note 3. Model validation predictions 615
- Bulk modulus 615
- Shear modulus 623
- Debye temperature 631
- Heat capacity at constant pressure 639
- Heat capacity at constant volume 647
- Thermal expansion coefficient 655

Supplementary Note 4. Comparison with experiments 663
- Bulk modulus 663
- Shear modulus 664
- Debye temperature 665

Supplementary References 666

SUPPLEMENTARY NOTE 1. FEATURE IMPORTANCE

variable	importance	mean
avg $\left(\Delta H_{\mathrm{fusion}}\lambda^{-1}\right)$	0.1161	10.42
avg $\left(V_{\mathrm{molar}}r_{\mathrm{cov}}^{-1}\right)$	0.1120	23.34
max $\left(\Delta H_{\mathrm{fusion}}\lambda^{-1}\right)$	0.0834	13.83
min $\left(\Delta H_{\mathrm{vapor}}V_{\mathrm{molar}}^{-1}\right)$	0.0578	7.64
avg $\left(\alpha_{\mathrm{P}}\Delta H_{\mathrm{vapor}}^{-1}\right)$	0.0199	0.16
avg (IP_2V_{molar})	0.0192	349.66
avg $(\Delta H_{\mathrm{atom}}r_{\mathrm{cov}})$	0.0183	411.19
$\sum\left(IP_2\Delta H_{\mathrm{atom}}^{-1}\right)$	0.0121	1.70
min $(C\lambda)$	0.0118	4.27
max $(\Delta EA_{\mathrm{bond}})$	0.0108	0.30

Supplementary Table 1. Most significant features of the metal/insulator classification model. The mean value of each feature across the training set is also provided.

variable	importance	mean
avg $\left(\chi Z_{\mathrm{eff}}^{-1}\right)$	0.0752	1.41
avg $\left(C\lambda^{-1}\right)$	0.0713	17.45
min (CV_{molar})	0.0452	4.07
avg (CZ_{eff})	0.0244	3.37
min $\left(\chi Z_{\mathrm{eff}}^{-1}\right)$	0.0220	0.66
avg (IP_3C)	0.0218	38.87
$\sum(\Delta H_{\mathrm{fusion}}-C)$	0.0156	2.41
avg $(Z_{\mathrm{eff}}r_{\mathrm{cov}})$	0.0147	5.32
max $\left(EA\alpha_{\mathrm{P}}^{-1}\right)$	0.0104	1.88
min (χC)	0.0103	1.11
min $\left(\Delta\left(V_{\mathrm{molar}}\right)_{\mathrm{bond}}\right)$	0.0073	1.96
avg $(\Delta IP_{\mathrm{bond}})$	0.0073	3.08
std $(\Delta IP_{\mathrm{bond}})$	0.0067	1.38
ρ	0.0048	6.03

Supplementary Table 2. Most significant features of the band gap energy regression model. The mean value of each feature across the training set is also provided.

variable	importance	mean
volume per atom	0.2155	19.87
med $(IP_2\Delta H_{\mathrm{atom}})$	0.0978	5640.00
avg $\left(V_{\mathrm{molar}}IP^{-1}\right)$	0.0522	1.71
min $(IP_3\Delta H_{\mathrm{atom}})$	0.0498	7347.37
min $(IP\Delta H_{\mathrm{atom}})$	0.0397	1907.13

Supplementary Table 3. Most significant features of the bulk modulus regression model. The mean value of each feature across the training set is also provided.

variable	importance	mean
volume per atom	0.2240	19.87
med $(IP_2\Delta H_{\mathrm{atom}})$	0.0451	5640.00
avg $(r_{\mathrm{cov}}V_{\mathrm{molar}})$	0.0436	17.92
avg $(V_{\mathrm{molar}}IP_3)$	0.0424	499.19
med $(r_{\mathrm{cov}}V_{\mathrm{molar}})$	0.0335	17.92

Supplementary Table 4. Most significant features of the shear modulus regression model. The mean value of each feature across the training set is also provided.

variable	importance	mean
avg $\left(V_{\mathrm{molar}}C^{-1}\right)$	0.1512	53.99
volume per atom	0.1439	19.87
avg $(m_{\mathrm{atom}}C)$	0.0812	23.22
avg $\left(m_{\mathrm{atom}}\Delta H_{\mathrm{atom}}^{-1}\right)$	0.0589	0.38
avg $\left(\Delta\left(p_{\mathrm{P}}\right)_{\mathrm{bond}}\right)$	0.0509	4.18
avg $(p_{\mathrm{P}}Z_{\mathrm{eff}})$	0.0455	28.66
c	0.0264	5.55
std $\left(\Delta H_{\mathrm{vapor}}\Delta H_{\mathrm{atom}}^{-1}\right)$	0.0198	16.20
avg $(\Delta EA_{\mathrm{bond}})$	0.0193	2.63
b	0.0179	5.32

Supplementary Table 5. Most significant features of the Debye temperature regression model. The mean value of each feature across the training set is also provided.

variable	importance	mean
volume per atom	0.1653	19.87
avg $(m_{\mathrm{atom}}C)$	0.1092	23.22
avg $\left(m_{\mathrm{atom}}\Delta H_{\mathrm{atom}}^{-1}\right)$	0.0829	0.38
avg $\left(V_{\mathrm{molar}}C^{-1}\right)$	0.0614	53.96
avg $\left(p_{\mathrm{P}}N_{\mathrm{V}}^{-1}\right)$	0.0317	1.27

Supplementary Table 6. Most significant features of the heat capacity (at constant pressure) regression model. The mean value of each feature across the training set is also provided.

variable	importance	mean
volume per atom	0.1330	19.87
avg $(m_{\mathrm{atom}}C)$	0.1098	23.22
avg $\left(m_{\mathrm{atom}}\Delta H_{\mathrm{atom}}^{-1}\right)$	0.0960	0.38
avg $\left(V_{\mathrm{molar}}C^{-1}\right)$	0.0950	53.96
avg $\left(\Delta\left(p_{\mathrm{P}}\right)_{\mathrm{bond}}\right)$	0.0642	4.18
avg $(p_{\mathrm{P}}Z_{\mathrm{eff}})$	0.0573	28.68
avg $\left(m_{\mathrm{atom}}IP_3^{-1}\right)$	0.0334	3.23

Supplementary Table 7. Most significant features of the heat capacity (at constant volume) regression model. The mean value of each feature across the training set is also provided.

variable	importance	mean
med $(IP_2\Delta H_{\mathrm{atom}})$	0.1153	5643.45
max $(IP\Delta H_{\mathrm{atom}})$	0.0993	3753.71
min $(g_{\mathrm{P}}\Delta H_{\mathrm{atom}})$	0.0849	1962.71
min $(\Delta H_{\mathrm{fusion}}-\Delta H_{\mathrm{atom}})$	0.0524	-440.01
avg $(IP\Delta H_{\mathrm{atom}})$	0.0411	2574.49

Supplementary Table 8. Most significant features of the thermal expansion coefficient regression model. The mean value of each feature across the training set is also provided.

SUPPLEMENTARY NOTE 2. FIVE-FOLD CROSS VALIDATION PREDICTIONS

Metal/insulator classification

system	calculated	predicted
Ac1Br3_ICSD_31578	insulator	insulator
Ac1Cl3_ICSD_31569	insulator	insulator
Ac1H2_ICSD_56392	metal	metal
Ac1_ICSD_43491	metal	metal
Ac2O3_ICSD_31750	insulator	insulator
Ag10C2F8_ICSD_407646	insulator	insulator
Ag13Al36Mg32_ICSD_55636	metal	metal
Ag13O6Os1_ICSD_413193	metal	metal
Ag15Cl3P4S16_ICSD_416586	insulator	insulator
Ag16Ca6N1_ICSD_78395	metal	metal
Ag17Mg54_ICSD_58327	metal	metal
Ag18O21Si6_ICSD_33907	insulator	insulator
Ag1Al1Li2_ICSD_57330	metal	metal
Ag1Al1O2_ICSD_95662	insulator	insulator
Ag1Al1O2_ICSD_99688	insulator	insulator
Ag1Al1S2_ICSD_25356	insulator	insulator
Ag1Al1S2_ICSD_28744	insulator	insulator
Ag1Al1Se2_ICSD_28745	insulator	insulator
Ag1Al1Te2_ICSD_28746	insulator	insulator
Ag1Al2Pr1_ICSD_604688	metal	metal
Ag1As1Ba1_ICSD_8278	metal	metal
Ag1As1C4F6N4S8_ICSD_61131	insulator	insulator
Ag1As1Ca1_ICSD_10017	metal	metal
Ag1As1Eu1_ICSD_24365	metal	metal
Ag1As1F6Se3_ICSD_418700	insulator	insulator
Ag1As1F7_ICSD_62510	insulator	insulator
Ag1As1Hg1S3_ICSD_31194	insulator	insulator
Ag1As1Hg2O4_ICSD_413087	insulator	insulator
Ag1As1K2_ICSD_1154	insulator	insulator
Ag1As1Mg1_ICSD_655132	metal	metal
Ag1As1Na2_ICSD_49007	insulator	insulator
Ag1As1O1Th1_ICSD_183138	metal	metal
Ag1As1Pb1S3_ICSD_26835	insulator	insulator
Ag1As1S1_ICSD_604740	metal	insulator
Ag1As1S2_ICSD_18101	insulator	insulator
Ag1As1Se1_ICSD_604753	metal	metal
Ag1As1Se2_ICSD_61708	insulator	insulator
Ag1As1Sr1_ICSD_49742	metal	insulator
Ag1As1Zn1_ICSD_604767	metal	metal
Ag1As2Ce1_ICSD_93150	metal	metal
Ag1As2Nd1_ICSD_174360	metal	metal
Ag1As2Pr1_ICSD_412816	metal	metal
Ag1As2Sm1_ICSD_174361	metal	metal
Ag1Au1Ba4O6_ICSD_72329	insulator	insulator
Ag1Au1Cd2_ICSD_57337	metal	metal
Ag1Au1Cl4_ICSD_1152	insulator	insulator
Ag1Au1Cl6Cs2_ICSD_24516	metal	insulator
Ag1Au1Cl6Cs2_ICSD_26162	insulator	insulator
Ag1Au1F4_ICSD_90071	insulator	insulator
Ag1Au1Te2_ICSD_55250	metal	metal

Supplementary Table 9. Five-fold cross validated predictions for the metal/insulator classification (1/598).

system	calculated	predicted
Ag1Au1Te4_ICSD_30874	metal	metal
Ag1Au1Zn2_ICSD_57340	metal	metal
Ag1Au2F8_ICSD_85416	insulator	insulator
Ag1Au3I8Rb2_ICSD_32031	insulator	insulator
Ag1B1C4N4_ICSD_411179	insulator	insulator
Ag1B1F4_ICSD_415320	insulator	insulator
Ag1B1F5_ICSD_80645	insulator	insulator
Ag1B2_ICSD_604797	metal	metal
Ag1B7O12Sr1_ICSD_406544	insulator	insulator
Ag1Ba1Bi1_ICSD_56978	metal	metal
Ag1Ba1Er1S3_ICSD_75074	insulator	insulator
Ag1Ba1Er1Se3_ICSD_659171	insulator	insulator
Ag1Ba1F1S1_ICSD_183713	insulator	insulator
Ag1Ba1F1Se1_ICSD_183714	insulator	insulator
Ag1Ba1F1Te1_ICSD_419382	insulator	insulator
Ag1Ba1La1Se3_ICSD_659172	insulator	insulator
Ag1Ba1Nd1S3_ICSD_659170	insulator	insulator
Ag1Ba1O9P3_ICSD_50672	insulator	insulator
Ag1Ba1P1_ICSD_41706	insulator	metal
Ag1Ba1Sb1_ICSD_56981	metal	metal
Ag1Ba1Se3Y1_ICSD_104237	insulator	insulator
Ag1Ba1Te3Y1_ICSD_88717	insulator	insulator
Ag1Ba1_ICSD_57342	metal	metal
Ag1Be12_ICSD_109313	metal	metal
Ag1Be2_ICSD_604819	metal	metal
Ag1Bi1Ca1_ICSD_416283	metal	metal
Ag1Bi1Ca1_ICSD_659377	metal	metal
Ag1Bi1Cl2S1_ICSD_413290	insulator	insulator
Ag1Bi1Cr2O8_ICSD_8224	insulator	insulator
Ag1Bi1Cr4O14_ICSD_14234	insulator	insulator
Ag1Bi1Eu1_ICSD_57348	metal	metal
Ag1Bi1K2_ICSD_1156	insulator	insulator
Ag1Bi1Li2_ICSD_57350	metal	metal
Ag1Bi1O3_ICSD_89432	metal	insulator
Ag1Bi1P2S6_ICSD_170639	insulator	insulator
Ag1Bi1S2_ICSD_44340	insulator	insulator
Ag1Bi1Se2_ICSD_26518	insulator	insulator
Ag1Bi1Se2_ICSD_26519	insulator	insulator
Ag1Bi1Se2_ICSD_604856	insulator	insulator
Ag1Bi1Sr1_ICSD_57019	metal	metal
Ag1Bi1Te2_ICSD_159345	metal	insulator
Ag1Bi1Te2_ICSD_43266	insulator	metal
Ag1Bi1Te2_ICSD_604866	insulator	metal
Ag1Bi1Yb1_ICSD_604874	metal	metal
Ag1Bi2Cl1S3_ICSD_412372	insulator	insulator
Ag1Bi2Cl1Se3_ICSD_412371	insulator	insulator
Ag1Bi3S5_ICSD_171229	insulator	metal
Ag1Bi6S9_ICSD_69456	metal	insulator
Ag1Br13Mo6_ICSD_412903	insulator	insulator
Ag1Br14W6_ICSD_410958	insulator	insulator

Supplementary Table 10. Five-fold cross validated predictions for the metal/insulator classification (2/598).

system	calculated	predicted
Ag1Br1H3N1_ICSD_169137	insulator	insulator
Ag1Br1Hg1S1_ICSD_411773	insulator	insulator
Ag1Br1O1Pb1_ICSD_33913	insulator	insulator
Ag1Br1_ICSD_157536	insulator	insulator
Ag1Br1_ICSD_56550	insulator	insulator
Ag1Br3Cs2_ICSD_150288	insulator	insulator
Ag1Br3Rb2_ICSD_150287	insulator	insulator
Ag1Br5Pb2_ICSD_300100	insulator	insulator
Ag1C1H3N2_ICSD_169135	insulator	insulator
Ag1C1N1O1_ICSD_18149	insulator	insulator
Ag1C1N1O1_ICSD_23832	insulator	insulator
Ag1C1N1O1_ICSD_23833	insulator	insulator
Ag1C1N1O1_ICSD_260378	insulator	insulator
Ag1C1N1O1_ICSD_27678	metal	insulator
Ag1C1N1_ICSD_85783	insulator	insulator
Ag1C1N3O2_ICSD_408288	insulator	insulator
Ag1C1_ICSD_181772	metal	metal
Ag1C1_ICSD_183175	metal	metal
Ag1C2Cl2F6N2Sb1_ICSD_64638	insulator	insulator
Ag1C2Cs1_ICSD_410873	insulator	insulator
Ag1C2F6H2N2Sb1_ICSD_63287	insulator	insulator
Ag1C2H2N3O1_ICSD_63100	insulator	insulator
Ag1C2K1N2S2_ICSD_280587	insulator	insulator
Ag1C2K1N2_ICSD_30275	insulator	insulator
Ag1C2K1_ICSD_410874	insulator	insulator
Ag1C2Li1_ICSD_410868	insulator	insulator
Ag1C2N2Na1_ICSD_65697	insulator	insulator
Ag1C2N3_ICSD_68453	insulator	insulator
Ag1C2N3_ICSD_843	insulator	insulator
Ag1C4H12N1Se5_ICSD_159458	insulator	insulator
Ag1C4N3_ICSD_43823	insulator	insulator
Ag1Ca1Ge1_ICSD_421236	metal	metal
Ag1Ca1P1_ICSD_10016	metal	insulator
Ag1Ca1Sb1_ICSD_56982	insulator	metal
Ag1Ca1_ICSD_57352	metal	metal
Ag1Cd1O4V1_ICSD_401350	insulator	insulator
Ag1Cd1Sb1_ICSD_52549	metal	metal
Ag1Cd1_ICSD_604906	metal	metal
Ag1Cd2Ce1_ICSD_57363	metal	metal
Ag1Cd2Ga1S4_ICSD_90459	insulator	insulator
Ag1Cd3F20Hf3_ICSD_78893	metal	insulator
Ag1Cd3F20Zr3_ICSD_78892	metal	insulator
Ag1Ce1Cu5_ICSD_657086	metal	metal
Ag1Ce1Ge1_ICSD_56983	metal	metal
Ag1Ce1Mg1_ICSD_657933	metal	metal
Ag1Ce1Mg2_ICSD_57373	metal	metal
Ag1Ce1Pb1_ICSD_107100	metal	metal
Ag1Ce1Sb2_ICSD_79425	metal	metal
Ag1Ce1Si2_ICSD_247421	metal	metal
Ag1Ce1Sn1_ICSD_55819	metal	metal

Supplementary Table 11. Five-fold cross validated predictions for the metal/insulator classification (3/598).

system	calculated	predicted
Ag1Ce1_ICSD_57368	metal	metal
Ag1Ce1_ICSD_604941	metal	metal
Ag1Ce3Ge1S7_ICSD_604947	insulator	insulator
Ag1Ce3S7Si1_ICSD_604959	insulator	insulator
Ag1Ce3Se7Si1_ICSD_604962	insulator	insulator
Ag1Cl1O2_ICSD_15407	insulator	insulator
Ag1Cl1O2_ICSD_16717	insulator	insulator
Ag1Cl1O3_ICSD_30227	insulator	insulator
Ag1Cl1O4Pb4_ICSD_68309	insulator	insulator
Ag1Cl1O4_ICSD_185363	insulator	insulator
Ag1Cl1O4_ICSD_185366	insulator	insulator
Ag1Cl1O4_ICSD_185367	insulator	insulator
Ag1Cl1O4_ICSD_33568	insulator	insulator
Ag1Cl1_ICSD_56539	insulator	insulator
Ag1Cl1_ICSD_56543	insulator	insulator
Ag1Cl1_ICSD_56545	insulator	insulator
Ag1Cl2Cs1_ICSD_150300	insulator	insulator
Ag1Cl3Cs2_ICSD_150286	insulator	insulator
Ag1Cl3Rb2_ICSD_280031	insulator	insulator
Ag1Co1O2_ICSD_180888	insulator	insulator
Ag1Co1O2_ICSD_180889	metal	insulator
Ag1Co1O2_ICSD_187261	insulator	insulator
Ag1Co1O2_ICSD_261608	insulator	insulator
Ag1Co1O4P1_ICSD_100520	insulator	insulator
Ag1Co3H2O12P3_ICSD_280774	insulator	insulator
Ag1Cr1O2_ICSD_4149	insulator	insulator
Ag1Cr1S2_ICSD_25628	metal	insulator
Ag1Cr1S2_ICSD_42395	insulator	metal
Ag1Cr1Se2_ICSD_25626	metal	metal
Ag1Cr1Se2_ICSD_68423	insulator	metal
Ag1Cr1Te2_ICSD_605002	metal	metal
Ag1Cr2H4O10Sc1_ICSD_156676	insulator	insulator
Ag1Cr2Te4_ICSD_71695	metal	metal
Ag1Cr3O8_ICSD_155507	insulator	insulator
Ag1Cs1F3_ICSD_23154	insulator	insulator
Ag1Cs1N2O6_ICSD_280069	insulator	insulator
Ag1Cs1O1_ICSD_25745	insulator	insulator
Ag1Cs1O1_ICSD_40160	insulator	insulator
Ag1Cs1O1_ICSD_49754	insulator	insulator
Ag1Cs1Se4_ICSD_87464	insulator	insulator
Ag1Cs2F4_ICSD_16254	insulator	insulator
Ag1Cs2F6K1_ICSD_16783	insulator	insulator
Ag1Cs2I3_ICSD_150291	insulator	insulator
Ag1Cs2S4V1_ICSD_50460	insulator	insulator
Ag1Cs5O9Si3_ICSD_51508	insulator	insulator
Ag1Cu1O2_ICSD_95089	insulator	metal
Ag1Cu1O4P1_ICSD_165596	insulator	insulator
Ag1Cu1O4P1_ICSD_35590	insulator	insulator
Ag1Cu1O4V1_ICSD_419201	insulator	insulator
Ag1Cu1S1_ICSD_30233	insulator	insulator

Supplementary Table 12. Five-fold cross validated predictions for the metal/insulator classification (4/598).

system	calculated	predicted
Ag1Cu1S1_ICSD_66580	insulator	insulator
Ag1Cu1S1_ICSD_66582	insulator	insulator
Ag1Cu1Te2_ICSD_42482	metal	metal
Ag1Cu4Dy1_ICSD_605006	metal	metal
Ag1Cu4Er1_ICSD_605007	metal	metal
Ag1Cu4Gd1_ICSD_605020	metal	metal
Ag1Cu4Ho1_ICSD_605023	metal	metal
Ag1Cu4Nd1_ICSD_605036	metal	metal
Ag1Cu4Tb1_ICSD_605048	metal	metal
Ag1Cu4Tm1_ICSD_605053	metal	metal
Ag1Cu4U1_ICSD_603921	metal	metal
Ag1Cu4Yb1_ICSD_605057	metal	metal
Ag1Dy1Ge1_ICSD_605077	metal	metal
Ag1Dy1Ge1_ICSD_86731	metal	metal
Ag1Dy1Mg1_ICSD_657937	metal	metal
Ag1Dy1Pb1_ICSD_107106	metal	metal
Ag1Dy1Sb2_ICSD_95133	metal	metal
Ag1Dy1Se2_ICSD_605083	insulator	insulator
Ag1Dy1Se2_ICSD_605084	insulator	insulator
Ag1Dy1Si1_ICSD_605085	metal	metal
Ag1Dy1Sn1_ICSD_55823	metal	metal
Ag1Dy1Te2_ICSD_154794	insulator	insulator
Ag1Dy1_ICSD_150538	metal	metal
Ag1Er1Ge1_ICSD_81558	metal	metal
Ag1Er1Ge1_ICSD_86733	metal	metal
Ag1Er1Mg1_ICSD_657938	metal	metal
Ag1Er1O8W2_ICSD_27743	insulator	insulator
Ag1Er1P2Se6_ICSD_420303	insulator	insulator
Ag1Er1Pb1_ICSD_107276	metal	metal
Ag1Er1S2_ICSD_423921	insulator	insulator
Ag1Er1Se2_ICSD_605115	insulator	insulator
Ag1Er1Si1_ICSD_605116	metal	metal
Ag1Er1Sn1_ICSD_417551	metal	metal
Ag1Er1Sn1_ICSD_55825	metal	metal
Ag1Er1Te2_ICSD_154791	insulator	insulator
Ag1Er1_ICSD_605102	metal	metal
Ag1Eu1O4Ti1_ICSD_78720	metal	insulator
Ag1Eu1P1_ICSD_52567	metal	metal
Ag1Eu1Sb1_ICSD_52568	metal	metal
Ag1Eu1_ICSD_58257	metal	metal
Ag1F10P1Xe2_ICSD_412662	insulator	insulator
Ag1F11Na1Zr2_ICSD_65179	insulator	insulator
Ag1F11Pd1Zr2_ICSD_65181	insulator	insulator
Ag1F12Sb2_ICSD_65186	insulator	insulator
Ag1F12Ta2_ICSD_62543	insulator	insulator
Ag1F1S1Sr1_ICSD_183709	insulator	insulator
Ag1F1Se1Sr1_ICSD_183710	insulator	insulator
Ag1F1Sr1Te1_ICSD_183711	insulator	insulator
Ag1F1_ICSD_18008	insulator	metal
Ag1F2O6S2_ICSD_422414	insulator	insulator

Supplementary Table 13. Five-fold cross validated predictions for the metal/insulator classification (5/598).

system	calculated	predicted
Ag1F2_ICSD_20453	metal	insulator
Ag1F2_ICSD_6277	insulator	insulator
Ag1F30O6S16Sb1Te6_ICSD_412325	insulator	insulator
Ag1F3K1_ICSD_189150	insulator	insulator
Ag1F3Rb1_ICSD_23153	insulator	insulator
Ag1F3Zn1_ICSD_28950	insulator	insulator
Ag1F3_ICSD_80477	insulator	insulator
Ag1F4K1_ICSD_72715	insulator	insulator
Ag1F4K2_ICSD_421461	insulator	insulator
Ag1F4Na1_ICSD_9903	insulator	insulator
Ag1F4Na2_ICSD_425149	insulator	insulator
Ag1F6Pd1_ICSD_51507	insulator	insulator
Ag1F6Sb1_ICSD_28676	insulator	insulator
Ag1F6Sn1_ICSD_51505	insulator	insulator
Ag1F6Ta1_ICSD_411796	insulator	insulator
Ag1F6Ti1_ICSD_51506	insulator	insulator
Ag1F7Ir1_ICSD_79880	metal	insulator
Ag1Fe1O2_ICSD_2786	metal	metal
Ag1Fe1O2_ICSD_31919	metal	metal
Ag1Fe1O6Se2_ICSD_90414	insulator	insulator
Ag1Fe1O7P2_ICSD_421413	insulator	insulator
Ag1Fe1O7V2_ICSD_166891	insulator	insulator
Ag1Fe1S2_ICSD_156643	metal	insulator
Ag1Fe1S2_ICSD_56263	metal	insulator
Ag1Fe1Te2_ICSD_43278	metal	metal
Ag1Fe2S3_ICSD_165661	insulator	metal
Ag1Ga1O2_ICSD_95665	insulator	insulator
Ag1Ga1P2Se6_ICSD_71971	insulator	insulator
Ag1Ga1S2_ICSD_156785	insulator	insulator
Ag1Ga1S2_ICSD_92052	insulator	insulator
Ag1Ga1Se2_ICSD_605211	insulator	insulator
Ag1Ga1Te2_ICSD_156128	insulator	insulator
Ag1Ga1_ICSD_104466	metal	metal
Ag1Gd1Ge1_ICSD_81556	metal	metal
Ag1Gd1Mg1_ICSD_657936	metal	metal
Ag1Gd1Mg2_ICSD_107733	metal	metal
Ag1Gd1Pb1_ICSD_107104	metal	metal
Ag1Gd1Se2_ICSD_602138	insulator	insulator
Ag1Gd1Si1_ICSD_605275	metal	metal
Ag1Gd1_ICSD_605255	metal	metal
Ag1Ge1Ho1_ICSD_86732	metal	metal
Ag1Ge1La1_ICSD_56985	metal	metal
Ag1Ge1La3S7_ICSD_80174	insulator	insulator
Ag1Ge1Pr3S7_ICSD_605303	insulator	insulator
Ag1Ge1Tb1_ICSD_86730	metal	metal
Ag1Ge1Yb1_ICSD_402450	metal	metal
Ag1H2Ni3O12P3_ICSD_280922	insulator	insulator
Ag1H2O4V1_ICSD_75941	insulator	insulator
Ag1H4Mo1N1S4_ICSD_236249	insulator	insulator
Ag1H4N1S4W1_ICSD_84370	insulator	insulator

Supplementary Table 14. Five-fold cross validated predictions for the metal/insulator classification (6/598).

system	calculated	predicted
Ag1Hf1Rb1Te3_ICSD_402632	insulator	insulator
Ag1Hf1_ICSD_605330	metal	metal
Ag1Hf2_ICSD_163152	metal	metal
Ag1Hg1I1S1_ICSD_411772	insulator	insulator
Ag1Hg1I1S1_ICSD_54796	insulator	insulator
Ag1Hg1O4V1_ICSD_414429	insulator	insulator
Ag1Hg2N1O5_ICSD_89685	insulator	insulator
Ag1Hg2O4P1_ICSD_2208	insulator	insulator
Ag1Hg2Ti1_ICSD_58276	metal	metal
Ag1Hg3O6Sb1_ICSD_170764	insulator	insulator
Ag1Ho1Pb1_ICSD_107107	metal	metal
Ag1Ho1S2_ICSD_40960	insulator	insulator
Ag1Ho1Se2_ICSD_156419	insulator	insulator
Ag1Ho1Se2_ICSD_605365	insulator	insulator
Ag1Ho1Sn1_ICSD_55824	metal	metal
Ag1Ho1Te2_ICSD_154795	insulator	insulator
Ag1Ho1_ICSD_605352	metal	metal
Ag1I1O4_ICSD_52380	insulator	insulator
Ag1I1Se3_ICSD_414116	insulator	insulator
Ag1I1Te3_ICSD_414117	insulator	insulator
Ag1I1_ICSD_161581	insulator	insulator
Ag1I1_ICSD_161582	insulator	insulator
Ag1I1_ICSD_164959	insulator	insulator
Ag1I1_ICSD_1899	insulator	insulator
Ag1I1_ICSD_28230	insulator	insulator
Ag1I1_ICSD_52361	insulator	insulator
Ag1I1_ICSD_56557	insulator	insulator
Ag1I1_ICSD_79678	insulator	insulator
Ag1I2Tl1_ICSD_26318	insulator	insulator
Ag1I3K2_ICSD_150289	insulator	insulator
Ag1I3O9Pd1_ICSD_174519	insulator	insulator
Ag1I3Rb2_ICSD_150290	insulator	insulator
Ag1I3Tl2_ICSD_78929	insulator	insulator
Ag1In1Li2_ICSD_58292	metal	metal
Ag1In1O2_ICSD_202429	insulator	insulator
Ag1In1O2_ICSD_95671	insulator	insulator
Ag1In1P2Se6_ICSD_71968	insulator	insulator
Ag1In1S2_ICSD_600585	insulator	insulator
Ag1In1S2_ICSD_605408	insulator	insulator
Ag1In1S2_ICSD_659385	insulator	insulator
Ag1In1Se2_ICSD_604401	metal	insulator
Ag1In1Se2_ICSD_605449	insulator	insulator
Ag1In1Te2_ICSD_104476	insulator	insulator
Ag1In1_ICSD_605384	metal	metal
Ag1In2Na3_ICSD_170868	metal	metal
Ag1In2_ICSD_58282	metal	metal
Ag1In2_ICSD_659383	metal	metal
Ag1In5Se8_ICSD_35597	insulator	insulator
Ag1In5Te8_ICSD_151871	insulator	insulator
Ag1K1O1_ICSD_188532	metal	metal

Supplementary Table 15. Five-fold cross validated predictions for the metal/insulator classification (7/598).

system	calculated	predicted
Ag1K1O1_ICSD_24818	insulator	insulator
Ag1K1O1_ICSD_25744	insulator	insulator
Ag1K2Nb1S4_ICSD_84292	insulator	insulator
Ag1K2P1_ICSD_402572	insulator	insulator
Ag1K2S4Sb1_ICSD_82144	insulator	insulator
Ag1K2S4Ta1_ICSD_84294	insulator	insulator
Ag1K2S4V1_ICSD_66840	insulator	insulator
Ag1K2Sb1_ICSD_1155	insulator	insulator
Ag1K2Se4V1_ICSD_50461	insulator	insulator
Ag1K3Se8Sn3_ICSD_416330	insulator	insulator
Ag1La1Mg1_ICSD_657932	metal	metal
Ag1La1O1S1_ICSD_89023	insulator	insulator
Ag1La1Pb1_ICSD_107099	metal	metal
Ag1La1Sb2_ICSD_95223	metal	metal
Ag1La1_ICSD_150963	metal	metal
Ag1La1_ICSD_58306	metal	metal
Ag1La3S7Si1_ICSD_409845	insulator	insulator
Ag1La3S7Sn1_ICSD_417316	insulator	insulator
Ag1La3Se7Si1_ICSD_605509	insulator	insulator
Ag1La3Se7Sn1_ICSD_417803	insulator	insulator
Ag1Li1_ICSD_247145	metal	metal
Ag1Li1_ICSD_58310	metal	metal
Ag1Li2Pb1_ICSD_58314	metal	metal
Ag1Li2Sb1_ICSD_52589	insulator	insulator
Ag1Li2Sn1_ICSD_58317	metal	metal
Ag1Li2Sn1_ICSD_58318	metal	metal
Ag1Lu1Pb1_ICSD_107278	metal	metal
Ag1Lu1Se2_ICSD_605535	insulator	insulator
Ag1Lu1Si1_ICSD_605536	metal	metal
Ag1Lu1Sn1_ICSD_245236	metal	metal
Ag1Lu1Sn1_ICSD_416377	metal	metal
Ag1Mg1Pr1_ICSD_657934	metal	metal
Ag1Mg1Sb1_ICSD_187149	metal	metal
Ag1Mg1Sb1_ICSD_187150	metal	metal
Ag1Mg1Sb1_ICSD_187151	metal	metal
Ag1Mg1Sm1_ICSD_657935	metal	metal
Ag1Mg1Y1_ICSD_104477	metal	metal
Ag1Mg1_ICSD_605538	metal	metal
Ag1Mg2Nd1_ICSD_110801	metal	metal
Ag1Mg2Pr1_ICSD_58329	metal	metal
Ag1Mg2Sm1_ICSD_58330	metal	metal
Ag1Mn1O4V1_ICSD_246202	insulator	insulator
Ag1Mn1O4_ICSD_30200	insulator	insulator
Ag1Mn3N1_ICSD_52592	metal	metal
Ag1Mo3O14P2_ICSD_74937	metal	insulator
Ag1Mo3Se3_ICSD_603624	metal	metal
Ag1Mo6S8_ICSD_600661	metal	metal
Ag1Mo6Se8_ICSD_605584	metal	metal
Ag1Mo6Te6_ICSD_40793	metal	metal
Ag1N1O2_ICSD_26750	insulator	insulator

Supplementary Table 16. Five-fold cross validated predictions for the metal/insulator classification (8/598).

system	calculated	predicted
Ag1N1O3_ICSD_1685	insulator	insulator
Ag1N1O3_ICSD_28103	insulator	insulator
Ag1N1O3_ICSD_35157	insulator	insulator
Ag1N1O3_ICSD_374	insulator	insulator
Ag1N1_ICSD_167874	metal	metal
Ag1N1_ICSD_185555	metal	metal
Ag1N1_ICSD_185565	metal	metal
Ag1N1_ICSD_185575	metal	metal
Ag1N2Na1O4_ICSD_20926	insulator	insulator
Ag1N3O4_ICSD_419628	insulator	insulator
Ag1N3_ICSD_183201	insulator	insulator
Ag1N3_ICSD_27135	insulator	insulator
Ag1Na1O1_ICSD_188524	insulator	insulator
Ag1Na1O1_ICSD_188531	metal	metal
Ag1Na1O3S2_ICSD_418297	insulator	insulator
Ag1Na2Sb1_ICSD_10010	insulator	insulator
Ag1Na3O2_ICSD_24817	insulator	insulator
Ag1Na3S2_ICSD_201800	insulator	insulator
Ag1Nb1O3_ICSD_164198	insulator	insulator
Ag1Nb1O3_ICSD_55647	insulator	insulator
Ag1Nb1O3_ICSD_55649	insulator	metal
Ag1Nb1Rb2S4_ICSD_50463	insulator	insulator
Ag1Nb1Rb2Se4_ICSD_402423	insulator	insulator
Ag1Nb3O8_ICSD_67244	insulator	insulator
Ag1Nb3S4_ICSD_605592	metal	metal
Ag1Nb3Te4_ICSD_605595	metal	metal
Ag1Nd1Pb1_ICSD_107102	metal	metal
Ag1Nd1Sn1_ICSD_155736	metal	metal
Ag1Nd1Sn1_ICSD_55821	metal	metal
Ag1Nd1_ICSD_605598	metal	metal
Ag1Ni1O2_ICSD_415451	metal	insulator
Ag1Ni1O2_ICSD_73974	metal	metal
Ag1Ni1Se2_ICSD_605616	metal	metal
Ag1Ni1Te2_ICSD_605619	metal	metal
Ag1O13P4Ta1_ICSD_86892	insulator	insulator
Ag1O1P1Th1_ICSD_183137	metal	insulator
Ag1O1P1Th1_ICSD_419463	insulator	metal
Ag1O1Rb1_ICSD_188526	insulator	insulator
Ag1O1Rb1_ICSD_188533	metal	insulator
Ag1O1Rb1_ICSD_40155	insulator	insulator
Ag1O1_ICSD_202055	insulator	metal
Ag1O1_ICSD_27667	metal	insulator
Ag1O1_ICSD_35662	metal	metal
Ag1O1_ICSD_43741	insulator	insulator
Ag1O2Rh1_ICSD_261561	insulator	insulator
Ag1O2Sc1_ICSD_422442	insulator	insulator
Ag1O2Sc1_ICSD_95668	insulator	insulator
Ag1O2Y1_ICSD_95674	insulator	insulator
Ag1O2Yb1_ICSD_163472	insulator	insulator
Ag1O3Sb1_ICSD_245292	insulator	insulator

Supplementary Table 17. Five-fold cross validated predictions for the metal/insulator classification (9/598).

system	calculated	predicted
Ag1O3Sb1_ICSD_25541	insulator	insulator
Ag1O3Ta1_ICSD_40830	insulator	insulator
Ag1O3Ta1_ICSD_40831	insulator	insulator
Ag1O3Te1Tl1_ICSD_169995	insulator	insulator
Ag1O3V1_ICSD_50645	insulator	insulator
Ag1O3V1_ICSD_82079	insulator	insulator
Ag1O4P1Zn1_ICSD_260974	insulator	insulator
Ag1O4Re1_ICSD_280086	insulator	insulator
Ag1O4Tc1_ICSD_281321	insulator	insulator
Ag1O5Se1V1_ICSD_417773	insulator	insulator
Ag1O5Te1V1_ICSD_417774	insulator	insulator
Ag1O6Tl1V2_ICSD_201934	insulator	insulator
Ag1P1Pd5_ICSD_605631	metal	metal
Ag1P1Pt5_ICSD_605633	metal	metal
Ag1P1S3_ICSD_653748	insulator	insulator
Ag1P1S4Zn1_ICSD_48197	insulator	insulator
Ag1P1Sr1_ICSD_52596	insulator	metal
Ag1P2S6Sc1_ICSD_63273	insulator	insulator
Ag1P2S6V1_ICSD_655222	insulator	insulator
Ag1P2Sc1Se6_ICSD_420302	insulator	insulator
Ag1P2Se6V1_ICSD_68143	insulator	insulator
Ag1P2_ICSD_35283	insulator	insulator
Ag1Pb1Pr1_ICSD_107101	insulator	metal
Ag1Pb1S3Sb1_ICSD_24257	insulator	insulator
Ag1Pb1S3Sb1_ICSD_8166	insulator	insulator
Ag1Pb1Tb1_ICSD_107105	metal	metal
Ag1Pb1Y1_ICSD_107275	metal	metal
Ag1Pd3Se1_ICSD_174642	metal	metal
Ag1Pr1Sn1_ICSD_55820	metal	metal
Ag1Pr1_ICSD_605674	metal	metal
Ag1Pr3S7Si1_ICSD_605682	insulator	insulator
Ag1Pr3Se7Si1_ICSD_605685	insulator	insulator
Ag1Pt3_ICSD_180887	metal	metal
Ag1Pt5Si1_ICSD_605693	metal	metal
Ag1Rb1S3U1_ICSD_249702	insulator	insulator
Ag1Rb1Se3U1_ICSD_249703	insulator	insulator
Ag1Rb1Se4_ICSD_87463	insulator	insulator
Ag1Rb2S4Ta1_ICSD_84295	insulator	insulator
Ag1Rb2S4V1_ICSD_66841	insulator	insulator
Ag1Rb2Se4V1_ICSD_50462	insulator	insulator
Ag1Rb3Se8Sn3_ICSD_416294	insulator	insulator
Ag1S1Tl1_ICSD_605754	insulator	insulator
Ag1S2Sb1_ICSD_16578	insulator	insulator
Ag1S2Sb1_ICSD_85130	insulator	insulator
Ag1S2Sb1_ICSD_94647	insulator	insulator
Ag1S2Yb1_ICSD_27090	metal	metal
Ag1S2Yb1_ICSD_27091	metal	metal
Ag1S3Ta1_ICSD_73804	metal	insulator
Ag1S3Ta1_ICSD_74442	metal	insulator
Ag1Sb1Sr1_ICSD_56990	metal	metal

Supplementary Table 18. Five-fold cross validated predictions for the metal/insulator classification (10/598).

system	calculated	predicted
Ag1Sb1Te2_ICSD_000000	metal	metal
Ag1Sb1Te2_ICSD_100000	metal	metal
Ag1Sb1Te2_ICSD_170662	metal	metal
Ag1Sb1Te2_ICSD_170663	metal	metal
Ag1Sb1Te2_ICSD_200000	metal	metal
Ag1Sb1Te2_ICSD_300000	metal	metal
Ag1Sb1Te2_ICSD_400000	metal	metal
Ag1Sb1Te2_ICSD_604040	metal	insulator
Ag1Sb1Te2_ICSD_704040	insulator	metal
Ag1Sb1Yb1_ICSD_83983	metal	metal
Ag1Sb3Zr5_ICSD_605789	metal	metal
Ag1Sc1Se2_ICSD_155115	insulator	insulator
Ag1Sc1Se2_ICSD_605797	insulator	insulator
Ag1Sc1_ICSD_58348	metal	metal
Ag1Sc6Te2_ICSD_94859	metal	metal
Ag1Se1Tl1_ICSD_100710	insulator	insulator
Ag1Se1_ICSD_605802	metal	metal
Ag1Se2Tb1_ICSD_605827	insulator	insulator
Ag1Se2Tl1_ICSD_30360	metal	insulator
Ag1Se2Y1_ICSD_605836	insulator	insulator
Ag1Se2Yb1_ICSD_605837	metal	insulator
Ag1Si1Sm1_ICSD_605842	metal	metal
Ag1Si1Tm1_ICSD_169472	metal	metal
Ag1Si1Tm1_ICSD_605849	metal	metal
Ag1Si1Y1_ICSD_605850	metal	metal
Ag1Si1Yb1_ICSD_52606	metal	metal
Ag1Sm1_ICSD_605855	metal	metal
Ag1Sn1Tb1_ICSD_55822	metal	metal
Ag1Sn1Tm1_ICSD_417550	metal	metal
Ag1Sn1Y1_ICSD_245234	metal	metal
Ag1Sn1Yb1_ICSD_410744	metal	metal
Ag1Sr1_ICSD_173623	metal	metal
Ag1Sr1_ICSD_58358	metal	metal
Ag1Tb1Te2_ICSD_154793	insulator	insulator
Ag1Tb1_ICSD_58363	metal	metal
Ag1Tb1_ICSD_58364	metal	metal
Ag1Te1Tl1_ICSD_23367	metal	insulator
Ag1Te1Tl1_ICSD_52609	insulator	metal
Ag1Te2Tl1_ICSD_43284	metal	metal
Ag1Te2Tl3_ICSD_61680	insulator	insulator
Ag1Te2Tm1_ICSD_156301	metal	metal
Ag1Te2Y1_ICSD_154792	insulator	insulator
Ag1Te2Y6_ICSD_160181	metal	metal
Ag1Te3_ICSD_37186	metal	metal
Ag1Th2_ICSD_150643	metal	metal
Ag1Ti1_ICSD_58369	metal	metal
Ag1Ti1_ICSD_605934	metal	metal
Ag1Ti2_ICSD_605935	metal	metal
Ag1Ti3_ICSD_58370	metal	metal
Ag1Tm1_ICSD_58372	metal	metal

Supplementary Table 19. Five-fold cross validated predictions for the metal/insulator classification (11/598).

system	calculated	predicted
Ag1Tm1_ICSD_605946	metal	metal
Ag1Tm1_ICSD_605952	metal	metal
Ag1Y1_ICSD_58376	metal	metal
Ag1Yb1_ICSD_58378	metal	metal
Ag1Yb1_ICSD_605970	metal	metal
Ag1Zn1_ICSD_150570	metal	metal
Ag1Zr1_ICSD_163155	metal	metal
Ag1Zr2_ICSD_605999	metal	metal
Ag1Zr3_ICSD_58392	metal	metal
Ag1_ICSD_56269	metal	metal
Ag1_ICSD_64707	metal	metal
Ag1_ICSD_77917	metal	metal
Ag2.67Cr1I0.67O4_ICSD_419833	insulator	insulator
Ag2Al1Sc1_ICSD_57333	metal	metal
Ag2Al7Ca3_ICSD_104173	metal	metal
Ag2As1Cs1S3_ICSD_421091	insulator	insulator
Ag2As1K1O4_ICSD_409793	insulator	insulator
Ag2As4Hg7I6_ICSD_391132	insulator	insulator
Ag2Ba1Ge1S4_ICSD_10040	insulator	insulator
Ag2Ba1Ge1Se4_ICSD_411405	insulator	insulator
Ag2Ba1Ge2_ICSD_25318	metal	metal
Ag2Ba1Hg2O4_ICSD_40835	insulator	insulator
Ag2Ba1Mn1O8V2_ICSD_408193	insulator	insulator
Ag2Ba1S2_ICSD_50183	insulator	insulator
Ag2Ba1S4Sn1_ICSD_41898	insulator	insulator
Ag2Ba1Se4Sn1_ICSD_170856	insulator	insulator
Ag2Ba1Sn2_ICSD_25332	metal	metal
Ag2Ba1Te2_ICSD_246048	insulator	insulator
Ag2Ba1_ICSD_240052	metal	metal
Ag2Ba3_ICSD_108847	metal	metal
Ag2Bi1O3_ICSD_410665	metal	insulator
Ag2Bi1O3_ICSD_415959	insulator	metal
Ag2Br14W6_ICSD_410959	insulator	insulator
Ag2Br1N1O3_ICSD_1311	insulator	insulator
Ag2Br6Hg7P8_ICSD_171256	insulator	insulator
Ag2C1Cl1N1O4S1_ICSD_410623	insulator	insulator
Ag2C1N2_ICSD_411091	insulator	insulator
Ag2C1O3_ICSD_281040	insulator	insulator
Ag2C1O3_ICSD_93988	insulator	insulator
Ag2C2O4_ICSD_109603	insulator	insulator
Ag2C4Cs1Cu1N4_ICSD_415572	insulator	insulator
Ag2C4Cu1K1N4_ICSD_415570	insulator	insulator
Ag2C4Cu1N4Rb1_ICSD_415571	insulator	insulator
Ag2C4N4S4Zn1_ICSD_71563	insulator	insulator
Ag2C4O4_ICSD_109770	insulator	insulator
Ag2Ca1Ge2_ICSD_25316	metal	metal
Ag2Ca1O12V4_ICSD_161369	insulator	insulator
Ag2Ca1_ICSD_57354	metal	metal
Ag2Ca4Si6_ICSD_410522	metal	metal
Ag2Cd1Ge1S4_ICSD_152753	insulator	insulator

Supplementary Table 20. Five-fold cross validated predictions for the metal/insulator classification (12/598).

system	calculated	predicted
Ag2Cd1Ge1S4_ICSD_423404	insulator	insulator
Ag2Cd1Mg1_ICSD_104404	metal	metal
Ag2Ce1Ge2_ICSD_246609	metal	metal
Ag2Ce1Ge2_ICSD_604945	metal	metal
Ag2Ce1In1_ICSD_57372	metal	metal
Ag2Ce1Si2_ICSD_52551	metal	metal
Ag2Cl1N1O3_ICSD_8013	insulator	insulator
Ag2Cl4Pd1_ICSD_65239	insulator	insulator
Ag2Cl6Cs2_ICSD_66067	metal	insulator
Ag2Cl6Re1_ICSD_156662	insulator	insulator
Ag2Cr1O4_ICSD_16298	insulator	insulator
Ag2Cr2O7_ICSD_2433	insulator	insulator
Ag2Cs2S4Ti1_ICSD_280645	insulator	insulator
Ag2Cu2O3_ICSD_51672	metal	metal
Ag2Dy1In1_ICSD_57382	metal	metal
Ag2Dy1_ICSD_150637	metal	metal
Ag2Er1In1_ICSD_58254	metal	metal
Ag2Er1_ICSD_605091	metal	metal
Ag2Eu1Si2_ICSD_106697	metal	metal
Ag2Eu1Si2_ICSD_605133	metal	metal
Ag2Eu1_ICSD_58258	metal	metal
Ag2Eu1_ICSD_605125	metal	metal
Ag2Eu3_ICSD_58259	metal	metal
Ag2F14Zn1Zr2_ICSD_422839	insulator	insulator
Ag2F1H2I1O1_ICSD_32660	insulator	insulator
Ag2F1_ICSD_68439	metal	metal
Ag2F5_ICSD_95832	insulator	insulator
Ag2Fe1S4Sn1_ICSD_42534	insulator	insulator
Ag2Ga1_ICSD_151175	metal	metal
Ag2Gd1In1_ICSD_185964	metal	metal
Ag2Gd1Si2_ICSD_52574	metal	metal
Ag2Gd1Si2_ICSD_605276	metal	metal
Ag2Gd1_ICSD_104473	metal	metal
Ag2Ge1In2S6_ICSD_159759	insulator	insulator
Ag2Ge1In2Se6_ICSD_88168	insulator	insulator
Ag2Ge1K2S4_ICSD_170843	insulator	insulator
Ag2Ge1Li1_ICSD_85307	metal	metal
Ag2Ge1O3_ICSD_167332	insulator	insulator
Ag2Ge1O4Zn1_ICSD_81328	metal	insulator
Ag2Ge1Pb1S4_ICSD_180802	insulator	insulator
Ag2Ge1Rb2S4_ICSD_170844	insulator	insulator
Ag2Ge1S3_ICSD_41711	insulator	insulator
Ag2Ge2Nd1_ICSD_247286	metal	metal
Ag2Ge2Pr1_ICSD_154450	metal	metal
Ag2Ge2Sr1_ICSD_25317	metal	metal
Ag2H12N4O4S1_ICSD_36585	insulator	insulator
Ag2H1O4P1_ICSD_30503	insulator	insulator
Ag2H3I1O6_ICSD_155415	insulator	insulator
Ag2H4O12S3_ICSD_408949	insulator	insulator
Ag2H4O6Te1_ICSD_240968	metal	insulator

Supplementary Table 21. Five-fold cross validated predictions for the metal/insulator classification (13/598).

system	calculated	predicted
Ag2H6N2O6Pd1S2_ICSD_280075	insulator	insulator
Ag2Hf1S3_ICSD_79251	insulator	insulator
Ag2Hg1I2S1_ICSD_413300	insulator	insulator
Ag2Hg1I4_ICSD_30264	insulator	insulator
Ag2Hg1I4_ICSD_6069	insulator	insulator
Ag2Hg1O2_ICSD_280333	insulator	insulator
Ag2Hg1S2_ICSD_201713	insulator	insulator
Ag2Hg1Se4Sn1_ICSD_95094	metal	insulator
Ag2Hg2O12Te3_ICSD_171006	insulator	insulator
Ag2Hg7I6P8_ICSD_171257	insulator	insulator
Ag2Ho1In1_ICSD_58280	metal	metal
Ag2Ho1_ICSD_605358	metal	metal
Ag2I10Tl6_ICSD_35389	insulator	insulator
Ag2I1N1O3_ICSD_8075	insulator	insulator
Ag2I6O18Ti1_ICSD_420852	insulator	insulator
Ag2In1La1_ICSD_58290	metal	metal
Ag2In1Li1_ICSD_171472	metal	metal
Ag2In1Mg1_ICSD_605394	metal	metal
Ag2In1Nd1_ICSD_58295	metal	metal
Ag2In1Pr1_ICSD_58297	metal	metal
Ag2In1Sc1_ICSD_58298	metal	metal
Ag2In1Sm1_ICSD_58299	metal	metal
Ag2In1Tb1_ICSD_56987	metal	metal
Ag2In1Tm1_ICSD_58303	metal	metal
Ag2In1Y1_ICSD_58304	metal	metal
Ag2In2S6Si1_ICSD_189391	insulator	insulator
Ag2In2Se6Si1_ICSD_154635	insulator	insulator
Ag2K1Nb1Se4_ICSD_412479	insulator	insulator
Ag2K1P1S4_ICSD_420033	insulator	insulator
Ag2K1S3Sb1_ICSD_420015	insulator	insulator
Ag2K1S4Sb1_ICSD_82143	insulator	insulator
Ag2K1Se4Ta1_ICSD_412477	insulator	insulator
Ag2K1_ICSD_150142	metal	metal
Ag2K2Se4Sn1_ICSD_90119	insulator	insulator
Ag2La1Si2_ICSD_52587	metal	metal
Ag2La1_ICSD_605497	metal	metal
Ag2Li1Sn1_ICSD_154086	metal	metal
Ag2Li3Si3_ICSD_85305	metal	metal
Ag2Lu1_ICSD_605531	metal	metal
Ag2Mg1Zn1_ICSD_605562	metal	metal
Ag2Mn1O4_ICSD_35762	metal	insulator
Ag2Mn1O8Sr1V2_ICSD_408052	insulator	insulator
Ag2Mo1O12Te4_ICSD_420406	insulator	insulator
Ag2Mo1O4_ICSD_36187	insulator	insulator
Ag2Mo2O7_ICSD_31027	insulator	insulator
Ag2Mo3O16Te3_ICSD_420405	insulator	insulator
Ag2Na1_ICSD_58337	metal	metal
Ag2Nb4O11_ICSD_180731	insulator	insulator
Ag2Nb4O11_ICSD_180733	insulator	insulator
Ag2Nd1Si2_ICSD_106695	metal	metal

Supplementary Table 22. Five-fold cross validated predictions for the metal/insulator classification (14/598).

system	calculated	predicted
Ag2Nd1_ICSD_58339	metal	metal
Ag2Nd1_ICSD_605603	metal	metal
Ag2Ni1O2_ICSD_160582	metal	metal
Ag2Ni1O2_ICSD_290597	metal	metal
Ag2O10U1W2_ICSD_98550	insulator	insulator
Ag2O11Ta4_ICSD_180734	insulator	insulator
Ag2O11V4_ICSD_93453	insulator	insulator
Ag2O12Sr1V4_ICSD_161371	insulator	insulator
Ag2O12U2V2_ICSD_81576	insulator	insulator
Ag2O1_ICSD_20368	metal	metal
Ag2O1_ICSD_246904	metal	metal
Ag2O2Pd1_ICSD_51499	insulator	metal
Ag2O3S1_ICSD_4433	insulator	insulator
Ag2O3Se1_ICSD_78388	insulator	insulator
Ag2O3Si1_ICSD_36589	insulator	insulator
Ag2O3Ti1_ICSD_84970	insulator	insulator
Ag2O3_ICSD_15999	metal	metal
Ag2O3_ICSD_59193	insulator	metal
Ag2O4S1_ICSD_69096	insulator	insulator
Ag2O4Se1_ICSD_413089	insulator	insulator
Ag2O4Si1Zn1_ICSD_81327	metal	insulator
Ag2O4Te1_ICSD_414464	insulator	insulator
Ag2O6P1V1_ICSD_73580	insulator	insulator
Ag2O6Te2_ICSD_415472	insulator	insulator
Ag2O6Te2_ICSD_417354	insulator	insulator
Ag2O7P2Pb1_ICSD_93967	insulator	insulator
Ag2O7P2Zn1_ICSD_90917	insulator	insulator
Ag2O7S2_ICSD_423166	insulator	insulator
Ag2O7Te2_ICSD_416281	metal	insulator
Ag2O7W2_ICSD_31028	insulator	insulator
Ag2P2S11Ti2_ICSD_84606	insulator	insulator
Ag2Pr1Si2_ICSD_106694	metal	metal
Ag2Pr1_ICSD_58346	metal	metal
Ag2Pr1_ICSD_605675	metal	metal
Ag2Rb1S4Sb1_ICSD_82145	insulator	insulator
Ag2Rh15S15_ICSD_605703	metal	metal
Ag2S1_ICSD_173999	metal	insulator
Ag2S1_ICSD_262633	metal	insulator
Ag2S1_ICSD_262634	metal	insulator
Ag2S1_ICSD_262637	insulator	insulator
Ag2S1_ICSD_98452	insulator	insulator
Ag2S1_ICSD_98453	insulator	insulator
Ag2S3Si1_ICSD_180764	insulator	insulator
Ag2S3Te1_ICSD_85135	insulator	insulator
Ag2S4Sn1Zn1_ICSD_605734	insulator	insulator
Ag2Sc1_ICSD_605791	metal	metal
Ag2Se1_ICSD_15213	metal	metal
Ag2Se1_ICSD_43242	metal	metal
Ag2Si1_ICSD_605838	metal	metal
Ag2Si2Sm1_ICSD_106696	metal	metal

Supplementary Table 23. Five-fold cross validated predictions for the metal/insulator classification (15/598).

system	calculated	predicted
Ag2Si2Sr1_ICSD_25330	metal	metal
Ag2Si2Tb1_ICSD_98341	metal	metal
Ag2Si2Yb1_ICSD_52607	metal	metal
Ag2Sn2Sr1_ICSD_414	metal	metal
Ag2Sr1_ICSD_58359	metal	metal
Ag2Sr3_ICSD_58360	metal	metal
Ag2Tb1_ICSD_605886	metal	metal
Ag2Te1_ICSD_73402	metal	metal
Ag2Th1_ICSD_15443	metal	metal
Ag2Tm1_ICSD_58373	metal	metal
Ag2Y1_ICSD_605957	metal	metal
Ag2Yb1_ICSD_58380	metal	metal
Ag2Yb1_ICSD_605966	metal	metal
Ag2Yb3_ICSD_605972	metal	metal
Ag2Zr1_ICSD_605995	metal	metal
Ag3Al2La1_ICSD_57329	metal	metal
Ag3As1O4_ICSD_35545	insulator	insulator
Ag3As1S3_ICSD_36352	insulator	insulator
Ag3As1S3_ICSD_419203	insulator	insulator
Ag3As1S4_ICSD_86227	insulator	insulator
Ag3As1Se3_ICSD_76519	insulator	metal
Ag3As1Se3_ICSD_82636	insulator	insulator
Ag3As2K3_ICSD_32016	insulator	insulator
Ag3Au1S2_ICSD_15732	insulator	insulator
Ag3Au1Se2_ICSD_171959	insulator	insulator
Ag3Au1Te2_ICSD_15733	insulator	metal
Ag3B1O3_ICSD_26521	metal	insulator
Ag3B1O3_ICSD_32721	insulator	insulator
Ag3Ba4Ge20_ICSD_56980	metal	metal
Ag3Br1S1_ICSD_174097	insulator	insulator
Ag3C1N3O6S1_ICSD_23513	insulator	insulator
Ag3C6Cd1N6Rb1_ICSD_75503	insulator	insulator
Ag3C6Co1N6_ICSD_16959	insulator	insulator
Ag3C6Fe1H6N8_ICSD_64627	metal	insulator
Ag3C6Fe1N6_ICSD_173553	metal	insulator
Ag3C6H6La1N6O3_ICSD_170986	insulator	insulator
Ag3C6H6N6O3Tb1_ICSD_249343	insulator	insulator
Ag3C6K1Mn1N6_ICSD_262507	insulator	insulator
Ag3C6K2N6Na1_ICSD_59725	insulator	insulator
Ag3C6K2N6Na1_ICSD_65699	insulator	insulator
Ag3Ca5_ICSD_57355	metal	metal
Ag3Ce1K2Te4_ICSD_86678	insulator	insulator
Ag3Co2O6Sb1_ICSD_174288	insulator	insulator
Ag3Cs1S2_ICSD_1033	insulator	insulator
Ag3Cs1Se5Tb2_ICSD_93684	insulator	insulator
Ag3Cu1S2_ICSD_163982	insulator	insulator
Ag3Cu1S2_ICSD_163983	insulator	insulator
Ag3Er3Ga8_ICSD_605107	metal	metal
Ag3F14Hf2_ICSD_65178	insulator	insulator
Ag3Fe1O8V2_ICSD_166890	insulator	insulator

Supplementary Table 24. Five-fold cross validated predictions for the metal/insulator classification (16/598).

system	calculated	predicted
Ag3Ga8Ho3_ICSD_605157	metal	metal
Ag3Ge3P6Sn2_ICSD_52575	insulator	insulator
Ag3I1S1_ICSD_201004	insulator	insulator
Ag3In1O8P2_ICSD_245001	insulator	insulator
Ag3In1_ICSD_58283	metal	metal
Ag3In2O12P3_ICSD_245002	insulator	insulator
Ag3K1Se2_ICSD_402643	insulator	insulator
Ag3K1Te2_ICSD_402230	insulator	insulator
Ag3Li17Sn6_ICSD_170571	metal	metal
Ag3Li1O2_ICSD_4204	insulator	insulator
Ag3Mg1_ICSD_58323	metal	metal
Ag3N1O3S1_ICSD_25523	insulator	insulator
Ag3N1O3Se1_ICSD_33581	insulator	insulator
Ag3Na1O2_ICSD_9627	insulator	insulator
Ag3Na1S2_ICSD_73198	insulator	insulator
Ag3Ni2O4_ICSD_172878	metal	metal
Ag3O4P1_ICSD_31078	metal	insulator
Ag3O4P1_ICSD_76968	insulator	insulator
Ag3O4Ru1_ICSD_59924	insulator	insulator
Ag3O4Sb1_ICSD_417675	insulator	insulator
Ag3O4V1_ICSD_249417	insulator	insulator
Ag3O4V1_ICSD_417470	insulator	insulator
Ag3O4_ICSD_202218	metal	insulator
Ag3P11_ICSD_26563	insulator	insulator
Ag3P1S4_ICSD_416585	insulator	insulator
Ag3P1Se4_ICSD_97760	insulator	insulator
Ag3P2S8Y1_ICSD_417658	insulator	insulator
Ag3P6Si3Sn2_ICSD_52595	insulator	insulator
Ag3P7Sn1_ICSD_411041	insulator	insulator
Ag3Pt1_ICSD_605689	metal	metal
Ag3Rb1S2_ICSD_1034	insulator	insulator
Ag3Rb1Se2_ICSD_90795	insulator	insulator
Ag3Rb1Se5Sm2_ICSD_91095	insulator	insulator
Ag3Rb1Te2_ICSD_90872	insulator	insulator
Ag3S2Tl1_ICSD_75976	insulator	insulator
Ag3S3Sb1_ICSD_181518	insulator	insulator
Ag3S3Sb1_ICSD_33714	insulator	insulator
Ag3S6Sb2Tl3_ICSD_160100	insulator	insulator
Ag3Sb1_ICSD_52600	metal	metal
Ag3Sb1_ICSD_64716	metal	metal
Ag3Sn1_ICSD_2721	metal	metal
Ag3Te2Tl1_ICSD_71081	metal	metal
Ag3Tm1_ICSD_58374	metal	metal
Ag3Yb5_ICSD_58382	metal	metal
Ag4C4N4S4_ICSD_159147	insulator	insulator
Ag4Cd1Ge2S7_ICSD_95121	insulator	insulator
Ag4Cu1O6Te1_ICSD_416931	metal	insulator
Ag4Dy3Sn4_ICSD_157373	metal	metal
Ag4Eu1Sb2_ICSD_424312	metal	metal
Ag4Ho3Sn4_ICSD_157374	metal	metal

Supplementary Table 25. Five-fold cross validated predictions for the metal/insulator classification (17/598).

system	calculated	predicted
Ag4I1O4P1_ICSD_245791	insulator	insulator
Ag4I2O4Se1_ICSD_418902	insulator	insulator
Ag4K2S3_ICSD_863	insulator	insulator
Ag4K2Se3_ICSD_54102	insulator	insulator
Ag4Lu1_ICSD_58321	metal	metal
Ag4Mn1S6Sb2_ICSD_156764	insulator	insulator
Ag4Mn1S6Sb2_ICSD_38360	insulator	insulator
Ag4Mn3O8_ICSD_414178	insulator	insulator
Ag4N2O2S1_ICSD_23111	insulator	insulator
Ag4N2O6Te1_ICSD_16006	metal	insulator
Ag4O4S1Te1_ICSD_421880	insulator	insulator
Ag4O4Si1_ICSD_418314	insulator	insulator
Ag4P2S6_ICSD_24782	insulator	insulator
Ag4P2Se6_ICSD_1727	insulator	insulator
Ag4Pr3Sn4_ICSD_164498	metal	metal
Ag4Rb2S3_ICSD_409727	insulator	insulator
Ag4S8Sn3_ICSD_164435	insulator	insulator
Ag4Sb2Sr1_ICSD_424311	metal	metal
Ag4Sc1_ICSD_58349	metal	metal
Ag5Ba1_ICSD_57344	metal	metal
Ag5Ba2La1S6_ICSD_67894	insulator	insulator
Ag5Ba2S6Y1_ICSD_659302	insulator	insulator
Ag5Cd8_ICSD_604897	metal	metal
Ag5Cl2P1S4_ICSD_416587	insulator	insulator
Ag5Cl5Hg4P8_ICSD_416360	insulator	insulator
Ag5Cs1Se3_ICSD_90871	insulator	insulator
Ag5Eu1_ICSD_58260	metal	metal
Ag5Eu1_ICSD_605121	metal	metal
Ag5Eu1_ICSD_605128	metal	metal
Ag5Hg1O6Sb1_ICSD_421241	metal	insulator
Ag5I1O6_ICSD_415893	metal	insulator
Ag5O4P1Se1_ICSD_420342	insulator	insulator
Ag5O4P1Te1_ICSD_420343	insulator	insulator
Ag5O4Si1_ICSD_165377	insulator	insulator
Ag5O6Pb2_ICSD_155044	metal	metal
Ag5O6Pb2_ICSD_24038	metal	metal
Ag5Rb1Se3_ICSD_50738	metal	metal
Ag5S4Sb1_ICSD_36347	insulator	insulator
Ag5Sr1_ICSD_58361	metal	metal
Ag5Zn8_ICSD_58389	metal	metal
Ag6Ba1O4_ICSD_9288	insulator	insulator
Ag6Ce1N9O27_ICSD_59256	metal	insulator
Ag6Cl1F3Mo2O7_ICSD_413082	insulator	insulator
Ag6Cr1O8Si1_ICSD_420804	metal	insulator
Ag6Ge10P12_ICSD_70055	insulator	insulator
Ag6Ge1O8S1_ICSD_300179	metal	metal
Ag6Ge2O7_ICSD_404356	insulator	insulator
Ag6K2S4_ICSD_73581	insulator	insulator
Ag6O2_ICSD_26557	metal	metal
Ag6O4Sr1_ICSD_10359	insulator	insulator

Supplementary Table 26. Five-fold cross validated predictions for the metal/insulator classification (18/598).

system	calculated	predicted
Ag6O7Si2_ICSD_404355	insulator	insulator
Ag6O8S1Si1_ICSD_6225	metal	metal
Ag7As1S6_ICSD_604743	insulator	insulator
Ag7As1Se6_ICSD_604758	metal	insulator
Ag7Ca2_ICSD_55510	metal	metal
Ag7F31Zr6_ICSD_92446	insulator	insulator
Ag7P1S6_ICSD_601451	insulator	insulator
Ag7P1Se6_ICSD_54055	insulator	insulator
Ag7P3S11_ICSD_414335	insulator	insulator
Ag7Yb2_ICSD_370003	metal	metal
Ag8Al4Li4_ICSD_414342	metal	metal
Ag8Ca19N7_ICSD_410770	metal	metal
Ag8Ca3_ICSD_107145	metal	metal
Ag8Ce6Sn8_ICSD_55826	metal	metal
Ag8Ge1S6_ICSD_100079	insulator	insulator
Ag8Ge1Se6_ICSD_10432	metal	metal
Ag8O4S2Si1_ICSD_2330	metal	insulator
Ag8Pr6Sn8_ICSD_55827	metal	metal
Ag8S1_ICSD_79770	metal	metal
Ag8S6Si1_ICSD_1054	insulator	insulator
Ag8S6Sn1_ICSD_42533	insulator	insulator
Ag8S6Ti1_ICSD_95648	insulator	insulator
Ag8Se6Sn1_ICSD_95093	metal	metal
Ag9Ga1Se6_ICSD_2411	metal	metal
Ag9Ge2I1O8_ICSD_380303	insulator	insulator
Ag9In4_ICSD_832	metal	metal
Al0.4C0.2Mo0.6_ICSD_606230	metal	metal
Al0.4C0.2Nb0.6_ICSD_606235	metal	metal
Al0.4C0.2Ta0.6_ICSD_606257	metal	metal
Al102Re24Si12_ICSD_95127	metal	metal
Al10Ba7_ICSD_420092	metal	metal
Al10Ce1Ru2_ICSD_59912	metal	metal
Al10Fe2Th1_ICSD_152354	metal	metal
Al10Fe2U1_ICSD_155072	metal	metal
Al10Fe2Y1_ICSD_107224	metal	metal
Al10Fe2Yb1_ICSD_151140	metal	metal
Al10H2O16_ICSD_23651	insulator	insulator
Al10Mn3_ICSD_57974	metal	metal
Al10Pr1Ru2_ICSD_186913	metal	metal
Al10Ru2U1_ICSD_182114	metal	metal
Al10Ru2Yb1_ICSD_186915	metal	metal
Al10V1_ICSD_58202	metal	metal
Al11La3_ICSD_608281	metal	metal
Al11Mn4_ICSD_10509	metal	metal
Al12Ca1O19_ICSD_34394	insulator	insulator
Al12Ca1O27Si4_ICSD_91233	insulator	insulator
Al12Ca8O24S2_ICSD_67589	insulator	insulator
Al12Ca8O24Te2_ICSD_86156	insulator	insulator
Al12Cd8O24S2_ICSD_78368	insulator	insulator
Al12Cd8O24Te2_ICSD_86155	insulator	insulator

Supplementary Table 27. Five-fold cross validated predictions for the metal/insulator classification (19/598).

system	calculated	predicted
Al12Ce3Ru4_ICSD_99134	metal	metal
Al12Dy5Fe4Mg5Si6_ICSD_262937	metal	metal
Al12I21La24_ICSD_416019	metal	metal
Al12Mg17_ICSD_158247	metal	metal
Al12Mn1_ICSD_608472	metal	metal
Al12Mo1_ICSD_608577	metal	metal
Al12Nd4O32Pb8_ICSD_406531	insulator	insulator
Al12O19Sr1_ICSD_2006	insulator	insulator
Al12O24S2Sr8_ICSD_67590	insulator	insulator
Al12O24Sr8Te2_ICSD_82609	insulator	insulator
Al12Re1_ICSD_109107	metal	metal
Al12Ru1_ICSD_609231	metal	metal
Al12Ta17_ICSD_609426	metal	metal
Al12Tc1_ICSD_58178	metal	metal
Al12W1_ICSD_58207	metal	metal
Al13Ba7_ICSD_170239	metal	metal
Al13Cr4Si4_ICSD_76116	metal	metal
Al13La16_ICSD_414423	metal	metal
Al13Os4_ICSD_58110	metal	metal
Al13Ru4_ICSD_58158	metal	metal
Al14Ca12O32_ICSD_164634	metal	insulator
Al14Ca13_ICSD_165239	metal	metal
Al14Mg13_ICSD_150647	metal	metal
Al14O25Sr4_ICSD_88527	insulator	insulator
Al16Ce2Pt9_ICSD_163921	metal	metal
Al16Co7Hf6_ICSD_606582	metal	metal
Al16Co7Ti6_ICSD_606678	metal	metal
Al16Co7Zr6_ICSD_606726	metal	metal
Al16Hf6Os7_ICSD_608133	metal	metal
Al16Hf6Pd7_ICSD_608137	metal	metal
Al16Hf6Pt7_ICSD_608140	metal	metal
Al16Hf6Rh7_ICSD_608144	metal	metal
Al16Ir7Sc6_ICSD_608246	metal	metal
Al16Ir7Ti6_ICSD_608248	metal	metal
Al16Ir7Zr6_ICSD_608254	metal	metal
Al16Ni7Sc6_ICSD_608840	metal	metal
Al16Ni7Ti6_ICSD_608909	metal	metal
Al16Ni7Zr6_ICSD_608966	metal	metal
Al16Os7Sc6_ICSD_609011	metal	metal
Al16Os7Ti6_ICSD_609012	metal	metal
Al16Os7Zr6_ICSD_609018	metal	metal
Al16Pd7Sc6_ICSD_609052	metal	metal
Al16Pd7Ti6_ICSD_609061	metal	metal
Al16Pd7Zr6_ICSD_609066	metal	metal
Al16Pt7Ti6_ICSD_609161	metal	metal
Al16Pt7Zr6_ICSD_609168	metal	metal
Al16Rh7Sc6_ICSD_609216	metal	metal
Al16Rh7Ti6_ICSD_609218	metal	metal
Al16Rh7Zr6_ICSD_609222	metal	metal
Al16Ru7Sc6_ICSD_609239	metal	metal

Supplementary Table 28. Five-fold cross validated predictions for the metal/insulator classification (20/598).

system	calculated	predicted
Al16Ru7Ti6_ICSD_609242	metal	metal
Al16Ru7Zr6_ICSD_609247	metal	metal
Al17Dy2_ICSD_607243	metal	metal
Al17Er2_ICSD_657488	metal	metal
Al17Mo4_ICSD_400887	metal	metal
Al17Pd8Si4_ICSD_52650	metal	metal
Al17Tb2_ICSD_609466	metal	metal
Al18Cr2Mg3_ICSD_57659	metal	metal
Al18Mg3Mn2_ICSD_608417	metal	metal
Al18Mg3Mo2_ICSD_608418	metal	metal
Al18Mg3Ta2_ICSD_608444	metal	metal
Al18Mg3Ti2_ICSD_608446	metal	metal
Al18Mg3V2_ICSD_608447	metal	metal
Al18Mg3W2_ICSD_608448	metal	metal
Al18Ni5U4_ICSD_58070	metal	metal
Al1As1Cu1O5_ICSD_91551	insulator	insulator
Al1As1F1Na1O4_ICSD_30205	insulator	insulator
Al1As1H4O6_ICSD_170740	insulator	insulator
Al1As1O4_ICSD_24512	insulator	insulator
Al1As1O4_ICSD_67228	insulator	insulator
Al1As1_ICSD_56967	metal	metal
Al1As1_ICSD_606008	insulator	insulator
Al1As1_ICSD_67771	insulator	insulator
Al1As2K1O7_ICSD_79711	insulator	insulator
Al1As2K2Na1_ICSD_73280	insulator	insulator
Al1As2Li1O7_ICSD_161498	insulator	insulator
Al1As2Li3Mo2O14_ICSD_260199	insulator	insulator
Al1As2Na1O7_ICSD_75142	insulator	insulator
Al1As2Na3_ICSD_63149	insulator	insulator
Al1As3Ca3_ICSD_32727	insulator	insulator
Al1As3Cl4S5_ICSD_415503	insulator	insulator
Al1As4Ca3Na3_ICSD_402309	insulator	insulator
Al1Au1Ca1_ICSD_370015	metal	metal
Al1Au1Ce1_ICSD_658143	metal	metal
Al1Au1Dy1_ICSD_602605	metal	metal
Al1Au1Er1_ICSD_602607	metal	metal
Al1Au1Eu1_ICSD_602609	metal	metal
Al1Au1Gd1_ICSD_602627	metal	metal
Al1Au1Ho1_ICSD_602606	metal	metal
Al1Au1La1_ICSD_602633	metal	metal
Al1Au1Nd1_ICSD_602636	metal	metal
Al1Au1O2_ICSD_95663	insulator	insulator
Al1Au1Pr1_ICSD_602635	metal	metal
Al1Au1Tb1_ICSD_602604	metal	metal
Al1Au1Ti1_ICSD_57506	metal	metal
Al1Au1Y1_ICSD_602610	metal	metal
Al1Au1Yb1_ICSD_658146	metal	metal
Al1Au1_ICSD_104649	metal	metal
Al1Au1_ICSD_57495	metal	metal
Al1Au2Hf1_ICSD_57503	metal	metal

Supplementary Table 29. Five-fold cross validated predictions for the metal/insulator classification (21/598).

system	calculated	predicted
Al1Au2Mn1_ICSD_57504	metal	metal
Al1Au2Sc1_ICSD_57505	metal	metal
Al1Au2Ti1_ICSD_57507	metal	metal
Al1Au2U1_ICSD_657425	metal	metal
Al1Au2_ICSD_57496	metal	metal
Al1Au2_ICSD_57497	metal	metal
Al1Au2_ICSD_606020	metal	metal
Al1Au4_ICSD_606018	metal	metal
Al1B14Er1_ICSD_602386	metal	metal
Al1B14Ho1_ICSD_655666	metal	metal
Al1B14Ni20_ICSD_408418	metal	metal
Al1B1Ba1F2O3_ICSD_409663	insulator	insulator
Al1B1Ba1F2O3_ICSD_91316	insulator	insulator
Al1B1Be1_ICSD_43351	metal	metal
Al1B1Ca1O4_ICSD_27647	insulator	insulator
Al1B1H3Na1O10P2_ICSD_409427	insulator	insulator
Al1B1Li2O4_ICSD_50612	insulator	insulator
Al1B1Mg1O4_ICSD_34349	insulator	insulator
Al1B1Mo1_ICSD_16777	metal	metal
Al1B1O3_ICSD_30538	insulator	insulator
Al1B1O4Pb1_ICSD_98572	insulator	insulator
Al1B2Cr2_ICSD_20083	metal	metal
Al1B2Fe2_ICSD_20322	metal	metal
Al1B2Li1O5_ICSD_51314	insulator	insulator
Al1B2Li3O6_ICSD_51754	insulator	insulator
Al1B2Mn2_ICSD_25518	metal	metal
Al1B2_ICSD_52282	metal	metal
Al1B3Ca1O7_ICSD_161813	insulator	insulator
Al1B4Cr3_ICSD_20082	metal	metal
Al1B4Lu1_ICSD_656223	metal	metal
Al1B4Yb1_ICSD_167533	metal	metal
Al1B4Yb1_ICSD_181368	metal	metal
Al1B4Yb1_ICSD_260796	metal	metal
Al1B6Yb2_ICSD_41404	metal	metal
Al1Ba1F5_ICSD_37033	insulator	insulator
Al1Ba1F5_ICSD_80565	insulator	insulator
Al1Ba1Ge1_ICSD_417662	metal	metal
Al1Ba1H1Si1_ICSD_162869	insulator	insulator
Al1Ba1La1O4_ICSD_62490	insulator	insulator
Al1Ba1Si1_ICSD_162866	metal	metal
Al1Ba2Cu2F11_ICSD_50483	insulator	insulator
Al1Ba2In1O5_ICSD_33805	insulator	insulator
Al1Ba3F9_ICSD_72718	insulator	insulator
Al1Ba3H1O4_ICSD_280520	insulator	insulator
Al1Be1H5_ICSD_156311	insulator	insulator
Al1Be1H5_ICSD_156312	insulator	insulator
Al1Be1La3S7_ICSD_606164	insulator	insulator
Al1Be1Na3O8Si2_ICSD_4334	insulator	insulator
Al1Bi1O3_ICSD_158756	insulator	insulator
Al1Bi1O3_ICSD_185508	insulator	insulator

Supplementary Table 30. Five-fold cross validated predictions for the metal/insulator classification (22/598).

system	calculated	predicted
Al1Bi1_ICSD_184567	metal	metal
Al1Bi2Br1Cl4Te2_ICSD_174525	insulator	insulator
Al1Br1Cl4Sb2Te2_ICSD_174524	insulator	insulator
Al1Br4Cs1_ICSD_83435	insulator	insulator
Al1Br4Cu1_ICSD_165608	insulator	insulator
Al1Br4Tl1_ICSD_419829	insulator	insulator
Al1Br7Se1_ICSD_401396	insulator	insulator
Al1C1.67Zr1_ICSD_606293	metal	metal
Al1C1Ce3_ICSD_43846	metal	metal
Al1C1Co3_ICSD_43847	metal	metal
Al1C1Cr2_ICSD_42918	metal	metal
Al1C1Dy3_ICSD_43848	metal	metal
Al1C1Er3_ICSD_43849	metal	metal
Al1C1Er3_ICSD_606192	metal	metal
Al1C1F5O3Sr2_ICSD_201803	insulator	insulator
Al1C1Fe3_ICSD_43853	metal	metal
Al1C1Gd3_ICSD_43854	metal	metal
Al1C1H2K1O5_ICSD_153303	insulator	insulator
Al1C1H2Na1O5_ICSD_100140	insulator	insulator
Al1C1Ho3_ICSD_43856	metal	metal
Al1C1La3_ICSD_56395	metal	metal
Al1C1Mn3_ICSD_43860	metal	metal
Al1C1Mn3_ICSD_606226	metal	metal
Al1C1Nb2_ICSD_606236	metal	metal
Al1C1Nd3_ICSD_57528	metal	metal
Al1C1O1Sc1_ICSD_419683	insulator	insulator
Al1C1Pr3_ICSD_185962	metal	metal
Al1C1Pt3_ICSD_43864	metal	metal
Al1C1Sc2_ICSD_160378	metal	metal
Al1C1Sc3_ICSD_50161	metal	metal
Al1C1Ta2_ICSD_181247	metal	metal
Al1C1Ta2_ICSD_187514	metal	metal
Al1C1Tb3_ICSD_43866	metal	metal
Al1C1Tb3_ICSD_606265	metal	metal
Al1C1Ti2_ICSD_606272	metal	metal
Al1C1Ti3_ICSD_163846	metal	metal
Al1C1Tm3_ICSD_43868	metal	metal
Al1C1V2_ICSD_606283	metal	metal
Al1C1W2_ICSD_165101	metal	metal
Al1C1Y3_ICSD_43869	metal	metal
Al1C1Zr2_ICSD_157761	metal	metal
Al1C2Ta3_ICSD_249399	metal	metal
Al1C2Ti3_ICSD_182475	metal	metal
Al1C3Nb4_ICSD_160755	metal	metal
Al1C3Ta4_ICSD_159456	metal	metal
Al1C3V4_ICSD_163549	metal	metal
Al1C8H24P1Sn2_ICSD_163054	insulator	insulator
Al1Ca1F14Mg3Na3_ICSD_168054	insulator	insulator
Al1Ca1F5_ICSD_171399	insulator	insulator
Al1Ca1F5_ICSD_69563	insulator	insulator

Supplementary Table 31. Five-fold cross validated predictions for the metal/insulator classification (23/598).

system	calculated	predicted
Al1Ca1F6Li1_ICSD_150332	insulator	insulator
Al1Ca1F6Na1_ICSD_80542	insulator	insulator
Al1Ca1H1O5Si1_ICSD_12127	insulator	insulator
Al1Ca1H1Si1_ICSD_162867	insulator	insulator
Al1Ca1H5_ICSD_156313	insulator	insulator
Al1Ca1H5_ICSD_156314	insulator	insulator
Al1Ca1H5_ICSD_172034	insulator	insulator
Al1Ca1O5Ta1_ICSD_50718	insulator	insulator
Al1Ca1O5Ta1_ICSD_99001	insulator	insulator
Al1Ca1Pd1_ICSD_370036	metal	metal
Al1Ca1Pt1_ICSD_603309	metal	metal
Al1Ca1Si1_ICSD_155192	metal	metal
Al1Ca1Si1_ICSD_155193	metal	metal
Al1Ca1Si1_ICSD_155853	metal	metal
Al1Ca2Cl1F2H8O12S2_ICSD_80437	insulator	insulator
Al1Ca2F7H2O1_ICSD_182268	insulator	insulator
Al1Ca2F7_ICSD_100308	insulator	insulator
Al1Ca2F9Pb1_ICSD_180336	insulator	insulator
Al1Ca2Na5O16P4_ICSD_400310	insulator	insulator
Al1Ca3N3_ICSD_410579	insulator	insulator
Al1Ca3Sb3_ICSD_36363	insulator	insulator
Al1Cd1Ce3S7_ICSD_606335	insulator	insulator
Al1Cd1F6Na1_ICSD_80559	insulator	insulator
Al1Cd1La3S7_ICSD_606339	insulator	insulator
Al1Ce1Co1_ICSD_20632	metal	metal
Al1Ce1Cu1_ICSD_606422	metal	metal
Al1Ce1H2Ir1_ICSD_261890	metal	metal
Al1Ce1H6_ICSD_247039	metal	insulator
Al1Ce1H6_ICSD_247040	insulator	metal
Al1Ce1Ir1_ICSD_261889	metal	metal
Al1Ce1Ni1_ICSD_57583	metal	metal
Al1Ce1O3_ICSD_245264	insulator	insulator
Al1Ce1O3_ICSD_245267	insulator	insulator
Al1Ce1O3_ICSD_245562	insulator	insulator
Al1Ce1O3_ICSD_245565	metal	insulator
Al1Ce1Pd1_ICSD_156904	metal	metal
Al1Ce1Pd1_ICSD_603264	metal	metal
Al1Ce1Pd2_ICSD_604242	metal	metal
Al1Ce1Pt1_ICSD_150172	metal	metal
Al1Ce1Rh1_ICSD_160052	metal	metal
Al1Ce1Ru1_ICSD_160051	metal	metal
Al1Ce1Si2_ICSD_82674	metal	metal
Al1Ce1_ICSD_181163	metal	metal
Al1Ce1_ICSD_57551	metal	metal
Al1Ce2Ru1_ICSD_422577	metal	metal
Al1Ce3Cr1S7_ICSD_606421	insulator	insulator
Al1Ce3Fe1S7_ICSD_606457	metal	insulator
Al1Ce3Mg1S7_ICSD_606475	insulator	insulator
Al1Ce3N1_ICSD_57581	metal	metal
Al1Ce3Ni1S7_ICSD_606499	metal	insulator

Supplementary Table 32. Five-fold cross validated predictions for the metal/insulator classification (24/598).

system	calculated	predicted
Al1Ce3S7Ti1_ICSD_606505	metal	insulator
Al1Ce3S7V1_ICSD_606506	insulator	insulator
Al1Ce3S7Zn1_ICSD_606507	insulator	insulator
Al1Ce3_ICSD_108787	metal	metal
Al1Ce3_ICSD_108788	metal	metal
Al1Ce3_ICSD_57554	metal	metal
Al1Ce3_ICSD_606349	metal	metal
Al1Ce3_ICSD_606369	metal	metal
Al1Cl1H6O4_ICSD_425880	insulator	insulator
Al1Cl1O1_ICSD_27812	insulator	insulator
Al1Cl3H12O6_ICSD_22071	insulator	insulator
Al1Cl3_ICSD_155670	metal	insulator
Al1Cl3_ICSD_39566	insulator	insulator
Al1Cl4Cs1_ICSD_8118	insulator	insulator
Al1Cl4Cu1_ICSD_165607	insulator	insulator
Al1Cl4Cu1_ICSD_35050	insulator	insulator
Al1Cl4Ga1_ICSD_62232	insulator	insulator
Al1Cl4In1_ICSD_170790	insulator	insulator
Al1Cl4Li1_ICSD_35277	insulator	insulator
Al1Cl4N1S2_ICSD_27210	insulator	insulator
Al1Cl4Na1_ICSD_71158	insulator	insulator
Al1Cl4Tl1_ICSD_419828	insulator	insulator
Al1Cl6I1_ICSD_26510	insulator	insulator
Al1Cl6I3_ICSD_26403	insulator	insulator
Al1Cl6N1S2_ICSD_14181	insulator	insulator
Al1Cl7S1_ICSD_35685	insulator	insulator
Al1Cl7Se1_ICSD_9064	insulator	insulator
Al1Cl7Te1_ICSD_26043	insulator	insulator
Al1Cl7Te1_ICSD_59133	insulator	insulator
Al1Cl8Nb1_ICSD_62029	insulator	insulator
Al1Co1Dy3S7_ICSD_606550	insulator	insulator
Al1Co1La3S7_ICSD_606601	insulator	insulator
Al1Co1Mn2_ICSD_160703	metal	metal
Al1Co1Pr3S7_ICSD_606645	insulator	insulator
Al1Co1Pu1_ICSD_606646	metal	metal
Al1Co1S7Y3_ICSD_606649	insulator	insulator
Al1Co1Th1_ICSD_606677	metal	metal
Al1Co1Ti2_ICSD_185879	metal	metal
Al1Co1U1_ICSD_606697	metal	metal
Al1Co1_ICSD_57596	metal	metal
Al1Co1_ICSD_606535	metal	metal
Al1Co2Cr1_ICSD_57600	metal	metal
Al1Co2Dy2_ICSD_107402	metal	metal
Al1Co2Fe1_ICSD_57607	metal	metal
Al1Co2Gd2_ICSD_412543	metal	metal
Al1Co2Hf1_ICSD_110809	metal	metal
Al1Co2Mn1_ICSD_606611	metal	metal
Al1Co2Nb1_ICSD_57620	metal	metal
Al1Co2Nd2_ICSD_107401	metal	metal
Al1Co2Pr2_ICSD_412545	metal	metal

Supplementary Table 33. Five-fold cross validated predictions for the metal/insulator classification (25/598).

system	calculated	predicted
Al1Co2Si2_ICSD_43353	metal	metal
Al1Co2Ta1_ICSD_606667	metal	metal
Al1Co2Tb2_ICSD_107403	metal	metal
Al1Co2Ti1_ICSD_606682	metal	metal
Al1Co2U2_ICSD_57641	metal	metal
Al1Co2V1_ICSD_606705	metal	metal
Al1Co2Zr1_ICSD_606738	metal	metal
Al1Co3_ICSD_187962	metal	metal
Al1Co3_ICSD_187992	metal	metal
Al1Cr1Cu2_ICSD_57653	metal	metal
Al1Cr1Fe2_ICSD_57654	metal	metal
Al1Cr1La3S7_ICSD_606794	insulator	insulator
Al1Cr1Ni2_ICSD_57662	metal	metal
Al1Cr1Ti2_ICSD_185876	metal	metal
Al1Cr2_ICSD_606746	metal	metal
Al1Cs1Cu1F6_ICSD_240292	insulator	insulator
Al1Cs1F4_ICSD_10012	insulator	insulator
Al1Cs1H8N4_ICSD_2537	insulator	insulator
Al1Cs1H8N4_ICSD_40169	metal	insulator
Al1Cs1O2_ICSD_28372	insulator	insulator
Al1Cs1O4Si1_ICSD_160822	insulator	insulator
Al1Cs1O4Si1_ICSD_186607	insulator	insulator
Al1Cs1O4Si1_ICSD_186610	insulator	insulator
Al1Cs1Te3_ICSD_300180	insulator	insulator
Al1Cs2F6Na1_ICSD_41801	insulator	insulator
Al1Cs3Ge2O7_ICSD_412140	insulator	insulator
Al1Cs6K3Sb4_ICSD_300152	insulator	insulator
Al1Cs6Sb3_ICSD_300128	insulator	insulator
Al1Cu1Dy1_ICSD_606896	metal	metal
Al1Cu1Er1_ICSD_184971	metal	metal
Al1Cu1F6K1_ICSD_59003	insulator	insulator
Al1Cu1Gd1_ICSD_602087	metal	metal
Al1Cu1Ho1_ICSD_184970	metal	metal
Al1Cu1Nd1_ICSD_607037	metal	metal
Al1Cu1O2_ICSD_32630	insulator	insulator
Al1Cu1O2_ICSD_95661	insulator	insulator
Al1Cu1Pr1_ICSD_607054	metal	metal
Al1Cu1Pr1_ICSD_607067	metal	metal
Al1Cu1Pt2_ICSD_107788	metal	metal
Al1Cu1S2_ICSD_165739	metal	insulator
Al1Cu1S2_ICSD_187058	insulator	insulator
Al1Cu1Se2_ICSD_165741	metal	insulator
Al1Cu1Se2_ICSD_603539	insulator	insulator
Al1Cu1Tb1_ICSD_607130	metal	metal
Al1Cu1Te2_ICSD_165743	metal	metal
Al1Cu1Te2_ICSD_28735	insulator	insulator
Al1Cu1Ti2_ICSD_185881	metal	metal
Al1Cu1Y1_ICSD_607186	metal	metal
Al1Cu1Yb1_ICSD_607190	metal	metal
Al1Cu1_ICSD_653749	metal	metal

Supplementary Table 34. Five-fold cross validated predictions for the metal/insulator classification (26/598).

system	calculated	predicted
Al1Cu2Hf1_ICSD_185283	metal	metal
Al1Cu2Mn1_ICSD_607009	metal	metal
Al1Cu2Re2_ICSD_57706	metal	metal
Al1Cu2Sc1_ICSD_57709	metal	metal
Al1Cu2Ti1_ICSD_656061	metal	metal
Al1Cu2Zr1_ICSD_607205	metal	metal
Al1Cu3U2_ICSD_23256	metal	metal
Al1Cu3_ICSD_150823	metal	metal
Al1Cu3_ICSD_151216	metal	metal
Al1Cu3_ICSD_185891	metal	metal
Al1Dy1Ge1_ICSD_607304	metal	metal
Al1Dy1Ni1_ICSD_107417	metal	metal
Al1Dy1Pd1_ICSD_607333	metal	metal
Al1Dy1Pt1_ICSD_607335	metal	metal
Al1Dy1Zn1_ICSD_607343	metal	metal
Al1Dy1_ICSD_57735	metal	metal
Al1Dy1_ICSD_57736	metal	metal
Al1Dy2Ge2_ICSD_172061	metal	metal
Al1Dy2Ni2_ICSD_607323	metal	metal
Al1Dy2_ICSD_607222	metal	metal
Al1Dy3Fe1S7_ICSD_607296	insulator	insulator
Al1Dy3N1_ICSD_607314	metal	metal
Al1Dy3Ni1S7_ICSD_607332	insulator	insulator
Al1Dy3Ni8_ICSD_607316	metal	metal
Al1Er1Ge1_ICSD_607405	metal	metal
Al1Er1Ni1_ICSD_607431	metal	metal
Al1Er1Pd1_ICSD_603267	metal	metal
Al1Er1Pd1_ICSD_607432	metal	metal
Al1Er1Pt1_ICSD_607433	metal	metal
Al1Er1Zn1_ICSD_607444	metal	metal
Al1Er1_ICSD_607346	metal	metal
Al1Er2Ni2_ICSD_607424	metal	metal
Al1Er2Si2_ICSD_92452	metal	metal
Al1Er2_ICSD_607355	metal	metal
Al1Er3N1_ICSD_607415	metal	metal
Al1Er3Ni8_ICSD_607417	metal	metal
Al1Eu1_ICSD_107525	metal	metal
Al1F16Rb2Tb3_ICSD_98487	metal	insulator
Al1F1Na1O4P1_ICSD_40522	insulator	insulator
Al1F1O4Sr3_ICSD_50736	insulator	insulator
Al1F2Na5O8P2_ICSD_62645	insulator	insulator
Al1F3H2O2Pb1_ICSD_79740	insulator	insulator
Al1F3K2O4S1_ICSD_161272	insulator	insulator
Al1F3_ICSD_130021	insulator	insulator
Al1F3_ICSD_202681	insulator	insulator
Al1F3_ICSD_29131	insulator	insulator
Al1F3_ICSD_36034	insulator	insulator
Al1F3_ICSD_72174	insulator	insulator
Al1F3_ICSD_79816	insulator	insulator
Al1F4H4N1_ICSD_33539	insulator	insulator

Supplementary Table 35. Five-fold cross validated predictions for the metal/insulator classification (27/598).

system	calculated	predicted
Al1F4K1_ICSD_16413	insulator	insulator
Al1F4K1_ICSD_166825	insulator	insulator
Al1F4K1_ICSD_285	insulator	insulator
Al1F4K1_ICSD_60525	insulator	insulator
Al1F4Na1_ICSD_166361	insulator	insulator
Al1F4Na1_ICSD_20572	insulator	insulator
Al1F4Rb1_ICSD_54120	insulator	insulator
Al1F4Rb1_ICSD_54123	insulator	insulator
Al1F4Rb1_ICSD_77914	insulator	insulator
Al1F4Tl1_ICSD_200637	insulator	insulator
Al1F4Tl1_ICSD_202455	insulator	insulator
Al1F5Fe1_ICSD_78012	insulator	insulator
Al1F5H10N2O1_ICSD_201652	insulator	insulator
Al1F5H2K2O1_ICSD_81863	insulator	insulator
Al1F5H4Mg1O2_ICSD_411650	insulator	insulator
Al1F5K2_ICSD_81864	insulator	insulator
Al1F5Mn1_ICSD_73812	insulator	insulator
Al1F5Mn1_ICSD_9912	insulator	insulator
Al1F5Tl2_ICSD_25616	insulator	insulator
Al1F6H12N3_ICSD_96591	insulator	insulator
Al1F6H18N6Ru1_ICSD_91763	insulator	insulator
Al1F6H6K1O2_ICSD_69434	insulator	insulator
Al1F6H8N2Na1_ICSD_249157	insulator	insulator
Al1F6K2Li1_ICSD_27672	insulator	insulator
Al1F6K2Li1_ICSD_408553	insulator	insulator
Al1F6K2Li1_ICSD_48149	insulator	insulator
Al1F6K2Na1_ICSD_164216	insulator	insulator
Al1F6K2Na1_ICSD_34201	insulator	insulator
Al1F6K3_ICSD_262078	insulator	insulator
Al1F6Li1Na2_ICSD_280906	insulator	insulator
Al1F6Li1Pd1_ICSD_73132	insulator	insulator
Al1F6Li1Sr1_ICSD_164563	insulator	insulator
Al1F6Li1Sr1_ICSD_68905	insulator	insulator
Al1F6Li1Yb1_ICSD_411131	insulator	insulator
Al1F6Li3_ICSD_34672	insulator	insulator
Al1F6Li3_ICSD_85171	insulator	insulator
Al1F6Na1Rb2_ICSD_290318	insulator	insulator
Al1F6Na3_ICSD_30201	insulator	insulator
Al1F6Na3_ICSD_74210	insulator	insulator
Al1F6Pd1Rb1_ICSD_78749	insulator	insulator
Al1F7Mg1Na2_ICSD_33509	insulator	insulator
Al1F7Mg1Na2_ICSD_33510	insulator	insulator
Al1F7Mg1Na2_ICSD_33511	insulator	insulator
Al1F7Na2Ni1_ICSD_72289	insulator	insulator
Al1F7Na2Zn1_ICSD_400729	insulator	insulator
Al1Fe1La3S7_ICSD_607565	insulator	insulator
Al1Fe1O3_ICSD_203203	insulator	insulator
Al1Fe1O5P1_ICSD_74760	insulator	insulator
Al1Fe1S7Tb3_ICSD_607622	insulator	insulator
Al1Fe1S7Y3_ICSD_607623	metal	insulator

Supplementary Table 36. Five-fold cross validated predictions for the metal/insulator classification (28/598).

system	calculated	predicted
Al1Fe1Ti2_ICSD_185878	metal	metal
Al1Fe1U1_ICSD_607706	metal	metal
Al1Fe1_ICSD_165165	metal	metal
Al1Fe1_ICSD_169548	metal	metal
Al1Fe1_ICSD_169549	metal	metal
Al1Fe2Mn1_ICSD_57806	metal	metal
Al1Fe2Mo1_ICSD_57807	metal	metal
Al1Fe2Ni1_ICSD_57808	metal	metal
Al1Fe2O4_ICSD_76977	metal	metal
Al1Fe2Ti1_ICSD_57827	metal	metal
Al1Fe2V1_ICSD_57832	insulator	metal
Al1Fe2_ICSD_57792	metal	metal
Al1Fe3_ICSD_607485	metal	metal
Al1Ga1Yb1_ICSD_607814	metal	metal
Al1Gd1Ge1_ICSD_607864	metal	metal
Al1Gd1Ni1_ICSD_607908	metal	metal
Al1Gd1O3_ICSD_59848	insulator	insulator
Al1Gd1Pd1_ICSD_240059	metal	metal
Al1Gd1Pd1_ICSD_240061	metal	metal
Al1Gd1Zn1_ICSD_607941	metal	metal
Al1Gd1_ICSD_150537	metal	metal
Al1Gd1_ICSD_607823	metal	metal
Al1Gd2Ge2_ICSD_95801	metal	metal
Al1Gd2_ICSD_607837	metal	metal
Al1Gd3Mg1S7_ICSD_607881	insulator	insulator
Al1Gd3Mn1S7_ICSD_607887	insulator	insulator
Al1Gd3N1_ICSD_607889	metal	metal
Al1Gd3S7Ti1_ICSD_607920	metal	insulator
Al1Gd3S7V1_ICSD_607921	insulator	insulator
Al1Gd3S7Zn1_ICSD_607922	insulator	insulator
Al1Ge1Ho1_ICSD_607952	metal	metal
Al1Ge1La1_ICSD_105149	metal	metal
Al1Ge1Li1O4_ICSD_67238	insulator	insulator
Al1Ge1Li1_ICSD_152087	insulator	metal
Al1Ge1Li3O5_ICSD_72098	insulator	insulator
Al1Ge1Mn1_ICSD_607974	metal	metal
Al1Ge1Na1_ICSD_10147	metal	metal
Al1Ge1O5Y1_ICSD_32744	insulator	insulator
Al1Ge1Pr1_ICSD_90160	metal	metal
Al1Ge1Sc1_ICSD_608009	metal	metal
Al1Ge1Sr1_ICSD_166379	metal	metal
Al1Ge1Tb1_ICSD_608017	metal	metal
Al1Ge1Y1_ICSD_76295	metal	metal
Al1Ge2Nd1O7_ICSD_35144	insulator	insulator
Al1Ge3Tb2_ICSD_152747	metal	metal
Al1Ge3Y2_ICSD_78969	metal	metal
Al1Ge3Yb2_ICSD_423345	metal	metal
Al1H0.67Ni1Zr1_ICSD_186405	metal	metal
Al1H12N3O15_ICSD_96764	insulator	insulator
Al1H12N3O15_ICSD_96765	insulator	insulator

Supplementary Table 37. Five-fold cross validated predictions for the metal/insulator classification (29/598).

system	calculated	predicted
Al1H1Li1O5P1_ICSD_68921	metal	insulator
Al1H1O10Si4_ICSD_33924	insulator	insulator
Al1H1O2_ICSD_166340	insulator	insulator
Al1H1O2_ICSD_169040	metal	metal
Al1H1O2_ICSD_173074	insulator	insulator
Al1H1O2_ICSD_173075	metal	metal
Al1H1O2_ICSD_173076	insulator	insulator
Al1H1O2_ICSD_173078	metal	metal
Al1H1O2_ICSD_59609	insulator	insulator
Al1H1O4Si1_ICSD_85555	insulator	insulator
Al1H1Si1Sr1_ICSD_162868	insulator	insulator
Al1H24Li1Mg10_ICSD_158274	insulator	insulator
Al1H2Li1O5Si1_ICSD_161497	insulator	insulator
Al1H2Na3O9P2_ICSD_84643	insulator	insulator
Al1H3O3_ICSD_164050	insulator	insulator
Al1H3O3_ICSD_181006	insulator	insulator
Al1H3O3_ICSD_181008	insulator	insulator
Al1H3O3_ICSD_240781	insulator	insulator
Al1H3O3_ICSD_26830	insulator	insulator
Al1H3_ICSD_15225	insulator	insulator
Al1H3_ICSD_182534	insulator	insulator
Al1H3_ICSD_182535	insulator	insulator
Al1H3_ICSD_182536	insulator	insulator
Al1H4K1O14P4_ICSD_63033	insulator	insulator
Al1H4K1_ICSD_99082	insulator	insulator
Al1H4K1_ICSD_99083	insulator	insulator
Al1H4Li1_ICSD_22247	insulator	insulator
Al1H4Na1_ICSD_165838	insulator	insulator
Al1H4Na1_ICSD_99257	insulator	insulator
Al1H4Th2_ICSD_43313	metal	metal
Al1H5Mg1_ICSD_165987	insulator	insulator
Al1H5O9P2_ICSD_34613	insulator	insulator
Al1H5Sr1_ICSD_156315	insulator	insulator
Al1H6I1O4_ICSD_424556	insulator	insulator
Al1H6K2Li1_ICSD_152890	insulator	insulator
Al1H6K2Li1_ICSD_245318	insulator	insulator
Al1H6K3_ICSD_152889	metal	insulator
Al1H6K3_ICSD_153683	insulator	insulator
Al1H6K3_ICSD_153684	insulator	insulator
Al1H6La1_ICSD_247038	insulator	insulator
Al1H6Li1Mg1_ICSD_165986	insulator	insulator
Al1H6Li1Na2_ICSD_152893	insulator	insulator
Al1H6Li3_ICSD_99217	insulator	insulator
Al1H6Na3_ICSD_153680	metal	insulator
Al1H6Na3_ICSD_154909	metal	insulator
Al1H6Nd1_ICSD_247044	insulator	metal
Al1H6Pr1_ICSD_247041	metal	insulator
Al1H6Pr1_ICSD_247042	insulator	metal
Al1H8K1N4_ICSD_2538	insulator	insulator
Al1H8N4Rb1_ICSD_40168	insulator	insulator

Supplementary Table 38. Five-fold cross validated predictions for the metal/insulator classification (30/598).

system	calculated	predicted
Al1Hf1Ni1_ICSD_608130	metal	metal
Al1Hf1Ni2_ICSD_608123	metal	metal
Al1Hf1Pd2_ICSD_57906	metal	metal
Al1Hf1Pt1_ICSD_261008	metal	metal
Al1Hf1Pt1_ICSD_608141	metal	metal
Al1Hf1Pt2_ICSD_608138	metal	metal
Al1Hf1_ICSD_107828	metal	metal
Al1Hf2_ICSD_608076	metal	metal
Al1Hf3N1_ICSD_602284	metal	metal
Al1Ho1Ni1_ICSD_107223	metal	metal
Al1Ho1Pd1_ICSD_608213	metal	metal
Al1Ho1Pt1_ICSD_608215	metal	metal
Al1Ho1_ICSD_608166	metal	metal
Al1Ho2Ni2_ICSD_608205	metal	metal
Al1Ho2Si2_ICSD_92451	metal	metal
Al1Ho2_ICSD_57910	metal	metal
Al1Ho3N1_ICSD_608196	metal	metal
Al1Ho3Ni8_ICSD_608198	metal	metal
Al1I2Pd5_ICSD_14164	metal	metal
Al1I3O9_ICSD_152758	insulator	insulator
Al1I3_ICSD_391247	insulator	insulator
Al1I4In1_ICSD_418802	insulator	insulator
Al1I4Na1_ICSD_400521	insulator	insulator
Al1I4Tl1_ICSD_419827	insulator	insulator
Al1I7Te1_ICSD_401395	insulator	insulator
Al1I8P1_ICSD_35403	insulator	insulator
Al1In1S3_ICSD_8257	insulator	insulator
Al1Ir1Th1_ICSD_608247	metal	metal
Al1Ir1U1_ICSD_608249	metal	metal
Al1Ir1U1_ICSD_608251	metal	metal
Al1Ir1_ICSD_57928	metal	metal
Al1K1Mo2O8_ICSD_28018	insulator	insulator
Al1K1O2_ICSD_151883	insulator	insulator
Al1K1O2_ICSD_169481	insulator	insulator
Al1K1O2_ICSD_262975	insulator	insulator
Al1K1O4Si1_ICSD_34350	insulator	insulator
Al1K1O4Si1_ICSD_83449	insulator	insulator
Al1K1O7P2_ICSD_2888	insulator	insulator
Al1K1O8S2_ICSD_6305	insulator	insulator
Al1K1O8Si3_ICSD_83536	insulator	insulator
Al1K1Sb4_ICSD_300157	insulator	insulator
Al1K1Te2_ICSD_411171	insulator	insulator
Al1K1Te2_ICSD_44703	insulator	insulator
Al1K2Li1P2_ICSD_77275	insulator	insulator
Al1K2Na1P2_ICSD_73279	insulator	insulator
Al1K6Na3Sb4_ICSD_401209	insulator	insulator
Al1La1O3_ICSD_153821	insulator	insulator
Al1La1O3_ICSD_153830	insulator	insulator
Al1La1O3_ICSD_153836	insulator	metal
Al1La1O3_ICSD_180416	insulator	insulator

Supplementary Table 39. Five-fold cross validated predictions for the metal/insulator classification (31/598).

system	calculated	predicted
Al1La1O3_ICSD_180417	insulator	insulator
Al1La1O3_ICSD_90549	insulator	insulator
Al1La1Pt1_ICSD_603295	metal	metal
Al1La1Si2_ICSD_94502	metal	metal
Al1La1_ICSD_54470	metal	metal
Al1La3Mg1S7_ICSD_608298	insulator	insulator
Al1La3Mn1S7_ICSD_608303	insulator	insulator
Al1La3N1_ICSD_185959	metal	metal
Al1La3Ni1S7_ICSD_608317	insulator	insulator
Al1La3S7Ti1_ICSD_608322	insulator	insulator
Al1La3S7V1_ICSD_608323	insulator	insulator
Al1La3S7Zn1_ICSD_608324	insulator	insulator
Al1La3_ICSD_57932	metal	metal
Al1La3_ICSD_603210	metal	metal
Al1La6Mg22_ICSD_154679	metal	metal
Al1Li1Mo2O8_ICSD_16175	insulator	insulator
Al1Li1Na2P2_ICSD_402083	insulator	insulator
Al1Li1O12P4_ICSD_74860	insulator	insulator
Al1Li1O2_ICSD_23815	insulator	insulator
Al1Li1O2_ICSD_28288	insulator	insulator
Al1Li1O4Si1_ICSD_22011	insulator	insulator
Al1Li1O4Si1_ICSD_22014	insulator	insulator
Al1Li1O4Si1_ICSD_32595	insulator	insulator
Al1Li1O4Si1_ICSD_38167	insulator	insulator
Al1Li1O4Si1_ICSD_97909	insulator	insulator
Al1Li1O6Si2_ICSD_158512	insulator	insulator
Al1Li1O6Si2_ICSD_30521	insulator	insulator
Al1Li1S2_ICSD_608360	insulator	insulator
Al1Li1Se2_ICSD_280225	insulator	insulator
Al1Li1Si1_ICSD_413257	insulator	insulator
Al1Li1Te2_ICSD_162672	insulator	insulator
Al1Li1_ICSD_240114	metal	metal
Al1Li1_ICSD_240125	metal	metal
Al1Li1_ICSD_262064	metal	metal
Al1Li1_ICSD_262069	metal	metal
Al1Li2Pd1_ICSD_105504	metal	metal
Al1Li2Pt1_ICSD_105506	metal	metal
Al1Li2Rh1_ICSD_105508	metal	metal
Al1Li3N2_ICSD_25565	insulator	insulator
Al1Li5O4_ICSD_1037	insulator	insulator
Al1Li5O4_ICSD_42697	insulator	insulator
Al1Lu1Ni1_ICSD_608382	metal	metal
Al1Lu1Pd1_ICSD_603268	metal	metal
Al1Lu1Pt1_ICSD_608394	metal	metal
Al1Lu1Si1_ICSD_415373	metal	metal
Al1Lu2Si2_ICSD_92454	metal	metal
Al1Lu3N1_ICSD_608380	metal	metal
Al1Lu3Ni8_ICSD_608381	metal	metal
Al1Mg1O5P1_ICSD_156822	insulator	insulator
Al1Mg1Si1_ICSD_153549	metal	metal

Supplementary Table 40. Five-fold cross validated predictions for the metal/insulator classification (32/598).

system	calculated	predicted
Al1Mg1Y1_ICSD_160908	metal	metal
Al1Mg3Pt2_ICSD_105510	metal	metal
Al1Mg4Si6_ICSD_54781	metal	metal
Al1Mn1Ni2_ICSD_57976	metal	metal
Al1Mn1Pd2_ICSD_57981	metal	metal
Al1Mn1Pr3S7_ICSD_608498	insulator	insulator
Al1Mn1Pt1_ICSD_57984	metal	metal
Al1Mn1Pt2_ICSD_57985	metal	metal
Al1Mn1Rh2_ICSD_57986	metal	metal
Al1Mn1Ti2_ICSD_185877	insulator	metal
Al1Mn1_ICSD_57969	metal	metal
Al1Mn1_ICSD_608471	metal	metal
Al1Mn2V1_ICSD_57994	metal	metal
Al1Mn3_ICSD_181801	metal	metal
Al1Mo1O7V1_ICSD_280775	insulator	insulator
Al1Mo2Na1O8_ICSD_281210	insulator	insulator
Al1Mo2O8Tl1_ICSD_250339	insulator	insulator
Al1Mo3_ICSD_57999	metal	metal
Al1Mo4S8_ICSD_36561	metal	metal
Al1Mo4S8_ICSD_36564	insulator	metal
Al1N1Na3O9P3_ICSD_75366	insulator	insulator
Al1N1Nd2O3_ICSD_201358	metal	insulator
Al1N1Nd3_ICSD_52638	metal	metal
Al1N1Pr3_ICSD_52639	metal	metal
Al1N1Tb3_ICSD_608634	metal	metal
Al1N1Ti2_ICSD_157766	metal	metal
Al1N1Ti3_ICSD_52642	metal	metal
Al1N1Zr2_ICSD_157763	metal	metal
Al1N1Zr3_ICSD_29521	metal	metal
Al1N1_ICSD_105522	insulator	insulator
Al1N1_ICSD_163950	insulator	insulator
Al1N1_ICSD_163951	insulator	insulator
Al1N1_ICSD_188512	insulator	insulator
Al1N1_ICSD_31169	insulator	insulator
Al1N3Nb4_ICSD_181352	metal	metal
Al1N3Ta4_ICSD_181353	metal	metal
Al1N3Ti4_ICSD_91772	metal	metal
Al1N3V4_ICSD_181348	metal	metal
Al1Na1O2_ICSD_22216	insulator	insulator
Al1Na1O2_ICSD_79404	insulator	insulator
Al1Na1O4Si1_ICSD_34884	insulator	insulator
Al1Na1O4Si1_ICSD_36324	insulator	insulator
Al1Na1O6Si2_ICSD_162546	insulator	insulator
Al1Na1O7P2_ICSD_400462	insulator	insulator
Al1Na1O8Si3_ICSD_77425	metal	insulator
Al1Na1Se2_ICSD_300173	insulator	insulator
Al1Na1Se2_ICSD_44704	insulator	insulator
Al1Na1Si1_ICSD_10146	metal	metal
Al1Na1Te2_ICSD_44701	insulator	insulator
Al1Na3P2_ICSD_402081	insulator	insulator

Supplementary Table 41. Five-fold cross validated predictions for the metal/insulator classification (33/598).

system	calculated	predicted
Al1Nb1Ni2_ICSD_608701	metal	metal
Al1Nb1O4_ICSD_24078	insulator	insulator
Al1Nb1Ru2_ICSD_105524	metal	metal
Al1Nb2_ICSD_601188	metal	metal
Al1Nb3_ICSD_608655	metal	metal
Al1Nd1Ni1_ICSD_608770	metal	metal
Al1Nd1O3_ICSD_35550	metal	metal
Al1Nd1O3_ICSD_90573	insulator	insulator
Al1Nd1Pd1_ICSD_603266	metal	metal
Al1Nd1Pd2_ICSD_604244	metal	metal
Al1Nd1Pt1_ICSD_150174	metal	metal
Al1Nd1_ICSD_181160	metal	metal
Al1Nd2Ni2_ICSD_608769	metal	metal
Al1Nd3Ni8_ICSD_608765	metal	metal
Al1Ni0.33Ru0.67U1_ICSD_164389	metal	metal
Al1Ni1Pr1_ICSD_608820	metal	metal
Al1Ni1Pr3S7_ICSD_608833	insulator	insulator
Al1Ni1Pu1_ICSD_608834	metal	metal
Al1Ni1S7Y3_ICSD_608836	insulator	insulator
Al1Ni1Tb1_ICSD_105534	metal	metal
Al1Ni1Th1_ICSD_608897	metal	metal
Al1Ni1Ti2_ICSD_185880	metal	metal
Al1Ni1U1_ICSD_608935	metal	metal
Al1Ni1Y1_ICSD_608947	metal	metal
Al1Ni1Yb1_ICSD_608960	metal	metal
Al1Ni1Zr1_ICSD_608978	metal	metal
Al1Ni1_ICSD_608805	metal	metal
Al1Ni2Pr2_ICSD_608823	metal	metal
Al1Ni2Sc1_ICSD_58050	metal	metal
Al1Ni2Ta1_ICSD_58055	metal	metal
Al1Ni2Tb2_ICSD_608886	metal	metal
Al1Ni2Ti1_ICSD_165902	metal	metal
Al1Ni2V1_ICSD_58071	metal	metal
Al1Ni2Y2_ICSD_160936	metal	metal
Al1Ni2Zr1_ICSD_608975	metal	metal
Al1Ni2Zr4_ICSD_164058	metal	metal
Al1Ni3_ICSD_107862	metal	metal
Al1Ni3_ICSD_604410	metal	metal
Al1Ni3_ICSD_608800	metal	metal
Al1Ni4U1_ICSD_58069	metal	metal
Al1Ni4Zr5_ICSD_415704	metal	metal
Al1Ni8Tb3_ICSD_608879	metal	metal
Al1Ni8Y3_ICSD_160938	metal	metal
Al1O1_ICSD_28920	metal	insulator
Al1O2Rb1_ICSD_28373	insulator	insulator
Al1O2Tl1_ICSD_29010	insulator	insulator
Al1O3Pr1_ICSD_35549	metal	metal
Al1O3Pr1_ICSD_90566	insulator	insulator
Al1O3Y1_ICSD_27100	insulator	insulator
Al1O3Y1_ICSD_4115	insulator	insulator

Supplementary Table 42. Five-fold cross validated predictions for the metal/insulator classification (34/598).

system	calculated	predicted
Al1O4P1_ICSD_158619	insulator	insulator
Al1O4P1_ICSD_159272	insulator	insulator
Al1O4P1_ICSD_159273	insulator	insulator
Al1O4P1_ICSD_162670	insulator	insulator
Al1O4P1_ICSD_24511	insulator	insulator
Al1O4P1_ICSD_261306	insulator	insulator
Al1O4P1_ICSD_279582	insulator	insulator
Al1O4P1_ICSD_417475	insulator	insulator
Al1O4P1_ICSD_50101	insulator	insulator
Al1O4P1_ICSD_66996	insulator	insulator
Al1O4P1_ICSD_66998	insulator	insulator
Al1O4P1_ICSD_66999	insulator	insulator
Al1O4P1_ICSD_9643	insulator	insulator
Al1O4P1_ICSD_98384	insulator	insulator
Al1O4Rb1Si1_ICSD_160823	insulator	insulator
Al1O4Rb1Si1_ICSD_4335	insulator	insulator
Al1O4Si1Tl1_ICSD_89722	insulator	insulator
Al1O4Ta1_ICSD_33885	insulator	insulator
Al1O4Ta1_ICSD_67676	insulator	insulator
Al1O4V2_ICSD_151457	metal	metal
Al1O4V2_ICSD_60413	metal	metal
Al1O4W1_ICSD_4164	insulator	insulator
Al1O9P3_ICSD_26759	insulator	insulator
Al1Os1_ICSD_609006	metal	metal
Al1P1Pt5_ICSD_609024	metal	metal
Al1P1S4_ICSD_15910	insulator	insulator
Al1P1S4_ICSD_56821	metal	insulator
Al1P1_ICSD_67770	insulator	insulator
Al1P1_ICSD_67783	insulator	insulator
Al1Pd1Pr1_ICSD_603265	metal	metal
Al1Pd1Sm1_ICSD_609058	metal	metal
Al1Pd1Tb1_ICSD_160951	metal	metal
Al1Pd1Tb1_ICSD_609059	metal	metal
Al1Pd1Th1_ICSD_609060	metal	metal
Al1Pd1Y1_ICSD_156923	metal	metal
Al1Pd1Y1_ICSD_609063	metal	metal
Al1Pd1Yb1_ICSD_370028	metal	metal
Al1Pd1_ICSD_58112	metal	metal
Al1Pd1_ICSD_58113	metal	metal
Al1Pd1_ICSD_58114	metal	metal
Al1Pd2Pr1_ICSD_604243	metal	metal
Al1Pd2Sc1_ICSD_58120	metal	metal
Al1Pd2Zr1_ICSD_58122	metal	metal
Al1Pd2_ICSD_58115	metal	metal
Al1Pd5_ICSD_245328	metal	metal
Al1Pr1Pt1_ICSD_150173	metal	metal
Al1Pr1Si2_ICSD_152802	metal	metal
Al1Pr1_ICSD_108897	metal	metal
Al1Pr1_ICSD_181159	metal	metal
Al1Pr1_ICSD_58123	metal	metal

Supplementary Table 43. Five-fold cross validated predictions for the metal/insulator classification (35/598).

system	calculated	predicted
Al1Pr3_ICSD_58124	metal	metal
Al1Pr3_ICSD_609101	metal	metal
Al1Pr3_ICSD_657240	metal	metal
Al1Pt1Sm1_ICSD_609157	metal	metal
Al1Pt1Tb1_ICSD_609159	metal	metal
Al1Pt1Th1_ICSD_609160	metal	metal
Al1Pt1Ti1_ICSD_163350	metal	metal
Al1Pt1U1_ICSD_609163	metal	metal
Al1Pt1Y1_ICSD_609165	metal	metal
Al1Pt1Yb1_ICSD_604063	metal	metal
Al1Pt1Zr1_ICSD_156266	metal	metal
Al1Pt1Zr1_ICSD_609169	metal	metal
Al1Pt1_ICSD_58128	metal	metal
Al1Pt1_ICSD_609146	metal	metal
Al1Pt2Zr1_ICSD_609167	metal	metal
Al1Pt2_ICSD_459	metal	metal
Al1Pt2_ICSD_58130	metal	metal
Al1Pt3_ICSD_107439	metal	metal
Al1Pt3_ICSD_609128	metal	metal
Al1Rb6Sb3_ICSD_300217	insulator	insulator
Al1Re1_ICSD_58146	metal	metal
Al1Re2_ICSD_58147	metal	metal
Al1Rh1Th1_ICSD_609217	metal	metal
Al1Rh1U1_ICSD_106246	metal	metal
Al1Rh1U1_ICSD_656982	metal	metal
Al1Rh1_ICSD_609212	metal	metal
Al1Ru1U1_ICSD_609243	metal	metal
Al1Ru1_ICSD_609238	metal	metal
Al1Sb1_ICSD_41994	metal	metal
Al1Sb1_ICSD_609290	insulator	metal
Al1Sb9Yb11_ICSD_186084	insulator	metal
Al1Sc1_ICSD_609299	metal	metal
Al1Sc1_ICSD_609303	metal	metal
Al1Sc2Si2_ICSD_52653	metal	metal
Al1Sc2_ICSD_183218	metal	metal
Al1Se2Tl1_ICSD_100130	insulator	insulator
Al1Si1Sm1_ICSD_151717	metal	metal
Al1Si1Sr1_ICSD_162865	metal	metal
Al1Si1Te3_ICSD_75001	insulator	insulator
Al1Si1Tm1_ICSD_415372	metal	metal
Al1Si1Y1_ICSD_99138	metal	metal
Al1Si2Tm2_ICSD_92453	metal	metal
Al1Si2Y2_ICSD_92450	metal	metal
Al1Si2Yb2_ICSD_92455	metal	metal
Al1Sm1_ICSD_58104	metal	metal
Al1Sm1_ICSD_609374	metal	metal
Al1Sm3_ICSD_58106	metal	metal
Al1Sn1_ICSD_107883	metal	metal
Al1Sn3Zr5_ICSD_106250	metal	metal
Al1Sr1_ICSD_344	metal	metal

Supplementary Table 44. Five-fold cross validated predictions for the metal/insulator classification (36/598).

system	calculated	predicted
Al1Ta2_ICSD_107888	metal	metal
Al1Ta2_ICSD_609429	metal	metal
Al1Tb1_ICSD_181161	metal	metal
Al1Tb1_ICSD_609450	metal	metal
Al1Tb2_ICSD_609457	metal	metal
Al1Tb3_ICSD_58173	metal	metal
Al1Tc2_ICSD_609480	metal	metal
Al1Th1_ICSD_609503	metal	metal
Al1Th2_ICSD_609491	metal	metal
Al1Ti1Zr2_ICSD_185119	metal	metal
Al1Ti1_ICSD_290012	metal	metal
Al1Ti1_ICSD_58187	metal	metal
Al1Ti2V1_ICSD_185875	metal	metal
Al1Ti2Zn1_ICSD_185882	metal	metal
Al1Ti3_ICSD_189695	metal	metal
Al1Ti3_ICSD_99779	metal	metal
Al1Tm1Zn1_ICSD_609569	metal	metal
Al1V3_ICSD_609619	metal	metal
Al1Y1Zn1_ICSD_609667	metal	metal
Al1Y1_ICSD_181158	metal	metal
Al1Y1_ICSD_58209	metal	metal
Al1Y2_ICSD_58211	metal	metal
Al1Y3_ICSD_58212	metal	metal
Al1Zr1_ICSD_58227	metal	metal
Al1Zr2_ICSD_150774	metal	metal
Al1Zr2_ICSD_609694	metal	metal
Al1Zr3_ICSD_609742	metal	metal
Al1_ICSD_187080	metal	metal
Al1_ICSD_240129	metal	metal
Al2.3Sr2.7_ICSD_609406	metal	metal
Al20Ca1Cr2_ICSD_606307	metal	metal
Al20Ce1Co2_ICSD_57562	metal	metal
Al20Ce1Cr2_ICSD_107751	metal	metal
Al20Ce1Ti2_ICSD_236257	metal	metal
Al20Ce1V2_ICSD_236262	metal	metal
Al20Cr2Er1_ICSD_606768	metal	metal
Al20Cr2Ho1_ICSD_606789	metal	metal
Al20Cr2La1_ICSD_236265	metal	metal
Al20Cr2Nd1_ICSD_606816	metal	metal
Al20Cr2Pr1_ICSD_236267	metal	metal
Al20Cr2Sm1_ICSD_236268	metal	metal
Al20Cr2Y1_ICSD_606867	metal	metal
Al20Cr2Yb1_ICSD_236269	metal	metal
Al20Eu1V2_ICSD_105131	metal	metal
Al20Gd1V2_ICSD_157333	metal	metal
Al20La1Ti2_ICSD_236256	metal	metal
Al20La1V2_ICSD_236261	metal	metal
Al20Pr1Ti2_ICSD_236258	metal	metal
Al20Pr1V2_ICSD_236263	metal	metal
Al20Sm1V2_ICSD_236264	metal	metal

Supplementary Table 45. Five-fold cross validated predictions for the metal/insulator classification (37/598).

system	calculated	predicted
Al20Ti2Yb1_ICSD_236260	metal	metal
Al20U1V2_ICSD_107251	metal	metal
Al21Pd8_ICSD_58119	metal	metal
Al21Pt8_ICSD_58136	metal	metal
Al22Na2O34_ICSD_34905	insulator	insulator
Al23Ce4Ni6_ICSD_240163	metal	metal
Al23Ni6Y4_ICSD_160933	metal	metal
Al23Ni6Yb4_ICSD_402094	metal	metal
Al23V4_ICSD_174002	metal	metal
Al24Ce3Cu8Mn1_ICSD_20315	metal	metal
Al2As2Cs2O7_ICSD_154363	insulator	insulator
Al2As3K3_ICSD_60950	insulator	insulator
Al2As4Ca3_ICSD_60161	insulator	insulator
Al2As4Sr3_ICSD_423787	insulator	insulator
Al2Au1_ICSD_606031	metal	metal
Al2Au2Ce1_ICSD_658114	metal	metal
Al2Au2Dy1_ICSD_658121	metal	metal
Al2Au2Gd1_ICSD_658119	metal	metal
Al2Au2La1_ICSD_658113	metal	metal
Al2Au2Nd1_ICSD_658116	metal	metal
Al2Au2Pr1_ICSD_658115	metal	metal
Al2Au2Sm1_ICSD_658117	metal	metal
Al2Au2Sr1_ICSD_658124	metal	metal
Al2Au2Tb1_ICSD_658120	metal	metal
Al2Au2Th1_ICSD_658122	metal	metal
Al2Au2U1_ICSD_658123	metal	metal
Al2B1Ce1Ru2_ICSD_186682	metal	metal
Al2B1La1Ru2_ICSD_174342	metal	metal
Al2B1Re3_ICSD_43842	metal	metal
Al2B2Ba1O7_ICSD_409171	insulator	insulator
Al2B2Ca1O7_ICSD_86785	insulator	insulator
Al2B2Cs2O7_ICSD_423471	insulator	insulator
Al2B2K2O7_ICSD_409420	insulator	insulator
Al2B2Na2O7_ICSD_93389	insulator	insulator
Al2B2O7Sr1_ICSD_89423	insulator	insulator
Al2B2O7Sr1_ICSD_91031	insulator	insulator
Al2B2Ru3_ICSD_43843	metal	metal
Al2Ba1Ge2O8_ICSD_1282	insulator	insulator
Al2Ba1Ge2_ICSD_98514	metal	metal
Al2Ba1Ge2_ICSD_98515	metal	metal
Al2Ba1O4_ICSD_21080	insulator	insulator
Al2Ba1O4_ICSD_246027	insulator	insulator
Al2Ba1O7Sb2_ICSD_154362	insulator	insulator
Al2Ba1S4_ICSD_35136	insulator	insulator
Al2Ba1Si2_ICSD_153384	metal	metal
Al2Ba1Si2_ICSD_153385	metal	metal
Al2Ba1Si2_ICSD_249561	metal	metal
Al2Ba1Te4_ICSD_41165	insulator	insulator
Al2Ba1_ICSD_57512	metal	metal
Al2Ba2Ca1F14Mg1_ICSD_20393	insulator	insulator

Supplementary Table 46. Five-fold cross validated predictions for the metal/insulator classification (38/598).

system	calculated	predicted
Al2Ba2Ga1.33_ICSD_415931	metal	metal
Al2Ba3F12_ICSD_413546	insulator	insulator
Al2Ba3N4_ICSD_410578	insulator	insulator
Al2Ba3O12Si3_ICSD_27386	insulator	insulator
Al2Ba5Ge7_ICSD_417659	metal	metal
Al2Ba7O19Sc6_ICSD_39442	insulator	insulator
Al2Be1O4_ICSD_34806	insulator	insulator
Al2Be2Cl2Na8O24Si8_ICSD_34665	insulator	insulator
Al2Be3O18Si6_ICSD_202091	insulator	insulator
Al2Bi2Br12_ICSD_414262	insulator	insulator
Al2Bi2Cl12_ICSD_414261	insulator	insulator
Al2Bi6Ca5_ICSD_36364	metal	metal
Al2Br6N2S2_ICSD_38378	insulator	insulator
Al2Br6N2Se2_ICSD_82802	insulator	insulator
Al2Br6_ICSD_83433	insulator	insulator
Al2Br6_ICSD_83434	insulator	insulator
Al2Br7K1_ICSD_2592	insulator	insulator
Al2Br8Ti1_ICSD_39243	insulator	insulator
Al2Br8Ti1_ICSD_40904	insulator	insulator
Al2C1Co1Ta3_ICSD_418908	metal	metal
Al2C1Ni1Ti3_ICSD_43862	metal	metal
Al2C2Mg1_ICSD_85739	insulator	insulator
Al2C3Th2_ICSD_81572	metal	metal
Al2C6H22O2Si2_ICSD_172440	insulator	insulator
Al2Ca1Cl8_ICSD_56730	insulator	insulator
Al2Ca1Ga2_ICSD_300209	metal	metal
Al2Ca1Ge2_ICSD_606320	metal	metal
Al2Ca1H4O10Si2_ICSD_80837	insulator	insulator
Al2Ca1H6O13Si3_ICSD_30967	insulator	insulator
Al2Ca1H8_ICSD_246482	insulator	insulator
Al2Ca1O4_ICSD_172780	insulator	insulator
Al2Ca1O4_ICSD_41661	insulator	insulator
Al2Ca1S4_ICSD_46016	insulator	insulator
Al2Ca1Si2_ICSD_20278	metal	metal
Al2Ca1Zn2_ICSD_57550	metal	metal
Al2Ca1_ICSD_418966	metal	metal
Al2Ca2Fe1H1O13Si3_ICSD_63661	insulator	insulator
Al2Ca2H1Mn1O13Si3_ICSD_26354	insulator	insulator
Al2Ca2O7Si1_ICSD_24588	insulator	insulator
Al2Ca2O9Sn2_ICSD_260890	insulator	insulator
Al2Ca3F14Na2_ICSD_202657	insulator	insulator
Al2Ca3F8H6O8S1_ICSD_31248	insulator	insulator
Al2Ca3Ge2_ICSD_52616	metal	metal
Al2Ca3Ge3_ICSD_31982	metal	metal
Al2Ca3H12O12_ICSD_34227	insulator	insulator
Al2Ca3N4_ICSD_280347	insulator	insulator
Al2Ca3N4_ICSD_280348	insulator	insulator
Al2Ca3N4_ICSD_280349	insulator	insulator
Al2Ca3O12Si3_ICSD_94614	insulator	insulator
Al2Ca3O6_ICSD_151369	metal	insulator

Supplementary Table 47. Five-fold cross validated predictions for the metal/insulator classification (39/598).

system	calculated	predicted
Al2Ca3Si2_ICSD_100126	metal	metal
Al2Ca5Sb6_ICSD_183853	insulator	insulator
Al2Cd1O4_ICSD_183382	insulator	insulator
Al2Cd1S4_ICSD_25634	insulator	insulator
Al2Cd1S4_ICSD_43025	insulator	insulator
Al2Cd1Se4_ICSD_174192	insulator	insulator
Al2Cd1Se4_ICSD_51423	insulator	insulator
Al2Cd1Te4_ICSD_25640	insulator	insulator
Al2Cd2Cl8_ICSD_59173	metal	insulator
Al2Cd3O12Si3_ICSD_27384	insulator	insulator
Al2Ce1Ga2_ICSD_55789	metal	metal
Al2Ce1Ge2_ICSD_604134	metal	metal
Al2Ce1Pd2_ICSD_658138	metal	metal
Al2Ce1Pt1_ICSD_658140	metal	metal
Al2Ce1Pt3_ICSD_658142	metal	metal
Al2Ce1Zn2_ICSD_57594	metal	metal
Al2Ce1_ICSD_57555	metal	metal
Al2Ce1_ICSD_606350	metal	metal
Al2Ce3I2_ICSD_409723	metal	metal
Al2Cl10N2P2_ICSD_391165	insulator	insulator
Al2Cl1F25Sr10_ICSD_202936	insulator	insulator
Al2Cl1H6Li1O6_ICSD_83509	insulator	insulator
Al2Cl2O5Sr3_ICSD_68365	insulator	insulator
Al2Cl6O1Sn1_ICSD_411777	insulator	insulator
Al2Cl8Co1_ICSD_22143	insulator	insulator
Al2Cl8Cu1_ICSD_80107	insulator	insulator
Al2Cl8Mg1_ICSD_62046	insulator	insulator
Al2Cl8Ni1_ICSD_417872	insulator	insulator
Al2Cl8Pd1_ICSD_15595	insulator	insulator
Al2Cl8Te4_ICSD_10322	insulator	insulator
Al2Cl8Ti1_ICSD_35154	metal	insulator
Al2Cl8Ti1_ICSD_39565	insulator	insulator
Al2Cl8V1_ICSD_415951	insulator	insulator
Al2Co1H10Zr6_ICSD_88069	metal	metal
Al2Co1O4_ICSD_290133	insulator	metal
Al2Co1Y1_ICSD_57645	metal	metal
Al2Co1Zr6_ICSD_150575	metal	metal
Al2Co3O12Si3_ICSD_27383	insulator	insulator
Al2Cs2O7Sb2_ICSD_154361	insulator	insulator
Al2Cs2O9P2_ICSD_280275	insulator	insulator
Al2Cs6Te6_ICSD_300181	insulator	insulator
Al2Cu1Ir1_ICSD_167666	metal	metal
Al2Cu1Mg1_ICSD_415062	metal	metal
Al2Cu1O4_ICSD_24491	metal	metal
Al2Cu1U1_ICSD_23257	metal	metal
Al2Cu1Yb1_ICSD_604213	metal	metal
Al2Cu1_ICSD_151384	metal	metal
Al2Cu1_ICSD_172800	metal	metal
Al2Cu9Nd1_ICSD_607036	metal	metal
Al2Dy1Ni1_ICSD_57759	metal	metal

Supplementary Table 48. Five-fold cross validated predictions for the metal/insulator classification (40/598).

system	calculated	predicted
Al2Dy1Si2_ICSD_89553	metal	metal
Al2Dy1_ICSD_607229	metal	metal
Al2Dy3H9Ni6_ICSD_657447	metal	metal
Al2Dy3Ni6_ICSD_607315	metal	metal
Al2Dy3_ICSD_150549	metal	metal
Al2Er1Ge2_ICSD_160311	metal	metal
Al2Er1Ni1_ICSD_57778	metal	metal
Al2Er1_ICSD_607354	metal	metal
Al2Er3H7Ni6_ICSD_657449	metal	metal
Al2Er3Ni6_ICSD_607416	metal	metal
Al2Er3_ICSD_607352	metal	metal
Al2Eu1Ge2_ICSD_607458	metal	metal
Al2Eu1S4_ICSD_607464	insulator	insulator
Al2Eu1Se4_ICSD_607466	insulator	insulator
Al2Eu1Si2_ICSD_411055	metal	metal
Al2Eu1Si2_ICSD_67353	metal	metal
Al2Eu1_ICSD_659102	metal	metal
Al2Eu2O7Sr1_ICSD_182157	metal	insulator
Al2F12Li3Na3_ICSD_31110	insulator	insulator
Al2F16Sr5_ICSD_411613	insulator	insulator
Al2F1K4Nb11O20_ICSD_65738	insulator	insulator
Al2F2Ge1O4_ICSD_409714	insulator	insulator
Al2F2K2O3_ICSD_421736	insulator	insulator
Al2Fe1O4_ICSD_187920	metal	insulator
Al2Fe1S4_ICSD_607619	insulator	metal
Al2Fe1Zr6_ICSD_402701	metal	metal
Al2Fe3O12Si3_ICSD_80671	insulator	insulator
Al2Fe3Si3_ICSD_422342	metal	metal
Al2Fe3Si4_ICSD_83665	metal	metal
Al2Ga2La1_ICSD_607781	metal	metal
Al2Ga2Pr1_ICSD_607795	metal	metal
Al2Ga2Yb1_ICSD_607817	metal	metal
Al2Gd1Ni1_ICSD_607899	metal	metal
Al2Gd1Si2_ICSD_41180	metal	metal
Al2Gd1_ICSD_607852	metal	metal
Al2Gd2O7Sr1_ICSD_33580	insulator	insulator
Al2Ge1H2O6_ICSD_78792	insulator	insulator
Al2Ge2La1_ICSD_604133	metal	metal
Al2Ge2Lu1_ICSD_412125	metal	metal
Al2Ge2Nd1_ICSD_412121	metal	metal
Al2Ge2O7_ICSD_201750	insulator	insulator
Al2Ge2Sr1_ICSD_608014	metal	metal
Al2Ge2Sr3_ICSD_52617	metal	metal
Al2Ge2Tb1_ICSD_412124	metal	metal
Al2Ge2Y1_ICSD_39053	metal	metal
Al2Ge2Yb1_ICSD_411056	metal	metal
Al2Ge4Sr3_ICSD_417968	metal	metal
Al2Ge8Sc11_ICSD_76361	metal	metal
Al2H10Ni1Zr6_ICSD_88070	metal	metal
Al2H12O12Sr3_ICSD_20529	insulator	insulator

Supplementary Table 49. Five-fold cross validated predictions for the metal/insulator classification (41/598).

system	calculated	predicted
Al2H12O15Se3_ICSD_72871	insulator	insulator
Al2H3O9P3_ICSD_74527	insulator	insulator
Al2H4K1Ni1O14P3_ICSD_75347	insulator	insulator
Al2H4Li2O14Si4_ICSD_88917	insulator	insulator
Al2H4Na2O12Si3_ICSD_31309	insulator	insulator
Al2H4O9Si2_ICSD_30996	insulator	insulator
Al2H4O9Si2_ICSD_63192	insulator	insulator
Al2H4O9Si2_ICSD_80083	insulator	insulator
Al2H4O9Si2_ICSD_87771	insulator	insulator
Al2H4O9Si2_ICSD_98133	insulator	insulator
Al2H8Mg1_ICSD_152538	insulator	insulator
Al2H9Ni6Y3_ICSD_657444	metal	metal
Al2Hf1_ICSD_608069	metal	metal
Al2Hf3_ICSD_60632	metal	metal
Al2Hf6Pt1_ICSD_261007	metal	metal
Al2Hg1S4_ICSD_25635	insulator	insulator
Al2Hg1S4_ICSD_608160	insulator	insulator
Al2Hg1Se4_ICSD_25638	insulator	insulator
Al2Hg1Se4_ICSD_608163	insulator	insulator
Al2Hg1Te4_ICSD_25641	insulator	insulator
Al2Ho1Ni1_ICSD_57919	metal	metal
Al2Ho1_ICSD_608178	metal	metal
Al2Ho3Ni6_ICSD_105154	metal	metal
Al2Ho3_ICSD_184632	metal	metal
Al2I12Sb2_ICSD_38253	insulator	insulator
Al2I1La2_ICSD_411690	metal	metal
Al2I2La3_ICSD_411691	metal	metal
Al2Ir1Li1_ICSD_105155	metal	metal
Al2K2O24P8_ICSD_260827	insulator	insulator
Al2K2O7Sb2_ICSD_280310	insulator	insulator
Al2K6O6_ICSD_74968	insulator	insulator
Al2K6Se6_ICSD_300172	insulator	insulator
Al2K6Te6_ICSD_300168	insulator	insulator
Al2La1Zn2_ICSD_105503	metal	metal
Al2La1_ICSD_604450	metal	metal
Al2La5Ru3_ICSD_167948	metal	metal
Al2Li1Pd1_ICSD_105505	metal	metal
Al2Li1Pt1_ICSD_105507	metal	metal
Al2Li1Rh1_ICSD_105509	metal	metal
Al2Li3_ICSD_57951	metal	metal
Al2Lu1_ICSD_608370	metal	metal
Al2Lu3_ICSD_57959	metal	metal
Al2Mg1O4_ICSD_161057	insulator	insulator
Al2Mg1O4_ICSD_182859	insulator	insulator
Al2Mg1S4_ICSD_107308	insulator	insulator
Al2Mg1S4_ICSD_79672	insulator	insulator
Al2Mg1Se4_ICSD_83363	insulator	insulator
Al2Mg1Si2_ICSD_156101	metal	metal
Al2Mg2Se5_ICSD_41928	insulator	insulator
Al2Mg3O12Si3_ICSD_24941	insulator	insulator

Supplementary Table 50. Five-fold cross validated predictions for the metal/insulator classification (42/598).

system	calculated	predicted
Al2Mn1S4_ICSD_608507	metal	insulator
Al2Mn1S4_ICSD_608509	insulator	insulator
Al2Mn1Te4_ICSD_608538	insulator	insulator
Al2Mn3O12Si3_ICSD_52396	insulator	insulator
Al2N1Nb3_ICSD_60644	metal	metal
Al2N1Ni1Ti3_ICSD_91338	metal	metal
Al2N2O3Si1Sr1_ICSD_408170	insulator	insulator
Al2N4Sr3_ICSD_74824	insulator	insulator
Al2Na6Se6_ICSD_300166	insulator	insulator
Al2Na6Te6_ICSD_300163	insulator	insulator
Al2Na7Sb5_ICSD_48168	insulator	insulator
Al2Nd1_ICSD_58027	metal	metal
Al2Ni1O1Ti3_ICSD_91339	metal	metal
Al2Ni1O4_ICSD_608815	metal	insulator
Al2Ni1Sc1_ICSD_370012	metal	metal
Al2Ni1Tb1_ICSD_608885	metal	metal
Al2Ni1Tm1_ICSD_58067	metal	metal
Al2Ni1Tm1_ICSD_608918	metal	metal
Al2Ni1Y1_ICSD_160930	metal	metal
Al2Ni1Yb1_ICSD_370014	metal	metal
Al2Ni1Zr6_ICSD_160911	metal	metal
Al2Ni3Pr1_ICSD_107864	metal	metal
Al2Ni6Tb3_ICSD_608878	metal	metal
Al2Ni6Y3_ICSD_160937	metal	metal
Al2O10Rb2Si3_ICSD_180324	insulator	insulator
Al2O12P3Rb3_ICSD_280211	insulator	insulator
Al2O12P3Tl3_ICSD_280212	insulator	insulator
Al2O12S3_ICSD_73249	insulator	insulator
Al2O12Si3Sr3_ICSD_27385	insulator	insulator
Al2O12W3_ICSD_73878	insulator	insulator
Al2O12W3_ICSD_73879	insulator	insulator
Al2O15Ti7_ICSD_202646	metal	insulator
Al2O1_ICSD_28919	metal	insulator
Al2O3_ICSD_161061	insulator	insulator
Al2O3_ICSD_161062	insulator	insulator
Al2O3_ICSD_169722	insulator	insulator
Al2O3_ICSD_173014	insulator	insulator
Al2O3_ICSD_30026	insulator	insulator
Al2O3_ICSD_82504	insulator	insulator
Al2O3_ICSD_84375	insulator	insulator
Al2O4Pb1_ICSD_33532	insulator	insulator
Al2O4Sr1_ICSD_160296	insulator	insulator
Al2O4Sr1_ICSD_160298	insulator	insulator
Al2O4Zn1_ICSD_94159	insulator	insulator
Al2O5Si1_ICSD_76936	insulator	insulator
Al2O5Si1_ICSD_85742	insulator	insulator
Al2O5Si1_ICSD_85745	insulator	insulator
Al2O5Si1_ICSD_85746	insulator	insulator
Al2O5Ti1_ICSD_27681	insulator	insulator
Al2O6Rb6_ICSD_74969	insulator	insulator

Supplementary Table 51. Five-fold cross validated predictions for the metal/insulator classification (43/598).

system	calculated	predicted
Al2O7Rb2Sb2_ICSD_154360	insulator	insulator
Al2O9Pb2Si2_ICSD_159977	insulator	insulator
Al2O9Tb4_ICSD_164882	insulator	insulator
Al2O9Y4_ICSD_51076	insulator	insulator
Al2Os1_ICSD_58108	insulator	metal
Al2P4Sr3_ICSD_409134	insulator	insulator
Al2Pb1S4_ICSD_609026	insulator	insulator
Al2Pb2Sr1_ICSD_25336	metal	metal
Al2Pd1Yb1_ICSD_658148	metal	metal
Al2Pd1_ICSD_58116	metal	metal
Al2Pd5Pu1_ICSD_166270	metal	metal
Al2Pd5U1_ICSD_161313	metal	metal
Al2Pd5Y1_ICSD_182836	metal	metal
Al2Pr1Si2_ICSD_152800	metal	metal
Al2Pr1Zn2_ICSD_106244	metal	metal
Al2Pr1_ICSD_609072	metal	metal
Al2Pr1_ICSD_609080	metal	metal
Al2Pt1_ICSD_609121	metal	metal
Al2Pu1_ICSD_58140	metal	metal
Al2Ru1_ICSD_58156	insulator	metal
Al2Ru1_ICSD_609228	metal	metal
Al2Ru1_ICSD_609234	insulator	metal
Al2S3_ICSD_609250	insulator	insulator
Al2S3_ICSD_73220	insulator	insulator
Al2S4Sr1_ICSD_609259	insulator	insulator
Al2S4Zn1_ICSD_609280	insulator	insulator
Al2S4Zn1_ICSD_76278	insulator	insulator
Al2Sb6Sr5_ICSD_62304	insulator	insulator
Al2Sb6Yb5_ICSD_409996	metal	metal
Al2Sc1_ICSD_609301	metal	metal
Al2Se3_ICSD_14373	insulator	insulator
Al2Se4Sr1_ICSD_49732	insulator	insulator
Al2Se4Zn1_ICSD_25636	insulator	insulator
Al2Se4Zn1_ICSD_609325	insulator	insulator
Al2Si2Sm1_ICSD_89551	metal	metal
Al2Si2Sr1_ICSD_419886	metal	metal
Al2Si2Sr3_ICSD_100127	metal	metal
Al2Si2Tb1_ICSD_89552	metal	metal
Al2Si2Y1_ICSD_89550	metal	metal
Al2Si2Yb1_ICSD_411054	metal	metal
Al2Sm1Zn2_ICSD_609398	metal	metal
Al2Sm1_ICSD_609382	metal	metal
Al2Sr1_ICSD_181800	metal	metal
Al2Sr1_ICSD_58166	metal	metal
Al2Sr3_ICSD_609416	metal	metal
Al2Tb1_ICSD_609469	metal	metal
Al2Tb3_ICSD_609454	metal	metal
Al2Te3_ICSD_406353	insulator	insulator
Al2Te4Zn1_ICSD_25639	insulator	insulator
Al2Te5_ICSD_78941	insulator	insulator

Supplementary Table 52. Five-fold cross validated predictions for the metal/insulator classification (44/598).

system	calculated	predicted
Al2Th1_ICSD_58181	metal	metal
Al2Th1_ICSD_58182	metal	metal
Al2Th3_ICSD_58183	metal	metal
Al2Ti1_ICSD_106252	metal	metal
Al2Tm1_ICSD_609563	metal	metal
Al2U1_ICSD_106979	metal	metal
Al2U1_ICSD_609586	metal	metal
Al2W1_ICSD_173662	metal	metal
Al2Y1_ICSD_609656	metal	metal
Al2Y3_ICSD_247100	metal	metal
Al2Yb1_ICSD_609677	metal	metal
Al2Zr1_ICSD_609730	metal	metal
Al2Zr3_ICSD_58231	metal	metal
Al30Mg23_ICSD_57965	metal	metal
Al3Au1Ce1_ICSD_658144	metal	metal
Al3Au8_ICSD_57502	metal	metal
Al3B2Ru4_ICSD_43844	metal	metal
Al3B4Gd1O12_ICSD_100831	insulator	insulator
Al3B4Nd1O12_ICSD_20800	insulator	insulator
Al3B4Nd1O12_ICSD_6175	insulator	insulator
Al3B4O12Pr1_ICSD_160892	insulator	insulator
Al3B4O12Y1_ICSD_20223	insulator	insulator
Al3B6Co20_ICSD_43833	metal	metal
Al3B6Ni20_ICSD_408417	metal	metal
Al3Ba10Ge7_ICSD_1078	metal	metal
Al3Bi5Cl12_ICSD_201993	insulator	insulator
Al3Br12La1_ICSD_72281	insulator	insulator
Al3Br12Pr1_ICSD_72282	insulator	insulator
Al3C1Zr5_ICSD_606294	metal	metal
Al3C2O5Sc3_ICSD_420953	insulator	insulator
Al3C3Co1W9_ICSD_421253	metal	metal
Al3C3Dy1_ICSD_606190	metal	metal
Al3C3Er1_ICSD_606194	metal	metal
Al3C3Gd1_ICSD_606207	metal	metal
Al3C3Ho1_ICSD_606218	metal	metal
Al3C3Sc1_ICSD_43477	insulator	insulator
Al3C3Sc1_ICSD_62308	insulator	insulator
Al3C3Tb1_ICSD_606267	metal	metal
Al3C3Tm1_ICSD_606281	insulator	metal
Al3C3U1_ICSD_43478	metal	metal
Al3C3Y1_ICSD_606289	metal	metal
Al3C4Hf2_ICSD_161588	metal	metal
Al3C4U2_ICSD_66750	metal	metal
Al3C4Zr2_ICSD_153859	metal	metal
Al3C5Hf3_ICSD_161587	metal	metal
Al3C5Zr2_ICSD_87349	metal	metal
Al3C5Zr3_ICSD_159412	metal	metal
Al3C6Zr4_ICSD_159413	metal	metal
Al3Ca2H1O13Si3_ICSD_9245	insulator	insulator
Al3Ca4Mg1_ICSD_152756	metal	metal

Supplementary Table 53. Five-fold cross validated predictions for the metal/insulator classification (45/598).

system	calculated	predicted
Al3Ca8_ICSD_165240	metal	metal
Al3Ce1Ni2_ICSD_602476	metal	metal
Al3Ce1Pd2_ICSD_603064	metal	metal
Al3Ce1_ICSD_150603	metal	metal
Al3Ce2Ge4_ICSD_50977	metal	metal
Al3Cl12Tb1_ICSD_410939	insulator	insulator
Al3Co14Ho2_ICSD_606594	metal	metal
Al3Co14Y2_ICSD_606723	metal	metal
Al3Co1Er2_ICSD_606559	metal	metal
Al3Cs2F12Na1_ICSD_646	insulator	insulator
Al3Cu1Gd1_ICSD_658383	metal	metal
Al3Cu1Nd1_ICSD_290391	metal	metal
Al3Cu1Pr1_ICSD_290390	metal	metal
Al3Cu2Gd1_ICSD_57681	metal	metal
Al3Cu2Ni3Zr12_ICSD_166652	insulator	metal
Al3Cu2_ICSD_57668	metal	metal
Al3Dy1_ICSD_57738	metal	metal
Al3Dy1_ICSD_57739	metal	metal
Al3Dy1_ICSD_607239	metal	metal
Al3Dy2Ge4_ICSD_607305	metal	metal
Al3Dy2Si2_ICSD_92449	metal	metal
Al3Er0.8Y0.2_ICSD_607439	metal	metal
Al3Er1Ni2_ICSD_55529	metal	metal
Al3Er1_ICSD_607353	metal	metal
Al3Er2Si2_ICSD_415148	metal	metal
Al3Er3Ge2Ni1_ICSD_171194	metal	metal
Al3Er5Ge4Ni3_ICSD_172068	metal	metal
Al3F12K2Na1_ICSD_40178	insulator	insulator
Al3F12Na1Rb2_ICSD_40177	insulator	insulator
Al3F14Na5_ICSD_26419	insulator	insulator
Al3F19Pb5_ICSD_203224	insulator	insulator
Al3Fe1Si2_ICSD_79710	metal	metal
Al3Fe2Si1_ICSD_422341	metal	metal
Al3Gd1O8Pb2_ICSD_404479	insulator	insulator
Al3Gd1Pd2_ICSD_105145	metal	metal
Al3Gd1_ICSD_607838	metal	metal
Al3Ge2Ni1Y3_ICSD_76297	metal	metal
Al3Ge4La2_ICSD_76359	metal	metal
Al3H14Na5_ICSD_246195	insulator	insulator
Al3H6K1O14S2_ICSD_12106	metal	insulator
Al3H6K1O14S2_ICSD_18141	insulator	insulator
Al3Hf1_ICSD_57896	metal	metal
Al3Hf1_ICSD_608082	metal	metal
Al3Hf2_ICSD_109105	metal	metal
Al3Hf4_ICSD_608091	metal	metal
Al3Hf5N1_ICSD_603995	metal	metal
Al3Hf5_ICSD_57898	metal	metal
Al3Ho1O8Pb2_ICSD_67819	insulator	insulator
Al3Ho1_ICSD_57913	metal	metal
Al3Ho1_ICSD_57914	metal	metal

Supplementary Table 54. Five-fold cross validated predictions for the metal/insulator classification (46/598).

system	calculated	predicted
Al3Ho2Si2_ICSD_415147	metal	metal
Al3I1Te3_ICSD_66030	insulator	insulator
Al3Ir1_ICSD_608235	metal	metal
Al3Ir1_ICSD_608237	metal	metal
Al3La1Pd2_ICSD_105500	metal	metal
Al3La1_ICSD_659413	metal	metal
Al3La5Ni2_ICSD_415701	metal	metal
Al3Li12Si4_ICSD_39597	metal	metal
Al3Li1_ICSD_57952	metal	metal
Al3Li8Si5_ICSD_51446	metal	metal
Al3Lu1O8Pb2_ICSD_67820	insulator	insulator
Al3Lu1_ICSD_608373	metal	metal
Al3Mo1_ICSD_105517	insulator	metal
Al3Nb1_ICSD_186000	metal	metal
Al3Nd1Ni2_ICSD_58034	metal	metal
Al3Nd1Pd2_ICSD_150136	metal	metal
Al3Nd1_ICSD_58028	metal	metal
Al3Nd1_ICSD_658317	metal	metal
Al3Ni1Y1_ICSD_160939	metal	metal
Al3Ni1_ICSD_608787	metal	metal
Al3Ni2Pr1_ICSD_58045	metal	metal
Al3Ni2Th1_ICSD_58061	metal	metal
Al3Ni2U1_ICSD_659220	metal	metal
Al3Ni2Y1_ICSD_160934	metal	metal
Al3Ni2_ICSD_608786	metal	metal
Al3Ni5_ICSD_58041	metal	metal
Al3O12Sc2Y3_ICSD_67055	insulator	insulator
Al3O27P9_ICSD_409479	insulator	insulator
Al3Os1U2_ICSD_58111	metal	metal
Al3Os2_ICSD_58109	metal	metal
Al3Pd2Sm1_ICSD_105540	metal	metal
Al3Pd2U1_ICSD_657574	metal	metal
Al3Pd2_ICSD_58117	metal	metal
Al3Pd5_ICSD_58118	metal	metal
Al3Pr1_ICSD_107867	metal	metal
Al3Pr1_ICSD_58126	metal	metal
Al3Pr1_ICSD_609096	metal	metal
Al3Pt2_ICSD_58134	metal	metal
Al3Pt2_ICSD_602213	metal	metal
Al3Pt5_ICSD_656681	metal	metal
Al3Pu1_ICSD_58141	metal	metal
Al3Pu1_ICSD_58142	metal	metal
Al3Pu1_ICSD_58143	metal	metal
Al3Pu1_ICSD_609172	metal	metal
Al3Pu1_ICSD_609187	metal	metal
Al3Pu1_ICSD_609188	metal	metal
Al3Ru1Sc2_ICSD_58159	metal	metal
Al3Ru1_ICSD_609233	metal	metal
Al3Ru2_ICSD_609226	metal	metal
Al3Ru2_ICSD_609232	metal	metal

Supplementary Table 55. Five-fold cross validated predictions for the metal/insulator classification (47/598).

system	calculated	predicted
Al3Sc1_ICSD_247449	metal	metal
Al3Sc1_ICSD_609305	metal	metal
Al3Si2Tb2_ICSD_415146	metal	metal
Al3Si2Tm2_ICSD_415150	metal	metal
Al3Si2Y2_ICSD_75228	metal	metal
Al3Ta1_ICSD_609440	metal	metal
Al3Ta5_ICSD_609439	metal	metal
Al3Tb0.8Zr0.2_ICSD_609475	metal	metal
Al3Tb1_ICSD_58175	metal	metal
Al3Tc2_ICSD_609482	metal	metal
Al3Th1_ICSD_609500	metal	metal
Al3Ti1_ICSD_167814	metal	metal
Al3Ti1_ICSD_609543	metal	metal
Al3Tm1_ICSD_58194	metal	metal
Al3U1_ICSD_658006	metal	metal
Al3V1_ICSD_167811	metal	metal
Al3V1_ICSD_167812	metal	metal
Al3Y1_ICSD_58216	metal	metal
Al3Y1_ICSD_58218	metal	metal
Al3Y1_ICSD_58220	metal	metal
Al3Y5_ICSD_603810	metal	metal
Al3Yb1_ICSD_658320	metal	metal
Al3Zr1_ICSD_609709	metal	metal
Al3Zr1_ICSD_609737	metal	metal
Al3Zr2_ICSD_609705	metal	metal
Al3Zr4_ICSD_150529	metal	metal
Al3Zr5_ICSD_58235	metal	metal
Al3Zr5_ICSD_603491	metal	metal
Al40Ba21_ICSD_170238	metal	metal
Al43Dy6Ti4_ICSD_150119	metal	metal
Al45Cr7_ICSD_57652	metal	metal
Al45V7_ICSD_58204	metal	metal
Al4As8K12_ICSD_300121	insulator	insulator
Al4Au1Er1Ge2_ICSD_415290	metal	metal
Al4B12Be4Cs1O28_ICSD_17018	metal	insulator
Al4B2Co1O10_ICSD_1975	insulator	insulator
Al4Ba1S7_ICSD_33237	insulator	insulator
Al4Ba1_ICSD_57513	metal	metal
Al4Ba7Ge9_ICSD_417660	metal	metal
Al4Be1Mg1O8_ICSD_36361	insulator	insulator
Al4Bi2O9_ICSD_26807	insulator	insulator
Al4Bi2S8_ICSD_408439	insulator	insulator
Al4Bi2Se8_ICSD_408440	insulator	insulator
Al4Bi4Cl16S4_ICSD_414154	insulator	insulator
Al4Bi4Cl16Se4_ICSD_414155	insulator	insulator
Al4Bi4Cl16Te4_ICSD_411714	insulator	insulator
Al4C1N3O1_ICSD_409682	insulator	insulator
Al4C1O4_ICSD_18204	insulator	insulator
Al4C3_ICSD_606173	insulator	metal
Al4C4Si1_ICSD_606255	metal	insulator

Supplementary Table 56. Five-fold cross validated predictions for the metal/insulator classification (48/598).

system	calculated	predicted
Al4C4Th1_ICSD_81573	metal	metal
Al4C5Hf2_ICSD_161586	metal	metal
Al4C5Zr2_ICSD_173676	metal	metal
Al4C6Hf3_ICSD_161585	metal	metal
Al4C6Zr3_ICSD_173677	metal	metal
Al4Ca1H2O12Si2_ICSD_34855	insulator	insulator
Al4Ca1O7_ICSD_16191	insulator	insulator
Al4Ca1_ICSD_151189	metal	metal
Al4Ce1Co1_ICSD_55598	metal	metal
Al4Ce1Ni1_ICSD_606496	metal	metal
Al4Ce1_ICSD_606379	metal	metal
Al4Ce3Si6_ICSD_82675	metal	metal
Al4Cl14Te4_ICSD_10323	insulator	insulator
Al4Co1La1_ICSD_9986	metal	metal
Al4Co1Nd1_ICSD_154678	metal	metal
Al4Co1Pr1_ICSD_600912	metal	metal
Al4Co1U1_ICSD_240136	metal	metal
Al4Cu2O7_ICSD_100355	metal	metal
Al4Cu9_ICSD_606890	metal	metal
Al4Dy1Ni1_ICSD_607321	metal	metal
Al4Er1Mo2_ICSD_607414	metal	metal
Al4Er1Ni1_ICSD_57779	metal	metal
Al4Eu1_ICSD_425968	metal	metal
Al4Eu1_ICSD_55427	metal	metal
Al4Eu1_ICSD_607445	metal	metal
Al4Eu1_ICSD_607452	metal	metal
Al4F9K3O8P2_ICSD_79700	insulator	insulator
Al4Fe8U1_ICSD_607702	metal	metal
Al4Gd1Mo2_ICSD_607888	metal	metal
Al4Ge2Ni1Tb2_ICSD_95799	metal	metal
Al4H1O14Ta3_ICSD_67673	insulator	insulator
Al4H3O15P3_ICSD_6193	insulator	insulator
Al4H6O18P6_ICSD_415615	insulator	insulator
Al4Ho1Ni1_ICSD_57920	metal	metal
Al4In3Sr11_ICSD_159149	metal	metal
Al4K12P8_ICSD_300130	insulator	insulator
Al4La17N33Si9_ICSD_416358	insulator	insulator
Al4La1_ICSD_57935	metal	metal
Al4La1_ICSD_57936	metal	metal
Al4Li9_ICSD_57953	metal	metal
Al4Mg2O18Si5_ICSD_86347	insulator	insulator
Al4Mg2Zn3_ICSD_180365	metal	metal
Al4Mn13O28Sb2Si2_ICSD_12137	insulator	insulator
Al4Mo1_ICSD_58000	metal	metal
Al4Mo2Yb1_ICSD_660236	metal	metal
Al4Na4P12Sr8_ICSD_409319	insulator	insulator
Al4Nd1_ICSD_608758	metal	metal
Al4Ni1Tb1_ICSD_58060	metal	metal
Al4Ni1Tm1_ICSD_58068	metal	metal
Al4Ni1Y1_ICSD_58077	metal	metal

Supplementary Table 57. Five-fold cross validated predictions for the metal/insulator classification (49/598).

system	calculated	predicted
Al4Ni3_ICSD_58042	metal	metal
Al4O15Sr6Y2_ICSD_262993	insulator	insulator
Al4O1Si6Sr10_ICSD_418387	metal	metal
Al4O7Sr1_ICSD_16751	insulator	insulator
Al4O7Sr1_ICSD_34803	insulator	insulator
Al4Pr1_ICSD_150507	metal	metal
Al4Pr1_ICSD_609088	metal	metal
Al4Pr3Si6_ICSD_152801	metal	metal
Al4Pu1_ICSD_150593	metal	metal
Al4Sm1_ICSD_609378	metal	metal
Al4Sm1_ICSD_609392	metal	metal
Al4Sn3Sr11_ICSD_159165	metal	metal
Al4Sr1_ICSD_609418	metal	metal
Al4Tb1_ICSD_609456	metal	metal
Al4Tc1_ICSD_609483	metal	metal
Al4U1_ICSD_150933	metal	metal
Al4W1_ICSD_58205	metal	metal
Al4Zr5_ICSD_167476	metal	metal
Al5.15Mg3.15_ICSD_150646	metal	metal
Al57Mn12_ICSD_14374	metal	metal
Al5B1O9_ICSD_167310	insulator	insulator
Al5Ba3_ICSD_420090	metal	metal
Al5Ba4_ICSD_1985	metal	metal
Al5Ba5Pb1_ICSD_416337	metal	metal
Al5Ba5Sn1_ICSD_416338	metal	metal
Al5Br2La4_ICSD_413191	metal	metal
Al5C3N1_ICSD_14398	insulator	insulator
Al5C3N1_ICSD_36303	insulator	insulator
Al5Ce1Ni2_ICSD_104630	metal	metal
Al5Ce1Pt3_ICSD_171199	metal	metal
Al5Co2_ICSD_30207	metal	metal
Al5Cu6Mg2_ICSD_57694	metal	metal
Al5Cu6U1_ICSD_188588	metal	metal
Al5Dy2Fe12_ICSD_604298	metal	metal
Al5Er3O12_ICSD_170147	insulator	insulator
Al5Eu3O12_ICSD_245326	insulator	insulator
Al5Gd3O12_ICSD_23849	insulator	insulator
Al5Ge8Yb7_ICSD_76360	metal	metal
Al5Ho3O12_ICSD_33603	insulator	insulator
Al5La1Ni2_ICSD_608306	metal	metal
Al5Lu3O12_ICSD_182354	insulator	insulator
Al5Lu3O12_ICSD_23846	insulator	insulator
Al5Mo1_ICSD_105519	metal	metal
Al5Mo1_ICSD_105520	metal	metal
Al5Mo1_ICSD_105521	metal	metal
Al5Na1O12Ti2_ICSD_15346	insulator	insulator
Al5Ni2Pr1_ICSD_58046	metal	metal
Al5Ni2Zr1_ICSD_58084	metal	metal
Al5O12Tb3_ICSD_33602	insulator	insulator
Al5O12Y3_ICSD_31496	insulator	insulator

Supplementary Table 58. Five-fold cross validated predictions for the metal/insulator classification (50/598).

system	calculated	predicted
Al5O12Yb3_ICSD_170159	insulator	insulator
Al5Re24_ICSD_609200	metal	metal
Al5Rh2_ICSD_58153	metal	metal
Al5W1_ICSD_58206	metal	metal
Al6Au1Dy2Si4_ICSD_281661	metal	metal
Al6Au1Si4Tb2_ICSD_281659	metal	metal
Al6Br2Ge6Li8O24_ICSD_87991	insulator	insulator
Al6Br2Ge6Na8O24_ICSD_65665	insulator	insulator
Al6Br2Na8O24Si6_ICSD_417676	insulator	insulator
Al6C3N2_ICSD_14399	metal	insulator
Al6Ca10Ge9_ICSD_417966	metal	metal
Al6Ca4O13_ICSD_16177	insulator	insulator
Al6Ca4O13_ICSD_245370	insulator	insulator
Al6Ca4O16S1_ICSD_28480	insulator	insulator
Al6Ca4O16W1_ICSD_28481	insulator	insulator
Al6Ca5O14_ICSD_1714	insulator	insulator
Al6Cl2Ge6Li8O24_ICSD_87990	insulator	insulator
Al6Cl2Ge6Na8O24_ICSD_65664	insulator	insulator
Al6Cl2K8O24Si6_ICSD_41191	insulator	insulator
Al6Cl2Li8O24Si6_ICSD_41186	insulator	insulator
Al6Cl2Na8O24Si6_ICSD_29443	insulator	insulator
Al6Cu1H16O28P4_ICSD_21062	metal	insulator
Al6Dy2Pt1Si4_ICSD_281660	metal	metal
Al6F21Na1Rb2_ICSD_68555	insulator	insulator
Al6Fe1_ICSD_607497	metal	metal
Al6Ge6I2Li8O24_ICSD_87992	insulator	insulator
Al6Ge6I2Na8O24_ICSD_65666	insulator	insulator
Al6I2Na8O24Si6_ICSD_68960	insulator	insulator
Al6Mn1_ICSD_57973	metal	metal
Al6Na8O24Si6_ICSD_56710	metal	insulator
Al6O11Sr2_ICSD_97713	insulator	insulator
Al6O16S1Sr4_ICSD_28482	insulator	insulator
Al6O16Sr4W1_ICSD_28483	insulator	insulator
Al6O1Si8Sr13_ICSD_418388	metal	metal
Al6Pt1Si4Tb2_ICSD_281658	metal	metal
Al6Re1_ICSD_58149	metal	metal
Al6Ru1_ICSD_58157	metal	metal
Al6Tc1_ICSD_58177	metal	metal
Al7Au3Ce1_ICSD_391101	metal	metal
Al7Au3Dy1_ICSD_391108	metal	metal
Al7Au3Er1_ICSD_391109	metal	metal
Al7Au3Gd1_ICSD_391102	metal	metal
Al7Au3Ho1_ICSD_391106	metal	metal
Al7Au3Lu1_ICSD_391105	metal	metal
Al7Au3Nd1_ICSD_391107	metal	metal
Al7Au3Pr1_ICSD_391104	metal	metal
Al7Au3Tb1_ICSD_391103	metal	metal
Al7Au3Yb1_ICSD_391110	metal	metal
Al7C3N3_ICSD_14400	metal	insulator
Al7Ca3Cu2_ICSD_57538	metal	metal

Supplementary Table 59. Five-fold cross validated predictions for the metal/insulator classification (51/598).

system	calculated	predicted
Al7Co1Cu2_ICSD_57603	metal	metal
Al7Cu16Dy6_ICSD_606894	metal	metal
Al7Cu16Er6_ICSD_606911	metal	metal
Al7Cu16H9Zr6_ICSD_76304	metal	metal
Al7Cu16Ho6_ICSD_606961	metal	metal
Al7Cu16Yb6_ICSD_607188	metal	metal
Al7Cu16Zr6_ICSD_656063	metal	metal
Al7Cu2Fe1_ICSD_57677	metal	metal
Al7S12Tl3_ICSD_72324	insulator	insulator
Al7Te10_ICSD_62659	insulator	insulator
Al7Th2_ICSD_58186	metal	metal
Al8Be1Mg3O16_ICSD_31227	insulator	insulator
Al8C3N4_ICSD_14401	metal	insulator
Al8Ca1Co2_ICSD_57534	metal	metal
Al8Ca1Cu4_ICSD_57539	metal	metal
Al8Ca1Fe4_ICSD_606314	metal	metal
Al8Ca1Mn4_ICSD_57545	metal	metal
Al8Ce1Cr4_ICSD_606419	metal	metal
Al8Ce1Cu4_ICSD_57566	metal	metal
Al8Ce1Fe4_ICSD_57574	metal	metal
Al8Ce1Mn4_ICSD_57579	metal	metal
Al8Co2Pr1_ICSD_600914	metal	metal
Al8Co2Sm1_ICSD_600915	metal	metal
Al8Cr4Dy1_ICSD_606767	metal	metal
Al8Cr4Er1_ICSD_606769	metal	metal
Al8Cr4Gd1_ICSD_156967	metal	metal
Al8Cr4Ho1_ICSD_606790	metal	metal
Al8Cr4La1_ICSD_606792	metal	metal
Al8Cr4Nd1_ICSD_606817	metal	metal
Al8Cr4Pr1_ICSD_606830	metal	metal
Al8Cr4Tb1_ICSD_606848	metal	metal
Al8Cr4Th1_ICSD_54964	metal	metal
Al8Cr4Th1_ICSD_606850	metal	metal
Al8Cr4Y1_ICSD_57664	metal	metal
Al8Cr4Y1_ICSD_606869	metal	metal
Al8Cr4Yb1_ICSD_606871	metal	metal
Al8Cr5_ICSD_606753	metal	metal
Al8Cu4Dy1_ICSD_606899	metal	metal
Al8Cu4Er1_ICSD_606913	metal	metal
Al8Cu4Eu1_ICSD_606928	metal	metal
Al8Cu4Gd1_ICSD_606934	metal	metal
Al8Cu4Ho1_ICSD_606964	metal	metal
Al8Cu4Lu1_ICSD_606997	metal	metal
Al8Cu4Nd1_ICSD_607039	metal	metal
Al8Cu4Pr1_ICSD_607058	metal	metal
Al8Cu4Tb1_ICSD_607133	metal	metal
Al8Cu4Th1_ICSD_607143	metal	metal
Al8Cu4U1_ICSD_57724	metal	metal
Al8Cu4Y1_ICSD_607184	metal	metal
Al8Cu4Yb1_ICSD_607191	metal	metal

Supplementary Table 60. Five-fold cross validated predictions for the metal/insulator classification (52/598).

system	calculated	predicted
Al8Dy1Fe4_ICSD_607289	metal	metal
Al8Dy1Mn4_ICSD_607312	metal	metal
Al8Er1Fe4_ICSD_607382	metal	metal
Al8Er1Mn4_ICSD_607412	metal	metal
Al8Eu1Mn4_ICSD_607460	metal	metal
Al8Fe1Mg3Si6_ICSD_27140	metal	metal
Al8Fe4Gd1_ICSD_607505	metal	metal
Al8Fe4Hf1_ICSD_607535	metal	metal
Al8Fe4Ho1_ICSD_607546	metal	metal
Al8Fe4La1_ICSD_607554	metal	metal
Al8Fe4Lu1_ICSD_607568	metal	metal
Al8Fe4Nd1_ICSD_607593	metal	metal
Al8Fe4Sc1_ICSD_164856	metal	metal
Al8Fe4Tb1_ICSD_57822	metal	metal
Al8Fe4Th1_ICSD_607671	metal	metal
Al8Fe4U1_ICSD_607699	metal	metal
Al8Fe4Y1_ICSD_607728	metal	metal
Al8Fe4Yb1_ICSD_607748	metal	metal
Al8Fe4Zr1_ICSD_607756	metal	metal
Al8Fe5Si9U6_ICSD_183358	metal	metal
Al8Fe5_ICSD_169547	metal	metal
Al8Ge3Sr14_ICSD_173215	metal	metal
Al8Ho1Mn4_ICSD_608195	metal	metal
Al8La1Mn4_ICSD_608299	metal	metal
Al8Lu1Mn4_ICSD_608379	metal	metal
Al8Mn4Nd1_ICSD_608479	metal	metal
Al8Mn4Pr1_ICSD_608494	metal	metal
Al8Mn4Sc1_ICSD_99142	metal	metal
Al8Mn4Tb1_ICSD_608537	metal	metal
Al8Mn4Th1_ICSD_608539	metal	metal
Al8Mn4U1_ICSD_105516	metal	metal
Al8Mn4U1_ICSD_608550	metal	metal
Al8Mn4U1_ICSD_608553	metal	metal
Al8Mn4Y1_ICSD_608564	metal	metal
Al8Mn4Yb1_ICSD_608570	metal	metal
Al8Mo3_ICSD_58002	insulator	metal
Al8Si38_ICSD_180298	metal	insulator
Al9Ba1Co2_ICSD_606143	metal	metal
Al9Ba1Fe2_ICSD_57518	metal	metal
Al9Ba1Ni2_ICSD_107746	metal	metal
Al9Ca1Co2_ICSD_57535	metal	metal
Al9Ca1Ni1_ICSD_63626	metal	metal
Al9Co2Eu1_ICSD_409394	metal	metal
Al9Co2Sr1_ICSD_57633	metal	metal
Al9Co2_ICSD_57598	metal	metal
Al9Co3U2_ICSD_157329	metal	metal
Al9Co3Y2_ICSD_104653	metal	metal
Al9Er1Ni3_ICSD_105031	metal	metal
Al9Fe1Mg3Si5_ICSD_96905	metal	metal
Al9Fe2H1O24Si4_ICSD_16769	insulator	insulator

Supplementary Table 61. Five-fold cross validated predictions for the metal/insulator classification (53/598).

system	calculated	predicted
Al9Fe2_ICSD_607498	metal	metal
Al9Gd2Ir3_ICSD_370034	metal	metal
Al9Ir2_ICSD_414302	metal	metal
Al9Ir2_ICSD_608238	metal	metal
Al9Mn3Si1_ICSD_76249	metal	metal
Al9Ni2Sr1_ICSD_608861	metal	metal
Al9Rh2_ICSD_414304	metal	metal
Al9Rh2_ICSD_58154	metal	metal
Al9Sr5_ICSD_62343	metal	metal
Ar1_ICSD_426923	insulator	insulator
Ar1_ICSD_77918	insulator	insulator
As1.01Ce1Se0.99_ICSD_249802	insulator	insulator
As11Rb3_ICSD_412872	insulator	insulator
As12Ce1Fe4_ICSD_610003	insulator	metal
As12Ce1Os4_ICSD_610010	metal	metal
As12Ce1Ru4_ICSD_610013	insulator	metal
As12Fe4La1_ICSD_23080	metal	metal
As12La1Os4_ICSD_610776	metal	metal
As12La1Ru4_ICSD_610778	metal	insulator
As12Nd1Os4_ICSD_611007	metal	metal
As12Ni2Re5_ICSD_35731	metal	metal
As12Os4Pr1_ICSD_611141	metal	metal
As12Os4Th1_ICSD_611145	insulator	metal
As12Pr1Ru4_ICSD_611222	metal	metal
As14Ba3_ICSD_1404	insulator	insulator
As1Au1Ba1_ICSD_420341	metal	metal
As1Au1Ca1_ICSD_404725	metal	metal
As1Au1Eu1_ICSD_43870	metal	metal
As1Au1K2S4_ICSD_85681	insulator	insulator
As1Au1Na2_ICSD_23254	insulator	insulator
As1B1Ba1O5_ICSD_404439	insulator	insulator
As1B1Ba3O3_ICSD_402682	insulator	insulator
As1B1O4_ICSD_413436	insulator	insulator
As1B1O4_ICSD_413438	insulator	insulator
As1B1O5Pb1_ICSD_404328	insulator	insulator
As1B1_ICSD_181292	insulator	insulator
As1B2P1_ICSD_181293	insulator	insulator
As1B6_ICSD_68151	insulator	metal
As1Ba1Cu1_ICSD_41705	metal	metal
As1Ba1Li1_ICSD_56445	insulator	insulator
As1Ba1O6V1_ICSD_83629	insulator	insulator
As1Ba1Pd1_ICSD_404724	metal	metal
As1Ba1Pt1_ICSD_59192	metal	metal
As1Ba2Fe1O3Sc1_ICSD_420654	metal	metal
As1Ba2_ICSD_87345	metal	metal
As1Be1Cs1O4_ICSD_74027	insulator	insulator
As1Be1Li1_ICSD_100004	insulator	insulator
As1Be1Na1_ICSD_100091	insulator	insulator
As1Bi1Ca2O6_ICSD_91475	insulator	insulator
As1Bi1Cu2O6_ICSD_88111	metal	insulator

Supplementary Table 62. Five-fold cross validated predictions for the metal/insulator classification (54/598).

system	calculated	predicted
As1Bi1Mg2O6_ICSD_73895	insulator	insulator
As1Bi1Mn1O5_ICSD_59721	insulator	insulator
As1Bi1Ni1O5_ICSD_92916	insulator	insulator
As1Bi1O4_ICSD_27199	insulator	insulator
As1Bi1O4_ICSD_30636	insulator	insulator
As1Bi1O5Pb1_ICSD_419124	insulator	insulator
As1Bi3Ni18S16_ICSD_203066	metal	metal
As1Br1Hg3O4_ICSD_411758	insulator	insulator
As1Br1Hg3S4_ICSD_280330	insulator	insulator
As1Br1Hg3Se4_ICSD_280331	insulator	insulator
As1Br3Ca3_ICSD_426	insulator	insulator
As1Br3F6S1_ICSD_202502	insulator	insulator
As1Br3F6Te1_ICSD_200689	insulator	insulator
As1Br3F6_ICSD_33811	insulator	insulator
As1Br3_ICSD_26774	insulator	insulator
As1C1Cl2F9S1_ICSD_60141	insulator	insulator
As1C1F11N2S3_ICSD_81839	insulator	insulator
As1C1F5N2O1S2_ICSD_16422	insulator	insulator
As1C1F6H5O1_ICSD_408996	insulator	insulator
As1C1F9H3N1O1_ICSD_41317	insulator	insulator
As1C1Nb2_ICSD_180617	metal	metal
As1C1V2_ICSD_43874	metal	metal
As1C1V3_ICSD_609887	metal	metal
As1C2Cs1H6Se2_ICSD_171201	insulator	insulator
As1C2F6H5O2_ICSD_407344	insulator	insulator
As1C2F6N1O2_ICSD_410737	insulator	insulator
As1C3H10I1_ICSD_171203	insulator	insulator
As1C3H6N1_ICSD_170721	insulator	insulator
As1C3N3_ICSD_35330	insulator	insulator
As1Ca1Co1H1O5_ICSD_240725	insulator	insulator
As1Ca1Cu1_ICSD_49741	insulator	metal
As1Ca1F1Fe1_ICSD_186504	metal	metal
As1Ca1F1Mg1O4_ICSD_26000	insulator	insulator
As1Ca1F1Mg1O4_ICSD_56862	insulator	insulator
As1Ca1H1Ni1O5_ICSD_202422	insulator	insulator
As1Ca1H1O5Zn1_ICSD_63285	insulator	insulator
As1Ca1Na1O4_ICSD_262123	insulator	insulator
As1Ca1Rb1_ICSD_409177	insulator	insulator
As1Ca1_ICSD_26263	metal	metal
As1Ca2Cl1O4_ICSD_26234	insulator	insulator
As1Ca2F13H4_ICSD_415156	insulator	insulator
As1Ca2I1_ICSD_65218	insulator	insulator
As1Ca2_ICSD_166865	insulator	metal
As1Ca3Cl3_ICSD_36002	insulator	insulator
As1Ca3N1_ICSD_56968	insulator	insulator
As1Ca3N1_ICSD_657356	insulator	insulator
As1Cd1Ce1O1_ICSD_88268	insulator	insulator
As1Cd1K1_ICSD_609963	insulator	insulator
As1Cd1Li1_ICSD_609965	metal	metal
As1Cd1Li1_ICSD_609966	metal	metal

Supplementary Table 63. Five-fold cross validated predictions for the metal/insulator classification (55/598).

system	calculated	predicted
As1Cd1Na1_ICSD_609968	metal	insulator
As1Cd1Pd5_ICSD_609974	metal	metal
As1Cd1Pt5_ICSD_609975	metal	metal
As1Cd1_ICSD_432	insulator	insulator
As1Cd2Cl2_ICSD_26013	insulator	insulator
As1Cd2F1O4_ICSD_202983	insulator	insulator
As1Cd3Cl3_ICSD_23306	insulator	insulator
As1Ce1F1O4_ICSD_166934	insulator	insulator
As1Ce1Fe1O1_ICSD_162819	metal	metal
As1Ce1Ni1O1_ICSD_174440	metal	metal
As1Ce1Ni1O1_ICSD_182625	metal	metal
As1Ce1O1Ru1_ICSD_188947	metal	metal
As1Ce1O4_ICSD_280982	insulator	insulator
As1Ce1Pd1_ICSD_71613	metal	metal
As1Ce1Rh1_ICSD_90869	metal	metal
As1Ce1S1_ICSD_42432	metal	metal
As1Ce1S1_ICSD_610017	metal	metal
As1Ce1_ICSD_609995	metal	metal
As1Cl13Nb1P1_ICSD_25110	insulator	insulator
As1Cl13P1Sb1_ICSD_25109	insulator	insulator
As1Cl13P1Ta1_ICSD_25111	insulator	insulator
As1Cl1Co2O4_ICSD_902	insulator	insulator
As1Cl1Cu2O4_ICSD_901	insulator	insulator
As1Cl1Hg3O4_ICSD_411757	insulator	insulator
As1Cl1Hg3S4_ICSD_280329	insulator	insulator
As1Cl1O2Pb1_ICSD_66246	insulator	insulator
As1Cl2Hg2_ICSD_39930	insulator	insulator
As1Cl3F5O1P1_ICSD_82761	insulator	insulator
As1Cl3F6S1_ICSD_60076	insulator	insulator
As1Cl3F6S3_ICSD_154804	metal	insulator
As1Cl3F6Se1_ICSD_66843	insulator	insulator
As1Cl3_ICSD_35133	insulator	insulator
As1Cl5_ICSD_412103	insulator	insulator
As1Co1Hf1_ICSD_406953	metal	metal
As1Co1La1O1_ICSD_167818	metal	metal
As1Co1Li1O4_ICSD_155305	insulator	insulator
As1Co1Mn1_ICSD_610084	metal	metal
As1Co1Nb1_ICSD_610089	metal	metal
As1Co1Nd1O1_ICSD_180774	metal	metal
As1Co1Ni1_ICSD_610094	metal	metal
As1Co1Rh1_ICSD_43896	metal	metal
As1Co1S1_ICSD_36395	insulator	insulator
As1Co1S1_ICSD_41857	metal	insulator
As1Co1S1_ICSD_41858	metal	insulator
As1Co1S1_ICSD_610107	insulator	metal
As1Co1S1_ICSD_69129	insulator	metal
As1Co1Se1_ICSD_41731	insulator	insulator
As1Co1_ICSD_43888	metal	metal
As1Co1_ICSD_610033	metal	metal
As1Co1_ICSD_610048	metal	metal

Supplementary Table 64. Five-fold cross validated predictions for the metal/insulator classification (56/598).

system	calculated	predicted
As1Co2Cr1_ICSD_180207	metal	metal
As1Co2_ICSD_610030	metal	metal
As1Cr1Ni1_ICSD_43913	metal	metal
As1Cr1Ni1_ICSD_610244	metal	metal
As1Cr1O4_ICSD_62132	insulator	insulator
As1Cr1Rh1_ICSD_43919	metal	metal
As1Cr1Rh1_ICSD_601513	metal	metal
As1Cr1Rh1_ICSD_610256	metal	metal
As1Cr1Ti1_ICSD_610275	metal	metal
As1Cr1_ICSD_107934	metal	metal
As1Cr1_ICSD_42640	metal	metal
As1Cr2_ICSD_610130	metal	metal
As1Cr2_ICSD_610131	metal	metal
As1Cr2_ICSD_610145	metal	metal
As1Cr2_ICSD_610153	metal	metal
As1Cr3N1_ICSD_25760	metal	metal
As1Cs1F4_ICSD_413041	insulator	insulator
As1Cs1F6_ICSD_408070	insulator	insulator
As1Cs1H12Mg1O10_ICSD_260150	insulator	insulator
As1Cs1O5Ti1_ICSD_280315	insulator	insulator
As1Cs1Se2_ICSD_65299	insulator	insulator
As1Cs2Li1O4_ICSD_36645	insulator	insulator
As1Cs2Na1O4_ICSD_36533	insulator	insulator
As1Cs3Ge1Se5_ICSD_415214	metal	insulator
As1Cs3O4_ICSD_412392	insulator	insulator
As1Cs3Se4_ICSD_404082	insulator	insulator
As1Cs3_ICSD_409668	metal	metal
As1Cs5P4S12_ICSD_260946	insulator	insulator
As1Cu1Eu1_ICSD_107941	metal	metal
As1Cu1F7_ICSD_413972	insulator	insulator
As1Cu1Ge1Hf1_ICSD_185859	metal	metal
As1Cu1Ge1Ti1_ICSD_185857	metal	metal
As1Cu1Ge1Zr1_ICSD_423103	metal	metal
As1Cu1H1O5Zn1_ICSD_160894	insulator	insulator
As1Cu1Hf1Si1_ICSD_185856	metal	metal
As1Cu1K2_ICSD_43936	insulator	insulator
As1Cu1Li2_ICSD_43938	metal	insulator
As1Cu1Mg1_ICSD_412296	metal	metal
As1Cu1Mn1_ICSD_423230	metal	metal
As1Cu1Mn1_ICSD_72413	metal	metal
As1Cu1Na2_ICSD_43937	insulator	insulator
As1Cu1O1Th1_ICSD_183136	metal	metal
As1Cu1O4Pb1_ICSD_61677	insulator	insulator
As1Cu1O4Tl1_ICSD_50458	insulator	insulator
As1Cu1O4Tl2_ICSD_407563	insulator	insulator
As1Cu1O4Tl2_ICSD_50456	insulator	insulator
As1Cu1S1_ICSD_23826	insulator	insulator
As1Cu1S1_ICSD_240925	insulator	insulator
As1Cu1Se1_ICSD_610356	metal	metal
As1Cu1Se2_ICSD_42884	insulator	metal

Supplementary Table 65. Five-fold cross validated predictions for the metal/insulator classification (57/598).

system	calculated	predicted
As1Cu1Si1Ti1_ICSD_185854	metal	metal
As1Cu1Si1Zr1_ICSD_185855	metal	metal
As1Cu1Sr1_ICSD_107943	insulator	metal
As1Cu1Te1_ICSD_610367	metal	metal
As1Cu1_ICSD_610308	metal	metal
As1Cu3S4_ICSD_42516	metal	metal
As1Cu3S4_ICSD_95547	insulator	metal
As1Cu3Se4_ICSD_610361	metal	metal
As1Cu3_ICSD_16840	metal	metal
As1Cu3_ICSD_655109	metal	metal
As1Cu4K1S4_ICSD_75430	insulator	insulator
As1Dy1O4_ICSD_16512	insulator	insulator
As1Dy1Pd1_ICSD_656672	insulator	insulator
As1Dy1Pt1_ICSD_610382	metal	metal
As1Dy1_ICSD_187521	metal	metal
As1Dy1_ICSD_43638	metal	metal
As1Er1O1Zn1_ICSD_420203	insulator	insulator
As1Er1Pd1_ICSD_656673	metal	insulator
As1Er1Pt1_ICSD_610395	metal	metal
As1Er1S1_ICSD_610398	metal	metal
As1Er1_ICSD_167763	metal	metal
As1Er1_ICSD_610387	metal	metal
As1Eu1Na1S4_ICSD_262583	insulator	insulator
As1Eu1O4_ICSD_409995	metal	insulator
As1Eu1Pt1_ICSD_60829	metal	metal
As1F11H1N1S1Xe1_ICSD_249654	insulator	insulator
As1F11H1N1Te1Xe1_ICSD_171447	insulator	insulator
As1F12I1_ICSD_249129	insulator	insulator
As1F1Fe1Sr1_ICSD_168774	metal	metal
As1F1Fe1Sr1_ICSD_186507	metal	metal
As1F3_ICSD_35132	insulator	insulator
As1F5H6N2_ICSD_412507	insulator	insulator
As1F5N4S4_ICSD_14083	insulator	insulator
As1F5_ICSD_65477	insulator	insulator
As1F6H2Li1O1_ICSD_59367	insulator	insulator
As1F6H3O1_ICSD_61236	insulator	insulator
As1F6H6Li1O3_ICSD_416608	insulator	insulator
As1F6I1Se6_ICSD_35351	insulator	insulator
As1F6I3_ICSD_15527	insulator	insulator
As1F6I5_ICSD_59115	insulator	insulator
As1F6In1_ICSD_417952	insulator	insulator
As1F6K1_ICSD_16663	insulator	insulator
As1F6K1_ICSD_2362	insulator	insulator
As1F6Li1_ICSD_74831	insulator	insulator
As1F6N1O2_ICSD_68906	insulator	insulator
As1F6N1S2_ICSD_62120	insulator	insulator
As1F6N2S3_ICSD_31787	insulator	insulator
As1F6N2S3_ICSD_4043	insulator	insulator
As1F6Na1_ICSD_184563	insulator	insulator
As1F6Na1_ICSD_184564	insulator	insulator

Supplementary Table 66. Five-fold cross validated predictions for the metal/insulator classification (58/598).

system	calculated	predicted
As1F6Rb1_ICSD_408069	insulator	insulator
As1F6Tl1_ICSD_417954	insulator	insulator
As1F7Kr1_ICSD_279624	insulator	insulator
As1F7Pb1_ICSD_411788	insulator	insulator
As1F7Sn1_ICSD_816	insulator	insulator
As1F8Sb1_ICSD_9920	insulator	insulator
As1F9O1S1_ICSD_10193	insulator	insulator
As1Fe1La1O1_ICSD_180435	metal	metal
As1Fe1La1O1_ICSD_180436	metal	metal
As1Fe1Li1O4_ICSD_245182	insulator	insulator
As1Fe1Li1_ICSD_166457	metal	metal
As1Fe1Li1_ICSD_168206	metal	metal
As1Fe1Li1_ICSD_187131	metal	metal
As1Fe1Li1_ICSD_187132	insulator	metal
As1Fe1Li1_ICSD_187133	metal	metal
As1Fe1Mn1_ICSD_610492	metal	metal
As1Fe1Mn1_ICSD_93239	metal	metal
As1Fe1Na1_ICSD_163143	metal	metal
As1Fe1Na1_ICSD_187136	metal	metal
As1Fe1Nb1_ICSD_610502	metal	metal
As1Fe1Nd1O1_ICSD_163546	metal	metal
As1Fe1Nd1O1_ICSD_164679	metal	metal
As1Fe1Nd1O1_ICSD_164680	metal	metal
As1Fe1Ni1_ICSD_610509	metal	metal
As1Fe1O1Pr1_ICSD_162833	metal	metal
As1Fe1O1Tb1_ICSD_422004	metal	metal
As1Fe1O3Sr2V1_ICSD_184994	metal	metal
As1Fe1O4_ICSD_73978	metal	insulator
As1Fe1S1_ICSD_15986	metal	insulator
As1Fe1S1_ICSD_15987	insulator	metal
As1Fe1S1_ICSD_185809	insulator	metal
As1Fe1S1_ICSD_43508	metal	insulator
As1Fe1Ta1_ICSD_610528	metal	metal
As1Fe1Te1_ICSD_610529	insulator	metal
As1Fe1Ti1_ICSD_610530	metal	metal
As1Fe1V1_ICSD_610532	metal	metal
As1Fe1_ICSD_42449	metal	metal
As1Fe1_ICSD_42451	metal	metal
As1Fe2Ti1_ICSD_186059	metal	metal
As1Fe2_ICSD_610470	metal	metal
As1Ga1O4_ICSD_33256	insulator	insulator
As1Ga1O4_ICSD_423937	insulator	insulator
As1Ga1Pd5_ICSD_610564	metal	metal
As1Ga1_ICSD_41992	metal	insulator
As1Ga1_ICSD_43950	metal	insulator
As1Ga1_ICSD_43951	metal	metal
As1Ga1_ICSD_610543	insulator	metal
As1Ga1_ICSD_67773	insulator	insulator
As1Ga2Rh5_ICSD_56973	metal	metal
As1Gd1O1Zn1_ICSD_420206	insulator	insulator

Supplementary Table 67. Five-fold cross validated predictions for the metal/insulator classification (59/598).

system	calculated	predicted
As1Gd1S1_ICSD_610596	metal	metal
As1Gd1_ICSD_610585	metal	metal
As1Ge1Nb1_ICSD_610611	metal	metal
As1Ge1Se1_ICSD_100828	insulator	insulator
As1Ge1_ICSD_17033	metal	metal
As1Ge1_ICSD_610598	insulator	insulator
As1H1Hg1O5Zn1_ICSD_281591	insulator	insulator
As1H1O4Pb1_ICSD_29552	insulator	insulator
As1H1O5Pb1Zn1_ICSD_98385	insulator	insulator
As1H1O5Zn2_ICSD_34868	insulator	insulator
As1H2Li1O4_ICSD_62024	insulator	insulator
As1H2Li1O5Zn1_ICSD_409396	insulator	insulator
As1H2Mn1O5_ICSD_71164	insulator	insulator
As1H36Li3N12Se4_ICSD_409539	insulator	insulator
As1H3_ICSD_24499	metal	insulator
As1H4Na1O5_ICSD_4284	insulator	insulator
As1H4Na1O6Zn1_ICSD_407316	insulator	insulator
As1H6N1O4_ICSD_66206	insulator	insulator
As1H6N1O4_ICSD_66208	insulator	insulator
As1H6N1O4_ICSD_66210	insulator	insulator
As1Hf1Os1_ICSD_610652	metal	metal
As1Hf1Ru1_ICSD_610654	metal	metal
As1Hf1Ru1_ICSD_610655	metal	metal
As1Hf1_ICSD_42915	metal	metal
As1Hf2_ICSD_610638	metal	metal
As1Hf3_ICSD_10490	metal	metal
As1Hf9Mo4_ICSD_610646	metal	metal
As1Hf9Re4_ICSD_610653	metal	metal
As1Hf9W4_ICSD_610661	metal	metal
As1Hg1K1_ICSD_10458	metal	insulator
As1Hg1Pd5_ICSD_610664	metal	metal
As1Hg1S3Tl1_ICSD_610665	metal	insulator
As1Hg3I1Se4_ICSD_280332	insulator	insulator
As1Ho1O4_ICSD_155919	insulator	insulator
As1Ho1Pd1_ICSD_71619	insulator	metal
As1Ho1Pt1_ICSD_610678	metal	metal
As1Ho1_ICSD_187364	metal	metal
As1Ho1_ICSD_610670	metal	metal
As1I1Se1_ICSD_200799	insulator	insulator
As1I3La3_ICSD_411803	metal	metal
As1I3_ICSD_56571	insulator	insulator
As1In1Pd5_ICSD_610706	metal	metal
As1In1Pt5_ICSD_610708	metal	metal
As1In1_ICSD_156105	metal	metal
As1In1_ICSD_41993	metal	metal
As1In1_ICSD_43972	metal	metal
As1In1_ICSD_610687	metal	metal
As1K1Li2_ICSD_78938	insulator	insulator
As1K1Mg1_ICSD_610753	insulator	insulator
As1K1Mo1O6_ICSD_203218	insulator	insulator

Supplementary Table 68. Five-fold cross validated predictions for the metal/insulator classification (60/598).

system	calculated	predicted
As1K1Ni1O4_ICSD_63544	insulator	insulator
As1K1O2_ICSD_413149	insulator	insulator
As1K1O5Ti1_ICSD_75322	insulator	insulator
As1K1S5Sn1_ICSD_281038	insulator	insulator
As1K1Se2_ICSD_65297	insulator	insulator
As1K1Sn1_ICSD_610765	insulator	insulator
As1K1Zn1_ICSD_10459	insulator	insulator
As1K1Zn1_ICSD_43985	insulator	insulator
As1K1_ICSD_409653	insulator	insulator
As1K3S3_ICSD_610764	insulator	insulator
As1K3Se11Ta2_ICSD_413600	insulator	insulator
As1K3Se3_ICSD_50492	insulator	insulator
As1K3_ICSD_26887	metal	insulator
As1La1Ni1O1_ICSD_246061	metal	metal
As1La1O1Ru1_ICSD_262831	metal	metal
As1La1O1Zn1_ICSD_420204	insulator	insulator
As1La1O3_ICSD_423416	insulator	insulator
As1La1O4_ICSD_415338	insulator	insulator
As1La1Pd1_ICSD_56974	metal	metal
As1La1Rh1_ICSD_95191	metal	metal
As1La1S1_ICSD_610779	insulator	metal
As1La1Te1_ICSD_280231	insulator	metal
As1La1_ICSD_106265	metal	metal
As1La1_ICSD_98427	metal	metal
As1Li1Mg1O4_ICSD_67523	insulator	insulator
As1Li1Mg1_ICSD_107954	insulator	insulator
As1Li1Mn1O4_ICSD_245181	insulator	insulator
As1Li1Mo1O6_ICSD_15035	insulator	insulator
As1Li1Mo1O6_ICSD_59822	insulator	insulator
As1Li1Mo2O9_ICSD_170039	insulator	insulator
As1Li1Ni1O4_ICSD_245184	insulator	insulator
As1Li1O3_ICSD_16617	insulator	insulator
As1Li1O3_ICSD_202862	insulator	insulator
As1Li1O4Rb2_ICSD_36644	insulator	insulator
As1Li1O5Ti1_ICSD_172582	insulator	insulator
As1Li1O5V1_ICSD_90991	insulator	insulator
As1Li1S2_ICSD_419061	insulator	insulator
As1Li1Se2_ICSD_248116	insulator	insulator
As1Li1Se2_ICSD_248118	insulator	insulator
As1Li1Zn1_ICSD_74504	insulator	insulator
As1Li1_ICSD_26472	insulator	insulator
As1Li2Na1O4_ICSD_73200	insulator	insulator
As1Li3O4_ICSD_75927	insulator	insulator
As1Li3S3_ICSD_59381	insulator	insulator
As1Li3_ICSD_26878	insulator	insulator
As1Li3_ICSD_610785	insulator	insulator
As1Lu1O4_ICSD_2506	insulator	insulator
As1Lu1Pd1_ICSD_656676	metal	metal
As1Lu1_ICSD_247648	metal	metal
As1Lu1_ICSD_43993	metal	metal

Supplementary Table 69. Five-fold cross validated predictions for the metal/insulator classification (61/598).

system	calculated	predicted
As1Mg1Na1_ICSD_610829	insulator	insulator
As1Mg1Pt5_ICSD_610830	metal	metal
As1Mn1Na1O4_ICSD_95087	insulator	insulator
As1Mn1Na1_ICSD_610878	metal	metal
As1Mn1Nd1O1_ICSD_50242	metal	metal
As1Mn1Ni1_ICSD_161716	metal	metal
As1Mn1Ni1_ICSD_610883	metal	metal
As1Mn1Ni1_ICSD_610884	metal	metal
As1Mn1O4_ICSD_165271	insulator	insulator
As1Mn1Pd1_ICSD_610906	metal	metal
As1Mn1Pd2_ICSD_107955	metal	metal
As1Mn1Rb1_ICSD_610910	metal	metal
As1Mn1Rh1_ICSD_610914	metal	metal
As1Mn1Ru1_ICSD_44004	metal	metal
As1Mn1Ru1_ICSD_601489	metal	metal
As1Mn1Ti1_ICSD_610927	metal	metal
As1Mn1_ICSD_16543	metal	metal
As1Mn1_ICSD_184925	metal	metal
As1Mn1_ICSD_250011	metal	metal
As1Mn1_ICSD_76408	metal	metal
As1Mn2_ICSD_610849	metal	metal
As1Mn3_ICSD_76409	metal	metal
As1Mo1O6Rb1_ICSD_280174	insulator	insulator
As1Mo1_ICSD_610957	metal	metal
As1N1V3_ICSD_610970	metal	metal
As1N2U2_ICSD_41947	metal	metal
As1Na1Ni1O4_ICSD_63353	insulator	insulator
As1Na1O2_ICSD_413148	insulator	insulator
As1Na1O3_ICSD_16654	insulator	insulator
As1Na1O5Ti1_ICSD_421302	insulator	insulator
As1Na1S2_ICSD_854	insulator	insulator
As1Na1Sr1_ICSD_402448	insulator	insulator
As1Na1Zn1_ICSD_610979	insulator	insulator
As1Na1_ICSD_182158	insulator	insulator
As1Na1_ICSD_182160	metal	metal
As1Na3S3_ICSD_645	insulator	insulator
As1Na3Se3_ICSD_50491	insulator	insulator
As1Na3_ICSD_182163	metal	insulator
As1Na3_ICSD_26883	metal	metal
As1Na3_ICSD_81565	metal	metal
As1Nb1Ni1_ICSD_610993	metal	metal
As1Nb1Si1_ICSD_187693	metal	metal
As1Nb1_ICSD_27040	metal	metal
As1Nb2Rb3Se11_ICSD_413598	insulator	insulator
As1Nb3Te3_ICSD_79934	metal	metal
As1Nb3_ICSD_1998	metal	metal
As1Nd1O1Ru1_ICSD_262832	metal	metal
As1Nd1O1Zn1_ICSD_85778	insulator	insulator
As1Nd1O4_ICSD_155918	insulator	insulator
As1Nd1Pd1_ICSD_71614	metal	metal

Supplementary Table 70. Five-fold cross validated predictions for the metal/insulator classification (62/598).

system	calculated	predicted
As1Nd1Pd1_ICSD_71615	metal	metal
As1Nd1S1_ICSD_611011	metal	metal
As1Nd1_ICSD_610998	metal	metal
As1Ni1Pd1_ICSD_611044	metal	metal
As1Ni1Rh1_ICSD_601484	metal	metal
As1Ni1S1_ICSD_93899	metal	metal
As1Ni1Se1_ICSD_93901	metal	metal
As1Ni1Ta1_ICSD_611079	metal	metal
As1Ni1Ti1_ICSD_611086	metal	metal
As1Ni1V1_ICSD_611097	metal	metal
As1Ni1_ICSD_611033	metal	metal
As1Ni1_ICSD_655881	metal	metal
As1Ni2Si1_ICSD_83753	metal	metal
As1O1Y1Zn1_ICSD_420205	insulator	insulator
As1O2Rb1_ICSD_413150	insulator	insulator
As1O3Sb1_ICSD_37187	insulator	insulator
As1O4P1_ICSD_31879	insulator	insulator
As1O4Sb1_ICSD_23316	insulator	insulator
As1O4Sc1_ICSD_155920	insulator	insulator
As1O4Tb1_ICSD_16329	insulator	insulator
As1O4Tb1_ICSD_200231	insulator	insulator
As1O4Tl1Zn1_ICSD_74812	insulator	insulator
As1O4Tl3_ICSD_407561	insulator	insulator
As1O4Y1_ICSD_24513	insulator	insulator
As1O4Yb1_ICSD_171193	metal	metal
As1O5P1_ICSD_36649	insulator	insulator
As1O5Rb1Sn1_ICSD_80977	insulator	insulator
As1O5Rb1Ti1_ICSD_280131	insulator	insulator
As1O5Sb1_ICSD_36650	insulator	insulator
As1Os1Zr1_ICSD_611146	metal	metal
As1P1U1_ICSD_611155	metal	metal
As1Pa1_ICSD_44041	metal	metal
As1Pb2Pd3_ICSD_106267	metal	metal
As1Pb3Zr5_ICSD_604204	metal	metal
As1Pd1S1_ICSD_611197	metal	metal
As1Pd1Se1_ICSD_611203	metal	metal
As1Pd1Sm1_ICSD_71616	metal	metal
As1Pd1Sr1_ICSD_404723	metal	metal
As1Pd1Tb1_ICSD_656671	insulator	metal
As1Pd1Yb1_ICSD_71620	metal	metal
As1Pd1Zr1_ICSD_92440	metal	metal
As1Pd2_ICSD_26279	metal	metal
As1Pd2_ICSD_611190	metal	metal
As1Pd3_ICSD_611183	metal	metal
As1Pd5Sb1_ICSD_30009	metal	metal
As1Pd5Tl1_ICSD_23540	metal	metal
As1Pd5_ICSD_44042	metal	metal
As1Pr1S1_ICSD_611223	metal	metal
As1Pr1_ICSD_52010	metal	metal
As1Pt1Sr1_ICSD_59187	metal	metal

Supplementary Table 71. Five-fold cross validated predictions for the metal/insulator classification (63/598).

system	calculated	predicted
As1Pt1Y1_ICSD_44047	metal	metal
As1Pt1Yb1_ICSD_611243	metal	metal
As1Pt5Tl1_ICSD_611241	metal	metal
As1Pt5Zn1_ICSD_611244	metal	metal
As1Pu1_ICSD_44048	metal	metal
As1Pu1_ICSD_44049	metal	metal
As1Rb1_ICSD_412594	insulator	insulator
As1Rb3Se16_ICSD_405959	insulator	insulator
As1Rb3Se4_ICSD_404080	insulator	insulator
As1Rb3_ICSD_25551	metal	insulator
As1Rh1Ti1_ICSD_601549	metal	metal
As1Rh1V1_ICSD_107965	metal	metal
As1Rh1_ICSD_42572	metal	metal
As1Rh2_ICSD_43511	metal	metal
As1Rh2_ICSD_611266	metal	metal
As1Ru1Te1_ICSD_611299	insulator	insulator
As1Ru1Ti1_ICSD_611300	metal	metal
As1Ru1Zr1_ICSD_35593	metal	metal
As1Ru1_ICSD_611293	metal	metal
As1S1Sm1_ICSD_611320	metal	metal
As1S1Sm1_ICSD_96227	metal	metal
As1S1Th1_ICSD_611325	metal	metal
As1S1Tm1_ICSD_611336	metal	metal
As1S1U1_ICSD_66945	metal	metal
As1S1_ICSD_180621	insulator	insulator
As1S2Tl1_ICSD_26514	metal	insulator
As1S2Tl1_ICSD_658371	insulator	insulator
As1S2_ICSD_424590	insulator	insulator
As1S3Tl3_ICSD_611332	insulator	insulator
As1S3Tl3_ICSD_79580	insulator	insulator
As1S4Tl3_ICSD_61057	insulator	insulator
As1Sb3Zr5_ICSD_611352	metal	metal
As1Sc1_ICSD_164628	metal	metal
As1Sc1_ICSD_44057	metal	metal
As1Se1Th1_ICSD_611384	metal	metal
As1Se1U1_ICSD_66946	metal	metal
As1Se3Tl3_ICSD_603666	insulator	insulator
As1Si1Ta1_ICSD_42355	metal	metal
As1Si1_ICSD_611404	insulator	insulator
As1Sm1_ICSD_611421	metal	metal
As1Sm1_ICSD_611422	metal	metal
As1Sn1_ICSD_44063	metal	metal
As1Sr1_ICSD_26264	metal	metal
As1Sr2_ICSD_44066	metal	metal
As1Ta1_ICSD_611451	metal	metal
As1Ta2_ICSD_611452	metal	metal
As1Ta3_ICSD_611459	metal	metal
As1Tb1_ICSD_43601	metal	metal
As1Te1Th1_ICSD_611471	metal	metal
As1Te1Ti1_ICSD_281420	metal	metal

Supplementary Table 72. Five-fold cross validated predictions for the metal/insulator classification (64/598).

system	calculated	predicted
As1Te1U1_ICSD_87139	metal	metal
As1Te1_ICSD_44071	metal	metal
As1Th1_ICSD_44073	metal	metal
As1Th1_ICSD_611485	metal	metal
As1Ti1Zr1_ICSD_92989	metal	metal
As1Ti1_ICSD_44981	metal	metal
As1Ti1_ICSD_611502	metal	metal
As1Ti3_ICSD_611498	metal	metal
As1Ti3_ICSD_611501	metal	metal
As1Tl1_ICSD_184574	metal	metal
As1Tm1_ICSD_43647	metal	metal
As1Tm1_ICSD_611521	metal	metal
As1U1_ICSD_44079	metal	metal
As1U1_ICSD_611545	metal	metal
As1V1_ICSD_42445	metal	metal
As1V1_ICSD_42446	metal	metal
As1V1_ICSD_44083	metal	metal
As1V3_ICSD_611575	metal	metal
As1Y1_ICSD_160147	metal	metal
As1Y1_ICSD_658803	metal	metal
As1Yb1_ICSD_44088	metal	metal
As1Zn1_ICSD_431	insulator	insulator
As1Zr1_ICSD_20291	metal	metal
As1Zr1_ICSD_44092	metal	metal
As1Zr3_ICSD_611620	metal	metal
As1_ICSD_158473	metal	metal
As1_ICSD_158474	metal	metal
As1_ICSD_609828	metal	metal
As1_ICSD_70100	metal	metal
As2Au1K5_ICSD_40698	insulator	insulator
As2B1Cs3_ICSD_300122	metal	insulator
As2B1K3_ICSD_300105	insulator	insulator
As2B1Rb3_ICSD_402082	insulator	insulator
As2B4Cl4_ICSD_80153	insulator	insulator
As2Ba1Cd2_ICSD_30917	insulator	insulator
As2Ba1Co2O8_ICSD_260062	insulator	insulator
As2Ba1Co2_ICSD_609848	metal	metal
As2Ba1Cr2_ICSD_609849	metal	metal
As2Ba1Cu2_ICSD_609850	metal	metal
As2Ba1Cu4_ICSD_89628	metal	metal
As2Ba1Cu6_ICSD_79256	metal	metal
As2Ba1Fe2_ICSD_180475	metal	metal
As2Ba1Fe2_ICSD_180490	metal	metal
As2Ba1Fe2_ICSD_182273	metal	metal
As2Ba1Fe2_ICSD_188346	metal	metal
As2Ba1Fe2_ICSD_189024	metal	metal
As2Ba1Ga2_ICSD_380478	insulator	insulator
As2Ba1Ge2_ICSD_26417	metal	insulator
As2Ba1Mg2_ICSD_30916	insulator	insulator
As2Ba1Mn2_ICSD_41794	metal	metal

Supplementary Table 73. Five-fold cross validated predictions for the metal/insulator classification (65/598).

system	calculated	predicted
As2Ba1Ni2O8_ICSD_27014	insulator	insulator
As2Ba1Ni2_ICSD_164196	metal	metal
As2Ba1Ni2_ICSD_185463	metal	metal
As2Ba1O1Ti2_ICSD_169074	metal	metal
As2Ba1Pd1_ICSD_405110	metal	metal
As2Ba1Pd2_ICSD_36376	metal	metal
As2Ba1Pd2_ICSD_61196	metal	metal
As2Ba1Pt1_ICSD_405112	metal	metal
As2Ba1Rh2_ICSD_416983	metal	metal
As2Ba1Ru2_ICSD_602110	metal	metal
As2Ba1Zn2_ICSD_12146	insulator	insulator
As2Ba1Zn2_ICSD_417000	metal	metal
As2Ba1_ICSD_414139	insulator	insulator
As2Ba2Cd1_ICSD_422941	insulator	metal
As2Ba2Ge1_ICSD_35151	insulator	insulator
As2Ba2Mn1O2Zn2_ICSD_85659	metal	metal
As2Ba2Mn2O1_ICSD_75454	insulator	metal
As2Ba2Se5_ICSD_60954	insulator	insulator
As2Ba2Zn1_ICSD_421423	insulator	insulator
As2Ba3O8_ICSD_404438	insulator	insulator
As2Ba4O1_ICSD_33905	insulator	insulator
As2Be1K4_ICSD_300111	insulator	insulator
As2Be2Ca1_ICSD_609867	insulator	insulator
As2Be2Mg1_ICSD_609872	insulator	insulator
As2Br1La3O7_ICSD_421948	insulator	insulator
As2Br2Cd2Hg2_ICSD_240354	insulator	insulator
As2Br3Hg4_ICSD_82312	insulator	insulator
As2Ca1Cd2_ICSD_100065	insulator	insulator
As2Ca1Co2_ICSD_609899	metal	metal
As2Ca1Cu1O7_ICSD_82623	insulator	insulator
As2Ca1Cu4_ICSD_32619	metal	metal
As2Ca1F20Xe4_ICSD_412759	insulator	insulator
As2Ca1Fe2_ICSD_166017	metal	metal
As2Ca1Fe2_ICSD_182277	metal	metal
As2Ca1Fe2_ICSD_182278	metal	metal
As2Ca1Ga2_ICSD_422526	metal	metal
As2Ca1H4O8_ICSD_2809	insulator	insulator
As2Ca1H6Mn1O10_ICSD_200702	insulator	insulator
As2Ca1Mg2_ICSD_100041	insulator	insulator
As2Ca1Mn2_ICSD_41792	metal	metal
As2Ca1Ni2_ICSD_188174	metal	metal
As2Ca1O6_ICSD_77379	insulator	insulator
As2Ca1Pd2_ICSD_604341	metal	metal
As2Ca1Ru2_ICSD_609914	metal	metal
As2Ca1Zn2_ICSD_609920	insulator	insulator
As2Ca2Cd1_ICSD_422578	insulator	insulator
As2Ca2Cd1_ICSD_422579	insulator	insulator
As2Ca2H4Mn1O10_ICSD_156223	insulator	insulator
As2Ca2O7_ICSD_32602	insulator	insulator
As2Ca4O1_ICSD_68203	insulator	insulator

Supplementary Table 74. Five-fold cross validated predictions for the metal/insulator classification (66/598).

system	calculated	predicted
As2Cd1Ge1_ICSD_609947	metal	metal
As2Cd1Hg4I4_ICSD_416973	insulator	insulator
As2Cd1K4_ICSD_300190	insulator	insulator
As2Cd1O6_ICSD_280576	insulator	insulator
As2Cd1Si1_ICSD_603895	insulator	insulator
As2Cd1Sn1_ICSD_609982	metal	metal
As2Cd1Sr2_ICSD_422940	insulator	insulator
As2Cd1_ICSD_16037	insulator	metal
As2Cd2Eu1_ICSD_422963	metal	metal
As2Cd2O7_ICSD_280579	insulator	insulator
As2Cd2Sr1_ICSD_23249	insulator	insulator
As2Cd3_ICSD_56167	metal	metal
As2Cd3_ICSD_609930	metal	metal
As2Cd4I3_ICSD_1964	insulator	insulator
As2Ce1Co2_ICSD_610002	metal	metal
As2Ce1Ir2_ICSD_186993	metal	metal
As2Ce1Li2_ICSD_32042	insulator	metal
As2Ce1Ni2_ICSD_610005	metal	metal
As2Ce1Ni2_ICSD_610006	metal	metal
As2Ce1Pd2_ICSD_604354	metal	metal
As2Ce1Pd3_ICSD_81775	metal	metal
As2Ce1Rh2_ICSD_610012	metal	metal
As2Ce1_ICSD_609997	insulator	insulator
As2Cl13Sb1_ICSD_26088	insulator	insulator
As2Cl2In2O5_ICSD_422701	insulator	insulator
As2Cl3F5O1_ICSD_82760	insulator	insulator
As2Cl3Hg3Tl1_ICSD_411520	insulator	insulator
As2Cl4F6_ICSD_33884	insulator	insulator
As2Cl6O2_ICSD_411196	insulator	insulator
As2Co1O6_ICSD_80350	insulator	insulator
As2Co1U1_ICSD_90313	metal	metal
As2Co1Zr6_ICSD_83932	metal	metal
As2Co1_ICSD_610034	metal	metal
As2Co1_ICSD_610039	metal	metal
As2Co2K1_ICSD_610072	metal	metal
As2Co2La1_ICSD_610073	metal	metal
As2Co2Nd1_ICSD_610090	metal	metal
As2Co2O6Sc2Sr4_ICSD_262356	metal	metal
As2Co2O7_ICSD_69001	insulator	insulator
As2Co2O8Sr1_ICSD_400764	insulator	insulator
As2Co2Pr1_ICSD_610099	metal	metal
As2Co2Sr1_ICSD_610122	metal	metal
As2Co2Th1_ICSD_610124	metal	metal
As2Co7O12_ICSD_403080	insulator	insulator
As2Cr1_ICSD_43898	metal	metal
As2Cr2Fe2O6Sr4_ICSD_180529	metal	metal
As2Cr2Sr1_ICSD_610273	metal	metal
As2Cs1Rh2_ICSD_610296	metal	metal
As2Cs1Ru2_ICSD_610297	metal	metal
As2Cs2F8O2_ICSD_6070	insulator	insulator

Supplementary Table 75. Five-fold cross validated predictions for the metal/insulator classification (67/598).

system	calculated	predicted
As2Cs2Pd1_ICSD_69647	insulator	insulator
As2Cs2Se9Sn1_ICSD_281267	insulator	insulator
As2Cs2Si1_ICSD_71225	insulator	insulator
As2Cs2Sn1_ICSD_71226	insulator	insulator
As2Cs4Te6_ICSD_405235	insulator	insulator
As2Cu1Dy1_ICSD_412552	metal	metal
As2Cu1Er1_ICSD_412553	metal	metal
As2Cu1F12O8S4_ICSD_411792	metal	insulator
As2Cu1Gd1_ICSD_91305	metal	metal
As2Cu1H16O20U2_ICSD_97287	metal	insulator
As2Cu1Ho1_ICSD_94440	metal	metal
As2Cu1K5_ICSD_40699	insulator	insulator
As2Cu1Lu1_ICSD_174358	metal	metal
As2Cu1Na4O8_ICSD_63058	insulator	insulator
As2Cu1O4_ICSD_16829	metal	insulator
As2Cu1O8Zn2_ICSD_18115	insulator	insulator
As2Cu1Tb1_ICSD_412551	metal	metal
As2Cu1Tm1_ICSD_412554	metal	metal
As2Cu1U1_ICSD_601780	metal	metal
As2Cu1Y1_ICSD_412550	metal	metal
As2Cu1Yb1_ICSD_412193	metal	metal
As2Cu1_ICSD_610303	metal	metal
As2Cu2O7_ICSD_162061	insulator	insulator
As2Cu2O7_ICSD_162062	insulator	insulator
As2Cu2Sr1_ICSD_610366	metal	metal
As2Cu2U1_ICSD_601803	metal	metal
As2Cu3K3_ICSD_32015	insulator	insulator
As2Cu3O8_ICSD_201733	insulator	insulator
As2Cu3O8_ICSD_24198	insulator	insulator
As2Cu3O8_ICSD_63057	insulator	insulator
As2Cu4Eu1_ICSD_89627	metal	metal
As2Cu4K1_ICSD_59207	metal	metal
As2Cu4Sr1_ICSD_89626	metal	metal
As2Cu5_ICSD_26253	metal	metal
As2Dy1K3S8_ICSD_420737	insulator	insulator
As2Dy1Ni4_ICSD_68526	metal	metal
As2Dy2Ni1_ICSD_610379	metal	metal
As2Er1Ni4_ICSD_610390	metal	metal
As2Er2Ni1_ICSD_610392	metal	metal
As2Eu1Fe2_ICSD_163210	metal	metal
As2Eu1Fe2_ICSD_163211	metal	metal
As2Eu1Fe2_ICSD_169687	metal	metal
As2Eu1Fe2_ICSD_185355	metal	metal
As2Eu1Mn2_ICSD_100583	metal	metal
As2Eu1Ni2_ICSD_610437	metal	metal
As2Eu1Pd2_ICSD_604348	metal	metal
As2Eu1Pt2_ICSD_418152	metal	metal
As2Eu1Rh2_ICSD_416982	metal	metal
As2Eu1Ru2_ICSD_610446	metal	metal
As2Eu1Zn2_ICSD_610450	metal	insulator

Supplementary Table 76. Five-fold cross validated predictions for the metal/insulator classification (68/598).

system	calculated	predicted
As2Eu2_ICSD_810	metal	metal
As2Eu3S8_ICSD_249612	insulator	insulator
As2Eu4O1_ICSD_1222	insulator	insulator
As2F12Hg3_ICSD_9323	insulator	insulator
As2F12Hg4_ICSD_35412	insulator	insulator
As2F12I4S2_ICSD_14074	insulator	insulator
As2F12I4_ICSD_37001	metal	insulator
As2F12Mn1O4S2_ICSD_411791	insulator	insulator
As2F12Mn1_ICSD_83635	insulator	insulator
As2F12N2S1Se2_ICSD_71357	insulator	insulator
As2F12N2S3_ICSD_54159	insulator	insulator
As2F12N2Se3_ICSD_73584	insulator	insulator
As2F16Mg1Xe2_ICSD_281694	insulator	insulator
As2F18Pb1Xe3_ICSD_391093	insulator	insulator
As2F2O1Sr2Ti2_ICSD_167013	metal	insulator
As2F7K1_ICSD_36332	insulator	insulator
As2F8K2O2_ICSD_9027	insulator	insulator
As2F8O2Rb2_ICSD_9028	insulator	insulator
As2Fe1Li1O7_ICSD_75180	insulator	insulator
As2Fe1_ICSD_610471	metal	metal
As2Fe2O6Sc2Sr4_ICSD_180528	metal	metal
As2Fe2Rb1_ICSD_167329	metal	metal
As2Fe2Sr1_ICSD_163208	metal	metal
As2Fe2Sr1_ICSD_163869	metal	metal
As2Fe4O11_ICSD_100442	insulator	insulator
As2Ga1K1O7_ICSD_82391	insulator	insulator
As2Ga1K2Li1_ICSD_401208	insulator	insulator
As2Ga1K2Na1_ICSD_300129	insulator	insulator
As2Ga1Li1Na2_ICSD_402111	insulator	insulator
As2Ga1Li1O7_ICSD_161500	insulator	insulator
As2Gd1Ni2_ICSD_610591	metal	metal
As2Ge1K2_ICSD_71222	insulator	insulator
As2Ge1Mg1_ICSD_182368	insulator	insulator
As2Ge1Te4_ICSD_68111	insulator	insulator
As2Ge1Zn1_ICSD_16735	metal	metal
As2Ge1_ICSD_23872	insulator	insulator
As2Ge2Te5_ICSD_68112	insulator	insulator
As2Ge3Te6_ICSD_68113	insulator	insulator
As2Ge4Te7_ICSD_68114	insulator	insulator
As2Ge5Te8_ICSD_63174	insulator	insulator
As2H16O16Zn3_ICSD_100492	insulator	insulator
As2H2K1O8Sc1_ICSD_59820	insulator	insulator
As2H3Li1O7_ICSD_172988	insulator	insulator
As2H4O9Ti1_ICSD_86771	insulator	insulator
As2H4O9V1_ICSD_69585	insulator	insulator
As2H6O11U1_ICSD_411217	insulator	insulator
As2H8O12Zn3_ICSD_290615	insulator	insulator
As2Hf1Ni4_ICSD_610651	metal	metal
As2Hf1_ICSD_42916	metal	metal
As2Hf1_ICSD_610636	metal	metal

Supplementary Table 77. Five-fold cross validated predictions for the metal/insulator classification (69/598).

system	calculated	predicted
As2Hf3_ICSD_610637	metal	metal
As2Hg1K4_ICSD_402573	insulator	insulator
As2Hg1O6_ICSD_409490	insulator	insulator
As2Hg2O6_ICSD_411230	insulator	insulator
As2Hg3O8_ICSD_72527	insulator	insulator
As2Hg4I3_ICSD_67227	insulator	insulator
As2Hg4O7_ICSD_391228	insulator	insulator
As2Hg6O8_ICSD_2604	insulator	insulator
As2Ho1Ni4_ICSD_610673	metal	metal
As2Ho2Ni1_ICSD_68142	metal	metal
As2In1K2Li1_ICSD_402147	insulator	insulator
As2Ir1_ICSD_610739	insulator	insulator
As2Ir2La1_ICSD_186992	metal	metal
As2K2Ni1_ICSD_300120	insulator	insulator
As2K2Pd1_ICSD_32009	insulator	insulator
As2K2Pt1_ICSD_610762	insulator	insulator
As2K2S6Sn1_ICSD_281039	insulator	insulator
As2K2Si1_ICSD_40426	insulator	insulator
As2K3La1O8_ICSD_421979	insulator	insulator
As2K3Nb1O9_ICSD_202980	insulator	insulator
As2K3Nd1S8_ICSD_240365	insulator	insulator
As2K4Zn1_ICSD_409919	insulator	insulator
As2La1Ni2_ICSD_610772	metal	metal
As2La1Ni2_ICSD_610773	metal	metal
As2La1Pd2_ICSD_604343	metal	metal
As2La1Rh2_ICSD_610777	metal	metal
As2La1Ru2_ICSD_602111	metal	metal
As2La1_ICSD_280294	insulator	insulator
As2La1_ICSD_610769	insulator	insulator
As2Li1O7Sc1_ICSD_161499	insulator	insulator
As2Li2Nd1_ICSD_23261	metal	metal
As2Li2Pr1_ICSD_23260	metal	metal
As2Li3Nd1_ICSD_49626	insulator	metal
As2Lu1Ni4_ICSD_610818	metal	metal
As2Mg1Si1_ICSD_182367	insulator	insulator
As2Mg2O7_ICSD_16885	insulator	insulator
As2Mg2Sr1_ICSD_610831	insulator	insulator
As2Mg2Zn1_ICSD_610834	metal	insulator
As2Mg3_ICSD_24485	insulator	insulator
As2Mg3_ICSD_25504	insulator	insulator
As2Mg3_ICSD_610824	insulator	insulator
As2Mn1O6_ICSD_80351	insulator	insulator
As2Mn1S5Tl2_ICSD_17035	insulator	insulator
As2Mn2O7_ICSD_69003	insulator	insulator
As2Mn2Sr1_ICSD_41793	metal	metal
As2Mn2Sr1_ICSD_49020	metal	metal
As2Mn2Yb1_ICSD_100585	metal	metal
As2Mn2Zn1_ICSD_610947	metal	metal
As2Mn3O2Sr2_ICSD_32010	metal	metal
As2Mn3_ICSD_73251	metal	metal

Supplementary Table 78. Five-fold cross validated predictions for the metal/insulator classification (70/598).

system	calculated	predicted
As2Mn3_ICSD_75510	metal	metal
As2Mo1_ICSD_610950	metal	metal
As2Mo2O12Sr1_ICSD_87794	insulator	insulator
As2Na1O7Sc1_ICSD_161501	insulator	insulator
As2Na1Sn2_ICSD_82366	metal	metal
As2Na1_ICSD_182161	metal	insulator
As2Na2_ICSD_421420	insulator	insulator
As2Na4O7_ICSD_2887	insulator	insulator
As2Na4O9Ti1_ICSD_59321	insulator	insulator
As2Na4Te4_ICSD_300185	insulator	insulator
As2Nb1Ni1_ICSD_38412	metal	metal
As2Nb1_ICSD_18143	metal	metal
As2Nd1Ni2_ICSD_611001	metal	metal
As2Nd1Ni2_ICSD_611004	metal	metal
As2Nd1Pd2_ICSD_604337	metal	metal
As2Nd1Rh2_ICSD_611010	metal	metal
As2Nd1_ICSD_1730	insulator	insulator
As2Ni1O6_ICSD_80349	insulator	insulator
As2Ni1Tb2_ICSD_611083	metal	metal
As2Ni1Tm2_ICSD_96762	metal	metal
As2Ni1U1_ICSD_601778	metal	metal
As2Ni1Zr2_ICSD_68141	metal	metal
As2Ni1_ICSD_34851	metal	metal
As2Ni1_ICSD_611031	metal	metal
As2Ni1_ICSD_76940	metal	metal
As2Ni2O6Sc2Sr4_ICSD_180270	metal	metal
As2Ni2O7_ICSD_69002	insulator	insulator
As2Ni2Pr1_ICSD_611047	metal	metal
As2Ni2Pr1_ICSD_611048	metal	metal
As2Ni2Sr1_ICSD_611078	metal	metal
As2Ni2U1_ICSD_611092	metal	metal
As2Ni3O8_ICSD_63708	insulator	insulator
As2Ni3O8_ICSD_63709	insulator	insulator
As2Ni4Sc1_ICSD_611064	metal	metal
As2Ni4Tb1_ICSD_611081	metal	metal
As2Ni4Y1_ICSD_611100	metal	metal
As2Ni4Yb1_ICSD_611105	metal	metal
As2Ni4Zr1_ICSD_611111	metal	metal
As2Ni5_ICSD_42681	metal	metal
As2O11W2_ICSD_15020	insulator	insulator
As2O12S3_ICSD_32586	insulator	insulator
As2O13Pb8_ICSD_99432	insulator	insulator
As2O13V4_ICSD_60781	insulator	insulator
As2O14Pb3Te1Zn3_ICSD_85574	insulator	insulator
As2O1Sr4_ICSD_33904	insulator	insulator
As2O1Yb4_ICSD_402951	insulator	metal
As2O3_ICSD_100434	insulator	insulator
As2O3_ICSD_183097	metal	insulator
As2O3_ICSD_27588	insulator	insulator
As2O3_ICSD_409611	insulator	insulator

Supplementary Table 79. Five-fold cross validated predictions for the metal/insulator classification (71/598).

system	calculated	predicted
As2O4Pb1_ICSD_65027	insulator	insulator
As2O4Zn1_ICSD_202249	insulator	insulator
As2O4_ICSD_10436	insulator	insulator
As2O5_ICSD_10015	insulator	insulator
As2O5_ICSD_987	insulator	insulator
As2O6Pb1_ICSD_81063	insulator	insulator
As2O6Pd1_ICSD_187098	insulator	insulator
As2O6S1_ICSD_4297	insulator	insulator
As2O6Sr1_ICSD_420296	insulator	insulator
As2O6Sr1_ICSD_420297	insulator	insulator
As2O6Zn3_ICSD_10400	insulator	insulator
As2O6Zn3_ICSD_417004	insulator	insulator
As2O7Ti1_ICSD_73476	insulator	insulator
As2O7Ti1_ICSD_73477	insulator	insulator
As2O8Pb3_ICSD_200414	insulator	insulator
As2O8Sr3_ICSD_420295	insulator	insulator
As2O8Zn3_ICSD_404199	insulator	insulator
As2O8Zn3_ICSD_404229	insulator	insulator
As2O9S2_ICSD_63530	insulator	insulator
As2O9V3_ICSD_424067	insulator	insulator
As2Os1_ICSD_611138	insulator	insulator
As2P2S7_ICSD_30706	insulator	insulator
As2Pa1_ICSD_611160	metal	metal
As2Pd1_ICSD_43101	metal	metal
As2Pd2Pr1_ICSD_604345	metal	metal
As2Pd2Sm1_ICSD_604347	metal	metal
As2Pd2Sr1_ICSD_36374	metal	metal
As2Pr1_ICSD_611219	insulator	insulator
As2Pt1Rb2_ICSD_107529	insulator	insulator
As2Pt1_ICSD_38428	insulator	metal
As2Pt2Sr1_ICSD_181396	metal	metal
As2Pt2Sr1_ICSD_418156	metal	metal
As2Rb2Si1_ICSD_60617	insulator	insulator
As2Rb2Sn1_ICSD_71223	insulator	insulator
As2Rh1_ICSD_42616	insulator	metal
As2Rh2Sr1_ICSD_417001	metal	metal
As2Rh2Sr1_ICSD_417002	metal	metal
As2Ru1_ICSD_611289	insulator	insulator
As2Ru2Sr1_ICSD_602109	metal	metal
As2S3_ICSD_185819	insulator	insulator
As2S6Sn1Tl2_ICSD_72907	insulator	insulator
As2Sc3_ICSD_16411	metal	metal
As2Sc3_ICSD_611355	metal	metal
As2Se3_ICSD_2600	insulator	insulator
As2Se3_ICSD_611373	insulator	insulator
As2Si1Zn1_ICSD_23707	insulator	insulator
As2Si1_ICSD_24801	metal	metal
As2Si1_ICSD_611405	insulator	insulator
As2Sn1Zn1_ICSD_43357	metal	metal
As2Sn2Sr1_ICSD_82371	metal	metal

Supplementary Table 80. Five-fold cross validated predictions for the metal/insulator classification (72/598).

system	calculated	predicted
As2Sr1Zn2_ICSD_23248	insulator	insulator
As2Ta1_ICSD_107966	metal	metal
As2Te3_ICSD_30981	insulator	insulator
As2Te3_ICSD_41040	insulator	insulator
As2Te3_ICSD_68110	insulator	insulator
As2Th1_ICSD_611488	metal	metal
As2Ti1_ICSD_611500	metal	metal
As2U1_ICSD_611529	metal	metal
As2U1_ICSD_611531	metal	metal
As2V1_ICSD_611574	metal	metal
As2V3_ICSD_999	metal	metal
As2W1_ICSD_611576	metal	metal
As2Yb1Zn2_ICSD_88231	insulator	metal
As2Zn1_ICSD_2021	insulator	insulator
As2Zn3_ICSD_24486	metal	metal
As2Zn3_ICSD_611595	metal	metal
As2Zn3_ICSD_611608	insulator	metal
As2Zr1_ICSD_168665	metal	metal
As2Zr1_ICSD_20292	metal	metal
As2Zr3_ICSD_611611	metal	metal
As3Ba1_ICSD_86402	metal	metal
As3Ba2Cd2_ICSD_420833	metal	metal
As3Ba3In1_ICSD_402338	insulator	insulator
As3Ba3Nb1O1_ICSD_408853	insulator	insulator
As3Ba3O1Ta1_ICSD_280155	insulator	insulator
As3Ba5Cl1O12_ICSD_260065	insulator	insulator
As3Ba5_ICSD_43872	metal	metal
As3Be3Cl1Li4O12_ICSD_74526	insulator	insulator
As3Bi2K3O12_ICSD_59887	insulator	insulator
As3Br1Cd2_ICSD_100815	insulator	insulator
As3Br1Cd2_ICSD_75170	insulator	insulator
As3Br1Hg2_ICSD_75169	insulator	insulator
As3C1V5_ICSD_609885	metal	metal
As3Ca1Fe4_ICSD_260320	metal	metal
As3Ca1Fe4_ICSD_260323	metal	metal
As3Ca1_ICSD_193	metal	metal
As3Ca2_ICSD_43876	insulator	insulator
As3Ca3Ga1_ICSD_60126	insulator	insulator
As3Ca5F1O12_ICSD_172996	insulator	insulator
As3Ca5_ICSD_609889	metal	insulator
As3Cd2I1_ICSD_40449	insulator	insulator
As3Cd2I1_ICSD_8216	insulator	insulator
As3Cd4K1_ICSD_262032	metal	metal
As3Cd4Rb1_ICSD_262037	metal	metal
As3Ce4_ICSD_43883	metal	metal
As3Cl1O12Pb5_ICSD_69960	insulator	insulator
As3Cl1O12Sr5_ICSD_260196	insulator	insulator
As3Cl1O9Pb5_ICSD_100168	insulator	insulator
As3Cl1O9Pb5_ICSD_31863	insulator	insulator
As3Co1Hf5_ICSD_85884	metal	metal

Supplementary Table 81. Five-fold cross validated predictions for the metal/insulator classification (73/598).

system	calculated	predicted
As3Co1_ICSD_34051	metal	metal
As3Cr2Na3O12_ICSD_280946	metal	insulator
As3Cr4_ICSD_66761	metal	metal
As3Cs1Zn4_ICSD_262030	metal	insulator
As3Cs5Ge1_ICSD_65718	metal	insulator
As3Cs5Si1_ICSD_65716	insulator	metal
As3Cs6In1_ICSD_300145	insulator	insulator
As3Cu13S16V1_ICSD_610353	metal	insulator
As3Cu2O1U2_ICSD_75132	metal	metal
As3Cu4K1O12_ICSD_63059	insulator	insulator
As3Eu1Ni5_ICSD_33916	metal	metal
As3Eu1_ICSD_86401	metal	metal
As3Eu4_ICSD_10440	metal	metal
As3Eu5_ICSD_2250	metal	metal
As3F1O12Sr5_ICSD_260044	insulator	insulator
As3F6Sb1Se4_ICSD_24413	insulator	insulator
As3H1Sr5_ICSD_173027	insulator	metal
As3H2Na1O9_ICSD_65011	insulator	insulator
As3H5O10_ICSD_14327	insulator	insulator
As3Hf1Na5_ICSD_66026	insulator	insulator
As3Hg1S6Tl1_ICSD_38363	insulator	insulator
As3Ir1_ICSD_15457	metal	metal
As3K5O10_ICSD_23302	insulator	insulator
As3K6Nb1O1_ICSD_409630	insulator	insulator
As3La1Si1_ICSD_68204	metal	insulator
As3La1Zn3_ICSD_261981	insulator	metal
As3La4_ICSD_610771	metal	metal
As3Mg4Na1O12_ICSD_59888	insulator	insulator
As3Mn4_ICSD_80375	metal	metal
As3Mo2_ICSD_41015	metal	metal
As3Na1O12Ti2_ICSD_421531	insulator	insulator
As3Na1O12Zr2_ICSD_97956	insulator	insulator
As3Na1Zn4_ICSD_262036	insulator	metal
As3Na5Sn1_ICSD_656058	insulator	insulator
As3Nb4_ICSD_15032	metal	metal
As3Nb5_ICSD_16417	metal	metal
As3Ni5Sr1_ICSD_33915	metal	metal
As3O1Sr3Ta1_ICSD_409567	insulator	insulator
As3Pr4_ICSD_611220	metal	metal
As3Rb1Zn4_ICSD_262038	insulator	metal
As3Rb5Si1_ICSD_300191	insulator	insulator
As3Rh1_ICSD_611268	metal	metal
As3Sc5_ICSD_41677	metal	metal
As3Sn4_ICSD_611426	metal	metal
As3Sr1_ICSD_41831	metal	metal
As3Sr2Zn2_ICSD_262413	insulator	insulator
As3Sr4_ICSD_402110	insulator	insulator
As3Sr5_ICSD_44067	metal	metal
As3Ti4_ICSD_611492	metal	metal
As3Ti5_ICSD_611496	metal	metal

Supplementary Table 82. Five-fold cross validated predictions for the metal/insulator classification (74/598).

system	calculated	predicted
As3Ti5_ICSD_611507	metal	metal
As3V4_ICSD_15049	metal	metal
As3V4_ICSD_23787	metal	metal
As3V5_ICSD_611571	metal	metal
As3W2_ICSD_43185	metal	metal
As3Yb4_ICSD_153669	metal	metal
As3Yb4_ICSD_42685	metal	metal
As4Ba1Cu8_ICSD_66017	metal	metal
As4Ba3Cd2_ICSD_424761	metal	metal
As4Ba3Li4_ICSD_280027	metal	metal
As4Ba3Si2_ICSD_41183	insulator	insulator
As4Ba3Sn2_ICSD_30702	insulator	insulator
As4Ba3Zn2_ICSD_424760	metal	metal
As4Ba4Si1_ICSD_26467	insulator	insulator
As4Ba4Ti1_ICSD_380115	insulator	insulator
As4Bi1Cl7Hg6_ICSD_411204	insulator	insulator
As4Br16Ga4Hg11_ICSD_411676	insulator	insulator
As4Br1K1O6_ICSD_65206	insulator	insulator
As4Br6Cd1Hg6_ICSD_417039	insulator	insulator
As4Br7Cr1Hg6_ICSD_411481	insulator	insulator
As4Br7Hg6Ti1_ICSD_411239	insulator	insulator
As4Br7Hg6Yb1_ICSD_413988	metal	insulator
As4C3_ICSD_163831	metal	insulator
As4C4F12_ICSD_9071	insulator	insulator
As4Ca1Na2O12_ICSD_409335	insulator	insulator
As4Ca1Rh6_ICSD_89612	metal	metal
As4Ca3Ge2_ICSD_16455	insulator	insulator
As4Ca3In2_ICSD_61336	insulator	insulator
As4Ca3Si2_ICSD_16456	insulator	insulator
As4Ca5H10O20_ICSD_21068	insulator	insulator
As4Cd2Ge1_ICSD_42132	insulator	insulator
As4Cd5Rb2_ICSD_290262	metal	insulator
As4Cl1Cu1S3_ICSD_419754	insulator	insulator
As4Cl1K1O6_ICSD_65205	insulator	insulator
As4Cl2Cu2S3_ICSD_419755	insulator	insulator
As4Cl7Hg6In1_ICSD_411820	insulator	insulator
As4Cl7Hg6Mo1_ICSD_411240	insulator	insulator
As4Cl7Hg6Ti1_ICSD_411238	insulator	insulator
As4Cs1F13_ICSD_281641	insulator	insulator
As4Cs4Se8_ICSD_171373	insulator	insulator
As4Cu12S12_ICSD_33588	metal	metal
As4Cu12S13_ICSD_26724	metal	metal
As4Cu3K2O12_ICSD_65416	insulator	insulator
As4Cu3_ICSD_41169	metal	metal
As4Cu6Hg3S12_ICSD_169803	insulator	insulator
As4Eu3Pd4_ICSD_79094	metal	metal
As4Eu3_ICSD_8211	insulator	metal
As4Ga1Na3Sr3_ICSD_402286	insulator	insulator
As4Ge1Sr4_ICSD_610619	insulator	insulator
As4Ge1Te7_ICSD_41107	insulator	insulator

Supplementary Table 83. Five-fold cross validated predictions for the metal/insulator classification (75/598).

system	calculated	predicted
As4Ge2Sr3_ICSD_16454	insulator	insulator
As4Ge3_ICSD_163833	metal	metal
As4Hf7_ICSD_610634	metal	metal
As4I1K1O6_ICSD_16889	insulator	insulator
As4K7Nb1_ICSD_380109	insulator	insulator
As4K7Ta1_ICSD_380110	insulator	insulator
As4Mg1Rh6_ICSD_89611	metal	metal
As4Mg1_ICSD_1079	insulator	insulator
As4Mn5_ICSD_73252	metal	metal
As4Mo5_ICSD_43187	metal	metal
As4Mo5_ICSD_610956	metal	metal
As4Na2O11_ICSD_62963	insulator	insulator
As4Na5_ICSD_182165	metal	insulator
As4Na8Ti1_ICSD_73310	insulator	insulator
As4Nb1Rb7_ICSD_380111	insulator	insulator
As4Nb5Pd4_ICSD_412866	metal	metal
As4Nb7_ICSD_610986	metal	metal
As4Pa3_ICSD_9818	metal	metal
As4Pb9S15_ICSD_18097	insulator	insulator
As4Rb5Ta1Tl2_ICSD_85784	insulator	insulator
As4Rh6Sr1_ICSD_89613	metal	metal
As4Rh6Yb1_ICSD_89614	metal	metal
As4S3_ICSD_188058	insulator	insulator
As4S4_ICSD_187210	insulator	insulator
As4S5_ICSD_16107	insulator	insulator
As4Se3_ICSD_611376	insulator	insulator
As4Se4_ICSD_2056	insulator	insulator
As4Se4_ICSD_2599	insulator	insulator
As4Si1Sr4_ICSD_611409	insulator	insulator
As4Si2Sr3_ICSD_16453	insulator	insulator
As4Sn3_ICSD_163834	insulator	metal
As4Sr3_ICSD_100110	insulator	metal
As4Sr4Ti1_ICSD_380113	insulator	insulator
As4Ta5_ICSD_36525	metal	metal
As4Th3_ICSD_611484	metal	metal
As4U3_ICSD_611525	metal	metal
As4Zr7_ICSD_611614	metal	metal
As5B1O20Pb6_ICSD_404329	insulator	insulator
As5Ba1Ni9_ICSD_33919	metal	metal
As5Cs3O9_ICSD_413151	insulator	insulator
As5I1Te7_ICSD_31877	insulator	insulator
As5K6Sn3_ICSD_71009	insulator	insulator
As6Ba1Pt4_ICSD_62519	insulator	insulator
As6Bi1K3Se12_ICSD_180763	insulator	insulator
As6Ca5Ga2_ICSD_27	metal	insulator
As6Ca5Sn2_ICSD_61037	insulator	insulator
As6Cs4_ICSD_409382	insulator	insulator
As6Cu7Se13_ICSD_15235	metal	insulator
As6Ge2Na10_ICSD_300187	metal	insulator
As6Ir7Mg4_ICSD_94393	metal	metal

Supplementary Table 84. Five-fold cross validated predictions for the metal/insulator classification (76/598).

system	calculated	predicted
As6Mg4Rh7_ICSD_94391	metal	metal
As6Na10Si2_ICSD_300188	metal	insulator
As6Na10Sn2_ICSD_40559	insulator	insulator
As6Pt4Sr1_ICSD_62518	insulator	insulator
As6Rb4_ICSD_409381	insulator	insulator
As6Rh7Yb4_ICSD_94392	metal	metal
As6Ru7U4_ICSD_90326	metal	metal
As6Sn2Sr5_ICSD_262646	metal	insulator
As7Ca4Ir8_ICSD_413846	metal	metal
As7Ce2Ni12_ICSD_610004	metal	metal
As7Dy2Ni12_ICSD_610376	metal	metal
As7Er2Ni12_ICSD_610389	metal	metal
As7Ho2Ni12_ICSD_610672	metal	metal
As7K2Ni12_ICSD_300138	metal	metal
As7Mg6Ni16_ICSD_78834	metal	metal
As7Mn6Ni16_ICSD_83742	metal	metal
As7Ni12Tb2_ICSD_611080	metal	metal
As7Ni12U2_ICSD_93169	metal	metal
As7Ni12Y2_ICSD_611099	metal	metal
As7Re3_ICSD_611260	metal	metal
As8Ge38I8_ICSD_22033	insulator	insulator
As8Ni11_ICSD_164878	metal	metal
As8Ni11_ICSD_34853	metal	metal
As8S9_ICSD_98792	insulator	insulator
Au0.17Gd0.17Sn0.17_ICSD_240884	metal	metal
Au1.01Cd0.99_ICSD_106276	metal	metal
Au10Ca4In3_ICSD_249601	metal	metal
Au10In3_ICSD_612020	metal	metal
Au1B2_ICSD_52699	metal	metal
Au1Ba1Bi1_ICSD_106269	metal	metal
Au1Ba1Gd1Se3_ICSD_88718	insulator	insulator
Au1Ba1P1_ICSD_420340	metal	metal
Au1Ba1Sb1_ICSD_106270	metal	metal
Au1Ba1Sb1_ICSD_280954	metal	metal
Au1Ba1_ICSD_419559	metal	metal
Au1Ba2Tl7_ICSD_98964	metal	metal
Au1Ba4C4K1O4_ICSD_40854	insulator	insulator
Au1Ba4Na1O8_ICSD_73189	insulator	insulator
Au1Be12_ICSD_109312	metal	metal
Au1Be1_ICSD_58396	metal	metal
Au1Be5_ICSD_58397	metal	metal
Au1Bi1Ca1_ICSD_58399	metal	metal
Au1Bi1Eu1_ICSD_611652	metal	metal
Au1Bi1K2_ICSD_380341	insulator	insulator
Au1Bi1Li2_ICSD_261786	insulator	metal
Au1Bi1Li2_ICSD_58400	metal	metal
Au1Bi1Na2_ICSD_261788	insulator	insulator
Au1Bi1Sr1_ICSD_106271	metal	metal
Au1Bi1Yb1_ICSD_106272	metal	metal
Au1Bi1Yb1_ICSD_611656	metal	metal

Supplementary Table 85. Five-fold cross validated predictions for the metal/insulator classification (77/598).

system	calculated	predicted
Au1Bi2O5_ICSD_82092	metal	insulator
Au1Bi5Na2O11_ICSD_164986	insulator	insulator
Au1Br1C2H8N4S2_ICSD_72684	insulator	insulator
Au1Br1F6_ICSD_93481	insulator	insulator
Au1Br1Se1_ICSD_2897	insulator	insulator
Au1Br1_ICSD_200286	insulator	insulator
Au1Br1_ICSD_200287	insulator	insulator
Au1Br4K1_ICSD_280033	insulator	insulator
Au1Br8Te1_ICSD_63129	insulator	insulator
Au1C1Cl1O1_ICSD_33526	insulator	insulator
Au1C1Cl3S2_ICSD_63379	insulator	insulator
Au1C1N1_ICSD_165175	insulator	insulator
Au1C1_ICSD_169406	metal	metal
Au1C2Cl1H3N1_ICSD_152108	insulator	insulator
Au1C2Cl1H8N4S2_ICSD_170718	insulator	insulator
Au1C2Cs1_ICSD_411251	insulator	insulator
Au1C2K1N2S2_ICSD_159001	insulator	insulator
Au1C2K1N2_ICSD_26498	insulator	insulator
Au1C2K1_ICSD_411255	insulator	insulator
Au1C2Li1_ICSD_411253	insulator	insulator
Au1C2N2Rb1S2_ICSD_159002	insulator	insulator
Au1C2N2Rb1_ICSD_66037	insulator	insulator
Au1C2Na1_ICSD_411254	insulator	insulator
Au1C2Rb1_ICSD_411252	insulator	insulator
Au1C4H12N1_ICSD_110302	insulator	insulator
Au1C4H2K1N4O1_ICSD_16043	insulator	insulator
Au1Ca1Cd1_ICSD_420574	metal	metal
Au1Ca1Ga1_ICSD_106273	metal	metal
Au1Ca1Ge1_ICSD_85836	metal	metal
Au1Ca1In1_ICSD_408579	metal	metal
Au1Ca1In2_ICSD_408882	metal	metal
Au1Ca1P1_ICSD_52661	metal	metal
Au1Ca1Sb1_ICSD_52662	metal	metal
Au1Ca1_ICSD_54978	metal	metal
Au1Ca2N1_ICSD_85528	metal	metal
Au1Ca3N1_ICSD_72942	metal	metal
Au1Ca3_ICSD_58401	metal	metal
Au1Cd1Ce1_ICSD_411542	metal	metal
Au1Cd1Eu1_ICSD_411544	metal	metal
Au1Cd1La1_ICSD_411543	metal	metal
Au1Cd1Sb1_ICSD_52663	metal	metal
Au1Cd1Yb1_ICSD_411545	metal	metal
Au1Cd1_ICSD_58408	metal	metal
Au1Cd1_ICSD_58409	metal	metal
Au1Cd1_ICSD_58410	metal	metal
Au1Cd3_ICSD_58411	metal	metal
Au1Ce1Cu5_ICSD_107971	metal	metal
Au1Ce1Ge1_ICSD_154287	metal	metal
Au1Ce1Ge1_ICSD_245768	metal	metal
Au1Ce1Ge1_ICSD_57176	metal	metal

Supplementary Table 86. Five-fold cross validated predictions for the metal/insulator classification (78/598).

system	calculated	predicted
Au1Ce1In1_ICSD_58421	metal	metal
Au1Ce1Pb1_ICSD_611722	metal	metal
Au1Ce1Sb1_ICSD_603059	metal	metal
Au1Ce1Sb2_ICSD_658239	metal	metal
Au1Ce1Sn1_ICSD_411539	metal	metal
Au1Ce1Sn1_ICSD_611730	metal	metal
Au1Ce1Zn1_ICSD_418712	metal	metal
Au1Ce1_ICSD_611709	metal	metal
Au1Ce1_ICSD_611711	metal	metal
Au1Ce2P3_ICSD_411550	metal	metal
Au1Ce2_ICSD_611710	metal	metal
Au1Cl1F3P1_ICSD_415842	insulator	insulator
Au1Cl1O1_ICSD_8190	insulator	insulator
Au1Cl1Te2_ICSD_16324	metal	insulator
Au1Cl1_ICSD_6052	insulator	insulator
Au1Cl3Cs1_ICSD_56472	metal	insulator
Au1Cl3_ICSD_22146	insulator	insulator
Au1Cl4Cs1_ICSD_423233	insulator	insulator
Au1Cl4K1_ICSD_73080	insulator	insulator
Au1Cl4N5S5_ICSD_61254	insulator	insulator
Au1Cl4P1_ICSD_15565	insulator	insulator
Au1Cl4Rb1_ICSD_26021	insulator	insulator
Au1Cl4Tl1_ICSD_62107	insulator	insulator
Au1Cl5S1_ICSD_39532	insulator	insulator
Au1Cl7S1_ICSD_62908	insulator	insulator
Au1Cl7Se1_ICSD_62511	insulator	insulator
Au1Cl7Te1_ICSD_61350	insulator	insulator
Au1Cl8P1_ICSD_62236	insulator	insulator
Au1Co1Na4O5_ICSD_36661	insulator	insulator
Au1Cr1S2_ICSD_88852	insulator	metal
Au1Cr1Te4_ICSD_150279	metal	metal
Au1Cr3O8_ICSD_155508	insulator	insulator
Au1Cs1F4_ICSD_152056	insulator	insulator
Au1Cs1K2O2_ICSD_62064	insulator	insulator
Au1Cs1N12_ICSD_416487	insulator	insulator
Au1Cs1O1_ICSD_409553	insulator	insulator
Au1Cs1Se3_ICSD_84002	insulator	insulator
Au1Cs1Te1_ICSD_71653	insulator	insulator
Au1Cs1_ICSD_150971	insulator	insulator
Au1Cs3Ge4_ICSD_413725	insulator	insulator
Au1Cs3Pb4_ICSD_107448	insulator	insulator
Au1Cu1F5_ICSD_62544	metal	insulator
Au1Cu1Se4_ICSD_93052	metal	metal
Au1Cu1Sn1_ICSD_611768	metal	metal
Au1Cu1Zn2_ICSD_150571	metal	metal
Au1Cu1_ICSD_42575	metal	metal
Au1Cu3_ICSD_150666	metal	metal
Au1Cu4Dy1_ICSD_611752	metal	metal
Au1Cu4Er1_ICSD_611753	metal	metal
Au1Cu4Ho1_ICSD_611759	metal	metal

Supplementary Table 87. Five-fold cross validated predictions for the metal/insulator classification (79/598).

system	calculated	predicted
Au1Cu4Tb1_ICSD_611769	metal	metal
Au1Cu4U1_ICSD_611772	metal	metal
Au1Cu4Yb1_ICSD_611773	metal	metal
Au1Dy1Ge1_ICSD_106279	metal	metal
Au1Dy1In1_ICSD_54951	metal	metal
Au1Dy1Ni4_ICSD_611799	metal	metal
Au1Dy1Pb1_ICSD_58443	metal	metal
Au1Dy1Sn1_ICSD_55000	metal	metal
Au1Dy1Sn1_ICSD_611806	metal	metal
Au1Dy1_ICSD_58439	metal	metal
Au1Dy1_ICSD_611786	metal	metal
Au1Dy2_ICSD_611788	metal	metal
Au1Er1Ge1_ICSD_107984	metal	metal
Au1Er1In1_ICSD_165161	metal	metal
Au1Er1Ni4_ICSD_611829	metal	metal
Au1Er1Pb1_ICSD_58450	metal	metal
Au1Er1Sn1_ICSD_245759	metal	metal
Au1Er1Sn1_ICSD_58451	metal	metal
Au1Er1_ICSD_58444	metal	metal
Au1Er1_ICSD_611819	metal	metal
Au1Er2_ICSD_611816	metal	metal
Au1Eu1Ge1_ICSD_80270	metal	metal
Au1Eu1In1_ICSD_404043	metal	metal
Au1Eu1P1_ICSD_52664	metal	metal
Au1Eu1Sb1_ICSD_422622	metal	metal
Au1Eu1Zn1_ICSD_420674	metal	metal
Au1Eu1_ICSD_611844	metal	metal
Au1Eu3O6_ICSD_411503	insulator	insulator
Au1F11Th2_ICSD_89619	insulator	insulator
Au1F11U2_ICSD_152058	insulator	insulator
Au1F12Sb2Xe2_ICSD_412106	insulator	insulator
Au1F16H4Sb2_ICSD_412237	metal	insulator
Au1F3_ICSD_16876	insulator	insulator
Au1F4K1_ICSD_9906	insulator	insulator
Au1F4Li1_ICSD_33953	insulator	insulator
Au1F4Li1_ICSD_9908	insulator	insulator
Au1F4Na1_ICSD_9905	insulator	insulator
Au1F4Rb1_ICSD_9907	insulator	insulator
Au1F6K1_ICSD_415874	insulator	insulator
Au1F6Li1_ICSD_165209	insulator	insulator
Au1F6O2_ICSD_171655	insulator	insulator
Au1F6Tl1_ICSD_95770	insulator	insulator
Au1Ga1Hf1_ICSD_156265	metal	metal
Au1Ga1Li2_ICSD_58461	metal	metal
Au1Ga1O2_ICSD_95666	insulator	insulator
Au1Ga1Zr1_ICSD_156264	metal	metal
Au1Ga1_ICSD_611867	metal	metal
Au1Ga2_ICSD_188888	metal	metal
Au1Ga2_ICSD_611874	metal	metal
Au1Gd1Ge1_ICSD_656872	metal	metal

Supplementary Table 88. Five-fold cross validated predictions for the metal/insulator classification (80/598).

system	calculated	predicted
Au1Gd1In1_ICSD_104011	metal	metal
Au1Gd1Mg1_ICSD_413335	metal	metal
Au1Gd1Pb1_ICSD_58467	metal	metal
Au1Gd1Sn1_ICSD_245762	metal	metal
Au1Gd1_ICSD_611906	metal	metal
Au1Gd1_ICSD_611908	metal	metal
Au1Gd2_ICSD_611913	metal	metal
Au1Gd3O6_ICSD_411500	insulator	insulator
Au1Ge1Ho1_ICSD_245770	metal	metal
Au1Ge1Ho1_ICSD_656875	metal	metal
Au1Ge1La1_ICSD_405322	metal	metal
Au1Ge1Li2_ICSD_52668	metal	metal
Au1Ge1Lu1_ICSD_81734	metal	metal
Au1Ge1Na1_ICSD_78866	metal	metal
Au1Ge1Nd1_ICSD_656870	metal	metal
Au1Ge1Pr1_ICSD_656869	metal	metal
Au1Ge1Sc1_ICSD_81733	metal	metal
Au1Ge1Tb1_ICSD_656873	metal	metal
Au1Ge1Y1_ICSD_405323	metal	metal
Au1Ge1Yb1_ICSD_656877	metal	metal
Au1Ge1Yb1_ICSD_85835	metal	metal
Au1Ge2In3Yb3_ICSD_261317	metal	metal
Au1Ge4K3_ICSD_413728	insulator	insulator
Au1Ge4Rb3_ICSD_413724	insulator	insulator
Au1H3Ti3_ICSD_611953	metal	metal
Au1Hf1_ICSD_611959	metal	metal
Au1Hf2_ICSD_611958	metal	metal
Au1Ho1In1_ICSD_54955	metal	metal
Au1Ho1Ni4_ICSD_611998	metal	metal
Au1Ho1Pb1_ICSD_58487	metal	metal
Au1Ho1Sn1_ICSD_55001	metal	metal
Au1Ho1Sn1_ICSD_58488	metal	metal
Au1Ho1Sn1_ICSD_612005	metal	metal
Au1Ho1_ICSD_58479	metal	metal
Au1Ho1_ICSD_611986	metal	metal
Au1Ho2_ICSD_58480	metal	metal
Au1I1Te1_ICSD_1661	insulator	insulator
Au1I1_ICSD_24619	insulator	insulator
Au1I2K5O2_ICSD_40376	insulator	insulator
Au1I4K1O12_ICSD_417267	insulator	insulator
Au1I4K1_ICSD_406966	metal	insulator
Au1In1La1_ICSD_58496	metal	metal
Au1In1Li2_ICSD_58497	metal	metal
Au1In1Nd1_ICSD_58499	metal	metal
Au1In1O2_ICSD_95672	metal	insulator
Au1In1Pr1_ICSD_612052	metal	metal
Au1In1Sm1_ICSD_58505	metal	metal
Au1In1Sr1_ICSD_391422	metal	metal
Au1In1Tb1_ICSD_54949	metal	metal
Au1In1Y1_ICSD_58513	metal	metal

Supplementary Table 89. Five-fold cross validated predictions for the metal/insulator classification (81/598).

system	calculated	predicted
Au1In2Na1_ICSD_107505	metal	metal
Au1In2Na3_ICSD_170867	metal	metal
Au1In2Yb1_ICSD_410436	metal	metal
Au1In2_ICSD_612012	metal	metal
Au1K1N12_ICSD_416489	insulator	insulator
Au1K1N4O12_ICSD_16141	insulator	insulator
Au1K1Na2O2_ICSD_61226	insulator	insulator
Au1K1O2_ICSD_15115	insulator	insulator
Au1K1O8S2_ICSD_412094	insulator	insulator
Au1K1P2S7_ICSD_165325	insulator	insulator
Au1K1S1_ICSD_202178	insulator	insulator
Au1K1S5_ICSD_402875	insulator	insulator
Au1K1Se1_ICSD_656713	insulator	insulator
Au1K1Se2_ICSD_84003	insulator	insulator
Au1K1Se5_ICSD_67372	insulator	insulator
Au1K1Te1_ICSD_655856	metal	metal
Au1K2P1S4_ICSD_85679	insulator	insulator
Au1K2P1_ICSD_300201	insulator	insulator
Au1K2Sb1_ICSD_380340	insulator	insulator
Au1K3O1_ICSD_79086	insulator	insulator
Au1K3P2Se8_ICSD_165321	insulator	insulator
Au1K3Se13_ICSD_67373	insulator	insulator
Au1K3Se2_ICSD_402000	insulator	insulator
Au1K3Sn4_ICSD_107444	insulator	insulator
Au1K5P2_ICSD_40700	insulator	insulator
Au1La1O3_ICSD_73873	insulator	insulator
Au1La1Pb1_ICSD_612104	metal	metal
Au1La1Sb2_ICSD_658238	metal	metal
Au1La1_ICSD_612099	metal	metal
Au1La1_ICSD_612101	metal	metal
Au1La2_ICSD_612100	metal	metal
Au1Li1Mg1Sn1_ICSD_16477	metal	metal
Au1Li1S1_ICSD_165259	insulator	insulator
Au1Li1S1_ICSD_280534	insulator	insulator
Au1Li1Sb1_ICSD_107996	metal	metal
Au1Li1Sn1_ICSD_412208	metal	metal
Au1Li2Pb1_ICSD_58528	metal	metal
Au1Li2Sb1_ICSD_58529	metal	insulator
Au1Li2Sn1_ICSD_58533	metal	metal
Au1Li2Tl1_ICSD_58534	metal	metal
Au1Li3O3_ICSD_15113	insulator	insulator
Au1Li3S2_ICSD_280535	insulator	insulator
Au1Li3_ICSD_58524	metal	metal
Au1Lu1Ni4_ICSD_612133	metal	metal
Au1Lu1Si1_ICSD_71999	metal	metal
Au1Lu1Sn1_ICSD_415825	metal	metal
Au1Lu1_ICSD_58535	metal	metal
Au1Lu2_ICSD_612129	metal	metal
Au1Mg1Sn1_ICSD_16475	metal	metal
Au1Mg1Yb1_ICSD_411303	metal	metal

Supplementary Table 90. Five-fold cross validated predictions for the metal/insulator classification (82/598).

system	calculated	predicted
Au1Mg1_ICSD_612143	metal	metal
Au1Mg2_ICSD_58540	metal	metal
Au1Mg3_ICSD_106287	metal	metal
Au1Mg3_ICSD_58541	metal	metal
Au1Mg3_ICSD_58542	metal	metal
Au1Mg3_ICSD_612138	metal	metal
Au1Mn1Sb1_ICSD_612167	metal	metal
Au1Mn1Sn1_ICSD_240860	metal	metal
Au1Mn1_ICSD_109348	metal	metal
Au1Mn1_ICSD_109349	metal	metal
Au1Mn2_ICSD_58548	metal	metal
Au1Mn3_ICSD_150552	metal	metal
Au1N12Rb1_ICSD_416488	insulator	insulator
Au1N1Sr2_ICSD_95826	metal	metal
Au1N1V3_ICSD_58547	metal	metal
Au1N1_ICSD_167866	metal	metal
Au1N1_ICSD_167882	metal	metal
Au1N2_ICSD_166464	metal	metal
Au1N2_ICSD_166465	metal	insulator
Au1Na1O2_ICSD_409547	insulator	insulator
Au1Na1Se2_ICSD_84004	insulator	insulator
Au1Na1Sn1_ICSD_58554	metal	metal
Au1Na1Sn1_ICSD_660108	metal	metal
Au1Na1Te1_ICSD_71650	metal	metal
Au1Na2O2Rb1_ICSD_411460	insulator	insulator
Au1Na2Sb1_ICSD_23255	insulator	insulator
Au1Na2Sn3_ICSD_107556	metal	metal
Au1Na2_ICSD_58527	metal	metal
Au1Na3O2_ICSD_62066	insulator	insulator
Au1Na3S2_ICSD_202329	insulator	insulator
Au1Na4Tl1_ICSD_107428	metal	metal
Au1Nb3_ICSD_612198	metal	metal
Au1Nd1Pb1_ICSD_612219	metal	metal
Au1Nd1Sb2_ICSD_658241	metal	metal
Au1Nd1Sn1_ICSD_54998	metal	metal
Au1Nd1Sn1_ICSD_612224	metal	metal
Au1Nd1_ICSD_58565	metal	metal
Au1Nd1_ICSD_612215	metal	metal
Au1Nd1_ICSD_612217	metal	metal
Au1Nd2P3_ICSD_411552	metal	metal
Au1Ni2Sn4_ICSD_150127	metal	metal
Au1Ni4Sc1_ICSD_612227	metal	metal
Au1Ni4Tb1_ICSD_612229	metal	metal
Au1Ni4Y1_ICSD_612233	metal	metal
Au1Ni4Yb1_ICSD_612234	metal	metal
Au1O1Rb1_ICSD_409552	insulator	insulator
Au1O1Rb3_ICSD_75499	insulator	insulator
Au1O2Rb1_ICSD_15116	insulator	insulator
Au1O2Sc1_ICSD_95669	insulator	insulator
Au1O2Y1_ICSD_95675	insulator	insulator

Supplementary Table 91. Five-fold cross validated predictions for the metal/insulator classification (83/598).

system	calculated	predicted
Au1O8Rb1S2_ICSD_412095	insulator	insulator
Au1O8Rb1Se2_ICSD_413989	insulator	insulator
Au1P1S4Tl2_ICSD_85680	insulator	insulator
Au1P1S4_ICSD_413009	insulator	insulator
Au1Pb1Pr1_ICSD_612247	metal	metal
Au1Pb1Tb1_ICSD_58569	metal	metal
Au1Pb1Y1_ICSD_58570	metal	metal
Au1Pb2_ICSD_56272	metal	metal
Au1Pb3_ICSD_58567	metal	metal
Au1Pb4Rb3_ICSD_107447	insulator	insulator
Au1Pd3_ICSD_180877	metal	metal
Au1Pr1Sb2_ICSD_658240	metal	metal
Au1Pr1Sn1_ICSD_54997	metal	metal
Au1Pr1Sn1_ICSD_612271	metal	metal
Au1Pr1_ICSD_58575	metal	metal
Au1Pr1_ICSD_612261	metal	metal
Au1Pr1_ICSD_612264	metal	metal
Au1Pr2_ICSD_150634	metal	metal
Au1Pt4U1_ICSD_612276	metal	metal
Au1Rb1S1_ICSD_71654	insulator	insulator
Au1Rb1Se1_ICSD_656714	insulator	insulator
Au1Rb1Se3U1_ICSD_420483	insulator	insulator
Au1Rb1Te1_ICSD_71652	insulator	insulator
Au1Rb1Te1_ICSD_75026	metal	insulator
Au1Rb1Te3U1_ICSD_420485	insulator	insulator
Au1Rb1_ICSD_58428	insulator	metal
Au1Rb2S4Sb1_ICSD_54507	insulator	insulator
Au1Rb3Sn4_ICSD_107445	insulator	insulator
Au1S1Ta5_ICSD_51139	metal	metal
Au1S2V1_ICSD_96089	metal	metal
Au1Sb1Sr1_ICSD_106292	metal	metal
Au1Sb1Tl1_ICSD_391381	metal	metal
Au1Sb1Yb1_ICSD_83984	metal	metal
Au1Sb2Sm1_ICSD_658242	metal	metal
Au1Sb2U1_ICSD_157378	metal	metal
Au1Sb2_ICSD_43107	metal	metal
Au1Sb3_ICSD_43504	metal	metal
Au1Sc1Si1_ICSD_71998	metal	metal
Au1Sc1Sn1_ICSD_245753	metal	metal
Au1Sc1Sn1_ICSD_612303	metal	metal
Au1Sc1_ICSD_58582	metal	metal
Au1Se1_ICSD_52674	metal	metal
Au1Se1_ICSD_73668	metal	metal
Au1Se1_ICSD_73700	metal	metal
Au1Se3Tl4_ICSD_612311	metal	metal
Au1Si1Th1_ICSD_75032	metal	metal
Au1Si1U1_ICSD_81907	metal	metal
Au1Si1Y1_ICSD_72000	metal	metal
Au1Si1Yb1_ICSD_89631	metal	metal
Au1Sm1Sn1_ICSD_245763	metal	metal

Supplementary Table 92. Five-fold cross validated predictions for the metal/insulator classification (84/598).

system	calculated	predicted
Au1Sm1Sn1_ICSD_245764	metal	metal
Au1Sm1Sn1_ICSD_612343	metal	metal
Au1Sm1_ICSD_58584	metal	metal
Au1Sm1_ICSD_612333	metal	metal
Au1Sm1_ICSD_612335	metal	metal
Au1Sn1Sr1_ICSD_412013	metal	metal
Au1Sn1Tb1_ICSD_54999	metal	metal
Au1Sn1Tb1_ICSD_612357	metal	metal
Au1Sn1Tm1_ICSD_245757	metal	metal
Au1Sn1Tm1_ICSD_245758	metal	metal
Au1Sn1Tm1_ICSD_416420	metal	metal
Au1Sn1Tm1_ICSD_58592	metal	metal
Au1Sn1U1_ICSD_612359	metal	metal
Au1Sn1U1_ICSD_657567	metal	metal
Au1Sn1Y1_ICSD_415826	metal	metal
Au1Sn1Y1_ICSD_612362	metal	metal
Au1Sn1_ICSD_612356	metal	metal
Au1Sn2_ICSD_58587	metal	metal
Au1Sn4_ICSD_612348	metal	metal
Au1Sr1_ICSD_58594	metal	metal
Au1Ta2_ICSD_612373	metal	metal
Au1Ta3_ICSD_58599	metal	metal
Au1Tb1_ICSD_58600	metal	metal
Au1Tb1_ICSD_612379	metal	metal
Au1Tb2_ICSD_612381	metal	metal
Au1Te2_ICSD_30905	metal	metal
Au1Te2_ICSD_612391	metal	metal
Au1Te2_ICSD_659326	metal	metal
Au1Te2_ICSD_659331	metal	metal
Au1Th1_ICSD_601380	metal	metal
Au1Th2_ICSD_58602	metal	metal
Au1Ti1_ICSD_58603	metal	metal
Au1Ti1_ICSD_612407	metal	metal
Au1Ti1_ICSD_612415	metal	metal
Au1Ti3_ICSD_58604	metal	metal
Au1Ti3_ICSD_612417	metal	metal
Au1Tl2_ICSD_102798	metal	metal
Au1Tl3_ICSD_186643	metal	metal
Au1Tm1_ICSD_58608	metal	metal
Au1Tm1_ICSD_612431	metal	metal
Au1Tm1_ICSD_612432	metal	metal
Au1V3_ICSD_58611	metal	metal
Au1V3_ICSD_612459	metal	metal
Au1Y1_ICSD_169022	metal	metal
Au1Y1_ICSD_58617	metal	metal
Au1Y2_ICSD_262044	metal	metal
Au1Yb1Zn1_ICSD_159306	metal	metal
Au1Yb1_ICSD_612473	metal	metal
Au1Yb1_ICSD_612495	metal	metal
Au1Yb2_ICSD_612475	metal	metal

Supplementary Table 93. Five-fold cross validated predictions for the metal/insulator classification (85/598).

system	calculated	predicted
Au1Zn1_ICSD_612506	metal	metal
Au1Zn3_ICSD_58626	metal	metal
Au1Zr2_ICSD_612512	metal	metal
Au1Zr3_ICSD_58630	metal	metal
Au1_ICSD_611625	metal	metal
Au2Ba1F12_ICSD_39316	insulator	insulator
Au2Ba1F8_ICSD_65289	insulator	insulator
Au2Ba1In2_ICSD_249562	metal	metal
Au2Ba1O4_ICSD_80327	insulator	insulator
Au2Ba1Sb2_ICSD_416298	metal	metal
Au2Ba1_ICSD_181532	metal	metal
Au2Ba3_ICSD_611635	metal	metal
Au2Be1_ICSD_150581	metal	metal
Au2Bi1Dy5_ICSD_156957	metal	metal
Au2Bi1Er5_ICSD_156959	metal	metal
Au2Bi1Ho5_ICSD_156958	metal	metal
Au2Bi1Tb5_ICSD_156956	metal	metal
Au2Bi1_ICSD_52284	metal	metal
Au2Br2H12N4_ICSD_80216	insulator	insulator
Au2Br6Cs2_ICSD_170696	insulator	insulator
Au2Br6Rb2_ICSD_9577	insulator	insulator
Au2Br6_ICSD_4069	insulator	insulator
Au2C10Cl8S8_ICSD_65114	insulator	insulator
Au2Ca1F12_ICSD_39315	insulator	insulator
Au2Ca1Ge2_ICSD_25333	metal	metal
Au2Ca1O4_ICSD_79801	insulator	insulator
Au2Ca1Si2_ICSD_412	metal	metal
Au2Ca1_ICSD_55542	metal	metal
Au2Ca2Pb1_ICSD_409531	metal	metal
Au2Ca5_ICSD_58403	metal	metal
Au2Cd1F12_ICSD_415873	insulator	insulator
Au2Cd1F8_ICSD_85413	insulator	insulator
Au2Cd2Rb2S4_ICSD_85582	insulator	insulator
Au2Ce1Ge2_ICSD_246610	metal	metal
Au2Ce1In1_ICSD_611714	metal	metal
Au2Ce1Si2_ICSD_418530	metal	metal
Au2Ce1Si2_ICSD_611726	metal	metal
Au2Ce1_ICSD_611706	metal	metal
Au2Ce3Sb3_ICSD_163719	metal	metal
Au2Cl6Cs2_ICSD_417370	insulator	insulator
Au2Cl6Cs2_ICSD_56470	insulator	insulator
Au2Cs1F7_ICSD_152057	insulator	insulator
Au2Cs2I6_ICSD_186066	insulator	insulator
Au2Cs2Se3_ICSD_85708	insulator	insulator
Au2Dy1In1_ICSD_58442	metal	metal
Au2Dy1Si2_ICSD_106691	metal	metal
Au2Dy1_ICSD_58440	metal	metal
Au2Dy2In1_ICSD_106280	metal	metal
Au2Dy5Sb1_ICSD_156952	metal	metal
Au2Er1In1_ICSD_58449	metal	metal

Supplementary Table 94. Five-fold cross validated predictions for the metal/insulator classification (86/598).

system	calculated	predicted
Au2Er1Si2_ICSD_106692	metal	metal
Au2Er1_ICSD_611807	metal	metal
Au2Er2In1_ICSD_658840	metal	metal
Au2Er2Sn1_ICSD_658830	metal	metal
Au2Er5Sb1_ICSD_156954	metal	metal
Au2Er7Te2_ICSD_168470	metal	metal
Au2Eu1Ge2_ICSD_421997	metal	metal
Au2Eu1Si2_ICSD_106690	metal	metal
Au2Eu1Si2_ICSD_611854	metal	metal
Au2Eu1_ICSD_611839	metal	metal
Au2Eu2Sn5_ICSD_410884	metal	metal
Au2F11H1Mg1_ICSD_415872	insulator	insulator
Au2F8Hg1_ICSD_85414	insulator	insulator
Au2F8Mg1_ICSD_65287	insulator	insulator
Au2F8Ni1_ICSD_65288	insulator	insulator
Au2F8Zn1_ICSD_65286	insulator	insulator
Au2Ga1_ICSD_58459	metal	metal
Au2Ga2Sr1_ICSD_370004	metal	metal
Au2Gd1In1_ICSD_185965	metal	metal
Au2Gd1In1_ICSD_58466	metal	metal
Au2Gd1Si2_ICSD_52667	metal	metal
Au2Gd1Si2_ICSD_55941	metal	metal
Au2Gd1Si2_ICSD_611929	metal	metal
Au2Gd1_ICSD_611904	metal	metal
Au2Gd1_ICSD_611914	metal	metal
Au2Gd2Sn1_ICSD_658833	metal	metal
Au2Ge2Nd1_ICSD_164728	metal	metal
Au2Ge2Sr1_ICSD_25334	metal	metal
Au2Ge2Th1_ICSD_52670	metal	metal
Au2Hf1In1_ICSD_58472	metal	metal
Au2Hf1_ICSD_611956	metal	metal
Au2Ho1In1_ICSD_58486	metal	metal
Au2Ho1Si2_ICSD_55944	metal	metal
Au2Ho1_ICSD_611984	metal	metal
Au2Ho2In1_ICSD_658839	metal	metal
Au2Ho2Sn1_ICSD_658829	metal	metal
Au2Ho5Sb1_ICSD_156953	metal	metal
Au2Ho7Te2_ICSD_262392	metal	metal
Au2In1Lu1_ICSD_58498	metal	metal
Au2In1Nd1_ICSD_58500	metal	metal
Au2In1Pr1_ICSD_58503	metal	metal
Au2In1Sc1_ICSD_58504	metal	metal
Au2In1Sm1_ICSD_58506	metal	metal
Au2In1Tb1_ICSD_58508	metal	metal
Au2In1Tb2_ICSD_658837	metal	metal
Au2In1Th1_ICSD_58509	metal	metal
Au2In1Ti1_ICSD_58510	metal	metal
Au2In1U1_ICSD_612076	metal	metal
Au2In1Y1_ICSD_58514	metal	metal
Au2In1Y2_ICSD_658835	metal	metal

Supplementary Table 95. Five-fold cross validated predictions for the metal/insulator classification (87/598).

system	calculated	predicted
Au2In1Yb1_ICSD_58516	metal	metal
Au2In1Zr1_ICSD_58517	metal	metal
Au2In2Sr1_ICSD_249563	metal	metal
Au2In4La1_ICSD_249529	metal	metal
Au2In4Nd1_ICSD_249532	metal	metal
Au2In4Pr1_ICSD_249531	metal	metal
Au2In4Yb1_ICSD_261042	metal	metal
Au2K1_ICSD_58520	metal	metal
Au2K2P2Se6_ICSD_165322	insulator	insulator
Au2K2S4Sn1_ICSD_74022	insulator	insulator
Au2La1Si2_ICSD_106686	metal	metal
Au2La1_ICSD_612096	metal	metal
Au2La4O9_ICSD_74989	insulator	insulator
Au2Lu1_ICSD_612125	metal	metal
Au2Lu5Te2_ICSD_261995	metal	metal
Au2Mn1_ICSD_612163	metal	metal
Au2Na1_ICSD_612178	metal	metal
Au2Nb1_ICSD_58558	metal	metal
Au2Nb3_ICSD_58559	metal	metal
Au2Nb3_ICSD_612191	metal	metal
Au2Nd1Si2_ICSD_106689	metal	metal
Au2Nd1Si2_ICSD_55939	metal	metal
Au2O10Se3_ICSD_170260	insulator	insulator
Au2O11Se4_ICSD_15495	insulator	insulator
Au2O3_ICSD_8014	insulator	insulator
Au2O4Sr1_ICSD_80328	insulator	insulator
Au2O7Se2_ICSD_37009	insulator	insulator
Au2P2Pb1_ICSD_412224	metal	metal
Au2P2Se6Tl2_ICSD_171216	insulator	insulator
Au2P3_ICSD_8058	insulator	insulator
Au2Pb1Yb2_ICSD_409532	metal	metal
Au2Pb1_ICSD_56261	metal	metal
Au2Pr1Si2_ICSD_106688	metal	metal
Au2Pr1_ICSD_58577	metal	metal
Au2Pu1Si2_ICSD_73040	metal	metal
Au2S1_ICSD_612282	insulator	metal
Au2Sb1Tb5_ICSD_156951	metal	metal
Au2Sc1_ICSD_612299	metal	metal
Au2Si2Sm1_ICSD_52675	metal	metal
Au2Si2Sr1_ICSD_413	metal	metal
Au2Si2Tb1_ICSD_55818	metal	metal
Au2Si2Th1_ICSD_52676	metal	metal
Au2Si2U1_ICSD_52677	metal	metal
Au2Si2U1_ICSD_612325	metal	metal
Au2Si2Y1_ICSD_55943	metal	metal
Au2Si2Yb1_ICSD_52678	metal	metal
Au2Sn1Tb2_ICSD_658834	metal	metal
Au2Sn1U1_ICSD_58593	metal	metal
Au2Sr1_ICSD_55543	metal	metal
Au2Sr3_ICSD_58596	metal	metal

Supplementary Table 96. Five-fold cross validated predictions for the metal/insulator classification (88/598).

system	calculated	predicted
Au2Tb1_ICSD_612375	metal	metal
Au2Th1_ICSD_15444	metal	metal
Au2Ti1_ICSD_58607	metal	metal
Au2Tm1_ICSD_58387	metal	metal
Au2Tm1_ICSD_612427	metal	metal
Au2Tm1_ICSD_612435	metal	metal
Au2Tm1_ICSD_612437	metal	metal
Au2U1_ICSD_58609	metal	metal
Au2U1_ICSD_612443	metal	metal
Au2V1_ICSD_58614	metal	metal
Au2Y1_ICSD_612462	metal	metal
Au2Y3_ICSD_262043	metal	metal
Au2Yb1_ICSD_58388	metal	metal
Au2Zr1_ICSD_612510	metal	metal
Au31Mn9_ICSD_58552	metal	metal
Au3C6Co1K1N6_ICSD_201056	insulator	insulator
Au3C6H6La1N6O3_ICSD_170989	insulator	insulator
Au3C6K1N6Ni1_ICSD_249724	insulator	insulator
Au3Ca1Ga1_ICSD_249634	metal	metal
Au3Ca2In4_ICSD_410702	metal	metal
Au3Ca3In1_ICSD_418239	metal	metal
Au3Ca5_ICSD_58404	metal	metal
Au3Cd5_ICSD_58416	metal	metal
Au3Cl8Rb3_ICSD_9578	insulator	insulator
Au3Cs1Ga2_ICSD_263058	metal	metal
Au3Cu1_ICSD_56266	metal	metal
Au3Dy1_ICSD_611784	metal	metal
Au3Dy3Sb4_ICSD_611802	insulator	insulator
Au3Er1_ICSD_611810	metal	metal
Au3Er3Ga8_ICSD_611824	metal	metal
Au3Er3Sb4_ICSD_611833	insulator	insulator
Au3Eu1In3_ICSD_245680	metal	metal
Au3F12La1_ICSD_78915	insulator	insulator
Au3Ga2Rb1_ICSD_263057	metal	metal
Au3Ga7Yb1_ICSD_402170	metal	metal
Au3Gd3Sb4_ICSD_611925	insulator	insulator
Au3Ge1Na1_ICSD_16463	insulator	metal
Au3Ge1Sr1_ICSD_262386	metal	metal
Au3Hf1_ICSD_611955	metal	metal
Au3Ho1_ICSD_611983	metal	metal
Au3Ho3Sb4_ICSD_612001	insulator	insulator
Au3In1_ICSD_612031	metal	metal
Au3In2_ICSD_612030	metal	metal
Au3In3Sr1_ICSD_245679	metal	metal
Au3In3Yb3_ICSD_261318	metal	metal
Au3K1Sn3_ICSD_249645	metal	metal
Au3K2_ICSD_65113	metal	metal
Au3K3Sb2_ICSD_78977	insulator	insulator
Au3La3Sb4_ICSD_612105	insulator	metal
Au3Li1Na2_ICSD_152090	metal	metal

Supplementary Table 97. Five-fold cross validated predictions for the metal/insulator classification (89/598).

system	calculated	predicted
Au3Li1Sn4_ICSD_412207	metal	metal
Au3Li1_ICSD_58525	metal	metal
Au3Lu1_ICSD_612128	metal	metal
Au3Lu3Sb4_ICSD_612134	insulator	insulator
Au3Na1Si1_ICSD_16462	insulator	metal
Au3Nd3Sb4_ICSD_612220	metal	metal
Au3O2Rb5_ICSD_91308	insulator	insulator
Au3P7Sn1_ICSD_416407	insulator	insulator
Au3Pd1_ICSD_180872	metal	metal
Au3Pr3Sb4_ICSD_612269	metal	metal
Au3Rb1Se2_ICSD_82541	insulator	metal
Au3Rb2Tl1_ICSD_249924	metal	metal
Au3Rb2_ICSD_106288	metal	metal
Au3Rb3Sb2_ICSD_78978	insulator	insulator
Au3Sb4Sm3_ICSD_612289	metal	metal
Au3Sb4Tb3_ICSD_612293	insulator	insulator
Au3Sb4Y3_ICSD_957	insulator	insulator
Au3Sm1_ICSD_58585	metal	metal
Au3Sn2Yb2_ICSD_710044	metal	metal
Au3Sn4U3_ICSD_612361	metal	metal
Au3Sr7_ICSD_58597	metal	metal
Au3Tb1_ICSD_612380	metal	metal
Au3Y1_ICSD_612464	metal	metal
Au3Yb1_ICSD_612472	metal	metal
Au3Yb5_ICSD_58621	metal	metal
Au3Yb7_ICSD_58622	metal	metal
Au3Zn1_ICSD_654236	metal	metal
Au3Zr1_ICSD_612509	metal	metal
Au4Ca1Cd2_ICSD_424033	metal	metal
Au4Ca3_ICSD_54547	metal	metal
Au4Ca5_ICSD_58406	metal	metal
Au4Cd1K2S4_ICSD_85583	insulator	insulator
Au4Ce1Si2_ICSD_154801	metal	metal
Au4Ce1Si2_ICSD_418529	metal	metal
Au4Cl8_ICSD_201436	insulator	insulator
Au4Cr1_ICSD_611737	metal	metal
Au4Er1_ICSD_611812	metal	metal
Au4Hf1_ICSD_611961	metal	metal
Au4Ho1_ICSD_58483	metal	metal
Au4In2K1_ICSD_249221	metal	metal
Au4In2Rb1_ICSD_249220	metal	metal
Au4In6K1_ICSD_249520	metal	metal
Au4In6Rb1_ICSD_249521	metal	metal
Au4K1Sn2_ICSD_58522	metal	metal
Au4K1Sn6_ICSD_249644	metal	metal
Au4Li15_ICSD_150973	metal	metal
Au4Lu1_ICSD_612131	metal	metal
Au4Mn1_ICSD_657182	metal	metal
Au4S3Tl2_ICSD_51235	insulator	insulator
Au4Sc1_ICSD_612300	metal	metal

Supplementary Table 98. Five-fold cross validated predictions for the metal/insulator classification (90/598).

system	calculated	predicted
Au4Th3_ICSD_601382	metal	metal
Au4Ti1_ICSD_612411	metal	metal
Au4V1_ICSD_612460	metal	metal
Au4Yb1_ICSD_612477	metal	metal
Au4Yb5_ICSD_58624	metal	metal
Au4Zr1_ICSD_58631	metal	metal
Au5Ba1_ICSD_58395	metal	metal
Au5Ca1_ICSD_58407	metal	metal
Au5Cd8_ICSD_611679	metal	metal
Au5Cs7O2_ICSD_411334	insulator	insulator
Au5Cs7O2_ICSD_95821	insulator	insulator
Au5Eu1_ICSD_58452	metal	metal
Au5In1K3_ICSD_249922	metal	metal
Au5K1_ICSD_106286	metal	metal
Au5K3Pb1_ICSD_107450	metal	metal
Au5Mn2_ICSD_612154	metal	metal
Au5O2Rb7_ICSD_95825	insulator	insulator
Au5Rb1_ICSD_612281	metal	metal
Au5Sn1_ICSD_58589	metal	metal
Au5Sr1_ICSD_612363	metal	metal
Au6Ba8Ge40_ICSD_181524	metal	metal
Au6Ba8Si40_ICSD_186541	metal	metal
Au6Dy1_ICSD_611790	metal	metal
Au6Ga3Sr2_ICSD_426465	metal	metal
Au6Hg5_ICSD_150475	metal	metal
Au6Ho1_ICSD_611991	metal	metal
Au6In5Na2_ICSD_55545	metal	metal
Au6Nd1_ICSD_612210	metal	metal
Au6Pr1_ICSD_2125	metal	metal
Au6Pr3Sn5_ICSD_419491	metal	metal
Au6Rb4S5_ICSD_82556	insulator	insulator
Au6Sr2Zn3_ICSD_426464	metal	metal
Au6Tb1_ICSD_612383	metal	metal
Au7Cd16Na6_ICSD_261960	metal	metal
Au7Cs4Sn2_ICSD_107449	metal	metal
Au7Ga2_ICSD_654822	metal	metal
Au7Ge2K4_ICSD_79111	metal	metal
Au7I1P10_ICSD_12162	insulator	insulator
Au7I1P10_ICSD_8059	insulator	insulator
Au7Rb3_ICSD_106291	metal	metal
Au7Rb4Sn2_ICSD_58581	metal	metal
Au9In4_ICSD_58494	metal	metal
B0.667N0.667_ICSD_162872	insulator	insulator
B10Ba2H2Na2O19_ICSD_95447	insulator	insulator
B10Ca1La2O19_ICSD_92866	insulator	insulator
B10Ce2Ni1_ICSD_91245	metal	metal
B10Co1Th2_ICSD_78822	metal	metal
B10Fe1Th2_ICSD_78821	metal	metal
B10Ho3Ni19_ICSD_39286	metal	metal
B10Nd2Ni1_ICSD_91246	metal	metal

Supplementary Table 99. Five-fold cross validated predictions for the metal/insulator classification (91/598).

system	calculated	predicted
B10Ni1Th2_ICSD_78823	metal	metal
B10O21Pb6_ICSD_2641	insulator	insulator
B11Li1_ICSD_164841	metal	metal
B11Li1_ICSD_164842	metal	metal
B11Li1_ICSD_164843	metal	metal
B11Li1_ICSD_164845	metal	metal
B12Ba7Ir12_ICSD_8156	metal	metal
B12Be1C2_ICSD_612548	metal	insulator
B12Br1Cs3H12_ICSD_414584	insulator	insulator
B12Br1H12K3_ICSD_414581	insulator	insulator
B12Br1H12Rb3_ICSD_414583	insulator	insulator
B12C2Li2_ICSD_415556	insulator	metal
B12C2Mg1_ICSD_416801	insulator	insulator
B12C3_ICSD_612562	insulator	metal
B12Cl1Cs3H12_ICSD_414586	insulator	insulator
B12Cl1H12Rb3_ICSD_414585	insulator	insulator
B12Cs2H12_ICSD_92501	insulator	insulator
B12Cs3H12I1_ICSD_98622	insulator	insulator
B12Dy1_ICSD_613592	metal	metal
B12Er1_ICSD_613695	metal	metal
B12Gd1_ICSD_614314	metal	metal
B12H12I1K3_ICSD_98619	insulator	insulator
B12H12I1Rb3_ICSD_98620	insulator	insulator
B12H12K2_ICSD_36148	insulator	insulator
B12H12Rb2_ICSD_20015	insulator	insulator
B12H12Tl2_ICSD_422433	insulator	insulator
B12H20Li2O4_ICSD_163690	insulator	insulator
B12H20N2_ICSD_98618	insulator	insulator
B12Hf1_ICSD_614421	metal	metal
B12Ho1_ICSD_614457	metal	metal
B12Lu1_ICSD_600741	metal	metal
B12Ni1_ICSD_614986	insulator	metal
B12O24Se2Zn8_ICSD_74057	insulator	insulator
B12P2_ICSD_62748	insulator	insulator
B12Pu1_ICSD_615219	metal	metal
B12S1_ICSD_150626	metal	insulator
B12Sc1_ICSD_615424	metal	metal
B12Sc1_ICSD_68028	metal	metal
B12Si3_ICSD_615435	metal	insulator
B12Tb1_ICSD_615534	metal	metal
B12Th1_ICSD_615581	metal	metal
B12Tm1_ICSD_615607	metal	metal
B12U1_ICSD_615635	metal	metal
B12Y1_ICSD_23860	metal	metal
B12Yb1_ICSD_603063	metal	metal
B12Zr1_ICSD_615752	metal	metal
B13C2Li1_ICSD_415557	insulator	metal
B13C2_ICSD_612568	metal	insulator
B13Co1Tb4_ICSD_44183	metal	metal
B13Co1Y4_ICSD_39466	metal	metal

Supplementary Table 100. Five-fold cross validated predictions for the metal/insulator classification (92/598).

system	calculated	predicted
B13Er4Ni1_ICSD_20700	metal	metal
B13N2_ICSD_162801	metal	insulator
B14Ho4Ni1_ICSD_409535	metal	metal
B16Ir19Mg10_ICSD_163909	metal	metal
B18Hg2Rb4Se18_ICSD_410758	insulator	insulator
B1Ba1Bi1O4_ICSD_154105	insulator	insulator
B1Ba1Bi1S4_ICSD_248222	insulator	insulator
B1Ba1Cl1F4_ICSD_188654	insulator	insulator
B1Ba1Cu1La1O5_ICSD_75318	metal	insulator
B1Ba1Cu1Nd1O5_ICSD_94205	metal	metal
B1Ba1F10P1_ICSD_420597	insulator	insulator
B1Ba1F1O3Zn1_ICSD_248042	insulator	insulator
B1Ba1F2Ga1O3_ICSD_91315	insulator	insulator
B1Ba1F3O1_ICSD_16684	insulator	insulator
B1Ba1Li1O3_ICSD_73218	insulator	insulator
B1Ba1Li1O3_ICSD_92843	insulator	insulator
B1Ba1Li1S3_ICSD_82352	insulator	insulator
B1Ba1Na1O3_ICSD_250086	insulator	insulator
B1Ba1O5P1_ICSD_50875	insulator	insulator
B1Ba1O5P1_ICSD_99842	insulator	insulator
B1Ba1Pd4_ICSD_247264	metal	metal
B1Ba1S4Sb1_ICSD_248221	insulator	insulator
B1Ba2Cl17Zr6_ICSD_203141	insulator	insulator
B1Ba2Cl1N2_ICSD_418947	insulator	insulator
B1Ba3O3P1_ICSD_402017	insulator	insulator
B1Ba3O7P1_ICSD_150328	insulator	insulator
B1Be2Cs1F2O3_ICSD_20000	insulator	insulator
B1Be2F1O3_ICSD_200264	insulator	insulator
B1Be2F1O3_ICSD_56847	insulator	insulator
B1Be2F2K1O3_ICSD_16178	insulator	insulator
B1Be2F2K1O3_ICSD_77277	insulator	insulator
B1Be2F2Na1O3_ICSD_75594	insulator	insulator
B1Be2F2O3Rb1_ICSD_260439	insulator	insulator
B1Be2F2O3Rb1_ICSD_4223	insulator	insulator
B1Be2H1O4_ICSD_34650	insulator	insulator
B1Be2_ICSD_108027	metal	metal
B1Bi1O3_ICSD_413621	insulator	insulator
B1Bi1O4Pb1_ICSD_183393	insulator	insulator
B1Bi1O4Pb1_ICSD_416639	insulator	insulator
B1Bi1_ICSD_184569	insulator	metal
B1Br1C4F4H11N1_ICSD_249155	insulator	insulator
B1Br1Eu2N2_ICSD_409982	insulator	insulator
B1Br1Mg2N2_ICSD_261620	insulator	insulator
B1Br1N2Sr2_ICSD_261795	insulator	insulator
B1Br20K8Zr6_ICSD_165310	insulator	insulator
B1Br3I3P1_ICSD_411438	insulator	insulator
B1Br3P4S3_ICSD_165186	insulator	insulator
B1Br3_ICSD_173374	insulator	insulator
B1C1Ca3Cl2N1_ICSD_33850	insulator	insulator
B1C1Cl2N1Sr3_ICSD_74914	insulator	insulator

Supplementary Table 101. Five-fold cross validated predictions for the metal/insulator classification (93/598).

system	calculated	predicted
B1C1Cl3La3_ICSD_415102	metal	metal
B1C1Dy1_ICSD_42477	metal	metal
B1C1F4H6N3_ICSD_202434	insulator	insulator
B1C1F6K1_ICSD_1194	insulator	insulator
B1C1Gd1_ICSD_612618	metal	metal
B1C1H5N2_ICSD_2165	insulator	insulator
B1C1Ho1_ICSD_612623	metal	metal
B1C1Li1_ICSD_245288	insulator	metal
B1C1Lu1Ni1_ICSD_75610	metal	metal
B1C1Mo2_ICSD_43318	metal	metal
B1C1Ni1Y1_ICSD_85283	metal	metal
B1C1Th1_ICSD_2368	metal	metal
B1C1U1_ICSD_612669	metal	metal
B1C1Y1_ICSD_42626	metal	metal
B1C2Cs1F8_ICSD_200869	insulator	insulator
B1C2N1_ICSD_93040	insulator	insulator
B1C2N1_ICSD_93041	insulator	insulator
B1C2Sc2_ICSD_88804	metal	metal
B1C3.33N1_ICSD_161278	insulator	insulator
B1C3Cs1F9N1O2_ICSD_410079	insulator	insulator
B1C3F4H10N1_ICSD_171202	insulator	insulator
B1C3H12N1_ICSD_249799	insulator	insulator
B1C3K2N3_ICSD_262588	insulator	insulator
B1C3Lu3_ICSD_411641	metal	metal
B1C4Cl1F4H11N1_ICSD_320194	insulator	insulator
B1C4Cs1N4_ICSD_414556	insulator	insulator
B1C4Cu1N4_ICSD_414557	insulator	insulator
B1C4H4N5_ICSD_414561	insulator	insulator
B1C4K1N4_ICSD_411180	insulator	insulator
B1C4K1O8_ICSD_281621	insulator	insulator
B1C4Li1N4_ICSD_414558	insulator	insulator
B1C4N4Na1_ICSD_414559	insulator	insulator
B1C4N4Rb1_ICSD_414563	insulator	insulator
B1C4N4Tl1_ICSD_414564	insulator	insulator
B1C4Na1O8_ICSD_281622	insulator	insulator
B1C5_ICSD_166553	metal	metal
B1C5_ICSD_166554	metal	metal
B1C5_ICSD_166555	metal	metal
B1C5_ICSD_166556	metal	metal
B1C7_ICSD_181952	metal	metal
B1C7_ICSD_181953	metal	metal
B1C7_ICSD_181954	metal	metal
B1C7_ICSD_181955	metal	metal
B1C7_ICSD_181956	metal	metal
B1Ca1Ga1O4_ICSD_93390	insulator	insulator
B1Ca1H1O3_ICSD_181633	insulator	insulator
B1Ca1H1O5Si1_ICSD_168627	insulator	insulator
B1Ca1H3Ni1O10P2_ICSD_409898	insulator	insulator
B1Ca1Li1O3_ICSD_99386	insulator	insulator
B1Ca1Ni4_ICSD_36504	metal	metal

Supplementary Table 102. Five-fold cross validated predictions for the metal/insulator classification (94/598).

system	calculated	predicted
B1Ca1O5P1_ICSD_87893	insulator	insulator
B1Ca2Cl1N2_ICSD_406361	insulator	insulator
B1Ca2Cl1O3_ICSD_342	insulator	insulator
B1Ca2F1N2_ICSD_50842	insulator	insulator
B1Ca2H1N2_ICSD_414006	insulator	insulator
B1Ca3N3_ICSD_95814	insulator	insulator
B1Cd1Cs1O3_ICSD_189199	insulator	insulator
B1Cd1Li1O3_ICSD_200615	insulator	insulator
B1Cd1Li1O3_ICSD_20191	insulator	insulator
B1Ce1Co4_ICSD_42101	metal	metal
B1Ce1N2_ICSD_410417	insulator	insulator
B1Ce1O3_ICSD_99689	insulator	insulator
B1Ce1O3_ICSD_99690	insulator	insulator
B1Ce1O5Si1_ICSD_28026	insulator	insulator
B1Ce1Pd3_ICSD_612819	metal	metal
B1Ce1Pt2_ICSD_90403	metal	metal
B1Ce1Pt3_ICSD_95051	metal	metal
B1Ce1Pt4_ICSD_55453	metal	metal
B1Ce1Rh3_ICSD_612823	metal	metal
B1Ce1S3_ICSD_421071	insulator	insulator
B1Cl1Cu1H4O4_ICSD_56963	metal	insulator
B1Cl1F4O2_ICSD_166598	insulator	insulator
B1Cl1F6_ICSD_68277	insulator	insulator
B1Cl1H4Na2O4_ICSD_32518	insulator	insulator
B1Cl1Mg2N2_ICSD_413908	insulator	insulator
B1Cl1N2Sr2_ICSD_50845	insulator	insulator
B1Cl2H4K1Zn1_ICSD_174294	insulator	insulator
B1Cl3_ICSD_27869	insulator	insulator
B1Cl6Sc4_ICSD_201977	metal	metal
B1Co1Mo1_ICSD_42894	metal	metal
B1Co1Re1_ICSD_20079	metal	metal
B1Co1W1_ICSD_613388	metal	metal
B1Co1_ICSD_612863	metal	metal
B1Co2Fe1_ICSD_603579	metal	metal
B1Co2_ICSD_42531	metal	metal
B1Co2_ICSD_612878	metal	metal
B1Co3Fe1Y1_ICSD_613036	metal	metal
B1Co3O5_ICSD_93484	insulator	insulator
B1Co3O7P1_ICSD_51317	insulator	insulator
B1Co3_ICSD_44339	metal	metal
B1Co3_ICSD_612882	metal	metal
B1Co4Dy1_ICSD_612899	metal	metal
B1Co4Er1_ICSD_612926	metal	metal
B1Co4Ho1_ICSD_613107	metal	metal
B1Co4La1_ICSD_613133	metal	metal
B1Co4Nd1_ICSD_157353	metal	metal
B1Co4Nd1_ICSD_97606	metal	metal
B1Co4Pr1_ICSD_97722	metal	metal
B1Co4Tb1_ICSD_613336	metal	metal
B1Co4Y1_ICSD_51272	metal	metal

Supplementary Table 103. Five-fold cross validated predictions for the metal/insulator classification (95/598).

system	calculated	predicted
B1Co4Y1_ICSD_613437	metal	metal
B1Cr1O3_ICSD_43311	insulator	insulator
B1Cr1O4Pb1_ICSD_97663	insulator	insulator
B1Cr1_ICSD_613479	metal	metal
B1Cr2O12P3_ICSD_409459	insulator	insulator
B1Cr2_ICSD_188474	metal	metal
B1Cr2_ICSD_76127	metal	metal
B1Cr5Si3_ICSD_613552	metal	metal
B1Cs1F4_ICSD_95828	insulator	insulator
B1Cs1H4_ICSD_27864	metal	metal
B1Cs1Li2S3_ICSD_411530	insulator	insulator
B1Cs1Na2O3_ICSD_67524	insulator	insulator
B1Cs1Se3_ICSD_411342	insulator	insulator
B1Cs3P2_ICSD_300123	insulator	insulator
B1Cs3S3_ICSD_391170	insulator	insulator
B1Cu1Ir1_ICSD_75029	metal	metal
B1Cu1S2_ICSD_156413	insulator	insulator
B1Cu1Se2_ICSD_613591	insulator	insulator
B1Cu2H5O6_ICSD_54883	insulator	insulator
B1Dy1Ni4_ICSD_613650	metal	metal
B1Dy1O3_ICSD_59849	insulator	insulator
B1Dy1Pd3_ICSD_44210	metal	metal
B1Dy1Rh3_ICSD_44211	metal	metal
B1Dy3O9W1_ICSD_250419	insulator	insulator
B1Dy5Ge3_ICSD_613639	metal	metal
B1Dy5Si3_ICSD_415746	metal	metal
B1Er1Fe4_ICSD_44215	metal	metal
B1Er1Ni4_ICSD_156976	metal	metal
B1Er1Pd3_ICSD_44233	metal	metal
B1Er1Rh3_ICSD_44237	metal	metal
B1Eu1Pd3_ICSD_44241	metal	metal
B1Eu1Rh3_ICSD_44242	metal	metal
B1F1H4N1O4P1_ICSD_170949	insulator	insulator
B1F1N2Sr2_ICSD_50843	insulator	insulator
B1F3H4O2_ICSD_26551	insulator	insulator
B1F3Mg3O3_ICSD_4226	insulator	insulator
B1F3Na2O4S1_ICSD_424150	insulator	insulator
B1F3_ICSD_24783	insulator	insulator
B1F4H2N3S3_ICSD_201705	insulator	insulator
B1F4H3O1_ICSD_15591	insulator	insulator
B1F4H4N1_ICSD_93978	insulator	insulator
B1F4H5N2_ICSD_245055	insulator	insulator
B1F4In1_ICSD_50218	insulator	insulator
B1F4K1_ICSD_22260	insulator	insulator
B1F4Li1_ICSD_171375	insulator	insulator
B1F4N1O2_ICSD_240721	insulator	insulator
B1F4Na1_ICSD_30349	insulator	insulator
B1F4Rb1_ICSD_24016	insulator	insulator
B1F4Tl1_ICSD_300222	insulator	insulator
B1F5Li2_ICSD_426821	insulator	insulator

Supplementary Table 104. Five-fold cross validated predictions for the metal/insulator classification (96/598).

system	calculated	predicted
B1F7N2O1S2_ICSD_414492	insulator	insulator
B1F7S1_ICSD_10337	insulator	insulator
B1F7Sn2_ICSD_15263	insulator	insulator
B1F8N1_ICSD_165425	insulator	insulator
B1F8N1_ICSD_63311	insulator	insulator
B1F9Sn3_ICSD_15264	insulator	insulator
B1Fe1Nb1_ICSD_20298	metal	metal
B1Fe1Nb1_ICSD_614046	metal	metal
B1Fe1Ni2O5_ICSD_69615	insulator	insulator
B1Fe1O3_ICSD_34474	insulator	insulator
B1Fe1O4Pb1_ICSD_97668	insulator	insulator
B1Fe1Ta1_ICSD_614205	metal	metal
B1Fe1W1_ICSD_614256	metal	metal
B1Fe1_ICSD_391329	metal	metal
B1Fe1_ICSD_613898	metal	metal
B1Fe2Ni1_ICSD_614129	metal	metal
B1Fe2O12P3_ICSD_260895	insulator	insulator
B1Fe2O4_ICSD_88450	insulator	insulator
B1Fe2_ICSD_160789	metal	metal
B1Fe2_ICSD_160790	metal	metal
B1Fe2_ICSD_16809	metal	metal
B1Fe2_ICSD_613907	metal	metal
B1Fe3O5_ICSD_164815	insulator	insulator
B1Fe3O5_ICSD_25101	metal	insulator
B1Fe3O6_ICSD_23863	insulator	insulator
B1Fe3_ICSD_184958	metal	metal
B1Fe3_ICSD_260757	metal	metal
B1Fe4Lu1_ICSD_614001	metal	metal
B1Ga1O4Pb1_ICSD_279600	insulator	insulator
B1Gd1N2_ICSD_657369	insulator	insulator
B1Gd1O3_ICSD_87779	insulator	insulator
B1Gd1Pd3_ICSD_163775	metal	metal
B1Gd1Pd3_ICSD_44309	metal	metal
B1Gd1Rh3_ICSD_44310	metal	metal
B1Gd1Rh3_ICSD_614376	metal	metal
B1Gd3O9W1_ICSD_250417	insulator	insulator
B1Gd5Si3_ICSD_614390	metal	metal
B1Ge1La1O5_ICSD_39262	insulator	insulator
B1Ge1Li1O4_ICSD_28106	insulator	insulator
B1Ge1Li1O4_ICSD_67535	insulator	insulator
B1Ge2K1O6_ICSD_281258	insulator	insulator
B1Ge2Ni6_ICSD_185118	metal	metal
B1Ge3La5_ICSD_614396	metal	metal
B1Ge3Nb5_ICSD_614400	metal	metal
B1Ge3Ta5_ICSD_614404	metal	metal
B1Ge3Tb5_ICSD_614406	metal	metal
B1Ge3V5_ICSD_614407	metal	metal
B1H10Li4N3_ICSD_161018	insulator	insulator
B1H1Li1_ICSD_153290	metal	insulator
B1H1Na2O3_ICSD_27211	insulator	insulator

Supplementary Table 105. Five-fold cross validated predictions for the metal/insulator classification (97/598).

system	calculated	predicted
B1H1O2_ICSD_34639	insulator	insulator
B1H2Li1_ICSD_153289	insulator	insulator
B1H2Na1O7Si2_ICSD_12134	insulator	insulator
B1H3In1Na1O10P2_ICSD_409585	insulator	insulator
B1H3Mg3O6_ICSD_250420	insulator	insulator
B1H3Na1O10P2V1_ICSD_409651	insulator	insulator
B1H3O3_ICSD_52290	insulator	insulator
B1H4K1_ICSD_160985	insulator	insulator
B1H4K1_ICSD_160986	insulator	insulator
B1H4Li1_ICSD_173101	insulator	insulator
B1H4Li1_ICSD_186262	insulator	insulator
B1H4Li1_ICSD_245569	insulator	insulator
B1H4Li1_ICSD_95207	insulator	insulator
B1H4N1O8P2Zn1_ICSD_410872	insulator	insulator
B1H4Na1O4_ICSD_167112	insulator	insulator
B1H4Na1_ICSD_159243	insulator	insulator
B1H4Na1_ICSD_181024	insulator	insulator
B1H4Na1_ICSD_182733	insulator	insulator
B1H4Na1_ICSD_182734	insulator	insulator
B1H4Na1_ICSD_261750	insulator	insulator
B1H4Na1_ICSD_261751	insulator	insulator
B1H5In1N1O9P2_ICSD_409584	insulator	insulator
B1H5Li1N1_ICSD_180548	insulator	insulator
B1H6N1_ICSD_165975	insulator	insulator
B1H6N1_ICSD_180547	insulator	insulator
B1H6N1_ICSD_181423	insulator	insulator
B1H7Li1N1_ICSD_169560	insulator	insulator
B1Hf1Rh3_ICSD_614447	metal	metal
B1Hf1_ICSD_614418	metal	metal
B1Hf9Mo4_ICSD_23788	metal	metal
B1Hf9Os4_ICSD_614445	metal	metal
B1Hf9Re4_ICSD_614446	metal	metal
B1Ho1Ni4_ICSD_409500	metal	metal
B1Ho1Pd3_ICSD_44417	metal	metal
B1Ho1Rh3_ICSD_44418	metal	metal
B1Ho5Si3_ICSD_415742	metal	metal
B1I12Zr6_ICSD_202103	metal	insulator
B1I1N2Sr2_ICSD_50598	insulator	insulator
B1I3P4S3_ICSD_165187	insulator	insulator
B1I3_ICSD_28328	insulator	insulator
B1In1La3_ICSD_107021	metal	metal
B1In1O3_ICSD_75254	insulator	insulator
B1In1Sc3_ICSD_44421	metal	metal
B1In2O12P3_ICSD_420643	insulator	insulator
B1Ir1Li1_ICSD_75027	metal	metal
B1Ir1Mg1_ICSD_409979	metal	metal
B1Ir1Pd1_ICSD_75030	metal	metal
B1Ir3Sc1_ICSD_44423	metal	metal
B1Ir6Zr2_ICSD_421246	metal	metal
B1K1Li2O3_ICSD_48177	insulator	insulator

Supplementary Table 106. Five-fold cross validated predictions for the metal/insulator classification (98/598).

system	calculated	predicted
B1K1Mg1O3_ICSD_174336	insulator	insulator
B1K1Na2O3_ICSD_33261	insulator	insulator
B1K1O6Si2_ICSD_380488	insulator	insulator
B1K2Li1O3_ICSD_60949	insulator	insulator
B1K2Na1P2_ICSD_300133	insulator	insulator
B1K3O13Sb4_ICSD_411501	insulator	insulator
B1K3P2_ICSD_300104	insulator	insulator
B1K3S3_ICSD_411607	insulator	insulator
B1La1N1Ni1_ICSD_77939	metal	metal
B1La1Ni4_ICSD_170617	metal	metal
B1La1O3_ICSD_15383	insulator	insulator
B1La1O5Si1_ICSD_83397	insulator	insulator
B1La1Pd3_ICSD_614637	metal	metal
B1La1Pt2_ICSD_98425	metal	metal
B1La1Rh3_ICSD_44430	metal	metal
B1La3O9W1_ICSD_39809	insulator	insulator
B1Li1Mn1O3_ICSD_94318	insulator	insulator
B1Li1O2_ICSD_200891	insulator	insulator
B1Li1O2_ICSD_34256	insulator	insulator
B1Li1O3Sr1_ICSD_92842	insulator	insulator
B1Li1O4Si1_ICSD_67536	insulator	insulator
B1Li1Pt3_ICSD_68091	metal	metal
B1Li1S3Sr1_ICSD_380105	insulator	insulator
B1Li1Si2_ICSD_425643	insulator	insulator
B1Li1_ICSD_153291	metal	metal
B1Li2Pd3_ICSD_246447	metal	metal
B1Li2Pt3_ICSD_246448	metal	metal
B1Li3N2_ICSD_155126	insulator	insulator
B1Li3N2_ICSD_155129	insulator	insulator
B1Li3N2_ICSD_655673	insulator	insulator
B1Li3O3_ICSD_9105	insulator	insulator
B1Li3S3_ICSD_75223	insulator	insulator
B1Lu1O3_ICSD_16525	metal	insulator
B1Lu1Pd3_ICSD_44437	metal	metal
B1Lu1Rh3_ICSD_44438	metal	metal
B1Lu5Si3_ICSD_415737	metal	metal
B1Mg1Na1O3_ICSD_249567	insulator	insulator
B1Mg3N3_ICSD_79623	insulator	insulator
B1Mg8Pt4_ICSD_245121	metal	metal
B1Mg8Rh4_ICSD_249110	metal	metal
B1Mn1O4Pb1_ICSD_97664	insulator	insulator
B1Mn1_ICSD_614725	metal	metal
B1Mn1_ICSD_614742	metal	metal
B1Mn2_ICSD_42529	metal	metal
B1Mn2_ICSD_614741	metal	metal
B1Mo1_ICSD_24280	metal	metal
B1Mo1_ICSD_614812	metal	metal
B1Mo2_ICSD_614814	metal	metal
B1Mo4Zr9_ICSD_614862	metal	metal
B1N1U1_ICSD_71067	metal	metal

Supplementary Table 107. Five-fold cross validated predictions for the metal/insulator classification (99/598).

system	calculated	predicted
B1N1_ICSD_162871	insulator	insulator
B1N1_ICSD_162875	insulator	insulator
B1N1_ICSD_162876	insulator	insulator
B1N1_ICSD_162877	insulator	insulator
B1N1_ICSD_162880	insulator	insulator
B1N1_ICSD_162882	insulator	insulator
B1N1_ICSD_162883	insulator	insulator
B1N1_ICSD_186246	insulator	insulator
B1N1_ICSD_27986	metal	insulator
B1N1_ICSD_42002	insulator	insulator
B1N1_ICSD_614873	insulator	insulator
B1N2Na3_ICSD_68619	insulator	insulator
B1N2Nd1_ICSD_657367	insulator	insulator
B1N3Ta2_ICSD_182349	metal	metal
B1Na1O2_ICSD_34645	insulator	insulator
B1Na1O3Sr1_ICSD_172420	insulator	insulator
B1Na1O4Si1_ICSD_39459	insulator	insulator
B1Na1Pt3_ICSD_68092	metal	metal
B1Na2O3Rb1_ICSD_67525	insulator	insulator
B1Na3O3_ICSD_1351	insulator	insulator
B1Na3P2_ICSD_300124	insulator	insulator
B1Na3S3_ICSD_411608	insulator	insulator
B1Nb1Ni1_ICSD_614909	metal	metal
B1Nb1Ni2O6_ICSD_32583	insulator	insulator
B1Nb1O4_ICSD_63202	insulator	insulator
B1Nb1_ICSD_614885	metal	metal
B1Nb5Si3_ICSD_614916	metal	metal
B1Nd1Ni4_ICSD_20882	metal	metal
B1Nd1O3_ICSD_240380	insulator	insulator
B1Nd1O3_ICSD_412407	insulator	insulator
B1Nd1Pd3_ICSD_44463	metal	metal
B1Nd1Pt2_ICSD_98426	metal	metal
B1Nd1Pt3_ICSD_97371	metal	metal
B1Nd1Rh3_ICSD_44464	metal	metal
B1Nd1S3_ICSD_421073	insulator	insulator
B1Ni1Ta1_ICSD_615023	metal	metal
B1Ni1_ICSD_26937	metal	metal
B1Ni2_ICSD_603804	metal	metal
B1Ni3_ICSD_106927	metal	metal
B1Ni4Tb1_ICSD_156975	metal	metal
B1Ni4Tm1_ICSD_44531	metal	metal
B1Ni4U1_ICSD_615060	metal	metal
B1Ni4Y1_ICSD_615076	metal	metal
B1Ni4Yb1_ICSD_615088	metal	metal
B1Ni6Si2_ICSD_185117	metal	metal
B1O2Tl1_ICSD_36404	insulator	insulator
B1O3P1Sr3_ICSD_401207	insulator	insulator
B1O3Pr1_ICSD_421745	insulator	insulator
B1O3Sc1_ICSD_65010	insulator	insulator
B1O3Ti1_ICSD_402039	metal	insulator

Supplementary Table 108. Five-fold cross validated predictions for the metal/insulator classification (100/598).

system	calculated	predicted
B1O3Tl3_ICSD_8084	insulator	insulator
B1O3V1_ICSD_45060	insulator	insulator
B1O3Y1_ICSD_44162	insulator	insulator
B1O3Yb1_ICSD_160141	metal	insulator
B1O3Yb1_ICSD_34663	metal	insulator
B1O4P1_ICSD_26890	insulator	insulator
B1O4P1_ICSD_413435	insulator	insulator
B1O4Ta1_ICSD_402404	insulator	insulator
B1O5P1Pb1_ICSD_93598	insulator	insulator
B1O5P1Sr1_ICSD_77519	insulator	insulator
B1O5P1Sr1_ICSD_97675	insulator	insulator
B1O7P1Zn3_ICSD_406386	insulator	insulator
B1O8P2Rb1Zn1_ICSD_410870	insulator	insulator
B1O9Tb3W1_ICSD_250418	insulator	insulator
B1Os1_ICSD_181304	metal	metal
B1P1S4_ICSD_24618	insulator	insulator
B1P1_ICSD_184570	insulator	insulator
B1P1_ICSD_615155	insulator	insulator
B1P2Rb3_ICSD_402084	insulator	insulator
B1Pb1Sc3_ICSD_44541	metal	metal
B1Pd2_ICSD_615164	metal	metal
B1Pd3Pr1_ICSD_44543	metal	metal
B1Pd3Tb1_ICSD_44545	metal	metal
B1Pd3Tm1_ICSD_44546	metal	metal
B1Pd3Yb1_ICSD_44547	metal	metal
B1Pd3_ICSD_94235	metal	metal
B1Pd4Sr1_ICSD_247263	metal	metal
B1Pr1Pt3_ICSD_97370	metal	metal
B1Pr1Pt4_ICSD_55454	metal	metal
B1Pr1Rh3_ICSD_44548	metal	metal
B1Pr1S3_ICSD_421072	insulator	insulator
B1Pt1_ICSD_24363	metal	metal
B1Pt2Y1_ICSD_156947	metal	metal
B1Pt2_ICSD_615207	metal	metal
B1Pu1_ICSD_43661	metal	metal
B1Rb1S3_ICSD_73084	insulator	insulator
B1Rb1Se3_ICSD_411343	insulator	insulator
B1Re3_ICSD_615241	metal	metal
B1Re4Zr9_ICSD_615287	metal	metal
B1Rh1_ICSD_150732	metal	metal
B1Rh2_ICSD_24699	metal	metal
B1Rh3Sc1_ICSD_44561	metal	metal
B1Rh3Tb1_ICSD_44565	metal	metal
B1Rh3Tm1_ICSD_44566	metal	metal
B1Rh3Tm1_ICSD_615332	metal	metal
B1Rh3U1_ICSD_615338	metal	metal
B1Rh3Y1_ICSD_44568	metal	metal
B1Rh3Yb1_ICSD_44570	metal	metal
B1Rh3Yb1_ICSD_615350	metal	metal
B1Rh3Zr1_ICSD_615353	metal	metal

Supplementary Table 109. Five-fold cross validated predictions for the metal/insulator classification (101/598).

system	calculated	predicted
B1Rh6Sn4_ICSD_77353	metal	metal
B1Rh6Ti2_ICSD_415337	metal	metal
B1S2Tl1_ICSD_71593	insulator	insulator
B1S3Tb1_ICSD_710042	insulator	insulator
B1S3Tl1_ICSD_73085	insulator	insulator
B1S3Tl3_ICSD_202528	insulator	insulator
B1Sb1_ICSD_184571	insulator	metal
B1Sc3Sn1_ICSD_167913	metal	metal
B1Sc3Tl1_ICSD_44586	metal	metal
B1Se3Tl3_ICSD_40375	insulator	insulator
B1Si2Ti6_ICSD_98945	metal	metal
B1Si3Tb5_ICSD_615438	metal	metal
B1Sn3Zr5_ICSD_656289	metal	metal
B1Ta1_ICSD_42954	metal	metal
B1Ta2_ICSD_76744	metal	metal
B1Ta4Te8_ICSD_410843	metal	metal
B1Tc1_ICSD_168895	metal	metal
B1Tc1_ICSD_168897	metal	metal
B1Tc1_ICSD_168899	metal	metal
B1Tc1_ICSD_168900	metal	metal
B1Tc1_ICSD_168901	metal	metal
B1Tc3_ICSD_615555	metal	metal
B1Ti1_ICSD_44595	metal	metal
B1Ti1_ICSD_615596	metal	metal
B1Ti2_ICSD_189385	metal	metal
B1V1_ICSD_615658	metal	metal
B1W1_ICSD_24281	metal	metal
B1W1_ICSD_615689	metal	metal
B1W2_ICSD_24279	metal	metal
B1W4Zr9_ICSD_615705	metal	metal
B1Zr1_ICSD_44605	metal	metal
B1_ICSD_189437	metal	metal
B1_ICSD_22300	metal	metal
B1_ICSD_240995	metal	metal
B1_ICSD_659240	insulator	metal
B20Na3_ICSD_410247	metal	metal
B24Fe80Nb12_ICSD_164057	metal	metal
B28_ICSD_164659	insulator	metal
B2Ba1Be2O6_ICSD_67975	insulator	insulator
B2Ba1Cu1O5_ICSD_84683	insulator	insulator
B2Ba1F8_ICSD_240991	insulator	insulator
B2Ba1Ga2O7_ICSD_91280	insulator	insulator
B2Ba1Ir2_ICSD_612532	metal	metal
B2Ba1O6Ti1_ICSD_183931	insulator	insulator
B2Ba1O6Zr1_ICSD_95527	insulator	insulator
B2Ba1Rh2_ICSD_8155	metal	metal
B2Ba1S4_ICSD_412516	insulator	insulator
B2Ba1Se6_ICSD_411967	insulator	insulator
B2Ba2Ca1O6_ICSD_80429	insulator	insulator
B2Ba2Cd1O6_ICSD_180954	insulator	insulator

Supplementary Table 110. Five-fold cross validated predictions for the metal/insulator classification (102/598).

system	calculated	predicted
B2Ba2Cl1Gd1O6_ICSD_262073	insulator	insulator
B2Ba2Cl1Ho1O6_ICSD_65933	insulator	insulator
B2Ba2Cl1O6Y1_ICSD_262072	insulator	insulator
B2Ba2Cl1O6Yb1_ICSD_65934	insulator	insulator
B2Ba2Mg1O6_ICSD_75986	insulator	insulator
B2Ba3N4_ICSD_412663	insulator	insulator
B2Ba3O12Ti3_ICSD_99460	insulator	insulator
B2Ba4N4O1_ICSD_400338	insulator	insulator
B2Be1C2_ICSD_418618	insulator	insulator
B2Be1Ca1O5_ICSD_72520	insulator	insulator
B2Be1_ICSD_186762	metal	metal
B2Be2Ca1F1K1O6_ICSD_183886	insulator	insulator
B2Be2Ca1F1Na1O6_ICSD_183885	insulator	insulator
B2Be2F1K1O6Sr1_ICSD_183887	insulator	insulator
B2Be2O7Sr2_ICSD_79025	insulator	insulator
B2Bi1F1O4_ICSD_172481	insulator	insulator
B2Bi2Ca1O7_ICSD_245016	insulator	insulator
B2Bi2O7Zn1_ICSD_152281	insulator	insulator
B2Br15Tb10_ICSD_410990	metal	insulator
B2Br3C3Ce6_ICSD_401744	metal	metal
B2Br4Cl6N2P2_ICSD_412556	insulator	insulator
B2Br6C4Gd8_ICSD_401745	metal	metal
B2C1Ce1Ir2_ICSD_95098	metal	metal
B2C1Ce1Ni2_ICSD_56645	metal	metal
B2C1Ce1Rh2_ICSD_95097	metal	metal
B2C1Ce1_ICSD_40164	metal	metal
B2C1Co2Pr1_ICSD_157583	metal	metal
B2C1Co2Tm1_ICSD_164575	metal	metal
B2C1Dy1Ni2_ICSD_79573	metal	metal
B2C1Dy1Rh2_ICSD_150156	metal	metal
B2C1Dy1_ICSD_42476	metal	metal
B2C1Er1Ni2_ICSD_246431	metal	metal
B2C1Er1Rh2_ICSD_150159	metal	metal
B2C1Eu1Ni2_ICSD_79571	metal	metal
B2C1Gd1Ni2_ICSD_79563	metal	metal
B2C1Gd1Rh2_ICSD_150154	metal	metal
B2C1Ho1Ni2_ICSD_56650	metal	metal
B2C1Ho1Rh2_ICSD_150158	metal	metal
B2C1Ho1_ICSD_41762	metal	metal
B2C1Ir2La1_ICSD_79567	metal	metal
B2C1La1Ni2_ICSD_79562	metal	metal
B2C1La1Pt2_ICSD_79565	metal	metal
B2C1La1Rh2_ICSD_150149	metal	metal
B2C1Lu1Ni2_ICSD_79564	metal	metal
B2C1Lu1_ICSD_44139	metal	metal
B2C1N1_ICSD_183790	metal	metal
B2C1N1_ICSD_183791	metal	metal
B2C1N1_ICSD_183792	metal	metal
B2C1N1_ICSD_183793	metal	metal
B2C1N1_ICSD_183794	metal	metal

Supplementary Table 111. Five-fold cross validated predictions for the metal/insulator classification (103/598).

system	calculated	predicted
B2C1Nd1Ni2_ICSD_79569	metal	metal
B2C1Nd1Rh2_ICSD_150152	metal	metal
B2C1Ni2Pr1_ICSD_157582	metal	metal
B2C1Ni2Tb1_ICSD_89155	metal	metal
B2C1Ni2Th1_ICSD_659009	metal	metal
B2C1Ni2Tm1_ICSD_56652	metal	metal
B2C1Ni2Y1_ICSD_83231	metal	metal
B2C1Ni2Yb1_ICSD_56653	metal	metal
B2C1Pr1Pt2_ICSD_157584	metal	metal
B2C1Pr1Rh2_ICSD_150151	metal	metal
B2C1Pt2Th1_ICSD_659011	metal	metal
B2C1Rh2Tb1_ICSD_150155	metal	metal
B2C1Rh2Th1_ICSD_659010	metal	metal
B2C1Rh2U1_ICSD_659008	metal	metal
B2C1Rh2Y1_ICSD_150157	metal	metal
B2C1Sc1_ICSD_54104	metal	metal
B2C1Tb1_ICSD_44141	metal	metal
B2C1Th1_ICSD_68415	metal	metal
B2C1Tm1_ICSD_41763	metal	metal
B2C1U1_ICSD_44142	metal	metal
B2C1U1_ICSD_69767	metal	metal
B2C1Y1_ICSD_41761	metal	metal
B2C2Ca1_ICSD_166515	metal	insulator
B2C2Ca1_ICSD_88019	insulator	metal
B2C2Ce1_ICSD_603066	metal	metal
B2C2Ce1_ICSD_88858	metal	metal
B2C2Dy1_ICSD_42475	metal	metal
B2C2Dy1_ICSD_88558	metal	metal
B2C2Er1_ICSD_612593	metal	metal
B2C2Er1_ICSD_94042	metal	metal
B2C2Gd1_ICSD_612619	metal	metal
B2C2Ho1_ICSD_612622	metal	metal
B2C2Ho1_ICSD_94041	metal	metal
B2C2La1_ICSD_612631	metal	metal
B2C2La1_ICSD_94035	metal	metal
B2C2Lu1_ICSD_94044	metal	metal
B2C2Mg1_ICSD_421839	insulator	insulator
B2C2Mg1_ICSD_79587	insulator	insulator
B2C2Nd1_ICSD_612651	metal	metal
B2C2Nd1_ICSD_88859	metal	metal
B2C2Nd1_ICSD_88860	metal	metal
B2C2Nd1_ICSD_94038	metal	metal
B2C2Pr1_ICSD_612652	metal	metal
B2C2Pr1_ICSD_94037	metal	metal
B2C2Sc1_ICSD_23834	metal	metal
B2C2Tb1_ICSD_612659	metal	metal
B2C2Tb1_ICSD_94039	metal	metal
B2C2Y1_ICSD_612672	metal	metal
B2C2Yb1_ICSD_612680	metal	metal
B2C3Tb2_ICSD_419476	metal	metal

Supplementary Table 112. Five-fold cross validated predictions for the metal/insulator classification (104/598).

system	calculated	predicted
B2C3Th3_ICSD_23077	metal	metal
B2C5Dy5_ICSD_410896	metal	metal
B2C6F8Fe1O6_ICSD_152380	insulator	insulator
B2C8Co1H4N8O2_ICSD_260263	metal	insulator
B2C8Cu1N8_ICSD_415546	insulator	insulator
B2C8Hg1N8_ICSD_412297	insulator	insulator
B2C8N8Zn1_ICSD_415547	insulator	insulator
B2Ca1H10N2_ICSD_246139	insulator	insulator
B2Ca1H12O10_ICSD_23016	insulator	insulator
B2Ca1H1_ICSD_168228	metal	metal
B2Ca1H2_ICSD_163473	metal	metal
B2Ca1H2_ICSD_168230	metal	metal
B2Ca1H2_ICSD_183133	insulator	metal
B2Ca1H2_ICSD_183134	metal	insulator
B2Ca1H3_ICSD_168232	metal	metal
B2Ca1H3_ICSD_168233	metal	metal
B2Ca1H8_ICSD_163262	insulator	insulator
B2Ca1H8_ICSD_163263	insulator	insulator
B2Ca1H8_ICSD_163480	insulator	insulator
B2Ca1H8_ICSD_164182	insulator	insulator
B2Ca1H8_ICSD_168224	insulator	insulator
B2Ca1H8_ICSD_168225	insulator	insulator
B2Ca1Ir2_ICSD_49738	metal	metal
B2Ca1Li4O6_ICSD_99503	insulator	insulator
B2Ca1O4_ICSD_20097	insulator	insulator
B2Ca1O4_ICSD_23241	insulator	insulator
B2Ca1O6Sn1_ICSD_30998	insulator	insulator
B2Ca1O8Si2_ICSD_26491	insulator	insulator
B2Ca1Rh2_ICSD_49737	metal	metal
B2Ca1Rh3_ICSD_66767	metal	metal
B2Ca1_ICSD_186764	metal	metal
B2Ca2O5_ICSD_66516	insulator	insulator
B2Ca3Ni7_ICSD_36505	metal	metal
B2Ca3O6_ICSD_23664	insulator	insulator
B2Cd1F1K1O6Zn2_ICSD_248025	insulator	insulator
B2Cd1H8_ICSD_262600	insulator	insulator
B2Cd2O5_ICSD_200621	insulator	insulator
B2Cd2O5_ICSD_281357	insulator	insulator
B2Cd3O6_ICSD_66170	insulator	insulator
B2Ce1Cl1O4_ICSD_413236	insulator	insulator
B2Ce1Co3_ICSD_612737	metal	metal
B2Ce1Co3_ICSD_612751	metal	metal
B2Ce1Ir3_ICSD_97341	metal	metal
B2Ce1Os2_ICSD_601367	metal	metal
B2Ce1Rh3_ICSD_40777	metal	metal
B2Ce1Rh3_ICSD_40778	metal	metal
B2Ce1Rh3_ICSD_612827	metal	metal
B2Ce1Ru1_ICSD_612853	metal	metal
B2Ce1Ru2_ICSD_40799	metal	metal
B2Ce1Ru2_ICSD_40800	metal	metal

Supplementary Table 113. Five-fold cross validated predictions for the metal/insulator classification (105/598).

system	calculated	predicted
B2Ce1Ru3_ICSD_612852	metal	metal
B2Ce2Ir5_ICSD_97343	metal	metal
B2Ce3Cl3O6_ICSD_413237	insulator	insulator
B2Ce3N4_ICSD_68610	metal	metal
B2Ce4N5_ICSD_51538	metal	metal
B2Cl1La1O4_ICSD_413209	insulator	insulator
B2Cl1O4Pr1_ICSD_95851	insulator	insulator
B2Cl4_ICSD_31693	insulator	insulator
B2Co1Ir5Mg2_ICSD_613129	metal	metal
B2Co1Mo2_ICSD_613166	metal	metal
B2Co1Nb1_ICSD_41893	metal	metal
B2Co1O4_ICSD_422063	insulator	insulator
B2Co1W2_ICSD_16776	metal	metal
B2Co2Dy1_ICSD_612907	metal	metal
B2Co2Er1_ICSD_612925	metal	metal
B2Co2Gd1_ICSD_602344	metal	metal
B2Co2Ho1_ICSD_613114	metal	metal
B2Co2La1_ICSD_423045	metal	metal
B2Co2Nd1_ICSD_44168	metal	metal
B2Co2Nd1_ICSD_613196	metal	metal
B2Co2O5_ICSD_24284	insulator	insulator
B2Co2Tb1_ICSD_613330	metal	metal
B2Co2Y1_ICSD_613423	metal	metal
B2Co3Dy1_ICSD_612896	metal	metal
B2Co3Er1_ICSD_612931	metal	metal
B2Co3Gd1_ICSD_23656	metal	metal
B2Co3Hf1_ICSD_23550	metal	metal
B2Co3Hf1_ICSD_613106	metal	metal
B2Co3Ho1_ICSD_87165	metal	metal
B2Co3Lu1_ICSD_44171	metal	metal
B2Co3O6_ICSD_24035	insulator	insulator
B2Co3Sc1_ICSD_44179	metal	metal
B2Co3Tb1_ICSD_246511	metal	metal
B2Co3U1_ICSD_44185	metal	metal
B2Co3Y1_ICSD_659053	metal	metal
B2Co3Yb1_ICSD_44189	metal	metal
B2Co3Zr1_ICSD_16179	metal	metal
B2Co3Zr1_ICSD_44191	metal	metal
B2Co3Zr1_ICSD_613452	metal	metal
B2Co5P1_ICSD_613222	metal	metal
B2Co5Ti3_ICSD_20140	metal	metal
B2Co7U3_ICSD_86379	metal	metal
B2Cr1Ir5Mg2_ICSD_613516	metal	metal
B2Cr1Nb2_ICSD_613529	metal	metal
B2Cr1Rh5Sc2_ICSD_51435	metal	metal
B2Cr1Ta2_ICSD_613557	metal	metal
B2Cr1_ICSD_44194	metal	metal
B2Cr2Ir1_ICSD_601518	metal	metal
B2Cs1Li5O6_ICSD_61203	insulator	insulator
B2Cs1Nb1O6_ICSD_79703	insulator	insulator

Supplementary Table 114. Five-fold cross validated predictions for the metal/insulator classification (106/598).

system	calculated	predicted
B2Cs1O6Ta1_ICSD_80423	insulator	insulator
B2Cs3Li2Na1O6_ICSD_36531	insulator	insulator
B2Cu1O4_ICSD_9087	metal	insulator
B2Cu1O6Pb2_ICSD_155317	insulator	insulator
B2Cu1O6Sr2_ICSD_202934	insulator	insulator
B2Cu2O6Sr1_ICSD_247206	insulator	insulator
B2Cu2O6Sr1_ICSD_247207	insulator	insulator
B2Cu2O6Sr1_ICSD_80592	insulator	insulator
B2Dy1Fe2_ICSD_613606	metal	metal
B2Dy1Ir2_ICSD_613641	metal	metal
B2Dy1Ir3_ICSD_613640	metal	metal
B2Dy1Ni2_ICSD_613653	metal	metal
B2Dy1Os1_ICSD_613666	metal	metal
B2Dy1Rh3_ICSD_613675	metal	metal
B2Dy1Ru1_ICSD_613687	metal	metal
B2Dy1Ru3_ICSD_613684	metal	metal
B2Dy1_ICSD_44205	metal	metal
B2Dy3Ni13_ICSD_108041	metal	metal
B2Dy3Ni7_ICSD_613658	metal	metal
B2Er1Fe2_ICSD_44218	metal	metal
B2Er1Fe2_ICSD_613745	metal	metal
B2Er1Ir3_ICSD_44230	metal	metal
B2Er1Ni2_ICSD_613779	metal	metal
B2Er1Os1_ICSD_613789	metal	metal
B2Er1Rh3_ICSD_72568	metal	metal
B2Er1Ru1_ICSD_613819	metal	metal
B2Er1Ru3_ICSD_44238	metal	metal
B2Er1_ICSD_613696	metal	metal
B2Er3Ni13_ICSD_601073	metal	metal
B2Er3Ni7_ICSD_44231	metal	metal
B2Eu1Rh3_ICSD_44243	metal	metal
B2Eu3O6_ICSD_86479	insulator	insulator
B2F10H1Nd1_ICSD_420467	insulator	insulator
B2F10H1Pr1_ICSD_420466	insulator	insulator
B2F3Gd3O6_ICSD_51140	insulator	insulator
B2F4_ICSD_27867	insulator	insulator
B2F8H6N2_ICSD_49614	insulator	insulator
B2Fe1Ir5Sc2_ICSD_85631	metal	metal
B2Fe1Mo2_ICSD_44292	metal	metal
B2Fe1Nb2_ICSD_614047	metal	metal
B2Fe1O4_ICSD_420403	insulator	insulator
B2Fe1Rh5Sc2_ICSD_51437	metal	metal
B2Fe1Ta2_ICSD_614207	metal	metal
B2Fe1W2_ICSD_43016	metal	metal
B2Fe1W2_ICSD_614261	metal	metal
B2Fe1_ICSD_425310	insulator	metal
B2Fe1_ICSD_613892	metal	metal
B2Fe2Gd1_ICSD_613920	metal	metal
B2Fe2H2K2O18P4_ICSD_407797	insulator	insulator
B2Fe2Ho1_ICSD_613974	metal	metal

Supplementary Table 115. Five-fold cross validated predictions for the metal/insulator classification (107/598).

system	calculated	predicted
B2Fe2Lu1_ICSD_604549	metal	metal
B2Fe2Tb1_ICSD_614209	metal	metal
B2Fe2Y1_ICSD_614264	metal	metal
B2Fe3U1_ICSD_44302	metal	metal
B2Fe5P1_ICSD_601531	metal	metal
B2Fe5Si1_ICSD_614183	metal	metal
B2Ga1Rh5Sc2_ICSD_51438	metal	metal
B2Ga2K2O7_ICSD_50039	insulator	insulator
B2Ga2O7Sr1_ICSD_91279	insulator	insulator
B2Ga6Ge6H8Na8O24_ICSD_188639	insulator	insulator
B2Gd1Os1_ICSD_614363	metal	metal
B2Gd1Rh3_ICSD_44311	metal	metal
B2Gd1Ru1_ICSD_614386	metal	metal
B2Gd1Ru2_ICSD_601355	metal	metal
B2Gd1Ru3_ICSD_44312	metal	metal
B2Gd1_ICSD_614307	metal	metal
B2Ge1Mg2Rh5_ICSD_51432	metal	metal
B2H16Na2O14_ICSD_30532	insulator	insulator
B2H4Li2O8_ICSD_100854	insulator	insulator
B2H6_ICSD_1312	insulator	insulator
B2H6_ICSD_413919	insulator	insulator
B2H8K1Li1_ICSD_173236	insulator	insulator
B2H8K1Na1_ICSD_163376	insulator	insulator
B2H8K1Na1_ICSD_163377	insulator	insulator
B2H8K1Na1_ICSD_163378	insulator	insulator
B2H8K1Na1_ICSD_163379	insulator	insulator
B2H8Mg1_ICSD_155717	insulator	insulator
B2H8Mg1_ICSD_161140	insulator	insulator
B2H8Mg1_ICSD_187436	insulator	insulator
B2H8Mg1_ICSD_187440	insulator	insulator
B2H8Mg1_ICSD_187459	insulator	insulator
B2H8Mg1_ICSD_262083	insulator	insulator
B2H8Zn1_ICSD_161376	insulator	insulator
B2Hf1_ICSD_167823	metal	metal
B2Hf3Ir5_ICSD_44316	metal	metal
B2Hg3O6_ICSD_409688	insulator	insulator
B2Ho1Ir3_ICSD_614472	metal	metal
B2Ho1Ni2_ICSD_39487	metal	metal
B2Ho1Os1_ICSD_614489	metal	metal
B2Ho1Rh3_ICSD_614497	metal	metal
B2Ho1Ru1_ICSD_614509	metal	metal
B2Ho1Ru3_ICSD_614506	metal	metal
B2Ho1_ICSD_44320	metal	metal
B2Ho3Ni13_ICSD_601055	metal	metal
B2Ho3Ni7_ICSD_614478	metal	metal
B2Ir1Mo2_ICSD_23786	metal	metal
B2Ir1V2_ICSD_601555	metal	metal
B2Ir2La1_ICSD_614525	metal	metal
B2Ir2Sr1_ICSD_8154	metal	metal
B2Ir2Tb1_ICSD_614567	metal	metal

Supplementary Table 116. Five-fold cross validated predictions for the metal/insulator classification (108/598).

system	calculated	predicted
B2Ir2Zn1_ICSD_71642	metal	metal
B2Ir3La1_ICSD_44422	metal	metal
B2Ir3Lu1_ICSD_614531	metal	metal
B2Ir3Nd1_ICSD_614546	metal	metal
B2Ir3Pr1_ICSD_97342	metal	metal
B2Ir3Pu1_ICSD_600578	metal	metal
B2Ir3Sc1_ICSD_614561	metal	metal
B2Ir3Tb1_ICSD_614566	metal	metal
B2Ir3Th1_ICSD_44425	metal	metal
B2Ir3U1_ICSD_44426	metal	metal
B2Ir3Y1_ICSD_99236	metal	metal
B2Ir3Yb1_ICSD_614581	metal	metal
B2Ir5Mg2Mn1_ICSD_614533	metal	metal
B2Ir5Mg2Si1_ICSD_69487	metal	metal
B2Ir5Sc2Si1_ICSD_85629	metal	metal
B2Ir5Sc2V1_ICSD_85630	metal	metal
B2Ir6Sn5_ICSD_78984	metal	metal
B2K1O6Ta1_ICSD_162214	insulator	insulator
B2K2La2O7_ICSD_174543	insulator	insulator
B2K2O6Zr1_ICSD_67982	insulator	insulator
B2K3Nb3O12_ICSD_85091	insulator	insulator
B2K3Nb3O12_ICSD_968	insulator	insulator
B2K3O12Ta3_ICSD_201143	insulator	insulator
B2K3O6Y1_ICSD_245925	insulator	insulator
B2La1Os2_ICSD_601366	metal	metal
B2La1Rh3_ICSD_614643	metal	metal
B2La1Ru2_ICSD_601360	metal	metal
B2La1Ru3_ICSD_44433	metal	metal
B2La3N4_ICSD_407033	metal	metal
B2La5N6_ICSD_410980	metal	metal
B2Li2Rh3_ICSD_417442	metal	metal
B2Li2S5_ICSD_401723	insulator	insulator
B2Li2Se5_ICSD_411410	insulator	insulator
B2Li2_ICSD_1	metal	metal
B2Li2_ICSD_2	metal	metal
B2Li3O6Sc1_ICSD_241234	insulator	insulator
B2Li3O6Sc1_ICSD_261256	insulator	insulator
B2Lu1Os3_ICSD_44435	metal	metal
B2Lu1Rh3_ICSD_614701	metal	metal
B2Lu1Ru3_ICSD_44439	metal	metal
B2Lu1_ICSD_57002	metal	metal
B2Mg1Ni3_ICSD_156018	metal	metal
B2Mg1Ni6.7_ICSD_44442	metal	metal
B2Mg1O6Sn1_ICSD_28266	insulator	insulator
B2Mg1O6Sr2_ICSD_240897	insulator	insulator
B2Mg1_ICSD_96906	metal	metal
B2Mg2O5_ICSD_24789	insulator	insulator
B2Mg2O5_ICSD_81229	insulator	insulator
B2Mg3Mn3O10_ICSD_30654	metal	insulator
B2Mg3O6_ICSD_24036	insulator	insulator

Supplementary Table 117. Five-fold cross validated predictions for the metal/insulator classification (109/598).

system	calculated	predicted
B2Mn1Mo2_ICSD_614755	metal	metal
B2Mn1O6Sn1_ICSD_79165	insulator	insulator
B2Mn1Rh5Sc2_ICSD_51436	metal	metal
B2Mn1W2_ICSD_44449	metal	metal
B2Mn1_ICSD_43664	metal	metal
B2Mn5P1_ICSD_109111	metal	metal
B2Mo1_ICSD_418398	metal	metal
B2Mo1_ICSD_76410	metal	metal
B2Mo2Nb1_ICSD_614821	metal	metal
B2Mo2Ni1_ICSD_43192	metal	metal
B2Mo2Os1_ICSD_614829	metal	metal
B2Mo2Ru1_ICSD_601573	metal	metal
B2Mo2Ta1_ICSD_614842	metal	metal
B2Mo3_ICSD_614800	metal	metal
B2Mo5Si1_ICSD_614839	metal	metal
B2N4Nd3_ICSD_407576	metal	metal
B2N4Pr3_ICSD_407282	metal	metal
B2Na1O5Sc1_ICSD_409522	insulator	insulator
B2Na2S5_ICSD_401724	insulator	insulator
B2Na3O6Sc1_ICSD_262733	insulator	insulator
B2Na4O5_ICSD_10061	insulator	insulator
B2Nb1Ni1_ICSD_614910	metal	metal
B2Nb1_ICSD_614884	metal	metal
B2Nb3_ICSD_614907	metal	metal
B2Nd1Os2_ICSD_601369	metal	metal
B2Nd1Rh3_ICSD_614963	metal	metal
B2Nd1Ru3_ICSD_614966	metal	metal
B2Ni13Tb3_ICSD_44468	metal	metal
B2Ni13Y3_ICSD_246557	metal	metal
B2Ni13Yb3_ICSD_603509	metal	metal
B2Ni1O4_ICSD_418385	insulator	insulator
B2Ni1Ta1_ICSD_100435	metal	metal
B2Ni1W2_ICSD_615069	metal	metal
B2Ni2Tb1_ICSD_57003	metal	metal
B2Ni2Y1_ICSD_615079	metal	metal
B2Ni3O6_ICSD_2016	insulator	insulator
B2Ni3Zn1_ICSD_261932	metal	metal
B2Ni6.67Sn1_ICSD_44466	metal	metal
B2Ni6.67Zn1_ICSD_44536	metal	metal
B2Ni7Tb3_ICSD_615028	metal	metal
B2Ni7Y3_ICSD_615073	metal	metal
B2O1_ICSD_41670	metal	insulator
B2O3_ICSD_24649	insulator	insulator
B2O3_ICSD_36066	insulator	insulator
B2O4Sr1_ICSD_203226	insulator	insulator
B2O4Sr1_ICSD_69102	insulator	insulator
B2O5Sr2_ICSD_50739	insulator	insulator
B2O5Zr1_ICSD_418931	insulator	insulator
B2O6Pb1Zn2_ICSD_171139	insulator	insulator
B2O6Sn1Sr1_ICSD_28267	insulator	insulator

Supplementary Table 118. Five-fold cross validated predictions for the metal/insulator classification (110/598).

system	calculated	predicted
B2O6Sr3_ICSD_93395	insulator	insulator
B2O6U1_ICSD_248127	insulator	insulator
B2O6U1_ICSD_49908	insulator	insulator
B2O7Se2_ICSD_172383	insulator	insulator
B2O8Si2Sr1_ICSD_83368	insulator	insulator
B2O9S2_ICSD_426544	insulator	insulator
B2Os1Pu1_ICSD_601842	metal	metal
B2Os1Sc1_ICSD_615126	metal	metal
B2Os1Tb1_ICSD_615131	metal	metal
B2Os1Y1_ICSD_615149	metal	metal
B2Os1Yb1_ICSD_615151	metal	metal
B2Os1_ICSD_615117	metal	metal
B2Os2Pr1_ICSD_601368	metal	metal
B2Os2Th1_ICSD_601346	metal	metal
B2Os3U1_ICSD_44539	metal	metal
B2Pd5_ICSD_43513	metal	metal
B2Pd7Y1_ICSD_260147	metal	metal
B2Pr1Rh3_ICSD_44550	metal	metal
B2Pr1Rh3_ICSD_615187	metal	metal
B2Pr1Rh3_ICSD_615193	metal	metal
B2Pr1Ru2_ICSD_601361	metal	metal
B2Pr1Ru3_ICSD_44551	metal	metal
B2Pu1Re1_ICSD_603981	metal	metal
B2Pu1Rh3_ICSD_600567	metal	metal
B2Pu1Ru1_ICSD_603992	metal	metal
B2Pu1Tc1_ICSD_603980	metal	metal
B2Pu1_ICSD_43660	metal	metal
B2Re1Ti2_ICSD_44554	metal	metal
B2Re1_ICSD_421522	metal	metal
B2Rh2Sr1_ICSD_8153	metal	metal
B2Rh3Tb1_ICSD_615319	metal	metal
B2Rh3Y1_ICSD_615340	metal	metal
B2Rh3Yb1_ICSD_615348	metal	metal
B2Rh5Sc3_ICSD_51434	metal	metal
B2Rh6Sn5_ICSD_77352	metal	metal
B2Ru1Tb1_ICSD_615373	metal	metal
B2Ru1Y1_ICSD_615399	metal	metal
B2Ru1_ICSD_421524	metal	metal
B2Ru2Th1_ICSD_601365	metal	metal
B2Ru3Tb1_ICSD_615376	metal	metal
B2Ru3Th1_ICSD_44574	metal	metal
B2Ru3Tm1_ICSD_44576	metal	metal
B2Ru3U1_ICSD_44579	metal	metal
B2Ru3Y1_ICSD_659500	metal	metal
B2Ru3Yb1_ICSD_44583	metal	metal
B2S4Sr1_ICSD_71594	insulator	insulator
B2S6Sr3_ICSD_412879	insulator	insulator
B2Sc1_ICSD_106903	metal	metal
B2Sc1_ICSD_615426	metal	metal
B2Si1V5_ICSD_615439	metal	metal

Supplementary Table 119. Five-fold cross validated predictions for the metal/insulator classification (111/598).

system	calculated	predicted
B2Ta1_ICSD_615525	metal	metal
B2Ta3_ICSD_107320	metal	metal
B2Tb1_ICSD_615535	metal	metal
B2Tc1_ICSD_615553	metal	metal
B2Ti1_ICSD_56723	metal	metal
B2Tm1_ICSD_44598	metal	metal
B2U1_ICSD_615636	metal	metal
B2V1_ICSD_30417	metal	metal
B2V3_ICSD_615662	metal	metal
B2W1_ICSD_23716	metal	metal
B2W1_ICSD_44601	metal	metal
B2Y1_ICSD_44602	metal	metal
B2Yb1_ICSD_44604	metal	metal
B2Zr1_ICSD_615755	metal	metal
B3Ba1Li1S6_ICSD_82353	insulator	insulator
B3Ba4N6Na1_ICSD_401210	insulator	insulator
B3Ba5Br1O9_ICSD_422557	insulator	insulator
B3Ba5C1N1O9_ICSD_414486	insulator	insulator
B3Ba5Cl1O9_ICSD_422556	insulator	insulator
B3Be2K1O7_ICSD_248202	insulator	insulator
B3Be2K1O7_ICSD_248203	insulator	insulator
B3Be2O7Rb1_ICSD_248205	insulator	insulator
B3Be3F4Na1O9Sr3_ICSD_423143	insulator	insulator
B3Bi1O6_ICSD_245893	insulator	insulator
B3Bi1O6_ICSD_416822	insulator	insulator
B3Br1La2_ICSD_417603	metal	metal
B3C2Gd2_ICSD_67980	metal	metal
B3C2Y2_ICSD_90269	metal	metal
B3C4Gd4_ICSD_280354	metal	metal
B3Ca1H7O9_ICSD_75922	insulator	insulator
B3Ca4Gd1O10_ICSD_86172	insulator	insulator
B3Ca4K1O9_ICSD_171422	insulator	insulator
B3Ca4La1O10_ICSD_180596	insulator	insulator
B3Ca4Li1N6_ICSD_83419	insulator	insulator
B3Ca4Na1O9_ICSD_171421	insulator	insulator
B3Ca5F1O9_ICSD_65763	insulator	insulator
B3Ce1O6_ICSD_413903	insulator	insulator
B3Ce1O6_ICSD_99691	insulator	insulator
B3Cl1Eu5O9_ICSD_422555	insulator	insulator
B3Cl1La2_ICSD_417604	metal	metal
B3Cl1O9Sr5_ICSD_71871	insulator	insulator
B3Cl1Pr2_ICSD_410727	metal	metal
B3Cl6N3_ICSD_14276	insulator	insulator
B3Co1Mo3_ICSD_613173	metal	metal
B3Co1V1_ICSD_44188	metal	metal
B3Co1W3_ICSD_25753	metal	metal
B3Co7Dy2_ICSD_612905	metal	metal
B3Co7Er2_ICSD_612929	metal	metal
B3Co7Ho2_ICSD_613119	metal	metal
B3Co7Tb2_ICSD_613342	metal	metal

Supplementary Table 120. Five-fold cross validated predictions for the metal/insulator classification (112/598).

system	calculated	predicted
B3Co7Y2_ICSD_613431	metal	metal
B3Co7Y2_ICSD_656888	metal	metal
B3Cr2_ICSD_601350	metal	metal
B3Cr5_ICSD_613474	metal	metal
B3Cs1H8_ICSD_65947	insulator	insulator
B3Er1Mo1_ICSD_65932	metal	metal
B3Eu4Li1N6_ICSD_400465	insulator	insulator
B3Gd1O6_ICSD_8083	insulator	insulator
B3Ge2Ni8_ICSD_54011	metal	metal
B3H12Y1_ICSD_169076	insulator	insulator
B3H2Na3O7_ICSD_1939	insulator	insulator
B3H3O14Zn8_ICSD_416894	insulator	insulator
B3H6N3_ICSD_401085	insulator	insulator
B3Ir4Zn1_ICSD_71640	metal	metal
B3Ir7Sn4_ICSD_78985	metal	metal
B3Ir7Zn5_ICSD_107494	metal	metal
B3K1O6Zn1_ICSD_174357	insulator	insulator
B3K3O6_ICSD_16005	insulator	insulator
B3K3S6_ICSD_79614	insulator	insulator
B3La1O6_ICSD_20355	insulator	insulator
B3La2Na3O9_ICSD_151884	insulator	insulator
B3La3N6_ICSD_410598	insulator	insulator
B3Li1N6Sr4_ICSD_402173	insulator	insulator
B3Li1O5_ICSD_415200	insulator	insulator
B3Li1S6Sr1_ICSD_79616	insulator	insulator
B3Mo1_ICSD_167734	metal	metal
B3Mo3Ni1_ICSD_614824	metal	metal
B3N6Na1Sr4_ICSD_92577	insulator	insulator
B3Na3O9Sc2_ICSD_245063	insulator	insulator
B3Na3S6_ICSD_79613	insulator	insulator
B3Nb2_ICSD_656213	metal	metal
B3Nd1O6_ICSD_20075	insulator	insulator
B3Ni1W3_ICSD_615070	metal	metal
B3Ni4_ICSD_150560	metal	metal
B3Ni4_ICSD_187403	metal	metal
B3O5Rb1_ICSD_91545	insulator	insulator
B3O5Tl1_ICSD_84855	insulator	insulator
B3O6Pr1_ICSD_95850	insulator	insulator
B3O6Rb3_ICSD_59826	insulator	insulator
B3O9Rb1Zn4_ICSD_92616	insulator	insulator
B3O9Sc1Sr3_ICSD_75339	insulator	insulator
B3Os2_ICSD_424879	metal	metal
B3Rb3S6_ICSD_79615	insulator	insulator
B3Re1U1_ICSD_68001	metal	metal
B3Re1_ICSD_24361	metal	metal
B3Re7_ICSD_615242	metal	metal
B3Rh7Zn5_ICSD_107495	metal	metal
B3Rh7_ICSD_615290	metal	metal
B3Ru2_ICSD_108082	metal	metal
B3Ru2_ICSD_23715	metal	metal

Supplementary Table 121. Five-fold cross validated predictions for the metal/insulator classification (113/598).

system	calculated	predicted
B3Ru7_ICSD_44343	metal	metal
B3Si1_ICSD_412621	insulator	metal
B3V2_ICSD_79258	metal	metal
B4Ba2O11Sc2_ICSD_86436	insulator	insulator
B4Ba5F2O10_ICSD_73905	insulator	insulator
B4Bi2Cu5O14_ICSD_260046	metal	insulator
B4C1Cl6O1_ICSD_280617	insulator	insulator
B4C1Dy2_ICSD_418403	metal	metal
B4C2Ca4H6Mg1O18_ICSD_80438	insulator	insulator
B4Ca1O7_ICSD_412710	insulator	insulator
B4Ca2Rh5_ICSD_66768	metal	metal
B4Ce1Co4_ICSD_16193	metal	metal
B4Ce1Co4_ICSD_654433	metal	metal
B4Ce1Cr1_ICSD_612757	metal	metal
B4Ce1Fe4_ICSD_612769	metal	metal
B4Ce1Ni1_ICSD_612813	metal	metal
B4Ce1O12Sc3_ICSD_90839	metal	insulator
B4Ce1Os4_ICSD_612815	metal	metal
B4Ce1Re1_ICSD_81494	metal	metal
B4Ce1Rh4_ICSD_612831	metal	metal
B4Ce1Ru4_ICSD_421307	metal	metal
B4Ce1_ICSD_417745	metal	metal
B4Cl4_ICSD_27872	insulator	insulator
B4Cl5La4_ICSD_418483	metal	metal
B4Co11Dy3_ICSD_97730	metal	metal
B4Co11Y3_ICSD_613415	metal	metal
B4Co1Gd1_ICSD_613074	metal	metal
B4Co1O7_ICSD_420402	insulator	insulator
B4Co1Sc1_ICSD_613278	metal	metal
B4Co1U1_ICSD_613378	metal	metal
B4Co2Mo1_ICSD_44175	metal	metal
B4Co4Ho1_ICSD_613122	metal	metal
B4Co4Nd1_ICSD_2372	metal	metal
B4Co4U1_ICSD_86377	metal	metal
B4Cr1Dy1_ICSD_658658	metal	metal
B4Cr1Gd1_ICSD_613512	metal	metal
B4Cr1Ho1_ICSD_613514	metal	metal
B4Cr1Pr1_ICSD_613542	metal	metal
B4Cr1Tb1_ICSD_613559	metal	metal
B4Cr1Th1_ICSD_81542	metal	metal
B4Cr1U1_ICSD_613562	metal	metal
B4Cr1Y1_ICSD_16171	metal	metal
B4Cr1_ICSD_186851	metal	metal
B4Cr1_ICSD_423380	metal	metal
B4Cr3_ICSD_40791	metal	metal
B4Cu1Er2O10_ICSD_401710	insulator	insulator
B4Cu1Ho2O10_ICSD_408029	insulator	insulator
B4Cu1Li6O10_ICSD_249215	insulator	insulator
B4Cu1O7_ICSD_391408	insulator	insulator
B4Dy1Fe1_ICSD_613623	metal	metal

Supplementary Table 122. Five-fold cross validated predictions for the metal/insulator classification (114/598).

system	calculated	predicted
B4Dy1Mn1_ICSD_613643	metal	metal
B4Dy1Mo1_ICSD_613645	insulator	insulator
B4Dy1Ni1_ICSD_613662	metal	metal
B4Dy1Os1_ICSD_613664	metal	metal
B4Dy1Re1_ICSD_613671	metal	metal
B4Dy1Rh4_ICSD_613679	metal	metal
B4Dy1Ru1_ICSD_613685	metal	metal
B4Dy1Ru4_ICSD_613688	metal	metal
B4Dy1V1_ICSD_613693	metal	metal
B4Dy1W1_ICSD_613694	insulator	insulator
B4Dy1_ICSD_68306	metal	metal
B4Er1Fe1_ICSD_613744	metal	metal
B4Er1Ir4_ICSD_659488	metal	metal
B4Er1Mn1_ICSD_613773	metal	metal
B4Er1Mo1_ICSD_613775	insulator	metal
B4Er1Ni1_ICSD_20894	metal	metal
B4Er1Ni1_ICSD_613777	metal	metal
B4Er1Os1_ICSD_613787	metal	metal
B4Er1Re1_ICSD_613797	metal	metal
B4Er1Rh1_ICSD_603852	metal	metal
B4Er1Rh4_ICSD_49631	metal	metal
B4Er1Ru1_ICSD_613817	metal	metal
B4Er1Ru4_ICSD_613822	metal	metal
B4Er1V1_ICSD_613825	metal	metal
B4Er1W1_ICSD_613826	insulator	insulator
B4Er1_ICSD_68650	metal	metal
B4Eu1Ir4_ICSD_44239	metal	metal
B4Eu2Rh5_ICSD_68540	metal	metal
B4Fe1Gd1_ICSD_613941	metal	metal
B4Fe1O7_ICSD_420401	insulator	insulator
B4Fe1Sc1_ICSD_614180	metal	metal
B4Fe1U1_ICSD_614250	metal	metal
B4Fe1Yb1_ICSD_603709	metal	metal
B4Fe1_ICSD_425311	metal	metal
B4Fe2Mo1_ICSD_44293	metal	metal
B4Fe3La1O12_ICSD_83506	insulator	insulator
B4Fe3Nd1O12_ICSD_83507	insulator	insulator
B4Fe3O12Tb1_ICSD_96455	insulator	insulator
B4Ga2Li6O12_ICSD_9987	insulator	insulator
B4Ga3Nd1O12_ICSD_200321	insulator	insulator
B4Gd1Mn1_ICSD_614344	metal	metal
B4Gd1Mo1_ICSD_614347	insulator	insulator
B4Gd1Ni1_ICSD_614359	metal	metal
B4Gd1Os1_ICSD_614361	metal	metal
B4Gd1Re1_ICSD_614368	metal	metal
B4Gd1Rh4_ICSD_601532	metal	metal
B4Gd1Ru1_ICSD_614384	metal	metal
B4Gd1V1_ICSD_614392	metal	metal
B4Gd1W1_ICSD_614393	insulator	insulator
B4H16Hf1_ICSD_35379	insulator	insulator

Supplementary Table 123. Five-fold cross validated predictions for the metal/insulator classification (115/598).

system	calculated	predicted
B4H16Na1Sc1_ICSD_166748	insulator	insulator
B4H16U1_ICSD_63132	insulator	insulator
B4Hf1Ir3_ICSD_614432	metal	metal
B4Hg1O7_ICSD_415347	insulator	insulator
B4Ho1Mn1_ICSD_614473	metal	metal
B4Ho1Mo1_ICSD_614475	insulator	insulator
B4Ho1Ni1_ICSD_614485	metal	metal
B4Ho1Os1_ICSD_614487	metal	metal
B4Ho1Re1_ICSD_614493	metal	metal
B4Ho1Rh4_ICSD_601533	metal	metal
B4Ho1Ru1_ICSD_614507	metal	metal
B4Ho1Ru4_ICSD_614511	metal	metal
B4Ho1V1_ICSD_658656	metal	metal
B4Ho1W1_ICSD_614514	insulator	insulator
B4Ho1_ICSD_109318	metal	metal
B4Ho2Ni1O10_ICSD_404814	insulator	insulator
B4In5Ir9_ICSD_249113	metal	metal
B4Ir3Sc1_ICSD_44424	metal	metal
B4Ir3Zr1_ICSD_614583	metal	metal
B4Ir4La1_ICSD_614523	metal	metal
B4Ir4Nd1_ICSD_100351	metal	metal
B4Ir4Tb1_ICSD_614568	metal	metal
B4Ir4Y1_ICSD_614580	metal	metal
B4La1O12Sc3_ICSD_83405	insulator	insulator
B4La1O12Sc3_ICSD_89012	insulator	insulator
B4La1O12Sc3_ICSD_89013	insulator	insulator
B4La1Re4_ICSD_614640	metal	metal
B4La1Ru4_ICSD_8078	metal	metal
B4La1_ICSD_2360	metal	metal
B4La2Ni5_ICSD_170618	metal	metal
B4La2Rh5_ICSD_68539	metal	metal
B4Li2O7_ICSD_163177	insulator	insulator
B4Lu1Ni1_ICSD_614685	metal	metal
B4Lu1Os1_ICSD_614693	metal	metal
B4Lu1Ru1_ICSD_614714	metal	metal
B4Lu1_ICSD_109319	metal	metal
B4Mg1Os3_ICSD_91243	metal	metal
B4Mg2Ru5_ICSD_61039	metal	metal
B4Mn1O7_ICSD_391409	insulator	insulator
B4Mn1Tb1_ICSD_614776	metal	metal
B4Mn1U1_ICSD_614778	metal	metal
B4Mn1Y1_ICSD_614788	metal	metal
B4Mn1_ICSD_15079	metal	metal
B4Mn2W1_ICSD_614782	metal	metal
B4Mn3_ICSD_614732	metal	metal
B4Mn4_ICSD_74985	metal	metal
B4Mo1Pu1_ICSD_614831	metal	metal
B4Mo1Tb1_ICSD_614843	insulator	insulator
B4Mo1Th1_ICSD_108898	metal	metal
B4Mo1U1_ICSD_614851	metal	metal

Supplementary Table 124. Five-fold cross validated predictions for the metal/insulator classification (116/598).

system	calculated	predicted
B4Mo1U1_ICSD_614852	metal	metal
B4Mo1Y1_ICSD_20081	insulator	metal
B4Mo1_ICSD_167735	metal	metal
B4Mo1_ICSD_182095	metal	metal
B4Nb3_ICSD_656212	metal	metal
B4Nd1Os4_ICSD_86393	metal	metal
B4Nd1Rh4_ICSD_601574	metal	metal
B4Nd1_ICSD_154656	metal	metal
B4Nd1_ICSD_93225	metal	metal
B4Nd2Ni5_ICSD_63510	metal	metal
B4Ni1O7_ICSD_391407	insulator	insulator
B4Ni1Sc1_ICSD_84184	metal	metal
B4Ni1Tb1_ICSD_615041	metal	metal
B4Ni1U1_ICSD_615063	metal	metal
B4Ni1Y1_ICSD_615085	metal	metal
B4Ni1Yb1_ICSD_603710	metal	metal
B4O11Sc2Sr2_ICSD_86435	insulator	insulator
B4O7Pb1_ICSD_185672	insulator	insulator
B4O7Sn1_ICSD_249206	insulator	insulator
B4O7Sr1_ICSD_27404	insulator	insulator
B4O7Zn1_ICSD_412688	insulator	insulator
B4Os1Pu1_ICSD_615123	metal	metal
B4Os1Pu1_ICSD_615124	metal	metal
B4Os1Tb1_ICSD_615129	metal	metal
B4Os1U1_ICSD_615139	metal	metal
B4Os1Y1_ICSD_615147	metal	metal
B4Os1Yb1_ICSD_615150	metal	metal
B4Os3Sc1_ICSD_91244	metal	metal
B4Os5Sc2_ICSD_603725	metal	metal
B4Pr1Rh4_ICSD_615190	metal	metal
B4Pr1Ru4_ICSD_421308	metal	metal
B4Pr1_ICSD_108073	metal	metal
B4Pu1Re1_ICSD_615226	metal	metal
B4Pu1Ru1_ICSD_615230	metal	metal
B4Pu1Ru1_ICSD_615231	metal	metal
B4Pu1Tc1_ICSD_615233	metal	metal
B4Pu1W1_ICSD_615235	metal	metal
B4Pu1_ICSD_43659	metal	metal
B4Re1Tb1_ICSD_615250	metal	metal
B4Re1Th1_ICSD_615254	metal	metal
B4Re1U1_ICSD_615261	metal	metal
B4Re1U1_ICSD_615262	metal	metal
B4Re1Y1_ICSD_615278	metal	metal
B4Re4Y1_ICSD_615286	metal	metal
B4Rh1Y1_ICSD_419358	metal	metal
B4Rh1Yb1_ICSD_419361	metal	metal
B4Rh4Tb1_ICSD_615322	metal	metal
B4Rh4Th1_ICSD_601578	metal	metal
B4Rh4Y1_ICSD_615346	metal	metal
B4Rh5Sr2_ICSD_66769	metal	metal

Supplementary Table 125. Five-fold cross validated predictions for the metal/insulator classification (117/598).

system	calculated	predicted
B4Rh5_ICSD_86395	metal	metal
B4Rh8Zn5_ICSD_417990	metal	metal
B4Ru1Tb1_ICSD_615371	metal	metal
B4Ru1U1_ICSD_615390	metal	metal
B4Ru1Y1_ICSD_615400	metal	metal
B4Ru1Yb1_ICSD_615408	metal	metal
B4Ru4Y1_ICSD_615406	metal	metal
B4Ta3_ICSD_602946	metal	metal
B4Tb1V1_ICSD_615551	metal	metal
B4Tb1W1_ICSD_615552	insulator	insulator
B4Tb1_ICSD_67223	metal	metal
B4Th1V1_ICSD_615586	metal	metal
B4Th1W1_ICSD_615588	metal	metal
B4Th1_ICSD_81545	metal	metal
B4Ti3_ICSD_615598	metal	metal
B4Tm1_ICSD_109209	metal	metal
B4U1V1_ICSD_615644	metal	metal
B4U1W1_ICSD_615649	metal	metal
B4U1W1_ICSD_615650	metal	metal
B4U1_ICSD_24681	metal	metal
B4V1Y1_ICSD_615679	metal	metal
B4V3_ICSD_615668	metal	metal
B4W1Y1_ICSD_615702	insulator	metal
B4W1_ICSD_615683	metal	metal
B4W1_ICSD_615684	metal	metal
B4Y1_ICSD_26777	metal	metal
B5Ba2Li1O10_ICSD_71875	insulator	insulator
B5Ca1Cl1H2O10Sr1_ICSD_91540	insulator	insulator
B5Ca1Na3O10_ICSD_61165	insulator	insulator
B5Ca2Cl1H2O10_ICSD_100261	insulator	insulator
B5Ca2Cl1H2O10_ICSD_74548	insulator	insulator
B5Ca2Os3_ICSD_59229	metal	metal
B5Ca3La3O15_ICSD_93394	insulator	insulator
B5Cs2Li3O10_ICSD_180730	insulator	insulator
B5Cu1O10Tb1_ICSD_401453	insulator	insulator
B5Eu2Os3_ICSD_59228	metal	metal
B5Gd2_ICSD_62067	metal	metal
B5H12N1O12_ICSD_90001	insulator	insulator
B5H2Li3O10_ICSD_20155	insulator	insulator
B5H8K1O12_ICSD_6292	insulator	insulator
B5H9_ICSD_24636	insulator	insulator
B5Mo2_ICSD_167733	metal	metal
B5Mo2_ICSD_614810	metal	metal
B5Na3O10Sr1_ICSD_260005	insulator	insulator
B5Na3Pt9_ICSD_68093	metal	metal
B5Nd2_ICSD_154655	metal	metal
B5W2_ICSD_20326	metal	metal
B5W2_ICSD_615696	metal	metal
B6Ba1Ni12_ICSD_100287	metal	metal
B6Ba1_ICSD_612529	metal	metal

Supplementary Table 126. Five-fold cross validated predictions for the metal/insulator classification (118/598).

system	calculated	predicted
B6Ba2Ca1O12_ICSD_30890	insulator	insulator
B6Ba2Cd1O12_ICSD_425704	insulator	insulator
B6Ba2Co1O12_ICSD_391014	insulator	insulator
B6Ba2Mg1O12_ICSD_290356	insulator	insulator
B6Ba2Mn1O12_ICSD_391013	insulator	insulator
B6Ba2Ni9_ICSD_100288	metal	metal
B6Ba2O12Zn1_ICSD_404486	insulator	insulator
B6Ba3Ge2O16_ICSD_261403	insulator	insulator
B6Br1K3O10_ICSD_172400	insulator	insulator
B6Ca1Ni12_ICSD_36507	metal	metal
B6Ca1_ICSD_44985	metal	metal
B6Ca2Ni21_ICSD_612695	metal	metal
B6Ca3Rh8_ICSD_66770	metal	metal
B6Ce1Co12_ICSD_612736	metal	metal
B6Ce1Cr2_ICSD_81544	metal	metal
B6Ce1_ICSD_612715	metal	metal
B6Ce2Ni21_ICSD_44151	metal	metal
B6Ce2Re3_ICSD_612821	metal	metal
B6Co12Dy1_ICSD_612901	metal	metal
B6Co12Er1_ICSD_612920	metal	metal
B6Co12La1_ICSD_656613	metal	metal
B6Co12Pr1_ICSD_613225	metal	metal
B6Co12Tb1_ICSD_613339	metal	metal
B6Co12Y1_ICSD_613420	metal	metal
B6Co20Ga3_ICSD_613040	metal	metal
B6Co20In3_ICSD_613126	metal	metal
B6Co20Ti3_ICSD_613353	metal	metal
B6Co20V3_ICSD_613386	metal	metal
B6Co21Ga2_ICSD_613042	metal	metal
B6Co21Ge2_ICSD_613090	metal	metal
B6Co21Hf2_ICSD_613099	metal	metal
B6Co21In2_ICSD_613127	metal	metal
B6Co21Mn2_ICSD_613157	metal	metal
B6Co21Mo2_ICSD_108036	metal	metal
B6Co21Nb2_ICSD_613174	metal	metal
B6Co21Sb2_ICSD_613274	metal	metal
B6Co21Sc2_ICSD_613281	metal	metal
B6Co21Ta2_ICSD_613326	metal	metal
B6Co21Ti2_ICSD_613352	metal	metal
B6Co21U2_ICSD_44187	metal	metal
B6Co21V2_ICSD_613383	metal	metal
B6Co21W2_ICSD_613397	metal	metal
B6Co21Zr2_ICSD_44192	metal	metal
B6Co22In1_ICSD_613128	metal	metal
B6Co22Sb1_ICSD_613275	metal	metal
B6Co23_ICSD_419735	metal	metal
B6Co4O13_ICSD_96561	insulator	insulator
B6Cr1H16_ICSD_170564	insulator	insulator
B6Cr1Sc2_ICSD_99646	metal	metal
B6Cr2Nd1_ICSD_613532	metal	metal

Supplementary Table 127. Five-fold cross validated predictions for the metal/insulator classification (119/598).

system	calculated	predicted
B6Cr2Th1_ICSD_81543	metal	metal
B6Cs1Li1O10_ICSD_75995	insulator	insulator
B6Cs2H6_ICSD_65508	insulator	insulator
B6Cs2O10_ICSD_25815	insulator	insulator
B6Dy1Ni12_ICSD_613649	metal	metal
B6Dy1_ICSD_613601	metal	metal
B6Dy2Ni3_ICSD_613652	metal	metal
B6Dy2Os1_ICSD_603479	metal	metal
B6Dy2Ru1_ICSD_603488	metal	metal
B6Dy4Fe3_ICSD_613611	metal	metal
B6Dy4O15_ICSD_412041	insulator	insulator
B6Er1_ICSD_613715	metal	metal
B6Er2Ni21_ICSD_613776	metal	metal
B6Er2Ni3_ICSD_655633	metal	metal
B6Er2Os1_ICSD_603495	metal	metal
B6Er2Ru1_ICSD_603499	metal	metal
B6Eu1Ni12_ICSD_86371	metal	metal
B6Eu1_ICSD_613830	metal	metal
B6Eu1_ICSD_613840	metal	metal
B6Eu1_ICSD_613842	metal	metal
B6Eu1_ICSD_613846	metal	metal
B6Eu1_ICSD_86268	metal	metal
B6Fe12La1_ICSD_656612	metal	metal
B6Fe23_ICSD_54786	metal	metal
B6Fe2U1_ICSD_167153	metal	metal
B6Fe3Ni20_ICSD_614125	metal	metal
B6Fe3Pr4_ICSD_614152	metal	metal
B6Fe3Tb4_ICSD_614214	metal	metal
B6Fe3Y4_ICSD_614271	metal	metal
B6Ga2Ni21_ICSD_614302	metal	metal
B6Ga3Ni20_ICSD_421820	metal	metal
B6Gd1_ICSD_76125	metal	metal
B6Gd2Os1_ICSD_603503	metal	metal
B6Gd2Re3_ICSD_614372	metal	metal
B6H10_ICSD_43253	insulator	insulator
B6H5K2N1O2_ICSD_67890	insulator	insulator
B6H6K2_ICSD_65507	insulator	insulator
B6Hf2Ni21_ICSD_614444	metal	metal
B6Hf3Ni20_ICSD_614442	metal	metal
B6Ho1_ICSD_614466	metal	metal
B6Ho2Ni3_ICSD_614480	metal	metal
B6Ho2Os1_ICSD_603477	metal	metal
B6Ho2Ru1_ICSD_603498	metal	metal
B6Ho4O15_ICSD_412991	insulator	insulator
B6In2Ni21_ICSD_380216	metal	metal
B6K1_ICSD_98987	metal	metal
B6La1Ni12_ICSD_39323	metal	metal
B6La1_ICSD_614595	metal	metal
B6La3Ru8_ICSD_159927	metal	metal
B6Lu1_ICSD_614680	metal	metal

Supplementary Table 128. Five-fold cross validated predictions for the metal/insulator classification (120/598).

system	calculated	predicted
B6Lu2Ni3_ICSD_68002	metal	metal
B6Mn2Ni21_ICSD_614758	metal	metal
B6Mn3Ni20_ICSD_614760	metal	metal
B6Na2O11Zn1_ICSD_167333	insulator	insulator
B6Nd1_ICSD_614939	metal	metal
B6Nd2Re3_ICSD_614953	metal	metal
B6Ni12Sr1_ICSD_100286	metal	metal
B6Ni12Tb1_ICSD_44476	metal	metal
B6Ni12Tb1_ICSD_615029	metal	metal
B6Ni12Y1_ICSD_615075	metal	metal
B6Ni20Sc3_ICSD_615002	metal	metal
B6Ni20Ta3_ICSD_615021	metal	metal
B6Ni20Ti3_ICSD_615043	metal	metal
B6Ni20V3_ICSD_615065	metal	metal
B6Ni20Zr3_ICSD_615099	metal	metal
B6Ni21Sb2_ICSD_614998	metal	metal
B6Ni21Sc2_ICSD_409959	metal	metal
B6Ni21Sn2_ICSD_109112	metal	metal
B6Ni21Ta2_ICSD_615022	metal	metal
B6Ni21Ti2_ICSD_615045	metal	metal
B6Ni21U2_ICSD_44533	metal	metal
B6Ni21Yb2_ICSD_615087	metal	metal
B6Ni21Zr2_ICSD_44193	metal	metal
B6Ni23_ICSD_54787	metal	metal
B6Ni3Tb2_ICSD_615033	metal	metal
B6Ni3Tm2_ICSD_615052	metal	metal
B6Ni3Y2_ICSD_615078	metal	metal
B6Ni3Yb2_ICSD_39394	metal	metal
B6O13Zn4_ICSD_100290	insulator	insulator
B6O18Sc1Sr6Y1_ICSD_67648	insulator	insulator
B6O1_ICSD_71065	insulator	insulator
B6Os1Tb2_ICSD_603504	metal	metal
B6Os1Y2_ICSD_603494	metal	metal
B6Os1Yb2_ICSD_603702	metal	metal
B6Os8Y3_ICSD_417473	metal	metal
B6Pr1_ICSD_615181	metal	metal
B6Pr2Re3_ICSD_86366	metal	metal
B6Pu1_ICSD_615214	metal	metal
B6Re1Y2_ICSD_16187	metal	metal
B6Rh1Sc2_ICSD_152716	metal	metal
B6Rh8Sr3_ICSD_66771	metal	metal
B6Ru1Tb2_ICSD_603496	metal	metal
B6Ru1Y2_ICSD_603493	metal	metal
B6Ru1Yb2_ICSD_603501	metal	metal
B6Si1_ICSD_20240	insulator	metal
B6Sr1_ICSD_615493	metal	metal
B6Tb1_ICSD_615549	metal	metal
B6Th1_ICSD_615568	metal	metal
B6Tm1_ICSD_615612	metal	metal
B6Y1_ICSD_615714	metal	metal

Supplementary Table 129. Five-fold cross validated predictions for the metal/insulator classification (121/598).

system	calculated	predicted
B6Yb1_ICSD_615747	metal	metal
B7Br1Cu3O13_ICSD_201347	insulator	insulator
B7Br1Mn3O13_ICSD_80420	insulator	insulator
B7Cl1Co3O13_ICSD_158297	insulator	insulator
B7Cl1Cr3O13_ICSD_4231	insulator	insulator
B7Cl1Mg3O13_ICSD_30351	insulator	insulator
B7Cl1O13Zn3_ICSD_55444	insulator	insulator
B7Co4Nb3_ICSD_62282	metal	metal
B7Cr1Er3_ICSD_62630	metal	metal
B7Cr1Ho3_ICSD_56994	metal	metal
B7Cr3I1O13_ICSD_62178	insulator	insulator
B7Cu3I1O13_ICSD_61058	insulator	insulator
B7Dy3Fe1_ICSD_613612	metal	metal
B7Dy3Mn1_ICSD_39509	metal	metal
B7Dy3Re1_ICSD_613670	metal	metal
B7Dy3W1_ICSD_656435	metal	metal
B7Er3Fe1_ICSD_613731	metal	metal
B7Er3Mn1_ICSD_613774	metal	metal
B7Er3Re1_ICSD_613796	metal	metal
B7Er3W1_ICSD_656437	metal	metal
B7Fe1Ho3_ICSD_613979	metal	metal
B7Fe1Tb3_ICSD_614215	metal	metal
B7Fe1Y3_ICSD_64596	metal	metal
B7Fe3I1O13_ICSD_78420	insulator	insulator
B7Gd3Mn1_ICSD_614343	metal	metal
B7Gd3Re1_ICSD_614367	metal	metal
B7Gd3W1_ICSD_656433	metal	metal
B7Ho1Mo3_ICSD_39943	metal	metal
B7Ho3Mn1_ICSD_614474	metal	metal
B7Ho3Re1_ICSD_614492	metal	metal
B7Ho3W1_ICSD_656436	metal	metal
B7I1Ni3O13_ICSD_27946	insulator	insulator
B7Mn1Tb3_ICSD_614777	metal	metal
B7Mn1Y3_ICSD_614789	metal	metal
B7Mo3Y1_ICSD_39873	metal	metal
B7Re1Tb3_ICSD_615249	metal	metal
B7Re1Y3_ICSD_2475	metal	metal
B7Re1Y3_ICSD_64595	metal	metal
B7Tb3W1_ICSD_656434	metal	metal
B7W1Y3_ICSD_39663	metal	metal
B8Ca1H4O15_ICSD_250323	insulator	insulator
B8Cu1Ho2O16_ICSD_408028	metal	insulator
B8La9Na3O27_ICSD_95753	insulator	insulator
B8Nb7Ru6_ICSD_263041	metal	metal
B8Ru11_ICSD_43663	metal	metal
B8Ru7Sr2_ICSD_33848	metal	metal
B8S16_ICSD_15267	insulator	insulator
B9Ba1Li1O15_ICSD_93013	insulator	insulator
B9Ba1Na1O15_ICSD_93014	insulator	insulator
B9Li1_ICSD_164625	metal	metal

Supplementary Table 130. Five-fold cross validated predictions for the metal/insulator classification (122/598).

system	calculated	predicted
B9Mg1N1_ICSD_280938	insulator	metal
Ba10N12Ti4_ICSD_79102	insulator	insulator
Ba10O24P6S1_ICSD_410785	insulator	insulator
Ba11Bi10_ICSD_51797	metal	metal
Ba11Bi14Cd8_ICSD_240338	metal	metal
Ba11Cd6Sb12_ICSD_418886	metal	metal
Ba11In6O3_ICSD_416096	metal	metal
Ba11Sb10_ICSD_413518	metal	insulator
Ba12Li8N6Na15_ICSD_417928	metal	metal
Ba14Ca1N6Na14_ICSD_81845	metal	metal
Ba14Li1N6Na14_ICSD_418457	metal	metal
Ba14Li5N6Na11_ICSD_418455	metal	metal
Ba14Li6N6Na10_ICSD_418456	metal	metal
Ba1Be13_ICSD_58633	metal	metal
Ba1Be1F1O4P1_ICSD_200922	insulator	insulator
Ba1Be1F4_ICSD_414412	insulator	insulator
Ba1Be1La2O5_ICSD_65292	insulator	insulator
Ba1Be1O4Si1_ICSD_86792	insulator	insulator
Ba1Be2N2_ICSD_415304	insulator	insulator
Ba1Be2O7Si2_ICSD_100030	insulator	insulator
Ba1Be2O7Si2_ICSD_263133	insulator	insulator
Ba1Bi1Cl1O2_ICSD_79532	insulator	insulator
Ba1Bi1Cu1_ICSD_106303	metal	metal
Ba1Bi1I1O2_ICSD_97511	insulator	insulator
Ba1Bi1Na1_ICSD_413810	insulator	metal
Ba1Bi1O3_ICSD_151895	insulator	insulator
Ba1Bi1O3_ICSD_151896	metal	insulator
Ba1Bi1O3_ICSD_172756	insulator	insulator
Ba1Bi1Se3_ICSD_10505	insulator	insulator
Ba1Bi2La1S6_ICSD_85461	insulator	insulator
Ba1Bi2Mg2_ICSD_100049	insulator	insulator
Ba1Bi2Mo4O16_ICSD_416023	insulator	insulator
Ba1Bi2Pd2_ICSD_416299	metal	metal
Ba1Bi3_ICSD_58634	metal	metal
Ba1Br1Cl1_ICSD_35458	insulator	insulator
Ba1Br1F1_ICSD_155005	insulator	insulator
Ba1Br1H1_ICSD_25544	insulator	insulator
Ba1Br2H4O2_ICSD_33949	insulator	insulator
Ba1Br2O6_ICSD_40287	insulator	insulator
Ba1Br2O6_ICSD_66035	insulator	insulator
Ba1Br2_ICSD_262675	insulator	insulator
Ba1Br2_ICSD_79892	insulator	insulator
Ba1Br6Th1_ICSD_78770	insulator	insulator
Ba1C1Cl1H2N1O1S1_ICSD_82878	insulator	insulator
Ba1C1Cl1N1S1_ICSD_94400	insulator	insulator
Ba1C1Cu1F2O3_ICSD_79864	insulator	insulator
Ba1C1F2Mn1O3_ICSD_95740	insulator	insulator
Ba1C1F2O3Zn1_ICSD_95739	insulator	insulator
Ba1C1N2_ICSD_75041	insulator	insulator
Ba1C1O3_ICSD_158389	insulator	insulator

Supplementary Table 131. Five-fold cross validated predictions for the metal/insulator classification (123/598).

system	calculated	predicted
Ba1C1O3_ICSD_91897	insulator	insulator
Ba1C1Si1_ICSD_168411	insulator	insulator
Ba1C1Si1_ICSD_168413	insulator	insulator
Ba1C2Ca1O6_ICSD_100477	insulator	insulator
Ba1C2Ca1O6_ICSD_157982	insulator	insulator
Ba1C2Ca2F2O6_ICSD_245746	insulator	insulator
Ba1C2Ce1F1O6_ICSD_37195	insulator	insulator
Ba1C2Ce1F1O6_ICSD_74178	insulator	insulator
Ba1C2F2O6Pb2_ICSD_280899	insulator	insulator
Ba1C2H2O4_ICSD_151335	insulator	insulator
Ba1C2H2O5_ICSD_151115	insulator	insulator
Ba1C2H4O6_ICSD_162707	insulator	insulator
Ba1C2Mg1O6_ICSD_24435	insulator	insulator
Ba1C2Mg1O6_ICSD_89038	insulator	insulator
Ba1C2N2S2_ICSD_94428	insulator	insulator
Ba1C2O4_ICSD_261703	insulator	insulator
Ba1C2_ICSD_168408	insulator	insulator
Ba1C2_ICSD_186576	insulator	insulator
Ba1C2_ICSD_88098	insulator	insulator
Ba1C2_ICSD_88102	insulator	insulator
Ba1C4Ce2F1Na1O12_ICSD_77499	insulator	insulator
Ba1C4H6O7_ICSD_62387	insulator	insulator
Ba1C4H8N4O4Pt1_ICSD_851	insulator	insulator
Ba1C4O4_ICSD_412830	insulator	insulator
Ba1C8N8Pt2Rb2_ICSD_409861	insulator	insulator
Ba1Ca1Fe4O7_ICSD_161791	insulator	insulator
Ba1Ca1Fe4O8_ICSD_15174	insulator	insulator
Ba1Ca1Ga4O8_ICSD_280042	insulator	insulator
Ba1Ca1Ge1_ICSD_52681	metal	metal
Ba1Ca1O4Si1_ICSD_67092	insulator	insulator
Ba1Ca1O7V2_ICSD_80810	insulator	insulator
Ba1Ca1Pb1_ICSD_615799	insulator	metal
Ba1Ca1Si1_ICSD_52682	metal	metal
Ba1Ca1Sn1_ICSD_58641	insulator	metal
Ba1Ca2Mg1O8Si2_ICSD_422406	insulator	insulator
Ba1Ca2N12P6_ICSD_415714	insulator	insulator
Ba1Ca2O9Si3_ICSD_24426	insulator	insulator
Ba1Ca4Co2N4_ICSD_409921	insulator	insulator
Ba1Ca4Cu2N4_ICSD_86066	insulator	insulator
Ba1Cd0.333O3Ta0.667_ICSD_156335	insulator	insulator
Ba1Cd11_ICSD_615809	metal	metal
Ba1Cd1F1Sb1_ICSD_421815	insulator	insulator
Ba1Cd1Ge1S4_ICSD_23343	insulator	insulator
Ba1Cd1Ge1_ICSD_106305	metal	metal
Ba1Cd1K2Sb2_ICSD_422272	metal	insulator
Ba1Cd1O7P2_ICSD_72673	insulator	insulator
Ba1Cd1O7V2_ICSD_80811	insulator	insulator
Ba1Cd1S2_ICSD_66655	insulator	insulator
Ba1Cd1Sb2Yb1_ICSD_422280	insulator	metal
Ba1Cd1_ICSD_58642	metal	metal

Supplementary Table 132. Five-fold cross validated predictions for the metal/insulator classification (124/598).

system	calculated	predicted
Ba1Cd2Ge2_ICSD_174263	metal	metal
Ba1Cd2P2_ICSD_615814	insulator	insulator
Ba1Cd2Sb2_ICSD_32021	metal	insulator
Ba1Cd2_ICSD_260668	metal	metal
Ba1Ce1Cu1S3_ICSD_659174	insulator	insulator
Ba1Ce1Cu1Se3_ICSD_659176	insulator	insulator
Ba1Ce1N2_ICSD_74791	insulator	insulator
Ba1Ce1O3_ICSD_29109	insulator	metal
Ba1Ce1O3_ICSD_79627	insulator	insulator
Ba1Ce1O3_ICSD_88590	insulator	insulator
Ba1Ce1O3_ICSD_94347	insulator	insulator
Ba1Ce2Co1S5_ICSD_93708	insulator	insulator
Ba1Ce2Mn1S5_ICSD_91228	insulator	insulator
Ba1Ce2S5Zn1_ICSD_93712	insulator	insulator
Ba1Cl1Cu1O4P1_ICSD_79883	insulator	insulator
Ba1Cl1F1_ICSD_35491	insulator	insulator
Ba1Cl1H1O1_ICSD_74721	insulator	insulator
Ba1Cl1H1_ICSD_37201	insulator	insulator
Ba1Cl1N3_ICSD_418423	metal	insulator
Ba1Cl1O2Sb1_ICSD_200962	insulator	insulator
Ba1Cl1O3V1_ICSD_50786	insulator	insulator
Ba1Cl2Cu2O6Te2_ICSD_85786	metal	insulator
Ba1Cl2H2O1_ICSD_60886	insulator	insulator
Ba1Cl2H4O2_ICSD_2254	insulator	insulator
Ba1Cl2H6O11_ICSD_65020	insulator	insulator
Ba1Cl2Hg2O2_ICSD_77509	insulator	insulator
Ba1Cl2O6_ICSD_40285	insulator	insulator
Ba1Cl2_ICSD_183924	insulator	insulator
Ba1Cl2_ICSD_2190	insulator	insulator
Ba1Cl2_ICSD_2191	insulator	insulator
Ba1Cl2_ICSD_79891	insulator	insulator
Ba1Cl4Pd1_ICSD_411837	insulator	insulator
Ba1Cl4Zn1_ICSD_410193	insulator	insulator
Ba1Cl4Zn1_ICSD_411951	insulator	insulator
Ba1Cl5Gd1_ICSD_407832	insulator	insulator
Ba1Co1Dy2O5_ICSD_74974	insulator	insulator
Ba1Co1Dy2O5_ICSD_85060	insulator	insulator
Ba1Co1Er2O5_ICSD_65469	insulator	insulator
Ba1Co1Er2O5_ICSD_73168	insulator	insulator
Ba1Co1Eu2O5_ICSD_78173	metal	metal
Ba1Co1F4_ICSD_261189	insulator	insulator
Ba1Co1F6Li1_ICSD_41178	insulator	insulator
Ba1Co1Gd2O5_ICSD_85059	metal	insulator
Ba1Co1Ho2O5_ICSD_65467	insulator	insulator
Ba1Co1K2N6O12_ICSD_23732	metal	metal
Ba1Co1K2N6O12_ICSD_61070	metal	metal
Ba1Co1La2S5_ICSD_95267	insulator	insulator
Ba1Co1Lu2O5_ICSD_74977	insulator	insulator
Ba1Co1Nd2O5_ICSD_202973	insulator	metal
Ba1Co1Nd2S5_ICSD_96554	insulator	insulator

Supplementary Table 133. Five-fold cross validated predictions for the metal/insulator classification (125/598).

system	calculated	predicted
Ba1Co1O2_ICSD_25813	insulator	metal
Ba1Co1O3_ICSD_88670	insulator	metal
Ba1Co1O4Si1_ICSD_73778	insulator	insulator
Ba1Co1O5Y2_ICSD_85061	insulator	insulator
Ba1Co1O7P2_ICSD_202853	insulator	insulator
Ba1Co1Pr2S5_ICSD_93709	metal	insulator
Ba1Co1S2_ICSD_246938	metal	metal
Ba1Co1S2_ICSD_75515	metal	metal
Ba1Co2Ge2_ICSD_420975	metal	metal
Ba1Co2H2O9P2_ICSD_84830	insulator	insulator
Ba1Co2La1O6_ICSD_153495	metal	metal
Ba1Co2Nd1O5_ICSD_96366	metal	metal
Ba1Co2Nd1O6_ICSD_154107	metal	metal
Ba1Co2O5Y1_ICSD_171432	metal	metal
Ba1Co2O5Y1_ICSD_171435	metal	metal
Ba1Co2O5Y1_ICSD_247220	metal	metal
Ba1Co2O7Si2_ICSD_74160	insulator	insulator
Ba1Co3H2O10V2_ICSD_236321	insulator	insulator
Ba1Co4Dy1O7_ICSD_59795	metal	metal
Ba1Co4Ho1O7_ICSD_420423	insulator	insulator
Ba1Co4Ho1O7_ICSD_420425	metal	metal
Ba1Co4Ho1O7_ICSD_420426	insulator	insulator
Ba1Co4Ho1O7_ICSD_420427	insulator	insulator
Ba1Co4In1O7_ICSD_59794	metal	metal
Ba1Co4Lu1O7_ICSD_59780	metal	metal
Ba1Co4O7.2Yb1_ICSD_172418	metal	metal
Ba1Co4O7Y1_ICSD_188853	insulator	metal
Ba1Co4O7Y1_ICSD_95745	insulator	metal
Ba1Co4O7Y1_ICSD_95747	metal	insulator
Ba1Cr10O15_ICSD_82100	metal	insulator
Ba1Cr1F5_ICSD_31705	insulator	insulator
Ba1Cr1F6_ICSD_10341	metal	insulator
Ba1Cr1O3_ICSD_35029	metal	insulator
Ba1Cr1O3_ICSD_35459	metal	insulator
Ba1Cr1O4_ICSD_188534	insulator	insulator
Ba1Cr1O4_ICSD_62560	insulator	insulator
Ba1Cr1S2_ICSD_165626	insulator	insulator
Ba1Cs2H6N2O12_ICSD_411040	insulator	insulator
Ba1Cu10P4_ICSD_83618	metal	metal
Ba1Cu13_ICSD_154391	metal	metal
Ba1Cu1Dy1Te3_ICSD_85601	insulator	insulator
Ba1Cu1Dy2O5_ICSD_93463	insulator	insulator
Ba1Cu1Er1S3_ICSD_78176	insulator	insulator
Ba1Cu1Er1Se3_ICSD_659168	insulator	insulator
Ba1Cu1Er2O5_ICSD_72165	insulator	insulator
Ba1Cu1F1S1_ICSD_183712	insulator	insulator
Ba1Cu1F1Se1_ICSD_75585	insulator	insulator
Ba1Cu1F1Te1_ICSD_245624	insulator	insulator
Ba1Cu1F4_ICSD_9930	insulator	insulator
Ba1Cu1F7Fe1_ICSD_60952	insulator	insulator

Supplementary Table 134. Five-fold cross validated predictions for the metal/insulator classification (126/598).

system	calculated	predicted
Ba1Cu1Fe1Lu1O5_ICSD_66404	metal	metal
Ba1Cu1Fe1O5Y1_ICSD_71355	metal	metal
Ba1Cu1Gd1Se3_ICSD_280698	insulator	insulator
Ba1Cu1Ho2O5_ICSD_72163	insulator	insulator
Ba1Cu1K2N6O12_ICSD_1830	metal	metal
Ba1Cu1La1S3_ICSD_659175	insulator	insulator
Ba1Cu1La1Se3_ICSD_659178	insulator	insulator
Ba1Cu1La1Te3_ICSD_88715	insulator	insulator
Ba1Cu1N1_ICSD_86064	insulator	insulator
Ba1Cu1Na2O8V2_ICSD_72364	insulator	insulator
Ba1Cu1Nd1S3_ICSD_659173	insulator	insulator
Ba1Cu1Nd2O5_ICSD_154576	insulator	insulator
Ba1Cu1O10Si4_ICSD_71864	insulator	insulator
Ba1Cu1O5Pr2_ICSD_85952	insulator	insulator
Ba1Cu1O5Y2_ICSD_72572	insulator	insulator
Ba1Cu1O6Se2_ICSD_202387	insulator	insulator
Ba1Cu1O6Se2_ICSD_202388	insulator	insulator
Ba1Cu1O7Te2_ICSD_404297	metal	insulator
Ba1Cu1O7V2_ICSD_40839	insulator	insulator
Ba1Cu1P1_ICSD_52684	metal	insulator
Ba1Cu1S3Sc1_ICSD_659165	insulator	insulator
Ba1Cu1S3Y1_ICSD_659166	insulator	insulator
Ba1Cu1Sb1_ICSD_57020	metal	metal
Ba1Cu1Se3Y1_ICSD_659169	insulator	insulator
Ba1Cu1Sn2_ICSD_58647	metal	metal
Ba1Cu1Te3Y1_ICSD_88716	insulator	insulator
Ba1Cu1_ICSD_58646	metal	metal
Ba1Cu2Ga1_ICSD_615828	metal	metal
Ba1Cu2Ge1S4_ICSD_10006	insulator	insulator
Ba1Cu2Ge1Se4_ICSD_411404	insulator	insulator
Ba1Cu2Ge2O7_ICSD_77133	insulator	insulator
Ba1Cu2O2_ICSD_9456	insulator	metal
Ba1Cu2O7Si2_ICSD_51281	insulator	insulator
Ba1Cu2P4_ICSD_68802	metal	metal
Ba1Cu2S2_ICSD_89573	insulator	insulator
Ba1Cu2S2_ICSD_89575	insulator	insulator
Ba1Cu2S4Sn1_ICSD_52685	insulator	insulator
Ba1Cu2Sb2_ICSD_236308	metal	metal
Ba1Cu2Se2_ICSD_89574	insulator	insulator
Ba1Cu2Se2_ICSD_89576	insulator	insulator
Ba1Cu2Se4Sn1_ICSD_170857	insulator	insulator
Ba1Cu2Te2_ICSD_51444	insulator	insulator
Ba1Cu3H2O10V2_ICSD_67726	insulator	insulator
Ba1Cu3O4_ICSD_65881	metal	insulator
Ba1Cu3O4_ICSD_83079	insulator	metal
Ba1Cu4O17P4V1_ICSD_406667	insulator	insulator
Ba1Cu4S3_ICSD_15138	insulator	insulator
Ba1Cu4S3_ICSD_15139	insulator	insulator
Ba1Cu5La4O12_ICSD_79398	insulator	metal
Ba1Cu8P4_ICSD_66016	metal	metal

Supplementary Table 135. Five-fold cross validated predictions for the metal/insulator classification (127/598).

system	calculated	predicted
Ba1Cu9Ge4_ICSD_412885	metal	metal
Ba1Cu9Si4_ICSD_412883	metal	metal
Ba1Cu9Sn4_ICSD_424220	metal	metal
Ba1Dy1Fe2O4.996_ICSD_99611	insulator	metal
Ba1Dy1Fe4O7_ICSD_166241	metal	metal
Ba1Dy2Ni1O5_ICSD_72627	insulator	insulator
Ba1Dy2O5Pd1_ICSD_404496	insulator	insulator
Ba1Dy2S4_ICSD_615841	insulator	insulator
Ba1Dy2Se4_ICSD_615842	insulator	insulator
Ba1Dy2Te4_ICSD_90334	insulator	insulator
Ba1Er1Mn2O5_ICSD_188495	metal	metal
Ba1Er1Mn2O5_ICSD_188496	metal	metal
Ba1Er2F8_ICSD_151699	insulator	insulator
Ba1Er2Ni1O5_ICSD_69323	insulator	insulator
Ba1Er2O10Si3_ICSD_167615	insulator	insulator
Ba1Er2S4_ICSD_188656	insulator	insulator
Ba1Er2Se4_ICSD_615844	insulator	insulator
Ba1Er2Te4_ICSD_90337	insulator	insulator
Ba1Eu1Fe2O5_ICSD_416716	metal	metal
Ba1Eu2Ni1O5_ICSD_68086	metal	metal
Ba1Eu2O5Pd1_ICSD_83232	metal	insulator
Ba1Eu2O5Pt1_ICSD_68085	metal	insulator
Ba1F10Te2_ICSD_81862	insulator	insulator
Ba1F10Zr2_ICSD_202530	insulator	insulator
Ba1F11Li1Zr2_ICSD_67512	insulator	insulator
Ba1F12Sb2_ICSD_39346	insulator	insulator
Ba1F1I1_ICSD_1128	insulator	insulator
Ba1F1O6P1U1_ICSD_249983	insulator	insulator
Ba1F2Fe2O7P2_ICSD_88824	insulator	insulator
Ba1F2_ICSD_181246	insulator	insulator
Ba1F2_ICSD_183923	insulator	insulator
Ba1F2_ICSD_41650	insulator	insulator
Ba1F3H1_ICSD_35409	insulator	insulator
Ba1F3Li1_ICSD_45310	insulator	insulator
Ba1F4Fe1_ICSD_82765	insulator	insulator
Ba1F4Mg1_ICSD_182596	insulator	insulator
Ba1F4Mg1_ICSD_182599	insulator	insulator
Ba1F4Mn1_ICSD_182603	insulator	insulator
Ba1F4Ni1_ICSD_261190	insulator	insulator
Ba1F4O1Ti1_ICSD_72740	insulator	insulator
Ba1F4Pd1_ICSD_108991	insulator	insulator
Ba1F4Sn1_ICSD_166207	insulator	insulator
Ba1F4Zn1_ICSD_402925	insulator	insulator
Ba1F5Ga1_ICSD_200316	insulator	insulator
Ba1F5Mn1_ICSD_38317	insulator	insulator
Ba1F5Sb1_ICSD_68455	insulator	insulator
Ba1F6Ge1_ICSD_26614	insulator	insulator
Ba1F6Ni1_ICSD_35396	insulator	insulator
Ba1F6Pb1_ICSD_25521	insulator	insulator
Ba1F6Rh1_ICSD_6038	metal	insulator

Supplementary Table 136. Five-fold cross validated predictions for the metal/insulator classification (128/598).

system	calculated	predicted
Ba1F6Si1_ICSD_26613	insulator	insulator
Ba1F6Sn1_ICSD_33788	insulator	insulator
Ba1F6Tb1_ICSD_59306	metal	insulator
Ba1F6Te1_ICSD_88416	insulator	insulator
Ba1F6Ti1_ICSD_33789	insulator	insulator
Ba1F6Zr1_ICSD_1697	insulator	insulator
Ba1F6Zr1_ICSD_36122	insulator	insulator
Ba1F7Fe1Zn1_ICSD_36001	insulator	insulator
Ba1F7Ga1Mn1_ICSD_201525	insulator	insulator
Ba1F7Na1Zr1_ICSD_67515	insulator	insulator
Ba1F7Ta1_ICSD_417251	insulator	insulator
Ba1F8Tm2_ICSD_20103	insulator	insulator
Ba1Fe1H1O5P1_ICSD_174376	insulator	insulator
Ba1Fe1K1O3_ICSD_412877	insulator	insulator
Ba1Fe1La2S5_ICSD_51602	insulator	insulator
Ba1Fe1Nd2S5_ICSD_51605	insulator	insulator
Ba1Fe1O10Si4_ICSD_156832	insulator	insulator
Ba1Fe1O10Si4_ICSD_31205	insulator	insulator
Ba1Fe1O3_ICSD_262132	metal	metal
Ba1Fe1Pr2S5_ICSD_51604	insulator	insulator
Ba1Fe2Gd1O5_ICSD_247569	metal	insulator
Ba1Fe2O4_ICSD_171001	insulator	insulator
Ba1Fe2O4_ICSD_2769	insulator	insulator
Ba1Fe2O5Y1_ICSD_281203	insulator	metal
Ba1Fe2P2_ICSD_169745	metal	metal
Ba1Fe2S3_ICSD_16307	insulator	metal
Ba1Fe2S4_ICSD_23081	insulator	metal
Ba1Fe2Se3_ICSD_290594	insulator	metal
Ba1Fe2Se3_ICSD_290595	insulator	insulator
Ba1Fe2Se3_ICSD_424315	insulator	insulator
Ba1Fe4O7Y1_ICSD_262841	metal	metal
Ba1Fe4O7Y1_ICSD_262842	metal	metal
Ba1Fe4O8Sr1_ICSD_1943	insulator	insulator
Ba1Fe4O8Sr1_ICSD_2536	metal	insulator
Ba1Fe4O8Sr1_ICSD_37011	insulator	insulator
Ba1Fe4Sb12_ICSD_658735	metal	metal
Ba1Ga1Ge1H1_ICSD_173573	insulator	insulator
Ba1Ga1Ge1_ICSD_615870	metal	metal
Ba1Ga1H1Si1_ICSD_173572	insulator	insulator
Ba1Ga1H1Sn1_ICSD_173574	insulator	insulator
Ba1Ga1H4_ICSD_240693	metal	insulator
Ba1Ga1H5_ICSD_240693	insulator	insulator
Ba1Ga1La1O4_ICSD_180536	insulator	insulator
Ba1Ga1Nd1O4_ICSD_69641	insulator	insulator
Ba1Ga2P2_ICSD_380479	insulator	insulator
Ba1Ga2Pt2_ICSD_50160	metal	metal
Ba1Ga2S4_ICSD_615871	insulator	insulator
Ba1Ga2Sb2_ICSD_280662	insulator	metal
Ba1Ga2Se4_ICSD_24386	insulator	insulator
Ba1Ga2_ICSD_246238	metal	metal

Supplementary Table 137. Five-fold cross validated predictions for the metal/insulator classification (129/598).

system	calculated	predicted
Ba1Ga4S7_ICSD_162960	insulator	insulator
Ba1Ga4_ICSD_55449	metal	metal
Ba1Gd2Ni1O5_ICSD_62758	metal	metal
Ba1Gd2O5Pd1_ICSD_83233	insulator	insulator
Ba1Gd2O5Pt1_ICSD_202171	insulator	insulator
Ba1Gd2O7Sc2_ICSD_167604	insulator	insulator
Ba1Gd2S4_ICSD_615879	insulator	insulator
Ba1Gd2Te4_ICSD_90332	metal	insulator
Ba1Ge12Pt4_ICSD_174551	metal	metal
Ba1Ge1Mn1_ICSD_615891	metal	metal
Ba1Ge1Ni2_ICSD_421431	metal	metal
Ba1Ge1O3_ICSD_23925	insulator	insulator
Ba1Ge1O8P2_ICSD_423040	insulator	insulator
Ba1Ge1Pt1_ICSD_106309	metal	metal
Ba1Ge1Zn1_ICSD_106310	metal	metal
Ba1Ge1_ICSD_659524	metal	metal
Ba1Ge2Li1_ICSD_162583	metal	metal
Ba1Ge2Li2Mg2_ICSD_409576	metal	metal
Ba1Ge2Mg2_ICSD_25312	metal	metal
Ba1Ge2Mn2_ICSD_404	metal	metal
Ba1Ge2Mn2_ICSD_80502	metal	metal
Ba1Ge2Mn2_ICSD_80503	metal	metal
Ba1Ge2O5_ICSD_60061	insulator	insulator
Ba1Ge2P2_ICSD_26416	insulator	insulator
Ba1Ge2Rh2_ICSD_77146	metal	metal
Ba1Ge2_ICSD_100129	metal	metal
Ba1Ge2_ICSD_615881	insulator	metal
Ba1Ge3Ir1_ICSD_174264	metal	metal
Ba1Ge3Mg4_ICSD_165631	metal	metal
Ba1Ge3O9Sn1_ICSD_10384	insulator	insulator
Ba1Ge3Pd1_ICSD_174261	metal	metal
Ba1Ge3Pt1_ICSD_409867	metal	metal
Ba1Ge3Rh1_ICSD_174265	metal	metal
Ba1Ge3_ICSD_261651	metal	metal
Ba1H1I1_ICSD_37203	insulator	insulator
Ba1H2N2O5_ICSD_200864	insulator	insulator
Ba1H2N2O5_ICSD_65641	insulator	insulator
Ba1H2Ni3O10V2_ICSD_424330	insulator	insulator
Ba1H2O14P4Si1_ICSD_41357	insulator	insulator
Ba1H2O14Se4V2_ICSD_79518	insulator	insulator
Ba1H2O4Sn2_ICSD_37115	insulator	insulator
Ba1H2O8S2_ICSD_62494	insulator	insulator
Ba1H2_ICSD_615909	insulator	insulator
Ba1H2_ICSD_62562	metal	metal
Ba1H3Li1_ICSD_23977	insulator	insulator
Ba1H3Li1_ICSD_416463	insulator	insulator
Ba1H3Na1Pd1_ICSD_165609	insulator	insulator
Ba1H4I2O2_ICSD_407360	insulator	insulator
Ba1H4O3_ICSD_63017	insulator	insulator
Ba1H4O4P2_ICSD_59934	insulator	insulator

Supplementary Table 138. Five-fold cross validated predictions for the metal/insulator classification (130/598).

system	calculated	predicted
Ba1H4O8P2_ICSD_1297	insulator	insulator
Ba1H6O8Si2_ICSD_26971	insulator	insulator
Ba1H7Re1_ICSD_247116	insulator	insulator
Ba1H8O5_ICSD_67109	insulator	insulator
Ba1H9Re1_ICSD_247104	insulator	insulator
Ba1H9Re1_ICSD_247107	insulator	insulator
Ba1H9Re1_ICSD_247109	insulator	insulator
Ba1H9Re1_ICSD_247110	insulator	insulator
Ba1H9Re1_ICSD_247111	insulator	insulator
Ba1H9Re1_ICSD_75460	insulator	insulator
Ba1Hf1N2_ICSD_184055	insulator	insulator
Ba1Hf1O8P2_ICSD_245690	insulator	insulator
Ba1Hf1O9Si3_ICSD_183835	insulator	insulator
Ba1Hg11_ICSD_107486	metal	metal
Ba1Hg1O2_ICSD_68616	insulator	insulator
Ba1Hg1O2_ICSD_83411	insulator	insulator
Ba1Hg1O5Ru1_ICSD_81070	insulator	insulator
Ba1Hg1S2_ICSD_32648	insulator	insulator
Ba1Hg1S4Sn1_ICSD_10456	insulator	insulator
Ba1Hg1Sn1_ICSD_106311	metal	metal
Ba1Hg1_ICSD_58654	metal	metal
Ba1Hg2Tl2_ICSD_260391	metal	metal
Ba1Hg2_ICSD_58655	metal	metal
Ba1Hg6_ICSD_424857	metal	metal
Ba1Ho2Ni1O5_ICSD_67930	insulator	insulator
Ba1Ho2O4_ICSD_154812	insulator	insulator
Ba1Ho2O5Pd1_ICSD_404497	insulator	insulator
Ba1Ho2S4_ICSD_615930	insulator	insulator
Ba1Ho2Te4_ICSD_90336	insulator	insulator
Ba1I2O6_ICSD_23276	insulator	insulator
Ba1I2_ICSD_15707	insulator	insulator
Ba1I2_ICSD_36210	insulator	insulator
Ba1I2_ICSD_79893	insulator	insulator
Ba1I6U1_ICSD_78772	insulator	insulator
Ba1In1_ICSD_414228	metal	metal
Ba1In2Ir1_ICSD_414434	metal	metal
Ba1In2La2O7_ICSD_95984	insulator	insulator
Ba1In2Nd2O7_ICSD_168283	insulator	insulator
Ba1In2O14P4_ICSD_180003	insulator	insulator
Ba1In2P2_ICSD_260564	insulator	insulator
Ba1In2Pt1_ICSD_411228	metal	metal
Ba1In2Rh1_ICSD_411232	metal	metal
Ba1In2Te4_ICSD_41168	insulator	insulator
Ba1In2_ICSD_58657	metal	metal
Ba1In4Ir1_ICSD_173616	metal	metal
Ba1In4_ICSD_414230	metal	metal
Ba1Ir1O3_ICSD_173848	metal	metal
Ba1Ir1O3_ICSD_173889	metal	metal
Ba1Ir1O3_ICSD_260303	metal	metal
Ba1Ir1P1_ICSD_73529	metal	metal

Supplementary Table 139. Five-fold cross validated predictions for the metal/insulator classification (131/598).

system	calculated	predicted
Ba1Ir2P2_ICSD_95757	metal	metal
Ba1Ir2Si2_ICSD_50159	metal	metal
Ba1K1Nb1S4_ICSD_415335	insulator	insulator
Ba1K1O4P1_ICSD_202430	insulator	insulator
Ba1K1O4V1_ICSD_418461	insulator	insulator
Ba1K1P1S4_ICSD_414639	insulator	insulator
Ba1K1P1Se4_ICSD_414637	insulator	insulator
Ba1K4O9Si3_ICSD_246254	insulator	insulator
Ba1K4S8V2_ICSD_240378	insulator	insulator
Ba1La1Mn2O6_ICSD_150703	metal	metal
Ba1La1Sb2Se6_ICSD_421269	insulator	insulator
Ba1La2Mn1S5_ICSD_90640	insulator	insulator
Ba1La2O14Te5_ICSD_417616	insulator	insulator
Ba1La2O5Pt1_ICSD_68794	insulator	insulator
Ba1La2O5Zn1_ICSD_172768	insulator	insulator
Ba1La2O7Sc2_ICSD_167599	insulator	insulator
Ba1La2S5Zn1_ICSD_93711	insulator	insulator
Ba1Li1P1_ICSD_416890	insulator	insulator
Ba1Li1P1_ICSD_56444	insulator	insulator
Ba1Li1Sb1_ICSD_280574	insulator	metal
Ba1Li1Si1_ICSD_42890	metal	metal
Ba1Li2Mg1O8P2_ICSD_236294	insulator	insulator
Ba1Li2Mg2Si2_ICSD_409575	metal	metal
Ba1Li2O4Si1_ICSD_180289	insulator	insulator
Ba1Li2Si1_ICSD_15642	metal	metal
Ba1Li4_ICSD_615944	metal	metal
Ba1Lu2S4_ICSD_422891	insulator	insulator
Ba1Mg0.333Nb0.667O3_ICSD_95497	insulator	insulator
Ba1Mg0.333O3Ta0.667_ICSD_95495	insulator	insulator
Ba1Mg1Na2O8P2_ICSD_262716	insulator	insulator
Ba1Mg1O4Si1_ICSD_73776	insulator	insulator
Ba1Mg1O7Te2_ICSD_262408	insulator	insulator
Ba1Mg1Pb1_ICSD_615958	metal	metal
Ba1Mg1Si1_ICSD_42459	metal	metal
Ba1Mg1Sn1_ICSD_615965	metal	metal
Ba1Mg2O8V2_ICSD_20429	insulator	insulator
Ba1Mg2P2_ICSD_30914	insulator	insulator
Ba1Mg2Sb2_ICSD_100047	insulator	insulator
Ba1Mg2Si2_ICSD_25311	metal	metal
Ba1Mg2_ICSD_170241	metal	metal
Ba1Mg4Si3_ICSD_165630	metal	metal
Ba1Mn1Nd2S5_ICSD_93715	metal	insulator
Ba1Mn1O3_ICSD_10250	insulator	insulator
Ba1Mn1O3_ICSD_10331	insulator	insulator
Ba1Mn1O3_ICSD_23874	insulator	insulator
Ba1Mn1O3_ICSD_66822	insulator	insulator
Ba1Mn1O3_ICSD_89995	insulator	insulator
Ba1Mn1O4Rb1_ICSD_80640	insulator	insulator
Ba1Mn1O7P2_ICSD_78658	insulator	insulator
Ba1Mn1Pr2S5_ICSD_91229	insulator	insulator

Supplementary Table 140. Five-fold cross validated predictions for the metal/insulator classification (132/598).

system	calculated	predicted
Ba1Mn1S2_ICSD_31453	insulator	insulator
Ba1Mn28_ICSD_615966	metal	metal
Ba1Mn2Nd1O5_ICSD_158889	metal	metal
Ba1Mn2Nd1O6_ICSD_150705	metal	metal
Ba1Mn2Nd2O7_ICSD_154537	metal	metal
Ba1Mn2O14Si4Sr2_ICSD_39593	insulator	insulator
Ba1Mn2O3_ICSD_10038	insulator	metal
Ba1Mn2O5Pr1_ICSD_158885	metal	metal
Ba1Mn2O5Y1_ICSD_86839	metal	metal
Ba1Mn2O5Y1_ICSD_88950	metal	metal
Ba1Mn2O6Pr1_ICSD_158886	metal	metal
Ba1Mn2O6Tb1_ICSD_154009	metal	metal
Ba1Mn2O6Tb1_ICSD_154010	metal	metal
Ba1Mn2O6Y1_ICSD_154011	metal	metal
Ba1Mn2O6Y1_ICSD_99700	metal	metal
Ba1Mn2O6Y1_ICSD_99701	metal	metal
Ba1Mn2O7Tb2_ICSD_99245	metal	insulator
Ba1Mn2O8_ICSD_23443	insulator	insulator
Ba1Mn2O8_ICSD_24128	insulator	insulator
Ba1Mn2P2_ICSD_10469	metal	metal
Ba1Mn2Sb2_ICSD_32019	metal	metal
Ba1Mn2Sb2_ICSD_419531	metal	metal
Ba1Mn2Sn2_ICSD_405	metal	metal
Ba1Mn3O38Ti18_ICSD_81584	metal	insulator
Ba1Mn3O6_ICSD_93226	metal	metal
Ba1Mo1O3_ICSD_43799	metal	metal
Ba1Mo1O4_ICSD_16166	insulator	insulator
Ba1Mo1O6Se1_ICSD_82255	insulator	insulator
Ba1Mo1O8P2_ICSD_79507	insulator	insulator
Ba1Mo2O11Se2_ICSD_82256	insulator	insulator
Ba1Mo2O12P2_ICSD_62833	insulator	insulator
Ba1Mo2O12P3_ICSD_68560	metal	insulator
Ba1Mo2O14P3_ICSD_153067	insulator	insulator
Ba1Mo2O14P4_ICSD_72339	insulator	insulator
Ba1Mo2O9Te1_ICSD_159460	insulator	insulator
Ba1Mo3Se3_ICSD_603615	metal	metal
Ba1Mo3Te3_ICSD_603674	metal	metal
Ba1Mo4O16P2_ICSD_75147	insulator	insulator
Ba1Mo6O10_ICSD_202739	metal	metal
Ba1Mo6S8_ICSD_65775	insulator	metal
Ba1Mo6S8_ICSD_85489	metal	metal
Ba1N10Si7_ICSD_405772	insulator	insulator
Ba1N12P6Sr2_ICSD_415716	insulator	insulator
Ba1N1Ni1_ICSD_40166	metal	insulator
Ba1N2O2Si2_ICSD_419450	insulator	insulator
Ba1N2O4_ICSD_29510	insulator	insulator
Ba1N2O6_ICSD_56087	insulator	insulator
Ba1N2Si1_ICSD_170265	insulator	insulator
Ba1N2Zr1_ICSD_74904	insulator	insulator
Ba1N2_ICSD_423723	metal	insulator

Supplementary Table 141. Five-fold cross validated predictions for the metal/insulator classification (133/598).

system	calculated	predicted
Ba1N4P2_ICSD_414350	insulator	insulator
Ba1N6_ICSD_412253	insulator	insulator
Ba1N7Si4Y1_ICSD_98276	insulator	insulator
Ba1N7Si4Yb1_ICSD_160649	insulator	insulator
Ba1N8O1Si6_ICSD_415272	insulator	insulator
Ba1N8Si6_ICSD_417444	insulator	insulator
Ba1Na1O7Sc1Si2_ICSD_166998	insulator	insulator
Ba1Na1P1_ICSD_402227	insulator	insulator
Ba1Na2O6Si2_ICSD_10217	insulator	insulator
Ba1Na2_ICSD_106313	metal	metal
Ba1Nb0.67O3Sr0.33_ICSD_186191	insulator	insulator
Ba1Nb1O3_ICSD_50275	metal	metal
Ba1Nb1S3_ICSD_659538	metal	metal
Ba1Nb2O11V2_ICSD_165097	insulator	insulator
Ba1Nb2O6_ICSD_39272	insulator	insulator
Ba1Nb2O6_ICSD_39320	insulator	insulator
Ba1Nb4O6_ICSD_42006	metal	metal
Ba1Nb7O9_ICSD_66344	metal	metal
Ba1Nb8O14_ICSD_79976	insulator	insulator
Ba1Nd2Ni1O5_ICSD_62627	metal	metal
Ba1Nd2O5Pd1_ICSD_62609	insulator	insulator
Ba1Nd2O5Pt1_ICSD_47178	insulator	insulator
Ba1Nd2O5Zn1_ICSD_68808	insulator	insulator
Ba1Nd2S4_ICSD_615994	insulator	insulator
Ba1Nd2S5Zn1_ICSD_93714	insulator	insulator
Ba1Ni10P6_ICSD_67899	metal	metal
Ba1Ni1O2_ICSD_15760	insulator	insulator
Ba1Ni1O3_ICSD_15761	insulator	insulator
Ba1Ni1O3_ICSD_175	insulator	insulator
Ba1Ni1O5Pr2_ICSD_75035	metal	metal
Ba1Ni1O5Tb2_ICSD_66078	insulator	insulator
Ba1Ni1O5Y2_ICSD_71327	insulator	insulator
Ba1Ni1O5Yb2_ICSD_67832	metal	metal
Ba1Ni1O5Yb2_ICSD_67834	metal	metal
Ba1Ni1O5Yb2_ICSD_72632	metal	metal
Ba1Ni1S2_ICSD_15289	metal	metal
Ba1Ni1S2_ICSD_246945	metal	metal
Ba1Ni1Sn3_ICSD_58662	metal	metal
Ba1Ni2O8P2_ICSD_280167	insulator	insulator
Ba1Ni2O8V2_ICSD_201621	insulator	insulator
Ba1Ni2P2_ICSD_85408	metal	metal
Ba1Ni2P4_ICSD_79104	metal	metal
Ba1Ni2P4_ICSD_79105	metal	metal
Ba1Ni2Si2_ICSD_14110	metal	metal
Ba1Ni4O8_ICSD_20898	insulator	insulator
Ba1Ni9P5_ICSD_33918	metal	metal
Ba1O11V6_ICSD_260347	metal	metal
Ba1O11V6_ICSD_260348	metal	metal
Ba1O11V6_ICSD_416456	metal	metal
Ba1O13Ti6_ICSD_2922	insulator	insulator

Supplementary Table 142. Five-fold cross validated predictions for the metal/insulator classification (134/598).

system	calculated	predicted
Ba1O14P4Ti2_ICSD_69103	insulator	insulator
Ba1O14P4V2_ICSD_66538	insulator	insulator
Ba1O14Sr4U3_ICSD_50812	insulator	insulator
Ba1O18V13_ICSD_97948	metal	metal
Ba1O1S1Zn1_ICSD_171239	insulator	insulator
Ba1O1_ICSD_15301	insulator	insulator
Ba1O1_ICSD_173921	insulator	insulator
Ba1O1_ICSD_58663	insulator	insulator
Ba1O2.8V1_ICSD_78164	insulator	insulator
Ba1O2Zn1_ICSD_25812	insulator	insulator
Ba1O2_ICSD_180398	insulator	insulator
Ba1O2_ICSD_80750	insulator	insulator
Ba1O3Pa1_ICSD_61315	metal	metal
Ba1O3Pb1_ICSD_245603	metal	insulator
Ba1O3Pb1_ICSD_51659	metal	metal
Ba1O3Pb1_ICSD_72269	metal	metal
Ba1O3Pb1_ICSD_94312	insulator	metal
Ba1O3Pb1_ICSD_94313	insulator	insulator
Ba1O3Pr1_ICSD_163752	insulator	insulator
Ba1O3Pr1_ICSD_163753	insulator	insulator
Ba1O3Pr1_ICSD_52336	metal	insulator
Ba1O3Pu1_ICSD_43768	metal	metal
Ba1O3Rh1_ICSD_15520	metal	insulator
Ba1O3Ru1_ICSD_10253	metal	metal
Ba1O3Ru1_ICSD_160287	metal	metal
Ba1O3Ru1_ICSD_246019	metal	metal
Ba1O3Ru1_ICSD_84652	metal	metal
Ba1O3Se1_ICSD_54156	insulator	insulator
Ba1O3Si1_ICSD_156705	insulator	insulator
Ba1O3Si1_ICSD_156706	insulator	insulator
Ba1O3Si1_ICSD_6245	insulator	insulator
Ba1O3Sn1_ICSD_184338	metal	insulator
Ba1O3Tb1_ICSD_2752	metal	insulator
Ba1O3Tb1_ICSD_86737	metal	insulator
Ba1O3Tb1_ICSD_99477	metal	insulator
Ba1O3Tb1_ICSD_99478	metal	metal
Ba1O3Tc1_ICSD_109077	insulator	insulator
Ba1O3Te1_ICSD_10107	insulator	insulator
Ba1O3Te1_ICSD_4320	insulator	insulator
Ba1O3Th1_ICSD_29110	insulator	insulator
Ba1O3Ti1_ICSD_100802	insulator	insulator
Ba1O3Ti1_ICSD_109327	metal	insulator
Ba1O3Ti1_ICSD_154346	insulator	insulator
Ba1O3Ti1_ICSD_186461	insulator	insulator
Ba1O3Ti1_ICSD_31155	insulator	insulator
Ba1O3Ti1_ICSD_34619	insulator	insulator
Ba1O3Ti1_ICSD_55221	insulator	insulator
Ba1O3Ti1_ICSD_73628	insulator	insulator
Ba1O3Ti1_ICSD_73640	insulator	insulator
Ba1O3V1_ICSD_78168	metal	insulator

Supplementary Table 143. Five-fold cross validated predictions for the metal/insulator classification (135/598).

system	calculated	predicted
Ba1O3Zr1_ICSD_90049	insulator	insulator
Ba1O4P1Rb1_ICSD_72001	insulator	insulator
Ba1O4S1_ICSD_186427	insulator	insulator
Ba1O4S1_ICSD_23743	insulator	insulator
Ba1O4S1_ICSD_33732	insulator	insulator
Ba1O4Se1_ICSD_409810	insulator	insulator
Ba1O4Si1Zn1_ICSD_73777	insulator	insulator
Ba1O4Tb2_ICSD_78661	insulator	insulator
Ba1O4U1_ICSD_36239	insulator	insulator
Ba1O4W1_ICSD_155512	insulator	insulator
Ba1O4W1_ICSD_155516	insulator	insulator
Ba1O4Y2_ICSD_89640	insulator	insulator
Ba1O5Pd1Pr2_ICSD_202172	insulator	insulator
Ba1O5Pd1Tb2_ICSD_202173	insulator	insulator
Ba1O5Pd1Y2_ICSD_202819	insulator	insulator
Ba1O5Se2_ICSD_54157	insulator	insulator
Ba1O5Si2_ICSD_10162	insulator	insulator
Ba1O5Tb2Zn1_ICSD_69721	insulator	insulator
Ba1O5Ti2_ICSD_157775	insulator	insulator
Ba1O5Ti2_ICSD_162369	insulator	insulator
Ba1O5Ti2_ICSD_2356	insulator	insulator
Ba1O5Ti2_ICSD_281548	insulator	insulator
Ba1O6P1V1_ICSD_66699	insulator	insulator
Ba1O6P2_ICSD_15714	insulator	insulator
Ba1O6Sb2_ICSD_74541	insulator	insulator
Ba1O6Te2_ICSD_8017	insulator	insulator
Ba1O6Ti1U1_ICSD_156646	insulator	insulator
Ba1O6V2_ICSD_80938	insulator	insulator
Ba1O7P2Zn1_ICSD_39396	insulator	insulator
Ba1O7Si2V1_ICSD_78029	insulator	insulator
Ba1O7Te2Zn1_ICSD_262409	insulator	insulator
Ba1O7U2_ICSD_22206	insulator	insulator
Ba1O8P2Sn1_ICSD_420129	insulator	insulator
Ba1O8P2Th1_ICSD_421965	insulator	insulator
Ba1O8P2Zn2_ICSD_300159	insulator	insulator
Ba1O8P2Zr1_ICSD_153124	insulator	insulator
Ba1O8P2Zr1_ICSD_173842	insulator	insulator
Ba1O8Si2U1_ICSD_79817	insulator	insulator
Ba1O8V3_ICSD_80299	insulator	insulator
Ba1O8V3_ICSD_82063	insulator	insulator
Ba1O9Si3Sn1_ICSD_10385	insulator	insulator
Ba1O9Si3Ti1_ICSD_290229	insulator	insulator
Ba1O9Si4_ICSD_80067	insulator	insulator
Ba1O9Te1W2_ICSD_281502	insulator	insulator
Ba1O9Ti4_ICSD_49575	insulator	insulator
Ba1O9V4_ICSD_173002	insulator	insulator
Ba1Os2P2_ICSD_602106	metal	metal
Ba1Os4Sb12_ICSD_658736	metal	metal
Ba1P10_ICSD_35295	insulator	insulator
Ba1P1Pt1_ICSD_59191	metal	metal

Supplementary Table 144. Five-fold cross validated predictions for the metal/insulator classification (136/598).

system	calculated	predicted
Ba1P2Pd2_ICSD_36375	metal	metal
Ba1P2Rh2_ICSD_50188	metal	metal
Ba1P2Ru2_ICSD_616013	metal	metal
Ba1P2Zn2_ICSD_12145	metal	insulator
Ba1P3Pt2_ICSD_62520	insulator	insulator
Ba1P3_ICSD_23618	insulator	metal
Ba1P4Pd2_ICSD_75020	metal	metal
Ba1P4Te2_ICSD_412643	insulator	insulator
Ba1P8_ICSD_96544	insulator	insulator
Ba1Pb1Zn1_ICSD_106315	metal	metal
Ba1Pb1_ICSD_616019	metal	metal
Ba1Pb3_ICSD_419973	metal	metal
Ba1Pd1S2_ICSD_63588	insulator	insulator
Ba1Pd1Sb2_ICSD_405111	metal	metal
Ba1Pd1Si1_ICSD_616034	metal	metal
Ba1Pd1Si3_ICSD_174266	metal	metal
Ba1Pd1Sn3_ICSD_58673	metal	metal
Ba1Pd1_ICSD_616028	metal	metal
Ba1Pd2S4_ICSD_79930	insulator	insulator
Ba1Pd2Sb2_ICSD_604350	metal	metal
Ba1Pd2Sb2_ICSD_61197	metal	metal
Ba1Pd2_ICSD_616027	metal	metal
Ba1Pd5_ICSD_58672	metal	metal
Ba1Pr2S5Zn1_ICSD_93713	insulator	insulator
Ba1Pt1Sb1_ICSD_59186	metal	metal
Ba1Pt1Si1_ICSD_604438	metal	metal
Ba1Pt1Si3_ICSD_174267	metal	metal
Ba1Pt1Sn3_ICSD_58677	metal	metal
Ba1Pt1_ICSD_55492	metal	metal
Ba1Pt2S3_ICSD_201146	insulator	insulator
Ba1Pt2S3_ICSD_55077	insulator	insulator
Ba1Pt2_ICSD_58675	metal	metal
Ba1Pt5_ICSD_58676	metal	metal
Ba1Rb1S4Ta1_ICSD_421845	insulator	insulator
Ba1Rh2Si2_ICSD_50158	metal	metal
Ba1Rh2_ICSD_108149	metal	metal
Ba1Ru2Sb2_ICSD_188980	metal	metal
Ba1Ru4Sb12_ICSD_42963	metal	metal
Ba1S1_ICSD_52690	insulator	insulator
Ba1S2Sn1_ICSD_2587	insulator	insulator
Ba1S2_ICSD_2004	insulator	insulator
Ba1S3Sn2_ICSD_26333	insulator	insulator
Ba1S3Ta1_ICSD_659541	metal	metal
Ba1S3Te1_ICSD_8	insulator	insulator
Ba1S3Ti1_ICSD_616083	metal	metal
Ba1S3U1_ICSD_23289	insulator	insulator
Ba1S3V1_ICSD_154181	metal	metal
Ba1S3V1_ICSD_154183	insulator	insulator
Ba1S3V1_ICSD_154184	insulator	insulator
Ba1S3V1_ICSD_15537	metal	metal

Supplementary Table 145. Five-fold cross validated predictions for the metal/insulator classification (137/598).

system	calculated	predicted
Ba1S3V1_ICSD_616099	insulator	metal
Ba1S3V1_ICSD_63230	metal	metal
Ba1S3Zr1_ICSD_165977	insulator	insulator
Ba1S3_ICSD_23637	insulator	insulator
Ba1S3_ICSD_26765	insulator	insulator
Ba1S4Sb2_ICSD_38372	insulator	insulator
Ba1S4Tb2_ICSD_616078	insulator	insulator
Ba1S4Y2_ICSD_616101	insulator	insulator
Ba1S4Yb2_ICSD_616102	metal	metal
Ba1Sb1Te3_ICSD_10506	insulator	insulator
Ba1Sb2Zn2_ICSD_32020	metal	metal
Ba1Sb2_ICSD_409517	metal	metal
Ba1Sb3_ICSD_49000	metal	metal
Ba1Sc2Te4_ICSD_416326	insulator	insulator
Ba1Se1_ICSD_43655	insulator	insulator
Ba1Se1_ICSD_52695	insulator	insulator
Ba1Se2_ICSD_16358	insulator	insulator
Ba1Se3Ta1_ICSD_616129	metal	metal
Ba1Se3Ti1_ICSD_616131	metal	metal
Ba1Se3V1_ICSD_10486	metal	metal
Ba1Se3Zr1_ICSD_616136	metal	metal
Ba1Se3_ICSD_16359	insulator	insulator
Ba1Se4Sm2_ICSD_616127	metal	insulator
Ba1Se4Y2_ICSD_422980	insulator	insulator
Ba1Se4Yb2_ICSD_616135	insulator	metal
Ba1Si1Zn1_ICSD_106316	metal	metal
Ba1Si1_ICSD_659548	metal	metal
Ba1Si2Zn2_ICSD_174269	metal	metal
Ba1Si2_ICSD_1237	metal	metal
Ba1Si2_ICSD_168407	insulator	metal
Ba1Si2_ICSD_168409	metal	metal
Ba1Si2_ICSD_20244	metal	metal
Ba1Si2_ICSD_602228	metal	metal
Ba1Si6_ICSD_245295	metal	metal
Ba1Sn1Zn1_ICSD_106317	metal	metal
Ba1Sn1_ICSD_58678	metal	metal
Ba1Sn2_ICSD_409958	metal	metal
Ba1Sn3_ICSD_419206	metal	metal
Ba1Sn5_ICSD_107305	metal	metal
Ba1Tb2Te4_ICSD_90333	insulator	insulator
Ba1Te1_ICSD_43656	insulator	insulator
Ba1Te1_ICSD_616164	insulator	insulator
Ba1Te2_ICSD_80280	insulator	insulator
Ba1Te3_ICSD_36366	insulator	insulator
Ba1Te4Y2_ICSD_90335	insulator	insulator
Ba1Tl2_ICSD_58681	metal	metal
Ba1Tl4_ICSD_261206	metal	metal
Ba1Zn13_ICSD_616177	metal	metal
Ba1Zn1_ICSD_58682	metal	metal
Ba1Zn2_ICSD_58683	metal	metal

Supplementary Table 146. Five-fold cross validated predictions for the metal/insulator classification (138/598).

system	calculated	predicted
Ba1Zn5_ICSD_418610	metal	metal
Ba1_ICSD_108091	metal	metal
Ba1_ICSD_109029	metal	metal
Ba1_ICSD_109030	metal	metal
Ba1_ICSD_52679	metal	metal
Ba1_ICSD_52680	metal	metal
Ba1_ICSD_89237	metal	metal
Ba21Ge2O5_ICSD_491557	metal	metal
Ba21O5Si2_ICSD_491556	metal	metal
Ba24Si100_ICSD_90703	metal	insulator
Ba2Bi1Dy1O6_ICSD_68612	insulator	insulator
Ba2Bi1Fe1S5_ICSD_261419	insulator	insulator
Ba2Bi1Ir1O6_ICSD_174289	metal	insulator
Ba2Bi1Ir1O6_ICSD_174290	insulator	metal
Ba2Bi1O6Sb1_ICSD_172761	insulator	insulator
Ba2Bi1O6Sb1_ICSD_172762	insulator	insulator
Ba2Bi1O6Ta1_ICSD_153120	insulator	insulator
Ba2Bi1O6Ta1_ICSD_154150	insulator	insulator
Ba2Bi1O6Y1_ICSD_65555	insulator	insulator
Ba2Bi1O6Yb1_ICSD_80902	insulator	metal
Ba2Bi1_ICSD_2141	metal	metal
Ba2Bi2Mn2O1_ICSD_16361	metal	metal
Ba2Bi2O6_ICSD_28164	insulator	insulator
Ba2Bi2Zn1_ICSD_421424	insulator	metal
Ba2Bi3_ICSD_170218	metal	metal
Ba2Bi4Cd3_ICSD_58636	metal	metal
Ba2Br1Cu1O2_ICSD_67395	insulator	insulator
Ba2Br1H3_ICSD_415127	insulator	insulator
Ba2Br1In1O3_ICSD_81878	insulator	insulator
Ba2Br1N1_ICSD_262056	insulator	insulator
Ba2Br2Cu3O4_ICSD_36128	metal	metal
Ba2Br2F10Pb4_ICSD_411087	insulator	insulator
Ba2Br2O1_ICSD_423479	insulator	insulator
Ba2Br5Cs1_ICSD_402191	insulator	insulator
Ba2C2Co1F2O6_ICSD_95721	insulator	insulator
Ba2C2F3O6Y1_ICSD_72733	insulator	insulator
Ba2C3Ce1F1O9_ICSD_72446	insulator	insulator
Ba2C3Cs2O9_ICSD_73170	insulator	insulator
Ba2C3F1La1O9_ICSD_250059	insulator	insulator
Ba2C4H12N2O10S2_ICSD_59807	insulator	insulator
Ba2Ca1Cr2Cu1F14_ICSD_419545	insulator	insulator
Ba2Ca1Cu1F14Fe2_ICSD_202761	insulator	insulator
Ba2Ca1Cu2Hg1O6_ICSD_75728	metal	metal
Ba2Ca1Cu2O7Tl1_ICSD_67128	metal	metal
Ba2Ca1Ir1O6_ICSD_74029	insulator	metal
Ba2Ca1Mo1O6_ICSD_45317	insulator	insulator
Ba2Ca1N4W1_ICSD_409472	insulator	insulator
Ba2Ca1O6Os1_ICSD_171989	metal	metal
Ba2Ca1O6Pd3_ICSD_73082	insulator	insulator
Ba2Ca1O6Re1_ICSD_109258	metal	metal

Supplementary Table 147. Five-fold cross validated predictions for the metal/insulator classification (139/598).

system	calculated	predicted
Ba2Ca1O6Re1_ICSD_171987	metal	metal
Ba2Ca1O6Te1_ICSD_246112	insulator	insulator
Ba2Ca1O6W1_ICSD_246111	insulator	insulator
Ba2Ca1O6W1_ICSD_246117	insulator	insulator
Ba2Ca2Cu3Hg1O8_ICSD_75735	metal	metal
Ba2Ca2Sn6_ICSD_249515	metal	metal
Ba2Cd1O6Re1_ICSD_109257	metal	metal
Ba2Cd1O6U1_ICSD_167503	insulator	insulator
Ba2Cd1S3_ICSD_66654	insulator	insulator
Ba2Cd1Se3_ICSD_66653	insulator	insulator
Ba2Cd1Te3_ICSD_88849	insulator	insulator
Ba2Cd1_ICSD_30083	metal	metal
Ba2Cd2K1Sb3_ICSD_420620	insulator	metal
Ba2Cd2Sb3_ICSD_420834	metal	metal
Ba2Ce1Cl7_ICSD_000	insulator	insulator
Ba2Ce1O6Pt1_ICSD_66408	insulator	insulator
Ba2Cl1Co2F7_ICSD_79479	insulator	insulator
Ba2Cl1Co4O7_ICSD_245991	insulator	insulator
Ba2Cl1Cu1O2_ICSD_1038	insulator	insulator
Ba2Cl1F3_ICSD_183926	insulator	insulator
Ba2Cl1F7Mn1Ni1_ICSD_79480	insulator	insulator
Ba2Cl1F7Ni2_ICSD_84782	insulator	insulator
Ba2Cl1F7Zn2_ICSD_79478	insulator	insulator
Ba2Cl1H3_ICSD_416893	insulator	insulator
Ba2Cl1In1O3_ICSD_81877	insulator	insulator
Ba2Cl1Mn1O7Si2_ICSD_281430	insulator	insulator
Ba2Cl1N1_ICSD_262135	insulator	insulator
Ba2Cl1P1_ICSD_28134	insulator	insulator
Ba2Cl1P7_ICSD_24398	insulator	insulator
Ba2Cl2Co1O6Se2_ICSD_280965	insulator	insulator
Ba2Cl2Cu3O4_ICSD_163501	metal	metal
Ba2Cl7Er1_ICSD_82494	insulator	insulator
Ba2Cl7Gd1_ICSD_82495	insulator	insulator
Ba2Cl7La1_ICSD_000	insulator	insulator
Ba2Cl7Sc1_ICSD_408056	insulator	insulator
Ba2Cl7Sc1_ICSD_56832	insulator	insulator
Ba2Cl7Y1_ICSD_000	insulator	insulator
Ba2Cl7Yb1_ICSD_000	metal	insulator
Ba2Co1Cu2O2S2_ICSD_261352	metal	metal
Ba2Co1F6_ICSD_21057	insulator	insulator
Ba2Co1Ge2O7_ICSD_290483	insulator	insulator
Ba2Co1Mo1O6_ICSD_184910	metal	metal
Ba2Co1O4_ICSD_16234	insulator	insulator
Ba2Co1O4_ICSD_92321	insulator	insulator
Ba2Co1O6Re1_ICSD_109254	insulator	metal
Ba2Co1O6U1_ICSD_245141	metal	insulator
Ba2Co1O6W1_ICSD_27425	metal	insulator
Ba2Co1O7Si2_ICSD_281293	insulator	insulator
Ba2Co1O7Si2_ICSD_81472	insulator	insulator
Ba2Co4O11Tb2_ICSD_94046	metal	metal

Supplementary Table 148. Five-fold cross validated predictions for the metal/insulator classification (140/598).

system	calculated	predicted
Ba2Co9O14_ICSD_161771	metal	metal
Ba2Cr1Mo1O6_ICSD_184907	metal	metal
Ba2Cr1O4_ICSD_73892	insulator	insulator
Ba2Cr1O6Ta1_ICSD_74410	insulator	insulator
Ba2Cr7O14_ICSD_2766	insulator	metal
Ba2Cs1Nb3O10_ICSD_93676	insulator	insulator
Ba2Cu1F6_ICSD_100028	insulator	insulator
Ba2Cu1F6_ICSD_21055	metal	insulator
Ba2Cu1Ge2O7_ICSD_77132	insulator	insulator
Ba2Cu1Hg1O4_ICSD_75723	metal	metal
Ba2Cu1I1O2_ICSD_67394	insulator	insulator
Ba2Cu1O3_ICSD_68217	metal	metal
Ba2Cu1O5Tl1_ICSD_66583	metal	metal
Ba2Cu1O6Te1_ICSD_88703	insulator	insulator
Ba2Cu1O6U1_ICSD_157853	insulator	insulator
Ba2Cu1O6U1_ICSD_169534	insulator	insulator
Ba2Cu1O6W1_ICSD_33569	insulator	insulator
Ba2Cu1O6W1_ICSD_72813	insulator	insulator
Ba2Cu1O7Si2_ICSD_20905	insulator	insulator
Ba2Cu1O7Si2_ICSD_97762	insulator	insulator
Ba2Cu1O8P2_ICSD_81457	insulator	insulator
Ba2Cu2Eu2O11Ti2_ICSD_66812	metal	metal
Ba2Cu2La1O8Ta1_ICSD_64639	metal	metal
Ba2Cu2Nb1Nd1O8_ICSD_44255	metal	metal
Ba2Cu2Nd2O11Ti2_ICSD_75342	metal	metal
Ba2Cu2O11Tb2Ti2_ICSD_79534	metal	metal
Ba2Cu2O12Si4_ICSD_71535	insulator	insulator
Ba2Cu2O7Tl1Y1_ICSD_74163	metal	metal
Ba2Cu2S5U1_ICSD_418464	insulator	insulator
Ba2Cu3Dy1O7_ICSD_81173	metal	metal
Ba2Cu3Er1O7.02_ICSD_63405	metal	metal
Ba2Cu3Eu1O7_ICSD_81171	metal	metal
Ba2Cu3Gd1O7_ICSD_56514	metal	metal
Ba2Cu3Ho1O6_ICSD_68047	metal	metal
Ba2Cu3Ho1O7_ICSD_68044	metal	metal
Ba2Cu3Ho1O7_ICSD_81174	metal	metal
Ba2Cu3La1O7_ICSD_81167	metal	metal
Ba2Cu3La1O8_ICSD_85291	metal	metal
Ba2Cu3Li1O6_ICSD_401239	insulator	insulator
Ba2Cu3Lu1O6_ICSD_98113	metal	metal
Ba2Cu3Na1O6_ICSD_72328	insulator	insulator
Ba2Cu3Nd1O6_ICSD_83074	metal	metal
Ba2Cu3Nd1O7_ICSD_86960	metal	metal
Ba2Cu3O6Y1_ICSD_67016	metal	metal
Ba2Cu3O7Pr1_ICSD_41452	metal	metal
Ba2Cu3O7Y1_ICSD_86958	metal	metal
Ba2Cu3O8Pr1_ICSD_86043	metal	metal
Ba2Cu3P4_ICSD_69494	metal	insulator
Ba2Cu3S6V1_ICSD_83969	insulator	insulator
Ba2Cu4Er1O8_ICSD_75687	metal	metal

Supplementary Table 149. Five-fold cross validated predictions for the metal/insulator classification (141/598).

system	calculated	predicted
Ba2Cu4Ho1O8_ICSD_75688	metal	metal
Ba2Cu4O8Y1_ICSD_74259	metal	metal
Ba2Cu5F14_ICSD_202138	insulator	insulator
Ba2Dy1Ga1Se5_ICSD_262886	insulator	insulator
Ba2Dy1Nb1O6_ICSD_109156	insulator	insulator
Ba2Dy1O6Re1_ICSD_25396	metal	metal
Ba2Dy1O6Sb1_ICSD_150863	insulator	insulator
Ba2Er1Ga1Se5_ICSD_262887	insulator	insulator
Ba2Er1Nb1O6_ICSD_245456	insulator	insulator
Ba2Er1O6Ru1_ICSD_59743	insulator	insulator
Ba2Er1O6Sb1_ICSD_245459	insulator	insulator
Ba2Eu1Nb1O6_ICSD_109154	metal	metal
Ba2Eu1Nb1O6_ICSD_245798	metal	metal
Ba2Eu1O6Re1_ICSD_25394	metal	metal
Ba2Eu1O6Sb1_ICSD_38330	metal	metal
Ba2Eu1O6Ta1_ICSD_160171	metal	metal
Ba2Eu3Si7_ICSD_83676	metal	metal
Ba2F10I2Pb4_ICSD_411088	insulator	insulator
Ba2F10Mg3_ICSD_50228	insulator	insulator
Ba2F10Ni3_ICSD_23364	insulator	insulator
Ba2F18Ni7_ICSD_65610	insulator	insulator
Ba2F18Zn7_ICSD_40925	insulator	insulator
Ba2F1In1O3_ICSD_79261	insulator	insulator
Ba2F1N1_ICSD_262049	insulator	insulator
Ba2F2Fe2O1S2_ICSD_249689	insulator	insulator
Ba2F2Fe2O1Se2_ICSD_249687	insulator	insulator
Ba2F2Mn2O1Se2_ICSD_183149	insulator	insulator
Ba2F2O2Pd1_ICSD_98141	metal	insulator
Ba2F2S3Sn1_ICSD_171343	insulator	insulator
Ba2F2Se3Sn1_ICSD_171344	insulator	insulator
Ba2F4O3W1_ICSD_33271	insulator	insulator
Ba2F6Ni1_ICSD_21056	insulator	insulator
Ba2F6O1Ti1_ICSD_72150	insulator	insulator
Ba2F6Pd1_ICSD_88802	insulator	insulator
Ba2F6Zn1_ICSD_21054	insulator	insulator
Ba2F8Zr1_ICSD_85720	insulator	insulator
Ba2Fe1Ge2O7_ICSD_22358	insulator	insulator
Ba2Fe1Mo1O6_ICSD_246543	insulator	metal
Ba2Fe1Mo1O6_ICSD_246546	metal	metal
Ba2Fe1O6Re1_ICSD_109252	metal	metal
Ba2Fe1O6Re1_ICSD_155174	metal	metal
Ba2Fe1O6Re1_ICSD_155175	metal	metal
Ba2Fe1O6U1_ICSD_27018	insulator	metal
Ba2Fe1O6W1_ICSD_95518	insulator	metal
Ba2Fe1O6W1_ICSD_95520	metal	insulator
Ba2Fe1O6W1_ICSD_99061	insulator	insulator
Ba2Fe1S3_ICSD_615850	insulator	insulator
Ba2Fe1S5Sb1_ICSD_261418	insulator	insulator
Ba2Fe1Se3_ICSD_615861	insulator	insulator
Ba2Fe2Li1N3_ICSD_71060	metal	metal

Supplementary Table 150. Five-fold cross validated predictions for the metal/insulator classification (142/598).

system	calculated	predicted
Ba2Fe3O8Y1_ICSD_67711	metal	metal
Ba2Ga1Ge1N1_ICSD_84073	metal	insulator
Ba2Ga1H1O14P4_ICSD_280043	insulator	insulator
Ba2Ga2S5_ICSD_38256	insulator	insulator
Ba2Gd1Mo1O6_ICSD_236354	metal	insulator
Ba2Gd1Mo1O6_ICSD_236363	metal	insulator
Ba2Gd1Nb1O6_ICSD_109155	insulator	insulator
Ba2Gd1Nb1O6_ICSD_172405	insulator	insulator
Ba2Gd1O6Sb1_ICSD_33618	metal	metal
Ba2Ge1P2_ICSD_35150	insulator	insulator
Ba2Ge1S4_ICSD_615898	insulator	insulator
Ba2Ge1Se2Te2_ICSD_414165	insulator	insulator
Ba2Ge1Se4_ICSD_414166	insulator	insulator
Ba2Ge1_ICSD_25351	metal	metal
Ba2Ge2Mg1O7_ICSD_419312	insulator	insulator
Ba2Ge2Mn1O7_ICSD_184630	insulator	insulator
Ba2Ge2O1S6Zn1_ICSD_14174	insulator	insulator
Ba2Ge2O7Zn1_ICSD_420550	insulator	insulator
Ba2Ge2O8Ti1_ICSD_281271	insulator	insulator
Ba2Ge2O8Ti1_ICSD_39133	insulator	insulator
Ba2Ge2Se5_ICSD_410791	insulator	insulator
Ba2Ge2Te5_ICSD_59001	insulator	insulator
Ba2Ge4Ni5_ICSD_423268	metal	metal
Ba2Ge4Pd5_ICSD_186936	metal	metal
Ba2Ge4S10_ICSD_66868	insulator	insulator
Ba2H1N1_ICSD_67510	insulator	insulator
Ba2H3I1_ICSD_423520	insulator	insulator
Ba2H4Pd1_ICSD_165182	insulator	metal
Ba2H7O7Tl1_ICSD_411058	insulator	insulator
Ba2Hf1S4_ICSD_80652	insulator	insulator
Ba2Hg1S3_ICSD_32647	insulator	insulator
Ba2Hg1_ICSD_30084	metal	metal
Ba2Hg3O14Pd7_ICSD_72312	metal	insulator
Ba2Ho1Nb1O6_ICSD_109157	insulator	insulator
Ba2Ho1O6Re1_ICSD_25397	metal	metal
Ba2Ho1O6Ru1_ICSD_99638	insulator	insulator
Ba2Ho1O6Sb1_ICSD_150862	insulator	insulator
Ba2Ho1O6Ta1_ICSD_158358	insulator	insulator
Ba2Ho1O6Ta1_ICSD_158359	insulator	insulator
Ba2I1Na1O6_ICSD_425446	insulator	insulator
Ba2I2O1_ICSD_391434	insulator	insulator
Ba2In1Nb1O6_ICSD_172166	insulator	insulator
Ba2In1O6Ru1_ICSD_15260	insulator	insulator
Ba2In1O6Ta1_ICSD_261481	insulator	insulator
Ba2In1Sb1Se5_ICSD_425179	insulator	insulator
Ba2In1Se5Y1_ICSD_262888	insulator	insulator
Ba2In1_ICSD_261714	metal	metal
Ba2In2O5_ICSD_89438	insulator	insulator
Ba2In2Se5_ICSD_67187	insulator	insulator
Ba2Ir1La1O6_ICSD_152678	insulator	metal

Supplementary Table 151. Five-fold cross validated predictions for the metal/insulator classification (143/598).

system	calculated	predicted
Ba2Ir1La1O6_ICSD_174140	insulator	insulator
Ba2Ir1La1O6_ICSD_247170	metal	insulator
Ba2Ir1Lu1O6_ICSD_88158	metal	insulator
Ba2Ir1Nd1O6_ICSD_247171	metal	insulator
Ba2Ir1O4_ICSD_189053	metal	metal
Ba2Ir1O6Pr1_ICSD_150323	insulator	metal
Ba2Ir1O6Pr1_ICSD_155828	insulator	metal
Ba2Ir1O6Sr1_ICSD_74030	insulator	metal
Ba2Ir1O6Tb1_ICSD_171491	metal	metal
Ba2Ir1O6Y1_ICSD_152679	metal	metal
Ba2Ir1O6Y1_ICSD_88155	metal	metal
Ba2Ir3O9_ICSD_54725	metal	metal
Ba2K8O24U6_ICSD_91785	insulator	insulator
Ba2La1O6Re1_ICSD_25392	metal	metal
Ba2La1O6Ru1_ICSD_100793	insulator	insulator
Ba2La1O6Ru1_ICSD_155549	insulator	insulator
Ba2La1O6Ru1_ICSD_37028	insulator	insulator
Ba2La1O6Sb1_ICSD_153136	insulator	insulator
Ba2La1O6Ta1_ICSD_160168	insulator	insulator
Ba2La1O6Ta1_ICSD_160169	insulator	insulator
Ba2La1O6Ta1_ICSD_160170	insulator	insulator
Ba2La2Mn1O12W2_ICSD_54667	metal	insulator
Ba2Li1N1_ICSD_245651	metal	metal
Ba2Li1N4Re1_ICSD_411453	insulator	insulator
Ba2Li1O6Os1_ICSD_412142	metal	metal
Ba2Li1O6Re1_ICSD_109259	insulator	insulator
Ba2Li3N4Nb1_ICSD_75516	insulator	insulator
Ba2Li3N4Ta1_ICSD_75031	insulator	insulator
Ba2Lu1Nb1O6_ICSD_109161	insulator	insulator
Ba2Lu1O6Ru1_ICSD_202869	insulator	insulator
Ba2Lu1O6Sb1_ICSD_33622	metal	insulator
Ba2Mg17_ICSD_150735	metal	metal
Ba2Mg1O6Re1_ICSD_98524	metal	metal
Ba2Mg1O6U1_ICSD_23099	insulator	insulator
Ba2Mg1O6W1_ICSD_183771	insulator	insulator
Ba2Mg1O7Si2_ICSD_183983	insulator	insulator
Ba2Mg1O7Si2_ICSD_81117	insulator	insulator
Ba2Mg3Si4_ICSD_408938	metal	metal
Ba2Mn1Mo1O6_ICSD_98739	metal	metal
Ba2Mn1O3_ICSD_15508	insulator	insulator
Ba2Mn1O6Re1_ICSD_109256	metal	metal
Ba2Mn1O6Te1_ICSD_407802	metal	insulator
Ba2Mn1O6U1_ICSD_21104	metal	insulator
Ba2Mn1O6W1_ICSD_51609	metal	metal
Ba2Mn1S3_ICSD_26231	insulator	insulator
Ba2Mn1Se3_ICSD_26230	insulator	insulator
Ba2Mn1Te3_ICSD_1151	insulator	insulator
Ba2Mn2O1Sb2_ICSD_16360	metal	metal
Ba2Mn8O16_ICSD_62096	metal	insulator
Ba2Mo1Nd1O6_ICSD_172390	insulator	metal

Supplementary Table 152. Five-fold cross validated predictions for the metal/insulator classification (144/598).

system	calculated	predicted
Ba2Mo1Nd1O6_ICSD_26229	metal	metal
Ba2Mo1Ni1O6_ICSD_98192	insulator	metal
Ba2Mo1O6V1_ICSD_185890	metal	metal
Ba2N1_ICSD_409851	metal	metal
Ba2N2Zn1_ICSD_80377	insulator	insulator
Ba2N3Nb1_ICSD_74906	insulator	insulator
Ba2N3Ta1_ICSD_74503	insulator	insulator
Ba2N3V1_ICSD_80177	insulator	insulator
Ba2N6Ni1O12_ICSD_24508	metal	insulator
Ba2N8Si5_ICSD_401501	insulator	insulator
Ba2Na1Nb5O15_ICSD_53267	insulator	insulator
Ba2Na1Ni3O6_ICSD_59588	insulator	insulator
Ba2Na1O1_ICSD_411905	metal	insulator
Ba2Na1O6Os1_ICSD_412143	metal	insulator
Ba2Na1O6Re1_ICSD_200876	insulator	metal
Ba2Nb15O32_ICSD_69991	metal	insulator
Ba2Nb1Nd1O6_ICSD_109152	metal	insulator
Ba2Nb1O6Pr1_ICSD_245457	metal	insulator
Ba2Nb1O6Tb1_ICSD_245455	insulator	insulator
Ba2Nb1O6Tm1_ICSD_109159	metal	insulator
Ba2Nb1O6V1_ICSD_185889	metal	insulator
Ba2Nb1O6Y1_ICSD_172407	insulator	insulator
Ba2Nb1O6Yb1_ICSD_109160	metal	metal
Ba2Nb2O10Te1_ICSD_405153	insulator	insulator
Ba2Nb5O9_ICSD_78023	metal	metal
Ba2Nb6O21Te2_ICSD_405107	insulator	insulator
Ba2Nd1O6Ru1_ICSD_155551	insulator	metal
Ba2Nd1O6Sb1_ICSD_38328	metal	metal
Ba2Ni1O6Re1_ICSD_109251	metal	insulator
Ba2Ni1O6Te1_ICSD_25005	insulator	insulator
Ba2Ni1O6U1_ICSD_169535	insulator	insulator
Ba2Ni1O6W1_ICSD_24984	insulator	insulator
Ba2Ni1Si3_ICSD_280338	metal	metal
Ba2Ni3_ICSD_423734	metal	metal
Ba2O10Si4_ICSD_100314	insulator	insulator
Ba2O11Te2V2_ICSD_261183	insulator	insulator
Ba2O13Ti6_ICSD_300030	insulator	insulator
Ba2O21Ta6Te2_ICSD_405108	insulator	insulator
Ba2O3Pd1_ICSD_202812	insulator	metal
Ba2O3Zn1_ICSD_36659	insulator	insulator
Ba2O4Pb1_ICSD_66544	insulator	insulator
Ba2O4Sn1_ICSD_81849	insulator	insulator
Ba2O4Ti1_ICSD_2625	insulator	insulator
Ba2O4V1_ICSD_72625	insulator	insulator
Ba2O4Zr1_ICSD_39707	insulator	insulator
Ba2O5Re1_ICSD_202393	insulator	insulator
Ba2O5Tl2_ICSD_6322	insulator	insulator
Ba2O5W1_ICSD_62489	insulator	insulator
Ba2O6Pb1U1_ICSD_167504	insulator	insulator
Ba2O6Pr1Pt1_ICSD_80636	metal	insulator

Supplementary Table 153. Five-fold cross validated predictions for the metal/insulator classification (145/598).

system	calculated	predicted
Ba2O6Pr1Ru1_ICSD_155550	metal	insulator
Ba2O6Pr1Sb1_ICSD_153137	insulator	insulator
Ba2O6Pr1Sb1_ICSD_77660	metal	metal
Ba2O6Re1Tb1_ICSD_25395	metal	metal
Ba2O6Re1Tm1_ICSD_25398	metal	metal
Ba2O6Re1Y1_ICSD_94215	metal	metal
Ba2O6Re1Yb1_ICSD_25399	metal	metal
Ba2O6Re1Zn1_ICSD_109255	metal	insulator
Ba2O6Ru1Tm1_ICSD_55713	metal	insulator
Ba2O6Ru1Y1_ICSD_202864	insulator	insulator
Ba2O6Ru1Yb1_ICSD_55714	metal	metal
Ba2O6Sb1Sc1_ICSD_33623	metal	insulator
Ba2O6Sb1Tb1_ICSD_38332	insulator	metal
Ba2O6Sb1Y1_ICSD_33624	metal	insulator
Ba2O6Sb1Y1_ICSD_84650	insulator	insulator
Ba2O6Sb1Yb1_ICSD_33621	metal	metal
Ba2O6Sb1Yb1_ICSD_38336	metal	metal
Ba2O6Sr1Te1_ICSD_246109	insulator	insulator
Ba2O6Sr1W1_ICSD_246114	insulator	insulator
Ba2O6Ta1Y1_ICSD_171176	insulator	insulator
Ba2O6Ta1Y1_ICSD_261453	insulator	insulator
Ba2O6Ta1Yb1_ICSD_91001	metal	metal
Ba2O6U1Zn1_ICSD_167502	insulator	insulator
Ba2O6W1Zn1_ICSD_423034	insulator	insulator
Ba2O7P2_ICSD_261125	insulator	insulator
Ba2O7Si2Zn1_ICSD_409588	insulator	insulator
Ba2O7U2_ICSD_63076	insulator	insulator
Ba2O8Si2Ti1_ICSD_201844	insulator	insulator
Ba2O8Si2V1_ICSD_51479	insulator	insulator
Ba2O9P2Te1_ICSD_416032	insulator	insulator
Ba2O9P2V1_ICSD_56849	metal	insulator
Ba2O9V3_ICSD_404713	insulator	insulator
Ba2P3Pd1_ICSD_75019	metal	insulator
Ba2Pb1_ICSD_58666	metal	metal
Ba2Re6S11_ICSD_201072	insulator	insulator
Ba2S3Zn1_ICSD_653999	insulator	insulator
Ba2S3_ICSD_70058	insulator	insulator
Ba2S4Si1_ICSD_42661	insulator	insulator
Ba2S4Sn1_ICSD_16273	insulator	insulator
Ba2S4Sn1_ICSD_42036	insulator	insulator
Ba2S4Ti1_ICSD_616084	insulator	insulator
Ba2S4Zr1_ICSD_80651	insulator	insulator
Ba2Sb1_ICSD_41837	metal	metal
Ba2Sb2Zn1_ICSD_421425	insulator	metal
Ba2Sb3_ICSD_61089	insulator	insulator
Ba2Sb6Sn3_ICSD_82529	metal	metal
Ba2Se4Si1_ICSD_49750	insulator	insulator
Ba2Se5Sn1_ICSD_248033	insulator	insulator
Ba2Si1Te4_ICSD_49751	insulator	insulator
Ba2Si1_ICSD_52698	metal	metal

Supplementary Table 154. Five-fold cross validated predictions for the metal/insulator classification (146/598).

system	calculated	predicted
Ba2Si46_ICSD_94262	metal	metal
Ba2Sn1Te5_ICSD_81371	metal	insulator
Ba2Sn1_ICSD_58679	metal	metal
Ba2Sn6Yb2_ICSD_249516	metal	metal
Ba2Zn1_ICSD_616175	metal	metal
Ba3Be1Cl18Zr6_ICSD_33993	insulator	insulator
Ba3Bi1Cl3O3_ICSD_69618	insulator	insulator
Ba3Bi1Ir2O9_ICSD_174292	insulator	metal
Ba3Bi1N1_ICSD_152055	insulator	insulator
Ba3Bi1Na1O6_ICSD_72839	insulator	insulator
Ba3Bi1O9Ru2_ICSD_72448	insulator	insulator
Ba3Bi2O16P4_ICSD_61061	insulator	insulator
Ba3Bi2O9Te1_ICSD_90842	insulator	insulator
Ba3Br2Fe2O5_ICSD_48179	insulator	insulator
Ba3C1Cl4O3_ICSD_174065	insulator	insulator
Ba3C1F7O3Sc1_ICSD_75255	insulator	insulator
Ba3C5F2La2O15_ICSD_72445	insulator	insulator
Ba3C60_ICSD_70063	metal	insulator
Ba3Ca1Ir2O9_ICSD_245253	metal	insulator
Ba3Ca1Ir2O9_ICSD_246280	metal	metal
Ba3Ca1O9Ru2_ICSD_152482	insulator	insulator
Ba3Ca1O9Ru2_ICSD_71209	insulator	insulator
Ba3Ca1O9Ru2_ICSD_73183	insulator	insulator
Ba3Ca1O9Sb2_ICSD_249664	insulator	insulator
Ba3Ca2N6Si2_ICSD_187336	insulator	insulator
Ba3Cd2Sb4_ICSD_173685	metal	metal
Ba3Ce1O9Ru2_ICSD_401912	metal	metal
Ba3Ce1O9Ru2_ICSD_94024	metal	metal
Ba3Cl2Cu2O4_ICSD_115	insulator	insulator
Ba3Cl2Fe2O5_ICSD_48178	insulator	insulator
Ba3Cl2O5W1_ICSD_63518	insulator	insulator
Ba3Co1Nb2O9_ICSD_150431	insulator	insulator
Ba3Co1O9Ru2_ICSD_50830	metal	insulator
Ba3Co1O9Ru2_ICSD_69092	insulator	metal
Ba3Co1O9Sb2_ICSD_151442	insulator	insulator
Ba3Cr1N3_ICSD_154802	metal	metal
Ba3Cr1O5_ICSD_73893	insulator	insulator
Ba3Cr1S5_ICSD_97539	metal	insulator
Ba3Cr2Mo1O9_ICSD_81071	insulator	insulator
Ba3Cr2O8_ICSD_9457	metal	insulator
Ba3Cr2O9W1_ICSD_81072	insulator	insulator
Ba3Cr2S6_ICSD_97540	insulator	insulator
Ba3Cu1O9Sb2_ICSD_2279	metal	insulator
Ba3Cu2Er2O10Pt1_ICSD_69569	insulator	insulator
Ba3Cu2Ho2O10Pt1_ICSD_62391	insulator	insulator
Ba3Cu2O10Pt1Y2_ICSD_62390	insulator	insulator
Ba3Dy1O9Ru2_ICSD_401914	insulator	insulator
Ba3Dy4O9_ICSD_72480	insulator	insulator
Ba3Er1O9Ru2_ICSD_401915	insulator	insulator
Ba3Er4O9_ICSD_72481	insulator	insulator

Supplementary Table 155. Five-fold cross validated predictions for the metal/insulator classification (147/598).

system	calculated	predicted
Ba3F12In2_ICSD_48182	insulator	insulator
Ba3Fe1N3_ICSD_36502	metal	metal
Ba3Fe1O5_ICSD_281029	metal	insulator
Ba3Fe1S5_ICSD_280	insulator	insulator
Ba3Fe3Nb1O14Si2_ICSD_162894	insulator	insulator
Ba3Fe3Se7_ICSD_16310	insulator	insulator
Ba3Ga2Ge4O14_ICSD_250124	insulator	insulator
Ba3Ga2N4_ICSD_82736	insulator	insulator
Ba3Ga2S6_ICSD_201421	insulator	insulator
Ba3Ga3Nb1O14Si2_ICSD_154215	insulator	insulator
Ba3Ge1O1_ICSD_50512	insulator	metal
Ba3Ge2N2_ICSD_81819	metal	metal
Ba3Ge4_ICSD_391060	metal	metal
Ba3Ge4_ICSD_391061	metal	metal
Ba3Ge5_ICSD_167163	metal	metal
Ba3Hg10In1_ICSD_290305	metal	metal
Ba3Ho4O9_ICSD_33807	insulator	insulator
Ba3I2P3_ICSD_391128	metal	insulator
Ba3In1O9Ru2_ICSD_15261	insulator	insulator
Ba3In2O11Zn5_ICSD_73192	insulator	insulator
Ba3In2P4_ICSD_402812	insulator	insulator
Ba3Ir1Na1O6_ICSD_405134	metal	metal
Ba3Ir2La1O9_ICSD_174293	metal	insulator
Ba3Ir2Li1O9_ICSD_413453	metal	metal
Ba3Ir2Mg1O9_ICSD_245251	metal	metal
Ba3Ir2Na1O9_ICSD_413452	insulator	metal
Ba3Ir2Ni1O9_ICSD_33530	metal	metal
Ba3Ir2O9Sr1_ICSD_245254	insulator	metal
Ba3Ir2O9Y1_ICSD_16365	metal	insulator
Ba3Ir2O9Y1_ICSD_16367	insulator	metal
Ba3La1O9Ru2_ICSD_51926	insulator	insulator
Ba3Li1N1_ICSD_245652	metal	metal
Ba3Li1O9Os2_ICSD_281247	metal	metal
Ba3Li4Sb4_ICSD_615946	metal	metal
Ba3Li4Sn8_ICSD_240016	metal	metal
Ba3Lu1O9Ru2_ICSD_51929	insulator	insulator
Ba3Lu4O9_ICSD_38383	insulator	insulator
Ba3Mg1O8Si2_ICSD_419862	insulator	insulator
Ba3Mg1O9Ru2_ICSD_33529	insulator	insulator
Ba3Mg1O9Sb2_ICSD_33527	insulator	insulator
Ba3Mn1N3_ICSD_80771	metal	metal
Ba3Mn1Nb2O9_ICSD_171479	metal	insulator
Ba3Mn2O8_ICSD_280045	insulator	insulator
Ba3N1Na1_ICSD_67497	metal	metal
Ba3N1Sb1_ICSD_152054	insulator	insulator
Ba3N1_ICSD_77730	metal	metal
Ba3N2O12Si6_ICSD_421322	insulator	insulator
Ba3N2O1Zn1_ICSD_55536	insulator	insulator
Ba3N4O9Si6_ICSD_415918	insulator	insulator
Ba3N8O6P6_ICSD_710077	insulator	insulator

Supplementary Table 156. Five-fold cross validated predictions for the metal/insulator classification (148/598).

system	calculated	predicted
Ba3Na1Nb1O6_ICSD_72330	insulator	insulator
Ba3Na1O6Ru1_ICSD_405133	insulator	insulator
Ba3Na1O6Ta1_ICSD_72331	insulator	insulator
Ba3Na1O9Os2_ICSD_281248	metal	insulator
Ba3Na1O9Ru2_ICSD_281127	metal	insulator
Ba3Nb2O18P4_ICSD_90878	insulator	insulator
Ba3Nb2O8_ICSD_95193	insulator	insulator
Ba3Nb2O9Sr1_ICSD_24390	insulator	insulator
Ba3Nb2O9Sr1_ICSD_37161	insulator	insulator
Ba3Nb2O9Zn1_ICSD_157044	insulator	insulator
Ba3Nb5O15_ICSD_69993	metal	insulator
Ba3Nb6O26Si4_ICSD_16029	insulator	insulator
Ba3Ni1O4_ICSD_30662	insulator	insulator
Ba3Ni1O9Ru2_ICSD_50831	insulator	insulator
Ba3Ni1O9Sb2_ICSD_1177	insulator	insulator
Ba3Ni1O9Ta2_ICSD_240281	insulator	insulator
Ba3O11Te4_ICSD_37069	insulator	insulator
Ba3O13P4_ICSD_280908	insulator	insulator
Ba3O1Pb1_ICSD_100791	metal	metal
Ba3O1Sb2_ICSD_280592	insulator	insulator
Ba3O1Sb4_ICSD_415032	insulator	insulator
Ba3O1Sn1_ICSD_100792	metal	metal
Ba3O20Si4Te1Zn6_ICSD_416231	insulator	insulator
Ba3O23Si4Ta6_ICSD_18316	insulator	insulator
Ba3O26Si4Ta6_ICSD_18317	insulator	insulator
Ba3O3S4V2_ICSD_279607	insulator	insulator
Ba3O4P2Se4_ICSD_414638	insulator	insulator
Ba3O5Si1_ICSD_1449	insulator	insulator
Ba3O6Sb2_ICSD_413764	insulator	insulator
Ba3O6W1_ICSD_76437	insulator	insulator
Ba3O8P2_ICSD_69450	insulator	insulator
Ba3O8V2_ICSD_78169	insulator	insulator
Ba3O9Ru2Sr1_ICSD_48102	insulator	insulator
Ba3O9Ru2Tb1_ICSD_95165	insulator	insulator
Ba3O9Ru2Y1_ICSD_51924	insulator	insulator
Ba3O9Ru2Yb1_ICSD_400598	metal	insulator
Ba3O9Ru2Zn1_ICSD_69090	insulator	insulator
Ba3O9Ru2Zr1_ICSD_172754	metal	metal
Ba3O9Sb2Sr1_ICSD_249663	insulator	insulator
Ba3O9Sr1Ta2_ICSD_24391	insulator	insulator
Ba3O9Sr1Ta2_ICSD_27496	insulator	insulator
Ba3O9Sr1Ta2_ICSD_37162	insulator	insulator
Ba3O9Te2_ICSD_100797	insulator	insulator
Ba3O9Y4_ICSD_87118	insulator	insulator
Ba3O9Yb4_ICSD_33239	metal	insulator
Ba3P14_ICSD_23629	metal	insulator
Ba3P2S8_ICSD_417422	insulator	insulator
Ba3P4Sn2_ICSD_80132	insulator	insulator
Ba3P4_ICSD_38322	insulator	insulator
Ba3P6Si4_ICSD_29261	insulator	insulator

Supplementary Table 157. Five-fold cross validated predictions for the metal/insulator classification (149/598).

system	calculated	predicted
Ba3Pb5_ICSD_165184	metal	metal
Ba3S5Si1_ICSD_26377	insulator	insulator
Ba3S5Ti1_ICSD_203087	insulator	insulator
Ba3S7Sn2_ICSD_166524	insulator	insulator
Ba3S7Zr2_ICSD_59271	insulator	insulator
Ba3S8Ta2_ICSD_202878	insulator	insulator
Ba3Si4_ICSD_419308	metal	metal
Ba3Sn5_ICSD_167166	metal	metal
Ba4Bi3_ICSD_96943	metal	metal
Ba4Br2Si3_ICSD_411640	insulator	insulator
Ba4Br6O1_ICSD_391435	insulator	insulator
Ba4C2Cu1Li1O10_ICSD_401122	insulator	insulator
Ba4C2Cu1Na1O10_ICSD_66776	insulator	insulator
Ba4Cd3S10Tb2_ICSD_411167	insulator	insulator
Ba4Ce1Mn3O12_ICSD_99661	insulator	insulator
Ba4Cl12Co2_ICSD_280274	insulator	insulator
Ba4Cl1O18Os6_ICSD_82910	metal	metal
Ba4Cl2N4W1_ICSD_50003	insulator	insulator
Ba4Cl6O1_ICSD_16026	insulator	insulator
Ba4Cu2Nd2O9_ICSD_73904	metal	insulator
Ba4Cu3O9Yb1_ICSD_69718	metal	metal
Ba4Cu6O13Y2_ICSD_390001	metal	metal
Ba4Er1O12Ru3_ICSD_174186	insulator	insulator
Ba4F24H1P3_ICSD_419143	insulator	insulator
Ba4Fe2I5S4_ICSD_173357	metal	insulator
Ba4Ga2S7_ICSD_201420	insulator	insulator
Ba4Ge1P4_ICSD_32560	insulator	insulator
Ba4Ge20Ni3_ICSD_57034	metal	metal
Ba4Ge20Pd3_ICSD_57035	metal	metal
Ba4Ge20Pt3_ICSD_615897	metal	metal
Ba4H0.666Nb2O9.333_ICSD_165531	insulator	insulator
Ba4Hf3S10_ICSD_602359	insulator	insulator
Ba4Ho1O12Ru3_ICSD_160868	insulator	insulator
Ba4I6O1_ICSD_280584	insulator	insulator
Ba4In2S8_ICSD_261680	insulator	insulator
Ba4Ir3O10_ICSD_95063	metal	metal
Ba4K1O1Sb3_ICSD_410747	insulator	insulator
Ba4Li1Nb3O12_ICSD_19009	insulator	insulator
Ba4Li1O12Ta3_ICSD_19010	insulator	insulator
Ba4Mg1N6Si2_ICSD_187335	insulator	insulator
Ba4Mn3Nd1O12_ICSD_156305	metal	insulator
Ba4Mn3O10_ICSD_51881	insulator	insulator
Ba4Mn3O12Pr1_ICSD_99662	insulator	insulator
Ba4Na1O12Sb3_ICSD_160173	insulator	insulator
Ba4O10Ru3_ICSD_90902	metal	insulator
Ba4O12Ru3Tb1_ICSD_160870	metal	insulator
Ba4O12Ru3Zr1_ICSD_47133	metal	metal
Ba4O16Si6_ICSD_100310	insulator	insulator
Ba4O1Rb1Sb3_ICSD_415036	insulator	insulator
Ba4O1Sb2_ICSD_402284	insulator	insulator

Supplementary Table 158. Five-fold cross validated predictions for the metal/insulator classification (150/598).

system	calculated	predicted
Ba4O6Pt1_ICSD_65706	insulator	insulator
Ba4O9Ta2_ICSD_166900	insulator	insulator
Ba4P3_ICSD_73206	insulator	insulator
Ba4P4Si1_ICSD_32559	insulator	insulator
Ba4P4Ti1_ICSD_380114	insulator	insulator
Ba4S10Zr3_ICSD_72656	insulator	insulator
Ba4Sb4Se11_ICSD_31500	insulator	insulator
Ba5Bi3_ICSD_41839	metal	metal
Ba5Bi5In4_ICSD_54853	metal	metal
Ba5Br1O12P3_ICSD_409482	insulator	insulator
Ba5Br2O9Ru2_ICSD_245668	insulator	insulator
Ba5Br6O4Si1_ICSD_73365	insulator	insulator
Ba5Cd2O2Sb4_ICSD_423458	insulator	insulator
Ba5Cl1Mn3O12_ICSD_61356	insulator	insulator
Ba5Cl1O12P3_ICSD_8191	insulator	insulator
Ba5Cl1O12V3_ICSD_170769	insulator	insulator
Ba5Cl1O15Os3_ICSD_80447	insulator	insulator
Ba5Cl1O15Re3_ICSD_73928	insulator	insulator
Ba5Cl2O9Ta2_ICSD_240872	insulator	insulator
Ba5Co5O14_ICSD_153698	insulator	insulator
Ba5Cr1N5_ICSD_82360	insulator	insulator
Ba5Er8O21Zn4_ICSD_400436	insulator	insulator
Ba5Fe4S11_ICSD_16372	insulator	insulator
Ba5Fe5O14_ICSD_391229	metal	insulator
Ba5Ga5Pb1_ICSD_416339	metal	metal
Ba5Ga5Sn1_ICSD_416340	metal	metal
Ba5Gd8O21Zn4_ICSD_94223	insulator	insulator
Ba5Ge3_ICSD_409375	metal	metal
Ba5Hf4S13_ICSD_71936	insulator	insulator
Ba5Ho8O21Zn4_ICSD_73186	insulator	insulator
Ba5I3P5_ICSD_391129	metal	insulator
Ba5In2Sb6_ICSD_62305	metal	insulator
Ba5Nb4O15_ICSD_157477	insulator	insulator
Ba5O10Ru2_ICSD_75386	insulator	insulator
Ba5O15Ta4_ICSD_16028	insulator	insulator
Ba5O21Yb8Zn4_ICSD_94228	metal	insulator
Ba5P4_ICSD_413273	insulator	insulator
Ba5P9_ICSD_98667	metal	insulator
Ba5Pb3_ICSD_58668	metal	metal
Ba5Sb3_ICSD_616108	metal	metal
Ba5Sb4_ICSD_280022	insulator	metal
Ba5Sb4_ICSD_52693	insulator	insulator
Ba5Si3_ICSD_24598	metal	metal
Ba5Sn3_ICSD_100084	metal	metal
Ba6C60_ICSD_70062	insulator	metal
Ba6Cl2Eu1F12_ICSD_411225	insulator	insulator
Ba6Cl2Ir1Nb2O12_ICSD_40693	metal	insulator
Ba6Cl2O12Pt1Ru2_ICSD_72280	insulator	insulator
Ba6Cl2O12Ru3_ICSD_99681	insulator	insulator
Ba6Co25S27_ICSD_71939	metal	metal

Supplementary Table 159. Five-fold cross validated predictions for the metal/insulator classification (151/598).

system	calculated	predicted
Ba6F26Mg7_ICSD_50217	insulator	insulator
Ba6Ga2P6_ICSD_402177	insulator	insulator
Ba6Ga5N1_ICSD_77731	metal	metal
Ba6Ge5N2_ICSD_154398	metal	metal
Ba6Hf5S16_ICSD_71935	insulator	insulator
Ba6Mg23_ICSD_109225	metal	metal
Ba6Mn5O16_ICSD_97017	insulator	insulator
Ba6N1Na16_ICSD_78394	metal	metal
Ba6N6O1Os2_ICSD_419467	metal	insulator
Ba6N6O1Re2_ICSD_419636	metal	insulator
Ba6Na2Nb2O17P2_ICSD_249742	insulator	insulator
Ba6Na2Si46_ICSD_409912	metal	metal
Ba6Nb8O30Ti2_ICSD_36081	insulator	insulator
Ba6Ni25S27_ICSD_83476	metal	metal
Ba6O17Ti4Y2_ICSD_96630	insulator	insulator
Ba6O18W4_ICSD_9725	insulator	insulator
Ba6P6Sn2_ICSD_35342	insulator	insulator
Ba6Si46_ICSD_94263	metal	metal
Ba7Br2O15Ru4_ICSD_73069	metal	metal
Ba7Cl2F12_ICSD_87084	insulator	insulator
Ba7Cl2O15Ru4_ICSD_71755	metal	metal
Ba7Cu1F34Fe6_ICSD_203173	insulator	insulator
Ba7Ir6O19_ICSD_74686	insulator	metal
Ba8Cu1Si16_ICSD_182550	metal	insulator
Ba8Cu6Ge40_ICSD_172837	metal	metal
Ba8Si46_ICSD_90093	metal	metal
Ba8Si6Sn1_ICSD_169999	insulator	metal
Ba9C1N10Nb2O1_ICSD_412664	insulator	insulator
Ba9Cl2Cu7O15_ICSD_9628	metal	insulator
Ba9N12Nb2_ICSD_402743	insulator	insulator
Ba9O24Sc2Si6_ICSD_75175	insulator	insulator
Be12Co1_ICSD_616202	metal	metal
Be12Cr1_ICSD_150599	metal	metal
Be12Fe1_ICSD_616264	metal	metal
Be12Mn1_ICSD_616329	metal	metal
Be12Mo1_ICSD_616339	metal	metal
Be12Nb1_ICSD_616353	metal	metal
Be12Pd1_ICSD_109314	metal	metal
Be12Pt1_ICSD_109315	metal	metal
Be12Ta1_ICSD_616426	metal	metal
Be12Ti1_ICSD_58745	metal	metal
Be12Ti1_ICSD_616454	metal	metal
Be12V1_ICSD_616492	metal	metal
Be12W1_ICSD_109316	metal	metal
Be13Ca1_ICSD_616187	metal	metal
Be13Ce1_ICSD_659553	metal	metal
Be13Dy1_ICSD_616260	metal	metal
Be13Er1_ICSD_603924	metal	metal
Be13Eu1_ICSD_616262	metal	metal
Be13Gd1_ICSD_616277	metal	metal

Supplementary Table 160. Five-fold cross validated predictions for the metal/insulator classification (152/598).

system	calculated	predicted
Be13Hf1_ICSD_616289	metal	metal
Be13Ho1_ICSD_616296	metal	metal
Be13La1_ICSD_616302	metal	metal
Be13Lu1_ICSD_616321	metal	metal
Be13Mg1_ICSD_616325	metal	metal
Be13Nd1_ICSD_616361	metal	metal
Be13Pa1_ICSD_616385	metal	metal
Be13Pr1_ICSD_616393	metal	metal
Be13Pu1_ICSD_58731	metal	metal
Be13Sb1_ICSD_834	metal	metal
Be13Sc1_ICSD_616416	metal	metal
Be13Sr1_ICSD_616423	metal	metal
Be13Tb1_ICSD_616437	metal	metal
Be13Th1_ICSD_58740	metal	metal
Be13Tm1_ICSD_603925	metal	metal
Be13Tm1_ICSD_616468	metal	metal
Be13U1_ICSD_58750	metal	metal
Be13Y1_ICSD_616507	metal	metal
Be13Yb1_ICSD_603926	metal	metal
Be13Zr1_ICSD_616516	metal	metal
Be15Cu8Ta6_ICSD_58704	metal	metal
Be17Ca12O29_ICSD_14359	insulator	insulator
Be17Hf2_ICSD_616284	metal	metal
Be17Hf2_ICSD_616285	metal	metal
Be17Nb2_ICSD_58724	metal	metal
Be17Os3_ICSD_616380	metal	metal
Be17Ru3_ICSD_58735	metal	metal
Be17Ta2_ICSD_616430	metal	metal
Be17Ti2_ICSD_109217	metal	metal
Be17Ti2_ICSD_616452	metal	metal
Be17Zr2_ICSD_616514	metal	metal
Be1Bi1La3S7_ICSD_616183	insulator	insulator
Be1Br2_ICSD_92584	insulator	insulator
Be1C1N2_ICSD_181041	insulator	insulator
Be1C1N2_ICSD_181042	insulator	insulator
Be1C4Cl2H16O4S2_ICSD_249588	insulator	insulator
Be1C4K2O8_ICSD_1446	insulator	insulator
Be1Ca1F1Na1O6Si2_ICSD_15314	insulator	insulator
Be1Ca2O7Si2_ICSD_31234	insulator	insulator
Be1Cl2_ICSD_173559	insulator	insulator
Be1Cl2_ICSD_173560	insulator	insulator
Be1Cl2_ICSD_173561	insulator	insulator
Be1Cl2_ICSD_31696	insulator	insulator
Be1Cl2_ICSD_78774	insulator	insulator
Be1Cl4Cs2_ICSD_49622	insulator	insulator
Be1Co1_ICSD_150619	metal	metal
Be1Co1_ICSD_58693	metal	metal
Be1Co1_ICSD_616204	metal	metal
Be1Cr1Dy3S7_ICSD_616230	insulator	insulator
Be1Cr1Er3S7_ICSD_616231	insulator	insulator

Supplementary Table 161. Five-fold cross validated predictions for the metal/insulator classification (153/598).

system	calculated	predicted
Be1Cr1La3S7_ICSD_616233	insulator	insulator
Be1Cr1Nd3S7_ICSD_616236	insulator	insulator
Be1Cr1S7Y3_ICSD_616240	insulator	insulator
Be1Cs1F3_ICSD_290358	insulator	insulator
Be1Cs1F3_ICSD_9870	insulator	insulator
Be1Cs1F4Li1_ICSD_42463	insulator	insulator
Be1Cs1F4Li1_ICSD_9434	insulator	insulator
Be1Cs1H3_ICSD_173446	insulator	insulator
Be1Cs2F4_ICSD_153084	insulator	insulator
Be1Cs2F4_ICSD_23152	insulator	insulator
Be1Cu12La1_ICSD_658880	metal	metal
Be1Cu1_ICSD_616241	metal	metal
Be1F2_ICSD_173557	insulator	insulator
Be1F2_ICSD_173558	insulator	insulator
Be1F2_ICSD_261194	insulator	insulator
Be1F2_ICSD_9481	insulator	insulator
Be1F3H4N1_ICSD_61060	insulator	insulator
Be1F4H4Li1N1_ICSD_9433	insulator	insulator
Be1F4H5Li1N2_ICSD_1903	insulator	insulator
Be1F4H8N2_ICSD_200916	insulator	insulator
Be1F4K1Li1_ICSD_2773	insulator	insulator
Be1F4K2_ICSD_153081	insulator	insulator
Be1F4K2_ICSD_50337	insulator	insulator
Be1F4Li2_ICSD_14360	insulator	insulator
Be1F4Na2_ICSD_20366	insulator	insulator
Be1F4Na2_ICSD_28105	insulator	insulator
Be1F4Pb1_ICSD_24568	insulator	insulator
Be1F4Rb2_ICSD_153082	insulator	insulator
Be1F4Rb2_ICSD_61800	insulator	insulator
Be1F4Tl2_ICSD_171183	insulator	insulator
Be1F4Yb1_ICSD_411132	insulator	insulator
Be1F5K3_ICSD_14114	insulator	insulator
Be1F6Ho1K1_ICSD_2143	insulator	insulator
Be1Ga1La3S7_ICSD_616274	insulator	insulator
Be1Ge1Na2O4_ICSD_80357	insulator	insulator
Be1H2O2_ICSD_50445	insulator	insulator
Be1H2O4Se1_ICSD_410094	insulator	insulator
Be1H2_ICSD_161367	insulator	insulator
Be1H3K1_ICSD_173445	insulator	insulator
Be1H3Li1_ICSD_162770	insulator	insulator
Be1H3Li1_ICSD_162772	insulator	insulator
Be1H3Li1_ICSD_162773	insulator	insulator
Be1H3Li1_ICSD_162774	insulator	insulator
Be1H3Li1_ICSD_162775	insulator	insulator
Be1H3Li1_ICSD_173447	insulator	insulator
Be1H3Na1_ICSD_158250	insulator	insulator
Be1H3Rb1_ICSD_173449	insulator	insulator
Be1H4N1O4P1_ICSD_85445	insulator	insulator
Be1H4Na2_ICSD_159452	insulator	insulator
Be1H4O4Sr1_ICSD_180022	insulator	insulator

Supplementary Table 162. Five-fold cross validated predictions for the metal/insulator classification (154/598).

system	calculated	predicted
Be1H8I2O10_ICSD_83330	insulator	insulator
Be1H8O8S1_ICSD_74572	insulator	insulator
Be1H8O8Se1_ICSD_150083	insulator	insulator
Be1Hf1Si1_ICSD_108103	metal	metal
Be1Hf1_ICSD_616286	metal	metal
Be1I2_ICSD_92585	insulator	insulator
Be1In1La3S7_ICSD_616297	insulator	insulator
Be1K1O4P1_ICSD_4255	insulator	insulator
Be1K1O9P3_ICSD_40866	insulator	insulator
Be1K4P2_ICSD_300110	insulator	insulator
Be1La3S7Sb1_ICSD_616308	insulator	insulator
Be1La3S7Sc1_ICSD_616309	insulator	insulator
Be1La3S7Ti1_ICSD_616310	insulator	insulator
Be1La3S7V1_ICSD_616311	insulator	insulator
Be1La3S7Yb1_ICSD_616312	metal	insulator
Be1Li1N1_ICSD_402341	insulator	insulator
Be1Li1P1_ICSD_616316	insulator	insulator
Be1Li1Sb1_ICSD_616318	insulator	insulator
Be1Li1_ICSD_188829	metal	metal
Be1Li2O4Si1_ICSD_2319	insulator	insulator
Be1Mo3_ICSD_58715	metal	metal
Be1N2Si1_ICSD_25704	insulator	insulator
Be1N2Si1_ICSD_44112	insulator	insulator
Be1N4P2_ICSD_421385	insulator	insulator
Be1Na1Sb1_ICSD_100092	insulator	insulator
Be1Na2O2_ICSD_67154	insulator	insulator
Be1Ni1_ICSD_616363	metal	metal
Be1O1_ICSD_163467	insulator	insulator
Be1O1_ICSD_163826	insulator	insulator
Be1O1_ICSD_18147	insulator	insulator
Be1O1_ICSD_34237	insulator	insulator
Be1O4S1_ICSD_44801	insulator	insulator
Be1O6P2_ICSD_100404	insulator	insulator
Be1P2_ICSD_2262	insulator	insulator
Be1Pd1_ICSD_58728	metal	metal
Be1Pd2_ICSD_107609	metal	metal
Be1Pd3_ICSD_107610	metal	metal
Be1Rh1_ICSD_58734	metal	metal
Be1S1_ICSD_186889	insulator	insulator
Be1S1_ICSD_52719	insulator	insulator
Be1Se1_ICSD_52720	insulator	insulator
Be1Se1_ICSD_616419	insulator	insulator
Be1Si1Zr1_ICSD_76123	metal	metal
Be1Ta2_ICSD_58738	metal	metal
Be1Te1_ICSD_290008	insulator	metal
Be1Te1_ICSD_52722	metal	insulator
Be1Ti1_ICSD_616457	metal	metal
Be1Zn13_ICSD_174029	metal	metal
Be1_ICSD_52263	metal	metal
Be1_ICSD_52706	metal	metal

Supplementary Table 163. Five-fold cross validated predictions for the metal/insulator classification (155/598).

system	calculated	predicted
Be1_ICSD_52708	metal	metal
Be22Mo1_ICSD_616336	metal	metal
Be22Re1_ICSD_616399	metal	metal
Be22Tc1_ICSD_616438	metal	metal
Be22W1_ICSD_616499	metal	metal
Be2C1_ICSD_183009	insulator	metal
Be2Ca1Ge2_ICSD_25337	metal	metal
Be2Ca1P2_ICSD_616191	insulator	insulator
Be2Ca3H10O20P4_ICSD_40671	insulator	insulator
Be2Cr1_ICSD_58697	metal	metal
Be2Cs1F5_ICSD_2801	insulator	insulator
Be2Cu1_ICSD_616247	metal	metal
Be2Dy2Ge1O7_ICSD_39121	insulator	insulator
Be2Er2Ge1O7_ICSD_39123	insulator	insulator
Be2F5Rb1_ICSD_28541	insulator	insulator
Be2F7H4Li2N1_ICSD_240273	insulator	insulator
Be2F7Li1Na2_ICSD_9430	insulator	insulator
Be2F7Li2Rb1_ICSD_72	insulator	insulator
Be2F8K2Pb1_ICSD_9902	insulator	insulator
Be2F8K2Sr1_ICSD_109005	insulator	insulator
Be2Fe1_ICSD_616265	metal	metal
Be2Gd2Ge1O7_ICSD_39120	insulator	insulator
Be2Ge1La2O7_ICSD_39117	insulator	insulator
Be2Ge1O7Pr2_ICSD_39118	insulator	insulator
Be2Ge1O7Y2_ICSD_39122	insulator	insulator
Be2Hf1_ICSD_616288	metal	metal
Be2Ho2O7Si1_ICSD_73521	insulator	insulator
Be2K2Na4O5_ICSD_33849	insulator	insulator
Be2K4O4_ICSD_23633	insulator	insulator
Be2La2O5_ICSD_36063	insulator	insulator
Be2Mg1N2_ICSD_413358	insulator	insulator
Be2Mg1P2_ICSD_616328	insulator	insulator
Be2Mn1_ICSD_58714	metal	metal
Be2Mo1_ICSD_616337	metal	metal
Be2Nb1_ICSD_58721	metal	metal
Be2Nb3_ICSD_58722	metal	metal
Be2O10P3Rb1_ICSD_72985	insulator	insulator
Be2O4Si1_ICSD_202275	insulator	insulator
Be2O7Si1Y2_ICSD_23233	insulator	insulator
Be2Re1_ICSD_58732	metal	metal
Be2Ru1_ICSD_616410	metal	metal
Be2Si1_ICSD_183010	metal	insulator
Be2Ta1_ICSD_58739	metal	metal
Be2Ta3_ICSD_616431	metal	metal
Be2Ti1_ICSD_616466	metal	metal
Be2V1_ICSD_58752	metal	metal
Be2W1_ICSD_58754	metal	metal
Be2Zn1_ICSD_168955	metal	metal
Be2Zr1_ICSD_58756	metal	metal
Be3Ca3F2Li2O12Si3_ICSD_39389	insulator	insulator

Supplementary Table 164. Five-fold cross validated predictions for the metal/insulator classification (156/598).

system	calculated	predicted
Be3Cd2F12Rb2_ICSD_15155	insulator	insulator
Be3Cl1Li4O12P3_ICSD_74525	insulator	insulator
Be3F12K2Mg2_ICSD_15152	insulator	insulator
Be3F12K2Mn2_ICSD_83658	insulator	insulator
Be3F12K2Zn2_ICSD_24962	insulator	insulator
Be3F9K1Zn1_ICSD_18022	insulator	insulator
Be3F9Rb1Zn1_ICSD_23133	insulator	insulator
Be3H4O10P2_ICSD_88664	insulator	insulator
Be3K4O5_ICSD_33808	insulator	insulator
Be3Mn4O12S1Si3_ICSD_2709	insulator	insulator
Be3N2_ICSD_185490	insulator	insulator
Be3N2_ICSD_185491	insulator	insulator
Be3N2_ICSD_25656	insulator	insulator
Be3N2_ICSD_412667	insulator	insulator
Be3Nb1_ICSD_58723	metal	metal
Be3O4Sr1_ICSD_26179	insulator	insulator
Be3P2_ICSD_187677	insulator	insulator
Be3P2_ICSD_616384	insulator	insulator
Be3Ru2_ICSD_616409	metal	metal
Be3Ta1_ICSD_616429	metal	metal
Be3Ti1_ICSD_616451	metal	metal
Be4C6K6O19_ICSD_412642	insulator	insulator
Be4Ca2N4_ICSD_413357	insulator	insulator
Be4H2O9Si2_ICSD_202360	insulator	insulator
Be4N4Sr2_ICSD_413356	insulator	insulator
Be4Na10O17Si4_ICSD_68750	insulator	insulator
Be4Na1O7Sb1_ICSD_27599	insulator	insulator
Be4O7Te1_ICSD_1322	insulator	insulator
Be5Co1_ICSD_616209	metal	metal
Be5Fe1_ICSD_616267	metal	metal
Be5Hf1_ICSD_58712	metal	metal
Be5Pd1_ICSD_616387	metal	metal
Be5Pt1_ICSD_616395	metal	metal
Be5Sc1_ICSD_58736	metal	metal
Be5Zr1_ICSD_58757	metal	metal
Be6Br2Li8O24P6_ICSD_80472	insulator	insulator
Be6Cd8O24S2Si6_ICSD_81485	insulator	insulator
Be6Cd8O24Se2Si6_ICSD_81486	insulator	insulator
Be6Cd8O24Si6Te2_ICSD_81487	insulator	insulator
Be6Fe8O24S2Si6_ICSD_201640	metal	metal
Be6Fe8O24Se2Si6_ICSD_97781	metal	metal
Be6Fe8O24Si6Te2_ICSD_97782	metal	metal
Be6Ge6Mn8O24S2_ICSD_83841	insulator	insulator
Be6Ge6Mn8O24Se2_ICSD_83842	insulator	insulator
Be6Mn8O24Se2Si6_ICSD_83839	insulator	insulator
Be6Mn8O24Si6Te2_ICSD_83840	insulator	insulator
Be6O24S2Si6Zn8_ICSD_201641	insulator	insulator
Bi0.67Nb1S2_ICSD_74700	metal	metal
Bi0.67S2Ta1_ICSD_108123	metal	metal
Bi12Cl12Pt1_ICSD_415739	insulator	insulator

Supplementary Table 165. Five-fold cross validated predictions for the metal/insulator classification (157/598).

system	calculated	predicted
Bi12I3Ni4_ICSD_405639	metal	metal
Bi12Mn1O20_ICSD_75079	metal	insulator
Bi12O20Si1_ICSD_422389	insulator	insulator
Bi12O20Zn1_ICSD_62479	metal	insulator
Bi14Br16Pd1_ICSD_412895	insulator	insulator
Bi14I12Ir1Si2_ICSD_410740	metal	insulator
Bi14I12Rh1Si2_ICSD_410739	metal	insulator
Bi16I4_ICSD_246145	metal	insulator
Bi1Br1Cd1S2_ICSD_171725	insulator	insulator
Bi1Br1Cd1Se2_ICSD_171726	insulator	insulator
Bi1Br1Cu3O8Se2_ICSD_280759	insulator	insulator
Bi1Br1Mn1S2_ICSD_415307	insulator	insulator
Bi1Br1O1_ICSD_61225	insulator	insulator
Bi1Br1O2Sr1_ICSD_97509	insulator	insulator
Bi1Br1S1_ICSD_25574	metal	metal
Bi1Br1S1_ICSD_31389	metal	metal
Bi1Br1_ICSD_1560	insulator	insulator
Bi1Br3_ICSD_100293	insulator	insulator
Bi1Br3_ICSD_100294	insulator	insulator
Bi1Br7Hg6Sb4_ICSD_411218	insulator	insulator
Bi1Br8O2W2_ICSD_424531	metal	insulator
Bi1C1Ca1F1O4_ICSD_87759	insulator	insulator
Bi1C3H6N3S6_ICSD_422349	insulator	insulator
Bi1C4N4Rb1S4_ICSD_164	insulator	insulator
Bi1C5H5K2O10_ICSD_109956	insulator	insulator
Bi1Ca1Cl1O2_ICSD_84635	insulator	insulator
Bi1Ca1Cu1_ICSD_57018	metal	metal
Bi1Ca1Li1_ICSD_616539	insulator	metal
Bi1Ca1O5V1_ICSD_73184	insulator	insulator
Bi1Ca2O6V1_ICSD_50939	insulator	insulator
Bi1Ca2_ICSD_42136	insulator	metal
Bi1Ca3N1_ICSD_106320	insulator	metal
Bi1Cd1Cl1O2_ICSD_280770	insulator	insulator
Bi1Cd1Cl1S2_ICSD_171724	insulator	insulator
Bi1Cd1I1Se2_ICSD_171727	insulator	insulator
Bi1Ce1O1S2_ICSD_80	insulator	insulator
Bi1Ce1Pd1_ICSD_161758	metal	insulator
Bi1Ce1Pd1_ICSD_616563	insulator	metal
Bi1Ce1Pt1_ICSD_616564	metal	metal
Bi1Ce1_ICSD_108105	metal	metal
Bi1Ce1_ICSD_187507	metal	metal
Bi1Ce1_ICSD_58765	metal	metal
Bi1Ce1_ICSD_58766	metal	metal
Bi1Ce1_ICSD_616547	metal	metal
Bi1Ce2O2_ICSD_419606	metal	metal
Bi1Cl1Cu3O8Se2_ICSD_54190	insulator	insulator
Bi1Cl1F8_ICSD_39555	insulator	insulator
Bi1Cl1Mn1S2_ICSD_172156	insulator	insulator
Bi1Cl1O1_ICSD_24608	insulator	insulator
Bi1Cl1O2Sr1_ICSD_84636	insulator	insulator

Supplementary Table 166. Five-fold cross validated predictions for the metal/insulator classification (158/598).

system	calculated	predicted
Bi1Cl1O3Se1_ICSD_411169	insulator	insulator
Bi1Cl1O3Se1_ICSD_98000	insulator	insulator
Bi1Cl1S1_ICSD_100173	insulator	insulator
Bi1Cl1Te1_ICSD_79362	insulator	insulator
Bi1Cl2Cu1S1_ICSD_413289	insulator	insulator
Bi1Cl2K1O4S1_ICSD_155203	insulator	insulator
Bi1Cl3O3Sr3_ICSD_80637	insulator	insulator
Bi1Cl3_ICSD_41179	insulator	insulator
Bi1Cl4N3S4_ICSD_9460	insulator	insulator
Bi1Cl6Cs1K2_ICSD_201983	insulator	insulator
Bi1Cl6Cs2Na1_ICSD_59195	insulator	insulator
Bi1Cl6Tl3_ICSD_421317	insulator	insulator
Bi1Cl8F4H3K6_ICSD_39524	insulator	insulator
Bi1Cl8O2W2_ICSD_424532	metal	insulator
Bi1Co1O3_ICSD_157833	insulator	insulator
Bi1Co1Zr1_ICSD_107120	insulator	metal
Bi1Co2Cr1_ICSD_167769	metal	metal
Bi1Co2Ho5_ICSD_161658	metal	metal
Bi1Cr1O3_ICSD_160454	insulator	insulator
Bi1Cr1O3_ICSD_160456	insulator	insulator
Bi1Cr2O8Rb1_ICSD_201624	insulator	insulator
Bi1Cs1F6_ICSD_15122	insulator	insulator
Bi1Cs1Ge1S4_ICSD_281168	insulator	insulator
Bi1Cs1O2_ICSD_406564	insulator	insulator
Bi1Cs1S2_ICSD_72975	insulator	insulator
Bi1Cs1S4Si1_ICSD_281169	insulator	insulator
Bi1Cs2F6K1_ICSD_9383	insulator	insulator
Bi1Cs2F6Na1_ICSD_9382	insulator	insulator
Bi1Cs2F6Rb1_ICSD_9384	insulator	insulator
Bi1Cs2F6Tl1_ICSD_9385	insulator	insulator
Bi1Cs3O3_ICSD_406563	insulator	insulator
Bi1Cs3Se3_ICSD_85410	insulator	insulator
Bi1Cs3_ICSD_58769	insulator	insulator
Bi1Cs3_ICSD_659568	insulator	insulator
Bi1Cu1Eu1_ICSD_58772	metal	metal
Bi1Cu1Mg1_ICSD_58774	metal	metal
Bi1Cu1O1Se1_ICSD_74475	insulator	metal
Bi1Cu1O1Te1_ICSD_159475	metal	insulator
Bi1Cu1O8W2_ICSD_67569	insulator	insulator
Bi1Cu1P2Se6_ICSD_170643	insulator	insulator
Bi1Cu1Pb1S3_ICSD_9120	insulator	insulator
Bi1Cu1Pt1S3_ICSD_180450	insulator	insulator
Bi1Cu1S2_ICSD_38779	insulator	insulator
Bi1Cu1Sr1_ICSD_58777	metal	metal
Bi1Cu1Yb1_ICSD_416142	metal	metal
Bi1Cu1Yb1_ICSD_416144	metal	metal
Bi1Cu1_ICSD_616577	metal	metal
Bi1Cu2O6P1_ICSD_75387	insulator	insulator
Bi1Cu3I1O8Se2_ICSD_54191	insulator	insulator
Bi1Cu3S3_ICSD_616615	insulator	insulator

Supplementary Table 167. Five-fold cross validated predictions for the metal/insulator classification (159/598).

system	calculated	predicted
Bi1Dy1Ni1_ICSD_58779	insulator	metal
Bi1Dy1Pd1_ICSD_616637	metal	metal
Bi1Dy1Pt1_ICSD_58780	metal	metal
Bi1Dy1Rh1_ICSD_51845	metal	metal
Bi1Dy1_ICSD_58778	metal	metal
Bi1Dy2O2_ICSD_423915	metal	metal
Bi1Er1Ge1O5_ICSD_96409	insulator	insulator
Bi1Er1Pt1_ICSD_58782	metal	metal
Bi1Er1_ICSD_656440	metal	metal
Bi1Er2O2_ICSD_422647	metal	metal
Bi1Er5Pt2_ICSD_107217	metal	metal
Bi1F1O1_ICSD_24096	insulator	insulator
Bi1F3_ICSD_1269	insulator	insulator
Bi1F3_ICSD_25567	insulator	insulator
Bi1F3_ICSD_655136	insulator	insulator
Bi1F4K1_ICSD_63166	insulator	insulator
Bi1F4Li1_ICSD_65404	insulator	insulator
Bi1F4Rb1_ICSD_63167	insulator	insulator
Bi1F5K2_ICSD_418777	insulator	insulator
Bi1F5_ICSD_25023	insulator	insulator
Bi1F6K1Rb2_ICSD_9387	insulator	insulator
Bi1F6K1_ICSD_25024	insulator	insulator
Bi1F6K1_ICSD_25025	insulator	insulator
Bi1F6Li1_ICSD_15119	insulator	insulator
Bi1F6Na1Rb2_ICSD_9386	insulator	insulator
Bi1F6Na1_ICSD_15120	insulator	insulator
Bi1F6Rb1_ICSD_15121	insulator	insulator
Bi1F7Kr1_ICSD_279626	insulator	insulator
Bi1Fe1O3_ICSD_162264	insulator	insulator
Bi1Fe1O3_ICSD_162834	metal	insulator
Bi1Fe1O3_ICSD_168320	insulator	insulator
Bi1Fe1O3_ICSD_168321	insulator	insulator
Bi1Fe1O3_ICSD_180498	insulator	insulator
Bi1Fe1O3_ICSD_188467	insulator	insulator
Bi1Fe1O3_ICSD_20288	metal	metal
Bi1Fe1O3_ICSD_20372	metal	insulator
Bi1Fe1O3_ICSD_22342	insulator	insulator
Bi1Fe2H1O9Si2_ICSD_200069	insulator	insulator
Bi1Ga1O3_ICSD_157550	insulator	insulator
Bi1Ga1O3_ICSD_171709	insulator	insulator
Bi1Ga1_ICSD_167768	metal	metal
Bi1Gd1Ni1_ICSD_58785	metal	metal
Bi1Gd1Pd1_ICSD_616671	metal	metal
Bi1Gd1Pt1_ICSD_58786	metal	metal
Bi1Gd1_ICSD_58783	metal	metal
Bi1Gd2O2_ICSD_261435	metal	metal
Bi1Ge1K1S4_ICSD_421486	insulator	insulator
Bi1Ge1Rb1S4_ICSD_281167	insulator	insulator
Bi1H1O12P4_ICSD_1967	insulator	insulator
Bi1H1O7Sr4_ICSD_419369	insulator	insulator

Supplementary Table 168. Five-fold cross validated predictions for the metal/insulator classification (160/598).

system	calculated	predicted
Bi1Hf2_ICSD_168286	metal	metal
Bi1Ho1Ni1_ICSD_616692	metal	insulator
Bi1Ho1Pd1_ICSD_616693	metal	metal
Bi1Ho1Pt1_ICSD_58788	metal	metal
Bi1Ho1_ICSD_187366	metal	metal
Bi1Ho1_ICSD_43545	metal	metal
Bi1Ho2O2_ICSD_423914	metal	metal
Bi1I1Mn1Se2_ICSD_415138	insulator	insulator
Bi1I1O1_ICSD_391354	insulator	insulator
Bi1I1O2Sr1_ICSD_97510	insulator	insulator
Bi1I1O3Te1_ICSD_56218	insulator	insulator
Bi1I1O3Te1_ICSD_92318	metal	insulator
Bi1I1O4_ICSD_262019	insulator	insulator
Bi1I1S1_ICSD_23631	metal	insulator
Bi1I1S1_ICSD_25575	insulator	insulator
Bi1I1Se1_ICSD_280311	insulator	insulator
Bi1I1Te1_ICSD_263109	insulator	insulator
Bi1I1_ICSD_1558	insulator	insulator
Bi1I1_ICSD_1559	insulator	insulator
Bi1I3_ICSD_187608	insulator	insulator
Bi1I3_ICSD_187610	metal	insulator
Bi1I3_ICSD_187611	insulator	insulator
Bi1I3_ICSD_20676	insulator	insulator
Bi1I3_ICSD_56570	insulator	insulator
Bi1I6Tl3_ICSD_417537	insulator	insulator
Bi1In1O3_ICSD_158758	insulator	insulator
Bi1In1O3_ICSD_171756	insulator	insulator
Bi1In1O3_ICSD_171757	insulator	insulator
Bi1In1S3_ICSD_290195	insulator	insulator
Bi1In1_ICSD_184573	metal	metal
Bi1In1_ICSD_616707	metal	metal
Bi1In2_ICSD_108108	metal	metal
Bi1In2_ICSD_1247	metal	metal
Bi1Ir1S1_ICSD_616740	insulator	insulator
Bi1Ir1Se1_ICSD_616741	insulator	insulator
Bi1K1Li6O6_ICSD_71035	insulator	insulator
Bi1K1Mg1_ICSD_616748	insulator	insulator
Bi1K1Mn1_ICSD_601586	metal	metal
Bi1K1O2_ICSD_407209	insulator	insulator
Bi1K1O3_ICSD_76976	insulator	insulator
Bi1K1O8W2_ICSD_391361	insulator	insulator
Bi1K1P2S6_ICSD_409686	insulator	insulator
Bi1K1P2Se6_ICSD_90153	insulator	insulator
Bi1K1S4Si1_ICSD_421485	insulator	insulator
Bi1K1_ICSD_55065	insulator	insulator
Bi1K2Rb1Se3_ICSD_85412	insulator	insulator
Bi1K2Sn1_ICSD_107616	metal	insulator
Bi1K3O3_ICSD_407293	insulator	insulator
Bi1K3P2S8_ICSD_81772	insulator	insulator
Bi1K3Se3_ICSD_78841	insulator	insulator

Supplementary Table 169. Five-fold cross validated predictions for the metal/insulator classification (161/598).

system	calculated	predicted
Bi1K3Te3_ICSD_300183	insulator	insulator
Bi1K3_ICSD_26885	metal	metal
Bi1K3_ICSD_409223	metal	metal
Bi1K3_ICSD_58793	metal	metal
Bi1K9O24U6_ICSD_66528	insulator	insulator
Bi1La1_ICSD_616762	metal	metal
Bi1La2O2_ICSD_422645	metal	metal
Bi1Li1Mg1_ICSD_108112	insulator	metal
Bi1Li1O2_ICSD_25385	insulator	insulator
Bi1Li1O3_ICSD_82277	metal	insulator
Bi1Li1O4Pd2_ICSD_202930	insulator	insulator
Bi1Li1Sr1_ICSD_58800	insulator	insulator
Bi1Li1Yb1_ICSD_602201	insulator	metal
Bi1Li1Zn1_ICSD_100115	metal	metal
Bi1Li1_ICSD_58796	metal	metal
Bi1Li3O4_ICSD_109087	insulator	insulator
Bi1Li3_ICSD_58797	insulator	metal
Bi1Li5O5_ICSD_203031	insulator	insulator
Bi1Lu1Ni1_ICSD_58802	metal	metal
Bi1Lu1O6Sr2_ICSD_88149	metal	insulator
Bi1Lu1_ICSD_58801	metal	metal
Bi1Mg1Ni1_ICSD_76253	metal	metal
Bi1Mg2O6P1_ICSD_73894	insulator	insulator
Bi1Mg2O6V1_ICSD_72175	insulator	insulator
Bi1Mn1Na1_ICSD_601587	metal	metal
Bi1Mn1O3_ICSD_245299	insulator	insulator
Bi1Mn1O3_ICSD_245300	insulator	insulator
Bi1Mn1O3_ICSD_50795	insulator	insulator
Bi1Mn1O5V1_ICSD_59720	insulator	insulator
Bi1Mn1Rb1_ICSD_616824	metal	metal
Bi1Mn1_ICSD_58805	metal	metal
Bi1Mn2O5_ICSD_169736	insulator	insulator
Bi1Mn2O6P1_ICSD_59673	insulator	insulator
Bi1N1Sr3_ICSD_152053	insulator	metal
Bi1N2Th2_ICSD_16064	metal	metal
Bi1N2U2_ICSD_16061	metal	metal
Bi1Na1O2_ICSD_10317	insulator	insulator
Bi1Na1O3_ICSD_27553	insulator	insulator
Bi1Na1O6Sr3_ICSD_419367	insulator	insulator
Bi1Na1_ICSD_58816	metal	metal
Bi1Na3O3_ICSD_23347	insulator	insulator
Bi1Na3O4_ICSD_10319	insulator	insulator
Bi1Na3_ICSD_26881	metal	insulator
Bi1Nb1O4_ICSD_415850	insulator	insulator
Bi1Nb1O4_ICSD_74338	insulator	insulator
Bi1Nb3_ICSD_58817	metal	metal
Bi1Nd1O6Sr2_ICSD_68528	metal	insulator
Bi1Nd1Pd1_ICSD_616867	metal	metal
Bi1Nd1_ICSD_44133	metal	metal
Bi1Nd2O2_ICSD_422646	metal	metal

Supplementary Table 170. Five-fold cross validated predictions for the metal/insulator classification (162/598).

system	calculated	predicted
Bi1Ni1Sc1_ICSD_58824	metal	metal
Bi1Ni1Se1_ICSD_616878	metal	metal
Bi1Ni1Tm1_ICSD_58825	metal	metal
Bi1Ni1Y1_ICSD_58826	metal	metal
Bi1Ni1Zr1_ICSD_107121	metal	metal
Bi1Ni1_ICSD_58820	metal	metal
Bi1Ni1_ICSD_616868	metal	metal
Bi1Ni9S8Te1_ICSD_107586	metal	metal
Bi1O1_ICSD_30361	metal	metal
Bi1O2Pr2_ICSD_419609	metal	metal
Bi1O2Rb1_ICSD_407208	insulator	insulator
Bi1O2Tb2_ICSD_423916	metal	metal
Bi1O2Y2_ICSD_261432	metal	metal
Bi1O2_ICSD_52731	metal	metal
Bi1O3Rb3_ICSD_407294	insulator	insulator
Bi1O3Rh1_ICSD_188150	insulator	insulator
Bi1O3Sc1_ICSD_171384	insulator	insulator
Bi1O3Sc1_ICSD_171385	insulator	insulator
Bi1O3Sc1_ICSD_181115	insulator	insulator
Bi1O3Sr1_ICSD_85173	insulator	insulator
Bi1O4P1_ICSD_189659	insulator	insulator
Bi1O4P1_ICSD_27469	insulator	insulator
Bi1O4Re1_ICSD_10481	metal	insulator
Bi1O4Sb1_ICSD_80821	insulator	insulator
Bi1O4Ta1_ICSD_415849	insulator	insulator
Bi1O4Ta1_ICSD_97423	insulator	insulator
Bi1O4V1_ICSD_100601	insulator	insulator
Bi1O4V1_ICSD_100733	insulator	insulator
Bi1O5P1Pb1_ICSD_419125	insulator	insulator
Bi1O5Pb1V1_ICSD_419123	insulator	insulator
Bi1O5Pb1V1_ICSD_419126	insulator	insulator
Bi1O6P1Zn2_ICSD_91234	insulator	insulator
Bi1O8P1Pb4_ICSD_50649	insulator	insulator
Bi1Os1Se1_ICSD_616892	insulator	insulator
Bi1P2S6Tl1_ICSD_249461	insulator	insulator
Bi1Pb1Pd2_ICSD_56278	metal	metal
Bi1Pb1Pd2_ICSD_58830	metal	metal
Bi1Pb3Pt1_ICSD_58834	metal	metal
Bi1Pd1Se1_ICSD_616956	metal	metal
Bi1Pd1Te1_ICSD_616961	metal	metal
Bi1Pd1Y1_ICSD_616964	metal	metal
Bi1Pd1Yb1_ICSD_58843	metal	metal
Bi1Pd1_ICSD_108171	metal	metal
Bi1Pd1_ICSD_54976	metal	metal
Bi1Pd1_ICSD_56279	metal	metal
Bi1Pd2Y1_ICSD_58842	metal	metal
Bi1Pd3_ICSD_58839	metal	metal
Bi1Pr1_ICSD_187510	metal	metal
Bi1Pr1_ICSD_187511	metal	metal
Bi1Pr1_ICSD_58844	metal	metal

Supplementary Table 171. Five-fold cross validated predictions for the metal/insulator classification (163/598).

system	calculated	predicted
Bi1Pt1Se1_ICSD_616991	metal	metal
Bi1Pt1Te1_ICSD_42549	metal	metal
Bi1Pt1Yb1_ICSD_246662	metal	metal
Bi1Pt1_ICSD_58845	metal	metal
Bi1Pt1_ICSD_616981	metal	metal
Bi1Pu1_ICSD_616994	metal	metal
Bi1Rb1S2_ICSD_52735	insulator	insulator
Bi1Rb1S4Si1_ICSD_281166	insulator	insulator
Bi1Rb1_ICSD_55066	insulator	insulator
Bi1Rb3Se3_ICSD_85411	insulator	insulator
Bi1Rb3_ICSD_616996	metal	metal
Bi1Rb3_ICSD_616997	metal	metal
Bi1Rh1S1_ICSD_617013	insulator	insulator
Bi1Rh1Se1_ICSD_617014	insulator	insulator
Bi1Rh1Sm1_ICSD_51844	metal	metal
Bi1Rh1_ICSD_185668	metal	metal
Bi1Rh1_ICSD_58852	metal	metal
Bi1S2Tl1_ICSD_172572	insulator	insulator
Bi1Sb1U1_ICSD_617069	metal	metal
Bi1Sc1_ICSD_58856	metal	metal
Bi1Se1_ICSD_44982	metal	metal
Bi1Se2Tl1_ICSD_43314	insulator	insulator
Bi1Sm1_ICSD_44129	metal	metal
Bi1Sm1_ICSD_617126	metal	metal
Bi1Sn1_ICSD_160383	metal	metal
Bi1Sr2_ICSD_41836	metal	metal
Bi1Tb1_ICSD_617166	metal	metal
Bi1Te1Th1_ICSD_617196	metal	metal
Bi1Te1U1_ICSD_617210	metal	metal
Bi1Te1_ICSD_100654	metal	insulator
Bi1Te1_ICSD_44984	metal	metal
Bi1Te2Tl1_ICSD_15412	insulator	metal
Bi1Th1_ICSD_58860	metal	metal
Bi1Ti2_ICSD_617225	metal	metal
Bi1Tl1_ICSD_53967	metal	metal
Bi1Tm1_ICSD_58865	metal	metal
Bi1Tm1_ICSD_617231	metal	metal
Bi1U1_ICSD_165127	metal	metal
Bi1U1_ICSD_58866	metal	metal
Bi1Y1_ICSD_58869	metal	metal
Bi1Zr1_ICSD_409756	metal	metal
Bi1_ICSD_187502	metal	metal
Bi1_ICSD_189806	metal	metal
Bi1_ICSD_409752	metal	metal
Bi1_ICSD_43033	metal	metal
Bi1_ICSD_51674	metal	metal
Bi1_ICSD_51675	metal	metal
Bi1_ICSD_52724	metal	metal
Bi1_ICSD_52725	metal	metal
Bi1_ICSD_616526	metal	metal

Supplementary Table 172. Five-fold cross validated predictions for the metal/insulator classification (164/598).

system	calculated	predicted
Bi1_ICSD_616528	metal	metal
Bi24Ge2O40_ICSD_68431	insulator	insulator
Bi24O40Pb2_ICSD_75392	insulator	insulator
Bi24O40Ti2_ICSD_75389	insulator	insulator
Bi2Br1Dy1O4_ICSD_92417	insulator	insulator
Bi2Br1Er1O4_ICSD_92420	insulator	insulator
Bi2Br1Eu1O4_ICSD_92414	insulator	insulator
Bi2Br1Gd1O4_ICSD_92415	insulator	insulator
Bi2Br1Ho1O4_ICSD_92419	insulator	insulator
Bi2Br1In1Se4_ICSD_159465	insulator	insulator
Bi2Br1Lu1O4_ICSD_92423	insulator	insulator
Bi2Br1Nd1O4_ICSD_92412	insulator	insulator
Bi2Br1O4Pr1_ICSD_92411	insulator	insulator
Bi2Br1O4Tb1_ICSD_92416	insulator	insulator
Bi2Br1O4Y1_ICSD_92418	insulator	insulator
Bi2Br1O4Yb1_ICSD_92422	metal	insulator
Bi2Br8Te4_ICSD_83806	insulator	insulator
Bi2Br9Cs3_ICSD_1142	insulator	insulator
Bi2Br9Cs3_ICSD_96723	insulator	insulator
Bi2C1O5_ICSD_94740	insulator	insulator
Bi2C2Ca1O8_ICSD_94741	insulator	insulator
Bi2Ca1Mg2_ICSD_261988	insulator	metal
Bi2Ca1Mn2_ICSD_41791	metal	metal
Bi2Ca1O9Ta2_ICSD_20667	metal	insulator
Bi2Ca1_ICSD_659277	metal	metal
Bi2Ca4O1_ICSD_416137	insulator	insulator
Bi2Cd1Cs2S5_ICSD_97427	insulator	insulator
Bi2Cd1Ge1O6_ICSD_82157	insulator	insulator
Bi2Ce1Ni2_ICSD_616562	metal	metal
Bi2Cl1Dy1O4_ICSD_92404	insulator	insulator
Bi2Cl1Er1O4_ICSD_92407	insulator	insulator
Bi2Cl1Eu1O4_ICSD_92401	insulator	insulator
Bi2Cl1Gd1O4_ICSD_92402	insulator	insulator
Bi2Cl1Ho1O4_ICSD_92406	insulator	insulator
Bi2Cl1In1S4_ICSD_484	insulator	insulator
Bi2Cl1Lu1O4_ICSD_92410	insulator	insulator
Bi2Cl1Nd1O4_ICSD_92399	insulator	insulator
Bi2Cl1O4Pr1_ICSD_92398	insulator	insulator
Bi2Cl1O4Tb1_ICSD_92403	insulator	insulator
Bi2Cl1O4Y1_ICSD_92405	insulator	insulator
Bi2Cl1O4Yb1_ICSD_92409	insulator	metal
Bi2Co1Hf6_ICSD_54566	metal	metal
Bi2Co1Ho6_ICSD_240170	metal	metal
Bi2Co1Mn1O6_ICSD_153856	insulator	insulator
Bi2Co1O7S1_ICSD_65135	insulator	insulator
Bi2Co1Tm6_ICSD_240171	metal	metal
Bi2Co1Y6_ICSD_240169	metal	metal
Bi2Cr1Fe1O6_ICSD_246426	insulator	insulator
Bi2Cs1Cu1S4_ICSD_93370	insulator	insulator
Bi2Cs1_ICSD_58771	metal	metal

Supplementary Table 173. Five-fold cross validated predictions for the metal/insulator classification (165/598).

system	calculated	predicted
Bi2Cs2Pd1_ICSD_658703	insulator	insulator
Bi2Cs2Pt1_ICSD_658701	insulator	insulator
Bi2Cs3I9_ICSD_1448	insulator	insulator
Bi2Cs3I9_ICSD_411633	insulator	insulator
Bi2Cu1K1S4_ICSD_91297	insulator	insulator
Bi2Cu1O12Se4_ICSD_66826	insulator	insulator
Bi2Cu1O4_ICSD_12104	insulator	insulator
Bi2Cu1O4_ICSD_68812	insulator	insulator
Bi2Cu1U1_ICSD_656844	metal	metal
Bi2Cu1Zr6_ICSD_182405	metal	metal
Bi2Dy1I1O4_ICSD_92431	insulator	insulator
Bi2Dy1Ni2_ICSD_616635	metal	metal
Bi2Er1I1O4_ICSD_89618	insulator	insulator
Bi2Er6Mn1_ICSD_159231	metal	metal
Bi2Eu1I1O4_ICSD_92428	insulator	insulator
Bi2Eu1Pd2_ICSD_604364	metal	metal
Bi2Eu1S4_ICSD_600801	metal	insulator
Bi2Eu1Se4_ICSD_600805	insulator	metal
Bi2Eu1_ICSD_659278	metal	metal
Bi2Fe1Ho6_ICSD_182408	metal	metal
Bi2Fe1Ho6_ICSD_96253	metal	metal
Bi2Fe1O6Ti1_ICSD_162263	metal	insulator
Bi2Fe1Tb6_ICSD_152647	metal	metal
Bi2Fe1Tb6_ICSD_152648	metal	metal
Bi2Fe4O9_ICSD_26808	insulator	insulator
Bi2Ga2In4S12_ICSD_410032	insulator	insulator
Bi2Ga4O9_ICSD_248245	insulator	insulator
Bi2Ga4S8_ICSD_408441	insulator	insulator
Bi2Ga4Se8_ICSD_408442	insulator	insulator
Bi2Gd1I1O4_ICSD_92429	metal	insulator
Bi2Ge1O5_ICSD_62488	insulator	insulator
Bi2Ge1O5_ICSD_65522	insulator	insulator
Bi2Ge1O5_ICSD_94334	insulator	insulator
Bi2Ge1Te4_ICSD_658633	insulator	insulator
Bi2Ge3O9_ICSD_100277	insulator	insulator
Bi2Ge3Te6_ICSD_16207	metal	insulator
Bi2H16Na6O14_ICSD_412583	insulator	insulator
Bi2Hf1_ICSD_616683	metal	metal
Bi2Hg1S4_ICSD_14189	insulator	insulator
Bi2Ho1I1O4_ICSD_92433	insulator	insulator
Bi2Ho5Pd1_ICSD_180046	metal	metal
Bi2Ho6Mn1_ICSD_159211	metal	metal
Bi2Ho6Rh1_ICSD_180044	metal	metal
Bi2I1La1O4_ICSD_92424	insulator	insulator
Bi2I1Lu1O4_ICSD_92437	insulator	insulator
Bi2I1Nd1O4_ICSD_92426	insulator	insulator
Bi2I1O4Pr1_ICSD_92425	insulator	insulator
Bi2I1O4Tb1_ICSD_92430	insulator	insulator
Bi2I1O4Y1_ICSD_92432	insulator	insulator
Bi2I1O4Yb1_ICSD_92436	metal	metal

Supplementary Table 174. Five-fold cross validated predictions for the metal/insulator classification (166/598).

system	calculated	predicted
Bi2I1Te1_ICSD_153858	metal	insulator
Bi2In4S9_ICSD_2839	insulator	insulator
Bi2Ir1_ICSD_616739	metal	metal
Bi2Ir2O7_ICSD_161103	metal	metal
Bi2K1_ICSD_55068	metal	metal
Bi2K3O12P3_ICSD_409582	insulator	insulator
Bi2K4O5_ICSD_408000	insulator	insulator
Bi2La1Li1_ICSD_415728	metal	metal
Bi2La1Li3_ICSD_616769	metal	metal
Bi2La1Ni2_ICSD_616770	metal	metal
Bi2Li3Y1_ICSD_616795	metal	metal
Bi2Li8O10Pd1_ICSD_73000	insulator	insulator
Bi2Mg1O6_ICSD_50005	metal	insulator
Bi2Mg2Sr1_ICSD_616807	insulator	insulator
Bi2Mg2Yb1_ICSD_261990	metal	metal
Bi2Mg3_ICSD_659569	metal	metal
Bi2Mn1Ni1O6_ICSD_159285	insulator	insulator
Bi2Mn1Ti4_ICSD_150145	metal	metal
Bi2Mo1O6_ICSD_14266	insulator	insulator
Bi2Mo1O6_ICSD_17070	insulator	insulator
Bi2Mo1O6_ICSD_37251	insulator	insulator
Bi2Na1O11Sb3_ICSD_79859	insulator	insulator
Bi2Nb2O9Pb1_ICSD_20668	insulator	insulator
Bi2Nd1Ni2_ICSD_616866	metal	metal
Bi2Ni1Ti4_ICSD_150148	metal	metal
Bi2Ni1U1_ICSD_656843	metal	metal
Bi2Ni2Pr1_ICSD_616870	metal	metal
Bi2Ni2Tb1_ICSD_616881	metal	metal
Bi2Ni3S2_ICSD_159360	metal	metal
Bi2Ni3S2_ICSD_159364	metal	metal
Bi2Ni3Se2_ICSD_616879	metal	metal
Bi2O10Pb2V2_ICSD_60577	insulator	insulator
Bi2O10Te2W1_ICSD_78917	insulator	insulator
Bi2O11Ti4_ICSD_79769	insulator	insulator
Bi2O13P4_ICSD_65133	insulator	insulator
Bi2O2S1_ICSD_29451	insulator	insulator
Bi2O2Se1_ICSD_2903	metal	insulator
Bi2O2Se1_ICSD_411143	insulator	metal
Bi2O3_ICSD_168807	metal	insulator
Bi2O3_ICSD_168808	insulator	insulator
Bi2O3_ICSD_168810	insulator	insulator
Bi2O3_ICSD_168811	insulator	insulator
Bi2O3_ICSD_168813	insulator	insulator
Bi2O3_ICSD_168814	insulator	insulator
Bi2O3_ICSD_168815	insulator	insulator
Bi2O3_ICSD_169686	insulator	insulator
Bi2O3_ICSD_186365	insulator	insulator
Bi2O3_ICSD_261777	insulator	insulator
Bi2O3_ICSD_27152	insulator	insulator
Bi2O3_ICSD_37367	metal	metal

Supplementary Table 175. Five-fold cross validated predictions for the metal/insulator classification (167/598).

system	calculated	predicted
Bi2O3_ICSD_421855	insulator	insulator
Bi2O3_ICSD_616890	insulator	insulator
Bi2O3_ICSD_62979	insulator	insulator
Bi2O4Pd1_ICSD_200145	insulator	insulator
Bi2O4Pd1_ICSD_9622	insulator	insulator
Bi2O4Sr1_ICSD_80668	insulator	insulator
Bi2O4_ICSD_79500	insulator	insulator
Bi2O5Se1_ICSD_409518	insulator	insulator
Bi2O5Si1_ICSD_30995	insulator	insulator
Bi2O5Sr2_ICSD_86415	insulator	insulator
Bi2O5Te1_ICSD_36446	insulator	insulator
Bi2O6Te1_ICSD_6239	insulator	insulator
Bi2O6Ti1Zn1_ICSD_162767	insulator	insulator
Bi2O6Ti1Zn1_ICSD_162768	insulator	insulator
Bi2O6Ti1Zn1_ICSD_186800	metal	insulator
Bi2O6Ti1Zn1_ICSD_186802	insulator	insulator
Bi2O6Ti1Zn1_ICSD_186803	insulator	insulator
Bi2O6U1_ICSD_1805	insulator	insulator
Bi2O6U1_ICSD_1806	insulator	insulator
Bi2O6W1_ICSD_23584	insulator	insulator
Bi2O6W1_ICSD_290469	metal	insulator
Bi2O6W1_ICSD_66579	insulator	insulator
Bi2O6W1_ICSD_67647	insulator	insulator
Bi2O7Os2_ICSD_161105	metal	metal
Bi2O7Pt2_ICSD_161104	insulator	metal
Bi2O7Rh2_ICSD_161099	metal	metal
Bi2O7Ru2_ICSD_166567	metal	metal
Bi2O7Sn2_ICSD_50311	insulator	metal
Bi2O7Ti2_ICSD_180394	insulator	insulator
Bi2O9Si3_ICSD_155234	insulator	insulator
Bi2O9W2_ICSD_88428	insulator	insulator
Bi2Pb1S4_ICSD_31859	insulator	insulator
Bi2Pb1Te4_ICSD_616936	insulator	insulator
Bi2Pb2Se5_ICSD_30372	insulator	insulator
Bi2Pb2Te5_ICSD_42708	metal	insulator
Bi2Pb3S6_ICSD_92981	insulator	insulator
Bi2Pd1_ICSD_56280	metal	metal
Bi2Pd1_ICSD_616946	metal	metal
Bi2Pd2Sr1_ICSD_416300	metal	metal
Bi2Pd2Sr1_ICSD_604338	metal	metal
Bi2Pd3S2_ICSD_159362	metal	metal
Bi2Pd3S2_ICSD_159366	metal	metal
Bi2Pd3S2_ICSD_616954	metal	metal
Bi2Pd3Se2_ICSD_261458	metal	metal
Bi2Pd5_ICSD_58840	metal	metal
Bi2Pt1_ICSD_58846	metal	metal
Bi2Pt1_ICSD_58847	metal	metal
Bi2Pt1_ICSD_616982	metal	metal
Bi2Rb1_ICSD_58850	metal	metal
Bi2Rh1_ICSD_43503	metal	metal

Supplementary Table 176. Five-fold cross validated predictions for the metal/insulator classification (168/598).

system	calculated	predicted
Bi2Rh3S2_ICSD_420725	metal	metal
Bi2Rh3Si2_ICSD_617015	metal	metal
Bi2S1Te2_ICSD_26720	insulator	metal
Bi2S3_ICSD_617021	insulator	insulator
Bi2S4Yb1_ICSD_600812	insulator	insulator
Bi2Se1Te2_ICSD_43512	metal	insulator
Bi2Se2Te1_ICSD_54838	insulator	insulator
Bi2Se2_ICSD_20458	metal	insulator
Bi2Se3_ICSD_171571	insulator	insulator
Bi2Se3_ICSD_617083	insulator	metal
Bi2Se3_ICSD_617096	insulator	insulator
Bi2Te3_ICSD_20289	metal	insulator
Bi2Te3_ICSD_617187	insulator	metal
Bi2Th1_ICSD_617224	metal	metal
Bi2Tl1_ICSD_617229	metal	metal
Bi2U1_ICSD_617241	metal	metal
Bi2Yb1_ICSD_617251	metal	metal
Bi2Zr1_ICSD_42880	metal	metal
Bi3Br1La5_ICSD_95241	metal	metal
Bi3Br1Se4_ICSD_411096	insulator	insulator
Bi3Ce4_ICSD_616553	metal	metal
Bi3Cl5O10Te4_ICSD_168978	insulator	insulator
Bi3Co1_ICSD_236383	metal	metal
Bi3Cs1Se5_ICSD_171610	insulator	insulator
Bi3Er5_ICSD_107266	metal	metal
Bi3Eu4_ICSD_616649	metal	metal
Bi3Eu5_ICSD_173031	metal	metal
Bi3F6N1_ICSD_79395	insulator	insulator
Bi3Fe1Mo2O12_ICSD_45	insulator	insulator
Bi3Gd4_ICSD_616662	metal	metal
Bi3Ho5_ICSD_107265	metal	metal
Bi3In4Pb1_ICSD_616725	metal	metal
Bi3In5_ICSD_1246	metal	metal
Bi3La4_ICSD_616759	metal	metal
Bi3Mn4N1O15_ICSD_260393	insulator	insulator
Bi3Na1O10V2_ICSD_88455	insulator	insulator
Bi3Nd4_ICSD_616855	metal	metal
Bi3Nd4_ICSD_616860	metal	metal
Bi3Ni1_ICSD_391336	metal	metal
Bi3O11Pt3_ICSD_108966	metal	insulator
Bi3O11Ru3_ICSD_4194	metal	insulator
Bi3O7Sb1_ICSD_51779	insulator	insulator
Bi3O8Re1_ICSD_185903	insulator	insulator
Bi3O8Re1_ICSD_185904	insulator	insulator
Bi3Pr4_ICSD_616969	metal	metal
Bi3Rb1S5_ICSD_654071	metal	insulator
Bi3Rh1_ICSD_58853	metal	metal
Bi3S1Te2_ICSD_107587	metal	insulator
Bi3Sc5_ICSD_107590	metal	metal
Bi3Se4_ICSD_20386	metal	insulator

Supplementary Table 177. Five-fold cross validated predictions for the metal/insulator classification (169/598).

system	calculated	predicted
Bi3Sm4_ICSD_617132	metal	metal
Bi3Sr1_ICSD_617156	metal	metal
Bi3Sr2_ICSD_164987	metal	metal
Bi3Sr4_ICSD_96944	metal	metal
Bi3Sr5_ICSD_617154	metal	metal
Bi3Tb4_ICSD_617161	metal	metal
Bi3Tb5_ICSD_617164	metal	metal
Bi3Th5_ICSD_617223	metal	metal
Bi3Yb4_ICSD_617248	metal	metal
Bi3Yb4_ICSD_617255	metal	metal
Bi4Br2O5_ICSD_412591	insulator	insulator
Bi4Br2O9Te2_ICSD_79508	insulator	insulator
Bi4Ce3Pd3_ICSD_419162	insulator	metal
Bi4Ce3Pt3_ICSD_616565	insulator	metal
Bi4Cl14Se4_ICSD_410910	insulator	insulator
Bi4Cl16Te14_ICSD_391157	insulator	insulator
Bi4Cl1O8Ta1_ICSD_59601	insulator	insulator
Bi4Cu3La3_ICSD_167250	insulator	metal
Bi4Cu3O14V2_ICSD_75222	metal	insulator
Bi4Cu4Mn3_ICSD_616588	metal	metal
Bi4Ge1Te7_ICSD_42891	insulator	insulator
Bi4Ge3O12_ICSD_108872	insulator	insulator
Bi4I2O5_ICSD_412590	insulator	insulator
Bi4I2Ru1_ICSD_406949	insulator	insulator
Bi4La3Pt3_ICSD_616774	metal	insulator
Bi4Li1Nb3O14_ICSD_391261	insulator	insulator
Bi4Mn5Ni2_ICSD_58812	metal	metal
Bi4O11Ta2_ICSD_50601	insulator	insulator
Bi4O12Si3_ICSD_69430	insulator	insulator
Bi4O12Ti3_ICSD_24735	metal	insulator
Bi4O7_ICSD_51778	insulator	insulator
Bi4Pb1Te7_ICSD_250249	insulator	insulator
Bi4Rh1_ICSD_58854	metal	metal
Bi4Se3_ICSD_617074	metal	metal
Bi4Sn1Te7_ICSD_236253	insulator	insulator
Bi4Te3_ICSD_30526	insulator	metal
Bi4Th3_ICSD_58862	metal	metal
Bi4U3_ICSD_58868	metal	metal
Bi5Cl12Ga3_ICSD_414089	insulator	insulator
Bi5Cu4S10_ICSD_1842	metal	metal
Bi5I1O7_ICSD_30978	insulator	insulator
Bi5La3Mg1_ICSD_415727	metal	metal
Bi5La3Sc1_ICSD_416609	metal	metal
Bi5Mn1Pr3_ICSD_173359	metal	metal
Bi6Cu2Pb2S12_ICSD_160417	insulator	insulator
Bi6In2Sr5_ICSD_415576	metal	metal
Bi6Se13Sr4_ICSD_62782	insulator	insulator
Bi7Cs3Se12_ICSD_61785	insulator	insulator
Bi7Cu4S12_ICSD_64709	metal	insulator
Bi7F11O5_ICSD_167074	insulator	insulator

Supplementary Table 178. Five-fold cross validated predictions for the metal/insulator classification (170/598).

system	calculated	predicted
Bi7O18Ta3_ICSD_280093	insulator	insulator
Bi8K2Se13_ICSD_72976	insulator	insulator
Bi9Ca9Cd4_ICSD_58760	metal	metal
Bi9Ca9Zn4_ICSD_8158	metal	metal
Bi9I3Rh2_ICSD_411136	insulator	insulator
Bi9Mn4Yb9_ICSD_166888	metal	metal
Br1.6Cl0.4Sr1_ICSD_35052	insulator	insulator
Br10Co1Er6_ICSD_424427	insulator	insulator
Br10Co1Tb6_ICSD_424433	metal	insulator
Br10Co1Y6_ICSD_424465	insulator	insulator
Br10Gd6Ir1_ICSD_424456	insulator	insulator
Br10Ir1Tb6_ICSD_424434	insulator	metal
Br10Ni1Tb6_ICSD_424435	insulator	insulator
Br10Ni1Y6_ICSD_424466	insulator	insulator
Br10O1Ta2Te4_ICSD_401906	insulator	insulator
Br10O4Pb9_ICSD_35381	insulator	insulator
Br10Pd1Y6_ICSD_424467	insulator	insulator
Br10Rb1Re3_ICSD_33911	insulator	insulator
Br10Re2Te6_ICSD_410190	insulator	insulator
Br10Ru1Tb6_ICSD_424436	metal	insulator
Br11Cs1Nb4_ICSD_26077	insulator	insulator
Br12C1Sc7_ICSD_61266	metal	insulator
Br12Co1Ho7_ICSD_424451	insulator	metal
Br12Co1Sc7_ICSD_424474	insulator	insulator
Br12Fe1Tb7_ICSD_424440	metal	insulator
Br12Os1Se2_ICSD_182315	insulator	insulator
Br12Ta2Te4_ICSD_401905	insulator	insulator
Br12Zr6_ICSD_41540	metal	insulator
Br14Cd1W6_ICSD_80887	insulator	insulator
Br14Mo6Pb1_ICSD_36573	insulator	insulator
Br14Rb2W6_ICSD_390028	insulator	insulator
Br14Ta6_ICSD_402031	insulator	insulator
Br15Co1Th6_ICSD_33926	insulator	metal
Br15Fe1Th6_ICSD_33925	metal	insulator
Br16Re4Te8_ICSD_78924	insulator	insulator
Br17Nb6Rb3S1_ICSD_400496	insulator	insulator
Br18Cs1Er1Ta6_ICSD_77666	insulator	insulator
Br18K4Nb6_ICSD_49687	insulator	insulator
Br18Nb6Tl4_ICSD_402033	insulator	insulator
Br1C1Cl1F1H3_ICSD_424851	insulator	insulator
Br1C1Cu1H2N2_ICSD_420807	insulator	insulator
Br1C1Gd2_ICSD_40561	metal	metal
Br1C1K1N2O4_ICSD_42826	insulator	insulator
Br1C1Pr2_ICSD_420348	metal	metal
Br1C2H8N1_ICSD_110459	insulator	insulator
Br1C2H8N1_ICSD_400651	insulator	insulator
Br1C2N2Ni1Rb1_ICSD_380470	insulator	insulator
Br1C3H10N1_ICSD_171150	insulator	insulator
Br1C4N3_ICSD_246789	insulator	insulator
Br1C5Mn1O5_ICSD_281349	insulator	insulator

Supplementary Table 179. Five-fold cross validated predictions for the metal/insulator classification (171/598).

system	calculated	predicted
Br1C5O5Re1_ICSD_66697	insulator	insulator
Br1C5O5Tc1_ICSD_167773	insulator	insulator
Br1C8H20Li1O4_ICSD_151064	insulator	insulator
Br1Ca1H1_ICSD_25542	insulator	insulator
Br1Ca2H3_ICSD_420928	insulator	insulator
Br1Ca2N1_ICSD_153105	insulator	insulator
Br1Cd1S2Sb1_ICSD_171723	insulator	insulator
Br1Cd2P3_ICSD_100817	insulator	insulator
Br1Cd2P3_ICSD_100880	insulator	insulator
Br1Ce2S5Sb1_ICSD_51484	insulator	insulator
Br1Ce3S8Si2_ICSD_39052	insulator	insulator
Br1Cl14P3_ICSD_80214	insulator	insulator
Br1Cl1_ICSD_424850	insulator	insulator
Br1Cr1O1_ICSD_27092	insulator	insulator
Br1Cr1S1_ICSD_69659	insulator	insulator
Br1Cr2O4P1_ICSD_410823	insulator	insulator
Br1Cs1F6_ICSD_65712	insulator	insulator
Br1Cs1I2_ICSD_23120	metal	insulator
Br1Cs1O3_ICSD_74769	insulator	insulator
Br1Cs1O4_ICSD_201158	insulator	insulator
Br1Cs1_ICSD_53834	insulator	insulator
Br1Cs1_ICSD_61516	insulator	insulator
Br1Cu1H6N2_ICSD_170947	insulator	insulator
Br1Cu1Hg1S1_ICSD_412308	insulator	insulator
Br1Cu1Hg1Se1_ICSD_411771	insulator	insulator
Br1Cu1La1Nb2O7_ICSD_88033	metal	insulator
Br1Cu1N4S4_ICSD_33515	insulator	insulator
Br1Cu1O2Sr2_ICSD_65470	insulator	insulator
Br1Cu1Se3_ICSD_71309	insulator	insulator
Br1Cu1Te2_ICSD_67252	insulator	insulator
Br1Cu1_ICSD_30091	insulator	insulator
Br1Cu1_ICSD_78275	insulator	insulator
Br1Cu1_ICSD_78278	insulator	insulator
Br1Cu1_ICSD_78280	insulator	insulator
Br1Cu6P1S5_ICSD_89450	insulator	insulator
Br1Dy1Mo1O4_ICSD_425268	insulator	insulator
Br1Dy1S1_ICSD_79106	insulator	insulator
Br1Er1Mo1O4_ICSD_425270	insulator	insulator
Br1Eu1I1_ICSD_59885	insulator	insulator
Br1Eu2P1_ICSD_202067	insulator	insulator
Br1Eu3O4_ICSD_34605	insulator	insulator
Br1F1Pb1_ICSD_155011	insulator	insulator
Br1F1Sr1_ICSD_159279	insulator	insulator
Br1F3Rb1Sb1_ICSD_200109	insulator	insulator
Br1F3_ICSD_39441	insulator	insulator
Br1F4K1_ICSD_16633	insulator	insulator
Br1F4K1_ICSD_16700	insulator	insulator
Br1F4N1O2_ICSD_412427	insulator	insulator
Br1F4Rb1_ICSD_65713	insulator	insulator
Br1F5O3Te1_ICSD_50200	insulator	insulator

Supplementary Table 180. Five-fold cross validated predictions for the metal/insulator classification (172/598).

system	calculated	predicted
Br1F5Sn3_ICSD_1383	insulator	insulator
Br1F5_ICSD_31690	insulator	insulator
Br1F6O2Sb1_ICSD_173608	insulator	insulator
Br1F8Sb1_ICSD_9886	insulator	insulator
Br1Fe1O3Sr2_ICSD_93509	insulator	insulator
Br1Gd1Mo1O4_ICSD_425266	insulator	insulator
Br1Gd1O1_ICSD_41071	insulator	insulator
Br1Gd3S8Si2_ICSD_411995	insulator	insulator
Br1H1Hg1O4_ICSD_29039	insulator	insulator
Br1H1K2O1_ICSD_78734	insulator	insulator
Br1H1O1Pb1_ICSD_404573	insulator	insulator
Br1H1O1Rb2_ICSD_78735	insulator	insulator
Br1H1O1Sr1_ICSD_407720	insulator	insulator
Br1H1Sr1_ICSD_25543	insulator	insulator
Br1H1_ICSD_28842	insulator	insulator
Br1H1_ICSD_63670	metal	metal
Br1H2Li1O1_ICSD_391154	insulator	insulator
Br1H2O6Rb1Se2Zn1_ICSD_409916	insulator	insulator
Br1H3Li4O3_ICSD_412730	insulator	insulator
Br1H4N1O4_ICSD_165085	insulator	insulator
Br1H4N1_ICSD_27724	insulator	insulator
Br1H4N1_ICSD_43300	insulator	insulator
Br1H4Na1O2_ICSD_8125	insulator	insulator
Br1H4P1_ICSD_23691	insulator	insulator
Br1H5O2_ICSD_34105	insulator	insulator
Br1H6Li1O7_ICSD_73706	insulator	insulator
Br1H6Na1O5_ICSD_74654	insulator	insulator
Br1Hf1N1_ICSD_51773	insulator	insulator
Br1Hf1N1_ICSD_95720	insulator	insulator
Br1Hg1I1_ICSD_109010	insulator	insulator
Br1Hg2N1_ICSD_24706	insulator	insulator
Br1Hg2P3_ICSD_74770	insulator	insulator
Br1Hg3I1Te2_ICSD_99125	insulator	insulator
Br1Ho1Mo1O4_ICSD_425269	insulator	insulator
Br1I1Pb1_ICSD_22138	metal	insulator
Br1In1O1_ICSD_24059	insulator	insulator
Br1In1O3Te1_ICSD_420301	insulator	insulator
Br1In1S4Sb2_ICSD_159467	insulator	insulator
Br1In1Sb2Se4_ICSD_159466	insulator	insulator
Br1In1Te1_ICSD_100705	insulator	insulator
Br1In1_ICSD_62239	insulator	insulator
Br1In3La5_ICSD_409631	metal	metal
Br1In5S5_ICSD_414219	insulator	insulator
Br1In5Se5_ICSD_414218	insulator	insulator
Br1K1O3_ICSD_74767	insulator	insulator
Br1K1O4_ICSD_15222	insulator	insulator
Br1K1_ICSD_290558	insulator	insulator
Br1K1_ICSD_52243	insulator	insulator
Br1K3Mo2O7_ICSD_2163	insulator	insulator
Br1K3O1_ICSD_33920	insulator	insulator

Supplementary Table 181. Five-fold cross validated predictions for the metal/insulator classification (173/598).

system	calculated	predicted
Br1La1O1_ICSD_24612	insulator	insulator
Br1La1O7Pb6_ICSD_249390	insulator	insulator
Br1La1_ICSD_23354	metal	metal
Br1La3S8Si2_ICSD_411996	insulator	insulator
Br1La5Sb3_ICSD_50725	metal	metal
Br1La5Sn3_ICSD_95239	metal	metal
Br1Li10N3_ICSD_78819	insulator	insulator
Br1Li1_ICSD_44274	insulator	insulator
Br1Li3O1_ICSD_67265	insulator	insulator
Br1Li6O5P1_ICSD_421480	insulator	insulator
Br1Lu1O1_ICSD_249338	insulator	insulator
Br1Lu1S1_ICSD_6082	insulator	insulator
Br1Mn1S2Sb1_ICSD_172782	insulator	insulator
Br1Mn1Sb1Se2_ICSD_172784	insulator	insulator
Br1Mo1Nd1O4_ICSD_425264	insulator	insulator
Br1Mo1O4Pr1_ICSD_425263	insulator	insulator
Br1Mo1O4Tb1_ICSD_425267	insulator	insulator
Br1Mo1S1_ICSD_163308	insulator	insulator
Br1Mo1Te4_ICSD_82245	insulator	insulator
Br1N1O3_ICSD_407765	insulator	insulator
Br1N1Sr2_ICSD_172600	insulator	insulator
Br1N1Ti1_ICSD_27395	insulator	insulator
Br1N1Zn2_ICSD_425736	insulator	insulator
Br1N1Zr1_ICSD_25507	insulator	insulator
Br1N1Zr1_ICSD_27393	insulator	insulator
Br1N1Zr1_ICSD_87797	insulator	insulator
Br1Na1O3_ICSD_1302	insulator	insulator
Br1Na1_ICSD_53822	insulator	insulator
Br1Nd1O3Te1_ICSD_92319	insulator	insulator
Br1Nd1O5Te2_ICSD_83400	metal	insulator
Br1O12P3Sr5_ICSD_87102	insulator	insulator
Br1O1Pr1_ICSD_262128	insulator	insulator
Br1O1Rb3_ICSD_77196	insulator	insulator
Br1O1Sc1_ICSD_170774	insulator	insulator
Br1O1Ti1_ICSD_155650	insulator	insulator
Br1O1Ti1_ICSD_240499	metal	insulator
Br1O1V1_ICSD_27010	insulator	insulator
Br1O1Y1_ICSD_0	insulator	insulator
Br1O1Yb1_ICSD_28532	metal	insulator
Br1O3Rb1_ICSD_74768	insulator	insulator
Br1O3Tl1_ICSD_76966	insulator	insulator
Br1O4Tl1_ICSD_65660	insulator	insulator
Br1O4Y3_ICSD_419384	insulator	insulator
Br1Rb1_ICSD_18017	insulator	insulator
Br1Rb1_ICSD_61522	insulator	insulator
Br1S1Sb1_ICSD_40918	insulator	insulator
Br1S1Sb1_ICSD_88585	insulator	insulator
Br1S1Sb1_ICSD_88586	insulator	insulator
Br1S1Sb1_ICSD_88587	insulator	insulator
Br1Se2Tl5_ICSD_75960	insulator	insulator

Supplementary Table 182. Five-fold cross validated predictions for the metal/insulator classification (174/598).

system	calculated	predicted
Br1Tb1_ICSD_23353	metal	metal
Br1Te2_ICSD_426519	insulator	insulator
Br1Tl1_ICSD_109144	insulator	insulator
Br1Tl1_ICSD_61519	insulator	insulator
Br1Tl1_ICSD_61532	insulator	insulator
Br1Zr1_ICSD_1168	metal	metal
Br1_ICSD_168172	metal	metal
Br1_ICSD_168174	metal	metal
Br1_ICSD_168175	metal	metal
Br2C12H36P4Tc1_ICSD_166912	metal	insulator
Br2C1Gd2_ICSD_47226	insulator	metal
Br2C1O3Pb2_ICSD_29114	insulator	insulator
Br2C1Se4_ICSD_38387	insulator	insulator
Br2C2Gd2_ICSD_47225	metal	metal
Br2C2H6N2S1_ICSD_408001	insulator	insulator
Br2C2H6S1_ICSD_407035	insulator	insulator
Br2C2Y2_ICSD_78871	metal	metal
Br2C4Cd1H8N8_ICSD_405833	insulator	insulator
Br2C4H11N1_ICSD_240878	insulator	insulator
Br2C4K2N4Pt1_ICSD_413705	insulator	insulator
Br2C4Mg2Na6O12_ICSD_27791	insulator	insulator
Br2C4N4Pt1Rb2_ICSD_852	insulator	insulator
Br2C6Ca1H24O6_ICSD_174396	insulator	insulator
Br2C6H18N4S4_ICSD_151023	insulator	insulator
Br2Ca1H12O6_ICSD_1141	insulator	insulator
Br2Ca1_ICSD_246715	insulator	insulator
Br2Ca1_ICSD_56766	insulator	insulator
Br2Ca2Cu1O2_ICSD_1028	metal	metal
Br2Ca3Si1_ICSD_89537	metal	metal
Br2Ca3Si1_ICSD_89538	insulator	metal
Br2Ca3Si1_ICSD_89539	metal	metal
Br2Ca3Si1_ICSD_89540	metal	metal
Br2Ca3Si1_ICSD_89542	metal	metal
Br2Ca3Si1_ICSD_89543	metal	metal
Br2Ca3Si1_ICSD_89544	metal	metal
Br2Cd1Sb2Se3_ICSD_159464	insulator	insulator
Br2Cd1_ICSD_25782	insulator	insulator
Br2Cd1_ICSD_31536	insulator	insulator
Br2Cd1_ICSD_52367	insulator	insulator
Br2Cd2Hg2S2_ICSD_185392	metal	insulator
Br2Ce1S2Sb1_ICSD_93664	metal	insulator
Br2Cl2Cr1Cs1H18N6O8_ICSD_280499	insulator	insulator
Br2Cl2Cu1Rb2_ICSD_15147	insulator	insulator
Br2Cl6I3Sb1_ICSD_26404	insulator	insulator
Br2Co1H12O12_ICSD_68733	metal	insulator
Br2Co1H12O14_ICSD_73397	insulator	insulator
Br2Co1H12O6_ICSD_40634	insulator	insulator
Br2Co1H4O2_ICSD_22085	insulator	insulator
Br2Co1O2Sr2_ICSD_151789	metal	metal
Br2Co1O3Sb2_ICSD_418858	insulator	insulator

Supplementary Table 183. Five-fold cross validated predictions for the metal/insulator classification (175/598).

system	calculated	predicted
Br2Co1_ICSD_52364	insulator	insulator
Br2Co5O12Se4_ICSD_416966	insulator	insulator
Br2Cr1_ICSD_23903	insulator	insulator
Br2Cs1F1_ICSD_69124	insulator	insulator
Br2Cs1Li1_ICSD_40479	insulator	insulator
Br2Cu1O2Sr2_ICSD_1178	metal	metal
Br2Cu1_ICSD_22079	metal	metal
Br2Cu1_ICSD_409450	metal	insulator
Br2Cu2O5Te2_ICSD_152960	insulator	insulator
Br2Cu2O6Sr2Te1_ICSD_172071	insulator	insulator
Br2Cu3O4Sr2_ICSD_29040	metal	metal
Br2Cu3O6Te2_ICSD_414443	insulator	insulator
Br2Dy1_ICSD_56781	metal	insulator
Br2Eu1_ICSD_60316	insulator	insulator
Br2F10Ge1_ICSD_321	insulator	insulator
Br2F12Pb7_ICSD_92293	insulator	insulator
Br2Fe1_ICSD_409571	metal	insulator
Br2Fe1_ICSD_52365	metal	metal
Br2Ga6Li8O24Si6_ICSD_87988	insulator	insulator
Br2Ga6Na8O24Si6_ICSD_417688	insulator	insulator
Br2Gd2Ge1_ICSD_249475	metal	metal
Br2Ge1_ICSD_100088	insulator	insulator
Br2Ge3La3_ICSD_414172	metal	metal
Br2H10N2O2_ICSD_61783	insulator	insulator
Br2H10N2O2_ICSD_61784	metal	insulator
Br2H12Mg1O6_ICSD_189844	insulator	insulator
Br2H12Ni1O14_ICSD_65047	insulator	insulator
Br2H12Sr7_ICSD_418949	insulator	insulator
Br2H2O1Sr1_ICSD_76502	insulator	insulator
Br2H4Mn1O2_ICSD_22084	insulator	insulator
Br2H6O5U1_ICSD_170208	insulator	insulator
Br2Hg1K2N2O12_ICSD_24405	insulator	insulator
Br2Hg1_ICSD_151889	insulator	insulator
Br2Hg1_ICSD_39319	insulator	insulator
Br2Hg2O6_ICSD_31925	insulator	insulator
Br2Hg2Pb1S2_ICSD_85585	insulator	insulator
Br2Hg2S2Sn1_ICSD_82329	insulator	insulator
Br2Hg2S2Sn1_ICSD_85584	insulator	insulator
Br2Hg2_ICSD_23721	insulator	insulator
Br2Hg3S2_ICSD_82787	insulator	insulator
Br2Hg3Se2_ICSD_99092	insulator	insulator
Br2Hg3Te2_ICSD_27402	insulator	insulator
Br2Hg5O4_ICSD_28232	metal	insulator
Br2K4O1_ICSD_68505	insulator	insulator
Br2La1S2Sb1_ICSD_93666	insulator	insulator
Br2La1_ICSD_65481	insulator	insulator
Br2La2P1_ICSD_418009	metal	metal
Br2Li5N1_ICSD_78836	insulator	insulator
Br2Mg1_ICSD_165973	insulator	insulator
Br2Mn1_ICSD_60250	insulator	insulator

Supplementary Table 184. Five-fold cross validated predictions for the metal/insulator classification (176/598).

system	calculated	predicted
Br2Mn1_ICSD_67500	insulator	insulator
Br2Mn1_ICSD_67517	insulator	insulator
Br2Mn2Sb2Se4_ICSD_172785	insulator	insulator
Br2Mo1O2_ICSD_422483	insulator	insulator
Br2Mo6S6_ICSD_100599	metal	insulator
Br2N3S2V1_ICSD_61088	insulator	insulator
Br2Na4O1_ICSD_67283	insulator	insulator
Br2Nb1O1_ICSD_416669	insulator	insulator
Br2Nb1Se2_ICSD_202821	insulator	insulator
Br2Nb1Se2_ICSD_202822	insulator	insulator
Br2Ni1_ICSD_22106	insulator	insulator
Br2Ni5O12Se4_ICSD_240325	insulator	insulator
Br2Ni5O12Te4_ICSD_96912	insulator	insulator
Br2O1Rb4_ICSD_411954	insulator	insulator
Br2O1S1_ICSD_62972	insulator	insulator
Br2O1V1_ICSD_24381	insulator	insulator
Br2O1_ICSD_50198	metal	insulator
Br2O2Pb3_ICSD_82923	insulator	insulator
Br2O3_ICSD_78369	insulator	insulator
Br2O5Sb4_ICSD_24030	insulator	insulator
Br2O6Pb3Se2_ICSD_422640	insulator	insulator
Br2O6Pb3Te2_ICSD_248131	insulator	insulator
Br2O6Sr1_ICSD_61158	insulator	insulator
Br2Pd1_ICSD_27443	insulator	insulator
Br2Re6S8_ICSD_40701	insulator	insulator
Br2Re6Se8_ICSD_202824	insulator	insulator
Br2S2_ICSD_37020	insulator	insulator
Br2Se2_ICSD_37017	insulator	insulator
Br2Se2_ICSD_37019	insulator	insulator
Br2Sn1_ICSD_411177	metal	insulator
Br2Sr1_ICSD_15972	metal	insulator
Br2Sr1_ICSD_262673	insulator	insulator
Br2Ti1_ICSD_26078	insulator	insulator
Br2V1_ICSD_246906	insulator	insulator
Br2Yb1_ICSD_26045	insulator	insulator
Br2Zn1_ICSD_26080	insulator	insulator
Br2_ICSD_201693	insulator	insulator
Br3C1Cd1H6N3_ICSD_71850	insulator	insulator
Br3C1Eu2Li1N2_ICSD_415050	insulator	insulator
Br3C1F1_ICSD_72370	insulator	insulator
Br3C1Li1N2Sr2_ICSD_415051	insulator	insulator
Br3C2H8Hg1N1_ICSD_110527	insulator	insulator
Br3Ca1Cs1_ICSD_77242	insulator	insulator
Br3Ca1In1_ICSD_54138	insulator	insulator
Br3Cd1Cs1_ICSD_24483	insulator	insulator
Br3Cd1Cs1_ICSD_281176	insulator	insulator
Br3Cd1Cs1_ICSD_41453	insulator	insulator
Br3Cd1H4N1_ICSD_71849	insulator	insulator
Br3Cd1H5N2_ICSD_71851	insulator	insulator
Br3Cd1In1_ICSD_79512	insulator	insulator

Supplementary Table 185. Five-fold cross validated predictions for the metal/insulator classification (177/598).

system	calculated	predicted
Br3Cd1Rb1_ICSD_808	insulator	insulator
Br3Cd1Tl1_ICSD_39808	insulator	insulator
Br3Cd4P2_ICSD_75508	insulator	insulator
Br3Ce1_ICSD_31582	metal	insulator
Br3Ce3Ga1_ICSD_409846	metal	metal
Br3Cr1In1_ICSD_54136	insulator	insulator
Br3Cr1_ICSD_24768	insulator	insulator
Br3Cs1Cu1_ICSD_10184	insulator	insulator
Br3Cs1Cu2_ICSD_49613	insulator	insulator
Br3Cs1Dy1_ICSD_300285	metal	insulator
Br3Cs1Ge1_ICSD_80317	insulator	insulator
Br3Cs1Ge1_ICSD_80319	insulator	insulator
Br3Cs1Ge1_ICSD_80320	insulator	insulator
Br3Cs1Hg1_ICSD_24479	metal	insulator
Br3Cs1Li2_ICSD_245981	insulator	insulator
Br3Cs1Mg1_ICSD_87260	insulator	insulator
Br3Cs1Mn1_ICSD_2782	insulator	insulator
Br3Cs1Pb1_ICSD_109295	insulator	insulator
Br3Cs1Pb1_ICSD_29073	insulator	insulator
Br3Cs1Sn1_ICSD_4071	insulator	insulator
Br3Cs1V1_ICSD_201833	insulator	insulator
Br3Cs1Yb1_ICSD_77243	insulator	insulator
Br3Cu1K2_ICSD_150293	insulator	insulator
Br3Cu1Rb2_ICSD_150295	insulator	insulator
Br3Cu2Rb1_ICSD_150306	insulator	insulator
Br3F3N3P3_ICSD_16099	insulator	insulator
Br3F6S1Sb1_ICSD_66842	insulator	insulator
Br3Fe1In1_ICSD_75469	insulator	insulator
Br3Fe1K1_ICSD_4064	insulator	insulator
Br3Fe1Tl1_ICSD_36217	insulator	insulator
Br3Fe1_ICSD_410924	insulator	insulator
Br3Ga1La3_ICSD_95072	metal	metal
Br3Ga1Li1_ICSD_61338	insulator	insulator
Br3Ga1_ICSD_413456	insulator	insulator
Br3Gd1_ICSD_2610	insulator	insulator
Br3Gd2N1_ICSD_402278	insulator	insulator
Br3Gd2_ICSD_9581	insulator	metal
Br3Gd5O10Se2_ICSD_419349	insulator	insulator
Br3Ge1Rb1_ICSD_65244	insulator	insulator
Br3Hg2Te1_ICSD_417407	insulator	insulator
Br3Hg3Sb2Tl1_ICSD_411521	insulator	insulator
Br3In1K1_ICSD_50510	insulator	insulator
Br3In1Mg1_ICSD_402533	insulator	insulator
Br3In1Mn1_ICSD_75470	insulator	insulator
Br3In1_ICSD_65198	insulator	insulator
Br3Ir1_ICSD_14212	insulator	insulator
Br3K1Mn1_ICSD_42441	insulator	insulator
Br3La1_ICSD_31581	insulator	insulator
Br3La1_ICSD_65479	insulator	insulator
Br3La3Ni1_ICSD_391469	metal	metal

Supplementary Table 186. Five-fold cross validated predictions for the metal/insulator classification (178/598).

system	calculated	predicted
Br3La3Si1_ICSD_411800	metal	metal
Br3La6Si7_ICSD_408032	metal	metal
Br3Li6N1_ICSD_16798	insulator	insulator
Br3Li6N1_ICSD_84092	insulator	insulator
Br3Mn1Rb1_ICSD_14077	insulator	insulator
Br3Mo1Te6_ICSD_82246	insulator	insulator
Br3N1Se2_ICSD_83696	insulator	insulator
Br3N3S4_ICSD_14049	insulator	insulator
Br3Nb1O1_ICSD_418089	insulator	insulator
Br3Ni1Rb1_ICSD_15011	insulator	insulator
Br3O1P1_ICSD_23243	insulator	insulator
Br3O1P1_ICSD_9137	insulator	insulator
Br3O1Pa1_ICSD_1892	insulator	insulator
Br3P1_ICSD_8052	insulator	insulator
Br3Pr1_ICSD_31583	insulator	insulator
Br3Pr1_ICSD_65077	insulator	insulator
Br3Pu1_ICSD_31588	metal	metal
Br3Rb1Ti1_ICSD_154257	insulator	insulator
Br3Rb1V1_ICSD_201831	insulator	insulator
Br3Rh1_ICSD_28245	insulator	insulator
Br3Ru1_ICSD_413691	metal	metal
Br3Ru1_ICSD_414042	metal	insulator
Br3Sb1_ICSD_14217	insulator	insulator
Br3Si1Tb3_ICSD_409655	metal	metal
Br3Tc1_ICSD_260162	insulator	insulator
Br3Ti1_ICSD_39242	insulator	insulator
Br3U1_ICSD_4070	insulator	insulator
Br3Zr1_ICSD_4068	metal	metal
Br4C1N2S3_ICSD_32746	insulator	insulator
Br4C2H12Hg1N6_ICSD_260202	insulator	insulator
Br4C4H8Si1_ICSD_171154	insulator	insulator
Br4C6Cd1Hg4N6S6_ICSD_412498	insulator	insulator
Br4C6O6Ru2_ICSD_48158	insulator	insulator
Br4C8O8W2_ICSD_49689	insulator	insulator
Br4Cd1Cs2_ICSD_40415	metal	insulator
Br4Cd1Cs2_ICSD_40416	insulator	insulator
Br4Cs1Ga1_ICSD_4037	insulator	insulator
Br4Cs1Tl1_ICSD_61180	insulator	insulator
Br4Cs2Hg1_ICSD_200910	insulator	insulator
Br4Cs2Hg1_ICSD_54047	metal	insulator
Br4Cs2I2Pd1_ICSD_240482	insulator	insulator
Br4Cs2I2Pd1_ICSD_412833	insulator	insulator
Br4Cs2Mn1_ICSD_27251	insulator	insulator
Br4Cs2O2U1_ICSD_20130	insulator	insulator
Br4Cs2Re6Se8_ICSD_88611	insulator	insulator
Br4Cs2Zn1_ICSD_41537	insulator	insulator
Br4Cs3Li1_ICSD_245976	insulator	insulator
Br4Cu1H12N2O2_ICSD_88887	metal	insulator
Br4Fe1Li2_ICSD_82200	insulator	insulator
Br4Fe1Tl1_ICSD_402064	insulator	insulator

Supplementary Table 187. Five-fold cross validated predictions for the metal/insulator classification (179/598).

system	calculated	predicted
Br4Ga1Li1_ICSD_61337	insulator	insulator
Br4Ga1Na1_ICSD_69650	insulator	insulator
Br4Ga2_ICSD_62665	insulator	insulator
Br4Ge1_ICSD_409856	insulator	insulator
Br4Ge1_ICSD_409857	insulator	insulator
Br4Ge4S6_ICSD_24370	insulator	insulator
Br4Hg3Te1_ICSD_82788	insulator	insulator
Br4I2Pd1Rb2_ICSD_412835	insulator	insulator
Br4In1K1_ICSD_409063	insulator	insulator
Br4In1Na1_ICSD_65462	insulator	insulator
Br4In2_ICSD_60851	insulator	insulator
Br4K1Tl1_ICSD_35418	insulator	insulator
Br4K2Pd1_ICSD_1982	insulator	insulator
Br4K2Pt1_ICSD_6063	insulator	insulator
Br4K2Zn1_ICSD_78449	insulator	insulator
Br4K2Zn1_ICSD_99702	insulator	insulator
Br4Li2Mg1_ICSD_73276	insulator	insulator
Br4Li2Mn1_ICSD_33865	insulator	insulator
Br4Li2Zn1_ICSD_73223	insulator	insulator
Br4Mn1Rb2_ICSD_8174	insulator	insulator
Br4Na2Zn1_ICSD_73224	insulator	insulator
Br4Nb1O1Tl1_ICSD_415203	insulator	insulator
Br4O1Rb6_ICSD_411955	insulator	insulator
Br4O1W1_ICSD_49547	insulator	insulator
Br4Os1Y4_ICSD_71513	insulator	metal
Br4Os1_ICSD_61042	insulator	insulator
Br4Pa1_ICSD_16123	metal	insulator
Br4Pd4Te3_ICSD_418003	insulator	insulator
Br4Rb2Zn1_ICSD_63000	insulator	insulator
Br4Rb2Zn1_ICSD_77077	insulator	insulator
Br4Rb2Zn1_ICSD_77078	insulator	insulator
Br4Rb2Zn1_ICSD_97854	insulator	insulator
Br4S1Tl6_ICSD_40521	insulator	insulator
Br4S1W1_ICSD_16126	insulator	insulator
Br4Si1_ICSD_710060	insulator	insulator
Br4Si1_ICSD_710061	insulator	insulator
Br4Sn1_ICSD_26033	insulator	insulator
Br4Tc1_ICSD_249920	metal	insulator
Br4Th1_ICSD_130013	insulator	insulator
Br4Th1_ICSD_6056	insulator	insulator
Br4Ti1_ICSD_22103	insulator	insulator
Br4Ti1_ICSD_39241	insulator	insulator
Br4Tl2_ICSD_14216	insulator	insulator
Br4U1_ICSD_2339	insulator	insulator
Br5C2Ce4_ICSD_418408	metal	metal
Br5C2La4_ICSD_418409	metal	metal
Br5Cd1Cs3_ICSD_39599	insulator	insulator
Br5Ce2_ICSD_167088	insulator	insulator
Br5Cs1Hg2_ICSD_200751	insulator	insulator
Br5Cs1Sn2_ICSD_151987	insulator	insulator

Supplementary Table 188. Five-fold cross validated predictions for the metal/insulator classification (180/598).

system	calculated	predicted
Br5Cs3Fe1_ICSD_4065	insulator	insulator
Br5Cs3Hg1_ICSD_200538	insulator	insulator
Br5Dy2Li1_ICSD_402192	metal	insulator
Br5In1Sn2_ICSD_152014	insulator	insulator
Br5K1Sn2_ICSD_152005	insulator	insulator
Br5K2Pr1_ICSD_48191	insulator	insulator
Br5K2U1_ICSD_72616	insulator	insulator
Br5La2_ICSD_65480	insulator	insulator
Br5Nb1_ICSD_409917	insulator	insulator
Br5Nb1_ICSD_67298	insulator	insulator
Br5Nd3S2_ICSD_60134	insulator	insulator
Br5P1_ICSD_22140	insulator	insulator
Br5Pa1_ICSD_22132	insulator	insulator
Br5Pb1Tl3_ICSD_15594	insulator	insulator
Br5Pb1Tl3_ICSD_200875	insulator	insulator
Br5Pb2Rb1_ICSD_151990	insulator	insulator
Br5Pr2_ICSD_62231	insulator	insulator
Br5Rb1Sn2_ICSD_151989	insulator	insulator
Br5Rb3Zn1_ICSD_41536	insulator	insulator
Br5Ru1Y4_ICSD_165336	insulator	insulator
Br5Sn2Tl1_ICSD_151988	insulator	insulator
Br5Ta1_ICSD_109324	insulator	insulator
Br5W1_ICSD_409227	insulator	insulator
Br6Ca1Th1_ICSD_78768	insulator	insulator
Br6Cd1Rb4_ICSD_60625	insulator	insulator
Br6Cs2Na1Y1_ICSD_65733	insulator	insulator
Br6Cs2Pt1_ICSD_77381	insulator	insulator
Br6Cs2Sn1_ICSD_158957	insulator	insulator
Br6Cs2Te1_ICSD_24151	insulator	insulator
Br6Cs2Te1_ICSD_65058	insulator	insulator
Br6Cs2U1_ICSD_20159	metal	insulator
Br6Cs2W1_ICSD_402441	metal	insulator
Br6Cs4Pb1_ICSD_25124	insulator	insulator
Br6Dy1Li1Rb2_ICSD_402536	insulator	insulator
Br6Eu4O1_ICSD_65172	insulator	insulator
Br6Gd1Na3_ICSD_82354	insulator	insulator
Br6Gd4Si1_ICSD_409839	metal	metal
Br6H12Mg1O6Zn2_ICSD_49914	insulator	insulator
Br6H12N4Sc2_ICSD_281573	insulator	insulator
Br6H12O6Zn3_ICSD_64614	insulator	insulator
Br6H16Hg1N4_ICSD_391363	insulator	insulator
Br6H16Hg1N4_ICSD_391364	insulator	insulator
Br6H6N2Sc2_ICSD_281574	insulator	insulator
Br6H8N2Re1_ICSD_412159	insulator	insulator
Br6Hg1Tl4_ICSD_9325	insulator	insulator
Br6Hg3Se2Zr1_ICSD_412467	insulator	insulator
Br6Hg7P4Sn1_ICSD_411860	insulator	insulator
Br6Hg7P4_ICSD_73818	insulator	insulator
Br6In2Th1_ICSD_80180	insulator	insulator
Br6In2Zr1_ICSD_54137	insulator	insulator

Supplementary Table 189. Five-fold cross validated predictions for the metal/insulator classification (181/598).

system	calculated	predicted
Br6K2Os1_ICSD_26770	metal	insulator
Br6K2Pt1_ICSD_23771	insulator	insulator
Br6K2Re1_ICSD_26623	insulator	insulator
Br6K2Se1_ICSD_36228	insulator	insulator
Br6K2Sn1_ICSD_158955	insulator	insulator
Br6K2Te1_ICSD_37270	insulator	insulator
Br6K2Te1_ICSD_65118	insulator	insulator
Br6Mo6S3_ICSD_35356	insulator	insulator
Br6N3P3_ICSD_27271	insulator	insulator
Br6Na1O2W2_ICSD_408934	metal	insulator
Br6Na3Sc1_ICSD_401335	insulator	insulator
Br6Na3Y1_ICSD_82355	insulator	insulator
Br6Nb2Se2_ICSD_35375	insulator	insulator
Br6Nb2Te2_ICSD_35376	insulator	insulator
Br6O1Sr4_ICSD_418452	insulator	insulator
Br6Pb1Rb4_ICSD_65300	insulator	insulator
Br6Pb2Pd1_ICSD_78874	insulator	insulator
Br6Pb4Se1_ICSD_21039	insulator	insulator
Br6Pd1Rb2_ICSD_36305	insulator	insulator
Br6Pd1Se2_ICSD_405774	insulator	insulator
Br6Rb2Sn1_ICSD_158956	insulator	insulator
Br6Rb2Te1_ICSD_49520	insulator	insulator
Br6Rb2Te1_ICSD_49521	insulator	insulator
Br6Rb2U1_ICSD_20158	metal	insulator
Br6Rb2U1_ICSD_82949	insulator	insulator
Br6Rb2W1_ICSD_402439	metal	metal
Br6Se8Ta3_ICSD_408596	metal	insulator
Br6Si1Tb4_ICSD_409703	metal	metal
Br6Sr1Th1_ICSD_78769	insulator	insulator
Br6Te1Tl2_ICSD_99127	insulator	insulator
Br6W1_ICSD_62048	insulator	insulator
Br7Fe1Se1_ICSD_39528	insulator	insulator
Br7Fe1Te1_ICSD_39531	insulator	insulator
Br7Ga1Se1_ICSD_78913	insulator	insulator
Br7Hg6In1Sb4_ICSD_420139	insulator	insulator
Br7Hg6Sb5_ICSD_411219	insulator	insulator
Br7In4_ICSD_401451	insulator	insulator
Br7Nb3S1_ICSD_81078	insulator	insulator
Br7P1_ICSD_26025	insulator	insulator
Br7S1Ta3_ICSD_51101	insulator	insulator
Br7Tb6_ICSD_14182	metal	metal
Br8Cs2Re2_ICSD_26060	insulator	insulator
Br8Ga2Pd1_ICSD_413229	insulator	insulator
Br8Gd5_ICSD_38042	metal	metal
Br8Ge38P8_ICSD_22031	insulator	insulator
Br8Hg2Pd1Rb2_ICSD_203188	insulator	insulator
Br8Li6Mg1_ICSD_73275	insulator	insulator
Br8N4P4_ICSD_2716	insulator	insulator
Br8Nb3_ICSD_25766	insulator	insulator
Br8S9Ta4_ICSD_171236	insulator	insulator

Supplementary Table 190. Five-fold cross validated predictions for the metal/insulator classification (182/598).

system	calculated	predicted
Br8Tb5_ICSD_38041	metal	metal
Br9Cs3Er2_ICSD_203114	insulator	insulator
Br9Cs3Ho2_ICSD_72798	insulator	insulator
Br9Cs3Mo2_ICSD_26213	insulator	insulator
Br9Cs3Tb2_ICSD_203109	insulator	insulator
Br9Cs3Y2_ICSD_37221	insulator	insulator
Br9Cs5Nb2S4_ICSD_410590	insulator	insulator
Br9In7_ICSD_78804	insulator	insulator
Br9K3Mo2_ICSD_202299	insulator	insulator
Br9Mo2Rb3_ICSD_202293	insulator	insulator
Br9Nb2Rb3_ICSD_402032	insulator	insulator
Br9Nb2S4Tl5_ICSD_418796	insulator	insulator
Br9O4Pb8Tl1_ICSD_36007	insulator	insulator
Br9Os2Rb3_ICSD_56896	insulator	insulator
Br9Rb3Sb2_ICSD_39823	insulator	insulator
Br9Rb3V2_ICSD_61235	insulator	insulator
Br9Ta1Te1_ICSD_410949	insulator	insulator
Br9Te1W1_ICSD_410948	insulator	insulator
C1.06Mo6Ni6_ICSD_77164	metal	metal
C10Cl1Co3O9_ICSD_200825	insulator	insulator
C10Cl4La8_ICSD_416907	metal	metal
C10Co1H14N8O2_ICSD_109797	metal	insulator
C10Cs1_ICSD_657337	insulator	insulator
C10Cu2H18N2O10_ICSD_171405	insulator	insulator
C10Fe22Mo12_ICSD_61141	metal	metal
C10H14N8Ni1O2_ICSD_109784	insulator	insulator
C10H18N2O10Rh2_ICSD_172424	insulator	insulator
C10Hg1Mn2O10_ICSD_71879	insulator	insulator
C10O10Os3S1_ICSD_201861	insulator	insulator
C11N4_ICSD_184896	insulator	metal
C11N4_ICSD_184897	metal	insulator
C12Cl12N12O4P4S12Sb4_ICSD_80097	insulator	insulator
C12Cl6Gd10_ICSD_420618	metal	metal
C12Co2N12Zn3_ICSD_157849	insulator	metal
C12Dy8Rh5_ICSD_617616	metal	metal
C12F10Se1_ICSD_410720	insulator	insulator
C12Fe2N12Zn3_ICSD_157850	metal	insulator
C12Gd8Rh5_ICSD_617956	metal	metal
C12H36I4Ni1O6S6_ICSD_170024	insulator	insulator
C12Ho8Rh5_ICSD_618102	metal	metal
C12Mn2O12Pt1_ICSD_41117	insulator	insulator
C12N6_ICSD_30815	insulator	insulator
C12Ni4O18P4_ICSD_14337	insulator	insulator
C12O12Pt1Re2_ICSD_60074	insulator	insulator
C12O12Ru4Se4_ICSD_92913	insulator	insulator
C12Rh5Tb8_ICSD_618726	metal	metal
C12Rh5Y8_ICSD_618734	metal	metal
C14Cl12H24Mn1N8Nb6_ICSD_110055	insulator	insulator
C14H24K1Mn1N8_ICSD_107665	insulator	insulator
C14H28Mn1N6O4S2_ICSD_409689	metal	insulator

Supplementary Table 191. Five-fold cross validated predictions for the metal/insulator classification (183/598).

system	calculated	predicted
C15Dy12Mn5_ICSD_81770	metal	metal
C15Os5Tb12_ICSD_86729	metal	metal
C16Co4O16Sn1_ICSD_67320	insulator	insulator
C16Cr2H24O8Sb4_ICSD_241130	insulator	insulator
C18Er10Mn13_ICSD_603261	metal	metal
C18Ho10Mn13_ICSD_603286	metal	metal
C18Lu10Mn13_ICSD_603277	metal	metal
C18Mn13Tb10_ICSD_603285	metal	metal
C18Mn13Y10_ICSD_603284	metal	metal
C18Ru12Th11_ICSD_79240	metal	metal
C19Er10Ru10_ICSD_59419	metal	metal
C19Er15_ICSD_76817	metal	metal
C19Ho15_ICSD_618080	metal	metal
C19Sc15_ICSD_42631	metal	metal
C19Y15_ICSD_619118	metal	metal
C1Ca1F1K1O3_ICSD_154682	insulator	insulator
C1Ca1F1O3Rb1_ICSD_262232	insulator	insulator
C1Ca1H12O9_ICSD_151488	insulator	insulator
C1Ca1H2O4_ICSD_100847	insulator	insulator
C1Ca1N2_ICSD_26677	insulator	insulator
C1Ca1O3_ICSD_150	insulator	insulator
C1Ca1O3_ICSD_161820	insulator	insulator
C1Ca1O3_ICSD_169933	insulator	insulator
C1Ca1O3_ICSD_181959	insulator	insulator
C1Ca1O3_ICSD_280991	insulator	insulator
C1Ca1O3_ICSD_52152	insulator	insulator
C1Ca1O3_ICSD_83607	insulator	insulator
C1Ca1Pd3_ICSD_108128	metal	metal
C1Ca2F2O3_ICSD_100607	insulator	insulator
C1Ca4N4_ICSD_167787	insulator	insulator
C1Ca7H4O23Si6_ICSD_73333	insulator	insulator
C1Cd1Cl2H4N2O1_ICSD_39960	insulator	insulator
C1Cd1N2_ICSD_95264	insulator	insulator
C1Cd1N2_ICSD_95265	insulator	insulator
C1Cd1O3_ICSD_20181	insulator	insulator
C1Cd1Ti2_ICSD_42924	metal	metal
C1Cd1_ICSD_183177	metal	metal
C1Cd1_ICSD_183178	metal	metal
C1Cd3H2O8Se1_ICSD_95816	insulator	insulator
C1Ce1Cr2Si2_ICSD_160692	metal	metal
C1Ce1F1O3_ICSD_81673	insulator	insulator
C1Ce1H2O6P2_ICSD_262898	insulator	insulator
C1Ce1Mo2Si2_ICSD_189334	metal	metal
C1Ce1P1Ru2_ICSD_260480	metal	metal
C1Ce1Rh3_ICSD_76774	metal	metal
C1Ce1_ICSD_76769	metal	metal
C1Ce3In1_ICSD_76770	metal	metal
C1Ce3In1_ICSD_98501	metal	metal
C1Ce3Pb1_ICSD_76773	metal	metal
C1Ce3Sn1_ICSD_76776	metal	metal

Supplementary Table 192. Five-fold cross validated predictions for the metal/insulator classification (184/598).

system	calculated	predicted
C1Ce3Tl1_ICSD_76778	metal	metal
C1Cl14Ti6_ICSD_75502	insulator	insulator
C1Cl14Zr6_ICSD_202915	insulator	insulator
C1Cl18Rb4Zr6_ICSD_165401	insulator	insulator
C1Cl18W6_ICSD_413026	insulator	insulator
C1Cl1Co1H18N6O3_ICSD_412059	insulator	insulator
C1Cl1Cu1H2N2_ICSD_247128	insulator	insulator
C1Cl1Cu1O1_ICSD_63490	insulator	insulator
C1Cl1F3O3S1_ICSD_96950	insulator	insulator
C1Cl1F3_ICSD_49696	insulator	insulator
C1Cl1F4I1_ICSD_280343	insulator	insulator
C1Cl1Gd2_ICSD_68798	metal	metal
C1Cl1H3O1S1_ICSD_107497	insulator	insulator
C1Cl1H4N5_ICSD_2762	insulator	insulator
C1Cl1H5N2O4S1_ICSD_39575	insulator	insulator
C1Cl1H5N2S1_ICSD_250292	insulator	insulator
C1Cl1H6N1O1_ICSD_241238	insulator	insulator
C1Cl1H6N3O1_ICSD_62660	insulator	insulator
C1Cl1Hg1N1S1_ICSD_409579	insulator	insulator
C1Cl1La1N2_ICSD_413904	insulator	insulator
C1Cl1La2N3_ICSD_412412	insulator	insulator
C1Cl1La2_ICSD_400894	metal	metal
C1Cl1N1O2S2_ICSD_91453	insulator	insulator
C1Cl1N1Pb1S1_ICSD_409835	insulator	insulator
C1Cl1N1_ICSD_16660	insulator	insulator
C1Cl1N5S3_ICSD_62270	insulator	insulator
C1Cl2Eu2N2_ICSD_391256	insulator	insulator
C1Cl2F2_ICSD_33947	insulator	insulator
C1Cl2F2_ICSD_33948	insulator	insulator
C1Cl2Gd2_ICSD_400348	insulator	insulator
C1Cl2H3N1O2S1_ICSD_151036	insulator	insulator
C1Cl2Hg2N2_ICSD_412608	insulator	insulator
C1Cl2Lu2_ICSD_62227	insulator	insulator
C1Cl2N2Sr2_ICSD_391257	insulator	insulator
C1Cl2O3Pb2_ICSD_36241	insulator	insulator
C1Cl2Sc2_ICSD_59124	insulator	metal
C1Cl3F1_ICSD_80891	insulator	insulator
C1Cl3F3Si1_ICSD_173735	insulator	insulator
C1Cl3Gd3_ICSD_37323	metal	metal
C1Cl3H3Te1_ICSD_281531	insulator	insulator
C1Cl4H5N1Si2_ICSD_410261	insulator	insulator
C1Cl5H6Hg2N3_ICSD_59241	insulator	insulator
C1Cl6H2O4Pb4_ICSD_88936	insulator	insulator
C1Cl6N1Sb1_ICSD_279639	insulator	insulator
C1Cl8Sc5_ICSD_60855	metal	metal
C1Co12Ge4U6_ICSD_181657	metal	metal
C1Co1Dy1_ICSD_61687	metal	metal
C1Co1Er1_ICSD_61689	metal	metal
C1Co1Fe2_ICSD_167128	metal	metal
C1Co1Gd1_ICSD_61685	metal	metal

Supplementary Table 193. Five-fold cross validated predictions for the metal/insulator classification (185/598).

system	calculated	predicted
C1Co1Ho1_ICSD_61688	metal	metal
C1Co1Lu1_ICSD_61691	metal	metal
C1Co1N2_ICSD_249387	metal	metal
C1Co1O3_ICSD_61066	insulator	insulator
C1Co1Tb1_ICSD_61686	metal	metal
C1Co1Tm1_ICSD_61690	metal	metal
C1Co1Y1_ICSD_96327	metal	metal
C1Co2Mn2_ICSD_44353	metal	metal
C1Co2Mo4_ICSD_76136	metal	metal
C1Co2Nb4_ICSD_617428	metal	metal
C1Co2Ta4_ICSD_617448	metal	metal
C1Co2W4_ICSD_617463	metal	metal
C1Co2_ICSD_617391	metal	metal
C1Co3Mg1_ICSD_76790	metal	metal
C1Co3Mo3_ICSD_617424	metal	metal
C1Co3Nb3_ICSD_617429	metal	metal
C1Co3Sc1_ICSD_76793	metal	metal
C1Co3Sn1_ICSD_108129	metal	metal
C1Co3Ta3_ICSD_76794	metal	metal
C1Co3W3_ICSD_166747	metal	metal
C1Co3Zn1_ICSD_76797	metal	metal
C1Co3_ICSD_43521	metal	metal
C1Co6Mo6_ICSD_617425	metal	metal
C1Co6W6_ICSD_165459	metal	metal
C1Cr1_ICSD_181709	metal	metal
C1Cr1_ICSD_603179	metal	metal
C1Cr2Ga1_ICSD_419116	metal	metal
C1Cr2Ge1_ICSD_166030	metal	metal
C1Cr2La1Si2_ICSD_160691	metal	metal
C1Cr2Pr1Si2_ICSD_152143	metal	metal
C1Cr2Si1_ICSD_183361	metal	metal
C1Cr3Ge1_ICSD_42629	metal	metal
C1Cr3Nb3_ICSD_76131	metal	metal
C1Cr3P1_ICSD_617527	metal	metal
C1Cr3_ICSD_603557	metal	metal
C1Cr3_ICSD_617486	metal	metal
C1Cr5Si3_ICSD_617535	metal	metal
C1Cs1F3O1_ICSD_69971	insulator	insulator
C1Cs1F3O3S1_ICSD_415056	insulator	insulator
C1Cs1H1N2O2_ICSD_170588	insulator	insulator
C1Cs1H1O2_ICSD_172202	insulator	insulator
C1Cs1H1O3_ICSD_300259	insulator	insulator
C1Cs1I14Zr6_ICSD_60917	metal	insulator
C1Cs1N1S1_ICSD_60875	insulator	insulator
C1Cs2H6O6_ICSD_411684	insulator	insulator
C1Cs2I18Zr7_ICSD_72606	insulator	metal
C1Cs2O3_ICSD_14156	insulator	insulator
C1Cs4O4_ICSD_245445	insulator	insulator
C1Cs4O4_ICSD_245446	insulator	insulator
C1Cs4O4_ICSD_245447	insulator	insulator

Supplementary Table 194. Five-fold cross validated predictions for the metal/insulator classification (186/598).

system	calculated	predicted
C1Cs4O4_ICSD_245449	insulator	insulator
C1Cs4O4_ICSD_245450	insulator	insulator
C1Cs4O4_ICSD_245451	insulator	insulator
C1Cs4O4_ICSD_245453	insulator	insulator
C1Cu1F1K1O3_ICSD_75401	insulator	insulator
C1Cu1H1O4Tl1_ICSD_74875	insulator	insulator
C1Cu1H1O5S1_ICSD_281184	insulator	insulator
C1Cu1H8N2O5P2_ICSD_279591	insulator	insulator
C1Cu1N1S1_ICSD_124	insulator	insulator
C1Cu1N1S1_ICSD_24372	insulator	insulator
C1Cu1N1S1_ICSD_32578	insulator	insulator
C1Cu1N2_ICSD_415220	metal	insulator
C1Cu1O3_ICSD_6179	insulator	insulator
C1Dy1Fe2Si1_ICSD_40773	metal	metal
C1Dy1H1O4_ICSD_409351	insulator	insulator
C1Dy1Rh3_ICSD_76814	metal	metal
C1Dy1Ru2Si1_ICSD_50633	metal	metal
C1Dy2Fe2Si2_ICSD_67467	metal	metal
C1Dy2N2O2_ICSD_416881	insulator	insulator
C1Dy2_ICSD_108130	metal	metal
C1Dy3Ga1_ICSD_76812	metal	metal
C1Dy3In1_ICSD_56400	metal	metal
C1Dy3Pb1_ICSD_56410	metal	metal
C1Dy3Sn1_ICSD_56406	metal	metal
C1Dy3Tl1_ICSD_76815	metal	metal
C1Dy5Ge3_ICSD_617597	metal	metal
C1Dy5Si3_ICSD_617618	metal	metal
C1Er1Rh3_ICSD_108131	metal	metal
C1Er2Fe2Si2_ICSD_617663	metal	metal
C1Er2N2O2_ICSD_416880	insulator	insulator
C1Er3Ga1_ICSD_76818	metal	metal
C1Er3In1_ICSD_76819	metal	metal
C1Er3Pb1_ICSD_76822	metal	metal
C1Er3Sn1_ICSD_76823	metal	metal
C1Er3Tl1_ICSD_76824	metal	metal
C1Er5Ge3_ICSD_617665	metal	metal
C1Er5Si3_ICSD_617683	metal	metal
C1Eu1Rh3_ICSD_76825	metal	metal
C1Eu2I3Li1N2_ICSD_170261	insulator	insulator
C1Eu2N2O2_ICSD_82267	metal	insulator
C1F1H3O2_ICSD_170925	insulator	insulator
C1F1H6N1_ICSD_110656	insulator	insulator
C1F1H7N3O3P1_ICSD_151288	insulator	insulator
C1F1K1O3Sr1_ICSD_262231	insulator	insulator
C1F1K3O3_ICSD_66028	insulator	insulator
C1F1La1N2_ICSD_419916	metal	insulator
C1F1La1O3_ICSD_26678	insulator	insulator
C1F1N1S1Sn1_ICSD_2418	insulator	insulator
C1F1O3Rb1Sr1_ICSD_262233	insulator	insulator
C1F1O3Rb3_ICSD_66029	insulator	insulator

Supplementary Table 195. Five-fold cross validated predictions for the metal/insulator classification (187/598).

system	calculated	predicted
C1F1O3Tl3_ICSD_2515	insulator	insulator
C1F2Gd2_ICSD_33912	insulator	insulator
C1F2Ho2_ICSD_71626	insulator	insulator
C1F2K2O6S2_ICSD_92475	insulator	insulator
C1F2N1O2P1_ICSD_248121	insulator	insulator
C1F2Na1O3Yb1_ICSD_172520	insulator	insulator
C1F2O3Pb2_ICSD_35413	insulator	insulator
C1F3H1O3S1_ICSD_65782	insulator	insulator
C1F3H2Na1O4S1_ICSD_151032	insulator	insulator
C1F3H3O4S1_ICSD_2615	insulator	insulator
C1F3H5O5S1_ICSD_2007	insulator	insulator
C1F3Hg1O3S1_ICSD_98942	insulator	insulator
C1F3I1N2O6_ICSD_78758	insulator	insulator
C1F3I1_ICSD_73268	insulator	insulator
C1F3Li1O3S1_ICSD_110018	insulator	insulator
C1F3O3Rb1S1_ICSD_415817	insulator	insulator
C1F4H6N3Sb1_ICSD_39318	insulator	insulator
C1F4_ICSD_66659	insulator	insulator
C1F5H7N4Zr1_ICSD_281003	insulator	insulator
C1F5N1S1_ICSD_413757	insulator	insulator
C1F5N1S2_ICSD_413754	insulator	insulator
C1F6H6N3P1_ICSD_203170	insulator	insulator
C1F7I1_ICSD_401706	insulator	insulator
C1Fe14La2_ICSD_20971	metal	metal
C1Fe16Si7U6_ICSD_160155	metal	metal
C1Fe1H5O4P1_ICSD_153033	insulator	insulator
C1Fe1N2_ICSD_419223	metal	metal
C1Fe1Na3O7P1_ICSD_77053	insulator	insulator
C1Fe1O3_ICSD_169791	insulator	insulator
C1Fe1O3_ICSD_169796	insulator	insulator
C1Fe2Ho2Si2_ICSD_617751	metal	metal
C1Fe2Mo4_ICSD_76135	metal	metal
C1Fe2Nd2Si2_ICSD_57048	metal	metal
C1Fe2Nd2Si2_ICSD_603157	metal	metal
C1Fe2Ni1_ICSD_167127	metal	metal
C1Fe2Si1Th1_ICSD_75133	metal	metal
C1Fe2Si2Tb2_ICSD_617824	metal	metal
C1Fe2Si2Y2_ICSD_617825	metal	metal
C1Fe2W2_ICSD_20233	metal	metal
C1Fe2_ICSD_162103	metal	metal
C1Fe2_ICSD_187138	metal	metal
C1Fe2_ICSD_76826	metal	metal
C1Fe3Mo3_ICSD_617778	metal	metal
C1Fe3Sn1_ICSD_76842	metal	metal
C1Fe3V3_ICSD_617880	metal	metal
C1Fe3W3_ICSD_76760	metal	metal
C1Fe3Zn1_ICSD_76763	metal	metal
C1Fe3Zr3_ICSD_617908	metal	metal
C1Fe3_ICSD_16593	metal	metal
C1Fe3_ICSD_187142	metal	metal

Supplementary Table 196. Five-fold cross validated predictions for the metal/insulator classification (188/598).

system	calculated	predicted
C1Fe3_ICSD_42542	metal	metal
C1Fe4_ICSD_187143	metal	metal
C1Fe4_ICSD_187144	metal	metal
C1Fe4_ICSD_44729	metal	metal
C1Fe6W6_ICSD_76761	metal	metal
C1Ga1Ho3_ICSD_76843	metal	metal
C1Ga1Mn3_ICSD_76845	metal	metal
C1Ga1Mo2_ICSD_180615	metal	metal
C1Ga1Mo4S8_ICSD_76848	metal	metal
C1Ga1N1Si1_ICSD_183047	insulator	insulator
C1Ga1N5Sr4_ICSD_171166	insulator	insulator
C1Ga1Nb2_ICSD_167969	metal	metal
C1Ga1Nd3_ICSD_76849	metal	metal
C1Ga1Pr3_ICSD_76851	metal	metal
C1Ga1Pt3_ICSD_617925	metal	metal
C1Ga1Sc2_ICSD_160379	metal	metal
C1Ga1Sc3_ICSD_50162	metal	metal
C1Ga1Ta2_ICSD_617926	metal	metal
C1Ga1Ti2_ICSD_419117	metal	metal
C1Ga1Tm3_ICSD_76993	metal	metal
C1Ga1V2_ICSD_167968	metal	metal
C1Ga1Y3_ICSD_56396	metal	metal
C1Ga1Yb3_ICSD_56398	metal	metal
C1Ga3N3Si1_ICSD_183050	insulator	insulator
C1Gd1H5O7P1_ICSD_161193	insulator	insulator
C1Gd1Rh3_ICSD_76997	metal	metal
C1Gd2I1_ICSD_68797	metal	metal
C1Gd3I3_ICSD_73199	metal	metal
C1Gd3In1_ICSD_76994	metal	metal
C1Gd3Pb1_ICSD_76996	metal	metal
C1Gd3Sn1_ICSD_76998	metal	metal
C1Gd3Tl1_ICSD_76999	metal	metal
C1Gd5Si3_ICSD_617958	metal	metal
C1Ge1H3N1O1_ICSD_201197	insulator	insulator
C1Ge1Mn3_ICSD_25775	metal	metal
C1Ge1Mn3_ICSD_44351	metal	metal
C1Ge1Nb2_ICSD_188618	metal	metal
C1Ge1Ni3_ICSD_77001	metal	metal
C1Ge1Ti2_ICSD_42921	metal	metal
C1Ge1V2_ICSD_166031	metal	metal
C1Ge1V3_ICSD_617981	metal	metal
C1Ge1_ICSD_182363	insulator	metal
C1Ge1_ICSD_182364	metal	insulator
C1Ge3Ho5_ICSD_617965	metal	metal
C1Ge3Mo5_ICSD_42922	metal	metal
C1Ge3Tb5_ICSD_617974	metal	metal
C1Ge3Y5_ICSD_617982	metal	metal
C1H1K1N2O2_ICSD_170586	insulator	insulator
C1H1K1N2_ICSD_401784	insulator	insulator
C1H1K1O2_ICSD_151294	insulator	insulator

Supplementary Table 197. Five-fold cross validated predictions for the metal/insulator classification (189/598).

system	calculated	predicted
C1H1K1O3_ICSD_157166	insulator	insulator
C1H1K1O3_ICSD_2074	metal	insulator
C1H1K1O3_ICSD_2075	metal	insulator
C1H1K1O3_ICSD_2076	metal	insulator
C1H1K1O3_ICSD_43015	insulator	insulator
C1H1Li1O2_ICSD_151274	insulator	insulator
C1H1N1O1Pb1S1_ICSD_79723	metal	insulator
C1H1N1_ICSD_187644	insulator	insulator
C1H1N1_ICSD_76418	insulator	insulator
C1H1N1_ICSD_76419	insulator	insulator
C1H1N2Na1_ICSD_2495	insulator	insulator
C1H1Na1O2_ICSD_109643	insulator	insulator
C1H1Na1O2_ICSD_151256	insulator	insulator
C1H1Na1O3_ICSD_18183	insulator	insulator
C1H1Na1O3_ICSD_26933	insulator	insulator
C1H1O2Rb1_ICSD_172203	insulator	insulator
C1H1O2Tl1_ICSD_110364	insulator	insulator
C1H1O2Tl1_ICSD_151295	insulator	insulator
C1H1O4Y1_ICSD_644	insulator	insulator
C1H1_ICSD_187642	insulator	insulator
C1H2N2_ICSD_40446	insulator	insulator
C1H2Na2O4_ICSD_15959	insulator	insulator
C1H2Nd1O6P2_ICSD_262900	metal	insulator
C1H2O2_ICSD_151248	insulator	insulator
C1H2O6P2Pu1_ICSD_262902	metal	insulator
C1H3Hg1N3_ICSD_761194	insulator	insulator
C1H3K1N2O1S1_ICSD_16412	insulator	insulator
C1H3Li1O3_ICSD_151231	insulator	insulator
C1H3N1O1Si1_ICSD_201196	insulator	insulator
C1H3N1O3Zn1_ICSD_41113	insulator	insulator
C1H3N5O4_ICSD_281338	insulator	insulator
C1H4Ho2O7_ICSD_62030	insulator	insulator
C1H4N1Na1O2_ICSD_830	insulator	insulator
C1H4N2O1_ICSD_151523	insulator	insulator
C1H4N2O1_ICSD_245371	insulator	insulator
C1H4N2O2S1_ICSD_165279	insulator	insulator
C1H4N2S1_ICSD_69145	insulator	insulator
C1H4N2S1_ICSD_956	insulator	insulator
C1H4N3Rb1_ICSD_422591	insulator	insulator
C1H4Na4O9P2_ICSD_19	insulator	insulator
C1H5N1O2_ICSD_151222	insulator	insulator
C1H5N1O3_ICSD_158914	insulator	insulator
C1H5N3O4_ICSD_34697	insulator	insulator
C1H6N2O2_ICSD_172855	insulator	insulator
C1H6N2S2_ICSD_421191	insulator	insulator
C1H6N2S2_ICSD_421192	insulator	insulator
C1H6N4O1_ICSD_2812	insulator	insulator
C1H6N4S1_ICSD_42827	insulator	insulator
C1H7N3O3_ICSD_163085	insulator	insulator
C1H9N4O4P1_ICSD_163005	insulator	insulator

Supplementary Table 198. Five-fold cross validated predictions for the metal/insulator classification (190/598).

system	calculated	predicted
C1H9N7O3_ICSD_8100	insulator	insulator
C1Hf1_ICSD_169399	metal	metal
C1Hf1_ICSD_185985	metal	metal
C1Hf1_ICSD_185992	insulator	metal
C1Hf1_ICSD_618004	metal	metal
C1Hf2In1_ICSD_163508	metal	metal
C1Hf2Pb1_ICSD_618050	metal	metal
C1Hf2S1_ICSD_162461	metal	metal
C1Hf2Sn1_ICSD_161073	metal	metal
C1Hf2Tl1_ICSD_618061	metal	metal
C1Hf3Zn3_ICSD_618069	metal	metal
C1Hg1N2_ICSD_411067	insulator	insulator
C1Hg1N2_ICSD_412278	insulator	insulator
C1Ho1Rh3_ICSD_77033	metal	metal
C1Ho1Ru2Si1_ICSD_50634	metal	metal
C1Ho2N2O2_ICSD_416882	insulator	insulator
C1Ho2N6Si4_ICSD_94032	insulator	insulator
C1Ho2_ICSD_77029	metal	metal
C1Ho3In1_ICSD_77032	metal	metal
C1Ho3Sn1_ICSD_77034	metal	metal
C1Ho3Tl1_ICSD_77035	metal	metal
C1Ho5Si3_ICSD_618104	metal	metal
C1I12Zr6_ICSD_60918	insulator	metal
C1I1N1_ICSD_77911	insulator	insulator
C1I4_ICSD_30789	insulator	insulator
C1I5Y4_ICSD_68014	metal	metal
C1In1La3_ICSD_80956	metal	metal
C1In1Mn3_ICSD_106306	metal	metal
C1In1Mn3_ICSD_657357	metal	metal
C1In1Nb2_ICSD_163519	metal	metal
C1In1Nd3_ICSD_77037	metal	metal
C1In1Nd3_ICSD_98502	metal	metal
C1In1Pr3_ICSD_77039	metal	metal
C1In1Pt3_ICSD_77040	metal	metal
C1In1Sc2_ICSD_163510	metal	metal
C1In1Sc3_ICSD_50163	metal	metal
C1In1Ta2_ICSD_163509	metal	metal
C1In1Tb3_ICSD_77042	metal	metal
C1In1Ti2_ICSD_180620	metal	metal
C1In1Ti3_ICSD_163844	metal	metal
C1In1Tm3_ICSD_77043	metal	metal
C1In1V2_ICSD_163505	metal	metal
C1In1Y3_ICSD_80955	metal	metal
C1In1Yb3_ICSD_56401	metal	metal
C1In1Zr2_ICSD_163516	metal	metal
C1Ir1_ICSD_169404	metal	metal
C1Ir1_ICSD_185983	metal	metal
C1Ir1_ICSD_185990	metal	metal
C1Ir1_ICSD_185997	metal	metal
C1Ir3Sc1_ICSD_77044	metal	metal

Supplementary Table 199. Five-fold cross validated predictions for the metal/insulator classification (191/598).

system	calculated	predicted
C1K1La1O4_ICSD_90735	metal	insulator
C1K1Li1O3_ICSD_84638	insulator	insulator
C1K1N1S1_ICSD_65075	insulator	insulator
C1K1N1_ICSD_173942	insulator	insulator
C1K1N1_ICSD_27351	insulator	insulator
C1K1N3O2_ICSD_408287	insulator	insulator
C1K1N3O6_ICSD_20483	insulator	insulator
C1K1O5Pu1_ICSD_15685	insulator	insulator
C1K2N2_ICSD_423580	insulator	insulator
C1K2O3_ICSD_10191	insulator	insulator
C1K2O3_ICSD_52535	insulator	insulator
C1K2O3_ICSD_662	insulator	insulator
C1K3O8V1_ICSD_60813	insulator	insulator
C1K4O4_ICSD_245417	insulator	insulator
C1K4O4_ICSD_245418	insulator	insulator
C1K4O4_ICSD_245419	insulator	insulator
C1K4O4_ICSD_245422	insulator	insulator
C1K4O4_ICSD_245424	insulator	insulator
C1K4O4_ICSD_245425	insulator	insulator
C1K4O4_ICSD_245427	insulator	insulator
C1K4O4_ICSD_245428	insulator	insulator
C1La1_ICSD_169398	metal	metal
C1La2N2O4Si1_ICSD_420943	insulator	insulator
C1La2O5_ICSD_51468	insulator	insulator
C1La3Pb1_ICSD_56409	metal	metal
C1La3Sn1_ICSD_77049	metal	metal
C1La3Tl1_ICSD_56402	metal	metal
C1Li1N1_ICSD_77321	insulator	insulator
C1Li1Na1O3_ICSD_36482	metal	insulator
C1Li1Na1O3_ICSD_89650	insulator	insulator
C1Li2N2_ICSD_200369	insulator	insulator
C1Li2O3_ICSD_66941	insulator	insulator
C1Li2O3_ICSD_96486	insulator	insulator
C1Li4O4_ICSD_245388	insulator	insulator
C1Li4O4_ICSD_245389	insulator	insulator
C1Li4O4_ICSD_245390	insulator	insulator
C1Li4O4_ICSD_245395	insulator	insulator
C1Li4O4_ICSD_245396	insulator	insulator
C1Li4O4_ICSD_245398	insulator	insulator
C1Li4O4_ICSD_245401	insulator	insulator
C1Li4O4_ICSD_245402	insulator	insulator
C1Li4O4_ICSD_245403	insulator	insulator
C1Li4O4_ICSD_245405	insulator	insulator
C1Lu1Rh3_ICSD_77151	metal	metal
C1Lu3Sn1_ICSD_56407	metal	metal
C1Lu3Tl1_ICSD_56404	metal	metal
C1Mg1N2_ICSD_44110	insulator	insulator
C1Mg1N2_ICSD_75039	insulator	insulator
C1Mg1Ni3_ICSD_77152	metal	metal
C1Mg1O3_ICSD_161821	insulator	insulator

Supplementary Table 200. Five-fold cross validated predictions for the metal/insulator classification (192/598).

system	calculated	predicted
C1Mg1O3_ICSD_52150	insulator	insulator
C1Mg1O3_ICSD_63663	insulator	insulator
C1Mg3Zn1_ICSD_44855	metal	metal
C1Mn1N2_ICSD_170135	insulator	insulator
C1Mn1Na3O7P1_ICSD_200789	insulator	insulator
C1Mn1O3_ICSD_28556	insulator	insulator
C1Mn3Mo3_ICSD_618261	metal	metal
C1Mn3Mo3_ICSD_76132	metal	metal
C1Mn3Sn1_ICSD_77153	metal	metal
C1Mn3W3_ICSD_618280	metal	metal
C1Mn3Zn1_ICSD_150829	metal	metal
C1Mn3_ICSD_603556	metal	metal
C1Mn3_ICSD_618248	metal	metal
C1Mn6_ICSD_187037	metal	metal
C1Mo1_ICSD_183166	metal	metal
C1Mo1_ICSD_43523	metal	metal
C1Mo1_ICSD_44987	metal	metal
C1Mo1_ICSD_618300	metal	metal
C1Mo1_ICSD_618301	metal	metal
C1Mo2Pr1Si2_ICSD_409892	metal	metal
C1Mo2_ICSD_246146	metal	metal
C1Mo2_ICSD_43669	metal	metal
C1Mo3Ni3_ICSD_76134	metal	metal
C1Mo3Re2_ICSD_618337	metal	metal
C1Mo4Ni2_ICSD_76137	metal	metal
C1N1Na1O1_ICSD_27138	insulator	insulator
C1N1Na1S1_ICSD_22273	insulator	insulator
C1N1Na1_ICSD_77172	insulator	insulator
C1N1Si1_ICSD_28391	metal	insulator
C1N1Th1_ICSD_2785	insulator	metal
C1N2Na2_ICSD_411341	insulator	insulator
C1N2Nd2O2_ICSD_82265	insulator	insulator
C1N2Ni1_ICSD_249388	insulator	metal
C1N2O2Pr2_ICSD_82264	insulator	insulator
C1N2O2Tm2_ICSD_416883	insulator	insulator
C1N2O2Y2_ICSD_245332	insulator	insulator
C1N2O2Y2_ICSD_245333	insulator	insulator
C1N2O2Yb2_ICSD_416884	metal	metal
C1N2Pb1_ICSD_16600	insulator	insulator
C1N2Pb1_ICSD_410915	insulator	insulator
C1N2Sr1_ICSD_182046	insulator	insulator
C1N2Sr1_ICSD_59860	insulator	insulator
C1N2Tl2_ICSD_417297	insulator	insulator
C1N2Zn1_ICSD_280523	insulator	insulator
C1N2_ICSD_247676	insulator	insulator
C1N2_ICSD_247677	insulator	insulator
C1N2_ICSD_247678	insulator	insulator
C1N2_ICSD_247679	insulator	insulator
C1N2_ICSD_247680	insulator	insulator
C1N3Ta2_ICSD_182350	metal	metal

Supplementary Table 201. Five-fold cross validated predictions for the metal/insulator classification (193/598).

system	calculated	predicted
C1N4S3_ICSD_62269	insulator	insulator
C1N4Si2_ICSD_93544	insulator	insulator
C1N4Sr4_ICSD_170798	insulator	insulator
C1N6O1_ICSD_174516	insulator	insulator
C1N6Si4Y2_ICSD_155158	insulator	insulator
C1Na2O3_ICSD_81013	insulator	insulator
C1Na2O3_ICSD_95549	insulator	insulator
C1Na2S3_ICSD_73094	insulator	insulator
C1Na3O7P1Sr1_ICSD_39559	insulator	insulator
C1Na4O4_ICSD_245406	insulator	insulator
C1Na4O4_ICSD_245407	insulator	insulator
C1Na4O4_ICSD_245408	insulator	insulator
C1Na4O4_ICSD_245410	insulator	insulator
C1Na4O4_ICSD_245411	insulator	insulator
C1Na4O4_ICSD_245412	insulator	insulator
C1Na4O4_ICSD_245414	insulator	insulator
C1Na4O4_ICSD_245416	insulator	insulator
C1Nb1Ru3_ICSD_77216	metal	metal
C1Nb1_ICSD_183164	metal	metal
C1Nb1_ICSD_189090	metal	metal
C1Nb1_ICSD_189092	metal	metal
C1Nb1_ICSD_189093	metal	metal
C1Nb1_ICSD_618481	metal	metal
C1Nb2P1_ICSD_43009	metal	metal
C1Nb2S1_ICSD_180619	metal	metal
C1Nb2S2_ICSD_95110	metal	metal
C1Nb2S2_ICSD_95111	metal	metal
C1Nb2Sn1_ICSD_618496	metal	metal
C1Nb2_ICSD_31973	metal	metal
C1Nb2_ICSD_43670	metal	metal
C1Nb2_ICSD_653912	metal	metal
C1Nb3Ni3_ICSD_618487	metal	metal
C1Nb4Ni2_ICSD_618488	metal	metal
C1Nb4Rh2_ICSD_618493	metal	metal
C1Nb4Zn2_ICSD_42938	metal	metal
C1Nd1Rh3_ICSD_77226	metal	metal
C1Nd3Pb1_ICSD_77225	metal	metal
C1Nd3Sn1_ICSD_77227	metal	metal
C1Nd3Tl1_ICSD_77228	metal	metal
C1Ni1O3_ICSD_61067	insulator	insulator
C1Ni1_ICSD_180457	metal	metal
C1Ni2Ta4_ICSD_618567	metal	metal
C1Ni2W4_ICSD_618587	metal	metal
C1Ni3W3_ICSD_166814	metal	metal
C1Ni3Zn1_ICSD_77230	metal	metal
C1Ni3_ICSD_17005	metal	metal
C1Ni6W6_ICSD_618591	metal	metal
C1O1S1_ICSD_33540	insulator	insulator
C1O1_ICSD_26962	insulator	insulator
C1O1_ICSD_40937	insulator	insulator

Supplementary Table 202. Five-fold cross validated predictions for the metal/insulator classification (194/598).

system	calculated	predicted
C1O2_ICSD_110775	insulator	insulator
C1O2_ICSD_110776	insulator	insulator
C1O2_ICSD_166852	insulator	insulator
C1O2_ICSD_188892	insulator	insulator
C1O2_ICSD_188893	insulator	insulator
C1O2_ICSD_290503	insulator	insulator
C1O2_ICSD_59378	insulator	insulator
C1O2_ICSD_98406	insulator	insulator
C1O2_ICSD_98407	insulator	insulator
C1O3Pb1_ICSD_247487	insulator	insulator
C1O3Rb2_ICSD_414122	insulator	insulator
C1O3Rb2_ICSD_414123	insulator	insulator
C1O3Sr1_ICSD_27293	insulator	insulator
C1O3Tl2_ICSD_4239	insulator	insulator
C1O3Zn1_ICSD_100679	insulator	insulator
C1O4Pb2_ICSD_91714	insulator	insulator
C1O4Rb4_ICSD_245429	insulator	insulator
C1O4Rb4_ICSD_245430	insulator	insulator
C1O4Rb4_ICSD_245431	insulator	insulator
C1O4Rb4_ICSD_245433	insulator	insulator
C1O4Rb4_ICSD_245437	insulator	insulator
C1O4Rb4_ICSD_245439	insulator	insulator
C1O4Rb4_ICSD_245440	insulator	insulator
C1O4Rb4_ICSD_245441	metal	insulator
C1O4Rb4_ICSD_245442	insulator	insulator
C1O4Rb4_ICSD_245444	insulator	insulator
C1O5U1_ICSD_27053	insulator	insulator
C1O5U1_ICSD_87760	insulator	insulator
C1O6P1Sn2_ICSD_50969	insulator	insulator
C1O7Si1Y2_ICSD_88878	insulator	insulator
C1Os1_ICSD_168277	insulator	metal
C1Os1_ICSD_181032	metal	metal
C1Os1_ICSD_185982	metal	insulator
C1Os1_ICSD_185989	metal	metal
C1Os1_ICSD_185996	insulator	metal
C1Os1_ICSD_43672	metal	metal
C1Os2Zr4_ICSD_618619	metal	metal
C1P1Ru2Tb1_ICSD_260482	metal	metal
C1P1V2_ICSD_181097	metal	metal
C1P1V3_ICSD_618621	metal	metal
C1P2V4_ICSD_42388	metal	metal
C1Pa1_ICSD_618623	metal	metal
C1Pb1Pd3_ICSD_108178	metal	metal
C1Pb1Pr3_ICSD_77233	metal	metal
C1Pb1Pt3_ICSD_77234	metal	metal
C1Pb1Sc3_ICSD_77235	metal	metal
C1Pb1Tb3_ICSD_77237	metal	metal
C1Pb1Ti2_ICSD_42926	metal	metal
C1Pb1Tm3_ICSD_56411	metal	metal
C1Pb1Y3_ICSD_56408	metal	metal

Supplementary Table 203. Five-fold cross validated predictions for the metal/insulator classification (195/598).

system	calculated	predicted
C1Pb1Zr2_ICSD_618634	metal	metal
C1Pd1_ICSD_181774	metal	metal
C1Pd1_ICSD_183174	metal	metal
C1Pd3Sn1_ICSD_618636	metal	metal
C1Pr1Rh3_ICSD_77304	metal	metal
C1Pr3Sn1_ICSD_77302	metal	metal
C1Pr3Tl1_ICSD_77303	metal	metal
C1Pt1_ICSD_169405	metal	metal
C1Pt1_ICSD_181113	metal	metal
C1Pt1_ICSD_185984	metal	metal
C1Pt1_ICSD_185991	metal	metal
C1Pt3Sn1_ICSD_108179	metal	metal
C1Pu1Rh3_ICSD_108181	metal	metal
C1Pu1Ru3_ICSD_108183	metal	metal
C1Pu1_ICSD_618665	metal	metal
C1Re1_ICSD_169402	metal	metal
C1Re1_ICSD_185981	metal	metal
C1Re1_ICSD_185988	metal	metal
C1Re1_ICSD_185995	metal	metal
C1Re1_ICSD_618702	metal	metal
C1Re2Si1Tb1_ICSD_50632	metal	metal
C1Re2W3_ICSD_618714	metal	metal
C1Re2_ICSD_180376	metal	metal
C1Rh1_ICSD_181773	metal	metal
C1Rh1_ICSD_183172	metal	metal
C1Rh3Sc1_ICSD_77385	metal	metal
C1Rh3Tb1_ICSD_77387	metal	metal
C1Rh3Tm1_ICSD_77388	metal	metal
C1Rh3Y1_ICSD_77389	metal	metal
C1Rh3Yb1_ICSD_77390	metal	metal
C1Ru1_ICSD_181771	insulator	metal
C1Ru1_ICSD_183169	metal	metal
C1Ru1_ICSD_188285	metal	metal
C1Ru1_ICSD_43671	metal	metal
C1Ru3Sc1_ICSD_77391	metal	metal
C1Ru3Ta1_ICSD_77392	metal	metal
C1Ru3Th1_ICSD_79241	metal	metal
C1Ru3V1_ICSD_77394	metal	metal
C1Ru3Zr1_ICSD_77282	metal	metal
C1S14_ICSD_26464	insulator	insulator
C1S1Ti2_ICSD_162459	metal	metal
C1S2Ta2_ICSD_23790	metal	metal
C1S2Ta2_ICSD_23791	metal	metal
C1S2_ICSD_33547	insulator	insulator
C1S3Tl2_ICSD_420223	insulator	insulator
C1Sc1_ICSD_181048	metal	metal
C1Sc1_ICSD_189086	metal	metal
C1Sc1_ICSD_189088	metal	metal
C1Sc1_ICSD_43524	metal	metal
C1Sc2Tl1_ICSD_160381	metal	metal

Supplementary Table 204. Five-fold cross validated predictions for the metal/insulator classification (196/598).

system	calculated	predicted
C1Sc2_ICSD_280743	metal	metal
C1Sc3Sn1_ICSD_77397	metal	metal
C1Sc3Tl1_ICSD_50164	metal	metal
C1Se2_ICSD_60374	insulator	insulator
C1Si1Ti2_ICSD_183359	metal	metal
C1Si1_ICSD_107204	insulator	insulator
C1Si1_ICSD_161173	insulator	insulator
C1Si1_ICSD_164974	insulator	metal
C1Si1_ICSD_18136	insulator	insulator
C1Si1_ICSD_182362	metal	insulator
C1Si1_ICSD_24168	insulator	insulator
C1Si1_ICSD_24169	insulator	insulator
C1Si1_ICSD_24170	insulator	insulator
C1Si1_ICSD_24261	insulator	insulator
C1Si1_ICSD_24630	insulator	insulator
C1Si1_ICSD_24631	insulator	insulator
C1Si1_ICSD_27635	insulator	insulator
C1Si1_ICSD_28303	insulator	insulator
C1Si1_ICSD_38150	insulator	insulator
C1Si1_ICSD_42858	insulator	insulator
C1Si1_ICSD_42859	insulator	insulator
C1Si1_ICSD_42862	insulator	insulator
C1Si1_ICSD_43827	insulator	insulator
C1Si3Tb5_ICSD_618785	metal	metal
C1Si3Y5_ICSD_618792	metal	metal
C1Si3Yb5_ICSD_618798	metal	metal
C1Sn1Tb3_ICSD_77403	metal	metal
C1Sn1Ti2_ICSD_161063	metal	metal
C1Sn1Tm3_ICSD_77404	metal	metal
C1Sn1Y3_ICSD_56405	metal	metal
C1Sn1Yb3_ICSD_77405	metal	metal
C1Sn1Zr2_ICSD_161068	metal	metal
C1Sn1_ICSD_182365	metal	metal
C1Sn1_ICSD_182366	metal	metal
C1Ta1_ICSD_169400	metal	metal
C1Ta1_ICSD_185986	metal	metal
C1Ta1_ICSD_185993	metal	metal
C1Ta1_ICSD_618830	metal	metal
C1Ta2_ICSD_409555	metal	metal
C1Tb2_ICSD_42762	metal	metal
C1Tb3Tl1_ICSD_77467	metal	metal
C1Tc1_ICSD_183167	metal	metal
C1Tc1_ICSD_183168	metal	metal
C1Th1_ICSD_618904	metal	metal
C1Ti1_ICSD_618951	metal	metal
C1Ti2Tl1_ICSD_618954	metal	metal
C1Ti2_ICSD_77473	metal	metal
C1Ti3Tl1_ICSD_163848	metal	metal
C1Ti3Zn3_ICSD_42932	metal	metal
C1Tl1Tm3_ICSD_77557	metal	metal

Supplementary Table 205. Five-fold cross validated predictions for the metal/insulator classification (197/598).

system	calculated	predicted
C1Tl1Y3_ICSD_42923	metal	metal
C1Tl1Yb3_ICSD_77558	metal	metal
C1Tl1Zr2_ICSD_618976	metal	metal
C1Tl1_ICSD_618972	metal	metal
C1U1_ICSD_619011	metal	metal
C1V1_ICSD_619067	metal	metal
C1V2_ICSD_601748	metal	metal
C1V3Zr3_ICSD_76130	metal	metal
C1W1_ICSD_162417	metal	metal
C1W1_ICSD_185987	metal	metal
C1W1_ICSD_185994	metal	metal
C1W1_ICSD_22258	metal	metal
C1W2_ICSD_162419	metal	metal
C1W2_ICSD_167897	metal	metal
C1W2_ICSD_167898	metal	metal
C1Y1_ICSD_181050	metal	metal
C1Y1_ICSD_183159	metal	metal
C1Y1_ICSD_183160	metal	metal
C1Y2_ICSD_96329	metal	metal
C1Zn3Zr3_ICSD_42934	metal	metal
C1Zr1_ICSD_181142	metal	metal
C1Zr1_ICSD_183162	insulator	metal
C1Zr1_ICSD_619149	metal	metal
C1_ICSD_181083	metal	insulator
C1_ICSD_182269	insulator	metal
C1_ICSD_182760	insulator	insulator
C1_ICSD_182761	insulator	insulator
C1_ICSD_186176	metal	insulator
C1_ICSD_186577	insulator	insulator
C1_ICSD_187639	metal	insulator
C1_ICSD_187640	metal	metal
C1_ICSD_188336	metal	insulator
C1_ICSD_28417	insulator	insulator
C1_ICSD_53780	insulator	insulator
C1_ICSD_655131	insulator	insulator
C1_ICSD_66466	insulator	insulator
C1_ICSD_66467	insulator	insulator
C1_ICSD_66468	insulator	insulator
C1_ICSD_66469	insulator	insulator
C1_ICSD_66470	insulator	insulator
C1_ICSD_88812	metal	insulator
C1_ICSD_88813	metal	insulator
C1_ICSD_88814	insulator	insulator
C1_ICSD_88815	metal	insulator
C1_ICSD_88817	metal	insulator
C1_ICSD_88821	insulator	insulator
C20_ICSD_185973	metal	insulator
C22F14_ICSD_411879	insulator	insulator
C2Ca11N10_ICSD_410862	insulator	insulator
C2Ca1H2O4_ICSD_151251	insulator	insulator

Supplementary Table 206. Five-fold cross validated predictions for the metal/insulator classification (198/598).

system	calculated	predicted
C2Ca1H2O5_ICSD_153499	insulator	insulator
C2Ca1H4N2O2S2_ICSD_412784	insulator	insulator
C2Ca1H4Na2O8_ICSD_9012	metal	insulator
C2Ca1H6N4O4_ICSD_9201	insulator	insulator
C2Ca1H6O7_ICSD_77096	insulator	insulator
C2Ca1H8O6P2_ICSD_261905	insulator	insulator
C2Ca1K2O6_ICSD_29442	insulator	insulator
C2Ca1Mg1O6_ICSD_66335	insulator	insulator
C2Ca1N2S2_ICSD_412783	insulator	insulator
C2Ca1O4_ICSD_246004	insulator	insulator
C2Ca1O4_ICSD_246005	metal	insulator
C2Ca1O6Sr1_ICSD_201461	insulator	insulator
C2Ca1_ICSD_24074	insulator	insulator
C2Ca1_ICSD_31092	insulator	insulator
C2Ca1_ICSD_410313	insulator	insulator
C2Ca1_ICSD_54184	insulator	insulator
C2Ca1_ICSD_54188	insulator	insulator
C2Ca1_ICSD_66663	insulator	insulator
C2Ca2Cl2H4O6_ICSD_95291	insulator	insulator
C2Ca2O10Te2_ICSD_4319	insulator	insulator
C2Cd1Cl2H8N4S2_ICSD_83430	insulator	insulator
C2Cd1Cl3H8N1_ICSD_110600	insulator	insulator
C2Cd1Cl4H12N2_ICSD_110649	insulator	insulator
C2Cd1H2O4_ICSD_151254	insulator	insulator
C2Cd1N2S2_ICSD_32597	insulator	insulator
C2Cd1N2_ICSD_20748	insulator	insulator
C2Cd1O4_ICSD_170029	insulator	insulator
C2Ce1Co1_ICSD_617336	metal	metal
C2Ce1Co1_ICSD_653742	metal	metal
C2Ce1Ni1_ICSD_617365	metal	metal
C2Ce1Rh1_ICSD_617370	metal	metal
C2Ce1Rh1_ICSD_655675	metal	metal
C2Ce1_ICSD_617325	metal	metal
C2Ce2Cl1_ICSD_422300	metal	metal
C2Ce2Re1_ICSD_617368	metal	metal
C2Ce2W1_ICSD_417669	metal	metal
C2Ce6I10_ICSD_415091	insulator	metal
C2Cl10N2Sb2_ICSD_279638	insulator	insulator
C2Cl10Sc7_ICSD_201849	metal	metal
C2Cl1F2H1O1S1_ICSD_417530	insulator	insulator
C2Cl1H5O1_ICSD_151284	insulator	insulator
C2Cl1H8N1O1Si1_ICSD_110528	insulator	insulator
C2Cl1H8N1_ICSD_110601	insulator	insulator
C2Cl1La2_ICSD_419171	metal	metal
C2Cl1Mg1Na3O6_ICSD_4237	insulator	insulator
C2Cl1N2Ni1Rb1_ICSD_380471	insulator	insulator
C2Cl2Cu1H8N12_ICSD_162996	metal	insulator
C2Cl2F1H13O8_ICSD_404343	insulator	insulator
C2Cl2H10N2Pt1_ICSD_240919	insulator	insulator
C2Cl2H12N4O4S2Zn1_ICSD_240218	insulator	insulator

Supplementary Table 207. Five-fold cross validated predictions for the metal/insulator classification (199/598).

system	calculated	predicted
C2Cl2H4Hg1N4_ICSD_405834	insulator	insulator
C2Cl2Hg3N4_ICSD_412609	insulator	insulator
C2Cl2O2Pt1_ICSD_82803	insulator	insulator
C2Cl2O4Pb2_ICSD_99805	insulator	insulator
C2Cl3Cu1H8N1_ICSD_400123	insulator	insulator
C2Cl3H8N1Pd1_ICSD_170106	insulator	insulator
C2Cl3N3S1_ICSD_86483	insulator	insulator
C2Cl4Cu1H10N2_ICSD_408537	insulator	insulator
C2Cl4N3P1_ICSD_81344	insulator	insulator
C2Cl4S2_ICSD_300291	insulator	insulator
C2Cl5La4_ICSD_418410	metal	metal
C2Cl5Pr4_ICSD_418384	metal	metal
C2Cl8N1Nb1_ICSD_402856	insulator	insulator
C2Cl8O4Sb2_ICSD_109900	insulator	insulator
C2Co1Dy1_ICSD_78553	metal	metal
C2Co1Er1_ICSD_617400	metal	metal
C2Co1Gd1_ICSD_617408	metal	metal
C2Co1H2N4_ICSD_236414	insulator	insulator
C2Co1H4O6_ICSD_59927	insulator	insulator
C2Co1H6N2O3S2_ICSD_87	insulator	insulator
C2Co1Ho1_ICSD_617411	metal	metal
C2Co1La1_ICSD_617416	insulator	metal
C2Co1N7Sr6_ICSD_162927	insulator	metal
C2Co1Nd1_ICSD_55570	metal	metal
C2Co1Nd1_ICSD_67375	metal	metal
C2Co1Pr1_ICSD_658525	metal	metal
C2Co1Pr1_ICSD_78551	insulator	metal
C2Co1Pu1_ICSD_617438	metal	metal
C2Co1Sc1_ICSD_62598	metal	metal
C2Co1Tb1_ICSD_617452	metal	metal
C2Co1Th1_ICSD_601561	metal	metal
C2Co1Y1_ICSD_57007	metal	metal
C2Co1Yb1_ICSD_617469	metal	metal
C2Co3O10Se2_ICSD_56465	insulator	insulator
C2Cr1Sc1_ICSD_80373	metal	metal
C2Cr1U1_ICSD_44493	metal	metal
C2Cr2V1_ICSD_20297	metal	metal
C2Cr3_ICSD_57009	metal	metal
C2Cr3_ICSD_653958	metal	metal
C2Cr3_ICSD_76798	metal	metal
C2Cs1F6N1O4S2_ICSD_281183	insulator	insulator
C2Cs1H1O4_ICSD_39364	insulator	insulator
C2Cs1H1_ICSD_107491	insulator	insulator
C2Cs1I3N2_ICSD_32521	insulator	insulator
C2Cs1K1_ICSD_189822	insulator	insulator
C2Cs1Na1_ICSD_189824	insulator	insulator
C2Cs1Rb1_ICSD_189823	insulator	insulator
C2Cs2F4O4Pt1_ICSD_109890	insulator	insulator
C2Cs2H2O5_ICSD_249314	insulator	insulator
C2Cs2H4O6_ICSD_39365	insulator	insulator

Supplementary Table 208. Five-fold cross validated predictions for the metal/insulator classification (200/598).

system	calculated	predicted
C2Cs2O4_ICSD_163582	insulator	insulator
C2Cs2Pd1_ICSD_94396	insulator	insulator
C2Cs2Pt1_ICSD_94397	insulator	insulator
C2Cs2_ICSD_51531	metal	insulator
C2Cs2_ICSD_51533	insulator	insulator
C2Cs2_ICSD_51534	insulator	metal
C2Cu1H6I1Se1_ICSD_174213	insulator	insulator
C2Cu1H6O7_ICSD_240734	insulator	insulator
C2Cu1K1N2_ICSD_27923	insulator	insulator
C2Cu1K1_ICSD_412037	insulator	insulator
C2Cu1K2O6_ICSD_200779	insulator	insulator
C2Cu1Na2O6_ICSD_10355	insulator	insulator
C2Cu1Rb1_ICSD_391118	insulator	insulator
C2Cu1Rb1_ICSD_412039	insulator	insulator
C2Cu3H2O8_ICSD_2934	insulator	insulator
C2Cu4H3N5_ICSD_414512	insulator	insulator
C2Cu5H8I4N2O4_ICSD_170205	insulator	insulator
C2Dy1Fe1_ICSD_617582	metal	metal
C2Dy1K1O6_ICSD_407227	insulator	insulator
C2Dy1Mo1_ICSD_617606	metal	metal
C2Dy1Ni1_ICSD_80485	metal	metal
C2Dy1W1_ICSD_617623	metal	metal
C2Dy1_ICSD_42896	metal	metal
C2Dy2Re1_ICSD_617615	metal	metal
C2Er1Fe1_ICSD_617647	metal	metal
C2Er1Mo1_ICSD_617671	metal	metal
C2Er1Ni1_ICSD_617680	metal	metal
C2Er1W1_ICSD_617691	metal	metal
C2Er1_ICSD_617625	metal	metal
C2Er2Re1_ICSD_69099	metal	metal
C2Er7I12N1_ICSD_401810	insulator	insulator
C2Eu1N2S2_ICSD_94426	insulator	insulator
C2Eu1_ICSD_380495	insulator	metal
C2Eu1_ICSD_380496	insulator	metal
C2Eu1_ICSD_617694	metal	metal
C2F1H12N6O3P1_ICSD_151292	insulator	insulator
C2F1N1O1S1_ICSD_261212	insulator	insulator
C2F1N1O1S1_ICSD_261213	insulator	insulator
C2F2O3_ICSD_414408	insulator	insulator
C2F3Li1N4Pr2_ICSD_419925	insulator	insulator
C2F4O4S1_ICSD_170271	insulator	insulator
C2F4Te2_ICSD_73583	insulator	insulator
C2F5H12N6Sb1_ICSD_79544	insulator	insulator
C2F5O4Rb1Sb2_ICSD_109634	insulator	insulator
C2F6Fe1H12O12S2_ICSD_109429	insulator	insulator
C2F6H12N2Si1_ICSD_110673	insulator	insulator
C2F6H12N2Si1_ICSD_240237	insulator	insulator
C2F6H12N6Si1_ICSD_59237	insulator	insulator
C2F6H12N6Ti1_ICSD_36529	insulator	insulator
C2F6H12O12S2V1_ICSD_68779	insulator	insulator

Supplementary Table 209. Five-fold cross validated predictions for the metal/insulator classification (201/598).

system	calculated	predicted
C2F6H18N8O2Si1_ICSD_280103	insulator	insulator
C2F6H1N1O4S2_ICSD_81464	insulator	insulator
C2F6H2N2O6S3_ICSD_59804	insulator	insulator
C2F6Na2O4Sb2_ICSD_109946	insulator	insulator
C2F6Na2O4Sb2_ICSD_200225	insulator	insulator
C2F6O3_ICSD_401780	insulator	insulator
C2F6Te2_ICSD_401990	insulator	insulator
C2Fe1H2N4_ICSD_419222	insulator	insulator
C2Fe1H4O6_ICSD_161344	insulator	insulator
C2Fe1H6N2O4_ICSD_260020	insulator	insulator
C2Fe1Ho1_ICSD_617740	metal	metal
C2Fe1N7Sr6_ICSD_409974	metal	insulator
C2Fe1Pu1_ICSD_617815	metal	metal
C2Fe1Sc1_ICSD_617820	metal	metal
C2Fe1Tb1_ICSD_617852	metal	metal
C2Fe1U1_ICSD_109116	metal	metal
C2Fe1U1_ICSD_601557	metal	metal
C2Fe5_ICSD_181367	metal	metal
C2Fe5_ICSD_43194	metal	metal
C2Ga2Ge4H10N2O12_ICSD_158855	insulator	insulator
C2Gd1H7O8S1_ICSD_163610	insulator	insulator
C2Gd1K1O6_ICSD_407226	insulator	insulator
C2Gd1Mo1_ICSD_617947	metal	metal
C2Gd1Ni1_ICSD_617952	metal	metal
C2Gd1Ru1_ICSD_80312	metal	metal
C2Gd1_ICSD_617934	metal	metal
C2Ge1Ti3_ICSD_180428	metal	metal
C2H10N2O5_ICSD_64934	insulator	insulator
C2H10N4Ni1O6_ICSD_48155	insulator	insulator
C2H10N8O6S2Zn1_ICSD_170757	insulator	insulator
C2H12N14O1_ICSD_280660	insulator	insulator
C2H12N6Ni1S2_ICSD_72515	insulator	insulator
C2H12N6O8S2Zn1_ICSD_34708	insulator	insulator
C2H14N4O8Te1_ICSD_203185	insulator	insulator
C2H18N18_ICSD_280661	insulator	insulator
C2H1K1N4Ni1S2_ICSD_1732	insulator	insulator
C2H1K1O4_ICSD_246788	insulator	insulator
C2H1K5N4_ICSD_409559	insulator	insulator
C2H1N3_ICSD_240778	insulator	insulator
C2H2K2O5_ICSD_246782	insulator	insulator
C2H2K2O6_ICSD_246300	insulator	insulator
C2H2N2O1_ICSD_30774	insulator	insulator
C2H2N4Ni1_ICSD_172908	insulator	insulator
C2H2Na2O6_ICSD_246301	insulator	insulator
C2H2O4Sr1_ICSD_260441	insulator	insulator
C2H2O4Sr1_ICSD_260442	insulator	insulator
C2H2O5Rb2_ICSD_240494	insulator	insulator
C2H3In1O5_ICSD_249482	insulator	insulator
C2H3K1O3_ICSD_77115	insulator	insulator
C2H3K1O4_ICSD_151266	insulator	insulator

Supplementary Table 210. Five-fold cross validated predictions for the metal/insulator classification (202/598).

system	calculated	predicted
C2H3Na1O5_ICSD_1296	insulator	insulator
C2H3O4Rb1_ICSD_151357	insulator	insulator
C2H4I1N2Na1O2_ICSD_67245	insulator	insulator
C2H4Mn1O6_ICSD_240902	insulator	insulator
C2H4Mn2O12P2_ICSD_281672	insulator	insulator
C2H4Mn4O14P2_ICSD_414680	metal	insulator
C2H4N2Ni1S4_ICSD_110002	insulator	insulator
C2H4N4O4_ICSD_172534	insulator	insulator
C2H4N4_ICSD_281051	insulator	insulator
C2H4O6V1_ICSD_151270	insulator	insulator
C2H4O6Zn1_ICSD_56466	insulator	insulator
C2H5N1O1_ICSD_56913	insulator	insulator
C2H5N3S2_ICSD_37081	insulator	insulator
C2H5Na3O8_ICSD_35191	insulator	insulator
C2H6Hg2O6S2_ICSD_151097	metal	insulator
C2H6K1N1O4S2_ICSD_281526	insulator	insulator
C2H6K2N2_ICSD_425116	metal	insulator
C2H6Mn1O6_ICSD_173098	insulator	insulator
C2H6N1O4Rb1S2_ICSD_281527	insulator	insulator
C2H6N2Ni1O4_ICSD_260019	insulator	insulator
C2H6N2Rb2_ICSD_425117	insulator	insulator
C2H6N4O1_ICSD_414279	insulator	insulator
C2H6N4O4Zn1_ICSD_9202	insulator	insulator
C2H6Ni1O6_ICSD_151342	insulator	insulator
C2H6O12U2_ICSD_172777	insulator	insulator
C2H6O1Se1_ICSD_171011	insulator	insulator
C2H6O4S1_ICSD_171689	insulator	insulator
C2H6O6S2Zn1_ICSD_109792	insulator	insulator
C2H6O6S2_ICSD_261156	insulator	insulator
C2H6O6Zn1_ICSD_151355	insulator	insulator
C2H6O6_ICSD_246777	insulator	insulator
C2H6S2_ICSD_249027	insulator	insulator
C2H6Se2_ICSD_249029	insulator	insulator
C2H6Te2_ICSD_249030	insulator	insulator
C2H8I1N1_ICSD_110536	insulator	insulator
C2H8I3N1Sn1_ICSD_402164	insulator	insulator
C2H8I3N1_ICSD_110538	insulator	insulator
C2H8In2O14Se2_ICSD_249780	insulator	insulator
C2H8N1O2P1S1_ICSD_110565	insulator	insulator
C2H8N1O4P1_ICSD_110400	insulator	insulator
C2H8N1O7V3_ICSD_110550	insulator	insulator
C2H8N2O3_ICSD_240961	insulator	insulator
C2H8N6O6S2_ICSD_32752	insulator	insulator
C2H8N6S2Zn1_ICSD_18146	insulator	insulator
C2H9K1Mg1O10_ICSD_63138	insulator	insulator
C2H9K1Ni1O10_ICSD_68710	insulator	insulator
C2H9N3O3_ICSD_163084	insulator	insulator
C2Hg1I1K1N2_ICSD_18198	insulator	insulator
C2Hg1N2O2_ICSD_245330	insulator	insulator
C2Hg1N2S2_ICSD_10304	insulator	insulator

Supplementary Table 211. Five-fold cross validated predictions for the metal/insulator classification (203/598).

system	calculated	predicted
C2Hg1N2Se2_ICSD_85760	insulator	insulator
C2Hg1N2_ICSD_30343	insulator	insulator
C2Hg1O4_ICSD_56459	insulator	insulator
C2Hg2N2O1_ICSD_29037	insulator	insulator
C2Ho1K1O6_ICSD_407223	insulator	insulator
C2Ho1Mo1_ICSD_618095	metal	metal
C2Ho1Ni1_ICSD_658471	metal	metal
C2Ho1_ICSD_602551	metal	metal
C2I10La6_ICSD_415092	insulator	insulator
C2I12Pr6Rb1_ICSD_417636	insulator	insulator
C2I1K1N2_ICSD_40370	insulator	insulator
C2I2Y2_ICSD_153862	metal	metal
C2I2_ICSD_246785	insulator	insulator
C2I3N2Rb1_ICSD_35411	insulator	insulator
C2I4O2Pt2_ICSD_68098	insulator	insulator
C2I7Y6_ICSD_68015	metal	metal
C2Ir1U2_ICSD_618138	metal	metal
C2Ir1_ICSD_181487	metal	metal
C2Ir1_ICSD_181488	metal	metal
C2Ir1_ICSD_181489	metal	metal
C2Ir1_ICSD_181490	metal	metal
C2K1N3_ICSD_411930	insulator	insulator
C2K1N3_ICSD_411931	insulator	insulator
C2K1N3_ICSD_411932	insulator	insulator
C2K1Nd1O6_ICSD_407225	insulator	insulator
C2K1O6Yb1_ICSD_407224	insulator	insulator
C2K2Mg1O6_ICSD_31295	insulator	insulator
C2K2N4O8_ICSD_109049	insulator	insulator
C2K2O4_ICSD_165561	insulator	insulator
C2K2O6_ICSD_412335	insulator	insulator
C2K2Pd1_ICSD_421489	insulator	insulator
C2K2Pt1_ICSD_421491	insulator	insulator
C2K2_ICSD_36142	insulator	insulator
C2K2_ICSD_89528	insulator	insulator
C2La1Li1N4_ICSD_420440	insulator	insulator
C2La1Ni1_ICSD_618179	metal	metal
C2La1Rh1_ICSD_63063	metal	metal
C2La1_ICSD_618168	metal	metal
C2La2O2_ICSD_462	insulator	insulator
C2Li2O4_ICSD_173993	insulator	insulator
C2Li2_ICSD_89535	insulator	insulator
C2Lu1Ni1_ICSD_618226	metal	metal
C2Lu1_ICSD_74671	metal	metal
C2Lu2Re1_ICSD_618232	metal	metal
C2Mg1Na2O6_ICSD_100482	insulator	insulator
C2Mg1_ICSD_88057	insulator	insulator
C2Mn5_ICSD_618246	metal	metal
C2Mo1Nd2_ICSD_417666	metal	metal
C2Mo1Pr2_ICSD_413345	metal	metal
C2Mo1Tb1_ICSD_618345	metal	metal

Supplementary Table 212. Five-fold cross validated predictions for the metal/insulator classification (204/598).

system	calculated	predicted
C2Mo1U1_ICSD_618359	metal	metal
C2Mo1Y1_ICSD_618370	metal	metal
C2N2Ni1S2_ICSD_31320	insulator	insulator
C2N2O6S2_ICSD_23342	insulator	insulator
C2N2Pb1S2_ICSD_143	insulator	insulator
C2N2S1_ICSD_25520	insulator	insulator
C2N2S2Se1_ICSD_26610	insulator	insulator
C2N2S2Sn1_ICSD_201193	insulator	insulator
C2N2S2Sr1_ICSD_94427	insulator	insulator
C2N2S2Zn1_ICSD_22359	insulator	insulator
C2N2S3_ICSD_25505	insulator	insulator
C2N2S4_ICSD_77184	insulator	insulator
C2N2S9_ICSD_77442	insulator	insulator
C2N2Se1_ICSD_173995	insulator	insulator
C2N2Se1_ICSD_27441	insulator	insulator
C2N2Se2_ICSD_171332	insulator	insulator
C2N2Se3_ICSD_171333	insulator	insulator
C2N2Zn1_ICSD_22392	insulator	insulator
C2N2_ICSD_15870	insulator	insulator
C2N3Na1_ICSD_280187	insulator	insulator
C2N3Rb1_ICSD_411934	insulator	insulator
C2N3Rb1_ICSD_411935	insulator	insulator
C2N4S4_ICSD_170226	insulator	insulator
C2N4Si1_ICSD_93543	insulator	insulator
C2Na1_ICSD_186177	metal	insulator
C2Na2O4_ICSD_171459	insulator	insulator
C2Na2Pd1_ICSD_411388	insulator	insulator
C2Na2Pt1_ICSD_50173	insulator	insulator
C2Na2_ICSD_28066	insulator	insulator
C2Na2_ICSD_95835	insulator	insulator
C2Nd1Ni1_ICSD_618540	metal	metal
C2Nd1Rh1_ICSD_63064	metal	metal
C2Nd1_ICSD_63560	metal	metal
C2Nd2W1_ICSD_417667	metal	metal
C2Ni1Pr1_ICSD_80482	metal	metal
C2Ni1Pu1_ICSD_618560	metal	metal
C2Ni1Sc1_ICSD_618562	metal	metal
C2Ni1Tb1_ICSD_618574	metal	metal
C2Ni1Tm1_ICSD_57054	metal	metal
C2Ni1U1_ICSD_601562	metal	metal
C2Ni1Y1_ICSD_618595	metal	metal
C2Ni1Yb1_ICSD_618600	metal	metal
C2O4Pb1_ICSD_109830	insulator	insulator
C2O4Rb2_ICSD_163587	insulator	insulator
C2O4Rb2_ICSD_165562	insulator	insulator
C2O4Sn1_ICSD_150101	insulator	insulator
C2O4Tl2_ICSD_170127	insulator	insulator
C2O6Rb2_ICSD_412971	insulator	insulator
C2Os1U2_ICSD_618614	metal	metal
C2Os1_ICSD_168279	metal	metal

Supplementary Table 213. Five-fold cross validated predictions for the metal/insulator classification (205/598).

system	calculated	predicted
C2Os1_ICSD_168280	insulator	metal
C2Pa1_ICSD_618625	metal	metal
C2Pd1Rb2_ICSD_94394	insulator	insulator
C2Pr1Rh1_ICSD_618649	metal	metal
C2Pr1_ICSD_602766	metal	metal
C2Pr2Re1_ICSD_69097	metal	metal
C2Pr2W1_ICSD_417668	metal	metal
C2Pt1Rb2_ICSD_94395	insulator	insulator
C2Pt1U2_ICSD_618659	metal	metal
C2Pu1W1_ICSD_618691	metal	metal
C2Rb2_ICSD_51529	insulator	insulator
C2Re1U1_ICSD_618709	metal	metal
C2Re1Y2_ICSD_69098	metal	metal
C2Re1_ICSD_184660	metal	metal
C2Re1_ICSD_184661	metal	metal
C2Re1_ICSD_184662	metal	metal
C2Re1_ICSD_184663	metal	metal
C2Re1_ICSD_184664	metal	metal
C2Rh1U2_ICSD_604010	metal	metal
C2Ru1U2_ICSD_603999	metal	metal
C2Ru1U2_ICSD_618746	metal	metal
C2S2Zr4_ICSD_43674	metal	metal
C2Si1Ti3_ICSD_88579	metal	metal
C2Si1_ICSD_187720	metal	insulator
C2Si1_ICSD_187721	metal	insulator
C2Sn1Ti3_ICSD_160572	metal	metal
C2Sr1_ICSD_410317	insulator	insulator
C2Sr1_ICSD_91051	insulator	insulator
C2Tb1W1_ICSD_618888	metal	metal
C2Tb1_ICSD_618871	metal	metal
C2Th1_ICSD_26568	metal	metal
C2Th1_ICSD_618912	metal	metal
C2Th1_ICSD_77470	metal	metal
C2Tm1W1_ICSD_618985	metal	metal
C2Tm1_ICSD_23687	metal	metal
C2U1V1_ICSD_23742	metal	metal
C2U1W1_ICSD_619032	metal	metal
C2U1W1_ICSD_68116	metal	metal
C2U1_ICSD_168167	metal	metal
C2U1_ICSD_604087	metal	metal
C2U1_ICSD_619006	insulator	metal
C2W1Y1_ICSD_619103	metal	metal
C2Y1_ICSD_95963	metal	metal
C2Yb1_ICSD_619131	insulator	metal
C3Ca2H1Li1_ICSD_188224	insulator	insulator
C3Ca2K2O9_ICSD_83431	insulator	insulator
C3Ca2Na2O9_ICSD_36237	insulator	insulator
C3Ca3Cl2_ICSD_33818	insulator	insulator
C3Cd1K1N3O3_ICSD_4097	insulator	insulator
C3Cd1N3Rb1S3_ICSD_14147	insulator	insulator

Supplementary Table 214. Five-fold cross validated predictions for the metal/insulator classification (206/598).

system	calculated	predicted
C3Ce1O13P1Sr3_ICSD_76608	insulator	insulator
C3Ce2F1Na1O9_ICSD_183233	insulator	insulator
C3Ce2Mo2_ICSD_417827	metal	metal
C3Ce2Ni5_ICSD_167515	metal	metal
C3Ce2_ICSD_74661	metal	metal
C3Cl1H10N1O1_ICSD_110267	insulator	insulator
C3Cl1H10N1O4_ICSD_110284	insulator	insulator
C3Cl1H10N1O4_ICSD_110285	insulator	insulator
C3Cl1H9Si1_ICSD_171369	insulator	insulator
C3Cl3Cs1O3Os1_ICSD_39588	insulator	insulator
C3Cl3Ga1H9O1P1_ICSD_173260	insulator	insulator
C3Cl3H10N1Sn1_ICSD_170096	insulator	insulator
C3Cl3N3_ICSD_86484	insulator	insulator
C3Cl5Gd6_ICSD_202547	metal	metal
C3Cl6H5O5Ru1Sb1_ICSD_24785	insulator	insulator
C3Cl7Mo1O1S2_ICSD_413426	insulator	insulator
C3Co1H3K1O6_ICSD_181922	insulator	insulator
C3Co1H3K1O6_ICSD_181923	insulator	insulator
C3Cr1Sc2_ICSD_39202	metal	metal
C3Cr2Dy2_ICSD_617492	metal	metal
C3Cr2Er2_ICSD_617494	metal	metal
C3Cr2Gd2_ICSD_617507	metal	metal
C3Cr2Ho2_ICSD_62083	metal	metal
C3Cr2N6_ICSD_421391	insulator	insulator
C3Cr2Tb2_ICSD_617537	metal	metal
C3Cr2Y2_ICSD_617562	metal	metal
C3Cr2Yb2_ICSD_602482	metal	metal
C3Cs1N3Se3_ICSD_4101	insulator	insulator
C3Cs2O9Sr2_ICSD_169231	insulator	insulator
C3Cu2H2N3O1Rb1_ICSD_281775	insulator	insulator
C3Dy2Fe17_ICSD_617591	metal	metal
C3Dy2_ICSD_2448	metal	metal
C3Er2Fe17_ICSD_617656	metal	metal
C3Er2Mo2_ICSD_617669	metal	metal
C3Er2_ICSD_86291	metal	metal
C3Eu4I3Li1N6_ICSD_170262	insulator	insulator
C3F1H7N1O5Sn1_ICSD_250206	insulator	insulator
C3F1La2Na1O9_ICSD_183234	insulator	insulator
C3F3H12N1_ICSD_110221	insulator	insulator
C3F3H6N3O2S1_ICSD_152106	insulator	insulator
C3F6_ICSD_151184	insulator	insulator
C3Fe17Ho2_ICSD_617748	metal	metal
C3Fe17Tb2_ICSD_657171	metal	metal
C3Fe17Y2_ICSD_617904	metal	metal
C3Fe1N1O4Tl1_ICSD_66317	insulator	insulator
C3Fe7_ICSD_76830	metal	metal
C3Ga1N1Si3_ICSD_183049	insulator	insulator
C3Gd2Mo2_ICSD_409822	metal	metal
C3Gd2_ICSD_602774	metal	metal
C3H10I1N1_ICSD_249166	insulator	insulator

Supplementary Table 215. Five-fold cross validated predictions for the metal/insulator classification (207/598).

system	calculated	predicted
C3H20N4O10_ICSD_281534	insulator	insulator
C3H2O4_ICSD_109620	insulator	insulator
C3H3Nd1O6_ICSD_260292	metal	insulator
C3H3O6Sc1_ICSD_109661	insulator	insulator
C3H3O6Sc1_ICSD_281595	insulator	insulator
C3H3O6Y1_ICSD_109739	insulator	insulator
C3H5K1N2O5_ICSD_260200	insulator	insulator
C3H6N1Sb1_ICSD_170722	insulator	insulator
C3H6N4O3_ICSD_30562	insulator	insulator
C3H6O3_ICSD_151224	insulator	insulator
C3H7N3O5_ICSD_80262	insulator	insulator
C3H8N2O1_ICSD_110759	metal	insulator
C3H9N3O2_ICSD_109968	insulator	insulator
C3Hg1K1N3O3_ICSD_100116	insulator	insulator
C3Hg1K1N3S3_ICSD_20752	insulator	insulator
C3Hg1K1N3S3_ICSD_85761	insulator	insulator
C3Hg1N3Rb1S3_ICSD_101	insulator	insulator
C3Ho2Mo2_ICSD_88511	metal	metal
C3Ho2_ICSD_618085	metal	metal
C3I6O1Y7_ICSD_67889	metal	metal
C3Ir1_ICSD_181492	metal	metal
C3Ir5_ICSD_181485	metal	metal
C3K4O11U1_ICSD_69130	insulator	insulator
C3La2Ni5_ICSD_655407	metal	metal
C3La2_ICSD_618149	metal	metal
C3Lu2N6_ICSD_240311	insulator	insulator
C3Lu2_ICSD_618221	metal	metal
C3Mg2_ICSD_71941	insulator	insulator
C3Mo2U2_ICSD_618357	metal	metal
C3N4_ICSD_246659	insulator	insulator
C3N4_ICSD_41950	insulator	insulator
C3N4_ICSD_41952	insulator	insulator
C3N4_ICSD_83261	insulator	insulator
C3N4_ICSD_83263	insulator	insulator
C3N4_ICSD_83265	insulator	insulator
C3N4_ICSD_97564	insulator	insulator
C3N6Yb2_ICSD_416892	metal	insulator
C3Nb4Si1_ICSD_169044	metal	metal
C3Nb4_ICSD_42758	metal	metal
C3Nb8Zn4_ICSD_108770	metal	metal
C3Nd2_ICSD_2447	metal	metal
C3O11Tl4U1_ICSD_61345	insulator	insulator
C3O1S7_ICSD_165374	insulator	insulator
C3O2_ICSD_411461	insulator	insulator
C3O9Rb2Sr2_ICSD_169232	insulator	insulator
C3Os2_ICSD_168275	insulator	metal
C3Os2_ICSD_168276	metal	insulator
C3Pr2_ICSD_2444	metal	metal
C3Pr2_ICSD_74662	metal	metal
C3Pu2_ICSD_24620	metal	metal

Supplementary Table 216. Five-fold cross validated predictions for the metal/insulator classification (208/598).

system	calculated	predicted
C3Re2U2_ICSD_618707	metal	metal
C3S8_ICSD_53591	insulator	insulator
C3Sc2_ICSD_77396	metal	metal
C3Sc4_ICSD_42760	insulator	metal
C3Tb2_ICSD_74663	metal	metal
C3Th2_ICSD_618903	metal	metal
C3Tm2_ICSD_86292	metal	metal
C3U2W2_ICSD_619030	metal	metal
C3U2_ICSD_77561	metal	metal
C3Y2_ICSD_601153	metal	metal
C3Yb2_ICSD_86293	metal	metal
C4Ca1Cu1H4O8_ICSD_151315	insulator	insulator
C4Ca1Mg3O12_ICSD_201729	insulator	insulator
C4Ca1N6_ICSD_411362	insulator	insulator
C4Cd1Cl2H8N2_ICSD_162987	insulator	insulator
C4Cd1H20N2O12S4_ICSD_406374	insulator	insulator
C4Cd1H4O6_ICSD_249685	insulator	insulator
C4Cd1H6O2S4_ICSD_110011	insulator	insulator
C4Cd1H8N6S4_ICSD_170723	insulator	insulator
C4Cd1Hg1N4S4_ICSD_4102	insulator	insulator
C4Cd1Hg1N4Se4_ICSD_249203	insulator	insulator
C4Cd1K2N4_ICSD_23994	insulator	insulator
C4Cd1N4S4Zn1_ICSD_280039	insulator	insulator
C4Cd1N4Se4Zn1_ICSD_249202	insulator	insulator
C4Cd1N6_ICSD_413333	insulator	insulator
C4Cd1N6_ICSD_413334	insulator	insulator
C4Cl1N3_ICSD_246790	insulator	insulator
C4Cl2Cs2N4Pt1_ICSD_421674	insulator	insulator
C4Cl2Cu1H8N8_ICSD_250269	metal	insulator
C4Cl2F12N3P2V1_ICSD_68388	insulator	insulator
C4Cl2F6Te1_ICSD_260283	insulator	insulator
C4Cl2H12O6V2_ICSD_168381	insulator	insulator
C4Cl2H16N8Ni1S4_ICSD_61123	insulator	insulator
C4Cl2K2N4Pt1_ICSD_413704	insulator	insulator
C4Cl2Mg2Na6O12_ICSD_27790	insulator	insulator
C4Cl2N4Pt1Rb2_ICSD_419370	insulator	insulator
C4Cl2O4Os1_ICSD_171560	insulator	insulator
C4Cl2O4Os1_ICSD_171561	insulator	insulator
C4Cl3Co1Ga1K1O4_ICSD_410883	insulator	insulator
C4Cl3Co1O4Sn1_ICSD_69685	insulator	insulator
C4Cl4Fe1H12N1_ICSD_159487	insulator	insulator
C4Cl4H12S2Sn1_ICSD_170801	insulator	insulator
C4Cl4H14N2Sn1_ICSD_170799	insulator	insulator
C4Cl4N4S4_ICSD_48164	insulator	insulator
C4Cl6H16N2Sn1_ICSD_110603	insulator	insulator
C4Cl6H16N2Ti1_ICSD_281532	insulator	insulator
C4Co1Cs1O4_ICSD_31360	insulator	insulator
C4Co1F3O4Si1_ICSD_16069	insulator	insulator
C4Co1H4N2O10V4_ICSD_158983	metal	insulator
C4Co1Hg1N4S4_ICSD_36062	insulator	insulator

Supplementary Table 217. Five-fold cross validated predictions for the metal/insulator classification (209/598).

system	calculated	predicted
C4Co1Hg1N4Se4_ICSD_172058	insulator	insulator
C4Co1Li1O4_ICSD_30854	insulator	insulator
C4Co1N6_ICSD_85618	insulator	insulator
C4Co1Na1O4_ICSD_30855	insulator	insulator
C4Co1O4Tl1_ICSD_6011	insulator	insulator
C4Co1Sc3_ICSD_173167	metal	metal
C4Co1Sc3_ICSD_236391	metal	metal
C4Cr1H4K1O10_ICSD_172524	insulator	insulator
C4Cr1H4O10Rb1_ICSD_281565	insulator	insulator
C4Cr1H8N6S4_ICSD_69067	insulator	insulator
C4Cr4U1_ICSD_60822	metal	metal
C4Cs1H12O14V1_ICSD_109663	insulator	insulator
C4Cs2H2N4O2Pt1_ICSD_418911	insulator	insulator
C4Cs2O4_ICSD_154357	insulator	insulator
C4Cu1H4Na2O10_ICSD_40090	metal	insulator
C4Cu1H4O8Sr1_ICSD_150120	insulator	insulator
C4Cu1H8O8_ICSD_170726	metal	insulator
C4Cu1Hg1N4S4_ICSD_174608	metal	insulator
C4Cu1K3N4_ICSD_76933	insulator	insulator
C4Dy4Ni13_ICSD_617613	metal	metal
C4Er2Fe1_ICSD_42968	metal	metal
C4Er2Mn1_ICSD_73169	metal	metal
C4Er3_ICSD_617626	metal	metal
C4Er4Ni13_ICSD_617674	metal	metal
C4F12Ge4S6_ICSD_62223	insulator	insulator
C4F12Ge4Se6_ICSD_62224	insulator	insulator
C4F12H17O20S4V1_ICSD_201678	insulator	insulator
C4F12N2O8S4Sr1_ICSD_163023	insulator	insulator
C4F12P4_ICSD_23899	insulator	insulator
C4F1H12N1O4Os1_ICSD_110743	insulator	insulator
C4F1H12O5Re1_ICSD_415419	insulator	insulator
C4F2H13N1_ICSD_155703	insulator	insulator
C4F3H9Si1_ICSD_162079	insulator	insulator
C4F6Hg1_ICSD_165635	insulator	insulator
C4F6Hg2O4_ICSD_2022	insulator	insulator
C4Fe1H12N10S4_ICSD_170705	insulator	insulator
C4Fe1H12O6S4_ICSD_203234	insulator	insulator
C4Fe1Hg1N4S4_ICSD_87888	insulator	insulator
C4Fe1Ho2_ICSD_617747	metal	metal
C4Fe1K2O4_ICSD_200025	insulator	insulator
C4Fe1Lu2_ICSD_617765	metal	metal
C4Fe1Sc3_ICSD_72863	metal	metal
C4Fe1Tb2_ICSD_617861	metal	metal
C4Fe1Y2_ICSD_617900	metal	metal
C4Fe1Yb2_ICSD_617906	metal	metal
C4Fe2H2O6_ICSD_109774	insulator	insulator
C4H10N8Ni1S2_ICSD_110013	insulator	insulator
C4H11N1O10_ICSD_249174	insulator	insulator
C4H12I1N1O3_ICSD_416527	insulator	insulator
C4H12I1N1_ICSD_55080	insulator	insulator

Supplementary Table 218. Five-fold cross validated predictions for the metal/insulator classification (210/598).

system	calculated	predicted
C4H12I3O2S2Tl1_ICSD_170738	insulator	insulator
C4H12Mn1N10_ICSD_110766	insulator	insulator
C4H12N2O2Se1_ICSD_402259	insulator	insulator
C4H12N2O2_ICSD_150985	insulator	insulator
C4H12O3P2_ICSD_170836	insulator	insulator
C4H12O4Si1_ICSD_171152	insulator	insulator
C4H1O4Rb1_ICSD_172323	insulator	insulator
C4H20N4Ni1O12Pd1_ICSD_417562	insulator	insulator
C4H20N4O12Pd1Zn1_ICSD_417563	insulator	insulator
C4H20N4O12Pt1Zn1_ICSD_417565	insulator	insulator
C4H2O4_ICSD_151146	insulator	insulator
C4H2O6Sn2_ICSD_96547	insulator	insulator
C4H4Mo2O8_ICSD_151313	insulator	insulator
C4H4N4O2Pt1_ICSD_409975	insulator	insulator
C4H4N4_ICSD_170078	insulator	insulator
C4H4Na5O14Sc1_ICSD_411988	insulator	insulator
C4H5Na3O8_ICSD_109935	insulator	insulator
C4H6Hg1N4O2_ICSD_418607	insulator	insulator
C4H6N2S2Sn1_ICSD_171668	insulator	insulator
C4H6O7Sr1_ICSD_109771	insulator	insulator
C4H7O11Y1_ICSD_109808	insulator	insulator
C4H8I4Sn1_ICSD_110024	insulator	insulator
C4H8Mg1N6O4_ICSD_414648	insulator	insulator
C4H8N1O10Ti1_ICSD_109869	metal	insulator
C4H8N2O4_ICSD_172054	insulator	insulator
C4H8O4Sn1_ICSD_151276	insulator	insulator
C4H8O8Zn1_ICSD_109772	insulator	insulator
C4H9N1O6Zn1_ICSD_240733	insulator	insulator
C4H9N1Se2_ICSD_163646	insulator	insulator
C4Hg1K2N4_ICSD_62084	insulator	insulator
C4Hg1Mn1N4S4_ICSD_87889	insulator	insulator
C4Hg1Mn1N4Se4_ICSD_240722	insulator	insulator
C4Hg1N4Rb2_ICSD_100685	insulator	insulator
C4Hg1N4S4Zn1_ICSD_280028	insulator	insulator
C4Hg1N4Se4Zn1_ICSD_188764	insulator	insulator
C4Ho3_ICSD_618074	metal	metal
C4Ho4Ni13_ICSD_618100	metal	metal
C4I15La10_ICSD_415094	metal	insulator
C4I2K2N4Pt1_ICSD_413706	insulator	insulator
C4I2N4Pt1Rb2_ICSD_421673	insulator	insulator
C4I2O4Ru1_ICSD_23919	insulator	insulator
C4Ir1Sc3_ICSD_415137	metal	metal
C4Ir1Sc3_ICSD_657402	metal	metal
C4Ir1_ICSD_181493	metal	metal
C4Ir1_ICSD_181496	metal	metal
C4Ir1_ICSD_181497	metal	metal
C4Ir1_ICSD_181498	metal	metal
C4K1N3_ICSD_77046	insulator	insulator
C4K1N4Tl1_ICSD_170125	insulator	insulator
C4K2N4Ni1_ICSD_24099	insulator	insulator

Supplementary Table 219. Five-fold cross validated predictions for the metal/insulator classification (211/598).

system	calculated	predicted
C4K2N4Pd1S4_ICSD_9473	insulator	insulator
C4K2N4Pd1_ICSD_413899	insulator	insulator
C4K2N4Pt1_ICSD_413898	insulator	insulator
C4K2N4Zn1_ICSD_23993	insulator	insulator
C4La1N8Rb1Si1_ICSD_419337	insulator	insulator
C4Li2O4_ICSD_154354	insulator	insulator
C4Mg1N6_ICSD_411361	insulator	insulator
C4Mg2Na6O16S1_ICSD_158962	insulator	insulator
C4Mg2Na6O16S1_ICSD_27792	insulator	insulator
C4Mn1N1O5_ICSD_15221	insulator	insulator
C4Mn1N6_ICSD_92075	insulator	insulator
C4N3Na1_ICSD_31926	insulator	insulator
C4N4Ni1Tl2_ICSD_249564	insulator	insulator
C4N4Ni1Zn1_ICSD_169760	insulator	insulator
C4N4Pd1Tl2_ICSD_249565	insulator	insulator
C4N4Pd1Zn1_ICSD_169759	insulator	insulator
C4N4Pt1Rb2_ICSD_171492	insulator	insulator
C4N4Pt1Tl2_ICSD_202570	insulator	insulator
C4N4Pt1Zn1_ICSD_169758	insulator	insulator
C4N6Ni1_ICSD_85617	insulator	insulator
C4N6Pb1_ICSD_412288	insulator	insulator
C4N6Sr1_ICSD_411363	insulator	insulator
C4Na2O4_ICSD_154355	insulator	insulator
C4Na2O8Sn1_ICSD_388	insulator	insulator
C4Ni13Tb4_ICSD_618575	metal	metal
C4Ni13Y4_ICSD_618594	metal	metal
C4Ni13Yb4_ICSD_618599	metal	metal
C4Ni1O4_ICSD_74196	insulator	insulator
C4Ni1Sc3_ICSD_657399	metal	metal
C4O4Pb1_ICSD_248144	insulator	insulator
C4O4Rb2_ICSD_154356	insulator	insulator
C4O4Ru1_ICSD_72577	insulator	insulator
C4O4Tl2_ICSD_260404	insulator	insulator
C4Os1Sc3_ICSD_420075	metal	metal
C4Os1Sc3_ICSD_420076	metal	metal
C4Os1Sc3_ICSD_657401	metal	metal
C4Rh1Sc3_ICSD_415136	metal	metal
C4Rh1Sc3_ICSD_657400	metal	metal
C4Ru1Sc3_ICSD_420074	metal	metal
C4Ru1Sc3_ICSD_72864	metal	metal
C4Sc3_ICSD_71145	metal	metal
C4U1W4_ICSD_42959	metal	metal
C4Y3_ICSD_619109	metal	metal
C5Ca4Ni3_ICSD_71440	metal	metal
C5Cd1Fe1N6O1_ICSD_109420	insulator	insulator
C5Cl1Mn1O5_ICSD_16135	insulator	insulator
C5Cl1O5Re1_ICSD_35625	insulator	insulator
C5Cl1O5Tc1_ICSD_167772	insulator	insulator
C5Cu1Fe1N6O1_ICSD_414021	insulator	insulator
C5Cu1H11N1O6_ICSD_110500	metal	insulator

Supplementary Table 220. Five-fold cross validated predictions for the metal/insulator classification (212/598).

system	calculated	predicted
C5Cu2H8N4_ICSD_281777	insulator	insulator
C5Dy1H9N2O8_ICSD_151327	insulator	insulator
C5Er1H9N2O8_ICSD_151326	insulator	insulator
C5Fe1H8N6O4_ICSD_83731	insulator	insulator
C5Fe1N6O1Tl2_ICSD_14184	insulator	insulator
C5Fe1O5_ICSD_23918	insulator	insulator
C5Fe1O5_ICSD_300118	insulator	insulator
C5H10O5_ICSD_151281	insulator	insulator
C5H12N2O1_ICSD_150997	insulator	insulator
C5Ho4_ICSD_84871	metal	metal
C5I1Mn1O5_ICSD_71878	insulator	insulator
C5I1O5Re1_ICSD_71219	insulator	insulator
C5I1O5Tc1_ICSD_85335	insulator	insulator
C5Ir4_ICSD_181486	insulator	metal
C5N4_ICSD_2130	insulator	insulator
C5Nb6_ICSD_167353	metal	metal
C5Ni2Yb4_ICSD_73156	metal	metal
C5Ru6Th2_ICSD_39204	metal	metal
C5Tb4_ICSD_84870	metal	metal
C5Ti8_ICSD_20822	metal	metal
C5V6_ICSD_71098	metal	metal
C5Y4_ICSD_84869	metal	metal
C60Eu6_ICSD_88616	metal	metal
C60K6_ICSD_66879	insulator	insulator
C60Rb6_ICSD_66880	insulator	insulator
C60Sr3_ICSD_75354	insulator	metal
C60Sr6_ICSD_75356	metal	insulator
C60_ICSD_74523	insulator	insulator
C60_ICSD_95370	insulator	insulator
C60_ICSD_96620	insulator	insulator
C68Co2O8_ICSD_166882	insulator	insulator
C6Ca2Cu1H6O12_ICSD_151316	metal	insulator
C6Cd1Cs2Fe1N6_ICSD_151696	insulator	insulator
C6Cd1N6Pd1_ICSD_6093	insulator	insulator
C6Cd2Fe1N6_ICSD_417826	insulator	insulator
C6Cd2N6Os1_ICSD_417821	insulator	insulator
C6Cd2N6Ru1_ICSD_417822	insulator	insulator
C6Cl2H18N4S2Se2_ICSD_151024	insulator	insulator
C6Cl3H21N2_ICSD_402690	insulator	insulator
C6Cl4La6_ICSD_416906	metal	metal
C6Cl6H16O14Ru3_ICSD_413219	insulator	insulator
C6Co1Cr1H18N12_ICSD_806	metal	insulator
C6Co1Cs2Li1N6_ICSD_60539	insulator	insulator
C6Co1Cs2N6Rb1_ICSD_411128	insulator	insulator
C6Co1Er1H8N6O4_ICSD_171622	insulator	insulator
C6Co1Er1N6_ICSD_171616	insulator	insulator
C6Co1Fe1H18N12_ICSD_411630	insulator	metal
C6Co1Fe1K2N6_ICSD_28669	metal	insulator
C6Co1H3N6_ICSD_28502	insulator	insulator
C6Co1K3N6_ICSD_23048	insulator	insulator

Supplementary Table 221. Five-fold cross validated predictions for the metal/insulator classification (213/598).

system	calculated	predicted
C6Co1Li1N6Rb2_ICSD_83393	insulator	insulator
C6Co2Fe1N6_ICSD_28670	metal	metal
C6Co2H18N12_ICSD_2561	insulator	insulator
C6Cr1Cs2Li1N6_ICSD_68068	insulator	insulator
C6Cr1O6_ICSD_8286	insulator	insulator
C6Cr21Mo2_ICSD_156148	metal	metal
C6Cr21W2_ICSD_156149	metal	metal
C6Cr23_ICSD_154719	metal	metal
C6Cr23_ICSD_181714	metal	metal
C6Cr3W20_ICSD_617559	metal	metal
C6Cs2Fe1Li1N6_ICSD_1001	metal	insulator
C6Cs2Fe1Li1N6_ICSD_60542	insulator	insulator
C6Cs2Fe1N6Ni1_ICSD_151706	insulator	insulator
C6Cs2Ir1Li1N6_ICSD_9032	insulator	insulator
C6Cs2N9Na1_ICSD_407809	insulator	insulator
C6Cu1Fe1K2N6_ICSD_28654	insulator	insulator
C6Cu1Fe1K2N6_ICSD_99499	insulator	insulator
C6Cu1Fe1Li2N6_ICSD_28656	insulator	insulator
C6Cu1Fe1N6Na2_ICSD_28657	insulator	insulator
C6Cu1Fe1N6Rb2_ICSD_28659	insulator	insulator
C6Cu1H4N8O4_ICSD_168724	metal	insulator
C6Cu1H8N6O2_ICSD_156682	metal	metal
C6Cu2F6O6_ICSD_260351	insulator	insulator
C6Cu2Fe1N6_ICSD_28653	metal	insulator
C6Dy12I17_ICSD_416955	metal	insulator
C6Er11Ni60_ICSD_617678	metal	metal
C6Eu1N9_ICSD_391267	insulator	insulator
C6Eu1_ICSD_169041	metal	metal
C6F12Fe1O6Sb2_ICSD_280005	insulator	insulator
C6F12O6Os1Sb2_ICSD_152388	insulator	insulator
C6F12O6Ru1Sb2_ICSD_152386	insulator	insulator
C6F5H3O3Se1_ICSD_410719	insulator	insulator
C6F6H24N2O2Sn1_ICSD_110229	insulator	insulator
C6F6Na4O12Sn4_ICSD_109824	insulator	insulator
C6Fe1H4N6_ICSD_30559	insulator	insulator
C6Fe1K2N6Ni1_ICSD_28667	insulator	insulator
C6Fe1K3N6_ICSD_31883	insulator	insulator
C6Fe1Mn2N6_ICSD_417824	insulator	insulator
C6Fe1N6Ni2_ICSD_28668	metal	insulator
C6Fe1N6Pb2_ICSD_51493	insulator	insulator
C6Fe1N6Sn2_ICSD_51494	insulator	insulator
C6Fe21Mo2_ICSD_156150	metal	metal
C6Fe21W2_ICSD_156151	metal	metal
C6Fe23_ICSD_168294	metal	metal
C6Fe23_ICSD_187035	metal	metal
C6Gd1N9_ICSD_416002	insulator	insulator
C6Gd3Mn2_ICSD_73213	metal	metal
C6H12N10O2S2Zn1_ICSD_405377	insulator	insulator
C6H12N6O8Pt1_ICSD_281359	insulator	insulator
C6H18I2Pb2_ICSD_405865	insulator	insulator

Supplementary Table 222. Five-fold cross validated predictions for the metal/insulator classification (214/598).

system	calculated	predicted
C6H18N3O1P1_ICSD_409222	insulator	insulator
C6H20N2S6_ICSD_110278	insulator	insulator
C6H8Ho1K1N6O4Ru1_ICSD_281332	insulator	insulator
C6I4O6Os2_ICSD_68901	insulator	insulator
C6K3Mn1N6_ICSD_24933	insulator	insulator
C6La1N9_ICSD_391272	insulator	insulator
C6Mn20Mo3_ICSD_618260	metal	metal
C6Mn20W3_ICSD_618279	metal	metal
C6Mn23_ICSD_154720	metal	metal
C6Mn2N6Os1_ICSD_417825	insulator	insulator
C6Mn2N6Ru1_ICSD_417823	insulator	insulator
C6Mn2Tb3_ICSD_73214	metal	metal
C6Mo1O6_ICSD_30809	insulator	insulator
C6N9Nd1_ICSD_391269	insulator	insulator
C6N9Pr1_ICSD_391270	insulator	insulator
C6Na2O6_ICSD_170805	insulator	insulator
C6O6W1_ICSD_300206	insulator	insulator
C6O8Tl4_ICSD_260372	insulator	insulator
C6Yb1_ICSD_601565	metal	metal
C7Ho4_ICSD_74817	insulator	metal
C7Ho4_ICSD_83381	metal	metal
C7Ir3_ICSD_181491	metal	metal
C7Lu4_ICSD_83382	metal	metal
C7Lu5Re2_ICSD_406416	metal	metal
C7Re2Sc5_ICSD_72382	metal	metal
C7V8_ICSD_22177	metal	metal
C7Y4_ICSD_86049	metal	metal
C8Ca1H16K2O24Zr1_ICSD_245276	insulator	insulator
C8Cd1H10O12_ICSD_249683	insulator	insulator
C8Cl1H20Li1O4_ICSD_151063	insulator	insulator
C8Cl4H24N2Pd1_ICSD_59900	insulator	insulator
C8Cl5La8_ICSD_419172	metal	metal
C8Co1H24Li1N8O12_ICSD_110738	insulator	insulator
C8Co2O8_ICSD_16567	insulator	insulator
C8Cs1_ICSD_74641	metal	insulator
C8Cu1H16N8Ni1_ICSD_411632	metal	insulator
C8Cu3H8Na2O16_ICSD_171669	insulator	insulator
C8F12O8Ru2_ICSD_171336	insulator	insulator
C8F12Pt1S4_ICSD_165606	insulator	insulator
C8F6H24N2Os1_ICSD_151185	metal	insulator
C8Ga2H26N4_ICSD_163002	insulator	insulator
C8Ge2H24In1P1_ICSD_163057	insulator	insulator
C8H16Hg1N6S2_ICSD_260899	insulator	insulator
C8H24Mn1N2Se10Sn4_ICSD_249783	insulator	insulator
C8H24N2O4S1_ICSD_52027	insulator	insulator
C8H4K2Ni1O6S4_ICSD_165299	insulator	insulator
C8I2Mo2O8_ICSD_36	insulator	insulator
C8K1O8Y1_ICSD_170216	insulator	insulator
C8K1_ICSD_70020	metal	metal
C8K5N8Nb1_ICSD_16458	insulator	insulator

Supplementary Table 223. Five-fold cross validated predictions for the metal/insulator classification (215/598).

system	calculated	predicted
C8O4Se2_ICSD_172229	insulator	insulator
C8Rb1_ICSD_200563	metal	metal
C8Re3U5_ICSD_65202	metal	metal
C8_ICSD_20351	insulator	insulator
C9Fe2O9_ICSD_31030	insulator	insulator
C9La7Os4_ICSD_419389	metal	metal
Ca10.7184_ICSD_163531	metal	metal
Ca10Li1Mg1Sb9_ICSD_171161	insulator	insulator
Ca10O24P6Se1_ICSD_410781	insulator	insulator
Ca10O25P6_ICSD_87727	insulator	insulator
Ca11Ga7_ICSD_58896	metal	metal
Ca11N8_ICSD_23208	metal	insulator
Ca11Sb10_ICSD_433	metal	insulator
Ca12Li6Mo4N16O3_ICSD_83865	insulator	insulator
Ca12Li6N16O3Re4_ICSD_411462	metal	insulator
Ca12Li6N16O3W4_ICSD_83866	insulator	insulator
Ca14Si19_ICSD_78963	insulator	metal
Ca17Hg9Li6_ICSD_420846	metal	metal
Ca19In8N7_ICSD_155929	metal	metal
Ca1Cd1Ge1_ICSD_52750	metal	metal
Ca1Cd1Pb1_ICSD_58877	metal	metal
Ca1Cd1Sn1_ICSD_58878	metal	metal
Ca1Cd1_ICSD_619188	metal	metal
Ca1Cd2Cu9_ICSD_424134	metal	metal
Ca1Cd2P2_ICSD_100063	insulator	insulator
Ca1Cd2Pd1_ICSD_425509	metal	metal
Ca1Cd2Sb2_ICSD_12151	insulator	insulator
Ca1Cd2_ICSD_420576	metal	metal
Ca1Cd2_ICSD_619190	metal	metal
Ca1Cl1F1_ICSD_1130	insulator	insulator
Ca1Cl1Fe1O2_ICSD_69869	insulator	insulator
Ca1Cl1Fe1O2_ICSD_96556	insulator	insulator
Ca1Cl1H1O1_ICSD_24403	insulator	insulator
Ca1Cl1H1_ICSD_25601	insulator	insulator
Ca1Cl2H12O6_ICSD_59142	insulator	insulator
Ca1Cl2H4O2_ICSD_960	insulator	insulator
Ca1Cl2H8O4_ICSD_16484	insulator	insulator
Ca1Cl2H8O4_ICSD_8061	insulator	insulator
Ca1Cl2O4_ICSD_171020	insulator	insulator
Ca1Cl2_ICSD_51238	insulator	insulator
Ca1Cl2_ICSD_56421	insulator	insulator
Ca1Cl2_ICSD_56769	insulator	insulator
Ca1Cl2_ICSD_86209	insulator	insulator
Ca1Cl6H24Mg2O12_ICSD_16326	insulator	insulator
Ca1Co1F6Li1_ICSD_32730	insulator	insulator
Ca1Co1O6Si2_ICSD_159546	insulator	insulator
Ca1Co1O7P2_ICSD_202852	insulator	insulator
Ca1Co2Ge2_ICSD_406	metal	metal
Ca1Co2O4_ICSD_245715	metal	metal
Ca1Co2P2_ICSD_10462	metal	metal

Supplementary Table 224. Five-fold cross validated predictions for the metal/insulator classification (216/598).

system	calculated	predicted
Ca1Co2Si2_ICSD_420974	metal	metal
Ca1Co3O14P4_ICSD_74047	insulator	insulator
Ca1Co4Cu3O12_ICSD_169095	metal	metal
Ca1Cr1F5_ICSD_10286	insulator	insulator
Ca1Cr1F5_ICSD_23174	insulator	insulator
Ca1Cr1F6Li1_ICSD_73985	insulator	insulator
Ca1Cr1F6_ICSD_10343	insulator	insulator
Ca1Cr1O10Si4_ICSD_83465	insulator	insulator
Ca1Cr1O4_ICSD_30283	insulator	insulator
Ca1Cr1O7P2_ICSD_411743	insulator	insulator
Ca1Cr2Cu3O12Sb2_ICSD_152283	metal	insulator
Ca1Cs1F3_ICSD_290448	insulator	insulator
Ca1Cs1H3_ICSD_168718	insulator	insulator
Ca1Cs1H3_ICSD_619213	insulator	insulator
Ca1Cs1N3O6_ICSD_56433	insulator	insulator
Ca1Cs2F4_ICSD_82616	insulator	insulator
Ca1Cs2H4_ICSD_261358	insulator	insulator
Ca1Cu1F4_ICSD_9928	insulator	insulator
Ca1Cu1Ge1_ICSD_100071	metal	metal
Ca1Cu1Ge1_ICSD_408580	metal	metal
Ca1Cu1Ge2O6_ICSD_170819	insulator	insulator
Ca1Cu1Ge2O6_ICSD_170823	insulator	insulator
Ca1Cu1H2O5Si1_ICSD_30926	insulator	insulator
Ca1Cu1In2_ICSD_106332	metal	metal
Ca1Cu1K2N6O12_ICSD_6183	metal	metal
Ca1Cu1O10Si4_ICSD_402012	insulator	insulator
Ca1Cu1O2_ICSD_84868	insulator	metal
Ca1Cu1O2_ICSD_93652	metal	metal
Ca1Cu1P1_ICSD_49740	insulator	insulator
Ca1Cu1Sb1_ICSD_659589	metal	metal
Ca1Cu1_ICSD_58880	metal	metal
Ca1Cu2Ge2_ICSD_619230	metal	metal
Ca1Cu2O3_ICSD_15094	metal	metal
Ca1Cu2O7Sr2Tl1_ICSD_74165	metal	metal
Ca1Cu2P2_ICSD_41756	metal	metal
Ca1Cu2Si2_ICSD_25314	metal	metal
Ca1Cu2Sn2_ICSD_417717	metal	metal
Ca1Cu3Ge4O12_ICSD_184279	insulator	insulator
Ca1Cu3Mn4O12_ICSD_156374	metal	metal
Ca1Cu3O12Pt4_ICSD_248230	insulator	insulator
Ca1Cu3O12Ru4_ICSD_51894	metal	metal
Ca1Cu3O12Sn4_ICSD_184281	insulator	insulator
Ca1Cu3O12Ti4_ICSD_161826	insulator	insulator
Ca1Cu3O12Ti4_ICSD_246526	insulator	insulator
Ca1Cu3O12Ti4_ICSD_30592	metal	insulator
Ca1Cu3O12V4_ICSD_250094	metal	insulator
Ca1Cu4In1_ICSD_370005	metal	metal
Ca1Cu4O24Re3Sr8_ICSD_97514	metal	insulator
Ca1Cu4P2_ICSD_62553	metal	metal
Ca1Cu5_ICSD_58882	metal	metal

Supplementary Table 225. Five-fold cross validated predictions for the metal/insulator classification (217/598).

system	calculated	predicted
Ca1Cu9Sn4_ICSD_417718	metal	metal
Ca1Er2S4_ICSD_619253	insulator	insulator
Ca1F1H4O5P1_ICSD_2802	insulator	insulator
Ca1F2_ICSD_51237	insulator	insulator
Ca1F2_ICSD_51239	insulator	insulator
Ca1F2_ICSD_51283	insulator	insulator
Ca1F2_ICSD_60368	insulator	insulator
Ca1F3K1_ICSD_153629	insulator	insulator
Ca1F3K1_ICSD_154073	insulator	insulator
Ca1F3Rb1_ICSD_201252	insulator	insulator
Ca1F4H2_ICSD_419144	insulator	insulator
Ca1F4Pd1_ICSD_32674	insulator	insulator
Ca1F4Zn1_ICSD_31366	insulator	insulator
Ca1F5Fe1_ICSD_412862	insulator	insulator
Ca1F5Mn1_ICSD_69632	insulator	insulator
Ca1F5Ti1_ICSD_49529	insulator	insulator
Ca1F6Ga1Li1_ICSD_152300	insulator	insulator
Ca1F6Li1Ni1_ICSD_32732	insulator	insulator
Ca1F6Pb1_ICSD_25522	insulator	insulator
Ca1F6Pd1_ICSD_26164	insulator	insulator
Ca1F6Pt1_ICSD_37443	insulator	insulator
Ca1F6Rh1_ICSD_42160	insulator	insulator
Ca1F6Si1_ICSD_183914	insulator	insulator
Ca1F6Sn1_ICSD_35713	insulator	insulator
Ca1F6Sn1_ICSD_35723	insulator	insulator
Ca1F6Sn2_ICSD_92907	insulator	insulator
Ca1F6Tb1_ICSD_59986	metal	insulator
Ca1F8Hf1Li2_ICSD_95248	insulator	insulator
Ca1Fe1Ge2O6_ICSD_290257	insulator	insulator
Ca1Fe1O2_ICSD_173438	metal	metal
Ca1Fe1O2_ICSD_246245	insulator	metal
Ca1Fe1O3_ICSD_92339	metal	insulator
Ca1Fe1O3_ICSD_92340	insulator	insulator
Ca1Fe1O3_ICSD_92342	insulator	insulator
Ca1Fe1O3_ICSD_92343	metal	insulator
Ca1Fe1O3_ICSD_92349	metal	insulator
Ca1Fe1O6Si2_ICSD_83438	insulator	insulator
Ca1Fe1O6Ti2_ICSD_79353	insulator	insulator
Ca1Fe2O4_ICSD_159751	metal	insulator
Ca1Fe2O4_ICSD_16695	insulator	metal
Ca1Fe2P2_ICSD_10463	metal	metal
Ca1Fe2P2_ICSD_54406	metal	metal
Ca1Fe2Si2_ICSD_425467	metal	metal
Ca1Fe3O12Ti4_ICSD_79277	insulator	metal
Ca1Fe3O5_ICSD_16354	insulator	insulator
Ca1Fe4O6_ICSD_16355	insulator	insulator
Ca1Fe4Sb12_ICSD_42961	metal	metal
Ca1Fe5O7_ICSD_16356	insulator	insulator
Ca1Ga1Ge1H1_ICSD_173567	insulator	insulator
Ca1Ga1Ge1_ICSD_66002	metal	metal

Supplementary Table 226. Five-fold cross validated predictions for the metal/insulator classification (218/598).

system	calculated	predicted
Ca1Ga1H1Si1_ICSD_173566	insulator	insulator
Ca1Ga1H1Sn1_ICSD_173568	insulator	insulator
Ca1Ga1H4_ICSD_240695	metal	insulator
Ca1Ga1La1O4_ICSD_96463	insulator	insulator
Ca1Ga1Li1N2_ICSD_415411	insulator	insulator
Ca1Ga1N1_ICSD_2027	metal	insulator
Ca1Ga1Pt1_ICSD_106340	metal	metal
Ca1Ga1_ICSD_419100	metal	metal
Ca1Ga2Ni1_ICSD_710075	metal	metal
Ca1Ga2P2_ICSD_422525	insulator	insulator
Ca1Ga2S4_ICSD_619292	insulator	insulator
Ca1Ga2_ICSD_260645	metal	metal
Ca1Ga2_ICSD_619281	metal	metal
Ca1Ga3Ni2_ICSD_58898	metal	metal
Ca1Ga4O7_ICSD_10351	insulator	insulator
Ca1Ga4_ICSD_54994	metal	metal
Ca1Ga4_ICSD_619282	metal	metal
Ca1Ge1Li2O4_ICSD_19024	insulator	insulator
Ca1Ge1Mg1_ICSD_42456	metal	metal
Ca1Ge1N2_ICSD_23523	insulator	insulator
Ca1Ge1Ni1_ICSD_418852	metal	metal
Ca1Ge1O3_ICSD_403086	insulator	insulator
Ca1Ge1O5Ti1_ICSD_171795	metal	insulator
Ca1Ge1O5Zr1_ICSD_154327	insulator	insulator
Ca1Ge1O8P2_ICSD_423043	insulator	insulator
Ca1Ge1Pt1_ICSD_106343	metal	metal
Ca1Ge1Sr1_ICSD_172006	insulator	metal
Ca1Ge1Zn1_ICSD_52772	metal	metal
Ca1Ge1_ICSD_185653	metal	metal
Ca1Ge2Ir2_ICSD_52755	metal	metal
Ca1Ge2Li1_ICSD_25328	metal	metal
Ca1Ge2Mn2_ICSD_80505	metal	metal
Ca1Ge2Mn2_ICSD_81743	metal	metal
Ca1Ge2Ni1_ICSD_240342	metal	metal
Ca1Ge2Ni2_ICSD_408	metal	metal
Ca1Ge2O5_ICSD_151581	insulator	insulator
Ca1Ge2O5_ICSD_156194	insulator	insulator
Ca1Ge2O5_ICSD_200614	insulator	insulator
Ca1Ge2O6Zn1_ICSD_59938	insulator	insulator
Ca1Ge2Pd2_ICSD_52759	metal	metal
Ca1Ge2Pt2_ICSD_619327	metal	metal
Ca1Ge2Rh2_ICSD_52760	metal	metal
Ca1Ge2Ru2_ICSD_52761	metal	metal
Ca1Ge2Zn2_ICSD_25319	metal	metal
Ca1Ge2_ICSD_185657	metal	metal
Ca1Ge2_ICSD_185658	metal	metal
Ca1Ge2_ICSD_245611	metal	metal
Ca1Ge2_ICSD_619304	metal	metal
Ca1Ge3_ICSD_262736	metal	metal
Ca1H10O8Zn2_ICSD_260592	insulator	insulator

Supplementary Table 227. Five-fold cross validated predictions for the metal/insulator classification (219/598).

system	calculated	predicted
Ca1H12N2O10S2_ICSD_410199	insulator	insulator
Ca1H1I1_ICSD_25545	insulator	insulator
Ca1H1K3O8P2_ICSD_35344	insulator	insulator
Ca1H1Mn1O5Si1_ICSD_83774	insulator	insulator
Ca1H1Na1O4Si1_ICSD_97911	insulator	insulator
Ca1H1Ni5_ICSD_189750	metal	metal
Ca1H1Ni5_ICSD_189752	metal	metal
Ca1H1Ni5_ICSD_189753	metal	metal
Ca1H1Ni5_ICSD_189754	metal	metal
Ca1H1O4P1_ICSD_87196	insulator	insulator
Ca1H2I2O7_ICSD_36635	insulator	insulator
Ca1H2K2O9S2_ICSD_157072	insulator	insulator
Ca1H2O12P4_ICSD_82359	insulator	insulator
Ca1H2O2_ICSD_15471	insulator	insulator
Ca1H2O5Si1Zn1_ICSD_34944	insulator	insulator
Ca1H2O7P2_ICSD_91818	insulator	insulator
Ca1H2_ICSD_157943	insulator	insulator
Ca1H2_ICSD_261635	insulator	insulator
Ca1H3K1_ICSD_168716	insulator	insulator
Ca1H3Li1_ICSD_168713	insulator	insulator
Ca1H3Na1_ICSD_168715	insulator	insulator
Ca1H3O4P1_ICSD_411737	insulator	insulator
Ca1H3Rb1_ICSD_65195	insulator	insulator
Ca1H4Mg1Ni1_ICSD_400003	insulator	insulator
Ca1H4O4P2_ICSD_44413	insulator	insulator
Ca1H4O6S1_ICSD_2059	insulator	insulator
Ca1H4O7Se2_ICSD_84441	insulator	insulator
Ca1H4Rb2_ICSD_65196	insulator	insulator
Ca1H5O6P1_ICSD_98804	insulator	insulator
Ca1H6K1N3_ICSD_1359	insulator	insulator
Ca1H6N3Rb1_ICSD_14014	insulator	insulator
Ca1H6O16S4_ICSD_85073	metal	insulator
Ca1H6O7P2_ICSD_36285	insulator	insulator
Ca1H6O9P2_ICSD_133	insulator	insulator
Ca1H8K2N12O4_ICSD_34953	insulator	insulator
Ca1H8O14P2V2_ICSD_67660	insulator	insulator
Ca1Hg11_ICSD_58902	metal	metal
Ca1Hg1O2_ICSD_80717	insulator	insulator
Ca1Hg1Pb1_ICSD_106345	metal	metal
Ca1Hg1Sn1_ICSD_602726	metal	metal
Ca1Hg1Sn1_ICSD_659596	metal	metal
Ca1Hg1_ICSD_58900	metal	metal
Ca1Hg2_ICSD_58901	metal	metal
Ca1Hg2_ICSD_619359	metal	metal
Ca1Hg3_ICSD_619356	metal	metal
Ca1Ho2S4_ICSD_619369	insulator	insulator
Ca1I2O6_ICSD_1391	insulator	insulator
Ca1I2_ICSD_52280	insulator	insulator
Ca1In1Pt1_ICSD_106348	metal	metal
Ca1In1Rh1_ICSD_410841	metal	metal

Supplementary Table 228. Five-fold cross validated predictions for the metal/insulator classification (220/598).

system	calculated	predicted
Ca1In1S4Yb1_ICSD_67655	metal	insulator
Ca1In1Se4Yb1_ICSD_67654	metal	insulator
Ca1In1_ICSD_414231	metal	metal
Ca1In1_ICSD_58905	metal	metal
Ca1In2Ni1_ICSD_619378	metal	metal
Ca1In2O4_ICSD_52390	insulator	insulator
Ca1In2P2_ICSD_260562	insulator	insulator
Ca1In2Pd1_ICSD_408881	metal	metal
Ca1In2Pt1_ICSD_408880	metal	metal
Ca1In2_ICSD_58686	metal	metal
Ca1In2_ICSD_58906	metal	metal
Ca1In2_ICSD_619376	metal	metal
Ca1In4Ir1_ICSD_410892	metal	metal
Ca1In4Ni1_ICSD_106347	metal	metal
Ca1In4Pd1_ICSD_410890	metal	metal
Ca1In4Rh1_ICSD_410891	metal	metal
Ca1Ir1O3_ICSD_159026	metal	metal
Ca1Ir1O3_ICSD_159037	metal	metal
Ca1Ir1O6Sr3_ICSD_81899	metal	metal
Ca1Ir1Si3_ICSD_180962	metal	metal
Ca1Ir1Sn2_ICSD_410773	metal	metal
Ca1Ir2P2_ICSD_95756	insulator	insulator
Ca1Ir2_ICSD_108146	metal	metal
Ca1Ir3Si7_ICSD_181449	metal	metal
Ca1K1O9P3_ICSD_281588	insulator	insulator
Ca1K2N4O8_ICSD_85505	insulator	insulator
Ca1K2N6Ni1O12_ICSD_26748	metal	metal
Ca1Li1N1_ICSD_107304	insulator	insulator
Ca1Li1Pb1_ICSD_409533	metal	metal
Ca1Li1Sb1_ICSD_52775	insulator	insulator
Ca1Li1Si2_ICSD_25327	metal	metal
Ca1Li1Sn1_ICSD_58911	metal	metal
Ca1Li2N4Si2_ICSD_421548	insulator	insulator
Ca1Li2O4Si1_ICSD_19023	insulator	insulator
Ca1Li2O7Ta2_ICSD_245841	insulator	insulator
Ca1Li2_ICSD_413207	metal	metal
Ca1Li2_ICSD_619390	metal	metal
Ca1Li4O8Si2Sr1_ICSD_79867	insulator	insulator
Ca1Lu2S4_ICSD_200013	insulator	insulator
Ca1Mg1Ni4_ICSD_107419	metal	metal
Ca1Mg1O6Si2_ICSD_159522	insulator	insulator
Ca1Mg1Pb1_ICSD_619403	metal	metal
Ca1Mg1Si1_ICSD_158276	metal	metal
Ca1Mg1Sn1_ICSD_42757	metal	metal
Ca1Mg2N2_ICSD_411175	insulator	insulator
Ca1Mg2O12S3_ICSD_418454	insulator	insulator
Ca1Mg2Sb2_ICSD_100045	insulator	insulator
Ca1Mg2_ICSD_370044	metal	metal
Ca1Mn1O3_ICSD_168903	metal	metal
Ca1Mn1O3_ICSD_168906	insulator	insulator

Supplementary Table 229. Five-fold cross validated predictions for the metal/insulator classification (221/598).

system	calculated	predicted
Ca1Mn1O3_ICSD_86645	insulator	insulator
Ca1Mn1O6Si2_ICSD_168042	insulator	insulator
Ca1Mn1O7P2_ICSD_78659	insulator	insulator
Ca1Mn1Sb2_ICSD_52705	metal	metal
Ca1Mn1Si1_ICSD_66949	metal	metal
Ca1Mn1Sn1_ICSD_66950	metal	metal
Ca1Mn28_ICSD_619408	metal	metal
Ca1Mn2O4_ICSD_93749	insulator	metal
Ca1Mn2P2_ICSD_49017	metal	metal
Ca1Mn2Sb2_ICSD_164898	metal	metal
Ca1Mn2Sb2_ICSD_164899	metal	metal
Ca1Mn7O12_ICSD_187416	metal	metal
Ca1Mo1O4_ICSD_60553	insulator	insulator
Ca1Mo1O6Sr2_ICSD_72815	insulator	insulator
Ca1Mo1O6Sr2_ICSD_72816	insulator	insulator
Ca1Mo4O16U1_ICSD_202234	insulator	insulator
Ca1Mo6S8_ICSD_619421	metal	metal
Ca1N1Ni1_ICSD_656846	metal	metal
Ca1N1O2Ta1_ICSD_161824	insulator	insulator
Ca1N2O6_ICSD_52351	insulator	insulator
Ca1N2Si1_ICSD_170267	insulator	insulator
Ca1N2_ICSD_423721	metal	insulator
Ca1N6_ICSD_35227	insulator	insulator
Ca1Na10Sn12_ICSD_240006	insulator	insulator
Ca1Na1O4V1_ICSD_32573	insulator	insulator
Ca1Na2O12V4_ICSD_161368	insulator	insulator
Ca1Na2O4Si1_ICSD_24235	insulator	insulator
Ca1Na2O7P2_ICSD_89468	insulator	insulator
Ca1Na2O8S2_ICSD_28429	insulator	insulator
Ca1Na4O18P6_ICSD_281508	insulator	insulator
Ca1Na4Sn6_ICSD_172208	metal	metal
Ca1Nb1O3_ICSD_51202	metal	insulator
Ca1Nb2O11P2_ICSD_72115	insulator	insulator
Ca1Nb2O4_ICSD_88779	insulator	insulator
Ca1Nb2O6_ICSD_15208	insulator	insulator
Ca1Ni1O6Si2_ICSD_159545	insulator	insulator
Ca1Ni1O6Si2_ICSD_246208	insulator	insulator
Ca1Ni2P2_ICSD_10461	metal	metal
Ca1Ni2Si1_ICSD_412411	metal	metal
Ca1Ni2Si2_ICSD_90579	metal	metal
Ca1Ni2Zn3_ICSD_245512	metal	metal
Ca1Ni2_ICSD_619440	metal	metal
Ca1Ni3O14P4_ICSD_74046	insulator	insulator
Ca1Ni3_ICSD_619444	metal	metal
Ca1Ni4O8_ICSD_40470	insulator	insulator
Ca1Ni4Sn2_ICSD_418133	metal	metal
Ca1Ni5_ICSD_619446	metal	metal
Ca1Ni5_ICSD_619452	metal	metal
Ca1O11P4_ICSD_74953	insulator	insulator
Ca1O11Ta4_ICSD_1854	insulator	insulator

Supplementary Table 230. Five-fold cross validated predictions for the metal/insulator classification (222/598).

system	calculated	predicted
Ca1O1S1Zn1_ICSD_245309	insulator	insulator
Ca1O1_ICSD_161831	insulator	insulator
Ca1O1_ICSD_90486	insulator	insulator
Ca1O24P6Sn4_ICSD_261116	insulator	insulator
Ca1O24P6Zr4_ICSD_151683	insulator	insulator
Ca1O2_ICSD_619462	insulator	insulator
Ca1O3Pt1_ICSD_173278	insulator	insulator
Ca1O3Rh1_ICSD_164774	metal	insulator
Ca1O3Rh1_ICSD_164775	insulator	metal
Ca1O3Ru1_ICSD_99451	metal	insulator
Ca1O3Si1_ICSD_20571	insulator	insulator
Ca1O3Si1_ICSD_240432	insulator	insulator
Ca1O3Si1_ICSD_240442	insulator	insulator
Ca1O3Si1_ICSD_240450	insulator	insulator
Ca1O3Si1_ICSD_240456	insulator	insulator
Ca1O3Si1_ICSD_240461	insulator	insulator
Ca1O3Si1_ICSD_240465	insulator	insulator
Ca1O3Si1_ICSD_240468	insulator	insulator
Ca1O3Si1_ICSD_40658	insulator	insulator
Ca1O3Si1_ICSD_87694	insulator	insulator
Ca1O3Sn1_ICSD_2373	insulator	insulator
Ca1O3Sn1_ICSD_27777	metal	insulator
Ca1O3Sn1_ICSD_56095	insulator	insulator
Ca1O3Tc1_ICSD_261341	insulator	metal
Ca1O3Te1_ICSD_260238	insulator	insulator
Ca1O3Ti1_ICSD_162919	insulator	insulator
Ca1O3Ti1_ICSD_165801	insulator	insulator
Ca1O3Ti1_ICSD_187294	insulator	insulator
Ca1O3Ti1_ICSD_29116	insulator	insulator
Ca1O3Ti1_ICSD_31864	insulator	insulator
Ca1O3Ti1_ICSD_50364	insulator	insulator
Ca1O3V1_ICSD_88978	insulator	insulator
Ca1O3Zr1_ICSD_56094	insulator	insulator
Ca1O3Zr1_ICSD_97466	insulator	insulator
Ca1O4Pd3_ICSD_186819	metal	metal
Ca1O4Pt2_ICSD_6159	metal	insulator
Ca1O4Rh2_ICSD_170597	insulator	metal
Ca1O4S1_ICSD_171053	insulator	insulator
Ca1O4S1_ICSD_79527	insulator	insulator
Ca1O4S1_ICSD_86316	insulator	insulator
Ca1O4Te1_ICSD_8097	insulator	insulator
Ca1O4Tl2_ICSD_413874	metal	insulator
Ca1O4U1_ICSD_31631	insulator	insulator
Ca1O4W1_ICSD_60548	insulator	insulator
Ca1O4Yb2_ICSD_27312	metal	insulator
Ca1O5Se2_ICSD_27209	insulator	insulator
Ca1O5Si1Sn1_ICSD_96819	insulator	insulator
Ca1O5Si1Sn1_ICSD_96820	insulator	insulator
Ca1O5Si1Ti1_ICSD_159343	insulator	insulator
Ca1O5Si2_ICSD_87498	insulator	insulator

Supplementary Table 231. Five-fold cross validated predictions for the metal/insulator classification (223/598).

system	calculated	predicted
Ca1O5Te2_ICSD_260041	insulator	insulator
Ca1O5V2_ICSD_82689	insulator	insulator
Ca1O6P2_ICSD_417710	insulator	insulator
Ca1O6P2_ICSD_60117	insulator	insulator
Ca1O6Sb2_ICSD_74539	insulator	insulator
Ca1O6Si2Zn1_ICSD_158143	insulator	insulator
Ca1O6Sr2W1_ICSD_36459	insulator	insulator
Ca1O6Sr2W1_ICSD_36460	insulator	insulator
Ca1O6Ta2_ICSD_24091	insulator	insulator
Ca1O6Ta2_ICSD_47121	insulator	insulator
Ca1O6V2_ICSD_21064	insulator	insulator
Ca1O7Si2Zr1_ICSD_203131	insulator	insulator
Ca1O7V3_ICSD_241204	insulator	insulator
Ca1O8P2U1_ICSD_81388	insulator	insulator
Ca1O8P2Zn2_ICSD_202571	insulator	insulator
Ca1O8Te3_ICSD_100661	insulator	insulator
Ca1O9P2V2_ICSD_79735	insulator	insulator
Ca1O9V4_ICSD_2556	insulator	insulator
Ca1P1Pt1_ICSD_60826	insulator	metal
Ca1P2Pd2_ICSD_36371	metal	metal
Ca1P2Rh2_ICSD_50185	metal	metal
Ca1P2Ru2_ICSD_602125	metal	metal
Ca1P2Zn2_ICSD_619477	insulator	insulator
Ca1P3_ICSD_74479	metal	insulator
Ca1Pb1Pd1_ICSD_106355	metal	metal
Ca1Pb1Pd1_ICSD_602349	metal	metal
Ca1Pb1Pt1_ICSD_602366	metal	metal
Ca1Pb1Sr1_ICSD_172008	insulator	metal
Ca1Pb1Zn1_ICSD_659603	metal	metal
Ca1Pb1_ICSD_58918	metal	metal
Ca1Pb3_ICSD_619485	metal	metal
Ca1Pd1Si1_ICSD_69790	metal	metal
Ca1Pd1Sn1_ICSD_58690	metal	metal
Ca1Pd1Sn1_ICSD_602357	metal	metal
Ca1Pd1Sn2_ICSD_410774	metal	metal
Ca1Pd1_ICSD_58926	metal	metal
Ca1Pd2Si2_ICSD_619506	metal	metal
Ca1Pd2_ICSD_58927	metal	metal
Ca1Pd5_ICSD_106357	metal	metal
Ca1Pt1Sb1_ICSD_60830	metal	metal
Ca1Pt1Si1_ICSD_63555	metal	metal
Ca1Pt1Si1_ICSD_72641	metal	metal
Ca1Pt1Si1_ICSD_72642	metal	metal
Ca1Pt1Si3_ICSD_181448	metal	metal
Ca1Pt1Sn1_ICSD_602355	metal	metal
Ca1Pt2_ICSD_109140	metal	metal
Ca1Pt2_ICSD_619516	metal	metal
Ca1Pt5_ICSD_58929	metal	metal
Ca1Rb1Sb1_ICSD_409178	insulator	insulator
Ca1Rh1Sn2_ICSD_410775	metal	metal

Supplementary Table 232. Five-fold cross validated predictions for the metal/insulator classification (224/598).

system	calculated	predicted
Ca1Rh2Si2_ICSD_425469	metal	metal
Ca1Rh2_ICSD_108145	metal	metal
Ca1S1_ICSD_186785	insulator	insulator
Ca1S3Zr1_ICSD_23286	insulator	insulator
Ca1S4Y2_ICSD_619557	insulator	insulator
Ca1S4Yb2_ICSD_619559	insulator	metal
Ca1Sb2Zn2_ICSD_12150	metal	metal
Ca1Sb2_ICSD_862	metal	metal
Ca1Se1_ICSD_167834	insulator	insulator
Ca1Se1_ICSD_53948	insulator	insulator
Ca1Si1Sr1_ICSD_172005	insulator	insulator
Ca1Si1Zn1_ICSD_52791	metal	metal
Ca1Si1_ICSD_25570	metal	metal
Ca1Si1_ICSD_619586	metal	metal
Ca1Si2Zn2_ICSD_59649	metal	metal
Ca1Si2_ICSD_154431	metal	metal
Ca1Si2_ICSD_154433	metal	metal
Ca1Si2_ICSD_32007	metal	metal
Ca1Si2_ICSD_409057	metal	metal
Ca1Si2_ICSD_41450	metal	metal
Ca1Si3_ICSD_263004	metal	metal
Ca1Sn1Sr1_ICSD_172007	insulator	insulator
Ca1Sn1Zn1_ICSD_106358	metal	metal
Ca1Sn1Zn1_ICSD_659612	metal	metal
Ca1Sn1_ICSD_55213	metal	metal
Ca1Sn3_ICSD_58934	metal	metal
Ca1Te1_ICSD_52792	metal	metal
Ca1Te1_ICSD_619616	insulator	insulator
Ca1Tl1_ICSD_619623	metal	metal
Ca1Tl3_ICSD_58942	metal	metal
Ca1Zn11_ICSD_619630	metal	metal
Ca1Zn13_ICSD_619636	metal	metal
Ca1Zn1_ICSD_58944	metal	metal
Ca1Zn2_ICSD_418878	metal	metal
Ca1Zn2_ICSD_58945	metal	metal
Ca1Zn5_ICSD_619638	metal	metal
Ca1_ICSD_107510	metal	metal
Ca1_ICSD_107511	metal	metal
Ca1_ICSD_162253	metal	metal
Ca1_ICSD_162255	metal	metal
Ca1_ICSD_162256	metal	metal
Ca1_ICSD_168758	metal	metal
Ca1_ICSD_168759	metal	metal
Ca1_ICSD_188391	metal	metal
Ca1_ICSD_52270	metal	metal
Ca1_ICSD_52748	metal	metal
Ca1_ICSD_53768	metal	metal
Ca22O36S2Si8_ICSD_26407	insulator	insulator
Ca2Cd1P2_ICSD_422580	insulator	insulator
Ca2Cd1Pd2_ICSD_425471	metal	metal

Supplementary Table 233. Five-fold cross validated predictions for the metal/insulator classification (225/598).

system	calculated	predicted
Ca2Cd1Pt2_ICSD_424554	metal	metal
Ca2Cd1Sb2_ICSD_173172	insulator	metal
Ca2Cd2K1Sb3_ICSD_420619	metal	insulator
Ca2Cl1Cr1O4_ICSD_15315	insulator	insulator
Ca2Cl1Cu1Nb3O10_ICSD_51262	metal	insulator
Ca2Cl1Fe1O3_ICSD_56880	insulator	insulator
Ca2Cl1N1_ICSD_153101	insulator	insulator
Ca2Cl1O4P1_ICSD_15316	insulator	insulator
Ca2Cl2Cu1O10Te4_ICSD_171007	insulator	insulator
Ca2Cl2Cu1O2_ICSD_1027	metal	metal
Ca2Cl2O3Si1_ICSD_200221	insulator	insulator
Ca2Co1Fe1O5_ICSD_169345	metal	insulator
Ca2Co1O3_ICSD_95439	insulator	insulator
Ca2Co1O7Si2_ICSD_94137	insulator	insulator
Ca2Cr4O16P4_ICSD_412383	insulator	insulator
Ca2Cs1O10Ta3_ICSD_89011	insulator	insulator
Ca2Cu1Fe1O3S1_ICSD_169992	insulator	insulator
Ca2Cu1Fe1O3Se1_ICSD_169993	insulator	insulator
Ca2Cu1O3_ICSD_202995	metal	metal
Ca2Cu1O3_ICSD_93651	metal	metal
Ca2Cu1P3Zn2_ICSD_89517	insulator	insulator
Ca2Cu1_ICSD_108865	metal	metal
Ca2Cu2Ga1_ICSD_58885	metal	metal
Ca2F1O6Ta2_ICSD_50048	metal	insulator
Ca2F2Mg5O22Si8_ICSD_22020	insulator	insulator
Ca2F4_ICSD_246961	insulator	insulator
Ca2Fe1H8O12P2_ICSD_200477	insulator	insulator
Ca2Fe1Mn1O5_ICSD_85125	metal	insulator
Ca2Fe1N2_ICSD_72389	insulator	insulator
Ca2Fe1O6W1_ICSD_81203	insulator	insulator
Ca2Fe1O6W1_ICSD_81204	insulator	insulator
Ca2Fe2O5_ICSD_161513	insulator	insulator
Ca2Fe7O11_ICSD_100827	insulator	insulator
Ca2Fe9O13_ICSD_100826	insulator	insulator
Ca2Ga1Mn1O5_ICSD_51464	metal	insulator
Ca2Ga1Mn1O5_ICSD_51466	metal	insulator
Ca2Ge1In2Sr1_ICSD_85466	metal	metal
Ca2Ge1N2_ICSD_280252	metal	insulator
Ca2Ge1O4_ICSD_16258	insulator	insulator
Ca2Ge1O4_ICSD_173468	insulator	insulator
Ca2Ge1O4_ICSD_182051	insulator	insulator
Ca2Ge1O4_ICSD_39157	metal	metal
Ca2Ge1S4_ICSD_619332	insulator	insulator
Ca2Ge1_ICSD_42455	insulator	metal
Ca2Ge2H2Ni2_ICSD_263028	metal	metal
Ca2Ge2In1Li1_ICSD_280690	metal	metal
Ca2Ge2Ni3_ICSD_421074	metal	metal
Ca2Ge2O7Zn1_ICSD_69387	insulator	insulator
Ca2Ge3Li1_ICSD_25323	metal	metal
Ca2Ge7O16_ICSD_200603	insulator	insulator

Supplementary Table 234. Five-fold cross validated predictions for the metal/insulator classification (226/598).

system	calculated	predicted
Ca2Ge7O16_ICSD_249322	insulator	insulator
Ca2H18O12Re6S8_ICSD_415689	insulator	insulator
Ca2H1N1_ICSD_292	insulator	insulator
Ca2H1Na1O9Si3_ICSD_26820	insulator	insulator
Ca2H2Mg5O24Si8_ICSD_9659	insulator	insulator
Ca2H4Mg1O10P2_ICSD_4258	insulator	insulator
Ca2H4Mn1O10P2_ICSD_156225	insulator	insulator
Ca2H4O13P3V1_ICSD_165406	insulator	insulator
Ca2H4O9P2_ICSD_1912	insulator	insulator
Ca2H6Ru1_ICSD_656082	insulator	insulator
Ca2Hf7O16_ICSD_4136	insulator	insulator
Ca2Hg1_ICSD_619362	metal	metal
Ca2I1N1_ICSD_65216	insulator	insulator
Ca2I1P1_ICSD_6068	insulator	insulator
Ca2In1Pd2_ICSD_418202	metal	metal
Ca2In1Pt2_ICSD_418203	metal	metal
Ca2In1_ICSD_413803	metal	metal
Ca2Ir1O4_ICSD_25500	insulator	metal
Ca2Ir2Si1_ICSD_95789	metal	metal
Ca2K2O12S3_ICSD_40988	insulator	insulator
Ca2K8O24U6_ICSD_91783	insulator	insulator
Ca2Li1Si3_ICSD_25322	metal	metal
Ca2Li6Mn2N6_ICSD_408324	insulator	insulator
Ca2Mg1O6W1_ICSD_281564	insulator	insulator
Ca2Mg1O7Si2_ICSD_158177	insulator	insulator
Ca2Mn1O6Sb1_ICSD_246090	insulator	insulator
Ca2Mn1O6Ta1_ICSD_246091	insulator	insulator
Ca2Mn1O6W1_ICSD_51615	metal	insulator
Ca2Mn2O5_ICSD_35130	metal	insulator
Ca2Mn3O8_ICSD_24847	insulator	insulator
Ca2N1_ICSD_90630	metal	metal
Ca2N2Zn1_ICSD_69049	insulator	insulator
Ca2N3P1_ICSD_72532	insulator	insulator
Ca2N3V1_ICSD_409644	insulator	insulator
Ca2N4Sr1W1_ICSD_401331	insulator	insulator
Ca2N8Si5_ICSD_79070	insulator	insulator
Ca2Na3O6Ta1_ICSD_280284	insulator	insulator
Ca2Nb2O7_ICSD_22411	insulator	insulator
Ca2Nb3O10Rb1_ICSD_260289	insulator	insulator
Ca2Ni7P4_ICSD_94448	metal	metal
Ca2O10Rb1Ta3_ICSD_89010	insulator	insulator
Ca2O12Rb2S3_ICSD_249339	insulator	insulator
Ca2O12Si4Zr1_ICSD_73801	insulator	insulator
Ca2O20Si8Th1_ICSD_64745	insulator	insulator
Ca2O4Pb1_ICSD_36629	insulator	insulator
Ca2O4Ru1_ICSD_94244	insulator	insulator
Ca2O4Si1_ICSD_182052	insulator	insulator
Ca2O4Si1_ICSD_182054	insulator	insulator
Ca2O4Si1_ICSD_245074	insulator	insulator
Ca2O4Si1_ICSD_280995	insulator	insulator

Supplementary Table 235. Five-fold cross validated predictions for the metal/insulator classification (227/598).

system	calculated	predicted
Ca2O4Si1_ICSD_40657	insulator	insulator
Ca2O4Sn1_ICSD_9011	insulator	insulator
Ca2O5U1_ICSD_23198	insulator	insulator
Ca2O6Pd1W1_ICSD_83258	insulator	insulator
Ca2O6Pd1W1_ICSD_83259	insulator	insulator
Ca2O6Si1Ti1_ICSD_83455	insulator	insulator
Ca2O7Os2_ICSD_97092	metal	metal
Ca2O7Ru2_ICSD_156409	metal	metal
Ca2O7Sb2_ICSD_166282	insulator	insulator
Ca2O7Sb2_ICSD_24245	metal	insulator
Ca2O7Si2Zn1_ICSD_30262	insulator	insulator
Ca2O7Ta2_ICSD_27121	insulator	insulator
Ca2O7V2_ICSD_421266	insulator	insulator
Ca2O8Pt3_ICSD_65412	insulator	insulator
Ca2O8Se3_ICSD_54155	insulator	insulator
Ca2O9Pb1Si3_ICSD_18098	insulator	insulator
Ca2P2S6_ICSD_405192	insulator	insulator
Ca2P2Se6_ICSD_412765	metal	insulator
Ca2P2_ICSD_83352	insulator	insulator
Ca2Pb1_ICSD_58920	metal	metal
Ca2Pt3Sn5_ICSD_410772	metal	metal
Ca2S4Si1_ICSD_619542	insulator	insulator
Ca2S4Sn1_ICSD_619548	insulator	insulator
Ca2S5Sb2_ICSD_201044	insulator	insulator
Ca2Sb1_ICSD_42135	insulator	insulator
Ca2Se4Si1_ICSD_619574	insulator	insulator
Ca2Si1_ICSD_187352	insulator	metal
Ca2Si1_ICSD_42453	insulator	metal
Ca2Sn1_ICSD_659611	metal	metal
Ca3Cd2_ICSD_30082	metal	metal
Ca3Cl2Ge1O4_ICSD_249329	insulator	insulator
Ca3Cl2O4Si1_ICSD_9088	insulator	insulator
Ca3Cl3P1_ICSD_202075	insulator	insulator
Ca3Co1Mn1O6_ICSD_93775	metal	insulator
Ca3Co1O6Rh1_ICSD_50809	insulator	insulator
Ca3Co1O6Sc1_ICSD_245503	insulator	insulator
Ca3Co2O6_ICSD_246282	metal	insulator
Ca3Co4Sn13_ICSD_619211	metal	metal
Ca3Cr1N3_ICSD_40205	insulator	insulator
Ca3Cr2O12Si3_ICSD_82743	insulator	insulator
Ca3Cr3O16P4_ICSD_412381	insulator	insulator
Ca3Cu1Ir1O6_ICSD_73188	metal	insulator
Ca3Cu1O6Rh1_ICSD_96482	metal	insulator
Ca3Cu2P4Zn2_ICSD_89515	insulator	insulator
Ca3Cu8Sn4_ICSD_417716	metal	metal
Ca3Fe1O6Rh1_ICSD_96484	insulator	insulator
Ca3Fe2Ge3O12_ICSD_28176	insulator	insulator
Ca3Fe2O12Si3_ICSD_202964	insulator	insulator
Ca3Ga2N4_ICSD_170442	insulator	insulator
Ca3Ga2O12Si3_ICSD_27387	insulator	insulator

Supplementary Table 236. Five-fold cross validated predictions for the metal/insulator classification (228/598).

system	calculated	predicted
Ca3Ga2Pd2_ICSD_107507	metal	metal
Ca3Ga2Pt2_ICSD_107506	metal	metal
Ca3Ga3O14Si2Ta1_ICSD_245381	insulator	insulator
Ca3Ga4Ni4_ICSD_58899	metal	metal
Ca3Ga4O9_ICSD_100356	insulator	insulator
Ca3Ga5_ICSD_58894	metal	metal
Ca3Ge13Ir4_ICSD_619308	metal	metal
Ca3Ge13Rh4_ICSD_619329	metal	metal
Ca3Ge1N1_ICSD_106342	metal	metal
Ca3Ge1O1_ICSD_181077	metal	metal
Ca3Ge2P4_ICSD_41181	insulator	insulator
Ca3Ge3O12Sc2_ICSD_20215	insulator	insulator
Ca3Ge3O12Y2_ICSD_280048	insulator	insulator
Ca3Ge4_ICSD_185654	insulator	metal
Ca3H2Pb1_ICSD_165616	metal	metal
Ca3H2Sn1_ICSD_165615	metal	metal
Ca3Hf1O9Si2_ICSD_79452	insulator	insulator
Ca3Hg1_ICSD_58903	metal	metal
Ca3Hg1_ICSD_619363	metal	metal
Ca3Hg2_ICSD_58904	metal	metal
Ca3I3P1_ICSD_9026	insulator	insulator
Ca3In1P3_ICSD_60125	insulator	insulator
Ca3In1_ICSD_58907	metal	metal
Ca3In2O12Si3_ICSD_27390	insulator	insulator
Ca3Ir4Si4_ICSD_95788	metal	metal
Ca3Ir4Sn13_ICSD_619385	metal	metal
Ca3Li1O6Os1_ICSD_248206	insulator	insulator
Ca3Li1O6Ru1_ICSD_96219	insulator	insulator
Ca3Li4N6Si2_ICSD_420675	insulator	insulator
Ca3Mn1N3_ICSD_67888	insulator	insulator
Ca3Mn1Ni1O6_ICSD_50805	insulator	insulator
Ca3Mn1O6Zn1_ICSD_50808	insulator	insulator
Ca3Mn2O12Si3_ICSD_27388	metal	insulator
Ca3Mn2O7_ICSD_55666	insulator	insulator
Ca3Mn2O7_ICSD_55667	metal	insulator
Ca3Mn2O7_ICSD_96697	insulator	insulator
Ca3N1P1_ICSD_106350	insulator	insulator
Ca3N1Pb1_ICSD_106351	metal	metal
Ca3N1Sb1_ICSD_106352	metal	metal
Ca3N1Sn1_ICSD_106353	metal	metal
Ca3N1Tl1_ICSD_411448	metal	metal
Ca3N2_ICSD_162797	insulator	insulator
Ca3N2_ICSD_169726	insulator	insulator
Ca3N2_ICSD_169727	insulator	insulator
Ca3N2_ICSD_419865	insulator	insulator
Ca3N2_ICSD_50991	insulator	insulator
Ca3N3V1_ICSD_72118	insulator	insulator
Ca3Na1O6Ru1_ICSD_50020	insulator	insulator
Ca3Na2O9Ta2_ICSD_280154	insulator	insulator
Ca3Ni3Si2_ICSD_412410	metal	metal

Supplementary Table 237. Five-fold cross validated predictions for the metal/insulator classification (229/598).

system	calculated	predicted
Ca3O12Sc2Si3_ICSD_156537	insulator	insulator
Ca3O12Si3V2_ICSD_182067	metal	insulator
Ca3O12Te2Zn3_ICSD_64791	insulator	insulator
Ca3O12Te2Zn3_ICSD_67045	insulator	insulator
Ca3O1Pb1_ICSD_100789	metal	metal
Ca3O1Sn1_ICSD_181084	metal	metal
Ca3O5Si1_ICSD_22501	insulator	insulator
Ca3O5Si1_ICSD_81100	insulator	insulator
Ca3O6Te1_ICSD_35085	insulator	insulator
Ca3O6Tl2_ICSD_413876	insulator	insulator
Ca3O6U1_ICSD_23200	insulator	insulator
Ca3O8P2_ICSD_158736	insulator	insulator
Ca3O8V2_ICSD_412273	insulator	insulator
Ca3O9Si2Zr1_ICSD_79453	insulator	insulator
Ca3O9Si3_ICSD_87716	insulator	insulator
Ca3Pb1_ICSD_58921	metal	metal
Ca3Pd1_ICSD_619504	metal	metal
Ca3Pd2_ICSD_619502	metal	metal
Ca3Rh4Sn13_ICSD_58931	metal	metal
Ca3Tl1_ICSD_58943	metal	metal
Ca3Tl5_ICSD_380324	metal	metal
Ca3Zn1_ICSD_58948	metal	metal
Ca4Cl6O1_ICSD_33883	insulator	insulator
Ca4Cu3P5Zn2_ICSD_89518	insulator	insulator
Ca4F1Na5O16P4_ICSD_20906	insulator	insulator
Ca4F2O7Si2_ICSD_64710	insulator	insulator
Ca4Ge1N4_ICSD_280251	insulator	insulator
Ca4Ge3Ni4_ICSD_421432	metal	metal
Ca4Hg9_ICSD_107690	metal	metal
Ca4Ir1O6_ICSD_81902	metal	insulator
Ca4Ir8P7_ICSD_413847	metal	metal
Ca4Mn3O10_ICSD_85669	insulator	metal
Ca4N4Ti1_ICSD_172879	insulator	insulator
Ca4O1P2_ICSD_68202	insulator	insulator
Ca4O1Sb2_ICSD_16353	insulator	insulator
Ca4O6Pd1_ICSD_88134	insulator	insulator
Ca4O6Pt1_ICSD_59252	insulator	insulator
Ca4Pd4Si3_ICSD_74358	metal	metal
Ca5Cl1O12P3_ICSD_24237	insulator	insulator
Ca5Co2N4_ICSD_409920	insulator	insulator
Ca5F1O12P3_ICSD_52385	insulator	insulator
Ca5F1O12V3_ICSD_172997	insulator	insulator
Ca5Fe1Pb3_ICSD_240158	metal	metal
Ca5Ga2Sb6_ICSD_36466	metal	insulator
Ca5Ga6O14_ICSD_63052	insulator	insulator
Ca5Ge3O11_ICSD_403085	insulator	insulator
Ca5Ge3_ICSD_181074	metal	metal
Ca5Ge3_ICSD_181076	metal	metal
Ca5H1O13P3_ICSD_180315	insulator	insulator
Ca5H1Sn3_ICSD_165190	metal	metal

Supplementary Table 238. Five-fold cross validated predictions for the metal/insulator classification (230/598).

system	calculated	predicted
Ca5Hg3_ICSD_106344	metal	metal
Ca5In2Sb6_ICSD_36467	insulator	metal
Ca5Ir1_ICSD_156887	metal	metal
Ca5N4O2W1_ICSD_409475	insulator	insulator
Ca5N6Si2_ICSD_414462	insulator	insulator
Ca5O14Te3_ICSD_245873	insulator	insulator
Ca5P6Pd6_ICSD_79096	metal	metal
Ca5P8_ICSD_74854	insulator	insulator
Ca5Pd2_ICSD_619503	metal	metal
Ca5Pt2_ICSD_619513	metal	metal
Ca5Pt3_ICSD_619514	metal	metal
Ca5Sb3_ICSD_2065	insulator	metal
Ca5Sb3_ICSD_619563	metal	metal
Ca5Si3_ICSD_93699	metal	metal
Ca5Zn3_ICSD_184360	metal	metal
Ca6Cr2H1N6_ICSD_281462	metal	insulator
Ca6Cu2Sn7_ICSD_171243	metal	metal
Ca6Fe1N5_ICSD_33796	metal	insulator
Ca6Ga1N5_ICSD_33795	metal	insulator
Ca6Ge1Li1_ICSD_108776	metal	metal
Ca6H6O13Si2_ICSD_39725	insulator	insulator
Ca6Mn1N5_ICSD_80184	insulator	metal
Ca7.8856_ICSD_163532	metal	metal
Ca7Cl2H12_ICSD_420927	insulator	insulator
Ca7Ge1_ICSD_43321	metal	metal
Ca7Ge6_ICSD_95171	metal	metal
Ca7N9Nb1Si2_ICSD_414461	insulator	insulator
Ca7Ni4Sn13_ICSD_106354	metal	metal
Ca7Sn6_ICSD_54618	metal	metal
Ca8In3_ICSD_58909	metal	metal
Ca9Sb9Zn4_ICSD_52787	metal	insulator
Cd11Ce1_ICSD_619646	metal	metal
Cd11Eu1_ICSD_619836	metal	metal
Cd11La1_ICSD_620068	metal	metal
Cd11Na2_ICSD_421373	metal	metal
Cd11Nd1_ICSD_620191	metal	metal
Cd11Pr1_ICSD_620277	metal	metal
Cd11Sr1_ICSD_620498	metal	metal
Cd11Th1_ICSD_620560	metal	metal
Cd13Cs1_ICSD_619730	metal	metal
Cd13K1_ICSD_620061	metal	metal
Cd13Rb1_ICSD_415888	metal	metal
Cd17La2_ICSD_102006	metal	metal
Cd1Ce1Cu1_ICSD_656594	metal	metal
Cd1Ce1Ni4_ICSD_415194	metal	metal
Cd1Ce1Pd1_ICSD_656600	metal	metal
Cd1Ce1Tl1_ICSD_619670	metal	metal
Cd1Ce1_ICSD_58949	metal	metal
Cd1Ce1_ICSD_58950	metal	metal
Cd1Ce2Pd2_ICSD_411010	metal	metal

Supplementary Table 239. Five-fold cross validated predictions for the metal/insulator classification (231/598).

system	calculated	predicted
Cd1Ce2Pt2_ICSD_411009	metal	metal
Cd1Cl1H1O1_ICSD_26752	insulator	insulator
Cd1Cl1H1O1_ICSD_91087	insulator	insulator
Cd1Cl1K4O15V5_ICSD_404097	insulator	insulator
Cd1Cl1O15Rb4V5_ICSD_406783	insulator	insulator
Cd1Cl1O15Tl4V5_ICSD_406784	insulator	insulator
Cd1Cl1O3V1_ICSD_50788	insulator	insulator
Cd1Cl1S2Sb1_ICSD_171722	insulator	insulator
Cd1Cl2O2Pb2_ICSD_168248	insulator	insulator
Cd1Cl2_ICSD_62202	insulator	insulator
Cd1Cl3Cs1_ICSD_24484	insulator	insulator
Cd1Cl3Cs1_ICSD_4039	insulator	insulator
Cd1Cl3K1_ICSD_31357	insulator	insulator
Cd1Cl3Rb1_ICSD_200206	insulator	insulator
Cd1Cl3Rb1_ICSD_33552	insulator	insulator
Cd1Cl3Tl1_ICSD_39807	insulator	insulator
Cd1Cl4Cs2_ICSD_16576	insulator	insulator
Cd1Cl4Cu1H8O4_ICSD_280299	insulator	insulator
Cd1Cl4Na2_ICSD_69344	insulator	insulator
Cd1Cl4Rb2_ICSD_51168	insulator	insulator
Cd1Cl6K4_ICSD_60753	insulator	insulator
Cd1Cl6Rb4_ICSD_39622	insulator	insulator
Cd1Cl8Na6_ICSD_35070	insulator	insulator
Cd1Co1Dy4_ICSD_417044	metal	metal
Cd1Co1F6Li1_ICSD_32731	insulator	insulator
Cd1Co1Ho4_ICSD_417045	metal	metal
Cd1Co1La4_ICSD_419067	metal	metal
Cd1Co1Tb4_ICSD_417043	metal	metal
Cd1Co3N1_ICSD_422858	metal	metal
Cd1Cr1F6_ICSD_10346	insulator	insulator
Cd1Cr1O4_ICSD_18118	insulator	insulator
Cd1Cr1O4_ICSD_18119	insulator	insulator
Cd1Cr2H14N4O8_ICSD_80187	insulator	insulator
Cd1Cr2O4_ICSD_37428	insulator	insulator
Cd1Cr2S4_ICSD_619692	insulator	insulator
Cd1Cr2Se4_ICSD_42022	insulator	insulator
Cd1Cr4Cs2H4O16_ICSD_154326	insulator	insulator
Cd1Cs1Dy1Te3_ICSD_173312	insulator	insulator
Cd1Cs1Er1Te3_ICSD_173313	insulator	insulator
Cd1Cs1F3_ICSD_290344	insulator	insulator
Cd1Cs1Ho1Te3_ICSD_173315	insulator	insulator
Cd1Cs1La1Te3_ICSD_173316	insulator	insulator
Cd1Cs1N3O6_ICSD_28649	metal	insulator
Cd1Cs1N3O6_ICSD_95536	insulator	insulator
Cd1Cs1Nd1Te3_ICSD_173318	insulator	insulator
Cd1Cs1Se3Y1_ICSD_281433	insulator	insulator
Cd1Cs1Tb1Te3_ICSD_173320	insulator	insulator
Cd1Cs2I4_ICSD_65654	insulator	insulator
Cd1Cs3I5_ICSD_403103	insulator	insulator
Cd1Cu1Dy1_ICSD_656598	metal	metal

Supplementary Table 240. Five-fold cross validated predictions for the metal/insulator classification (232/598).

system	calculated	predicted
Cd1Cu1F4_ICSD_73478	insulator	insulator
Cd1Cu1O4V1_ICSD_401454	insulator	insulator
Cd1Cu1Pr1_ICSD_656595	metal	metal
Cd1Cu1Sb1_ICSD_52796	metal	metal
Cd1Cu1Sb1_ICSD_619779	metal	metal
Cd1Cu2Gd2_ICSD_419851	metal	metal
Cd1Cu2Ge1S4_ICSD_26150	insulator	insulator
Cd1Cu2Ge1Se4_ICSD_619750	metal	metal
Cd1Cu2Ge1Te4_ICSD_165094	metal	metal
Cd1Cu2Nd2_ICSD_261436	metal	metal
Cd1Cu2Nd2_ICSD_421934	metal	metal
Cd1Cu2S4Si1_ICSD_619770	insulator	insulator
Cd1Cu2S4Sn1_ICSD_619774	metal	insulator
Cd1Cu2Se4Si1_ICSD_619783	insulator	insulator
Cd1Cu2Se4Sn1_ICSD_95118	metal	metal
Cd1Cu2Si1Te4_ICSD_656151	insulator	metal
Cd1Cu2Sn1Te4_ICSD_656157	metal	metal
Cd1Cu2Yb5_ICSD_423860	metal	metal
Cd1Cu2Zr1_ICSD_58961	metal	metal
Cd1Cu2_ICSD_150582	metal	metal
Cd1Cu2_ICSD_58955	metal	metal
Cd1Cu3O12Ti4_ICSD_39467	insulator	insulator
Cd1Cu4Er1_ICSD_415196	metal	metal
Cd1Cu4Ho1_ICSD_415195	metal	metal
Cd1Cu4Yb1_ICSD_415198	metal	metal
Cd1Dy1Ga1_ICSD_619800	metal	metal
Cd1Dy1Ni4_ICSD_185112	metal	metal
Cd1Dy1_ICSD_58962	metal	metal
Cd1Dy2Ge2_ICSD_424175	metal	metal
Cd1Dy2Pd2_ICSD_157388	metal	metal
Cd1Dy2S4_ICSD_52798	insulator	insulator
Cd1Dy2Se4_ICSD_246499	insulator	insulator
Cd1Dy2Te4_ICSD_619806	metal	insulator
Cd1Dy4Rh1_ICSD_417047	metal	metal
Cd1Er1Ga1_ICSD_619819	metal	metal
Cd1Er1Ni4_ICSD_185114	metal	metal
Cd1Er1_ICSD_619817	metal	metal
Cd1Er2Ge2_ICSD_424177	metal	metal
Cd1Er2S4_ICSD_100518	insulator	insulator
Cd1Er2Se4_ICSD_619834	insulator	insulator
Cd1Eu1Pd1_ICSD_412311	metal	metal
Cd1Eu1Sn1_ICSD_619856	metal	metal
Cd1Eu1_ICSD_619835	metal	metal
Cd1F1H1O1_ICSD_27754	insulator	insulator
Cd1F2_ICSD_183501	insulator	insulator
Cd1F2_ICSD_28864	insulator	insulator
Cd1F3K1_ICSD_44788	insulator	insulator
Cd1F3Rb1_ICSD_49587	insulator	insulator
Cd1F5Mn1_ICSD_69633	insulator	insulator
Cd1F6H12O6Sn2_ICSD_166590	insulator	insulator

Supplementary Table 241. Five-fold cross validated predictions for the metal/insulator classification (233/598).

system	calculated	predicted
Cd1F6Pd1_ICSD_26166	insulator	insulator
Cd1F6Pt1_ICSD_37445	insulator	insulator
Cd1F6Rh1_ICSD_42162	insulator	insulator
Cd1F6Sn1_ICSD_25017	insulator	insulator
Cd1F6Tb1_ICSD_59987	metal	insulator
Cd1F6Ti1_ICSD_16233	insulator	insulator
Cd1F7Rb1Zr1_ICSD_245783	insulator	insulator
Cd1F7Tl1Zr1_ICSD_245784	insulator	insulator
Cd1Fe2O4_ICSD_619857	insulator	insulator
Cd1Ga1In1S4_ICSD_20785	insulator	metal
Cd1Ga1In1S4_ICSD_2465	metal	insulator
Cd1Ga1Nd1_ICSD_619868	metal	metal
Cd1Ga1Pr1_ICSD_619870	metal	metal
Cd1Ga2O4_ICSD_159739	insulator	insulator
Cd1Ga2S4_ICSD_619878	insulator	insulator
Cd1Ga2Se4_ICSD_30908	insulator	insulator
Cd1Ga2Te4_ICSD_656264	insulator	insulator
Cd1Gd1Pd1_ICSD_411775	metal	metal
Cd1Gd1_ICSD_108212	metal	metal
Cd1Gd2Ge2_ICSD_424173	metal	metal
Cd1Gd2Pd2_ICSD_157387	metal	metal
Cd1Gd4Ir1_ICSD_419612	metal	metal
Cd1Gd4Ni1_ICSD_419610	metal	metal
Cd1Gd4Pd1_ICSD_419611	metal	metal
Cd1Gd4Pt1_ICSD_419613	metal	metal
Cd1Ge1Li2O4_ICSD_20031	insulator	insulator
Cd1Ge1Li2S4_ICSD_249872	insulator	insulator
Cd1Ge1Li2_ICSD_52803	metal	metal
Cd1Ge1O3_ICSD_23531	insulator	insulator
Cd1Ge1O3_ICSD_30971	insulator	insulator
Cd1Ge1O3_ICSD_77134	insulator	insulator
Cd1Ge1P2_ICSD_52804	metal	metal
Cd1Ge1P2_ICSD_600509	insulator	insulator
Cd1Ge1Sr1_ICSD_619960	metal	metal
Cd1Ge1Te4Tl2_ICSD_172502	insulator	insulator
Cd1Ge1Yb1_ICSD_619963	metal	metal
Cd1Ge2Ho2_ICSD_424176	metal	metal
Cd1Ge2Nd2_ICSD_424171	metal	metal
Cd1Ge2O5_ICSD_23039	insulator	insulator
Cd1Ge2Pr2_ICSD_424170	metal	metal
Cd1Ge2Tb2_ICSD_424174	metal	metal
Cd1Ge2Tm2_ICSD_424178	metal	metal
Cd1Ge2Y2_ICSD_424169	metal	metal
Cd1Ge2Yb2_ICSD_424179	metal	metal
Cd1H10N4O8S2_ICSD_240376	insulator	insulator
Cd1H1N1O4_ICSD_35355	insulator	insulator
Cd1H1O3P1_ICSD_240864	insulator	insulator
Cd1H2I3O1Rb1_ICSD_809	insulator	insulator
Cd1H2O2_ICSD_165225	insulator	insulator
Cd1H2O4Se1_ICSD_59347	insulator	insulator

Supplementary Table 242. Five-fold cross validated predictions for the metal/insulator classification (234/598).

system	calculated	predicted
Cd1H2O5Se1_ICSD_26897	insulator	insulator
Cd1H3N1O5_ICSD_36557	insulator	insulator
Cd1H4Mg1N4O10_ICSD_64618	insulator	insulator
Cd1H4Na2O10S2_ICSD_98681	insulator	insulator
Cd1H4Na2O10Se2_ICSD_98682	insulator	insulator
Cd1H4O4P2_ICSD_171022	insulator	insulator
Cd1H6N1O5P1_ICSD_200085	insulator	insulator
Cd1H6O7P2_ICSD_1382	insulator	insulator
Cd1Hf1_ICSD_619964	metal	metal
Cd1Hf2_ICSD_619965	metal	metal
Cd1Hg1O2_ICSD_74848	insulator	insulator
Cd1Hg2O6S1_ICSD_413287	insulator	insulator
Cd1Hg2O6Se1_ICSD_413288	insulator	insulator
Cd1Hg2Ti1_ICSD_58987	metal	metal
Cd1Hg2_ICSD_58982	metal	metal
Cd1Ho1Ni4_ICSD_185113	metal	metal
Cd1Ho1_ICSD_619987	metal	metal
Cd1Ho2S4_ICSD_620002	insulator	insulator
Cd1Ho2Se4_ICSD_620011	insulator	insulator
Cd1Ho4Rh1_ICSD_417048	metal	metal
Cd1I2O6_ICSD_1397	insulator	insulator
Cd1I2O6_ICSD_415011	insulator	insulator
Cd1I2_ICSD_108921	insulator	insulator
Cd1I2_ICSD_108924	insulator	insulator
Cd1I2_ICSD_108926	insulator	insulator
Cd1I2_ICSD_20745	insulator	insulator
Cd1I2_ICSD_27292	metal	insulator
Cd1I2_ICSD_28384	insulator	insulator
Cd1I2_ICSD_37377	insulator	insulator
Cd1I2_ICSD_37378	insulator	insulator
Cd1I2_ICSD_38116	insulator	insulator
Cd1I2_ICSD_42173	insulator	insulator
Cd1I2_ICSD_42198	insulator	insulator
Cd1I2_ICSD_42205	insulator	insulator
Cd1I2_ICSD_42207	insulator	insulator
Cd1I2_ICSD_42210	insulator	insulator
Cd1I2_ICSD_42227	insulator	insulator
Cd1I2_ICSD_42233	insulator	insulator
Cd1I2_ICSD_42258	insulator	insulator
Cd1I2_ICSD_42262	insulator	insulator
Cd1I2_ICSD_42268	insulator	insulator
Cd1I2_ICSD_42269	insulator	insulator
Cd1I2_ICSD_43443	insulator	insulator
Cd1I2_ICSD_43852	insulator	insulator
Cd1I2_ICSD_44792	insulator	insulator
Cd1I2_ICSD_44793	insulator	insulator
Cd1I2_ICSD_9025	insulator	insulator
Cd1I2_ICSD_9190	insulator	insulator
Cd1I4S8Sb6_ICSD_27733	insulator	insulator
Cd1I6In4_ICSD_66290	insulator	insulator

Supplementary Table 243. Five-fold cross validated predictions for the metal/insulator classification (235/598).

system	calculated	predicted
Cd1I6Tl4_ICSD_66292	insulator	insulator
Cd1In1S2_ICSD_52810	metal	metal
Cd1In2O4_ICSD_159740	insulator	insulator
Cd1In2O4_ICSD_52389	insulator	insulator
Cd1In2S4_ICSD_601181	insulator	insulator
Cd1In2Se4_ICSD_151954	insulator	insulator
Cd1In2Se4_ICSD_151955	insulator	insulator
Cd1In2Se4_ICSD_52811	metal	insulator
Cd1In2Se4_ICSD_620049	insulator	insulator
Cd1In2Te4_ICSD_25651	insulator	insulator
Cd1Ir1O6Sr3_ICSD_81904	insulator	insulator
Cd1K1N3O6_ICSD_28647	insulator	metal
Cd1K1N3O6_ICSD_95538	insulator	insulator
Cd1K1Sb1_ICSD_620064	metal	insulator
Cd1K2N12_ICSD_659621	insulator	insulator
Cd1K2O2_ICSD_25004	insulator	insulator
Cd1K2O7P2_ICSD_12117	insulator	insulator
Cd1K2Pb1_ICSD_10041	metal	metal
Cd1K2Sb2Sr1_ICSD_422273	insulator	metal
Cd1K2Se4Sn1_ICSD_185468	insulator	insulator
Cd1K2Sn1_ICSD_620066	metal	metal
Cd1K4P2_ICSD_61084	insulator	insulator
Cd1K6O4_ICSD_62053	insulator	insulator
Cd1K6Te4_ICSD_420087	insulator	insulator
Cd1La1Pd1_ICSD_656599	metal	metal
Cd1La1Tl1_ICSD_620091	metal	metal
Cd1La1_ICSD_102005	metal	metal
Cd1La1_ICSD_601467	metal	metal
Cd1La2Rh2_ICSD_107389	metal	metal
Cd1La4Ru1_ICSD_419069	metal	metal
Cd1Li1O4P1_ICSD_65050	insulator	insulator
Cd1Li1O4P1_ICSD_71862	insulator	insulator
Cd1Li1P1_ICSD_52813	metal	metal
Cd1Li1_ICSD_620092	metal	metal
Cd1Li2Pb1_ICSD_102015	metal	metal
Cd1Li2Pb1_ICSD_102016	metal	metal
Cd1Li2S4Sn1_ICSD_249873	insulator	insulator
Cd1Li2Sb1_ICSD_52815	metal	metal
Cd1Li2Sn1_ICSD_108223	metal	metal
Cd1Lu1Ni4_ICSD_423941	metal	metal
Cd1Lu1_ICSD_620119	metal	metal
Cd1Lu2S4_ICSD_620127	insulator	insulator
Cd1Lu2Se4_ICSD_620129	insulator	insulator
Cd1Mg1_ICSD_102022	metal	metal
Cd1Mg3_ICSD_620134	metal	metal
Cd1Mn2O4_ICSD_24258	insulator	insulator
Cd1Mo1O4_ICSD_30371	insulator	insulator
Cd1Mo1O6P1_ICSD_82090	insulator	insulator
Cd1Mo1O6Te1_ICSD_93794	insulator	insulator
Cd1Mo3O12Th1_ICSD_26012	insulator	insulator

Supplementary Table 244. Five-fold cross validated predictions for the metal/insulator classification (236/598).

system	calculated	predicted
Cd1Mo6S8_ICSD_620169	metal	metal
Cd1Mo6Se8_ICSD_620174	metal	metal
Cd1N1Ni3_ICSD_422859	metal	metal
Cd1N1_ICSD_183197	metal	metal
Cd1N1_ICSD_185576	metal	metal
Cd1N1_ICSD_186891	metal	metal
Cd1N1_ICSD_186892	metal	metal
Cd1N1_ICSD_186894	metal	metal
Cd1N2O6_ICSD_297	insulator	insulator
Cd1N3O6Rb1_ICSD_28648	metal	insulator
Cd1N3O6Rb1_ICSD_95537	insulator	insulator
Cd1N3O6Tl1_ICSD_28650	insulator	metal
Cd1N3O6Tl1_ICSD_95539	insulator	insulator
Cd1Na1O4P1_ICSD_10153	insulator	insulator
Cd1Na1O4P1_ICSD_6210	insulator	insulator
Cd1Na1O4V1_ICSD_151411	insulator	insulator
Cd1Na1Sb1_ICSD_12159	metal	insulator
Cd1Na2O8S2_ICSD_173033	insulator	insulator
Cd1Na2Pb1_ICSD_102033	metal	metal
Cd1Na2S4Sn1_ICSD_281233	insulator	insulator
Cd1Na2Sb2Yb1_ICSD_422274	insulator	metal
Cd1Na2Sn1_ICSD_102034	metal	metal
Cd1Na4P2_ICSD_67262	insulator	insulator
Cd1Nd1Ni4_ICSD_185109	metal	metal
Cd1Nd1Tl1_ICSD_620196	metal	metal
Cd1Nd1_ICSD_102036	metal	metal
Cd1Nd1_ICSD_186649	metal	metal
Cd1Nd2Ni2_ICSD_414597	metal	metal
Cd1Ni1_ICSD_102038	metal	metal
Cd1Ni1_ICSD_620198	metal	metal
Cd1Ni2Pr2_ICSD_414596	metal	metal
Cd1Ni4Sc1_ICSD_185106	metal	metal
Cd1Ni4Tb1_ICSD_185111	metal	metal
Cd1Ni4Y1_ICSD_423939	metal	metal
Cd1Ni4Yb1_ICSD_185116	metal	metal
Cd1O14P4V2_ICSD_74554	insulator	insulator
Cd1O1_ICSD_161837	metal	insulator
Cd1O1_ICSD_181735	metal	metal
Cd1O2Rb2_ICSD_62054	insulator	insulator
Cd1O2_ICSD_36151	insulator	insulator
Cd1O3S1_ICSD_62640	insulator	insulator
Cd1O3S1_ICSD_62641	insulator	insulator
Cd1O3Se1_ICSD_75273	insulator	insulator
Cd1O3Se1_ICSD_75274	insulator	insulator
Cd1O3Sn1_ICSD_180401	insulator	insulator
Cd1O3Te1_ICSD_60067	insulator	insulator
Cd1O3Ti1_ICSD_15989	insulator	insulator
Cd1O3Ti1_ICSD_16686	insulator	insulator
Cd1O3Ti1_ICSD_33667	metal	insulator
Cd1O3Ti1_ICSD_62151	insulator	insulator

Supplementary Table 245. Five-fold cross validated predictions for the metal/insulator classification (237/598).

system	calculated	predicted
Cd1O3V1_ICSD_88387	insulator	insulator
Cd1O4Pd3_ICSD_16539	metal	metal
Cd1O4Rh2_ICSD_28954	insulator	insulator
Cd1O4S1_ICSD_130014	insulator	insulator
Cd1O4S1_ICSD_60571	insulator	insulator
Cd1O4S1_ICSD_9723	insulator	insulator
Cd1O4U1_ICSD_20504	insulator	insulator
Cd1O4U1_ICSD_26427	insulator	insulator
Cd1O4U1_ICSD_26428	insulator	insulator
Cd1O4V2_ICSD_28961	metal	metal
Cd1O4W1_ICSD_186159	insulator	insulator
Cd1O5Se2_ICSD_75230	insulator	insulator
Cd1O6P2_ICSD_15861	insulator	insulator
Cd1O6P2_ICSD_8070	insulator	insulator
Cd1O6Pt1Sr3_ICSD_280518	insulator	insulator
Cd1O6Pt3_ICSD_35407	insulator	insulator
Cd1O6Sb2_ICSD_181929	insulator	insulator
Cd1O6Sr2W1_ICSD_245684	insulator	insulator
Cd1O6Sr2W1_ICSD_71839	insulator	insulator
Cd1O6Sr2W1_ICSD_71840	insulator	insulator
Cd1O6V2_ICSD_15926	insulator	insulator
Cd1O7S2_ICSD_63067	insulator	insulator
Cd1P1Pd5_ICSD_620223	metal	metal
Cd1P1Pt5_ICSD_620224	metal	metal
Cd1P1S3_ICSD_61393	insulator	insulator
Cd1P1S3_ICSD_80875	insulator	insulator
Cd1P2Rb2Se6_ICSD_50959	insulator	insulator
Cd1P2Si1_ICSD_44260	insulator	insulator
Cd1P2Sn1_ICSD_620246	metal	insulator
Cd1P2_ICSD_26199	insulator	insulator
Cd1P2_ICSD_620210	insulator	insulator
Cd1P2_ICSD_62336	insulator	insulator
Cd1P4_ICSD_25605	insulator	insulator
Cd1Pb1Yb1_ICSD_620264	metal	metal
Cd1Pd1Pr1_ICSD_106366	metal	metal
Cd1Pd1_ICSD_620267	metal	metal
Cd1Pd2Pr2_ICSD_157385	metal	metal
Cd1Pd2Sm2_ICSD_157386	metal	metal
Cd1Pd2Yb2_ICSD_157389	metal	metal
Cd1Pd5Se1_ICSD_620273	metal	metal
Cd1Pr1Tl1_ICSD_620292	metal	metal
Cd1Pr1_ICSD_102049	metal	metal
Cd1Pr1_ICSD_182833	metal	metal
Cd1Pt1_ICSD_620297	metal	metal
Cd1Pt2Zn1_ICSD_102057	metal	metal
Cd1Rh1Tb4_ICSD_417046	metal	metal
Cd1Ru1Y4_ICSD_419070	metal	metal
Cd1S1_ICSD_31075	insulator	insulator
Cd1S1_ICSD_600773	insulator	insulator
Cd1S1_ICSD_60629	insulator	insulator

Supplementary Table 246. Five-fold cross validated predictions for the metal/insulator classification (238/598).

system	calculated	predicted
Cd1S1_ICSD_620315	insulator	insulator
Cd1S2Ta1_ICSD_620350	metal	metal
Cd1S2Tl1_ICSD_164949	metal	metal
Cd1S2Tl1_ICSD_20320	metal	metal
Cd1S2_ICSD_620305	insulator	insulator
Cd1S4Sc2_ICSD_94994	insulator	insulator
Cd1S4Tm2_ICSD_620360	metal	insulator
Cd1S4Y2_ICSD_620371	insulator	insulator
Cd1S4Yb2_ICSD_37409	metal	metal
Cd1Sb1Tl1_ICSD_9572	metal	metal
Cd1Sb1_ICSD_620395	insulator	metal
Cd1Sb2Sn1_ICSD_44259	metal	metal
Cd1Sb2Yb2_ICSD_173171	metal	metal
Cd1Sc1_ICSD_620407	metal	metal
Cd1Sc2Se4_ICSD_620411	insulator	insulator
Cd1Se1_ICSD_180931	insulator	insulator
Cd1Se1_ICSD_181026	metal	insulator
Cd1Se1_ICSD_415785	insulator	insulator
Cd1Se2Tl1_ICSD_620451	metal	insulator
Cd1Se2_ICSD_620416	insulator	insulator
Cd1Se4Y2_ICSD_620457	insulator	insulator
Cd1Se4Yb2_ICSD_620459	metal	metal
Cd1Sm1Tl1_ICSD_620482	metal	metal
Cd1Sm1_ICSD_102063	metal	metal
Cd1Sn1Te4Tl2_ICSD_172503	insulator	insulator
Cd1Sn1Yb1_ICSD_620489	metal	metal
Cd1Sr1_ICSD_102066	metal	metal
Cd1Tb1_ICSD_108236	metal	metal
Cd1Te1_ICSD_108237	metal	metal
Cd1Te1_ICSD_150941	insulator	insulator
Cd1Te1_ICSD_246692	metal	insulator
Cd1Te1_ICSD_620514	insulator	insulator
Cd1Te1_ICSD_620526	metal	metal
Cd1Te1_ICSD_67863	insulator	metal
Cd1Te2Tl1_ICSD_620548	metal	metal
Cd1Te2Zn1_ICSD_181019	insulator	insulator
Cd1Te4Tl2_ICSD_620549	metal	insulator
Cd1Ti1_ICSD_102076	metal	metal
Cd1Ti2_ICSD_102077	metal	metal
Cd1Tm1_ICSD_620576	insulator	metal
Cd1Tm1_ICSD_620577	metal	metal
Cd1V3_ICSD_102082	metal	metal
Cd1V3_ICSD_620584	metal	metal
Cd1Y1_ICSD_188181	metal	metal
Cd1Yb1_ICSD_620602	metal	metal
Cd1Zr1_ICSD_620612	metal	metal
Cd1Zr2_ICSD_620613	metal	metal
Cd1_ICSD_53770	metal	metal
Cd23Th6_ICSD_620562	metal	metal
Cd2Ce1_ICSD_58951	metal	metal

Supplementary Table 247. Five-fold cross validated predictions for the metal/insulator classification (239/598).

system	calculated	predicted
Cd2Ce1_ICSD_619647	metal	metal
Cd2Ce1_ICSD_619656	metal	metal
Cd2Cl1H3O3_ICSD_39285	insulator	insulator
Cd2Cl1P3_ICSD_100818	insulator	insulator
Cd2Cl1P3_ICSD_100879	insulator	insulator
Cd2Cl2P1_ICSD_412647	insulator	insulator
Cd2Cs5Tl11_ICSD_165200	metal	metal
Cd2Cu1Er1_ICSD_99139	metal	metal
Cd2Cu1O8P2_ICSD_249777	insulator	insulator
Cd2Dy1_ICSD_58964	metal	metal
Cd2Dy1_ICSD_619796	metal	metal
Cd2Er1_ICSD_58968	metal	metal
Cd2Er1_ICSD_619813	metal	metal
Cd2Er1_ICSD_619816	metal	metal
Cd2Eu1P2_ICSD_78987	insulator	metal
Cd2Eu1Sb2_ICSD_78988	metal	metal
Cd2Eu1_ICSD_58972	metal	metal
Cd2F1O4P1_ICSD_2361	insulator	insulator
Cd2F8Tb1_ICSD_86146	metal	insulator
Cd2Ga2Sr1_ICSD_370021	metal	metal
Cd2Gd1_ICSD_58976	metal	metal
Cd2Gd1_ICSD_619915	metal	metal
Cd2Ge2Sr1_ICSD_25331	metal	metal
Cd2H2O6S1_ICSD_652	insulator	insulator
Cd2H4O4_ICSD_40186	insulator	insulator
Cd2Hg1_ICSD_58983	metal	metal
Cd2Ho1_ICSD_58989	metal	metal
Cd2Ho1_ICSD_619991	metal	metal
Cd2I1P3_ICSD_100816	insulator	insulator
Cd2I1P3_ICSD_100881	insulator	insulator
Cd2I7Tl3_ICSD_63340	insulator	insulator
Cd2Ir1Na3O6_ICSD_404507	insulator	insulator
Cd2K2O12S3_ICSD_200291	insulator	insulator
Cd2K2O12S3_ICSD_40279	insulator	insulator
Cd2K2O3_ICSD_16223	insulator	insulator
Cd2La1Pd1_ICSD_417029	metal	metal
Cd2La1_ICSD_601468	metal	metal
Cd2La1_ICSD_620079	metal	metal
Cd2Lu1_ICSD_620120	metal	metal
Cd2Mn3O8_ICSD_16957	insulator	insulator
Cd2Na14O9_ICSD_2195	insulator	insulator
Cd2Na4O10Si3_ICSD_20185	insulator	insulator
Cd2Nb2O7_ICSD_169000	insulator	insulator
Cd2Nb2O7_ICSD_33672	insulator	insulator
Cd2Nb2O7_ICSD_75601	insulator	insulator
Cd2Nd1Pd1_ICSD_425064	metal	metal
Cd2Nd1_ICSD_620185	metal	metal
Cd2O12Rb2S3_ICSD_281315	insulator	insulator
Cd2O12S3Tl2_ICSD_66714	insulator	insulator
Cd2O4Si1_ICSD_161025	insulator	insulator

Supplementary Table 248. Five-fold cross validated predictions for the metal/insulator classification (240/598).

system	calculated	predicted
Cd2O4Si1_ICSD_50531	insulator	insulator
Cd2O4Sn1_ICSD_69297	insulator	insulator
Cd2O5U1_ICSD_166737	insulator	insulator
Cd2O7Os2_ICSD_155769	metal	metal
Cd2O7P2_ICSD_23542	insulator	insulator
Cd2O7Re2_ICSD_28445	metal	metal
Cd2O7Ru2_ICSD_86773	metal	metal
Cd2O7Sb2_ICSD_165168	insulator	insulator
Cd2O7Sb2_ICSD_77064	insulator	insulator
Cd2O7Ta2_ICSD_77061	insulator	insulator
Cd2O7Tc2_ICSD_180008	metal	insulator
Cd2O7V2_ICSD_24191	insulator	insulator
Cd2P2Se6_ICSD_620234	insulator	insulator
Cd2P2Sr1_ICSD_30912	insulator	insulator
Cd2Pd1Sr1_ICSD_425494	metal	metal
Cd2Pr1_ICSD_102050	metal	metal
Cd2Pr1_ICSD_601470	metal	metal
Cd2Pr1_ICSD_620282	metal	metal
Cd2Sb2Sr1_ICSD_12153	insulator	metal
Cd2Sb2Yb1_ICSD_78989	insulator	metal
Cd2Sm1_ICSD_102065	metal	metal
Cd2Sm1_ICSD_620476	metal	metal
Cd2Sr1_ICSD_420579	metal	metal
Cd2Tb1_ICSD_102069	metal	metal
Cd2Tb1_ICSD_620503	metal	metal
Cd2Th1_ICSD_15446	metal	metal
Cd2Tm1_ICSD_102079	metal	metal
Cd2Y1_ICSD_102084	metal	metal
Cd2Yb1_ICSD_620601	metal	metal
Cd2Zr3_ICSD_102092	metal	metal
Cd32K6_ICSD_421370	metal	metal
Cd3Ce1_ICSD_619645	metal	metal
Cd3Cl2O2_ICSD_300028	insulator	insulator
Cd3Cl3P1_ICSD_201025	insulator	insulator
Cd3Cs2K2O5_ICSD_60895	insulator	insulator
Cd3Dy1_ICSD_58965	metal	metal
Cd3Er1_ICSD_2834	metal	metal
Cd3Gd1_ICSD_58977	metal	metal
Cd3Ge3O12Sc2_ICSD_20216	insulator	insulator
Cd3Ho1_ICSD_58990	metal	metal
Cd3In1_ICSD_109285	metal	metal
Cd3K2Se4_ICSD_710063	insulator	insulator
Cd3Mg1_ICSD_102027	metal	metal
Cd3N2_ICSD_416908	metal	metal
Cd3Na2O10Si3_ICSD_28416	insulator	insulator
Cd3Na2O14P4_ICSD_93593	insulator	insulator
Cd3Nb1_ICSD_102035	metal	metal
Cd3O10Se3_ICSD_280951	insulator	insulator
Cd3O5Si1_ICSD_23170	insulator	insulator
Cd3O6Te1_ICSD_35084	insulator	insulator

Supplementary Table 249. Five-fold cross validated predictions for the metal/insulator classification (241/598).

system	calculated	predicted
Cd3O8P2_ICSD_20202	insulator	insulator
Cd3P2_ICSD_24488	metal	metal
Cd3P2_ICSD_620218	metal	metal
Cd3P2_ICSD_620219	metal	metal
Cd3P3Pr1_ICSD_88676	metal	metal
Cd3Sb3Tl2_ICSD_76500	insulator	metal
Cd3Sc1_ICSD_102062	metal	metal
Cd3Sr5_ICSD_106369	metal	metal
Cd3Tb1_ICSD_102070	metal	metal
Cd3Tb1_ICSD_102071	metal	metal
Cd3Th1_ICSD_620565	metal	metal
Cd3Y1_ICSD_102085	metal	metal
Cd3Y1_ICSD_2835	metal	metal
Cd3Zr1_ICSD_102093	metal	metal
Cd41Ir8_ICSD_189255	metal	metal
Cd4Cl3P2_ICSD_50938	insulator	insulator
Cd4Cl6O13Te6_ICSD_171334	insulator	insulator
Cd4F6O1_ICSD_74031	insulator	insulator
Cd4Ge1S6_ICSD_26214	insulator	insulator
Cd4Ge1Se6_ICSD_600752	insulator	insulator
Cd4I3P2_ICSD_23307	insulator	insulator
Cd4I3Sb2_ICSD_80589	insulator	insulator
Cd4K1O12V3_ICSD_807	insulator	insulator
Cd4K1P3_ICSD_262033	metal	insulator
Cd4K6Se13Sn3_ICSD_413739	insulator	insulator
Cd4La6Pd13_ICSD_421178	metal	metal
Cd4N12P6S1_ICSD_71019	insulator	insulator
Cd4Pd13Pr6_ICSD_417049	metal	metal
Cd4S6Si1_ICSD_620339	insulator	insulator
Cd4Se6Si1_ICSD_96265	insulator	insulator
Cd5Cl1O12P3_ICSD_130	insulator	insulator
Cd5Cu2_ICSD_130027	metal	metal
Cd5Th1_ICSD_620559	metal	metal
Cd6Sb12Sr11_ICSD_413701	insulator	metal
Cd6Sb5_ICSD_52832	metal	metal
Cd7Cl6P4_ICSD_38044	insulator	insulator
Cd7P10_ICSD_200596	insulator	insulator
Cd7Sc1_ICSD_55553	metal	metal
Cd7Th6_ICSD_102075	metal	metal
Cd9K14Tl21_ICSD_400782	metal	metal
Ce1Cl1O1_ICSD_412069	metal	insulator
Ce1Cl1O1_ICSD_72154	insulator	metal
Ce1Cl1Te1_ICSD_426281	insulator	insulator
Ce1Cl1Te1_ICSD_426282	metal	insulator
Ce1Cl3_ICSD_31575	insulator	insulator
Ce1Cl6Cs2_ICSD_14339	insulator	insulator
Ce1Co1Ge1H1_ICSD_98944	metal	metal
Ce1Co1Ge2_ICSD_620738	metal	metal
Ce1Co1Ge3_ICSD_620734	metal	metal
Ce1Co1In5_ICSD_102108	metal	metal

Supplementary Table 250. Five-fold cross validated predictions for the metal/insulator classification (242/598).

system	calculated	predicted
Ce1Co1Sb2_ICSD_657920	metal	metal
Ce1Co1Sb2_ICSD_657991	metal	metal
Ce1Co1Sb3_ICSD_419539	metal	metal
Ce1Co1Si1_ICSD_620788	metal	metal
Ce1Co1Si2_ICSD_16501	metal	metal
Ce1Co1Si3_ICSD_620795	metal	metal
Ce1Co2Ge2_ICSD_106847	metal	metal
Ce1Co2N12O24Rb5_ICSD_24534	metal	metal
Ce1Co2P2_ICSD_85893	metal	metal
Ce1Co2P2_ICSD_85895	metal	metal
Ce1Co2Si2_ICSD_657673	metal	metal
Ce1Co2_ICSD_620652	metal	metal
Ce1Co2_ICSD_620681	metal	metal
Ce1Co5_ICSD_54671	metal	metal
Ce1Co5_ICSD_620665	metal	metal
Ce1Co5_ICSD_620696	metal	metal
Ce1Co5_ICSD_659629	metal	metal
Ce1Co8Mn4_ICSD_620758	metal	metal
Ce1Co9Ge4_ICSD_166197	metal	metal
Ce1Co9Si4_ICSD_169484	metal	metal
Ce1Cr1Ge3_ICSD_158977	metal	metal
Ce1Cr1O3_ICSD_28931	insulator	metal
Ce1Cr1S3_ICSD_88975	insulator	insulator
Ce1Cs1Cu1S3_ICSD_156245	insulator	insulator
Ce1Cs1Hg1Se3_ICSD_281442	insulator	insulator
Ce1Cs1S2_ICSD_73533	insulator	insulator
Ce1Cu1Ge1_ICSD_106377	metal	metal
Ce1Cu1Ge1_ICSD_99673	metal	metal
Ce1Cu1Ge2_ICSD_620854	metal	metal
Ce1Cu1In1_ICSD_620875	metal	metal
Ce1Cu1Mg2_ICSD_102135	metal	metal
Ce1Cu1O1S1_ICSD_96344	insulator	insulator
Ce1Cu1O1Te1_ICSD_416521	insulator	insulator
Ce1Cu1Pb1_ICSD_620898	metal	metal
Ce1Cu1S2_ICSD_415076	insulator	insulator
Ce1Cu1Sb2_ICSD_658226	metal	metal
Ce1Cu1Se2_ICSD_99676	insulator	insulator
Ce1Cu1Si1_ICSD_30760	metal	metal
Ce1Cu1Si2_ICSD_620916	metal	metal
Ce1Cu1Sn1_ICSD_416788	metal	metal
Ce1Cu1Sn1_ICSD_600999	metal	metal
Ce1Cu1_ICSD_102122	metal	metal
Ce1Cu2Ge2_ICSD_246608	metal	metal
Ce1Cu2In1_ICSD_106379	metal	metal
Ce1Cu2Li1P2_ICSD_620884	metal	metal
Ce1Cu2Mg1_ICSD_157110	metal	metal
Ce1Cu2N12O24Tl5_ICSD_24547	metal	metal
Ce1Cu2Sb2_ICSD_658101	metal	metal
Ce1Cu2Si2_ICSD_620930	metal	metal
Ce1Cu2Sn2_ICSD_620958	metal	metal

Supplementary Table 251. Five-fold cross validated predictions for the metal/insulator classification (243/598).

system	calculated	predicted
Ce1Cu2Zn3_ICSD_99222	metal	metal
Ce1Cu2_ICSD_102124	metal	metal
Ce1Cu2_ICSD_620830	metal	metal
Ce1Cu3Mn4O12_ICSD_169042	metal	metal
Ce1Cu4Sn1_ICSD_110771	metal	metal
Ce1Cu5Sn1_ICSD_101011	metal	metal
Ce1Cu5_ICSD_620841	metal	metal
Ce1Cu6_ICSD_102127	metal	metal
Ce1Cu6_ICSD_174100	metal	metal
Ce1Cu6_ICSD_620842	metal	metal
Ce1Cu9Mg2_ICSD_245210	metal	metal
Ce1Dy1S3_ICSD_200015	insulator	insulator
Ce1Er3S6_ICSD_620979	insulator	insulator
Ce1F1S1_ICSD_31939	metal	insulator
Ce1F3_ICSD_4	insulator	insulator
Ce1F3_ICSD_42470	insulator	insulator
Ce1F3_ICSD_64720	insulator	insulator
Ce1F4K1_ICSD_23229	insulator	insulator
Ce1F7Zr1_ICSD_39776	insulator	insulator
Ce1Fe1Sb2_ICSD_657915	metal	metal
Ce1Fe1Si1_ICSD_656908	metal	metal
Ce1Fe1Si2_ICSD_621071	metal	metal
Ce1Fe2Ge2_ICSD_106824	metal	metal
Ce1Fe2K5N12O24_ICSD_24531	metal	metal
Ce1Fe2N12O24Tl5_ICSD_24532	metal	metal
Ce1Fe2Si2_ICSD_621068	metal	metal
Ce1Fe2Si2_ICSD_657671	metal	metal
Ce1Fe2_ICSD_620992	metal	metal
Ce1Fe2_ICSD_620994	metal	metal
Ce1Fe4P12_ICSD_621050	insulator	metal
Ce1Fe4Sb12_ICSD_621065	metal	metal
Ce1Fe5_ICSD_102147	metal	metal
Ce1Ga1Ni1_ICSD_102171	metal	metal
Ce1Ga1O3_ICSD_76048	metal	metal
Ce1Ga1Zn1_ICSD_621158	metal	metal
Ce1Ga1_ICSD_621093	metal	metal
Ce1Ga1_ICSD_621099	metal	metal
Ce1Ga2Ni1_ICSD_188330	metal	metal
Ce1Ga2_ICSD_102165	metal	metal
Ce1Ga2_ICSD_102166	metal	metal
Ce1Ga2_ICSD_621110	metal	metal
Ce1Ga2_ICSD_621122	metal	metal
Ce1Ga3Pd2_ICSD_602952	metal	metal
Ce1Ga6Pd1_ICSD_240161	metal	metal
Ce1Ga6_ICSD_102167	metal	metal
Ce1Ge12Pt4_ICSD_174553	metal	metal
Ce1Ge1Ir1_ICSD_621210	metal	metal
Ce1Ge1Li2_ICSD_52859	metal	metal
Ce1Ge1Mn1_ICSD_80010	metal	metal
Ce1Ge1Mn1_ICSD_85858	metal	metal

Supplementary Table 252. Five-fold cross validated predictions for the metal/insulator classification (244/598).

system	calculated	predicted
Ce1Ge1Ni1_ICSD_99671	metal	metal
Ce1Ge1Pd2_ICSD_621240	metal	metal
Ce1Ge1Pt1_ICSD_410358	metal	metal
Ce1Ge1Rh1_ICSD_414508	metal	metal
Ce1Ge1Sc1_ICSD_52866	metal	metal
Ce1Ge1Ti1_ICSD_107618	metal	metal
Ce1Ge1Zn1_ICSD_163343	metal	metal
Ce1Ge1Zn1_ICSD_420186	metal	metal
Ce1Ge1_ICSD_621180	metal	metal
Ce1Ge1_ICSD_621196	metal	metal
Ce1Ge2Ir2_ICSD_621212	metal	metal
Ce1Ge2Li1_ICSD_52860	metal	metal
Ce1Ge2Mn2_ICSD_621222	metal	metal
Ce1Ge2Ni1_ICSD_603231	metal	metal
Ce1Ge2Ni2_ICSD_106849	metal	metal
Ce1Ge2Pd2_ICSD_621242	metal	metal
Ce1Ge2Pt2_ICSD_52863	metal	metal
Ce1Ge2Pt2_ICSD_61371	metal	metal
Ce1Ge2Pt2_ICSD_621247	metal	metal
Ce1Ge2Rh2_ICSD_621251	metal	metal
Ce1Ge2Ru2_ICSD_621263	metal	metal
Ce1Ge2_ICSD_621193	metal	metal
Ce1Ge3Ir1_ICSD_161868	metal	metal
Ce1Ge3Rh1_ICSD_161867	metal	metal
Ce1Ge3Rh1_ICSD_621252	metal	metal
Ce1Ge3Ru1_ICSD_246698	metal	metal
Ce1Ge3V1_ICSD_164892	metal	metal
Ce1Ge4Ni9_ICSD_184400	metal	metal
Ce1Ge4Rh6_ICSD_425231	metal	metal
Ce1Ge5_ICSD_246812	metal	metal
Ce1H1Ru1Si1_ICSD_161825	metal	metal
Ce1H1Se1_ICSD_78956	metal	insulator
Ce1H2Ni1Sn1_ICSD_157930	metal	metal
Ce1H2_ICSD_44875	metal	metal
Ce1H3_ICSD_621290	metal	metal
Ce1H8O12S2_ICSD_417358	insulator	insulator
Ce1H8O7P3_ICSD_88339	insulator	insulator
Ce1Hg1Pd1_ICSD_657951	metal	metal
Ce1Hg1_ICSD_621334	metal	metal
Ce1Hg2_ICSD_102181	metal	metal
Ce1Hg3_ICSD_621337	metal	metal
Ce1I1S1_ICSD_23490	insulator	insulator
Ce1I4O12_ICSD_20033	insulator	insulator
Ce1In1Ir1_ICSD_100002	metal	metal
Ce1In1Ni1_ICSD_167627	metal	metal
Ce1In1Ni1_ICSD_621377	metal	metal
Ce1In1Pd1_ICSD_157625	metal	metal
Ce1In1Pd1_ICSD_160860	metal	metal
Ce1In1Pd1_ICSD_59883	metal	metal
Ce1In1Pd1_ICSD_602649	metal	metal

Supplementary Table 253. Five-fold cross validated predictions for the metal/insulator classification (245/598).

system	calculated	predicted
Ce1In1Pd2_ICSD_106406	metal	metal
Ce1In1Pt1_ICSD_418186	metal	metal
Ce1In1Pt1_ICSD_621382	metal	metal
Ce1In1Pt4_ICSD_621380	metal	metal
Ce1In1Rh1_ICSD_602648	metal	metal
Ce1In1Rh1_ICSD_621385	metal	metal
Ce1In1Zn1_ICSD_621394	metal	metal
Ce1In2Ir1_ICSD_414474	metal	metal
Ce1In2Ni9_ICSD_600164	metal	metal
Ce1In2Pd1_ICSD_150132	metal	metal
Ce1In2Pt2_ICSD_54553	metal	metal
Ce1In3_ICSD_171678	metal	metal
Ce1In4Pd2_ICSD_54733	metal	metal
Ce1In5Ir1_ICSD_150225	metal	metal
Ce1In5Rh1_ICSD_110778	metal	metal
Ce1Ir1P1_ICSD_414515	metal	metal
Ce1Ir1Sb1_ICSD_420488	metal	metal
Ce1Ir1Si1_ICSD_413854	metal	metal
Ce1Ir1Si2_ICSD_602571	metal	metal
Ce1Ir1Sn1_ICSD_415903	metal	metal
Ce1Ir2P2_ICSD_186990	metal	metal
Ce1Ir2Si2_ICSD_411678	metal	metal
Ce1Ir2Si2_ICSD_621424	metal	metal
Ce1Ir2Si2_ICSD_621426	metal	metal
Ce1Ir2Sn2_ICSD_621436	metal	metal
Ce1Ir2Zn20_ICSD_290421	metal	metal
Ce1Ir2_ICSD_621398	metal	metal
Ce1Ir3Si2_ICSD_52877	metal	metal
Ce1Ir3Si2_ICSD_79221	metal	metal
Ce1Ir3_ICSD_621405	metal	metal
Ce1Ir3_ICSD_99244	metal	metal
Ce1Ir5_ICSD_102195	metal	metal
Ce1Ir5_ICSD_621409	metal	metal
Ce1K1S2_ICSD_621439	insulator	insulator
Ce1K1S4Si1_ICSD_87946	insulator	insulator
Ce1K1S4Si1_ICSD_95774	insulator	insulator
Ce1K2O8P2_ICSD_173150	insulator	insulator
Ce1K3O8P2_ICSD_65193	insulator	insulator
Ce1Li1O2_ICSD_47116	insulator	insulator
Ce1Li1Si2_ICSD_621473	metal	metal
Ce1Li1Sn1_ICSD_106409	metal	metal
Ce1Li1Sn2_ICSD_102212	metal	metal
Ce1Li2N2_ICSD_34003	insulator	insulator
Ce1Li2P2_ICSD_42016	insulator	insulator
Ce1Li2Sb2_ICSD_36018	metal	metal
Ce1Li8O6_ICSD_61219	insulator	insulator
Ce1Mg12_ICSD_621495	metal	metal
Ce1Mg1Ni4_ICSD_107421	metal	metal
Ce1Mg1Pd1_ICSD_412401	metal	metal
Ce1Mg1Pt1_ICSD_412400	metal	metal

Supplementary Table 254. Five-fold cross validated predictions for the metal/insulator classification (246/598).

system	calculated	predicted
Ce1Mg1Sn1_ICSD_182477	metal	metal
Ce1Mg1Tl1_ICSD_414440	metal	metal
Ce1Mg1Zn2_ICSD_249342	metal	metal
Ce1Mg1_ICSD_102215	metal	metal
Ce1Mg2Ni9_ICSD_55615	metal	metal
Ce1Mg2Si2_ICSD_16180	metal	metal
Ce1Mg2_ICSD_621499	metal	metal
Ce1Mg3_ICSD_621489	metal	metal
Ce1Mn1Ni4_ICSD_262487	metal	metal
Ce1Mn1O1Sb1_ICSD_419356	metal	metal
Ce1Mn1Si1_ICSD_621538	metal	metal
Ce1Mn1Si1_ICSD_75051	metal	metal
Ce1Mn1Si1_ICSD_85850	metal	metal
Ce1Mn1Si2_ICSD_106410	metal	metal
Ce1Mn1Si2_ICSD_106411	metal	metal
Ce1Mn2Si2_ICSD_621535	metal	metal
Ce1Mo6S8_ICSD_603456	metal	metal
Ce1Mo6Se8_ICSD_621544	insulator	metal
Ce1N12Ni2O24Tl5_ICSD_24542	metal	metal
Ce1N1O2Si1_ICSD_9751	insulator	insulator
Ce1N1_ICSD_621559	metal	metal
Ce1N2Sr1_ICSD_95805	insulator	insulator
Ce1Na1Se2_ICSD_621570	insulator	insulator
Ce1Nb1O4_ICSD_414675	insulator	insulator
Ce1Ni1P1_ICSD_621627	metal	metal
Ce1Ni1Sb2_ICSD_84529	metal	metal
Ce1Ni1Si1_ICSD_152749	metal	metal
Ce1Ni1Si2_ICSD_42711	metal	metal
Ce1Ni1Sn1_ICSD_102236	metal	metal
Ce1Ni1Sn2_ICSD_621687	metal	metal
Ce1Ni1Zn1_ICSD_656582	metal	metal
Ce1Ni1_ICSD_621583	metal	metal
Ce1Ni1_ICSD_621584	metal	metal
Ce1Ni2P2_ICSD_621626	metal	metal
Ce1Ni2Sb2_ICSD_602480	metal	metal
Ce1Ni2Si2_ICSD_621678	metal	metal
Ce1Ni2Sn2_ICSD_602690	metal	metal
Ce1Ni2Sn2_ICSD_621697	metal	metal
Ce1Ni2_ICSD_621609	metal	metal
Ce1Ni4Sn2_ICSD_102238	metal	metal
Ce1Ni5Sn1_ICSD_621684	metal	metal
Ce1Ni5_ICSD_102232	metal	metal
Ce1Ni5_ICSD_621615	metal	metal
Ce1Ni9Si4_ICSD_98408	metal	metal
Ce1O16Zr7_ICSD_164737	insulator	insulator
Ce1O1P1Ru1_ICSD_80201	metal	metal
Ce1O1P1Zn1_ICSD_416475	insulator	insulator
Ce1O1Sb1Zn1_ICSD_85449	insulator	metal
Ce1O1_ICSD_52886	metal	metal
Ce1O2_ICSD_169031	insulator	insulator

Supplementary Table 255. Five-fold cross validated predictions for the metal/insulator classification (247/598).

system	calculated	predicted
Ce1O2_ICSD_189287	insulator	insulator
Ce1O2_ICSD_28753	insulator	metal
Ce1O3Sr1_ICSD_154927	insulator	insulator
Ce1O3V1_ICSD_162747	insulator	metal
Ce1O3V1_ICSD_28926	metal	metal
Ce1O4P1_ICSD_182582	insulator	insulator
Ce1O4P1_ICSD_182583	metal	insulator
Ce1O4P1_ICSD_182586	insulator	insulator
Ce1O4P1_ICSD_184550	insulator	insulator
Ce1O4P1_ICSD_22265	insulator	insulator
Ce1O4P1_ICSD_27859	insulator	insulator
Ce1O4P1_ICSD_31563	insulator	insulator
Ce1O4P1_ICSD_39135	insulator	insulator
Ce1O4Si1_ICSD_92040	insulator	insulator
Ce1O4Sr2_ICSD_86768	insulator	insulator
Ce1O4Ta1_ICSD_415427	insulator	insulator
Ce1O4V1_ICSD_183203	insulator	insulator
Ce1O4V1_ICSD_183206	metal	insulator
Ce1O4V1_ICSD_78075	insulator	insulator
Ce1O4Zr1_ICSD_164740	insulator	insulator
Ce1O6Se2_ICSD_60778	insulator	insulator
Ce1O7P2_ICSD_160452	insulator	insulator
Ce1O8Zr3_ICSD_164738	insulator	insulator
Ce1O9P3_ICSD_417805	insulator	insulator
Ce1O9Ta3_ICSD_66281	insulator	insulator
Ce1Os1Si1_ICSD_41256	metal	metal
Ce1Os1Si3_ICSD_621741	metal	metal
Ce1Os2Si2_ICSD_621738	metal	metal
Ce1Os2_ICSD_102241	metal	metal
Ce1Os2_ICSD_621725	metal	metal
Ce1Os4P12_ICSD_621731	insulator	metal
Ce1Os4Sb12_ICSD_621737	metal	metal
Ce1P12Ru4_ICSD_621765	insulator	metal
Ce1P1Pd1_ICSD_69672	metal	metal
Ce1P1S1_ICSD_621769	insulator	insulator
Ce1P1_ICSD_52888	metal	metal
Ce1P1_ICSD_603003	metal	metal
Ce1P2Pt4_ICSD_409972	metal	metal
Ce1P2Rh2_ICSD_40764	metal	metal
Ce1P2Ru2_ICSD_602130	metal	metal
Ce1P2_ICSD_108172	metal	insulator
Ce1P2_ICSD_621753	insulator	metal
Ce1P5_ICSD_409181	metal	metal
Ce1P5_ICSD_621759	insulator	metal
Ce1P5_ICSD_99178	metal	metal
Ce1Pb1Rh1_ICSD_161337	metal	metal
Ce1Pb3_ICSD_621779	metal	metal
Ce1Pd1Sb1_ICSD_621834	metal	metal
Ce1Pd1Sb2_ICSD_658218	metal	metal
Ce1Pd1Sb3_ICSD_602477	metal	metal

Supplementary Table 256. Five-fold cross validated predictions for the metal/insulator classification (248/598).

system	calculated	predicted
Ce1Pd1Sn1_ICSD_102254	metal	metal
Ce1Pd1Sn1_ICSD_416969	metal	metal
Ce1Pd1Sn1_ICSD_416970	metal	metal
Ce1Pd1Tl1_ICSD_102255	metal	metal
Ce1Pd1Zn1_ICSD_420207	metal	metal
Ce1Pd1Zn1_ICSD_420208	metal	metal
Ce1Pd1_ICSD_621787	metal	metal
Ce1Pd1_ICSD_621821	metal	metal
Ce1Pd2Sb2_ICSD_604352	metal	metal
Ce1Pd2Si2_ICSD_621847	metal	metal
Ce1Pd2Si2_ICSD_621852	metal	metal
Ce1Pd2Sn2_ICSD_621854	metal	metal
Ce1Pd2Zn3_ICSD_419163	metal	metal
Ce1Pd3S4_ICSD_621832	metal	metal
Ce1Pd3_ICSD_621789	metal	metal
Ce1Pd5_ICSD_102247	metal	metal
Ce1Pt1Si1_ICSD_621906	metal	metal
Ce1Pt1Si2_ICSD_106420	metal	metal
Ce1Pt1Sn1_ICSD_106421	metal	metal
Ce1Pt1Sn1_ICSD_415492	metal	metal
Ce1Pt1Zn1_ICSD_418546	metal	metal
Ce1Pt1_ICSD_621865	metal	metal
Ce1Pt2Si2_ICSD_621898	metal	metal
Ce1Pt2Si2_ICSD_621903	metal	metal
Ce1Pt2Sn2_ICSD_621912	metal	metal
Ce1Pt2_ICSD_621878	metal	metal
Ce1Pt3_ICSD_102258	metal	metal
Ce1Pt5Sb1_ICSD_183604	metal	metal
Ce1Pt5_ICSD_602756	metal	metal
Ce1Pt5_ICSD_621867	metal	metal
Ce1Rb1S2_ICSD_81395	insulator	insulator
Ce1Rb1Se2_ICSD_281068	insulator	insulator
Ce1Rb1Te2_ICSD_413329	metal	insulator
Ce1Re4Si2_ICSD_27747	metal	metal
Ce1Rh1Sb1_ICSD_90870	metal	metal
Ce1Rh1Si2_ICSD_602570	metal	metal
Ce1Rh1Si3_ICSD_621955	metal	metal
Ce1Rh1Sn1_ICSD_657265	metal	metal
Ce1Rh1_ICSD_604327	metal	metal
Ce1Rh2Si1_ICSD_99133	metal	metal
Ce1Rh2Si2_ICSD_621951	metal	metal
Ce1Rh2Sn2_ICSD_621965	metal	metal
Ce1Rh2_ICSD_102268	metal	metal
Ce1Rh3_ICSD_102270	metal	metal
Ce1Rh6Si4_ICSD_247289	metal	metal
Ce1Ru1Si1_ICSD_41252	metal	metal
Ce1Ru1Si2_ICSD_656786	metal	metal
Ce1Ru1Si3_ICSD_161866	metal	metal
Ce1Ru1Si3_ICSD_621998	metal	metal
Ce1Ru1Sn3_ICSD_600556	metal	metal

Supplementary Table 257. Five-fold cross validated predictions for the metal/insulator classification (249/598).

system	calculated	predicted
Ce1Ru1Sn3_ICSD_622003	metal	metal
Ce1Ru2Si2_ICSD_657435	metal	metal
Ce1Ru2_ICSD_102276	metal	metal
Ce1Ru3Si2_ICSD_52898	metal	metal
Ce1Ru4Sb12_ICSD_621988	metal	metal
Ce1Ru4Sn6_ICSD_406894	metal	metal
Ce1S1_ICSD_183954	metal	metal
Ce1S1_ICSD_622029	metal	metal
Ce1S2_ICSD_418404	insulator	insulator
Ce1S2_ICSD_653996	insulator	insulator
Ce1S2_ICSD_656240	metal	metal
Ce1S3Sc1_ICSD_400014	insulator	insulator
Ce1S3Yb1_ICSD_622073	insulator	metal
Ce1S6Yb3_ICSD_42129	metal	metal
Ce1Sb1Te1_ICSD_601692	metal	metal
Ce1Sb1Te1_ICSD_93136	metal	metal
Ce1Sb1_ICSD_52903	metal	metal
Ce1Sb1_ICSD_622102	metal	metal
Ce1Sb2_ICSD_622083	metal	metal
Ce1Sc1Si1_ICSD_87159	metal	metal
Ce1Se1_ICSD_183955	metal	metal
Ce1Se1_ICSD_622126	metal	metal
Ce1Se2Tl1_ICSD_622131	insulator	insulator
Ce1Se2Tl1_ICSD_622133	insulator	insulator
Ce1Se2_ICSD_108265	metal	metal
Ce1Se2_ICSD_52908	metal	metal
Ce1Se2_ICSD_622120	metal	metal
Ce1Se2_ICSD_622124	metal	metal
Ce1Se3Yb1_ICSD_99666	insulator	metal
Ce1Si1_ICSD_622140	metal	metal
Ce1Si2_ICSD_622153	metal	metal
Ce1Si5_ICSD_174529	metal	metal
Ce1Sn1Zn1_ICSD_152624	metal	metal
Ce1Sn3_ICSD_622248	metal	metal
Ce1Te1_ICSD_183956	metal	metal
Ce1Te1_ICSD_622277	metal	metal
Ce1Te2Tl1_ICSD_622280	insulator	insulator
Ce1Te2_ICSD_622263	metal	metal
Ce1Tl1Zn1_ICSD_622301	metal	metal
Ce1Tl3_ICSD_102295	metal	metal
Ce1Zn11_ICSD_602894	metal	metal
Ce1Zn1_ICSD_108270	metal	metal
Ce1Zn2_ICSD_102305	metal	metal
Ce1Zn2_ICSD_622320	metal	metal
Ce1Zn3_ICSD_150633	metal	metal
Ce1Zn3_ICSD_622345	metal	metal
Ce1Zn5_ICSD_622328	metal	metal
Ce1Zn5_ICSD_622339	metal	metal
Ce1_ICSD_41824	metal	metal
Ce1_ICSD_43381	metal	metal

Supplementary Table 258. Five-fold cross validated predictions for the metal/insulator classification (250/598).

system	calculated	predicted
Ce1_ICSD_43569	metal	metal
Ce1_ICSD_52844	metal	metal
Ce1_ICSD_601481	metal	metal
Ce1_ICSD_620630	metal	metal
Ce2Cl3N1_ICSD_261615	insulator	insulator
Ce2Cl6H28O14_ICSD_280974	metal	insulator
Ce2Co1Ga9Ge2_ICSD_173618	metal	metal
Ce2Co1Si3_ICSD_83895	metal	metal
Ce2Co5Fe12N3_ICSD_603443	metal	metal
Ce2Co5Fe12_ICSD_603342	metal	metal
Ce2Cr1N3_ICSD_78826	metal	metal
Ce2Cu2Mg1_ICSD_107322	metal	metal
Ce2F4Se1_ICSD_21011	insulator	insulator
Ce2Fe17N3_ICSD_67562	metal	metal
Ce2Fe17_ICSD_106387	metal	metal
Ce2Fe17_ICSD_620990	metal	metal
Ce2Fe2O3S2_ICSD_181167	insulator	metal
Ce2Ga12Pd1_ICSD_171487	metal	metal
Ce2Ge2In1_ICSD_415108	metal	metal
Ce2Ge2Mg1_ICSD_413849	metal	metal
Ce2Ge3Li2_ICSD_63036	metal	metal
Ce2Ge3O9_ICSD_83342	insulator	insulator
Ce2Ge3Zn6_ICSD_98233	metal	metal
Ce2H1Li1O3_ICSD_56745	insulator	insulator
Ce2H5_ICSD_621314	metal	metal
Ce2I2Si2_ICSD_407246	metal	metal
Ce2In1Ni2_ICSD_150615	metal	metal
Ce2In1Pd2_ICSD_107337	metal	metal
Ce2In1Pt2_ICSD_410293	metal	metal
Ce2In1_ICSD_102186	metal	metal
Ce2Mg17Ru4_ICSD_424261	metal	metal
Ce2Mg1Ni2_ICSD_411012	metal	metal
Ce2Mg1Si2_ICSD_83915	metal	metal
Ce2Mn1N3_ICSD_50579	metal	metal
Ce2Mn2O3Se2_ICSD_181935	insulator	metal
Ce2N2Se1_ICSD_421995	insulator	insulator
Ce2Ni12P7_ICSD_621628	metal	metal
Ce2Ni2Sn1_ICSD_55495	metal	metal
Ce2Ni2Zn1_ICSD_416816	metal	metal
Ce2Ni7P4_ICSD_658790	metal	metal
Ce2Ni7_ICSD_621585	metal	metal
Ce2O12W3_ICSD_401920	metal	insulator
Ce2O2S1_ICSD_154584	insulator	insulator
Ce2O2Sb1_ICSD_9100	metal	metal
Ce2O2Te1_ICSD_9101	insulator	insulator
Ce2O3_ICSD_154587	insulator	insulator
Ce2O3_ICSD_160221	insulator	insulator
Ce2O3_ICSD_26865	insulator	insulator
Ce2O3_ICSD_96202	insulator	insulator
Ce2O4Se1Si1_ICSD_59941	insulator	insulator

Supplementary Table 259. Five-fold cross validated predictions for the metal/insulator classification (251/598).

system	calculated	predicted
Ce2O4Si1Te1_ICSD_59940	metal	insulator
Ce2O7Zr2_ICSD_168595	insulator	insulator
Ce2O9Si1Ti2_ICSD_410201	insulator	insulator
Ce2Pd21Si6_ICSD_174079	metal	metal
Ce2Pd9Sb3_ICSD_81853	metal	metal
Ce2Pt7Si4_ICSD_246168	metal	metal
Ce2Rh15Si7_ICSD_280622	metal	metal
Ce2Rh1Si3_ICSD_106425	metal	metal
Ce2Rh1Si3_ICSD_164827	metal	metal
Ce2Rh3Si5_ICSD_247297	metal	metal
Ce2Ru1Zn4_ICSD_418547	metal	metal
Ce2S3_ICSD_89499	insulator	insulator
Ce2S5Si1_ICSD_97318	insulator	insulator
Ce2S5Sn1_ICSD_622053	metal	metal
Ce2Sb1_ICSD_409587	metal	metal
Ce2Sb1_ICSD_52904	metal	metal
Ce2Si7_ICSD_174528	metal	metal
Ce2Zn17_ICSD_622318	metal	metal
Ce3Cl1S8Si2_ICSD_412222	insulator	insulator
Ce3Cl3O6Ta1_ICSD_202554	insulator	insulator
Ce3Co4Sn13_ICSD_102118	metal	metal
Ce3Co8Si1_ICSD_620784	metal	metal
Ce3Cu1Ge1S7_ICSD_620858	insulator	insulator
Ce3Cu1Ge1Se7_ICSD_620859	insulator	insulator
Ce3Cu1S7Si1_ICSD_418242	insulator	insulator
Ce3Cu1S7Sn1_ICSD_155937	insulator	insulator
Ce3Cu1Se7Si1_ICSD_156282	insulator	insulator
Ce3Cu1Se7Sn1_ICSD_152824	insulator	insulator
Ce3Cu3Sb4_ICSD_658638	metal	metal
Ce3Ga1_ICSD_102168	metal	metal
Ce3Ga1_ICSD_102169	metal	metal
Ce3Ga1_ICSD_621097	metal	metal
Ce3Ge13Ir4_ICSD_621211	metal	metal
Ce3Ge13Os4_ICSD_621237	metal	metal
Ce3Ge6Pd20_ICSD_106395	metal	metal
Ce3I1S8Si2_ICSD_86173	insulator	insulator
Ce3In1_ICSD_98500	metal	metal
Ce3Ir4Sn13_ICSD_621433	metal	metal
Ce3N11Si6_ICSD_401679	metal	insulator
Ce3N1S3_ICSD_416218	insulator	insulator
Ce3Ni6Si2_ICSD_621650	metal	metal
Ce3O16Zr5_ICSD_164739	insulator	insulator
Ce3O8Zr1_ICSD_164742	insulator	insulator
Ce3Pb1_ICSD_108259	metal	metal
Ce3Pd20Si6_ICSD_75013	metal	metal
Ce3Pd5Si1_ICSD_93244	metal	metal
Ce3Pt3Sb4_ICSD_621896	insulator	metal
Ce3Pt4_ICSD_621874	metal	metal
Ce3Rh2Si2_ICSD_247291	metal	metal
Ce3Rh4Sn13_ICSD_621967	metal	metal

Supplementary Table 260. Five-fold cross validated predictions for the metal/insulator classification (252/598).

system	calculated	predicted
Ce3Ru4Sn13_ICSD_423135	metal	metal
Ce3S4_ICSD_31602	metal	metal
Ce3S4_ICSD_622018	metal	metal
Ce3Se4_ICSD_622113	metal	metal
Ce3Si2_ICSD_622176	metal	metal
Ce3Si2_ICSD_622187	metal	metal
Ce3Sn1_ICSD_622224	metal	metal
Ce3Sn1_ICSD_622230	metal	metal
Ce3Te4_ICSD_622276	metal	metal
Ce3Tl1_ICSD_102296	metal	metal
Ce4Cl3N1S3_ICSD_420115	insulator	insulator
Ce4Cr1S7_ICSD_603696	insulator	insulator
Ce4Cu1S7_ICSD_620904	insulator	insulator
Ce4Ge3S12_ICSD_418634	metal	insulator
Ce4O4S3_ICSD_2578	insulator	insulator
Ce4O4Se3_ICSD_419127	insulator	insulator
Ce4Pd12Sn25_ICSD_189075	metal	metal
Ce4Pt12Sn25_ICSD_150777	metal	metal
Ce4Rh12Si1_ICSD_247295	metal	metal
Ce4Rh4Si3_ICSD_247292	metal	metal
Ce4Ru3_ICSD_151120	metal	metal
Ce4Sb3_ICSD_622086	metal	metal
Ce5O16Zr3_ICSD_164741	insulator	insulator
Ce5Si3_ICSD_108267	metal	metal
Ce5Si4_ICSD_165733	metal	metal
Ce5Sn3_ICSD_622219	metal	metal
Ce6Ni6P17_ICSD_2243	metal	metal
Ce6P17Pd6_ICSD_30851	metal	metal
Ce7O12_ICSD_88754	insulator	insulator
Ce7O16Zr1_ICSD_164743	insulator	insulator
Ce8Pd24Sb1_ICSD_83378	metal	metal
Cl10Co1Tb6_ICSD_424437	insulator	insulator
Cl10Cs3O1Re2_ICSD_31	insulator	insulator
Cl10Fe1W2_ICSD_422521	insulator	insulator
Cl10Gd6O12Si4_ICSD_416000	insulator	insulator
Cl10K4O1Re2_ICSD_108904	insulator	insulator
Cl10K4O1Ru2_ICSD_8054	insulator	insulator
Cl10K4O1W2_ICSD_1923	insulator	insulator
Cl10Mo2N4S4_ICSD_47102	insulator	insulator
Cl10Mo2S4Se2_ICSD_84359	insulator	insulator
Cl10Nb1P1_ICSD_26096	insulator	insulator
Cl10Nb2O1Te4_ICSD_49920	insulator	insulator
Cl10Nb2P8S20_ICSD_416095	insulator	insulator
Cl10Nb2_ICSD_16757	insulator	insulator
Cl10O12Si4Y6_ICSD_94464	insulator	insulator
Cl10O7Si6_ICSD_409628	insulator	insulator
Cl10P1Ta1_ICSD_26097	insulator	insulator
Cl10P1U1_ICSD_35464	insulator	insulator
Cl10S4Se2W2_ICSD_84360	insulator	insulator
Cl10Sc7_ICSD_1018	metal	metal

Supplementary Table 261. Five-fold cross validated predictions for the metal/insulator classification (253/598).

system	calculated	predicted
Cl10U2_ICSD_26089	insulator	insulator
Cl10W2_ICSD_2398	insulator	insulator
Cl11Cs1Hg5_ICSD_39767	insulator	insulator
Cl11Hg5Tl1_ICSD_14108	insulator	insulator
Cl11K1Nb2_ICSD_420871	insulator	insulator
Cl11N1Re2S2_ICSD_38379	insulator	insulator
Cl11N3P4_ICSD_71913	insulator	insulator
Cl12Hf1Se2_ICSD_404545	insulator	insulator
Cl12I2Nb6_ICSD_65968	insulator	insulator
Cl12Mo1Se2_ICSD_404535	insulator	insulator
Cl12Mo1Te2_ICSD_404224	insulator	insulator
Cl12N1P2Ta1_ICSD_280896	insulator	insulator
Cl12N1Sc7_ICSD_201976	metal	insulator
Cl12O2S2Ti2_ICSD_410940	insulator	insulator
Cl12O2S2Ti2_ICSD_410941	insulator	insulator
Cl12O2S2Zr2_ICSD_16128	insulator	insulator
Cl12O4P2Sb2_ICSD_49749	insulator	insulator
Cl12Os1S2_ICSD_74680	insulator	insulator
Cl12Os1Te2_ICSD_74682	insulator	insulator
Cl12Re1Te2_ICSD_68656	insulator	insulator
Cl12Re2S1_ICSD_411053	insulator	insulator
Cl12Sc7_ICSD_36424	insulator	metal
Cl12Se2Zr1_ICSD_404533	insulator	insulator
Cl12Ta2Te4_ICSD_401907	insulator	insulator
Cl12Te4W2_ICSD_66023	insulator	insulator
Cl12Te4W2_ICSD_74789	insulator	insulator
Cl12Zr6_ICSD_41539	metal	insulator
Cl13Li1Mo6_ICSD_410368	insulator	insulator
Cl14Hg1Mo6_ICSD_26046	insulator	insulator
Cl14Mo4O4Tl2_ICSD_408774	insulator	insulator
Cl14Mo6Pb1_ICSD_36572	insulator	insulator
Cl14Nb6O4Ti2_ICSD_406839	insulator	insulator
Cl14O8P4V2_ICSD_420125	insulator	insulator
Cl15Co1Zr6_ICSD_71148	insulator	metal
Cl15Ni1Zr6_ICSD_71149	metal	insulator
Cl16Ge6_ICSD_86673	insulator	insulator
Cl16Mo1Te3_ICSD_404954	insulator	insulator
Cl16O6P4Sn2_ICSD_26182	insulator	insulator
Cl16Re4S4Te4_ICSD_165333	insulator	insulator
Cl16Re4Se4Te4_ICSD_165334	insulator	insulator
Cl16Re4Te8_ICSD_165335	insulator	insulator
Cl16Ru2S6_ICSD_410112	insulator	insulator
Cl16Si6_ICSD_2767	insulator	insulator
Cl17P1W6_ICSD_422270	insulator	insulator
Cl18Cs2Pb1Ta6_ICSD_88711	insulator	insulator
Cl18Cs2W6_ICSD_109513	insulator	insulator
Cl18Cu2Nb6Rb2_ICSD_402275	insulator	insulator
Cl18In2Li2Nb6_ICSD_75071	insulator	insulator
Cl18In4Ta6_ICSD_78835	insulator	insulator
Cl18K2Mn1Nb6_ICSD_82102	insulator	insulator

Supplementary Table 262. Five-fold cross validated predictions for the metal/insulator classification (254/598).

system	calculated	predicted
Cl18K2Nb6Sr1_ICSD_280933	insulator	insulator
Cl18K2W6_ICSD_260305	insulator	insulator
Cl18K2Zr7_ICSD_15278	metal	insulator
Cl18K4Nb6_ICSD_41118	insulator	insulator
Cl18K4Ta6_ICSD_59851	insulator	insulator
Cl18Mo2P2_ICSD_410649	insulator	insulator
Cl18Nb6Rb4_ICSD_402272	insulator	insulator
Cl18Nb6Tl2V1_ICSD_96347	insulator	insulator
Cl18P2Re2_ICSD_410188	insulator	insulator
Cl18P2Sn2_ICSD_60093	insulator	insulator
Cl18P2Ti2_ICSD_23137	insulator	insulator
Cl18Rb2W6_ICSD_260304	insulator	insulator
Cl18W6_ICSD_51513	insulator	insulator
Cl1Co1O3Sr2_ICSD_91750	insulator	insulator
Cl1Cr1Cs1O3_ICSD_6076	insulator	insulator
Cl1Cr1K1O3_ICSD_26591	insulator	insulator
Cl1Cr1O1_ICSD_4086	insulator	insulator
Cl1Cr1O3Rb1_ICSD_9030	insulator	insulator
Cl1Cr3O12Sr5_ICSD_37096	insulator	insulator
Cl1Cs1F3Sb1_ICSD_200296	insulator	insulator
Cl1Cs1Mn2O7V2_ICSD_418764	insulator	insulator
Cl1Cs1N1O4Tc1_ICSD_65802	insulator	insulator
Cl1Cs1O4_ICSD_33566	insulator	insulator
Cl1Cs1O4_ICSD_63364	insulator	insulator
Cl1Cs1_ICSD_52274	insulator	insulator
Cl1Cs1_ICSD_655032	insulator	insulator
Cl1Cs3O1_ICSD_401664	insulator	insulator
Cl1Cu1F10_ICSD_35387	insulator	insulator
Cl1Cu1H1O1_ICSD_38384	metal	insulator
Cl1Cu1H1O1_ICSD_91088	insulator	insulator
Cl1Cu1H3N1_ICSD_170946	insulator	insulator
Cl1Cu1Hg1S1_ICSD_412310	insulator	insulator
Cl1Cu1Hg1Se1_ICSD_16450	insulator	insulator
Cl1Cu1K4O15V5_ICSD_401042	insulator	insulator
Cl1Cu1La1Nb2O7_ICSD_88032	insulator	metal
Cl1Cu1La1O7Ta2_ICSD_51261	insulator	insulator
Cl1Cu1Se2_ICSD_68292	insulator	insulator
Cl1Cu1Te2_ICSD_641	insulator	insulator
Cl1Cu1_ICSD_60711	insulator	insulator
Cl1Cu1_ICSD_78272	insulator	insulator
Cl1Cu1_ICSD_78273	insulator	insulator
Cl1Cu2H3O3_ICSD_51677	metal	insulator
Cl1Cu2H3O3_ICSD_64956	metal	insulator
Cl1Cu3S3Te1_ICSD_85789	insulator	insulator
Cl1Cu6In1O8_ICSD_69612	metal	insulator
Cl1Cu6O8Y1_ICSD_188351	metal	insulator
Cl1Dy1H2O2_ICSD_260833	insulator	insulator
Cl1Dy1Mo1O4_ICSD_420052	insulator	insulator
Cl1Dy1O3Se1_ICSD_418537	insulator	insulator
Cl1Dy1O3Ti1_ICSD_73096	insulator	insulator

Supplementary Table 263. Five-fold cross validated predictions for the metal/insulator classification (255/598).

system	calculated	predicted
Cl1Dy1S1_ICSD_417434	insulator	insulator
Cl1Er1H1_ICSD_203143	metal	insulator
Cl1Er1H2O2_ICSD_260835	insulator	insulator
Cl1Er1Mo1O4_ICSD_420053	insulator	insulator
Cl1Er1O3Se1_ICSD_412861	insulator	insulator
Cl1Er1S1_ICSD_21009	insulator	insulator
Cl1Eu1F1_ICSD_108938	insulator	insulator
Cl1Eu1Mo1O4_ICSD_420054	metal	insulator
Cl1Eu1O1_ICSD_54682	metal	insulator
Cl1Eu1O3Se1_ICSD_418534	metal	insulator
Cl1Eu1O3Ti1_ICSD_73095	insulator	insulator
Cl1F10Sb1_ICSD_280731	insulator	insulator
Cl1F1Na6O8S2_ICSD_29330	insulator	insulator
Cl1F1O1Pb2_ICSD_200213	insulator	insulator
Cl1F1O7Pb2Se2Ti1_ICSD_189152	insulator	insulator
Cl1F1Pb1_ICSD_155010	insulator	insulator
Cl1F1Sn1_ICSD_647	insulator	insulator
Cl1F1Sr1_ICSD_159278	insulator	insulator
Cl1F1Yb1_ICSD_35390	insulator	insulator
Cl1F1_ICSD_406442	insulator	insulator
Cl1F3K1Sb1_ICSD_20656	insulator	insulator
Cl1F3Sn2_ICSD_200032	insulator	insulator
Cl1F3_ICSD_19079	insulator	insulator
Cl1F4N1O1_ICSD_50216	insulator	insulator
Cl1F5Ge1O2_ICSD_60108	insulator	insulator
Cl1F5N1Re1_ICSD_33542	insulator	insulator
Cl1F6K3Re1_ICSD_80170	insulator	insulator
Cl1F6O2Sb1_ICSD_173609	insulator	insulator
Cl1F8Nb1_ICSD_39501	insulator	insulator
Cl1F8Sb1_ICSD_9899	insulator	insulator
Cl1F8Ta1_ICSD_39502	insulator	insulator
Cl1Fe1Li1Mo1O4_ICSD_65346	insulator	insulator
Cl1Fe1Mo1O4_ICSD_202187	insulator	insulator
Cl1Fe1N3O3_ICSD_281495	insulator	insulator
Cl1Fe1O1_ICSD_27136	metal	insulator
Cl1Fe1O3Sr2_ICSD_93508	insulator	insulator
Cl1Fe1O4W1_ICSD_80798	insulator	insulator
Cl1Fe24K6Li1S26_ICSD_10398	metal	insulator
Cl1Fe2H3O12Te4_ICSD_50706	insulator	insulator
Cl1Fe2O4P1_ICSD_171	insulator	insulator
Cl1Fe3O8Pb4_ICSD_15521	insulator	insulator
Cl1Fe3O8Pb4_ICSD_9205	insulator	insulator
Cl1Ga1Te1_ICSD_15582	insulator	insulator
Cl1Gd1H2O2_ICSD_260831	insulator	insulator
Cl1Gd1Mo1O4_ICSD_420055	insulator	insulator
Cl1Gd1O1_ICSD_59232	insulator	insulator
Cl1Gd1O3Se1_ICSD_418535	insulator	insulator
Cl1Gd1O4S1_ICSD_410323	insulator	insulator
Cl1Gd1O4W1_ICSD_35292	insulator	insulator
Cl1Gd1O5Te2_ICSD_88674	insulator	insulator

Supplementary Table 264. Five-fold cross validated predictions for the metal/insulator classification (256/598).

system	calculated	predicted
Cl1Ge1H3_ICSD_62109	insulator	insulator
Cl1H12N7P2_ICSD_405490	insulator	insulator
Cl1H13O6_ICSD_2235	insulator	insulator
Cl1H1Hg1O4_ICSD_29038	insulator	insulator
Cl1H1Hg1O4_ICSD_59874	insulator	insulator
Cl1H1O1Pb1_ICSD_28035	insulator	insulator
Cl1H1O1Pb1_ICSD_74291	insulator	insulator
Cl1H1O1Pb1_ICSD_76932	insulator	insulator
Cl1H1O1Sr1_ICSD_407719	insulator	insulator
Cl1H1O3Pb3_ICSD_94064	insulator	insulator
Cl1H1Sc1_ICSD_40981	metal	insulator
Cl1H1Sr1_ICSD_37200	insulator	insulator
Cl1H1_ICSD_28844	insulator	insulator
Cl1H1_ICSD_29079	metal	metal
Cl1H2Hg1N1_ICSD_92480	insulator	insulator
Cl1H2Ho1O2_ICSD_260834	insulator	insulator
Cl1H2Li1O1_ICSD_281198	insulator	insulator
Cl1H2Lu1O2_ICSD_260841	insulator	insulator
Cl1H2Na1O5_ICSD_173	insulator	insulator
Cl1H2Nd1O2_ICSD_20047	insulator	insulator
Cl1H2O2Tb1_ICSD_260832	insulator	insulator
Cl1H2O2Yb1_ICSD_260837	metal	insulator
Cl1H2O2Yb1_ICSD_260840	metal	metal
Cl1H2O6Rb1Se2Zn1_ICSD_409915	insulator	insulator
Cl1H3Li4O3_ICSD_74929	insulator	insulator
Cl1H3O5_ICSD_23866	insulator	insulator
Cl1H4N1O1_ICSD_14204	insulator	insulator
Cl1H4N1O1_ICSD_85479	insulator	insulator
Cl1H4N1O2_ICSD_59921	insulator	insulator
Cl1H4N1O4_ICSD_6241	insulator	insulator
Cl1H4Na1O2_ICSD_2313	insulator	insulator
Cl1H4Na1O6_ICSD_425697	insulator	insulator
Cl1H5N2O4_ICSD_9894	insulator	insulator
Cl1H5O2_ICSD_15353	insulator	insulator
Cl1H6Li1O7_ICSD_32534	insulator	insulator
Cl1H6Na1O5_ICSD_1954	insulator	insulator
Cl1Hf1N1_ICSD_261539	insulator	insulator
Cl1Hg2O12Rb3S3_ICSD_436	insulator	insulator
Cl1Hg2O12S3Tl3_ICSD_435	insulator	insulator
Cl1Hg2P3_ICSD_74771	insulator	insulator
Cl1Hg3I1S2_ICSD_250174	insulator	insulator
Cl1Hg3O4P1_ICSD_411756	insulator	insulator
Cl1Ho1Mo1O4_ICSD_420056	insulator	insulator
Cl1Ho1O3Se1_ICSD_418538	insulator	insulator
Cl1Ho1O3Te1_ICSD_95844	insulator	insulator
Cl1I1Sn1_ICSD_23262	insulator	insulator
Cl1I1_ICSD_23886	insulator	insulator
Cl1I1_ICSD_26032	insulator	insulator
Cl1In1K6Te4_ICSD_79543	insulator	insulator
Cl1In1O1_ICSD_24058	insulator	insulator

Supplementary Table 265. Five-fold cross validated predictions for the metal/insulator classification (257/598).

system	calculated	predicted
Cl1In1O3Te1_ICSD_279576	insulator	insulator
Cl1In1S4Sb2_ICSD_159468	insulator	insulator
Cl1In1Te1_ICSD_21031	insulator	insulator
Cl1In1_ICSD_2429	insulator	insulator
Cl1In1_ICSD_2431	metal	insulator
Cl1In1_ICSD_2432	insulator	insulator
Cl1In1_ICSD_425449	insulator	insulator
Cl1In5S5_ICSD_414221	insulator	insulator
Cl1In5Se5_ICSD_414220	insulator	insulator
Cl1K1Mo1O5P1_ICSD_50988	insulator	insulator
Cl1K1O2_ICSD_59935	insulator	insulator
Cl1K1O3_ICSD_16690	insulator	insulator
Cl1K1O3_ICSD_26685	insulator	insulator
Cl1K1O3_ICSD_9483	insulator	insulator
Cl1K1O4_ICSD_33562	insulator	insulator
Cl1K1O4_ICSD_413444	insulator	insulator
Cl1K1_ICSD_240512	insulator	insulator
Cl1K1_ICSD_290514	insulator	insulator
Cl1K2Na1O6S2_ICSD_24677	insulator	insulator
Cl1K3O1S3W1_ICSD_10318	insulator	insulator
Cl1La1Nb2O6_ICSD_35428	insulator	insulator
Cl1La1O1_ICSD_24611	insulator	insulator
Cl1La1O4S1_ICSD_408944	insulator	insulator
Cl1La1Se1_ICSD_188001	insulator	insulator
Cl1La1Te1_ICSD_426279	insulator	insulator
Cl1La1_ICSD_24410	metal	metal
Cl1La3O8Si2_ICSD_65023	insulator	insulator
Cl1La3S8Si2_ICSD_412221	insulator	insulator
Cl1La5Sn3_ICSD_95238	metal	metal
Cl1Li1O2_ICSD_59936	insulator	insulator
Cl1Li1O4_ICSD_165579	insulator	insulator
Cl1Li1_ICSD_52235	insulator	insulator
Cl1Li4N1_ICSD_84649	insulator	insulator
Cl1Li6O5P1_ICSD_421479	insulator	insulator
Cl1Lu1O3Se1_ICSD_241239	insulator	insulator
Cl1Mg2O4P1_ICSD_2844	insulator	insulator
Cl1Mn1O3Sr2_ICSD_96044	metal	insulator
Cl1Mn1O3_ICSD_416749	insulator	insulator
Cl1Mn1S2Sb1_ICSD_151925	insulator	insulator
Cl1Mn2O4P1_ICSD_2813	insulator	insulator
Cl1Mo1O4Tb1_ICSD_420059	insulator	insulator
Cl1Mo1O4Tm1_ICSD_420060	insulator	insulator
Cl1Mo1O4Y1_ICSD_249647	insulator	insulator
Cl1Mo1O4Yb1_ICSD_420061	metal	insulator
Cl1Mo1O5P1Rb1_ICSD_50989	insulator	insulator
Cl1N1O1_ICSD_411511	insulator	insulator
Cl1N1O4S1_ICSD_27059	insulator	insulator
Cl1N1O6_ICSD_25817	insulator	insulator
Cl1N1Sr2_ICSD_410769	insulator	insulator
Cl1N1Ti1_ICSD_27396	insulator	insulator

Supplementary Table 266. Five-fold cross validated predictions for the metal/insulator classification (258/598).

system	calculated	predicted
Cl1N1Zn2_ICSD_425734	insulator	insulator
Cl1N1Zr1_ICSD_25506	insulator	insulator
Cl1N1Zr1_ICSD_93740	insulator	insulator
Cl1N3O2S3_ICSD_36378	insulator	insulator
Cl1N3_ICSD_424502	insulator	insulator
Cl1N5S4_ICSD_14085	insulator	insulator
Cl1N5S5_ICSD_62528	insulator	insulator
Cl1Na1O3_ICSD_26684	insulator	insulator
Cl1Na1O4_ICSD_172567	insulator	insulator
Cl1Na1O4_ICSD_33567	insulator	insulator
Cl1Na1O4_ICSD_51730	insulator	insulator
Cl1Na1_ICSD_100633	insulator	insulator
Cl1Na1_ICSD_43434	insulator	insulator
Cl1Na3O1_ICSD_67319	insulator	insulator
Cl1Nb1O8Pb2Se2_ICSD_189153	insulator	insulator
Cl1Nd1O5Te2_ICSD_88673	insulator	insulator
Cl1Nd1Te1_ICSD_426777	insulator	insulator
Cl1Nd3O8Si2_ICSD_92490	insulator	insulator
Cl1O12P3Pb5_ICSD_24238	insulator	insulator
Cl1O12P3Sr5_ICSD_2089	insulator	insulator
Cl1O12Sr5V3_ICSD_171381	insulator	insulator
Cl1O1Ti1_ICSD_39314	insulator	insulator
Cl1O1V1_ICSD_27011	insulator	insulator
Cl1O1Y1_ICSD_31667	insulator	insulator
Cl1O1Y1_ICSD_60585	insulator	insulator
Cl1O1Yb1_ICSD_6077	insulator	insulator
Cl1O2Pb1Sb1_ICSD_86229	insulator	insulator
Cl1O2Rb1_ICSD_162802	insulator	insulator
Cl1O2_ICSD_67665	insulator	insulator
Cl1O3Pb1V1_ICSD_420548	insulator	insulator
Cl1O3Rb1_ICSD_10283	insulator	insulator
Cl1O3Sb1Te1_ICSD_86095	insulator	insulator
Cl1O3Se1Tb1_ICSD_418536	insulator	insulator
Cl1O3Se1Yb1_ICSD_418541	metal	insulator
Cl1O3Sr1V1_ICSD_50787	insulator	insulator
Cl1O3Tl1_ICSD_76434	insulator	insulator
Cl1O4P1Sn2_ICSD_16519	insulator	insulator
Cl1O4Pb1Re1_ICSD_171147	insulator	insulator
Cl1O4Rb1S1Zn1_ICSD_386	insulator	insulator
Cl1O4Rb1_ICSD_33565	insulator	insulator
Cl1O4Rb1_ICSD_51728	insulator	insulator
Cl1O4S1Tl1Zn1_ICSD_387	insulator	insulator
Cl1O4Sb3_ICSD_410039	insulator	insulator
Cl1O4Tl1_ICSD_33564	insulator	insulator
Cl1O9Re1S2_ICSD_419178	insulator	insulator
Cl1Rb1_ICSD_18016	insulator	insulator
Cl1Rb1_ICSD_61521	insulator	insulator
Cl1Rh1Te1_ICSD_56853	insulator	insulator
Cl1Sc1_ICSD_1004	metal	metal
Cl1Tb1_ICSD_23352	metal	metal

Supplementary Table 267. Five-fold cross validated predictions for the metal/insulator classification (259/598).

system	calculated	predicted
Cl1Tl1_ICSD_109143	insulator	insulator
Cl1Tl1_ICSD_29107	insulator	insulator
Cl1Tl1_ICSD_61518	insulator	insulator
Cl1Y1_ICSD_61107	metal	metal
Cl1Zr1_ICSD_20145	metal	metal
Cl1Zr1_ICSD_868	metal	metal
Cl2Co1H12O6_ICSD_34429	insulator	insulator
Cl2Co1H4O2_ICSD_34651	insulator	insulator
Cl2Co1O2Sr2_ICSD_59697	metal	metal
Cl2Co1O6Se2Sr2_ICSD_419615	metal	insulator
Cl2Co1O8_ICSD_33288	insulator	insulator
Cl2Co1_ICSD_15939	insulator	insulator
Cl2Co2O3Te1_ICSD_415798	insulator	insulator
Cl2Co5O12Se4_ICSD_416965	insulator	insulator
Cl2Cr1H12I1N4_ICSD_48186	insulator	insulator
Cl2Cr1O2_ICSD_416750	insulator	insulator
Cl2Cr1_ICSD_27490	insulator	insulator
Cl2Cr3F6K2_ICSD_60897	insulator	insulator
Cl2Cs1Cu2I1_ICSD_60960	insulator	insulator
Cl2Cs1I1_ICSD_14260	insulator	insulator
Cl2Cs1K1O8_ICSD_22504	insulator	insulator
Cl2Cs1Li1_ICSD_35397	insulator	insulator
Cl2Cs1Li1_ICSD_423634	insulator	insulator
Cl2Cu1H4O2_ICSD_15087	insulator	insulator
Cl2Cu1H4O4Pb2_ICSD_81589	insulator	insulator
Cl2Cu1H6N2_ICSD_180189	insulator	insulator
Cl2Cu1K2O4S1_ICSD_22364	insulator	insulator
Cl2Cu1O2Sr2_ICSD_67067	metal	metal
Cl2Cu1O6Se2Sr2_ICSD_174067	insulator	insulator
Cl2Cu1_ICSD_66645	insulator	insulator
Cl2Cu2O1_ICSD_96610	insulator	insulator
Cl2Cu2O5Te2_ICSD_89978	insulator	insulator
Cl2Cu2O6Pb1Se2_ICSD_189154	insulator	insulator
Cl2Cu2O6Pb1Te2_ICSD_189155	insulator	insulator
Cl2Cu2O6Se2Sr1_ICSD_174068	insulator	insulator
Cl2Cu2O6Sr1Te2_ICSD_416653	insulator	insulator
Cl2Cu3H6Mg1O6_ICSD_240663	insulator	insulator
Cl2Cu3H6Ni1O6_ICSD_415857	metal	insulator
Cl2Cu3H6O6Zn1_ICSD_425834	insulator	metal
Cl2Cu3O4Sr2_ICSD_427	metal	metal
Cl2Cu3O6Se2_ICSD_240496	insulator	insulator
Cl2Cu3O6Se2_ICSD_92165	insulator	insulator
Cl2Cu5O8Se2_ICSD_8163	insulator	insulator
Cl2F12Pb7_ICSD_10402	insulator	insulator
Cl2F1Tb1_ICSD_418279	insulator	insulator
Cl2F2I2O2_ICSD_411237	insulator	insulator
Cl2F2N3O2P1S2_ICSD_2352	insulator	insulator
Cl2F6I1Sb1_ICSD_15526	insulator	insulator
Cl2Fe1H4O2_ICSD_15597	insulator	insulator
Cl2Fe1H8O4_ICSD_9488	insulator	insulator

Supplementary Table 268. Five-fold cross validated predictions for the metal/insulator classification (260/598).

system	calculated	predicted
Cl2Fe1_ICSD_64830	metal	insulator
Cl2Fe1_ICSD_64831	insulator	insulator
Cl2Fe5O18Te6_ICSD_245637	insulator	insulator
Cl2Ga6Li8O24Si6_ICSD_87987	insulator	insulator
Cl2Ga6Na8O24Si6_ICSD_417705	insulator	insulator
Cl2Ge3La3_ICSD_414173	metal	metal
Cl2H12Mg1O6_ICSD_34694	insulator	insulator
Cl2H12N4O8Zn1_ICSD_74790	insulator	insulator
Cl2H12Ni1O6_ICSD_22284	insulator	insulator
Cl2H12O6Sr1_ICSD_48110	insulator	insulator
Cl2H12Sr7_ICSD_418948	insulator	insulator
Cl2H1K3O6Pt1S2_ICSD_16439	insulator	insulator
Cl2H1N1O4S2_ICSD_165233	insulator	insulator
Cl2H2Mg1O1_ICSD_172993	insulator	insulator
Cl2H2Mo1O3_ICSD_25031	insulator	insulator
Cl2H2O1Sr1_ICSD_60883	insulator	insulator
Cl2H4Mg1O10_ICSD_261132	insulator	insulator
Cl2H4Mg1O2_ICSD_172992	insulator	insulator
Cl2H4Mn1O2_ICSD_15596	insulator	insulator
Cl2H4O6Zn1_ICSD_8231	insulator	insulator
Cl2H6N2_ICSD_23145	insulator	insulator
Cl2H6N2_ICSD_240903	insulator	insulator
Cl2H8Mg1O12_ICSD_261131	insulator	insulator
Cl2H8Mg1O12_ICSD_262271	insulator	insulator
Cl2H8Mn1N4_ICSD_25784	insulator	insulator
Cl2H8N2O2Pt1_ICSD_35759	insulator	insulator
Cl2H8N2O2Pt1_ICSD_35760	insulator	insulator
Cl2H8N4Zn1_ICSD_15875	insulator	insulator
Cl2Hg1O2Pb2_ICSD_74973	insulator	insulator
Cl2Hg1_ICSD_23277	insulator	insulator
Cl2Hg2O6_ICSD_412963	metal	insulator
Cl2Hg2_ICSD_31173	insulator	insulator
Cl2Hg3O2_ICSD_28399	insulator	insulator
Cl2Hg3S2_ICSD_27399	insulator	insulator
Cl2Hg3Se2_ICSD_27400	insulator	insulator
Cl2Hg3Te2_ICSD_27401	insulator	insulator
Cl2Hg4O2_ICSD_16662	insulator	insulator
Cl2Hg5O4_ICSD_24593	metal	insulator
Cl2I1N3S4_ICSD_412782	insulator	insulator
Cl2I2Ta1_ICSD_69688	insulator	insulator
Cl2K2N2O4Pd1_ICSD_39851	metal	insulator
Cl2K5Na1O12S4_ICSD_24676	insulator	insulator
Cl2La3Si3_ICSD_408031	metal	metal
Cl2Li5N1_ICSD_84763	insulator	insulator
Cl2Mg1O8_ICSD_261619	insulator	insulator
Cl2Mg1_ICSD_17063	insulator	insulator
Cl2Mg1_ICSD_51244	insulator	insulator
Cl2Mg1_ICSD_51245	insulator	insulator
Cl2Mg1_ICSD_51246	insulator	insulator
Cl2Mg1_ICSD_51247	insulator	insulator

Supplementary Table 269. Five-fold cross validated predictions for the metal/insulator classification (261/598).

system	calculated	predicted
Cl2Mg1_ICSD_56147	insulator	insulator
Cl2Mn1_ICSD_33752	insulator	insulator
Cl2N1Sc2_ICSD_59125	metal	insulator
Cl2N2O4Pd1Rb2_ICSD_93914	insulator	insulator
Cl2N2S2Te1_ICSD_170631	insulator	insulator
Cl2N2S3_ICSD_23974	insulator	insulator
Cl2N3S2V1_ICSD_30742	insulator	insulator
Cl2N4O12S10_ICSD_72781	insulator	insulator
Cl2N4S4_ICSD_26373	insulator	insulator
Cl2Nb1S2_ICSD_10484	insulator	insulator
Cl2Nb1Se2_ICSD_10483	insulator	insulator
Cl2Nd2O7Ta2_ICSD_108807	insulator	insulator
Cl2Ni1O8_ICSD_33289	insulator	insulator
Cl2Ni1_ICSD_14208	insulator	insulator
Cl2Ni2O8Sr1Te3_ICSD_418683	insulator	insulator
Cl2Ni7O18Te6_ICSD_240326	insulator	insulator
Cl2O1Os1_ICSD_83884	metal	metal
Cl2O1Pd2_ICSD_61333	insulator	insulator
Cl2O1Ru1_ICSD_83883	metal	insulator
Cl2O1S1_ICSD_62971	insulator	insulator
Cl2O1V1_ICSD_24380	insulator	insulator
Cl2O1_ICSD_407768	insulator	insulator
Cl2O2Pb3_ICSD_23521	insulator	insulator
Cl2O2U1_ICSD_36056	insulator	insulator
Cl2O4Pb1_ICSD_68484	insulator	insulator
Cl2O4Pb4_ICSD_30292	metal	insulator
Cl2O4Sr1_ICSD_171021	insulator	insulator
Cl2O5Sb4_ICSD_2233	insulator	insulator
Cl2O6Pb1_ICSD_40286	insulator	insulator
Cl2O6Pb2Pd1Se2_ICSD_423573	insulator	insulator
Cl2O6Pb2Pd1Te2_ICSD_423574	insulator	insulator
Cl2O6Pb3Te2_ICSD_281171	insulator	insulator
Cl2O6Sr1_ICSD_61157	insulator	insulator
Cl2O6_ICSD_62063	insulator	insulator
Cl2O7Pr2Ta2_ICSD_108806	insulator	insulator
Cl2O7_ICSD_63680	insulator	insulator
Cl2Pb1_ICSD_15806	metal	insulator
Cl2Pb1_ICSD_43344	insulator	insulator
Cl2Pb1_ICSD_81978	insulator	insulator
Cl2Pd1Se6_ICSD_405207	insulator	insulator
Cl2Pd1_ICSD_404624	insulator	insulator
Cl2Pd1_ICSD_421219	insulator	insulator
Cl2Pd1_ICSD_421220	insulator	insulator
Cl2Pd1_ICSD_421221	insulator	insulator
Cl2Pt1_ICSD_28527	insulator	insulator
Cl2Re6S8_ICSD_67658	insulator	insulator
Cl2Re6Se8_ICSD_35623	insulator	insulator
Cl2Ru1Te8_ICSD_422366	insulator	insulator
Cl2S1_ICSD_38351	insulator	insulator
Cl2S2_ICSD_37016	metal	insulator

Supplementary Table 270. Five-fold cross validated predictions for the metal/insulator classification (262/598).

system	calculated	predicted
Cl2Se2_ICSD_37018	insulator	insulator
Cl2Si1_ICSD_85526	insulator	insulator
Cl2Sn1_ICSD_15452	insulator	insulator
Cl2Sn1_ICSD_81977	insulator	insulator
Cl2Sn1_ICSD_81979	metal	insulator
Cl2Sr1_ICSD_18011	insulator	insulator
Cl2Te3_ICSD_105	metal	insulator
Cl2Ti1_ICSD_23177	insulator	insulator
Cl2Ti1_ICSD_38219	insulator	insulator
Cl2Tm1_ICSD_447	insulator	insulator
Cl2V1_ICSD_15901	insulator	insulator
Cl2V1_ICSD_246905	insulator	insulator
Cl2Yb1_ICSD_6054	insulator	insulator
Cl2Zn1_ICSD_1692	insulator	insulator
Cl2Zn1_ICSD_26152	insulator	insulator
Cl2Zn1_ICSD_26154	insulator	insulator
Cl2Zr1_ICSD_20144	insulator	insulator
Cl2_ICSD_201696	insulator	insulator
Cl2_ICSD_22406	insulator	insulator
Cl3Co1Cs1_ICSD_27511	insulator	insulator
Cl3Co1Rb1_ICSD_24305	insulator	insulator
Cl3Co1Tl1_ICSD_155189	insulator	insulator
Cl3Co1Tl1_ICSD_155191	insulator	insulator
Cl3Cr1Cs1_ICSD_36132	metal	insulator
Cl3Cr1Cs1_ICSD_41802	insulator	insulator
Cl3Cr1Rb1_ICSD_2225	insulator	insulator
Cl3Cr1Rb1_ICSD_56837	insulator	insulator
Cl3Cr1Rb1_ICSD_8184	insulator	insulator
Cl3Cr1_ICSD_22080	insulator	insulator
Cl3Cr1_ICSD_22081	insulator	insulator
Cl3Cr1_ICSD_33578	insulator	insulator
Cl3Cs1Cu1_ICSD_26666	insulator	insulator
Cl3Cs1Cu2_ICSD_14201	insulator	insulator
Cl3Cs1Fe1_ICSD_300249	insulator	insulator
Cl3Cs1Ge1_ICSD_23121	insulator	insulator
Cl3Cs1Ge1_ICSD_75215	insulator	insulator
Cl3Cs1Hg1_ICSD_24482	metal	insulator
Cl3Cs1Li2_ICSD_245974	insulator	insulator
Cl3Cs1Li2_ICSD_423635	insulator	insulator
Cl3Cs1Mg1_ICSD_54167	insulator	insulator
Cl3Cs1Mn1_ICSD_2555	insulator	insulator
Cl3Cs1Ni1_ICSD_60262	insulator	insulator
Cl3Cs1Pb1_ICSD_109294	insulator	insulator
Cl3Cs1Pb1_ICSD_201250	insulator	insulator
Cl3Cs1Sc1_ICSD_10474	metal	insulator
Cl3Cs1Sn1_ICSD_14199	insulator	insulator
Cl3Cs1Sn1_ICSD_28082	insulator	insulator
Cl3Cs1V1_ICSD_201832	insulator	insulator
Cl3Cs1Yb1_ICSD_31461	insulator	insulator
Cl3Cu1K1_ICSD_15590	insulator	insulator

Supplementary Table 271. Five-fold cross validated predictions for the metal/insulator classification (263/598).

system	calculated	predicted
Cl3Cu1K2_ICSD_150292	insulator	insulator
Cl3Cu1K2_ICSD_30656	metal	insulator
Cl3Cu1Rb1_ICSD_15523	insulator	insulator
Cl3Cu1Rb1_ICSD_40944	insulator	insulator
Cl3Cu1Rb1_ICSD_56838	insulator	insulator
Cl3Cu1Rb1_ICSD_84212	metal	insulator
Cl3Cu1Rb2_ICSD_150294	insulator	insulator
Cl3Dy1_ICSD_40064	insulator	insulator
Cl3Er1H2O13_ICSD_410764	insulator	insulator
Cl3Eu1_ICSD_23148	metal	insulator
Cl3F2Rb2Sb1_ICSD_200497	insulator	insulator
Cl3F2Sb1_ICSD_200039	insulator	insulator
Cl3F2Sb1_ICSD_380014	insulator	insulator
Cl3F3N3P3_ICSD_16098	insulator	insulator
Cl3Fe1K1_ICSD_10134	insulator	insulator
Cl3Fe1Mn7O10_ICSD_69062	insulator	insulator
Cl3Fe1_ICSD_27500	insulator	insulator
Cl3Fe1_ICSD_39765	insulator	insulator
Cl3Ga1_ICSD_30336	insulator	insulator
Cl3Ga1_ICSD_413455	insulator	insulator
Cl3Gd1_ICSD_22270	insulator	insulator
Cl3Gd2N1_ICSD_47224	insulator	insulator
Cl3Gd2_ICSD_9580	insulator	insulator
Cl3Ge1Rb1_ICSD_12149	insulator	insulator
Cl3H12N5O1Os1_ICSD_39306	insulator	insulator
Cl3H12O6V1_ICSD_418528	insulator	insulator
Cl3H1La3O6Ta1_ICSD_62189	insulator	insulator
Cl3H4K1Mn1O2_ICSD_14170	insulator	insulator
Cl3H6La1O9_ICSD_29439	insulator	insulator
Cl3Hg1Na1_ICSD_38302	insulator	insulator
Cl3I1N1V1_ICSD_10470	insulator	insulator
Cl3Ir1Te6_ICSD_423672	insulator	insulator
Cl3Ir1_ICSD_23171	insulator	insulator
Cl3Ir1_ICSD_25716	metal	metal
Cl3K1Mn1_ICSD_32710	insulator	insulator
Cl3K2N3O5Os1_ICSD_39424	insulator	insulator
Cl3K2N3O5Ru1_ICSD_97229	insulator	insulator
Cl3K2N3O6Pt1_ICSD_20709	insulator	insulator
Cl3La1_ICSD_31574	insulator	insulator
Cl3La2N1_ICSD_165789	insulator	insulator
Cl3La3O6U1_ICSD_73201	insulator	insulator
Cl3La3O6W1_ICSD_35595	insulator	insulator
Cl3La3O7Si2_ICSD_82386	insulator	insulator
Cl3La4N1S3_ICSD_74902	insulator	insulator
Cl3Mg1Rb1_ICSD_15279	insulator	insulator
Cl3Mg1Rb1_ICSD_4036	insulator	insulator
Cl3Mn1Na1_ICSD_2552	insulator	insulator
Cl3Mn1Rb1_ICSD_36125	insulator	insulator
Cl3Mn1Tl1_ICSD_23167	insulator	insulator
Cl3Mn1Tl1_ICSD_32712	insulator	insulator

Supplementary Table 272. Five-fold cross validated predictions for the metal/insulator classification (264/598).

system	calculated	predicted
Cl3Mo1N1_ICSD_15117	insulator	insulator
Cl3Mo1O1_ICSD_4411	insulator	insulator
Cl3Mo1S2_ICSD_28062	insulator	insulator
Cl3Mo1_ICSD_26109	insulator	insulator
Cl3Mo1_ICSD_83878	insulator	insulator
Cl3N1Nd2_ICSD_79944	insulator	insulator
Cl3N1Nd4S3_ICSD_92443	insulator	insulator
Cl3N1Pr2_ICSD_79943	insulator	insulator
Cl3N1Se2_ICSD_84030	insulator	insulator
Cl3N1W1_ICSD_165376	insulator	insulator
Cl3N1Y2_ICSD_65829	insulator	insulator
Cl3N1_ICSD_4034	insulator	insulator
Cl3N3S3_ICSD_30558	insulator	insulator
Cl3N3Ti1_ICSD_15996	insulator	insulator
Cl3Nb1O1_ICSD_26471	insulator	insulator
Cl3Nb1O1_ICSD_412071	insulator	insulator
Cl3Nd1_ICSD_31577	insulator	insulator
Cl3Nd3O6U1_ICSD_73203	insulator	insulator
Cl3Ni1Rb1_ICSD_15010	insulator	insulator
Cl3O10Se2Tb5_ICSD_154235	insulator	insulator
Cl3O12Yb1_ICSD_85762	metal	insulator
Cl3O1P1_ICSD_9128	insulator	insulator
Cl3O1V1_ICSD_250365	insulator	insulator
Cl3O2Re1_ICSD_416056	insulator	insulator
Cl3O6Pr3U1_ICSD_73202	insulator	insulator
Cl3O6Pr3W1_ICSD_20626	insulator	insulator
Cl3P1_ICSD_32027	insulator	insulator
Cl3Pr1_ICSD_202925	insulator	insulator
Cl3Pr1_ICSD_65079	insulator	insulator
Cl3Pr3Ru1_ICSD_173604	metal	metal
Cl3Pu1_ICSD_31572	metal	metal
Cl3Pu1_ICSD_38195	metal	metal
Cl3Rb1Ti1_ICSD_49747	insulator	insulator
Cl3Rb1V1_ICSD_201830	insulator	insulator
Cl3Rh1_ICSD_25764	insulator	insulator
Cl3Ru1_ICSD_20717	insulator	insulator
Cl3Ru1_ICSD_22092	metal	insulator
Cl3Ru1_ICSD_414041	insulator	insulator
Cl3S8Sb1_ICSD_35741	insulator	insulator
Cl3Sb1_ICSD_8258	insulator	insulator
Cl3Sc1_ICSD_74517	insulator	insulator
Cl3Tb1_ICSD_23938	insulator	insulator
Cl3Tb1_ICSD_63541	insulator	insulator
Cl3Tb1_ICSD_63543	insulator	insulator
Cl3Tc1_ICSD_261105	insulator	insulator
Cl3Tc1_ICSD_262639	metal	insulator
Cl3Ti1_ICSD_26069	metal	insulator
Cl3Ti1_ICSD_26070	insulator	insulator
Cl3Ti1_ICSD_29035	insulator	insulator
Cl3Ti1_ICSD_39426	insulator	insulator

Supplementary Table 273. Five-fold cross validated predictions for the metal/insulator classification (265/598).

system	calculated	predicted
Cl3Ti1_ICSD_39427	insulator	insulator
Cl3Ti1_ICSD_39429	insulator	insulator
Cl3Ti1_ICSD_39430	insulator	insulator
Cl3Tm1_ICSD_35398	metal	insulator
Cl3U1_ICSD_2353	insulator	insulator
Cl3V1_ICSD_38237	insulator	insulator
Cl3Y1_ICSD_15684	insulator	insulator
Cl3Y2_ICSD_23337	insulator	insulator
Cl3Zr1_ICSD_35315	metal	metal
Cl3Zr1_ICSD_43292	metal	insulator
Cl4Co1K2_ICSD_661	insulator	insulator
Cl4Co1Li2_ICSD_73227	insulator	insulator
Cl4Co1Rb2_ICSD_87515	insulator	insulator
Cl4Co1Rb2_ICSD_87517	insulator	insulator
Cl4Cr1Cs2_ICSD_41571	insulator	insulator
Cl4Cr1Li2_ICSD_202627	insulator	insulator
Cl4Cr1Li2_ICSD_403035	insulator	insulator
Cl4Cr1Na2_ICSD_66201	insulator	insulator
Cl4Cr1Rb2_ICSD_41570	insulator	insulator
Cl4Cr1Rb2_ICSD_47129	metal	insulator
Cl4Cr1Rb2_ICSD_9858	insulator	insulator
Cl4Cs1Ga1_ICSD_201670	insulator	insulator
Cl4Cs2Hg1_ICSD_39568	insulator	insulator
Cl4Cs2I2Pd1_ICSD_240484	insulator	insulator
Cl4Cs2Mg1_ICSD_9005	insulator	insulator
Cl4Cs2O2U1_ICSD_56859	insulator	insulator
Cl4Cs2O2U1_ICSD_66542	insulator	insulator
Cl4Cs2Pd1_ICSD_95812	insulator	insulator
Cl4Cs2Yb1_ICSD_49623	insulator	insulator
Cl4Cs3Li1_ICSD_245969	insulator	insulator
Cl4Cs6O1_ICSD_411634	insulator	insulator
Cl4Cu1Ga1_ICSD_300103	insulator	insulator
Cl4Cu1H12N2O2_ICSD_23750	metal	metal
Cl4Cu1H4K2O2_ICSD_16052	insulator	insulator
Cl4Cu1H4O2Rb2_ICSD_71867	insulator	insulator
Cl4Cu1Rb2_ICSD_15145	insulator	insulator
Cl4Cu3O12Te4Yb2_ICSD_419113	metal	insulator
Cl4Cu4S4W1_ICSD_171240	metal	insulator
Cl4Er1Na1_ICSD_82364	insulator	insulator
Cl4F1Sb1_ICSD_74783	insulator	insulator
Cl4F1Ta1_ICSD_27413	insulator	insulator
Cl4F5N1Te1W1_ICSD_37149	insulator	insulator
Cl4F6Ir1_ICSD_411351	metal	insulator
Cl4F6P1Sb1_ICSD_71876	insulator	insulator
Cl4Fe1K1_ICSD_63469	metal	insulator
Cl4Fe1Na1_ICSD_16994	insulator	insulator
Cl4Fe1Rb1_ICSD_63470	insulator	insulator
Cl4Ga1K1_ICSD_281297	insulator	insulator
Cl4Ga1Li1_ICSD_60849	insulator	insulator
Cl4Ga1Rb1_ICSD_409650	insulator	insulator

Supplementary Table 274. Five-fold cross validated predictions for the metal/insulator classification (266/598).

system	calculated	predicted
Cl4Ga1Tl1_ICSD_419825	insulator	insulator
Cl4Ga2H2_ICSD_165558	insulator	insulator
Cl4Ga2_ICSD_14218	insulator	insulator
Cl4Gd1Li1_ICSD_38326	insulator	insulator
Cl4Gd1Na1_ICSD_33784	insulator	insulator
Cl4Gd2S1_ICSD_56742	insulator	insulator
Cl4Ge1_ICSD_280880	insulator	insulator
Cl4H10O5Sn1_ICSD_413631	insulator	insulator
Cl4H18K1N6O8Ru1_ICSD_280489	insulator	insulator
Cl4H18N6O8Rb1Ru1_ICSD_280492	metal	insulator
Cl4H26O14Sc2_ICSD_39838	insulator	insulator
Cl4H2Hg1K2O1_ICSD_30211	insulator	insulator
Cl4H4N1Tl1_ICSD_14020	insulator	insulator
Cl4H8Si5_ICSD_262931	insulator	insulator
Cl4Hf1_ICSD_402054	insulator	insulator
Cl4Hg2K2O3S1_ICSD_419256	insulator	insulator
Cl4Hg3O1_ICSD_35649	insulator	insulator
Cl4Hg3S2Zn1_ICSD_420783	insulator	insulator
Cl4Hg4I2S1_ICSD_422546	insulator	insulator
Cl4I1K1_ICSD_30203	insulator	insulator
Cl4I2O6Pb3_ICSD_247144	insulator	insulator
Cl4K1Tl1_ICSD_14105	insulator	insulator
Cl4K2Mg1_ICSD_4035	insulator	insulator
Cl4K2O2Os1_ICSD_36231	insulator	insulator
Cl4K2Pd1_ICSD_2723	insulator	insulator
Cl4K2Pt1_ICSD_68768	insulator	insulator
Cl4K2Zn1_ICSD_68824	insulator	insulator
Cl4K2Zn1_ICSD_80861	insulator	insulator
Cl4Li2Zn1_ICSD_202743	insulator	insulator
Cl4Li2Zn1_ICSD_73222	insulator	insulator
Cl4Lu1Na1_ICSD_78994	insulator	insulator
Cl4Mg1Na2_ICSD_69343	insulator	insulator
Cl4Mn1Na2_ICSD_9136	insulator	insulator
Cl4Mn1Rb2_ICSD_1139	insulator	insulator
Cl4Mo1O1_ICSD_41418	insulator	insulator
Cl4N1Re1_ICSD_419181	insulator	insulator
Cl4N1V1_ICSD_28128	insulator	insulator
Cl4N4S4Te1_ICSD_416253	insulator	insulator
Cl4Na1Sc1_ICSD_402273	insulator	insulator
Cl4Na2Pd1_ICSD_202971	insulator	insulator
Cl4Na2Ti1_ICSD_400264	insulator	insulator
Cl4Na2Zn1_ICSD_402063	insulator	insulator
Cl4O1Os1_ICSD_417247	insulator	insulator
Cl4O1Rb6_ICSD_405193	insulator	insulator
Cl4O1W1_ICSD_426524	insulator	insulator
Cl4Os1Sc4_ICSD_421532	insulator	insulator
Cl4Os1_ICSD_1165	insulator	insulator
Cl4Pa1_ICSD_9309	metal	insulator
Cl4Pd1Tl2_ICSD_89601	insulator	insulator
Cl4Pt1_ICSD_22073	insulator	insulator

Supplementary Table 275. Five-fold cross validated predictions for the metal/insulator classification (267/598).

system	calculated	predicted
Cl4Rb2Zn1_ICSD_35657	insulator	insulator
Cl4Rb2Zn1_ICSD_63188	insulator	insulator
Cl4Rb2Zn1_ICSD_68569	insulator	insulator
Cl4Re1_ICSD_10293	metal	insulator
Cl4Re6Se8Tl2_ICSD_162300	insulator	insulator
Cl4S1Sm2_ICSD_89534	insulator	insulator
Cl4S1Tl6_ICSD_35289	insulator	insulator
Cl4S1W1_ICSD_16125	insulator	insulator
Cl4Se1_ICSD_15578	insulator	insulator
Cl4Si1_ICSD_62279	insulator	insulator
Cl4Sn1_ICSD_411242	insulator	insulator
Cl4Sr1Zn1_ICSD_410192	insulator	insulator
Cl4Ta1_ICSD_402406	insulator	insulator
Cl4Tc1_ICSD_26055	insulator	insulator
Cl4Th1_ICSD_26197	insulator	insulator
Cl4Th1_ICSD_6055	insulator	insulator
Cl4Ti1_ICSD_280981	insulator	insulator
Cl4Tl2_ICSD_4031	insulator	insulator
Cl4U1_ICSD_202331	insulator	metal
Cl4V1_ICSD_250364	insulator	insulator
Cl4W1_ICSD_165263	metal	insulator
Cl4Zr1_ICSD_26049	insulator	insulator
Cl5Co1Cs3_ICSD_14087	insulator	insulator
Cl5Co1Cs3_ICSD_15245	insulator	insulator
Cl5Co1Tl3_ICSD_10137	insulator	insulator
Cl5Cs1Pb2_ICSD_249888	insulator	insulator
Cl5Cs1Pd2_ICSD_411722	insulator	insulator
Cl5Cs1Sn2_ICSD_93791	insulator	insulator
Cl5Cs3Hg1_ICSD_630	insulator	insulator
Cl5Cs3Li2_ICSD_245971	insulator	insulator
Cl5Cs3Zn1_ICSD_240876	insulator	insulator
Cl5Dy2Li1_ICSD_35763	metal	insulator
Cl5Fe1N4S4_ICSD_47103	insulator	insulator
Cl5Fe1Tl3_ICSD_10138	insulator	insulator
Cl5K1Pb2_ICSD_416431	insulator	insulator
Cl5K1Sn2_ICSD_152003	insulator	insulator
Cl5K2Mo1O1_ICSD_28215	insulator	insulator
Cl5K2N1O1Ru1_ICSD_20713	insulator	insulator
Cl5K2N1Os1_ICSD_27560	insulator	insulator
Cl5K2N1Os1_ICSD_43729	insulator	insulator
Cl5K2Sb1_ICSD_6059	insulator	insulator
Cl5K2U1_ICSD_41551	insulator	insulator
Cl5Li1Tm2_ICSD_201996	insulator	insulator
Cl5Li1Yb2_ICSD_77273	insulator	insulator
Cl5Mo1_ICSD_26500	insulator	insulator
Cl5Mo1_ICSD_84620	insulator	insulator
Cl5N3O1P2S1_ICSD_43	insulator	insulator
Cl5Nb1_ICSD_66537	insulator	insulator
Cl5Nb3O2_ICSD_91781	insulator	insulator
Cl5O2U2_ICSD_23084	metal	insulator

Supplementary Table 276. Five-fold cross validated predictions for the metal/insulator classification (268/598).

system	calculated	predicted
Cl5O4Re2_ICSD_12111	insulator	insulator
Cl5O4Re2_ICSD_416429	insulator	insulator
Cl5P1_ICSD_26661	insulator	insulator
Cl5P1_ICSD_76731	metal	insulator
Cl5P4S4Ta1_ICSD_419157	insulator	insulator
Cl5Pa1_ICSD_15391	insulator	insulator
Cl5Pb1Tl3_ICSD_1262	insulator	insulator
Cl5Pb2Tl1_ICSD_417	insulator	insulator
Cl5Sb1_ICSD_250363	insulator	insulator
Cl6Co1Fe1H18N6_ICSD_32718	metal	insulator
Cl6Cr1Na3_ICSD_62035	insulator	insulator
Cl6Cs1Sb1_ICSD_155691	insulator	insulator
Cl6Cs1W1_ICSD_1242	metal	insulator
Cl6Cs2Er1Na1_ICSD_50361	insulator	insulator
Cl6Cs2Ge1_ICSD_28892	insulator	insulator
Cl6Cs2Hg1Pd1_ICSD_36616	insulator	insulator
Cl6Cs2Ho1Na1_ICSD_245366	insulator	insulator
Cl6Cs2In1Li1_ICSD_65735	insulator	insulator
Cl6Cs2Ir1_ICSD_69142	metal	insulator
Cl6Cs2Li1Sc1_ICSD_65734	insulator	insulator
Cl6Cs2Li1Y1_ICSD_65731	insulator	insulator
Cl6Cs2Mo1_ICSD_409808	insulator	insulator
Cl6Cs2Na1Tb1_ICSD_96062	insulator	insulator
Cl6Cs2Pb1_ICSD_26713	insulator	insulator
Cl6Cs2Pb1_ICSD_29033	insulator	insulator
Cl6Cs2Pt1_ICSD_26709	insulator	insulator
Cl6Cs2Pt1_ICSD_29032	insulator	insulator
Cl6Cs2Re1_ICSD_64613	insulator	insulator
Cl6Cs2Se1_ICSD_26693	insulator	insulator
Cl6Cs2Sn1_ICSD_26699	insulator	insulator
Cl6Cs2Ta1_ICSD_240711	metal	insulator
Cl6Cs2Te1_ICSD_26704	insulator	insulator
Cl6Cs2Th1_ICSD_16658	insulator	insulator
Cl6Cs2Ti1_ICSD_26690	insulator	insulator
Cl6Cs2U1_ICSD_202332	insulator	insulator
Cl6Cs2Zr1_ICSD_26695	insulator	insulator
Cl6Cs4Pb1_ICSD_35703	insulator	insulator
Cl6Er1Na3_ICSD_65145	insulator	insulator
Cl6Eu4O1_ICSD_65171	insulator	insulator
Cl6Fe1H12O6Pt1_ICSD_163595	insulator	insulator
Cl6Fe1Hf1_ICSD_39817	insulator	insulator
Cl6Fe1K3Na1_ICSD_170747	insulator	insulator
Cl6Fe1K3Na1_ICSD_23182	metal	insulator
Cl6Fe1Zr1_ICSD_39666	insulator	insulator
Cl6Ga1N1Se2_ICSD_71903	insulator	insulator
Cl6Ga1Sb1_ICSD_24786	insulator	insulator
Cl6Gd3N1_ICSD_63582	insulator	insulator
Cl6H16N5O3Rh1_ICSD_411835	insulator	insulator
Cl6H18In2N6_ICSD_281343	insulator	insulator
Cl6H22O10Sn1_ICSD_27180	insulator	insulator

Supplementary Table 277. Five-fold cross validated predictions for the metal/insulator classification (269/598).

system	calculated	predicted
Cl6H4N1Sb1_ICSD_412875	insulator	insulator
Cl6H4N1W1_ICSD_412876	insulator	insulator
Cl6H5O2Sb1_ICSD_280168	insulator	insulator
Cl6H8N2Pt1_ICSD_97927	insulator	insulator
Cl6H8N2Re1_ICSD_8044	insulator	insulator
Cl6H8N2Sn1_ICSD_605	insulator	insulator
Cl6Hf1Hg3Se2_ICSD_412466	insulator	insulator
Cl6Hf1Te4_ICSD_401589	insulator	insulator
Cl6Hf1Te8_ICSD_410306	insulator	insulator
Cl6Hg3S2Zr1_ICSD_412469	insulator	insulator
Cl6Hg3Se2Zr1_ICSD_412468	insulator	insulator
Cl6Hg3Te2U1_ICSD_419437	insulator	insulator
Cl6Hg6P4Sn1_ICSD_411863	insulator	insulator
Cl6I1P1_ICSD_26594	insulator	insulator
Cl6I1Se6W1_ICSD_78789	insulator	insulator
Cl6I2_ICSD_24714	insulator	insulator
Cl6In1Na3_ICSD_154092	insulator	insulator
Cl6K2Mn1_ICSD_9679	insulator	insulator
Cl6K2Mo1_ICSD_26643	metal	insulator
Cl6K2Nb1_ICSD_245749	metal	insulator
Cl6K2Os1_ICSD_68764	metal	metal
Cl6K2Pd1_ICSD_65037	insulator	insulator
Cl6K2Pt1_ICSD_52033	insulator	insulator
Cl6K2Pu1_ICSD_202526	insulator	insulator
Cl6K2Re1_ICSD_23769	insulator	insulator
Cl6K2Ru1_ICSD_76275	metal	insulator
Cl6K2Sn1_ICSD_1669	insulator	insulator
Cl6K2Sn1_ICSD_1670	insulator	insulator
Cl6K2Sn1_ICSD_6058	insulator	insulator
Cl6K2Ta1_ICSD_59894	metal	metal
Cl6K2Tc1_ICSD_22096	insulator	metal
Cl6K2Tc2_ICSD_63512	insulator	insulator
Cl6K2Ti1_ICSD_26934	insulator	insulator
Cl6K2W1_ICSD_409840	metal	metal
Cl6K3Mn1Na1_ICSD_290109	insulator	insulator
Cl6K4Mn1_ICSD_24475	insulator	insulator
Cl6Mn1Rb2_ICSD_9347	insulator	insulator
Cl6Mo1Tl2_ICSD_408777	insulator	insulator
Cl6N1S2Sb1_ICSD_2441	insulator	insulator
Cl6N1Sc4_ICSD_201978	metal	metal
Cl6N2O2V1_ICSD_413012	insulator	insulator
Cl6N3P3_ICSD_16136	insulator	insulator
Cl6N6S4W2_ICSD_405886	insulator	insulator
Cl6Na1Nb1_ICSD_36518	insulator	insulator
Cl6Na1Sb1_ICSD_36517	insulator	insulator
Cl6Na3Y1_ICSD_59886	insulator	insulator
Cl6Nb1Rb2_ICSD_245747	metal	insulator
Cl6Nb1Rb2_ICSD_245748	insulator	insulator
Cl6O1S8Ti4_ICSD_64624	insulator	insulator
Cl6O1Sr4_ICSD_402497	insulator	insulator

Supplementary Table 278. Five-fold cross validated predictions for the metal/insulator classification (270/598).

system	calculated	predicted
Cl6O1Yb4_ICSD_202169	insulator	insulator
Cl6O3Si3_ICSD_71444	insulator	insulator
Cl6Pb1Rb2_ICSD_26712	insulator	insulator
Cl6Pd1Rb2_ICSD_33710	insulator	insulator
Cl6Pd1S2_ICSD_39434	insulator	insulator
Cl6Pt1Rb2_ICSD_26708	insulator	insulator
Cl6Pt1S2_ICSD_66013	insulator	insulator
Cl6Pt1Tl2_ICSD_26710	insulator	insulator
Cl6Rb2Se1_ICSD_26692	insulator	insulator
Cl6Rb2Sn1_ICSD_9022	insulator	insulator
Cl6Rb2Te1_ICSD_26703	insulator	insulator
Cl6Rb2Ti1_ICSD_26689	insulator	insulator
Cl6Rb2W1_ICSD_409632	metal	metal
Cl6Rb2Zr1_ICSD_26694	insulator	insulator
Cl6Re1_ICSD_425145	metal	insulator
Cl6Re6Te16_ICSD_405813	insulator	insulator
Cl6S8W1_ICSD_65685	insulator	insulator
Cl6Se4Zr1_ICSD_412285	insulator	insulator
Cl6Sn1Tl2_ICSD_26700	insulator	insulator
Cl6Te1Tl2_ICSD_26705	insulator	insulator
Cl6Tl2W1_ICSD_409229	metal	insulator
Cl6W1_ICSD_425147	insulator	insulator
Cl6W1_ICSD_425148	insulator	insulator
Cl7Fe1Se1_ICSD_39529	insulator	insulator
Cl7Fe1Te1_ICSD_39530	insulator	insulator
Cl7Ga2In1_ICSD_418803	insulator	insulator
Cl7Ga3_ICSD_67279	insulator	insulator
Cl7Hg6P4Ti1_ICSD_411415	insulator	insulator
Cl7I1S1_ICSD_200374	insulator	insulator
Cl7In1Y2_ICSD_201429	insulator	insulator
Cl7K1Mo2O2_ICSD_410954	insulator	insulator
Cl7Mo1O1Se1_ICSD_410101	insulator	insulator
Cl7Mo2O2Tl1_ICSD_408775	insulator	insulator
Cl7N2Sb1Se3_ICSD_406352	insulator	insulator
Cl7Nb3Se5_ICSD_10066	insulator	insulator
Cl7Nb3Te1_ICSD_79213	insulator	insulator
Cl8Co1Li6_ICSD_202401	insulator	insulator
Cl8Cr1Li5_ICSD_67743	insulator	insulator
Cl8Cu1Ga2_ICSD_201414	metal	insulator
Cl8Fe1Li6_ICSD_73217	insulator	insulator
Cl8Fe1P1_ICSD_22232	insulator	insulator
Cl8Ga2Ni1_ICSD_417870	insulator	insulator
Cl8Hg2Pd1Rb2_ICSD_203187	insulator	insulator
Cl8Hg2Pd1Tl2_ICSD_203186	insulator	insulator
Cl8I1P1_ICSD_60420	insulator	insulator
Cl8I1Sb1_ICSD_26509	insulator	insulator
Cl8I3Sb1_ICSD_26402	insulator	insulator
Cl8Ir1K3Sn1_ICSD_69143	insulator	insulator
Cl8Li6V1_ICSD_100834	insulator	insulator
Cl8Mn1Na6_ICSD_1845	insulator	insulator

Supplementary Table 279. Five-fold cross validated predictions for the metal/insulator classification (271/598).

system	calculated	predicted
Cl8Mn3Na2_ICSD_1846	insulator	insulator
Cl8N1Sc5_ICSD_60856	metal	metal
Cl8N4P4_ICSD_23897	insulator	insulator
Cl8N4P4_ICSD_33711	insulator	insulator
Cl8N6Sb2_ICSD_10353	insulator	insulator
Cl8Na2Ti3_ICSD_401026	insulator	insulator
Cl8Nb3_ICSD_408645	insulator	insulator
Cl8O12Si8_ICSD_79706	insulator	insulator
Cl8O2Se2Sn1_ICSD_35477	insulator	insulator
Cl8O2Se4Te3W2_ICSD_380129	insulator	insulator
Cl8O4Si4_ICSD_71445	insulator	insulator
Cl8O9Re4_ICSD_10302	insulator	insulator
Cl8Pt1S2_ICSD_66012	insulator	insulator
Cl8Sc5_ICSD_36403	metal	metal
Cl9Cr2Cs3_ICSD_94384	insulator	insulator
Cl9Cs1Mn4_ICSD_34750	insulator	insulator
Cl9Cs3Fe2_ICSD_22074	insulator	insulator
Cl9Cs3In2_ICSD_2491	insulator	insulator
Cl9Cs3Ru2_ICSD_201057	insulator	insulator
Cl9Cs3Sb2_ICSD_26039	insulator	insulator
Cl9Cs3Sc2_ICSD_24215	insulator	insulator
Cl9Cs3Ti2_ICSD_402407	insulator	insulator
Cl9Cs3Tl2_ICSD_26657	insulator	insulator
Cl9Cs3V2_ICSD_61234	insulator	insulator
Cl9Cs3W2_ICSD_202296	insulator	insulator
Cl9Cs3Y2_ICSD_409540	insulator	insulator
Cl9In5_ICSD_37217	insulator	insulator
Cl9In7_ICSD_71062	insulator	insulator
Cl9K3Mo2_ICSD_202298	insulator	insulator
Cl9K3W2_ICSD_16760	insulator	insulator
Cl9Mo2Rb3_ICSD_202292	insulator	insulator
Cl9Nb1Te1_ICSD_410946	insulator	insulator
Cl9Nb2S4Tl5_ICSD_417942	insulator	insulator
Cl9P1Sn1_ICSD_60094	insulator	insulator
Cl9P1Te1_ICSD_21060	insulator	insulator
Cl9P1V1_ICSD_1047	insulator	insulator
Cl9Re3_ICSD_14209	insulator	insulator
Cl9S1Sb1_ICSD_59130	insulator	insulator
Cl9S1U1_ICSD_37116	insulator	insulator
Cl9Ta1Te1_ICSD_410947	insulator	insulator
Cl9Te1W1_ICSD_410945	insulator	insulator
Co12Dy2P7_ICSD_622698	metal	metal
Co12Er2P7_ICSD_622843	metal	metal
Co12Ho2P7_ICSD_84086	metal	metal
Co12Mg2P7_ICSD_94409	metal	metal
Co12Mn5Y2_ICSD_624190	metal	metal
Co12Nb2P7_ICSD_87195	metal	metal
Co12Nd2P7_ICSD_624414	metal	metal
Co12P7Sc2_ICSD_624619	metal	metal
Co12P7Tb2_ICSD_624642	metal	metal

Supplementary Table 280. Five-fold cross validated predictions for the metal/insulator classification (272/598).

system	calculated	predicted
Co12P7Ti2_ICSD_624647	metal	metal
Co12P7U2_ICSD_602730	metal	metal
Co12P7Yb2_ICSD_97184	metal	metal
Co12P7Zr2_ICSD_624676	metal	metal
Co13La1_ICSD_623975	metal	metal
Co15Ga2Nd2_ICSD_623133	metal	metal
Co16Ge7Hf6_ICSD_109129	metal	metal
Co16Ge7Nb6_ICSD_109128	metal	metal
Co16Ge7Ta6_ICSD_109130	metal	metal
Co16Ge7Zr6_ICSD_109131	metal	metal
Co16Hf6Si7_ICSD_109120	metal	metal
Co16Nb6Si7_ICSD_53047	metal	metal
Co16Nb6Si7_ICSD_624311	metal	metal
Co16Sc6Si7_ICSD_624967	metal	metal
Co16Si7Ta6_ICSD_109121	metal	metal
Co16Si7Ti6_ICSD_625090	metal	metal
Co16Si7Zr6_ICSD_625143	metal	metal
Co17Dy2_ICSD_108276	metal	metal
Co17Dy2_ICSD_602645	metal	metal
Co17Dy2_ICSD_622609	metal	metal
Co17Dy2_ICSD_658887	metal	metal
Co17Er2_ICSD_622718	metal	metal
Co17Ho2_ICSD_55101	metal	metal
Co17Ho2_ICSD_623812	metal	metal
Co17Nd2_ICSD_102561	metal	metal
Co17Tb2_ICSD_625360	metal	metal
Co17Th2_ICSD_23225	metal	metal
Co17Th2_ICSD_625454	metal	metal
Co17Th2_ICSD_625458	metal	metal
Co17Y2_ICSD_108324	metal	metal
Co17Y2_ICSD_625556	metal	metal
Co17Yb2_ICSD_625653	metal	metal
Co1Cr1Ge1_ICSD_409451	metal	metal
Co1Cr1O4_ICSD_23492	insulator	insulator
Co1Cr1Pt2_ICSD_102321	metal	metal
Co1Cr1Si1_ICSD_165248	metal	metal
Co1Cr1_ICSD_187979	metal	metal
Co1Cr2O4_ICSD_61612	metal	metal
Co1Cr2S4_ICSD_43039	metal	metal
Co1Cr2S4_ICSD_622501	metal	metal
Co1Cr2Se4_ICSD_601038	insulator	metal
Co1Cr4H4K2O16_ICSD_154325	insulator	insulator
Co1Cs1F3_ICSD_15091	insulator	insulator
Co1Cs1K2O2_ICSD_74889	insulator	insulator
Co1Cs2F6K1_ICSD_6037	metal	insulator
Co1Cs2I4_ICSD_87897	insulator	insulator
Co1Cs2O3_ICSD_6154	insulator	insulator
Co1Cs2O4Si1_ICSD_93878	insulator	insulator
Co1Cs2S2_ICSD_67389	insulator	insulator
Co1Cu12La1_ICSD_55509	metal	metal

Supplementary Table 281. Five-fold cross validated predictions for the metal/insulator classification (273/598).

system	calculated	predicted
Co1Cu1O2_ICSD_27803	insulator	metal
Co1Cu1O7P2_ICSD_75379	insulator	insulator
Co1Cu2Ge1S4_ICSD_622541	insulator	insulator
Co1Cu2O3_ICSD_33996	metal	metal
Co1Cu2S4Si1_ICSD_99292	insulator	insulator
Co1Cu2S4Sn1_ICSD_99294	insulator	insulator
Co1Cu2Se4Sn1_ICSD_99296	metal	metal
Co1Cu2Sn1_ICSD_151207	metal	metal
Co1Dy1Ga1_ICSD_163702	metal	metal
Co1Dy1Ga5_ICSD_622659	metal	metal
Co1Dy1Ge1_ICSD_601865	metal	metal
Co1Dy1Ge2_ICSD_622671	metal	metal
Co1Dy1In5_ICSD_622689	metal	metal
Co1Dy1Si1_ICSD_88272	insulator	metal
Co1Dy1Si2_ICSD_622703	metal	metal
Co1Dy1Sn1_ICSD_106459	metal	insulator
Co1Dy1Sn1_ICSD_54584	insulator	insulator
Co1Dy1Sn2_ICSD_240097	metal	metal
Co1Dy2Ga8_ICSD_622660	metal	metal
Co1Dy2In8_ICSD_622690	metal	metal
Co1Dy2Si2_ICSD_99213	metal	metal
Co1Dy3_ICSD_106901	metal	metal
Co1Dy6Te2_ICSD_247432	metal	metal
Co1Dy8Ga3_ICSD_622663	metal	metal
Co1Dy8In3_ICSD_189215	metal	metal
Co1Er1Ga1_ICSD_102373	metal	metal
Co1Er1Ga5_ICSD_622798	metal	metal
Co1Er1Ge1_ICSD_601878	metal	metal
Co1Er1Ge2_ICSD_622811	metal	metal
Co1Er1In5_ICSD_153908	metal	metal
Co1Er1Si1_ICSD_601847	metal	metal
Co1Er1Si2_ICSD_73620	metal	metal
Co1Er1Sn1_ICSD_106463	insulator	metal
Co1Er1Sn2_ICSD_240099	metal	metal
Co1Er2Ga8_ICSD_169772	metal	metal
Co1Er2Ge2_ICSD_622815	metal	metal
Co1Er2In8_ICSD_622827	metal	metal
Co1Er2Si2_ICSD_99214	metal	metal
Co1Er3Si3_ICSD_152534	metal	metal
Co1Er3_ICSD_622736	metal	metal
Co1Er6Te2_ICSD_247434	metal	metal
Co1Er7I12_ICSD_424430	insulator	metal
Co1Er8Ga3_ICSD_622802	metal	metal
Co1Er8In3_ICSD_290605	metal	metal
Co1Eu1Ge3_ICSD_52951	metal	metal
Co1F11Na1Zr2_ICSD_81221	insulator	insulator
Co1F12O4S2Sb2_ICSD_411789	insulator	insulator
Co1F1H4Na1O6S1_ICSD_169111	metal	insulator
Co1F1Li1O4S1_ICSD_167202	metal	insulator
Co1F1Na1O4S1_ICSD_290052	insulator	insulator

Supplementary Table 282. Five-fold cross validated predictions for the metal/insulator classification (274/598).

system	calculated	predicted
Co1F2_ICSD_26604	insulator	insulator
Co1F3K1_ICSD_15425	metal	insulator
Co1F3Na1_ICSD_4404	insulator	insulator
Co1F3_ICSD_16672	insulator	metal
Co1F3_ICSD_29133	metal	insulator
Co1F3_ICSD_77618	metal	insulator
Co1F4K2_ICSD_33522	insulator	insulator
Co1F4Li1_ICSD_202919	insulator	insulator
Co1F4Rb2_ICSD_69683	insulator	insulator
Co1F6H12O6Si1_ICSD_2900	insulator	insulator
Co1F6H12O6Sn1_ICSD_75984	insulator	insulator
Co1F6H12O6Sn2_ICSD_27799	insulator	insulator
Co1F6K1Rb2_ICSD_42147	insulator	insulator
Co1F6Li1Sr1_ICSD_41177	insulator	insulator
Co1F6Na1Rb2_ICSD_42148	insulator	insulator
Co1F6Pt1_ICSD_37447	insulator	insulator
Co1F6Rb2_ICSD_9701	insulator	insulator
Co1F6Sn1_ICSD_25014	insulator	insulator
Co1F6Zr1_ICSD_83724	insulator	metal
Co1Fe15_ICSD_155846	metal	metal
Co1Fe1Ga1Mn1_ICSD_186830	metal	metal
Co1Fe1Ge1Mn1_ICSD_186831	metal	metal
Co1Fe1La3S7_ICSD_603954	insulator	insulator
Co1Fe1P1_ICSD_622955	metal	metal
Co1Fe1Si1_ICSD_165250	metal	metal
Co1Fe1_ICSD_187982	metal	metal
Co1Fe2Ga1_ICSD_102385	metal	metal
Co1Fe2Ge1_ICSD_52954	metal	metal
Co1Fe2O4_ICSD_166200	metal	metal
Co1Fe2O4_ICSD_184063	metal	metal
Co1Fe2Se4_ICSD_622979	metal	metal
Co1Fe3_ICSD_155843	metal	metal
Co1Fe7_ICSD_155845	metal	metal
Co1Ga12U4_ICSD_156307	metal	metal
Co1Ga1Hf1_ICSD_623085	metal	metal
Co1Ga1Ho1_ICSD_623088	metal	metal
Co1Ga1Mn1V1_ICSD_183629	metal	metal
Co1Ga1Mn2_ICSD_160704	metal	metal
Co1Ga1Ti2_ICSD_185663	metal	metal
Co1Ga1U1_ICSD_164348	metal	metal
Co1Ga1U1_ICSD_623224	metal	metal
Co1Ga1Zr1_ICSD_623254	metal	metal
Co1Ga1_ICSD_657494	metal	metal
Co1Ga2Gd1_ICSD_102429	metal	metal
Co1Ga2Hf6_ICSD_623077	metal	metal
Co1Ga2Mn1_ICSD_102437	metal	metal
Co1Ga2S4_ICSD_623159	insulator	insulator
Co1Ga2Zr6_ICSD_20876	metal	metal
Co1Ga3Ho8_ICSD_623092	metal	metal
Co1Ga3Tb8_ICSD_623199	metal	metal

Supplementary Table 283. Five-fold cross validated predictions for the metal/insulator classification (275/598).

system	calculated	predicted
Co1Ga3_ICSD_102425	metal	metal
Co1Ga4Hf1_ICSD_623081	metal	metal
Co1Ga4Th1_ICSD_55670	metal	metal
Co1Ga5Gd1_ICSD_623064	metal	metal
Co1Ga5Ho1_ICSD_655672	metal	metal
Co1Ga5Lu1_ICSD_623110	metal	metal
Co1Ga5Tb1_ICSD_623195	metal	metal
Co1Ga5Tm1_ICSD_623215	metal	metal
Co1Ga5U1_ICSD_106469	metal	metal
Co1Ga5Y1_ICSD_623236	metal	metal
Co1Ga5Yb1_ICSD_162956	metal	metal
Co1Ga8Ho2_ICSD_42426	metal	metal
Co1Ga8Lu2_ICSD_623111	metal	metal
Co1Ga8Tb2_ICSD_623196	metal	metal
Co1Ga8Tm2_ICSD_169773	metal	metal
Co1Ga8Y2_ICSD_623237	metal	metal
Co1Gd1Ge2_ICSD_623352	metal	metal
Co1Gd1O3_ICSD_45153	insulator	metal
Co1Gd1Si2_ICSD_623396	metal	metal
Co1Gd1Si3_ICSD_658380	metal	metal
Co1Gd7I12_ICSD_245279	metal	insulator
Co1Ge1Hf1_ICSD_623439	metal	metal
Co1Ge1Ho1_ICSD_601867	metal	metal
Co1Ge1La1_ICSD_85860	metal	metal
Co1Ge1Mg1_ICSD_418853	metal	metal
Co1Ge1Mn1_ICSD_52968	metal	metal
Co1Ge1Mn1_ICSD_623495	metal	metal
Co1Ge1Mn2_ICSD_160707	metal	metal
Co1Ge1Na2O4_ICSD_23425	insulator	insulator
Co1Ge1Nb1_ICSD_623540	metal	metal
Co1Ge1Nd1_ICSD_85861	metal	metal
Co1Ge1O3_ICSD_26814	insulator	insulator
Co1Ge1Pr1_ICSD_623566	metal	metal
Co1Ge1Sc1_ICSD_623584	metal	metal
Co1Ge1Ta1_ICSD_623611	metal	metal
Co1Ge1Tb1_ICSD_52986	metal	metal
Co1Ge1Te1_ICSD_160511	insulator	metal
Co1Ge1Ti1_ICSD_42943	metal	metal
Co1Ge1Ti1_ICSD_623629	metal	metal
Co1Ge1Ti2_ICSD_184992	metal	metal
Co1Ge1V1_ICSD_623660	metal	metal
Co1Ge1Y1_ICSD_623669	metal	metal
Co1Ge1Zr1_ICSD_623685	metal	metal
Co1Ge1_ICSD_43677	metal	metal
Co1Ge1_ICSD_623422	metal	metal
Co1Ge1_ICSD_623423	metal	metal
Co1Ge2Ho1_ICSD_623450	metal	metal
Co1Ge2Ho2_ICSD_623457	metal	metal
Co1Ge2La1_ICSD_623464	metal	metal
Co1Ge2Nd1_ICSD_623543	metal	metal

Supplementary Table 284. Five-fold cross validated predictions for the metal/insulator classification (276/598).

system	calculated	predicted
Co1Ge2O7Sr2_ICSD_262399	insulator	insulator
Co1Ge2Pr1_ICSD_623562	metal	metal
Co1Ge2Tb1_ICSD_623619	metal	metal
Co1Ge2Tb2_ICSD_52987	metal	metal
Co1Ge2Y1_ICSD_623665	metal	metal
Co1Ge2Yb1_ICSD_623674	metal	metal
Co1Ge2_ICSD_52964	metal	metal
Co1Ge3La1_ICSD_42373	metal	metal
Co1Ge3Nd1_ICSD_50359	metal	metal
Co1Ge3Nd1_ICSD_623542	metal	metal
Co1Ge3Pr1_ICSD_161875	metal	metal
Co1H10O16P4_ICSD_31310	metal	insulator
Co1H1O2_ICSD_22285	insulator	insulator
Co1H1O2_ICSD_56288	insulator	insulator
Co1H1O5P1Zn1_ICSD_416823	insulator	insulator
Co1H2Mo1O7Se1_ICSD_249955	insulator	insulator
Co1H2O2_ICSD_88940	insulator	insulator
Co1H2O5S1_ICSD_71346	insulator	insulator
Co1H2O5Se1_ICSD_66747	insulator	insulator
Co1H4K2O10Se2_ICSD_98686	insulator	insulator
Co1H4Th1_ICSD_261442	metal	metal
Co1H6K2O8P2_ICSD_59884	insulator	insulator
Co1H6N1O5P1_ICSD_280044	insulator	insulator
Co1H8I2O10_ICSD_408060	insulator	insulator
Co1H8O12Re2_ICSD_402964	metal	insulator
Co1H8O14P2V2_ICSD_281336	insulator	insulator
Co1H8O16P4Tl2_ICSD_416650	metal	insulator
Co1Hf1P1_ICSD_623786	metal	metal
Co1Hf1Sb1_ICSD_108294	metal	metal
Co1Hf1Si1_ICSD_623795	metal	metal
Co1Hf1Sn1_ICSD_107472	metal	metal
Co1Hf1Sn1_ICSD_623801	metal	metal
Co1Hf1_ICSD_623772	metal	metal
Co1Hf2P1_ICSD_84827	metal	metal
Co1Hf2_ICSD_623763	metal	metal
Co1Hf4P1_ICSD_623784	metal	metal
Co1Hf9Mo4_ICSD_623782	metal	metal
Co1Hf9Re4_ICSD_623788	metal	metal
Co1Hf9W4_ICSD_623810	metal	metal
Co1Ho1In5_ICSD_102492	metal	metal
Co1Ho1Si1_ICSD_107115	metal	metal
Co1Ho1Si2_ICSD_67010	metal	metal
Co1Ho1Sn1_ICSD_106978	metal	insulator
Co1Ho1Sn1_ICSD_108173	insulator	metal
Co1Ho2In8_ICSD_623870	metal	metal
Co1Ho3_ICSD_102484	metal	metal
Co1Ho6Te2_ICSD_247433	metal	metal
Co1Ho8In3_ICSD_189216	metal	metal
Co1I10Y6_ICSD_424841	insulator	insulator
Co1I12Sc7_ICSD_63303	insulator	insulator

Supplementary Table 285. Five-fold cross validated predictions for the metal/insulator classification (277/598).

system	calculated	predicted
Co1I12Tb7_ICSD_424442	insulator	metal
Co1I2_ICSD_52368	insulator	insulator
Co1I4Rb2_ICSD_1029	insulator	insulator
Co1In1Mn2_ICSD_160705	metal	metal
Co1In1Ti2_ICSD_185664	metal	metal
Co1In2S4_ICSD_623937	insulator	insulator
Co1In2Se4_ICSD_658918	insulator	insulator
Co1In3_ICSD_102500	metal	metal
Co1In3_ICSD_623918	metal	metal
Co1In5La1_ICSD_150264	metal	metal
Co1In5Nd1_ICSD_623923	metal	metal
Co1In5Pr1_ICSD_246914	metal	metal
Co1In5Tb1_ICSD_623944	metal	metal
Co1In5Tm1_ICSD_106969	metal	metal
Co1In5Y1_ICSD_623947	metal	metal
Co1In5Yb1_ICSD_163767	metal	metal
Co1In5Yb1_ICSD_412910	metal	metal
Co1In8Pr2_ICSD_623926	metal	metal
Co1In8Tm2_ICSD_106970	metal	metal
Co1In8Y2_ICSD_623948	metal	metal
Co1K1O2_ICSD_15770	insulator	insulator
Co1K1O2_ICSD_4199	insulator	insulator
Co1K2N6O12Pb1_ICSD_23115	metal	metal
Co1K2O12P4_ICSD_2069	insulator	insulator
Co1K2O2_ICSD_74940	insulator	insulator
Co1K2O6Se2_ICSD_71536	metal	insulator
Co1K2S2_ICSD_623958	insulator	insulator
Co1K2Se2_ICSD_67390	insulator	insulator
Co1K3N6O12_ICSD_26746	insulator	metal
Co1K3O2_ICSD_73212	insulator	insulator
Co1K6S4_ICSD_68603	insulator	insulator
Co1K6Se4_ICSD_68605	insulator	insulator
Co1La1O1P1_ICSD_80203	metal	metal
Co1La1O3_ICSD_161383	metal	metal
Co1La1O3_ICSD_189375	metal	metal
Co1La1O3_ICSD_28921	metal	metal
Co1La1Sb2_ICSD_657919	metal	metal
Co1La1Sb2_ICSD_657990	metal	metal
Co1La1Sb3_ICSD_419540	metal	metal
Co1La1Si1_ICSD_658265	metal	metal
Co1La1Si3_ICSD_624028	metal	metal
Co1La3O6S3W1_ICSD_380405	insulator	insulator
Co1La3_ICSD_150531	metal	metal
Co1La4Mg1_ICSD_416990	metal	metal
Co1La4S7_ICSD_624013	insulator	insulator
Co1Li1N2Sr2_ICSD_72387	insulator	insulator
Co1Li1O2_ICSD_161384	insulator	insulator
Co1Li1O2_ICSD_180989	metal	insulator
Co1Li1O2_ICSD_182442	insulator	metal
Co1Li1O4P1_ICSD_247497	insulator	insulator

Supplementary Table 286. Five-fold cross validated predictions for the metal/insulator classification (278/598).

system	calculated	predicted
Co1Li2O8W2_ICSD_92852	insulator	insulator
Co1Li6O4_ICSD_62688	insulator	insulator
Co1Li8O6_ICSD_21026	insulator	insulator
Co1Mg1_ICSD_106482	metal	metal
Co1Mn1O3_ICSD_31854	insulator	insulator
Co1Mn1P1_ICSD_16483	metal	metal
Co1Mn1P1_ICSD_41556	metal	metal
Co1Mn1Sb1_ICSD_157563	metal	metal
Co1Mn1Sb1_ICSD_624133	metal	metal
Co1Mn1Si1_ICSD_41917	metal	metal
Co1Mn1Si1_ICSD_53006	metal	metal
Co1Mn1_ICSD_187981	metal	metal
Co1Mn2Sb1_ICSD_160709	metal	metal
Co1Mn2Si1_ICSD_160706	metal	metal
Co1Mn2Sn1_ICSD_160708	metal	metal
Co1Mn2Sn1_ICSD_183043	metal	metal
Co1Mo1O4_ICSD_23808	insulator	insulator
Co1Mo1O4_ICSD_281235	insulator	insulator
Co1Mo1O6Sr2_ICSD_153543	metal	metal
Co1Mo1O6Sr2_ICSD_153544	insulator	insulator
Co1Mo1O6Te1_ICSD_93795	insulator	insulator
Co1Mo1P1_ICSD_2421	metal	metal
Co1Mo1P2_ICSD_624219	metal	metal
Co1Mo1_ICSD_187980	metal	metal
Co1Mo2S4_ICSD_2018	insulator	metal
Co1Mo2S4_ICSD_26173	metal	metal
Co1Mo4Zr9_ICSD_624247	metal	metal
Co1N1_ICSD_184377	metal	metal
Co1N1_ICSD_79936	metal	metal
Co1N2O6_ICSD_412004	insulator	insulator
Co1N3_ICSD_162105	metal	metal
Co1N3_ICSD_162106	metal	metal
Co1N6Na3O12_ICSD_280730	insulator	insulator
Co1N6Na3O12_ICSD_39573	insulator	insulator
Co1N6O12Pb1Rb2_ICSD_23116	metal	metal
Co1Na1O2_ICSD_6152	insulator	insulator
Co1Na1O4P1_ICSD_280175	insulator	insulator
Co1Na1O4P1_ICSD_82752	insulator	insulator
Co1Na2O14P4Zr1_ICSD_65704	insulator	insulator
Co1Na2S2_ICSD_67386	insulator	insulator
Co1Na3O2_ICSD_73211	insulator	insulator
Co1Na4O3_ICSD_10473	insulator	insulator
Co1Na4O3_ICSD_10501	insulator	insulator
Co1Na5O2S1_ICSD_412978	insulator	insulator
Co1Na6S4_ICSD_624260	insulator	insulator
Co1Na6Se4_ICSD_68604	insulator	insulator
Co1Nb1O4_ICSD_16377	insulator	insulator
Co1Nb1P1_ICSD_49727	metal	metal
Co1Nb1Sb1_ICSD_107129	metal	metal
Co1Nb1Si1_ICSD_624322	metal	metal

Supplementary Table 287. Five-fold cross validated predictions for the metal/insulator classification (279/598).

system	calculated	predicted
Co1Nb1Sn1_ICSD_102552	metal	metal
Co1Nb1Sn1_ICSD_102553	metal	metal
Co1Nb1Te2_ICSD_73739	metal	metal
Co1Nb1_ICSD_187977	metal	metal
Co1Nb2S4_ICSD_624299	metal	metal
Co1Nb3S6_ICSD_53016	metal	metal
Co1Nb4P1_ICSD_9967	metal	metal
Co1Nb4S8_ICSD_624300	metal	metal
Co1Nb4Se8_ICSD_624305	metal	metal
Co1Nb4Si1_ICSD_43233	metal	metal
Co1Nd1O3_ICSD_28923	metal	metal
Co1Nd1O3_ICSD_82078	metal	metal
Co1Nd1P1_ICSD_624416	metal	metal
Co1Nd1Sb2_ICSD_657922	metal	metal
Co1Nd1Sb2_ICSD_657993	metal	metal
Co1Nd1Sb3_ICSD_419541	metal	metal
Co1Nd1Si1_ICSD_80513	metal	metal
Co1Nd1Si2_ICSD_83743	metal	metal
Co1Nd1Si2_ICSD_83744	metal	metal
Co1Nd1Si3_ICSD_624433	metal	metal
Co1Ni1O12P4_ICSD_37136	insulator	insulator
Co1Ni1Si1_ICSD_165256	metal	metal
Co1Ni1Sn1_ICSD_102582	metal	metal
Co1Ni1_ICSD_187983	metal	metal
Co1Ni2S4_ICSD_624469	metal	metal
Co1O10P2V2_ICSD_419532	insulator	insulator
Co1O1_ICSD_174027	metal	metal
Co1O1_ICSD_29082	insulator	metal
Co1O1_ICSD_29226	metal	metal
Co1O1_ICSD_43458	insulator	insulator
Co1O2Pd1_ICSD_31917	metal	metal
Co1O2Pt1_ICSD_31916	metal	metal
Co1O2Rb3_ICSD_94437	insulator	insulator
Co1O2_ICSD_88722	insulator	insulator
Co1O2_ICSD_95440	insulator	insulator
Co1O3Pr1_ICSD_28922	metal	metal
Co1O3Pr1_ICSD_88738	metal	metal
Co1O3Se1_ICSD_496	insulator	insulator
Co1O3Si1_ICSD_17054	insulator	insulator
Co1O3Sr1_ICSD_108896	metal	insulator
Co1O3Sr1_ICSD_184078	metal	metal
Co1O3Te1_ICSD_500	insulator	insulator
Co1O3Ti1_ICSD_16548	insulator	insulator
Co1O4P1_ICSD_246224	insulator	insulator
Co1O4Re1_ICSD_72872	insulator	metal
Co1O4Rh2_ICSD_109301	insulator	metal
Co1O4S1_ICSD_16985	insulator	insulator
Co1O4S1_ICSD_18176	insulator	insulator
Co1O4S1_ICSD_33736	insulator	insulator
Co1O4Sb2_ICSD_262626	insulator	insulator

Supplementary Table 288. Five-fold cross validated predictions for the metal/insulator classification (280/598).

system	calculated	predicted
Co1O4Se1_ICSD_109072	insulator	insulator
Co1O4Sr2_ICSD_246483	metal	metal
Co1O4U1_ICSD_26939	insulator	insulator
Co1O4W1_ICSD_15851	insulator	insulator
Co1O6Os1Sr2_ICSD_236435	metal	metal
Co1O6Pb2Te1_ICSD_169192	metal	insulator
Co1O6Pb2Te1_ICSD_169196	metal	metal
Co1O6Pb2Te1_ICSD_405782	insulator	metal
Co1O6Pb2W1_ICSD_77912	insulator	insulator
Co1O6Pt3_ICSD_35338	insulator	insulator
Co1O6Re1Sr2_ICSD_173488	metal	metal
Co1O6Sb2_ICSD_203094	insulator	insulator
Co1O6Sr2W1_ICSD_245470	insulator	metal
Co1O6Sr2W1_ICSD_28598	metal	insulator
Co1O6Sr3Zn1_ICSD_189230	insulator	insulator
Co1O6Ta2_ICSD_166673	insulator	insulator
Co1O6U2_ICSD_166250	insulator	insulator
Co1O6U2_ICSD_28127	insulator	insulator
Co1O6V2_ICSD_188911	insulator	insulator
Co1O6V2_ICSD_263002	insulator	insulator
Co1O6V2_ICSD_46003	insulator	insulator
Co1O8Re2_ICSD_51015	insulator	insulator
Co1P1Pd1_ICSD_624599	metal	metal
Co1P1Pr1_ICSD_624605	metal	metal
Co1P1Sc1_ICSD_624621	metal	metal
Co1P1Se1_ICSD_624624	insulator	insulator
Co1P1Sm1_ICSD_624632	metal	metal
Co1P1Ta1_ICSD_624636	metal	metal
Co1P1Ta4_ICSD_624637	metal	metal
Co1P1Ti1_ICSD_624646	metal	metal
Co1P1V1_ICSD_624659	metal	metal
Co1P1W1_ICSD_624662	metal	metal
Co1P1Zr1_ICSD_49726	metal	metal
Co1P1Zr2_ICSD_84825	metal	metal
Co1P1Zr4_ICSD_624677	metal	metal
Co1P1_ICSD_43401	metal	metal
Co1P2U1_ICSD_656838	metal	metal
Co1P2W1_ICSD_624660	metal	metal
Co1P2_ICSD_38316	metal	insulator
Co1P3Si3_ICSD_82221	insulator	insulator
Co1P3_ICSD_624594	metal	metal
Co1Pd2Se2_ICSD_416513	metal	metal
Co1Pd2Te2_ICSD_416514	metal	metal
Co1Pr1Sb2_ICSD_657921	metal	metal
Co1Pr1Si1_ICSD_624757	metal	metal
Co1Pr1Si1_ICSD_658267	metal	metal
Co1Pt1_ICSD_102620	metal	metal
Co1Pt1_ICSD_624775	metal	metal
Co1Pt1_ICSD_624781	metal	metal
Co1Pt3_ICSD_107048	metal	metal

Supplementary Table 289. Five-fold cross validated predictions for the metal/insulator classification (281/598).

system	calculated	predicted
Co1Rb2S2_ICSD_67388	insulator	insulator
Co1Rb2Se2_ICSD_67391	insulator	insulator
Co1Rh2S4_ICSD_624812	insulator	metal
Co1Rh2Sn1_ICSD_624817	metal	metal
Co1S17U8_ICSD_624880	insulator	insulator
Co1S1Sb1_ICSD_624862	insulator	insulator
Co1S1Sb1_ICSD_624864	insulator	insulator
Co1S1_ICSD_624831	insulator	metal
Co1S2_ICSD_624852	metal	insulator
Co1S4V2_ICSD_624884	metal	metal
Co1S5U2_ICSD_624883	insulator	insulator
Co1Sb1Ta1_ICSD_107128	metal	metal
Co1Sb1Ti1_ICSD_624919	insulator	metal
Co1Sb1V1_ICSD_107117	metal	metal
Co1Sb1Zr1_ICSD_108317	metal	metal
Co1Sb1_ICSD_624893	metal	metal
Co1Sb1_ICSD_624909	metal	metal
Co1Sb2_ICSD_161492	metal	metal
Co1Sb2_ICSD_43500	metal	metal
Co1Sb2_ICSD_624899	metal	metal
Co1Sb2_ICSD_76120	metal	metal
Co1Sb3Zr5_ICSD_624928	metal	metal
Co1Sb3_ICSD_34048	metal	metal
Co1Sc1Si1_ICSD_165245	metal	metal
Co1Sc1Sn1_ICSD_624977	metal	metal
Co1Sc1_ICSD_102642	metal	metal
Co1Sc2Si2_ICSD_41746	metal	metal
Co1Sc2Si2_ICSD_624952	metal	metal
Co1Sc2_ICSD_102643	metal	metal
Co1Sc3_ICSD_2407	metal	metal
Co1Se17U8_ICSD_601673	metal	metal
Co1Se1_ICSD_53959	metal	metal
Co1Se1_ICSD_56136	metal	metal
Co1Se2_ICSD_246908	metal	metal
Co1Se2_ICSD_42540	metal	metal
Co1Se2_ICSD_624982	metal	metal
Co1Se4Ti2_ICSD_625002	metal	metal
Co1Si1Sm1_ICSD_625037	metal	metal
Co1Si1Ta1_ICSD_625050	metal	metal
Co1Si1Tb1_ICSD_80516	metal	metal
Co1Si1Tb1_ICSD_88213	metal	metal
Co1Si1Ti1_ICSD_42735	metal	metal
Co1Si1Ti1_ICSD_53079	metal	metal
Co1Si1Ti2_ICSD_188945	metal	metal
Co1Si1U1_ICSD_50081	metal	metal
Co1Si1V1_ICSD_409847	metal	metal
Co1Si1Zr1_ICSD_625144	metal	metal
Co1Si1_ICSD_155928	metal	metal
Co1Si1_ICSD_625027	metal	metal
Co1Si2Sm1_ICSD_625036	metal	metal

Supplementary Table 290. Five-fold cross validated predictions for the metal/insulator classification (282/598).

system	calculated	predicted
Co1Si2Tb1_ICSD_99115	metal	metal
Co1Si2Tm1_ICSD_625095	metal	metal
Co1Si2Y1_ICSD_625129	metal	metal
Co1Si2Zr2_ICSD_653752	metal	metal
Co1Si2_ICSD_625030	metal	metal
Co1Si3Sm1_ICSD_625038	metal	metal
Co1Si3Tb1_ICSD_625062	metal	metal
Co1Si3Th1_ICSD_625074	metal	metal
Co1Sn1Tb1_ICSD_106977	metal	metal
Co1Sn1Th1_ICSD_61484	metal	metal
Co1Sn1Ti1_ICSD_657174	metal	metal
Co1Sn1Tm1_ICSD_625294	metal	metal
Co1Sn1U1_ICSD_601799	metal	metal
Co1Sn1U1_ICSD_625297	metal	metal
Co1Sn1V1_ICSD_185079	metal	metal
Co1Sn1Y1_ICSD_601850	metal	metal
Co1Sn1Zr1_ICSD_102686	metal	metal
Co1Sn1_ICSD_161110	metal	metal
Co1Sn1_ICSD_161113	metal	metal
Co1Sn1_ICSD_161115	metal	metal
Co1Sn1_ICSD_161117	metal	metal
Co1Sn1_ICSD_161118	metal	metal
Co1Sn1_ICSD_55564	metal	metal
Co1Sn1_ICSD_625264	metal	metal
Co1Sn2Tb1_ICSD_240096	metal	metal
Co1Sn2_ICSD_102672	metal	metal
Co1Sn2_ICSD_247484	metal	metal
Co1Sn2_ICSD_625263	metal	metal
Co1Sn3_ICSD_166871	metal	metal
Co1Ta1Te2_ICSD_73738	metal	metal
Co1Ta1_ICSD_187978	metal	metal
Co1Ta2_ICSD_625322	metal	metal
Co1Ta4Te4_ICSD_659269	metal	metal
Co1Tb3_ICSD_625381	metal	metal
Co1Tb6Te2_ICSD_247431	metal	metal
Co1Te1_ICSD_625410	metal	metal
Co1Te2_ICSD_625401	metal	metal
Co1Te2_ICSD_625403	metal	metal
Co1Te2_ICSD_625406	metal	metal
Co1Th1_ICSD_102699	metal	metal
Co1Th1_ICSD_261441	metal	metal
Co1Ti1_ICSD_625477	metal	metal
Co1Ti2_ICSD_102706	metal	metal
Co1Ti2_ICSD_625483	metal	metal
Co1U1_ICSD_625521	insulator	metal
Co1U6_ICSD_108323	metal	metal
Co1V1_ICSD_187976	metal	metal
Co1V3_ICSD_102716	metal	metal
Co1W1_ICSD_187974	metal	metal
Co1Y1_ICSD_20954	metal	metal

Supplementary Table 291. Five-fold cross validated predictions for the metal/insulator classification (283/598).

system	calculated	predicted
Co1Y3_ICSD_625576	metal	metal
Co1Zn13_ICSD_102736	metal	metal
Co1Zr1_ICSD_102738	metal	metal
Co1Zr2_ICSD_102740	metal	metal
Co1Zr2_ICSD_625665	metal	metal
Co1Zr2_ICSD_625682	metal	metal
Co1Zr3_ICSD_102741	metal	metal
Co1Zr3_ICSD_625670	metal	metal
Co1_ICSD_165725	metal	metal
Co1_ICSD_44990	metal	metal
Co1_ICSD_53806	metal	metal
Co1_ICSD_622436	metal	metal
Co1_ICSD_622439	metal	metal
Co23Hf6_ICSD_623762	metal	metal
Co23Zr6_ICSD_102744	metal	metal
Co2Cr1Ga1_ICSD_102318	metal	metal
Co2Cr1In1_ICSD_416260	metal	metal
Co2Cr1Sb1_ICSD_180208	metal	metal
Co2Cs1S2_ICSD_622529	metal	metal
Co2Cs1Se2_ICSD_622533	metal	metal
Co2Cu1Ge2_ICSD_62856	metal	metal
Co2Cu1S4_ICSD_622571	metal	metal
Co2Dy1Ge2_ICSD_622667	metal	metal
Co2Dy1In1_ICSD_658675	metal	metal
Co2Dy1Si2_ICSD_622705	metal	metal
Co2Dy1_ICSD_622647	metal	metal
Co2Dy3Ge4_ICSD_622666	metal	metal
Co2Er1Ge2_ICSD_106878	metal	metal
Co2Er1Si2_ICSD_31426	metal	metal
Co2Er1_ICSD_622733	metal	metal
Co2Er3Ge4_ICSD_622806	metal	metal
Co2Er5Te2_ICSD_150370	metal	metal
Co2Eu1P2_ICSD_47200	metal	metal
Co2Fe1Ga1_ICSD_247653	metal	metal
Co2Fe1Ge1_ICSD_622905	metal	metal
Co2Fe1In1_ICSD_102392	metal	metal
Co2Fe1Si1_ICSD_622985	metal	metal
Co2Ga1Hf1_ICSD_623080	metal	metal
Co2Ga1La1_ICSD_623100	metal	metal
Co2Ga1Mn1_ICSD_102438	metal	metal
Co2Ga1Nb1_ICSD_623126	metal	metal
Co2Ga1Ni1_ICSD_157788	metal	metal
Co2Ga1Ni1_ICSD_169731	metal	metal
Co2Ga1Ni1_ICSD_169733	metal	metal
Co2Ga1Ta1_ICSD_102451	metal	metal
Co2Ga1Ti1_ICSD_623209	metal	metal
Co2Ga1V1_ICSD_623230	metal	metal
Co2Ga7Mg3_ICSD_157364	metal	metal
Co2Gd1Ge2_ICSD_106848	metal	metal
Co2Gd1Si2_ICSD_106820	metal	metal

Supplementary Table 292. Five-fold cross validated predictions for the metal/insulator classification (284/598).

system	calculated	predicted
Co2Gd1Zn20_ICSD_152112	metal	metal
Co2Gd1_ICSD_102465	metal	metal
Co2Gd1_ICSD_623288	metal	metal
Co2Gd1_ICSD_623341	metal	metal
Co2Gd4Mg3_ICSD_417035	metal	metal
Co2Ge1Li1_ICSD_25324	metal	metal
Co2Ge1Mn1_ICSD_623496	metal	metal
Co2Ge1O4_ICSD_21115	metal	insulator
Co2Ge1Ti1_ICSD_169469	metal	metal
Co2Ge1Zn1_ICSD_52994	metal	metal
Co2Ge1_ICSD_108289	metal	metal
Co2Ge1_ICSD_623417	metal	metal
Co2Ge1_ICSD_623418	metal	metal
Co2Ge1_ICSD_623430	metal	metal
Co2Ge1_ICSD_623433	metal	metal
Co2Ge2Ho1_ICSD_55336	metal	metal
Co2Ge2Ho1_ICSD_623454	metal	metal
Co2Ge2La1_ICSD_81750	metal	metal
Co2Ge2Nd1_ICSD_106877	metal	metal
Co2Ge2Nd1_ICSD_55787	metal	metal
Co2Ge2Sr1_ICSD_407	metal	metal
Co2Ge2Tb1_ICSD_86030	metal	metal
Co2Ge2Th1_ICSD_43288	metal	metal
Co2Ge2Tm1_ICSD_107213	metal	metal
Co2Ge2U1_ICSD_623651	metal	metal
Co2Ge2U1_ICSD_623653	metal	metal
Co2Ge2Y1_ICSD_81748	metal	metal
Co2Ge2Yb1_ICSD_623671	metal	metal
Co2Ge3Sc3_ICSD_106479	metal	metal
Co2Ge4Ho3_ICSD_623445	metal	metal
Co2Ge4Tb3_ICSD_62292	metal	metal
Co2Ge4Y3_ICSD_623661	metal	metal
Co2H1O5P1_ICSD_79333	insulator	insulator
Co2Hf1Si2_ICSD_623793	metal	metal
Co2Hf1Sn1_ICSD_102483	metal	metal
Co2Hf1_ICSD_623776	metal	metal
Co2Hf3Si3_ICSD_623799	metal	metal
Co2Ho1In1_ICSD_658676	metal	metal
Co2Ho1Si2_ICSD_55909	metal	metal
Co2Ho1_ICSD_108296	metal	metal
Co2I1Pr2_ICSD_416273	metal	metal
Co2I1Tb2_ICSD_418080	metal	metal
Co2In1Tb1_ICSD_370040	metal	metal
Co2In1Y1_ICSD_658677	metal	metal
Co2In1Zr2_ICSD_404847	metal	metal
Co2In5Zr4_ICSD_55578	metal	metal
Co2In9K1_ICSD_260251	metal	metal
Co2K1S2_ICSD_623956	metal	metal
Co2K1Se2_ICSD_182837	metal	metal
Co2K2Na4O5_ICSD_64678	insulator	insulator

Supplementary Table 293. Five-fold cross validated predictions for the metal/insulator classification (285/598).

system	calculated	predicted
Co2K2O12S3_ICSD_81082	insulator	insulator
Co2K4O5Rb2_ICSD_33797	insulator	insulator
Co2K5N12O24Y1_ICSD_24536	metal	metal
Co2K6O7_ICSD_6153	insulator	insulator
Co2K9S7_ICSD_82347	metal	insulator
Co2La1P2_ICSD_87582	metal	metal
Co2La1Si2_ICSD_52999	metal	metal
Co2La1_ICSD_102510	metal	metal
Co2La2O3Se2_ICSD_261191	insulator	metal
Co2La3S7_ICSD_624016	insulator	insulator
Co2Lu1_ICSD_603225	metal	metal
Co2Lu1_ICSD_624045	metal	metal
Co2Lu1_ICSD_624055	metal	metal
Co2Mg1_ICSD_624068	metal	metal
Co2Mg3Tb4_ICSD_417036	metal	metal
Co2Mn1Sb1_ICSD_53002	metal	metal
Co2Mn1Si1_ICSD_624143	metal	metal
Co2Mn1Sn1_ICSD_102532	metal	metal
Co2Mo3N1_ICSD_96417	metal	metal
Co2Mo3O8_ICSD_10143	insulator	insulator
Co2N12O24Tl5Y1_ICSD_24537	metal	metal
Co2N1Nb4_ICSD_624252	metal	metal
Co2N1Ta4_ICSD_624254	metal	metal
Co2N1V4_ICSD_624256	metal	metal
Co2N1Zr4_ICSD_624258	metal	metal
Co2N1_ICSD_152812	metal	metal
Co2Na3O6Sb1_ICSD_245538	insulator	insulator
Co2Na4O5Rb2_ICSD_64680	insulator	insulator
Co2Na5S5_ICSD_201439	metal	insulator
Co2Na6O6_ICSD_99580	insulator	insulator
Co2Na7O6Rb1_ICSD_66006	insulator	insulator
Co2Nb1Sn1_ICSD_624326	metal	metal
Co2Nb1_ICSD_102549	metal	metal
Co2Nb1_ICSD_624270	metal	metal
Co2Nb1_ICSD_624286	metal	metal
Co2Nb2Te4_ICSD_71196	metal	metal
Co2Nb3Si1_ICSD_53017	metal	metal
Co2Nb4Pd1Se12_ICSD_41026	metal	metal
Co2Nd1P2_ICSD_73652	metal	metal
Co2Nd1Si2_ICSD_31420	metal	metal
Co2Nd1Si2_ICSD_31421	metal	metal
Co2Nd1_ICSD_154725	metal	metal
Co2Nd1_ICSD_154728	metal	metal
Co2Nd1_ICSD_154731	metal	metal
Co2Nd1_ICSD_154733	metal	metal
Co2Nd1_ICSD_154734	metal	metal
Co2Nd1_ICSD_154736	metal	metal
Co2Nd1_ICSD_154738	metal	metal
Co2Nd1_ICSD_624353	metal	metal
Co2Nd1_ICSD_624374	metal	metal

Supplementary Table 294. Five-fold cross validated predictions for the metal/insulator classification (286/598).

system	calculated	predicted
Co2Nd1_ICSD_624395	metal	metal
Co2Ni1S4_ICSD_624468	metal	metal
Co2Ni1Se4_ICSD_624485	metal	metal
Co2O12P4_ICSD_300027	insulator	insulator
Co2O14P4Si1_ICSD_82403	insulator	insulator
Co2O3Rb2_ICSD_95833	insulator	insulator
Co2O4Rb5_ICSD_73190	insulator	insulator
Co2O4Si1_ICSD_260092	insulator	insulator
Co2O4Si1_ICSD_8132	insulator	insulator
Co2O4Si1_ICSD_859	insulator	insulator
Co2O6P2Sc2Sr4_ICSD_262357	metal	metal
Co2O6Sr3_ICSD_182288	metal	metal
Co2O7P2_ICSD_59291	insulator	insulator
Co2O7P2_ICSD_74542	insulator	insulator
Co2O7Sb2_ICSD_247302	insulator	insulator
Co2O7V2_ICSD_2357	insulator	insulator
Co2O8P2Sr1_ICSD_73960	insulator	insulator
Co2O8Te3_ICSD_50702	insulator	insulator
Co2P1_ICSD_107550	metal	metal
Co2P1_ICSD_94379	metal	metal
Co2P2Pr1_ICSD_73650	metal	metal
Co2P2S6_ICSD_624616	insulator	insulator
Co2P2Sm1_ICSD_624631	metal	metal
Co2P2Sr1_ICSD_10466	metal	metal
Co2P2Th1_ICSD_624645	metal	metal
Co2P2U1_ICSD_67932	metal	metal
Co2P2U1_ICSD_78577	metal	metal
Co2Pd1Se12Ta4_ICSD_73319	metal	metal
Co2Pr1Si2_ICSD_31418	metal	metal
Co2Pr1_ICSD_624752	metal	metal
Co2Pu1Si2_ICSD_604282	metal	metal
Co2Pu1Si2_ICSD_73027	metal	metal
Co2Pu1_ICSD_102630	metal	metal
Co2Pu1_ICSD_624789	metal	metal
Co2Rb1S2_ICSD_624795	metal	metal
Co2Rb1Se2_ICSD_624798	metal	metal
Co2S2Tl1_ICSD_100438	insulator	metal
Co2S6Ta9_ICSD_624869	metal	metal
Co2Sc1Si2_ICSD_624959	metal	metal
Co2Sc1Sn1_ICSD_102646	metal	metal
Co2Sc1_ICSD_150739	metal	metal
Co2Sc3Si3_ICSD_41745	metal	metal
Co2Sc3Si3_ICSD_624954	metal	metal
Co2Se2Tl1_ICSD_625007	metal	metal
Co2Se2Tl1_ICSD_95138	metal	metal
Co2Si1Ti1_ICSD_625083	metal	metal
Co2Si1V1_ICSD_659022	metal	metal
Co2Si1_ICSD_165251	metal	metal
Co2Si2Sm1_ICSD_53073	metal	metal
Co2Si2Tb1_ICSD_53077	metal	metal

Supplementary Table 295. Five-fold cross validated predictions for the metal/insulator classification (287/598).

system	calculated	predicted
Co2Si2Tb1_ICSD_55924	metal	metal
Co2Si2Th1_ICSD_68210	metal	metal
Co2Si2U1_ICSD_53084	metal	metal
Co2Si2U1_ICSD_625105	metal	metal
Co2Si2Y1_ICSD_53088	metal	metal
Co2Si2Yb1_ICSD_53089	metal	metal
Co2Si2Zr1_ICSD_625135	metal	metal
Co2Si3Zr3_ICSD_625139	metal	metal
Co2Si3_ICSD_625021	metal	insulator
Co2Sm1_ICSD_102665	metal	metal
Co2Sn1Ti1_ICSD_625282	metal	metal
Co2Sn1U2_ICSD_602789	metal	metal
Co2Sn1V1_ICSD_625303	metal	metal
Co2Sn1Zr1_ICSD_625314	metal	metal
Co2Sn1_ICSD_102673	metal	metal
Co2Sn1_ICSD_625262	metal	metal
Co2Sn2Th1_ICSD_602653	metal	metal
Co2Sn2U1_ICSD_602870	metal	metal
Co2Ta1_ICSD_108152	metal	metal
Co2Ta1_ICSD_625325	metal	metal
Co2Ta1_ICSD_625326	metal	metal
Co2Ta1_ICSD_625334	metal	metal
Co2Ta1_ICSD_625347	metal	metal
Co2Tb1_ICSD_102695	metal	metal
Co2Tb1_ICSD_152583	metal	metal
Co2Tb1_ICSD_157055	metal	metal
Co2Tb1_ICSD_625375	metal	metal
Co2Ti1_ICSD_625463	metal	metal
Co2Ti1_ICSD_625482	metal	metal
Co2U1Zn20_ICSD_183633	metal	metal
Co2U1_ICSD_102713	metal	metal
Co2U1_ICSD_102714	metal	metal
Co2U1_ICSD_625514	metal	metal
Co2Y1_ICSD_625633	metal	metal
Co2Y3_ICSD_1844	metal	metal
Co2Yb1_ICSD_625660	metal	metal
Co2Yb1_ICSD_625661	metal	metal
Co2Zr1_ICSD_603796	metal	metal
Co3Cr1_ICSD_187968	metal	metal
Co3Cs2S4_ICSD_65256	insulator	insulator
Co3Cu2Ho1_ICSD_622555	metal	metal
Co3Dy1_ICSD_602917	metal	metal
Co3Dy2Ga9_ICSD_622665	metal	metal
Co3Dy2Si5_ICSD_622709	metal	metal
Co3Dy2Si5_ICSD_88274	metal	metal
Co3Dy4Ga16_ICSD_261781	metal	metal
Co3Er1Ga2_ICSD_622797	metal	metal
Co3Er1_ICSD_172411	metal	metal
Co3Er1_ICSD_622722	metal	metal
Co3Er1_ICSD_622769	metal	metal

Supplementary Table 296. Five-fold cross validated predictions for the metal/insulator classification (288/598).

system	calculated	predicted
Co3Er2Ga9_ICSD_622804	metal	metal
Co3Er2Si5_ICSD_622857	metal	metal
Co3Er4Ga16_ICSD_261783	metal	metal
Co3Fe13_ICSD_155844	metal	metal
Co3Fe1_ICSD_187971	metal	metal
Co3Fe5_ICSD_155841	metal	metal
Co3Ga16Ho4_ICSD_261782	metal	metal
Co3Ga16Tb4_ICSD_422806	metal	metal
Co3Ga16Y4_ICSD_261784	metal	metal
Co3Ga1Y3_ICSD_10044	metal	metal
Co3Ga2Ho1_ICSD_623089	metal	metal
Co3Ga2Tb1_ICSD_102452	metal	metal
Co3Ga9Ho2_ICSD_20952	metal	metal
Co3Ge1Mn2_ICSD_52972	metal	metal
Co3Hf2Si4_ICSD_20958	metal	metal
Co3Ho1_ICSD_102486	metal	metal
Co3Ho1_ICSD_602909	metal	metal
Co3Ho2Si5_ICSD_623902	metal	metal
Co3In1N1_ICSD_247066	metal	metal
Co3In1S2Sn1_ICSD_425137	metal	metal
Co3In2S2_ICSD_10011	metal	metal
Co3La2_ICSD_102511	metal	metal
Co3La2_ICSD_623979	metal	metal
Co3La3O8_ICSD_86176	metal	metal
Co3La4O10_ICSD_85592	metal	metal
Co3Lu1_ICSD_624039	metal	metal
Co3Mn1_ICSD_187970	metal	metal
Co3Mo1_ICSD_187969	metal	metal
Co3Mo1_ICSD_624216	metal	metal
Co3Mo3N1_ICSD_180409	metal	metal
Co3N1W3_ICSD_85883	metal	metal
Co3Nb1_ICSD_150944	metal	metal
Co3Nb1_ICSD_187966	metal	metal
Co3Nb2Si1_ICSD_53018	metal	metal
Co3Ni1_ICSD_187972	metal	metal
Co3O1Ti3_ICSD_29054	metal	metal
Co3O4_ICSD_69373	insulator	metal
Co3O8P2_ICSD_38260	insulator	insulator
Co3O8P2_ICSD_6208	insulator	insulator
Co3O8P2_ICSD_9850	metal	insulator
Co3O8U2_ICSD_86237	insulator	insulator
Co3O8V2_ICSD_2645	insulator	insulator
Co3S2Sn2_ICSD_173764	metal	metal
Co3S4_ICSD_106489	insulator	metal
Co3Sb4Th3_ICSD_93872	metal	metal
Co3Sb4U3_ICSD_624924	insulator	metal
Co3Sc2Si1_ICSD_9966	metal	metal
Co3Sc2Si5_ICSD_624961	metal	metal
Co3Se4_ICSD_99989	metal	metal
Co3Si1_ICSD_625031	metal	metal

Supplementary Table 297. Five-fold cross validated predictions for the metal/insulator classification (289/598).

system	calculated	predicted
Co3Si5Tb2_ICSD_88273	metal	metal
Co3Si5U2_ICSD_20930	metal	metal
Co3Sn2_ICSD_625259	metal	metal
Co3Ta1_ICSD_187985	metal	metal
Co3Ta1_ICSD_187991	metal	metal
Co3Ta1_ICSD_24581	metal	metal
Co3Tb1_ICSD_625395	metal	metal
Co3Th1_ICSD_625418	metal	metal
Co3Th1_ICSD_625433	metal	metal
Co3Th7_ICSD_625455	metal	metal
Co3Ti1_ICSD_187990	metal	metal
Co3Ti1_ICSD_625468	metal	metal
Co3Ti1_ICSD_625484	metal	metal
Co3V1_ICSD_102718	metal	metal
Co3V1_ICSD_187965	metal	metal
Co3V1_ICSD_187988	metal	metal
Co3V1_ICSD_187994	metal	metal
Co3W1_ICSD_102722	metal	metal
Co3W1_ICSD_187987	metal	metal
Co3W1_ICSD_187993	metal	metal
Co3Y1_ICSD_159894	metal	metal
Co3Y1_ICSD_603098	metal	metal
Co3Y1_ICSD_625596	metal	metal
Co3Y1_ICSD_625636	metal	metal
Co4Dy3Ge13_ICSD_622668	metal	metal
Co4Dy7Ge12In1_ICSD_158000	metal	metal
Co4Er3Ge13_ICSD_622808	metal	metal
Co4Ge12Ho7In1_ICSD_158001	metal	metal
Co4Ge12In1Yb7_ICSD_158002	metal	metal
Co4Ge13Ho3_ICSD_623447	metal	metal
Co4Ge13Lu3_ICSD_623466	metal	metal
Co4Ge13Tb3_ICSD_623616	metal	metal
Co4Ge13Y3_ICSD_623663	metal	metal
Co4Ge13Yb3_ICSD_623672	metal	metal
Co4La3Sn13_ICSD_600229	metal	metal
Co4Nb4Si7_ICSD_624323	metal	metal
Co4Nd1P12_ICSD_604478	metal	metal
Co4P12Pr1_ICSD_604477	metal	metal
Co4P12Yb1_ICSD_604479	metal	metal
Co4Pr3Sn13_ICSD_600231	metal	metal
Co4Sb12Sn1_ICSD_87886	metal	metal
Co4Sc5Si10_ICSD_30519	metal	metal
Co4Si7Ta4_ICSD_625051	metal	metal
Co4Sn13Tb3_ICSD_423113	metal	metal
Co4Sn13Yb3_ICSD_625310	metal	metal
Co4Sn1U1_ICSD_159198	metal	metal
Co5Dy1_ICSD_102345	metal	metal
Co5Dy1_ICSD_659060	metal	metal
Co5Er1_ICSD_102365	metal	metal
Co5Er1_ICSD_622763	metal	metal

Supplementary Table 298. Five-fold cross validated predictions for the metal/insulator classification (290/598).

system	calculated	predicted
Co5Fe11_ICSD_155842	metal	metal
Co5Ga1Y5_ICSD_102490	metal	metal
Co5Gd1_ICSD_102466	metal	metal
Co5Ge7_ICSD_43386	metal	metal
Co5H4La1_ICSD_158278	metal	metal
Co5Ho1_ICSD_102488	metal	metal
Co5Ho1_ICSD_623848	metal	metal
Co5Ho1_ICSD_623864	metal	metal
Co5La1P3_ICSD_86373	metal	metal
Co5La1_ICSD_158277	metal	metal
Co5La1_ICSD_623973	metal	metal
Co5La1_ICSD_623977	metal	metal
Co5La1_ICSD_623990	metal	metal
Co5La1_ICSD_623992	metal	metal
Co5La1_ICSD_624006	metal	metal
Co5Nd1_ICSD_102559	metal	metal
Co5Nd1_ICSD_624387	metal	metal
Co5Nd1_ICSD_624396	metal	metal
Co5Nd1_ICSD_657142	metal	metal
Co5O15Sr6_ICSD_155311	insulator	metal
Co5P3Y1_ICSD_40505	metal	metal
Co5Pr1_ICSD_624687	metal	metal
Co5Sm1_ICSD_102667	metal	metal
Co5Tb1_ICSD_625383	metal	metal
Co5Tb1_ICSD_625389	metal	metal
Co5Th1_ICSD_102702	metal	metal
Co5Th1_ICSD_625424	metal	metal
Co5Th1_ICSD_625430	metal	metal
Co5Th1_ICSD_625431	metal	metal
Co5Y1_ICSD_625573	metal	metal
Co5Y1_ICSD_625579	metal	metal
Co5Y1_ICSD_625597	metal	metal
Co6F18Rb6_ICSD_410388	insulator	insulator
Co6Ge6Hf1_ICSD_623442	metal	metal
Co6Ge6Li1_ICSD_41459	metal	metal
Co6Ge6Li1_ICSD_659653	metal	metal
Co6Ge6Mg1_ICSD_623475	metal	metal
Co6Ge6Sc1_ICSD_623581	metal	metal
Co6Ge6Sc1_ICSD_623590	metal	metal
Co6Ge6Tb1_ICSD_623625	metal	metal
Co6Ge6Ti1_ICSD_623631	metal	metal
Co6Ge6Zr1_ICSD_41460	metal	metal
Co6Li1P4_ICSD_69692	metal	metal
Co6Mg1P4_ICSD_94412	metal	metal
Co6Nb7_ICSD_624281	metal	metal
Co6Nd12Sn1_ICSD_240094	metal	metal
Co6Ta7_ICSD_625335	metal	metal
Co7Dy2_ICSD_658884	metal	metal
Co7Er2_ICSD_102366	metal	metal
Co7Fe9_ICSD_155840	metal	metal

Supplementary Table 299. Five-fold cross validated predictions for the metal/insulator classification (291/598).

system	calculated	predicted
Co7Ge6Hf4_ICSD_623435	metal	metal
Co7Ge6Sc4_ICSD_623580	metal	metal
Co7Ge6Ti4_ICSD_623638	metal	metal
Co7Ge6Zr4_ICSD_52996	metal	metal
Co7Hf2_ICSD_623764	metal	metal
Co7Ho2_ICSD_623816	metal	metal
Co7In14Zr9_ICSD_55561	metal	metal
Co7La2_ICSD_623993	metal	metal
Co7Mo6_ICSD_102544	metal	metal
Co7Nb6_ICSD_102550	metal	metal
Co7Th2_ICSD_102703	metal	metal
Co7W6_ICSD_102724	metal	metal
Co7Y2_ICSD_625563	metal	metal
Co8Fe1S8_ICSD_622969	metal	metal
Co8La1P5_ICSD_30701	metal	metal
Co8Mn4Nd1_ICSD_624077	metal	metal
Co8Mn4Pr1_ICSD_624111	metal	metal
Co8Ni1S8_ICSD_624466	metal	metal
Co8Pd1S8_ICSD_624682	metal	metal
Co8Rh1S8_ICSD_624810	metal	metal
Co8Ru1S8_ICSD_624822	metal	metal
Co9Er1Si2_ICSD_622849	metal	metal
Co9La1Si4_ICSD_156467	metal	metal
Co9Nd1Si4_ICSD_79971	metal	metal
Co9S8_ICSD_31753	insulator	metal
Co9S8_ICSD_40046	metal	metal
Co9Se8_ICSD_44857	metal	metal
Co9Se8_ICSD_624990	insulator	metal
Co9Si2Tb1_ICSD_625053	metal	metal
Co9Si2Th1_ICSD_81931	metal	metal
Co9Si2Y1_ICSD_625124	metal	metal
Co9Si4Tb1_ICSD_72092	metal	metal
Co9Si4Y1_ICSD_657471	metal	metal
Cr0.125Ga0.875P1_ICSD_181038	metal	insulator
Cr0.25Nb1S2_ICSD_42657	metal	metal
Cr1Cs1F4_ICSD_2278	insulator	insulator
Cr1Cs1H8N2O8_ICSD_261393	insulator	insulator
Cr1Cs1I3_ICSD_23382	insulator	insulator
Cr1Cs1I3_ICSD_8105	metal	insulator
Cr1Cs1Li1O4_ICSD_63417	insulator	insulator
Cr1Cs1P2S7_ICSD_260893	insulator	insulator
Cr1Cs2F6K1_ICSD_9705	insulator	insulator
Cr1Cs2F6_ICSD_29007	metal	insulator
Cr1Cu1O2_ICSD_163253	insulator	insulator
Cr1Cu1O2_ICSD_82065	insulator	metal
Cr1Cu1O3S1Sr2_ICSD_84962	insulator	insulator
Cr1Cu1O4_ICSD_60825	insulator	insulator
Cr1Cu1S2_ICSD_25627	metal	metal
Cr1Cu1S2_ICSD_42393	insulator	metal
Cr1Cu1Se2_ICSD_24798	metal	metal

Supplementary Table 300. Five-fold cross validated predictions for the metal/insulator classification (292/598).

system	calculated	predicted
Cr1Cu1Se2_ICSD_25625	metal	metal
Cr1Cu4O20Sr8Tl3_ICSD_81375	metal	insulator
Cr1Dy1O4_ICSD_167718	insulator	insulator
Cr1Dy1O4_ICSD_93788	insulator	insulator
Cr1Dy1O6Te1_ICSD_164943	insulator	insulator
Cr1Dy4Ga12_ICSD_424496	metal	metal
Cr1Er1O4_ICSD_247773	insulator	insulator
Cr1Er1O4_ICSD_96406	insulator	insulator
Cr1Er1O6Te1_ICSD_164945	insulator	insulator
Cr1Eu1O4_ICSD_51696	insulator	metal
Cr1Eu1O6Te1_ICSD_164940	insulator	insulator
Cr1F2_ICSD_31827	insulator	insulator
Cr1F3H18O9_ICSD_2379	insulator	insulator
Cr1F3K1_ICSD_172844	insulator	insulator
Cr1F3K1_ICSD_27690	insulator	insulator
Cr1F3O1_ICSD_59123	insulator	insulator
Cr1F3_ICSD_59972	insulator	insulator
Cr1F4Na1_ICSD_37108	insulator	insulator
Cr1F4Na2_ICSD_67250	insulator	insulator
Cr1F4Sr1_ICSD_26105	metal	insulator
Cr1F4Sr1_ICSD_9929	insulator	metal
Cr1F4_ICSD_78778	insulator	insulator
Cr1F5Mn1_ICSD_1617	insulator	insulator
Cr1F5Rb1_ICSD_418674	insulator	insulator
Cr1F5Rb2_ICSD_2343	insulator	insulator
Cr1F5_ICSD_419661	insulator	insulator
Cr1F6H18Mn1N6_ICSD_2728	insulator	insulator
Cr1F6H8N2Na1_ICSD_418735	insulator	insulator
Cr1F6Hg1_ICSD_10347	insulator	insulator
Cr1F6K1Rb2_ICSD_9707	insulator	insulator
Cr1F6K1_ICSD_418672	insulator	insulator
Cr1F6K2Na1_ICSD_40965	insulator	insulator
Cr1F6Li2_ICSD_10350	insulator	insulator
Cr1F6Mg1_ICSD_10344	insulator	insulator
Cr1F6Mn1Na1_ICSD_938	insulator	insulator
Cr1F6Mo1_ICSD_50507	insulator	insulator
Cr1F6Na1Rb2_ICSD_9706	insulator	insulator
Cr1F6Na1_ICSD_418671	insulator	insulator
Cr1F6Na3_ICSD_27070	insulator	insulator
Cr1F6Nb1_ICSD_75384	insulator	insulator
Cr1F6Rb1_ICSD_418673	insulator	insulator
Cr1F6Rb2_ICSD_29006	insulator	insulator
Cr1F6Sr1_ICSD_10342	metal	insulator
Cr1F6Zr1_ICSD_35719	metal	insulator
Cr1F7Pb2_ICSD_174348	insulator	insulator
Cr1F7Rb3_ICSD_9597	insulator	insulator
Cr1F8H2K2_ICSD_418678	insulator	insulator
Cr1F8H2Na2_ICSD_418677	insulator	insulator
Cr1Fe1P1_ICSD_161257	metal	metal
Cr1Fe2Ga1_ICSD_102755	metal	metal

Supplementary Table 301. Five-fold cross validated predictions for the metal/insulator classification (293/598).

system	calculated	predicted
Cr1Fe2Se4_ICSD_151873	metal	metal
Cr1Fe2Se4_ICSD_625966	metal	metal
Cr1Fe2Sn1_ICSD_185999	metal	metal
Cr1Fe2_ICSD_188276	metal	metal
Cr1Fe3_ICSD_188234	metal	metal
Cr1Fe3_ICSD_188258	metal	metal
Cr1Ga2S4_ICSD_626045	metal	insulator
Cr1Ga2S4_ICSD_626052	metal	insulator
Cr1Ga4_ICSD_626026	metal	metal
Cr1Gd1O4_ICSD_51903	insulator	insulator
Cr1Gd1O6Te1_ICSD_164941	insulator	insulator
Cr1Ge1Nb1_ICSD_42061	metal	metal
Cr1Ge1_ICSD_42417	metal	metal
Cr1Ge1_ICSD_602162	metal	metal
Cr1Ge2Li1O6_ICSD_174432	insulator	insulator
Cr1Ge2Na1O6_ICSD_260079	insulator	insulator
Cr1Ge3La1_ICSD_158976	metal	metal
Cr1Ge3Nd1_ICSD_186694	metal	metal
Cr1Ge3Nd1_ICSD_186696	metal	metal
Cr1Ge3Nd1_ICSD_186697	metal	metal
Cr1Ge3Pr1_ICSD_158978	metal	metal
Cr1H10N4O8S2_ICSD_279585	insulator	insulator
Cr1H12N3O8_ICSD_261390	metal	insulator
Cr1H1O2_ICSD_64754	insulator	insulator
Cr1H1O2_ICSD_88574	insulator	insulator
Cr1H1_ICSD_626118	metal	metal
Cr1H1_ICSD_76745	metal	metal
Cr1H2_ICSD_26630	metal	metal
Cr1H8N2O4_ICSD_16047	insulator	insulator
Cr1Hf1Si1_ICSD_626157	metal	metal
Cr1Hg1O4_ICSD_2224	insulator	insulator
Cr1Hg1O4_ICSD_416147	insulator	insulator
Cr1Hg1O4_ICSD_416148	insulator	insulator
Cr1Hg1O6Pb2_ICSD_280778	insulator	insulator
Cr1Hg5O5S2_ICSD_76860	insulator	insulator
Cr1Hg5O6_ICSD_81605	insulator	insulator
Cr1Ho1O4_ICSD_169873	insulator	insulator
Cr1Ho1O4_ICSD_173263	insulator	insulator
Cr1Ho1O6Te1_ICSD_164944	insulator	insulator
Cr1I1K1O6_ICSD_24341	insulator	insulator
Cr1I2_ICSD_2596	insulator	insulator
Cr1I2_ICSD_4073	insulator	insulator
Cr1I3Rb1_ICSD_15504	insulator	insulator
Cr1I6Tl4_ICSD_15502	insulator	insulator
Cr1I6Tl4_ICSD_38143	insulator	insulator
Cr1In2S4_ICSD_626203	metal	insulator
Cr1Ir3_ICSD_626213	metal	metal
Cr1K1O2_ICSD_40267	insulator	insulator
Cr1K1O2_ICSD_425293	insulator	insulator
Cr1K1O7V2_ICSD_245133	insulator	insulator

Supplementary Table 302. Five-fold cross validated predictions for the metal/insulator classification (294/598).

system	calculated	predicted
Cr1K1P2S7_ICSD_91754	insulator	insulator
Cr1K1S2_ICSD_25723	insulator	insulator
Cr1K2O4_ICSD_2402	insulator	insulator
Cr1K3Mn1O8_ICSD_26803	insulator	insulator
Cr1K3O4_ICSD_108934	insulator	insulator
Cr1K3O8_ICSD_30404	insulator	insulator
Cr1La1O3_ICSD_29120	insulator	metal
Cr1La1O3_ICSD_91270	insulator	insulator
Cr1La1O3_ICSD_91271	insulator	insulator
Cr1La1O4_ICSD_81938	metal	insulator
Cr1La1O6Te1_ICSD_164936	insulator	insulator
Cr1La1S3_ICSD_51123	insulator	insulator
Cr1La1Sb3_ICSD_83907	metal	metal
Cr1La1Se3_ICSD_626229	insulator	insulator
Cr1La3O6S3W1_ICSD_380404	insulator	insulator
Cr1La4S7_ICSD_603686	insulator	insulator
Cr1Li1Mo2O8_ICSD_247056	insulator	insulator
Cr1Li1O2_ICSD_167479	insulator	insulator
Cr1Li1O4Rb1_ICSD_72548	insulator	insulator
Cr1Li1O4Rb1_ICSD_72552	insulator	insulator
Cr1Li1O6Si2_ICSD_55163	insulator	insulator
Cr1Li1O7P2_ICSD_240965	insulator	insulator
Cr1Li1O8W2_ICSD_165780	insulator	insulator
Cr1Li1S2_ICSD_626233	insulator	metal
Cr1Li2O4_ICSD_1972	insulator	insulator
Cr1Lu1O4_ICSD_26988	metal	insulator
Cr1Mg1O4_ICSD_18117	insulator	insulator
Cr1Mg1O4_ICSD_18120	insulator	insulator
Cr1Mn2O9Pb2_ICSD_185692	insulator	insulator
Cr1Mo1O7V1_ICSD_408563	insulator	insulator
Cr1Mo2S4_ICSD_626322	metal	metal
Cr1N1Nb1_ICSD_23779	metal	metal
Cr1N1_ICSD_181079	metal	metal
Cr1N1_ICSD_53146	metal	metal
Cr1N2W1_ICSD_84639	metal	metal
Cr1N3Sr3_ICSD_154803	metal	metal
Cr1N3Th2_ICSD_108340	metal	metal
Cr1N3U2_ICSD_87244	metal	metal
Cr1Na1O2_ICSD_24595	insulator	insulator
Cr1Na1O6Si2_ICSD_9670	insulator	insulator
Cr1Na1O8W2_ICSD_161849	insulator	insulator
Cr1Na1S2_ICSD_660322	insulator	insulator
Cr1Na1Se2_ICSD_42391	insulator	insulator
Cr1Na2O4_ICSD_26330	insulator	insulator
Cr1Na4O4_ICSD_62676	insulator	insulator
Cr1Nb1P1_ICSD_53189	metal	metal
Cr1Nb1Si1_ICSD_42060	metal	metal
Cr1Nb2Se4_ICSD_23476	metal	metal
Cr1Nb2_ICSD_188270	metal	metal
Cr1Nb3_ICSD_188240	metal	metal

Supplementary Table 303. Five-fold cross validated predictions for the metal/insulator classification (295/598).

system	calculated	predicted
Cr1Nb3_ICSD_188252	metal	metal
Cr1Nb4Se8_ICSD_53191	metal	metal
Cr1Nb4Si3_ICSD_601734	metal	metal
Cr1Nd1O3_ICSD_156328	metal	metal
Cr1Nd1O3_ICSD_28933	metal	metal
Cr1Nd1O4_ICSD_90461	metal	metal
Cr1Nd1O4_ICSD_93786	metal	metal
Cr1Nd1O6Te1_ICSD_164938	insulator	insulator
Cr1Nd1S3_ICSD_88976	metal	insulator
Cr1Ni1O4_ICSD_18116	insulator	insulator
Cr1Ni1P1_ICSD_600596	metal	metal
Cr1Ni1P1_ICSD_626435	metal	metal
Cr1Ni1P1_ICSD_626440	metal	metal
Cr1Ni1Sb1_ICSD_182484	metal	metal
Cr1Ni1Si1_ICSD_165255	metal	metal
Cr1Ni2_ICSD_188273	metal	metal
Cr1Ni3_ICSD_188231	metal	metal
Cr1Ni3_ICSD_188243	metal	metal
Cr1O1.01_ICSD_61633	metal	metal
Cr1O2_ICSD_185887	metal	insulator
Cr1O2_ICSD_185888	metal	insulator
Cr1O2_ICSD_186838	insulator	metal
Cr1O2_ICSD_202838	metal	metal
Cr1O2_ICSD_290479	metal	metal
Cr1O3Pb1_ICSD_160196	metal	metal
Cr1O3Pr1_ICSD_28932	metal	metal
Cr1O3Sr1_ICSD_108903	metal	metal
Cr1O3Y1_ICSD_28909	insulator	insulator
Cr1O3_ICSD_16031	insulator	insulator
Cr1O4P1_ICSD_62159	insulator	insulator
Cr1O4Pb1_ICSD_28386	metal	insulator
Cr1O4Pb1_ICSD_91735	insulator	insulator
Cr1O4Pr1_ICSD_26978	metal	metal
Cr1O4Re1_ICSD_173875	metal	insulator
Cr1O4Sr1_ICSD_159266	insulator	insulator
Cr1O4Sr1_ICSD_160793	insulator	insulator
Cr1O4Sr2_ICSD_245595	metal	metal
Cr1O4Tb1_ICSD_169015	insulator	insulator
Cr1O4Tb1_ICSD_26983	insulator	insulator
Cr1O4Tl2_ICSD_247477	insulator	insulator
Cr1O4Tm1_ICSD_51905	metal	insulator
Cr1O4U1_ICSD_15724	metal	insulator
Cr1O4U1_ICSD_15859	insulator	metal
Cr1O4V1_ICSD_36244	insulator	insulator
Cr1O4V1_ICSD_79865	insulator	insulator
Cr1O4W1_ICSD_36213	metal	insulator
Cr1O4W1_ICSD_8269	insulator	insulator
Cr1O4Y1_ICSD_157933	insulator	insulator
Cr1O4Y1_ICSD_157934	insulator	insulator
Cr1O4Yb1_ICSD_26987	insulator	metal

Supplementary Table 304. Five-fold cross validated predictions for the metal/insulator classification (296/598).

system	calculated	predicted
Cr1O5Pb2_ICSD_34831	insulator	insulator
Cr1O5Ti2_ICSD_65219	metal	insulator
Cr1O6Sr2Ta1_ICSD_157544	insulator	insulator
Cr1O6Sr2Zr1_ICSD_181750	metal	insulator
Cr1O6Ta2_ICSD_15306	insulator	insulator
Cr1O6Ta2_ICSD_9390	insulator	insulator
Cr1O6Tb1Te1_ICSD_164942	insulator	insulator
Cr1O6Te1Y1_ICSD_164948	insulator	insulator
Cr1O6Te1Yb1_ICSD_164947	insulator	insulator
Cr1O7Rb1V2_ICSD_245134	insulator	insulator
Cr1P1Pd1_ICSD_626517	metal	metal
Cr1P1S4_ICSD_821	metal	insulator
Cr1P1S4_ICSD_937	insulator	insulator
Cr1P1Se3_ICSD_626521	metal	insulator
Cr1P1Ti1_ICSD_96721	metal	metal
Cr1P1Zr1_ICSD_626529	metal	metal
Cr1P1_ICSD_626502	metal	metal
Cr1P2_ICSD_2526	metal	metal
Cr1P4_ICSD_2790	metal	metal
Cr1Pr2Se4_ICSD_626543	metal	metal
Cr1Pt1Sb1_ICSD_626558	metal	metal
Cr1Pt3_ICSD_102834	metal	metal
Cr1Rh2Sn1_ICSD_626578	metal	metal
Cr1Rh3_ICSD_108348	metal	metal
Cr1S17U8_ICSD_1990	metal	insulator
Cr1S1_ICSD_16723	insulator	metal
Cr1S1_ICSD_49666	metal	metal
Cr1S1_ICSD_626593	metal	metal
Cr1S2Tl1_ICSD_201396	metal	metal
Cr1S3Sb1_ICSD_74601	insulator	insulator
Cr1S6Ta3_ICSD_626633	metal	metal
Cr1Sb1Se3_ICSD_84866	insulator	insulator
Cr1Sb1_ICSD_626689	metal	metal
Cr1Sb2_ICSD_246891	metal	metal
Cr1Sb2_ICSD_42601	metal	metal
Cr1Sb2_ICSD_86811	metal	metal
Cr1Sb3Yb1_ICSD_158115	metal	metal
Cr1Sb5U3_ICSD_658820	metal	metal
Cr1Se1_ICSD_53957	metal	metal
Cr1Se1_ICSD_626721	metal	metal
Cr1Se2Tl1_ICSD_626736	metal	metal
Cr1Se2_ICSD_626718	metal	metal
Cr1Si1Te3_ICSD_626809	insulator	insulator
Cr1Si1Zr1_ICSD_626850	metal	metal
Cr1Si1_ICSD_16837	metal	metal
Cr1Si1_ICSD_182503	metal	metal
Cr1Si2_ICSD_71499	metal	metal
Cr1Si2_ICSD_71502	insulator	metal
Cr1Si2_ICSD_96026	insulator	metal
Cr1Sn2_ICSD_106521	metal	metal

Supplementary Table 305. Five-fold cross validated predictions for the metal/insulator classification (297/598).

system	calculated	predicted
Cr1Te1_ICSD_169766	metal	metal
Cr1Te1_ICSD_169767	metal	metal
Cr1Te1_ICSD_81719	metal	metal
Cr1Te1_ICSD_93159	metal	metal
Cr1Te2Tl1_ICSD_152836	metal	metal
Cr1_ICSD_108326	metal	metal
Cr1_ICSD_165726	metal	metal
Cr1_ICSD_41505	metal	metal
Cr1_ICSD_43526	metal	metal
Cr1_ICSD_625712	metal	metal
Cr1_ICSD_625716	metal	metal
Cr2Cs3I9_ICSD_201965	insulator	insulator
Cr2Cs3I9_ICSD_201966	insulator	insulator
Cr2Cs6O7_ICSD_174316	insulator	insulator
Cr2Cu1O4_ICSD_16708	insulator	insulator
Cr2Cu1O4_ICSD_246081	metal	insulator
Cr2Cu1S4_ICSD_53111	metal	metal
Cr2Cu1S4_ICSD_625765	metal	metal
Cr2Cu1S4_ICSD_625769	metal	metal
Cr2Cu1Se4_ICSD_625807	metal	metal
Cr2Cu1Te4_ICSD_625828	metal	metal
Cr2Dy1Ge2_ICSD_625833	metal	metal
Cr2Dy1Si2_ICSD_625841	metal	metal
Cr2Er1Si2_ICSD_167206	metal	metal
Cr2Er1Si2_ICSD_625852	metal	metal
Cr2F5_ICSD_80639	insulator	insulator
Cr2Fe1K1O8_ICSD_34472	insulator	insulator
Cr2Fe1O4_ICSD_183961	metal	metal
Cr2Fe1O4_ICSD_183967	metal	metal
Cr2Fe1S4_ICSD_43268	metal	metal
Cr2Fe1S4_ICSD_625937	metal	metal
Cr2Fe1S4_ICSD_625941	metal	metal
Cr2Fe1Se4_ICSD_155121	metal	metal
Cr2Fe1Se4_ICSD_43042	insulator	metal
Cr2Fe1_ICSD_188279	metal	metal
Cr2Fe3O12Si3_ICSD_27375	insulator	insulator
Cr2Ga1N1_ICSD_53124	metal	metal
Cr2Ga1S4_ICSD_626049	metal	insulator
Cr2Ga6Hf3_ICSD_600971	metal	metal
Cr2Ga6Zr3_ICSD_600972	metal	metal
Cr2Gd1Si2_ICSD_67472	metal	metal
Cr2Ge2Te6_ICSD_79270	insulator	metal
Cr2Ge2Th1_ICSD_43285	metal	metal
Cr2Ge3Mn3O12_ICSD_262732	insulator	insulator
Cr2H4In1Na1O10_ICSD_172070	insulator	insulator
Cr2H4K2Mg1O10_ICSD_96781	insulator	insulator
Cr2H4Li2O9_ICSD_20207	insulator	insulator
Cr2Hf1_ICSD_109213	metal	metal
Cr2Hf1_ICSD_626144	metal	metal
Cr2Hf1_ICSD_626155	metal	metal

Supplementary Table 306. Five-fold cross validated predictions for the metal/insulator classification (298/598).

system	calculated	predicted
Cr2Hg1O4_ICSD_245275	insulator	insulator
Cr2Hg1S4_ICSD_626162	insulator	insulator
Cr2Hg1Se4_ICSD_600792	metal	insulator
Cr2Ho1Si2_ICSD_67474	metal	metal
Cr2Ho1Si2_ICSD_90408	metal	metal
Cr2K2O7_ICSD_15824	insulator	insulator
Cr2K2O7_ICSD_280204	insulator	insulator
Cr2K3Na1O8_ICSD_74557	insulator	insulator
Cr2K3Na1O8_ICSD_74559	insulator	insulator
Cr2Li14N8O1_ICSD_151435	insulator	insulator
Cr2Li2N8O1Sr6_ICSD_411983	insulator	insulator
Cr2Mg1O4_ICSD_160954	insulator	insulator
Cr2Mg1O4_ICSD_290589	insulator	insulator
Cr2Mg3O12Si3_ICSD_27371	insulator	insulator
Cr2Mn1O4_ICSD_167400	insulator	insulator
Cr2Mn1S4_ICSD_164401	insulator	metal
Cr2Mn3O12Si3_ICSD_77436	insulator	insulator
Cr2N1_ICSD_67400	metal	metal
Cr2Nb1_ICSD_188264	metal	metal
Cr2Nb1_ICSD_626379	metal	metal
Cr2Nb1_ICSD_626381	metal	metal
Cr2Nb2Se10_ICSD_35044	metal	metal
Cr2Nb4Si5_ICSD_76064	metal	metal
Cr2Nd1Si2_ICSD_108343	metal	metal
Cr2Ni1O4_ICSD_280061	insulator	insulator
Cr2Ni1O4_ICSD_280062	insulator	insulator
Cr2Ni1O4_ICSD_37427	metal	insulator
Cr2Ni1S4_ICSD_16884	metal	metal
Cr2Ni1_ICSD_188278	metal	metal
Cr2O11Te4_ICSD_1	insulator	insulator
Cr2O3_ICSD_167280	insulator	metal
Cr2O4Si1_ICSD_75639	insulator	insulator
Cr2O4Si1_ICSD_89073	insulator	insulator
Cr2O4Zn1_ICSD_290591	insulator	insulator
Cr2O4Zn1_ICSD_290592	insulator	insulator
Cr2O6Re1_ICSD_173874	insulator	insulator
Cr2O6Te1_ICSD_24794	insulator	insulator
Cr2O6U1_ICSD_274	insulator	insulator
Cr2O6W1_ICSD_20314	insulator	insulator
Cr2O7Rb2_ICSD_15034	insulator	insulator
Cr2O7Rb2_ICSD_409745	insulator	insulator
Cr2O7Tl2_ICSD_413950	insulator	insulator
Cr2O8Sr3_ICSD_85055	metal	insulator
Cr2P1_ICSD_82148	metal	metal
Cr2Pu1Si2_ICSD_106959	metal	metal
Cr2Pu1Si2_ICSD_602707	metal	metal
Cr2S3_ICSD_16720	metal	metal
Cr2S3_ICSD_626604	metal	metal
Cr2S4Ti1_ICSD_626640	insulator	metal
Cr2S4Zn1_ICSD_164168	insulator	insulator

Supplementary Table 307. Five-fold cross validated predictions for the metal/insulator classification (299/598).

system	calculated	predicted
Cr2S8Tl1V3_ICSD_74296	insulator	insulator
Cr2Se3_ICSD_626703	metal	metal
Cr2Se3_ICSD_626708	metal	metal
Cr2Se4Zn1_ICSD_626751	insulator	metal
Cr2Si2Sm1_ICSD_626799	metal	metal
Cr2Si2Tb1_ICSD_167205	metal	metal
Cr2Si2Tb1_ICSD_67473	metal	metal
Cr2Si2Th1_ICSD_181877	metal	metal
Cr2Si2Th1_ICSD_184657	metal	metal
Cr2Si2U1_ICSD_626828	metal	metal
Cr2Si2Y1_ICSD_626847	metal	metal
Cr2Si2Yb1_ICSD_626849	metal	metal
Cr2Ta1_ICSD_185190	metal	metal
Cr2Ta1_ICSD_626867	metal	metal
Cr2Te3_ICSD_15039	metal	metal
Cr2Te4Ti1_ICSD_626896	metal	metal
Cr2Te4V1_ICSD_626901	metal	metal
Cr2Ti1_ICSD_102851	metal	metal
Cr2Ti1_ICSD_102853	metal	metal
Cr2Ti1_ICSD_626909	metal	metal
Cr2Zr1_ICSD_106984	metal	metal
Cr2Zr1_ICSD_626933	metal	metal
Cr2Zr1_ICSD_626938	metal	metal
Cr2Zr1_ICSD_626940	metal	metal
Cr2Zr1_ICSD_626943	metal	metal
Cr2Zr1_ICSD_626944	metal	metal
Cr2Zr1_ICSD_626951	metal	metal
Cr2Zr1_ICSD_626956	metal	metal
Cr3Cu1O8_ICSD_155506	insulator	insulator
Cr3Fe1_ICSD_188249	metal	metal
Cr3Ga1N1_ICSD_53125	metal	metal
Cr3Ga1_ICSD_626025	metal	metal
Cr3Ge1N1_ICSD_25751	metal	metal
Cr3Ge1_ICSD_186002	metal	metal
Cr3H1O8_ICSD_156386	metal	insulator
Cr3H6K1O14S2_ICSD_75977	insulator	insulator
Cr3In1O8_ICSD_155504	insulator	insulator
Cr3Ir1N1_ICSD_53132	metal	metal
Cr3Ir1_ICSD_102780	metal	metal
Cr3K1O8_ICSD_15921	insulator	insulator
Cr3Li1O8_ICSD_155851	insulator	insulator
Cr3N1Nb3_ICSD_626345	metal	metal
Cr3N1P1_ICSD_626346	metal	metal
Cr3N1Pd1_ICSD_53141	metal	metal
Cr3N1Pt1_ICSD_53142	metal	metal
Cr3N1Rh1_ICSD_53143	metal	metal
Cr3N1Sn1_ICSD_53145	metal	metal
Cr3N1Ta3_ICSD_626352	metal	metal
Cr3Na1O8_ICSD_82620	insulator	insulator
Cr3Nb1_ICSD_188235	metal	metal

Supplementary Table 308. Five-fold cross validated predictions for the metal/insulator classification (300/598).

system	calculated	predicted
Cr3Nb1_ICSD_188247	metal	metal
Cr3Nb1_ICSD_188259	metal	metal
Cr3Ni1_ICSD_188260	metal	metal
Cr3Ni2Si1_ICSD_53197	metal	metal
Cr3O12Tl2_ICSD_201793	insulator	insulator
Cr3O1_ICSD_15904	metal	metal
Cr3O8P2_ICSD_261640	metal	insulator
Cr3O8Rb1_ICSD_82622	insulator	insulator
Cr3O8Tl1_ICSD_155505	insulator	insulator
Cr3O8_ICSD_155847	insulator	insulator
Cr3Os1_ICSD_102829	metal	metal
Cr3P1_ICSD_23560	metal	metal
Cr3Pt1_ICSD_102836	metal	metal
Cr3Pt1_ICSD_102837	metal	metal
Cr3Rb1S5_ICSD_73143	insulator	insulator
Cr3Rh1_ICSD_626570	metal	metal
Cr3Ru1_ICSD_659684	metal	metal
Cr3S4_ICSD_81886	metal	metal
Cr3S5Tl1_ICSD_23632	insulator	insulator
Cr3Se4_ICSD_601348	metal	metal
Cr3Si1_ICSD_186001	metal	metal
Cr3Si5_ICSD_626782	metal	metal
Cr3Te4_ICSD_626873	metal	metal
Cr4Cs2Cu1H4O16_ICSD_156678	insulator	insulator
Cr4Cu1Fe1S8_ICSD_53108	insulator	metal
Cr4Cu1H4O16Rb2_ICSD_156677	metal	insulator
Cr4Cu1In1Se8_ICSD_250246	metal	insulator
Cr4Fe8Y1_ICSD_168240	metal	metal
Cr4H4K2O16Zn1_ICSD_96785	insulator	insulator
Cr4Nb2Si5_ICSD_42905	metal	metal
Cr4Nb2Si5_ICSD_626410	metal	metal
Cr4Si5Zr2_ICSD_99577	metal	metal
Cr5Cs1S8_ICSD_2566	insulator	insulator
Cr5Cs1Te8_ICSD_602370	metal	metal
Cr5Ge3_ICSD_626088	metal	metal
Cr5In1Se8_ICSD_601719	insulator	insulator
Cr5K1S8_ICSD_2568	insulator	insulator
Cr5K1Se8_ICSD_602350	insulator	insulator
Cr5K1Te8_ICSD_602368	metal	metal
Cr5Na1Se8_ICSD_602328	insulator	insulator
Cr5P3Zr1_ICSD_409488	metal	metal
Cr5Rb1S8_ICSD_2567	insulator	insulator
Cr5Rb1Se8_ICSD_602314	insulator	insulator
Cr5Rb1Te8_ICSD_602369	metal	metal
Cr5S6_ICSD_43043	metal	metal
Cr5S8Tl1_ICSD_40821	insulator	insulator
Cr5S8_ICSD_626594	metal	metal
Cr5Se8Tl1_ICSD_88229	insulator	insulator
Cr5Se8_ICSD_626705	metal	metal
Cr5Si3_ICSD_15683	metal	metal

Supplementary Table 309. Five-fold cross validated predictions for the metal/insulator classification (301/598).

system	calculated	predicted
Cr6Gd1Ge6_ICSD_603291	metal	metal
Cr6Ge6Ho1_ICSD_83999	metal	metal
Cr6Ge6Sc1_ICSD_626107	metal	metal
Cr6Ni16Si7_ICSD_53198	metal	metal
Cr7Nb6_ICSD_188282	metal	metal
Cr8K2O16_ICSD_183641	metal	metal
Cr8K2O16_ICSD_491	metal	metal
Cr8O16Rb2_ICSD_100521	metal	metal
Cr8O21_ICSD_155849	insulator	insulator
Cs0.33Mo1O3_ICSD_65418	insulator	insulator
Cs18O6Tl8_ICSD_421376	metal	metal
Cs1Cu1F4_ICSD_35264	insulator	insulator
Cs1Cu1La2Se4_ICSD_93680	insulator	insulator
Cs1Cu1O1_ICSD_40161	insulator	insulator
Cs1Cu1O2_ICSD_15097	insulator	insulator
Cs1Cu1S4_ICSD_402075	insulator	insulator
Cs1Cu1Se4_ICSD_75195	insulator	insulator
Cs1Cu2I3_ICSD_150305	insulator	insulator
Cs1Cu3Er2Se5_ICSD_413675	insulator	insulator
Cs1Cu3O2_ICSD_413342	insulator	insulator
Cs1Cu3S2_ICSD_23326	insulator	insulator
Cs1Cu4S3_ICSD_23325	metal	metal
Cs1Cu4Se3_ICSD_75196	metal	metal
Cs1Dy1S2_ICSD_73540	insulator	insulator
Cs1Dy1Te3Zn1_ICSD_170189	insulator	insulator
Cs1Er1S2_ICSD_602875	insulator	insulator
Cs1Er1S2_ICSD_73542	insulator	insulator
Cs1Er1Te3Zn1_ICSD_170192	insulator	insulator
Cs1Eu1F3_ICSD_49577	insulator	insulator
Cs1Eu2I5_ICSD_41099	insulator	insulator
Cs1F1O2S1_ICSD_93076	insulator	insulator
Cs1F1O3S1_ICSD_82520	insulator	insulator
Cs1F1_ICSD_44288	insulator	insulator
Cs1F1_ICSD_53832	insulator	insulator
Cs1F1_ICSD_61563	insulator	insulator
Cs1F2H1O6S2_ICSD_82521	insulator	insulator
Cs1F2H1_ICSD_45858	insulator	insulator
Cs1F2Li1_ICSD_18020	insulator	insulator
Cs1F2Li1_ICSD_245965	insulator	insulator
Cs1F2N1O4S2_ICSD_81866	insulator	insulator
Cs1F2O2P1_ICSD_16875	insulator	insulator
Cs1F3Hg1_ICSD_15168	insulator	insulator
Cs1F3Mg1_ICSD_49584	insulator	insulator
Cs1F3Mn1_ICSD_31765	insulator	insulator
Cs1F3Mo1O2_ICSD_9710	insulator	insulator
Cs1F3Ni1_ICSD_15092	insulator	insulator
Cs1F3Ni1_ICSD_61278	insulator	insulator
Cs1F3O1V1_ICSD_249402	insulator	insulator
Cs1F3Pb1_ICSD_93438	insulator	insulator
Cs1F3Pb1_ICSD_93439	insulator	insulator

Supplementary Table 310. Five-fold cross validated predictions for the metal/insulator classification (302/598).

system	calculated	predicted
Cs1F3Sr1_ICSD_49578	insulator	insulator
Cs1F3Yb1_ICSD_49579	insulator	insulator
Cs1F4Fe1_ICSD_6043	insulator	insulator
Cs1F4Li3_ICSD_245968	insulator	insulator
Cs1F4Mn1_ICSD_14104	metal	insulator
Cs1F4Mn1_ICSD_72396	insulator	insulator
Cs1F4Ti1_ICSD_27230	insulator	insulator
Cs1F4V1_ICSD_167354	insulator	insulator
Cs1F5Pd1Rb2_ICSD_35286	insulator	insulator
Cs1F5Pd2_ICSD_35284	insulator	insulator
Cs1F5Tb1_ICSD_59235	metal	insulator
Cs1F5Te1_ICSD_200252	insulator	insulator
Cs1F6Nb1_ICSD_183851	insulator	insulator
Cs1F6Re1_ICSD_201511	insulator	insulator
Cs1F6Ru1_ICSD_28694	insulator	insulator
Cs1F6Sb1_ICSD_201886	insulator	insulator
Cs1F7Mo1_ICSD_78390	insulator	insulator
Cs1F7Rb2Si1_ICSD_9592	insulator	insulator
Cs1F7Sb2_ICSD_14119	insulator	insulator
Cs1F7W1_ICSD_78391	insulator	insulator
Cs1F7Xe1_ICSD_404986	insulator	insulator
Cs1F8Re1_ICSD_410829	insulator	insulator
Cs1Fe1I4_ICSD_66811	insulator	insulator
Cs1Fe1O2_ICSD_180532	insulator	insulator
Cs1Fe1O2_ICSD_421173	insulator	insulator
Cs1Fe1O8S2_ICSD_245667	insulator	insulator
Cs1Fe2Se3_ICSD_81549	metal	metal
Cs1Ga1H1O10P3_ICSD_409692	insulator	insulator
Cs1Ga1H4_ICSD_169711	insulator	insulator
Cs1Ga1H4_ICSD_169712	insulator	insulator
Cs1Ga1S2_ICSD_41888	insulator	insulator
Cs1Ga1S3_ICSD_281723	insulator	insulator
Cs1Ga3_ICSD_102863	metal	metal
Cs1Gd1S2_ICSD_602861	insulator	insulator
Cs1Gd1S2_ICSD_73538	insulator	insulator
Cs1Ge1I3_ICSD_62559	insulator	insulator
Cs1Ge1S5Ta1_ICSD_417875	insulator	insulator
Cs1Ge1_ICSD_43517	insulator	insulator
Cs1H12Mg1O10P1_ICSD_281563	insulator	insulator
Cs1H1Mn1O10P3_ICSD_51089	insulator	insulator
Cs1H1O3S1_ICSD_56615	insulator	insulator
Cs1H1O3Se1_ICSD_202718	insulator	insulator
Cs1H1O4S1_ICSD_91808	insulator	insulator
Cs1H1O4Se1_ICSD_290645	insulator	insulator
Cs1H1_ICSD_53235	insulator	insulator
Cs1H1_ICSD_53236	insulator	insulator
Cs1H2O4P1_ICSD_200895	insulator	insulator
Cs1H3Mg1_ICSD_162260	insulator	insulator
Cs1H3O2_ICSD_280909	insulator	insulator
Cs1H4Li1N2_ICSD_62654	insulator	insulator

Supplementary Table 311. Five-fold cross validated predictions for the metal/insulator classification (303/598).

system	calculated	predicted
Cs1H4Li1O5S1_ICSD_200780	insulator	insulator
Cs1H6Li2N3_ICSD_65151	insulator	insulator
Cs1Hg1La1Se3_ICSD_281441	insulator	insulator
Cs1Hg1N3O6_ICSD_28645	metal	metal
Cs1Hg1Nd1Se3_ICSD_281444	insulator	insulator
Cs1Hg1Se3Y1_ICSD_281440	insulator	insulator
Cs1Ho1K2O8P2_ICSD_64760	insulator	insulator
Cs1Ho1S2_ICSD_73541	insulator	insulator
Cs1Ho1Te3Zn1_ICSD_170191	insulator	insulator
Cs1I14Zr6_ICSD_35146	metal	insulator
Cs1I1O3_ICSD_33665	metal	insulator
Cs1I1O3_ICSD_61009	insulator	insulator
Cs1I1O4_ICSD_83375	insulator	insulator
Cs1I1_ICSD_53835	insulator	insulator
Cs1I1_ICSD_61517	insulator	insulator
Cs1I1_ICSD_9204	insulator	insulator
Cs1I3Mg1_ICSD_87262	insulator	insulator
Cs1I3Mn1_ICSD_15950	insulator	insulator
Cs1I3Pb1_ICSD_161481	insulator	insulator
Cs1I3Sn1_ICSD_14070	insulator	insulator
Cs1I3Sn1_ICSD_69995	insulator	insulator
Cs1I3Sn1_ICSD_69997	insulator	insulator
Cs1I3Ti1_ICSD_154258	insulator	insulator
Cs1I3V1_ICSD_26454	insulator	insulator
Cs1I4Li3_ICSD_245989	insulator	insulator
Cs1I4Tl1_ICSD_59110	insulator	insulator
Cs1In3O5_ICSD_23630	insulator	insulator
Cs1In3_ICSD_102867	metal	metal
Cs1K2O8P2Sc1_ICSD_61787	insulator	insulator
Cs1K2O8P2Yb1_ICSD_280936	insulator	insulator
Cs1K2Sb1_ICSD_53237	insulator	insulator
Cs1K2_ICSD_102870	metal	metal
Cs1La1Nb2O7_ICSD_82378	insulator	insulator
Cs1La1Te3Zn1_ICSD_170184	insulator	insulator
Cs1Li1Mo1O4_ICSD_20805	insulator	insulator
Cs1Li1O4S1_ICSD_63181	insulator	insulator
Cs1Li1O4S1_ICSD_63182	insulator	insulator
Cs1Li1O4S1_ICSD_88900	insulator	insulator
Cs1Lu1S2_ICSD_73545	insulator	insulator
Cs1Mg1O4P1_ICSD_260423	insulator	insulator
Cs1Mn1O4_ICSD_16819	insulator	insulator
Cs1Mn1P1_ICSD_627022	metal	metal
Cs1Mn1Sb1_ICSD_41869	metal	metal
Cs1Mo3S3_ICSD_627033	metal	metal
Cs1Mo3Se3_ICSD_604515	metal	metal
Cs1Mo3Te3_ICSD_603673	insulator	metal
Cs1Mo6Se7_ICSD_655517	metal	metal
Cs1N1O2_ICSD_29106	metal	insulator
Cs1N1O2_ICSD_50328	insulator	insulator
Cs1N1O3_ICSD_16339	insulator	insulator

Supplementary Table 312. Five-fold cross validated predictions for the metal/insulator classification (304/598).

system	calculated	predicted
Cs1N1O3_ICSD_35555	insulator	insulator
Cs1N2Nb1_ICSD_72546	insulator	insulator
Cs1N3O4S2_ICSD_281172	insulator	insulator
Cs1N3_ICSD_627047	insulator	insulator
Cs1Na1O3Ti1_ICSD_78752	insulator	insulator
Cs1Na1S1_ICSD_41323	insulator	insulator
Cs1Na1Se1_ICSD_41322	insulator	insulator
Cs1Na1Te1_ICSD_405538	insulator	insulator
Cs1Na2_ICSD_627052	metal	metal
Cs1Nb1O3_ICSD_1266	insulator	insulator
Cs1Nb3O10Sr2_ICSD_93675	insulator	insulator
Cs1Nd1O2_ICSD_27336	insulator	insulator
Cs1O10Sr2Ta3_ICSD_93677	insulator	insulator
Cs1O2Sb1_ICSD_59329	insulator	insulator
Cs1O2Y1_ICSD_49652	insulator	insulator
Cs1O2_ICSD_627062	metal	metal
Cs1O3V1_ICSD_1489	insulator	insulator
Cs1O3_ICSD_33847	insulator	insulator
Cs1O4P1Zn1_ICSD_85456	insulator	insulator
Cs1O4P1Zn1_ICSD_85457	insulator	insulator
Cs1O4Re1_ICSD_72817	insulator	insulator
Cs1O4Re1_ICSD_74505	insulator	insulator
Cs1O5V2_ICSD_850	insulator	insulator
Cs1O6Os2_ICSD_246507	metal	metal
Cs1O6W2_ICSD_72634	metal	insulator
Cs1O8S2V1_ICSD_73736	insulator	insulator
Cs1O8V3_ICSD_50010	insulator	insulator
Cs1P1Pb1S4_ICSD_409821	insulator	insulator
Cs1P1Pb1Se4_ICSD_165348	insulator	insulator
Cs1P2S7V1_ICSD_59910	insulator	insulator
Cs1P3Zn4_ICSD_262031	insulator	insulator
Cs1Pr1S2_ICSD_73548	insulator	insulator
Cs1S2Sb1_ICSD_200798	insulator	insulator
Cs1S2Sm1_ICSD_73536	metal	insulator
Cs1S2Tb1_ICSD_602889	insulator	insulator
Cs1S2Tb1_ICSD_73539	insulator	insulator
Cs1S2Yb1_ICSD_603271	metal	metal
Cs1S2Yb1_ICSD_73544	metal	metal
Cs1S6Sb1_ICSD_67977	insulator	insulator
Cs1Sb1_ICSD_14031	insulator	insulator
Cs1Sb1_ICSD_412154	insulator	insulator
Cs1Sb2Se4_ICSD_61220	insulator	insulator
Cs1Se2Yb1_ICSD_59913	metal	insulator
Cs1Si1_ICSD_43520	insulator	insulator
Cs1Si1_ICSD_627104	insulator	insulator
Cs1Te4_ICSD_47182	insulator	insulator
Cs1Te5Ti1U1_ICSD_79885	metal	insulator
Cs1Te8Ti5_ICSD_416704	metal	metal
Cs1Tl1_ICSD_165344	metal	metal
Cs1_ICSD_109020	metal	metal

Supplementary Table 313. Five-fold cross validated predictions for the metal/insulator classification (305/598).

system	calculated	predicted
Cs1_ICSD_109021	metal	metal
Cs1_ICSD_109022	metal	metal
Cs1_ICSD_173928	metal	metal
Cs1_ICSD_173929	metal	metal
Cs1_ICSD_173930	metal	metal
Cs1_ICSD_53232	metal	metal
Cs1_ICSD_76941	metal	metal
Cs2.67O16Te5.33_ICSD_261688	metal	insulator
Cs2Cu1F6K1_ICSD_59102	insulator	insulator
Cs2Cu1F6_ICSD_65259	insulator	insulator
Cs2Cu1N6O12Pb1_ICSD_2381	metal	metal
Cs2Cu1N6O12Pb1_ICSD_8284	metal	metal
Cs2Cu1O2_ICSD_39535	metal	insulator
Cs2Cu2Sb2Se5_ICSD_88681	insulator	insulator
Cs2Cu5Se4_ICSD_54101	metal	insulator
Cs2Er1F6Na1_ICSD_23143	insulator	insulator
Cs2F10O1Sb2_ICSD_21016	insulator	insulator
Cs2F1O3P1_ICSD_172345	insulator	insulator
Cs2F4Hg1_ICSD_72353	insulator	insulator
Cs2F5Mn1_ICSD_69675	insulator	insulator
Cs2F6Fe1K1_ICSD_42144	insulator	insulator
Cs2F6Fe1Na1_ICSD_65503	insulator	insulator
Cs2F6Fe1Tl1_ICSD_6036	insulator	insulator
Cs2F6Ga1Li1_ICSD_9004	insulator	insulator
Cs2F6Ga1Na1_ICSD_55698	insulator	insulator
Cs2F6Ge1_ICSD_35547	insulator	insulator
Cs2F6Hf1_ICSD_25600	insulator	insulator
Cs2F6In1Na1_ICSD_24921	insulator	insulator
Cs2F6K1Mn1_ICSD_91036	insulator	insulator
Cs2F6K1Mn1_ICSD_91037	insulator	insulator
Cs2F6K1Mo1_ICSD_4053	insulator	insulator
Cs2F6K1Rh1_ICSD_4056	insulator	insulator
Cs2F6K1Ti1_ICSD_67146	insulator	insulator
Cs2F6K1V1_ICSD_27344	insulator	insulator
Cs2F6K1Y1_ICSD_25367	insulator	insulator
Cs2F6Mn1Na1_ICSD_37005	metal	insulator
Cs2F6Mn1_ICSD_47201	insulator	insulator
Cs2F6Mn1_ICSD_76272	insulator	insulator
Cs2F6Mo1Tl1_ICSD_15772	insulator	insulator
Cs2F6Na1Sc1_ICSD_22116	insulator	insulator
Cs2F6Na1Tl1_ICSD_22118	insulator	insulator
Cs2F6Na1Y1_ICSD_25368	insulator	insulator
Cs2F6Nb1_ICSD_72832	insulator	insulator
Cs2F6Rb1Y1_ICSD_25366	insulator	insulator
Cs2F6Si1_ICSD_26871	insulator	insulator
Cs2F6Si1_ICSD_38548	insulator	insulator
Cs2F6Sn1_ICSD_281	insulator	insulator
Cs2F6Tl1V1_ICSD_27343	insulator	insulator
Cs2F6Zr1_ICSD_25598	insulator	insulator
Cs2Fe2K4O5_ICSD_65942	insulator	insulator

Supplementary Table 314. Five-fold cross validated predictions for the metal/insulator classification (306/598).

system	calculated	predicted
Cs2Ga1Li3O4_ICSD_69491	insulator	insulator
Cs2Ge1Li2O4_ICSD_36532	insulator	insulator
Cs2Ge1Te4_ICSD_78830	insulator	insulator
Cs2Ge2Se5_ICSD_411030	insulator	insulator
Cs2Ge3O9Sn1_ICSD_19031	insulator	insulator
Cs2Ge3O9Ti1_ICSD_19030	insulator	insulator
Cs2Ge4Na2_ICSD_409262	metal	insulator
Cs2H12N6Sn1_ICSD_79987	insulator	insulator
Cs2H4Mg1_ICSD_162261	insulator	insulator
Cs2H6N2P4_ICSD_413072	insulator	insulator
Cs2H8O10Sb2_ICSD_422768	insulator	insulator
Cs2Hf1I6_ICSD_201308	insulator	metal
Cs2Hg1I4_ICSD_63110	insulator	insulator
Cs2Hg1I4_ICSD_9361	insulator	insulator
Cs2Hg1O2_ICSD_25513	insulator	insulator
Cs2Hg27_ICSD_418849	metal	metal
Cs2Hg3I8_ICSD_4074	insulator	insulator
Cs2Hg3S8Sn2_ICSD_85598	insulator	insulator
Cs2Hg6S7_ICSD_260060	insulator	insulator
Cs2I4O11_ICSD_413942	insulator	insulator
Cs2I4Zn1_ICSD_82932	insulator	insulator
Cs2I5Li3_ICSD_35655	insulator	insulator
Cs2I6O18Zr1_ICSD_413881	insulator	insulator
Cs2I6Pd1_ICSD_280189	insulator	insulator
Cs2I6Pd1_ICSD_280190	insulator	insulator
Cs2I6Pt1_ICSD_37193	insulator	insulator
Cs2I6Sn1_ICSD_22105	metal	insulator
Cs2I6Te1_ICSD_38105	insulator	insulator
Cs2I8_ICSD_44621	insulator	insulator
Cs2K1O14Si4U2_ICSD_249936	insulator	insulator
Cs2Li1Mn1O4_ICSD_72361	insulator	insulator
Cs2Li1O4V1_ICSD_40219	insulator	insulator
Cs2Li1S4V1_ICSD_414186	insulator	insulator
Cs2Li2O4Si1_ICSD_33809	insulator	insulator
Cs2Li2O4Ti1_ICSD_33810	insulator	insulator
Cs2Mn1O4_ICSD_39503	insulator	insulator
Cs2Mn1S2_ICSD_65455	insulator	insulator
Cs2Mn1S6Te2_ICSD_165378	insulator	insulator
Cs2Mn1Se2_ICSD_65458	insulator	insulator
Cs2Mo1O2S2_ICSD_423982	insulator	insulator
Cs2N2Tb6Te7_ICSD_419405	insulator	insulator
Cs2Na1O4V1_ICSD_65963	insulator	insulator
Cs2Ni1O2_ICSD_423923	insulator	insulator
Cs2Ni1P2S6_ICSD_93485	insulator	insulator
Cs2O12Sr1V4_ICSD_250105	insulator	insulator
Cs2O12Te4_ICSD_59168	insulator	insulator
Cs2O13V5_ICSD_849	metal	insulator
Cs2O1_ICSD_27919	insulator	insulator
Cs2O2Pb1_ICSD_2268	insulator	insulator
Cs2O2Zn1_ICSD_2378	insulator	insulator

Supplementary Table 315. Five-fold cross validated predictions for the metal/insulator classification (307/598).

system	calculated	predicted
Cs2O2_ICSD_25529	insulator	insulator
Cs2O3Pb1_ICSD_2908	insulator	insulator
Cs2O3Pb1_ICSD_62140	insulator	insulator
Cs2O3Pr1_ICSD_1181	insulator	insulator
Cs2O3Sn2_ICSD_24392	insulator	insulator
Cs2O3Te1_ICSD_59164	insulator	insulator
Cs2O3Zr1_ICSD_67345	insulator	insulator
Cs2O3_ICSD_627061	metal	insulator
Cs2O4Ru1_ICSD_33799	insulator	insulator
Cs2O4U1_ICSD_20581	insulator	insulator
Cs2O6S2_ICSD_200865	insulator	insulator
Cs2O7P2Sr1_ICSD_39507	insulator	insulator
Cs2O7U2_ICSD_72588	insulator	insulator
Cs2O8S2_ICSD_26726	insulator	insulator
Cs2O9Si3Zr1_ICSD_93133	insulator	insulator
Cs2P2Pt1_ICSD_658699	insulator	insulator
Cs2P2S6_ICSD_33277	insulator	insulator
Cs2P2Si1_ICSD_71224	insulator	insulator
Cs2Pd3S4_ICSD_26250	insulator	insulator
Cs2Pd3Se4_ICSD_33892	insulator	insulator
Cs2Pt1Te2_ICSD_627076	insulator	insulator
Cs2Pt1_ICSD_413241	insulator	metal
Cs2S1_ICSD_183207	insulator	insulator
Cs2S2_ICSD_200474	insulator	insulator
Cs2S3Ti1_ICSD_49739	insulator	insulator
Cs2S3_ICSD_14094	insulator	insulator
Cs2S5_ICSD_201073	insulator	insulator
Cs2S6Sn2_ICSD_73008	insulator	insulator
Cs2S6_ICSD_24678	insulator	insulator
Cs2S7Sb4_ICSD_2193	insulator	insulator
Cs2S8Sb4_ICSD_67976	insulator	insulator
Cs2Se1_ICSD_41687	insulator	insulator
Cs2Se3Zr1_ICSD_409294	insulator	insulator
Cs2Se3_ICSD_14095	insulator	insulator
Cs2Se4W1_ICSD_627102	insulator	insulator
Cs2Se5_ICSD_60087	insulator	insulator
Cs2Se6Sn2_ICSD_402842	insulator	insulator
Cs2Se6Te2_ICSD_84993	insulator	insulator
Cs2Si2Te6_ICSD_61397	insulator	insulator
Cs2Sn1Te4_ICSD_74826	insulator	insulator
Cs2Te1_ICSD_36501	insulator	insulator
Cs2Te2_ICSD_83351	insulator	insulator
Cs2Te3_ICSD_53245	insulator	insulator
Cs2Te5_ICSD_36017	insulator	insulator
Cs3Cu8Se6_ICSD_14012	metal	metal
Cs3Er2I9_ICSD_300232	insulator	insulator
Cs3F3H12N4_ICSD_421716	insulator	insulator
Cs3F3Mo2O6_ICSD_200691	insulator	insulator
Cs3F4Li1_ICSD_245962	insulator	insulator
Cs3F5Li2_ICSD_245964	insulator	insulator

Supplementary Table 316. Five-fold cross validated predictions for the metal/insulator classification (308/598).

system	calculated	predicted
Cs3F6Tl1_ICSD_19076	insulator	insulator
Cs3F6Y1_ICSD_19078	insulator	insulator
Cs3F7Ge1_ICSD_202917	insulator	insulator
Cs3F7Mn1_ICSD_9598	insulator	insulator
Cs3F7Ni1_ICSD_9600	insulator	insulator
Cs3F7Si1_ICSD_9588	insulator	insulator
Cs3F7Ti1_ICSD_9594	insulator	insulator
Cs3Fe1S3_ICSD_71991	insulator	insulator
Cs3H1O8Se2_ICSD_40439	insulator	insulator
Cs3Hg20_ICSD_240038	metal	metal
Cs3I9Nb2_ICSD_421295	insulator	insulator
Cs3I9Sb2_ICSD_84989	insulator	insulator
Cs3I9Sb2_ICSD_89695	insulator	insulator
Cs3I9Y2_ICSD_26564	insulator	insulator
Cs3I9Zr2_ICSD_26565	insulator	insulator
Cs3N11P6_ICSD_51397	insulator	insulator
Cs3Ni1O2_ICSD_424578	insulator	insulator
Cs3O1_ICSD_15695	metal	insulator
Cs3O3Sb1_ICSD_279580	insulator	insulator
Cs3O4P1_ICSD_161678	insulator	insulator
Cs3O4Sb1_ICSD_411211	insulator	insulator
Cs3O8Ta1_ICSD_30408	insulator	insulator
Cs3P1Se4_ICSD_415021	insulator	insulator
Cs3P7_ICSD_62259	insulator	insulator
Cs3S4Sb1_ICSD_409619	insulator	insulator
Cs3Sb1Se3_ICSD_89609	insulator	insulator
Cs3Sb1Se4_ICSD_404083	insulator	insulator
Cs3Sb1_ICSD_53243	insulator	metal
Cs4F10Mg3_ICSD_16084	insulator	insulator
Cs4Fe1O3_ICSD_423336	insulator	insulator
Cs4Fe1O4_ICSD_174309	insulator	insulator
Cs4Ge2S8_ICSD_281352	insulator	insulator
Cs4Ge2Se6_ICSD_415215	insulator	insulator
Cs4Ge2Se8_ICSD_74832	insulator	insulator
Cs4Ge4S10_ICSD_409455	insulator	insulator
Cs4H8O4P2Se6_ICSD_260478	insulator	insulator
Cs4I13Os1Pr6_ICSD_81475	metal	insulator
Cs4Ir1O4_ICSD_72287	insulator	insulator
Cs4O3Pb1_ICSD_65246	insulator	insulator
Cs4O4Pb1_ICSD_16363	insulator	insulator
Cs4O4Sn1_ICSD_65970	insulator	insulator
Cs4O4Zr1_ICSD_65160	insulator	insulator
Cs4O8P4S4_ICSD_20543	insulator	insulator
Cs4P2Pd1Se8_ICSD_165313	insulator	insulator
Cs4P2Se10_ICSD_418434	insulator	insulator
Cs4P2Se9_ICSD_81298	insulator	insulator
Cs4P6_ICSD_65185	insulator	insulator
Cs4S6Si2_ICSD_409176	insulator	insulator
Cs4Sb2_ICSD_92474	metal	insulator
Cs4Se10Si4_ICSD_409816	insulator	insulator

Supplementary Table 317. Five-fold cross validated predictions for the metal/insulator classification (309/598).

system	calculated	predicted
Cs4Se4Si1_ICSD_409480	insulator	insulator
Cs4Se6Sn2_ICSD_67251	insulator	insulator
Cs4Se8Si2_ICSD_409815	insulator	insulator
Cs5Ge1P3_ICSD_65717	metal	metal
Cs5Hg19_ICSD_240040	metal	metal
Cs5P3Si1_ICSD_65715	metal	insulator
Cs5Te3_ICSD_34000	insulator	insulator
Cs6Fe2O5_ICSD_174313	insulator	insulator
Cs6Fe2O5_ICSD_73134	insulator	insulator
Cs6Ga1K3Sb4_ICSD_300150	insulator	insulator
Cs6Ga2Se6_ICSD_35293	insulator	insulator
Cs6Ge2Se6_ICSD_409512	insulator	insulator
Cs6I8Re6S8_ICSD_88610	insulator	insulator
Cs6In2O6_ICSD_62644	metal	insulator
Cs6Ni1O8Si2_ICSD_409506	insulator	insulator
Cs6O7Si2_ICSD_411665	insulator	insulator
Cs6P2Se14Sn2_ICSD_87578	insulator	insulator
Cs8Fe2O7_ICSD_174315	insulator	insulator
Cs8Ga4Se10_ICSD_30810	insulator	insulator
Cs8Na16Si136_ICSD_240030	metal	metal
Cs8O19Ta6_ICSD_411856	insulator	insulator
Cs9F27Ni9_ICSD_410393	insulator	insulator
Cs9Fe1O4_ICSD_420323	metal	metal
Cs9Ga1O4_ICSD_420320	metal	metal
Cs9In1O4_ICSD_260842	metal	metal
Cs9O4Sc1_ICSD_420322	metal	metal
Cu10Sb3_ICSD_44480	metal	metal
Cu10Sn3_ICSD_103105	metal	metal
Cu10Sn3_ICSD_187900	metal	metal
Cu10Sn3_ICSD_629279	metal	metal
Cu11O26V6_ICSD_201626	insulator	insulator
Cu11Sb3_ICSD_103093	metal	metal
Cu11Sn8Yb5_ICSD_413486	metal	metal
Cu12S13Sb4_ICSD_62116	metal	metal
Cu13La1_ICSD_628215	metal	metal
Cu15Si4_ICSD_36254	metal	metal
Cu16Mg6Si7_ICSD_628339	metal	metal
Cu1Dy1Ge1_ICSD_82553	metal	metal
Cu1Dy1In1_ICSD_627158	metal	metal
Cu1Dy1O1Se1_ICSD_80359	insulator	insulator
Cu1Dy1O8W2_ICSD_73749	insulator	insulator
Cu1Dy1Pb1Se3_ICSD_152517	insulator	insulator
Cu1Dy1Pb1_ICSD_107274	metal	metal
Cu1Dy1Pb1_ICSD_627160	metal	metal
Cu1Dy1S2_ICSD_172847	insulator	insulator
Cu1Dy1S2_ICSD_627161	insulator	insulator
Cu1Dy1Sb2_ICSD_658232	metal	metal
Cu1Dy1Si1_ICSD_61481	metal	metal
Cu1Dy1Sn1_ICSD_405201	metal	metal
Cu1Dy1Sn1_ICSD_627194	metal	metal

Supplementary Table 318. Five-fold cross validated predictions for the metal/insulator classification (310/598).

system	calculated	predicted
Cu1Dy1_ICSD_627125	metal	metal
Cu1Dy2Ge4O12_ICSD_95168	insulator	insulator
Cu1Dy2Rb1Se4_ICSD_91092	insulator	insulator
Cu1Dy3Ge1S7_ICSD_627147	insulator	insulator
Cu1Dy3Ge1Se7_ICSD_280032	insulator	insulator
Cu1Dy3S7Si1_ICSD_156279	insulator	insulator
Cu1Dy3S7Sn1_ICSD_155943	insulator	insulator
Cu1Dy3Se7Si1_ICSD_627179	insulator	insulator
Cu1Dy3Se7Sn1_ICSD_152552	insulator	insulator
Cu1Er1Ge1_ICSD_82555	metal	metal
Cu1Er1In1_ICSD_627226	metal	metal
Cu1Er1O8W2_ICSD_73747	insulator	insulator
Cu1Er1P2_ICSD_94444	metal	metal
Cu1Er1Pb1Se3_ICSD_152519	insulator	insulator
Cu1Er1Pb1_ICSD_107284	metal	metal
Cu1Er1Pb1_ICSD_627233	metal	metal
Cu1Er1S2_ICSD_156291	insulator	insulator
Cu1Er1S2_ICSD_172849	insulator	insulator
Cu1Er1Sb2_ICSD_658234	metal	metal
Cu1Er1Si1_ICSD_30768	metal	metal
Cu1Er1Sn1_ICSD_627260	metal	metal
Cu1Er1_ICSD_102881	metal	metal
Cu1Er2Ge4O12_ICSD_89987	insulator	insulator
Cu1Er2K1Se4_ICSD_95029	insulator	insulator
Cu1Er3Ge1S7_ICSD_155080	insulator	insulator
Cu1Er3S7Si1_ICSD_627245	insulator	insulator
Cu1Er3S7Sn1_ICSD_627247	insulator	insulator
Cu1Eu1F1S1_ICSD_172797	insulator	insulator
Cu1Eu1F1Se1_ICSD_172799	insulator	insulator
Cu1Eu1F1Te1_ICSD_419381	insulator	insulator
Cu1Eu1O1Se1_ICSD_159258	metal	insulator
Cu1Eu1O2_ICSD_18106	metal	metal
Cu1Eu1P1_ICSD_53253	metal	metal
Cu1Eu1Sb1_ICSD_53254	metal	metal
Cu1Eu1Sn2_ICSD_416795	metal	metal
Cu1Eu1_ICSD_108374	metal	metal
Cu1Eu1_ICSD_627270	metal	metal
Cu1Eu2O4_ICSD_71183	metal	metal
Cu1F11Na1Zr2_ICSD_78869	insulator	insulator
Cu1F16Sb2Xe2_ICSD_249655	insulator	insulator
Cu1F1H4O5P1_ICSD_33923	insulator	insulator
Cu1F1Na0.125S1Sr0.875_ICSD_157434	metal	insulator
Cu1F1Na1O4S1_ICSD_262273	insulator	insulator
Cu1F1Nb1O3_ICSD_200533	insulator	insulator
Cu1F1S1Sr1_ICSD_157433	insulator	insulator
Cu1F1Se1Sr1_ICSD_172798	insulator	insulator
Cu1F1Sr1Te1_ICSD_183708	insulator	insulator
Cu1F1_ICSD_52273	metal	insulator
Cu1F20Sb2Xe4_ICSD_249656	metal	insulator
Cu1F24Sb2Xe6_ICSD_416303	insulator	insulator

Supplementary Table 319. Five-fold cross validated predictions for the metal/insulator classification (311/598).

system	calculated	predicted
Cu1F2H12N2O8P2_ICSD_74551	metal	insulator
Cu1F2H4O2_ICSD_27866	insulator	insulator
Cu1F2O3W1_ICSD_60759	insulator	insulator
Cu1F2_ICSD_9790	insulator	insulator
Cu1F3K1_ICSD_21111	insulator	insulator
Cu1F3K1_ICSD_27689	metal	insulator
Cu1F3K1_ICSD_8000	insulator	insulator
Cu1F3K1_ICSD_9856	insulator	insulator
Cu1F3Na1_ICSD_69655	insulator	insulator
Cu1F3Rb1_ICSD_109293	insulator	insulator
Cu1F3Rb1_ICSD_69656	insulator	insulator
Cu1F3Tl1_ICSD_43457	metal	insulator
Cu1F4H12N2O2_ICSD_39518	metal	insulator
Cu1F4K2_ICSD_31688	insulator	insulator
Cu1F4Na2_ICSD_34602	insulator	insulator
Cu1F4Sr1_ICSD_9927	insulator	insulator
Cu1F6H8O4Si1_ICSD_39564	insulator	insulator
Cu1F6K2Na1_ICSD_22111	insulator	insulator
Cu1F6Pt1_ICSD_64660	insulator	insulator
Cu1F6Sn1_ICSD_25016	metal	insulator
Cu1F6Sn1_ICSD_36514	insulator	insulator
Cu1F6Sr2_ICSD_26106	metal	insulator
Cu1F6Sr2_ICSD_9926	insulator	insulator
Cu1F6Zr1_ICSD_30116	metal	insulator
Cu1F6Zr1_ICSD_30117	insulator	insulator
Cu1F8H12O6Sb2_ICSD_20925	metal	metal
Cu1Fe1O2_ICSD_246913	insulator	metal
Cu1Fe1O2_ICSD_66546	metal	metal
Cu1Fe1O2_ICSD_98488	metal	insulator
Cu1Fe1O3S1Sr2_ICSD_84963	insulator	insulator
Cu1Fe1Pt2_ICSD_627330	metal	metal
Cu1Fe1S2_ICSD_2518	metal	metal
Cu1Fe1S2_ICSD_27653	metal	metal
Cu1Fe1Se2_ICSD_656334	metal	metal
Cu1Fe2Ge2_ICSD_62330	metal	metal
Cu1Fe2In1Se4_ICSD_160047	insulator	metal
Cu1Fe2S3_ICSD_29095	metal	insulator
Cu1Fe2S3_ICSD_42104	metal	insulator
Cu1Ga1Hf1_ICSD_627409	metal	metal
Cu1Ga1I4_ICSD_400817	insulator	insulator
Cu1Ga1O2_ICSD_60846	insulator	insulator
Cu1Ga1O2_ICSD_95664	insulator	insulator
Cu1Ga1O3S1Sr2_ICSD_83630	insulator	insulator
Cu1Ga1Pt2_ICSD_102928	metal	metal
Cu1Ga1S2_ICSD_187021	insulator	insulator
Cu1Ga1Se2_ICSD_603766	insulator	insulator
Cu1Ga1Te2_ICSD_627551	insulator	insulator
Cu1Ga1U1_ICSD_602613	metal	metal
Cu1Ga1Zr1_ICSD_627583	metal	metal
Cu1Ga2O10Se3_ICSD_249926	insulator	insulator

Supplementary Table 320. Five-fold cross validated predictions for the metal/insulator classification (312/598).

system	calculated	predicted
Cu1Ga2_ICSD_102906	metal	metal
Cu1Gd1Ge1_ICSD_416030	metal	metal
Cu1Gd1O1Se1_ICSD_80358	insulator	insulator
Cu1Gd1S2_ICSD_415077	metal	insulator
Cu1Gd1S3Sr1_ICSD_250435	insulator	insulator
Cu1Gd1Sb2_ICSD_153568	metal	metal
Cu1Gd1Sb2_ICSD_658230	metal	metal
Cu1Gd1Si1_ICSD_108359	metal	metal
Cu1Gd1Si1_ICSD_30764	metal	metal
Cu1Gd1Sn1_ICSD_151171	metal	metal
Cu1Gd1Sn1_ICSD_600994	metal	metal
Cu1Gd1Sn1_ICSD_627665	metal	metal
Cu1Gd1_ICSD_102949	metal	metal
Cu1Gd1_ICSD_627589	metal	metal
Cu1Gd2K1Mo4O16_ICSD_404016	insulator	insulator
Cu1Gd2K1S4_ICSD_408854	insulator	insulator
Cu1Gd2O12Se4_ICSD_412945	metal	insulator
Cu1Gd2O4_ICSD_65015	metal	metal
Cu1Gd2Rb1Se4_ICSD_91091	insulator	insulator
Cu1Gd3Ge1S7_ICSD_627608	insulator	insulator
Cu1Gd3S7Si1_ICSD_627636	insulator	insulator
Cu1Gd3S7Sn1_ICSD_627638	insulator	insulator
Cu1Ge1Hf1_ICSD_290194	metal	metal
Cu1Ge1Hf1_ICSD_627690	metal	metal
Cu1Ge1Ho1_ICSD_603200	metal	metal
Cu1Ge1Ho1_ICSD_82554	metal	metal
Cu1Ge1Ho3S7_ICSD_159888	insulator	insulator
Cu1Ge1La3S7_ICSD_627711	insulator	insulator
Cu1Ge1La3Se7_ICSD_95026	insulator	insulator
Cu1Ge1Li2_ICSD_627718	metal	metal
Cu1Ge1Mg1_ICSD_627730	metal	metal
Cu1Ge1Mn2_ICSD_184949	metal	metal
Cu1Ge1Nd3S7_ICSD_627749	insulator	insulator
Cu1Ge1Nd3Se7_ICSD_627750	insulator	insulator
Cu1Ge1O3_ICSD_96085	metal	insulator
Cu1Ge1Pr3S7_ICSD_627770	insulator	insulator
Cu1Ge1Pr3Se7_ICSD_154744	insulator	insulator
Cu1Ge1S7Tb3_ICSD_155078	insulator	insulator
Cu1Ge1S7Y3_ICSD_154626	insulator	insulator
Cu1Ge1Sc1_ICSD_627808	metal	metal
Cu1Ge1Se7Tb3_ICSD_154748	insulator	insulator
Cu1Ge1Se7Y3_ICSD_157111	insulator	insulator
Cu1Ge1Tb1_ICSD_82552	metal	metal
Cu1Ge1U1_ICSD_57201	metal	metal
Cu1Ge1Yb1_ICSD_245766	metal	metal
Cu1Ge1Yb1_ICSD_414432	metal	metal
Cu1Ge1Yb1_ICSD_603203	metal	metal
Cu1Ge1Zr1_ICSD_627886	metal	metal
Cu1Ge2Hf1_ICSD_627688	metal	metal
Cu1Ge2La1_ICSD_627707	metal	metal

Supplementary Table 321. Five-fold cross validated predictions for the metal/insulator classification (313/598).

system	calculated	predicted
Cu1Ge2Nd1_ICSD_627744	metal	metal
Cu1Ge2Nd2O8_ICSD_81073	insulator	insulator
Cu1Ge2O7Sr2_ICSD_86843	insulator	insulator
Cu1Ge2Pr1_ICSD_627765	metal	metal
Cu1Ge2Zr1_ICSD_627883	metal	metal
Cu1Ge4Ho2O12_ICSD_89986	insulator	insulator
Cu1Ge4O12Tb2_ICSD_95167	insulator	insulator
Cu1H10O9Se1_ICSD_280783	metal	metal
Cu1H12I4N4_ICSD_4072	metal	insulator
Cu1H12N10O8_ICSD_412606	metal	metal
Cu1H12N2O12_ICSD_171146	insulator	insulator
Cu1H12N2O6Sn1_ICSD_103	metal	insulator
Cu1H18N6O10V2_ICSD_417860	metal	insulator
Cu1H1I1O4_ICSD_41804	insulator	insulator
Cu1H1O4P1_ICSD_260593	insulator	insulator
Cu1H1O5S1Tl1_ICSD_36581	metal	insulator
Cu1H1O5Se1Tl1_ICSD_36582	insulator	insulator
Cu1H1_ICSD_44859	insulator	metal
Cu1H2K1O5P1_ICSD_9	insulator	insulator
Cu1H2O2_ICSD_68459	insulator	insulator
Cu1H2O4Si1_ICSD_20199	insulator	insulator
Cu1H2O5S1_ICSD_68184	metal	insulator
Cu1H2O5Se1_ICSD_39887	insulator	insulator
Cu1H2O6Pb1S1_ICSD_164673	insulator	metal
Cu1H2O6Se2_ICSD_51676	insulator	insulator
Cu1H3O7V2_ICSD_281323	insulator	insulator
Cu1H4O4P2_ICSD_280918	insulator	insulator
Cu1H4O4P2_ICSD_280921	insulator	insulator
Cu1H5O5P1_ICSD_260208	insulator	insulator
Cu1H6N1O5P1_ICSD_250189	insulator	insulator
Cu1H6N4O4_ICSD_72461	insulator	insulator
Cu1H6O14Pb4S2_ICSD_380382	metal	insulator
Cu1H6O5Sr1_ICSD_154808	insulator	insulator
Cu1H6O7S1_ICSD_34679	insulator	insulator
Cu1Hf0.5P1_ICSD_627904	metal	metal
Cu1Hf1Hg2_ICSD_102969	metal	metal
Cu1Hf1K1S3_ICSD_409293	insulator	insulator
Cu1Hf1Na1Se3_ICSD_402578	insulator	insulator
Cu1Hf1S3Tl1_ICSD_82562	insulator	insulator
Cu1Hf1Se3Tl1_ICSD_82563	insulator	insulator
Cu1Hf1Si1_ICSD_627916	metal	metal
Cu1Hf1Si2_ICSD_627918	metal	metal
Cu1Hf2Na1Se5_ICSD_98476	insulator	insulator
Cu1Hf2Sb3_ICSD_93243	metal	metal
Cu1Hf2_ICSD_627900	metal	metal
Cu1Hf4P1_ICSD_627906	metal	metal
Cu1Hf4Si4_ICSD_421374	metal	metal
Cu1Hf5Pb3_ICSD_107314	metal	metal
Cu1Hf5Sn3_ICSD_107310	metal	metal
Cu1Hg1I1S1_ICSD_413429	insulator	insulator

Supplementary Table 322. Five-fold cross validated predictions for the metal/insulator classification (314/598).

system	calculated	predicted
Cu1Hg2Ti1_ICSD_102972	metal	metal
Cu1Hg2Zr1_ICSD_102973	metal	metal
Cu1Ho1In1_ICSD_162738	metal	metal
Cu1Ho1P2_ICSD_94443	metal	metal
Cu1Ho1Pb1Se3_ICSD_154669	insulator	insulator
Cu1Ho1Pb1_ICSD_107283	metal	metal
Cu1Ho1Pb1_ICSD_627959	metal	metal
Cu1Ho1S2_ICSD_172848	insulator	insulator
Cu1Ho1S2_ICSD_627960	insulator	insulator
Cu1Ho1Sb2_ICSD_153571	metal	metal
Cu1Ho1Si1_ICSD_61482	metal	metal
Cu1Ho1Sn1_ICSD_601006	metal	metal
Cu1Ho1_ICSD_102974	metal	metal
Cu1Ho2K1Mo4O16_ICSD_380015	insulator	insulator
Cu1Ho2K1S4_ICSD_97561	insulator	insulator
Cu1Ho2K1Se4_ICSD_95028	insulator	insulator
Cu1Ho2O5Sr1_ICSD_172226	insulator	insulator
Cu1Ho3S7Si1_ICSD_159886	insulator	insulator
Cu1Ho3S7Sn1_ICSD_155944	insulator	insulator
Cu1Ho3Se7Si1_ICSD_159887	insulator	insulator
Cu1I1P4Se4_ICSD_410913	insulator	insulator
Cu1I1Te2_ICSD_67253	insulator	insulator
Cu1I1_ICSD_163445	metal	insulator
Cu1I1_ICSD_23990	insulator	insulator
Cu1I1_ICSD_246688	insulator	insulator
Cu1I1_ICSD_246690	insulator	insulator
Cu1I1_ICSD_24772	insulator	insulator
Cu1I1_ICSD_30363	insulator	insulator
Cu1I1_ICSD_84217	insulator	insulator
Cu1I2O6_ICSD_2232	insulator	insulator
Cu1I2O6_ICSD_4327	insulator	insulator
Cu1I3Na1O9_ICSD_62367	metal	insulator
Cu1In1La1_ICSD_628010	metal	metal
Cu1In1Lu1_ICSD_628015	metal	metal
Cu1In1Mo2O8_ICSD_73097	insulator	insulator
Cu1In1Nd1_ICSD_162733	metal	metal
Cu1In1O2_ICSD_91058	insulator	insulator
Cu1In1O2_ICSD_95670	insulator	insulator
Cu1In1O8W2_ICSD_74944	insulator	insulator
Cu1In1P2Se6_ICSD_71969	insulator	insulator
Cu1In1Pr1_ICSD_628048	metal	metal
Cu1In1Pt2_ICSD_108380	metal	metal
Cu1In1S2_ICSD_600239	insulator	insulator
Cu1In1Se2_ICSD_73351	metal	insulator
Cu1In1Ta2Te4_ICSD_161365	metal	metal
Cu1In1Tb1_ICSD_260991	metal	metal
Cu1In1Te2_ICSD_628160	insulator	insulator
Cu1In1Y1_ICSD_628181	metal	metal
Cu1In1_ICSD_180112	metal	metal
Cu1In2O4_ICSD_39475	metal	insulator

Supplementary Table 323. Five-fold cross validated predictions for the metal/insulator classification (315/598).

system	calculated	predicted
Cu1In2Se4_ICSD_183470	metal	insulator
Cu1In2_ICSD_180113	metal	metal
Cu1In2_ICSD_187899	metal	metal
Cu1Ir1O6Sr3_ICSD_36628	insulator	metal
Cu1Ir2S4_ICSD_75532	metal	metal
Cu1K1Na2O2_ICSD_47105	insulator	insulator
Cu1K1Nd2S4_ICSD_97558	insulator	insulator
Cu1K1O1_ICSD_188529	metal	metal
Cu1K1O1_ICSD_37325	insulator	insulator
Cu1K1O1_ICSD_40158	insulator	insulator
Cu1K1O2_ICSD_203081	insulator	insulator
Cu1K1O4P1_ICSD_100172	insulator	insulator
Cu1K1Pd1Se5_ICSD_281423	insulator	insulator
Cu1K1S1_ICSD_49008	insulator	insulator
Cu1K1S3Th1_ICSD_170864	insulator	insulator
Cu1K1S3U1_ICSD_249701	insulator	insulator
Cu1K1S3Zr1_ICSD_80624	insulator	insulator
Cu1K1S4Tb2_ICSD_97560	insulator	insulator
Cu1K1S4Y2_ICSD_97557	insulator	insulator
Cu1K1Se1_ICSD_12157	insulator	insulator
Cu1K1Se3U1_ICSD_156246	insulator	insulator
Cu1K1Se3Zr1_ICSD_80625	insulator	insulator
Cu1K1Te1_ICSD_12158	insulator	insulator
Cu1K2N6O12Pb1_ICSD_1274	metal	metal
Cu1K2N6O12Pb1_ICSD_16127	metal	metal
Cu1K2N6O12Pb1_ICSD_36135	insulator	metal
Cu1K2N6O12Sr1_ICSD_178	metal	metal
Cu1K2Nb1S4_ICSD_402422	insulator	insulator
Cu1K2Nb1Se4_ICSD_73324	insulator	insulator
Cu1K2O12P4_ICSD_23754	insulator	insulator
Cu1K2P1_ICSD_61082	insulator	insulator
Cu1K2S3Sb1_ICSD_415483	insulator	insulator
Cu1K2S4V1_ICSD_81414	insulator	insulator
Cu1K2Sb1_ICSD_53298	insulator	insulator
Cu1K2Se4Ta1_ICSD_72427	insulator	insulator
Cu1K2Se4V1_ICSD_84303	insulator	insulator
Cu1K3O2_ICSD_48180	insulator	insulator
Cu1K5Sb2_ICSD_32032	insulator	insulator
Cu1K6O8Si2_ICSD_85404	insulator	insulator
Cu1La1Mg4_ICSD_418214	metal	metal
Cu1La1O1S1_ICSD_201046	insulator	insulator
Cu1La1O1Se1_ICSD_96758	insulator	insulator
Cu1La1O1Te1_ICSD_154591	insulator	insulator
Cu1La1O2_ICSD_18102	insulator	insulator
Cu1La1O3_ICSD_73554	metal	metal
Cu1La1O8W2_ICSD_68614	insulator	insulator
Cu1La1Pb1_ICSD_628235	metal	metal
Cu1La1S1Te1_ICSD_88012	insulator	insulator
Cu1La1S2_ICSD_24375	insulator	insulator
Cu1La1S2_ICSD_415078	insulator	insulator

Supplementary Table 324. Five-fold cross validated predictions for the metal/insulator classification (316/598).

system	calculated	predicted
Cu1La1Sb2_ICSD_658225	metal	metal
Cu1La1Se2_ICSD_99675	insulator	insulator
Cu1La1Si1_ICSD_84207	metal	metal
Cu1La1Sn1_ICSD_416545	metal	metal
Cu1La1_ICSD_628213	metal	metal
Cu1La2O4_ICSD_173894	metal	metal
Cu1La2O4_ICSD_261659	metal	metal
Cu1La2O4_ICSD_56528	metal	metal
Cu1La2O4_ICSD_67837	metal	metal
Cu1La2O4_ICSD_73910	metal	metal
Cu1La2S4_ICSD_628243	insulator	insulator
Cu1La3S7Sn1_ICSD_628248	insulator	insulator
Cu1La3Se7Si1_ICSD_628256	insulator	insulator
Cu1La3Se7Sn1_ICSD_152545	insulator	insulator
Cu1La4S7_ICSD_628240	metal	insulator
Cu1Li1O1_ICSD_188527	metal	metal
Cu1Li1O1_ICSD_49755	insulator	insulator
Cu1Li1O2_ICSD_74978	insulator	insulator
Cu1Li1O4V1_ICSD_65677	insulator	insulator
Cu1Li1O9P3_ICSD_2808	insulator	insulator
Cu1Li2O2_ICSD_174134	insulator	insulator
Cu1Li2O2_ICSD_67150	insulator	insulator
Cu1Li2O7P2_ICSD_160321	insulator	insulator
Cu1Li2O8W2_ICSD_92854	insulator	insulator
Cu1Li2P1_ICSD_240250	metal	insulator
Cu1Li2Sb1_ICSD_181259	insulator	metal
Cu1Li2Sn1_ICSD_103043	metal	metal
Cu1Li3O3_ICSD_4201	insulator	insulator
Cu1Lu1Pb1_ICSD_107286	metal	metal
Cu1Lu1S2_ICSD_628310	insulator	insulator
Cu1Lu1Sb2_ICSD_658237	metal	metal
Cu1Lu1Si1_ICSD_30771	metal	metal
Cu1Lu1Sn1_ICSD_416546	metal	metal
Cu1Lu1Sn1_ICSD_628323	metal	metal
Cu1Mg1P1_ICSD_8221	insulator	metal
Cu1Mg1Sb1_ICSD_77364	metal	metal
Cu1Mg1Sn1_ICSD_103054	metal	metal
Cu1Mg1Sn1_ICSD_628345	metal	metal
Cu1Mg1Y1_ICSD_163695	metal	metal
Cu1Mg2_ICSD_103047	metal	metal
Cu1Mn1O2_ICSD_158960	insulator	metal
Cu1Mn1O3S1Sr2_ICSD_91199	metal	insulator
Cu1Mn1O4V1_ICSD_170136	insulator	insulator
Cu1Mn1P1_ICSD_72411	metal	metal
Cu1Mn1Sb1_ICSD_53311	metal	metal
Cu1Mn2O4_ICSD_174000	metal	metal
Cu1Mn2Sb1_ICSD_184950	metal	metal
Cu1Mn3N1_ICSD_53306	metal	metal
Cu1Mn3N1_ICSD_628356	metal	metal
Cu1Mo1O6Sr2_ICSD_186038	metal	insulator

Supplementary Table 325. Five-fold cross validated predictions for the metal/insulator classification (317/598).

system	calculated	predicted
Cu1Mo6S8_ICSD_628433	metal	metal
Cu1Mo6Se8_ICSD_628449	metal	metal
Cu1N1Ni3_ICSD_183372	metal	metal
Cu1N1_ICSD_161756	metal	metal
Cu1N2O6_ICSD_28477	insulator	insulator
Cu1N2Ta1_ICSD_71136	insulator	metal
Cu1N3_ICSD_30633	insulator	insulator
Cu1N6O12Pb1Rb2_ICSD_372	metal	metal
Cu1N6O12Pb1Tl2_ICSD_16	metal	metal
Cu1N6_ICSD_24340	metal	insulator
Cu1Na1O1_ICSD_15099	insulator	insulator
Cu1Na1O1_ICSD_188528	metal	metal
Cu1Na1O2_ICSD_80561	insulator	insulator
Cu1Na1O4P1_ICSD_35451	insulator	insulator
Cu1Na1O4P1_ICSD_73398	metal	insulator
Cu1Na1S3Ti1_ICSD_73886	insulator	insulator
Cu1Na1S3Zr1_ICSD_73887	insulator	insulator
Cu1Na1Se1_ICSD_12155	insulator	insulator
Cu1Na1Se3Zr1_ICSD_73888	insulator	insulator
Cu1Na1Te1_ICSD_12156	insulator	insulator
Cu1Na1Te3Zr1_ICSD_73889	metal	insulator
Cu1Na2O2_ICSD_422751	insulator	insulator
Cu1Na2O7P2_ICSD_80418	insulator	insulator
Cu1Na2P1_ICSD_1153	insulator	insulator
Cu1Na5O2S1_ICSD_412977	insulator	insulator
Cu1Nb1O3_ICSD_201899	insulator	insulator
Cu1Nb1Rb2S4_ICSD_84304	insulator	insulator
Cu1Nb1Rb2Se4_ICSD_84305	insulator	insulator
Cu1Nb1Te2_ICSD_414338	metal	metal
Cu1Nb2S4_ICSD_628465	metal	metal
Cu1Nb3Te4_ICSD_628496	metal	metal
Cu1Nb4Si1_ICSD_628492	metal	metal
Cu1Nd1O1S1_ICSD_96346	insulator	metal
Cu1Nd1O1Te1_ICSD_416523	metal	insulator
Cu1Nd1O2_ICSD_83052	metal	insulator
Cu1Nd1O8W2_ICSD_40381	insulator	insulator
Cu1Nd1S2_ICSD_51405	insulator	insulator
Cu1Nd1Sb2_ICSD_93155	metal	metal
Cu1Nd1Se2_ICSD_95827	insulator	insulator
Cu1Nd1Si1_ICSD_30762	metal	metal
Cu1Nd1Sn1_ICSD_151169	metal	metal
Cu1Nd1Sn1_ICSD_601011	metal	metal
Cu1Nd1Sn1_ICSD_628539	metal	metal
Cu1Nd1_ICSD_246591	metal	metal
Cu1Nd1_ICSD_628504	metal	metal
Cu1Nd2O4_ICSD_202885	metal	metal
Cu1Nd2O4_ICSD_86754	metal	metal
Cu1Nd2Rb1S4_ICSD_93678	insulator	insulator
Cu1Nd3S7Si1_ICSD_156275	insulator	insulator
Cu1Nd3S7Sn1_ICSD_155938	insulator	insulator

Supplementary Table 326. Five-fold cross validated predictions for the metal/insulator classification (318/598).

system	calculated	predicted
Cu1Nd3Se7Si1_ICSD_628529	insulator	insulator
Cu1Nd3Se7Sn1_ICSD_152548	insulator	insulator
Cu1Ni1S3Sb1_ICSD_628553	insulator	insulator
Cu1Ni1Sb2_ICSD_57210	metal	metal
Cu1Ni2Sb1_ICSD_53320	metal	metal
Cu1Ni2Sn1_ICSD_103068	metal	metal
Cu1Ni2Ti1_ICSD_628580	metal	metal
Cu1O1P1Th1_ICSD_182311	metal	metal
Cu1O1P1U1_ICSD_79579	metal	metal
Cu1O1Pr1S1_ICSD_96345	metal	insulator
Cu1O1Rb1_ICSD_15100	insulator	insulator
Cu1O1Rb1_ICSD_188523	insulator	insulator
Cu1O1Rb1_ICSD_188530	metal	metal
Cu1O1_ICSD_61323	metal	metal
Cu1O1_ICSD_92367	metal	metal
Cu1O2Pr1_ICSD_246822	insulator	metal
Cu1O2Rb1_ICSD_15096	insulator	insulator
Cu1O2Rh1_ICSD_29214	insulator	insulator
Cu1O2Sc1_ICSD_151929	insulator	insulator
Cu1O2Sc1_ICSD_55689	insulator	insulator
Cu1O2Sr1_ICSD_202992	metal	metal
Cu1O2Y1_ICSD_60848	insulator	insulator
Cu1O2Y1_ICSD_95673	insulator	insulator
Cu1O2_ICSD_150886	metal	metal
Cu1O2_ICSD_54126	metal	metal
Cu1O2_ICSD_89236	metal	metal
Cu1O2_ICSD_96699	metal	metal
Cu1O3Se1_ICSD_29506	insulator	insulator
Cu1O3Se1_ICSD_498	insulator	insulator
Cu1O3Sr2_ICSD_151811	metal	metal
Cu1O3Ta1_ICSD_164934	insulator	insulator
Cu1O3Te1_ICSD_202451	metal	insulator
Cu1O3V1_ICSD_19046	insulator	insulator
Cu1O3V1_ICSD_9414	insulator	insulator
Cu1O4P1Rb1_ICSD_89904	metal	insulator
Cu1O4P1Tl1_ICSD_50457	insulator	insulator
Cu1O4Pr2_ICSD_202884	metal	metal
Cu1O4Pr2_ICSD_91072	metal	metal
Cu1O4Pr2_ICSD_91073	metal	metal
Cu1O4Rh2_ICSD_23486	metal	metal
Cu1O4Rh2_ICSD_88962	metal	metal
Cu1O4S1_ICSD_71017	insulator	insulator
Cu1O4Se1_ICSD_109073	insulator	insulator
Cu1O4U1_ICSD_36071	insulator	insulator
Cu1O4W1_ICSD_169005	metal	insulator
Cu1O4W1_ICSD_4189	metal	insulator
Cu1O5Se2_ICSD_603	insulator	insulator
Cu1O5Sr2Tl1_ICSD_86427	metal	metal
Cu1O5Te2_ICSD_2542	insulator	insulator
Cu1O6Os1Sr2_ICSD_245819	metal	metal

Supplementary Table 327. Five-fold cross validated predictions for the metal/insulator classification (319/598).

system	calculated	predicted
Cu1O6Pt1Sr3_ICSD_66347	insulator	insulator
Cu1O6Rh1Sr3_ICSD_51800	insulator	metal
Cu1O6S2Tl2_ICSD_2896	insulator	insulator
Cu1O6Sb2_ICSD_30410	metal	insulator
Cu1O6Sb2_ICSD_628623	metal	insulator
Cu1O6Sb2_ICSD_84789	insulator	insulator
Cu1O6Se2Sr1_ICSD_202389	insulator	insulator
Cu1O6Sr2Te1_ICSD_88702	insulator	insulator
Cu1O6Sr2W1_ICSD_33571	insulator	insulator
Cu1O6Ta2_ICSD_83367	insulator	insulator
Cu1O6Te1Tl4_ICSD_187561	insulator	insulator
Cu1O6V2_ICSD_21067	insulator	insulator
Cu1O6V2_ICSD_28151	insulator	insulator
Cu1O7Rb4Si2_ICSD_85405	insulator	insulator
Cu1O7Si2Sr2_ICSD_86842	insulator	insulator
Cu1O7Sr1Te2_ICSD_261937	metal	insulator
Cu1O7Sr1V2_ICSD_39620	insulator	insulator
Cu1O8P2Sr2_ICSD_51714	insulator	insulator
Cu1O8Tb1W2_ICSD_74943	insulator	insulator
Cu1O8W2Y1_ICSD_36622	insulator	insulator
Cu1P1Si1Zr1_ICSD_59594	metal	metal
Cu1P1Sr1_ICSD_53323	insulator	metal
Cu1P1Zr4_ICSD_108398	metal	metal
Cu1P2U1_ICSD_40763	metal	metal
Cu1P2Y1_ICSD_95177	metal	metal
Cu1P2_ICSD_35282	insulator	insulator
Cu1Pb1Pr1_ICSD_628687	metal	metal
Cu1Pb1S3Y1_ICSD_152555	insulator	insulator
Cu1Pb1Se3Tb1_ICSD_152516	insulator	insulator
Cu1Pb1Se3Yb1_ICSD_152521	metal	insulator
Cu1Pb1Tb1_ICSD_107282	metal	metal
Cu1Pb1Tm1_ICSD_107285	metal	metal
Cu1Pb1Y1_ICSD_107004	metal	metal
Cu1Pb1Yb1_ICSD_628692	metal	metal
Cu1Pd1Rb1Se5_ICSD_281424	insulator	insulator
Cu1Pd1_ICSD_181913	metal	metal
Cu1Pr1S2_ICSD_95829	insulator	insulator
Cu1Pr1Sb2_ICSD_93153	metal	metal
Cu1Pr1Se2_ICSD_99677	insulator	insulator
Cu1Pr1Se3Sr1_ICSD_414181	insulator	insulator
Cu1Pr1Si1_ICSD_30761	metal	metal
Cu1Pr1Sn1_ICSD_628741	metal	metal
Cu1Pr1Sn2_ICSD_628742	metal	metal
Cu1Pr1_ICSD_628710	metal	metal
Cu1Pr3S7Si1_ICSD_628722	insulator	insulator
Cu1Pr3S7Sn1_ICSD_155939	insulator	insulator
Cu1Pr3Se7Si1_ICSD_156283	insulator	insulator
Cu1Pt1_ICSD_107118	metal	metal
Cu1Pt7_ICSD_108775	metal	metal
Cu1Rb1S3U1_ICSD_249704	insulator	insulator

Supplementary Table 328. Five-fold cross validated predictions for the metal/insulator classification (320/598).

system	calculated	predicted
Cu1Rb1Se4_ICSD_404225	insulator	insulator
Cu1Rb2S4Ta1_ICSD_414271	insulator	insulator
Cu1Rb2S4V1_ICSD_84302	insulator	insulator
Cu1Rb2Se4Ta1_ICSD_414272	insulator	insulator
Cu1Rh2S4_ICSD_41900	metal	metal
Cu1Rh2S4_ICSD_628767	metal	metal
Cu1Rh2Se4_ICSD_628775	metal	metal
Cu1Rh2Sn1_ICSD_103092	metal	metal
Cu1S1_ICSD_24586	metal	metal
Cu1S1_ICSD_32105	metal	metal
Cu1S1_ICSD_63328	metal	metal
Cu1S2Sb1_ICSD_628829	insulator	insulator
Cu1S2Sc1_ICSD_15298	insulator	insulator
Cu1S2Tb1_ICSD_415490	insulator	insulator
Cu1S2Tl1_ICSD_628931	metal	insulator
Cu1S2Tm1_ICSD_172850	metal	metal
Cu1S2Tm1_ICSD_628936	metal	metal
Cu1S2Y1_ICSD_152553	insulator	insulator
Cu1S2Y1_ICSD_92458	insulator	insulator
Cu1S2Yb1_ICSD_152805	metal	metal
Cu1S2Yb1_ICSD_172851	metal	metal
Cu1S2_ICSD_628781	metal	metal
Cu1S2_ICSD_628782	metal	metal
Cu1S2_ICSD_628791	metal	metal
Cu1S3Ta1_ICSD_187747	insulator	insulator
Cu1S3Tl1Zr1_ICSD_82560	insulator	insulator
Cu1S4Ti2_ICSD_170228	metal	metal
Cu1S4Ti2_ICSD_628916	metal	metal
Cu1S4V2_ICSD_10035	metal	metal
Cu1S4Zr2_ICSD_27027	metal	metal
Cu1S7Si1Tb3_ICSD_156278	insulator	insulator
Cu1S7Si1Y3_ICSD_152792	insulator	insulator
Cu1S7Sn1Tb3_ICSD_155942	insulator	insulator
Cu1S7Sn1Y3_ICSD_152554	insulator	insulator
Cu1Sb1Se2_ICSD_30358	insulator	insulator
Cu1Sb1Sr1_ICSD_53339	metal	metal
Cu1Sb1Yb1_ICSD_57021	metal	metal
Cu1Sb1Yb1_ICSD_83982	metal	metal
Cu1Sb2Tb1_ICSD_658231	metal	metal
Cu1Sb2Ti5_ICSD_159248	metal	metal
Cu1Sb2U1_ICSD_656842	metal	metal
Cu1Sb2Y1_ICSD_658224	metal	metal
Cu1Sb2Yb1_ICSD_658236	metal	metal
Cu1Sb3Ti2_ICSD_93241	metal	metal
Cu1Sb3Zr2_ICSD_93242	metal	metal
Cu1Sc1Si1_ICSD_86391	metal	metal
Cu1Sc1Si1_ICSD_86392	metal	metal
Cu1Sc1Sn1_ICSD_416543	metal	metal
Cu1Sc1Sn1_ICSD_629024	metal	metal
Cu1Sc1_ICSD_103095	metal	metal

Supplementary Table 329. Five-fold cross validated predictions for the metal/insulator classification (321/598).

system	calculated	predicted
Cu1Se1_ICSD_94678	metal	metal
Cu1Se1_ICSD_94685	metal	metal
Cu1Se1_ICSD_94686	metal	metal
Cu1Se2Tb1_ICSD_157555	insulator	insulator
Cu1Se2Tl1_ICSD_28743	metal	metal
Cu1Se2_ICSD_629033	metal	metal
Cu1Se2_ICSD_629046	metal	metal
Cu1Se3Th1Tl1_ICSD_262810	insulator	insulator
Cu1Se3Tl1Zr1_ICSD_82561	insulator	insulator
Cu1Se7Si1Tb3_ICSD_629074	insulator	insulator
Cu1Se7Si1Y3_ICSD_152793	insulator	insulator
Cu1Se7Sn1Tb3_ICSD_152551	insulator	insulator
Cu1Si1Tb1_ICSD_629188	metal	metal
Cu1Si1Ti1_ICSD_629204	metal	metal
Cu1Si1Tm1_ICSD_30769	metal	metal
Cu1Si1Y1_ICSD_30772	metal	metal
Cu1Si1Yb1_ICSD_30770	metal	metal
Cu1Si1Zr1_ICSD_629245	metal	metal
Cu1Si2Zr1_ICSD_629236	metal	metal
Cu1Sn1Tb1_ICSD_601004	metal	metal
Cu1Sn1Ti1_ICSD_54657	metal	metal
Cu1Sn1U1_ICSD_629297	metal	metal
Cu1Sn1Y1_ICSD_416544	metal	metal
Cu1Sn1Y1_ICSD_600998	metal	metal
Cu1Sn1_ICSD_629278	metal	metal
Cu1Sn2Sr1_ICSD_103110	metal	metal
Cu1Sn3Zr5_ICSD_656297	metal	metal
Cu1Sr1_ICSD_247133	metal	metal
Cu1Tb1_ICSD_629319	metal	metal
Cu1Te1_ICSD_93966	metal	metal
Cu1Te2_ICSD_629329	metal	metal
Cu1Te3Ti1Tl1_ICSD_81571	insulator	insulator
Cu1Te3Tl4_ICSD_629347	metal	metal
Cu1Th2_ICSD_103121	metal	metal
Cu1Th2_ICSD_150501	metal	metal
Cu1Ti1_ICSD_103127	metal	metal
Cu1Ti1_ICSD_103128	metal	metal
Cu1Ti1_ICSD_600100	metal	metal
Cu1Ti1_ICSD_629389	metal	metal
Cu1Ti2_ICSD_15807	metal	metal
Cu1Ti2_ICSD_629399	metal	metal
Cu1Ti3_ICSD_103130	metal	metal
Cu1Tm1_ICSD_629415	metal	metal
Cu1Tm1_ICSD_629417	metal	metal
Cu1Y1_ICSD_185943	metal	metal
Cu1Y1_ICSD_629426	metal	metal
Cu1Yb1_ICSD_629442	metal	metal
Cu1Zn1_ICSD_629462	metal	metal
Cu1Zr1_ICSD_103163	metal	metal
Cu1Zr1_ICSD_167596	metal	metal

Supplementary Table 330. Five-fold cross validated predictions for the metal/insulator classification (322/598).

system	calculated	predicted
Cu1Zr1_ICSD_167598	metal	metal
Cu1Zr2_ICSD_151845	metal	metal
Cu1Zr2_ICSD_151846	metal	metal
Cu1Zr3_ICSD_629481	metal	metal
Cu1_ICSD_183263	metal	metal
Cu1_ICSD_627117	metal	metal
Cu23Dy6_ICSD_627129	metal	metal
Cu23Lu6_ICSD_628304	metal	metal
Cu23Tb6_ICSD_629317	metal	metal
Cu23Yb6_ICSD_629447	metal	metal
Cu2Dy1Ge2_ICSD_627141	metal	metal
Cu2Dy1In1_ICSD_102877	metal	metal
Cu2Dy1In3_ICSD_108369	metal	metal
Cu2Dy1S2_ICSD_627162	metal	metal
Cu2Dy1Si2_ICSD_627185	metal	metal
Cu2Dy1_ICSD_627121	metal	metal
Cu2Dy2In1_ICSD_627148	metal	metal
Cu2Er1Ge2_ICSD_53251	metal	metal
Cu2Er1In1_ICSD_102885	metal	metal
Cu2Er1Si2_ICSD_165439	metal	metal
Cu2Er1_ICSD_627200	metal	metal
Cu2Er2In1_ICSD_627222	metal	metal
Cu2Eu1Ge2_ICSD_87255	metal	metal
Cu2Eu1K1Te4_ICSD_280193	metal	insulator
Cu2Eu1S4Sn1_ICSD_419600	insulator	insulator
Cu2Eu1Sb2_ICSD_77197	metal	metal
Cu2Eu1Si2_ICSD_627287	metal	metal
Cu2Eu1Sn2_ICSD_182050	metal	metal
Cu2Eu1_ICSD_627266	metal	metal
Cu2Eu3P4Zn2_ICSD_89516	metal	metal
Cu2F6Hg2S1_ICSD_156367	metal	insulator
Cu2Fe1Ge1S4_ICSD_627304	insulator	insulator
Cu2Fe1Ge1Se4_ICSD_627313	insulator	insulator
Cu2Fe1S2_ICSD_171909	insulator	metal
Cu2Fe1S2_ICSD_171910	metal	metal
Cu2Fe1S4Si1_ICSD_627355	insulator	insulator
Cu2Fe1S4Sn1_ICSD_627358	insulator	insulator
Cu2Fe1S4Sn1_ICSD_90827	metal	insulator
Cu2Fe1Se4Si1_ICSD_627368	insulator	insulator
Cu2Fe1Se4Sn1_ICSD_627369	metal	metal
Cu2Fe1Sn1_ICSD_151206	metal	metal
Cu2Fe4S7_ICSD_15973	insulator	metal
Cu2Ga1Sc1_ICSD_102930	metal	metal
Cu2Ga1Sr1_ICSD_102938	metal	metal
Cu2Gd1Ge2_ICSD_106854	metal	metal
Cu2Gd1In1_ICSD_102953	metal	metal
Cu2Gd1In1_ICSD_627620	metal	metal
Cu2Gd1O8Ru1Sr2_ICSD_85244	metal	metal
Cu2Gd1Si2_ICSD_164072	metal	metal
Cu2Gd1Si2_ICSD_627655	metal	metal

Supplementary Table 331. Five-fold cross validated predictions for the metal/insulator classification (323/598).

system	calculated	predicted
Cu2Ge1Hg1S4_ICSD_152762	insulator	insulator
Cu2Ge1Hg1S4_ICSD_187020	insulator	insulator
Cu2Ge1Hg1Se4_ICSD_152761	metal	metal
Cu2Ge1Hg1Te4_ICSD_656155	metal	metal
Cu2Ge1Li1_ICSD_15129	metal	metal
Cu2Ge1Mg1S4_ICSD_425555	insulator	insulator
Cu2Ge1Mn1S4_ICSD_627735	insulator	insulator
Cu2Ge1Mn1Se4_ICSD_627741	insulator	insulator
Cu2Ge1O4_ICSD_100796	metal	insulator
Cu2Ge1S3_ICSD_627779	metal	metal
Cu2Ge1S3_ICSD_85138	insulator	metal
Cu2Ge1S4Sr1_ICSD_10005	insulator	insulator
Cu2Ge1S4Zn1_ICSD_152752	insulator	insulator
Cu2Ge1S4Zn1_ICSD_627793	insulator	insulator
Cu2Ge1Se3_ICSD_160386	metal	metal
Cu2Ge1Se4Sr1_ICSD_411406	insulator	insulator
Cu2Ge1Se4Zn1_ICSD_627831	metal	metal
Cu2Ge1Te3_ICSD_151872	metal	metal
Cu2Ge1Te4Zn1_ICSD_152751	metal	metal
Cu2Ge2Ho1_ICSD_627700	metal	metal
Cu2Ge2La1_ICSD_81756	metal	metal
Cu2Ge2Nd1_ICSD_152916	metal	metal
Cu2Ge2Pr1_ICSD_152911	metal	metal
Cu2Ge2Sr1_ICSD_424224	metal	metal
Cu2Ge2Tb1_ICSD_169355	metal	metal
Cu2Ge2Th1_ICSD_55344	metal	metal
Cu2Ge2U1_ICSD_85998	metal	metal
Cu2Ge2Y1_ICSD_52764	metal	metal
Cu2Ge2Yb1_ICSD_424225	metal	metal
Cu2H12N8O10_ICSD_281342	insulator	insulator
Cu2H14O18S2Zn3_ICSD_404835	metal	insulator
Cu2H1K3O14P4_ICSD_62742	insulator	insulator
Cu2H1O5P1_ICSD_39679	insulator	insulator
Cu2H3N1O6_ICSD_31353	metal	insulator
Cu2H4Na2O13Si4_ICSD_414048	insulator	insulator
Cu2Hf1In1_ICSD_54594	metal	metal
Cu2Hf1P2_ICSD_53287	metal	metal
Cu2Hf1Te3_ICSD_41072	insulator	insulator
Cu2Hf3Se8Tl2_ICSD_81554	insulator	insulator
Cu2Hg1I4_ICSD_30265	insulator	insulator
Cu2Hg1I4_ICSD_79679	insulator	insulator
Cu2Hg1S4Si1_ICSD_627928	insulator	insulator
Cu2Hg1S4Sn1_ICSD_627929	metal	metal
Cu2Hg1Se4Si1_ICSD_627935	insulator	insulator
Cu2Hg1Se4Sn1_ICSD_262976	metal	metal
Cu2Hg1Si1Te4_ICSD_656152	metal	insulator
Cu2Hg1Sn1Te4_ICSD_656158	metal	metal
Cu2Ho1In1_ICSD_102979	metal	metal
Cu2Ho1S2_ICSD_627961	metal	metal
Cu2Ho1Si2_ICSD_53290	metal	metal

Supplementary Table 332. Five-fold cross validated predictions for the metal/insulator classification (324/598).

system	calculated	predicted
Cu2Ho1_ICSD_627942	metal	metal
Cu2Ho2In1_ICSD_627951	metal	metal
Cu2I3Rb1_ICSD_65250	insulator	insulator
Cu2I6Tl4_ICSD_65962	insulator	insulator
Cu2In1La1_ICSD_106404	metal	metal
Cu2In1La2_ICSD_411708	metal	metal
Cu2In1Lu1_ICSD_102993	metal	metal
Cu2In1Mn1_ICSD_102996	metal	metal
Cu2In1Nd1_ICSD_103004	metal	metal
Cu2In1Pr1_ICSD_106558	metal	metal
Cu2In1Sc1_ICSD_103009	metal	metal
Cu2In1Tb1_ICSD_152556	metal	metal
Cu2In1Tb2_ICSD_628133	metal	metal
Cu2In1Ti1_ICSD_103020	metal	metal
Cu2In1Tm1_ICSD_103022	metal	metal
Cu2In1Y1_ICSD_103023	metal	metal
Cu2In1Y2_ICSD_628177	metal	metal
Cu2In1Yb2_ICSD_107315	metal	metal
Cu2In1Zr1_ICSD_103026	metal	metal
Cu2In1_ICSD_180111	metal	metal
Cu2In2S6Si1_ICSD_157375	insulator	insulator
Cu2K1S4V1_ICSD_402924	insulator	insulator
Cu2K1Se2_ICSD_98403	metal	metal
Cu2K1Se4Ta1_ICSD_73957	insulator	insulator
Cu2K1Se4V1_ICSD_280059	insulator	insulator
Cu2K2S4Th1_ICSD_170865	insulator	insulator
Cu2K2Te5_ICSD_280533	metal	insulator
Cu2K3O4_ICSD_380487	insulator	insulator
Cu2La1Mg1_ICSD_155984	metal	metal
Cu2La1Si2_ICSD_106841	metal	metal
Cu2La1Sn2_ICSD_602618	metal	metal
Cu2La1_ICSD_103028	metal	metal
Cu2La2Mg1_ICSD_411709	metal	metal
Cu2La2O5_ICSD_73399	metal	metal
Cu2La6S14Si2_ICSD_23519	insulator	insulator
Cu2Li1O2_ICSD_71221	metal	insulator
Cu2Li1P1_ICSD_628282	metal	metal
Cu2Li1P2Tb1_ICSD_35589	metal	metal
Cu2Li1P2Y1_ICSD_628287	metal	metal
Cu2Li1P2Yb1_ICSD_628288	metal	metal
Cu2Li1P2_ICSD_247090	metal	metal
Cu2Li1Si1_ICSD_15128	metal	metal
Cu2Li1Sn1_ICSD_150602	metal	metal
Cu2Li2O6Te1_ICSD_189655	insulator	insulator
Cu2Li3O4_ICSD_66509	metal	insulator
Cu2Lu1Si2_ICSD_106846	metal	metal
Cu2Lu1Sn2_ICSD_603428	metal	metal
Cu2Mg1Nd2_ICSD_411710	metal	metal
Cu2Mg1O3_ICSD_4202	metal	insulator
Cu2Mg1Se4Si1_ICSD_425556	insulator	insulator

Supplementary Table 333. Five-fold cross validated predictions for the metal/insulator classification (325/598).

system	calculated	predicted
Cu2Mg1Y2_ICSD_411711	metal	metal
Cu2Mg1_ICSD_174171	metal	metal
Cu2Mn1O2S2Sr2_ICSD_84734	metal	metal
Cu2Mn1S4Si1_ICSD_628375	insulator	insulator
Cu2Mn1S4Sn1_ICSD_628379	metal	insulator
Cu2Mn1Sb1_ICSD_53312	metal	metal
Cu2Mn1Se4Si1_ICSD_628396	insulator	insulator
Cu2Mn1Se4Sn1_ICSD_155904	metal	metal
Cu2Mn1Sn1_ICSD_628408	metal	metal
Cu2Mn3O8_ICSD_971	metal	insulator
Cu2Mo1O7Se1_ICSD_249957	insulator	insulator
Cu2N12O24Tl5Y1_ICSD_24549	metal	metal
Cu2Na1O2_ICSD_169713	insulator	insulator
Cu2Na2O11Si4_ICSD_240930	insulator	insulator
Cu2Na2O6Te1_ICSD_170637	insulator	insulator
Cu2Na2S4Zr1_ICSD_79536	metal	insulator
Cu2Na3O6Sb1_ICSD_153037	insulator	insulator
Cu2Nb6S8_ICSD_628469	metal	metal
Cu2Nd1Si2_ICSD_164070	metal	metal
Cu2Nd1_ICSD_628499	metal	metal
Cu2Nd1_ICSD_656902	metal	metal
Cu2Ni1O2S2Sr2_ICSD_88424	metal	metal
Cu2Ni1Sn1_ICSD_151208	metal	metal
Cu2Ni1Zn1_ICSD_103079	metal	metal
Cu2O10P2U1_ICSD_59598	insulator	insulator
Cu2O1Ti4_ICSD_73017	metal	metal
Cu2O1_ICSD_180846	insulator	metal
Cu2O2Pb1_ICSD_400657	insulator	insulator
Cu2O2S2Sr2Zn1_ICSD_84735	insulator	metal
Cu2O2Sr1_ICSD_25002	insulator	metal
Cu2O2Tl1_ICSD_36535	insulator	insulator
Cu2O3Sr1_ICSD_416903	metal	metal
Cu2O3Sr1_ICSD_99042	metal	metal
Cu2O3Sr2_ICSD_68676	metal	insulator
Cu2O4P1_ICSD_80181	insulator	insulator
Cu2O4S1_ICSD_40452	insulator	insulator
Cu2O4Se1_ICSD_60652	insulator	insulator
Cu2O4Se1_ICSD_60653	insulator	insulator
Cu2O4W1_ICSD_202669	insulator	insulator
Cu2O4W1_ICSD_62058	insulator	insulator
Cu2O5S1_ICSD_34649	insulator	insulator
Cu2O7P2_ICSD_157107	insulator	insulator
Cu2O7P2_ICSD_27436	insulator	insulator
Cu2O7Sr2Tl1Y1_ICSD_74164	metal	metal
Cu2O7V2_ICSD_164189	insulator	insulator
Cu2O7V2_ICSD_171028	insulator	insulator
Cu2O7V2_ICSD_1831	insulator	insulator
Cu2O7V2_ICSD_34756	insulator	insulator
Cu2O8Te3_ICSD_50704	insulator	insulator
Cu2P2Th1_ICSD_100270	metal	metal

Supplementary Table 334. Five-fold cross validated predictions for the metal/insulator classification (326/598).

system	calculated	predicted
Cu2P2U1_ICSD_601802	metal	metal
Cu2P2Zr1_ICSD_35585	metal	metal
Cu2P7_ICSD_35281	insulator	insulator
Cu2Pb1S4Si1_ICSD_152763	insulator	insulator
Cu2Pr1Si2_ICSD_106876	metal	metal
Cu2Pr1Si2_ICSD_53325	metal	metal
Cu2Pr1Sn2_ICSD_628744	metal	metal
Cu2Pr1_ICSD_628707	metal	metal
Cu2Pu1Si2_ICSD_602699	metal	metal
Cu2Rb1S4V1_ICSD_280516	insulator	insulator
Cu2Rb2S4Sn1_ICSD_74020	insulator	insulator
Cu2Rb2S4Ti1_ICSD_280644	insulator	insulator
Cu2S13U6_ICSD_61417	insulator	insulator
Cu2S1_ICSD_16550	insulator	metal
Cu2S1_ICSD_20560	metal	metal
Cu2S1_ICSD_628818	metal	metal
Cu2S2Tb1_ICSD_628910	metal	metal
Cu2S2Tl1_ICSD_40495	metal	metal
Cu2S2Y1_ICSD_628962	metal	insulator
Cu2S2Yb1_ICSD_628970	insulator	metal
Cu2S3Si1_ICSD_24132	metal	insulator
Cu2S3Si1_ICSD_70057	insulator	metal
Cu2S3Si1_ICSD_88235	insulator	metal
Cu2S3Sn1_ICSD_107606	metal	metal
Cu2S4Si1Zn1_ICSD_628869	insulator	insulator
Cu2S4Sn1Sr1_ICSD_356	insulator	insulator
Cu2S4Sn1Sr1_ICSD_43131	insulator	insulator
Cu2S4Sn1Zn1_ICSD_184478	metal	metal
Cu2S4Sn1Zn1_ICSD_185597	metal	insulator
Cu2S4Sn1Zn1_ICSD_628895	insulator	metal
Cu2S4W1_ICSD_72529	insulator	insulator
Cu2S4W1_ICSD_98909	insulator	insulator
Cu2S7U3_ICSD_82155	insulator	insulator
Cu2Sb1_ICSD_412295	metal	metal
Cu2Sc1Si2_ICSD_60379	metal	metal
Cu2Sc1_ICSD_629012	metal	metal
Cu2Se1_ICSD_30230	metal	metal
Cu2Se1_ICSD_56025	metal	metal
Cu2Se2Tl1_ICSD_601713	metal	metal
Cu2Se3Si1_ICSD_88236	insulator	insulator
Cu2Se3Sn1_ICSD_97966	metal	metal
Cu2Se4Si1Zn1_ICSD_629079	insulator	insulator
Cu2Se4Sn1Zn1_ICSD_184474	metal	metal
Cu2Se4Sn1Zn1_ICSD_189278	metal	metal
Cu2Se7U3_ICSD_82156	insulator	insulator
Cu2Si1Te4Zn1_ICSD_656150	insulator	metal
Cu2Si2Sr1_ICSD_25313	metal	metal
Cu2Si2Tb1_ICSD_55784	metal	metal
Cu2Si2Th1_ICSD_629199	metal	metal
Cu2Si2Tm1_ICSD_53349	metal	metal

Supplementary Table 335. Five-fold cross validated predictions for the metal/insulator classification (327/598).

system	calculated	predicted
Cu2Si2U1_ICSD_629218	metal	metal
Cu2Si2Y1_ICSD_164066	metal	metal
Cu2Si2Yb1_ICSD_164078	metal	metal
Cu2Sn1Te3_ICSD_160882	metal	metal
Cu2Sn1Te4Zn1_ICSD_629293	metal	metal
Cu2Sn1U1_ICSD_657423	metal	metal
Cu2Sn2Sr1_ICSD_182102	metal	metal
Cu2Sn2U1_ICSD_602654	metal	metal
Cu2Sn2Y1_ICSD_603426	metal	metal
Cu2Sn5Yb4_ICSD_409510	metal	metal
Cu2Tb1_ICSD_241220	metal	metal
Cu2Te1_ICSD_655706	metal	metal
Cu2Te2Tl1_ICSD_629346	metal	metal
Cu2Te3Ti1_ICSD_402631	insulator	insulator
Cu2Te3Zr1_ICSD_56463	insulator	insulator
Cu2Th1_ICSD_15442	metal	metal
Cu2Y1_ICSD_604016	metal	metal
Cu2Yb1_ICSD_103147	metal	metal
Cu2Yb1_ICSD_629448	metal	metal
Cu2Zn1Zr1_ICSD_103161	metal	metal
Cu3Dy1Ga2_ICSD_108368	metal	metal
Cu3Dy1Mn4O12_ICSD_153871	metal	metal
Cu3Dy1O8Pb2Sr2_ICSD_71483	metal	metal
Cu3Dy3Sb4_ICSD_658643	insulator	insulator
Cu3Er1O8Pb2Sr2_ICSD_71485	metal	metal
Cu3Er1Te3_ICSD_154741	insulator	insulator
Cu3Er2K1Se5_ICSD_174372	insulator	insulator
Cu3Er2Rb1S5_ICSD_93682	insulator	insulator
Cu3Er2Rb1Se5_ICSD_96686	insulator	insulator
Cu3Er3Sb4_ICSD_658645	insulator	insulator
Cu3Eu1Mn4O12_ICSD_153869	metal	metal
Cu3Eu1O8Pb2Sr2_ICSD_71481	metal	metal
Cu3F4H2O2_ICSD_98699	insulator	insulator
Cu3F7Na1_ICSD_202587	insulator	insulator
Cu3Fe4La1O12_ICSD_185583	metal	metal
Cu3Fe4O12Sr1_ICSD_262857	metal	metal
Cu3Ga8V2_ICSD_416815	metal	metal
Cu3Ga8W2_ICSD_416814	metal	metal
Cu3Gd1Mn4O12_ICSD_153870	insulator	metal
Cu3Gd1O8Pb2Sr2_ICSD_71482	metal	metal
Cu3Gd3Sb4_ICSD_658641	insulator	insulator
Cu3Ge1_ICSD_53266	metal	metal
Cu3Ge1_ICSD_627687	metal	metal
Cu3Ge4Na2O12_ICSD_171630	metal	insulator
Cu3Ge5K2O14_ICSD_410828	insulator	insulator
Cu3H6O11V2_ICSD_68994	metal	insulator
Cu3Ho1Mn4O12_ICSD_153872	metal	metal
Cu3Ho1O8Pb2Sr2_ICSD_71484	metal	metal
Cu3Ho2K1Se5_ICSD_174371	insulator	insulator
Cu3Ho2Rb1S5_ICSD_59858	insulator	insulator

Supplementary Table 336. Five-fold cross validated predictions for the metal/insulator classification (328/598).

system	calculated	predicted
Cu3Ho3Sb4_ICSD_658644	insulator	insulator
Cu3I2O4Sr2_ICSD_55711	metal	metal
Cu3In2O16P4_ICSD_411872	metal	insulator
Cu3K1S2_ICSD_100001	insulator	insulator
Cu3K1Te2_ICSD_100830	insulator	metal
Cu3K2O12Se4_ICSD_50541	insulator	insulator
Cu3K3P2_ICSD_12163	insulator	insulator
Cu3La1O12Ru4_ICSD_51897	metal	metal
Cu3La3Sb4_ICSD_658637	metal	metal
Cu3Lu1Mn4O12_ICSD_180106	metal	metal
Cu3Mg2Si1_ICSD_53304	metal	metal
Cu3Mn4O12Pr1_ICSD_153867	metal	metal
Cu3Mn4O12Th1_ICSD_34316	metal	metal
Cu3Mn4O12Tm1_ICSD_153873	metal	metal
Cu3Mn4O12Y1_ICSD_38418	metal	metal
Cu3Mn4O12Yb1_ICSD_153874	metal	metal
Cu3N1Pd0.25_ICSD_180239	metal	metal
Cu3N1_ICSD_167838	insulator	metal
Cu3N1_ICSD_180236	metal	metal
Cu3N1_ICSD_180237	metal	metal
Cu3N1_ICSD_55222	metal	metal
Cu3N5Sr6_ICSD_83962	insulator	insulator
Cu3Na1O12Ru4_ICSD_95716	metal	metal
Cu3Na1Te2_ICSD_60860	metal	metal
Cu3Nb1S4_ICSD_183994	insulator	insulator
Cu3Nb1Se4_ICSD_628485	insulator	insulator
Cu3Nb1Te4_ICSD_628495	insulator	insulator
Cu3Nd1O12Ru4_ICSD_51896	metal	metal
Cu3Nd1O8Pb2Sr2_ICSD_71479	metal	metal
Cu3Nd3Sb4_ICSD_57207	metal	metal
Cu3Nd3Sb4_ICSD_658635	metal	metal
Cu3O12Pb1Sn4_ICSD_162102	insulator	insulator
Cu3O12Ru4Sr1_ICSD_51895	metal	metal
Cu3O12Sn4Sr1_ICSD_162101	insulator	insulator
Cu3O1Ti3_ICSD_29056	metal	metal
Cu3O2S2Sr2_ICSD_88423	metal	metal
Cu3O4V1_ICSD_418372	insulator	insulator
Cu3O5Sr2_ICSD_416904	metal	metal
Cu3O6Te1_ICSD_189131	insulator	insulator
Cu3O6W1_ICSD_15001	insulator	insulator
Cu3O8Pb2Sr2Y1_ICSD_74154	metal	metal
Cu3O8V2_ICSD_27184	insulator	insulator
Cu3O8V2_ICSD_27310	insulator	insulator
Cu3P1S4_ICSD_412240	insulator	insulator
Cu3P1Se4_ICSD_95412	insulator	insulator
Cu3P1_ICSD_16247	metal	metal
Cu3P1_ICSD_26775	metal	metal
Cu3P1_ICSD_628628	metal	metal
Cu3Pd1_ICSD_103083	metal	metal
Cu3Pd1_ICSD_103084	metal	metal

Supplementary Table 337. Five-fold cross validated predictions for the metal/insulator classification (329/598).

system	calculated	predicted
Cu3Pd1_ICSD_103086	metal	metal
Cu3Pd1_ICSD_107169	metal	metal
Cu3Pr3Sb4_ICSD_658639	metal	metal
Cu3Pt1_ICSD_628749	metal	metal
Cu3Rb1S2_ICSD_409646	insulator	insulator
Cu3S2Tl1_ICSD_23290	insulator	insulator
Cu3S3Sb1_ICSD_403113	insulator	insulator
Cu3S3Sb1_ICSD_53332	metal	metal
Cu3S4Sb1_ICSD_30368	metal	metal
Cu3S4Sb1_ICSD_42672	metal	insulator
Cu3S4Sb1_ICSD_42673	metal	metal
Cu3S4Ta1_ICSD_183995	insulator	insulator
Cu3S4V1_ICSD_15490	insulator	insulator
Cu3S8Ta2Tl3_ICSD_406210	insulator	insulator
Cu3Sb1Se3_ICSD_401095	insulator	insulator
Cu3Sb1Se4_ICSD_628993	metal	metal
Cu3Sb1_ICSD_53337	metal	metal
Cu3Sb1_ICSD_628990	metal	metal
Cu3Sb4Tb3_ICSD_658642	insulator	insulator
Cu3Sb4U3_ICSD_657096	metal	metal
Cu3Sb4Y3_ICSD_658636	insulator	metal
Cu3Se2Tl1_ICSD_629126	insulator	metal
Cu3Se2_ICSD_239	metal	metal
Cu3Se4Ta1_ICSD_108406	insulator	insulator
Cu3Se4V1_ICSD_629148	insulator	insulator
Cu3Sn1_ICSD_103103	metal	metal
Cu3Sn1_ICSD_162569	metal	metal
Cu3Sn1_ICSD_629268	metal	metal
Cu3Sn4U3_ICSD_629298	metal	metal
Cu3Ta1Te4_ICSD_629308	insulator	insulator
Cu3Te4V1_ICSD_629351	insulator	insulator
Cu3Ti1_ICSD_600128	metal	metal
Cu3Ti2_ICSD_629380	metal	metal
Cu4Dy1In1_ICSD_627150	metal	metal
Cu4Dy3Ge4_ICSD_627142	metal	metal
Cu4Dy3Si4_ICSD_55837	metal	metal
Cu4Dy3Sn4_ICSD_604589	metal	metal
Cu4Dy4K2S9_ICSD_97562	insulator	insulator
Cu4Dy4K2Se9_ICSD_174374	insulator	insulator
Cu4Er1Pd1_ICSD_627234	metal	metal
Cu4Er3Ge4_ICSD_98338	metal	metal
Cu4Er3Si4_ICSD_55650	metal	metal
Cu4Er3Sn4_ICSD_604569	metal	metal
Cu4Eu1Sn2_ICSD_416796	metal	metal
Cu4Gd1In1_ICSD_627611	metal	metal
Cu4Ge2Hf3_ICSD_53269	metal	metal
Cu4Ge2Ni1S7_ICSD_627755	metal	insulator
Cu4Ge2Zr3_ICSD_53286	metal	metal
Cu4Ge4Ho3_ICSD_98336	metal	metal
Cu4Ge4Sc3_ICSD_604581	metal	metal

Supplementary Table 338. Five-fold cross validated predictions for the metal/insulator classification (330/598).

system	calculated	predicted
Cu4Ge4Tb3_ICSD_98331	metal	metal
Cu4Ge4Y3_ICSD_627876	metal	metal
Cu4Ge4Yb3_ICSD_627879	metal	metal
Cu4Hf3Si2_ICSD_53288	metal	metal
Cu4Hf3Si4_ICSD_87201	metal	metal
Cu4Ho3Si4_ICSD_627979	metal	metal
Cu4Ho3Sn4_ICSD_627987	metal	metal
Cu4Ho4K2S9_ICSD_97563	insulator	insulator
Cu4In1Mg1_ICSD_628018	metal	metal
Cu4In1Mn1_ICSD_424277	metal	metal
Cu4In1Sc1_ICSD_416528	metal	metal
Cu4In1Tb1_ICSD_152559	metal	metal
Cu4In1Y1_ICSD_628179	metal	metal
Cu4In1Yb1_ICSD_628189	metal	metal
Cu4In9Se16_ICSD_53295	metal	insulator
Cu4K1S3_ICSD_23336	metal	metal
Cu4K1Sb2_ICSD_602179	metal	metal
Cu4K1Se3_ICSD_280072	metal	metal
Cu4K2Se9Y4_ICSD_174375	insulator	insulator
Cu4Mg1Sn1_ICSD_108141	metal	metal
Cu4Mg1Y1_ICSD_163696	metal	metal
Cu4Mn1Sn1_ICSD_628407	metal	metal
Cu4Mo6Se8_ICSD_171431	metal	metal
Cu4Na1O12Te2Tl3_ICSD_421466	insulator	insulator
Cu4Na1S4_ICSD_81306	metal	metal
Cu4Na1Sb2_ICSD_59206	metal	metal
Cu4Na2S3_ICSD_100829	insulator	metal
Cu4Na3S4_ICSD_10004	metal	insulator
Cu4Nb5Si4_ICSD_25756	metal	metal
Cu4Ni1S7Si2_ICSD_100778	insulator	insulator
Cu4Ni1Yb1_ICSD_628598	metal	metal
Cu4O3_ICSD_100566	metal	metal
Cu4P8Si1_ICSD_78967	insulator	insulator
Cu4Pd1U1_ICSD_658161	metal	metal
Cu4Pd1Yb1_ICSD_628704	metal	metal
Cu4Pd1_ICSD_103087	metal	metal
Cu4Rb1S3_ICSD_628758	metal	metal
Cu4Rb1Se3_ICSD_628760	metal	metal
Cu4S3Tl1_ICSD_32607	metal	metal
Cu4S4Sn1_ICSD_833	metal	insulator
Cu4S4Ti1_ICSD_82558	insulator	metal
Cu4Sc1Sn1_ICSD_629023	metal	metal
Cu4Sc3Si4_ICSD_629021	metal	metal
Cu4Se3Tl1_ICSD_629128	metal	metal
Cu4Si2Zr3_ICSD_26260	metal	metal
Cu4Si4Tb3_ICSD_629185	metal	metal
Cu4Si4Y3_ICSD_629226	metal	metal
Cu4Si4Yb3_ICSD_629232	metal	metal
Cu4Si4Zr3_ICSD_87200	metal	metal
Cu4Sn2Sr1_ICSD_422455	metal	metal

Supplementary Table 339. Five-fold cross validated predictions for the metal/insulator classification (331/598).

system	calculated	predicted
Cu4Sn4Tb3_ICSD_162827	metal	metal
Cu4Sn4Y3_ICSD_604582	metal	metal
Cu4Ti1_ICSD_629401	metal	metal
Cu5Dy1_ICSD_102875	metal	metal
Cu5Er1_ICSD_627207	metal	metal
Cu5Eu1_ICSD_627265	metal	metal
Cu5Gd1_ICSD_102950	metal	metal
Cu5Gd1_ICSD_627590	metal	metal
Cu5Gd1_ICSD_659698	metal	metal
Cu5H4O12V2_ICSD_54831	insulator	insulator
Cu5Ho1_ICSD_102978	metal	metal
Cu5Ho1_ICSD_604234	metal	metal
Cu5In1Th1_ICSD_107268	metal	metal
Cu5K1O13V3_ICSD_400802	insulator	insulator
Cu5K2Te5_ICSD_71204	metal	insulator
Cu5La1_ICSD_628218	metal	metal
Cu5Li2O14Si4_ICSD_2409	insulator	insulator
Cu5Lu1_ICSD_103045	metal	metal
Cu5Na1S3_ICSD_61514	metal	insulator
Cu5Na2O14Si4_ICSD_416179	insulator	insulator
Cu5Na8O10_ICSD_415349	insulator	insulator
Cu5Nd1_ICSD_659711	metal	metal
Cu5O10P2_ICSD_1292	insulator	insulator
Cu5O4Rb3_ICSD_35246	insulator	insulator
Cu5Pr1_ICSD_103088	metal	metal
Cu5S7Si2_ICSD_628848	metal	insulator
Cu5Sn1Th1_ICSD_107269	metal	metal
Cu5Sn1U1_ICSD_54541	metal	metal
Cu5Sn4_ICSD_187904	metal	metal
Cu5Sr1_ICSD_103113	metal	metal
Cu5Tb1_ICSD_108415	metal	metal
Cu5Tb1_ICSD_629316	metal	metal
Cu5Tm1_ICSD_103140	metal	metal
Cu5U1_ICSD_629421	metal	metal
Cu5Y1_ICSD_629436	metal	metal
Cu5Yb1_ICSD_103149	metal	metal
Cu5Zn8_ICSD_103158	metal	metal
Cu5Zr1_ICSD_103165	metal	metal
Cu6Fe2S8Sn1_ICSD_40047	metal	insulator
Cu6Ga21Li13_ICSD_106534	metal	metal
Cu6Ga5Mg2_ICSD_240092	metal	metal
Cu6Gd1_ICSD_163977	metal	metal
Cu6La1_ICSD_103032	metal	metal
Cu6La1_ICSD_103033	metal	metal
Cu6La4Mn1S10_ICSD_96415	insulator	insulator
Cu6Nd1_ICSD_150110	metal	metal
Cu6Nd1_ICSD_628509	metal	metal
Cu6O10Sr4_ICSD_50089	metal	metal
Cu6O8Pb1_ICSD_27585	metal	insulator
Cu6O8Pb1_ICSD_280596	insulator	metal

Supplementary Table 340. Five-fold cross validated predictions for the metal/insulator classification (332/598).

system	calculated	predicted
Cu6Pr1_ICSD_628709	metal	metal
Cu6Pu1_ICSD_628756	metal	metal
Cu6Sn5Yb3_ICSD_413485	metal	metal
Cu6Sn5_ICSD_158248	metal	metal
Cu6Th1_ICSD_103125	metal	metal
Cu7Hf6Zn16_ICSD_627923	metal	metal
Cu7P1S6_ICSD_628639	insulator	insulator
Cu7P1Se6_ICSD_628648	insulator	insulator
Cu7S4_ICSD_16011	metal	insulator
Cu7Te4_ICSD_629337	metal	metal
Cu7Zn16Zr6_ICSD_629468	metal	metal
Cu8Ge8Nd6_ICSD_83190	metal	metal
Cu8Hf3_ICSD_396	metal	metal
Cu8K3S6_ICSD_10054	metal	metal
Cu8K3Se6_ICSD_85728	metal	metal
Cu8O1_ICSD_62764	metal	metal
Cu8Rb3S6_ICSD_10055	metal	metal
Cu8Rb3Se6_ICSD_14011	metal	metal
Cu8S6Si1_ICSD_24374	insulator	metal
Cu9Dy1Mg2_ICSD_245216	metal	metal
Cu9Fe9S16_ICSD_2649	metal	metal
Cu9Ga4_ICSD_627389	metal	metal
Cu9Ge4Sr1_ICSD_412884	metal	metal
Cu9Ho1Mg2_ICSD_245217	metal	metal
Cu9In4_ICSD_187898	metal	metal
Cu9La1Mg2_ICSD_245209	metal	metal
Cu9Mg2Nd1_ICSD_245212	metal	metal
Cu9Mg2Pr1_ICSD_245211	metal	metal
Cu9Mg2Tb1_ICSD_416327	metal	metal
Cu9Mg2Y1_ICSD_245208	metal	metal
Cu9Mg2Yb1_ICSD_245218	metal	metal
Cu9Nd1Sn4_ICSD_107250	metal	metal
Cu9S5_ICSD_41263	metal	metal
Cu9Si4Sr1_ICSD_412882	metal	metal
Cu9Sn4Sr1_ICSD_422456	metal	metal
Cu9Sn4Yb1_ICSD_154446	metal	metal
Dy11Sn10_ICSD_103372	metal	metal
Dy12Fe32O2_ICSD_9639	metal	metal
Dy1F1O1_ICSD_184010	insulator	insulator
Dy1Fe1O3_ICSD_280091	insulator	insulator
Dy1Fe1Si1_ICSD_629664	metal	metal
Dy1Fe2Ge2_ICSD_103175	metal	metal
Dy1Fe2Si2_ICSD_629657	metal	metal
Dy1Fe2_ICSD_629613	metal	metal
Dy1Fe3_ICSD_629569	metal	metal
Dy1Fe3_ICSD_629573	metal	metal
Dy1Fe3_ICSD_629588	metal	metal
Dy1Fe4Ge2_ICSD_154830	metal	metal
Dy1Fe4Ge2_ICSD_154831	metal	metal
Dy1Fe4Si2_ICSD_247480	metal	metal

Supplementary Table 341. Five-fold cross validated predictions for the metal/insulator classification (333/598).

system	calculated	predicted
Dy1Fe5_ICSD_103173	metal	metal
Dy1Fe6Ge6_ICSD_656771	metal	metal
Dy1Fe6Sn6_ICSD_151163	metal	metal
Dy1Ga1Ni1_ICSD_629716	metal	metal
Dy1Ga1Pd1_ICSD_629728	metal	metal
Dy1Ga1Pd2_ICSD_656966	metal	metal
Dy1Ga1Pt1_ICSD_629731	metal	metal
Dy1Ga1Zn1_ICSD_629739	metal	metal
Dy1Ga1_ICSD_103184	metal	metal
Dy1Ga2Ni1_ICSD_629726	metal	metal
Dy1Ga2Pd1_ICSD_600150	metal	metal
Dy1Ga2_ICSD_657002	metal	metal
Dy1Ga3Ni2_ICSD_106968	metal	metal
Dy1Ga3Ru1_ICSD_629734	metal	metal
Dy1Ga3_ICSD_103187	metal	metal
Dy1Ga3_ICSD_629703	metal	metal
Dy1Ga3_ICSD_629704	metal	metal
Dy1Ga4Ni1_ICSD_423065	metal	metal
Dy1Ge1Ir1_ICSD_88165	metal	metal
Dy1Ge1Li1_ICSD_601244	metal	metal
Dy1Ge1Mn1_ICSD_97794	metal	metal
Dy1Ge1Ni1_ICSD_657256	metal	metal
Dy1Ge1Pd1_ICSD_391464	metal	metal
Dy1Ge1Pt1_ICSD_90294	metal	metal
Dy1Ge1Rh1_ICSD_629803	metal	metal
Dy1Ge1Ru1_ICSD_85914	metal	metal
Dy1Ge1Sn1_ICSD_261753	metal	metal
Dy1Ge1Ti1_ICSD_85907	metal	metal
Dy1Ge1Ti1_ICSD_93644	metal	metal
Dy1Ge1_ICSD_53360	metal	metal
Dy1Ge2Ir1_ICSD_600505	metal	metal
Dy1Ge2Mn2_ICSD_629783	metal	metal
Dy1Ge2Ni1_ICSD_658575	metal	metal
Dy1Ge2Ni2_ICSD_629792	metal	metal
Dy1Ge2Pd2_ICSD_53363	metal	metal
Dy1Ge2Pt1_ICSD_66977	metal	metal
Dy1Ge2Pt2_ICSD_53364	metal	metal
Dy1Ge2Pt2_ICSD_629802	metal	metal
Dy1Ge2Rh2_ICSD_55930	metal	metal
Dy1Ge2Ru2_ICSD_55931	metal	metal
Dy1Ge6Mn6_ICSD_57228	metal	metal
Dy1H2_ICSD_629826	metal	metal
Dy1H3O3_ICSD_200095	insulator	insulator
Dy1H3_ICSD_629827	metal	insulator
Dy1H4K1O9P2_ICSD_167522	insulator	insulator
Dy1Hg1_ICSD_629835	metal	metal
Dy1Hg2_ICSD_103196	metal	metal
Dy1Hg2_ICSD_629834	metal	metal
Dy1Hg3_ICSD_629833	metal	metal
Dy1I1S1_ICSD_79107	insulator	insulator

Supplementary Table 342. Five-fold cross validated predictions for the metal/insulator classification (334/598).

system	calculated	predicted
Dy1In1Ir1_ICSD_414472	metal	metal
Dy1In1Mg1_ICSD_55102	metal	metal
Dy1In1Ni1_ICSD_629861	metal	metal
Dy1In1Pd1_ICSD_629863	metal	metal
Dy1In1Pd2_ICSD_103207	metal	metal
Dy1In1Pt1_ICSD_154892	metal	metal
Dy1In1Pt2_ICSD_629865	metal	metal
Dy1In1Pt4_ICSD_629864	metal	metal
Dy1In1Rh1_ICSD_103209	metal	metal
Dy1In1Zn1_ICSD_629871	metal	metal
Dy1In1_ICSD_103201	metal	metal
Dy1In2Ni1_ICSD_629858	metal	metal
Dy1In2Ni9_ICSD_600171	metal	metal
Dy1In3_ICSD_629856	metal	metal
Dy1In5Rh1_ICSD_155804	metal	metal
Dy1Ir1Si1_ICSD_93218	metal	metal
Dy1Ir1Si3_ICSD_629884	metal	metal
Dy1Ir2Si2_ICSD_57230	metal	metal
Dy1Ir2Si2_ICSD_629888	metal	metal
Dy1Ir2_ICSD_629882	metal	metal
Dy1Ir3_ICSD_103213	metal	metal
Dy1K1S2_ICSD_44946	insulator	insulator
Dy1Li1O12P4_ICSD_260001	insulator	insulator
Dy1Li1O2_ICSD_422562	insulator	insulator
Dy1Li1O2_ICSD_45512	insulator	insulator
Dy1Li1S2_ICSD_44958	insulator	insulator
Dy1Li1Se2_ICSD_44964	insulator	insulator
Dy1Li1Sn1_ICSD_603144	metal	metal
Dy1Li1Sn2_ICSD_629904	metal	metal
Dy1Mg1Pd1_ICSD_657929	metal	metal
Dy1Mg1Sn1_ICSD_183524	metal	metal
Dy1Mg1_ICSD_629912	metal	metal
Dy1Mg2_ICSD_656103	metal	metal
Dy1Mg3_ICSD_656104	metal	metal
Dy1Mn12_ICSD_26824	metal	metal
Dy1Mn1O3_ICSD_157396	metal	insulator
Dy1Mn1O3_ICSD_91711	insulator	metal
Dy1Mn1Si1_ICSD_88061	metal	metal
Dy1Mn2O5_ICSD_18308	insulator	insulator
Dy1Mn2Si2_ICSD_629944	metal	metal
Dy1Mn2_ICSD_163470	metal	metal
Dy1Mn2_ICSD_602151	metal	metal
Dy1Mo6S8_ICSD_629953	metal	metal
Dy1Mo6Se8_ICSD_629954	metal	metal
Dy1N1_ICSD_629962	insulator	metal
Dy1Na1S2_ICSD_629966	insulator	insulator
Dy1Na1Se2_ICSD_629969	insulator	insulator
Dy1Ni10Si2_ICSD_97036	metal	metal
Dy1Ni1P1_ICSD_630012	metal	metal
Dy1Ni1Sb1_ICSD_103339	insulator	insulator

Supplementary Table 343. Five-fold cross validated predictions for the metal/insulator classification (335/598).

system	calculated	predicted
Dy1Ni1Sb2_ICSD_658215	metal	metal
Dy1Ni1Si2_ICSD_85839	metal	metal
Dy1Ni1Sn1_ICSD_103340	metal	metal
Dy1Ni1Zn1_ICSD_656586	metal	metal
Dy1Ni1_ICSD_109242	metal	metal
Dy1Ni2P2_ICSD_88184	metal	metal
Dy1Ni2Sb2_ICSD_630016	metal	metal
Dy1Ni2Si2_ICSD_630029	metal	metal
Dy1Ni2_ICSD_629995	metal	metal
Dy1Ni3_ICSD_629980	metal	metal
Dy1Ni3_ICSD_630007	metal	metal
Dy1Ni4P2_ICSD_601112	metal	metal
Dy1Ni5_ICSD_629994	metal	metal
Dy1O14P5_ICSD_240973	insulator	insulator
Dy1O19Ta7_ICSD_203232	insulator	insulator
Dy1O1P1Zn1_ICSD_418526	insulator	insulator
Dy1O2Rb1_ICSD_15159	insulator	insulator
Dy1O4P1_ICSD_79756	insulator	insulator
Dy1O4Ta1_ICSD_415435	insulator	insulator
Dy1O4V1_ICSD_247716	insulator	insulator
Dy1O4V1_ICSD_37365	insulator	insulator
Dy1O6Re1Sr2_ICSD_25402	metal	metal
Dy1O6Rh1Sr3_ICSD_51055	insulator	insulator
Dy1O8Rb1S2_ICSD_20896	insulator	insulator
Dy1O8Rb1W2_ICSD_22361	insulator	insulator
Dy1O9P3_ICSD_416844	insulator	insulator
Dy1Os2Si2_ICSD_630053	metal	metal
Dy1Os2_ICSD_630052	metal	metal
Dy1P1Pt1_ICSD_53373	metal	metal
Dy1P1S1_ICSD_630062	insulator	insulator
Dy1P1_ICSD_187519	metal	metal
Dy1P1_ICSD_630056	metal	metal
Dy1P2Pt8_ICSD_418227	metal	metal
Dy1P2Ru2_ICSD_602101	metal	metal
Dy1P5_ICSD_409184	insulator	metal
Dy1Pb1Pd1_ICSD_657945	metal	metal
Dy1Pb3_ICSD_630066	metal	metal
Dy1Pd1Sb1_ICSD_415945	metal	metal
Dy1Pd1Sb1_ICSD_630088	metal	metal
Dy1Pd1Sn1_ICSD_103348	metal	metal
Dy1Pd1Tl1_ICSD_103353	metal	metal
Dy1Pd1Zn1_ICSD_183341	metal	metal
Dy1Pd1_ICSD_106595	metal	metal
Dy1Pd1_ICSD_630079	metal	metal
Dy1Pd2Si1_ICSD_630089	metal	metal
Dy1Pd2Si2_ICSD_71250	metal	metal
Dy1Pd2Sn1_ICSD_103349	metal	metal
Dy1Pd3_ICSD_103344	metal	metal
Dy1Pt1Sb1_ICSD_53374	insulator	metal
Dy1Pt1Si1_ICSD_630134	metal	metal

Supplementary Table 344. Five-fold cross validated predictions for the metal/insulator classification (336/598).

system	calculated	predicted
Dy1Pt1Sn1_ICSD_630137	metal	metal
Dy1Pt1_ICSD_630123	metal	metal
Dy1Pt2Si1_ICSD_630131	metal	metal
Dy1Pt2Si2_ICSD_601620	metal	metal
Dy1Pt2Si2_ICSD_604564	metal	metal
Dy1Pt2_ICSD_103355	metal	metal
Dy1Pt3_ICSD_103356	metal	metal
Dy1Rb1S2_ICSD_81402	insulator	insulator
Dy1Re2_ICSD_630138	metal	metal
Dy1Rh1Sn1_ICSD_630165	metal	metal
Dy1Rh1Zn1_ICSD_419897	metal	metal
Dy1Rh1_ICSD_103360	metal	metal
Dy1Rh2Si2_ICSD_630161	metal	metal
Dy1Rh2Zn20_ICSD_152113	metal	metal
Dy1Rh2_ICSD_103362	metal	metal
Dy1Rh3Si2_ICSD_103364	metal	metal
Dy1Rh5_ICSD_103363	metal	metal
Dy1Ru1Si1_ICSD_88065	metal	metal
Dy1Ru2Si2_ICSD_630182	metal	metal
Dy1Ru2Zn20_ICSD_152111	metal	metal
Dy1Ru2_ICSD_630179	metal	metal
Dy1S1_ICSD_57239	metal	metal
Dy1S2Tl1_ICSD_57244	insulator	insulator
Dy1S2_ICSD_53378	metal	metal
Dy1S2_ICSD_630195	metal	metal
Dy1Sb1Sc1_ICSD_421440	metal	metal
Dy1Sb1Zr1_ICSD_152640	metal	metal
Dy1Sb1_ICSD_630240	metal	metal
Dy1Sb2_ICSD_630230	metal	metal
Dy1Se1_ICSD_53380	metal	metal
Dy1Se1_ICSD_53381	metal	metal
Dy1Se2Tl1_ICSD_106991	insulator	insulator
Dy1Se2_ICSD_630258	metal	metal
Dy1Si1.7_ICSD_415597	metal	metal
Dy1Si1Ti1_ICSD_88116	metal	metal
Dy1Si1_ICSD_164012	metal	metal
Dy1Si1_ICSD_165434	metal	metal
Dy1Si1_ICSD_180212	metal	metal
Dy1Si1_ICSD_180224	metal	metal
Dy1Si1_ICSD_180225	metal	metal
Dy1Si2_ICSD_150663	metal	metal
Dy1Si2_ICSD_20248	metal	metal
Dy1Sn2_ICSD_168674	metal	metal
Dy1Sn3_ICSD_103370	metal	metal
Dy1Tc2_ICSD_630325	metal	metal
Dy1Te1_ICSD_43640	metal	metal
Dy1Te2Tl1_ICSD_630345	insulator	insulator
Dy1Te2_ICSD_630332	metal	metal
Dy1Th1_ICSD_109244	metal	metal
Dy1Tl1Zn1_ICSD_630365	metal	metal

Supplementary Table 345. Five-fold cross validated predictions for the metal/insulator classification (337/598).

system	calculated	predicted
Dy1Tl1_ICSD_103375	metal	metal
Dy1Tl3_ICSD_103376	metal	metal
Dy1Zn12_ICSD_630387	metal	metal
Dy1Zn1_ICSD_630374	metal	metal
Dy1Zn2_ICSD_630384	metal	metal
Dy1Zn3_ICSD_103382	metal	metal
Dy1Zn5_ICSD_103383	metal	metal
Dy1_ICSD_157920	metal	metal
Dy1_ICSD_52518	metal	metal
Dy1_ICSD_53356	metal	metal
Dy1_ICSD_53358	metal	metal
Dy1_ICSD_629536	metal	metal
Dy2Fe17N3_ICSD_629636	metal	metal
Dy2Fe17_ICSD_604297	metal	metal
Dy2Fe3Si5_ICSD_41835	metal	metal
Dy2Fe3Si5_ICSD_629666	metal	metal
Dy2Ga9Ru3_ICSD_411437	metal	metal
Dy2Ge2In1_ICSD_172492	metal	metal
Dy2Ge2Mg1_ICSD_423452	metal	metal
Dy2Ge3Pt9_ICSD_415206	metal	metal
Dy2Hf2O7_ICSD_245138	insulator	insulator
Dy2In1Ni2_ICSD_629859	metal	metal
Dy2In1Ni2_ICSD_629860	metal	metal
Dy2In1Pd2_ICSD_658298	metal	metal
Dy2In1_ICSD_103205	metal	metal
Dy2Mg1Si2_ICSD_415117	metal	metal
Dy2Mo3Si4_ICSD_600648	metal	metal
Dy2Mo4O18Sb2_ICSD_417674	insulator	insulator
Dy2N3_ICSD_629957	metal	metal
Dy2Ni12P7_ICSD_602458	metal	metal
Dy2Ni17_ICSD_630000	metal	metal
Dy2Ni3Si5_ICSD_84170	metal	metal
Dy2Ni7_ICSD_629978	metal	metal
Dy2O13Te5_ICSD_413664	insulator	insulator
Dy2O1S2_ICSD_36603	insulator	insulator
Dy2O1S2_ICSD_36604	insulator	insulator
Dy2O2S1_ICSD_109332	insulator	insulator
Dy2O2Te1_ICSD_89565	insulator	insulator
Dy2O3_ICSD_160213	insulator	insulator
Dy2O3_ICSD_160228	insulator	insulator
Dy2O3_ICSD_185606	insulator	insulator
Dy2O7Ru2_ICSD_78151	insulator	insulator
Dy2O7Sn2_ICSD_82960	insulator	insulator
Dy2O7Tc2_ICSD_109080	insulator	insulator
Dy2O7Ti2_ICSD_188553	insulator	insulator
Dy2O7V2_ICSD_160863	insulator	insulator
Dy2Pb1Pd2_ICSD_99196	metal	metal
Dy2Pt1_ICSD_630109	metal	metal
Dy2Rh1Si3_ICSD_630163	metal	metal
Dy2Rh3Si5_ICSD_35134	metal	metal

Supplementary Table 346. Five-fold cross validated predictions for the metal/insulator classification (338/598).

system	calculated	predicted
Dy2Rh3Si5_ICSD_600471	metal	metal
Dy2S3_ICSD_66532	insulator	insulator
Dy2S4Sr1_ICSD_630212	insulator	insulator
Dy2S4Zn1_ICSD_630225	insulator	insulator
Dy2S5Sn1_ICSD_630211	metal	insulator
Dy2Sb5_ICSD_107594	metal	metal
Dy2Se3_ICSD_419	insulator	insulator
Dy2Se4Sr1_ICSD_630270	insulator	insulator
Dy2Si4Ti3_ICSD_96129	metal	metal
Dy2Sn5_ICSD_160543	metal	metal
Dy2Tl1_ICSD_103378	metal	metal
Dy2Zn17_ICSD_103384	metal	metal
Dy2Zn17_ICSD_630377	metal	metal
Dy3F1O12Se4_ICSD_411748	insulator	insulator
Dy3Fe1Ga1S7_ICSD_236399	insulator	insulator
Dy3Fe1Ga1Se7_ICSD_236400	insulator	insulator
Dy3Fe2Si3_ICSD_84008	metal	metal
Dy3Fe5O12_ICSD_23856	insulator	insulator
Dy3Ga1O6_ICSD_99495	insulator	insulator
Dy3Ga2Mn3Si1_ICSD_99076	metal	metal
Dy3Ga2Ni6_ICSD_629715	metal	metal
Dy3Ga5O12_ICSD_409391	insulator	insulator
Dy3Ge13Ir4_ICSD_629774	metal	metal
Dy3Ge13Os4_ICSD_629795	metal	metal
Dy3Ge13Rh4_ICSD_629805	metal	metal
Dy3Ge13Ru4_ICSD_629815	metal	metal
Dy3Ge3Ru2_ICSD_425772	metal	metal
Dy3Ge4_ICSD_84342	metal	metal
Dy3Ge5_ICSD_419458	metal	metal
Dy3In1N1_ICSD_98504	metal	metal
Dy3In1_ICSD_108423	metal	metal
Dy3In5_ICSD_629850	metal	metal
Dy3Ir1_ICSD_629872	metal	metal
Dy3N1S3_ICSD_416219	insulator	insulator
Dy3N1Se3_ICSD_420156	insulator	insulator
Dy3Ni1Si2_ICSD_630021	metal	metal
Dy3Ni1_ICSD_629977	metal	metal
Dy3Ni2_ICSD_2334	metal	metal
Dy3Ni6Si2_ICSD_630030	metal	metal
Dy3O7Re1_ICSD_99254	insulator	insulator
Dy3Os1_ICSD_630049	metal	metal
Dy3Pd2_ICSD_107682	metal	metal
Dy3Pd4_ICSD_630078	metal	metal
Dy3Pt1_ICSD_630113	metal	metal
Dy3Pt4_ICSD_630122	metal	metal
Dy3Rh1_ICSD_630158	metal	metal
Dy3Ru1_ICSD_630178	metal	metal
Dy3S4_ICSD_630201	metal	metal
Dy3S7Sc2_ICSD_630205	metal	metal
Dy3Se4_ICSD_630261	metal	metal

Supplementary Table 347. Five-fold cross validated predictions for the metal/insulator classification (339/598).

system	calculated	predicted
Dy3Tl5_ICSD_630360	metal	metal
Dy3Zn11_ICSD_630370	metal	metal
Dy4Fe1Ga12_ICSD_249998	metal	metal
Dy4Ga12Ni1_ICSD_629724	metal	metal
Dy4Ga12Pd1_ICSD_629729	metal	metal
Dy4Ge4In1Ni2_ICSD_240335	metal	metal
Dy4Ge6Ir7_ICSD_629773	metal	metal
Dy4Ge6Rh7_ICSD_629804	metal	metal
Dy4In1Rh1_ICSD_417514	metal	metal
Dy4Mg1Rh1_ICSD_417454	metal	metal
Dy4Ni12Sn25_ICSD_160045	metal	metal
Dy4Sb3_ICSD_630245	metal	metal
Dy5Ge3_ICSD_629755	metal	metal
Dy5In4Ni2_ICSD_418923	metal	metal
Dy5In4Pd2_ICSD_165135	metal	metal
Dy5Ir2_ICSD_629875	metal	metal
Dy5Ir3_ICSD_629876	metal	metal
Dy5Ir3_ICSD_629878	metal	metal
Dy5Ir4Si10_ICSD_629891	metal	metal
Dy5Mg24_ICSD_109137	metal	metal
Dy5Ni1Pb3_ICSD_152604	metal	metal
Dy5Ni2Sb1_ICSD_81765	metal	metal
Dy5Pb3_ICSD_103342	metal	metal
Dy5Pt3_ICSD_630107	metal	metal
Dy5Rh3_ICSD_630149	metal	metal
Dy5Ru2_ICSD_103366	metal	metal
Dy5S7_ICSD_630189	metal	metal
Dy5Sb3_ICSD_601792	metal	metal
Dy5Si3_ICSD_630292	metal	metal
Dy5Sn3_ICSD_103371	metal	metal
Dy5Tl3_ICSD_630359	metal	metal
Dy5Tl3_ICSD_630364	metal	metal
Dy6Fe1Sb2_ICSD_156085	metal	metal
Dy6Fe1Sb2_ICSD_96250	metal	metal
Dy6Fe1Te2_ICSD_91340	metal	metal
Dy6Fe23_ICSD_629567	metal	metal
Dy6Mn23_ICSD_629921	metal	metal
Dy6Zn23_ICSD_630373	metal	metal
Dy7Ir2Te2_ICSD_420220	metal	metal
Er11Ge10_ICSD_630596	metal	metal
Er11In6Si4_ICSD_184239	metal	metal
Er11Sn10_ICSD_631167	metal	metal
Er14In3Pd3_ICSD_418560	metal	metal
Er17Ru6Te3_ICSD_391431	metal	metal
Er1F1Mo1O4_ICSD_419253	insulator	insulator
Er1F1Na1O4P1_ICSD_94521	insulator	insulator
Er1F1O1_ICSD_184012	insulator	insulator
Er1F1O3Se1_ICSD_419954	insulator	insulator
Er1F1S1_ICSD_94476	insulator	insulator
Er1F3_ICSD_81411	insulator	insulator

Supplementary Table 348. Five-fold cross validated predictions for the metal/insulator classification (340/598).

system	calculated	predicted
Er1F5K2_ICSD_20404	insulator	insulator
Er1F6Na1Rb2_ICSD_23142	insulator	insulator
Er1Fe1Ge2_ICSD_630459	metal	metal
Er1Fe2Ge2_ICSD_260818	metal	metal
Er1Fe2O4_ICSD_67700	metal	metal
Er1Fe2Si2_ICSD_630499	metal	metal
Er1Fe2_ICSD_630423	metal	metal
Er1Fe2_ICSD_630432	metal	metal
Er1Fe3_ICSD_602052	metal	metal
Er1Fe4Ge2_ICSD_156069	metal	metal
Er1Fe4Ge2_ICSD_156072	metal	metal
Er1Fe4Ge2_ICSD_85886	metal	metal
Er1Fe6Ga6_ICSD_630453	metal	metal
Er1Fe6Ge6_ICSD_656391	metal	metal
Er1Ga1Ir1_ICSD_630550	metal	metal
Er1Ga1Ni1_ICSD_630555	metal	metal
Er1Ga1Pd1_ICSD_630568	metal	metal
Er1Ga1Pt1_ICSD_630571	metal	metal
Er1Ga1Rh1_ICSD_630573	metal	metal
Er1Ga1Zn1_ICSD_261028	metal	metal
Er1Ga1Zn1_ICSD_630581	metal	metal
Er1Ga1_ICSD_630532	metal	metal
Er1Ga2Ni1_ICSD_630566	metal	metal
Er1Ga2Pd1_ICSD_600162	metal	metal
Er1Ga2_ICSD_103230	metal	metal
Er1Ga3Ru1_ICSD_630575	metal	metal
Er1Ga3_ICSD_602415	metal	metal
Er1Ga4Ni1_ICSD_423066	metal	metal
Er1Ga4Ti2_ICSD_630579	metal	metal
Er1Ga4V2_ICSD_103238	metal	metal
Er1Ge1Ir1_ICSD_630615	metal	metal
Er1Ge1Li1_ICSD_76287	metal	metal
Er1Ge1Mn1_ICSD_97799	metal	metal
Er1Ge1Mn1_ICSD_97800	metal	metal
Er1Ge1Na1O4_ICSD_54199	insulator	insulator
Er1Ge1Ni1_ICSD_630636	metal	metal
Er1Ge1Pd2_ICSD_151861	metal	metal
Er1Ge1Pt1_ICSD_630642	metal	metal
Er1Ge1Rh1_ICSD_86113	metal	metal
Er1Ge1Ru1_ICSD_88190	metal	metal
Er1Ge1Sn1_ICSD_261755	metal	metal
Er1Ge1Ti1_ICSD_93649	metal	metal
Er1Ge1_ICSD_630607	metal	metal
Er1Ge2Ir1_ICSD_93338	metal	metal
Er1Ge2Mn2_ICSD_630623	metal	metal
Er1Ge2Ni1_ICSD_170341	metal	metal
Er1Ge2Ni1_ICSD_658573	metal	metal
Er1Ge2Ni2_ICSD_630635	metal	metal
Er1Ge2Pt1_ICSD_93335	metal	metal
Er1Ge2Rh2_ICSD_630646	metal	metal

Supplementary Table 349. Five-fold cross validated predictions for the metal/insulator classification (341/598).

system	calculated	predicted
Er1Ge2Ru2_ICSD_630652	metal	metal
Er1Ge6Mn6_ICSD_107030	metal	metal
Er1H1O2_ICSD_34473	insulator	insulator
Er1H1_ICSD_187370	metal	metal
Er1H1_ICSD_187373	metal	metal
Er1H2_ICSD_187374	metal	metal
Er1H2_ICSD_630659	metal	metal
Er1H3O3_ICSD_200097	insulator	insulator
Er1H3_ICSD_187369	insulator	metal
Er1H3_ICSD_187372	metal	metal
Er1H3_ICSD_187375	metal	metal
Er1Hg1_ICSD_630672	metal	metal
Er1Hg2_ICSD_103246	metal	metal
Er1Hg3_ICSD_630671	metal	metal
Er1I1Se1_ICSD_50194	insulator	insulator
Er1In1Ni1_ICSD_165162	metal	metal
Er1In1Pd1_ICSD_630695	metal	metal
Er1In1Pd2_ICSD_103253	metal	metal
Er1In1Pt1_ICSD_154843	metal	metal
Er1In1Pt4_ICSD_630697	metal	metal
Er1In1Rh1_ICSD_412915	metal	metal
Er1In1Zn1_ICSD_630703	metal	metal
Er1In1_ICSD_103248	metal	metal
Er1In2Ni9_ICSD_600173	metal	metal
Er1In3_ICSD_657242	metal	metal
Er1Ir1Si1_ICSD_93220	metal	metal
Er1Ir1Sn1_ICSD_630724	metal	metal
Er1Ir1_ICSD_103257	metal	metal
Er1Ir2Si2_ICSD_57231	metal	metal
Er1Ir2_ICSD_103258	metal	metal
Er1K1O12P4_ICSD_200795	insulator	insulator
Er1K1O2_ICSD_27002	insulator	insulator
Er1K1O8W2_ICSD_157832	insulator	insulator
Er1K1S2_ICSD_44948	insulator	insulator
Er1K1Te2_ICSD_71560	insulator	insulator
Er1Li1O12P4_ICSD_33242	insulator	insulator
Er1Li1O2_ICSD_95380	insulator	insulator
Er1Li1O2_ICSD_95381	insulator	insulator
Er1Li1S2_ICSD_630736	insulator	insulator
Er1Li1Se2_ICSD_44966	insulator	insulator
Er1Li1Sn1_ICSD_603146	metal	metal
Er1Li1Sn2_ICSD_630741	metal	metal
Er1Mg1Pd1_ICSD_657930	metal	metal
Er1Mg1Sn1_ICSD_183529	metal	metal
Er1Mg1_ICSD_103260	metal	metal
Er1Mg2_ICSD_164275	metal	metal
Er1Mn12_ICSD_26826	metal	metal
Er1Mn1Ni4_ICSD_630772	metal	metal
Er1Mn1Si1_ICSD_88062	metal	metal
Er1Mn2Si2_ICSD_630781	metal	metal

Supplementary Table 350. Five-fold cross validated predictions for the metal/insulator classification (342/598).

system	calculated	predicted
Er1Mn2_ICSD_106610	metal	metal
Er1Mn2_ICSD_106832	metal	metal
Er1Mo6S8_ICSD_603465	metal	metal
Er1Mo6Se8_ICSD_630792	metal	metal
Er1N1_ICSD_167759	metal	metal
Er1N1_ICSD_169223	insulator	metal
Er1Na1O2_ICSD_2739	insulator	insulator
Er1Na1O2_ICSD_97544	insulator	insulator
Er1Na1O8S2_ICSD_90775	insulator	insulator
Er1Na1P2S6_ICSD_415672	insulator	insulator
Er1Na1S2_ICSD_73481	insulator	insulator
Er1Na1Se2_ICSD_50195	insulator	insulator
Er1Ni10Si2_ICSD_97038	metal	metal
Er1Ni1P1_ICSD_630859	metal	metal
Er1Ni1Sb1_ICSD_103273	insulator	insulator
Er1Ni1Si2_ICSD_658583	metal	metal
Er1Ni1Sn1_ICSD_103275	metal	metal
Er1Ni1Zn1_ICSD_656587	metal	metal
Er1Ni1_ICSD_630832	metal	metal
Er1Ni2P2_ICSD_88187	metal	metal
Er1Ni2Sb2_ICSD_630862	metal	metal
Er1Ni2Si2_ICSD_54158	metal	metal
Er1Ni2_ICSD_630848	metal	metal
Er1Ni3_ICSD_152845	metal	metal
Er1Ni3_ICSD_630836	metal	metal
Er1Ni4P2_ICSD_601114	metal	metal
Er1Ni4_ICSD_630812	metal	metal
Er1Ni5_ICSD_103267	metal	metal
Er1O14P5_ICSD_23304	insulator	insulator
Er1O2Rb1_ICSD_15161	insulator	insulator
Er1O3V1_ICSD_161781	insulator	insulator
Er1O4P1_ICSD_184547	insulator	insulator
Er1O4Ta1_ICSD_415437	insulator	insulator
Er1O4V1_ICSD_69118	insulator	insulator
Er1O4V1_ICSD_78080	insulator	insulator
Er1O6Re1Sr2_ICSD_25405	metal	metal
Er1O6Rh1Sr3_ICSD_51057	insulator	insulator
Er1O9P3_ICSD_20935	insulator	insulator
Er1Os2Si2_ICSD_57258	metal	metal
Er1Os2_ICSD_630903	metal	metal
Er1P1Pd1_ICSD_69673	insulator	metal
Er1P1S1_ICSD_630913	insulator	insulator
Er1P1_ICSD_167761	metal	metal
Er1P1_ICSD_53407	metal	metal
Er1P2Ru2_ICSD_602113	metal	metal
Er1Pb1Pd1_ICSD_657946	metal	metal
Er1Pb3_ICSD_103277	metal	metal
Er1Pd1Sb1_ICSD_57259	insulator	metal
Er1Pd1Si1_ICSD_408081	metal	metal
Er1Pd1Sn1_ICSD_657627	metal	metal

Supplementary Table 351. Five-fold cross validated predictions for the metal/insulator classification (343/598).

system	calculated	predicted
Er1Pd1Sn1_ICSD_657630	metal	metal
Er1Pd1Tl1_ICSD_103286	metal	metal
Er1Pd1Zn1_ICSD_656593	metal	metal
Er1Pd1_ICSD_630924	metal	metal
Er1Pd2Si1_ICSD_151859	metal	metal
Er1Pd2Si2_ICSD_630942	metal	metal
Er1Pd2Sn1_ICSD_103284	metal	metal
Er1Pd3S4_ICSD_630936	metal	metal
Er1Pd3_ICSD_656120	metal	metal
Er1Pt1Sb1_ICSD_53409	metal	metal
Er1Pt1Si1_ICSD_630989	metal	metal
Er1Pt1Sn1_ICSD_630991	metal	metal
Er1Pt1Zn1_ICSD_423115	metal	metal
Er1Pt1_ICSD_630968	metal	metal
Er1Pt2Si1_ICSD_630985	metal	metal
Er1Pt2Si2_ICSD_630986	metal	metal
Er1Pt2Si2_ICSD_630990	metal	metal
Er1Pt2Sn1_ICSD_103292	metal	metal
Er1Pt2_ICSD_630983	metal	metal
Er1Pt3_ICSD_103289	metal	metal
Er1Rb1S2_ICSD_81404	insulator	insulator
Er1Rb1Se2_ICSD_281075	insulator	insulator
Er1Re2_ICSD_630993	metal	metal
Er1Rh1Si1_ICSD_53410	metal	metal
Er1Rh1Sn1_ICSD_411825	metal	metal
Er1Rh1_ICSD_631008	metal	metal
Er1Rh2Si2_ICSD_602058	metal	metal
Er1Rh2_ICSD_631016	metal	metal
Er1Rh3Si2_ICSD_53412	metal	metal
Er1Rh3Si2_ICSD_62924	metal	metal
Er1Rh5_ICSD_103297	metal	metal
Er1Ru1Si1_ICSD_88067	metal	metal
Er1Ru2Si2_ICSD_631050	metal	metal
Er1Ru2_ICSD_631040	metal	metal
Er1S1_ICSD_631056	metal	metal
Er1S2Tl1_ICSD_106620	insulator	insulator
Er1S2_ICSD_53415	metal	metal
Er1S2_ICSD_631062	metal	metal
Er1Sb1Zr1_ICSD_152643	metal	metal
Er1Sb1_ICSD_53417	metal	metal
Er1Sb2_ICSD_631094	metal	metal
Er1Se1_ICSD_600371	metal	metal
Er1Se2Tl1_ICSD_106993	insulator	insulator
Er1Se2_ICSD_631111	metal	metal
Er1Se2_ICSD_631116	metal	metal
Er1Si1Ti1_ICSD_88118	metal	metal
Er1Si1_ICSD_106621	metal	metal
Er1Si1_ICSD_631134	metal	metal
Er1Si2_ICSD_631150	metal	metal
Er1Si2_ICSD_631164	metal	metal

Supplementary Table 352. Five-fold cross validated predictions for the metal/insulator classification (344/598).

system	calculated	predicted
Er1Sn2Zn1_ICSD_183312	metal	metal
Er1Sn2_ICSD_168682	metal	metal
Er1Sn3_ICSD_103305	metal	metal
Er1Tc2_ICSD_631172	metal	metal
Er1Te1_ICSD_631173	metal	metal
Er1Te2Tl1_ICSD_631187	insulator	insulator
Er1Te2_ICSD_631180	metal	metal
Er1Tl1Zn1_ICSD_631211	metal	metal
Er1Tl1_ICSD_103312	metal	metal
Er1Tl1_ICSD_631208	metal	metal
Er1Tl3_ICSD_103313	metal	metal
Er1Zn12_ICSD_631224	metal	metal
Er1Zn1_ICSD_631214	metal	metal
Er1Zn2_ICSD_631213	metal	metal
Er1Zn3_ICSD_631233	metal	metal
Er1Zn5_ICSD_103318	metal	metal
Er1Zn5_ICSD_103319	metal	metal
Er1_ICSD_247674	metal	metal
Er1_ICSD_53386	metal	metal
Er1_ICSD_53388	metal	metal
Er2Eu1S4_ICSD_630396	insulator	insulator
Er2Eu1Se4_ICSD_630398	insulator	insulator
Er2Fe17_ICSD_106602	metal	metal
Er2Fe1Ga8_ICSD_600152	metal	metal
Er2Fe1Si2_ICSD_99216	metal	metal
Er2Fe3Si5_ICSD_630508	metal	metal
Er2Ge1Rh3_ICSD_630648	metal	metal
Er2Ge2Mg1_ICSD_423456	metal	metal
Er2Ge2O7_ICSD_16164	insulator	insulator
Er2Ge5Ru3_ICSD_602054	metal	metal
Er2Ge5_ICSD_88203	metal	metal
Er2Hf2O7_ICSD_162006	insulator	insulator
Er2In1Ni2_ICSD_630688	metal	metal
Er2In1Ni2_ICSD_630690	metal	metal
Er2In1Pd2_ICSD_54487	metal	metal
Er2In1_ICSD_103251	metal	metal
Er2Mg1Se4_ICSD_630754	insulator	insulator
Er2Mg1_ICSD_55548	metal	metal
Er2Mn2O7_ICSD_202517	insulator	insulator
Er2Mo3Si4_ICSD_600622	metal	metal
Er2Ni12P7_ICSD_602512	metal	metal
Er2Ni2Sn1_ICSD_425431	metal	metal
Er2Ni7_ICSD_630835	metal	metal
Er2O11Te4_ICSD_413661	insulator	insulator
Er2O13Te5_ICSD_413666	insulator	insulator
Er2O1S2_ICSD_67379	insulator	insulator
Er2O2S1_ICSD_109334	insulator	insulator
Er2O2Se1_ICSD_25810	insulator	insulator
Er2O3_ICSD_160215	insulator	insulator
Er2O3_ICSD_160230	insulator	insulator

Supplementary Table 353. Five-fold cross validated predictions for the metal/insulator classification (345/598).

system	calculated	predicted
Er2O3_ICSD_39189	insulator	insulator
Er2O5Si1_ICSD_419309	insulator	insulator
Er2O7Ru2_ICSD_97533	insulator	insulator
Er2O7Si2_ICSD_74779	insulator	insulator
Er2O7Sn2_ICSD_84749	insulator	insulator
Er2O7Tc2_ICSD_109081	insulator	insulator
Er2O7Ti2_ICSD_24209	insulator	insulator
Er2O7V2_ICSD_160866	insulator	insulator
Er2O9Se3_ICSD_411749	insulator	insulator
Er2Pb1Pd2_ICSD_99198	metal	metal
Er2Pb1S4_ICSD_630919	insulator	insulator
Er2Pb1Se4_ICSD_630921	insulator	insulator
Er2Pt1_ICSD_630961	metal	metal
Er2Rh1Si3_ICSD_97376	metal	metal
Er2Rh3Si1_ICSD_631024	metal	metal
Er2Ru3Si5_ICSD_68792	metal	metal
Er2S3_ICSD_631065	insulator	insulator
Er2S3_ICSD_72292	insulator	insulator
Er2S3_ICSD_73558	insulator	insulator
Er2S4Sr1_ICSD_631075	insulator	insulator
Er2S4Yb1_ICSD_631087	insulator	insulator
Er2S4Zn1_ICSD_631088	insulator	insulator
Er2Se3_ICSD_40617	metal	metal
Er2Se4Sr1_ICSD_631123	insulator	insulator
Er2Se4Yb1_ICSD_631130	insulator	insulator
Er2Sn5_ICSD_167894	metal	metal
Er2Te3_ICSD_50501	metal	metal
Er2Zn17_ICSD_103321	metal	metal
Er2Zn17_ICSD_631219	metal	metal
Er3F10K1_ICSD_418210	insulator	insulator
Er3Fe1Si3_ICSD_152532	metal	metal
Er3Fe5O12_ICSD_71464	insulator	insulator
Er3Ga1O6_ICSD_99497	insulator	insulator
Er3Ga1S6_ICSD_630576	insulator	insulator
Er3Ga2Mn3Si1_ICSD_99078	metal	metal
Er3Ga2Ni6_ICSD_630554	metal	metal
Er3Ga2_ICSD_630544	metal	metal
Er3Ga5O12_ICSD_9238	insulator	insulator
Er3Ga5_ICSD_602413	metal	metal
Er3Ga9Pt2_ICSD_658055	metal	metal
Er3Ge13Ir4_ICSD_630617	metal	metal
Er3Ge13Os4_ICSD_630639	metal	metal
Er3Ge13Rh4_ICSD_630645	metal	metal
Er3Ge13Ru4_ICSD_630655	metal	metal
Er3Ge3Ru2_ICSD_425774	metal	metal
Er3Ge4_ICSD_80501	metal	metal
Er3In1N1_ICSD_98506	metal	metal
Er3In5_ICSD_630683	metal	metal
Er3Ir1_ICSD_630704	metal	metal
Er3Ir4Si13_ICSD_600539	metal	metal

Supplementary Table 354. Five-fold cross validated predictions for the metal/insulator classification (346/598).

system	calculated	predicted
Er3La1S6_ICSD_630730	insulator	insulator
Er3Nd1S6_ICSD_630810	insulator	insulator
Er3Ni1Si3_ICSD_152535	metal	metal
Er3Ni1_ICSD_630815	metal	metal
Er3Ni2_ICSD_2150	metal	metal
Er3Ni6Si2_ICSD_630878	metal	metal
Er3Os1_ICSD_630900	metal	metal
Er3Os4Si13_ICSD_600536	metal	metal
Er3P4Pd7_ICSD_74936	metal	metal
Er3P6Pd20_ICSD_409903	metal	metal
Er3Pd2_ICSD_107685	metal	metal
Er3Pd4_ICSD_630930	metal	metal
Er3Pd8Sb4_ICSD_174471	metal	metal
Er3Pt1_ICSD_630965	metal	metal
Er3Pt4_ICSD_630975	metal	metal
Er3Rh1_ICSD_103299	metal	metal
Er3Ru1_ICSD_631037	metal	metal
Er3Ru2_ICSD_106615	metal	metal
Er3Ru2_ICSD_631044	metal	metal
Er3Se6Sm1_ICSD_417135	metal	insulator
Er3Tl5_ICSD_631205	metal	metal
Er4Fe1Ga12_ICSD_248000	metal	metal
Er4Ga12Ni1_ICSD_630564	metal	metal
Er4Ga12Pd1_ICSD_630569	metal	metal
Er4Ge4In1Ni2_ICSD_240334	metal	metal
Er4Ge6Ir7_ICSD_630616	metal	metal
Er4Ge6Os7_ICSD_630637	metal	metal
Er4Ge6Rh7_ICSD_630644	metal	metal
Er4I5_ICSD_200828	metal	metal
Er4In1Ir1_ICSD_418265	metal	metal
Er4In1Rh1_ICSD_417515	metal	metal
Er4Sb3_ICSD_600631	metal	metal
Er5Ga3_ICSD_602420	metal	metal
Er5Ge3_ICSD_106605	metal	metal
Er5In3_ICSD_630682	metal	metal
Er5In4Ni2_ICSD_630691	metal	metal
Er5In4Pd2_ICSD_165137	metal	metal
Er5Ir2_ICSD_630708	metal	metal
Er5Ir3_ICSD_630711	metal	metal
Er5Ir4Si10_ICSD_630722	metal	metal
Er5Mg24_ICSD_151537	metal	metal
Er5Ni1Pb3_ICSD_152606	metal	metal
Er5Ni2Te2_ICSD_150369	metal	metal
Er5Pb3_ICSD_630914	metal	metal
Er5Pt3_ICSD_630959	metal	metal
Er5Rh3_ICSD_103300	metal	metal
Er5Ru2_ICSD_631047	metal	metal
Er5S7_ICSD_631059	metal	metal
Er5Sb3_ICSD_156884	metal	metal
Er5Si3_ICSD_631139	metal	metal

Supplementary Table 355. Five-fold cross validated predictions for the metal/insulator classification (347/598).

system	calculated	predicted
Er5Sn3_ICSD_54582	metal	metal
Er5Tl3_ICSD_631201	metal	metal
Er5Tl3_ICSD_631209	metal	metal
Er6Fe23_ICSD_630446	metal	metal
Er6Ga1Ni2_ICSD_630567	metal	metal
Er6I10Ir1_ICSD_424428	insulator	insulator
Er6I10Ni1_ICSD_424429	insulator	insulator
Er6I7_ICSD_14183	metal	metal
Er6Mn1Te2_ICSD_182404	metal	metal
Er6Mn23_ICSD_630765	metal	metal
Er6Mn23_ICSD_630767	metal	metal
Er6Ni2Sn1_ICSD_162140	metal	metal
Er6Zn23_ICSD_103322	metal	metal
Er7Fe1I12_ICSD_424431	metal	insulator
Er7Ni2Te2_ICSD_280628	metal	metal
Er7Rh3_ICSD_103301	metal	metal
Eu0.034Mn0.966_ICSD_631390	metal	metal
Eu1F1Mo1O4_ICSD_419248	insulator	insulator
Eu1F1O1_ICSD_184006	insulator	insulator
Eu1F2_ICSD_260882	insulator	insulator
Eu1F3_ICSD_95244	insulator	insulator
Eu1F4Mg1_ICSD_86246	insulator	insulator
Eu1F7Sn1_ICSD_71585	insulator	insulator
Eu1Fe2Si2_ICSD_631248	metal	metal
Eu1Fe4P12_ICSD_79925	metal	metal
Eu1Ga1Ge1_ICSD_249599	metal	metal
Eu1Ga2Se4_ICSD_631269	insulator	insulator
Eu1Ga2_ICSD_103388	metal	metal
Eu1Ga2_ICSD_58688	metal	metal
Eu1Ga2_ICSD_631256	metal	metal
Eu1Ga4_ICSD_602026	metal	metal
Eu1Ge12Pt4_ICSD_174556	metal	metal
Eu1Ge1Li2S4_ICSD_281012	insulator	insulator
Eu1Ge1Na1O4_ICSD_186847	insulator	insulator
Eu1Ge1Pt1_ICSD_106432	metal	metal
Eu1Ge1Pt1_ICSD_401561	metal	metal
Eu1Ge1Sc1_ICSD_601016	metal	metal
Eu1Ge1Zn1_ICSD_246862	metal	metal
Eu1Ge1Zn1_ICSD_246863	metal	metal
Eu1Ge1Zn1_ICSD_246865	metal	metal
Eu1Ge1Zn1_ICSD_246866	metal	metal
Eu1Ge1Zn1_ICSD_246871	metal	metal
Eu1Ge1_ICSD_52702	metal	metal
Eu1Ge1_ICSD_631288	metal	metal
Eu1Ge2Ir1_ICSD_404599	metal	metal
Eu1Ge2Ir2_ICSD_631293	metal	metal
Eu1Ge2Ni2_ICSD_631297	metal	metal
Eu1Ge2Ni2_ICSD_87254	metal	metal
Eu1Ge2Rh2_ICSD_631308	metal	metal
Eu1Ge2Rh2_ICSD_76354	metal	metal

Supplementary Table 356. Five-fold cross validated predictions for the metal/insulator classification (348/598).

system	calculated	predicted
Eu1Ge2Ru2_ICSD_631312	metal	metal
Eu1Ge2Zn2_ICSD_51757	metal	metal
Eu1Ge2Zn2_ICSD_59650	metal	metal
Eu1Ge2_ICSD_150337	metal	metal
Eu1Ge2_ICSD_43274	metal	metal
Eu1Ge3Ir1_ICSD_631294	metal	metal
Eu1Ge3Ni1_ICSD_600943	metal	metal
Eu1Ge3Pt1_ICSD_409869	metal	metal
Eu1Ge3Rh1_ICSD_631309	metal	metal
Eu1Ge4Ni9_ICSD_600942	metal	metal
Eu1H1O12P4_ICSD_250030	insulator	insulator
Eu1H2_ICSD_631320	insulator	metal
Eu1H3Li1_ICSD_416461	insulator	insulator
Eu1H4Mg1Ni1_ICSD_657978	insulator	insulator
Eu1Hg1Pb1_ICSD_602720	metal	metal
Eu1Hg1Sn1_ICSD_602727	metal	metal
Eu1Hg1_ICSD_103396	metal	metal
Eu1Hg2_ICSD_631334	metal	metal
Eu1Hg3_ICSD_631332	metal	metal
Eu1Ho2S4_ICSD_631336	insulator	insulator
Eu1Ho2Se4_ICSD_631338	insulator	insulator
Eu1I2_ICSD_22131	insulator	insulator
Eu1I2_ICSD_260561	insulator	insulator
Eu1I2_ICSD_56816	insulator	insulator
Eu1In1Pd1_ICSD_103400	metal	metal
Eu1In1Pd1_ICSD_631348	metal	metal
Eu1In1Pt1_ICSD_405049	metal	metal
Eu1In1Pt4_ICSD_631349	metal	metal
Eu1In1_ICSD_106626	metal	metal
Eu1In2S4_ICSD_631350	metal	insulator
Eu1In2Se4_ICSD_631352	insulator	insulator
Eu1In2_ICSD_103398	metal	metal
Eu1In2_ICSD_631341	metal	metal
Eu1In4_ICSD_106627	metal	metal
Eu1Ir1P1_ICSD_602838	metal	metal
Eu1Ir1Si3_ICSD_631358	metal	metal
Eu1Ir1Sn2_ICSD_410700	metal	metal
Eu1Ir2P2_ICSD_73530	insulator	metal
Eu1Ir2Si2_ICSD_414147	metal	metal
Eu1Ir2Si2_ICSD_631357	metal	metal
Eu1Ir2_ICSD_631354	metal	metal
Eu1K1Na1Nb1O5_ICSD_420036	insulator	insulator
Eu1K1Na1O5Ta1_ICSD_173777	metal	insulator
Eu1K1O4P1_ICSD_36647	insulator	insulator
Eu1K1O8W2_ICSD_173634	metal	insulator
Eu1K1P1S4_ICSD_279622	insulator	insulator
Eu1K1P1Se4_ICSD_280200	insulator	insulator
Eu1K1S2_ICSD_631365	metal	metal
Eu1K1S4Si1_ICSD_280649	metal	insulator
Eu1K3O8P2_ICSD_94536	metal	insulator

Supplementary Table 357. Five-fold cross validated predictions for the metal/insulator classification (349/598).

system	calculated	predicted
Eu1Li1O12P4_ICSD_416878	insulator	insulator
Eu1Li1O2_ICSD_422560	insulator	insulator
Eu1Li1O4Ti1_ICSD_81858	insulator	insulator
Eu1Li1O8S2_ICSD_200222	insulator	insulator
Eu1Li1P1Se4_ICSD_280199	insulator	insulator
Eu1Li2O4Si1_ICSD_50442	insulator	insulator
Eu1Mg1Pd1_ICSD_412725	metal	metal
Eu1Mg1_ICSD_161745	metal	metal
Eu1Mg2Sb2_ICSD_412653	insulator	insulator
Eu1Mg2_ICSD_103403	metal	metal
Eu1Mn2P2_ICSD_51782	metal	metal
Eu1Mn2Sb2_ICSD_100584	metal	metal
Eu1Mo2O8Tl1_ICSD_152174	metal	insulator
Eu1Mo6S8_ICSD_62155	metal	metal
Eu1Mo6S8_ICSD_65774	metal	metal
Eu1Mo6Se8_ICSD_600678	metal	metal
Eu1N1_ICSD_44772	metal	metal
Eu1N2O2Si2_ICSD_416046	insulator	insulator
Eu1N7Si4Y1_ICSD_150460	insulator	insulator
Eu1Na10Sn12_ICSD_172209	insulator	insulator
Eu1Na1O4Ti1_ICSD_81536	metal	insulator
Eu1Na1S2_ICSD_631411	metal	insulator
Eu1Nb2O6_ICSD_247276	insulator	insulator
Eu1Ni1Si2_ICSD_631449	metal	metal
Eu1Ni2P2_ICSD_15258	metal	metal
Eu1Ni2P2_ICSD_631434	metal	metal
Eu1Ni2Sb2_ICSD_631439	metal	metal
Eu1Ni5P3_ICSD_40812	metal	metal
Eu1Ni5_ICSD_103413	metal	metal
Eu1O1_ICSD_53437	insulator	metal
Eu1O2Rb1_ICSD_27334	insulator	insulator
Eu1O2_ICSD_631464	metal	insulator
Eu1O3Ti1_ICSD_187102	insulator	metal
Eu1O3Ti1_ICSD_187108	insulator	insulator
Eu1O3Ti1_ICSD_187204	insulator	insulator
Eu1O4P1Rb1_ICSD_36648	insulator	insulator
Eu1O4S1_ICSD_68244	insulator	insulator
Eu1O4Sb1_ICSD_245019	insulator	insulator
Eu1O4Ta1_ICSD_415432	insulator	insulator
Eu1O4V1_ICSD_15608	metal	insulator
Eu1O4V1_ICSD_164835	metal	insulator
Eu1O4W1_ICSD_185361	insulator	insulator
Eu1O6Rh1Sr3_ICSD_51053	insulator	insulator
Eu1O8Rb1S2_ICSD_48002	insulator	insulator
Eu1Os2P2_ICSD_602107	metal	metal
Eu1Os4Sb12_ICSD_55664	metal	metal
Eu1P12Ru4_ICSD_631497	metal	metal
Eu1P1Pd1_ICSD_57274	metal	metal
Eu1P1Pt1_ICSD_631489	metal	metal
Eu1P1Sn1_ICSD_95081	metal	metal

Supplementary Table 358. Five-fold cross validated predictions for the metal/insulator classification (350/598).

system	calculated	predicted
Eu1P1_ICSD_631476	metal	metal
Eu1P2Pd2_ICSD_631486	metal	metal
Eu1P2Rh2_ICSD_50187	metal	metal
Eu1P2Ru2_ICSD_602098	metal	metal
Eu1P2Ru2_ICSD_61126	metal	metal
Eu1P2Zn2_ICSD_631503	insulator	insulator
Eu1Pb1_ICSD_103417	metal	metal
Eu1Pb3_ICSD_103418	metal	metal
Eu1Pd1Sb1_ICSD_422624	metal	metal
Eu1Pd1Si1_ICSD_631539	metal	metal
Eu1Pd1Sn1_ICSD_657622	metal	metal
Eu1Pd1Tl2_ICSD_172230	metal	metal
Eu1Pd1Zn1_ICSD_420683	metal	metal
Eu1Pd1_ICSD_631522	metal	metal
Eu1Pd2Sb2_ICSD_168263	metal	metal
Eu1Pd2Sb2_ICSD_47171	metal	metal
Eu1Pd2Si2_ICSD_602027	metal	metal
Eu1Pd2Si2_ICSD_631536	metal	metal
Eu1Pd2Si2_ICSD_631543	metal	metal
Eu1Pd2_ICSD_103423	metal	metal
Eu1Pd3S4_ICSD_84667	metal	metal
Eu1Pd3_ICSD_103425	metal	metal
Eu1Pt1Sb1_ICSD_60831	metal	metal
Eu1Pt1Si1_ICSD_604427	metal	metal
Eu1Pt1Si1_ICSD_631566	metal	metal
Eu1Pt1Sn1_ICSD_107571	metal	metal
Eu1Pt1Zn1_ICSD_420684	metal	metal
Eu1Pt2Si2_ICSD_631565	metal	metal
Eu1Pt2Si2_ICSD_631567	metal	metal
Eu1Pt2_ICSD_103431	metal	metal
Eu1Rb1S2_ICSD_81399	metal	insulator
Eu1Rh1Si3_ICSD_631577	metal	metal
Eu1Rh2Si2_ICSD_602028	metal	metal
Eu1Rh2_ICSD_103432	metal	metal
Eu1Ru1Sn3_ICSD_380383	metal	metal
Eu1Ru2Si2_ICSD_631586	metal	metal
Eu1Ru4Sb12_ICSD_79928	metal	metal
Eu1S1_ICSD_183957	insulator	insulator
Eu1S1_ICSD_631595	insulator	insulator
Eu1S2Tl1_ICSD_631635	metal	insulator
Eu1S4Sb2_ICSD_600799	metal	insulator
Eu1Sb2Se4_ICSD_600803	metal	insulator
Eu1Sb2Zn2_ICSD_380375	metal	metal
Eu1Sb2_ICSD_10081	metal	metal
Eu1Sc2Se4_ICSD_391438	metal	insulator
Eu1Se1_ICSD_183958	insulator	insulator
Eu1Se1_ICSD_631657	insulator	insulator
Eu1Se4Y2_ICSD_631671	insulator	insulator
Eu1Si1Zn1_ICSD_106634	metal	metal
Eu1Si1_ICSD_53446	metal	metal

Supplementary Table 359. Five-fold cross validated predictions for the metal/insulator classification (351/598).

system	calculated	predicted
Eu1Si2Zn2_ICSD_51756	metal	metal
Eu1Si2_ICSD_103436	metal	metal
Eu1Si2_ICSD_631674	metal	metal
Eu1Si2_ICSD_631683	metal	metal
Eu1Si6_ICSD_416576	metal	metal
Eu1Sn1Zn1_ICSD_405050	metal	metal
Eu1Sn1_ICSD_108434	metal	metal
Eu1Sn2Zn2_ICSD_162267	metal	metal
Eu1Sn3_ICSD_108435	metal	metal
Eu1Te1_ICSD_33612	insulator	metal
Eu1Te1_ICSD_53447	metal	insulator
Eu1Tl1_ICSD_103437	metal	metal
Eu1Tl1_ICSD_631702	metal	metal
Eu1Tl2_ICSD_103438	metal	metal
Eu1Tl3_ICSD_103439	metal	metal
Eu1Tl3_ICSD_631704	metal	metal
Eu1Zn13_ICSD_103446	metal	metal
Eu1Zn1_ICSD_103442	metal	metal
Eu1Zn2_ICSD_103443	metal	metal
Eu1Zn5_ICSD_103444	metal	metal
Eu1Zn5_ICSD_631712	metal	metal
Eu1_ICSD_44498	metal	metal
Eu1_ICSD_53423	metal	metal
Eu1_ICSD_631240	metal	metal
Eu2F1K5O13Si4_ICSD_249458	insulator	insulator
Eu2Fe1H6_ICSD_631243	metal	insulator
Eu2Ga2Ge1S7_ICSD_262242	insulator	insulator
Eu2Ge1S4_ICSD_8242	insulator	insulator
Eu2Ge1Se4_ICSD_412227	insulator	insulator
Eu2Ge1Se4_ICSD_412228	insulator	insulator
Eu2H6Ru1_ICSD_656083	insulator	metal
Eu2Hf2O7_ICSD_173953	metal	insulator
Eu2I1P1_ICSD_202068	insulator	insulator
Eu2Ir2O7_ICSD_173948	metal	metal
Eu2Li1Si3_ICSD_416629	metal	metal
Eu2Mn1O7Si2_ICSD_261225	metal	insulator
Eu2Mo2O7_ICSD_173946	metal	metal
Eu2O2S1_ICSD_109330	metal	insulator
Eu2O2Te1_ICSD_89562	metal	metal
Eu2O3_ICSD_659185	insulator	metal
Eu2O3_ICSD_8056	insulator	insulator
Eu2O4Si1_ICSD_1510	insulator	insulator
Eu2O4Si1_ICSD_23615	insulator	insulator
Eu2O4V1_ICSD_89000	metal	metal
Eu2O7Pb2_ICSD_173951	metal	insulator
Eu2O7Pt2_ICSD_173952	metal	insulator
Eu2O7Ru2_ICSD_109306	metal	metal
Eu2O7Si2_ICSD_34586	insulator	insulator
Eu2O7Sn2_ICSD_84754	metal	insulator
Eu2O7Ti2_ICSD_92767	insulator	insulator

Supplementary Table 360. Five-fold cross validated predictions for the metal/insulator classification (352/598).

system	calculated	predicted
Eu2O7Zr2_ICSD_173950	metal	metal
Eu2Pd1Si3_ICSD_391246	metal	metal
Eu2S4Si1_ICSD_95775	insulator	insulator
Eu2S4Sn1_ICSD_413022	insulator	insulator
Eu2Si1_ICSD_96114	metal	metal
Eu3F10Rb1_ICSD_14027	insulator	insulator
Eu3Ga1O6_ICSD_99492	insulator	insulator
Eu3Ga4Ni4_ICSD_103395	metal	metal
Eu3In1O1_ICSD_415804	metal	metal
Eu3Ir1O7_ICSD_86476	metal	metal
Eu3Ni6Si2_ICSD_631451	metal	metal
Eu3O12Sb5_ICSD_67023	insulator	insulator
Eu3O6Ta1_ICSD_4148	metal	metal
Eu3O7Os1_ICSD_170874	metal	metal
Eu3O7Ru1_ICSD_93668	metal	insulator
Eu3Pd2_ICSD_103428	metal	metal
Eu3Rh4Sn13_ICSD_102358	metal	metal
Eu3S4_ICSD_100522	metal	metal
Eu3S4_ICSD_151633	metal	metal
Eu4I6O1_ICSD_281554	insulator	insulator
Eu4Ir1_ICSD_103402	metal	metal
Eu4O1Sb2_ICSD_402953	insulator	metal
Eu5O15Ta4_ICSD_166562	insulator	insulator
Eu5Sb3_ICSD_173030	metal	metal
F10H1P1Pb2_ICSD_419141	insulator	insulator
F10Hg3Nb2O4S1_ICSD_60920	insulator	insulator
F10Hg3O4S1Ta2_ICSD_60922	insulator	insulator
F10In3Rb1_ICSD_200052	insulator	insulator
F10K1Tb3_ICSD_28214	insulator	insulator
F10K1Y3_ICSD_155137	insulator	insulator
F10K1Yb3_ICSD_28258	insulator	insulator
F10Na1Sb3_ICSD_1968	insulator	insulator
F10Nb1Sb1_ICSD_16095	insulator	insulator
F10Pb3Zr1_ICSD_100600	insulator	insulator
F11Fe1Na1Zr2_ICSD_81220	insulator	insulator
F11Hf2Na1V1_ICSD_94460	insulator	insulator
F11I2Sb2_ICSD_6031	metal	insulator
F11Kr3Sb1_ICSD_279628	insulator	insulator
F11La1Zr2_ICSD_424909	insulator	insulator
F11Mn1Na1Zr2_ICSD_81219	insulator	insulator
F11Na1Ni1Zr2_ICSD_81222	insulator	insulator
F11Na1Pd1Zr2_ICSD_77241	insulator	insulator
F11Na1Ti1Zr2_ICSD_78867	metal	insulator
F11Na1V1Zr2_ICSD_78868	insulator	insulator
F11Na1Zn1Zr2_ICSD_81223	insulator	insulator
F11Pr1Zr2_ICSD_72156	insulator	insulator
F12Fe2Li3Na3_ICSD_17056	insulator	insulator
F12Ge5_ICSD_10295	insulator	insulator
F12H8O4Pt1Sb2_ICSD_421861	metal	insulator
F12I4Sb2_ICSD_63301	insulator	insulator

Supplementary Table 361. Five-fold cross validated predictions for the metal/insulator classification (353/598).

system	calculated	predicted
F12K1Tb3_ICSD_51125	metal	insulator
F12K2O1Zr3_ICSD_281350	insulator	insulator
F12Mn1Sb2_ICSD_411522	insulator	insulator
F12O1Tl2Zr3_ICSD_48003	insulator	insulator
F12O8Os2Xe2_ICSD_260293	insulator	insulator
F12P4Pt1_ICSD_418726	insulator	insulator
F12Sb2Te4_ICSD_201222	insulator	insulator
F13H4P1Sr2_ICSD_415154	insulator	insulator
F13K1Sb4_ICSD_24740	insulator	insulator
F13K1Sb4_ICSD_4049	insulator	insulator
F13N1O2Xe2_ICSD_404987	insulator	insulator
F13Na5Zr2_ICSD_14133	insulator	insulator
F14H2Mg1Sb2_ICSD_412893	insulator	insulator
F14K4Sb2Sn1_ICSD_241228	insulator	insulator
F14N2Ni1_ICSD_26397	insulator	insulator
F14Sb4_ICSD_35709	insulator	insulator
F14Sr4Zn3_ICSD_72991	insulator	insulator
F15Nb6_ICSD_415950	metal	insulator
F15Pr1Zr3_ICSD_72838	insulator	insulator
F16Ge7_ICSD_405946	insulator	insulator
F18Mg6Rb6_ICSD_410385	insulator	insulator
F18Ni6Rb6_ICSD_410390	insulator	insulator
F18P2Pb1Xe3_ICSD_249570	insulator	insulator
F18P2Sr1Xe3_ICSD_249568	insulator	insulator
F1Fe1H4Na1O6S1_ICSD_169110	insulator	insulator
F1Fe1Li1O4S1_ICSD_180389	insulator	insulator
F1Fe1Na1O4S1_ICSD_262276	insulator	insulator
F1Fe1O3Sr2_ICSD_93510	insulator	insulator
F1Fe1O3Te1_ICSD_241141	insulator	insulator
F1Fe1O4S1_ICSD_182945	insulator	insulator
F1Ga1O3Te1_ICSD_241140	insulator	insulator
F1Ga1O4Sr3_ICSD_50734	insulator	insulator
F1Gd1Mo1O4_ICSD_419249	insulator	insulator
F1Gd1O1_ICSD_184007	insulator	insulator
F1Gd1O4S1_ICSD_410324	insulator	insulator
F1Gd1S1_ICSD_93355	insulator	insulator
F1Gd3O12Se4_ICSD_411086	insulator	insulator
F1H10N2O4P1_ICSD_2803	insulator	insulator
F1H1Hg1O1_ICSD_200744	insulator	insulator
F1H1Hg1O3Te1_ICSD_413078	insulator	insulator
F1H1Mg1O1_ICSD_186501	insulator	insulator
F1H1Na1O4P1Sn1_ICSD_96768	insulator	insulator
F1H1O1_ICSD_63681	insulator	insulator
F1H1O3S1_ICSD_65781	insulator	insulator
F1H2K1Li1O4P1_ICSD_2044	insulator	insulator
F1H2O1Rb1_ICSD_250327	insulator	insulator
F1H3K1O3P1_ICSD_9151	insulator	insulator
F1H3O1_ICSD_24382	insulator	insulator
F1H3O4S1_ICSD_33866	insulator	insulator
F1H3Si1_ICSD_60065	insulator	insulator

Supplementary Table 362. Five-fold cross validated predictions for the metal/insulator classification (354/598).

system	calculated	predicted
F1H4K1O2_ICSD_165373	insulator	insulator
F1H4K1O4_ICSD_22366	insulator	insulator
F1H4Li1N1O3P1_ICSD_1439	insulator	insulator
F1H4Mg1Na1O6S1_ICSD_201712	insulator	insulator
F1H4N1_ICSD_14294	insulator	insulator
F1H4Na1Ni1O6S1_ICSD_169112	insulator	insulator
F1H5N2_ICSD_6019	insulator	insulator
F1H6N1Na1O4P1_ICSD_240974	insulator	insulator
F1H6N1O2_ICSD_28552	insulator	insulator
F1H6Na1O6Te1_ICSD_42	insulator	insulator
F1H7N2_ICSD_419918	insulator	insulator
F1Hg2O3P1_ICSD_414292	insulator	insulator
F1Ho1Mo1O4_ICSD_419252	insulator	insulator
F1Ho1O1_ICSD_184011	insulator	insulator
F1Ho1O3Se1_ICSD_419000	insulator	insulator
F1Ho1S1_ICSD_94475	insulator	insulator
F1Ho3O12Se4_ICSD_419001	insulator	insulator
F1I1O2_ICSD_280804	insulator	insulator
F1I1Pb1_ICSD_155012	insulator	insulator
F1I1Sr1_ICSD_155009	insulator	insulator
F1In1O1_ICSD_2521	insulator	insulator
F1In1O3Te1_ICSD_260009	insulator	insulator
F1K1Nb4O5_ICSD_88880	metal	metal
F1K1O2S1_ICSD_89602	insulator	insulator
F1K1O2Se1_ICSD_78398	insulator	insulator
F1K1O3Xe1_ICSD_15219	insulator	insulator
F1K1O4S1Sn1_ICSD_94532	insulator	insulator
F1K1O6Te3_ICSD_95727	insulator	insulator
F1K1_ICSD_61558	insulator	insulator
F1K1_ICSD_64686	insulator	insulator
F1K2O3P1_ICSD_200441	insulator	insulator
F1K3O4S1_ICSD_81835	insulator	insulator
F1K3O4Se1_ICSD_82750	insulator	insulator
F1La1O1_ICSD_30622	insulator	insulator
F1La1O1_ICSD_76427	insulator	insulator
F1La1S1_ICSD_93350	insulator	insulator
F1La1Se1_ICSD_21010	insulator	insulator
F1Li1Ni1O4S1_ICSD_180391	insulator	insulator
F1Li1O3S1_ICSD_1384	insulator	insulator
F1Li1O4P1V1_ICSD_184601	insulator	insulator
F1Li1O4Si1Sr2_ICSD_79805	insulator	insulator
F1Li1_ICSD_18012	insulator	insulator
F1Li1_ICSD_184904	insulator	insulator
F1Lu1O3Se1_ICSD_417449	insulator	insulator
F1Lu1S1_ICSD_89549	insulator	insulator
F1Mg1Na1O4S1_ICSD_262272	insulator	insulator
F1Mg2N1_ICSD_17021	insulator	insulator
F1Mn2O4P1_ICSD_2814	insulator	insulator
F1Mo1O4Tb1_ICSD_419250	insulator	insulator
F1Mo1O4Y1_ICSD_249646	insulator	insulator

Supplementary Table 363. Five-fold cross validated predictions for the metal/insulator classification (355/598).

system	calculated	predicted
F1N1O1_ICSD_411510	insulator	insulator
F1N1O3Xe1_ICSD_174511	insulator	insulator
F1N1Th1_ICSD_236315	insulator	insulator
F1N3O2S4_ICSD_14028	insulator	insulator
F1N3Ta2_ICSD_182353	metal	metal
F1Na1Nb6O15_ICSD_24109	insulator	insulator
F1Na1O4P1Y1_ICSD_51463	insulator	insulator
F1Na1O4S1Zn1_ICSD_262274	insulator	insulator
F1Na1_ICSD_44276	insulator	insulator
F1Na3O4S1_ICSD_9519	insulator	insulator
F1Na3O4W1_ICSD_417289	insulator	insulator
F1Na5O16Si4Y4_ICSD_20795	insulator	insulator
F1Nd1O1_ICSD_95655	insulator	insulator
F1Nd1S1_ICSD_94471	insulator	insulator
F1Nd1Se1_ICSD_108968	insulator	insulator
F1Nd1Te1_ICSD_108969	insulator	insulator
F1O1Sb1_ICSD_19019	insulator	insulator
F1O1Sc1_ICSD_100564	insulator	insulator
F1O1Tb1_ICSD_184009	insulator	insulator
F1O1Y1_ICSD_184004	insulator	insulator
F1O1Y1_ICSD_76426	insulator	insulator
F1O1Yb1_ICSD_75557	insulator	insulator
F1O2Rb1S1_ICSD_93064	insulator	insulator
F1O3P1Rb2_ICSD_172343	insulator	insulator
F1O3P1Sn1_ICSD_2039	insulator	insulator
F1O3S1Tl1_ICSD_424114	insulator	insulator
F1O3Se1Y1_ICSD_418898	insulator	insulator
F1O3Se1Yb1_ICSD_419953	insulator	insulator
F1O3Tc1_ICSD_249509	insulator	insulator
F1O4P1V1_ICSD_183878	insulator	insulator
F1O4Rb1S1_ICSD_8275	insulator	insulator
F1O4S1Y1_ICSD_92288	insulator	insulator
F1Pr1S1_ICSD_94470	insulator	insulator
F1Rb1_ICSD_53828	insulator	insulator
F1Rb1_ICSD_61562	insulator	insulator
F1S1Tb1_ICSD_94474	insulator	insulator
F1S1Y1_ICSD_2597	insulator	insulator
F1S1Y1_ICSD_87130	insulator	insulator
F1Se1Y1_ICSD_1827	insulator	insulator
F1Tl1_ICSD_16112	insulator	insulator
F1Tl1_ICSD_16113	insulator	insulator
F1Tl1_ICSD_90992	insulator	insulator
F1Tl1_ICSD_90993	insulator	insulator
F1Tl1_ICSD_9893	insulator	insulator
F1_ICSD_426939	insulator	insulator
F20O2Re2Sb2_ICSD_201546	insulator	insulator
F24Sb2Xe6Zn1_ICSD_416302	insulator	insulator
F25K1Th6_ICSD_2711	insulator	insulator
F2Fe1_ICSD_73729	insulator	insulator
F2Fe2H1O3P1_ICSD_391478	insulator	insulator

Supplementary Table 364. Five-fold cross validated predictions for the metal/insulator classification (356/598).

system	calculated	predicted
F2Fe2O1S2Sr2_ICSD_249688	insulator	insulator
F2Fe2O1Se2Sr2_ICSD_249690	insulator	insulator
F2Ge1_ICSD_18030	insulator	insulator
F2H1K1_ICSD_9345	insulator	insulator
F2H1Li1_ICSD_23883	insulator	insulator
F2H1N1O4S2_ICSD_50523	insulator	insulator
F2H1N1_ICSD_404200	insulator	insulator
F2H1Na1_ICSD_26870	insulator	insulator
F2H1P1S1_ICSD_63001	insulator	insulator
F2H1P1_ICSD_406360	insulator	insulator
F2H1Rb1_ICSD_45859	insulator	insulator
F2H2N1P1_ICSD_201528	insulator	insulator
F2H2O5S1Sn2_ICSD_39455	insulator	insulator
F2H4N1O2P1_ICSD_16152	insulator	insulator
F2H4O1_ICSD_24383	insulator	insulator
F2H5In1N2_ICSD_87965	insulator	insulator
F2H8O4Zn1_ICSD_14064	insulator	insulator
F2Hg1_ICSD_33614	insulator	insulator
F2Hg2_ICSD_27700	insulator	insulator
F2Hg3S2_ICSD_16927	insulator	insulator
F2I1K1O2_ICSD_26600	insulator	insulator
F2I1Na1O2_ICSD_260003	insulator	insulator
F2I2O6S2_ICSD_74647	insulator	insulator
F2K1Mg2Na1O10Si4_ICSD_98196	insulator	insulator
F2K1O2P1_ICSD_26640	insulator	insulator
F2K2O5P2_ICSD_1659	insulator	insulator
F2K3Na1O6P2_ICSD_1904	insulator	insulator
F2Kr1_ICSD_23534	insulator	insulator
F2Kr1_ICSD_279623	insulator	insulator
F2Li1Rb1_ICSD_18019	insulator	insulator
F2Mg1_ICSD_51242	insulator	insulator
F2Mg1_ICSD_51243	insulator	insulator
F2Mg1_ICSD_94279	insulator	insulator
F2Mg1_ICSD_94280	insulator	insulator
F2Mg1_ICSD_94283	insulator	insulator
F2Mn1_ICSD_12167	insulator	insulator
F2Mn1_ICSD_20365	insulator	insulator
F2Mn1_ICSD_53985	insulator	insulator
F2N1P1_ICSD_9684	insulator	insulator
F2N3O1P1_ICSD_248120	insulator	insulator
F2Na1Nb1O2_ICSD_23238	insulator	insulator
F2Na1O2V1_ICSD_75418	insulator	insulator
F2Ni1_ICSD_34307	insulator	insulator
F2Ni1_ICSD_73726	insulator	insulator
F2O1Pb2_ICSD_10416	insulator	insulator
F2O1Pb2_ICSD_76964	insulator	insulator
F2O1Se1_ICSD_12110	insulator	insulator
F2O1Te1_ICSD_88415	insulator	insulator
F2O2P1Rb1_ICSD_1980	insulator	insulator
F2O2S1_ICSD_62968	insulator	insulator

Supplementary Table 365. Five-fold cross validated predictions for the metal/insulator classification (357/598).

system	calculated	predicted
F2O2U1_ICSD_31630	insulator	insulator
F2O2Xe1_ICSD_10203	insulator	insulator
F2O3Os1_ICSD_73732	insulator	insulator
F2O3S1Xe1_ICSD_10357	insulator	insulator
F2O3Te2_ICSD_82162	insulator	insulator
F2O4Rb1S1Sb1_ICSD_32709	insulator	insulator
F2O7Te2V2_ICSD_173748	insulator	insulator
F2Pb1_ICSD_154995	insulator	insulator
F2Pb1_ICSD_86738	insulator	insulator
F2Pd1_ICSD_100567	insulator	insulator
F2Pd1_ICSD_16763	insulator	insulator
F2S3Sn1Sr2_ICSD_171346	insulator	insulator
F2Sb2Se4Sr2_ICSD_171430	insulator	insulator
F2Se3Sn1Sr2_ICSD_171345	insulator	insulator
F2Sn1_ICSD_10485	insulator	insulator
F2Sn1_ICSD_14194	insulator	insulator
F2Sn1_ICSD_14195	insulator	insulator
F2Sr1_ICSD_260881	insulator	insulator
F2Sr1_ICSD_262350	insulator	insulator
F2Ti1_ICSD_65410	insulator	insulator
F2V1_ICSD_201245	insulator	insulator
F2Xe1_ICSD_26625	insulator	insulator
F2Zn1_ICSD_20364	insulator	insulator
F2Zn1_ICSD_280605	insulator	insulator
F2_ICSD_15821	metal	insulator
F2_ICSD_16262	insulator	insulator
F2_ICSD_22271	insulator	insulator
F3Fe1K0.6_ICSD_2603	metal	insulator
F3Fe1K1_ICSD_15424	metal	insulator
F3Fe1Na1_ICSD_68981	insulator	insulator
F3Fe1Rb1_ICSD_49586	metal	insulator
F3Fe1_ICSD_202047	insulator	insulator
F3Fe1_ICSD_240399	insulator	insulator
F3Fe1_ICSD_29132	insulator	insulator
F3Ga1H3N1_ICSD_89503	insulator	insulator
F3Ga1_ICSD_409507	insulator	insulator
F3H1Sr1_ICSD_35408	insulator	insulator
F3H2P1_ICSD_406359	insulator	insulator
F3H6O3V1_ICSD_69571	metal	insulator
F3Hg1Rb1_ICSD_15169	insulator	insulator
F3Ho1_ICSD_200955	insulator	insulator
F3I1O1_ICSD_4076	insulator	insulator
F3I1Sn2_ICSD_2419	insulator	insulator
F3I1_ICSD_411036	insulator	insulator
F3In1_ICSD_18028	insulator	insulator
F3Ir1_ICSD_16676	metal	insulator
F3Ir1_ICSD_77619	insulator	metal
F3K1Mg1_ICSD_56096	insulator	insulator
F3K1Mn1_ICSD_246921	insulator	insulator
F3K1Mn1_ICSD_35507	insulator	insulator

Supplementary Table 366. Five-fold cross validated predictions for the metal/insulator classification (358/598).

system	calculated	predicted
F3K1Mn1_ICSD_37254	insulator	insulator
F3K1Mn1_ICSD_4054	insulator	insulator
F3K1Mn1_ICSD_89343	insulator	insulator
F3K1Ni1_ICSD_44786	insulator	insulator
F3K1O1Te1_ICSD_201155	insulator	insulator
F3K1Pd1_ICSD_73167	insulator	insulator
F3K1V1_ICSD_28145	insulator	insulator
F3K1Zn1_ICSD_63157	insulator	insulator
F3K2O2V1_ICSD_9122	insulator	insulator
F3K3N3O9Sb1_ICSD_35677	insulator	insulator
F3K3O4Ti1_ICSD_39273	insulator	insulator
F3K3O4Ti1_ICSD_39811	insulator	insulator
F3La1_ICSD_164054	insulator	insulator
F3La1_ICSD_164055	insulator	insulator
F3La1_ICSD_167553	insulator	insulator
F3La1_ICSD_16964	insulator	insulator
F3La1_ICSD_246323	insulator	insulator
F3La1_ICSD_28538	insulator	insulator
F3La1_ICSD_34108	insulator	insulator
F3La1_ICSD_96134	insulator	insulator
F3La3O9Si3_ICSD_89522	insulator	insulator
F3Mg1Na1_ICSD_156157	insulator	insulator
F3Mg1Na1_ICSD_156158	insulator	insulator
F3Mg1Na1_ICSD_171813	insulator	insulator
F3Mg1Na1_ICSD_90283	insulator	insulator
F3Mg1Rb1_ICSD_49585	insulator	insulator
F3Mg3N1_ICSD_262326	insulator	insulator
F3Mn1Na1_ICSD_65770	insulator	insulator
F3Mn1Rb1_ICSD_43722	insulator	insulator
F3Mn1_ICSD_19080	insulator	insulator
F3Mn1_ICSD_73113	insulator	insulator
F3Mo1Na3O3_ICSD_97452	insulator	insulator
F3Mo1_ICSD_30612	insulator	metal
F3Mo1_ICSD_68527	insulator	insulator
F3N3S3_ICSD_21015	insulator	insulator
F3Na1Ni1_ICSD_423598	insulator	insulator
F3Na1Ni1_ICSD_9008	insulator	insulator
F3Na1V1_ICSD_60611	insulator	insulator
F3Na1Zn1_ICSD_69349	insulator	insulator
F3Na7O12S3Sn1_ICSD_250242	insulator	insulator
F3Nb1_ICSD_60246	metal	insulator
F3Nd1_ICSD_155557	insulator	insulator
F3Nd1_ICSD_16967	insulator	insulator
F3Nd1_ICSD_63049	insulator	insulator
F3Ni1_ICSD_87943	insulator	insulator
F3O1Rb1V1_ICSD_249403	insulator	insulator
F3O1V1_ICSD_249507	insulator	insulator
F3O2Re1_ICSD_415421	insulator	insulator
F3O4P1Sn3_ICSD_37133	insulator	insulator
F3Pb1Rb1_ICSD_49591	insulator	insulator

Supplementary Table 367. Five-fold cross validated predictions for the metal/insulator classification (359/598).

system	calculated	predicted
F3Pd1Rb1_ICSD_73166	insulator	insulator
F3Pd1_ICSD_16675	metal	insulator
F3Pd1_ICSD_29135	metal	insulator
F3Pr1_ICSD_16966	insulator	insulator
F3Pr1_ICSD_77741	insulator	insulator
F3Pu1_ICSD_29013	metal	insulator
F3Rb1V1_ICSD_28146	insulator	insulator
F3Rb1Yb1_ICSD_49590	insulator	insulator
F3Rb1Zn1_ICSD_41618	insulator	insulator
F3Rh1_ICSD_16674	metal	insulator
F3Rh1_ICSD_29134	insulator	insulator
F3Rh1_ICSD_62262	insulator	metal
F3Ru1_ICSD_16673	insulator	insulator
F3Sb1_ICSD_30411	insulator	insulator
F3Sc1_ICSD_261083	insulator	insulator
F3Sc1_ICSD_30215	insulator	insulator
F3Sc1_ICSD_36011	insulator	insulator
F3Ta1_ICSD_30613	metal	insulator
F3Tb1_ICSD_167473	insulator	insulator
F3Ti1_ICSD_28783	metal	insulator
F3Ti1_ICSD_52159	insulator	insulator
F3Ti1_ICSD_52163	metal	insulator
F3Tl1_ICSD_10365	insulator	insulator
F3U1_ICSD_24966	insulator	insulator
F3V1_ICSD_30624	insulator	insulator
F3Y1_ICSD_15961	insulator	insulator
F3Y1_ICSD_6023	insulator	insulator
F3Yb1_ICSD_9844	insulator	insulator
F4Fe1K1_ICSD_60615	insulator	insulator
F4Fe1Na1_ICSD_15501	insulator	insulator
F4Fe1Rb1_ICSD_21071	insulator	insulator
F4Fe1Rb1_ICSD_21072	insulator	insulator
F4Fe1Rb1_ICSD_85499	insulator	insulator
F4Fe1Rb1_ICSD_85500	insulator	insulator
F4Ga1H4N1_ICSD_410849	insulator	insulator
F4Ge1_ICSD_202558	insulator	insulator
F4H1K1O1Te1_ICSD_155199	insulator	insulator
F4H1Mn1Na2O3P1_ICSD_424942	insulator	insulator
F4H1O1Rb1Te1_ICSD_155198	insulator	insulator
F4H1P1_ICSD_406358	insulator	insulator
F4H2O3Te2_ICSD_9653	insulator	insulator
F4H3K1_ICSD_202107	insulator	insulator
F4H3N1Zr1_ICSD_50233	insulator	insulator
F4H6N2Si1_ICSD_78902	insulator	insulator
F4H6O1_ICSD_32569	insulator	insulator
F4H7In1N2O1_ICSD_35	insulator	insulator
F4H7N1_ICSD_38337	insulator	insulator
F4H8N2O1V1_ICSD_23317	insulator	insulator
F4H8N2_ICSD_87361	insulator	insulator
F4Hf1_ICSD_66008	insulator	insulator

Supplementary Table 368. Five-fold cross validated predictions for the metal/insulator classification (360/598).

system	calculated	predicted
F4Hg1Rb2_ICSD_72352	insulator	insulator
F4In1Li1_ICSD_66693	insulator	insulator
F4Ir1_ICSD_23483	insulator	insulator
F4K1Mn1_ICSD_72394	insulator	insulator
F4K1Mo1Na1O2_ICSD_422707	insulator	insulator
F4K1O1V1_ICSD_9295	insulator	insulator
F4K1Pr1_ICSD_281289	insulator	insulator
F4K1Sc1_ICSD_72736	insulator	insulator
F4K2Mg1_ICSD_33519	insulator	insulator
F4K2Mn1_ICSD_23183	insulator	insulator
F4K2Ni1_ICSD_15576	insulator	insulator
F4K2O1Te1_ICSD_412955	insulator	insulator
F4K2Pd1_ICSD_33888	insulator	insulator
F4K2Zn1_ICSD_100298	insulator	insulator
F4La1Rb1_ICSD_262425	insulator	insulator
F4Li1Lu1_ICSD_152948	insulator	insulator
F4Li1Mn1_ICSD_62655	insulator	insulator
F4Li1O2Re1_ICSD_280011	insulator	insulator
F4Li1Sc1_ICSD_413966	insulator	insulator
F4Li1Y1_ICSD_27896	insulator	insulator
F4Li1Y1_ICSD_55692	insulator	insulator
F4Li1Yb1_ICSD_9914	insulator	insulator
F4Mg1Rb2_ICSD_69681	insulator	insulator
F4Mg1Sr1_ICSD_86248	insulator	insulator
F4Mn1Na1_ICSD_71456	insulator	insulator
F4Mn1Rb1_ICSD_73587	insulator	insulator
F4N4S4_ICSD_18202	insulator	insulator
F4Na1Sb1_ICSD_24750	insulator	insulator
F4Na1Ti1_ICSD_389	insulator	insulator
F4Na1V1_ICSD_407013	insulator	insulator
F4Na2O2W1_ICSD_201173	insulator	insulator
F4Na2Pd1_ICSD_71101	insulator	insulator
F4Nb1_ICSD_25768	insulator	insulator
F4Ni1Rb2_ICSD_69682	insulator	insulator
F4O1Os1_ICSD_417246	insulator	insulator
F4O1Ru1_ICSD_417249	insulator	insulator
F4O1Tc1_ICSD_16143	insulator	insulator
F4O1U1_ICSD_200058	insulator	insulator
F4O1W1_ICSD_10393	insulator	insulator
F4O2Sn4_ICSD_948	insulator	insulator
F4Pb1Pd1_ICSD_108992	insulator	insulator
F4Pb1Sn1_ICSD_152954	insulator	insulator
F4Pb1_ICSD_78895	insulator	insulator
F4Pd1Sr1_ICSD_108990	insulator	insulator
F4Pd1_ICSD_1555	insulator	insulator
F4Pt1_ICSD_71579	insulator	insulator
F4Ru1_ICSD_165398	insulator	insulator
F4S1W1_ICSD_37245	insulator	insulator
F4S2Yb3_ICSD_92497	metal	insulator
F4Sb1Tl1_ICSD_201084	insulator	insulator

Supplementary Table 369. Five-fold cross validated predictions for the metal/insulator classification (361/598).

system	calculated	predicted
F4Se1_ICSD_85451	insulator	insulator
F4Si1_ICSD_24500	insulator	insulator
F4Sn1_ICSD_16794	insulator	insulator
F4Sr1Zn1_ICSD_16807	insulator	insulator
F4Tc1_ICSD_249918	insulator	insulator
F4Te1_ICSD_9869	insulator	insulator
F4V1_ICSD_65785	insulator	insulator
F4Xe1_ICSD_26626	insulator	insulator
F4Zr1_ICSD_35100	insulator	insulator
F5Fe1H2K2O1_ICSD_16090	insulator	insulator
F5Fe1H4Mn1O2_ICSD_202193	insulator	insulator
F5Fe1H4Mn1O2_ICSD_62362	insulator	insulator
F5Fe1H4O2Zn1_ICSD_202194	insulator	insulator
F5Fe2H4O2_ICSD_201799	insulator	insulator
F5Ga1H4Mn1O2_ICSD_245172	insulator	insulator
F5H2K2Mn1O1_ICSD_26071	insulator	insulator
F5H2K2O1V1_ICSD_59818	insulator	insulator
F5H2Mn1O1Rb2_ICSD_2435	insulator	insulator
F5H2Mn1O1Tl2_ICSD_71517	insulator	insulator
F5H2Mn1O1Tl2_ICSD_71518	insulator	insulator
F5H2N1Te1_ICSD_171449	insulator	insulator
F5H3N1P1_ICSD_36010	insulator	insulator
F5H4Mn1O2V1_ICSD_80157	metal	insulator
F5H8N1_ICSD_38338	insulator	insulator
F5H8N2Nb1O2_ICSD_200233	insulator	insulator
F5Hf1Rb1_ICSD_95846	insulator	insulator
F5I1N2O4_ICSD_406388	insulator	insulator
F5I1_ICSD_6021	insulator	insulator
F5K1Li1Y1_ICSD_39668	insulator	insulator
F5K1Na1Nb1O1_ICSD_422708	insulator	insulator
F5K1Te1_ICSD_26051	insulator	insulator
F5K2Pd1Rb1_ICSD_33887	insulator	insulator
F5K2Sb1_ICSD_24742	insulator	insulator
F5K2Sb1_ICSD_39634	insulator	insulator
F5K2Ti1_ICSD_261469	insulator	insulator
F5Li2Mn1_ICSD_202394	insulator	insulator
F5Lu3O2_ICSD_80365	insulator	insulator
F5Mn1Rb2_ICSD_82218	insulator	insulator
F5Mo1_ICSD_26644	metal	insulator
F5N3W1_ICSD_201198	insulator	insulator
F5Na1Te1_ICSD_202879	insulator	insulator
F5Na2Sb1_ICSD_28061	insulator	insulator
F5Nb1_ICSD_26647	insulator	insulator
F5O1Os1_ICSD_16872	insulator	insulator
F5O1Os1_ICSD_240332	insulator	insulator
F5O1Sn2_ICSD_409393	insulator	insulator
F5O2S1Sb1_ICSD_67273	insulator	insulator
F5O2Sb3_ICSD_62476	insulator	insulator
F5O4Tc1Xe1_ICSD_280249	insulator	insulator
F5P1_ICSD_62554	insulator	insulator

Supplementary Table 370. Five-fold cross validated predictions for the metal/insulator classification (362/598).

system	calculated	predicted
F5Pd1Rb3_ICSD_35285	insulator	insulator
F5Pd2Rb1_ICSD_72299	insulator	insulator
F5Sb1Sr1_ICSD_68454	insulator	insulator
F5Ta1_ICSD_171155	insulator	insulator
F5Te1Tl1_ICSD_90619	insulator	insulator
F5Tl1Zr1_ICSD_26558	insulator	insulator
F5U1_ICSD_31657	insulator	insulator
F5U1_ICSD_659	insulator	insulator
F6Fe1H12N3_ICSD_96590	metal	insulator
F6Fe1H12O6Sn1_ICSD_68452	insulator	insulator
F6Fe1H8N2Na1_ICSD_418736	insulator	insulator
F6Fe1K1Rb2_ICSD_9001	insulator	insulator
F6Fe1K2Na1_ICSD_61277	insulator	insulator
F6Fe1Li1Mn1_ICSD_35231	insulator	insulator
F6Fe1Li1Mn1_ICSD_35233	insulator	insulator
F6Fe1Li1Rb2_ICSD_16400	insulator	insulator
F6Fe1Li1Sr1_ICSD_32729	insulator	insulator
F6Fe1Na1Rb2_ICSD_40966	insulator	insulator
F6Fe1Na1Rb2_ICSD_42146	insulator	insulator
F6Fe1Na3_ICSD_20157	insulator	insulator
F6Fe1Nb1_ICSD_63307	insulator	insulator
F6Fe1Nb1_ICSD_63309	insulator	insulator
F6Fe1Sn1_ICSD_25011	metal	insulator
F6Fe1Zr1_ICSD_35716	metal	insulator
F6Fe1Zr1_ICSD_35725	insulator	metal
F6Fe2Li1_ICSD_202705	insulator	insulator
F6Fe2Rb1_ICSD_186747	insulator	insulator
F6Ga1H8N2Na1_ICSD_418737	insulator	insulator
F6Ga1K1Rb2_ICSD_416605	insulator	insulator
F6Ga1Li1Mn1_ICSD_4045	insulator	insulator
F6Ga1Li1Pd1_ICSD_78748	insulator	insulator
F6Ga1Li1Rb2_ICSD_50468	insulator	insulator
F6Ge1H12Mg1O6_ICSD_401096	insulator	insulator
F6Ge1K2_ICSD_24026	insulator	insulator
F6Ge1K2_ICSD_30310	insulator	insulator
F6Ge1Li2_ICSD_23406	insulator	insulator
F6Ge1Li2_ICSD_23407	insulator	insulator
F6Ge1Na2_ICSD_30344	insulator	insulator
F6Ge1Na2_ICSD_69623	insulator	insulator
F6Ge1Rb2_ICSD_25662	insulator	insulator
F6Ge1Rb2_ICSD_26633	insulator	insulator
F6Ge1Rb2_ICSD_68982	insulator	insulator
F6H10O4Si1_ICSD_40388	insulator	insulator
F6H12Mn1O6Ti1_ICSD_202786	insulator	insulator
F6H12Ni1O6Sn1_ICSD_75985	insulator	insulator
F6H12O6Sn2Zn1_ICSD_166589	insulator	insulator
F6H12O6Ti1Zn1_ICSD_62375	insulator	insulator
F6H2N3Sb1_ICSD_41354	insulator	insulator
F6H3O1Sb1_ICSD_66552	insulator	insulator
F6H6N2Si1_ICSD_35702	insulator	insulator

Supplementary Table 371. Five-fold cross validated predictions for the metal/insulator classification (363/598).

system	calculated	predicted
F6H8In1N2Na1_ICSD_79099	insulator	insulator
F6H8N2Sn1_ICSD_409509	insulator	insulator
F6H8N2Ti1_ICSD_24834	insulator	insulator
F6H8N2Ti1_ICSD_24835	insulator	insulator
F6Hf1K2_ICSD_29514	insulator	insulator
F6Hf1K2_ICSD_47243	insulator	insulator
F6Hf1K2_ICSD_47244	insulator	insulator
F6Hf1K2_ICSD_47246	insulator	insulator
F6Hf1Rb2_ICSD_25599	insulator	insulator
F6Hf1V1_ICSD_94455	insulator	insulator
F6Hg1Rh1_ICSD_42163	metal	insulator
F6Hg2Mn2S1_ICSD_27328	metal	insulator
F6Hg2Ni2O1_ICSD_27329	insulator	insulator
F6Hg2Ni2S1_ICSD_4083	insulator	insulator
F6Hg2O1Zn2_ICSD_27330	insulator	insulator
F6Hg3Nb1_ICSD_62027	metal	insulator
F6Hg3S2Si1_ICSD_23605	insulator	insulator
F6Hg4O1_ICSD_99995	metal	insulator
F6Ho1Na1Rb2_ICSD_86273	insulator	insulator
F6Ho1Na1Rb2_ICSD_86274	insulator	insulator
F6I5Sb1_ICSD_27557	insulator	insulator
F6In1K2Na1_ICSD_22113	insulator	insulator
F6Ir1K2_ICSD_95779	insulator	insulator
F6Ir1Li1_ICSD_165207	metal	insulator
F6Ir1Rb2_ICSD_240955	insulator	insulator
F6Ir1_ICSD_171654	insulator	insulator
F6K1Mo1Rb2_ICSD_15773	insulator	insulator
F6K1Mo1Tl2_ICSD_15775	insulator	insulator
F6K1Na1Sn1_ICSD_39590	insulator	insulator
F6K1Nb1_ICSD_16729	insulator	insulator
F6K1Ni1Rb2_ICSD_42149	metal	insulator
F6K1Os1_ICSD_27664	insulator	insulator
F6K1P1_ICSD_25576	insulator	insulator
F6K1P1_ICSD_56255	insulator	insulator
F6K1Rb2Rh1_ICSD_27339	insulator	insulator
F6K1Rb2Sc1_ICSD_81679	insulator	insulator
F6K1Rb2Sc1_ICSD_81681	insulator	insulator
F6K1Rb2Sc1_ICSD_81683	insulator	insulator
F6K1Rb2Ti1_ICSD_42142	insulator	insulator
F6K1Rb2V1_ICSD_27345	insulator	insulator
F6K1Rb2Y1_ICSD_73472	insulator	insulator
F6K1Rh1_ICSD_165215	insulator	insulator
F6K1Ru1_ICSD_28692	insulator	insulator
F6K1Sb1_ICSD_42509	insulator	insulator
F6K1Sb1_ICSD_632	insulator	insulator
F6K1Sb1_ICSD_76422	insulator	insulator
F6K2Mn1Na1_ICSD_61117	insulator	insulator
F6K2Mn1_ICSD_47213	insulator	insulator
F6K2Mn1_ICSD_60417	insulator	insulator
F6K2Mo1Na1_ICSD_15777	insulator	insulator

Supplementary Table 372. Five-fold cross validated predictions for the metal/insulator classification (364/598).

system	calculated	predicted
F6K2Na1Ni1_ICSD_42151	insulator	insulator
F6K2Na1Rh1_ICSD_27342	insulator	insulator
F6K2Na1Sc1_ICSD_22112	insulator	insulator
F6K2Na1Tl1_ICSD_22114	insulator	insulator
F6K2Na1V1_ICSD_186883	insulator	insulator
F6K2Na1Y1_ICSD_22115	insulator	insulator
F6K2Ni1_ICSD_6046	insulator	insulator
F6K2O3Ta2_ICSD_15866	insulator	insulator
F6K2Pd1_ICSD_27486	insulator	insulator
F6K2Pt1_ICSD_87360	insulator	insulator
F6K2Re1_ICSD_1528	insulator	insulator
F6K2Rh1_ICSD_28779	insulator	insulator
F6K2Si1_ICSD_158483	insulator	insulator
F6K2Si1_ICSD_38546	insulator	insulator
F6K2Tb1_ICSD_245618	metal	insulator
F6K2Th1_ICSD_31609	insulator	insulator
F6K2Ti1_ICSD_24659	insulator	insulator
F6K2U1_ICSD_31604	insulator	insulator
F6K2U1_ICSD_31610	insulator	insulator
F6K2Zr1_ICSD_14126	insulator	insulator
F6K2Zr1_ICSD_865	insulator	insulator
F6K3Mo1_ICSD_4403	insulator	insulator
F6K3N1O3Si1_ICSD_159390	insulator	insulator
F6K3W1_ICSD_51264	insulator	insulator
F6K3Y1_ICSD_416296	insulator	insulator
F6Li1Mn1Ti1_ICSD_69047	insulator	insulator
F6Li1Mn1V1_ICSD_167073	insulator	insulator
F6Li1Nb1_ICSD_165202	insulator	insulator
F6Li1Ni1Sr1_ICSD_32733	insulator	insulator
F6Li1Os1_ICSD_165214	insulator	insulator
F6Li1P1_ICSD_74830	insulator	insulator
F6Li1Pt1_ICSD_165208	insulator	insulator
F6Li1Rh1_ICSD_95776	metal	insulator
F6Li1Ru1_ICSD_165203	insulator	insulator
F6Li1Sb1_ICSD_23924	insulator	insulator
F6Li1Ta1_ICSD_165205	insulator	insulator
F6Li1V2_ICSD_35382	insulator	insulator
F6Li2Mn1_ICSD_15791	insulator	insulator
F6Li2Mo1_ICSD_23173	insulator	insulator
F6Li2Nb1_ICSD_201755	insulator	insulator
F6Li2O3Ta2_ICSD_405777	insulator	insulator
F6Li2Pd1_ICSD_165212	insulator	insulator
F6Li2Pd1_ICSD_26167	metal	insulator
F6Li2Pt1_ICSD_165216	insulator	insulator
F6Li2Pt1_ICSD_26168	metal	insulator
F6Li2Rh1_ICSD_165211	insulator	insulator
F6Li2Rh1_ICSD_6044	insulator	insulator
F6Li2Rh1_ICSD_95778	metal	insulator
F6Li2Ru1_ICSD_165210	insulator	insulator
F6Li2Ti1_ICSD_18313	insulator	insulator

Supplementary Table 373. Five-fold cross validated predictions for the metal/insulator classification (365/598).

system	calculated	predicted
F6Li2Zr1_ICSD_155020	insulator	insulator
F6Li2Zr1_ICSD_2644	insulator	insulator
F6Li2Zr1_ICSD_409667	insulator	insulator
F6Mg1Pb1_ICSD_15106	insulator	insulator
F6Mg1Pd1_ICSD_26163	insulator	insulator
F6Mg1Rh1_ICSD_42159	insulator	insulator
F6Mn1Na1Rb2_ICSD_9708	insulator	insulator
F6Mn1Na3_ICSD_66315	insulator	insulator
F6Mn1Pt1_ICSD_73114	insulator	insulator
F6Mn1Rb2_ICSD_25578	insulator	insulator
F6Mn1Rb2_ICSD_25579	insulator	insulator
F6Mn1Sn1_ICSD_25010	insulator	insulator
F6Mo1Na1Rb2_ICSD_15774	insulator	insulator
F6Mo1Na1Tl2_ICSD_15776	insulator	insulator
F6Mo1Na1_ICSD_27484	metal	insulator
F6Mo1_ICSD_171653	insulator	insulator
F6Mo1_ICSD_1879	insulator	insulator
F6N1Re1_ICSD_33543	insulator	insulator
F6N1S2Sb1_ICSD_170871	insulator	insulator
F6N3P3_ICSD_26649	insulator	insulator
F6Na1Ni1Rb2_ICSD_42150	insulator	insulator
F6Na1P1_ICSD_90615	insulator	insulator
F6Na1Rb2Rh1_ICSD_27341	insulator	insulator
F6Na1Rb2Ti1_ICSD_42143	insulator	insulator
F6Na1Rb2V1_ICSD_27346	insulator	insulator
F6Na1Rb2Y1_ICSD_25369	insulator	insulator
F6Na1Sb1_ICSD_25538	insulator	insulator
F6Na1Sb1_ICSD_56251	insulator	insulator
F6Na2Nb1_ICSD_201756	insulator	insulator
F6Na2Si1_ICSD_30348	insulator	insulator
F6Na2Si1_ICSD_40917	insulator	insulator
F6Na2Si1_ICSD_61274	insulator	insulator
F6Na2Sn1_ICSD_23156	metal	insulator
F6Na2Sn1_ICSD_68980	insulator	insulator
F6Na2Th1_ICSD_418145	insulator	insulator
F6Na2Ti1_ICSD_24477	insulator	insulator
F6Na2Ti1_ICSD_40916	insulator	insulator
F6Na2U1_ICSD_31605	insulator	insulator
F6Na2U1_ICSD_31623	insulator	insulator
F6Na2U1_ICSD_8034	insulator	insulator
F6Na2Zr1_ICSD_9877	insulator	insulator
F6Na3Nb1O1_ICSD_35761	insulator	insulator
F6Na3Ni1_ICSD_26073	insulator	insulator
F6Na3Sc1_ICSD_401761	insulator	insulator
F6Na3V1_ICSD_27347	insulator	insulator
F6Nb1Rb2_ICSD_72831	insulator	insulator
F6Nb1V1_ICSD_73353	metal	insulator
F6Ni1Pb1_ICSD_15108	insulator	insulator
F6Ni1Pt1_ICSD_78905	insulator	insulator
F6Ni1Rb2_ICSD_29005	insulator	insulator

Supplementary Table 374. Five-fold cross validated predictions for the metal/insulator classification (366/598).

system	calculated	predicted
F6Ni1Rh1_ICSD_42164	insulator	insulator
F6Ni1Sn1_ICSD_25015	insulator	insulator
F6Ni1Sr1_ICSD_30114	insulator	insulator
F6Ni2Rb1_ICSD_31781	insulator	insulator
F6O1W1Xe1_ICSD_1853	insulator	insulator
F6O2P1Sb1_ICSD_51535	insulator	insulator
F6O2Pt1_ICSD_78851	insulator	insulator
F6O2Ru1_ICSD_78850	metal	insulator
F6O2Sb1_ICSD_78849	insulator	insulator
F6O4Pb1Zr3_ICSD_60959	insulator	insulator
F6O9Si2Ti1Y4_ICSD_20003	insulator	insulator
F6Os1_ICSD_171657	insulator	insulator
F6P1Tl1_ICSD_28899	insulator	insulator
F6Pa1Rb1_ICSD_36078	insulator	insulator
F6Pb1Pt1_ICSD_4057	insulator	insulator
F6Pb1Sr1_ICSD_25629	metal	insulator
F6Pb1Te1_ICSD_81865	insulator	insulator
F6Pb1Zn1_ICSD_15107	insulator	insulator
F6Pb1Zr1_ICSD_4051	insulator	insulator
F6Pb2_ICSD_23467	insulator	insulator
F6Pd1Pt1_ICSD_64661	insulator	insulator
F6Pd1Rb2_ICSD_28675	insulator	insulator
F6Pd1Zn1_ICSD_26165	insulator	insulator
F6Pd1Zr1_ICSD_73133	insulator	insulator
F6Pt1Rb2_ICSD_35108	insulator	insulator
F6Pt1Zn1_ICSD_37444	insulator	insulator
F6Rb1Ru1_ICSD_28693	insulator	insulator
F6Rb1Sb1_ICSD_408071	insulator	insulator
F6Rb2Rh1_ICSD_28780	insulator	insulator
F6Rb2Si1_ICSD_38547	insulator	insulator
F6Rb2Tb1_ICSD_99248	metal	insulator
F6Rb2Th1_ICSD_25829	insulator	insulator
F6Rb2U1_ICSD_14116	insulator	insulator
F6Rb2Zr1_ICSD_25597	insulator	insulator
F6Rb3Tl1_ICSD_19075	insulator	insulator
F6Rb3Y1_ICSD_19077	insulator	insulator
F6Rh1Sr1_ICSD_42158	metal	insulator
F6Rh1Zn1_ICSD_42161	insulator	insulator
F6S1_ICSD_214	insulator	insulator
F6S1_ICSD_41229	insulator	insulator
F6S1_ICSD_63334	insulator	insulator
F6S1_ICSD_71013	insulator	insulator
F6Sb1Tl1_ICSD_36264	insulator	insulator
F6Si1Tl2_ICSD_38549	insulator	insulator
F6Sn1Tl2_ICSD_410801	insulator	insulator
F6Sn1Zn1_ICSD_25012	insulator	insulator
F6Sn2_ICSD_33786	insulator	insulator
F6Te1_ICSD_67609	insulator	insulator
F6Ti1Tl2_ICSD_410802	insulator	insulator
F6Ti1Tl3_ICSD_42154	metal	insulator

Supplementary Table 375. Five-fold cross validated predictions for the metal/insulator classification (367/598).

system	calculated	predicted
F6Ti1Zr1_ICSD_94456	insulator	insulator
F6U1_ICSD_9000	insulator	insulator
F6V1Zr1_ICSD_94454	insulator	insulator
F6W1_ICSD_36220	insulator	insulator
F6Xe2_ICSD_18128	insulator	insulator
F7Fe1Na2Ni1_ICSD_1511	insulator	insulator
F7Fe1Na2Ni1_ICSD_202870	insulator	insulator
F7Ga1Na2Ni1_ICSD_408111	insulator	insulator
F7Gd1Sn1_ICSD_78898	insulator	insulator
F7H1I1N1O1_ICSD_71582	insulator	insulator
F7H1K2Ti1_ICSD_187321	insulator	insulator
F7H1K3Nb1O1_ICSD_26620	insulator	insulator
F7Ho2K1_ICSD_27196	insulator	insulator
F7I1Xe1_ICSD_26059	insulator	insulator
F7I1_ICSD_18191	insulator	insulator
F7In1Sr2_ICSD_38307	insulator	insulator
F7In2K1_ICSD_1307	insulator	insulator
F7K1Sb2_ICSD_14118	insulator	insulator
F7K1Sc2_ICSD_47227	insulator	insulator
F7K1Ti2_ICSD_174209	insulator	insulator
F7K1Yb2_ICSD_16350	insulator	insulator
F7K2Nb1_ICSD_14132	insulator	insulator
F7K2Pa1_ICSD_24144	insulator	insulator
F7K2Ta1_ICSD_260876	insulator	insulator
F7K3N2O6Sb2_ICSD_200740	metal	insulator
F7K3Si1_ICSD_23875	insulator	insulator
F7La1Sn1_ICSD_78896	insulator	insulator
F7Li3Th1_ICSD_1726	insulator	insulator
F7Mn1Rb1Zr1_ICSD_78042	insulator	insulator
F7Mn1Rb3_ICSD_9599	insulator	insulator
F7Mn1Tl1Zr1_ICSD_78041	insulator	insulator
F7Na2Ni1V1_ICSD_407015	insulator	insulator
F7Ni1Rb3_ICSD_9601	insulator	insulator
F7Pb1Ta1_ICSD_417253	insulator	insulator
F7Pb2Rh1_ICSD_37141	insulator	insulator
F7Rb3Si1_ICSD_9589	insulator	insulator
F7Rb3Ti1_ICSD_9595	insulator	insulator
F7Re1_ICSD_78311	insulator	insulator
F7Sb1Xe1_ICSD_411868	insulator	insulator
F7Sc1Sr2_ICSD_74360	insulator	insulator
F7Sn1Tl1_ICSD_78899	insulator	insulator
F7Sn1Y1_ICSD_71586	insulator	insulator
F7Sn1Y1_ICSD_74963	insulator	insulator
F7Sr1Ta1_ICSD_417254	insulator	insulator
F8Fe3H4O2_ICSD_37140	insulator	insulator
F8Fe4O8Sr6_ICSD_262347	metal	insulator
F8H10In2N2O2_ICSD_97067	insulator	insulator
F8H12O6Zr2_ICSD_1309	insulator	insulator
F8H1K3Sn1_ICSD_26993	insulator	insulator
F8H1Na3Ti1_ICSD_14131	insulator	insulator

Supplementary Table 376. Five-fold cross validated predictions for the metal/insulator classification (368/598).

system	calculated	predicted
F8I2Pb5_ICSD_36334	insulator	insulator
F8K3Ta1_ICSD_248063	insulator	insulator
F8Li1Na1Y2_ICSD_201623	insulator	insulator
F8Li1Na1Yb2_ICSD_202052	insulator	insulator
F8N2O2Xe1_ICSD_23535	insulator	insulator
F8N4P4_ICSD_31125	insulator	insulator
F8Na2U1_ICSD_165293	insulator	insulator
F8Na3Pa1_ICSD_16153	insulator	insulator
F8Na3Ta1_ICSD_260875	insulator	insulator
F8Sn3_ICSD_32592	insulator	insulator
F8Sn3_ICSD_61162	insulator	insulator
F9K1Te2_ICSD_84364	insulator	insulator
F9K1U2_ICSD_16065	insulator	insulator
F9K5Th1_ICSD_14127	insulator	insulator
F9Na1Th2_ICSD_245846	insulator	insulator
F9Nb1Se1_ICSD_9898	insulator	insulator
F9Sb1Xe2_ICSD_90622	insulator	insulator
F9U2_ICSD_27858	metal	insulator
Fe0.25S2Ta1_ICSD_417150	metal	metal
Fe1.125Se1_ICSD_167195	metal	insulator
Fe11Si5_ICSD_77369	metal	metal
Fe12Ho2P7_ICSD_632334	metal	metal
Fe12O19Sr1_ICSD_16158	metal	metal
Fe12P7Tb2_ICSD_633107	metal	metal
Fe12P7Tb2_ICSD_633108	metal	metal
Fe12P7Zr2_ICSD_25757	metal	metal
Fe12Y1_ICSD_168236	metal	metal
Fe13Ge3_ICSD_150584	metal	metal
Fe16Hf6Si7_ICSD_150951	metal	metal
Fe16N2_ICSD_185976	metal	metal
Fe16Nb6Si7_ICSD_107097	metal	metal
Fe16Si7Ta6_ICSD_107098	metal	metal
Fe16Si7Zr6_ICSD_601928	metal	metal
Fe17N3Nd2_ICSD_603452	metal	metal
Fe17N3Nd2_ICSD_632695	metal	metal
Fe17N3Pr2_ICSD_632713	metal	metal
Fe17N3Tb2_ICSD_632743	metal	metal
Fe17Nd2_ICSD_103550	metal	metal
Fe17Nd2_ICSD_107530	metal	metal
Fe17Nd2_ICSD_164327	metal	metal
Fe17Pr2_ICSD_103593	metal	metal
Fe17Tb2_ICSD_187854	metal	metal
Fe17Tb2_ICSD_633846	metal	metal
Fe17Th2_ICSD_656795	metal	metal
Fe17Y2_ICSD_604267	metal	metal
Fe17Y2_ICSD_634081	metal	metal
Fe1Ga12Ho4_ICSD_249999	metal	metal
Fe1Ga12Tb4_ICSD_249997	metal	metal
Fe1Ga12U4_ICSD_156306	metal	metal
Fe1Ga12Y4_ICSD_249996	metal	metal

Supplementary Table 377. Five-fold cross validated predictions for the metal/insulator classification (369/598).

system	calculated	predicted
Fe1Ga1Ni2_ICSD_187492	metal	metal
Fe1Ga1Ti2_ICSD_185660	metal	metal
Fe1Ga2Hf6_ICSD_631770	metal	metal
Fe1Ga2S4_ICSD_100706	insulator	insulator
Fe1Ga2Se4_ICSD_631817	insulator	insulator
Fe1Ga3_ICSD_103447	metal	insulator
Fe1Ga3_ICSD_631760	insulator	metal
Fe1Ga5U1_ICSD_600547	metal	metal
Fe1Ga6Ge6Tb4_ICSD_281082	metal	metal
Fe1Ga8Ho2_ICSD_180132	metal	metal
Fe1Ga8Lu2_ICSD_600147	metal	metal
Fe1Ga8Tb2_ICSD_600178	metal	metal
Fe1Ga8Tm2_ICSD_106640	metal	metal
Fe1Gd1O3_ICSD_16644	metal	insulator
Fe1Gd1Sb2_ICSD_657989	metal	metal
Fe1Gd1Si1_ICSD_631952	metal	metal
Fe1Ge1Hf1_ICSD_23577	metal	metal
Fe1Ge1Hf1_ICSD_632036	metal	metal
Fe1Ge1O3_ICSD_89787	metal	insulator
Fe1Ge1Sc1_ICSD_632114	metal	metal
Fe1Ge1Si1_ICSD_150274	insulator	metal
Fe1Ge1_ICSD_159921	metal	metal
Fe1Ge1_ICSD_43055	metal	metal
Fe1Ge1_ICSD_632006	metal	metal
Fe1Ge1_ICSD_632022	metal	metal
Fe1Ge2Ho1_ICSD_632046	metal	metal
Fe1Ge2La1_ICSD_632049	metal	metal
Fe1Ge2Li1O6_ICSD_162951	insulator	insulator
Fe1Ge2Na1O6_ICSD_180307	insulator	insulator
Fe1Ge2_ICSD_150276	metal	metal
Fe1Ge2_ICSD_632024	metal	metal
Fe1Ge3La1_ICSD_161874	metal	metal
Fe1Ge3Pr1_ICSD_658628	insulator	metal
Fe1H10N4O8S2_ICSD_240720	insulator	insulator
Fe1H1Li1O5P1_ICSD_167610	insulator	insulator
Fe1H1O2_ICSD_1544	insulator	insulator
Fe1H1O2_ICSD_167358	insulator	insulator
Fe1H1O2_ICSD_245057	metal	insulator
Fe1H1O2_ICSD_24885	insulator	insulator
Fe1H1Ti1_ICSD_602022	metal	metal
Fe1H1_ICSD_187145	metal	metal
Fe1H2K1O6P2_ICSD_423281	insulator	insulator
Fe1H2O5P1_ICSD_167029	insulator	insulator
Fe1H2O5S1_ICSD_71345	insulator	insulator
Fe1H2O5V1_ICSD_87710	insulator	insulator
Fe1H2O6P2Rb1_ICSD_423278	insulator	insulator
Fe1H2Ti1_ICSD_601978	metal	metal
Fe1H3_ICSD_187146	metal	metal
Fe1H3_ICSD_187147	metal	metal
Fe1H4K1O14P4_ICSD_250262	metal	insulator

Supplementary Table 378. Five-fold cross validated predictions for the metal/insulator classification (370/598).

system	calculated	predicted
Fe1H4K2O10S2_ICSD_87958	insulator	insulator
Fe1H4_ICSD_187148	metal	metal
Fe1H5N1O10P3_ICSD_20782	insulator	insulator
Fe1H6Mg2_ICSD_107500	insulator	insulator
Fe1H6Sr2_ICSD_632211	insulator	insulator
Fe1H8O12Re2_ICSD_402963	insulator	insulator
Fe1Hf1P1_ICSD_86280	metal	metal
Fe1Hf1Si1_ICSD_632263	metal	metal
Fe1Hf1Si2_ICSD_62614	metal	metal
Fe1Hf2_ICSD_632245	metal	metal
Fe1Hf4P1_ICSD_632255	metal	metal
Fe1Hf9Mo4_ICSD_632254	metal	metal
Fe1Hf9Re4_ICSD_632258	metal	metal
Fe1Hf9W4_ICSD_632275	metal	metal
Fe1Ho1Si1_ICSD_632349	metal	metal
Fe1Ho6Sb2_ICSD_96251	metal	metal
Fe1Ho7I12_ICSD_260155	metal	metal
Fe1I2_ICSD_52369	metal	insulator
Fe1I3O9_ICSD_286	insulator	insulator
Fe1I3Tl1_ICSD_26422	insulator	insulator
Fe1I4Rb2_ICSD_8010	insulator	insulator
Fe1In1O3_ICSD_80469	insulator	metal
Fe1In1Ti2_ICSD_185661	metal	metal
Fe1In2S4_ICSD_632381	insulator	insulator
Fe1Ir3_ICSD_56264	metal	metal
Fe1K1Mo2O8_ICSD_153864	insulator	insulator
Fe1K1Mo2O8_ICSD_28020	insulator	insulator
Fe1K1O2_ICSD_421186	insulator	insulator
Fe1K1O8S2_ICSD_26004	insulator	insulator
Fe1K1S2_ICSD_632398	metal	insulator
Fe1K1S2_ICSD_68383	insulator	metal
Fe1K1Se2_ICSD_40780	metal	insulator
Fe1K2Li3O4_ICSD_33280	insulator	insulator
Fe1K2Na1O3_ICSD_36380	insulator	insulator
Fe1K2O4_ICSD_2876	insulator	insulator
Fe1K2P2S6_ICSD_300226	insulator	insulator
Fe1K2P2S6_ICSD_657803	insulator	insulator
Fe1K2P2Se6_ICSD_165358	insulator	insulator
Fe1K3O2_ICSD_73215	insulator	insulator
Fe1K3Se3_ICSD_89586	insulator	insulator
Fe1La1O1P1_ICSD_391428	metal	metal
Fe1La1O3_ICSD_236176	insulator	metal
Fe1La1O3_ICSD_29118	insulator	metal
Fe1La1Sb2_ICSD_657914	metal	metal
Fe1La1Si1_ICSD_85853	metal	metal
Fe1La1Si2_ICSD_632434	metal	metal
Fe1La3Mn1S7_ICSD_35581	insulator	insulator
Fe1La3Ni1S7_ICSD_603955	insulator	insulator
Fe1La3O6S3W1_ICSD_380402	insulator	insulator
Fe1La3O6_ICSD_421426	insulator	insulator

Supplementary Table 379. Five-fold cross validated predictions for the metal/insulator classification (371/598).

system	calculated	predicted
Fe1La4O1Se6_ICSD_391306	insulator	insulator
Fe1La4S7_ICSD_632416	insulator	insulator
Fe1La5O16Re3_ICSD_96339	insulator	insulator
Fe1Li1Mo2O8_ICSD_241134	insulator	insulator
Fe1Li1O2_ICSD_51759	metal	metal
Fe1Li1O2_ICSD_78712	metal	metal
Fe1Li1O4P1_ICSD_161479	insulator	insulator
Fe1Li1O4P1_ICSD_97766	insulator	insulator
Fe1Li1O4Si1_ICSD_183763	insulator	insulator
Fe1Li1O4Si1_ICSD_186523	insulator	insulator
Fe1Li1O6Si2_ICSD_9669	insulator	insulator
Fe1Li1O7P2_ICSD_95751	insulator	insulator
Fe1Li1O8W2_ICSD_16170	insulator	insulator
Fe1Li1O9P3_ICSD_20610	insulator	insulator
Fe1Li1P1_ICSD_166456	metal	metal
Fe1Li1P1_ICSD_187134	metal	metal
Fe1Li1P1_ICSD_187135	insulator	metal
Fe1Li2O4Si1_ICSD_161306	insulator	insulator
Fe1Li2O4Si1_ICSD_185581	insulator	insulator
Fe1Li2O4Si1_ICSD_261336	insulator	insulator
Fe1Li2S2_ICSD_632443	metal	metal
Fe1Li3N2_ICSD_68523	metal	insulator
Fe1Li4N2_ICSD_68700	insulator	metal
Fe1Lu2S4_ICSD_37422	metal	metal
Fe1Lu6Sb2_ICSD_96249	metal	metal
Fe1Mn1P1_ICSD_632538	metal	metal
Fe1Mn1P1_ICSD_632548	metal	metal
Fe1Mn2N8Sr8_ICSD_409950	metal	metal
Fe1Mo1O4_ICSD_43012	insulator	insulator
Fe1Mo1O6Sr2_ICSD_169377	insulator	insulator
Fe1Mo1O6Sr2_ICSD_172212	insulator	insulator
Fe1Mo1O7V1_ICSD_408562	insulator	insulator
Fe1Mo1P1_ICSD_632646	metal	metal
Fe1Mo2Na1O8_ICSD_166610	insulator	insulator
Fe1Mo2O8Rb1_ICSD_245665	metal	insulator
Fe1Mo2O8Rb1_ICSD_245666	metal	insulator
Fe1Mo2S4_ICSD_94749	insulator	metal
Fe1Mo2S4_ICSD_94751	insulator	metal
Fe1Mo4Zr9_ICSD_632686	metal	metal
Fe1Mo6S8_ICSD_632656	metal	metal
Fe1N1Ni1_ICSD_53505	metal	metal
Fe1N1_ICSD_184375	metal	metal
Fe1N1_ICSD_41258	metal	metal
Fe1N2Sr2_ICSD_72390	insulator	metal
Fe1N2W1_ICSD_81488	metal	metal
Fe1Na1O2_ICSD_27117	insulator	insulator
Fe1Na1O2_ICSD_33775	insulator	insulator
Fe1Na1O2_ICSD_75588	metal	insulator
Fe1Na1O4P1_ICSD_56292	insulator	insulator
Fe1Na1O4Ti1_ICSD_36090	insulator	insulator

Supplementary Table 380. Five-fold cross validated predictions for the metal/insulator classification (372/598).

system	calculated	predicted
Fe1Na1O6Si2_ICSD_10221	insulator	insulator
Fe1Na1O8S2_ICSD_166768	insulator	insulator
Fe1Na1O8Se2_ICSD_74001	insulator	insulator
Fe1Na1O8W2_ICSD_161847	insulator	insulator
Fe1Na1S2_ICSD_37026	insulator	metal
Fe1Na2O4_ICSD_50478	insulator	insulator
Fe1Na3O8P2_ICSD_93790	insulator	insulator
Fe1Na4O3_ICSD_1410	insulator	insulator
Fe1Na4O4_ICSD_89702	insulator	insulator
Fe1Na6S4_ICSD_72303	insulator	insulator
Fe1Na6Se4_ICSD_72304	insulator	insulator
Fe1Nb1O4_ICSD_14016	insulator	insulator
Fe1Nb1O4_ICSD_429	insulator	insulator
Fe1Nb1O4_ICSD_8128	insulator	insulator
Fe1Nb1P1_ICSD_632794	metal	metal
Fe1Nb1Sb1_ICSD_83928	insulator	insulator
Fe1Nb1Si1_ICSD_632827	metal	metal
Fe1Nb1Te2_ICSD_71520	metal	metal
Fe1Nb2O6_ICSD_31943	insulator	insulator
Fe1Nb2S4_ICSD_632799	metal	metal
Fe1Nb2Se4_ICSD_632807	metal	metal
Fe1Nb3S6_ICSD_42687	metal	metal
Fe1Nb3_ICSD_188227	metal	metal
Fe1Nb3_ICSD_188251	metal	metal
Fe1Nb4P1_ICSD_632795	metal	metal
Fe1Nb4S8_ICSD_632800	metal	metal
Fe1Nb4Se8_ICSD_632812	metal	metal
Fe1Nb4Si1_ICSD_632825	metal	metal
Fe1Nd1Si1_ICSD_85854	metal	metal
Fe1Nd1Si2_ICSD_67089	metal	metal
Fe1Ni1P1_ICSD_632939	metal	metal
Fe1Ni1Pt2_ICSD_42564	metal	metal
Fe1Ni1Si1_ICSD_165243	metal	metal
Fe1Ni1Si1_ICSD_165244	metal	metal
Fe1Ni1_ICSD_103555	metal	metal
Fe1Ni2O4_ICSD_109150	metal	metal
Fe1Ni2S4_ICSD_42590	metal	metal
Fe1Ni2Se4_ICSD_632972	metal	metal
Fe1Ni2_ICSD_188272	metal	metal
Fe1Ni3_ICSD_188242	metal	metal
Fe1Ni3_ICSD_188254	metal	metal
Fe1Ni3_ICSD_632929	metal	metal
Fe1O13Te6_ICSD_417293	insulator	insulator
Fe1O1P1Pr1_ICSD_80200	metal	metal
Fe1O1_ICSD_633029	metal	metal
Fe1O1_ICSD_82236	metal	metal
Fe1O2Rb1_ICSD_421188	insulator	insulator
Fe1O2Sr1_ICSD_418606	metal	metal
Fe1O2Tl1_ICSD_29011	insulator	insulator
Fe1O3Si1_ICSD_75929	insulator	insulator

Supplementary Table 381. Five-fold cross validated predictions for the metal/insulator classification (373/598).

system	calculated	predicted
Fe1O3Sr1_ICSD_92335	metal	metal
Fe1O3Ti1_ICSD_246515	insulator	insulator
Fe1O3Ti1_ICSD_246516	insulator	insulator
Fe1O3Ti1_ICSD_30665	insulator	insulator
Fe1O3Y1_ICSD_23822	metal	insulator
Fe1O3Zr1_ICSD_247122	insulator	insulator
Fe1O4P1_ICSD_155065	insulator	insulator
Fe1O4P1_ICSD_281079	insulator	insulator
Fe1O4P1_ICSD_290337	insulator	insulator
Fe1O4P1_ICSD_38062	insulator	insulator
Fe1O4P1_ICSD_412735	insulator	insulator
Fe1O4S1_ICSD_23507	insulator	insulator
Fe1O4S1_ICSD_240947	insulator	insulator
Fe1O4Sb2_ICSD_155152	insulator	insulator
Fe1O4Sb2_ICSD_4459	insulator	insulator
Fe1O4Si1_ICSD_186518	metal	insulator
Fe1O4Si1_ICSD_186521	insulator	insulator
Fe1O4Si1_ICSD_186524	metal	insulator
Fe1O4Sr2_ICSD_74421	metal	metal
Fe1O4U1_ICSD_15709	insulator	insulator
Fe1O4V1_ICSD_82161	insulator	insulator
Fe1O4V2_ICSD_109149	metal	metal
Fe1O4V2_ICSD_28962	metal	metal
Fe1O4W1_ICSD_26811	insulator	insulator
Fe1O5Ti2_ICSD_37231	insulator	insulator
Fe1O6Re1Sr2_ICSD_150702	metal	metal
Fe1O6Sb2_ICSD_40344	metal	insulator
Fe1O6Sr2W1_ICSD_78677	metal	insulator
Fe1O6Sr2W1_ICSD_78678	insulator	metal
Fe1O6Ta2_ICSD_201754	insulator	insulator
Fe1O7Rb1Se2_ICSD_90415	insulator	insulator
Fe1O8Rb1Se2_ICSD_75550	insulator	insulator
Fe1P1S1_ICSD_633086	metal	insulator
Fe1P1S3_ICSD_61392	insulator	insulator
Fe1P1Se1_ICSD_633093	metal	insulator
Fe1P1Se3_ICSD_86272	metal	insulator
Fe1P1Ta1_ICSD_633105	metal	metal
Fe1P1Ta4_ICSD_86378	metal	metal
Fe1P1Ti1_ICSD_633111	metal	metal
Fe1P1V1_ICSD_633116	metal	metal
Fe1P1Zr1_ICSD_43204	metal	metal
Fe1P1_ICSD_633046	metal	metal
Fe1P2_ICSD_42904	insulator	metal
Fe1P4Si4_ICSD_79005	insulator	insulator
Fe1P4_ICSD_2413	insulator	insulator
Fe1P4_ICSD_2442	insulator	insulator
Fe1P4_ICSD_65415	insulator	insulator
Fe1Pd1_ICSD_180896	metal	metal
Fe1Pd1_ICSD_633142	metal	metal
Fe1Pd2Se2_ICSD_416515	metal	metal

Supplementary Table 382. Five-fold cross validated predictions for the metal/insulator classification (374/598).

system	calculated	predicted
Fe1Pd3_ICSD_103582	metal	metal
Fe1Pr1Sb2_ICSD_657916	metal	metal
Fe1Pr1Sb3_ICSD_168458	metal	metal
Fe1Pr1Si1_ICSD_633164	metal	metal
Fe1Pr1Si2_ICSD_67087	metal	metal
Fe1Pt1Sb1_ICSD_633196	metal	metal
Fe1Pt1_ICSD_42589	metal	metal
Fe1Pt1_ICSD_633191	metal	metal
Fe1Pt1_ICSD_659004	metal	metal
Fe1Pt3_ICSD_633188	metal	metal
Fe1Rb1S2_ICSD_202384	metal	insulator
Fe1Rb1S2_ICSD_633207	insulator	insulator
Fe1Rb1Se2_ICSD_40781	insulator	metal
Fe1Rh1_ICSD_108465	metal	metal
Fe1Rh2S4_ICSD_633231	metal	metal
Fe1Rh2Se4_ICSD_603354	insulator	metal
Fe1Rh2Sn1_ICSD_633234	metal	metal
Fe1Ru2Si1_ICSD_633245	metal	metal
Fe1Ru2Sn1_ICSD_103615	metal	metal
Fe1S17U8_ICSD_633354	insulator	insulator
Fe1S1Sb1_ICSD_24161	insulator	metal
Fe1S1_ICSD_29302	metal	metal
Fe1S1_ICSD_35006	insulator	metal
Fe1S1_ICSD_43694	insulator	metal
Fe1S1_ICSD_51003	metal	insulator
Fe1S1_ICSD_53528	metal	metal
Fe1S1_ICSD_633265	insulator	metal
Fe1S1_ICSD_68845	insulator	metal
Fe1S1_ICSD_68847	metal	insulator
Fe1S1_ICSD_68848	metal	insulator
Fe1S1_ICSD_87499	metal	insulator
Fe1S1_ICSD_87501	insulator	metal
Fe1S1_ICSD_87502	metal	metal
Fe1S1_ICSD_87503	insulator	metal
Fe1S2Tl1_ICSD_150822	metal	metal
Fe1S2Tl1_ICSD_63381	insulator	metal
Fe1S2_ICSD_109374	insulator	insulator
Fe1S2_ICSD_633289	insulator	insulator
Fe1S4Sb2_ICSD_93911	insulator	insulator
Fe1S4Sc2_ICSD_74405	metal	insulator
Fe1S4Ti2_ICSD_42563	metal	metal
Fe1S4V2_ICSD_35634	insulator	metal
Fe1S4V2_ICSD_633355	metal	insulator
Fe1S4V2_ICSD_633361	metal	insulator
Fe1S4Yb2_ICSD_37419	metal	metal
Fe1S5Th2_ICSD_633325	insulator	insulator
Fe1S5U2_ICSD_41899	insulator	insulator
Fe1S6Ti3_ICSD_53533	insulator	metal
Fe1S8Ti4_ICSD_53534	metal	metal
Fe1Sb1Se1_ICSD_633399	metal	insulator

Supplementary Table 383. Five-fold cross validated predictions for the metal/insulator classification (375/598).

system	calculated	predicted
Fe1Sb1Te1_ICSD_633405	metal	metal
Fe1Sb1Ti1_ICSD_53537	metal	metal
Fe1Sb1V1_ICSD_181132	metal	metal
Fe1Sb1V1_ICSD_53539	insulator	metal
Fe1Sb1Zn1_ICSD_90397	metal	metal
Fe1Sb1_ICSD_53535	metal	metal
Fe1Sb1_ICSD_53971	metal	metal
Fe1Sb1_ICSD_633396	metal	metal
Fe1Sb2Sc6_ICSD_96247	metal	metal
Fe1Sb2Tb6_ICSD_154772	metal	metal
Fe1Sb2Ti5_ICSD_96143	metal	metal
Fe1Sb2Tm6_ICSD_96252	metal	metal
Fe1Sb2Y6_ICSD_96248	metal	metal
Fe1Sb2Zr6_ICSD_156949	metal	metal
Fe1Sb2_ICSD_186635	insulator	metal
Fe1Sb2_ICSD_186640	metal	metal
Fe1Sb2_ICSD_633389	metal	insulator
Fe1Sc1Si1_ICSD_84203	metal	metal
Fe1Sc2Si2_ICSD_633455	metal	metal
Fe1Sc2Si2_ICSD_84205	metal	metal
Fe1Sc6Te2_ICSD_90081	metal	metal
Fe1Se0.875_ICSD_167196	insulator	metal
Fe1Se1_ICSD_163557	metal	metal
Fe1Se1_ICSD_165957	insulator	metal
Fe1Se1_ICSD_169271	metal	metal
Fe1Se1_ICSD_169307	metal	metal
Fe1Se1_ICSD_182545	metal	insulator
Fe1Se1_ICSD_185465	insulator	metal
Fe1Se1_ICSD_53541	metal	metal
Fe1Se1_ICSD_53542	insulator	metal
Fe1Se1_ICSD_633467	metal	metal
Fe1Se1_ICSD_633480	metal	metal
Fe1Se2Tl1_ICSD_100354	metal	insulator
Fe1Se2Tl1_ICSD_633509	metal	metal
Fe1Se2_ICSD_633475	insulator	metal
Fe1Se2_ICSD_633489	insulator	metal
Fe1Se4Ti2_ICSD_633504	insulator	metal
Fe1Se5Th2_ICSD_633494	insulator	insulator
Fe1Se5U2_ICSD_601612	metal	insulator
Fe1Si1Ta1_ICSD_633568	metal	metal
Fe1Si1Tb1_ICSD_57297	metal	metal
Fe1Si1Tb1_ICSD_633578	metal	metal
Fe1Si1Ti1_ICSD_41157	metal	metal
Fe1Si1U1_ICSD_55537	metal	metal
Fe1Si1Y1_ICSD_633643	metal	metal
Fe1Si1Yb1_ICSD_633660	metal	metal
Fe1Si1Zr1_ICSD_633674	metal	metal
Fe1Si1_ICSD_52334	metal	insulator
Fe1Si1_ICSD_81495	insulator	metal
Fe1Si2Tb2_ICSD_99215	metal	metal

Supplementary Table 384. Five-fold cross validated predictions for the metal/insulator classification (376/598).

system	calculated	predicted
Fe1Si2Ti1_ICSD_166099	metal	metal
Fe1Si2Ti1_ICSD_31992	metal	metal
Fe1Si2_ICSD_23408	insulator	metal
Fe1Si2_ICSD_633533	metal	metal
Fe1Sn1Ti1_ICSD_106680	metal	metal
Fe1Sn1_ICSD_150620	metal	metal
Fe1Sn1_ICSD_150691	metal	metal
Fe1Sn1_ICSD_633747	metal	metal
Fe1Sn2_ICSD_633761	metal	metal
Fe1Ta1Te3_ICSD_66513	metal	metal
Fe1Te1_ICSD_180602	metal	metal
Fe1Te1_ICSD_56142	metal	metal
Fe1Te2Zr6_ICSD_82530	metal	metal
Fe1Te2_ICSD_633866	insulator	metal
Fe1Te2_ICSD_633869	metal	metal
Fe1Ti1_ICSD_96139	metal	metal
Fe1Ti2_ICSD_633947	metal	metal
Fe1U6_ICSD_103678	metal	metal
Fe1V1_ICSD_103681	metal	metal
Fe1V3_ICSD_634028	metal	metal
Fe1Zn13_ICSD_240050	metal	metal
Fe1Zr2_ICSD_102804	metal	metal
Fe1Zr2_ICSD_103713	metal	metal
Fe1Zr2_ICSD_634145	metal	metal
Fe1Zr3_ICSD_634167	metal	metal
Fe1_ICSD_165727	metal	metal
Fe1_ICSD_171002	metal	metal
Fe1_ICSD_185725	metal	metal
Fe1_ICSD_185737	metal	metal
Fe1_ICSD_44863	metal	metal
Fe1_ICSD_631723	metal	metal
Fe1_ICSD_631727	metal	metal
Fe1_ICSD_631730	metal	metal
Fe23Ho6_ICSD_103503	metal	metal
Fe23Lu6_ICSD_632454	metal	metal
Fe23Tb6_ICSD_633832	metal	metal
Fe23Y6_ICSD_634086	metal	metal
Fe23Yb6_ICSD_634132	metal	metal
Fe23Zr6_ICSD_634176	metal	metal
Fe2Ga1Mn1_ICSD_186721	metal	metal
Fe2Ga1Mn1_ICSD_186722	metal	metal
Fe2Ga1Ni1_ICSD_103460	metal	metal
Fe2Ga1Ti1_ICSD_103469	metal	metal
Fe2Ga1V1_ICSD_631850	insulator	metal
Fe2Ga2S5_ICSD_49912	metal	insulator
Fe2Ga2S5_ICSD_631804	insulator	metal
Fe2Gd1Ge2_ICSD_106825	metal	metal
Fe2Gd1_ICSD_103477	metal	metal
Fe2Gd1_ICSD_631879	metal	metal
Fe2Gd2I1_ICSD_73157	metal	metal

Supplementary Table 385. Five-fold cross validated predictions for the metal/insulator classification (377/598).

system	calculated	predicted
Fe2Ge1Mn1_ICSD_186065	metal	metal
Fe2Ge1Mo3N1_ICSD_185929	metal	metal
Fe2Ge1O4_ICSD_93973	insulator	metal
Fe2Ge1S4_ICSD_23526	insulator	insulator
Fe2Ge1Se4_ICSD_87086	insulator	insulator
Fe2Ge1Ti1_ICSD_186057	insulator	metal
Fe2Ge1_ICSD_108442	metal	metal
Fe2Ge1_ICSD_632005	metal	metal
Fe2Ge1_ICSD_632028	metal	metal
Fe2Ge2Ho1_ICSD_164212	metal	metal
Fe2Ge2La1_ICSD_81747	metal	metal
Fe2Ge2Nd1_ICSD_53467	metal	metal
Fe2Ge2Pr1_ICSD_53472	metal	metal
Fe2Ge2Tb1_ICSD_632133	metal	metal
Fe2Ge2Th1_ICSD_55341	metal	metal
Fe2Ge2Tm1_ICSD_106829	metal	metal
Fe2Ge2Y1_ICSD_81745	metal	metal
Fe2Ge2Yb1_ICSD_53482	metal	metal
Fe2Ge2Yb1_ICSD_632164	metal	metal
Fe2Ge3Mn3O12_ICSD_18111	insulator	insulator
Fe2H1Na1O8P2_ICSD_171329	insulator	insulator
Fe2H1O9Sb1Si2_ICSD_200068	insulator	insulator
Fe2H2O1Zr4_ICSD_108768	metal	metal
Fe2Hf1Si2_ICSD_20933	metal	metal
Fe2Hf1_ICSD_632242	metal	metal
Fe2Hf1_ICSD_632247	metal	metal
Fe2Hf1_ICSD_632249	metal	metal
Fe2Hf1_ICSD_632252	metal	metal
Fe2Hf3Si3_ICSD_632265	metal	metal
Fe2Ho1Si2_ICSD_632346	metal	metal
Fe2Ho1_ICSD_632290	metal	metal
Fe2Ho1_ICSD_632293	metal	metal
Fe2I1La2_ICSD_409622	metal	metal
Fe2In1O4_ICSD_157323	metal	metal
Fe2In1Ti1_ICSD_186056	metal	metal
Fe2K1S2_ICSD_186572	insulator	metal
Fe2K1S3_ICSD_100180	metal	insulator
Fe2K1S3_ICSD_99507	insulator	metal
Fe2K1Se2_ICSD_186573	metal	metal
Fe2K1Se3_ICSD_424672	metal	metal
Fe2K1Te2_ICSD_186574	metal	metal
Fe2K2Na4O6_ICSD_36612	insulator	insulator
Fe2K2Nb4O13_ICSD_50038	insulator	insulator
Fe2K3Na1O8_ICSD_159909	insulator	insulator
Fe2K3O4_ICSD_94465	metal	insulator
Fe2K4O5_ICSD_154372	insulator	insulator
Fe2K6O5_ICSD_174312	insulator	insulator
Fe2K6O6_ICSD_6149	insulator	insulator
Fe2K6S6_ICSD_425068	insulator	insulator
Fe2K9O8_ICSD_174307	metal	insulator

Supplementary Table 386. Five-fold cross validated predictions for the metal/insulator classification (378/598).

system	calculated	predicted
Fe2La1P2_ICSD_632411	metal	metal
Fe2La1Si2_ICSD_53491	metal	metal
Fe2La2O3Se2_ICSD_183143	metal	insulator
Fe2La2S5_ICSD_1478	insulator	insulator
Fe2La3S7_ICSD_603953	insulator	insulator
Fe2Li1N3Sr2_ICSD_71059	metal	metal
Fe2Li2O4_ICSD_31149	insulator	metal
Fe2Lu1Si2_ICSD_632475	metal	metal
Fe2Lu1_ICSD_103515	metal	metal
Fe2Mg1O4_ICSD_157694	insulator	insulator
Fe2Mg1O4_ICSD_24493	insulator	metal
Fe2Mg1O4_ICSD_94080	insulator	insulator
Fe2Mg3O12Si3_ICSD_27373	insulator	insulator
Fe2Mn1O4_ICSD_170910	insulator	insulator
Fe2Mn1O4_ICSD_24497	insulator	insulator
Fe2Mn1Si1_ICSD_659018	metal	metal
Fe2Mn3O12Si3_ICSD_77433	metal	insulator
Fe2Mo1_ICSD_632621	metal	metal
Fe2Mo1_ICSD_632626	metal	metal
Fe2Mo3Na1O12_ICSD_281398	metal	insulator
Fe2Mo3Na3O12_ICSD_281400	insulator	insulator
Fe2Mo3O8_ICSD_61069	insulator	insulator
Fe2Mo4N1_ICSD_162275	metal	metal
Fe2N1Nb4_ICSD_632693	metal	metal
Fe2N1Ta4_ICSD_632740	metal	metal
Fe2N1Zr4_ICSD_632768	metal	metal
Fe2N1_ICSD_150889	metal	metal
Fe2N1_ICSD_152811	metal	metal
Fe2N1_ICSD_33575	metal	metal
Fe2N1_ICSD_44612	metal	metal
Fe2Na1O3_ICSD_200009	metal	metal
Fe2Na1O3_ICSD_424349	metal	insulator
Fe2Na2O1Se2_ICSD_186502	insulator	insulator
Fe2Nb1_ICSD_188274	metal	metal
Fe2Nb1_ICSD_632773	metal	metal
Fe2Nb1_ICSD_632791	metal	metal
Fe2Nd1Si2_ICSD_169532	metal	metal
Fe2Nd1Si2_ICSD_632872	metal	metal
Fe2Nd1Si2_ICSD_632874	metal	metal
Fe2Nd1_ICSD_103548	metal	metal
Fe2Nd1_ICSD_632845	metal	metal
Fe2Ni1O4_ICSD_158834	metal	metal
Fe2Ni1O4_ICSD_182237	metal	metal
Fe2Ni1O4_ICSD_52387	metal	metal
Fe2Ni1P1_ICSD_153485	metal	metal
Fe2Ni1_ICSD_188275	metal	metal
Fe2O12P4_ICSD_33740	insulator	insulator
Fe2O12S3_ICSD_21018	insulator	insulator
Fe2O12Se4Sr1_ICSD_81569	insulator	insulator
Fe2O14P4Pb1_ICSD_83648	insulator	insulator

Supplementary Table 387. Five-fold cross validated predictions for the metal/insulator classification (379/598).

system	calculated	predicted
Fe2O14P4Sr1_ICSD_79719	insulator	insulator
Fe2O1Ti4_ICSD_100839	metal	metal
Fe2O3Pr2S2_ICSD_181168	metal	metal
Fe2O3Pr2Se2_ICSD_183145	metal	metal
Fe2O3_ICSD_108905	metal	metal
Fe2O3_ICSD_15840	metal	insulator
Fe2O3_ICSD_161287	insulator	insulator
Fe2O3_ICSD_189439	insulator	insulator
Fe2O3_ICSD_96076	insulator	insulator
Fe2O3_ICSD_96077	metal	insulator
Fe2O4Si1_ICSD_158572	insulator	insulator
Fe2O4Si1_ICSD_169083	insulator	insulator
Fe2O4Si1_ICSD_185523	insulator	insulator
Fe2O4Si1_ICSD_87462	insulator	insulator
Fe2O4Ti1_ICSD_187588	metal	metal
Fe2O4Y1_ICSD_67701	metal	metal
Fe2O4Yb1_ICSD_4192	metal	metal
Fe2O4Zn1_ICSD_76981	metal	metal
Fe2O4Zn1_ICSD_91935	metal	metal
Fe2O5P1_ICSD_32641	insulator	insulator
Fe2O5P1_ICSD_80554	metal	insulator
Fe2O5Rb4_ICSD_154291	insulator	insulator
Fe2O5Te1_ICSD_8282	insulator	insulator
Fe2O5Ti1_ICSD_24416	insulator	insulator
Fe2O5Ti1_ICSD_36183	insulator	insulator
Fe2O6P2Sc2Sr4_ICSD_168586	metal	metal
Fe2O6Rb6_ICSD_154371	insulator	insulator
Fe2O6Te1_ICSD_24795	insulator	insulator
Fe2O6W1_ICSD_1147	insulator	insulator
Fe2O7P2_ICSD_17062	insulator	insulator
Fe2O7P2_ICSD_240243	insulator	insulator
Fe2O7P2_ICSD_36208	insulator	insulator
Fe2O7Sr1Tb2_ICSD_19038	insulator	insulator
Fe2O7Sr3_ICSD_2648	metal	metal
Fe2P1_ICSD_246858	metal	metal
Fe2P1_ICSD_603609	metal	metal
Fe2P2S6_ICSD_657319	insulator	insulator
Fe2P2Sr1_ICSD_10467	metal	metal
Fe2P2U1_ICSD_633115	metal	metal
Fe2Pr1Si2_ICSD_106663	metal	metal
Fe2Pr1Si2_ICSD_53521	metal	metal
Fe2Pr1_ICSD_103590	metal	metal
Fe2Pr1_ICSD_103591	metal	metal
Fe2Pr1_ICSD_633150	metal	metal
Fe2Pu1Si2_ICSD_602683	metal	metal
Fe2Pu1_ICSD_633202	metal	metal
Fe2Rb1S3_ICSD_99506	insulator	insulator
Fe2Rb1Se3_ICSD_81547	metal	metal
Fe2Rb1Te3_ICSD_81548	metal	metal
Fe2Rb6S6_ICSD_425088	insulator	insulator

Supplementary Table 388. Five-fold cross validated predictions for the metal/insulator classification (380/598).

system	calculated	predicted
Fe2Re3_ICSD_108565	metal	metal
Fe2S2Tl1_ICSD_633338	metal	metal
Fe2S3Tl1_ICSD_166049	metal	metal
Fe2S4Si1_ICSD_88517	insulator	insulator
Fe2S6Ta9_ICSD_63592	metal	metal
Fe2Sb1Ti1_ICSD_186060	metal	metal
Fe2Sc1Si2_ICSD_87157	metal	metal
Fe2Sc1_ICSD_633414	metal	metal
Fe2Sc1_ICSD_633417	metal	metal
Fe2Sc1_ICSD_633420	metal	metal
Fe2Sc1_ICSD_633423	metal	metal
Fe2Sc1_ICSD_633427	metal	metal
Fe2Sc1_ICSD_633428	metal	metal
Fe2Sc3Si3_ICSD_76347	metal	metal
Fe2Se2Tl1_ICSD_633510	metal	metal
Fe2Se4V1_ICSD_633517	metal	metal
Fe2Se8Ta11_ICSD_40377	metal	metal
Fe2Si1V1_ICSD_53555	metal	metal
Fe2Si1_ICSD_100094	metal	metal
Fe2Si2Tb1_ICSD_55801	metal	metal
Fe2Si2Tb1_ICSD_633572	metal	metal
Fe2Si2Th1_ICSD_68209	metal	metal
Fe2Si2U1_ICSD_53552	metal	metal
Fe2Si2U1_ICSD_633606	metal	metal
Fe2Si2U1_ICSD_633617	metal	metal
Fe2Si2Y1_ICSD_53556	metal	metal
Fe2Si2Yb1_ICSD_633658	metal	metal
Fe2Si2Zr1_ICSD_633666	metal	metal
Fe2Si3Zr3_ICSD_633671	metal	metal
Fe2Sm1_ICSD_633697	metal	metal
Fe2Sn1Ti1_ICSD_103642	metal	insulator
Fe2Sn1Ti1_ICSD_186058	insulator	metal
Fe2Sn1U2_ICSD_106681	metal	metal
Fe2Sn1V1_ICSD_103644	metal	metal
Fe2Sn1_ICSD_103637	metal	metal
Fe2Ta1_ICSD_633780	metal	metal
Fe2Ta1_ICSD_633784	metal	metal
Fe2Ta1_ICSD_633796	metal	metal
Fe2Tb1Zn20_ICSD_152109	metal	metal
Fe2Tb1_ICSD_2351	metal	metal
Fe2Tb1_ICSD_633817	metal	metal
Fe2Tb1_ICSD_633821	metal	metal
Fe2Ti1_ICSD_103663	metal	metal
Fe2Ti1_ICSD_657043	metal	metal
Fe2U1_ICSD_103680	metal	metal
Fe2U1_ICSD_633998	metal	metal
Fe2U1_ICSD_634006	metal	metal
Fe2U1_ICSD_634010	metal	metal
Fe2W1_ICSD_634067	metal	metal
Fe2Y1_ICSD_103698	metal	metal

Supplementary Table 389. Five-fold cross validated predictions for the metal/insulator classification (381/598).

system	calculated	predicted
Fe2Yb1_ICSD_103703	metal	metal
Fe2Yb1_ICSD_634128	metal	metal
Fe2Zr1_ICSD_634155	metal	metal
Fe2Zr1_ICSD_634160	metal	metal
Fe32Ho12O2_ICSD_9827	metal	metal
Fe32O2Y12_ICSD_9640	metal	metal
Fe3Ga1_ICSD_108436	metal	metal
Fe3Ga1_ICSD_631740	metal	metal
Fe3Ga1_ICSD_631761	metal	metal
Fe3Ge1_ICSD_53462	metal	metal
Fe3Ge1_ICSD_631986	metal	metal
Fe3Ge1_ICSD_632004	metal	metal
Fe3Ge1_ICSD_632027	metal	metal
Fe3H28O30S4_ICSD_15207	insulator	insulator
Fe3H6K1O14S2_ICSD_189302	insulator	insulator
Fe3H6Na1O14S2_ICSD_160409	insulator	insulator
Fe3H6O14P1Pb1S1_ICSD_68352	insulator	insulator
Fe3Ho2Si5_ICSD_632350	metal	metal
Fe3Mn2O12Si3_ICSD_27378	metal	insulator
Fe3Mo3N1_ICSD_632633	metal	metal
Fe3N1Nb3_ICSD_632692	metal	metal
Fe3N1Ni1_ICSD_44865	metal	metal
Fe3N1Ni1_ICSD_632709	metal	metal
Fe3N1Pd1_ICSD_53506	metal	metal
Fe3N1Pt1_ICSD_632719	metal	metal
Fe3N1Rh1_ICSD_153274	metal	metal
Fe3N1W3_ICSD_59255	metal	metal
Fe3N1_ICSD_260758	metal	metal
Fe3N1_ICSD_33576	metal	metal
Fe3N1_ICSD_79982	metal	metal
Fe3N1_ICSD_79983	metal	metal
Fe3N1_ICSD_79984	metal	metal
Fe3N8Sr8_ICSD_409949	metal	insulator
Fe3Nb1_ICSD_188244	metal	metal
Fe3Ni1_ICSD_188233	metal	metal
Fe3Ni1_ICSD_188245	metal	metal
Fe3Ni1_ICSD_188257	metal	metal
Fe3O12Si3V2_ICSD_27376	insulator	insulator
Fe3O1Ti3_ICSD_29053	metal	metal
Fe3O4_ICSD_159976	metal	metal
Fe3O4_ICSD_263010	insulator	insulator
Fe3O4_ICSD_31156	metal	metal
Fe3O4_ICSD_35002	insulator	metal
Fe3O4_ICSD_65338	insulator	metal
Fe3O4_ICSD_84611	metal	insulator
Fe3O4_ICSD_87697	metal	metal
Fe3O4_ICSD_92356	metal	metal
Fe3O4_ICSD_98086	metal	insulator
Fe3O6Rb5_ICSD_174319	insulator	insulator
Fe3O7P1_ICSD_36207	insulator	insulator

Supplementary Table 390. Five-fold cross validated predictions for the metal/insulator classification (382/598).

system	calculated	predicted
Fe3O7Yb2_ICSD_8293	metal	metal
Fe3O8P2_ICSD_56293	insulator	insulator
Fe3P1_ICSD_633057	metal	metal
Fe3Pd1_ICSD_103586	metal	metal
Fe3Pt1_ICSD_633190	metal	metal
Fe3Pt1_ICSD_99787	metal	metal
Fe3S1_ICSD_633291	metal	metal
Fe3S4Tl2_ICSD_41430	metal	metal
Fe3S4_ICSD_42537	metal	metal
Fe3S4_ICSD_44885	metal	metal
Fe3S4_ICSD_633292	metal	metal
Fe3Sc2Si5_ICSD_633453	metal	metal
Fe3Se4_ICSD_150568	metal	metal
Fe3Si1Zr2_ICSD_53558	metal	metal
Fe3Si1_ICSD_633531	metal	metal
Fe3Si5Tb2_ICSD_633581	metal	metal
Fe3Si5U2_ICSD_633609	metal	metal
Fe3Si5Y2_ICSD_633652	metal	metal
Fe3Si5Yb2_ICSD_633661	metal	metal
Fe3Sn1_ICSD_103638	metal	metal
Fe3Sn1_ICSD_24569	metal	metal
Fe3Sn1_ICSD_633757	metal	metal
Fe3Sn2_ICSD_71	metal	metal
Fe3Tb1_ICSD_103650	metal	metal
Fe3Tb1_ICSD_164279	metal	metal
Fe3Tb1_ICSD_633844	metal	metal
Fe3Te3Tl1_ICSD_633881	metal	metal
Fe3Th1_ICSD_633920	metal	metal
Fe3Th7_ICSD_633908	metal	metal
Fe3Y1_ICSD_634092	metal	metal
Fe4Ge2Yb1_ICSD_39898	metal	metal
Fe4La1P12_ICSD_168583	metal	metal
Fe4La1Sb12_ICSD_53490	metal	metal
Fe4Mn8Y1_ICSD_632605	metal	metal
Fe4N1_ICSD_60195	metal	metal
Fe4Na1Sb12_ICSD_246577	metal	metal
Fe4Nd1P12_ICSD_632859	metal	metal
Fe4Nd1Sb12_ICSD_79927	metal	metal
Fe4O11Sr4_ICSD_249009	metal	insulator
Fe4O5_ICSD_185515	insulator	insulator
Fe4P12Pr1_ICSD_93363	metal	metal
Fe4P12Sm1_ICSD_633097	metal	metal
Fe4P12Tb1_ICSD_245293	metal	metal
Fe4P12Th1_ICSD_200827	insulator	metal
Fe4P12U1_ICSD_89093	metal	metal
Fe4P12Yb1_ICSD_156464	metal	metal
Fe4P1_ICSD_43551	metal	metal
Fe4P2Sc1_ICSD_68525	metal	metal
Fe4P2Zr1_ICSD_633122	metal	metal
Fe4Rb8S10_ICSD_425071	insulator	insulator

Supplementary Table 391. Five-fold cross validated predictions for the metal/insulator classification (383/598).

system	calculated	predicted
Fe4Sb12Sr1_ICSD_658733	metal	metal
Fe4Sc1Si2_ICSD_633444	metal	metal
Fe4Si2Tm1_ICSD_261030	metal	metal
Fe4Si2Y1_ICSD_186048	metal	metal
Fe4Si2Zr1_ICSD_87172	metal	metal
Fe4Si7Zr4_ICSD_633672	metal	metal
Fe4Sn4Zr3_ICSD_168652	metal	metal
Fe5Gd1_ICSD_103480	metal	metal
Fe5H1O8_ICSD_166135	insulator	insulator
Fe5H2La1_ICSD_160916	metal	metal
Fe5H7La1_ICSD_160915	metal	metal
Fe5H7La1_ICSD_160917	metal	metal
Fe5Ho1_ICSD_103502	metal	metal
Fe5Ho1_ICSD_632281	metal	metal
Fe5Li1O8_ICSD_35769	metal	insulator
Fe5Nd1_ICSD_103549	metal	metal
Fe5Nd3O12_ICSD_260556	insulator	insulator
Fe5O12Pr3_ICSD_422690	insulator	insulator
Fe5O12Si3_ICSD_27377	insulator	insulator
Fe5O12Tb3_ICSD_22320	insulator	insulator
Fe5O12Y3_ICSD_88504	insulator	insulator
Fe5O12Yb3_ICSD_23854	metal	metal
Fe5Si3U1_ICSD_157999	metal	metal
Fe5Si3_ICSD_161130	metal	metal
Fe5Si3_ICSD_161132	metal	metal
Fe5Si3_ICSD_99967	metal	metal
Fe5Sm1_ICSD_103629	metal	metal
Fe5Th1_ICSD_103656	metal	metal
Fe5Th1_ICSD_150500	metal	metal
Fe5Th1_ICSD_633902	metal	metal
Fe5Y1_ICSD_184905	metal	metal
Fe6Ga6Ho1_ICSD_180129	metal	metal
Fe6Ga6Sc1_ICSD_103465	metal	metal
Fe6Ge4Li1_ICSD_41381	metal	metal
Fe6Ge5_ICSD_2145	metal	metal
Fe6Ge6Hf1_ICSD_85903	metal	metal
Fe6Ge6Ho1_ICSD_656772	metal	metal
Fe6Ge6Lu1_ICSD_84189	metal	metal
Fe6Ge6Mg1_ICSD_188716	metal	metal
Fe6Ge6Nb1_ICSD_92177	metal	metal
Fe6Ge6Sc1_ICSD_632112	metal	metal
Fe6Ge6Sc1_ICSD_92170	metal	metal
Fe6Ge6Tb1_ICSD_86194	metal	metal
Fe6Ge6Zr1_ICSD_92175	metal	metal
Fe6Sc1Sn6_ICSD_633457	metal	metal
Fe6Sn6Tb1_ICSD_106583	metal	metal
Fe6Ta7_ICSD_633795	metal	metal
Fe7Mo6_ICSD_632622	metal	metal
Fe7Nb6_ICSD_188281	metal	metal
Fe7O10Si1_ICSD_26171	metal	insulator

Supplementary Table 392. Five-fold cross validated predictions for the metal/insulator classification (384/598).

system	calculated	predicted
Fe7S8_ICSD_8064	metal	insulator
Fe7Se8_ICSD_16882	metal	metal
Fe7Ta6_ICSD_633791	metal	metal
Fe7Th2_ICSD_103657	metal	metal
Fe7W6_ICSD_103694	metal	metal
Fe8Rb24S24_ICSD_425089	insulator	insulator
Fe8Tb1V4_ICSD_633855	metal	metal
Fe9La1Si4_ICSD_79590	metal	metal
Ga0.4N0.2V0.6_ICSD_634722	metal	metal
Ga0.5N0.5O0.5Zn0.5_ICSD_160638	insulator	insulator
Ga10Ni1Pr3_ICSD_20663	metal	metal
Ga12Gd4Pd1_ICSD_634239	metal	metal
Ga12Ho4Ni1_ICSD_634391	metal	metal
Ga12Ho4Pd1_ICSD_634396	metal	metal
Ga12La2Pd1_ICSD_171486	metal	metal
Ga12Lu4Pd1_ICSD_634587	metal	metal
Ga12Ni1Tm4_ICSD_634969	metal	metal
Ga12Pd1Tb4_ICSD_635088	metal	metal
Ga12Pd1Tm4_ICSD_635093	metal	metal
Ga12Pd1U4_ICSD_156309	metal	metal
Ga12Pd1Y4_ICSD_635099	metal	metal
Ga12Pt1Tb4_ICSD_153211	metal	metal
Ga12Pt1Y4_ICSD_153909	metal	metal
Ga12Rh1U4_ICSD_156308	metal	metal
Ga16Hf6Rh7_ICSD_634325	metal	metal
Ga16Hf6Ru7_ICSD_634327	metal	metal
Ga16Ir7Sc6_ICSD_634454	metal	metal
Ga16Ir7Ti6_ICSD_634458	metal	metal
Ga16Os7Sc6_ICSD_635026	metal	metal
Ga16Pt7Ti6_ICSD_635156	metal	metal
Ga16Rh7Sc6_ICSD_635212	metal	metal
Ga16Rh7Ti6_ICSD_635215	metal	metal
Ga16Rh7Zr6_ICSD_635224	metal	metal
Ga16Ru7Zr6_ICSD_635240	metal	metal
Ga1Gd1Mg1_ICSD_54616	metal	metal
Ga1Gd1Zn1_ICSD_634245	metal	metal
Ga1Gd1_ICSD_109240	metal	metal
Ga1Gd3O6_ICSD_420733	insulator	insulator
Ga1Ge1H1Sr1_ICSD_173570	insulator	insulator
Ga1Ge1Li1_ICSD_25310	metal	metal
Ga1Ge1Li3O5_ICSD_72101	insulator	insulator
Ga1Ge1Mn1_ICSD_634253	metal	metal
Ga1Ge1Mn1_ICSD_634255	metal	metal
Ga1Ge1N1Sr2_ICSD_420413	insulator	metal
Ga1Ge1Sr1_ICSD_634281	metal	metal
Ga1Ge1Te1_ICSD_35386	metal	insulator
Ga1Ge1Yb1_ICSD_152569	metal	metal
Ga1Ge2Na5_ICSD_169702	metal	metal
Ga1H1O2_ICSD_409671	insulator	insulator
Ga1H1O4Se1_ICSD_422882	insulator	insulator

Supplementary Table 393. Five-fold cross validated predictions for the metal/insulator classification (385/598).

system	calculated	predicted
Ga1H1Si1Sr1_ICSD_173569	insulator	insulator
Ga1H1Sn1Sr1_ICSD_173571	insulator	insulator
Ga1H2Na3O9P2_ICSD_84644	insulator	insulator
Ga1H4K1_ICSD_169709	insulator	insulator
Ga1H4Li1_ICSD_169706	insulator	insulator
Ga1H4Li1_ICSD_169707	insulator	insulator
Ga1H4Mg1_ICSD_240696	metal	insulator
Ga1H4Na1_ICSD_67309	insulator	insulator
Ga1H4Rb1_ICSD_169710	insulator	insulator
Ga1H4Sr1_ICSD_240697	metal	insulator
Ga1H5Sr1_ICSD_240697	insulator	insulator
Ga1Hf1Ni1_ICSD_634315	metal	metal
Ga1Hf1Ni2_ICSD_103734	metal	metal
Ga1Hf1Pd1_ICSD_156269	metal	metal
Ga1Hf1Pt1_ICSD_156268	metal	metal
Ga1Hf1Rh1_ICSD_634326	metal	metal
Ga1Hf1_ICSD_103726	metal	metal
Ga1Hf2Sb3_ICSD_189076	metal	metal
Ga1Hf2_ICSD_102809	metal	metal
Ga1Ho1Mn1_ICSD_106705	metal	metal
Ga1Ho1Ni1_ICSD_103751	metal	metal
Ga1Ho1O3_ICSD_420711	insulator	insulator
Ga1Ho1Pd1_ICSD_54590	metal	metal
Ga1Ho1Pt1_ICSD_634398	metal	metal
Ga1Ho1_ICSD_634376	metal	metal
Ga1Ho3O6_ICSD_99496	insulator	insulator
Ga1I3La3_ICSD_409561	metal	metal
Ga1I3Li1_ICSD_202642	insulator	insulator
Ga1I3O9_ICSD_171008	insulator	insulator
Ga1I3_ICSD_413457	insulator	insulator
Ga1I4K1_ICSD_400816	insulator	insulator
Ga1I4Tl1_ICSD_419824	insulator	insulator
Ga1I7Te1_ICSD_407982	insulator	insulator
Ga1In1O3_ICSD_30339	insulator	insulator
Ga1In1O5Zn2_ICSD_380305	insulator	insulator
Ga1In1O7Zn4_ICSD_247275	insulator	insulator
Ga1In1S3_ICSD_62929	insulator	insulator
Ga1In1Se2_ICSD_601131	metal	insulator
Ga1In1Se3_ICSD_62930	insulator	insulator
Ga1In1Te2_ICSD_60083	metal	insulator
Ga1Ir1Li2_ICSD_106708	metal	metal
Ga1Ir1Li2_ICSD_107085	metal	metal
Ga1Ir1Th1_ICSD_634457	metal	metal
Ga1Ir1U1_ICSD_634461	metal	metal
Ga1Ir1Y1_ICSD_634463	metal	metal
Ga1Ir1_ICSD_103760	metal	metal
Ga1K1O14Te6_ICSD_280793	insulator	insulator
Ga1K1S2_ICSD_107928	insulator	insulator
Ga1K1Sb4_ICSD_300158	insulator	insulator
Ga1K2Li3O4_ICSD_35290	insulator	insulator

Supplementary Table 394. Five-fold cross validated predictions for the metal/insulator classification (386/598).

system	calculated	predicted
Ga1K2Na1P2_ICSD_300112	insulator	insulator
Ga1K6Na3Sb4_ICSD_77274	insulator	insulator
Ga1La1Mg1_ICSD_413276	metal	metal
Ga1La1Ni1_ICSD_62281	metal	metal
Ga1La1O1Se2_ICSD_48024	metal	insulator
Ga1La1O3_ICSD_161782	insulator	insulator
Ga1La1O3_ICSD_51039	insulator	insulator
Ga1La1O3_ICSD_76047	metal	metal
Ga1La1O3_ICSD_83349	insulator	insulator
Ga1La1Pd2_ICSD_656959	metal	metal
Ga1La1Pt1_ICSD_634512	metal	metal
Ga1La1Zn1_ICSD_634520	metal	metal
Ga1La1_ICSD_634484	metal	metal
Ga1La2N3_ICSD_160089	insulator	insulator
Ga1La3Mn1S7_ICSD_634493	insulator	insulator
Ga1La3_ICSD_603198	metal	metal
Ga1Li1Mg2_ICSD_103782	metal	metal
Ga1Li1Mo2O8_ICSD_241135	insulator	insulator
Ga1Li1O2_ICSD_18152	insulator	insulator
Ga1Li1O2_ICSD_28388	insulator	insulator
Ga1Li1O6Si2_ICSD_80108	insulator	insulator
Ga1Li1O6Si2_ICSD_80924	insulator	insulator
Ga1Li1O8W2_ICSD_28008	insulator	insulator
Ga1Li1S2_ICSD_96914	insulator	insulator
Ga1Li1Se2_ICSD_634546	insulator	insulator
Ga1Li1Si1_ICSD_103786	insulator	metal
Ga1Li1Te2_ICSD_162555	insulator	insulator
Ga1Li1_ICSD_634528	metal	metal
Ga1Li2Pd1_ICSD_634537	metal	metal
Ga1Li2Pt1_ICSD_106713	metal	metal
Ga1Li2Rh1_ICSD_106714	metal	metal
Ga1Li2_ICSD_659761	metal	metal
Ga1Li3N2_ICSD_419137	insulator	insulator
Ga1Li3Na2O4_ICSD_37071	insulator	insulator
Ga1Li3O5Si1_ICSD_72100	insulator	insulator
Ga1Li5O4_ICSD_9082	insulator	insulator
Ga1Lu1Ni1_ICSD_634564	metal	metal
Ga1Lu1Pd1_ICSD_634586	metal	metal
Ga1Lu1_ICSD_634550	metal	metal
Ga1Mg1Mo4O7_ICSD_93228	metal	metal
Ga1Mg1Pr1_ICSD_413285	metal	metal
Ga1Mg1Sm1_ICSD_413286	metal	metal
Ga1Mg1Tm1_ICSD_413278	metal	metal
Ga1Mg1Y1_ICSD_160909	metal	metal
Ga1Mg1_ICSD_103789	metal	metal
Ga1Mg2_ICSD_103790	metal	metal
Ga1Mn1Ni2_ICSD_167566	metal	metal
Ga1Mn1Ni2_ICSD_187493	metal	metal
Ga1Mn1Ni2_ICSD_634654	metal	metal
Ga1Mn1Pt1_ICSD_103807	metal	metal

Supplementary Table 395. Five-fold cross validated predictions for the metal/insulator classification (387/598).

system	calculated	predicted
Ga1Mn1Pt1_ICSD_108482	metal	metal
Ga1Mn1Ti2_ICSD_189697	insulator	metal
Ga1Mn1_ICSD_634628	metal	metal
Ga1Mn2V1_ICSD_103813	metal	metal
Ga1Mn3N1_ICSD_87399	metal	metal
Ga1Mn3_ICSD_603351	metal	metal
Ga1Mn3_ICSD_634607	metal	metal
Ga1Mo3_ICSD_634697	metal	metal
Ga1Mo4S8Si1_ICSD_53580	metal	metal
Ga1Mo4S8_ICSD_106732	insulator	metal
Ga1Mo4S8_ICSD_634707	metal	metal
Ga1Mo4Se4Te4_ICSD_49568	metal	insulator
Ga1Mo4Se8_ICSD_36563	insulator	metal
Ga1Mo4Se8_ICSD_634710	insulator	metal
Ga1N1Nd3_ICSD_103821	metal	metal
Ga1N1V3_ICSD_634723	metal	metal
Ga1N1_ICSD_159250	insulator	insulator
Ga1N1_ICSD_181358	insulator	insulator
Ga1N1_ICSD_185155	insulator	insulator
Ga1N1_ICSD_41500	insulator	insulator
Ga1N3Sr3_ICSD_281259	insulator	insulator
Ga1N5O14_ICSD_280268	insulator	insulator
Ga1N5Sr6_ICSD_281260	insulator	insulator
Ga1Na1O2_ICSD_36652	insulator	insulator
Ga1Na1O6Si2_ICSD_156699	insulator	insulator
Ga1Na1Te2_ICSD_44702	insulator	insulator
Ga1Na3P4Sr3_ICSD_402285	insulator	insulator
Ga1Nb1Ni2_ICSD_103839	metal	metal
Ga1Nb1O4_ICSD_18187	insulator	insulator
Ga1Nb3_ICSD_108484	metal	metal
Ga1Nb3_ICSD_634746	metal	metal
Ga1Nb3_ICSD_634748	metal	metal
Ga1Nb4S8_ICSD_158312	metal	metal
Ga1Nb4Se8_ICSD_84196	metal	metal
Ga1Nb5Sn2_ICSD_103842	metal	metal
Ga1Nd1O3_ICSD_24856	insulator	insulator
Ga1Nd1O3_ICSD_76050	metal	metal
Ga1Nd1_ICSD_634805	metal	metal
Ga1Nd3O6_ICSD_99490	insulator	insulator
Ga1Nd3_ICSD_634799	metal	metal
Ga1Ni1Pu1_ICSD_634900	metal	metal
Ga1Ni1Tb1_ICSD_634939	metal	metal
Ga1Ni1Th1_ICSD_634951	metal	metal
Ga1Ni1Ti1_ICSD_103885	metal	metal
Ga1Ni1Ti2_ICSD_185666	metal	metal
Ga1Ni1U1_ICSD_600394	metal	metal
Ga1Ni1Y1_ICSD_634986	metal	metal
Ga1Ni1Zr1_ICSD_635009	metal	metal
Ga1Ni1_ICSD_103853	metal	metal
Ga1Ni2Sc1_ICSD_103874	metal	metal

Supplementary Table 396. Five-fold cross validated predictions for the metal/insulator classification (388/598).

system	calculated	predicted
Ga1Ni2Ta1_ICSD_103881	metal	metal
Ga1Ni2Ti1_ICSD_103886	metal	metal
Ga1Ni2U1_ICSD_603100	metal	metal
Ga1Ni2V1_ICSD_103892	metal	metal
Ga1Ni2Y2_ICSD_634983	metal	metal
Ga1Ni2Zr1_ICSD_103902	metal	metal
Ga1Ni2_ICSD_634869	metal	metal
Ga1Ni3_ICSD_103856	metal	metal
Ga1O2Tl1_ICSD_33579	insulator	insulator
Ga1O3Pr1_ICSD_76049	insulator	metal
Ga1O3Y1_ICSD_1999	insulator	insulator
Ga1O4P1_ICSD_155446	insulator	insulator
Ga1O4P1_ICSD_30883	insulator	insulator
Ga1O4Rb5_ICSD_420328	insulator	insulator
Ga1O6Sb1Sr2_ICSD_157014	insulator	insulator
Ga1O6Sb1Sr2_ICSD_157017	insulator	insulator
Ga1O6Tb3_ICSD_99494	insulator	insulator
Ga1O6Y3_ICSD_155086	insulator	insulator
Ga1P1Pd5_ICSD_635043	metal	metal
Ga1P1Pt5_ICSD_635044	metal	metal
Ga1P1S4_ICSD_2613	insulator	insulator
Ga1P1_ICSD_40868	metal	insulator
Ga1P1_ICSD_635031	metal	insulator
Ga1P1_ICSD_67772	insulator	insulator
Ga1P1_ICSD_77087	insulator	metal
Ga1Pd1Sc1_ICSD_635080	metal	metal
Ga1Pd1Tb1_ICSD_54586	metal	metal
Ga1Pd1Y1_ICSD_635097	metal	metal
Ga1Pd1Yb1_ICSD_635102	metal	metal
Ga1Pd1_ICSD_635060	metal	metal
Ga1Pd2Sc1_ICSD_103909	metal	metal
Ga1Pd2Y1_ICSD_635098	metal	metal
Ga1Pd2_ICSD_409939	metal	metal
Ga1Pr1Zn1_ICSD_635129	metal	metal
Ga1Pr1_ICSD_103912	metal	metal
Ga1Pr1_ICSD_635104	metal	metal
Ga1Pr1_ICSD_635123	metal	metal
Ga1Pr3_ICSD_103914	metal	metal
Ga1Pt1Sc1_ICSD_635150	metal	metal
Ga1Pt1Tb1_ICSD_106747	metal	metal
Ga1Pt1Th1_ICSD_635155	metal	metal
Ga1Pt1Ti1_ICSD_156263	metal	metal
Ga1Pt1Y1_ICSD_635163	metal	metal
Ga1Pt1Yb1_ICSD_635165	metal	metal
Ga1Pt1Zr1_ICSD_152132	metal	metal
Ga1Pt1Zr1_ICSD_635168	metal	metal
Ga1Pt1_ICSD_635132	metal	metal
Ga1Pt2_ICSD_103919	metal	metal
Ga1Pt3_ICSD_103922	metal	metal
Ga1Pt3_ICSD_635134	metal	metal

Supplementary Table 397. Five-fold cross validated predictions for the metal/insulator classification (389/598).

system	calculated	predicted
Ga1Pt3_ICSD_635145	metal	metal
Ga1Pu1Rh1_ICSD_635198	metal	metal
Ga1Pu1_ICSD_103930	metal	metal
Ga1Pu3_ICSD_635171	metal	metal
Ga1Rh1Th1_ICSD_635214	metal	metal
Ga1Rh1Ti1_ICSD_108492	metal	metal
Ga1Rh1U1_ICSD_635219	metal	metal
Ga1Rh1Y1_ICSD_635221	metal	metal
Ga1Rh1Zr1_ICSD_635225	metal	metal
Ga1Rh1_ICSD_657338	metal	metal
Ga1Ru1U1_ICSD_600396	metal	metal
Ga1Ru1U1_ICSD_635235	metal	metal
Ga1Ru1_ICSD_103950	metal	metal
Ga1S1_ICSD_40824	insulator	insulator
Ga1S1_ICSD_635244	insulator	insulator
Ga1S2Tl1_ICSD_180614	insulator	insulator
Ga1S8Ti4_ICSD_260772	metal	insulator
Ga1S8V4_ICSD_603133	metal	metal
Ga1Sb1_ICSD_44979	metal	metal
Ga1Sc1_ICSD_103954	metal	metal
Ga1Se1_ICSD_2002	insulator	insulator
Ga1Se1_ICSD_20237	metal	insulator
Ga1Se1_ICSD_635382	insulator	insulator
Ga1Se1_ICSD_660262	insulator	insulator
Ga1Se1_ICSD_73388	insulator	insulator
Ga1Se2Tl1_ICSD_100131	insulator	insulator
Ga1Se2Tl1_ICSD_1573	insulator	insulator
Ga1Se2Tl1_ICSD_157752	insulator	insulator
Ga1Se4Ta4Te4_ICSD_182921	metal	metal
Ga1Se8Ta4_ICSD_170660	metal	metal
Ga1Se8V4_ICSD_603125	metal	metal
Ga1Sn1Sr1_ICSD_166387	metal	metal
Ga1Sn2V2_ICSD_106752	metal	metal
Ga1Sn3Zr5_ICSD_106753	metal	metal
Ga1Tb1_ICSD_103979	metal	metal
Ga1Te1_ICSD_43328	insulator	insulator
Ga1Te1_ICSD_8249	metal	insulator
Ga1Te2Tl1_ICSD_635523	insulator	insulator
Ga1Th2_ICSD_102812	metal	metal
Ga1Ti1_ICSD_103989	metal	metal
Ga1Ti2_ICSD_182430	metal	metal
Ga1Ti2_ICSD_189699	metal	metal
Ga1Ti3_ICSD_103991	metal	metal
Ga1Ti3_ICSD_635554	metal	metal
Ga1Tm1Zn1_ICSD_635590	metal	metal
Ga1Tm1_ICSD_635577	metal	metal
Ga1V3_ICSD_104021	metal	metal
Ga1V3_ICSD_635603	metal	metal
Ga1V3_ICSD_635607	metal	metal
Ga1V3_ICSD_635624	metal	metal

Supplementary Table 398. Five-fold cross validated predictions for the metal/insulator classification (390/598).

system	calculated	predicted
Ga1V3_ICSD_635625	metal	metal
Ga1V3_ICSD_635636	metal	metal
Ga1Y1Zn1_ICSD_635662	metal	metal
Ga1Y1_ICSD_104030	metal	metal
Ga1Yb1_ICSD_635668	metal	metal
Ga1Zr2_ICSD_102806	metal	metal
Ga1_ICSD_12173	metal	metal
Ga1_ICSD_12174	metal	metal
Ga1_ICSD_165978	metal	metal
Ga1_ICSD_165979	metal	metal
Ga1_ICSD_23247	metal	metal
Ga1_ICSD_2513	metal	metal
Ga1_ICSD_2795	metal	metal
Ga1_ICSD_43539	metal	metal
Ga1_ICSD_654170	metal	metal
Ga2Gd1_ICSD_107426	metal	metal
Ga2Gd1_ICSD_601806	metal	metal
Ga2Gd1_ICSD_634204	metal	metal
Ga2Gd1_ICSD_634210	metal	metal
Ga2Gd1_ICSD_634223	metal	metal
Ga2Ge1La2S8_ICSD_262241	insulator	insulator
Ga2Ge1Li2S6_ICSD_161763	metal	insulator
Ga2Ge4O14Pb3_ICSD_250123	insulator	insulator
Ga2H3O9P3_ICSD_74528	insulator	insulator
Ga2H6O12Te3_ICSD_240957	insulator	insulator
Ga2Hf6Ni1_ICSD_634312	metal	metal
Ga2Hg1S4_ICSD_189739	insulator	insulator
Ga2Hg1Se4_ICSD_188545	insulator	insulator
Ga2Hg1Te4_ICSD_53575	insulator	insulator
Ga2Ho1Ni1_ICSD_634393	metal	metal
Ga2Ho1Pd1_ICSD_600151	metal	metal
Ga2Ho1_ICSD_110149	metal	metal
Ga2Ho3Mn3Si1_ICSD_99077	metal	metal
Ga2Ho3Ni6_ICSD_634381	metal	metal
Ga2Ho3_ICSD_634368	metal	metal
Ga2I2Y2_ICSD_417149	metal	metal
Ga2I3_ICSD_24822	insulator	insulator
Ga2I8Pd1_ICSD_413230	insulator	insulator
Ga2In6O8Pt1_ICSD_411505	insulator	insulator
Ga2Ir1Li1_ICSD_106709	metal	metal
Ga2K2Na4O6_ICSD_62136	insulator	insulator
Ga2K6O6_ICSD_2269	insulator	insulator
Ga2K6Se6_ICSD_300170	insulator	insulator
Ga2K6Te6_ICSD_300171	insulator	insulator
Ga2La1Ni1_ICSD_634496	metal	metal
Ga2La1_ICSD_409562	metal	metal
Ga2La2O1S6Zn1_ICSD_61044	insulator	insulator
Ga2La3Ni2_ICSD_20939	metal	metal
Ga2Li1Pd1_ICSD_106712	metal	metal
Ga2Li1Pt1_ICSD_107094	metal	metal

Supplementary Table 399. Five-fold cross validated predictions for the metal/insulator classification (391/598).

system	calculated	predicted
Ga2Li1Rh1_ICSD_106715	metal	metal
Ga2Li1Ru1_ICSD_106716	metal	metal
Ga2Li3_ICSD_659760	metal	metal
Ga2Lu3Mn3Si1_ICSD_99080	metal	metal
Ga2Mg1O4_ICSD_86507	insulator	insulator
Ga2Mg1S4_ICSD_15350	insulator	insulator
Ga2Mg1Sc2_ICSD_260213	metal	metal
Ga2Mg1_ICSD_103792	metal	metal
Ga2Mg1_ICSD_23226	metal	metal
Ga2Mg5_ICSD_103794	metal	metal
Ga2Mn1S4_ICSD_634668	insulator	insulator
Ga2Mn1Se4_ICSD_634676	insulator	insulator
Ga2Mn1Se4_ICSD_659100	insulator	insulator
Ga2Mn1Te4_ICSD_67402	metal	insulator
Ga2Mn2Pr6S14_ICSD_634657	insulator	insulator
Ga2Mn2S5_ICSD_634669	insulator	insulator
Ga2Mn3Si1Tb3_ICSD_99075	metal	metal
Ga2Mn3Si1Tm3_ICSD_99079	metal	metal
Ga2N4Sr3_ICSD_170441	insulator	insulator
Ga2Nb3_ICSD_103832	metal	metal
Ga2Nd1Ni1_ICSD_103850	metal	metal
Ga2Nd1_ICSD_103849	metal	metal
Ga2Nd1_ICSD_106734	metal	metal
Ga2Nd1_ICSD_634804	metal	metal
Ga2Nd1_ICSD_658432	metal	metal
Ga2Nd3Ni6_ICSD_634818	metal	metal
Ga2Ni1S4_ICSD_59230	metal	insulator
Ga2Ni1S4_ICSD_634901	insulator	insulator
Ga2Ni1Sc1_ICSD_103875	metal	metal
Ga2Ni1Tb1_ICSD_634949	metal	metal
Ga2Ni1Y1_ICSD_103895	metal	metal
Ga2Ni1Yb1_ICSD_262737	metal	metal
Ga2Ni1Zr6_ICSD_635001	metal	metal
Ga2Ni3Y1_ICSD_103896	metal	metal
Ga2Ni6Tb3_ICSD_634938	metal	metal
Ga2Ni6Y3_ICSD_634985	metal	metal
Ga2O11Te4_ICSD_94349	insulator	insulator
Ga2O3_ICSD_162252	insulator	insulator
Ga2O3_ICSD_184327	insulator	insulator
Ga2O3_ICSD_635016	insulator	insulator
Ga2O4Pb1_ICSD_33533	insulator	insulator
Ga2O4Pb1_ICSD_80129	insulator	insulator
Ga2O4Sr1_ICSD_15539	insulator	insulator
Ga2O4Zn1_ICSD_81107	insulator	insulator
Ga2O6Rb6_ICSD_2270	insulator	insulator
Ga2O6Te1_ICSD_78346	insulator	insulator
Ga2O9Te3_ICSD_261175	insulator	insulator
Ga2Os1_ICSD_103785	insulator	metal
Ga2Pb1Se4_ICSD_635052	insulator	insulator
Ga2Pd1Tb1_ICSD_600149	metal	metal

Supplementary Table 400. Five-fold cross validated predictions for the metal/insulator classification (392/598).

system	calculated	predicted
Ga2Pd1Y1_ICSD_600148	metal	metal
Ga2Pd1Yb1_ICSD_600145	metal	metal
Ga2Pd5_ICSD_103904	metal	metal
Ga2Pr1_ICSD_103916	metal	metal
Ga2Pr1_ICSD_635108	metal	metal
Ga2Pt1_ICSD_103925	metal	metal
Ga2Pu1_ICSD_635181	metal	metal
Ga2Ru1_ICSD_635228	insulator	metal
Ga2S2Te1_ICSD_8028	insulator	insulator
Ga2S3_ICSD_409550	insulator	insulator
Ga2S3_ICSD_488	insulator	insulator
Ga2S4Sr1_ICSD_635274	insulator	insulator
Ga2S4Zn1_ICSD_635300	insulator	insulator
Ga2S4Zn1_ICSD_635305	insulator	insulator
Ga2S5Sn2_ICSD_37173	insulator	insulator
Ga2Sc1_ICSD_103955	metal	metal
Ga2Sc1_ICSD_103956	metal	metal
Ga2Se2Te1_ICSD_64617	insulator	insulator
Ga2Se3_ICSD_35028	insulator	insulator
Ga2Se4Sr1_ICSD_635401	insulator	insulator
Ga2Se4Yb1_ICSD_635414	insulator	insulator
Ga2Se4Zn1_ICSD_168594	insulator	insulator
Ga2Sr1Te4_ICSD_41166	insulator	insulator
Ga2Sr1_ICSD_150473	metal	metal
Ga2Ta3_ICSD_635490	metal	metal
Ga2Tb1_ICSD_106754	metal	metal
Ga2Te3_ICSD_657608	metal	metal
Ga2Te4Zn1_ICSD_44888	insulator	insulator
Ga2Te5_ICSD_1085	insulator	insulator
Ga2Th1_ICSD_103985	metal	metal
Ga2Th3_ICSD_103988	metal	metal
Ga2Tm1_ICSD_104006	metal	metal
Ga2U1_ICSD_104013	metal	metal
Ga2Y1_ICSD_104032	metal	metal
Ga2Yb1_ICSD_104035	metal	metal
Ga2Yb1_ICSD_107582	metal	metal
Ga2Zr1_ICSD_104041	metal	metal
Ga2Zr3_ICSD_635692	metal	metal
Ga3Gd1Ru1_ICSD_634241	insulator	metal
Ga3Hf1_ICSD_103728	metal	metal
Ga3Hf2_ICSD_103729	metal	metal
Ga3Hf3Nb2_ICSD_103733	metal	metal
Ga3Hf5_ICSD_634295	metal	metal
Ga3Ho1Ru1_ICSD_634401	metal	metal
Ga3Ho1_ICSD_110129	metal	metal
Ga3Ho1_ICSD_602234	metal	metal
Ga3Ho1_ICSD_602235	metal	metal
Ga3Ho1_ICSD_634364	metal	metal
Ga3Ho5_ICSD_634357	metal	metal
Ga3Ho5_ICSD_658182	metal	metal

Supplementary Table 401. Five-fold cross validated predictions for the metal/insulator classification (393/598).

system	calculated	predicted
Ga3I1Te3_ICSD_61678	insulator	insulator
Ga3I1Te3_ICSD_66031	insulator	insulator
Ga3Ir1_ICSD_634441	metal	metal
Ga3K1_ICSD_20664	insulator	metal
Ga3La1Pd2_ICSD_106710	metal	metal
Ga3Lu1_ICSD_106721	metal	metal
Ga3N5Sr3_ICSD_170443	insulator	insulator
Ga3Na3O12Te2_ICSD_418645	insulator	insulator
Ga3Nb1_ICSD_634760	metal	metal
Ga3Nd1_ICSD_103847	metal	metal
Ga3Ni1U1_ICSD_106826	metal	metal
Ga3Ni2Ti4_ICSD_103888	metal	metal
Ga3Ni2_ICSD_634859	metal	metal
Ga3Ni3Zn1_ICSD_103898	metal	metal
Ga3Ni5_ICSD_103861	metal	metal
Ga3O14Si2Sr3Ta1_ICSD_421591	insulator	insulator
Ga3O7P1_ICSD_59364	insulator	insulator
Ga3Os1Tb1_ICSD_412144	metal	metal
Ga3Os1_ICSD_635024	insulator	metal
Ga3Pd5_ICSD_635071	metal	metal
Ga3Pd7_ICSD_107292	metal	metal
Ga3Pt2_ICSD_103926	metal	metal
Ga3Pt5_ICSD_103927	metal	metal
Ga3Pu1_ICSD_246481	metal	metal
Ga3Rb1S5_ICSD_419822	insulator	insulator
Ga3Rb1_ICSD_103943	insulator	metal
Ga3Re1_ICSD_635203	metal	metal
Ga3Rh1_ICSD_635208	metal	metal
Ga3Ru1Tb1_ICSD_635232	metal	metal
Ga3Ru1Tm1_ICSD_106748	metal	metal
Ga3Ru1Y1_ICSD_635239	metal	metal
Ga3Ru1_ICSD_412078	insulator	metal
Ga3Sc1_ICSD_635343	metal	metal
Ga3Sc5_ICSD_635341	metal	metal
Ga3Ta1_ICSD_103977	metal	metal
Ga3Ta5_ICSD_103978	metal	metal
Ga3Tb1_ICSD_103981	metal	metal
Ga3Tb1_ICSD_635497	metal	metal
Ga3Ti1_ICSD_103993	metal	metal
Ga3Ti2_ICSD_103995	metal	metal
Ga3Tm1_ICSD_104007	metal	metal
Ga3Tm1_ICSD_104008	metal	metal
Ga3Tm1_ICSD_635583	metal	metal
Ga3U1_ICSD_104015	metal	metal
Ga3U1_ICSD_635598	metal	metal
Ga3Y5_ICSD_150544	metal	metal
Ga3Zr1_ICSD_104043	metal	metal
Ga41Mo8_ICSD_634698	metal	metal
Ga4Ge1O8_ICSD_202044	insulator	insulator
Ga4Ho1Ni1_ICSD_423079	metal	metal

Supplementary Table 402. Five-fold cross validated predictions for the metal/insulator classification (394/598).

system	calculated	predicted
Ga4Ho1Ti2_ICSD_634403	metal	metal
Ga4Li1Y1_ICSD_98666	metal	metal
Ga4Mn1_ICSD_634630	metal	metal
Ga4Na1_ICSD_103824	metal	metal
Ga4Nb5_ICSD_103837	metal	metal
Ga4Ni1Tb1_ICSD_634948	metal	metal
Ga4Ni1Tm1_ICSD_423069	metal	metal
Ga4Ni1Y1_ICSD_634990	metal	metal
Ga4Ni1Yb1_ICSD_154676	metal	metal
Ga4Ni3_ICSD_103864	metal	metal
Ga4O7Sr1_ICSD_10316	insulator	insulator
Ga4O8Ti1_ICSD_155638	insulator	insulator
Ga4O9Sr3_ICSD_51546	insulator	insulator
Ga4Pd7Zn3_ICSD_103911	metal	metal
Ga4Sc1Ti2_ICSD_635352	metal	metal
Ga4Sc1V2_ICSD_635354	metal	metal
Ga4Sr1_ICSD_635459	metal	metal
Ga4Ti2Y1_ICSD_103999	metal	metal
Ga4V2Zr1_ICSD_635647	metal	metal
Ga4Yb1_ICSD_104036	metal	metal
Ga4Yb1_ICSD_635673	metal	metal
Ga5Gd3O12_ICSD_184931	insulator	insulator
Ga5H1O8_ICSD_236277	insulator	insulator
Ga5Hf1Ni1_ICSD_634319	metal	metal
Ga5Ho3O12_ICSD_409390	insulator	insulator
Ga5Ir1U1_ICSD_103639	metal	metal
Ga5La3O14Sn1_ICSD_412328	insulator	insulator
Ga5Li1O8_ICSD_33716	insulator	insulator
Ga5Lu3Ni1_ICSD_634575	metal	metal
Ga5Mg2_ICSD_23227	metal	metal
Ga5Mn2_ICSD_634639	metal	metal
Ga5Mn8_ICSD_634632	metal	metal
Ga5N1Sr6_ICSD_77645	metal	metal
Ga5Na1O12Ti2_ICSD_108816	insulator	insulator
Ga5Nd3O12_ICSD_84872	insulator	insulator
Ga5Ni1U1_ICSD_600552	metal	metal
Ga5Ni1_ICSD_165723	metal	metal
Ga5Ni8Zn36_ICSD_103900	metal	metal
Ga5O12Tb3_ICSD_184934	insulator	insulator
Ga5O12Y3_ICSD_80148	insulator	insulator
Ga5O12Yb3_ICSD_23851	insulator	insulator
Ga5Os1U1_ICSD_600559	metal	metal
Ga5Pd1U1_ICSD_600553	metal	metal
Ga5Pd1_ICSD_103908	metal	metal
Ga5Pt1U1_ICSD_600554	metal	metal
Ga5Rh1U1_ICSD_600550	metal	metal
Ga5Rh3_ICSD_240179	metal	metal
Ga5Ru1U1_ICSD_600548	metal	metal
Ga5Ta6_ICSD_635465	metal	metal
Ga5V2_ICSD_635642	metal	metal

Supplementary Table 403. Five-fold cross validated predictions for the metal/insulator classification (395/598).

system	calculated	predicted
Ga5V6_ICSD_635633	metal	metal
Ga5W2_ICSD_635650	metal	metal
Ga6Gd2Mn1_ICSD_103721	metal	metal
Ga6Ge6Mn1Y4_ICSD_248234	metal	metal
Ga6Hf3Mn2_ICSD_103732	metal	metal
Ga6I2Li8O24Si6_ICSD_87989	insulator	insulator
Ga6La1Pd1_ICSD_240162	metal	metal
Ga6La1_ICSD_103768	metal	metal
Ga6La1_ICSD_634486	metal	metal
Ga6Mn1Tb2_ICSD_600191	metal	metal
Ga6Pu1_ICSD_103942	metal	metal
Ga6Y1_ICSD_104033	metal	metal
Ga7Ni3_ICSD_408313	metal	metal
Ga7Pd3_ICSD_174527	metal	metal
Ga7Pt3_ICSD_180363	metal	metal
Ga7Rb1_ICSD_103946	metal	metal
Ga7Te10_ICSD_400668	insulator	insulator
Ga8Ho3Ir3_ICSD_634379	metal	metal
Ga8Ru1U2_ICSD_106749	metal	metal
Ga9Ho3Pd2_ICSD_634397	metal	metal
Ga9Ni13_ICSD_103866	metal	metal
Ga9O2S13Tl3_ICSD_61256	insulator	insulator
Gd1Ge1Ir1_ICSD_88163	metal	metal
Gd1Ge1K1S4_ICSD_603183	insulator	insulator
Gd1Ge1Li1_ICSD_32030	metal	metal
Gd1Ge1Na1O4_ICSD_85496	insulator	insulator
Gd1Ge1Sc1_ICSD_86068	metal	metal
Gd1Ge1Ti1_ICSD_85905	metal	metal
Gd1Ge1Zn1_ICSD_163344	metal	metal
Gd1Ge1_ICSD_635724	metal	metal
Gd1Ge2Mn2_ICSD_53609	metal	metal
Gd1Ge2Ni2_ICSD_106851	metal	metal
Gd1Ge2Pd2_ICSD_52771	metal	metal
Gd1Ge2Pd2_ICSD_53610	metal	metal
Gd1Ge2Pt2_ICSD_53611	metal	metal
Gd1Ge2Rh2_ICSD_53612	metal	metal
Gd1Ge2_ICSD_635723	metal	metal
Gd1H2_ICSD_635801	metal	metal
Gd1H3O3_ICSD_200093	insulator	insulator
Gd1H3_ICSD_44740	metal	metal
Gd1H3_ICSD_77970	metal	insulator
Gd1Hg1_ICSD_104046	metal	metal
Gd1Hg2_ICSD_635811	metal	metal
Gd1Hg3_ICSD_635810	metal	metal
Gd1I1S1_ICSD_416462	insulator	insulator
Gd1I1S1_ICSD_59203	metal	metal
Gd1In1Ir1_ICSD_414478	metal	metal
Gd1In1Mg1_ICSD_54617	metal	metal
Gd1In1Pd1_ICSD_415506	metal	metal
Gd1In1Pd2_ICSD_104054	metal	metal

Supplementary Table 404. Five-fold cross validated predictions for the metal/insulator classification (396/598).

system	calculated	predicted
Gd1In1Pt1_ICSD_104055	metal	metal
Gd1In1Pt4_ICSD_635842	metal	metal
Gd1In1Rh1_ICSD_104056	metal	metal
Gd1In1Zn1_ICSD_635852	metal	metal
Gd1In1_ICSD_104048	metal	metal
Gd1In1_ICSD_104049	metal	metal
Gd1In3_ICSD_104051	metal	metal
Gd1In3_ICSD_635829	metal	metal
Gd1Ir1Si3_ICSD_635872	metal	metal
Gd1Ir2Si2_ICSD_104062	metal	metal
Gd1Ir2Si2_ICSD_635869	metal	metal
Gd1Ir2Si2_ICSD_635877	metal	metal
Gd1Ir2_ICSD_635863	metal	metal
Gd1Ir3_ICSD_104061	metal	metal
Gd1K1Na1Nb1O5_ICSD_420037	insulator	insulator
Gd1K1Na1O5Ta1_ICSD_419854	insulator	insulator
Gd1K1O12P4_ICSD_171710	insulator	insulator
Gd1K1O3Pd1_ICSD_417105	insulator	insulator
Gd1K1O8W2_ICSD_68249	insulator	insulator
Gd1K1S2_ICSD_44945	insulator	insulator
Gd1K1Te2_ICSD_98657	insulator	insulator
Gd1K1Te4_ICSD_391205	metal	insulator
Gd1K2O8Rb1V2_ICSD_39370	insulator	insulator
Gd1K3O8P2_ICSD_260894	insulator	insulator
Gd1Li1O12P4_ICSD_416442	insulator	insulator
Gd1Li1O2_ICSD_27769	insulator	insulator
Gd1Li1O2_ICSD_422561	insulator	insulator
Gd1Li1Se2_ICSD_44961	insulator	insulator
Gd1Li1Sn1_ICSD_603171	metal	metal
Gd1Lu3S6_ICSD_30073	metal	insulator
Gd1Mg1Pd1_ICSD_412441	metal	metal
Gd1Mg1Pt1_ICSD_412443	metal	metal
Gd1Mg1_ICSD_161746	metal	metal
Gd1Mg1_ICSD_635913	metal	metal
Gd1Mg2Ni9_ICSD_55619	metal	metal
Gd1Mg2_ICSD_104066	metal	metal
Gd1Mg2_ICSD_104067	metal	metal
Gd1Mg3_ICSD_104068	metal	metal
Gd1Mg3_ICSD_635907	metal	metal
Gd1Mn12_ICSD_26823	metal	metal
Gd1Mn1Si1_ICSD_85852	metal	metal
Gd1Mn2Si2_ICSD_54947	metal	metal
Gd1Mn2_ICSD_104073	metal	metal
Gd1Mn2_ICSD_635929	metal	metal
Gd1Mo6S8_ICSD_635984	metal	metal
Gd1N1_ICSD_635993	metal	metal
Gd1Na1O2_ICSD_97542	insulator	insulator
Gd1Na1O4Si1_ICSD_16188	insulator	insulator
Gd1Na1O4Ti1_ICSD_81537	insulator	insulator
Gd1Na1O4Ti1_ICSD_82006	insulator	insulator

Supplementary Table 405. Five-fold cross validated predictions for the metal/insulator classification (397/598).

system	calculated	predicted
Gd1Na1S2_ICSD_37332	insulator	insulator
Gd1Na1Se2_ICSD_636002	insulator	insulator
Gd1Ni1Sb1_ICSD_636074	insulator	insulator
Gd1Ni1Si2_ICSD_601070	metal	metal
Gd1Ni1Si2_ICSD_636080	metal	metal
Gd1Ni1_ICSD_104087	metal	metal
Gd1Ni1_ICSD_636039	metal	metal
Gd1Ni2P2_ICSD_636061	metal	metal
Gd1Ni2Si2_ICSD_53619	metal	metal
Gd1Ni2_ICSD_104088	metal	metal
Gd1Ni4P2_ICSD_601102	metal	metal
Gd1Ni5_ICSD_636010	metal	metal
Gd1O1P1Zn1_ICSD_418524	insulator	insulator
Gd1O1_ICSD_24981	metal	metal
Gd1O2Rb1_ICSD_27335	insulator	insulator
Gd1O2_ICSD_6318	metal	insulator
Gd1O3Sc1_ICSD_65513	insulator	insulator
Gd1O4P1_ICSD_184554	insulator	insulator
Gd1O4P1_ICSD_184560	insulator	insulator
Gd1O4P1_ICSD_79753	insulator	insulator
Gd1O4Ta1_ICSD_415433	insulator	insulator
Gd1O4V1_ICSD_81703	insulator	insulator
Gd1O6Re1Sr2_ICSD_25400	metal	metal
Gd1O6Ru1Sr2_ICSD_183381	insulator	insulator
Gd1O8Rb1W2_ICSD_152962	insulator	insulator
Gd1O9P3_ICSD_155301	insulator	insulator
Gd1Os2Si2_ICSD_636121	metal	metal
Gd1Os2_ICSD_104105	metal	metal
Gd1P1Pd1_ICSD_57315	metal	metal
Gd1P1Pt1_ICSD_53623	metal	metal
Gd1P1_ICSD_53622	metal	metal
Gd1P2Ru2_ICSD_602099	metal	metal
Gd1P5_ICSD_114	metal	metal
Gd1Pb3_ICSD_104107	metal	metal
Gd1Pb3_ICSD_636136	metal	metal
Gd1Pd1Sb1_ICSD_415946	metal	metal
Gd1Pd1Sb1_ICSD_636173	metal	metal
Gd1Pd1Sb2_ICSD_658222	metal	metal
Gd1Pd1Sn1_ICSD_104114	metal	metal
Gd1Pd1Tl1_ICSD_104116	metal	metal
Gd1Pd1Zn1_ICSD_183339	metal	metal
Gd1Pd1_ICSD_636150	metal	metal
Gd1Pd2Si2_ICSD_106828	metal	metal
Gd1Pd2Si2_ICSD_57317	metal	metal
Gd1Pd2_ICSD_106831	metal	metal
Gd1Pd3_ICSD_104109	metal	metal
Gd1Pd3_ICSD_636146	metal	metal
Gd1Pd3_ICSD_636155	metal	metal
Gd1Pt1Sb1_ICSD_53624	metal	metal
Gd1Pt1Sb1_ICSD_57318	metal	metal

Supplementary Table 406. Five-fold cross validated predictions for the metal/insulator classification (398/598).

system	calculated	predicted
Gd1Pt1Sn1_ICSD_417532	metal	metal
Gd1Pt1Sn1_ICSD_636241	metal	metal
Gd1Pt1_ICSD_636215	metal	metal
Gd1Pt2Si2_ICSD_602025	metal	metal
Gd1Pt2Si2_ICSD_636238	metal	metal
Gd1Pt2Sn1_ICSD_104124	metal	metal
Gd1Pt2_ICSD_104121	metal	metal
Gd1Pt2_ICSD_150737	metal	metal
Gd1Pt2_ICSD_636205	metal	metal
Gd1Pt2_ICSD_636217	metal	metal
Gd1Pt3_ICSD_104122	metal	metal
Gd1Rb1S2_ICSD_81400	insulator	insulator
Gd1Rb1Se2_ICSD_281072	insulator	insulator
Gd1Rh1Sn1_ICSD_413353	metal	metal
Gd1Rh1_ICSD_104125	metal	metal
Gd1Rh2Si2_ICSD_53626	metal	metal
Gd1Rh2Si2_ICSD_602051	metal	metal
Gd1Rh2Si2_ICSD_636280	metal	metal
Gd1Rh2_ICSD_104127	metal	metal
Gd1Rh3Si2_ICSD_53627	metal	metal
Gd1Rh5_ICSD_104128	metal	metal
Gd1Ru1Si1_ICSD_41255	metal	metal
Gd1Ru2_ICSD_106830	metal	metal
Gd1Ru2_ICSD_150522	metal	metal
Gd1Ru4Sn6_ICSD_410808	metal	metal
Gd1S1_ICSD_636313	metal	metal
Gd1S2Tl1_ICSD_57323	insulator	insulator
Gd1S2_ICSD_104131	metal	metal
Gd1S2_ICSD_636318	metal	metal
Gd1S3Sc1_ICSD_173844	insulator	insulator
Gd1Sb1Zr1_ICSD_107068	metal	metal
Gd1Sb1_ICSD_43635	metal	metal
Gd1Sb1_ICSD_636379	metal	metal
Gd1Sb1_ICSD_636380	metal	metal
Gd1Sb2_ICSD_636367	metal	metal
Gd1Sc1Si1_ICSD_182419	metal	metal
Gd1Sc1Si1_ICSD_86067	metal	metal
Gd1Se1_ICSD_636402	metal	metal
Gd1Se2Tl1_ICSD_106989	insulator	insulator
Gd1Se2Tl1_ICSD_636407	insulator	insulator
Gd1Se2_ICSD_636398	metal	metal
Gd1Si1Ti1_ICSD_88114	metal	metal
Gd1Si1_ICSD_636423	metal	metal
Gd1Si2_ICSD_150661	metal	metal
Gd1Si2_ICSD_53633	metal	metal
Gd1Si5_ICSD_174530	metal	metal
Gd1Sn1Zn1_ICSD_152621	metal	metal
Gd1Sn3_ICSD_104133	metal	metal
Gd1Sn3_ICSD_182147	metal	metal
Gd1Sn4Ti6_ICSD_166193	metal	metal

Supplementary Table 407. Five-fold cross validated predictions for the metal/insulator classification (399/598).

system	calculated	predicted
Gd1Te1_ICSD_104137	metal	metal
Gd1Te2Tl1_ICSD_636475	metal	insulator
Gd1Te2_ICSD_636465	metal	metal
Gd1Th1_ICSD_109243	metal	metal
Gd1Tl1Zn1_ICSD_636493	metal	metal
Gd1Tl1_ICSD_104140	metal	metal
Gd1Tl3_ICSD_104141	metal	metal
Gd1Tl3_ICSD_104142	metal	metal
Gd1Tl3_ICSD_636486	metal	metal
Gd1Zn12_ICSD_636505	metal	metal
Gd1Zn12_ICSD_636519	metal	metal
Gd1Zn1_ICSD_636518	metal	metal
Gd1Zn2_ICSD_636499	metal	metal
Gd1Zn5_ICSD_104150	metal	metal
Gd1_ICSD_102659	metal	metal
Gd1_ICSD_104045	metal	metal
Gd1_ICSD_20502	metal	metal
Gd1_ICSD_52517	metal	metal
Gd1_ICSD_53607	metal	metal
Gd2Ge1O5_ICSD_61372	insulator	insulator
Gd2Ge2In1_ICSD_172490	metal	metal
Gd2Ge2Mg1_ICSD_93403	metal	metal
Gd2Ge3Zn6_ICSD_98237	metal	metal
Gd2Hf2O7_ICSD_236213	insulator	insulator
Gd2In1Ni2_ICSD_635833	metal	metal
Gd2In1Pd2_ICSD_658296	metal	metal
Gd2In1_ICSD_104052	metal	metal
Gd2Mo2O7_ICSD_159776	metal	insulator
Gd2O1S2_ICSD_409708	insulator	insulator
Gd2O1Se2_ICSD_280201	insulator	insulator
Gd2O2S1_ICSD_636114	insulator	insulator
Gd2O2Se1_ICSD_25808	insulator	insulator
Gd2O2Te1_ICSD_89563	insulator	insulator
Gd2O3_ICSD_160211	insulator	insulator
Gd2O3_ICSD_162247	insulator	insulator
Gd2O3_ICSD_184528	insulator	insulator
Gd2O3_ICSD_40473	insulator	insulator
Gd2O4Si1Te1_ICSD_409569	insulator	insulator
Gd2O4Sr1_ICSD_96232	insulator	insulator
Gd2O5Si1_ICSD_27728	insulator	insulator
Gd2O6Te1_ICSD_412447	insulator	insulator
Gd2O6W1_ICSD_62887	insulator	insulator
Gd2O6W1_ICSD_62888	insulator	insulator
Gd2O7Ru2_ICSD_79332	metal	insulator
Gd2O7Sc2Sr1_ICSD_167052	insulator	insulator
Gd2O7Si2_ICSD_20316	insulator	insulator
Gd2O7Sn2_ICSD_84753	insulator	insulator
Gd2O7Ti2_ICSD_164024	insulator	insulator
Gd2O7Zr2_ICSD_185396	metal	insulator
Gd2O7Zr2_ICSD_188035	insulator	metal

Supplementary Table 408. Five-fold cross validated predictions for the metal/insulator classification (400/598).

system	calculated	predicted
Gd2Pt1_ICSD_636221	metal	metal
Gd2Rh1Si3_ICSD_636281	metal	metal
Gd2S3_ICSD_33785	insulator	insulator
Gd2S5Sn1_ICSD_636350	metal	metal
Gd2S5Zr1_ICSD_425080	insulator	insulator
Gd2Se3_ICSD_636396	insulator	insulator
Gd2Te3_ICSD_63649	metal	metal
Gd2Tl1_ICSD_636491	metal	metal
Gd3Ge5_ICSD_416583	metal	metal
Gd3I3Si1_ICSD_67361	metal	metal
Gd3In1N1_ICSD_98503	metal	metal
Gd3In1Se6_ICSD_280242	insulator	insulator
Gd3Ir1_ICSD_635853	metal	metal
Gd3N1S3_ICSD_416220	insulator	insulator
Gd3Nb1O7_ICSD_174285	insulator	insulator
Gd3Ni1_ICSD_104095	metal	metal
Gd3Ni6Si2_ICSD_601071	metal	metal
Gd3O12Sb5_ICSD_67024	insulator	insulator
Gd3O3Sb1_ICSD_380460	insulator	insulator
Gd3O7Os1_ICSD_170875	metal	insulator
Gd3O7Re1_ICSD_99252	insulator	insulator
Gd3O7Ru1_ICSD_92067	insulator	insulator
Gd3Os1_ICSD_636118	metal	metal
Gd3P4Pd7_ICSD_409873	metal	metal
Gd3Pd2_ICSD_107680	metal	metal
Gd3Pd4_ICSD_636163	metal	metal
Gd3Pt1_ICSD_636202	metal	metal
Gd3Pt4_ICSD_636211	metal	metal
Gd3Rh1_ICSD_636259	metal	metal
Gd3Ru1_ICSD_636293	metal	metal
Gd3S4_ICSD_636332	metal	metal
Gd3Se4_ICSD_636389	metal	metal
Gd4I5Si1_ICSD_67360	metal	metal
Gd4O4Se4Ti1_ICSD_93873	insulator	insulator
Gd4Sb3_ICSD_636381	metal	metal
Gd5Ge3_ICSD_43262	metal	metal
Gd5Si3_ICSD_636431	metal	metal
Gd5Si3_ICSD_99641	metal	metal
Gd6Ni1Te2_ICSD_260932	metal	metal
Gd6Zn23_ICSD_636504	metal	metal
Gd7Pd3_ICSD_104112	metal	metal
Ge10Ir4Y5_ICSD_636767	metal	metal
Ge10Os4Y5_ICSD_637487	metal	metal
Ge10Tb11_ICSD_637971	metal	metal
Ge12La1Pt4_ICSD_174552	metal	metal
Ge12Nd1Pt4_ICSD_174555	metal	metal
Ge12Pr1Pt4_ICSD_174554	metal	metal
Ge136_ICSD_245948	insulator	metal
Ge136_ICSD_245955	metal	insulator
Ge13Ho3Ir4_ICSD_636631	metal	metal

Supplementary Table 409. Five-fold cross validated predictions for the metal/insulator classification (401/598).

system	calculated	predicted
Ge13Ho3Os4_ICSD_636650	metal	metal
Ge13Ho3Rh4_ICSD_636664	metal	metal
Ge13Ho3Ru4_ICSD_636675	metal	metal
Ge13Ir4Lu3_ICSD_636713	metal	metal
Ge13Ir4Nd3_ICSD_636718	metal	metal
Ge13Ir4Pr3_ICSD_636725	metal	metal
Ge13Ir4Tb3_ICSD_636741	metal	metal
Ge13Ir4Y3_ICSD_636763	metal	metal
Ge13Ir4Yb3_ICSD_636769	metal	metal
Ge13Lu3Rh4_ICSD_636932	metal	metal
Ge13Nd3Os4_ICSD_637296	metal	metal
Ge13Nd3Rh4_ICSD_637309	metal	metal
Ge13Os4Pr3_ICSD_637469	metal	metal
Ge13Os4Tb3_ICSD_637474	metal	metal
Ge13Os4Y3_ICSD_637488	metal	metal
Ge13Os4Yb3_ICSD_637490	metal	metal
Ge13Rh4Tb3_ICSD_637696	metal	metal
Ge13Rh4Y3_ICSD_637724	metal	metal
Ge13Rh4Yb3_ICSD_637733	metal	metal
Ge13Ru4Tb3_ICSD_637760	metal	metal
Ge13Ru4Y3_ICSD_53900	metal	metal
Ge13Ru4Yb3_ICSD_637775	metal	metal
Ge1H2O4Sr1_ICSD_30320	insulator	insulator
Ge1H3K3O2S3_ICSD_418259	insulator	insulator
Ge1H4_ICSD_183081	metal	metal
Ge1H4_ICSD_183082	metal	metal
Ge1H4_ICSD_183083	metal	metal
Ge1H4_ICSD_183084	insulator	insulator
Ge1Hf1Ir1_ICSD_636558	metal	metal
Ge1Hf1Mn1_ICSD_636559	metal	metal
Ge1Hf1Ni1_ICSD_636577	metal	metal
Ge1Hf1O4_ICSD_202080	insulator	insulator
Ge1Hf1Os1_ICSD_636578	metal	metal
Ge1Hf1Pd1_ICSD_418027	metal	metal
Ge1Hf1Pt1_ICSD_86691	metal	metal
Ge1Hf1Rh1_ICSD_636581	metal	metal
Ge1Hf1Rh1_ICSD_636582	metal	metal
Ge1Hf1Ru1_ICSD_636584	metal	metal
Ge1Hf1S1_ICSD_25742	metal	metal
Ge1Hf1Se1_ICSD_25741	metal	metal
Ge1Hf1Te1_ICSD_25743	metal	metal
Ge1Hf1Te4_ICSD_241122	insulator	insulator
Ge1Hf1V1_ICSD_636594	metal	metal
Ge1Hf1V1_ICSD_88211	metal	metal
Ge1Hf2_ICSD_42524	metal	metal
Ge1Hf2_ICSD_636554	metal	metal
Ge1Hf3_ICSD_636550	metal	metal
Ge1Hf9Mo4_ICSD_636562	metal	metal
Ge1Hf9Re4_ICSD_636579	metal	metal
Ge1Hg1Li2_ICSD_53646	metal	metal

Supplementary Table 410. Five-fold cross validated predictions for the metal/insulator classification (402/598).

system	calculated	predicted
Ge1Hg1Te4Tl2_ICSD_172504	insulator	insulator
Ge1Hg2O4_ICSD_26340	insulator	insulator
Ge1Hg2Se4_ICSD_636599	insulator	insulator
Ge1Hg4S6_ICSD_636597	insulator	insulator
Ge1Ho1Ir1_ICSD_636629	metal	metal
Ge1Ho1Li1_ICSD_76288	metal	metal
Ge1Ho1Mn1_ICSD_97797	metal	metal
Ge1Ho1Mn1_ICSD_97798	metal	metal
Ge1Ho1Na1O4_ICSD_95964	insulator	insulator
Ge1Ho1Ni1_ICSD_57062	metal	metal
Ge1Ho1Pd1_ICSD_391462	metal	metal
Ge1Ho1Pd2_ICSD_636654	metal	metal
Ge1Ho1Pt1_ICSD_636660	metal	metal
Ge1Ho1Rh1_ICSD_85995	metal	metal
Ge1Ho1Ru1_ICSD_88189	metal	metal
Ge1Ho1Sn1_ICSD_261754	metal	metal
Ge1Ho1Ti1_ICSD_95023	metal	metal
Ge1Ho1_ICSD_636627	metal	metal
Ge1I1O6Rb1_ICSD_73613	insulator	insulator
Ge1I2La2_ICSD_414170	insulator	metal
Ge1I2La2_ICSD_414171	insulator	metal
Ge1I2Y2_ICSD_249477	insulator	metal
Ge1I2_ICSD_23176	insulator	insulator
Ge1I2_ICSD_27674	insulator	insulator
Ge1I3La3_ICSD_414174	metal	metal
Ge1I3Rb1_ICSD_40216	insulator	insulator
Ge1I3Rb1_ICSD_85041	insulator	insulator
Ge1I3Tl1_ICSD_201144	insulator	insulator
Ge1I4_ICSD_67895	insulator	insulator
Ge1I6K2O18_ICSD_2826	insulator	insulator
Ge1In1Li1O4_ICSD_62229	insulator	insulator
Ge1In1Li1_ICSD_53650	metal	metal
Ge1In2Li2S6_ICSD_262643	insulator	insulator
Ge1In2Li2Se6_ICSD_262645	insulator	insulator
Ge1In4La3_ICSD_81469	metal	metal
Ge1In4S4_ICSD_400222	insulator	insulator
Ge1In7Ir1O8_ICSD_417829	insulator	insulator
Ge1Ir1La1_ICSD_636698	metal	metal
Ge1Ir1Mn3_ICSD_150394	metal	metal
Ge1Ir1Nb1_ICSD_411883	metal	metal
Ge1Ir1Nd1_ICSD_636717	metal	metal
Ge1Ir1Sc1_ICSD_636730	metal	metal
Ge1Ir1Tb1_ICSD_88160	metal	metal
Ge1Ir1Ti1_ICSD_636746	metal	metal
Ge1Ir1Y1_ICSD_636761	metal	metal
Ge1Ir1Yb1_ICSD_414433	metal	metal
Ge1Ir1Zr1_ICSD_636771	metal	metal
Ge1Ir1_ICSD_52126	metal	metal
Ge1K1La1S4_ICSD_73970	insulator	insulator
Ge1K1La1Se4_ICSD_73971	insulator	insulator

Supplementary Table 411. Five-fold cross validated predictions for the metal/insulator classification (403/598).

system	calculated	predicted
Ge1K1N1O1_ICSD_60002	insulator	insulator
Ge1K1Nd1S4_ICSD_603182	insulator	insulator
Ge1K1O5P1_ICSD_39735	insulator	insulator
Ge1K1O5Ta1_ICSD_39585	insulator	insulator
Ge1K1Pr1Se4_ICSD_409812	insulator	insulator
Ge1K1S4Tb1_ICSD_409811	insulator	insulator
Ge1K1S4Y1_ICSD_603205	insulator	insulator
Ge1K1_ICSD_43515	insulator	insulator
Ge1K2Se4_ICSD_78828	insulator	insulator
Ge1K2Te4_ICSD_38341	insulator	insulator
Ge1K4O4_ICSD_37271	insulator	insulator
Ge1K4S4_ICSD_418257	insulator	insulator
Ge1K4Se4_ICSD_418258	insulator	insulator
Ge1La1Li2_ICSD_636809	metal	metal
Ge1La1Mn1_ICSD_80007	metal	metal
Ge1La1Mn1_ICSD_85857	metal	metal
Ge1La1Pd2_ICSD_189328	metal	metal
Ge1La1Pt1_ICSD_636837	metal	metal
Ge1La1Ru1_ICSD_85862	metal	metal
Ge1La1Ti1_ICSD_107617	metal	metal
Ge1La1_ICSD_108512	metal	metal
Ge1La1_ICSD_413736	metal	metal
Ge1La2Mg1O6_ICSD_97016	insulator	insulator
Ge1La2O5_ICSD_59727	insulator	insulator
Ge1La3_ICSD_603211	metal	metal
Ge1Li1Mg2_ICSD_53671	metal	metal
Ge1Li1Nd1O4_ICSD_200774	insulator	insulator
Ge1Li1Nd1_ICSD_76289	metal	metal
Ge1Li1Ni2_ICSD_53673	metal	metal
Ge1Li1O4Sc1_ICSD_62481	insulator	insulator
Ge1Li1O5Ta1_ICSD_39211	insulator	insulator
Ge1Li1Pd2_ICSD_42130	metal	metal
Ge1Li1Pr1_ICSD_59177	metal	metal
Ge1Li1Rh2_ICSD_417739	metal	metal
Ge1Li1Tb1_ICSD_601256	metal	metal
Ge1Li1Te2_ICSD_35676	insulator	insulator
Ge1Li1Y1_ICSD_32029	metal	metal
Ge1Li1Yb1_ICSD_59178	metal	metal
Ge1Li1Zn1_ICSD_171496	metal	metal
Ge1Li1_ICSD_42062	metal	metal
Ge1Li2Nd1_ICSD_636889	metal	metal
Ge1Li2O3_ICSD_100403	insulator	insulator
Ge1Li2O4Rb2_ICSD_61087	insulator	insulator
Ge1Li2O5Ti1_ICSD_250297	insulator	insulator
Ge1Li2O5V1_ICSD_86777	insulator	insulator
Ge1Li2Pb1S4_ICSD_281011	insulator	insulator
Ge1Li2Pd1_ICSD_53674	metal	metal
Ge1Li2Pr1_ICSD_636899	metal	metal
Ge1Li2Sn1_ICSD_636906	metal	metal
Ge1Li2Zn1_ICSD_171498	insulator	metal

Supplementary Table 412. Five-fold cross validated predictions for the metal/insulator classification (404/598).

system	calculated	predicted
Ge1Li2Zn1_ICSD_53677	metal	insulator
Ge1Li2Zn1_ICSD_53678	metal	metal
Ge1Li4S4_ICSD_95649	insulator	insulator
Ge1Li8O6_ICSD_65175	insulator	insulator
Ge1Lu1Na1O4_ICSD_54196	insulator	insulator
Ge1Lu1Ni1_ICSD_88247	metal	metal
Ge1Lu1Rh1_ICSD_90195	metal	metal
Ge1Lu1Ru1_ICSD_90194	metal	metal
Ge1Lu1Ti1_ICSD_88209	metal	metal
Ge1Mg1Mn1_ICSD_66948	metal	metal
Ge1Mg1N2_ICSD_23502	insulator	insulator
Ge1Mg1N4Sr3_ICSD_97382	insulator	insulator
Ge1Mg1O3_ICSD_160840	insulator	insulator
Ge1Mg1O3_ICSD_171787	insulator	insulator
Ge1Mg1O3_ICSD_35533	insulator	insulator
Ge1Mg1O3_ICSD_35534	insulator	insulator
Ge1Mg1P2_ICSD_182369	insulator	insulator
Ge1Mg1Sr1_ICSD_42458	metal	metal
Ge1Mg1Yb1_ICSD_57079	metal	metal
Ge1Mg2O4_ICSD_1086	insulator	insulator
Ge1Mg2O4_ICSD_63533	insulator	insulator
Ge1Mg2O4_ICSD_86505	insulator	insulator
Ge1Mg2S4_ICSD_23525	insulator	insulator
Ge1Mg2_ICSD_181764	metal	metal
Ge1Mg2_ICSD_52283	insulator	metal
Ge1Mg2_ICSD_636942	metal	metal
Ge1Mn1N2_ICSD_23528	insulator	insulator
Ge1Mn1Nb1_ICSD_42911	metal	metal
Ge1Mn1Nb1_ICSD_637001	metal	metal
Ge1Mn1Nd1_ICSD_80014	metal	metal
Ge1Mn1Ni1_ICSD_161715	metal	metal
Ge1Mn1Ni1_ICSD_637010	metal	metal
Ge1Mn1Ni1_ICSD_637011	metal	metal
Ge1Mn1Ni1_ICSD_637029	metal	metal
Ge1Mn1Ni2_ICSD_53687	metal	metal
Ge1Mn1O3_ICSD_69591	insulator	insulator
Ge1Mn1O4Zn1_ICSD_166628	insulator	insulator
Ge1Mn1O5Pr1_ICSD_80421	insulator	insulator
Ge1Mn1Pd1_ICSD_41156	metal	metal
Ge1Mn1Pd2_ICSD_53705	metal	metal
Ge1Mn1Pr1_ICSD_80011	metal	metal
Ge1Mn1Pr1_ICSD_85859	metal	metal
Ge1Mn1Rh1_ICSD_41155	metal	metal
Ge1Mn1Rh1_ICSD_637069	metal	metal
Ge1Mn1Rh2_ICSD_637070	metal	metal
Ge1Mn1Sc1_ICSD_600156	metal	metal
Ge1Mn1Ta1_ICSD_637098	metal	metal
Ge1Mn1Tb1_ICSD_82568	metal	metal
Ge1Mn1Tb1_ICSD_84094	metal	metal
Ge1Mn1Te4Tl2_ICSD_172506	insulator	insulator

Supplementary Table 413. Five-fold cross validated predictions for the metal/insulator classification (405/598).

system	calculated	predicted
Ge1Mn1Ti2_ICSD_189706	metal	metal
Ge1Mn1Tm1_ICSD_97792	metal	metal
Ge1Mn1Y1_ICSD_97806	metal	metal
Ge1Mn1Yb1_ICSD_95003	metal	metal
Ge1Mn1Yb1_ICSD_95004	metal	metal
Ge1Mn1Zr1_ICSD_637130	metal	metal
Ge1Mn1_ICSD_187195	metal	metal
Ge1Mn2O4_ICSD_22046	insulator	insulator
Ge1Mn2O4_ICSD_23587	insulator	insulator
Ge1Mn2O4_ICSD_6312	insulator	insulator
Ge1Mn2S4_ICSD_24142	metal	insulator
Ge1Mn2S4_ICSD_656252	insulator	insulator
Ge1Mn2Se4_ICSD_391296	insulator	insulator
Ge1Mn2Te4_ICSD_165681	metal	metal
Ge1Mn2_ICSD_184947	insulator	metal
Ge1Mn2_ICSD_52020	metal	metal
Ge1Mn3_ICSD_603343	metal	metal
Ge1Mn3_ICSD_97743	metal	metal
Ge1Mo3_ICSD_637137	metal	metal
Ge1N1V3_ICSD_637164	metal	metal
Ge1N2O1Si1_ICSD_169122	insulator	insulator
Ge1N2O1Si1_ICSD_169123	insulator	insulator
Ge1N2Sr2_ICSD_153302	metal	insulator
Ge1N2Sr2_ICSD_82534	metal	insulator
Ge1N2Zn1_ICSD_656277	insulator	insulator
Ge1Na1Nb1O5_ICSD_39209	insulator	insulator
Ge1Na1O4Tb1_ICSD_54197	insulator	insulator
Ge1Na1O4Tm1_ICSD_54198	insulator	insulator
Ge1Na1O4Y1_ICSD_28213	insulator	insulator
Ge1Na1O4Y1_ICSD_85497	insulator	insulator
Ge1Na1O4Yb1_ICSD_54195	insulator	insulator
Ge1Na1O5Sb1_ICSD_39646	insulator	insulator
Ge1Na1O5Ta1_ICSD_39210	insulator	insulator
Ge1Na1_ICSD_43275	insulator	metal
Ge1Na2O3_ICSD_1622	insulator	insulator
Ge1Na2O5Ti1_ICSD_160149	insulator	insulator
Ge1Na2O5Ti1_ICSD_20129	insulator	insulator
Ge1Na2O6Te1_ICSD_88789	insulator	insulator
Ge1Na2S3_ICSD_23455	insulator	insulator
Ge1Na2Se3_ICSD_49003	insulator	insulator
Ge1Na2Zn1_ICSD_240728	metal	metal
Ge1Na4O4_ICSD_61496	insulator	insulator
Ge1Nb1Ni1_ICSD_637228	metal	metal
Ge1Nb1Pt1_ICSD_186940	metal	metal
Ge1Nb1Rh1_ICSD_637230	metal	metal
Ge1Nb1Sb1_ICSD_637231	metal	metal
Ge1Nb3Te6_ICSD_72208	metal	metal
Ge1Nb3_ICSD_108521	metal	metal
Ge1Nb3_ICSD_637198	metal	metal
Ge1Nd1Rh1_ICSD_82548	metal	metal

Supplementary Table 414. Five-fold cross validated predictions for the metal/insulator classification (406/598).

system	calculated	predicted
Ge1Nd1Ru1_ICSD_602385	metal	metal
Ge1Nd1Sc1_ICSD_152903	metal	metal
Ge1Nd1Ti1_ICSD_93640	metal	metal
Ge1Nd1_ICSD_637286	metal	metal
Ge1Ni1Pt2_ICSD_87348	metal	metal
Ge1Ni1Sc1_ICSD_86365	metal	metal
Ge1Ni1Ta1_ICSD_53861	metal	metal
Ge1Ni1Tb1_ICSD_57101	metal	metal
Ge1Ni1Ti1_ICSD_53862	metal	metal
Ge1Ni1U1_ICSD_603104	metal	metal
Ge1Ni1V1_ICSD_637435	metal	metal
Ge1Ni1Y1_ICSD_637440	metal	metal
Ge1Ni1Yb1_ICSD_637443	metal	metal
Ge1Ni1Zr1_ICSD_637451	metal	metal
Ge1Ni1_ICSD_52124	metal	metal
Ge1Ni2O4_ICSD_69508	insulator	insulator
Ge1Ni2Sr1_ICSD_419659	metal	metal
Ge1Ni2Zn1_ICSD_53865	metal	metal
Ge1Ni2Zn3_ICSD_52179	metal	metal
Ge1Ni2_ICSD_53743	metal	metal
Ge1Ni2_ICSD_637339	metal	metal
Ge1Ni3_ICSD_600112	metal	metal
Ge1O2_ICSD_158598	insulator	insulator
Ge1O2_ICSD_158600	insulator	insulator
Ge1O2_ICSD_23783	insulator	insulator
Ge1O2_ICSD_281600	insulator	insulator
Ge1O2_ICSD_53869	insulator	insulator
Ge1O2_ICSD_59626	insulator	insulator
Ge1O2_ICSD_66592	insulator	insulator
Ge1O2_ICSD_94241	insulator	insulator
Ge1O3Pb1_ICSD_172521	insulator	insulator
Ge1O3Pb1_ICSD_185694	metal	insulator
Ge1O3Sr1_ICSD_28603	insulator	insulator
Ge1O3Zn1_ICSD_183725	insulator	insulator
Ge1O3Zn1_ICSD_33722	insulator	insulator
Ge1O4Sr2_ICSD_56382	insulator	insulator
Ge1O4Th1_ICSD_156945	insulator	insulator
Ge1O4Th1_ICSD_202081	insulator	insulator
Ge1O4U1_ICSD_16639	insulator	insulator
Ge1O4Zr1_ICSD_29262	insulator	insulator
Ge1O5Pb3_ICSD_100275	insulator	insulator
Ge1O5Pb3_ICSD_200517	insulator	insulator
Ge1O5Sr1Ti1_ICSD_158144	insulator	insulator
Ge1O6Se2_ICSD_422884	insulator	insulator
Ge1O6Sr1Te1_ICSD_88790	insulator	insulator
Ge1O6Te2_ICSD_260278	insulator	insulator
Ge1O7P2_ICSD_74876	insulator	insulator
Ge1O8P2Sr1_ICSD_423041	insulator	insulator
Ge1O8P2Sr1_ICSD_423042	insulator	insulator
Ge1O8Zr3_ICSD_29263	insulator	insulator

Supplementary Table 415. Five-fold cross validated predictions for the metal/insulator classification (407/598).

system	calculated	predicted
Ge1Os1Sc1_ICSD_637470	metal	metal
Ge1Os1Zr1_ICSD_637491	metal	metal
Ge1P1_ICSD_17032	metal	metal
Ge1P1_ICSD_53874	metal	metal
Ge1P1_ICSD_637492	insulator	insulator
Ge1P2Zn1_ICSD_637513	insulator	insulator
Ge1P3_ICSD_16294	metal	insulator
Ge1P4Sr4_ICSD_32558	insulator	insulator
Ge1Pb1S3_ICSD_2090	insulator	insulator
Ge1Pb2S4_ICSD_16272	insulator	insulator
Ge1Pd1S3_ICSD_408505	insulator	insulator
Ge1Pd1Sc1_ICSD_637547	metal	metal
Ge1Pd1Ti1_ICSD_409925	metal	metal
Ge1Pd1Zr1_ICSD_637568	metal	metal
Ge1Pd1_ICSD_659798	metal	metal
Ge1Pd2Tb1_ICSD_637553	metal	metal
Ge1Pd2Y1_ICSD_637566	metal	metal
Ge1Pd2_ICSD_76140	metal	metal
Ge1Pd5_ICSD_637537	metal	metal
Ge1Pr1Ru1_ICSD_602393	metal	metal
Ge1Pr1Sc1_ICSD_159197	metal	metal
Ge1Pr1Ti1_ICSD_93637	metal	metal
Ge1Pr1_ICSD_42376	metal	metal
Ge1Pr1_ICSD_42377	metal	metal
Ge1Pr1_ICSD_42379	metal	metal
Ge1Pr2Rh3_ICSD_637614	metal	metal
Ge1Pt1S1_ICSD_637646	insulator	insulator
Ge1Pt1Sc1_ICSD_637648	metal	metal
Ge1Pt1Se1_ICSD_637651	insulator	insulator
Ge1Pt1Sr1_ICSD_57115	metal	metal
Ge1Pt1Tb1_ICSD_90293	metal	metal
Ge1Pt1Ti1_ICSD_188965	insulator	metal
Ge1Pt1U1_ICSD_246606	metal	metal
Ge1Pt1U1_ICSD_92071	metal	metal
Ge1Pt1Y1_ICSD_637661	metal	metal
Ge1Pt1Yb1_ICSD_419135	metal	metal
Ge1Pt1Zr1_ICSD_637663	metal	metal
Ge1Pt1_ICSD_637633	metal	metal
Ge1Pt2_ICSD_76141	metal	metal
Ge1Pt3_ICSD_57114	metal	metal
Ge1Pt3_ICSD_77962	metal	metal
Ge1Pu3_ICSD_157517	metal	metal
Ge1Rb1S5Ta1_ICSD_417874	insulator	insulator
Ge1Rb1_ICSD_43516	insulator	insulator
Ge1Rh1Sc1_ICSD_637681	metal	metal
Ge1Rh1Sc1_ICSD_637682	metal	metal
Ge1Rh1Ta1_ICSD_421092	metal	metal
Ge1Rh1Tb1_ICSD_85993	metal	metal
Ge1Rh1Te1_ICSD_260373	insulator	metal
Ge1Rh1Tm1_ICSD_85997	metal	metal

Supplementary Table 416. Five-fold cross validated predictions for the metal/insulator classification (408/598).

system	calculated	predicted
Ge1Rh1U1_ICSD_637720	metal	metal
Ge1Rh1Y1_ICSD_637723	metal	metal
Ge1Rh1Yb1_ICSD_76355	metal	metal
Ge1Rh1Zr1_ICSD_637737	metal	metal
Ge1Rh1_ICSD_53890	metal	metal
Ge1Rh1_ICSD_637675	metal	metal
Ge1Rh2_ICSD_637674	metal	metal
Ge1Rh3Y2_ICSD_76345	metal	metal
Ge1Ru1Sc1_ICSD_79595	metal	metal
Ge1Ru1Tb1_ICSD_85912	metal	metal
Ge1Ru1Tm1_ICSD_85941	metal	metal
Ge1Ru1_ICSD_637744	insulator	metal
Ge1Ru3U2_ICSD_658172	metal	metal
Ge1S1Th1_ICSD_15269	metal	metal
Ge1S1U1_ICSD_87315	metal	metal
Ge1S1Zr1_ICSD_15573	metal	metal
Ge1S1_ICSD_1256	metal	metal
Ge1S1_ICSD_155418	insulator	insulator
Ge1S1_ICSD_38165	metal	insulator
Ge1S1_ICSD_637785	insulator	insulator
Ge1S2_ICSD_167194	insulator	insulator
Ge1S2_ICSD_31685	metal	insulator
Ge1S2_ICSD_44	insulator	insulator
Ge1S2_ICSD_85527	insulator	insulator
Ge1S3Sn1_ICSD_637796	insulator	insulator
Ge1S4Sr2_ICSD_637797	insulator	insulator
Ge1S4Tl4_ICSD_100161	insulator	insulator
Ge1S4Yb2_ICSD_637815	insulator	metal
Ge1S8V4_ICSD_50502	metal	metal
Ge1Sb1Ti1_ICSD_260557	metal	metal
Ge1Sb1Zr1_ICSD_85000	metal	metal
Ge1Sb1_ICSD_42472	metal	metal
Ge1Sb4Te7_ICSD_42875	insulator	insulator
Ge1Sc1Tb1_ICSD_159190	metal	metal
Ge1Sc1_ICSD_169409	metal	metal
Ge1Sc1_ICSD_637831	metal	metal
Ge1Se1Th1_ICSD_637884	metal	metal
Ge1Se1U1_ICSD_26276	metal	metal
Ge1Se1Zr1_ICSD_15574	metal	metal
Ge1Se1_ICSD_41738	metal	insulator
Ge1Se1_ICSD_53906	insulator	insulator
Ge1Se1_ICSD_637854	insulator	insulator
Ge1Se2_ICSD_50761	insulator	insulator
Ge1Se4Sr2_ICSD_413023	insulator	insulator
Ge1Se4Yb2_ICSD_637890	insulator	insulator
Ge1Se8V4_ICSD_50503	metal	metal
Ge1Sn1Tb1_ICSD_261752	metal	metal
Ge1Sr1_ICSD_52703	metal	metal
Ge1Sr2_ICSD_53923	insulator	metal
Ge1Ta3_ICSD_108870	metal	metal

Supplementary Table 417. Five-fold cross validated predictions for the metal/insulator classification (409/598).

system	calculated	predicted
Ge1Ta3_ICSD_56027	metal	metal
Ge1Tb1Ti1_ICSD_93642	metal	metal
Ge1Tb1_ICSD_637999	metal	metal
Ge1Te1Th1_ICSD_638015	metal	metal
Ge1Te1U1_ICSD_87317	metal	metal
Ge1Te1Zr1_ICSD_15575	metal	metal
Ge1Te1_ICSD_188458	insulator	insulator
Ge1Te1_ICSD_56042	insulator	insulator
Ge1Te1_ICSD_56604	insulator	insulator
Ge1Te1_ICSD_638005	insulator	insulator
Ge1Te1_ICSD_638009	insulator	insulator
Ge1Te4Zr1_ICSD_240906	insulator	insulator
Ge1Te5Tl2_ICSD_69035	metal	insulator
Ge1Te6Ti1_ICSD_73014	insulator	insulator
Ge1Th1_ICSD_44502	metal	metal
Ge1Th2_ICSD_56045	metal	metal
Ge1Ti1Tm1_ICSD_85910	metal	metal
Ge1Ti1Y1_ICSD_85904	metal	metal
Ge1Ti1Y1_ICSD_90234	metal	metal
Ge1Ti2_ICSD_189708	metal	metal
Ge1Tm1_ICSD_638068	metal	metal
Ge1V1Zr1_ICSD_88210	metal	metal
Ge1V3_ICSD_186004	metal	metal
Ge1Y1Zn1_ICSD_163345	metal	metal
Ge1Y1_ICSD_638131	metal	metal
Ge1Yb2_ICSD_96115	metal	metal
Ge1Zr1_ICSD_638161	metal	metal
Ge1_ICSD_167204	metal	metal
Ge1_ICSD_173520	insulator	metal
Ge1_ICSD_173890	metal	metal
Ge1_ICSD_181070	metal	metal
Ge1_ICSD_181072	metal	metal
Ge1_ICSD_181073	metal	metal
Ge1_ICSD_189803	metal	metal
Ge1_ICSD_189805	metal	metal
Ge1_ICSD_245956	metal	metal
Ge1_ICSD_246628	metal	metal
Ge1_ICSD_53642	metal	metal
Ge1_ICSD_636530	metal	metal
Ge1_ICSD_636533	metal	metal
Ge2Hf1Rh1_ICSD_99176	metal	metal
Ge2Hf1_ICSD_16698	metal	metal
Ge2Hf3_ICSD_636549	metal	metal
Ge2Hg3K2S8_ICSD_281506	insulator	insulator
Ge2Ho1Ir1_ICSD_93337	metal	metal
Ge2Ho1Mn2_ICSD_636635	metal	metal
Ge2Ho1Ni1_ICSD_658574	metal	metal
Ge2Ho1Ni2_ICSD_636645	metal	metal
Ge2Ho1Pd2_ICSD_99112	metal	metal
Ge2Ho1Pt1_ICSD_88227	metal	metal

Supplementary Table 418. Five-fold cross validated predictions for the metal/insulator classification (410/598).

system	calculated	predicted
Ge2Ho1Rh2_ICSD_636665	metal	metal
Ge2Ho1Ru2_ICSD_53648	metal	metal
Ge2Ho2In1_ICSD_172493	metal	metal
Ge2Ho2Mg1_ICSD_423453	metal	metal
Ge2Ho2Os1_ICSD_76350	metal	metal
Ge2Ho2Rh1_ICSD_88198	metal	metal
Ge2I2La2_ICSD_59801	metal	metal
Ge2In1La2_ICSD_87511	metal	metal
Ge2In1Li1Sr2_ICSD_280691	metal	metal
Ge2In1Li1Yb2_ICSD_174355	insulator	metal
Ge2In1Pr2_ICSD_414072	metal	metal
Ge2In1Tb2_ICSD_172491	metal	metal
Ge2In1Yb2_ICSD_172494	metal	metal
Ge2In2O7_ICSD_20053	insulator	insulator
Ge2In2O7_ICSD_74896	insulator	insulator
Ge2In6O9Pt1_ICSD_170897	insulator	insulator
Ge2Ir1La1_ICSD_636702	metal	metal
Ge2Ir1Tb1_ICSD_93336	metal	metal
Ge2Ir1Y1_ICSD_76509	metal	metal
Ge2Ir1Yb2_ICSD_412707	metal	metal
Ge2Ir2La1_ICSD_636699	metal	metal
Ge2Ir2Nd1_ICSD_636719	metal	metal
Ge2Ir2Pr1_ICSD_180145	metal	metal
Ge2Ir2Sr1_ICSD_165737	metal	metal
Ge2Ir2Tb1_ICSD_636742	metal	metal
Ge2Ir2Th1_ICSD_636745	metal	metal
Ge2Ir2U1_ICSD_53660	metal	metal
Ge2Ir2U1_ICSD_636759	metal	metal
Ge2Ir3La1_ICSD_424816	metal	metal
Ge2K2O6Zn1_ICSD_65740	insulator	insulator
Ge2K2O7Pb2_ICSD_30247	insulator	insulator
Ge2K2O7Zr1_ICSD_88843	insulator	insulator
Ge2K2Pb1S6_ICSD_170601	insulator	insulator
Ge2K4Se6_ICSD_300233	insulator	insulator
Ge2K6S6_ICSD_47111	insulator	insulator
Ge2K6Se6_ICSD_47112	insulator	insulator
Ge2K6Te6_ICSD_10108	insulator	insulator
Ge2La1Li1_ICSD_601105	metal	metal
Ge2La1Mn2_ICSD_636812	metal	metal
Ge2La1Mn2_ICSD_636814	metal	metal
Ge2La1Ni1_ICSD_85600	metal	metal
Ge2La1Ni2_ICSD_636821	metal	metal
Ge2La1Pd1_ICSD_636826	metal	metal
Ge2La1Pd2_ICSD_53662	metal	metal
Ge2La1Pt1_ICSD_636838	metal	metal
Ge2La1Pt2_ICSD_416421	metal	metal
Ge2La1Pt2_ICSD_416423	metal	metal
Ge2La1Pt2_ICSD_53665	metal	metal
Ge2La1Pt2_ICSD_659355	metal	metal
Ge2La1Rh1_ICSD_636850	metal	metal

Supplementary Table 419. Five-fold cross validated predictions for the metal/insulator classification (411/598).

system	calculated	predicted
Ge2La1Rh2_ICSD_636847	metal	metal
Ge2La1Ru2_ICSD_81758	metal	metal
Ge2La1_ICSD_636796	metal	metal
Ge2La1_ICSD_636807	metal	metal
Ge2La2O7_ICSD_202606	insulator	insulator
Ge2La3Ni1_ICSD_20520	metal	metal
Ge2La3Ru2_ICSD_636861	metal	metal
Ge2La5Si1_ICSD_636877	metal	metal
Ge2Li1N3_ICSD_636887	insulator	insulator
Ge2Li1Nd1_ICSD_601107	metal	metal
Ge2Li1Pr1_ICSD_601106	metal	metal
Ge2Li1Sr1_ICSD_162582	metal	metal
Ge2Li1Yb1_ICSD_409768	metal	metal
Ge2Li2O5_ICSD_28178	insulator	insulator
Ge2Li4N6Sr3_ICSD_96422	insulator	insulator
Ge2Li4Zr1_ICSD_99161	metal	metal
Ge2Li7_ICSD_42063	metal	metal
Ge2Lu1Ni2_ICSD_106852	metal	metal
Ge2Lu2O7_ICSD_39929	insulator	insulator
Ge2Mg1Nd2_ICSD_423449	metal	metal
Ge2Mg1O7Sr2_ICSD_420522	insulator	insulator
Ge2Mg1Pr2_ICSD_413850	metal	metal
Ge2Mg1Tb2_ICSD_423450	metal	metal
Ge2Mg1Tm2_ICSD_423454	metal	metal
Ge2Mg1Y2_ICSD_423457	metal	metal
Ge2Mn1O7Sr2_ICSD_84033	insulator	insulator
Ge2Mn1Pd3_ICSD_637060	metal	metal
Ge2Mn2Sr1_ICSD_403	metal	metal
Ge2Mn2Tb1_ICSD_107109	metal	metal
Ge2Mn2Th1_ICSD_55340	metal	metal
Ge2Mn2Y1_ICSD_86266	metal	metal
Ge2Mn2Yb1_ICSD_90443	metal	metal
Ge2Mn5_ICSD_40462	metal	metal
Ge2Mo1_ICSD_16822	metal	metal
Ge2Mo1_ICSD_637139	metal	metal
Ge2N2O1_ICSD_200842	insulator	insulator
Ge2N2Sr3_ICSD_82533	metal	metal
Ge2N3Na1_ICSD_47100	insulator	insulator
Ge2N4Si1_ICSD_183103	insulator	insulator
Ge2N6Sr5_ICSD_419164	insulator	insulator
Ge2Na1O6Sc1_ICSD_22503	insulator	insulator
Ge2Na1O6V1_ICSD_99742	metal	insulator
Ge2Na2O7Zr1_ICSD_20402	insulator	insulator
Ge2Na2Se5_ICSD_40453	insulator	insulator
Ge2Na6S7_ICSD_25388	insulator	insulator
Ge2Na6Se6_ICSD_61400	insulator	insulator
Ge2Na6Te6_ICSD_47113	insulator	insulator
Ge2Nb1_ICSD_637208	metal	metal
Ge2Nd1Ni1_ICSD_57094	metal	metal
Ge2Nd1Ni1_ICSD_637291	metal	metal

Supplementary Table 420. Five-fold cross validated predictions for the metal/insulator classification (412/598).

system	calculated	predicted
Ge2Nd1Ni2_ICSD_53728	metal	metal
Ge2Nd1Pd1_ICSD_637300	metal	metal
Ge2Nd1Pd2_ICSD_247283	metal	metal
Ge2Nd1Pt2_ICSD_637303	metal	metal
Ge2Nd1Pt2_ICSD_637305	metal	metal
Ge2Nd1Rh1_ICSD_637311	metal	metal
Ge2Nd1Rh2_ICSD_637310	metal	metal
Ge2Nd1Ru2_ICSD_53739	metal	metal
Ge2Nd1_ICSD_637255	metal	metal
Ge2Nd2Zn15_ICSD_94220	metal	metal
Ge2Ni1Pr1_ICSD_637376	metal	metal
Ge2Ni1Tb1_ICSD_657199	metal	metal
Ge2Ni1Tb3_ICSD_185872	metal	metal
Ge2Ni1Y1_ICSD_637438	metal	metal
Ge2Ni1_ICSD_90341	metal	metal
Ge2Ni2Pr1_ICSD_167938	metal	metal
Ge2Ni2Pr1_ICSD_93413	metal	metal
Ge2Ni2Sr1_ICSD_637397	metal	metal
Ge2Ni2Tb1_ICSD_150833	metal	metal
Ge2Ni2Th1_ICSD_55343	metal	metal
Ge2Ni2U1_ICSD_637428	metal	metal
Ge2Ni2Y1_ICSD_62933	metal	metal
Ge2Ni2Yb1_ICSD_637442	metal	metal
Ge2Ni3Sr1_ICSD_419660	metal	metal
Ge2Ni5_ICSD_637367	metal	metal
Ge2Ni7Zn6_ICSD_52178	metal	metal
Ge2O7Sr2Zn1_ICSD_420521	insulator	insulator
Ge2O7Yb2_ICSD_65618	insulator	metal
Ge2Os1_ICSD_43690	insulator	metal
Ge2Os2Th1_ICSD_637475	metal	metal
Ge2P2Rb6Se14_ICSD_261951	insulator	insulator
Ge2P4Sr3_ICSD_41182	insulator	insulator
Ge2Pd1Tb1_ICSD_106940	metal	metal
Ge2Pd2Sr1_ICSD_165985	metal	metal
Ge2Pd2Tb1_ICSD_53883	metal	metal
Ge2Pd2Th1_ICSD_53884	metal	metal
Ge2Pd2U1_ICSD_53885	metal	metal
Ge2Pd2Yb1_ICSD_637567	metal	metal
Ge2Pr1Rh2_ICSD_167942	metal	metal
Ge2Pr1Rh2_ICSD_167944	metal	metal
Ge2Pr1Ru2_ICSD_167945	metal	metal
Ge2Pr1_ICSD_637607	metal	metal
Ge2Pr1_ICSD_658011	metal	metal
Ge2Pt1Tb1_ICSD_95142	metal	metal
Ge2Pt1Y1_ICSD_601773	metal	metal
Ge2Pt1_ICSD_637638	metal	metal
Ge2Pt2Tb1_ICSD_637655	metal	metal
Ge2Pt2Th1_ICSD_53888	metal	metal
Ge2Pt2Th1_ICSD_637658	metal	metal
Ge2Pt2U1_ICSD_53889	metal	metal

Supplementary Table 421. Five-fold cross validated predictions for the metal/insulator classification (413/598).

system	calculated	predicted
Ge2Pt2U1_ICSD_657185	metal	metal
Ge2Pt2Y1_ICSD_637662	metal	metal
Ge2Rb4S6_ICSD_409729	insulator	insulator
Ge2Rb4Se6_ICSD_402719	insulator	insulator
Ge2Re1_ICSD_637671	metal	metal
Ge2Rh2Sr1_ICSD_77145	metal	metal
Ge2Rh2Tb1_ICSD_637700	metal	metal
Ge2Rh2Th1_ICSD_53895	metal	metal
Ge2Rh2U1_ICSD_53896	metal	metal
Ge2Rh2U1_ICSD_603847	metal	metal
Ge2Rh2U1_ICSD_637716	metal	metal
Ge2Rh2Y1_ICSD_637725	metal	metal
Ge2Rh2Yb1_ICSD_637734	metal	metal
Ge2Ru1Tb2_ICSD_91969	metal	metal
Ge2Ru2Sr1_ICSD_77144	metal	metal
Ge2Ru2Tb1_ICSD_53897	metal	metal
Ge2Ru2Tb1_ICSD_637754	metal	metal
Ge2Ru2Th1_ICSD_637761	metal	metal
Ge2Ru2Y1_ICSD_52766	metal	metal
Ge2Ru2Yb1_ICSD_637774	metal	metal
Ge2S6Tl4_ICSD_2240	insulator	insulator
Ge2Sb2Te5_ICSD_42876	insulator	insulator
Ge2Sc1_ICSD_637839	metal	metal
Ge2Se6Tl4_ICSD_35043	insulator	insulator
Ge2Sr1Zn2_ICSD_411	metal	metal
Ge2Sr1_ICSD_10000	metal	metal
Ge2Sr1_ICSD_152799	insulator	metal
Ge2Ta1_ICSD_637963	metal	metal
Ge2Tb1_ICSD_638002	metal	metal
Ge2Te6Tl6_ICSD_49658	insulator	insulator
Ge2Th1_ICSD_44500	metal	metal
Ge2Th1_ICSD_56046	metal	metal
Ge2Th1_ICSD_638030	metal	metal
Ge2Th3_ICSD_638039	metal	metal
Ge2Ti1_ICSD_638053	metal	metal
Ge2Tm1_ICSD_638067	metal	metal
Ge2U1_ICSD_56049	metal	metal
Ge2U1_ICSD_82702	metal	metal
Ge2V1_ICSD_638088	metal	metal
Ge2W1_ICSD_638115	metal	metal
Ge2W1_ICSD_9978	metal	metal
Ge2Y1_ICSD_76344	metal	metal
Ge2Zr1_ICSD_638152	metal	metal
Ge30In16K8_ICSD_636686	metal	metal
Ge38I8P8_ICSD_16533	insulator	insulator
Ge3Hf5_ICSD_44361	metal	metal
Ge3Ho2Pt9_ICSD_415205	metal	metal
Ge3Ho3Ir2_ICSD_82875	metal	metal
Ge3Ho3Ru2_ICSD_425773	metal	metal
Ge3Ho5_ICSD_636616	metal	metal

Supplementary Table 422. Five-fold cross validated predictions for the metal/insulator classification (414/598).

system	calculated	predicted
Ge3I5La6_ICSD_414175	metal	metal
Ge3Ir1La1_ICSD_636700	metal	metal
Ge3Ir1Sr1_ICSD_168861	metal	metal
Ge3Ir2S3_ICSD_636729	metal	metal
Ge3Ir2Se3_ICSD_636733	metal	metal
Ge3Ir2Tb3_ICSD_425777	metal	metal
Ge3K1O9Ta1_ICSD_10380	insulator	insulator
Ge3La1Os1_ICSD_636822	metal	metal
Ge3La1Rh1_ICSD_636848	metal	metal
Ge3La1Ru1_ICSD_636856	metal	metal
Ge3La1Ti1_ICSD_154047	metal	metal
Ge3La1V1_ICSD_164891	metal	metal
Ge3La1_ICSD_261501	metal	metal
Ge3La2Li2_ICSD_262431	metal	metal
Ge3La2O9_ICSD_83341	insulator	insulator
Ge3La2Zn6_ICSD_98232	metal	metal
Ge3La4S12_ICSD_2046	insulator	insulator
Ge3La4_ICSD_76313	metal	metal
Ge3La5_ICSD_636790	metal	metal
Ge3Li2Lu3_ICSD_57074	metal	metal
Ge3Li2Ni1O8_ICSD_68339	insulator	insulator
Ge3Lu3Ni2_ICSD_88246	metal	metal
Ge3Lu3Ru2_ICSD_425776	metal	metal
Ge3Lu5_ICSD_601111	metal	metal
Ge3Mn5_ICSD_76138	metal	metal
Ge3Mo5_ICSD_637143	metal	metal
Ge3Mo5_ICSD_77281	metal	metal
Ge3N4_ICSD_156339	insulator	insulator
Ge3N4_ICSD_637162	insulator	insulator
Ge3N4_ICSD_658934	insulator	insulator
Ge3N4_ICSD_87767	insulator	insulator
Ge3N4_ICSD_97569	insulator	insulator
Ge3Na1P3_ICSD_184000	insulator	insulator
Ge3Nb1O9Rb1_ICSD_10382	insulator	insulator
Ge3Nb5_ICSD_2003	metal	metal
Ge3Nb5_ICSD_44741	metal	metal
Ge3Nd2Zn6_ICSD_98235	metal	metal
Ge3Ni5_ICSD_53746	metal	metal
Ge3O12Sb4_ICSD_421470	insulator	insulator
Ge3O12Sr3Y2_ICSD_80582	insulator	insulator
Ge3O25P6Si2_ICSD_202643	insulator	insulator
Ge3O8Zn2_ICSD_27103	insulator	insulator
Ge3O9Rb1Ta1_ICSD_10381	insulator	insulator
Ge3O9Sb2_ICSD_95735	insulator	insulator
Ge3O9Ta1Tl1_ICSD_10383	insulator	insulator
Ge3O9Ti1Tl2_ICSD_19029	insulator	insulator
Ge3Os2_ICSD_95593	insulator	metal
Ge3Pd1Sr1_ICSD_168862	metal	metal
Ge3Pr1Rh1_ICSD_160385	metal	metal
Ge3Pr1V1_ICSD_164893	metal	metal

Supplementary Table 423. Five-fold cross validated predictions for the metal/insulator classification (415/598).

system	calculated	predicted
Ge3Pr2Zn6_ICSD_98234	metal	metal
Ge3Pr4_ICSD_637571	metal	metal
Ge3Pt1Sr1_ICSD_168863	metal	metal
Ge3Pt2_ICSD_42912	metal	metal
Ge3Pt9Tb2_ICSD_415207	metal	metal
Ge3Pu1_ICSD_157520	metal	metal
Ge3Rh5_ICSD_53891	metal	metal
Ge3Ru2Tb3_ICSD_425771	metal	metal
Ge3Ru2_ICSD_95589	insulator	metal
Ge3Sb5Yb8_ICSD_170575	metal	metal
Ge3Sc5_ICSD_602017	metal	metal
Ge3Sr5_ICSD_409374	metal	metal
Ge3Ta5_ICSD_44744	metal	metal
Ge3Ta5_ICSD_637960	metal	metal
Ge3Tb5_ICSD_56034	metal	metal
Ge3Ti5_ICSD_638051	metal	metal
Ge3U1_ICSD_638072	metal	metal
Ge3V1_ICSD_638098	metal	metal
Ge3V5_ICSD_44504	metal	metal
Ge3V5_ICSD_638081	metal	metal
Ge3W5_ICSD_638119	metal	metal
Ge3Y5_ICSD_638128	metal	metal
Ge3Yb2Zn3_ICSD_152947	metal	metal
Ge3Yb5_ICSD_638147	metal	metal
Ge3Zr5_ICSD_76315	metal	metal
Ge40In6K8_ICSD_400448	metal	metal
Ge46K8_ICSD_26300	metal	metal
Ge4Hf2Nb3_ICSD_172870	metal	metal
Ge4Hf3Nb2_ICSD_172872	metal	metal
Ge4Hf3Ni4_ICSD_86690	metal	metal
Ge4Hf4Nb1_ICSD_172869	metal	metal
Ge4Hf5_ICSD_76306	metal	metal
Ge4Ho3Pd4_ICSD_96158	metal	metal
Ge4Ho3_ICSD_86103	metal	metal
Ge4Ho4In1Ni2_ICSD_240336	metal	metal
Ge4I4S6_ICSD_24371	insulator	insulator
Ge4In1Ni2Tm4_ICSD_240337	metal	metal
Ge4Ir1_ICSD_42773	metal	metal
Ge4Ir1_ICSD_636695	metal	metal
Ge4K4Se10_ICSD_300262	insulator	insulator
Ge4La1Ni9_ICSD_184406	metal	metal
Ge4La1Rh6_ICSD_426482	metal	metal
Ge4La3Li4_ICSD_262432	metal	metal
Ge4La5_ICSD_636782	metal	metal
Ge4Li15_ICSD_43235	metal	metal
Ge4Li4Yb5_ICSD_57076	metal	metal
Ge4Li9_ICSD_25308	metal	metal
Ge4Na1O12P3_ICSD_240922	insulator	insulator
Ge4Na4S10_ICSD_25382	insulator	insulator
Ge4Na4Se10_ICSD_300263	insulator	insulator

Supplementary Table 424. Five-fold cross validated predictions for the metal/insulator classification (416/598).

system	calculated	predicted
Ge4Na8Se10_ICSD_61401	insulator	insulator
Ge4Na8Te10_ICSD_37183	insulator	insulator
Ge4Nb3Zr2_ICSD_20809	metal	metal
Ge4Nb5_ICSD_172871	metal	metal
Ge4Ni4Sc3_ICSD_53857	metal	metal
Ge4Ni9Pr1_ICSD_600192	metal	metal
Ge4O9Pb1_ICSD_201282	insulator	insulator
Ge4Pd4Yb3_ICSD_411182	metal	metal
Ge4Pr1Rh6_ICSD_426483	metal	metal
Ge4Rh1_ICSD_637677	metal	metal
Ge4S10Tl4_ICSD_640	insulator	insulator
Ge4Se10Tl4_ICSD_26415	insulator	insulator
Ge4Y3_ICSD_88105	metal	metal
Ge4Zr5_ICSD_638154	metal	metal
Ge5Ho2Rh3_ICSD_63616	metal	metal
Ge5Ho3_ICSD_54493	metal	metal
Ge5Ir3La2_ICSD_86499	metal	metal
Ge5Ir3Y2_ICSD_86503	metal	metal
Ge5Ir4_ICSD_636696	metal	metal
Ge5La2Rh3_ICSD_86502	metal	metal
Ge5La2Ru3_ICSD_86500	metal	metal
Ge5Mg14O24_ICSD_9371	insulator	insulator
Ge5N2Sr6_ICSD_154397	metal	metal
Ge5Nd1_ICSD_246814	metal	metal
Ge5Nd1_ICSD_710017	metal	metal
Ge5Nd3_ICSD_53726	metal	metal
Ge5O25P6_ICSD_9212	insulator	insulator
Ge5Pr1_ICSD_246813	metal	metal
Ge5Tb1_ICSD_247400	metal	metal
Ge5Tb3_ICSD_419457	metal	metal
Ge5Ti6_ICSD_15304	metal	metal
Ge5Y3_ICSD_20612	metal	metal
Ge5Yb3_ICSD_600981	metal	metal
Ge5Yb3_ICSD_97355	metal	metal
Ge6Hf1Mn6_ICSD_155117	metal	metal
Ge6Ho1Mn6_ICSD_656761	metal	metal
Ge6Ho4Ir7_ICSD_636630	metal	metal
Ge6Ho4Rh7_ICSD_636663	metal	metal
Ge6Ir7Lu4_ICSD_636712	metal	metal
Ge6Ir7Mn4_ICSD_153071	metal	metal
Ge6Ir7Sc4_ICSD_636731	metal	metal
Ge6Ir7Tb4_ICSD_636740	metal	metal
Ge6Ir7Tm4_ICSD_636749	metal	metal
Ge6Ir7Y4_ICSD_636762	metal	metal
Ge6Ir7Yb4_ICSD_636768	metal	metal
Ge6La11Ni4_ICSD_62290	metal	metal
Ge6Li1Ni6_ICSD_41463	metal	metal
Ge6Li2Sr4_ICSD_414573	metal	metal
Ge6Li2Sr4_ICSD_414574	metal	metal
Ge6Li2Sr4_ICSD_414575	metal	metal

Supplementary Table 425. Five-fold cross validated predictions for the metal/insulator classification (417/598).

system	calculated	predicted
Ge6Lu4Os7_ICSD_636925	metal	metal
Ge6Lu4Rh7_ICSD_90200	metal	metal
Ge6Lu4Ru7_ICSD_90199	metal	metal
Ge6Mg1Ni6_ICSD_636950	metal	metal
Ge6Mn6Sc1_ICSD_637078	metal	metal
Ge6Mn6Sc1_ICSD_656754	metal	metal
Ge6Mn6Tb1_ICSD_656759	metal	metal
Ge6Mn6Y1_ICSD_656755	metal	metal
Ge6Mn6Zr1_ICSD_89433	metal	metal
Ge6Os7Sc4_ICSD_637471	metal	metal
Ge6Os7U4_ICSD_637483	metal	metal
Ge6Os7Yb4_ICSD_637489	metal	metal
Ge6Pt4Y3_ICSD_76293	metal	metal
Ge6Rh7Sc4_ICSD_84201	metal	metal
Ge6Rh7Yb4_ICSD_413983	metal	metal
Ge6Ru7Sc4_ICSD_637747	metal	metal
Ge6Ru7U4_ICSD_637767	metal	metal
Ge6Tc7U4_ICSD_80521	metal	metal
Ge7H8N2O15_ICSD_407222	insulator	insulator
Ge7Hf6Ni16_ICSD_109125	metal	metal
Ge7Ir3_ICSD_53656	metal	metal
Ge7Mg6Ni16_ICSD_53680	metal	metal
Ge7Mn6Ni16_ICSD_637032	metal	metal
Ge7Nb6Ni16_ICSD_109124	metal	metal
Ge7Ni16Sc6_ICSD_637387	metal	metal
Ge7Ni16Ta6_ICSD_637403	metal	metal
Ge7Ni16Ti6_ICSD_109123	metal	metal
Ge7Ni16Zr6_ICSD_637449	metal	metal
Ge7Re3_ICSD_84208	metal	metal
Ge8Li7Rb1_ICSD_411606	metal	metal
Ge8Na6Pt8_ICSD_32038	metal	metal
Ge8Yb3_ICSD_97356	metal	metal
Ge9Li4O20_ICSD_34361	insulator	insulator
Ge9Nd2_ICSD_263089	metal	metal
Ge9Pd25_ICSD_87204	metal	metal
H0.333Y1_ICSD_152860	metal	metal
H0.667Y1_ICSD_152861	metal	metal
H10In1N3O14_ICSD_417341	insulator	insulator
H10In2O17S3_ICSD_391355	insulator	insulator
H10Mn1N1Na1O10P2_ICSD_413407	insulator	insulator
H10Mn1N4O8S2_ICSD_249335	metal	insulator
H10N2O8P2_ICSD_14169	insulator	insulator
H10N3Nd1O11S3_ICSD_410898	insulator	insulator
H10N4O4S1_ICSD_24103	insulator	insulator
H10O10S1V1_ICSD_23308	metal	insulator
H10O8S1_ICSD_2783	insulator	insulator
H10O8Se1_ICSD_404659	insulator	insulator
H10O8Se1_ICSD_404660	metal	insulator
H11N2O4P1_ICSD_32572	insulator	insulator
H11Na2O8P1_ICSD_1602	insulator	insulator

Supplementary Table 426. Five-fold cross validated predictions for the metal/insulator classification (418/598).

system	calculated	predicted
H12I8Mg1O6_ICSD_32507	insulator	insulator
H12K2N6Sn1_ICSD_79985	insulator	insulator
H12Li3O10V1_ICSD_174238	insulator	insulator
H12Li4N4O12P4_ICSD_415548	insulator	insulator
H12Mg1N6O6_ICSD_61681	insulator	insulator
H12Mg1O12S2_ICSD_1834	metal	insulator
H12Mg1O9S1_ICSD_48112	insulator	insulator
H12Mg1O9S2_ICSD_35664	insulator	insulator
H12Mg1O9Se1_ICSD_48115	insulator	insulator
H12Mg1O9Te1_ICSD_48114	insulator	insulator
H12Mn1N2O10S2_ICSD_410200	insulator	insulator
H12Mn2N4O8Zn1_ICSD_162384	insulator	insulator
H12N10O8Pd1_ICSD_412607	insulator	insulator
H12N16Pd2_ICSD_410384	insulator	insulator
H12N2Ni1O10S2_ICSD_410198	insulator	insulator
H12N2O14U1_ICSD_23825	insulator	insulator
H12N3Nb1O8_ICSD_161455	insulator	insulator
H12N4O4P2_ICSD_6211	insulator	insulator
H12N6Na2Sn1_ICSD_83963	insulator	insulator
H12N6Ni1O4_ICSD_201209	insulator	insulator
H12Na2O9Si1_ICSD_9171	insulator	insulator
H12Ni1O9S1_ICSD_26149	insulator	insulator
H12O12S2Zn1_ICSD_1836	insulator	insulator
H12O18P4Sr2_ICSD_61237	insulator	insulator
H13Mg1O9P1_ICSD_74520	insulator	insulator
H14La2Ni10_ICSD_260376	metal	metal
H14N4O6Ru1S2_ICSD_40202	insulator	insulator
H14N4O8S2_ICSD_73623	insulator	insulator
H15Th4_ICSD_638495	metal	metal
H16Mg1N1O10P1_ICSD_29385	insulator	insulator
H16Mg1O10P2_ICSD_250187	insulator	insulator
H16Mg6Nb1_ICSD_169055	metal	metal
H16Mg6Ti1_ICSD_169053	metal	metal
H16Mg6V1_ICSD_169054	metal	metal
H16Mg7Nb1_ICSD_169052	metal	metal
H16Mg7Ti1_ICSD_169050	metal	metal
H16Mg7V1_ICSD_169051	metal	metal
H16N2O16P4Zn1_ICSD_59919	insulator	insulator
H16N4O12P4_ICSD_16148	insulator	insulator
H16Ni1O10P2_ICSD_281216	insulator	insulator
H16O20S3Y2_ICSD_281358	insulator	insulator
H1Hg1N1O4_ICSD_6186	insulator	insulator
H1Ho1O2_ICSD_2944	insulator	insulator
H1Ho1Se1_ICSD_78957	insulator	metal
H1I1Li2O1_ICSD_74930	insulator	insulator
H1I1O3_ICSD_415140	insulator	insulator
H1I1O3_ICSD_66643	insulator	insulator
H1I1O4Zn1_ICSD_185598	insulator	insulator
H1I1Sr1_ICSD_25546	insulator	insulator
H1In1O2_ICSD_166255	insulator	insulator

Supplementary Table 427. Five-fold cross validated predictions for the metal/insulator classification (419/598).

system	calculated	predicted
H1In1O2_ICSD_166257	insulator	insulator
H1In1O2_ICSD_24093	insulator	insulator
H1In1O4Se1_ICSD_415267	insulator	insulator
H1K1O1_ICSD_61047	insulator	insulator
H1K1O3Se1_ICSD_20862	insulator	insulator
H1K1O3Si1_ICSD_30892	insulator	insulator
H1K1O7Se2V1_ICSD_82535	insulator	insulator
H1K1O8P2Zn2_ICSD_412588	insulator	insulator
H1K1_ICSD_183287	insulator	insulator
H1K2N1O6S2_ICSD_15880	insulator	insulator
H1K3O8S2_ICSD_249551	insulator	insulator
H1K6Na15Tl18_ICSD_81301	metal	metal
H1La1N1O4_ICSD_413563	metal	insulator
H1La1Ni1Sn1_ICSD_157925	metal	metal
H1La1O1_ICSD_48122	insulator	insulator
H1La1O2_ICSD_60675	insulator	insulator
H1La2Li1O3_ICSD_56744	insulator	insulator
H1Li1Nd2O3_ICSD_56747	insulator	insulator
H1Li1O3P1Tl1_ICSD_201040	insulator	insulator
H1Li1O3Se1_ICSD_8255	insulator	insulator
H1Li1O4S1_ICSD_82373	insulator	insulator
H1Li1Pd1_ICSD_246613	metal	metal
H1Li1S1_ICSD_98019	insulator	insulator
H1Li1S1_ICSD_98020	insulator	insulator
H1Li1S1_ICSD_98021	insulator	insulator
H1Li1_ICSD_173192	insulator	insulator
H1Li2N1_ICSD_168791	insulator	insulator
H1Li2N1_ICSD_169858	insulator	insulator
H1Li2O8P2Sc1_ICSD_409955	insulator	insulator
H1Li3O4Te1_ICSD_38036	insulator	insulator
H1Li4N1_ICSD_409633	insulator	insulator
H1Mg1Na3O8P2_ICSD_81226	insulator	insulator
H1Mg1Ni1_ICSD_187257	metal	metal
H1Mg2O5P1_ICSD_40828	insulator	insulator
H1Mn1O2_ICSD_27457	insulator	insulator
H1Mn1O2_ICSD_84948	metal	insulator
H1Mn1O2_ICSD_84949	insulator	insulator
H1Mn1O7P2_ICSD_415152	insulator	insulator
H1Mn1_ICSD_108535	metal	metal
H1Mo1_ICSD_108537	metal	metal
H1N1O4Zn1_ICSD_415537	insulator	insulator
H1N1Sr1_ICSD_410656	insulator	insulator
H1N2O9P1Pb2_ICSD_260268	insulator	insulator
H1N3Si2_ICSD_202970	insulator	insulator
H1Na1O1_ICSD_41231	insulator	insulator
H1Na1O1_ICSD_61045	insulator	insulator
H1Na1O4S1_ICSD_1436	insulator	insulator
H1Na1O4Se1_ICSD_88893	insulator	insulator
H1Na1O4Si1Zn1_ICSD_88001	insulator	insulator
H1Na1O7P2Zn1_ICSD_39491	insulator	insulator

Supplementary Table 428. Five-fold cross validated predictions for the metal/insulator classification (420/598).

system	calculated	predicted
H1Na1O8P2Zn2_ICSD_73954	insulator	insulator
H1Na1_ICSD_261353	insulator	insulator
H1Na1_ICSD_261354	insulator	insulator
H1Na2O3P1_ICSD_155976	metal	insulator
H1Na2O5P1Zn1_ICSD_280153	insulator	insulator
H1Nd1Ni1Sn1_ICSD_157928	metal	metal
H1Nd1O2_ICSD_162390	insulator	insulator
H1Ni1O2_ICSD_169980	metal	insulator
H1Ni1O2_ICSD_169981	insulator	metal
H1Ni1Pr1Sn1_ICSD_157927	metal	metal
H1Ni1Ti2_ICSD_108767	metal	metal
H1Ni1_ICSD_56077	metal	metal
H1Ni2_ICSD_201088	metal	metal
H1O10P3Pb2_ICSD_2494	insulator	insulator
H1O1Rb1_ICSD_61048	insulator	insulator
H1O2Tb1_ICSD_6164	insulator	insulator
H1O2Y1_ICSD_28442	insulator	insulator
H1O2Yb1_ICSD_26341	insulator	metal
H1O3P1Sn1_ICSD_25034	insulator	insulator
H1O4P1Sn1_ICSD_658	insulator	insulator
H1O4Rb1S1_ICSD_36326	insulator	insulator
H1O5P1Zn2_ICSD_62245	insulator	insulator
H1O5P1Zn2_ICSD_79334	insulator	insulator
H1O5P1Zn2_ICSD_81313	insulator	insulator
H1O5Rb5Si1_ICSD_417054	insulator	insulator
H1O8P2Rb1Zn2_ICSD_280530	insulator	insulator
H1O8Rb3S2_ICSD_60050	insulator	insulator
H1Pd1Ti2_ICSD_167656	metal	metal
H1Pd1_ICSD_638418	metal	metal
H1Pd3Tl1_ICSD_247273	metal	metal
H1Rb1_ICSD_183292	insulator	insulator
H1Rh1_ICSD_56168	metal	metal
H1Se1Y1_ICSD_72008	insulator	metal
H1Si1_ICSD_41478	insulator	insulator
H1Si3Y5_ICSD_638464	metal	metal
H1Ta2_ICSD_41774	metal	metal
H1Ti1_ICSD_168325	metal	metal
H1V2_ICSD_61422	metal	metal
H1V2_ICSD_61423	metal	metal
H1W1_ICSD_247587	metal	metal
H1Zr1_ICSD_169451	metal	metal
H1_ICSD_426942	insulator	insulator
H2.25La1_ICSD_187682	metal	metal
H20N4O2P4S8_ICSD_16451	insulator	insulator
H24Li2N8Te2_ICSD_409556	insulator	insulator
H2Hf1_ICSD_24721	metal	metal
H2Hf2Pd1_ICSD_638175	metal	metal
H2Hg1O5Se1_ICSD_1712	insulator	insulator
H2Hg2O11S2Zn2_ICSD_413515	insulator	insulator
H2Ho1_ICSD_56057	metal	metal

Supplementary Table 429. Five-fold cross validated predictions for the metal/insulator classification (421/598).

system	calculated	predicted
H2I2O1Sr1_ICSD_76503	insulator	insulator
H2I3K1O1_ICSD_26338	insulator	insulator
H2In1O5P1_ICSD_86674	insulator	insulator
H2K1Mn1O5P1_ICSD_71177	insulator	insulator
H2K1N1O3S1_ICSD_15324	insulator	insulator
H2K1N1_ICSD_25619	insulator	insulator
H2K1O2P1_ICSD_59833	insulator	insulator
H2K1O3P1S1_ICSD_75217	insulator	insulator
H2K1O4P1_ICSD_186817	insulator	insulator
H2K1O4P1_ICSD_29258	insulator	insulator
H2K1O4P1_ICSD_31151	insulator	insulator
H2K1O4P1_ICSD_87895	insulator	insulator
H2K1O6P2V1_ICSD_423276	metal	insulator
H2K2Mn3O10V2_ICSD_203214	insulator	insulator
H2K2O5Ru1_ICSD_36593	insulator	insulator
H2K2O7P2_ICSD_36043	insulator	insulator
H2K3O10Si3Tb1_ICSD_413425	insulator	insulator
H2La1N1O7S1_ICSD_415675	insulator	insulator
H2La1Ni1Sn1_ICSD_157929	metal	metal
H2La1_ICSD_638229	metal	metal
H2Li1N1O3_ICSD_37180	insulator	insulator
H2Li1N1Sr2_ICSD_417994	insulator	insulator
H2Li1N1_ICSD_168792	insulator	insulator
H2Li1O2P1_ICSD_59823	insulator	insulator
H2Li1O3P1_ICSD_15045	insulator	insulator
H2Li1O3P1_ICSD_7	insulator	insulator
H2Li1O5P1Zn1_ICSD_79351	insulator	insulator
H2Li2Mg1N2_ICSD_165890	insulator	insulator
H2Li2O5S1_ICSD_20617	insulator	insulator
H2Li2O5Se1_ICSD_281276	insulator	insulator
H2Li2Pd1_ICSD_108534	metal	metal
H2Lu1_ICSD_56067	metal	metal
H2Mg0.125Ti0.875_ICSD_168835	metal	metal
H2Mg1Mo1O5_ICSD_202695	insulator	insulator
H2Mg1Ni1_ICSD_187258	metal	metal
H2Mg1O2_ICSD_79032	insulator	insulator
H2Mg1O5S1_ICSD_26304	insulator	insulator
H2Mg1O5Se1_ICSD_66745	insulator	insulator
H2Mg1O6Se2_ICSD_59310	insulator	insulator
H2Mg1O8S2_ICSD_62325	insulator	insulator
H2Mg1O8Se2_ICSD_66966	insulator	insulator
H2Mg1_ICSD_155807	insulator	insulator
H2Mg1_ICSD_155808	insulator	insulator
H2Mg1_ICSD_155809	insulator	insulator
H2Mg1_ICSD_155810	insulator	insulator
H2Mg1_ICSD_155811	insulator	insulator
H2Mg1_ICSD_166234	metal	insulator
H2Mg2O3_ICSD_95472	insulator	insulator
H2Mg3O4_ICSD_95471	insulator	insulator
H2Mg4O5_ICSD_95470	insulator	insulator

Supplementary Table 430. Five-fold cross validated predictions for the metal/insulator classification (422/598).

system	calculated	predicted
H2Mg5O6_ICSD_95469	insulator	insulator
H2Mn1O2_ICSD_23591	insulator	insulator
H2Mn1O5P1_ICSD_62220	insulator	insulator
H2Mn1O5S1_ICSD_71344	metal	insulator
H2Mn1O7P2_ICSD_419085	insulator	insulator
H2Mn1O8S2_ICSD_408752	insulator	insulator
H2Mn2O6S1_ICSD_413556	insulator	insulator
H2Mn3O10Se3_ICSD_281509	insulator	insulator
H2Mn6O17P4_ICSD_412152	insulator	insulator
H2N1Na1_ICSD_34290	insulator	insulator
H2N2Na2O4_ICSD_10182	insulator	insulator
H2N2O5Sr1_ICSD_38403	insulator	insulator
H2N2S6_ICSD_2535	insulator	insulator
H2Na2Pd1_ICSD_68071	metal	insulator
H2Na3O6Os1_ICSD_172060	insulator	insulator
H2Nb1_ICSD_56074	metal	metal
H2Nd1Ni1Sn1_ICSD_157932	metal	metal
H2Nd1_ICSD_56076	metal	metal
H2Ni1O2_ICSD_169978	insulator	insulator
H2Ni1O5S1_ICSD_71347	insulator	insulator
H2Ni1O5Se1_ICSD_66748	insulator	insulator
H2Ni1Pr1Sn1_ICSD_157931	metal	metal
H2O10P2Ti2_ICSD_160167	insulator	insulator
H2O13Ti6_ICSD_186994	insulator	insulator
H2O1_ICSD_27837	insulator	insulator
H2O1_ICSD_27844	metal	insulator
H2O1_ICSD_31683	insulator	insulator
H2O1_ICSD_64771	insulator	insulator
H2O2P1Rb1_ICSD_250131	insulator	insulator
H2O2Pb1_ICSD_20701	insulator	insulator
H2O2Zn1_ICSD_50447	insulator	insulator
H2O2_ICSD_34253	insulator	insulator
H2O3Se1_ICSD_31929	insulator	insulator
H2O4P1Rb1_ICSD_69317	insulator	insulator
H2O4P1Rb1_ICSD_69318	insulator	insulator
H2O4P1Tl1_ICSD_8051	insulator	insulator
H2O4P1Tl1_ICSD_81068	insulator	insulator
H2O4Pb3_ICSD_60054	insulator	insulator
H2O4S1_ICSD_38429	insulator	insulator
H2O4S1_ICSD_82732	insulator	insulator
H2O4S1_ICSD_95409	insulator	insulator
H2O4Se1_ICSD_60942	insulator	insulator
H2O4U1_ICSD_2867	insulator	insulator
H2O4U1_ICSD_9153	insulator	insulator
H2O5S1Zn1_ICSD_71348	insulator	insulator
H2O5Se1V1_ICSD_69994	insulator	insulator
H2O5Se1Zn1_ICSD_66749	insulator	insulator
H2O6P2Rb1V1_ICSD_423279	insulator	insulator
H2O6P2Zr1_ICSD_201970	insulator	insulator
H2O6P2Zr1_ICSD_74463	insulator	insulator

Supplementary Table 431. Five-fold cross validated predictions for the metal/insulator classification (423/598).

system	calculated	predicted
H2O6S1Ti1_ICSD_72973	insulator	insulator
H2O6Se2Sr1_ICSD_410935	insulator	insulator
H2O8Pb4S1_ICSD_84850	insulator	insulator
H2O8S2Zn1_ICSD_63418	insulator	insulator
H2O8Se2Zn1_ICSD_66967	insulator	insulator
H2Pd1Zr2_ICSD_638425	metal	metal
H2Pr1_ICSD_56080	metal	metal
H2Pu1_ICSD_638441	metal	metal
H2S1_ICSD_168207	insulator	insulator
H2S1_ICSD_24582	metal	insulator
H2S1_ICSD_50333	insulator	insulator
H2Sc1_ICSD_638457	metal	metal
H2Se1_ICSD_24584	metal	insulator
H2Sm1_ICSD_44878	metal	metal
H2Sm1_ICSD_638466	metal	metal
H2Sm1_ICSD_638472	metal	metal
H2Sr1_ICSD_163569	insulator	insulator
H2Sr1_ICSD_163576	insulator	insulator
H2Ta1_ICSD_56172	metal	metal
H2Tb1_ICSD_56175	metal	metal
H2Th1_ICSD_180580	metal	metal
H2Th1_ICSD_180581	metal	metal
H2Ti1_ICSD_166235	metal	metal
H2Ti1_ICSD_168834	metal	metal
H2Ti1_ICSD_169605	metal	metal
H2Tm1_ICSD_56187	metal	metal
H2Tm1_ICSD_638513	metal	metal
H2V1_ICSD_638528	metal	metal
H2W1_ICSD_247593	metal	metal
H2Y1_ICSD_638535	metal	metal
H2Yb1_ICSD_56195	metal	metal
H2Yb1_ICSD_638550	metal	metal
H2Zr1_ICSD_56198	metal	metal
H2Zr1_ICSD_638551	metal	metal
H2_ICSD_28344	insulator	insulator
H2_ICSD_28465	insulator	insulator
H2_ICSD_28539	insulator	insulator
H2_ICSD_62003	insulator	insulator
H2_ICSD_67425	insulator	insulator
H3Ho1O3_ICSD_200096	metal	insulator
H3Ho1_ICSD_638194	insulator	metal
H3I1Li1N1_ICSD_55064	insulator	insulator
H3I1Si1_ICSD_65044	insulator	insulator
H3I1Sr2_ICSD_422181	insulator	insulator
H3I3N2_ICSD_27416	insulator	insulator
H3K1Li2O3_ICSD_65148	insulator	insulator
H3K1Mg1_ICSD_181940	metal	insulator
H3K1Mg1_ICSD_638209	insulator	insulator
H3K1N1O3P1_ICSD_23291	insulator	insulator
H3K1O2_ICSD_47114	insulator	insulator

Supplementary Table 432. Five-fold cross validated predictions for the metal/insulator classification (424/598).

system	calculated	predicted
H3K1O6Se2_ICSD_2838	insulator	insulator
H3K1Si1_ICSD_65954	insulator	insulator
H3La1O12Se3_ICSD_411831	insulator	insulator
H3La1O3_ICSD_167480	insulator	insulator
H3La1_ICSD_638226	metal	metal
H3Li1Mg1_ICSD_181326	insulator	insulator
H3Li1O2_ICSD_9138	insulator	insulator
H3Li1O6Se2_ICSD_8243	insulator	insulator
H3Li1Sr1_ICSD_23978	insulator	insulator
H3Li4Mg1N3_ICSD_165891	insulator	insulator
H3Mg1Na1_ICSD_159174	insulator	insulator
H3Mg1Ni1_ICSD_187259	metal	metal
H3N1Na1O3P1_ICSD_16608	insulator	insulator
H3N1O3S1_ICSD_802	insulator	insulator
H3N1O6Sr2_ICSD_415799	insulator	insulator
H3N1_ICSD_84461	insulator	insulator
H3N3Pb1S2_ICSD_49538	insulator	insulator
H3Na1O2_ICSD_33909	insulator	insulator
H3Na1O5S1_ICSD_28259	insulator	insulator
H3Na1O6Se2_ICSD_34523	metal	insulator
H3Na1O6Si2_ICSD_39749	insulator	insulator
H3Na1O8S2Zn2_ICSD_61213	insulator	insulator
H3Nd1O3_ICSD_167482	metal	insulator
H3Ni1Yb1_ICSD_638405	metal	metal
H3Ni1Zr1_ICSD_638406	metal	metal
H3O2Rb1_ICSD_47115	insulator	insulator
H3O3P1_ICSD_33790	insulator	insulator
H3O3Tb1_ICSD_1224	insulator	insulator
H3O3Y1_ICSD_200098	insulator	insulator
H3O4P1Sr1_ICSD_411738	insulator	insulator
H3O4P1_ICSD_15887	insulator	insulator
H3O9P3Sb2_ICSD_26555	insulator	insulator
H3O9P3Sc2_ICSD_409724	insulator	insulator
H3P1_ICSD_24498	metal	insulator
H3Pa1_ICSD_76030	metal	metal
H3Pd1Ti2_ICSD_167659	metal	metal
H3Pd1Yb1_ICSD_638422	metal	metal
H3Pd1Zr2_ICSD_601955	metal	metal
H3Pu1_ICSD_638440	metal	metal
H3Tb1_ICSD_638485	insulator	metal
H3U1_ICSD_44994	metal	metal
H3U1_ICSD_60546	metal	metal
H3W1_ICSD_247598	metal	metal
H3Y1_ICSD_154809	insulator	metal
H3Y1_ICSD_180317	metal	metal
H3Y1_ICSD_41548	metal	metal
H3Yb1_ICSD_56196	metal	metal
H4.001N1.667Rb1_ICSD_418413	insulator	insulator
H4Hg2O9P2_ICSD_413085	insulator	insulator
H4I1N1O4_ICSD_280083	insulator	insulator

Supplementary Table 433. Five-fold cross validated predictions for the metal/insulator classification (425/598).

system	calculated	predicted
H4I1Na1O2_ICSD_23134	insulator	insulator
H4I1P1_ICSD_22083	insulator	insulator
H4I2O8Zn1_ICSD_410881	insulator	insulator
H4I3N1Pb1_ICSD_240915	insulator	insulator
H4I3N1_ICSD_48202	insulator	insulator
H4I3O11Yb1_ICSD_413352	insulator	insulator
H4I4K2O14Zn1_ICSD_260726	insulator	insulator
H4K2Mg1_ICSD_68358	insulator	insulator
H4K2Mn3O12P4_ICSD_260451	insulator	insulator
H4K2N4O10Pd1_ICSD_164218	metal	insulator
H4K2O6Os1_ICSD_409612	insulator	insulator
H4K2Zn1_ICSD_187277	insulator	insulator
H4La1N1O8S2_ICSD_20895	insulator	insulator
H4Li2Mg1_ICSD_181325	insulator	insulator
H4Li4Rh1_ICSD_26226	metal	metal
H4Mg1Na2O8S2_ICSD_35767	insulator	insulator
H4Mg1Ni4Y1_ICSD_169537	metal	metal
H4Mg2Ni1_ICSD_162413	insulator	insulator
H4Mg3O5_ICSD_95473	insulator	insulator
H4Mg3O9Si2_ICSD_75933	insulator	insulator
H4N1O3V1_ICSD_164689	insulator	insulator
H4N1O4Re1_ICSD_1394	insulator	insulator
H4N1O4Tc1_ICSD_10427	insulator	insulator
H4N2Ni1_ICSD_69723	insulator	insulator
H4N2O2S1_ICSD_62521	metal	insulator
H4N2O3_ICSD_27453	insulator	insulator
H4N2O3_ICSD_2772	insulator	insulator
H4N2O3_ICSD_37128	insulator	insulator
H4N2O8Zn1_ICSD_9286	insulator	insulator
H4N4S4_ICSD_15394	insulator	insulator
H4N4_ICSD_2236	insulator	insulator
H4Na2O10Se2Zn1_ICSD_98685	insulator	insulator
H4Na2O5Ti1_ICSD_183668	insulator	insulator
H4Na2O8S4_ICSD_40833	insulator	insulator
H4Na2O8Se2_ICSD_71786	insulator	insulator
H4Na2Pt1_ICSD_638356	insulator	insulator
H4Na6O18S4Zn1_ICSD_61246	insulator	insulator
H4Ni2O10P2Sr1_ICSD_261127	insulator	insulator
H4O10Si2Zn4_ICSD_422820	insulator	insulator
H4O10V4_ICSD_260368	insulator	insulator
H4O11P2Ti2_ICSD_84307	insulator	insulator
H4O12S3Sr1_ICSD_404139	insulator	insulator
H4O3Sr1_ICSD_63016	insulator	insulator
H4O4P2Pb1_ICSD_59933	insulator	insulator
H4O4P2Sr1_ICSD_59932	insulator	insulator
H4O4P2Zn1_ICSD_66911	insulator	insulator
H4O4V2_ICSD_41123	insulator	insulator
H4O6P2Sr1_ICSD_412173	insulator	insulator
H4O8P2Pb1_ICSD_86778	insulator	insulator
H4O9P2V1_ICSD_20182	insulator	insulator

Supplementary Table 434. Five-fold cross validated predictions for the metal/insulator classification (426/598).

system	calculated	predicted
H4Pd1Ti2_ICSD_167660	metal	metal
H4S6Si4_ICSD_36379	insulator	insulator
H4Si1_ICSD_159307	insulator	insulator
H4Sn1_ICSD_168178	metal	metal
H4Sn1_ICSD_168179	metal	metal
H4Sn1_ICSD_168180	metal	metal
H4Sn1_ICSD_168181	metal	metal
H5I1O6_ICSD_25790	insulator	insulator
H5K1O8P2_ICSD_23097	insulator	insulator
H5K2O6Sb1_ICSD_422765	insulator	insulator
H5K3O14P4_ICSD_30511	insulator	insulator
H5K3Zn1_ICSD_187417	insulator	insulator
H5Li1N2O4S1_ICSD_2051	insulator	insulator
H5Li4Rh1_ICSD_26225	metal	insulator
H5N1O3Se1_ICSD_171372	insulator	insulator
H5Na3O16Se4_ICSD_411720	insulator	insulator
H5Nd2_ICSD_638383	metal	metal
H5O8P2Rb1_ICSD_20915	insulator	insulator
H5O8P2Tl1_ICSD_30509	insulator	insulator
H5Rb3Zn1_ICSD_187418	insulator	insulator
H5W1_ICSD_247606	metal	metal
H5W1_ICSD_247609	metal	metal
H6Hf1Na2O6_ICSD_88890	insulator	insulator
H6Hf1O10P2_ICSD_94481	insulator	insulator
H6Hg1I3N1O1_ICSD_409701	insulator	insulator
H6Ir1Li1Mg1_ICSD_180122	insulator	insulator
H6Ir1Na3_ICSD_40575	insulator	insulator
H6K2O6Pb1_ICSD_92466	insulator	insulator
H6K2O6Sn1_ICSD_92465	insulator	insulator
H6La1O12P3_ICSD_240963	insulator	insulator
H6Li1Mn1O7_ICSD_33776	insulator	insulator
H6Li1N1O6_ICSD_48040	insulator	insulator
H6Li2N3Rb1_ICSD_56394	insulator	insulator
H6Li2Pt1_ICSD_236283	insulator	insulator
H6Li4Ru1_ICSD_638272	insulator	insulator
H6Mg1O6Si1_ICSD_185677	insulator	insulator
H6Mg2Na2Ni1_ICSD_260394	insulator	insulator
H6N1Nd1O10S1_ICSD_416245	insulator	insulator
H6N1Ni1O5P1_ICSD_424553	insulator	insulator
H6N1O2P1_ICSD_250178	insulator	insulator
H6N1O4P1_ICSD_246299	insulator	insulator
H6N1O4P1_ICSD_29257	insulator	insulator
H6N2O4S1_ICSD_16055	insulator	insulator
H6N2O4S1_ICSD_1977	insulator	insulator
H6N3O12Tl1_ICSD_1723	insulator	insulator
H6N3O1P1_ICSD_16151	insulator	insulator
H6N4O4Pd1_ICSD_420291	insulator	insulator
H6N4O4Pt1_ICSD_420292	insulator	insulator
H6Na1O6P1_ICSD_66	insulator	insulator
H6Na2O6Sn1_ICSD_92464	insulator	insulator

Supplementary Table 435. Five-fold cross validated predictions for the metal/insulator classification (427/598).

system	calculated	predicted
H6Na2Pt1_ICSD_236284	insulator	insulator
H6Na4Ru1_ICSD_638362	insulator	insulator
H6Nd1O12P3_ICSD_426743	insulator	insulator
H6O10P2Zr1_ICSD_94480	insulator	insulator
H6O12P3Pr1_ICSD_155271	insulator	insulator
H6O12P3Sc1_ICSD_20432	insulator	insulator
H6O12Sc2Se3_ICSD_391277	insulator	insulator
H6O1Si2_ICSD_30501	insulator	insulator
H6O5Sr1Zn1_ICSD_50177	insulator	insulator
H6O8Se2Zn1_ICSD_200525	insulator	insulator
H6Os1Sr2_ICSD_638409	insulator	insulator
H6Ru1Sr2_ICSD_655142	insulator	insulator
H6W1_ICSD_247611	metal	metal
H7La1Ni5_ICSD_96245	metal	metal
H7La1Ni5_ICSD_96246	metal	metal
H7La2O11P3_ICSD_249116	insulator	insulator
H7Mg3Tc1_ICSD_262724	insulator	insulator
H7N1O6_ICSD_1902	insulator	insulator
H7N2O4P1_ICSD_15083	insulator	insulator
H7Na5O18Se4_ICSD_40276	insulator	insulator
H7Th1Zr2_ICSD_56180	metal	metal
H8I1N4P1_ICSD_400461	insulator	insulator
H8I1N4P1_ICSD_74814	insulator	insulator
H8K1N3_ICSD_414496	insulator	insulator
H8K1N4Y1_ICSD_202010	insulator	insulator
H8K2Ni1O16P4_ICSD_281592	insulator	insulator
H8K2O14V2_ICSD_67322	insulator	insulator
H8K2O16P4Zn1_ICSD_281290	insulator	insulator
H8K4O4P2Se6_ICSD_260476	insulator	insulator
H8La1O7P3_ICSD_88015	insulator	insulator
H8Mg1O14V4_ICSD_185911	insulator	insulator
H8Mg5O9_ICSD_95474	insulator	insulator
H8Mn1O16P4Tl2_ICSD_416649	insulator	insulator
H8Mo1N2O2S2_ICSD_41688	insulator	insulator
H8N10Zn1_ICSD_421952	insulator	insulator
H8N2O4S1_ICSD_34258	insulator	insulator
H8N2O4Se1_ICSD_838	insulator	insulator
H8N2S5_ICSD_30984	insulator	insulator
H8N8Na1O12Rh1_ICSD_169810	insulator	insulator
H8Ni1O10P2_ICSD_417429	metal	insulator
H8Ni1O12Re2_ICSD_402773	insulator	insulator
H8Ni1O16P4Tl2_ICSD_416651	insulator	insulator
H8O10P2Zn1_ICSD_417431	insulator	insulator
H8O12Si8_ICSD_75244	insulator	insulator
H8O16P4Tl2Zn1_ICSD_416652	insulator	insulator
H8O8Sn1Sr2_ICSD_91102	insulator	insulator
H8O8U1_ICSD_167992	insulator	insulator
H8Si1_ICSD_169739	insulator	insulator
H8Si1_ICSD_169740	insulator	insulator
H9.975La3.99_ICSD_638228	metal	metal

Supplementary Table 436. Five-fold cross validated predictions for the metal/insulator classification (428/598).

system	calculated	predicted
H9K1O5_ICSD_36598	insulator	insulator
H9K2Re1_ICSD_27563	insulator	insulator
H9N1Si3_ICSD_201428	insulator	insulator
H9N2O4P1_ICSD_2799	insulator	insulator
He1_ICSD_22384	insulator	insulator
He1_ICSD_426943	insulator	insulator
He1_ICSD_44394	insulator	insulator
He1_ICSD_44395	insulator	insulator
He2Ne1_ICSD_659143	insulator	insulator
Hf0.17Re0.83_ICSD_638804	metal	metal
Hf1.5Mn1.5_ICSD_638599	metal	metal
Hf10Mo3Si1_ICSD_603654	metal	metal
Hf1I1N1_ICSD_51774	insulator	insulator
Hf1I3_ICSD_23947	metal	insulator
Hf1I4_ICSD_109275	insulator	insulator
Hf1I4_ICSD_200826	insulator	insulator
Hf1I4_ICSD_62473	insulator	insulator
Hf1In1Ni2_ICSD_54595	metal	metal
Hf1Ir1Si1_ICSD_638584	metal	metal
Hf1Ir1Sn1_ICSD_638586	metal	metal
Hf1Ir1_ICSD_185632	metal	metal
Hf1Ir1_ICSD_185634	metal	metal
Hf1Ir3_ICSD_185651	metal	metal
Hf1La3Sb5_ICSD_83906	metal	metal
Hf1Mn1Si1_ICSD_53024	metal	metal
Hf1Mn2_ICSD_104214	metal	metal
Hf1Mn6Sn6_ICSD_54930	metal	metal
Hf1Mo1P1_ICSD_88151	metal	metal
Hf1Mo1Si1_ICSD_638634	metal	metal
Hf1Mo2O8_ICSD_98065	insulator	insulator
Hf1Mo2_ICSD_638610	metal	metal
Hf1Mo2_ICSD_638621	metal	metal
Hf1N1O3Ta1_ICSD_186407	insulator	insulator
Hf1N1O3Ta1_ICSD_186408	insulator	insulator
Hf1N1O3Ta1_ICSD_186409	insulator	insulator
Hf1N1_ICSD_167875	metal	metal
Hf1N1_ICSD_183420	metal	metal
Hf1N1_ICSD_638649	metal	metal
Hf1N2Sr1_ICSD_82538	insulator	insulator
Hf1N2_ICSD_290427	metal	insulator
Hf1N2_ICSD_290428	insulator	metal
Hf1N2_ICSD_290429	insulator	insulator
Hf1N3Ta2_ICSD_186423	metal	metal
Hf1Nb1P1_ICSD_75009	metal	metal
Hf1Ni1P1_ICSD_638712	metal	metal
Hf1Ni1Si1_ICSD_638723	metal	metal
Hf1Ni1Sn1_ICSD_638731	insulator	metal
Hf1Ni1_ICSD_638689	metal	metal
Hf1Ni2Sb1_ICSD_638718	metal	metal
Hf1Ni2Si2_ICSD_638721	metal	metal

Supplementary Table 437. Five-fold cross validated predictions for the metal/insulator classification (429/598).

system	calculated	predicted
Hf1Ni2Sn1_ICSD_104250	metal	metal
Hf1Ni2_ICSD_104231	metal	metal
Hf1Ni3_ICSD_2414	metal	metal
Hf1Ni3_ICSD_2415	metal	metal
Hf1Ni4P2_ICSD_638711	metal	metal
Hf1Ni5_ICSD_150940	metal	metal
Hf1O0.1667_ICSD_174039	metal	metal
Hf1O1S1_ICSD_23327	insulator	insulator
Hf1O2_ICSD_173965	insulator	insulator
Hf1O2_ICSD_173966	insulator	insulator
Hf1O2_ICSD_173968	insulator	insulator
Hf1O2_ICSD_180834	insulator	insulator
Hf1O2_ICSD_187015	insulator	insulator
Hf1O2_ICSD_83863	insulator	insulator
Hf1O3Pb1_ICSD_161702	insulator	insulator
Hf1O3Pb1_ICSD_174110	insulator	insulator
Hf1O3Pb1_ICSD_33194	insulator	insulator
Hf1O3Sr1_ICSD_161594	insulator	insulator
Hf1O3Sr1_ICSD_161595	insulator	insulator
Hf1O3Sr1_ICSD_161597	insulator	insulator
Hf1O3Sr1_ICSD_161598	insulator	insulator
Hf1O3Sr1_ICSD_164620	insulator	insulator
Hf1O3Sr1_ICSD_164622	insulator	insulator
Hf1O3Sr1_ICSD_84280	insulator	insulator
Hf1O4Si1_ICSD_187732	insulator	insulator
Hf1O7V2_ICSD_90134	insulator	insulator
Hf1O8Te3_ICSD_9078	insulator	insulator
Hf1O8W2_ICSD_188441	insulator	insulator
Hf1Os1P1_ICSD_638751	metal	metal
Hf1Os1Si1_ICSD_638752	metal	metal
Hf1Os1Si1_ICSD_638753	metal	metal
Hf1Os1_ICSD_104252	metal	metal
Hf1Os2_ICSD_150513	metal	metal
Hf1Os3_ICSD_185650	metal	metal
Hf1P1Ru1_ICSD_53035	metal	metal
Hf1P1V1_ICSD_656389	metal	metal
Hf1P1_ICSD_42914	metal	metal
Hf1P2S6_ICSD_47228	insulator	insulator
Hf1P2_ICSD_638760	metal	metal
Hf1Pb1S3_ICSD_65668	insulator	insulator
Hf1Pd1Si1_ICSD_90279	metal	metal
Hf1Pd1Sn1_ICSD_106773	insulator	insulator
Hf1Pd1_ICSD_185630	metal	metal
Hf1Pd1_ICSD_185631	metal	metal
Hf1Pd3_ICSD_104254	metal	metal
Hf1Pd3_ICSD_185649	metal	metal
Hf1Pd5_ICSD_168289	metal	metal
Hf1Pt1Si1_ICSD_638791	metal	metal
Hf1Pt1Sn1_ICSD_104261	metal	metal
Hf1Pt1_ICSD_104257	metal	metal

Supplementary Table 438. Five-fold cross validated predictions for the metal/insulator classification (430/598).

system	calculated	predicted
Hf1Pt1_ICSD_638781	metal	metal
Hf1Pt3_ICSD_185652	metal	metal
Hf1Pt3_ICSD_638782	metal	metal
Hf1Re1Si1_ICSD_638814	metal	metal
Hf1Re2_ICSD_638810	metal	metal
Hf1Rh1Sb1_ICSD_53036	metal	metal
Hf1Rh1Si1_ICSD_638834	metal	metal
Hf1Rh1Sn1_ICSD_107474	metal	metal
Hf1Rh1Sn1_ICSD_638836	metal	metal
Hf1Rh1_ICSD_104265	metal	metal
Hf1Rh3_ICSD_104266	metal	metal
Hf1Ru1Sb1_ICSD_107125	metal	metal
Hf1Ru1Si1_ICSD_638841	metal	metal
Hf1Ru1_ICSD_104268	metal	metal
Hf1Ru3_ICSD_185647	metal	metal
Hf1S1Si1_ICSD_25735	metal	metal
Hf1S2_ICSD_182677	insulator	insulator
Hf1S3Sn1_ICSD_65667	insulator	insulator
Hf1S3_ICSD_638846	insulator	insulator
Hf1S4Tl4_ICSD_261210	insulator	insulator
Hf1S5Y2_ICSD_185	insulator	insulator
Hf1Sb1_ICSD_107591	metal	metal
Hf1Sb2_ICSD_638876	metal	metal
Hf1Sb2_ICSD_66780	metal	metal
Hf1Sb5U3_ICSD_154389	metal	metal
Hf1Se1Si1_ICSD_25736	metal	metal
Hf1Se2_ICSD_182678	insulator	metal
Hf1Se3Tl2_ICSD_186071	insulator	insulator
Hf1Se3_ICSD_638898	insulator	insulator
Hf1Si1Te1_ICSD_25737	metal	metal
Hf1Si1V1_ICSD_638931	metal	metal
Hf1Si1_ICSD_638911	metal	metal
Hf1Si2_ICSD_638923	metal	metal
Hf1Sn1_ICSD_104272	metal	metal
Hf1Sn2_ICSD_638941	metal	metal
Hf1Sn2_ICSD_638946	metal	metal
Hf1Tc1_ICSD_104277	metal	metal
Hf1Tc2_ICSD_638956	metal	metal
Hf1Te2_ICSD_638959	metal	metal
Hf1Te3_ICSD_638962	metal	metal
Hf1Te4Tl4_ICSD_168651	insulator	insulator
Hf1Te5_ICSD_657474	metal	metal
Hf1V2_ICSD_187951	metal	metal
Hf1V2_ICSD_187953	metal	metal
Hf1V2_ICSD_280388	metal	metal
Hf1W2_ICSD_639010	metal	metal
Hf1Zn22_ICSD_639015	metal	metal
Hf1Zn2_ICSD_104292	metal	metal
Hf1Zn2_ICSD_639018	metal	metal
Hf1_ICSD_187430	metal	metal

Supplementary Table 439. Five-fold cross validated predictions for the metal/insulator classification (431/598).

system	calculated	predicted
Hf1_ICSD_41519	metal	metal
Hf1_ICSD_53022	metal	metal
Hf1_ICSD_76412	metal	metal
Hf2Hg1_ICSD_638564	metal	metal
Hf2Ho2O7_ICSD_162005	insulator	insulator
Hf2In5_ICSD_404531	metal	metal
Hf2Ir1_ICSD_638574	metal	metal
Hf2La2O7_ICSD_153815	insulator	insulator
Hf2La2O7_ICSD_173790	metal	insulator
Hf2Mn1_ICSD_638594	metal	metal
Hf2N3Ta1_ICSD_186420	metal	metal
Hf2Nd2O7_ICSD_236210	insulator	insulator
Hf2Ni1P1_ICSD_405616	metal	metal
Hf2Ni1_ICSD_102808	metal	metal
Hf2Ni2Sn1_ICSD_107096	metal	metal
Hf2Ni2Sn1_ICSD_407546	metal	metal
Hf2Ni3Si4_ICSD_40442	metal	metal
Hf2O7Y2_ICSD_153819	insulator	insulator
Hf2O9P2_ICSD_416013	insulator	insulator
Hf2Os1_ICSD_638748	metal	metal
Hf2P1Sb1_ICSD_638764	metal	metal
Hf2P1_ICSD_24350	metal	metal
Hf2Pd1_ICSD_104255	metal	metal
Hf2Pd1_ICSD_638772	metal	metal
Hf2Pt1_ICSD_638787	metal	metal
Hf2Rh1_ICSD_638817	metal	metal
Hf2S1_ICSD_43203	metal	metal
Hf2Si1_ICSD_638912	metal	metal
Hf2Te1_ICSD_83965	metal	metal
Hf2Tl1_ICSD_168287	metal	metal
Hf2Tl1_ICSD_168954	metal	metal
Hf2Zn1_ICSD_639016	metal	metal
Hf3In4_ICSD_638568	metal	metal
Hf3N1Zn3_ICSD_42937	metal	metal
Hf3N4_ICSD_97997	insulator	metal
Hf3Ni2Si3_ICSD_200067	metal	metal
Hf3Ni7_ICSD_2417	metal	metal
Hf3P1_ICSD_35439	metal	metal
Hf3P2_ICSD_36117	metal	metal
Hf3P3Pd4_ICSD_280272	metal	metal
Hf3Rh5_ICSD_104267	metal	metal
Hf3Sb1_ICSD_638878	metal	metal
Hf3Sc1_ICSD_168284	metal	metal
Hf3Si2_ICSD_638925	metal	metal
Hf3Te2_ICSD_75936	metal	metal
Hf4Ni1P1_ICSD_638708	metal	metal
Hf5In4Rh2_ICSD_412706	metal	metal
Hf5Ir3_ICSD_638575	metal	metal
Hf5Ir3_ICSD_638578	metal	metal
Hf5K4O12_ICSD_180186	insulator	insulator

Supplementary Table 440. Five-fold cross validated predictions for the metal/insulator classification (432/598).

system	calculated	predicted
Hf5Ni1Sn3_ICSD_638730	metal	metal
Hf5Pb1_ICSD_168288	metal	metal
Hf5Sb3Zn1_ICSD_42913	metal	metal
Hf5Sb3_ICSD_410750	metal	metal
Hf5Sb9_ICSD_413979	metal	metal
Hf5Sc1_ICSD_168285	metal	metal
Hf5Si3_ICSD_638909	metal	metal
Hf5Si4_ICSD_638914	metal	metal
Hf5Sn3_ICSD_183487	metal	metal
Hf5Sn4_ICSD_107313	metal	metal
Hf5Te4_ICSD_154358	metal	metal
Hf6Ni16Si7_ICSD_109223	metal	metal
Hf6Ni1Sb2_ICSD_85966	metal	metal
Hf7Ni10_ICSD_638690	metal	metal
Hf7P4_ICSD_405208	metal	metal
Hf8Ni21_ICSD_2416	metal	metal
Hf9Mo4Ni1_ICSD_638623	metal	metal
Hf9Mo4P1_ICSD_638625	metal	metal
Hf9Mo4S1_ICSD_638628	metal	metal
Hf9Mo4Se1_ICSD_638630	metal	metal
Hf9Mo4Si1_ICSD_260996	metal	metal
Hf9Ni11_ICSD_638706	metal	metal
Hf9Ni1Re4_ICSD_638713	metal	metal
Hf9Ni1W4_ICSD_638734	metal	metal
Hf9P1Re4_ICSD_638761	metal	metal
Hf9P1W4_ICSD_638765	metal	metal
Hf9Re4S1_ICSD_638811	metal	metal
Hf9Re4Se1_ICSD_638812	metal	metal
Hf9Re4Si1_ICSD_638813	metal	metal
Hf9S1V4_ICSD_81822	metal	metal
Hf9S1W4_ICSD_638867	metal	metal
Hf9Se1W4_ICSD_638906	metal	metal
Hg11K1_ICSD_410566	metal	metal
Hg11K3_ICSD_240039	metal	metal
Hg11Rb1_ICSD_410567	metal	metal
Hg11Sr1_ICSD_247134	metal	metal
Hg19Rb5_ICSD_107452	metal	metal
Hg1Ho1_ICSD_104297	metal	metal
Hg1Ho2O4_ICSD_69734	insulator	insulator
Hg1I1N1O3_ICSD_32580	insulator	insulator
Hg1I2O6_ICSD_98546	insulator	insulator
Hg1I2_ICSD_281133	insulator	insulator
Hg1I2_ICSD_412988	insulator	insulator
Hg1I2_ICSD_67069	insulator	insulator
Hg1I6Tl4_ICSD_14018	insulator	insulator
Hg1I6Tl4_ICSD_250133	insulator	insulator
Hg1In1_ICSD_104299	metal	metal
Hg1In2S4_ICSD_56081	insulator	insulator
Hg1In2Se4_ICSD_25649	insulator	insulator
Hg1In2Te4_ICSD_639056	insulator	insulator

Supplementary Table 441. Five-fold cross validated predictions for the metal/insulator classification (433/598).

system	calculated	predicted
Hg1K1Sb1_ICSD_56201	metal	metal
Hg1K1_ICSD_104302	metal	metal
Hg1K2O2_ICSD_66275	insulator	insulator
Hg1K2S2_ICSD_74500	insulator	insulator
Hg1K2Se4Sn1_ICSD_413308	insulator	insulator
Hg1K4P2_ICSD_67263	insulator	insulator
Hg1K6S4_ICSD_266	insulator	insulator
Hg1K6Se4_ICSD_639060	insulator	insulator
Hg1La1Pd1_ICSD_657950	metal	metal
Hg1La1_ICSD_639065	metal	metal
Hg1Li1_ICSD_639075	metal	metal
Hg1Li3_ICSD_104309	metal	metal
Hg1Lu1_ICSD_639078	metal	metal
Hg1Mg1_ICSD_104314	metal	metal
Hg1Mg2_ICSD_104315	metal	metal
Hg1Mg3_ICSD_639092	metal	metal
Hg1Mn1_ICSD_639098	metal	metal
Hg1Mo1O4_ICSD_2533	insulator	insulator
Hg1Mo6S8_ICSD_639106	metal	metal
Hg1N3O6Rb1_ICSD_28644	metal	insulator
Hg1N3O6Tl1_ICSD_28646	insulator	metal
Hg1N6_ICSD_21029	insulator	insulator
Hg1Na1O4P1_ICSD_32511	insulator	insulator
Hg1Na1_ICSD_104326	metal	metal
Hg1Na2O2_ICSD_27409	insulator	insulator
Hg1Na2Pb1_ICSD_261791	metal	metal
Hg1Na2S2_ICSD_74499	insulator	insulator
Hg1Na3_ICSD_408694	metal	metal
Hg1Na4P2_ICSD_67260	insulator	insulator
Hg1Nd1_ICSD_104330	metal	metal
Hg1Nd2O4_ICSD_41121	metal	insulator
Hg1Ni1_ICSD_100029	metal	metal
Hg1O14P4V2_ICSD_78510	insulator	insulator
Hg1O1_ICSD_16627	metal	metal
Hg1O1_ICSD_24062	insulator	insulator
Hg1O1_ICSD_32561	insulator	insulator
Hg1O1_ICSD_40316	insulator	insulator
Hg1O1_ICSD_639125	insulator	insulator
Hg1O2Rb2_ICSD_25514	insulator	insulator
Hg1O2Sr1_ICSD_165068	insulator	insulator
Hg1O2Sr1_ICSD_69739	insulator	insulator
Hg1O2_ICSD_24774	insulator	insulator
Hg1O2_ICSD_655816	insulator	insulator
Hg1O3Se1_ICSD_412547	insulator	insulator
Hg1O3Se1_ICSD_79694	insulator	insulator
Hg1O3Sn1_ICSD_260029	metal	insulator
Hg1O3Te1_ICSD_61673	insulator	insulator
Hg1O3Ti1_ICSD_19005	insulator	insulator
Hg1O3V1_ICSD_82242	insulator	insulator
Hg1O4Re1_ICSD_74885	insulator	insulator

Supplementary Table 442. Five-fold cross validated predictions for the metal/insulator classification (434/598).

system	calculated	predicted
Hg1O4S1_ICSD_28402	insulator	insulator
Hg1O4S1_ICSD_31870	insulator	insulator
Hg1O4Se1_ICSD_412403	insulator	insulator
Hg1O4W1_ICSD_169667	insulator	insulator
Hg1O4W1_ICSD_169671	insulator	insulator
Hg1O6V2_ICSD_26996	insulator	insulator
Hg1O7P2Pd1_ICSD_420533	insulator	insulator
Hg1O8P2Pd2_ICSD_420532	insulator	insulator
Hg1P1Pd5_ICSD_639128	metal	metal
Hg1P1Pt5_ICSD_639129	metal	metal
Hg1Pb1Sr1_ICSD_602710	metal	metal
Hg1Pb1Yb1_ICSD_108545	metal	metal
Hg1Pd1Pr1_ICSD_657952	metal	metal
Hg1Pd1Sm1_ICSD_106784	metal	metal
Hg1Pd1_ICSD_40321	metal	metal
Hg1Pd1_ICSD_639137	metal	metal
Hg1Pd5Se1_ICSD_639142	metal	metal
Hg1Pr1_ICSD_104336	metal	metal
Hg1Pt1_ICSD_104337	metal	metal
Hg1Pt2Se3_ICSD_185808	metal	insulator
Hg1Pt3_ICSD_639147	metal	metal
Hg1Rb1Sb1Se3_ICSD_89018	insulator	insulator
Hg1Rb2Sn1Te4_ICSD_262390	insulator	insulator
Hg1Rb6S4_ICSD_639158	insulator	insulator
Hg1Rb6Se4_ICSD_639159	insulator	insulator
Hg1S1_ICSD_169614	insulator	insulator
Hg1S1_ICSD_169621	metal	metal
Hg1S1_ICSD_31129	metal	metal
Hg1S1_ICSD_639164	metal	metal
Hg1S1_ICSD_81923	insulator	insulator
Hg1S2Ta1_ICSD_639178	metal	metal
Hg1Sc1_ICSD_104342	metal	metal
Hg1Se1_ICSD_639193	metal	metal
Hg1Se1_ICSD_639194	metal	metal
Hg1Se1_ICSD_639202	insulator	insulator
Hg1Se4Si1Tl2_ICSD_247763	insulator	insulator
Hg1Se4Sn1Tl2_ICSD_247764	insulator	insulator
Hg1Sn1Sr1_ICSD_602716	metal	metal
Hg1Sn1Te4Tl2_ICSD_172505	metal	insulator
Hg1Sn1Yb1_ICSD_602718	metal	metal
Hg1Sr1_ICSD_104345	metal	metal
Hg1Sr3_ICSD_639226	metal	metal
Hg1Tb1_ICSD_104348	metal	metal
Hg1Te1_ICSD_162603	insulator	insulator
Hg1Te1_ICSD_162604	metal	metal
Hg1Te1_ICSD_162605	metal	metal
Hg1Te1_ICSD_56216	metal	metal
Hg1Te1_ICSD_639251	insulator	insulator
Hg1Te1_ICSD_639258	metal	metal
Hg1Th2_ICSD_104351	metal	metal

Supplementary Table 443. Five-fold cross validated predictions for the metal/insulator classification (435/598).

system	calculated	predicted
Hg1Ti1_ICSD_104355	metal	metal
Hg1Ti3_ICSD_104356	metal	metal
Hg1Ti3_ICSD_104357	metal	metal
Hg1Tl1_ICSD_104360	metal	metal
Hg1Tm1_ICSD_104365	metal	metal
Hg1Y1_ICSD_639300	metal	metal
Hg1Yb1_ICSD_639303	metal	metal
Hg1Zr1_ICSD_639318	metal	metal
Hg1Zr3_ICSD_104375	metal	metal
Hg1_ICSD_157861	metal	metal
Hg1_ICSD_174006	metal	metal
Hg1_ICSD_426945	metal	metal
Hg1_ICSD_56897	metal	metal
Hg1_ICSD_79804	metal	metal
Hg20Rb3_ICSD_240037	metal	metal
Hg2Ho1_ICSD_104298	metal	metal
Hg2Ho1_ICSD_639033	metal	metal
Hg2I1Na1O2_ICSD_36110	insulator	insulator
Hg2I1O1_ICSD_33275	insulator	insulator
Hg2I2Pb1S2_ICSD_59204	insulator	insulator
Hg2I2_ICSD_36189	insulator	insulator
Hg2I4_ICSD_241174	metal	insulator
Hg2K1_ICSD_104303	metal	metal
Hg2La1_ICSD_104306	metal	metal
Hg2La1_ICSD_639067	metal	metal
Hg2Mg1_ICSD_104319	metal	metal
Hg2Mo1O4_ICSD_90083	insulator	insulator
Hg2Mo1O4_ICSD_90084	insulator	insulator
Hg2Mo2O7_ICSD_51511	insulator	insulator
Hg2Mo2O7_ICSD_83374	insulator	insulator
Hg2N2O4_ICSD_60055	insulator	insulator
Hg2N2O6_ICSD_412933	insulator	insulator
Hg2N6_ICSD_98661	insulator	insulator
Hg2Na1_ICSD_104327	metal	metal
Hg2Na3_ICSD_104328	metal	metal
Hg2Nb2O7_ICSD_22226	insulator	insulator
Hg2Nd1_ICSD_104331	metal	metal
Hg2O3Se1_ICSD_412303	insulator	insulator
Hg2O3Se1_ICSD_412304	insulator	insulator
Hg2O4S1_ICSD_15005	insulator	insulator
Hg2O4Se1_ICSD_15006	insulator	insulator
Hg2O4W1_ICSD_90085	insulator	insulator
Hg2O5Te1_ICSD_98671	insulator	insulator
Hg2O7Os2_ICSD_159801	metal	metal
Hg2O7P2_ICSD_59280	insulator	insulator
Hg2O7Ru2_ICSD_420818	metal	metal
Hg2O7Sb2_ICSD_160088	metal	insulator
Hg2O7V2_ICSD_2517	insulator	insulator
Hg2P2S6_ICSD_2564	insulator	insulator
Hg2P2S7_ICSD_2490	insulator	insulator

Supplementary Table 444. Five-fold cross validated predictions for the metal/insulator classification (436/598).

system	calculated	predicted
Hg2P2Se6_ICSD_639132	insulator	insulator
Hg2Pt1_ICSD_104339	metal	metal
Hg2Rb1_ICSD_408698	metal	metal
Hg2Rh1_ICSD_106786	metal	metal
Hg2Se4Sn1_ICSD_95061	metal	insulator
Hg2Sr1_ICSD_104346	metal	metal
Hg2Sr1_ICSD_104347	metal	metal
Hg2Sr3_ICSD_247135	metal	metal
Hg2Tb1_ICSD_639230	metal	metal
Hg2Th1_ICSD_639269	metal	metal
Hg2U1_ICSD_639291	metal	metal
Hg2Y1_ICSD_104368	metal	metal
Hg2Yb1_ICSD_104371	metal	metal
Hg3Ho1_ICSD_639032	metal	metal
Hg3I2S2_ICSD_411154	insulator	insulator
Hg3I2Se2_ICSD_99093	insulator	insulator
Hg3I2Te2_ICSD_65806	insulator	insulator
Hg3La1_ICSD_639068	metal	metal
Hg3Li1_ICSD_639074	metal	metal
Hg3Lu1_ICSD_639079	metal	metal
Hg3Mg5_ICSD_104320	metal	metal
Hg3Na2S4_ICSD_71597	insulator	insulator
Hg3O10Se3_ICSD_280914	insulator	insulator
Hg3O5Se4_ICSD_418001	insulator	insulator
Hg3O6S1_ICSD_24147	insulator	insulator
Hg3O6S1_ICSD_96753	insulator	insulator
Hg3O6Te1_ICSD_30325	insulator	insulator
Hg3O6Te1_ICSD_98672	insulator	insulator
Hg3Rb2S8Sn2_ICSD_85599	insulator	insulator
Hg3S4Tl2_ICSD_182745	insulator	insulator
Hg3Sc1_ICSD_104343	metal	metal
Hg3Se4Tl2_ICSD_182746	insulator	insulator
Hg3Sr1_ICSD_247136	metal	metal
Hg3Sr1_ICSD_247140	metal	metal
Hg3Tb1_ICSD_639229	metal	metal
Hg3Te4Tl2_ICSD_182747	insulator	insulator
Hg3Th1_ICSD_639268	metal	metal
Hg3Tl1_ICSD_104362	metal	metal
Hg3U1_ICSD_639292	metal	metal
Hg3Y1_ICSD_104369	metal	metal
Hg3Yb1_ICSD_639305	metal	metal
Hg3Zr1_ICSD_639314	metal	metal
Hg4I3Sb2_ICSD_80588	insulator	insulator
Hg4N2O8_ICSD_59156	insulator	insulator
Hg4N2O8_ICSD_61101	metal	insulator
Hg4Ni1_ICSD_151197	metal	metal
Hg4Pt1_ICSD_655081	metal	metal
Hg4S6Si1_ICSD_95227	insulator	insulator
Hg4Se6Si1_ICSD_95228	metal	insulator
Hg5Mn2_ICSD_104324	metal	metal

Supplementary Table 445. Five-fold cross validated predictions for the metal/insulator classification (437/598).

system	calculated	predicted
Hg5Pd2_ICSD_104334	metal	metal
Hg6O7Si2_ICSD_69123	insulator	insulator
Hg6O8P2_ICSD_410760	insulator	insulator
Hg7K2_ICSD_107482	metal	metal
Hg7O10P2_ICSD_411300	insulator	insulator
Hg7Rb2_ICSD_107483	metal	metal
Hg9Li6Sr17_ICSD_420847	metal	metal
Hg9O16P4_ICSD_411299	insulator	insulator
Ho11In6Si4_ICSD_184238	metal	metal
Ho11Sn10_ICSD_639754	metal	metal
Ho16Mg24Zn122_ICSD_240202	metal	metal
Ho1I1S1_ICSD_425285	insulator	insulator
Ho1In1Ir1_ICSD_414477	metal	metal
Ho1In1Mg1_ICSD_55103	metal	metal
Ho1In1Ni1_ICSD_639339	metal	metal
Ho1In1Pd1_ICSD_639340	metal	metal
Ho1In1Pt1_ICSD_411457	metal	metal
Ho1In1Pt2_ICSD_639343	metal	metal
Ho1In1Pt4_ICSD_639342	metal	metal
Ho1In1Rh1_ICSD_412916	metal	metal
Ho1In1Zn1_ICSD_639346	metal	metal
Ho1In1_ICSD_104410	metal	metal
Ho1In2Ni9_ICSD_600163	metal	metal
Ho1In3_ICSD_657246	metal	metal
Ho1In5Rh1_ICSD_155805	metal	metal
Ho1Ir1Si1_ICSD_93222	metal	metal
Ho1Ir1Si3_ICSD_639360	metal	metal
Ho1Ir1Sn1_ICSD_639364	metal	metal
Ho1Ir1_ICSD_104415	metal	metal
Ho1Ir2Si2_ICSD_639357	metal	metal
Ho1Ir2Si2_ICSD_639359	metal	metal
Ho1Ir2_ICSD_104416	metal	metal
Ho1K1O8W2_ICSD_182626	insulator	insulator
Ho1K1S2_ICSD_44947	insulator	insulator
Ho1Li1O2_ICSD_109172	insulator	insulator
Ho1Li1S2_ICSD_639371	insulator	insulator
Ho1Li1Se2_ICSD_44965	insulator	insulator
Ho1Li1Sn1_ICSD_603145	metal	metal
Ho1Li1Sn2_ICSD_639377	metal	metal
Ho1Mg1Sn1_ICSD_183527	metal	metal
Ho1Mg1_ICSD_161749	metal	metal
Ho1Mg2_ICSD_639381	metal	metal
Ho1Mn12_ICSD_26825	metal	metal
Ho1Mn1O3_ICSD_182632	insulator	insulator
Ho1Mn1Si1_ICSD_83989	metal	metal
Ho1Mn2O5_ICSD_84354	insulator	insulator
Ho1Mn2Si2_ICSD_55813	metal	metal
Ho1Mn2_ICSD_104421	metal	metal
Ho1Mn2_ICSD_180150	metal	metal
Ho1Mn2_ICSD_602152	metal	metal

Supplementary Table 446. Five-fold cross validated predictions for the metal/insulator classification (438/598).

system	calculated	predicted
Ho1Mn2_ICSD_639394	metal	metal
Ho1Mn2_ICSD_639399	metal	metal
Ho1Mn6Sn6_ICSD_240184	metal	metal
Ho1Mn6Sn6_ICSD_57390	metal	metal
Ho1Mo6S8_ICSD_639427	metal	metal
Ho1Mo6Se8_ICSD_639431	metal	metal
Ho1N1_ICSD_169207	insulator	insulator
Ho1N1_ICSD_187360	metal	metal
Ho1Na1S2_ICSD_56229	insulator	insulator
Ho1Na1Se2_ICSD_639444	insulator	insulator
Ho1Nb1O4_ICSD_73392	insulator	insulator
Ho1Ni10Si2_ICSD_97037	metal	metal
Ho1Ni1P1_ICSD_639495	metal	metal
Ho1Ni1Sb1_ICSD_639501	insulator	insulator
Ho1Ni1Sb2_ICSD_658216	metal	metal
Ho1Ni1Si2_ICSD_658584	metal	metal
Ho1Ni1Sn1_ICSD_57391	metal	metal
Ho1Ni1_ICSD_106792	metal	metal
Ho1Ni2P2_ICSD_639493	metal	metal
Ho1Ni2Sb2_ICSD_639499	metal	metal
Ho1Ni2Si2_ICSD_56232	metal	metal
Ho1Ni2_ICSD_639485	metal	metal
Ho1Ni3_ICSD_639455	metal	metal
Ho1Ni4P2_ICSD_601113	metal	metal
Ho1Ni5_ICSD_639458	metal	metal
Ho1Ni5_ICSD_639471	metal	metal
Ho1O2Rb1_ICSD_15160	insulator	insulator
Ho1O3V1_ICSD_10018	insulator	insulator
Ho1O4P1_ICSD_246677	insulator	insulator
Ho1O4Ta1_ICSD_415436	insulator	insulator
Ho1O4Ta1_ICSD_79499	insulator	insulator
Ho1O4V1_ICSD_78079	insulator	insulator
Ho1O6Re1Sr2_ICSD_25403	metal	metal
Ho1O6Rh1Sr3_ICSD_51056	insulator	insulator
Ho1Os2Si2_ICSD_90340	metal	metal
Ho1Os2_ICSD_639530	metal	metal
Ho1P1S1_ICSD_639545	insulator	insulator
Ho1P1_ICSD_187362	metal	metal
Ho1P1_ICSD_43542	metal	metal
Ho1P2Ru2_ICSD_602102	metal	metal
Ho1P5_ICSD_409185	insulator	insulator
Ho1Pb3_ICSD_104431	metal	metal
Ho1Pd1Sb1_ICSD_108547	insulator	metal
Ho1Pd1Sn1_ICSD_639580	metal	metal
Ho1Pd1Sn1_ICSD_99207	metal	metal
Ho1Pd1Tl1_ICSD_104439	metal	metal
Ho1Pd1Zn1_ICSD_183342	metal	metal
Ho1Pd1_ICSD_104433	metal	metal
Ho1Pd1_ICSD_639565	metal	metal
Ho1Pd2Si1_ICSD_639571	metal	metal

Supplementary Table 447. Five-fold cross validated predictions for the metal/insulator classification (439/598).

system	calculated	predicted
Ho1Pd2Si2_ICSD_602292	metal	metal
Ho1Pd2Sn1_ICSD_104437	metal	metal
Ho1Pd3S4_ICSD_639568	metal	metal
Ho1Pd3_ICSD_659826	metal	metal
Ho1Pt1Sb1_ICSD_56237	metal	metal
Ho1Pt1Si1_ICSD_639611	metal	metal
Ho1Pt1Sn1_ICSD_104444	metal	metal
Ho1Pt1_ICSD_639599	metal	metal
Ho1Pt2Si1_ICSD_639610	metal	metal
Ho1Pt2Si2_ICSD_639612	metal	metal
Ho1Pt2_ICSD_104441	metal	metal
Ho1Pt3_ICSD_659827	metal	metal
Ho1Rb1S2_ICSD_81403	insulator	insulator
Ho1Rb1Se2_ICSD_281074	insulator	insulator
Ho1Re2_ICSD_639615	metal	metal
Ho1Rh1Si1_ICSD_56240	metal	metal
Ho1Rh1Sn1_ICSD_639638	metal	metal
Ho1Rh1Zn1_ICSD_419898	metal	metal
Ho1Rh1_ICSD_639620	metal	metal
Ho1Rh2Si2_ICSD_602159	metal	metal
Ho1Rh2_ICSD_104447	metal	metal
Ho1Rh3Si2_ICSD_56243	metal	metal
Ho1Ru1Si1_ICSD_88066	metal	metal
Ho1Ru2Si2_ICSD_639648	metal	metal
Ho1Ru2_ICSD_639647	metal	metal
Ho1S1_ICSD_639662	metal	metal
Ho1S1_ICSD_66357	metal	metal
Ho1S2Tl1_ICSD_639673	insulator	insulator
Ho1S2_ICSD_56245	metal	metal
Ho1S2_ICSD_639660	metal	metal
Ho1Sb1Zr1_ICSD_152642	metal	metal
Ho1Sb1_ICSD_639701	metal	metal
Ho1Sb2_ICSD_26220	metal	metal
Ho1Se1_ICSD_43547	metal	metal
Ho1Se2Tl1_ICSD_639712	insulator	insulator
Ho1Se2_ICSD_639707	metal	metal
Ho1Si1.7_ICSD_415598	metal	metal
Ho1Si1Ti1_ICSD_88117	metal	metal
Ho1Si1_ICSD_154701	metal	metal
Ho1Si1_ICSD_182225	metal	metal
Ho1Si2_ICSD_639748	metal	metal
Ho1Si2_ICSD_639750	metal	metal
Ho1Si3_ICSD_710068	metal	metal
Ho1Sn2Zn1_ICSD_183311	metal	metal
Ho1Sn2_ICSD_168677	metal	metal
Ho1Sn3_ICSD_104449	metal	metal
Ho1Tc2_ICSD_639759	metal	metal
Ho1Te1_ICSD_639766	metal	metal
Ho1Te2Tl1_ICSD_639771	insulator	insulator
Ho1Te2_ICSD_639765	metal	metal

Supplementary Table 448. Five-fold cross validated predictions for the metal/insulator classification (440/598).

system	calculated	predicted
Ho1Tl1_ICSD_104454	metal	metal
Ho1Tl3_ICSD_104456	metal	metal
Ho1Zn12_ICSD_639795	metal	metal
Ho1Zn1_ICSD_639797	metal	metal
Ho1Zn2_ICSD_104460	metal	metal
Ho1Zn3_ICSD_104461	metal	metal
Ho1Zn5_ICSD_639802	metal	metal
Ho1_ICSD_102660	metal	metal
Ho1_ICSD_56224	metal	metal
Ho1_ICSD_56227	metal	metal
Ho1_ICSD_639322	metal	metal
Ho2In1Ni2_ICSD_639335	metal	metal
Ho2In1Ni2_ICSD_639337	metal	metal
Ho2In1Pd2_ICSD_54486	metal	metal
Ho2In1_ICSD_639328	metal	metal
Ho2Mg1Se4_ICSD_639387	insulator	insulator
Ho2Mo3Si4_ICSD_600649	metal	metal
Ho2N3_ICSD_639435	metal	metal
Ho2Ni12P7_ICSD_602484	metal	metal
Ho2Ni2Pb1_ICSD_54612	metal	metal
Ho2Ni2Sn1_ICSD_425430	metal	metal
Ho2Ni3Si5_ICSD_84171	metal	metal
Ho2O2S1_ICSD_109333	insulator	insulator
Ho2O2Se1_ICSD_25809	insulator	insulator
Ho2O3_ICSD_160214	insulator	insulator
Ho2O3_ICSD_41268	insulator	insulator
Ho2O3_ICSD_420710	insulator	insulator
Ho2O7Ru2_ICSD_96730	insulator	insulator
Ho2O7Sn2_ICSD_84748	insulator	insulator
Ho2Pb1Pd2_ICSD_99197	metal	metal
Ho2Pb1S4_ICSD_639552	insulator	insulator
Ho2Pt1_ICSD_639592	metal	metal
Ho2Rh1Si3_ICSD_97373	metal	metal
Ho2S3_ICSD_22252	insulator	insulator
Ho2S3_ICSD_72291	insulator	insulator
Ho2S4Sr1_ICSD_639669	insulator	insulator
Ho2S4Zn1_ICSD_639680	insulator	insulator
Ho2Sb5_ICSD_412685	metal	metal
Ho2Se3_ICSD_408345	metal	metal
Ho2Se4Yb1_ICSD_639718	insulator	insulator
Ho2Zn17_ICSD_639794	metal	metal
Ho2Zn17_ICSD_639799	metal	metal
Ho3In1N1_ICSD_98505	metal	metal
Ho3In5_ICSD_639331	metal	metal
Ho3Ir1_ICSD_639347	metal	metal
Ho3Ir4Si13_ICSD_600540	metal	metal
Ho3Ni1_ICSD_639451	metal	metal
Ho3Ni2_ICSD_639447	metal	metal
Ho3Ni2_ICSD_639449	metal	metal
Ho3Ni6Si2_ICSD_639508	metal	metal

Supplementary Table 449. Five-fold cross validated predictions for the metal/insulator classification (441/598).

system	calculated	predicted
Ho3O3Sb1_ICSD_380458	insulator	insulator
Ho3O6Sc1_ICSD_23480	insulator	insulator
Ho3O7Re1_ICSD_245106	insulator	insulator
Ho3O7Ta1_ICSD_55656	insulator	insulator
Ho3Os1_ICSD_639528	metal	metal
Ho3Os4Si13_ICSD_600537	metal	metal
Ho3Pd2_ICSD_107684	metal	metal
Ho3Pd4_ICSD_639562	metal	metal
Ho3Pr1S6_ICSD_639588	insulator	insulator
Ho3Pt1_ICSD_639596	metal	metal
Ho3Pt4_ICSD_639601	metal	metal
Ho3Ru1_ICSD_639642	metal	metal
Ho3Tl5_ICSD_639785	metal	metal
Ho4In1Ir1_ICSD_418270	metal	metal
Ho4In1Rh1_ICSD_417517	metal	metal
Ho4Sb3_ICSD_639686	metal	metal
Ho5In3_ICSD_639329	metal	metal
Ho5In3_ICSD_639330	metal	metal
Ho5In4Pd2_ICSD_165136	metal	metal
Ho5Ir2_ICSD_639350	metal	metal
Ho5Ir3_ICSD_639351	metal	metal
Ho5Ir3_ICSD_639353	metal	metal
Ho5Ir4Si10_ICSD_639362	metal	metal
Ho5Mg24_ICSD_639380	metal	metal
Ho5Ni1Pb3_ICSD_152605	metal	metal
Ho5Ni2Sb1_ICSD_91136	metal	metal
Ho5Pb3_ICSD_639546	metal	metal
Ho5Pd1Sb2_ICSD_180045	metal	metal
Ho5Pt3_ICSD_639590	metal	metal
Ho5Rh3_ICSD_104448	metal	metal
Ho5Ru2_ICSD_639641	metal	metal
Ho5S7_ICSD_639657	metal	metal
Ho5Sb3_ICSD_56248	metal	metal
Ho5Si3_ICSD_639730	metal	metal
Ho5Sn3_ICSD_104450	metal	metal
Ho5Tl3_ICSD_639784	metal	metal
Ho5Tl3_ICSD_639788	metal	metal
Ho6Mn1Te2_ICSD_182412	metal	metal
Ho6Mn1Te2_ICSD_182414	metal	metal
Ho6Mn23_ICSD_104422	metal	metal
Ho6N2Se6_ICSD_420157	insulator	insulator
Ho6O12W1_ICSD_92038	insulator	insulator
Ho6Zn23_ICSD_639793	metal	metal
Ho7I12Os1_ICSD_424454	metal	metal
I0.33Nb1Se4_ICSD_1092	metal	insulator
I10Ir1Tb6_ICSD_424438	insulator	metal
I10Ir1Y6_ICSD_74650	metal	insulator
I10Ir2Te16_ICSD_425201	insulator	insulator
I10La6Os1_ICSD_409817	insulator	metal
I10Os1Tb6_ICSD_424439	metal	insulator

Supplementary Table 450. Five-fold cross validated predictions for the metal/insulator classification (442/598).

system	calculated	predicted
I10Os1Y6_ICSD_74649	metal	metal
I10Pt1Y6_ICSD_424468	insulator	insulator
I10Ru1Y6_ICSD_74645	insulator	metal
I12K4O1Ti4_ICSD_413504	insulator	insulator
I12La6Os1Sr1_ICSD_94879	metal	metal
I12Nb2Te8_ICSD_78371	insulator	insulator
I12Ru2Te14_ICSD_425196	insulator	insulator
I14K1Zr6_ICSD_201675	metal	insulator
I14Mo6Pb1_ICSD_36574	insulator	insulator
I15La10Os2_ICSD_83856	insulator	metal
I1In1Te1_ICSD_100703	insulator	insulator
I1In1_ICSD_55184	insulator	insulator
I1In3Te3_ICSD_66032	insulator	insulator
I1K1Ni1O6_ICSD_60313	insulator	insulator
I1K1O3_ICSD_28545	metal	insulator
I1K1O3_ICSD_423584	insulator	insulator
I1K1O3_ICSD_424864	insulator	insulator
I1K1O3_ICSD_9329	insulator	insulator
I1K1O3_ICSD_97995	insulator	insulator
I1K1O4_ICSD_83377	insulator	insulator
I1K1_ICSD_22158	insulator	insulator
I1K1_ICSD_61555	insulator	insulator
I1K3O1_ICSD_36512	insulator	insulator
I1K3O5_ICSD_4325	insulator	insulator
I1La1O1_ICSD_24613	insulator	insulator
I1La1_ICSD_83678	metal	metal
I1La2Ni2_ICSD_165262	metal	metal
I1La3S8Si2_ICSD_280940	insulator	insulator
I1La5Pb3_ICSD_409778	metal	metal
I1La5Sn3_ICSD_95240	metal	metal
I1Li1Mo1O6_ICSD_156006	insulator	insulator
I1Li1O3_ICSD_20012	metal	insulator
I1Li1O3_ICSD_20032	insulator	insulator
I1Li1O3_ICSD_2642	insulator	insulator
I1Li1O3_ICSD_40364	insulator	insulator
I1Li1O3_ICSD_41199	insulator	insulator
I1Li1O4_ICSD_400552	insulator	insulator
I1Li1_ICSD_414242	insulator	insulator
I1Li1_ICSD_44275	insulator	insulator
I1Li1_ICSD_44781	insulator	insulator
I1Li6P1S5_ICSD_421083	insulator	insulator
I1Li7N2_ICSD_16799	metal	insulator
I1Lu1O1_ICSD_240948	insulator	insulator
I1Mn1Sb1Se2_ICSD_281558	insulator	insulator
I1N1Sr2_ICSD_172602	insulator	insulator
I1N1Ti1_ICSD_27394	insulator	insulator
I1N1Zn2_ICSD_425735	insulator	insulator
I1N1Zr1_ICSD_27392	insulator	insulator
I1N1Zr1_ICSD_51772	insulator	insulator
I1N3Rb2_ICSD_202077	insulator	insulator

Supplementary Table 451. Five-fold cross validated predictions for the metal/insulator classification (443/598).

system	calculated	predicted
I1Na1Ni1O6_ICSD_23543	insulator	insulator
I1Na1O3_ICSD_20168	insulator	insulator
I1Na1O3_ICSD_33664	metal	insulator
I1Na1O4_ICSD_14287	insulator	insulator
I1Na1_ICSD_52240	insulator	insulator
I1Nb1O2_ICSD_418061	insulator	insulator
I1Nb1Se1_ICSD_201283	insulator	insulator
I1Nb2Te6_ICSD_71516	insulator	insulator
I1Ni2Pr2_ICSD_84702	metal	metal
I1O1Sc1_ICSD_419335	insulator	insulator
I1O1Y1_ICSD_0	insulator	insulator
I1O3Rb1_ICSD_2825	insulator	insulator
I1O3Tl1_ICSD_62106	insulator	insulator
I1O3Tl1_ICSD_76967	metal	insulator
I1O4Rb1_ICSD_89510	insulator	insulator
I1O4Tl1_ICSD_52342	insulator	insulator
I1O5Rb3_ICSD_4326	insulator	insulator
I1O6Rb1Sn1_ICSD_73614	insulator	insulator
I1O7Sb5_ICSD_146	insulator	insulator
I1O7Sb5_ICSD_62369	insulator	insulator
I1P3Se4_ICSD_62673	insulator	insulator
I1Pd1Te1_ICSD_50987	metal	insulator
I1Rb1_ICSD_53831	insulator	insulator
I1Rb1_ICSD_61523	insulator	insulator
I1S1Sb1_ICSD_25572	insulator	insulator
I1S1Sb1_ICSD_28264	insulator	insulator
I1S1Sb1_ICSD_28265	insulator	insulator
I1S1Sb1_ICSD_28305	insulator	insulator
I1S1Sb1_ICSD_41589	insulator	insulator
I1S1Sb1_ICSD_85298	insulator	insulator
I1S1Sb1_ICSD_85299	insulator	insulator
I1S1Sm1_ICSD_2510	metal	insulator
I1S8Si2Tb3_ICSD_411784	insulator	insulator
I1Sb1Se1_ICSD_31292	metal	insulator
I1Sb1Se1_ICSD_35470	insulator	insulator
I1Sb1Te1_ICSD_35471	insulator	insulator
I1Se2Tl5_ICSD_152031	insulator	insulator
I1Se8Ta2_ICSD_35190	metal	insulator
I1Ta1Te4_ICSD_67533	insulator	insulator
I1Te1_ICSD_108	insulator	insulator
I1Te1_ICSD_109	insulator	insulator
I1Tl1_ICSD_55194	insulator	insulator
I1Tl1_ICSD_60491	insulator	insulator
I1Tl1_ICSD_61520	insulator	insulator
I1_ICSD_109039	metal	metal
I1_ICSD_109040	metal	metal
I2K4O9_ICSD_25694	insulator	insulator
I2La1_ICSD_72191	metal	metal
I2La2O4Si1_ICSD_72653	insulator	insulator
I2La2P1_ICSD_418010	metal	metal

Supplementary Table 452. Five-fold cross validated predictions for the metal/insulator classification (444/598).

system	calculated	predicted
I2La2Te1_ICSD_240698	metal	metal
I2Mg1_ICSD_281551	insulator	insulator
I2Mn1_ICSD_33673	insulator	insulator
I2N2S1_ICSD_72363	insulator	insulator
I2Na4O1_ICSD_67112	insulator	insulator
I2Nb1O1_ICSD_36255	insulator	insulator
I2Nd1_ICSD_72190	metal	metal
I2Ni1_ICSD_22108	insulator	insulator
I2O1Sr2_ICSD_409896	insulator	insulator
I2O1Ta1_ICSD_80109	insulator	insulator
I2O2Pb3_ICSD_201857	insulator	insulator
I2O4_ICSD_182670	insulator	insulator
I2O5_ICSD_182671	insulator	insulator
I2O6Pb1_ICSD_74493	insulator	insulator
I2O6Pb3Se2_ICSD_422641	insulator	insulator
I2O6S1_ICSD_6220	insulator	insulator
I2O6Zn1_ICSD_54086	insulator	insulator
I2O7Ti1_ICSD_424632	insulator	insulator
I2O8Rb1V1_ICSD_281304	insulator	insulator
I2O8U1_ICSD_92833	insulator	insulator
I2P4S3_ICSD_26219	insulator	insulator
I2P4S3_ICSD_26485	insulator	insulator
I2P4Se3_ICSD_14281	insulator	insulator
I2P4Se3_ICSD_36365	insulator	insulator
I2Pb1_ICSD_108914	insulator	insulator
I2Pb1_ICSD_23762	insulator	insulator
I2Pb1_ICSD_23763	insulator	insulator
I2Pb1_ICSD_23764	insulator	insulator
I2Pb1_ICSD_24263	insulator	insulator
I2Pb1_ICSD_24264	insulator	insulator
I2Pb1_ICSD_24265	insulator	insulator
I2Pb1_ICSD_60186	insulator	insulator
I2Pb1_ICSD_60327	insulator	insulator
I2Pb1_ICSD_77324	insulator	insulator
I2Pd1Te1_ICSD_409062	insulator	insulator
I2Pd1_ICSD_23169	insulator	insulator
I2Pd1_ICSD_25120	insulator	insulator
I2Pr1_ICSD_10510	metal	metal
I2Pt1_ICSD_60760	insulator	insulator
I2Sn1_ICSD_2831	insulator	insulator
I2Sr1_ICSD_203137	insulator	insulator
I2Sr1_ICSD_37050	insulator	insulator
I2Te2Th1_ICSD_94402	insulator	insulator
I2Th1_ICSD_22233	metal	metal
I2Tm1_ICSD_43731	insulator	metal
I2V1_ICSD_246907	insulator	insulator
I2Yb1_ICSD_77907	insulator	insulator
I2Zn1_ICSD_48016	metal	insulator
I2Zn1_ICSD_77058	insulator	insulator
I2Zr1_ICSD_24807	insulator	insulator

Supplementary Table 453. Five-fold cross validated predictions for the metal/insulator classification (445/598).

system	calculated	predicted
I2Zr1_ICSD_26418	insulator	insulator
I2_ICSD_20329	insulator	insulator
I2_ICSD_67616	insulator	insulator
I3In1O9_ICSD_154654	insulator	insulator
I3In1O9_ICSD_250450	insulator	insulator
I3Ir1Y3_ICSD_71524	metal	metal
I3K1Tm1_ICSD_72343	metal	insulator
I3La3P1_ICSD_411801	metal	metal
I3La3Pb1_ICSD_409796	metal	metal
I3La3Ru1_ICSD_280447	metal	metal
I3La3Sb1_ICSD_411804	metal	metal
I3La4Si4_ICSD_407247	metal	metal
I3La5Si5_ICSD_407248	metal	metal
I3Li1Sc1_ICSD_73339	metal	insulator
I3Mn1Tl1_ICSD_26420	insulator	insulator
I3Na1O8_ICSD_418336	insulator	insulator
I3Nb1O1_ICSD_418088	insulator	insulator
I3Nb1_ICSD_109145	metal	insulator
I3O13Rb1V2_ICSD_281306	insulator	insulator
I3O1W1_ICSD_65183	insulator	insulator
I3O9Sc1_ICSD_411945	insulator	insulator
I3O9Tl1_ICSD_247059	insulator	insulator
I3Os1Pr3_ICSD_84701	metal	metal
I3P1_ICSD_311	insulator	insulator
I3Pb1Tl1_ICSD_1199	insulator	insulator
I3Pb2S2Sb1_ICSD_418344	insulator	insulator
I3Pr3Pt1_ICSD_71515	metal	metal
I3Rb1Sn1_ICSD_400934	insulator	insulator
I3Rb1Yb1_ICSD_86314	insulator	insulator
I3Rb1_ICSD_61348	insulator	insulator
I3Ru1Y3_ICSD_71523	metal	metal
I3S2Sb1Sn2_ICSD_23344	insulator	insulator
I3Sb1_ICSD_30906	insulator	insulator
I3Sb1_ICSD_56572	insulator	insulator
I3Ti1_ICSD_23172	insulator	insulator
I3Tl1_ICSD_61349	insulator	insulator
I3U1_ICSD_1851	insulator	insulator
I3U1_ICSD_201171	insulator	insulator
I3Y1_ICSD_170773	insulator	insulator
I3Zr1_ICSD_32704	metal	metal
I3Zr1_ICSD_74648	metal	insulator
I4In1Li1_ICSD_36599	insulator	insulator
I4In1Na1_ICSD_65463	insulator	insulator
I4In2_ICSD_46034	insulator	insulator
I4Li2Zn1_ICSD_402062	insulator	insulator
I4Mg2O12_ICSD_415820	insulator	insulator
I4Mn2O12_ICSD_415822	insulator	insulator
I4Nb1_ICSD_23916	insulator	insulator
I4O12Zn2_ICSD_415821	insulator	insulator
I4O12Zr1_ICSD_15405	insulator	insulator

Supplementary Table 454. Five-fold cross validated predictions for the metal/insulator classification (446/598).

system	calculated	predicted
I4O12_ICSD_80196	insulator	insulator
I4O1Te9Ti4_ICSD_82142	insulator	insulator
I4O20Tl2U3_ICSD_93862	insulator	insulator
I4P2_ICSD_36293	insulator	insulator
I4Pt1_ICSD_15173	insulator	insulator
I4Rb2Zn1_ICSD_56425	insulator	insulator
I4S1Tl6_ICSD_29265	insulator	insulator
I4Se1Tl6_ICSD_40520	insulator	insulator
I4Si1_ICSD_91745	insulator	insulator
I4Sn1_ICSD_18010	insulator	insulator
I4Sn1_ICSD_38140	insulator	insulator
I4Ta1_ICSD_391406	insulator	insulator
I4Te1_ICSD_37226	insulator	insulator
I4Ti1_ICSD_39820	insulator	insulator
I4Tl2Zn1_ICSD_37099	insulator	insulator
I4U1_ICSD_16464	insulator	insulator
I4Zr1_ICSD_62243	insulator	insulator
I4Zr1_ICSD_8068	insulator	insulator
I5In1Pb2_ICSD_152000	insulator	insulator
I5In1Sn2_ICSD_151995	insulator	insulator
I5In1Sr2_ICSD_152022	insulator	insulator
I5In3Sn1_ICSD_74762	insulator	insulator
I5K1Sn2_ICSD_152010	insulator	insulator
I5K2U1_ICSD_41550	insulator	insulator
I5La2_ICSD_409702	metal	insulator
I5Nb1_ICSD_10457	insulator	insulator
I5Nb1_ICSD_25503	insulator	insulator
I5Pb1Tl3_ICSD_85309	insulator	insulator
I5Pr2_ICSD_56830	metal	insulator
I5Pr4Ru1_ICSD_71161	metal	metal
I6In1O18Rb3_ICSD_422058	insulator	insulator
I6In2_ICSD_23136	insulator	insulator
I6K2Pt1_ICSD_37190	insulator	insulator
I6K2Tc1_ICSD_65510	insulator	insulator
I6K2Te1_ICSD_23649	metal	insulator
I6Mo1O18Rb2_ICSD_413880	insulator	insulator
I6Na2U1_ICSD_78683	insulator	insulator
I6Nb2Se2_ICSD_410743	insulator	insulator
I6Nb2Te2_ICSD_35377	insulator	insulator
I6O18Rb2Zr1_ICSD_414091	insulator	insulator
I6O18Tl4_ICSD_247060	insulator	insulator
I6O1Sr4_ICSD_280585	insulator	insulator
I6Pb1Th1_ICSD_73139	insulator	insulator
I6Pb1Tl4_ICSD_100069	insulator	insulator
I6Pb5S2_ICSD_21040	insulator	insulator
I6Pd1Rb2_ICSD_92478	metal	insulator
I6Pt1Rb2_ICSD_37191	insulator	insulator
I6Pt2_ICSD_47120	insulator	insulator
I6Rb2Sn1_ICSD_22104	metal	insulator
I6Rb2Te1_ICSD_36009	insulator	insulator

Supplementary Table 455. Five-fold cross validated predictions for the metal/insulator classification (447/598).

system	calculated	predicted
I6Rb4Yb1_ICSD_72344	insulator	insulator
I6S1Sn4_ICSD_12165	insulator	insulator
I6Si2_ICSD_83643	insulator	insulator
I6Sn1Th1_ICSD_73138	insulator	insulator
I6Te1Tl2_ICSD_99128	insulator	insulator
I7In1Te1_ICSD_407981	insulator	insulator
I7Nb3Te1_ICSD_86723	insulator	insulator
I7Nb3Te1_ICSD_86724	insulator	insulator
I7Se1Ta3_ICSD_82791	insulator	insulator
I7Ta3Te1_ICSD_82792	insulator	insulator
I8Nb3_ICSD_25767	insulator	insulator
I8Pt3_ICSD_60761	insulator	insulator
I9Re3_ICSD_25114	insulator	insulator
In11La5Ni6_ICSD_639855	metal	metal
In11Nd5Ni6_ICSD_410093	metal	metal
In11Sr3_ICSD_240132	metal	metal
In13Pd6Yb2_ICSD_391130	metal	metal
In14K3Mg20_ICSD_172036	metal	metal
In1Ir1Li2_ICSD_107089	metal	metal
In1Ir1Li2_ICSD_639824	metal	metal
In1Ir1Sm1_ICSD_414471	metal	metal
In1Ir1Tb1_ICSD_414476	metal	metal
In1Ir1Tb4_ICSD_418269	metal	metal
In1Ir1Y4_ICSD_418567	metal	metal
In1Ir2U2_ICSD_602811	metal	metal
In1K1O2_ICSD_380401	insulator	insulator
In1K1O8W2_ICSD_10187	insulator	insulator
In1K1P2S7_ICSD_91755	insulator	insulator
In1K1Te2_ICSD_25347	insulator	insulator
In1K2Na1Sb2_ICSD_77181	insulator	insulator
In1K2P2S7_ICSD_248034	insulator	insulator
In1K3P2_ICSD_73281	insulator	insulator
In1La1Ni1_ICSD_639858	metal	metal
In1La1Ni2_ICSD_106800	metal	metal
In1La1O3_ICSD_281549	insulator	insulator
In1La1Pd1_ICSD_157624	metal	metal
In1La1Pd1_ICSD_639859	metal	metal
In1La1Pd2_ICSD_249793	metal	metal
In1La1Pt1_ICSD_51956	metal	metal
In1La1Pt4_ICSD_639860	metal	metal
In1La1Rh1_ICSD_51957	metal	metal
In1La1Zn1_ICSD_639873	metal	metal
In1La1_ICSD_51948	metal	metal
In1La2Ni2_ICSD_639854	metal	metal
In1La2Pd2_ICSD_180806	metal	metal
In1La2Pd2_ICSD_658291	metal	metal
In1La2Rh2_ICSD_106975	metal	metal
In1La2_ICSD_51949	metal	metal
In1La3N1_ICSD_98494	metal	metal
In1La3_ICSD_639851	metal	metal

Supplementary Table 456. Five-fold cross validated predictions for the metal/insulator classification (448/598).

system	calculated	predicted
In1Li1Mg1Mo3O12_ICSD_63519	insulator	insulator
In1Li1Mo2O8_ICSD_91269	insulator	insulator
In1Li1Mo3O8_ICSD_30579	insulator	insulator
In1Li1O2_ICSD_639886	insulator	insulator
In1Li1O6Si2_ICSD_55178	insulator	insulator
In1Li1O7P2_ICSD_60935	insulator	insulator
In1Li1O8W2_ICSD_423127	insulator	insulator
In1Li1Se2_ICSD_56531	insulator	insulator
In1Li1Se2_ICSD_56532	insulator	insulator
In1Li1Se2_ICSD_656944	insulator	insulator
In1Li1Sn1_ICSD_639905	metal	metal
In1Li1Te2_ICSD_639906	insulator	insulator
In1Li1_ICSD_51959	metal	metal
In1Li2Mg1_ICSD_51965	metal	metal
In1Li2Pd1_ICSD_107088	metal	metal
In1Li2Pt1_ICSD_106805	metal	metal
In1Li2Rh1_ICSD_107087	metal	metal
In1Li2_ICSD_51961	metal	metal
In1Lu1Pd1_ICSD_639921	metal	metal
In1Lu1Pd2_ICSD_51971	metal	metal
In1Lu1Pt1_ICSD_108550	metal	metal
In1Lu2Ni2_ICSD_639917	metal	metal
In1Lu2Ni2_ICSD_639919	metal	metal
In1Lu2Pd2_ICSD_54490	metal	metal
In1Lu2_ICSD_51968	metal	metal
In1Lu3N1_ICSD_98508	metal	metal
In1Mg1Ni2_ICSD_51982	metal	metal
In1Mg1Sm1_ICSD_413808	metal	metal
In1Mg1Tm1_ICSD_55104	metal	metal
In1Mg1Y1_ICSD_160910	metal	metal
In1Mg1_ICSD_51972	metal	metal
In1Mg2_ICSD_51974	metal	metal
In1Mg3_ICSD_109832	metal	metal
In1Mg3_ICSD_51975	metal	metal
In1Mg3_ICSD_639928	metal	metal
In1Mn1Ni2_ICSD_51985	metal	metal
In1Mn1O3_ICSD_67671	metal	insulator
In1Mn1Pd2_ICSD_51988	metal	metal
In1Mn1Ti2_ICSD_189700	insulator	metal
In1Mo1S2_ICSD_603787	metal	metal
In1Mo2O8Rb1_ICSD_10186	insulator	insulator
In1Mo3Se3_ICSD_53098	metal	metal
In1Mo3Te3_ICSD_53100	metal	metal
In1Mo6S8_ICSD_639993	metal	metal
In1Mo6S8_ICSD_639998	metal	metal
In1Mo6Se8_ICSD_23272	metal	metal
In1N1Nd3_ICSD_53101	metal	metal
In1N1Nd3_ICSD_98496	metal	metal
In1N1Ni3_ICSD_247065	metal	metal
In1N1Pr3_ICSD_98495	metal	metal

Supplementary Table 457. Five-fold cross validated predictions for the metal/insulator classification (449/598).

system	calculated	predicted
In1N1Sc3_ICSD_98499	metal	metal
In1N1Tb3_ICSD_98498	metal	metal
In1N1Ti2_ICSD_640030	metal	metal
In1N1Ti3_ICSD_42929	metal	metal
In1N1Tm3_ICSD_98507	metal	metal
In1N1Zr2_ICSD_640031	metal	metal
In1N1_ICSD_157514	metal	metal
In1N1_ICSD_162684	metal	metal
In1N1_ICSD_41501	metal	metal
In1Na1O2_ICSD_34600	insulator	insulator
In1Na1O6Si2_ICSD_183781	insulator	insulator
In1Na1O8W2_ICSD_16263	insulator	insulator
In1Na1S2_ICSD_640037	insulator	insulator
In1Na1Se2_ICSD_25558	insulator	insulator
In1Na1Te2_ICSD_25346	insulator	insulator
In1Na1_ICSD_640032	metal	metal
In1Na3O4Rb2_ICSD_33868	insulator	insulator
In1Na3O8P2_ICSD_87833	insulator	insulator
In1Na5O4_ICSD_69630	insulator	insulator
In1Na5S4_ICSD_300175	insulator	insulator
In1Nb1S2_ICSD_74702	metal	metal
In1Nb1Se2_ICSD_640056	metal	metal
In1Nb3_ICSD_51996	metal	metal
In1Nb3_ICSD_51997	metal	metal
In1Nb3_ICSD_640048	metal	metal
In1Nd1Ni2_ICSD_658679	metal	metal
In1Nd1Pd1_ICSD_153004	metal	metal
In1Nd1Pt1_ICSD_59429	metal	metal
In1Nd1Pt4_ICSD_640081	metal	metal
In1Nd1Rh1_ICSD_59430	metal	metal
In1Nd1Zn1_ICSD_640092	metal	metal
In1Nd2Pd2_ICSD_54481	metal	metal
In1Nd2Rh2_ICSD_106976	metal	metal
In1Nd2_ICSD_59421	metal	metal
In1Nd3_ICSD_640070	metal	metal
In1Ni1O6Sr3_ICSD_81660	metal	insulator
In1Ni1Tb1_ICSD_55844	metal	metal
In1Ni1Th1_ICSD_640155	metal	metal
In1Ni1Ti2_ICSD_185667	metal	metal
In1Ni1Tm1_ICSD_640163	metal	metal
In1Ni1Y1_ICSD_59455	metal	metal
In1Ni1_ICSD_161126	metal	metal
In1Ni1_ICSD_59434	metal	metal
In1Ni2Sc1_ICSD_59446	metal	metal
In1Ni2Sc2_ICSD_107333	metal	metal
In1Ni2Tb2_ICSD_167636	metal	metal
In1Ni2Tb2_ICSD_640151	metal	metal
In1Ni2Ti1_ICSD_59451	metal	metal
In1Ni2Ti2_ICSD_107330	metal	metal
In1Ni2Tm2_ICSD_640161	metal	metal

Supplementary Table 458. Five-fold cross validated predictions for the metal/insulator classification (450/598).

system	calculated	predicted
In1Ni2U1_ICSD_106862	metal	metal
In1Ni2U2_ICSD_246629	metal	metal
In1Ni2Y2_ICSD_640169	metal	metal
In1Ni2Y2_ICSD_640170	metal	metal
In1Ni2Zr1_ICSD_54546	metal	metal
In1Ni2Zr2_ICSD_107372	metal	metal
In1Ni2_ICSD_59437	metal	metal
In1Ni3_ICSD_151196	metal	metal
In1Ni3_ICSD_185623	metal	metal
In1Ni3_ICSD_59439	metal	metal
In1Ni4Tm1_ICSD_415030	metal	metal
In1Ni4U1_ICSD_59454	metal	metal
In1Ni4U1_ICSD_658029	metal	metal
In1Ni4Zr1_ICSD_59462	metal	metal
In1O1Pr3_ICSD_240907	metal	metal
In1O3Y1_ICSD_251	insulator	insulator
In1O4P1_ICSD_16618	insulator	insulator
In1O4P1_ICSD_85579	insulator	insulator
In1O4V1_ICSD_155162	insulator	insulator
In1O4V1_ICSD_80360	insulator	insulator
In1O6Rh1Sr3_ICSD_51138	insulator	insulator
In1O6Sr2Ta1_ICSD_188420	insulator	insulator
In1O8Rb1W2_ICSD_24859	insulator	insulator
In1O8Se2V1_ICSD_261603	insulator	insulator
In1O9P3_ICSD_421261	insulator	insulator
In1P1Pd5_ICSD_640193	metal	metal
In1P1Pt5_ICSD_640194	metal	metal
In1P1S4_ICSD_1699	insulator	insulator
In1P1_ICSD_180911	insulator	metal
In1P1_ICSD_53104	metal	metal
In1P1_ICSD_600858	insulator	metal
In1P2Se6Tl1_ICSD_171212	insulator	insulator
In1P3Sr3_ICSD_65056	insulator	insulator
In1P3_ICSD_37073	metal	insulator
In1Pb1Rh3S2_ICSD_640213	metal	metal
In1Pd1Sr1_ICSD_640249	metal	metal
In1Pd1Tb1_ICSD_640250	metal	metal
In1Pd1Th1_ICSD_640251	metal	metal
In1Pd1U1_ICSD_640255	metal	metal
In1Pd1Y1_ICSD_640260	metal	metal
In1Pd1Yb1_ICSD_415508	metal	metal
In1Pd1_ICSD_59474	metal	metal
In1Pd2Pr2_ICSD_54480	metal	metal
In1Pd2Pu2_ICSD_107353	metal	metal
In1Pd2Sc1_ICSD_59481	metal	metal
In1Pd2Sr2_ICSD_391432	metal	metal
In1Pd2Tb2_ICSD_658297	metal	metal
In1Pd2Th2_ICSD_658304	metal	metal
In1Pd2Tm1_ICSD_59483	metal	metal
In1Pd2Tm2_ICSD_658301	metal	metal

Supplementary Table 459. Five-fold cross validated predictions for the metal/insulator classification (451/598).

system	calculated	predicted
In1Pd2U2_ICSD_106867	metal	metal
In1Pd2Y1_ICSD_59484	metal	metal
In1Pd2Y2_ICSD_658303	metal	metal
In1Pd2Yb2_ICSD_107347	metal	metal
In1Pd2Zr2_ICSD_107332	metal	metal
In1Pd2_ICSD_640234	metal	metal
In1Pd3_ICSD_247189	metal	metal
In1Pd3_ICSD_247195	metal	metal
In1Pd3_ICSD_247197	metal	metal
In1Pd3_ICSD_59476	metal	metal
In1Pr1Pt4_ICSD_640275	metal	metal
In1Pr1Rh1_ICSD_411076	metal	metal
In1Pr1Zn1_ICSD_640282	metal	metal
In1Pr1_ICSD_59487	metal	metal
In1Pr3_ICSD_640265	metal	metal
In1Pt1Sc1_ICSD_411459	metal	metal
In1Pt1Sr1_ICSD_410704	metal	metal
In1Pt1Tb1_ICSD_411458	metal	metal
In1Pt1Th1_ICSD_640313	metal	metal
In1Pt1Tm1_ICSD_59503	metal	metal
In1Pt1U1_ICSD_640320	metal	metal
In1Pt1Y1_ICSD_59504	metal	metal
In1Pt1_ICSD_59491	metal	metal
In1Pt2Pu2_ICSD_150162	metal	metal
In1Pt2Sc1_ICSD_59502	metal	metal
In1Pt2Sr2_ICSD_391433	metal	metal
In1Pt2Tb1_ICSD_640312	metal	metal
In1Pt2U1_ICSD_640317	metal	metal
In1Pt2U2_ICSD_602812	metal	metal
In1Pt2Y1_ICSD_59505	metal	metal
In1Pt2Zr1_ICSD_59506	metal	metal
In1Pt3_ICSD_59494	metal	metal
In1Pt4Sm1_ICSD_640310	metal	metal
In1Pt4Tb1_ICSD_640311	metal	metal
In1Pt5Si1_ICSD_640309	metal	metal
In1Pu1_ICSD_640329	metal	metal
In1Pu2Rh2_ICSD_150163	metal	metal
In1Pu3_ICSD_59510	metal	metal
In1Rb1Te2_ICSD_75346	insulator	insulator
In1Rh1Sm1_ICSD_412918	metal	metal
In1Rh1Tb1_ICSD_412917	metal	metal
In1Rh1Tb4_ICSD_417518	metal	metal
In1Rh1Tm1_ICSD_412914	metal	metal
In1Rh1U1_ICSD_640340	metal	metal
In1Rh1Y1_ICSD_59517	metal	metal
In1Rh1Yb1_ICSD_412913	metal	metal
In1Rh1_ICSD_640334	metal	metal
In1Rh2U2_ICSD_106868	metal	metal
In1Ru3_ICSD_640342	metal	metal
In1S1_ICSD_409645	insulator	insulator

Supplementary Table 460. Five-fold cross validated predictions for the metal/insulator classification (452/598).

system	calculated	predicted
In1S1_ICSD_640349	insulator	insulator
In1S2Ta1_ICSD_640379	metal	metal
In1S2Ta1_ICSD_74703	metal	metal
In1S2Tl1_ICSD_108557	insulator	insulator
In1S2Tl1_ICSD_600748	insulator	insulator
In1S2Tl1_ICSD_600863	insulator	insulator
In1S2Tl1_ICSD_640393	insulator	insulator
In1S2Tl1_ICSD_654688	insulator	insulator
In1S2W1_ICSD_640400	metal	metal
In1S3Sb1_ICSD_300207	insulator	insulator
In1Sb1_ICSD_10022	metal	metal
In1Sb1_ICSD_156995	metal	metal
In1Sb1_ICSD_157947	metal	metal
In1Sb1_ICSD_44641	metal	metal
In1Sb1_ICSD_44642	metal	metal
In1Sb1_ICSD_640414	metal	metal
In1Sb1_ICSD_640432	metal	metal
In1Sb1_ICSD_659843	metal	metal
In1Sc2_ICSD_59522	metal	metal
In1Sc3_ICSD_15908	metal	metal
In1Sc3_ICSD_59523	metal	metal
In1Se1_ICSD_640490	insulator	insulator
In1Se1_ICSD_640503	insulator	insulator
In1Se1_ICSD_640505	insulator	insulator
In1Se1_ICSD_71083	insulator	insulator
In1Se2Ta1_ICSD_640520	metal	metal
In1Se2Tl1_ICSD_600818	insulator	insulator
In1Se2Tl1_ICSD_640528	metal	insulator
In1Si1Te3_ICSD_657680	insulator	insulator
In1Si1Te3_ICSD_66356	metal	insulator
In1Sm1_ICSD_59525	metal	metal
In1Sm2_ICSD_59526	metal	metal
In1Sm3_ICSD_640543	metal	metal
In1Sm3_ICSD_640546	metal	metal
In1Sr3_ICSD_59542	metal	metal
In1Tb2_ICSD_59544	metal	metal
In1Te1_ICSD_169421	metal	metal
In1Te1_ICSD_169431	metal	metal
In1Te1_ICSD_44652	metal	metal
In1Te2Tl1_ICSD_600697	insulator	insulator
In1Th1_ICSD_102123	metal	metal
In1Th2_ICSD_59549	metal	metal
In1Ti2_ICSD_189702	metal	metal
In1Ti3_ICSD_189701	metal	metal
In1Tm1_ICSD_59561	metal	metal
In1Tm2_ICSD_59562	metal	metal
In1Y1_ICSD_640692	metal	metal
In1Y2_ICSD_108567	metal	metal
In1Yb1Zn1_ICSD_640716	metal	metal
In1Yb1_ICSD_640702	metal	metal

Supplementary Table 461. Five-fold cross validated predictions for the metal/insulator classification (453/598).

system	calculated	predicted
In1Yb2_ICSD_59571	metal	metal
In1Zr3_ICSD_640717	metal	metal
In1_ICSD_53777	metal	metal
In24S32_ICSD_640361	metal	insulator
In2Ir1La1_ICSD_414479	metal	metal
In2Ir1Li1_ICSD_106798	metal	metal
In2Ir1Sr1_ICSD_410401	metal	metal
In2La1Ni9_ICSD_600218	metal	metal
In2La1Pd1_ICSD_413448	metal	metal
In2La1Rh1_ICSD_412431	metal	metal
In2La1_ICSD_51951	metal	metal
In2Li1Pd1_ICSD_106803	metal	metal
In2Li1Pt1_ICSD_106804	metal	metal
In2Li1Rh1_ICSD_106806	metal	metal
In2Li1Ru1_ICSD_639896	metal	metal
In2Li2S6Si1_ICSD_262642	insulator	insulator
In2Li2Se6Si1_ICSD_262644	insulator	insulator
In2Li3_ICSD_10051	metal	metal
In2Mg1O4_ICSD_157770	insulator	insulator
In2Mg1S4_ICSD_53096	insulator	insulator
In2Mg1_ICSD_423608	metal	metal
In2Mg5_ICSD_109097	metal	metal
In2Mn1S4_ICSD_639973	insulator	insulator
In2Mn1Se4_ICSD_639980	metal	insulator
In2Mn1Te4_ICSD_60053	insulator	metal
In2Mn1Te4_ICSD_639984	insulator	insulator
In2Mn2O7_ICSD_56517	insulator	metal
In2Na6S6_ICSD_300165	insulator	insulator
In2Ni1S4_ICSD_640128	metal	insulator
In2Ni1Tb1_ICSD_59450	metal	metal
In2Ni1Y1_ICSD_59457	metal	metal
In2Ni1Yb1_ICSD_414077	metal	metal
In2Ni21P6_ICSD_68998	metal	metal
In2Ni3S2_ICSD_640135	metal	metal
In2Ni3Se2_ICSD_640141	metal	metal
In2Ni9Tb1_ICSD_600180	metal	metal
In2Ni9Y1_ICSD_59458	metal	metal
In2O3_ICSD_169429	insulator	insulator
In2O3_ICSD_181834	insulator	insulator
In2O3_ICSD_181836	insulator	insulator
In2O3_ICSD_24325	insulator	insulator
In2O5P1_ICSD_413858	insulator	insulator
In2O5V1_ICSD_417999	metal	insulator
In2O6Te1_ICSD_60260	insulator	insulator
In2O7Si2_ICSD_1458	insulator	insulator
In2O7Si2_ICSD_74897	insulator	insulator
In2P2Sr1_ICSD_260563	insulator	insulator
In2P4Sr3_ICSD_61335	insulator	insulator
In2Pb1S4_ICSD_100687	insulator	insulator
In2Pd1Sr1_ICSD_410402	metal	metal

Supplementary Table 462. Five-fold cross validated predictions for the metal/insulator classification (454/598).

system	calculated	predicted
In2Pd1Yb1_ICSD_410437	metal	metal
In2Pt1Sr1_ICSD_410400	metal	metal
In2Pt1Yb1_ICSD_424100	metal	metal
In2Pt1_ICSD_640307	metal	metal
In2Pt3_ICSD_59498	metal	metal
In2Rb4S5_ICSD_23253	insulator	insulator
In2Rb6S6_ICSD_23252	insulator	insulator
In2Rh1Sm1_ICSD_412430	metal	metal
In2Rh1Sr1_ICSD_410403	metal	metal
In2Rh1Yb1_ICSD_152048	metal	metal
In2Rh3S2_ICSD_640339	metal	metal
In2S3_ICSD_12148	insulator	insulator
In2S3_ICSD_23844	insulator	insulator
In2S4Zn1_ICSD_16200	insulator	metal
In2S4Zn1_ICSD_42668	metal	insulator
In2S4Zn1_ICSD_44637	insulator	insulator
In2S4Zn1_ICSD_603024	insulator	metal
In2S4Zn1_ICSD_81811	insulator	insulator
In2S5Zn2_ICSD_42443	metal	insulator
In2S5Zn2_ICSD_42666	metal	insulator
In2S6Zn3_ICSD_655835	metal	insulator
In2S7Sn3_ICSD_68366	insulator	insulator
In2Sb6Sr5_ICSD_36468	insulator	metal
In2Sb6Yb5_ICSD_91336	metal	metal
In2Se1_ICSD_44646	metal	insulator
In2Se3_ICSD_108563	insulator	insulator
In2Se3_ICSD_1376	insulator	insulator
In2Se3_ICSD_17008	metal	metal
In2Se3_ICSD_602266	insulator	insulator
In2Se3_ICSD_640489	metal	metal
In2Se4Yb1_ICSD_640533	insulator	insulator
In2Se4Zn1_ICSD_25647	insulator	insulator
In2Sr1Te4_ICSD_41167	insulator	insulator
In2Sr1_ICSD_59543	metal	metal
In2Te1_ICSD_640618	metal	metal
In2Te3_ICSD_44364	metal	insulator
In2Te3_ICSD_640609	metal	metal
In2Te4Zn1_ICSD_25650	insulator	insulator
In2Te5_ICSD_640615	insulator	insulator
In2Yb1_ICSD_59572	metal	metal
In3Ir1_ICSD_240201	metal	metal
In3Ir1_ICSD_639819	metal	metal
In3La1_ICSD_639843	metal	metal
In3Li13_ICSD_51963	metal	metal
In3Lu1_ICSD_639910	metal	metal
In3Mg1Sr1_ICSD_249592	metal	metal
In3Mg1_ICSD_51978	metal	metal
In3Na7Se8_ICSD_300178	insulator	insulator
In3Nd1_ICSD_640065	metal	metal
In3Ni2_ICSD_640116	metal	metal

Supplementary Table 463. Five-fold cross validated predictions for the metal/insulator classification (455/598).

system	calculated	predicted
In3O12Sb5_ICSD_68240	insulator	insulator
In3O8P2_ICSD_66831	insulator	insulator
In3Pd2_ICSD_59478	metal	metal
In3Pd5_ICSD_417906	metal	metal
In3Pr1_ICSD_59489	metal	metal
In3Pr1_ICSD_640267	metal	metal
In3Pt2_ICSD_640301	metal	metal
In3Pu1_ICSD_640330	metal	metal
In3Rb1S5_ICSD_59667	insulator	insulator
In3Rb2_ICSD_370026	metal	metal
In3Rh1_ICSD_407549	metal	metal
In3Rh2Sr2_ICSD_410985	metal	metal
In3Rh2Ti3_ICSD_410967	metal	metal
In3Ru1_ICSD_59518	insulator	metal
In3Ru1_ICSD_640343	insulator	metal
In3S5Tl1_ICSD_402124	insulator	insulator
In3Sc1_ICSD_640463	metal	metal
In3Sm1_ICSD_59529	metal	metal
In3Sr1_ICSD_640581	metal	metal
In3Sr5_ICSD_413805	metal	metal
In3Tb1_ICSD_640594	metal	metal
In3Te4_ICSD_44655	metal	metal
In3Th1_ICSD_640646	metal	metal
In3Tm1_ICSD_640670	metal	metal
In3Tm1_ICSD_640675	metal	metal
In3Tm1_ICSD_640678	metal	metal
In3U1_ICSD_640683	metal	metal
In3Y1_ICSD_59568	metal	metal
In3Yb1_ICSD_640714	metal	metal
In3Zr1_ICSD_59578	metal	metal
In4Ir1Sr1_ICSD_417836	metal	metal
In4K1_ICSD_51947	metal	metal
In4La1Ni1_ICSD_411746	metal	metal
In4Li5_ICSD_639879	metal	metal
In4Ni1Sr1_ICSD_418178	metal	metal
In4Ni1Yb1_ICSD_640172	metal	metal
In4Ni2Sc5_ICSD_414505	metal	metal
In4O7Rb2_ICSD_6321	insulator	insulator
In4P8Rb12_ICSD_402811	insulator	insulator
In4Pd1Sr1_ICSD_418179	metal	metal
In4Pd1Yb1_ICSD_411398	metal	metal
In4Pt1Sr1_ICSD_418180	metal	metal
In4Pt1Yb1_ICSD_412908	metal	metal
In4Rb1_ICSD_59514	metal	metal
In4Rh1Yb1_ICSD_411393	metal	metal
In4Rh2Sc5_ICSD_414506	metal	metal
In4S4Sn1_ICSD_36513	insulator	insulator
In4Se3_ICSD_425490	insulator	insulator
In4Se4Sn1_ICSD_56324	insulator	insulator
In4Sr1_ICSD_240131	metal	metal

Supplementary Table 464. Five-fold cross validated predictions for the metal/insulator classification (456/598).

system	calculated	predicted
In4Sr1_ICSD_240135	metal	metal
In4Te3_ICSD_79121	insulator	metal
In4Ti3_ICSD_59553	metal	metal
In5Ir1La1_ICSD_150266	metal	metal
In5Ir1Pr1_ICSD_246916	metal	metal
In5Ir1Yb1_ICSD_413163	metal	metal
In5K1S8_ICSD_40875	insulator	insulator
In5La1Rh1_ICSD_150265	metal	metal
In5La4S13_ICSD_20381	metal	insulator
In5Na24O15_ICSD_63570	metal	insulator
In5Nd1Rh1_ICSD_155802	metal	metal
In5Pr1Rh1_ICSD_246915	metal	metal
In5Rb1S8_ICSD_40877	insulator	insulator
In5Rh1Tb1_ICSD_155803	metal	metal
In5Rh1Yb1_ICSD_412909	metal	metal
In5S6Tl1_ICSD_56388	insulator	insulator
In5S7Tl1_ICSD_402125	insulator	insulator
In5Sb3_ICSD_640434	metal	metal
In5Se7Tl1_ICSD_402126	insulator	insulator
In5Tb3_ICSD_640589	metal	metal
In5Th3_ICSD_59552	metal	metal
In5Ti2_ICSD_401730	metal	metal
In5Ti8_ICSD_240166	metal	metal
In5Y3_ICSD_165364	metal	metal
In6O12Re1_ICSD_245005	insulator	insulator
In6O12Te1_ICSD_245526	insulator	insulator
In6O12W1_ICSD_27197	insulator	insulator
In6S7_ICSD_640348	insulator	insulator
In6Se7_ICSD_640500	insulator	insulator
In6Se7_ICSD_9135	insulator	insulator
In7Pd3_ICSD_408314	metal	metal
In7Pt3_ICSD_409596	metal	metal
In7Sr11_ICSD_159151	metal	metal
In7Te10_ICSD_400669	insulator	insulator
In8Ir1Yb2_ICSD_414480	metal	metal
In8N7Sr19_ICSD_155930	metal	metal
In8Nd2Pd1_ICSD_180117	metal	metal
In8Pd1Pr2_ICSD_180116	metal	metal
In9K1Ni2_ICSD_260252	metal	metal
In9Ni13_ICSD_154798	metal	metal
In9Pt13_ICSD_59501	metal	metal
Ir13Sc57_ICSD_600485	metal	metal
Ir1K0.25O2_ICSD_80336	metal	metal
Ir1K1Li6O6_ICSD_61692	metal	insulator
Ir1K1O3_ICSD_4200	metal	insulator
Ir1K3Li1O4_ICSD_75045	insulator	insulator
Ir1K3N6O12_ICSD_28638	metal	metal
Ir1K4O4_ICSD_47223	insulator	insulator
Ir1La1P1_ICSD_414514	metal	metal
Ir1La1Si1_ICSD_640758	metal	metal

Supplementary Table 465. Five-fold cross validated predictions for the metal/insulator classification (457/598).

system	calculated	predicted
Ir1La1Si3_ICSD_38342	metal	metal
Ir1La3O7_ICSD_181572	insulator	insulator
Ir1La3_ICSD_603213	metal	metal
Ir1La4Mg1_ICSD_418746	metal	metal
Ir1Li1O6Pr2_ICSD_414149	metal	insulator
Ir1Li1O6Sr3_ICSD_81905	metal	insulator
Ir1Li1Sn4_ICSD_412252	metal	metal
Ir1Li1_ICSD_104487	metal	metal
Ir1Li2Mg1_ICSD_180120	metal	metal
Ir1Li2Sn1_ICSD_640769	metal	metal
Ir1Li8O6_ICSD_61217	insulator	insulator
Ir1Lu1Si1_ICSD_413857	metal	metal
Ir1Lu1Sn1_ICSD_640793	metal	metal
Ir1Lu1_ICSD_640773	metal	metal
Ir1Mg1O6Sr2_ICSD_77835	insulator	metal
Ir1Mg1O6Sr3_ICSD_84737	metal	insulator
Ir1Mg1Tb4_ICSD_418743	metal	metal
Ir1Mg3_ICSD_104494	metal	metal
Ir1Mg3_ICSD_106871	metal	metal
Ir1Mn1Sb1_ICSD_44660	metal	metal
Ir1Mn1Si1_ICSD_246548	metal	metal
Ir1Mn1Sn1_ICSD_104498	metal	metal
Ir1Mn1_ICSD_104496	metal	metal
Ir1Mn3N1_ICSD_44659	metal	metal
Ir1Mn3Si1_ICSD_151748	metal	metal
Ir1Mo1_ICSD_104499	metal	metal
Ir1Mo3_ICSD_640823	metal	metal
Ir1N1_ICSD_167880	metal	metal
Ir1N1_ICSD_183155	metal	metal
Ir1N1_ICSD_186241	metal	metal
Ir1N1_ICSD_186244	metal	metal
Ir1N1_ICSD_186245	metal	metal
Ir1N1_ICSD_187717	metal	metal
Ir1N1_ICSD_187719	metal	metal
Ir1N2_ICSD_240755	insulator	metal
Ir1N2_ICSD_240756	metal	metal
Ir1N2_ICSD_290442	metal	metal
Ir1N2_ICSD_290443	metal	metal
Ir1N2_ICSD_290444	metal	metal
Ir1N6Na3O12_ICSD_39572	insulator	insulator
Ir1N6O12Rb3_ICSD_28639	metal	metal
Ir1N6O12Tl3_ICSD_28641	metal	metal
Ir1Na1O3_ICSD_261371	metal	metal
Ir1Na1O6Sr3_ICSD_81906	metal	metal
Ir1Na2O3_ICSD_187130	metal	insulator
Ir1Na4O4_ICSD_67826	insulator	insulator
Ir1Nb1Si1_ICSD_411882	metal	metal
Ir1Nb1Te4_ICSD_656451	metal	metal
Ir1Nb1_ICSD_104510	metal	metal
Ir1Nb1_ICSD_640828	metal	metal

Supplementary Table 466. Five-fold cross validated predictions for the metal/insulator classification (458/598).

system	calculated	predicted
Ir1Nb3_ICSD_640833	metal	metal
Ir1Nd1P1_ICSD_414517	metal	metal
Ir1Nd1Si1_ICSD_640874	metal	metal
Ir1Nd1Si3_ICSD_640872	metal	metal
Ir1Ni1O6Sr3_ICSD_80285	insulator	insulator
Ir1O2_ICSD_640883	metal	metal
Ir1O4Sr2_ICSD_45974	metal	metal
Ir1O6Sr2Tb1_ICSD_90497	metal	insulator
Ir1O6Sr3Zn1_ICSD_82660	insulator	insulator
Ir1O6Sr4_ICSD_72926	metal	metal
Ir1O7Pr3_ICSD_86473	insulator	insulator
Ir1O9P3_ICSD_419717	insulator	insulator
Ir1P1Pr1_ICSD_414516	metal	metal
Ir1P1Sc1_ICSD_422403	metal	metal
Ir1P1Se1_ICSD_640901	insulator	insulator
Ir1P1Sr1_ICSD_155712	metal	metal
Ir1P1Te1_ICSD_601289	insulator	insulator
Ir1P2_ICSD_174231	insulator	insulator
Ir1P3_ICSD_640899	metal	metal
Ir1Pb1_ICSD_104538	metal	metal
Ir1Pt4U1_ICSD_640921	metal	metal
Ir1S1Sb1_ICSD_41400	insulator	insulator
Ir1S1Sb1_ICSD_74630	insulator	insulator
Ir1S2_ICSD_640949	metal	insulator
Ir1S2_ICSD_640950	insulator	insulator
Ir1Sb1Se1_ICSD_640966	insulator	insulator
Ir1Sb1Sm1_ICSD_51843	metal	metal
Ir1Sb1Te1_ICSD_640967	insulator	insulator
Ir1Sb1Yb1_ICSD_422623	metal	metal
Ir1Sb1_ICSD_44481	metal	metal
Ir1Sb2_ICSD_43502	insulator	metal
Ir1Sb3_ICSD_640958	metal	metal
Ir1Sc1Si1_ICSD_420416	metal	metal
Ir1Sc1_ICSD_104557	metal	metal
Ir1Sc2_ICSD_657999	metal	metal
Ir1Sc3Si3_ICSD_421256	metal	metal
Ir1Se2_ICSD_640985	insulator	metal
Ir1Si1Ta1_ICSD_411884	metal	metal
Ir1Si1Tb1_ICSD_93217	metal	metal
Ir1Si1Ti1_ICSD_641017	metal	metal
Ir1Si1U1_ICSD_44665	metal	metal
Ir1Si1Y1_ICSD_641028	metal	metal
Ir1Si1Yb1_ICSD_411736	metal	metal
Ir1Si1Zr1_ICSD_641044	metal	metal
Ir1Si1_ICSD_57397	metal	metal
Ir1Si3Sm1_ICSD_641004	metal	metal
Ir1Si3Tb1_ICSD_641008	metal	metal
Ir1Si3Th1_ICSD_641014	metal	metal
Ir1Si3U1_ICSD_641022	metal	metal
Ir1Si3Y1_ICSD_641038	metal	metal

Supplementary Table 467. Five-fold cross validated predictions for the metal/insulator classification (459/598).

system	calculated	predicted
Ir1Si3_ICSD_26218	metal	metal
Ir1Si3_ICSD_43418	metal	metal
Ir1Sn1U1_ICSD_641059	metal	metal
Ir1Sn1Yb1_ICSD_641062	metal	metal
Ir1Sn1Zr1_ICSD_104563	metal	metal
Ir1Sn1Zr1_ICSD_107470	metal	metal
Ir1Sn1_ICSD_641051	metal	metal
Ir1Sn2_ICSD_641054	metal	metal
Ir1Sn4_ICSD_641053	metal	metal
Ir1Ta1Te4_ICSD_73322	metal	metal
Ir1Ta1_ICSD_641067	metal	metal
Ir1Tb3_ICSD_641077	metal	metal
Ir1Te1_ICSD_44870	metal	metal
Ir1Te2_ICSD_189404	metal	metal
Ir1Te2_ICSD_90086	metal	metal
Ir1Te2_ICSD_93891	metal	metal
Ir1Te2_ICSD_93893	metal	metal
Ir1Te2_ICSD_93894	metal	metal
Ir1Te2_ICSD_93895	metal	metal
Ir1Te2_ICSD_93896	metal	metal
Ir1Th1_ICSD_641097	metal	metal
Ir1Ti1_ICSD_182146	metal	metal
Ir1Ti1_ICSD_188188	metal	metal
Ir1Ti3_ICSD_50296	metal	metal
Ir1Tl3_ICSD_186645	metal	metal
Ir1Tm1_ICSD_104580	metal	metal
Ir1U1_ICSD_104582	metal	metal
Ir1U1_ICSD_104583	metal	metal
Ir1V1_ICSD_169384	metal	metal
Ir1V1_ICSD_169387	metal	metal
Ir1V1_ICSD_169390	metal	metal
Ir1V1_ICSD_169393	metal	metal
Ir1V3_ICSD_641151	metal	metal
Ir1W1_ICSD_104596	metal	metal
Ir1Y1_ICSD_104600	metal	metal
Ir1Y3_ICSD_641173	metal	metal
Ir1Yb1_ICSD_104603	metal	metal
Ir1Zr1_ICSD_104606	metal	metal
Ir1Zr1_ICSD_657956	metal	metal
Ir1Zr2_ICSD_641204	metal	metal
Ir1Zr2_ICSD_641208	metal	metal
Ir1Zr3_ICSD_104607	metal	metal
Ir1_ICSD_64992	metal	metal
Ir2La1Si2_ICSD_188327	metal	metal
Ir2La1Si2_ICSD_604622	metal	metal
Ir2La1_ICSD_640736	metal	metal
Ir2La5_ICSD_640756	metal	metal
Ir2Lu1_ICSD_104491	metal	metal
Ir2Lu5_ICSD_104493	metal	metal
Ir2Mg1_ICSD_414084	metal	metal

Supplementary Table 468. Five-fold cross validated predictions for the metal/insulator classification (460/598).

system	calculated	predicted
Ir2N1Zr4_ICSD_640826	metal	metal
Ir2Nd1Si2_ICSD_640868	metal	metal
Ir2Nd1Si2_ICSD_640869	metal	metal
Ir2Nd1Si2_ICSD_640870	metal	metal
Ir2Nd1Si2_ICSD_640871	metal	metal
Ir2Nd1_ICSD_640859	metal	metal
Ir2O7Pr2_ICSD_156436	metal	metal
Ir2O7Y2_ICSD_187534	metal	metal
Ir2P1_ICSD_640898	metal	metal
Ir2P2Sm1_ICSD_186991	metal	metal
Ir2P2Sr1_ICSD_73531	insulator	insulator
Ir2Pr1_ICSD_104541	metal	metal
Ir2Pu1Si2_ICSD_73039	metal	metal
Ir2Pu1_ICSD_104544	metal	metal
Ir2S3Sn3_ICSD_640953	metal	metal
Ir2S3_ICSD_640948	insulator	insulator
Ir2Sc1_ICSD_104559	metal	metal
Ir2Si1_ICSD_640995	metal	metal
Ir2Si2Sm1_ICSD_641000	metal	metal
Ir2Si2Sm1_ICSD_641003	metal	metal
Ir2Si2Sm3_ICSD_411734	metal	metal
Ir2Si2Tb1_ICSD_55762	metal	metal
Ir2Si2Tb1_ICSD_604511	metal	metal
Ir2Si2Th1_ICSD_603978	metal	metal
Ir2Si2Th1_ICSD_641012	metal	metal
Ir2Si2U1_ICSD_44666	metal	metal
Ir2Si2U1_ICSD_62716	metal	metal
Ir2Si2Y1_ICSD_181769	metal	metal
Ir2Si2Y1_ICSD_641030	metal	metal
Ir2Si2Y1_ICSD_641036	metal	metal
Ir2Sr1_ICSD_104564	metal	metal
Ir2Tb1_ICSD_104569	metal	metal
Ir2Tb5_ICSD_641079	metal	metal
Ir2Th1_ICSD_641102	metal	metal
Ir2Tm1_ICSD_104581	metal	metal
Ir2U1Zn20_ICSD_183636	metal	metal
Ir2U1_ICSD_641143	metal	metal
Ir2Y1_ICSD_641180	metal	metal
Ir2Y5_ICSD_641177	metal	metal
Ir2Yb1_ICSD_104604	metal	metal
Ir2Zn11_ICSD_104605	metal	metal
Ir2Zr1_ICSD_104608	metal	metal
Ir3La1_ICSD_640746	metal	metal
Ir3La2Sb4_ICSD_423170	metal	metal
Ir3La3O11_ICSD_200472	metal	metal
Ir3La5_ICSD_640734	metal	metal
Ir3La7_ICSD_640737	metal	metal
Ir3Li1_ICSD_104488	metal	metal
Ir3Mg13_ICSD_413721	metal	metal
Ir3Mg1Si7_ICSD_249150	metal	metal

Supplementary Table 469. Five-fold cross validated predictions for the metal/insulator classification (461/598).

system	calculated	predicted
Ir3Mg3Si8_ICSD_416868	metal	metal
Ir3Mo1_ICSD_104506	metal	metal
Ir3Nb1_ICSD_104515	metal	metal
Ir3Nd1_ICSD_640856	metal	metal
Ir3Pa1_ICSD_104537	metal	metal
Ir3Pu5_ICSD_978	metal	metal
Ir3S8_ICSD_640951	metal	metal
Ir3Sb4U3_ICSD_640968	metal	metal
Ir3Sc1Si7_ICSD_15244	metal	metal
Ir3Sc1_ICSD_108568	metal	metal
Ir3Se8_ICSD_40823	metal	metal
Ir3Si1_ICSD_54975	metal	metal
Ir3Si4_ICSD_42731	metal	metal
Ir3Si5Y2_ICSD_641029	metal	metal
Ir3Sn7_ICSD_150762	metal	metal
Ir3Ta1_ICSD_641074	metal	metal
Ir3Tb5_ICSD_641080	metal	metal
Ir3Tb5_ICSD_641082	metal	metal
Ir3Th7_ICSD_641096	metal	metal
Ir3Ti1_ICSD_641129	metal	metal
Ir3U1_ICSD_104588	metal	metal
Ir3V1_ICSD_641154	metal	metal
Ir3W1_ICSD_641166	metal	metal
Ir3Y1_ICSD_641184	metal	metal
Ir3Y5_ICSD_641178	metal	metal
Ir3Y5_ICSD_641185	metal	metal
Ir3Yb5_ICSD_641190	metal	metal
Ir3Yb5_ICSD_641191	metal	metal
Ir3Zr1_ICSD_641206	metal	metal
Ir3Zr5_ICSD_641203	metal	metal
Ir4La3Sn13_ICSD_603036	metal	metal
Ir4Lu3Si13_ICSD_600538	metal	metal
Ir4Lu5Si10_ICSD_168655	metal	metal
Ir4Mg29_ICSD_55515	metal	metal
Ir4Nd3Sn13_ICSD_603091	metal	metal
Ir4Pr3Sn13_ICSD_603090	metal	metal
Ir4Pr3Sn13_ICSD_640916	metal	metal
Ir4Sc11_ICSD_10438	metal	metal
Ir4Sc5Si10_ICSD_640980	metal	metal
Ir4Si10Y5_ICSD_641033	metal	metal
Ir4Sm3Sn13_ICSD_603092	metal	metal
Ir4Sn13Sr3_ICSD_641056	metal	metal
Ir4Sn13Yb3_ICSD_641063	metal	metal
Ir4Sn4Sr3_ICSD_410992	metal	metal
Ir5La1_ICSD_640740	metal	metal
Ir5Nd1_ICSD_104521	metal	metal
Ir5Sn7_ICSD_76912	metal	metal
Ir5Th1_ICSD_641108	metal	metal
Ir7La2_ICSD_640739	metal	metal
Ir7Mg44_ICSD_104495	metal	metal

Supplementary Table 470. Five-fold cross validated predictions for the metal/insulator classification (462/598).

system	calculated	predicted
Ir7Sc4Si6_ICSD_640978	metal	metal
K0.33Mo1O3_ICSD_1053	insulator	insulator
K1.33Na0.67O5Si2_ICSD_170786	insulator	insulator
K10Pb48_ICSD_410090	metal	metal
K19.194_ICSD_157569	metal	metal
K19.206_ICSD_157567	metal	metal
K19.214_ICSD_157564	metal	metal
K1La1Na1Nb1O5_ICSD_94743	insulator	insulator
K1La1Na1O5Ta1_ICSD_419855	insulator	insulator
K1La1O12P4_ICSD_161016	insulator	insulator
K1La1O12P4_ICSD_33241	insulator	insulator
K1La1O2_ICSD_27001	insulator	insulator
K1La1O3Pd1_ICSD_417108	insulator	insulator
K1La1O4Ti1_ICSD_261365	insulator	insulator
K1La1S2_ICSD_44942	insulator	insulator
K1La1S4Si1_ICSD_414544	insulator	insulator
K1La1S4Si1_ICSD_603184	insulator	insulator
K1La1Se4Si1_ICSD_603185	insulator	insulator
K1La1Te2_ICSD_98654	insulator	insulator
K1La2Nb1O6_ICSD_160450	insulator	insulator
K1Li1Mn1O2_ICSD_49021	insulator	insulator
K1Li1O1_ICSD_30964	insulator	insulator
K1Li1O2Zn1_ICSD_49022	insulator	insulator
K1Li1O4S1_ICSD_56106	insulator	insulator
K1Li1O4S1_ICSD_71365	insulator	insulator
K1Li1O4S1_ICSD_71366	insulator	insulator
K1Li1O4S1_ICSD_71367	insulator	insulator
K1Li1O4S1_ICSD_88832	insulator	insulator
K1Li1O5Si2_ICSD_82457	insulator	insulator
K1Li1S1_ICSD_290450	insulator	insulator
K1Li1Se1_ICSD_290451	insulator	insulator
K1Li1Te1_ICSD_290452	insulator	insulator
K1Li4Nb1O5_ICSD_73124	insulator	insulator
K1Li6O6Ta1_ICSD_73159	insulator	insulator
K1Lu1O8W2_ICSD_155155	insulator	insulator
K1Mg1O9P3_ICSD_28012	insulator	insulator
K1Mg1P1_ICSD_641231	insulator	insulator
K1Mn1Na1O2_ICSD_61403	insulator	insulator
K1Mn1O4P1_ICSD_78840	insulator	insulator
K1Mn1O4_ICSD_44625	insulator	insulator
K1Mn1O8Se2_ICSD_80430	insulator	insulator
K1Mn1Sb1_ICSD_602269	insulator	metal
K1Mn1Te2_ICSD_87983	metal	metal
K1Mo12S14_ICSD_171363	metal	metal
K1Mo2O8Sc1_ICSD_28019	insulator	insulator
K1Mo2O8Y1_ICSD_20478	insulator	insulator
K1Mo3S3_ICSD_30752	metal	metal
K1Mo3Se3_ICSD_604517	metal	metal
K1Mo3Te3_ICSD_604500	metal	metal
K1Mo4O6_ICSD_68533	metal	metal

Supplementary Table 471. Five-fold cross validated predictions for the metal/insulator classification (463/598).

system	calculated	predicted
K1Mo4O6_ICSD_73807	metal	metal
K1Mo6S8_ICSD_641241	metal	metal
K1N1O2_ICSD_26764	metal	insulator
K1N1O2_ICSD_36202	insulator	insulator
K1N1O2_ICSD_86117	insulator	insulator
K1N1O3_ICSD_28077	insulator	insulator
K1N1O3_ICSD_71398	insulator	insulator
K1N1_ICSD_184991	metal	metal
K1N3_ICSD_1145	metal	insulator
K1N3_ICSD_155168	insulator	insulator
K1Na1Nb1Nd1O5_ICSD_420038	insulator	insulator
K1Na1Nb1O5Pr1_ICSD_420039	insulator	insulator
K1Na1Nd1O5Ta1_ICSD_419856	insulator	insulator
K1Na1O1_ICSD_32743	insulator	insulator
K1Na1O2Zn1_ICSD_38327	insulator	insulator
K1Na1O3Ti1_ICSD_47177	insulator	insulator
K1Na1O4S1_ICSD_26014	insulator	insulator
K1Na1O4S1_ICSD_77343	insulator	insulator
K1Na1O5Pr1Ta1_ICSD_419857	insulator	insulator
K1Na1O6V2_ICSD_2199	insulator	insulator
K1Na1O7S2_ICSD_413050	insulator	insulator
K1Na1S1_ICSD_62658	insulator	insulator
K1Na1Se1_ICSD_67278	insulator	insulator
K1Na1Te1_ICSD_67276	insulator	insulator
K1Na2Ni1O2_ICSD_79878	insulator	insulator
K1Na2Sb1_ICSD_44332	insulator	insulator
K1Na2_ICSD_260784	metal	metal
K1Na3O5W1_ICSD_40249	insulator	insulator
K1Nb1Nd2O6_ICSD_160451	insulator	insulator
K1Nb1O3_ICSD_9532	insulator	insulator
K1Nb1O3_ICSD_9533	insulator	insulator
K1Nb1O3_ICSD_9534	insulator	insulator
K1Nb1S2_ICSD_26286	insulator	insulator
K1Nb1Se2_ICSD_26288	insulator	insulator
K1Nd1O12P4_ICSD_4254	insulator	insulator
K1Nd1O3Pd1_ICSD_417107	insulator	insulator
K1Nd1O8S2_ICSD_200268	insulator	insulator
K1Nd1O8W2_ICSD_9364	insulator	insulator
K1Nd1S2_ICSD_641272	insulator	insulator
K1Nd1Te2_ICSD_98656	insulator	insulator
K1Nd1Te4_ICSD_412792	insulator	metal
K1Ni1O4P1_ICSD_81271	insulator	insulator
K1Ni1O9P3_ICSD_91527	insulator	insulator
K1Ni1P1S4_ICSD_79482	insulator	insulator
K1Ni2S2_ICSD_641273	metal	metal
K1Ni2Se2_ICSD_424686	metal	metal
K1Ni2Se2_ICSD_641276	metal	metal
K1O11V6_ICSD_410568	metal	metal
K1O11V6_ICSD_410569	metal	metal
K1O12P3Th2_ICSD_30432	insulator	insulator

Supplementary Table 472. Five-fold cross validated predictions for the metal/insulator classification (464/598).

system	calculated	predicted
K1O12P3Zr2_ICSD_4427	insulator	insulator
K1O12P4Pr1_ICSD_86289	insulator	insulator
K1O12P4Y1_ICSD_241230	insulator	insulator
K1O12Se2V3_ICSD_81231	insulator	insulator
K1O12Th2V3_ICSD_8232	insulator	insulator
K1O13P4Ta1_ICSD_20735	insulator	insulator
K1O1Tl1_ICSD_1570	insulator	insulator
K1O2Pr1_ICSD_40266	insulator	insulator
K1O2Sb1_ICSD_411214	insulator	insulator
K1O2Sc1_ICSD_34958	insulator	insulator
K1O2Tl1_ICSD_33553	insulator	insulator
K1O2Y1_ICSD_49650	insulator	insulator
K1O2_ICSD_180563	metal	metal
K1O2_ICSD_38246	metal	metal
K1O3Pd2_ICSD_248051	insulator	insulator
K1O3Sb1_ICSD_33546	insulator	insulator
K1O3Sb1_ICSD_41203	insulator	insulator
K1O3Sb1_ICSD_77333	insulator	insulator
K1O3Ta1_ICSD_184922	insulator	insulator
K1O3U1_ICSD_71241	insulator	insulator
K1O3V1_ICSD_1486	insulator	insulator
K1O3_ICSD_47163	insulator	insulator
K1O4P1Sr1_ICSD_83598	insulator	insulator
K1O4P1Zn1_ICSD_88955	insulator	insulator
K1O4Pb1V1_ICSD_80816	insulator	insulator
K1O4Re1_ICSD_72501	insulator	insulator
K1O4Ru1_ICSD_26612	insulator	insulator
K1O4Sr1V1_ICSD_404250	insulator	insulator
K1O4Sr1V1_ICSD_91782	insulator	insulator
K1O4Tc1_ICSD_61	insulator	insulator
K1O5S2Ti2Y2_ICSD_96952	metal	insulator
K1O5Sb3_ICSD_28493	insulator	insulator
K1O6Os2_ICSD_172620	metal	metal
K1O6Os2_ICSD_419880	metal	metal
K1O7P2Y1_ICSD_75171	insulator	insulator
K1O7Sb1Zn4_ICSD_421546	insulator	insulator
K1O8P2Sb1_ICSD_61788	insulator	insulator
K1O8Pr1S2_ICSD_200543	insulator	insulator
K1O8Ru4_ICSD_1562	metal	metal
K1O8S2V1_ICSD_59175	insulator	insulator
K1O8V3_ICSD_421064	insulator	insulator
K1O8W2Y1_ICSD_90378	insulator	insulator
K1O8W2Yb1_ICSD_280877	insulator	insulator
K1P1Pb1S4_ICSD_171379	insulator	insulator
K1P1Pd1S4_ICSD_165315	insulator	insulator
K1P1S5Ti1_ICSD_82262	insulator	insulator
K1P1Se5Ti1_ICSD_81297	insulator	insulator
K1P1Zn1_ICSD_12160	insulator	insulator
K1P1_ICSD_14010	insulator	insulator
K1P2Rh2_ICSD_641292	metal	metal

Supplementary Table 473. Five-fold cross validated predictions for the metal/insulator classification (465/598).

system	calculated	predicted
K1P2S6Sb1_ICSD_409751	insulator	insulator
K1P2S7V1_ICSD_91756	insulator	insulator
K1P2Sb1Se6_ICSD_90152	insulator	insulator
K1P3Zn4_ICSD_262034	insulator	insulator
K1Pb2_ICSD_104613	metal	metal
K1Pr1S2_ICSD_44943	metal	insulator
K1Pr1Se4Si1_ICSD_416622	insulator	insulator
K1Pr1Te2_ICSD_98655	metal	insulator
K1Pr1Te4_ICSD_391204	metal	insulator
K1Rb1S1_ICSD_72326	insulator	insulator
K1S1_ICSD_182223	metal	insulator
K1S2Sb1_ICSD_641324	insulator	insulator
K1S2Sm1_ICSD_44944	metal	insulator
K1S2Sn1_ICSD_23448	metal	metal
K1S2Sn1_ICSD_641326	metal	metal
K1S2Tb1_ICSD_641329	insulator	insulator
K1S2Ti1_ICSD_166489	metal	insulator
K1S2Ti1_ICSD_641335	insulator	metal
K1S2Y1_ICSD_641347	insulator	insulator
K1S2Yb1_ICSD_44949	metal	metal
K1S2Zr1_ICSD_641350	metal	insulator
K1S4Si1Y1_ICSD_602576	insulator	insulator
K1S4Si1Yb1_ICSD_409825	metal	insulator
K1S8V5_ICSD_602318	insulator	insulator
K1Sb1Se2_ICSD_100125	insulator	insulator
K1Sb1Se2_ICSD_44678	insulator	insulator
K1Sb1Sn1_ICSD_40816	insulator	insulator
K1Sb1Zn1_ICSD_12161	insulator	insulator
K1Sb1Zn1_ICSD_44680	insulator	insulator
K1Sb1_ICSD_56529	insulator	insulator
K1Sb2_ICSD_80945	insulator	metal
K1Se2Sm1_ICSD_602818	insulator	metal
K1Se2Yb1_ICSD_409679	metal	metal
K1Se8Ti5_ICSD_602313	insulator	insulator
K1Se8V5_ICSD_602325	metal	insulator
K1Si1_ICSD_641367	insulator	insulator
K1Sm1Te2_ICSD_73547	metal	insulator
K1Te2Y1_ICSD_419995	insulator	insulator
K1Tl1_ICSD_262063	metal	metal
K1Tl1_ICSD_262071	metal	metal
K1Zn13_ICSD_641377	metal	metal
K1_ICSD_161378	metal	metal
K1_ICSD_161379	metal	metal
K1_ICSD_161380	metal	metal
K1_ICSD_165995	metal	metal
K1_ICSD_165996	metal	metal
K1_ICSD_44669	metal	metal
K1_ICSD_44670	metal	metal
K2Li14O14Pb3_ICSD_35250	insulator	insulator
K2Li1O4V1_ICSD_65945	insulator	insulator

Supplementary Table 474. Five-fold cross validated predictions for the metal/insulator classification (466/598).

system	calculated	predicted
K2Li4O6U1_ICSD_65158	insulator	insulator
K2Li6O8Pb2_ICSD_37084	insulator	insulator
K2Mg1P2Se6_ICSD_413168	insulator	insulator
K2Mg2O12S3_ICSD_40986	insulator	insulator
K2Mg2O7Si2_ICSD_185362	insulator	insulator
K2Mn1O4_ICSD_22245	insulator	insulator
K2Mn1O6Se2_ICSD_71540	insulator	insulator
K2Mn1O7V2_ICSD_417892	insulator	insulator
K2Mn1P2S6_ICSD_658727	insulator	insulator
K2Mn1P2Se6_ICSD_165356	insulator	insulator
K2Mn1S2_ICSD_65453	insulator	insulator
K2Mn1Se2_ICSD_65456	insulator	insulator
K2Mn1Te2_ICSD_65459	insulator	insulator
K2Mn2O12S3_ICSD_158733	insulator	insulator
K2Mn2O15Si4Zn4_ICSD_20121	insulator	insulator
K2Mn2O3_ICSD_61038	insulator	insulator
K2Mn3S4_ICSD_411172	insulator	insulator
K2Mo1O1S3_ICSD_423988	insulator	insulator
K2Mo1O2S2_ICSD_423986	insulator	insulator
K2Mo1O2S2_ICSD_423987	insulator	insulator
K2Mo1O3S1_ICSD_423989	insulator	insulator
K2Mo1O4_ICSD_150842	insulator	insulator
K2Mo1S4_ICSD_409563	insulator	insulator
K2Mo2O7_ICSD_20133	insulator	insulator
K2Mo3O10_ICSD_24118	insulator	insulator
K2Mo9S11_ICSD_280261	metal	metal
K2N2O5S1_ICSD_31031	insulator	insulator
K2N6Ni1O12Pb1_ICSD_1937	metal	metal
K2N6Ni1O12Sr1_ICSD_179	insulator	metal
K2Nb5O15Y1_ICSD_82147	insulator	insulator
K2Ni1O2_ICSD_201891	insulator	insulator
K2Ni1P2_ICSD_300119	insulator	insulator
K2Ni2O12S3_ICSD_40987	insulator	insulator
K2Ni3S4_ICSD_641275	insulator	insulator
K2O11Sb4_ICSD_2061	insulator	insulator
K2O12P3Pr1Zr1_ICSD_250163	insulator	insulator
K2O12P3Ti2_ICSD_202888	insulator	insulator
K2O12P4Sr1_ICSD_15725	insulator	insulator
K2O12S3Zn2_ICSD_82873	insulator	insulator
K2O12Te4_ICSD_1728	insulator	insulator
K2O13Ti6_ICSD_184901	insulator	insulator
K2O14Te2U3_ICSD_414064	insulator	insulator
K2O15Si6Ti1_ICSD_158934	insulator	insulator
K2O16V8_ICSD_100596	metal	insulator
K2O1_ICSD_180571	insulator	insulator
K2O2Pd1_ICSD_6158	insulator	insulator
K2O2Zn1_ICSD_34603	insulator	insulator
K2O2_ICSD_36641	insulator	insulator
K2O3Pb1_ICSD_15929	insulator	insulator
K2O3Pb1_ICSD_22063	insulator	insulator

Supplementary Table 475. Five-fold cross validated predictions for the metal/insulator classification (467/598).

system	calculated	predicted
K2O3Pb2_ICSD_1412	insulator	insulator
K2O3S1_ICSD_60762	insulator	insulator
K2O3Sn1_ICSD_16265	insulator	insulator
K2O3Sn2_ICSD_2216	insulator	insulator
K2O3Sn2_ICSD_40463	insulator	insulator
K2O3Te1_ICSD_65640	insulator	insulator
K2O3Ti1_ICSD_162216	insulator	insulator
K2O3Zr1_ICSD_16264	insulator	insulator
K2O4Ru1_ICSD_415749	insulator	insulator
K2O4S1_ICSD_27956	metal	insulator
K2O4U1_ICSD_380361	insulator	insulator
K2O4W1_ICSD_26181	insulator	insulator
K2O4Zn3_ICSD_62146	insulator	insulator
K2O5S2_ICSD_16701	insulator	insulator
K2O5Ti2_ICSD_36097	insulator	insulator
K2O5Zr2_ICSD_18301	insulator	insulator
K2O6S2Se1_ICSD_25027	insulator	insulator
K2O6S2_ICSD_36154	insulator	insulator
K2O6Si2Zn1_ICSD_79705	insulator	insulator
K2O7S2_ICSD_249741	insulator	insulator
K2O7U2_ICSD_202984	insulator	insulator
K2O7W2_ICSD_67284	insulator	insulator
K2O7Zn6_ICSD_1120	insulator	insulator
K2O8P2V1_ICSD_200773	insulator	insulator
K2O8Pb1S2_ICSD_27652	insulator	insulator
K2O8Rb1Tb1V2_ICSD_39369	insulator	insulator
K2O8S2_ICSD_16972	insulator	insulator
K2O8V3_ICSD_1925	insulator	insulator
K2O9Si3Sn1_ICSD_19027	insulator	insulator
K2O9Si3Ti1_ICSD_19025	insulator	insulator
K2O9Si3Ti1_ICSD_412920	insulator	insulator
K2O9Si3Zr1_ICSD_56898	insulator	insulator
K2O9Si4_ICSD_31201	insulator	insulator
K2P2Pd1S6_ICSD_165316	insulator	insulator
K2P2Pd1_ICSD_15584	insulator	insulator
K2P2S6_ICSD_33278	insulator	insulator
K2P2Se6_ICSD_173417	insulator	insulator
K2P2Si1_ICSD_36367	insulator	insulator
K2Pd1S2_ICSD_641296	insulator	insulator
K2Pd1Se2_ICSD_641298	insulator	insulator
K2Pd1Te2_ICSD_641302	insulator	insulator
K2Pd3S4_ICSD_41885	insulator	insulator
K2Pd3S6U1_ICSD_262563	insulator	insulator
K2Pt1S15_ICSD_12164	insulator	insulator
K2Pt1S2_ICSD_26258	insulator	insulator
K2Pt1Se2_ICSD_40430	insulator	insulator
K2Pt1Te2_ICSD_40432	insulator	insulator
K2Pt4S6_ICSD_40062	insulator	insulator
K2Pt4Se6_ICSD_69438	insulator	insulator
K2S1_ICSD_183837	insulator	insulator

Supplementary Table 476. Five-fold cross validated predictions for the metal/insulator classification (468/598).

system	calculated	predicted
K2S1_ICSD_412535	insulator	insulator
K2S2_ICSD_43406	insulator	insulator
K2S3Te1_ICSD_280023	insulator	insulator
K2S3Ti1_ICSD_72377	insulator	insulator
K2S3_ICSD_1263	insulator	insulator
K2S5Sn2_ICSD_73007	insulator	insulator
K2S5_ICSD_641320	insulator	insulator
K2S7Sb4_ICSD_25329	insulator	insulator
K2Sb4Se8_ICSD_402886	insulator	insulator
K2Se1_ICSD_168448	insulator	insulator
K2Se2_ICSD_73172	insulator	insulator
K2Se3Te1_ICSD_63012	insulator	insulator
K2Se3_ICSD_1264	insulator	insulator
K2Se4Sn2_ICSD_153907	metal	insulator
K2Se4W1_ICSD_59242	insulator	insulator
K2Se5Sn2_ICSD_72379	insulator	insulator
K2Se5_ICSD_72380	insulator	insulator
K2Se8Sn4_ICSD_72386	insulator	insulator
K2Sn1Te5_ICSD_36215	insulator	insulator
K2Te1_ICSD_182742	insulator	insulator
K2Te2Zn1_ICSD_420088	insulator	insulator
K2Te2_ICSD_96740	insulator	insulator
K2Te2_ICSD_96741	insulator	insulator
K2Te3U1_ICSD_89546	insulator	insulator
K2Te3Zr1_ICSD_410734	insulator	insulator
K2Te3_ICSD_2453	insulator	insulator
K3Li1Na2O6Te1_ICSD_65978	insulator	insulator
K3Mn1O4_ICSD_108935	insulator	insulator
K3Mn2O8_ICSD_165117	metal	insulator
K3Mn2O8_ICSD_55420	metal	insulator
K3N11P6_ICSD_50211	insulator	insulator
K3N1O3_ICSD_37204	insulator	insulator
K3N1O4_ICSD_36632	insulator	insulator
K3N1_ICSD_99999	metal	insulator
K3N6O12Rh1_ICSD_108777	metal	metal
K3Na1O6Th2_ICSD_38325	insulator	insulator
K3Na1O8Ru2_ICSD_416039	insulator	insulator
K3Na1O8Ru2_ICSD_416040	insulator	insulator
K3Na1O8S2_ICSD_186839	insulator	insulator
K3Na1O8Se2_ICSD_54168	insulator	insulator
K3Nb1O8_ICSD_30405	insulator	insulator
K3Nd1O8P2_ICSD_8274	insulator	insulator
K3Nd1O8V2_ICSD_84234	insulator	insulator
K3Ni1O2_ICSD_262579	insulator	insulator
K3Ni1O2_ICSD_73216	insulator	insulator
K3Ni2O4_ICSD_14157	insulator	insulator
K3O14V5_ICSD_24068	insulator	insulator
K3O3S1V1_ICSD_419400	insulator	insulator
K3O3Sb1_ICSD_279579	insulator	insulator
K3O4Pd2_ICSD_245610	metal	insulator

Supplementary Table 477. Five-fold cross validated predictions for the metal/insulator classification (469/598).

system	calculated	predicted
K3O4Pt2_ICSD_14158	metal	insulator
K3O4Sb1_ICSD_280867	insulator	insulator
K3O4V1_ICSD_108936	insulator	insulator
K3O4V1_ICSD_4138	insulator	insulator
K3O7Sc1Si2_ICSD_413432	insulator	insulator
K3O8Ta1_ICSD_30406	insulator	insulator
K3O8V3_ICSD_100782	insulator	insulator
K3P1S4_ICSD_24599	insulator	insulator
K3P1Se16_ICSD_280849	insulator	insulator
K3P1_ICSD_25550	insulator	insulator
K3P5Ru1Se10_ICSD_406200	insulator	insulator
K3S3Sb1_ICSD_641323	insulator	insulator
K3S4Sb1_ICSD_402642	insulator	insulator
K3S4Sb1_ICSD_41895	insulator	insulator
K3Sb1Se3_ICSD_89607	insulator	insulator
K3Sb1Se4_ICSD_65142	insulator	insulator
K3Sb1Te3_ICSD_300182	insulator	insulator
K3Sb1_ICSD_641351	insulator	insulator
K3Sb1_ICSD_656327	insulator	metal
K4Mo8O52P12_ICSD_35463	insulator	insulator
K4N2O14S4_ICSD_16879	insulator	insulator
K4N6Ni1O12_ICSD_24529	insulator	insulator
K4Na2O6Te1_ICSD_202073	insulator	insulator
K4Na2O6Tl2_ICSD_74956	insulator	insulator
K4Ni3O6_ICSD_426450	insulator	insulator
K4O12W3Zn1_ICSD_200518	insulator	insulator
K4O12Zr5_ICSD_14024	insulator	insulator
K4O21Te2U5_ICSD_153175	insulator	insulator
K4O3Sb2_ICSD_280170	insulator	insulator
K4O4Pb1_ICSD_37268	insulator	insulator
K4O4Sn1_ICSD_158	insulator	insulator
K4O7P2_ICSD_187833	insulator	insulator
K4O7V2_ICSD_250388	insulator	insulator
K4O8P2_ICSD_418250	insulator	insulator
K4P2Pd1S8_ICSD_165312	insulator	insulator
K4P2Zn1_ICSD_67261	insulator	insulator
K4P3_ICSD_64625	metal	insulator
K4P6_ICSD_33259	insulator	insulator
K4P8Te4_ICSD_249937	insulator	insulator
K4S11Ta2_ICSD_410672	insulator	insulator
K4S14Ti3_ICSD_68154	insulator	insulator
K4Se6Sn2_ICSD_300234	insulator	insulator
K4Si4_ICSD_409852	insulator	insulator
K4Sn4_ICSD_409435	insulator	metal
K5.978_ICSD_157565	metal	metal
K5Sb4_ICSD_56530	metal	insulator
K5Te3_ICSD_66024	insulator	insulator
K6Mg1Na14Tl18_ICSD_236347	metal	metal
K6Mg1O4_ICSD_2340	insulator	insulator
K6Mn1S4_ICSD_65448	insulator	insulator

Supplementary Table 478. Five-fold cross validated predictions for the metal/insulator classification (470/598).

system	calculated	predicted
K6Mn1Se4_ICSD_65450	insulator	insulator
K6Mn1Te4_ICSD_65452	insulator	insulator
K6N4O3W2_ICSD_73549	insulator	insulator
K6Na14Tl18Zn1_ICSD_236348	metal	metal
K6O5Pb2_ICSD_74873	insulator	insulator
K6O9Se2_ICSD_240701	insulator	insulator
K6Se6Sn2_ICSD_410863	insulator	insulator
K6Si2Te6_ICSD_1238	insulator	insulator
K6Sn2Te6_ICSD_10109	insulator	insulator
K7Li1Si8_ICSD_61144	insulator	insulator
K8O24Sr2U6_ICSD_91784	insulator	insulator
K8Sb4Sn1_ICSD_44679	insulator	insulator
K8Si46_ICSD_15641	metal	metal
K8Sn46_ICSD_25301	metal	metal
K8Tl11_ICSD_370009	metal	metal
Kr1_ICSD_43726	insulator	insulator
Kr1_ICSD_9785	insulator	insulator
La10O3Si8_ICSD_173575	metal	metal
La1Li1O12P4_ICSD_416877	insulator	insulator
La1Li1O4Ti1_ICSD_91843	insulator	insulator
La1Li1Sn2_ICSD_641391	metal	metal
La1Li2O7Ta2_ICSD_88496	metal	insulator
La1Li3P2_ICSD_49627	insulator	insulator
La1Li3Sb2_ICSD_49625	metal	metal
La1Lu1O3_ICSD_51449	insulator	insulator
La1Mg12_ICSD_168466	metal	metal
La1Mg1Na1O6Te1_ICSD_78532	insulator	insulator
La1Mg1Na1O6W1_ICSD_174386	insulator	insulator
La1Mg1Na1O6W1_ICSD_40497	insulator	insulator
La1Mg1Ni4_ICSD_184164	metal	metal
La1Mg1Pd1_ICSD_657924	metal	metal
La1Mg1Rh1_ICSD_412113	metal	metal
La1Mg1Tl1_ICSD_414439	metal	metal
La1Mg1_ICSD_641411	metal	metal
La1Mg2Ni1_ICSD_96152	metal	metal
La1Mg2Ni9_ICSD_55614	metal	metal
La1Mg2Pd1_ICSD_158253	metal	metal
La1Mg2_ICSD_641407	metal	metal
La1Mg3_ICSD_104661	metal	metal
La1Mn1O1Sb1_ICSD_419355	metal	metal
La1Mn1O3_ICSD_162001	metal	metal
La1Mn1O3_ICSD_188401	metal	metal
La1Mn1O3_ICSD_54919	insulator	metal
La1Mn1O3_ICSD_83761	metal	insulator
La1Mn1Si1_ICSD_85849	metal	metal
La1Mn1Si2_ICSD_104670	metal	metal
La1Mn2Si2_ICSD_80508	metal	metal
La1Mn2Si2_ICSD_80509	metal	metal
La1Mn3Ni2_ICSD_96116	metal	metal
La1Mo2O8Rb1_ICSD_42700	insulator	insulator

Supplementary Table 479. Five-fold cross validated predictions for the metal/insulator classification (471/598).

system	calculated	predicted
La1Mo6S8_ICSD_641455	metal	metal
La1Mo6Se8_ICSD_641459	metal	metal
La1N1O2Si1_ICSD_9752	insulator	insulator
La1N1_ICSD_162194	metal	metal
La1N1_ICSD_162195	insulator	metal
La1N1_ICSD_641466	metal	metal
La1N2O1Ta1_ICSD_411138	insulator	insulator
La1N3O6_ICSD_281587	insulator	insulator
La1Na1O4Ti1_ICSD_82003	insulator	insulator
La1Na1O6Se2_ICSD_68467	insulator	insulator
La1Na1O8S2_ICSD_20354	insulator	insulator
La1Na1Se2_ICSD_641485	insulator	insulator
La1Nb1O4_ICSD_37139	insulator	insulator
La1Nb1O4_ICSD_73390	insulator	insulator
La1Nb1O6Te1_ICSD_413007	insulator	insulator
La1Ni1O1P1_ICSD_391427	metal	metal
La1Ni1O2_ICSD_153058	metal	metal
La1Ni1O3_ICSD_67717	metal	insulator
La1Ni1P1_ICSD_641552	metal	metal
La1Ni1Sb1_ICSD_79979	metal	metal
La1Ni1Sb2_ICSD_658208	metal	metal
La1Ni1Si1_ICSD_78264	metal	metal
La1Ni1Si2_ICSD_658592	metal	metal
La1Ni1Sn1_ICSD_108571	metal	metal
La1Ni1Sn2_ICSD_641595	metal	metal
La1Ni1Zn1_ICSD_416819	metal	metal
La1Ni1_ICSD_641489	metal	metal
La1Ni2P2_ICSD_641551	metal	metal
La1Ni2Rh3_ICSD_183387	metal	metal
La1Ni2Si2_ICSD_641572	metal	metal
La1Ni2Sn2_ICSD_603430	metal	metal
La1Ni2Sn2_ICSD_641594	metal	metal
La1Ni2_ICSD_104673	metal	metal
La1Ni3Rh2_ICSD_183385	metal	metal
La1Ni3_ICSD_641509	metal	metal
La1Ni4Sn2_ICSD_641589	metal	metal
La1Ni5P3_ICSD_38311	metal	metal
La1Ni5Sn1_ICSD_641587	metal	metal
La1Ni5_ICSD_657601	metal	metal
La1Ni9Si2_ICSD_641567	metal	metal
La1Ni9Si4_ICSD_98409	metal	metal
La1O1P1Zn1_ICSD_85777	insulator	insulator
La1O1Sb1Zn1_ICSD_182893	metal	insulator
La1O2Rb1_ICSD_27331	insulator	insulator
La1O3Ru1_ICSD_75569	insulator	metal
La1O3Ti1_ICSD_28908	metal	insulator
La1O3Tl1_ICSD_200088	insulator	insulator
La1O3Tm1_ICSD_419665	insulator	insulator
La1O3V1_ICSD_28925	metal	metal
La1O3V1_ICSD_86557	insulator	insulator

Supplementary Table 480. Five-fold cross validated predictions for the metal/insulator classification (472/598).

system	calculated	predicted
La1O3Y1_ICSD_89455	insulator	insulator
La1O3Yb1_ICSD_15093	insulator	insulator
La1O4P1_ICSD_184549	insulator	insulator
La1O4P1_ICSD_21066	insulator	insulator
La1O4P1_ICSD_31564	insulator	insulator
La1O4P1_ICSD_79747	insulator	insulator
La1O4P1_ICSD_92155	insulator	insulator
La1O4Pd2_ICSD_79340	metal	metal
La1O4Ta1_ICSD_415429	insulator	insulator
La1O4Ta1_ICSD_97688	insulator	insulator
La1O4V1_ICSD_155240	insulator	insulator
La1O4V1_ICSD_400	insulator	insulator
La1O4V1_ICSD_411083	insulator	insulator
La1O7Rb1Ta2_ICSD_81870	insulator	insulator
La1O8Rb1W2_ICSD_27737	insulator	insulator
La1O9P3_ICSD_202640	insulator	insulator
La1O9Sb3_ICSD_55720	insulator	insulator
La1O9V3_ICSD_411084	insulator	insulator
La1Os1Si3_ICSD_641618	metal	metal
La1Os2Si2_ICSD_641620	metal	metal
La1Os2_ICSD_641607	metal	metal
La1Os2_ICSD_641612	metal	metal
La1Os4P12_ICSD_641615	metal	metal
La1Os4Sb12_ICSD_183085	metal	metal
La1P12Ru4_ICSD_641633	metal	metal
La1P1Pd1_ICSD_57405	metal	metal
La1P1Pt1_ICSD_417912	metal	metal
La1P1S1_ICSD_641637	insulator	insulator
La1P1_ICSD_641626	metal	metal
La1P2Rh2_ICSD_641632	metal	metal
La1P2Ru2_ICSD_40795	metal	metal
La1P2_ICSD_42015	insulator	metal
La1P5_ICSD_357	insulator	insulator
La1P5_ICSD_96545	metal	metal
La1P7_ICSD_41938	insulator	insulator
La1Pb1Pd1_ICSD_657940	metal	metal
La1Pb3_ICSD_641640	metal	metal
La1Pd1Sb1_ICSD_641676	metal	metal
La1Pd1Sb2_ICSD_658217	metal	metal
La1Pd1Sn1_ICSD_419190	metal	metal
La1Pd1Sn1_ICSD_422484	metal	metal
La1Pd1Sn2_ICSD_641683	metal	metal
La1Pd1Tl1_ICSD_104699	metal	metal
La1Pd1Zn1_ICSD_183335	metal	metal
La1Pd1_ICSD_641672	metal	metal
La1Pd2Sb2_ICSD_604351	metal	metal
La1Pd2Si2_ICSD_601623	metal	metal
La1Pd3S4_ICSD_61031	metal	metal
La1Pd3_ICSD_641659	metal	metal
La1Pd5_ICSD_104698	metal	metal

Supplementary Table 481. Five-fold cross validated predictions for the metal/insulator classification (473/598).

system	calculated	predicted
La1Pt1Si2_ICSD_57406	metal	metal
La1Pt1Sn1_ICSD_416896	metal	metal
La1Pt1Sn1_ICSD_416901	metal	metal
La1Pt1_ICSD_641701	metal	metal
La1Pt2Si2_ICSD_44700	metal	metal
La1Pt2Si2_ICSD_641715	metal	metal
La1Pt2_ICSD_641687	metal	metal
La1Pt3_ICSD_104701	metal	metal
La1Pt5_ICSD_150749	metal	metal
La1Rb1S2_ICSD_81394	insulator	insulator
La1Rb1S4Si1_ICSD_414545	insulator	insulator
La1Rb1Se2_ICSD_281067	insulator	insulator
La1Rh1Si1_ICSD_641749	metal	metal
La1Rh1Si2_ICSD_602316	metal	metal
La1Rh1Si3_ICSD_604624	metal	metal
La1Rh1Sn1_ICSD_415594	metal	metal
La1Rh1Sn2_ICSD_410732	metal	metal
La1Rh1Sn2_ICSD_641754	metal	metal
La1Rh1Zn1_ICSD_419081	metal	metal
La1Rh1_ICSD_656950	metal	metal
La1Rh2Si2_ICSD_641752	metal	metal
La1Rh2Sn4_ICSD_641757	metal	metal
La1Rh2_ICSD_656951	metal	metal
La1Rh3Si2_ICSD_44708	metal	metal
La1Rh3Si2_ICSD_641743	metal	metal
La1Rh3_ICSD_641723	metal	metal
La1Rh3_ICSD_641724	metal	metal
La1Rh3_ICSD_641738	metal	metal
La1Ru1Si1_ICSD_41249	metal	metal
La1Ru1Si2_ICSD_71960	metal	metal
La1Ru1Si3_ICSD_641787	metal	metal
La1Ru1Sn3_ICSD_104715	metal	metal
La1Ru2Si2_ICSD_641785	metal	metal
La1Ru2_ICSD_104713	metal	metal
La1Ru3Si2_ICSD_100785	metal	metal
La1Ru4Sb12_ICSD_641783	metal	metal
La1S1_ICSD_183951	metal	metal
La1S1_ICSD_641804	metal	metal
La1S1_ICSD_77831	metal	metal
La1S2_ICSD_641839	metal	metal
La1S2_ICSD_656243	metal	insulator
La1S2_ICSD_659874	insulator	insulator
La1S3Yb1_ICSD_99664	metal	insulator
La1Sb1Te1_ICSD_601624	metal	metal
La1Sb1_ICSD_44806	metal	metal
La1Sb1_ICSD_641893	metal	metal
La1Sb2_ICSD_641892	metal	metal
La1Sc1Si1_ICSD_641915	metal	metal
La1Se1.9_ICSD_69730	insulator	insulator
La1Se1_ICSD_183952	metal	metal

Supplementary Table 482. Five-fold cross validated predictions for the metal/insulator classification (474/598).

system	calculated	predicted
La1Se1_ICSD_29395	metal	metal
La1Se1_ICSD_77829	metal	metal
La1Se2_ICSD_641932	metal	metal
La1Se3Yb1_ICSD_99665	metal	insulator
La1Si10_ICSD_246806	metal	metal
La1Si1_ICSD_408030	metal	metal
La1Si1_ICSD_641960	metal	metal
La1Si2_ICSD_641982	metal	metal
La1Si5_ICSD_246809	metal	metal
La1Sn1Zn1_ICSD_152623	metal	metal
La1Sn1_ICSD_641992	metal	metal
La1Sn2_ICSD_657383	metal	metal
La1Sn3_ICSD_151359	metal	metal
La1Te1_ICSD_183953	metal	metal
La1Te1_ICSD_642022	metal	metal
La1Te2_ICSD_23957	metal	metal
La1Te2_ICSD_90911	metal	metal
La1Tl1Zn1_ICSD_642075	metal	metal
La1Tl1_ICSD_104730	metal	metal
La1Tl3_ICSD_104731	metal	metal
La1Zn11_ICSD_150493	metal	metal
La1Zn13_ICSD_642091	metal	metal
La1Zn1_ICSD_104735	metal	metal
La1Zn2_ICSD_642080	metal	metal
La1Zn4_ICSD_262792	metal	metal
La1Zn5_ICSD_159122	metal	metal
La1_ICSD_43568	metal	metal
La1_ICSD_43573	metal	metal
La1_ICSD_43574	metal	metal
La1_ICSD_641383	metal	metal
La2Li2Si3_ICSD_423611	metal	metal
La2Mg17_ICSD_104663	metal	metal
La2Mg1Ni2_ICSD_411081	metal	metal
La2Mn2O3Se2_ICSD_181387	insulator	metal
La2Mo1O12Te3_ICSD_171245	insulator	insulator
La2Mo1O6_ICSD_25611	insulator	insulator
La2Mo2O7_ICSD_202189	metal	insulator
La2Ni1O4_ICSD_56319	insulator	insulator
La2Ni1O4_ICSD_98562	insulator	metal
La2Ni2Zn1_ICSD_416817	metal	metal
La2Ni3_ICSD_660167	metal	metal
La2Ni7_ICSD_641494	metal	metal
La2O12W3_ICSD_78180	insulator	insulator
La2O2S1_ICSD_154583	insulator	insulator
La2O2S2_ICSD_2455	insulator	insulator
La2O2S2_ICSD_68498	insulator	insulator
La2O2Se1_ICSD_25804	insulator	insulator
La2O2Te1_ICSD_89557	insulator	insulator
La2O3_ICSD_160205	insulator	insulator
La2O3_ICSD_160220	insulator	insulator

Supplementary Table 483. Five-fold cross validated predictions for the metal/insulator classification (475/598).

system	calculated	predicted
La2O3_ICSD_184531	insulator	insulator
La2O3_ICSD_26864	insulator	insulator
La2O4Pd1_ICSD_40262	metal	insulator
La2O4Se1Si1_ICSD_69552	insulator	insulator
La2O5Pd2_ICSD_65031	insulator	insulator
La2O5Si1_ICSD_109414	insulator	insulator
La2O6Re1V1_ICSD_169471	metal	metal
La2O6S1_ICSD_66823	insulator	insulator
La2O6Te1_ICSD_62134	insulator	insulator
La2O7Sn2_ICSD_153813	insulator	insulator
La2O7Ti2_ICSD_164027	insulator	insulator
La2O7Zr2_ICSD_154752	insulator	metal
La2O7Zr2_ICSD_184093	metal	insulator
La2O9Si1Ti2_ICSD_75583	insulator	insulator
La2O9W2_ICSD_93721	insulator	insulator
La2Pb1Pd2_ICSD_99190	metal	metal
La2Rh1Si3_ICSD_641751	metal	metal
La2Rh3Si5_ICSD_40427	metal	metal
La2Rh3Sn5_ICSD_104709	metal	metal
La2Rh7_ICSD_641735	metal	metal
La2Rh7_ICSD_641740	metal	metal
La2S3_ICSD_15151	insulator	insulator
La2S5Si1_ICSD_240952	insulator	insulator
La2S5Sn1_ICSD_641853	metal	metal
La2S5Zr1_ICSD_425078	insulator	insulator
La2Sb1_ICSD_423290	metal	metal
La2Se9U2_ICSD_248052	metal	insulator
La2Zn17_ICSD_642087	metal	metal
La3Li5Nb2O12_ICSD_68251	insulator	insulator
La3Li5O12Ta2_ICSD_68252	insulator	insulator
La3Mn1O6S3W1_ICSD_380406	metal	insulator
La3Mn4Sn4_ICSD_656265	metal	metal
La3N1S3_ICSD_416221	insulator	insulator
La3Nb1O7_ICSD_10058	insulator	insulator
La3Ni1O6S3W1_ICSD_380403	metal	insulator
La3Ni1_ICSD_603212	metal	metal
La3Ni2O6_ICSD_249209	metal	metal
La3Ni3Zn1_ICSD_416818	metal	metal
La3O10Os2_ICSD_10104	metal	insulator
La3O10Re2_ICSD_202001	insulator	insulator
La3O11Ru3_ICSD_2206	metal	insulator
La3O3Sb1_ICSD_380456	insulator	insulator
La3O7Os1_ICSD_59664	insulator	insulator
La3O7Ru1_ICSD_416718	insulator	insulator
La3O7Sb1_ICSD_246732	insulator	insulator
La3O7Ta1_ICSD_168909	insulator	insulator
La3O7Ta1_ICSD_55655	insulator	insulator
La3O8Re1_ICSD_38323	insulator	insulator
La3Os1_ICSD_641610	metal	metal
La3Pb13Rh4_ICSD_641650	metal	metal

Supplementary Table 484. Five-fold cross validated predictions for the metal/insulator classification (476/598).

system	calculated	predicted
La3Pd4Zn4_ICSD_182774	metal	metal
La3Pd4_ICSD_641663	metal	metal
La3Pd5Si1_ICSD_260033	metal	metal
La3Pt2_ICSD_641685	metal	metal
La3Pt4Zn4_ICSD_182775	metal	metal
La3Pt4_ICSD_641693	metal	metal
La3Rh2_ICSD_656947	insulator	metal
La3Rh4Sn13_ICSD_102351	metal	metal
La3Ru1_ICSD_641781	metal	metal
La3Ru4Sn13_ICSD_423134	metal	metal
La3S4_ICSD_641834	metal	metal
La3Sb5Ti1_ICSD_80907	metal	metal
La3Sb5Zr1_ICSD_83905	metal	metal
La3Se4_ICSD_641920	metal	metal
La3Si2_ICSD_44696	metal	metal
La3Sn1_ICSD_603209	metal	metal
La3Sn4_ICSD_423418	metal	metal
La3Sn5_ICSD_165185	metal	metal
La3Te4_ICSD_642029	metal	metal
La3Tl1_ICSD_642071	metal	metal
La3Tl5_ICSD_642069	metal	metal
La4Mg1Ru1_ICSD_418013	metal	metal
La4Mn1O1S6_ICSD_391305	insulator	insulator
La4Mn1O1Se6_ICSD_391303	insulator	insulator
La4Mn1S7_ICSD_641433	insulator	insulator
La4N2S3_ICSD_415780	insulator	insulator
La4N2Te3_ICSD_414591	insulator	insulator
La4Ni1S7_ICSD_641558	insulator	insulator
La4O10Re2_ICSD_81	insulator	insulator
La4O12Ti3_ICSD_91952	insulator	insulator
La4O19Os6_ICSD_100099	metal	metal
La4O19Re6_ICSD_22207	metal	metal
La4O19Ru6_ICSD_100098	metal	metal
La4O4Se3_ICSD_419128	insulator	insulator
La4O7Pd1_ICSD_65032	insulator	insulator
La4Pb3_ICSD_641648	metal	metal
La4Rh3_ICSD_656948	metal	metal
La4Sb3_ICSD_641896	metal	metal
La5N1O2S4_ICSD_420918	insulator	insulator
La5Pb3_ICSD_641643	metal	metal
La5Pb4_ICSD_23564	metal	metal
La5Ru2_ICSD_641763	metal	metal
La5Sb3_ICSD_641888	metal	metal
La5Si3_ICSD_641952	metal	metal
La5Si3_ICSD_641974	metal	metal
La5Si4_ICSD_641953	metal	metal
La5Sn1Zn2_ICSD_261925	metal	metal
La5Sn3_ICSD_641987	metal	metal
La5Sn3_ICSD_641990	metal	metal
La5Tl3_ICSD_642068	metal	metal

Supplementary Table 485. Five-fold cross validated predictions for the metal/insulator classification (477/598).

system	calculated	predicted
La6Ni6P17_ICSD_2242	metal	metal
La6O12U1_ICSD_202585	insulator	insulator
La6O18Re4_ICSD_30535	insulator	insulator
La6P17Pd6_ICSD_30850	metal	metal
La7Ni16_ICSD_104682	metal	metal
La7Ni2Zn1_ICSD_159116	metal	metal
La7Ni3_ICSD_104680	metal	metal
La7Pt3_ICSD_641692	metal	metal
La7Rh3_ICSD_656946	metal	metal
La8Ni4O17_ICSD_73921	insulator	insulator
Li0.33S2Ti1_ICSD_31342	metal	metal
Li0.33Se2Ti1_ICSD_31343	metal	metal
Li0.667Mn0.667Ni0.333O2_ICSD_90112	insulator	insulator
Li0.667O5V2_ICSD_25383	metal	insulator
Li10O9Zn4_ICSD_23634	insulator	insulator
Li10Pb3_ICSD_642250	metal	metal
Li12Mg3Si4_ICSD_39596	metal	metal
Li13Si4_ICSD_660135	metal	metal
Li13Sn5_ICSD_104786	metal	metal
Li14O14Pb3Rb2_ICSD_35405	insulator	insulator
Li14O14Pr3Rb2_ICSD_203142	insulator	insulator
Li15Pd4_ICSD_642260	metal	metal
Li15Si4_ICSD_167674	metal	metal
Li16N8Nb2O1_ICSD_174443	insulator	insulator
Li16N8O1Ta2_ICSD_71696	insulator	insulator
Li17Sn4_ICSD_240046	metal	metal
Li1Lu1Sn2_ICSD_642111	metal	metal
Li1Mg1N1_ICSD_167573	insulator	insulator
Li1Mg1O4P1_ICSD_201138	insulator	insulator
Li1Mg1O4V1_ICSD_63477	insulator	insulator
Li1Mg1P1_ICSD_44807	metal	insulator
Li1Mg1Pd1Sb1_ICSD_44808	metal	metal
Li1Mg1Pd1Sn1_ICSD_16478	metal	metal
Li1Mg1Pt1Sb1_ICSD_44809	metal	metal
Li1Mg1Pt1Sn1_ICSD_104749	metal	metal
Li1Mg2Si1_ICSD_181252	metal	metal
Li1Mg2Tl1_ICSD_104752	metal	metal
Li1Mg4O12V3_ICSD_250192	insulator	insulator
Li1Mg8Si4_ICSD_181249	metal	metal
Li1Mn1O2_ICSD_154823	insulator	insulator
Li1Mn1O2_ICSD_15768	metal	insulator
Li1Mn1O2_ICSD_40486	insulator	insulator
Li1Mn1O2_ICSD_98164	insulator	metal
Li1Mn1O4P1_ICSD_97763	insulator	insulator
Li1Mn1O4V1_ICSD_247772	insulator	insulator
Li1Mn1O4_ICSD_73115	insulator	insulator
Li1Mn1O7P2_ICSD_415153	insulator	insulator
Li1Mn1Se2_ICSD_50817	metal	metal
Li1Mn1Te2_ICSD_110773	metal	metal
Li1Mn2O4_ICSD_89459	metal	insulator

Supplementary Table 486. Five-fold cross validated predictions for the metal/insulator classification (478/598).

system	calculated	predicted
Li1Mo1O2_ICSD_165326	metal	metal
Li1Mo1O2_ICSD_183608	metal	insulator
Li1Mo1O4Rb1_ICSD_20641	insulator	insulator
Li1Mo1O7P2_ICSD_68522	insulator	insulator
Li1Mo1S2_ICSD_150688	insulator	metal
Li1Mo2O8Sb1_ICSD_80595	insulator	insulator
Li1Mo2O8Tl3_ICSD_84291	insulator	insulator
Li1Mo2O8Y1_ICSD_23313	insulator	insulator
Li1Mo3O8Sc1_ICSD_28525	insulator	insulator
Li1Mo3O8Y1_ICSD_28526	insulator	insulator
Li1Mo3Se3_ICSD_604519	metal	metal
Li1Mo6S8_ICSD_642160	metal	metal
Li1Mo6Se8_ICSD_642167	metal	metal
Li1N1Na2_ICSD_92310	metal	metal
Li1N1Ni1_ICSD_247028	metal	metal
Li1N1O1Si1_ICSD_34106	insulator	insulator
Li1N1O3_ICSD_67981	insulator	insulator
Li1N1Sr1_ICSD_87414	insulator	insulator
Li1N1Zn1_ICSD_16790	insulator	insulator
Li1N2Na5_ICSD_92315	metal	metal
Li1N2Na5_ICSD_92316	metal	metal
Li1N2P1_ICSD_32713	insulator	insulator
Li1N2U1_ICSD_98663	insulator	metal
Li1N3Si2_ICSD_89524	insulator	insulator
Li1N3_ICSD_155166	insulator	insulator
Li1N4Re1Sr2_ICSD_411454	insulator	insulator
Li1Na1O4S1_ICSD_14364	insulator	insulator
Li1Na1O6V2_ICSD_20836	insulator	insulator
Li1Na1Se1_ICSD_67359	insulator	insulator
Li1Na1Te1_ICSD_67274	insulator	insulator
Li1Nb1O2_ICSD_300243	insulator	insulator
Li1Nb1O3_ICSD_169693	insulator	insulator
Li1Nb1O3_ICSD_28298	insulator	insulator
Li1Nb1O4Zn1_ICSD_75526	insulator	insulator
Li1Nb1O4Zn1_ICSD_75597	insulator	insulator
Li1Nb1O4Zn1_ICSD_85735	insulator	insulator
Li1Nb1O6Sr3_ICSD_380359	insulator	insulator
Li1Nb1O6W1_ICSD_202779	insulator	insulator
Li1Nb1S2_ICSD_26284	insulator	metal
Li1Nb3O8_ICSD_2921	insulator	insulator
Li1Nd1O4Ti1_ICSD_91844	insulator	insulator
Li1Nd1Sn1_ICSD_54393	metal	metal
Li1Nd1Sn2_ICSD_642207	metal	metal
Li1Nd2Si2_ICSD_642206	metal	metal
Li1Ni1O2_ICSD_164803	metal	insulator
Li1Ni1O2_ICSD_164853	insulator	insulator
Li1Ni1O4P1_ICSD_184770	insulator	insulator
Li1Ni1O4P1_ICSD_97767	insulator	insulator
Li1Ni1O8Rb5Si2_ICSD_40269	insulator	insulator
Li1Ni2P2_ICSD_36511	metal	metal

Supplementary Table 487. Five-fold cross validated predictions for the metal/insulator classification (479/598).

system	calculated	predicted
Li1Ni2Si1_ICSD_44819	metal	metal
Li1Ni2Sn1_ICSD_25325	metal	metal
Li1Ni6Si6_ICSD_41464	metal	metal
Li1O12P3Sn2_ICSD_83832	insulator	insulator
Li1O12P3Zr2_ICSD_201935	insulator	insulator
Li1O12P4Tb1_ICSD_240703	insulator	insulator
Li1O12Te2V3_ICSD_249325	insulator	insulator
Li1O13V6_ICSD_280870	metal	metal
Li1O1Rb1_ICSD_65170	insulator	insulator
Li1O2Pr1_ICSD_56752	insulator	insulator
Li1O2Rh1_ICSD_29213	insulator	metal
Li1O2Rh1_ICSD_59179	insulator	insulator
Li1O2Ru1_ICSD_48007	metal	insulator
Li1O2Sc1_ICSD_31316	insulator	insulator
Li1O2Tb1_ICSD_21013	insulator	insulator
Li1O2Ti1_ICSD_164158	metal	insulator
Li1O2Ti1_ICSD_48128	metal	insulator
Li1O2Tl1_ICSD_33633	insulator	insulator
Li1O2V1_ICSD_202541	insulator	insulator
Li1O2Y1_ICSD_45511	insulator	insulator
Li1O2Yb1_ICSD_33955	metal	insulator
Li1O2_ICSD_180561	metal	insulator
Li1O2_ICSD_642216	metal	metal
Li1O3Rb1Zn2_ICSD_67145	insulator	insulator
Li1O3Re1_ICSD_35012	metal	insulator
Li1O3Sb1_ICSD_24051	insulator	insulator
Li1O3Ta1_ICSD_9537	insulator	insulator
Li1O3U1_ICSD_22310	insulator	insulator
Li1O3V1_ICSD_23477	insulator	insulator
Li1O3V1_ICSD_2899	insulator	insulator
Li1O3_ICSD_180565	metal	insulator
Li1O4P1Pb1_ICSD_39206	insulator	insulator
Li1O4P1Zn1_ICSD_79352	metal	insulator
Li1O4Rb1S1_ICSD_174059	insulator	insulator
Li1O4Rb2V1_ICSD_40218	insulator	insulator
Li1O4Ru2_ICSD_290491	insulator	metal
Li1O4Si1Y1_ICSD_34079	metal	insulator
Li1O4Ti2_ICSD_78482	metal	insulator
Li1O4V2_ICSD_89352	insulator	metal
Li1O5P1Ti1_ICSD_39534	insulator	insulator
Li1O5P1Ti1_ICSD_39761	insulator	insulator
Li1O5Si1Ta1_ICSD_39648	insulator	insulator
Li1O5V2_ICSD_23817	insulator	insulator
Li1O5V2_ICSD_27245	insulator	insulator
Li1O5V2_ICSD_50982	insulator	insulator
Li1O5V2_ICSD_88639	insulator	insulator
Li1O6Re1Sr2_ICSD_246730	insulator	insulator
Li1O6Re1Sr2_ICSD_418991	insulator	insulator
Li1O6Ru1Sr3_ICSD_50022	insulator	insulator
Li1O6Sb1W1_ICSD_202748	insulator	insulator

Supplementary Table 488. Five-fold cross validated predictions for the metal/insulator classification (480/598).

system	calculated	predicted
Li1O6Sc1Si2_ICSD_152075	insulator	insulator
Li1O6Sc1Si2_ICSD_152079	insulator	insulator
Li1O6Si2Ti1_ICSD_96292	insulator	insulator
Li1O6Si2V1_ICSD_55164	insulator	insulator
Li1O6Sr3Ta1_ICSD_380360	insulator	insulator
Li1O7P2Sc1_ICSD_91496	insulator	insulator
Li1O7P2Ti1_ICSD_166164	insulator	insulator
Li1O7P2V1_ICSD_80551	insulator	insulator
Li1O8Pr1W2_ICSD_200520	insulator	insulator
Li1O8Ta3_ICSD_1318	insulator	insulator
Li1O8V3_ICSD_16689	insulator	insulator
Li1O9P3Pb1_ICSD_1123	insulator	insulator
Li1P1S4Zn1_ICSD_95785	insulator	insulator
Li1P1Sr1_ICSD_416889	insulator	insulator
Li1P1Sr1_ICSD_56443	insulator	insulator
Li1P1Zn1_ICSD_44824	metal	insulator
Li1P1Zn1_ICSD_642242	insulator	metal
Li1P1_ICSD_642222	insulator	insulator
Li1P5_ICSD_88710	insulator	insulator
Li1P7_ICSD_23621	insulator	insulator
Li1Pb1Pd2_ICSD_104767	metal	metal
Li1Pb1Yb1_ICSD_409534	metal	metal
Li1Pb1_ICSD_104763	metal	metal
Li1Pb1_ICSD_642249	metal	metal
Li1Pd1Si3_ICSD_39861	metal	metal
Li1Pd1_ICSD_104770	metal	metal
Li1Pd1_ICSD_642257	metal	metal
Li1Pd2Sn1_ICSD_642271	metal	metal
Li1Pd2Sn6_ICSD_152299	metal	metal
Li1Pd2Tl1_ICSD_54365	metal	metal
Li1Pd7_ICSD_642266	metal	metal
Li1Pr1Sn1_ICSD_54392	metal	metal
Li1Pr1Sn2_ICSD_642277	metal	metal
Li1Pt1_ICSD_104777	metal	metal
Li1Pt2_ICSD_104778	metal	metal
Li1Pt7_ICSD_642281	metal	metal
Li1Rb2S4V1_ICSD_414185	insulator	insulator
Li1Rh1Sn4_ICSD_412251	metal	metal
Li1Rh1_ICSD_150551	metal	metal
Li1Rh2Si2_ICSD_420085	metal	metal
Li1Rh3_ICSD_642290	metal	metal
Li1Ru1Sn4_ICSD_412250	metal	metal
Li1S1_ICSD_182221	metal	insulator
Li1S2Sb1_ICSD_40457	insulator	insulator
Li1S2Sc1_ICSD_642305	insulator	insulator
Li1S2Sn1_ICSD_23451	metal	metal
Li1S2Sn1_ICSD_642314	metal	insulator
Li1S2Ti1_ICSD_189826	metal	insulator
Li1S2Ti1_ICSD_642322	insulator	metal
Li1S2V1_ICSD_16303	metal	metal

Supplementary Table 489. Five-fold cross validated predictions for the metal/insulator classification (481/598).

system	calculated	predicted
Li1S2V1_ICSD_642325	metal	metal
Li1S2Y1_ICSD_44957	insulator	insulator
Li1S2Yb1_ICSD_642336	metal	insulator
Li1Sb1Sn1_ICSD_603169	metal	metal
Li1Sb1Sr1_ICSD_172216	insulator	insulator
Li1Sb1Zn1_ICSD_642350	insulator	metal
Li1Se2Tb1_ICSD_44962	insulator	insulator
Li1Se2Y1_ICSD_44963	insulator	insulator
Li1Se2Zr1_ICSD_642364	metal	insulator
Li1Si1Y1_ICSD_262362	metal	metal
Li1Si1_ICSD_78364	metal	metal
Li1Si2Y2_ICSD_8160	metal	metal
Li1Sm1Sn2_ICSD_642381	metal	metal
Li1Sn1Y1_ICSD_32041	metal	metal
Li1Sn1_ICSD_104782	metal	metal
Li1Sn2Tb1_ICSD_642386	metal	metal
Li1Te2Ti1_ICSD_44908	insulator	metal
Li1Te3_ICSD_935	insulator	insulator
Li1Tl1_ICSD_104789	metal	metal
Li1Tl1_ICSD_262068	metal	metal
Li1Tl1_ICSD_262070	metal	metal
Li1Zn13_ICSD_421842	metal	metal
Li1Zn1_ICSD_642409	metal	metal
Li1_ICSD_109011	metal	metal
Li1_ICSD_109012	metal	metal
Li1_ICSD_161377	metal	metal
Li1_ICSD_182499	metal	metal
Li1_ICSD_44613	metal	metal
Li1_ICSD_44760	metal	metal
Li1_ICSD_57408	metal	metal
Li1_ICSD_642105	metal	metal
Li21Si5_ICSD_167675	metal	metal
Li22Sn5_ICSD_642384	metal	metal
Li23Sr6_ICSD_104788	metal	metal
Li2Mg1Mn3O8_ICSD_90648	insulator	insulator
Li2Mg1Pb1_ICSD_174025	metal	metal
Li2Mg1Si1_ICSD_167141	insulator	metal
Li2Mg1Si1_ICSD_181255	insulator	metal
Li2Mg1Tl1_ICSD_104753	metal	metal
Li2Mg8Si4_ICSD_181250	metal	metal
Li2Mn1O2_ICSD_37327	metal	insulator
Li2Mn1O3_ICSD_21022	insulator	insulator
Li2Mn1O4Si1_ICSD_161305	insulator	insulator
Li2Mn3O8Zn1_ICSD_26995	insulator	insulator
Li2N1Na1_ICSD_92308	metal	insulator
Li2N1O2P1_ICSD_188494	insulator	insulator
Li2N2Na4_ICSD_92309	insulator	metal
Li2N2Th1_ICSD_16232	insulator	insulator
Li2N2U1_ICSD_642191	metal	insulator
Li2N2Zr1_ICSD_78790	insulator	insulator

Supplementary Table 490. Five-fold cross validated predictions for the metal/insulator classification (482/598).

system	calculated	predicted
Li2N2_ICSD_423831	metal	insulator
Li2N5Ta3_ICSD_40568	metal	insulator
Li2Na1Sb1_ICSD_57410	insulator	insulator
Li2Na6O10W2_ICSD_49027	insulator	insulator
Li2Nb2O7Sr1_ICSD_88463	insulator	insulator
Li2Nb2O7Sr1_ICSD_88467	insulator	insulator
Li2Nd1Sb2_ICSD_36020	metal	metal
Li2Ni12P7_ICSD_39598	metal	metal
Li2Ni1O2_ICSD_25000	insulator	insulator
Li2Ni1O2_ICSD_73874	insulator	insulator
Li2Ni1O3_ICSD_153094	insulator	insulator
Li2Ni1O8W2_ICSD_92853	insulator	insulator
Li2O13Ti6_ICSD_182966	insulator	insulator
Li2O13V6_ICSD_59305	metal	metal
Li2O1_ICSD_108886	insulator	insulator
Li2O1_ICSD_642219	insulator	insulator
Li2O20P4U3_ICSD_246191	insulator	insulator
Li2O2Pd1_ICSD_19007	insulator	insulator
Li2O2_ICSD_180557	insulator	insulator
Li2O2_ICSD_50658	insulator	insulator
Li2O3Pb1_ICSD_35182	insulator	insulator
Li2O3Pr1_ICSD_154704	insulator	insulator
Li2O3Re1_ICSD_200999	insulator	insulator
Li2O3Ru1_ICSD_202611	insulator	insulator
Li2O3Ru1_ICSD_78721	insulator	insulator
Li2O3Si1_ICSD_853	insulator	insulator
Li2O3Sn1_ICSD_21053	insulator	insulator
Li2O3Te1_ICSD_38416	insulator	insulator
Li2O3Te1_ICSD_4317	insulator	insulator
Li2O3Ti1_ICSD_261239	insulator	insulator
Li2O3Zr1_ICSD_31941	insulator	insulator
Li2O3Zr1_ICSD_94896	insulator	insulator
Li2O4Rb2Si1_ICSD_61086	insulator	insulator
Li2O4S1_ICSD_153807	insulator	insulator
Li2O4S1_ICSD_30276	insulator	insulator
Li2O4Si1Sr1_ICSD_167334	insulator	insulator
Li2O4Te1_ICSD_1485	insulator	insulator
Li2O4U1_ICSD_200297	insulator	insulator
Li2O4U1_ICSD_20578	insulator	insulator
Li2O4W1_ICSD_10479	insulator	insulator
Li2O4W1_ICSD_14196	insulator	insulator
Li2O4W1_ICSD_15395	insulator	insulator
Li2O5Si1Ti1_ICSD_78059	insulator	insulator
Li2O5Si1V1_ICSD_86775	insulator	insulator
Li2O5Si2_ICSD_78562	insulator	insulator
Li2O6Sn1Te1_ICSD_40265	insulator	insulator
Li2O6Te1Zr1_ICSD_71489	insulator	insulator
Li2O7Sr1Ta2_ICSD_246277	insulator	insulator
Li2O7Sr1Ta2_ICSD_88464	insulator	metal
Li2O7Ti3_ICSD_426263	insulator	insulator

Supplementary Table 491. Five-fold cross validated predictions for the metal/insulator classification (483/598).

system	calculated	predicted
Li2O7W2_ICSD_1897	insulator	insulator
Li2P2Pr1_ICSD_23259	metal	metal
Li2P2Pr1_ICSD_642229	metal	metal
Li2Pd1Sb1_ICSD_44816	metal	metal
Li2Pd1_ICSD_642268	metal	metal
Li2Pr1Sb2_ICSD_36019	metal	metal
Li2Pt1Sb1_ICSD_642284	metal	metal
Li2Pt1_ICSD_104780	metal	metal
Li2S1_ICSD_657597	insulator	insulator
Li2S1_ICSD_91283	insulator	insulator
Li2S1_ICSD_91284	insulator	insulator
Li2S3Te1_ICSD_415120	insulator	insulator
Li2S3U1_ICSD_88095	metal	insulator
Li2Sb1_ICSD_100020	metal	insulator
Li2Se1_ICSD_642355	insulator	insulator
Li2Se3Te1_ICSD_415121	insulator	insulator
Li2Si1Zn1_ICSD_16221	metal	metal
Li2Si1Zn1_ICSD_642379	metal	metal
Li2Si1Zn1_ICSD_642380	metal	metal
Li2Si1_ICSD_642372	metal	metal
Li2Sn1Zn1_ICSD_104787	metal	metal
Li2Sn5_ICSD_26200	metal	metal
Li2Sr3_ICSD_15703	metal	metal
Li2Te1_ICSD_642398	insulator	insulator
Li2Tl1_ICSD_642401	metal	metal
Li3Mg8Si4_ICSD_181251	metal	metal
Li3N1_ICSD_156903	insulator	insulator
Li3N1_ICSD_660252	insulator	insulator
Li3N2Na3_ICSD_92311	metal	metal
Li3N2Na3_ICSD_92312	metal	metal
Li3N2Sc1_ICSD_98140	insulator	insulator
Li3N4Nb1Sr2_ICSD_84715	insulator	insulator
Li3N4Ni4Sr3_ICSD_33902	metal	metal
Li3N4Sr2Ta1_ICSD_84716	insulator	insulator
Li3Nb1O4_ICSD_30246	insulator	insulator
Li3Nd3O12W2_ICSD_245640	insulator	insulator
Li3Ni2O6Sb1_ICSD_183999	insulator	insulator
Li3O13V6_ICSD_59962	metal	metal
Li3O4P1_ICSD_10257	insulator	insulator
Li3O4P1_ICSD_20208	insulator	insulator
Li3O4Sb1_ICSD_82864	insulator	insulator
Li3O4Ta1_ICSD_37126	insulator	insulator
Li3O4U1_ICSD_109090	insulator	insulator
Li3O4V1_ICSD_19002	insulator	insulator
Li3O6Sb1Zn2_ICSD_69189	insulator	insulator
Li3P1_ICSD_26880	insulator	insulator
Li3Pb1_ICSD_104764	metal	metal
Li3Pd1_ICSD_104775	metal	metal
Li3S3Sb1_ICSD_424834	insulator	insulator
Li3Sb1_ICSD_26879	insulator	insulator

Supplementary Table 492. Five-fold cross validated predictions for the metal/insulator classification (484/598).

system	calculated	predicted
Li3Sb1_ICSD_642341	insulator	insulator
Li3Tl1_ICSD_10052	metal	metal
Li4Mo1O5_ICSD_40270	insulator	insulator
Li4N2Na2_ICSD_92305	insulator	metal
Li4N2Na2_ICSD_92306	insulator	metal
Li4N2Na2_ICSD_92307	metal	insulator
Li4N3Ta1_ICSD_412585	insulator	insulator
Li4N6Si2Sr3_ICSD_421259	insulator	insulator
Li4O4Pb1_ICSD_38350	insulator	insulator
Li4O4Si1_ICSD_98615	insulator	insulator
Li4O4Ti1_ICSD_75164	insulator	insulator
Li4O5Se1_ICSD_92395	insulator	insulator
Li4O5Te1_ICSD_2403	insulator	insulator
Li4O5U1_ICSD_20452	insulator	insulator
Li4O5W1_ICSD_108819	insulator	insulator
Li4O7P2_ICSD_246859	metal	insulator
Li4O7P2_ICSD_59243	insulator	insulator
Li4P2Sr1_ICSD_416888	insulator	insulator
Li4Pt3Si1_ICSD_186537	metal	metal
Li4Sb4Sr3_ICSD_25307	metal	metal
Li5N2Na1_ICSD_92313	insulator	insulator
Li5N2Na1_ICSD_92314	insulator	insulator
Li5N3Ni3_ICSD_411152	metal	metal
Li5N4Re1_ICSD_92467	insulator	insulator
Li5Na1Sn4_ICSD_12142	metal	metal
Li5Na2Sn4_ICSD_104760	metal	metal
Li5O4Tl1_ICSD_16219	insulator	insulator
Li5O5Sb1_ICSD_203030	insulator	insulator
Li5O6Re1_ICSD_30590	insulator	insulator
Li5O6Re1_ICSD_38381	insulator	insulator
Li5Sn2_ICSD_104783	metal	metal
Li5Tl2_ICSD_10053	metal	metal
Li6Mn2N6Sr2_ICSD_412874	insulator	insulator
Li6Mo1N4_ICSD_66095	insulator	insulator
Li6N4W1_ICSD_153620	insulator	insulator
Li6O4Zn1_ICSD_62137	insulator	insulator
Li6O6Te1_ICSD_40247	insulator	insulator
Li6O6U1_ICSD_25350	insulator	insulator
Li6O6U1_ICSD_48209	insulator	insulator
Li6O7Si2_ICSD_25752	insulator	insulator
Li6O7Zr2_ICSD_41321	insulator	insulator
Li7Mn1N4_ICSD_154076	insulator	insulator
Li7N4Nb1_ICSD_71547	insulator	insulator
Li7N4Ni1_ICSD_642178	metal	insulator
Li7N4P1_ICSD_642182	insulator	insulator
Li7N4Ta1_ICSD_67560	insulator	insulator
Li7N4V1_ICSD_409501	insulator	insulator
Li7N4V1_ICSD_44368	insulator	insulator
Li7N4V1_ICSD_96941	insulator	insulator
Li7O6Sb1_ICSD_15631	insulator	insulator

Supplementary Table 493. Five-fold cross validated predictions for the metal/insulator classification (485/598).

system	calculated	predicted
Li7O6Ta1_ICSD_74950	insulator	insulator
Li7O8Rb1Si2_ICSD_33864	insulator	insulator
Li7P3S11_ICSD_157654	insulator	insulator
Li7Pb2_ICSD_104765	metal	metal
Li7Si2_ICSD_24600	metal	metal
Li7Si3_ICSD_167672	metal	metal
Li7Sn2_ICSD_104784	metal	metal
Li7Sn3_ICSD_104785	metal	metal
Li8N2Se1_ICSD_247256	insulator	insulator
Li8N2Te1_ICSD_247257	insulator	insulator
Li8O6Pb1_ICSD_47104	insulator	insulator
Li8O6Pt1_ICSD_61218	insulator	insulator
Li8O6Sn1_ICSD_15104	insulator	insulator
Li8O6Tb1_ICSD_49026	metal	insulator
Li8Pb3_ICSD_15694	metal	metal
Li9N1S3_ICSD_240749	insulator	insulator
Lu1Mg1_ICSD_161753	metal	metal
Lu1Mn1O3_ICSD_15882	metal	insulator
Lu1Mn2_ICSD_642431	metal	metal
Lu1Mn6Sn6_ICSD_57416	metal	metal
Lu1N1_ICSD_169211	insulator	metal
Lu1Na1O8Pd6_ICSD_416619	metal	metal
Lu1Ni1P1_ICSD_642455	metal	metal
Lu1Ni1Pb1_ICSD_160550	metal	metal
Lu1Ni1Sb1_ICSD_642458	metal	metal
Lu1Ni1Si2_ICSD_642460	metal	metal
Lu1Ni1Sn1_ICSD_642464	metal	metal
Lu1Ni2Si2_ICSD_642459	metal	metal
Lu1Ni2Sn1_ICSD_104807	metal	metal
Lu1Ni2_ICSD_104804	metal	metal
Lu1Ni4Sn1_ICSD_642467	metal	metal
Lu1Ni5Sn1_ICSD_167138	metal	metal
Lu1Ni5_ICSD_104806	metal	metal
Lu1O3Pr1_ICSD_50758	insulator	insulator
Lu1O3V1_ICSD_185830	insulator	insulator
Lu1O4P1_ICSD_162338	insulator	insulator
Lu1O4P1_ICSD_162340	insulator	insulator
Lu1O4P1_ICSD_162341	insulator	insulator
Lu1O4P1_ICSD_162346	insulator	insulator
Lu1O4P1_ICSD_201133	insulator	insulator
Lu1O4P1_ICSD_2505	metal	insulator
Lu1O4V1_ICSD_160959	insulator	insulator
Lu1O4V1_ICSD_162326	insulator	insulator
Lu1O4V1_ICSD_246685	insulator	insulator
Lu1O4V1_ICSD_419281	insulator	insulator
Lu1O4V1_ICSD_419285	insulator	insulator
Lu1O4V1_ICSD_419288	insulator	insulator
Lu1Os2Si2_ICSD_642482	metal	metal
Lu1Os2_ICSD_150526	metal	metal
Lu1P1Pt1_ICSD_44915	metal	metal

Supplementary Table 494. Five-fold cross validated predictions for the metal/insulator classification (486/598).

system	calculated	predicted
Lu1P1S4_ICSD_412857	insulator	insulator
Lu1P1_ICSD_44914	metal	metal
Lu1Pb2_ICSD_104811	metal	metal
Lu1Pb3_ICSD_104812	metal	metal
Lu1Pd1Sn1_ICSD_642510	metal	metal
Lu1Pd1Zn1_ICSD_183346	metal	metal
Lu1Pd1_ICSD_642499	metal	metal
Lu1Pd2Si1_ICSD_642506	metal	metal
Lu1Pd2Si2_ICSD_603882	metal	metal
Lu1Pd2Sn1_ICSD_104817	metal	metal
Lu1Pd3_ICSD_104815	metal	metal
Lu1Pt1Sb1_ICSD_44916	metal	metal
Lu1Pt1Sn1_ICSD_642538	metal	metal
Lu1Pt1_ICSD_642529	metal	metal
Lu1Pt3_ICSD_642530	metal	metal
Lu1Rb1S2_ICSD_81407	insulator	insulator
Lu1Rh1Si1_ICSD_90193	metal	metal
Lu1Rh1_ICSD_104821	metal	metal
Lu1Rh2Si2_ICSD_90198	metal	metal
Lu1Rh2_ICSD_642546	metal	metal
Lu1Ru1_ICSD_104824	metal	metal
Lu1Ru2Si2_ICSD_90197	metal	metal
Lu1Ru2_ICSD_150525	metal	metal
Lu1S1_ICSD_642561	metal	metal
Lu1S2Tl1_ICSD_642581	insulator	insulator
Lu1S2_ICSD_44918	metal	insulator
Lu1Sb1Zr1_ICSD_107074	metal	metal
Lu1Sb1_ICSD_247647	metal	metal
Lu1Sb1_ICSD_642588	metal	metal
Lu1Sb2_ICSD_642589	metal	metal
Lu1Se1_ICSD_44921	metal	metal
Lu1Se2Tl1_ICSD_642602	insulator	insulator
Lu1Si1Ti1_ICSD_88208	metal	metal
Lu1Si2_ICSD_642611	metal	metal
Lu1Sn2Zn1_ICSD_183314	metal	metal
Lu1Tc2_ICSD_642618	metal	metal
Lu1Te1_ICSD_44922	metal	metal
Lu1Te2Tl1_ICSD_642625	insulator	insulator
Lu1Te2_ICSD_642622	metal	metal
Lu1Tl3_ICSD_104829	metal	metal
Lu1Tl3_ICSD_642631	metal	metal
Lu1Zn1_ICSD_104830	metal	metal
Lu1_ICSD_43586	metal	metal
Lu1_ICSD_44910	metal	metal
Lu1_ICSD_642415	metal	metal
Lu2Mg1S4_ICSD_37420	insulator	insulator
Lu2Mg1Se4_ICSD_44912	insulator	insulator
Lu2Mn1S4_ICSD_37421	insulator	insulator
Lu2Mn1Se4_ICSD_642434	insulator	insulator
Lu2Mn2O7_ICSD_56518	insulator	insulator

Supplementary Table 495. Five-fold cross validated predictions for the metal/insulator classification (487/598).

system	calculated	predicted
Lu2O13Te5_ICSD_413669	insulator	insulator
Lu2O2S1_ICSD_109336	insulator	insulator
Lu2O3_ICSD_160217	metal	insulator
Lu2O3_ICSD_160232	insulator	insulator
Lu2O3_ICSD_33659	insulator	insulator
Lu2O5Si1_ICSD_89624	insulator	insulator
Lu2O7Si2_ICSD_412249	insulator	insulator
Lu2O7Sn2_ICSD_82965	insulator	insulator
Lu2O7V2_ICSD_160599	metal	insulator
Lu2O7V2_ICSD_32688	insulator	metal
Lu2Pb1Pd2_ICSD_99200	metal	metal
Lu2Pt1_ICSD_642523	metal	metal
Lu2S3_ICSD_26974	insulator	insulator
Lu2S4Sr1_ICSD_642578	insulator	insulator
Lu2S4Zn1_ICSD_35089	insulator	insulator
Lu3Os4Si13_ICSD_600535	metal	metal
Lu5Ni1Pb3_ICSD_152608	metal	metal
Lu5Pb3_ICSD_168438	metal	metal
Lu5Pd2Sb1_ICSD_87452	metal	metal
Lu5Rh4Si10_ICSD_642550	metal	metal
Lu5Si3_ICSD_414146	metal	metal
Lu5Sn3_ICSD_642614	metal	metal
Lu5Tl3_ICSD_642632	metal	metal
Lu6Ni2Sn1_ICSD_642470	metal	metal
Lu6O12U1_ICSD_23967	insulator	insulator
Lu7Te1_ICSD_97005	metal	metal
Mg12Nd1_ICSD_104837	metal	metal
Mg12Pr1_ICSD_104856	metal	metal
Mg17Sr2_ICSD_642861	metal	metal
Mg1Mn2O4_ICSD_16858	insulator	insulator
Mg1Mo1N2_ICSD_185913	insulator	insulator
Mg1Mo1O4_ICSD_20418	insulator	insulator
Mg1Mo1O6Sr2_ICSD_187662	insulator	insulator
Mg1Mo2O7_ICSD_1314	insulator	insulator
Mg1N2Si1_ICSD_186509	insulator	insulator
Mg1N2Si1_ICSD_23501	insulator	insulator
Mg1N2Si1_ICSD_44109	insulator	insulator
Mg1Na1Sb1_ICSD_41797	insulator	insulator
Mg1Na2Sn1_ICSD_262676	metal	metal
Mg1Nb2O6_ICSD_85008	insulator	insulator
Mg1Nd1Ni4_ICSD_107423	metal	metal
Mg1Nd1Pt1_ICSD_412356	metal	metal
Mg1Nd1Sn1_ICSD_182480	metal	metal
Mg1Nd1_ICSD_104834	metal	metal
Mg1Nd1_ICSD_161742	metal	metal
Mg1Nd2Ni2_ICSD_107329	metal	metal
Mg1Ni1Sb1_ICSD_44925	metal	metal
Mg1Ni1Tb1_ICSD_166874	metal	metal
Mg1Ni1_ICSD_187255	metal	metal
Mg1Ni1_ICSD_187256	metal	metal

Supplementary Table 496. Five-fold cross validated predictions for the metal/insulator classification (488/598).

system	calculated	predicted
Mg1Ni2P1_ICSD_74784	metal	metal
Mg1Ni2Pr2_ICSD_107328	metal	metal
Mg1Ni2Pr2_ICSD_411080	metal	metal
Mg1Ni2Sb1_ICSD_104841	metal	metal
Mg1Ni2Sn1_ICSD_104842	metal	metal
Mg1Ni2_ICSD_104838	metal	metal
Mg1Ni2_ICSD_642687	metal	metal
Mg1Ni4Pr1_ICSD_107422	metal	metal
Mg1Ni4Y1_ICSD_107033	metal	metal
Mg1Ni6Si6_ICSD_642706	metal	metal
Mg1O1_ICSD_161842	insulator	insulator
Mg1O1_ICSD_162607	insulator	insulator
Mg1O1_ICSD_166273	insulator	insulator
Mg1O1_ICSD_181457	insulator	insulator
Mg1O1_ICSD_181459	insulator	insulator
Mg1O1_ICSD_181462	insulator	insulator
Mg1O1_ICSD_181463	insulator	insulator
Mg1O1_ICSD_181464	insulator	insulator
Mg1O1_ICSD_181465	insulator	insulator
Mg1O1_ICSD_181466	insulator	insulator
Mg1O1_ICSD_26958	insulator	insulator
Mg1O2_ICSD_41732	insulator	insulator
Mg1O3Se1_ICSD_494	insulator	insulator
Mg1O3Si1_ICSD_158959	insulator	insulator
Mg1O3Si1_ICSD_26489	insulator	insulator
Mg1O3Si1_ICSD_30893	insulator	insulator
Mg1O3Si1_ICSD_34074	insulator	insulator
Mg1O3Si1_ICSD_41659	insulator	insulator
Mg1O3Si1_ICSD_80669	insulator	insulator
Mg1O3Si1_ICSD_89805	insulator	insulator
Mg1O3Ti1_ICSD_169657	insulator	insulator
Mg1O3V1_ICSD_15927	metal	insulator
Mg1O3V1_ICSD_284	insulator	insulator
Mg1O4P1Rb1_ICSD_421738	insulator	insulator
Mg1O4P1Rb1_ICSD_421740	insulator	insulator
Mg1O4Rh2_ICSD_109299	insulator	insulator
Mg1O4S1_ICSD_16759	insulator	insulator
Mg1O4S1_ICSD_240893	insulator	insulator
Mg1O4Sb2_ICSD_4122	insulator	insulator
Mg1O4Se1_ICSD_109070	insulator	insulator
Mg1O4Ti2_ICSD_184695	insulator	insulator
Mg1O4Ti2_ICSD_184696	metal	insulator
Mg1O4V2_ICSD_60412	metal	metal
Mg1O4W1_ICSD_67905	insulator	insulator
Mg1O5Se2_ICSD_402917	insulator	insulator
Mg1O5Te2_ICSD_4318	insulator	insulator
Mg1O5Ti2_ICSD_37232	insulator	insulator
Mg1O5V2_ICSD_50981	insulator	insulator
Mg1O6Pb2Te1_ICSD_86139	insulator	insulator
Mg1O6Pb2W1_ICSD_67880	insulator	insulator

Supplementary Table 497. Five-fold cross validated predictions for the metal/insulator classification (489/598).

system	calculated	predicted
Mg1O6Pt1Sr3_ICSD_281299	insulator	insulator
Mg1O6Pt3_ICSD_35340	insulator	insulator
Mg1O6Re1Sr2_ICSD_182569	metal	metal
Mg1O6Re1Sr2_ICSD_98525	metal	metal
Mg1O6Rh1Sr3_ICSD_84738	metal	insulator
Mg1O6Sb2_ICSD_40345	insulator	insulator
Mg1O6Sr2W1_ICSD_152576	insulator	insulator
Mg1O6Sr2W1_ICSD_155308	insulator	insulator
Mg1O6Ta2_ICSD_150419	insulator	insulator
Mg1O6V2_ICSD_10391	insulator	insulator
Mg1O7Si2Sr2_ICSD_183978	insulator	insulator
Mg1P1Pd5_ICSD_642727	metal	metal
Mg1P1Pt5_ICSD_44928	metal	metal
Mg1P1S3_ICSD_642729	insulator	insulator
Mg1P2Si1_ICSD_22189	insulator	insulator
Mg1P4Rh6_ICSD_89610	metal	metal
Mg1P4_ICSD_113	insulator	insulator
Mg1Pb1Sr1_ICSD_642747	metal	metal
Mg1Pb1Yb1_ICSD_602715	metal	metal
Mg1Pd1Pr1_ICSD_657926	metal	metal
Mg1Pd1Sb1_ICSD_44825	metal	metal
Mg1Pd1Sm1_ICSD_657927	metal	metal
Mg1Pd1Y1_ICSD_54272	metal	metal
Mg1Pd1Yb1_ICSD_412726	metal	metal
Mg1Pd1Yb4_ICSD_423919	metal	metal
Mg1Pd1_ICSD_104847	metal	metal
Mg1Pd2Tb2_ICSD_412728	metal	metal
Mg1Pd2_ICSD_150227	metal	metal
Mg1Pd3_ICSD_150228	metal	metal
Mg1Pd3_ICSD_153055	metal	metal
Mg1Pm1_ICSD_161743	metal	metal
Mg1Pr1Pt1_ICSD_412100	metal	metal
Mg1Pr1Sn1_ICSD_182479	metal	metal
Mg1Pr1Sn1_ICSD_183535	metal	metal
Mg1Pr1Sn1_ICSD_183537	metal	metal
Mg1Pr1_ICSD_104851	metal	metal
Mg1Pt1Sb1_ICSD_44826	metal	metal
Mg1Pt1Sm1_ICSD_412102	metal	metal
Mg1Pt1_ICSD_109237	metal	metal
Mg1Pt3_ICSD_104857	metal	metal
Mg1Pu2_ICSD_109362	metal	metal
Mg1Rh1_ICSD_104859	metal	metal
Mg1Ru1Sn4_ICSD_412492	metal	metal
Mg1S1_ICSD_159401	insulator	insulator
Mg1S1_ICSD_44930	insulator	insulator
Mg1S4Sc2_ICSD_37423	insulator	insulator
Mg1S4Yb2_ICSD_642803	metal	metal
Mg1Sc1_ICSD_161737	metal	metal
Mg1Sc2Se4_ICSD_642814	insulator	insulator
Mg1Se17U8_ICSD_601593	insulator	metal

Supplementary Table 498. Five-fold cross validated predictions for the metal/insulator classification (490/598).

system	calculated	predicted
Mg1Se1_ICSD_159398	insulator	insulator
Mg1Se1_ICSD_53946	insulator	insulator
Mg1Se1_ICSD_658986	insulator	insulator
Mg1Se2_ICSD_642815	insulator	insulator
Mg1Se4Tm2_ICSD_76051	metal	insulator
Mg1Se4Y2_ICSD_76052	insulator	insulator
Mg1Se4Yb2_ICSD_76053	metal	metal
Mg1Si1Sr1_ICSD_42457	metal	metal
Mg1Si1Yb1_ICSD_602717	metal	metal
Mg1Si2Sr1_ICSD_78999	metal	metal
Mg1Si2Yb2_ICSD_391441	metal	metal
Mg1Sm1Tl1_ICSD_414441	metal	metal
Mg1Sn1Sr1_ICSD_104872	metal	metal
Mg1Sn1Tb1_ICSD_183523	metal	metal
Mg1Sn1Yb1_ICSD_183538	metal	metal
Mg1Sr1_ICSD_104873	metal	metal
Mg1Tb1_ICSD_161747	metal	metal
Mg1Tb2_ICSD_55549	metal	metal
Mg1Te1_ICSD_168346	insulator	insulator
Mg1Te1_ICSD_168347	insulator	insulator
Mg1Te1_ICSD_168349	insulator	insulator
Mg1Te1_ICSD_642882	insulator	insulator
Mg1Te2_ICSD_41733	insulator	insulator
Mg1Tl1Y1_ICSD_414438	metal	metal
Mg1Tl1_ICSD_104884	metal	metal
Mg1Tm1_ICSD_104885	metal	metal
Mg1Tm1_ICSD_104886	metal	metal
Mg1Tm1_ICSD_161751	metal	metal
Mg1Y1Zn1_ICSD_160907	metal	metal
Mg1Y1_ICSD_161738	metal	metal
Mg1Zn1_ICSD_151402	metal	metal
Mg1Zn2_ICSD_108587	metal	metal
Mg1Zn2_ICSD_150576	metal	metal
Mg1Zn5_ICSD_151403	metal	metal
Mg1_ICSD_180453	metal	metal
Mg1_ICSD_426953	metal	metal
Mg1_ICSD_642652	metal	metal
Mg23Sr6_ICSD_104876	metal	metal
Mg23Th6_ICSD_109226	metal	metal
Mg24Tb5_ICSD_642874	metal	metal
Mg24Y5_ICSD_109138	metal	metal
Mg2Mn1O8P2_ICSD_100784	insulator	insulator
Mg2Mo3O12Tl2_ICSD_250400	insulator	insulator
Mg2Mo3O8_ICSD_248080	insulator	insulator
Mg2N1Na2O9P3_ICSD_186877	insulator	insulator
Mg2N2Sr1_ICSD_410826	insulator	insulator
Mg2N3P1_ICSD_27229	insulator	insulator
Mg2Nd1_ICSD_104835	metal	metal
Mg2Nd1_ICSD_164273	metal	metal
Mg2Nd1_ICSD_642678	metal	metal

Supplementary Table 499. Five-fold cross validated predictions for the metal/insulator classification (491/598).

system	calculated	predicted
Mg2Ni1_ICSD_30713	metal	metal
Mg2Ni3P1_ICSD_72409	metal	metal
Mg2Ni3Si1_ICSD_44926	metal	metal
Mg2O12P4_ICSD_4280	insulator	insulator
Mg2O4Si1_ICSD_171065	insulator	insulator
Mg2O4Si1_ICSD_27533	insulator	insulator
Mg2O4Si1_ICSD_27534	insulator	insulator
Mg2O4Si1_ICSD_27535	insulator	insulator
Mg2O4Si1_ICSD_27536	insulator	insulator
Mg2O4Si1_ICSD_27537	insulator	insulator
Mg2O4Si1_ICSD_83783	insulator	insulator
Mg2O4Si1_ICSD_83792	insulator	insulator
Mg2O4Sn1_ICSD_187038	insulator	insulator
Mg2O4V1_ICSD_76980	metal	insulator
Mg2O7P2_ICSD_22328	insulator	insulator
Mg2O7V2_ICSD_2321	insulator	insulator
Mg2O8Te3_ICSD_424305	insulator	insulator
Mg2P2Se6_ICSD_642731	insulator	insulator
Mg2Pb1_ICSD_409428	metal	metal
Mg2Pb1_ICSD_642744	metal	metal
Mg2Pd1Sr1_ICSD_425493	metal	metal
Mg2Pd1_ICSD_409837	metal	metal
Mg2Pr1_ICSD_104853	metal	metal
Mg2Pt1Si1_ICSD_68513	metal	metal
Mg2Pt1_ICSD_245120	metal	metal
Mg2Rh1_ICSD_54254	metal	metal
Mg2S4Si1_ICSD_642791	insulator	insulator
Mg2S4Sn1_ICSD_642793	insulator	insulator
Mg2Sb2Sr1_ICSD_100046	insulator	insulator
Mg2Sb2Yb1_ICSD_412655	insulator	insulator
Mg2Se4Si1_ICSD_642818	insulator	insulator
Mg2Se4Sn1_ICSD_642819	insulator	insulator
Mg2Si10Sr11_ICSD_50001	metal	metal
Mg2Si1_ICSD_108584	metal	metal
Mg2Si1_ICSD_167512	metal	metal
Mg2Si1_ICSD_181102	metal	metal
Mg2Si1_ICSD_657540	insulator	metal
Mg2Sm1_ICSD_104867	metal	metal
Mg2Sn1_ICSD_104869	metal	metal
Mg2Sn1_ICSD_181766	metal	metal
Mg2Sn1_ICSD_182695	metal	metal
Mg2Sr1_ICSD_642864	metal	metal
Mg2Tb1_ICSD_642872	metal	metal
Mg2Th1_ICSD_104883	metal	metal
Mg2Th1_ICSD_150498	metal	metal
Mg2Tl1_ICSD_150630	metal	metal
Mg2Tm1_ICSD_380503	metal	metal
Mg2Tm1_ICSD_55551	metal	metal
Mg2Y1_ICSD_642903	metal	metal
Mg2Yb1_ICSD_412666	metal	metal

Supplementary Table 500. Five-fold cross validated predictions for the metal/insulator classification (492/598).

system	calculated	predicted
Mg2Zn11_ICSD_104898	metal	metal
Mg3Mn1Ni2_ICSD_182431	metal	metal
Mg3Mn2O12Si3_ICSD_27374	insulator	insulator
Mg3N2_ICSD_408145	insulator	insulator
Mg3Nb6O11_ICSD_62662	insulator	insulator
Mg3Nd1_ICSD_54271	metal	metal
Mg3Ni20P6_ICSD_72350	metal	metal
Mg3Ni2Ti1_ICSD_187996	metal	metal
Mg3O12Si3V2_ICSD_27372	metal	insulator
Mg3O6Te1_ICSD_6309	insulator	insulator
Mg3O8P2_ICSD_261231	insulator	insulator
Mg3O8P2_ICSD_9849	metal	insulator
Mg3O8V2_ICSD_21085	insulator	insulator
Mg3P2_ICSD_24489	insulator	insulator
Mg3P2_ICSD_642724	insulator	insulator
Mg3Pd1_ICSD_409824	metal	metal
Mg3Pd1_ICSD_642752	metal	metal
Mg3Pr1_ICSD_104854	metal	metal
Mg3Pr1_ICSD_642770	metal	metal
Mg3Pt1_ICSD_104858	metal	metal
Mg3Pt1_ICSD_54253	metal	metal
Mg3Rh1_ICSD_413720	metal	metal
Mg3Ru2_ICSD_104862	metal	metal
Mg3Sb2_ICSD_181285	insulator	insulator
Mg3Sb2_ICSD_52335	insulator	insulator
Mg3Sm1_ICSD_104868	metal	metal
Mg3Sm1_ICSD_642837	metal	metal
Mg3Tb1_ICSD_642871	metal	metal
Mg4N12P6S1_ICSD_642665	insulator	insulator
Mg4Nb2O9_ICSD_91748	insulator	insulator
Mg4O9Ta2_ICSD_65301	insulator	insulator
Mg4P6Rh7_ICSD_94390	metal	metal
Mg4Sr1_ICSD_104874	metal	metal
Mg5Na2O16P4_ICSD_75271	insulator	insulator
Mg5Pd10Si16_ICSD_95899	metal	metal
Mg5Pd2_ICSD_150567	metal	metal
Mg5Pt10Si16_ICSD_95900	metal	metal
Mg5Rh2_ICSD_104860	metal	metal
Mg5Si6_ICSD_54780	metal	metal
Mg5Si6_ICSD_85529	metal	metal
Mg5Tl2_ICSD_150631	metal	metal
Mg6Mn1O8_ICSD_76174	insulator	insulator
Mg6Ni16Si7_ICSD_159261	metal	metal
Mg6Si3.3_ICSD_157682	metal	metal
Mg9Sn5_ICSD_55577	metal	metal
Mn0.25Nb1S2_ICSD_42655	metal	metal
Mn0.25S2Ta1_ICSD_42660	metal	metal
Mn11Pd21_ICSD_130005	metal	metal
Mn12Th1_ICSD_104986	metal	metal
Mn12Y1_ICSD_26821	metal	metal

Supplementary Table 501. Five-fold cross validated predictions for the metal/insulator classification (493/598).

system	calculated	predicted
Mn12Y1_ICSD_643911	metal	metal
Mn1Mo1N2_ICSD_81489	insulator	metal
Mn1Mo1O4_ICSD_15615	insulator	insulator
Mn1Mo1O4_ICSD_61078	insulator	insulator
Mn1Mo1O6Sr2_ICSD_187667	metal	metal
Mn1Mo1O6Sr2_ICSD_187669	metal	insulator
Mn1Mo1O6Sr2_ICSD_99088	metal	metal
Mn1Mo1O6Te1_ICSD_163979	insulator	insulator
Mn1N1_ICSD_106932	metal	metal
Mn1N1_ICSD_184928	metal	metal
Mn1N2O6_ICSD_412003	insulator	insulator
Mn1N2Si1_ICSD_172194	insulator	metal
Mn1N2Si1_ICSD_200019	metal	insulator
Mn1N2W1_ICSD_80029	metal	metal
Mn1N3Sr3_ICSD_80772	metal	metal
Mn1N3Th2_ICSD_87243	metal	metal
Mn1Na1Nd1O6W1_ICSD_159098	insulator	insulator
Mn1Na1O2_ICSD_155330	insulator	insulator
Mn1Na1O2_ICSD_16271	insulator	insulator
Mn1Na1O4Rb2_ICSD_170771	insulator	insulator
Mn1Na1O4V1_ICSD_260949	insulator	insulator
Mn1Na1O6Si2_ICSD_62513	insulator	insulator
Mn1Na1O6Tb1W1_ICSD_159099	insulator	insulator
Mn1Na1P1_ICSD_601595	metal	metal
Mn1Na1Sb1_ICSD_601596	metal	metal
Mn1Na1Se2_ICSD_50818	metal	insulator
Mn1Na1Te2_ICSD_110774	metal	metal
Mn1Na2O4_ICSD_39504	insulator	insulator
Mn1Na2O7P2_ICSD_86669	insulator	insulator
Mn1Na2O8S2_ICSD_249878	metal	insulator
Mn1Na6O4_ICSD_420410	insulator	insulator
Mn1Na6S4_ICSD_95067	insulator	insulator
Mn1Na6Se4_ICSD_65449	insulator	insulator
Mn1Na6Te4_ICSD_65451	insulator	insulator
Mn1Nb1O6Sr2_ICSD_181751	metal	metal
Mn1Nb1P1_ICSD_68280	metal	metal
Mn1Nb1Si1_ICSD_24185	metal	metal
Mn1Nb2O6_ICSD_31944	insulator	insulator
Mn1Nb2O8Zn2_ICSD_202667	insulator	insulator
Mn1Nb2S4_ICSD_643015	metal	metal
Mn1Nb3O6_ICSD_89477	metal	insulator
Mn1Nb3S6_ICSD_104913	metal	metal
Mn1Nb4Se8_ICSD_643025	metal	metal
Mn1Nd1O1P1_ICSD_50241	metal	metal
Mn1Nd1O1Sb1_ICSD_50243	metal	metal
Mn1Nd1Si1_ICSD_643062	metal	metal
Mn1Nd1Si1_ICSD_75056	metal	metal
Mn1Nd1Si1_ICSD_75057	metal	metal
Mn1Nd1Si1_ICSD_85851	metal	metal
Mn1Nd1Si2_ICSD_643055	metal	metal

Supplementary Table 502. Five-fold cross validated predictions for the metal/insulator classification (494/598).

system	calculated	predicted
Mn1Nd1Si2_ICSD_85927	metal	metal
Mn1Ni1O3_ICSD_31853	insulator	insulator
Mn1Ni1P1_ICSD_161714	metal	metal
Mn1Ni1P1_ICSD_600852	metal	metal
Mn1Ni1P1_ICSD_643091	metal	metal
Mn1Ni1P1_ICSD_643093	metal	metal
Mn1Ni1Sb1_ICSD_161717	metal	metal
Mn1Ni1Sb1_ICSD_643120	metal	metal
Mn1Ni1Si1_ICSD_108590	metal	metal
Mn1Ni1Si1_ICSD_161713	metal	metal
Mn1Ni1Si1_ICSD_165241	metal	metal
Mn1Ni1Si1_ICSD_643132	metal	metal
Mn1Ni1Si1_ICSD_76084	metal	metal
Mn1Ni1_ICSD_104917	metal	metal
Mn1Ni2Sb1_ICSD_76080	metal	metal
Mn1Ni2Sn1_ICSD_104927	metal	metal
Mn1Ni3_ICSD_104920	metal	metal
Mn1Ni4Y1_ICSD_643173	metal	metal
Mn1Ni6O8_ICSD_40584	insulator	metal
Mn1O1_ICSD_262928	metal	metal
Mn1O1_ICSD_643192	metal	metal
Mn1O2.6Sr1_ICSD_159660	metal	metal
Mn1O2_ICSD_150462	insulator	insulator
Mn1O2_ICSD_171866	insulator	insulator
Mn1O2_ICSD_20227	insulator	metal
Mn1O2_ICSD_54114	metal	insulator
Mn1O2_ICSD_643187	metal	insulator
Mn1O3Se1_ICSD_495	insulator	insulator
Mn1O3Sn1_ICSD_29203	insulator	insulator
Mn1O3Sr1_ICSD_157936	insulator	insulator
Mn1O3Sr1_ICSD_185417	insulator	insulator
Mn1O3Sr1_ICSD_188415	metal	metal
Mn1O3Sr1_ICSD_202615	insulator	insulator
Mn1O3Tb1_ICSD_180803	metal	insulator
Mn1O3Ti1_ICSD_171579	insulator	insulator
Mn1O3Ti1_ICSD_184650	insulator	insulator
Mn1O3Y1_ICSD_166217	metal	metal
Mn1O3Y1_ICSD_181182	metal	metal
Mn1O3Y1_ICSD_422986	metal	insulator
Mn1O4P1Rb1_ICSD_157359	insulator	insulator
Mn1O4P1_ICSD_99859	insulator	insulator
Mn1O4Pb2_ICSD_33990	insulator	insulator
Mn1O4Rb1V1_ICSD_246201	insulator	insulator
Mn1O4Rb1_ICSD_89507	insulator	insulator
Mn1O4Rb2_ICSD_79083	insulator	insulator
Mn1O4Re1_ICSD_170685	metal	insulator
Mn1O4Rh2_ICSD_109300	metal	insulator
Mn1O4S1_ICSD_27648	insulator	insulator
Mn1O4Sb2_ICSD_60407	insulator	insulator
Mn1O4Se1_ICSD_109071	insulator	insulator

Supplementary Table 503. Five-fold cross validated predictions for the metal/insulator classification (495/598).

system	calculated	predicted
Mn1O4Sr2_ICSD_26560	metal	metal
Mn1O4Ti2_ICSD_22383	metal	metal
Mn1O4U1_ICSD_26938	insulator	insulator
Mn1O4V2_ICSD_109148	metal	metal
Mn1O4W1_ICSD_67926	insulator	insulator
Mn1O5Se2_ICSD_27518	insulator	insulator
Mn1O5Se2_ICSD_73936	insulator	insulator
Mn1O5Si2_ICSD_85554	insulator	insulator
Mn1O5Te2_ICSD_15844	insulator	insulator
Mn1O6Pb2Re1_ICSD_182001	metal	metal
Mn1O6Pt3_ICSD_35337	insulator	insulator
Mn1O6Sb1Sr2_ICSD_39351	insulator	insulator
Mn1O6Sb2_ICSD_202952	insulator	insulator
Mn1O6Se2_ICSD_69801	insulator	insulator
Mn1O6Sr2Zr1_ICSD_164699	insulator	metal
Mn1O6Ta2_ICSD_15857	insulator	insulator
Mn1O6V2_ICSD_40850	insulator	insulator
Mn1O7Si2Sr2_ICSD_261227	insulator	insulator
Mn1O7Te1V2_ICSD_59328	insulator	insulator
Mn1O8Re2_ICSD_51014	insulator	insulator
Mn1O8Ta2Zn2_ICSD_85042	insulator	insulator
Mn1P1Rb1_ICSD_643229	metal	metal
Mn1P1Rh1_ICSD_643230	metal	metal
Mn1P1S3_ICSD_643231	insulator	insulator
Mn1P1Se3_ICSD_54140	insulator	insulator
Mn1P1Ta1_ICSD_85947	metal	metal
Mn1P1Zr1_ICSD_76095	metal	metal
Mn1P1_ICSD_30412	metal	metal
Mn1P1_ICSD_643200	metal	metal
Mn1P2Rb2S6_ICSD_280907	insulator	insulator
Mn1P4_ICSD_1829	insulator	insulator
Mn1Pb1Rh2_ICSD_104936	metal	metal
Mn1Pd1Sb1_ICSD_76099	metal	metal
Mn1Pd1Te1_ICSD_40908	metal	metal
Mn1Pd1Te1_ICSD_44996	metal	metal
Mn1Pd1_ICSD_104938	metal	metal
Mn1Pd1_ICSD_104940	metal	metal
Mn1Pd2Sb1_ICSD_643312	metal	metal
Mn1Pd2Sn1_ICSD_643327	metal	metal
Mn1Pd2_ICSD_247774	metal	metal
Mn1Pd3_ICSD_104941	metal	metal
Mn1Pr1Si1_ICSD_75052	metal	metal
Mn1Pr1Si1_ICSD_75053	metal	metal
Mn1Pr1Si1_ICSD_75054	metal	metal
Mn1Pr1Si2_ICSD_643350	metal	metal
Mn1Pt1Sb1_ICSD_643376	metal	metal
Mn1Pt1Sb1_ICSD_643377	metal	metal
Mn1Pt1Sn1_ICSD_104955	metal	metal
Mn1Pt1Sn1_ICSD_643387	metal	metal
Mn1Pt1_ICSD_104949	metal	metal

Supplementary Table 504. Five-fold cross validated predictions for the metal/insulator classification (496/598).

system	calculated	predicted
Mn1Pt3_ICSD_104951	metal	metal
Mn1Rb1Sb1_ICSD_643395	insulator	metal
Mn1Rb1Se2_ICSD_50819	metal	insulator
Mn1Rb1Te2_ICSD_87984	metal	metal
Mn1Rb2S2_ICSD_65454	insulator	insulator
Mn1Rb2Se2_ICSD_65457	insulator	insulator
Mn1Rb2Te2_ICSD_65460	insulator	insulator
Mn1Rh1Sb1_ICSD_76199	metal	metal
Mn1Rh1Sb1_ICSD_76201	metal	metal
Mn1Rh1Si1_ICSD_76203	metal	metal
Mn1Rh1_ICSD_104960	metal	metal
Mn1Rh2Sb1_ICSD_76202	metal	metal
Mn1Rh2Sn1_ICSD_643428	metal	metal
Mn1S17U8_ICSD_643484	insulator	insulator
Mn1S1_ICSD_44764	metal	insulator
Mn1S1_ICSD_643445	insulator	insulator
Mn1S1_ICSD_643455	insulator	insulator
Mn1S1_ICSD_76205	insulator	insulator
Mn1S2_ICSD_36545	insulator	insulator
Mn1S2_ICSD_643441	insulator	insulator
Mn1S4Sb2_ICSD_411178	insulator	insulator
Mn1S4Sc2_ICSD_37424	insulator	insulator
Mn1S4Yb2_ICSD_643495	metal	metal
Mn1S6Ta3_ICSD_643472	metal	metal
Mn1S8Tl1V4_ICSD_643478	metal	insulator
Mn1Sb1_ICSD_53970	metal	metal
Mn1Sc1Si1_ICSD_86369	metal	metal
Mn1Sc2Se4_ICSD_74407	insulator	insulator
Mn1Se1_ICSD_24252	insulator	insulator
Mn1Se1_ICSD_643569	insulator	insulator
Mn1Se1_ICSD_643594	insulator	insulator
Mn1Se1_ICSD_76218	insulator	insulator
Mn1Se2_ICSD_24020	insulator	insulator
Mn1Se3Th1_ICSD_99582	insulator	metal
Mn1Se4Yb2_ICSD_76225	metal	metal
Mn1Si1Sm1_ICSD_643651	metal	metal
Mn1Si1Ta1_ICSD_643653	metal	metal
Mn1Si1Tb1_ICSD_88060	metal	metal
Mn1Si1Ti1_ICSD_643671	metal	metal
Mn1Si1Ti2_ICSD_189703	metal	metal
Mn1Si1Y1_ICSD_643705	metal	metal
Mn1Si1Yb1_ICSD_95000	metal	metal
Mn1Si1Zr1_ICSD_643708	metal	metal
Mn1Si1_ICSD_643618	metal	metal
Mn1Si2Ti1_ICSD_31991	metal	metal
Mn1Sn1Sr1_ICSD_66951	metal	metal
Mn1Sn1Te4Tl2_ICSD_172507	insulator	insulator
Mn1Sn1Ti2_ICSD_189709	metal	metal
Mn1Sn2_ICSD_102799	metal	metal
Mn1Sn2_ICSD_24571	metal	metal

Supplementary Table 505. Five-fold cross validated predictions for the metal/insulator classification (497/598).

system	calculated	predicted
Mn1Te1_ICSD_181324	insulator	metal
Mn1Te1_ICSD_643793	metal	metal
Mn1Te1_ICSD_643799	insulator	metal
Mn1Te1_ICSD_76240	metal	insulator
Mn1Te2_ICSD_643803	metal	metal
Mn1U6_ICSD_150486	metal	metal
Mn1V1_ICSD_643868	metal	metal
Mn1Zn1_ICSD_105017	metal	metal
Mn1Zn3_ICSD_105020	metal	metal
Mn1_ICSD_164349	metal	metal
Mn1_ICSD_41509	metal	metal
Mn1_ICSD_41775	metal	metal
Mn23Nd6_ICSD_602440	metal	metal
Mn23Pr6_ICSD_643337	metal	metal
Mn23Tb6_ICSD_643774	metal	metal
Mn23Th6_ICSD_104987	metal	metal
Mn23Y6_ICSD_105008	metal	metal
Mn23Y6_ICSD_105010	metal	metal
Mn23Yb6_ICSD_643920	metal	metal
Mn2Mo3O8_ICSD_10144	insulator	insulator
Mn2Mo3O8_ICSD_248082	insulator	insulator
Mn2Na14O9_ICSD_1563	insulator	insulator
Mn2Na2O3_ICSD_409981	insulator	insulator
Mn2Na2S3_ICSD_36209	insulator	insulator
Mn2Na2Se3_ICSD_50820	insulator	insulator
Mn2Na4O5_ICSD_1026	metal	insulator
Mn2Nb1_ICSD_643006	metal	metal
Mn2Nb1_ICSD_643008	metal	metal
Mn2Nb1_ICSD_643010	metal	metal
Mn2Nb1_ICSD_643012	metal	metal
Mn2Nd1Si2_ICSD_54942	metal	metal
Mn2Nd1Si2_ICSD_658306	metal	metal
Mn2Nd1Si2_ICSD_72478	metal	metal
Mn2Nd1Si2_ICSD_72479	metal	metal
Mn2Nd1_ICSD_108589	metal	metal
Mn2Ni1O4_ICSD_201398	metal	metal
Mn2O12P4_ICSD_412558	insulator	insulator
Mn2O12Rb2S3_ICSD_172522	insulator	insulator
Mn2O14P4Si1_ICSD_412559	insulator	insulator
Mn2O3Ta1_ICSD_15995	metal	metal
Mn2O3_ICSD_159865	metal	insulator
Mn2O4Si1_ICSD_88026	insulator	insulator
Mn2O4Ti1_ICSD_22313	insulator	insulator
Mn2O4Zn1_ICSD_39196	insulator	insulator
Mn2O5Pr1_ICSD_84351	insulator	insulator
Mn2O5Sr2_ICSD_417812	metal	metal
Mn2O5Y1_ICSD_159271	insulator	insulator
Mn2O6Rb6_ICSD_245999	insulator	insulator
Mn2O7P2_ICSD_47137	insulator	insulator
Mn2O7Sb2_ICSD_247301	metal	insulator

Supplementary Table 506. Five-fold cross validated predictions for the metal/insulator classification (498/598).

system	calculated	predicted
Mn2O7Sc2_ICSD_83277	insulator	insulator
Mn2O7Tl2_ICSD_83268	metal	insulator
Mn2O7V2_ICSD_81994	insulator	insulator
Mn2O7Y2_ICSD_202516	insulator	insulator
Mn2O8Te3_ICSD_82490	insulator	insulator
Mn2O9Pb2Si2_ICSD_31846	insulator	insulator
Mn2P1_ICSD_643211	metal	metal
Mn2P1_ICSD_643215	metal	metal
Mn2P1_ICSD_643223	metal	metal
Mn2P2Sr1_ICSD_49019	metal	metal
Mn2P2Sr1_ICSD_77669	metal	metal
Mn2Pr1Si2_ICSD_86256	metal	metal
Mn2Pu1Si2_ICSD_604299	metal	metal
Mn2Pu1_ICSD_643390	metal	metal
Mn2Ru1Si1_ICSD_182830	metal	metal
Mn2Ru1Sn1_ICSD_182829	metal	metal
Mn2S4Si1_ICSD_643464	insulator	insulator
Mn2S4Sn1_ICSD_78760	metal	insulator
Mn2S4Zn1_ICSD_643504	metal	insulator
Mn2Sb1_ICSD_163332	metal	metal
Mn2Sb1_ICSD_184948	metal	metal
Mn2Sb1_ICSD_643526	metal	metal
Mn2Sb1_ICSD_643534	metal	metal
Mn2Sb2Sr1_ICSD_416094	metal	metal
Mn2Sb2Sr1_ICSD_41790	metal	metal
Mn2Sb2Yb1_ICSD_154766	metal	metal
Mn2Sc1_ICSD_169435	metal	metal
Mn2Se4Si1_ICSD_643595	insulator	insulator
Mn2Se4Sn1_ICSD_643596	insulator	insulator
Mn2Se4Zn1_ICSD_643609	metal	insulator
Mn2Si2Tb1_ICSD_106811	metal	metal
Mn2Si2Th1_ICSD_41977	metal	metal
Mn2Si2U1_ICSD_643683	metal	metal
Mn2Si2U1_ICSD_643685	metal	metal
Mn2Si2U1_ICSD_656522	metal	metal
Mn2Si2Y1_ICSD_86263	metal	metal
Mn2Si2Yb1_ICSD_106814	metal	metal
Mn2Sn1Te4_ICSD_169812	metal	metal
Mn2Sn1W1_ICSD_104980	metal	metal
Mn2Sn1_ICSD_104977	metal	metal
Mn2Sn1_ICSD_643735	metal	metal
Mn2Sn1_ICSD_643741	metal	metal
Mn2Sn5Yb4_ICSD_419134	metal	metal
Mn2Ta1_ICSD_643761	metal	metal
Mn2Ta1_ICSD_643763	metal	metal
Mn2Ta1_ICSD_643764	metal	metal
Mn2Tb1_ICSD_104982	metal	metal
Mn2Tb1_ICSD_54278	metal	metal
Mn2Tb1_ICSD_602123	metal	metal
Mn2Te4Zn1_ICSD_643810	metal	metal

Supplementary Table 507. Five-fold cross validated predictions for the metal/insulator classification (499/598).

system	calculated	predicted
Mn2Th1_ICSD_604013	metal	metal
Mn2Th1_ICSD_643817	metal	metal
Mn2Ti1_ICSD_104989	metal	metal
Mn2Ti1_ICSD_109270	metal	metal
Mn2Ti1_ICSD_643823	metal	metal
Mn2Ti1_ICSD_643824	metal	metal
Mn2Ti1_ICSD_643834	metal	metal
Mn2Tm1_ICSD_104994	metal	metal
Mn2U1_ICSD_104995	metal	metal
Mn2U1_ICSD_104996	metal	metal
Mn2U1_ICSD_643857	metal	metal
Mn2Y1_ICSD_54374	metal	metal
Mn2Y1_ICSD_603250	metal	metal
Mn2Y1_ICSD_643877	metal	metal
Mn2Y1_ICSD_643915	metal	metal
Mn2Zr1_ICSD_109269	metal	metal
Mn2Zr1_ICSD_246490	metal	metal
Mn2Zr1_ICSD_643937	metal	metal
Mn2Zr1_ICSD_643942	metal	metal
Mn3N1Ni1_ICSD_76056	metal	metal
Mn3N1Pd1_ICSD_76057	metal	metal
Mn3N1Pt1_ICSD_76058	metal	metal
Mn3N1Rh1_ICSD_642970	metal	metal
Mn3N1Rh1_ICSD_76060	metal	metal
Mn3N1Sn1_ICSD_76062	metal	metal
Mn3N1Ta3_ICSD_642979	metal	metal
Mn3N1Zn1_ICSD_76070	metal	metal
Mn3N2_ICSD_84202	metal	metal
Mn3Na2O7_ICSD_92858	insulator	insulator
Mn3Nb6O11_ICSD_62661	insulator	metal
Mn3Ni20P6_ICSD_72351	metal	metal
Mn3Ni2Si1_ICSD_20441	metal	metal
Mn3O10Sr4_ICSD_40301	insulator	insulator
Mn3O12Si3V2_ICSD_27380	metal	insulator
Mn3O1Ti3_ICSD_29052	metal	metal
Mn3O4_ICSD_188902	metal	metal
Mn3O4_ICSD_188903	metal	metal
Mn3O4_ICSD_68174	insulator	metal
Mn3O6Te1_ICSD_245054	insulator	insulator
Mn3O6W1_ICSD_74952	insulator	insulator
Mn3O8P2_ICSD_415107	metal	insulator
Mn3O8V2_ICSD_422975	insulator	insulator
Mn3P1_ICSD_643224	metal	metal
Mn3P6Pd20_ICSD_156065	metal	metal
Mn3P6Pd20_ICSD_156066	metal	metal
Mn3Pt1_ICSD_104953	metal	metal
Mn3Rb2Se4_ICSD_78931	metal	insulator
Mn3Rh1_ICSD_104961	metal	metal
Mn3Rh1_ICSD_104962	metal	metal
Mn3Sb1_ICSD_163333	metal	metal

Supplementary Table 508. Five-fold cross validated predictions for the metal/insulator classification (500/598).

system	calculated	predicted
Mn3Si1_ICSD_643621	metal	metal
Mn3Si3V2_ICSD_643689	metal	metal
Mn3Sn1_ICSD_188333	metal	metal
Mn3Sn1_ICSD_643730	metal	metal
Mn3Sn2_ICSD_55622	metal	metal
Mn4N1_ICSD_44369	metal	metal
Mn4N1_ICSD_642956	metal	metal
Mn4Na3Te6_ICSD_87985	metal	metal
Mn4Nb2O9_ICSD_29216	insulator	insulator
Mn4Nb2Si5_ICSD_643027	metal	metal
Mn4O7Si1_ICSD_36273	insulator	insulator
Mn4Si5Ta2_ICSD_76230	metal	metal
Mn4Si7_ICSD_183036	insulator	metal
Mn56Yb2_ICSD_643923	metal	metal
Mn5O12Si3_ICSD_27382	metal	insulator
Mn5O16Pb3V2_ICSD_95653	insulator	insulator
Mn5O8V1_ICSD_262806	insulator	insulator
Mn5O8V1_ICSD_262807	insulator	insulator
Mn5O8_ICSD_16956	insulator	insulator
Mn5Si3_ICSD_24359	metal	metal
Mn5Si3_ICSD_33713	metal	metal
Mn5Si3_ICSD_643622	metal	metal
Mn5Si3_ICSD_643628	metal	metal
Mn6Ni16P7_ICSD_35466	metal	metal
Mn6Ni16Si7_ICSD_162574	metal	metal
Mn6Sc1Sn6_ICSD_54273	metal	metal
Mn6Sc1Sn6_ICSD_643563	metal	metal
Mn6Sn6Tb1_ICSD_54277	metal	metal
Mn6Sn6Tb1_ICSD_643748	metal	metal
Mn6Sn6Tm1_ICSD_107455	metal	metal
Mn6Sn6Y1_ICSD_240144	metal	metal
Mn6Sn6Y1_ICSD_240146	metal	metal
Mn6Sn6Zr1_ICSD_54929	metal	metal
Mn7Na1O12_ICSD_19022	metal	metal
Mn7O12Pb1_ICSD_262742	metal	insulator
Mo1.33Ru0.67_ICSD_644234	metal	metal
Mo12Rb2Se14_ICSD_89004	metal	metal
Mo13P9U1_ICSD_94407	metal	metal
Mo15Se19_ICSD_69004	metal	metal
Mo1N1Na3O3_ICSD_98673	insulator	insulator
Mo1N1Na5O4_ICSD_55113	insulator	insulator
Mo1N1Ta1_ICSD_100437	metal	metal
Mo1N1_ICSD_106926	metal	metal
Mo1N1_ICSD_159438	metal	metal
Mo1N1_ICSD_159439	metal	metal
Mo1N1_ICSD_159442	metal	metal
Mo1N1_ICSD_168364	metal	metal
Mo1N1_ICSD_168369	metal	metal
Mo1N1_ICSD_168372	metal	metal
Mo1N1_ICSD_185560	metal	metal

Supplementary Table 509. Five-fold cross validated predictions for the metal/insulator classification (501/598).

system	calculated	predicted
Mo1N1_ICSD_43559	metal	metal
Mo1N1_ICSD_45000	metal	metal
Mo1N1_ICSD_60168	metal	metal
Mo1N2Na4O2_ICSD_73102	insulator	insulator
Mo1N3Na3_ICSD_67565	insulator	insulator
Mo1Na1O2_ICSD_166514	metal	insulator
Mo1Na1O5Sb1_ICSD_413147	insulator	insulator
Mo1Na2O12Te4_ICSD_88010	insulator	insulator
Mo1Na2O4_ICSD_151971	insulator	insulator
Mo1Na2O4_ICSD_44523	insulator	insulator
Mo1Na2O6Se1_ICSD_412998	insulator	insulator
Mo1Na4O5_ICSD_411635	insulator	insulator
Mo1Ni1O4_ICSD_174488	insulator	insulator
Mo1Ni1O4_ICSD_81059	insulator	insulator
Mo1Ni1O4_ICSD_81060	insulator	insulator
Mo1Ni1O6Sr2_ICSD_155733	insulator	insulator
Mo1Ni1O6Sr2_ICSD_157021	insulator	insulator
Mo1Ni1P1_ICSD_1133	metal	metal
Mo1Ni1P2_ICSD_644023	metal	metal
Mo1Ni1P8_ICSD_71037	insulator	insulator
Mo1Ni3_ICSD_105046	metal	metal
Mo1Ni4_ICSD_105047	metal	metal
Mo1O11P3Si1_ICSD_202450	insulator	insulator
Mo1O1Rb2S3_ICSD_423994	insulator	insulator
Mo1O2Rb2S2_ICSD_423992	insulator	insulator
Mo1O2Rb2S2_ICSD_423993	insulator	insulator
Mo1O2_ICSD_23722	metal	metal
Mo1O2_ICSD_99714	metal	metal
Mo1O3Sr1_ICSD_174205	metal	metal
Mo1O3Sr1_ICSD_174206	metal	insulator
Mo1O3Sr1_ICSD_71994	metal	metal
Mo1O3Tl0.33_ICSD_201931	insulator	insulator
Mo1O3_ICSD_644058	insulator	insulator
Mo1O3_ICSD_80577	insulator	insulator
Mo1O4Pb1_ICSD_56111	insulator	insulator
Mo1O4Rb2_ICSD_24904	insulator	insulator
Mo1O4Sr1_ICSD_245802	insulator	insulator
Mo1O4Sr2_ICSD_152123	metal	metal
Mo1O4Tl2_ICSD_280056	insulator	insulator
Mo1O4Tl2_ICSD_421983	insulator	insulator
Mo1O4Zn1_ICSD_17030	insulator	insulator
Mo1O4Zn1_ICSD_236417	insulator	insulator
Mo1O5P1_ICSD_169098	insulator	insulator
Mo1O5P1_ICSD_36095	insulator	insulator
Mo1O5Pb2_ICSD_29270	insulator	insulator
Mo1O5U1_ICSD_82460	insulator	insulator
Mo1O5V1_ICSD_27315	insulator	insulator
Mo1O6Os1Pb2_ICSD_188743	metal	metal
Mo1O6Os1Sn2_ICSD_188742	metal	metal
Mo1O6Rh2_ICSD_200014	metal	insulator

Supplementary Table 510. Five-fold cross validated predictions for the metal/insulator classification (502/598).

system	calculated	predicted
Mo1O6Sb2_ICSD_30699	insulator	insulator
Mo1O6Sr2Zn1_ICSD_262320	insulator	insulator
Mo1O6Sr2Zn1_ICSD_28602	insulator	insulator
Mo1O6Te1Zn1_ICSD_163981	insulator	insulator
Mo1O7P2_ICSD_202810	insulator	insulator
Mo1O7Se1Zn2_ICSD_261699	insulator	insulator
Mo1O7Te1Zn2_ICSD_261700	insulator	insulator
Mo1O7Te2_ICSD_73	insulator	insulator
Mo1O8V2_ICSD_25378	insulator	insulator
Mo1O8V2_ICSD_28471	metal	insulator
Mo1P1Rh1_ICSD_644093	metal	metal
Mo1P1Ru1_ICSD_98405	metal	metal
Mo1P1Zr1_ICSD_88150	metal	metal
Mo1P1_ICSD_186875	metal	metal
Mo1P1_ICSD_186876	metal	metal
Mo1P1_ICSD_644091	metal	metal
Mo1P2_ICSD_43331	metal	metal
Mo1P4_ICSD_105057	metal	metal
Mo1Pt1_ICSD_644160	metal	metal
Mo1Pt2Si3_ICSD_174199	metal	metal
Mo1Pt2_ICSD_161107	metal	metal
Mo1Pt3Si4_ICSD_174200	metal	metal
Mo1Pt3_ICSD_161108	metal	metal
Mo1Rb2S4_ICSD_280054	insulator	insulator
Mo1Rb2Se4_ICSD_644182	insulator	insulator
Mo1Rh1_ICSD_108608	metal	metal
Mo1Rh3_ICSD_105085	metal	metal
Mo1S2_ICSD_38401	insulator	metal
Mo1S2_ICSD_644249	metal	metal
Mo1S2_ICSD_95570	insulator	metal
Mo1Sb2Se1_ICSD_280615	metal	metal
Mo1Se2_ICSD_16948	insulator	insulator
Mo1Se2_ICSD_644335	insulator	insulator
Mo1Si1Zr1_ICSD_644448	metal	metal
Mo1Si2_ICSD_644411	metal	metal
Mo1Si2_ICSD_71503	insulator	metal
Mo1Sn2_ICSD_77114	metal	metal
Mo1Te2_ICSD_14349	metal	metal
Mo1Te2_ICSD_644476	insulator	metal
Mo1Ti1_ICSD_168951	metal	metal
Mo1U2_ICSD_105107	metal	metal
Mo1Zn6_ICSD_260686	metal	metal
Mo1Zn7_ICSD_644500	metal	metal
Mo1_ICSD_187346	metal	metal
Mo1_ICSD_187347	metal	metal
Mo1_ICSD_41513	metal	metal
Mo2N1_ICSD_30593	metal	metal
Mo2Na1O12P3_ICSD_202860	metal	insulator
Mo2Na2O7_ICSD_24312	insulator	insulator
Mo2Nd1O8Tl1_ICSD_161960	metal	insulator

Supplementary Table 511. Five-fold cross validated predictions for the metal/insulator classification (503/598).

system	calculated	predicted
Mo2Ni3O11Se1_ICSD_249582	insulator	insulator
Mo2Ni3O11Te1_ICSD_249583	insulator	insulator
Mo2Ni6P3_ICSD_20825	metal	metal
Mo2O11P2_ICSD_27157	insulator	insulator
Mo2O12Y5_ICSD_60957	insulator	insulator
Mo2O16P4Sr1_ICSD_411191	insulator	insulator
Mo2O7Tb2_ICSD_159772	metal	insulator
Mo2O7Y2_ICSD_202522	metal	metal
Mo2O8Pr1Rb1_ICSD_6290	insulator	insulator
Mo2O8Pr1Tl1_ICSD_152176	metal	insulator
Mo2O8Pr1Tl1_ICSD_421051	insulator	insulator
Mo2O8Sc1Tl1_ICSD_250340	insulator	insulator
Mo2O8U1_ICSD_28088	insulator	insulator
Mo2O8Zr1_ICSD_169483	insulator	insulator
Mo2O8Zr1_ICSD_280435	insulator	insulator
Mo2O8Zr1_ICSD_59999	insulator	insulator
Mo2O8Zr1_ICSD_98167	insulator	insulator
Mo2O9Zn3_ICSD_401828	insulator	insulator
Mo2S2Sb1_ICSD_97682	metal	metal
Mo2S3_ICSD_76371	metal	metal
Mo2S4V1_ICSD_201787	metal	metal
Mo2S4V1_ICSD_644314	metal	metal
Mo2Zr1_ICSD_105115	metal	metal
Mo3N1Ni2_ICSD_180405	metal	metal
Mo3N1Ni3_ICSD_162274	metal	metal
Mo3N1Pd2_ICSD_93502	metal	metal
Mo3N1Pt2_ICSD_150314	metal	metal
Mo3Na1Se3_ICSD_603625	metal	metal
Mo3Na1Te3_ICSD_603641	metal	metal
Mo3Na2O12Zr1_ICSD_200913	insulator	insulator
Mo3Na2O16Te3_ICSD_171758	insulator	insulator
Mo3O12Sc2_ICSD_20838	insulator	insulator
Mo3O12Tb2_ICSD_200857	insulator	insulator
Mo3O14P2Sr1_ICSD_74938	insulator	insulator
Mo3O16Pr5_ICSD_174567	metal	insulator
Mo3O8Sc1Zn1_ICSD_40534	insulator	insulator
Mo3O8Zn2_ICSD_260410	insulator	insulator
Mo3Os1_ICSD_644078	metal	metal
Mo3P1_ICSD_43238	metal	metal
Mo3P1_ICSD_644085	metal	metal
Mo3Pt1_ICSD_161106	metal	metal
Mo3Rb1S3_ICSD_30753	metal	metal
Mo3Rb1Se3_ICSD_604516	metal	metal
Mo3Rb1Te3_ICSD_603672	metal	metal
Mo3Sb7_ICSD_173974	metal	metal
Mo3Se3Tl1_ICSD_644381	metal	metal
Mo3Se4_ICSD_16316	metal	metal
Mo3Si1_ICSD_35755	metal	metal
Mo3Si2_ICSD_644412	metal	metal
Mo3Si4Tb2_ICSD_600647	metal	metal

Supplementary Table 512. Five-fold cross validated predictions for the metal/insulator classification (504/598).

system	calculated	predicted
Mo3Si4U2_ICSD_658196	metal	metal
Mo3Si4Y2_ICSD_76379	metal	metal
Mo3Sn1_ICSD_644455	metal	metal
Mo3Te3Tl1_ICSD_603670	metal	metal
Mo3Ti1_ICSD_168952	metal	metal
Mo3Zr1_ICSD_105118	metal	metal
Mo4N14Sr10_ICSD_413932	insulator	insulator
Mo4N1Ni2_ICSD_162277	metal	metal
Mo4Na1O6_ICSD_40962	metal	metal
Mo4Na5O16Tb1_ICSD_200928	insulator	insulator
Mo4Ni1Zr9_ICSD_644049	metal	metal
Mo4O11_ICSD_15815	metal	insulator
Mo4O15Tb2_ICSD_280784	insulator	insulator
Mo4O6Sn1_ICSD_92839	metal	metal
Mo5O16U1_ICSD_80162	insulator	insulator
Mo5Re24_ICSD_109289	metal	metal
Mo5Si3_ICSD_644410	metal	metal
Mo5Si3_ICSD_76377	metal	metal
Mo6Nd1S8_ICSD_603458	metal	metal
Mo6Nd1S8_ICSD_644010	metal	metal
Mo6Nd1Se8_ICSD_644011	metal	metal
Mo6Ni1Te8_ICSD_644045	metal	metal
Mo6O17Tl1_ICSD_62699	metal	insulator
Mo6O24Rb8Zr1_ICSD_165116	insulator	insulator
Mo6Pb1S8_ICSD_644102	metal	metal
Mo6Pb1Se8_ICSD_644122	metal	metal
Mo6Pr1S8_ICSD_603457	metal	metal
Mo6Pr1Se8_ICSD_644146	metal	metal
Mo6S8Sb1_ICSD_604444	metal	metal
Mo6S8Sm1_ICSD_600668	metal	metal
Mo6S8Sn1_ICSD_644281	metal	metal
Mo6S8Sr1_ICSD_644296	metal	metal
Mo6S8Tb1_ICSD_603462	metal	metal
Mo6S8Tl1_ICSD_644306	metal	metal
Mo6S8Y1_ICSD_644317	metal	metal
Mo6S8Yb1_ICSD_46014	metal	metal
Mo6S8Yb1_ICSD_644324	metal	metal
Mo6S8_ICSD_201579	metal	metal
Mo6Se8Sm1_ICSD_644358	metal	metal
Mo6Se8Sn1_ICSD_644359	metal	metal
Mo6Se8Tb1_ICSD_644370	metal	metal
Mo6Se8Tl1_ICSD_644379	metal	metal
Mo6Se8Y1_ICSD_644389	metal	metal
Mo6Se8Yb1_ICSD_644390	metal	metal
Mo6Te8_ICSD_59375	metal	metal
Mo8P5_ICSD_15058	metal	metal
Mo9Rb2S11_ICSD_280262	metal	metal
Mo9Se11_ICSD_170348	metal	metal
Mo9Se11_ICSD_40789	metal	metal
N0.2V0.6Zn0.4_ICSD_644866	metal	metal

Supplementary Table 513. Five-fold cross validated predictions for the metal/insulator classification (505/598).

system	calculated	predicted
N10Si7Sr1_ICSD_154166	insulator	insulator
N11P6Rb3_ICSD_51396	insulator	insulator
N11Pr3Si6_ICSD_402178	insulator	metal
N12P6S1Zn4_ICSD_76440	insulator	insulator
N12Rb2Zn1_ICSD_31314	insulator	insulator
N18W1_ICSD_413860	insulator	insulator
N1Na1O2_ICSD_174026	insulator	insulator
N1Na1O3_ICSD_15333	insulator	insulator
N1Na1Sn1_ICSD_172471	insulator	insulator
N1Na1_ICSD_184990	metal	metal
N1Na3O9P3Ti1_ICSD_172056	insulator	insulator
N1Na3_ICSD_165989	metal	metal
N1Na3_ICSD_165991	metal	insulator
N1Na3_ICSD_165992	metal	insulator
N1Na3_ICSD_165994	metal	metal
N1Na3_ICSD_421117	metal	metal
N1Na5O4W1_ICSD_74934	insulator	insulator
N1Nb1O1_ICSD_1031	insulator	insulator
N1Nb1_ICSD_183184	metal	metal
N1Nb1_ICSD_185442	metal	metal
N1Nb1_ICSD_185549	metal	metal
N1Nb1_ICSD_236423	metal	metal
N1Nb1_ICSD_644536	metal	metal
N1Nb1_ICSD_644540	metal	metal
N1Nb1_ICSD_644558	metal	metal
N1Nb2_ICSD_167805	metal	metal
N1Nb3Zn3_ICSD_42939	metal	metal
N1Nb4Ni2_ICSD_644564	metal	metal
N1Nd1O4_ICSD_260072	insulator	insulator
N1Nd1_ICSD_644583	metal	metal
N1Nd3Pb1_ICSD_76397	metal	metal
N1Nd3Sn1_ICSD_76398	metal	metal
N1Nd3Tl1_ICSD_76400	metal	metal
N1Ni1_ICSD_161755	metal	metal
N1Ni2Ta4_ICSD_644599	metal	metal
N1Ni2V4_ICSD_644603	metal	metal
N1Ni2W3_ICSD_86170	metal	metal
N1Ni2Zr4_ICSD_644605	metal	metal
N1Ni3Sn1_ICSD_183371	metal	metal
N1Ni3Zn1_ICSD_183370	metal	metal
N1Ni3_ICSD_76402	metal	metal
N1Ni3_ICSD_76635	metal	metal
N1Ni4_ICSD_76403	metal	metal
N1O1Se2Tb3_ICSD_420917	insulator	insulator
N1O1Ta1Zr1_ICSD_76012	metal	metal
N1O1Ta1_ICSD_173005	insulator	insulator
N1O1Ta1_ICSD_20321	metal	insulator
N1O1Ta1_ICSD_2363	insulator	insulator
N1O2Si1Y1_ICSD_44163	insulator	insulator
N1O2Sr1Ta1_ICSD_411137	insulator	insulator

Supplementary Table 514. Five-fold cross validated predictions for the metal/insulator classification (506/598).

system	calculated	predicted
N1O2Tl1_ICSD_50326	insulator	insulator
N1O3Rb1_ICSD_16338	insulator	insulator
N1O3Rb1_ICSD_35103	insulator	insulator
N1O3Tl1_ICSD_1818	insulator	insulator
N1Os1_ICSD_167513	metal	metal
N1Os1_ICSD_167514	metal	metal
N1Os1_ICSD_167863	metal	metal
N1Os1_ICSD_167879	metal	metal
N1Os1_ICSD_187715	metal	metal
N1Os1_ICSD_187716	metal	metal
N1Os2Zr4_ICSD_644614	metal	metal
N1P1V3_ICSD_644620	metal	metal
N1P3V5_ICSD_29285	metal	metal
N1Pa1_ICSD_644621	metal	metal
N1Pd1_ICSD_167857	metal	metal
N1Pd1_ICSD_169394	metal	metal
N1Pd1_ICSD_169396	metal	metal
N1Pd1_ICSD_169397	metal	metal
N1Pd1_ICSD_185564	metal	metal
N1Pd1_ICSD_185574	metal	metal
N1Pm1_ICSD_169216	metal	metal
N1Pr1_ICSD_168641	metal	metal
N1Pr1_ICSD_168642	metal	metal
N1Pr1_ICSD_168643	metal	metal
N1Pr1_ICSD_168644	metal	metal
N1Pr1_ICSD_181482	metal	metal
N1Pr1_ICSD_45161	metal	metal
N1Pr1_ICSD_644624	metal	metal
N1Pt1_ICSD_163863	metal	metal
N1Pt1_ICSD_163864	metal	metal
N1Pt1_ICSD_163865	metal	metal
N1Pt2Zr4_ICSD_644637	metal	metal
N1Pu1_ICSD_644640	metal	metal
N1Re1_ICSD_167878	metal	metal
N1Re1_ICSD_181297	metal	metal
N1Re1_ICSD_181299	metal	metal
N1Re1_ICSD_181300	metal	metal
N1Re1_ICSD_187711	metal	metal
N1Re1_ICSD_187713	metal	metal
N1Re1_ICSD_187714	metal	metal
N1Re2Zr4_ICSD_644656	metal	metal
N1Re2_ICSD_169887	metal	metal
N1Re3_ICSD_188352	metal	metal
N1Rh1_ICSD_183192	metal	metal
N1Rh1_ICSD_185553	metal	metal
N1Rh1_ICSD_185563	metal	metal
N1Rh1_ICSD_185573	metal	metal
N1Rh2Zr4_ICSD_644657	metal	metal
N1Ru1_ICSD_167855	metal	metal
N1Ru1_ICSD_183190	metal	metal

Supplementary Table 515. Five-fold cross validated predictions for the metal/insulator classification (507/598).

system	calculated	predicted
N1Ru1_ICSD_185562	metal	metal
N1Ru1_ICSD_185572	metal	metal
N1Ru2Zr4_ICSD_644658	metal	metal
N1S1_ICSD_41967	metal	insulator
N1S3Tb3_ICSD_416217	insulator	insulator
N1Sb1Sr3_ICSD_152052	insulator	insulator
N1Sc1_ICSD_157501	insulator	metal
N1Sc1_ICSD_180830	metal	insulator
N1Sn3Zr5_ICSD_656291	metal	metal
N1Sr1_ICSD_411555	metal	insulator
N1Sr2_ICSD_411612	metal	metal
N1Ta1_ICSD_105123	metal	metal
N1Ta1_ICSD_167876	metal	metal
N1Ta1_ICSD_167901	metal	metal
N1Ta1_ICSD_180958	metal	metal
N1Ta1_ICSD_183426	metal	metal
N1Ta1_ICSD_186417	metal	metal
N1Ta1_ICSD_644712	metal	metal
N1Ta1_ICSD_76457	metal	metal
N1Ta2_ICSD_167808	metal	metal
N1Tb1_ICSD_644738	metal	metal
N1Tc1_ICSD_182012	metal	metal
N1Tc1_ICSD_182015	metal	metal
N1Tc1_ICSD_185571	metal	metal
N1Tc1_ICSD_187707	metal	metal
N1Tc1_ICSD_187708	metal	metal
N1Tc1_ICSD_187709	metal	metal
N1Tc1_ICSD_187710	metal	metal
N1Tc2_ICSD_182011	metal	metal
N1Tc3_ICSD_182010	metal	metal
N1Th1_ICSD_61757	metal	metal
N1Ti1_ICSD_183416	metal	metal
N1Ti1_ICSD_644776	metal	metal
N1Ti2_ICSD_23403	metal	metal
N1Ti2_ICSD_33715	metal	metal
N1Ti3Tl1_ICSD_42931	metal	metal
N1Ti3Zn3_ICSD_644790	metal	metal
N1Tl1_ICSD_184575	metal	metal
N1Tl1_ICSD_186556	metal	metal
N1Tl1_ICSD_186557	metal	metal
N1Tm1_ICSD_57149	metal	metal
N1Tm1_ICSD_644802	metal	metal
N1U1_ICSD_60377	metal	metal
N1U1_ICSD_644816	metal	metal
N1V1_ICSD_169821	metal	metal
N1V1_ICSD_183421	metal	metal
N1V1_ICSD_188217	metal	metal
N1V1_ICSD_236425	metal	metal
N1V2_ICSD_167802	metal	metal
N1W1_ICSD_167861	metal	metal

Supplementary Table 516. Five-fold cross validated predictions for the metal/insulator classification (508/598).

system	calculated	predicted
N1W1_ICSD_167877	metal	metal
N1W1_ICSD_644869	metal	metal
N1W2_ICSD_30354	metal	metal
N1Y1_ICSD_161078	insulator	metal
N1Y1_ICSD_161079	metal	insulator
N1Y1_ICSD_183180	insulator	metal
N1Y1_ICSD_185493	metal	metal
N1Y1_ICSD_185557	insulator	metal
N1Y1_ICSD_185567	metal	metal
N1Y1_ICSD_37413	insulator	metal
N1Yb1_ICSD_169225	metal	metal
N1Zn3Zr3_ICSD_42935	metal	metal
N1Zr1_ICSD_161885	metal	metal
N1Zr1_ICSD_181144	metal	metal
N1Zr1_ICSD_183182	metal	metal
N1Zr1_ICSD_185558	metal	metal
N1Zr1_ICSD_185568	metal	metal
N1Zr1_ICSD_44506	metal	metal
N1_ICSD_187643	insulator	insulator
N1_ICSD_95247	metal	insulator
N21P3_ICSD_416415	insulator	insulator
N24O2P12Zn8_ICSD_417324	insulator	insulator
N2Na1Nb1_ICSD_72557	insulator	insulator
N2Na1P1_ICSD_411818	insulator	insulator
N2Na1Ta1_ICSD_76382	insulator	insulator
N2Na2O2_ICSD_66978	insulator	insulator
N2Na4O2W1_ICSD_73101	insulator	insulator
N2Ni1O6_ICSD_28327	insulator	insulator
N2Ni1Sr2_ICSD_91273	insulator	metal
N2O12Se3_ICSD_78900	insulator	insulator
N2O14S3_ICSD_16615	insulator	insulator
N2O1Si2_ICSD_34025	insulator	insulator
N2O1_ICSD_22397	insulator	insulator
N2O2S2Se1_ICSD_69978	insulator	insulator
N2O2S3_ICSD_41538	insulator	insulator
N2O2S4_ICSD_410547	insulator	insulator
N2O2_ICSD_27871	metal	insulator
N2O3_ICSD_66362	insulator	insulator
N2O4_ICSD_164631	insulator	insulator
N2O4_ICSD_201140	insulator	insulator
N2O4_ICSD_28331	insulator	insulator
N2O4_ICSD_29047	insulator	insulator
N2O4_ICSD_31175	insulator	insulator
N2O5S3_ICSD_14151	insulator	insulator
N2O5_ICSD_30639	insulator	insulator
N2O6Pb1_ICSD_56086	insulator	insulator
N2O6Sr1_ICSD_35494	insulator	insulator
N2O8Pb2Se1_ICSD_60893	insulator	insulator
N2Os1_ICSD_160621	metal	metal
N2Os1_ICSD_185513	metal	metal

Supplementary Table 517. Five-fold cross validated predictions for the metal/insulator classification (509/598).

system	calculated	predicted
N2Os1_ICSD_260545	metal	metal
N2Os1_ICSD_260549	metal	metal
N2Os1_ICSD_290439	metal	metal
N2Os1_ICSD_290441	metal	metal
N2P1U2_ICSD_23216	metal	metal
N2P1U2_ICSD_41945	metal	metal
N2Pb1S2_ICSD_49539	insulator	insulator
N2Pt1_ICSD_166462	insulator	insulator
N2Pt1_ICSD_166463	insulator	metal
N2Pt1_ICSD_290445	metal	metal
N2Pt1_ICSD_290446	metal	metal
N2Re1_ICSD_187441	metal	metal
N2Re1_ICSD_187442	metal	metal
N2Re1_ICSD_187444	metal	metal
N2Re1_ICSD_187446	metal	metal
N2Re1_ICSD_187447	metal	metal
N2Re1_ICSD_187448	metal	metal
N2Re1_ICSD_187449	metal	metal
N2Re1_ICSD_187450	metal	metal
N2Re1_ICSD_187451	metal	metal
N2Re1_ICSD_187452	metal	metal
N2Re1_ICSD_290436	metal	metal
N2Re1_ICSD_290438	metal	metal
N2Rh1_ICSD_160624	metal	metal
N2Rh1_ICSD_240760	metal	metal
N2Ru1_ICSD_240754	metal	metal
N2Ru1_ICSD_260550	metal	metal
N2S1Th2_ICSD_23213	insulator	insulator
N2S1U2_ICSD_23217	metal	metal
N2S1Zr2_ICSD_96970	insulator	insulator
N2S1Zr2_ICSD_96971	insulator	insulator
N2S2_ICSD_37353	insulator	metal
N2S4_ICSD_201218	insulator	insulator
N2Sb1Th2_ICSD_16062	metal	metal
N2Sb1U2_ICSD_644665	metal	metal
N2Se1Th2_ICSD_23211	insulator	insulator
N2Se1U2_ICSD_41943	metal	metal
N2Se1Zr2_ICSD_424266	insulator	insulator
N2Se3Tb4_ICSD_412928	insulator	insulator
N2Si1Sr1_ICSD_170270	insulator	insulator
N2Si1Zn1_ICSD_656276	insulator	insulator
N2Sr1Ti1_ICSD_85770	insulator	insulator
N2Sr1Zr1_ICSD_82537	insulator	insulator
N2Sr1_ICSD_423722	metal	metal
N2Sr2Zn1_ICSD_80376	insulator	insulator
N2Ta1_ICSD_290431	metal	metal
N2Ta1_ICSD_290432	metal	metal
N2Te1Th2_ICSD_16063	insulator	insulator
N2Te1U2_ICSD_16060	insulator	metal
N2U1V1_ICSD_644843	metal	metal

Supplementary Table 518. Five-fold cross validated predictions for the metal/insulator classification (510/598).

system	calculated	predicted
N2U1_ICSD_105129	insulator	metal
N2W1_ICSD_290433	insulator	metal
N2W1_ICSD_290434	insulator	insulator
N2W1_ICSD_290435	metal	metal
N2W1_ICSD_30364	metal	metal
N2Zn3_ICSD_84918	insulator	insulator
N2Zr1_ICSD_262746	metal	insulator
N2_ICSD_15819	insulator	insulator
N2_ICSD_24891	insulator	insulator
N2_ICSD_24892	insulator	insulator
N2_ICSD_28179	insulator	insulator
N2_ICSD_40936	insulator	insulator
N2_ICSD_644518	insulator	insulator
N3Na1Si2_ICSD_72466	insulator	insulator
N3Na1_ICSD_155167	insulator	metal
N3Na1_ICSD_24006	insulator	insulator
N3Na1_ICSD_29373	metal	insulator
N3Na1_ICSD_29383	insulator	insulator
N3Na1_ICSD_644523	insulator	insulator
N3Na3W1_ICSD_75364	insulator	insulator
N3Nb1Sr2_ICSD_51028	insulator	insulator
N3Nb4_ICSD_76389	metal	metal
N3O11Rb1U1_ICSD_23806	insulator	insulator
N3O1Ta2_ICSD_182352	metal	metal
N3P1Zn2_ICSD_422150	insulator	insulator
N3Rb1_ICSD_16963	insulator	insulator
N3Rb1_ICSD_25007	insulator	insulator
N3Rh1_ICSD_162107	metal	metal
N3Rh1_ICSD_162108	metal	metal
N3Sr2V1_ICSD_80176	insulator	insulator
N3Ta1Th1_ICSD_247344	metal	insulator
N3Ta1Th1_ICSD_247345	insulator	metal
N3Ta1Ti2_ICSD_186418	metal	metal
N3Ta1Zr2_ICSD_186419	metal	metal
N3Ta2Ti1_ICSD_186421	metal	metal
N3Ta2Zr1_ICSD_186422	metal	metal
N3Tb2_ICSD_644736	metal	metal
N3Th2_ICSD_76637	metal	insulator
N3Tl1_ICSD_34268	insulator	insulator
N3U2_ICSD_644812	metal	metal
N3U2_ICSD_644840	metal	metal
N3V2_ICSD_182700	metal	metal
N3W2_ICSD_186207	metal	metal
N4O12Pd1Rb2_ICSD_158878	insulator	insulator
N4O14Rb2U1_ICSD_28095	insulator	insulator
N4O2S4_ICSD_37165	insulator	insulator
N4O3S4_ICSD_25508	insulator	insulator
N4O3Si3Y2_ICSD_89931	insulator	insulator
N4O8Zr7_ICSD_245752	insulator	insulator
N4Se4_ICSD_14325	insulator	insulator

Supplementary Table 519. Five-fold cross validated predictions for the metal/insulator classification (511/598).

system	calculated	predicted
N4Se4_ICSD_74838	insulator	insulator
N4Si3_ICSD_156337	insulator	insulator
N4Si3_ICSD_164618	insulator	insulator
N4Si3_ICSD_16752	insulator	insulator
N4Si3_ICSD_67241	insulator	insulator
N4Si3_ICSD_74744	insulator	insulator
N4Si3_ICSD_74750	insulator	insulator
N4Si3_ICSD_97567	insulator	insulator
N4Si3_ICSD_98636	insulator	insulator
N4Sn3_ICSD_89525	metal	insulator
N4Ta2_ICSD_182351	metal	metal
N4Th3_ICSD_14336	insulator	metal
N4W3_ICSD_186209	metal	metal
N4Zr3_ICSD_78945	insulator	insulator
N4Zr3_ICSD_97998	insulator	metal
N5Na1Ta3_ICSD_84911	metal	insulator
N5Nb4_ICSD_644542	metal	metal
N5P3_ICSD_391073	insulator	insulator
N5P3_ICSD_411857	insulator	insulator
N5Ta3_ICSD_16253	insulator	metal
N5Ta4_ICSD_29328	metal	metal
N6Na3O12Rh1_ICSD_39571	insulator	insulator
N6Nb5_ICSD_26252	metal	metal
N6Ni1O12Pb2_ICSD_24510	metal	insulator
N6Ni1O12Sr2_ICSD_24509	metal	insulator
N6O1P4_ICSD_79420	insulator	insulator
N6S5_ICSD_200444	insulator	insulator
N6Sr1_ICSD_30337	insulator	insulator
N6Ta5_ICSD_76461	metal	metal
N7Na1P4_ICSD_410629	insulator	insulator
N7Si4Sr1Y1_ICSD_150459	insulator	insulator
N7Si4Sr1Yb1_ICSD_160650	insulator	insulator
N7Si4Sr1Yb1_ICSD_405625	metal	insulator
N8O6P6Sr3_ICSD_163603	insulator	insulator
N8Si5Sr2_ICSD_401500	insulator	insulator
N8Si6Sr1_ICSD_391265	insulator	insulator
N9Sb1_ICSD_413359	insulator	insulator
Na10P6Sn2_ICSD_300189	insulator	insulator
Na10Sn12Sr1_ICSD_240007	insulator	insulator
Na10Sn12Yb1_ICSD_172210	insulator	insulator
Na11O16U5_ICSD_15137	metal	insulator
Na15Pb4_ICSD_105160	metal	metal
Na15Sn4_ICSD_105167	metal	metal
Na1Nb1O2_ICSD_73111	insulator	insulator
Na1Nb1O3_ICSD_23239	insulator	insulator
Na1Nb1O3_ICSD_247314	insulator	insulator
Na1Nb1O3_ICSD_247318	insulator	insulator
Na1Nb1O3_ICSD_280098	insulator	insulator
Na1Nb1O3_ICSD_280100	insulator	insulator
Na1Nb1O3_ICSD_28566	insulator	insulator

Supplementary Table 520. Five-fold cross validated predictions for the metal/insulator classification (512/598).

system	calculated	predicted
Na1Nb1O3_ICSD_28570	insulator	insulator
Na1Nb1O3_ICSD_76432	insulator	insulator
Na1Nb1O3_ICSD_9645	insulator	insulator
Na1Nb1O6Sr3_ICSD_418494	insulator	insulator
Na1Nb1S2_ICSD_26285	insulator	insulator
Na1Nb1Se2_ICSD_26287	insulator	insulator
Na1Nb3O8_ICSD_63203	insulator	insulator
Na1Nd1O2_ICSD_31317	insulator	insulator
Na1Nd1O4Ti1_ICSD_82004	insulator	insulator
Na1Nd1O8S2_ICSD_90774	insulator	insulator
Na1Nd1S2_ICSD_644913	insulator	insulator
Na1Nd1Se2_ICSD_644914	insulator	insulator
Na1Ni1O2_ICSD_153372	insulator	insulator
Na1O11V6_ICSD_419551	insulator	metal
Na1O12P3Sn4_ICSD_409786	insulator	insulator
Na1O12P3Ti2_ICSD_203042	insulator	insulator
Na1O12P3V3_ICSD_202911	metal	insulator
Na1O12P3Zr2_ICSD_467	insulator	insulator
Na1O13Ti8_ICSD_69110	metal	insulator
Na1O2Pr1_ICSD_61682	metal	insulator
Na1O2Rh1_ICSD_66280	insulator	insulator
Na1O2Ru1_ICSD_170347	insulator	insulator
Na1O2Sc1_ICSD_25729	insulator	insulator
Na1O2Tb1_ICSD_61683	insulator	insulator
Na1O2Ti1_ICSD_43439	insulator	insulator
Na1O2Tl1_ICSD_644919	insulator	insulator
Na1O2V1_ICSD_420136	insulator	insulator
Na1O2V1_ICSD_420137	insulator	insulator
Na1O2V1_ICSD_420138	insulator	insulator
Na1O2Y1_ICSD_2740	insulator	insulator
Na1O2_ICSD_26583	metal	insulator
Na1O2_ICSD_87178	metal	insulator
Na1O3Rb1Ti1_ICSD_78753	insulator	insulator
Na1O3Sb1_ICSD_25540	insulator	insulator
Na1O3Sb1_ICSD_78416	insulator	insulator
Na1O3Sb1_ICSD_98978	insulator	insulator
Na1O3Ta1_ICSD_16687	insulator	insulator
Na1O3Ta1_ICSD_23322	insulator	insulator
Na1O3Ta1_ICSD_28606	insulator	insulator
Na1O3Ta1_ICSD_28609	insulator	insulator
Na1O3Ta1_ICSD_28610	insulator	insulator
Na1O3Ta1_ICSD_980	insulator	insulator
Na1O3V1_ICSD_29450	insulator	insulator
Na1O3W1_ICSD_28866	metal	insulator
Na1O3_ICSD_180566	insulator	metal
Na1O4P1Zn1_ICSD_87557	insulator	insulator
Na1O4Pd3_ICSD_245608	metal	metal
Na1O4Pr1Ti1_ICSD_422048	insulator	insulator
Na1O4Pt3_ICSD_185930	metal	insulator
Na1O4Re1_ICSD_78831	insulator	insulator

Supplementary Table 521. Five-fold cross validated predictions for the metal/insulator classification (513/598).

system	calculated	predicted
Na1O4Rh2_ICSD_170598	metal	insulator
Na1O4Ru2_ICSD_172608	metal	metal
Na1O4Si1Y1_ICSD_20161	insulator	insulator
Na1O4Si1Y1_ICSD_30384	insulator	insulator
Na1O4Ti1Y1_ICSD_81538	insulator	insulator
Na1O4V2_ICSD_159905	metal	insulator
Na1O5P1Ti1_ICSD_67538	insulator	insulator
Na1O5P1V1_ICSD_33944	insulator	insulator
Na1O5V2_ICSD_409210	insulator	insulator
Na1O6Os2_ICSD_246504	metal	metal
Na1O6Ru1Sr3_ICSD_401345	insulator	insulator
Na1O6S2Tl3_ICSD_1626	insulator	insulator
Na1O6Sb1Sr3_ICSD_51259	insulator	insulator
Na1O6Sc1Si2_ICSD_74553	insulator	insulator
Na1O6Si2Ti1_ICSD_281615	insulator	insulator
Na1O6Si2Ti1_ICSD_39194	insulator	insulator
Na1O6Si2V1_ICSD_78342	insulator	insulator
Na1O6Si2Y1_ICSD_171255	insulator	insulator
Na1O6Sr3Ta1_ICSD_418491	insulator	insulator
Na1O7P2Y1_ICSD_83657	insulator	insulator
Na1O8P1W2_ICSD_93368	metal	insulator
Na1O8Pr1Se2_ICSD_63408	insulator	insulator
Na1O8Rb3Ru2_ICSD_416038	insulator	insulator
Na1O8S2V1_ICSD_71760	insulator	insulator
Na1O8Sb5_ICSD_28494	insulator	insulator
Na1O8Ta3_ICSD_63204	insulator	insulator
Na1P1Pd1S4_ICSD_97456	insulator	insulator
Na1P1Sn1_ICSD_409010	insulator	insulator
Na1P1Sr1_ICSD_416887	insulator	insulator
Na1P1Zn1_ICSD_61083	insulator	insulator
Na1P1_ICSD_14009	insulator	insulator
Na1P2S6Sb1_ICSD_155269	insulator	insulator
Na1P2S6Sb1_ICSD_155270	insulator	insulator
Na1P5_ICSD_99177	insulator	insulator
Na1Pb3_ICSD_644932	metal	metal
Na1Pr1Se2_ICSD_644941	insulator	insulator
Na1Pr1Te2_ICSD_413184	metal	insulator
Na1Pt2_ICSD_644945	metal	metal
Na1S1_ICSD_182222	metal	insulator
Na1S2Sb1_ICSD_200597	insulator	insulator
Na1S2Sb1_ICSD_49016	insulator	insulator
Na1S2Sb1_ICSD_71091	insulator	insulator
Na1S2Sc1_ICSD_644971	insulator	insulator
Na1S2Sn1_ICSD_23450	metal	insulator
Na1S2Tb1_ICSD_644985	insulator	insulator
Na1S2Ti1_ICSD_26305	metal	insulator
Na1S2V1_ICSD_644993	metal	insulator
Na1S2V1_ICSD_644994	metal	insulator
Na1S2V1_ICSD_76541	insulator	metal
Na1S2Y1_ICSD_644997	insulator	insulator

Supplementary Table 522. Five-fold cross validated predictions for the metal/insulator classification (514/598).

system	calculated	predicted
Na1S2Yb1_ICSD_645003	metal	insulator
Na1S7Si1Y3_ICSD_412445	insulator	insulator
Na1Sb1Zn1_ICSD_645023	metal	insulator
Na1Sb1_ICSD_26473	insulator	insulator
Na1Se2Tb1_ICSD_645031	insulator	insulator
Na1Se2V1_ICSD_76548	insulator	metal
Na1Se2V1_ICSD_77597	metal	insulator
Na1Se2Y1_ICSD_645037	insulator	insulator
Na1Se2Yb1_ICSD_409678	metal	insulator
Na1Sn2_ICSD_170467	metal	metal
Na1Sn5_ICSD_408045	metal	metal
Na1Tl1_ICSD_108619	metal	metal
Na1Tl1_ICSD_262065	metal	metal
Na1Tl1_ICSD_262066	metal	metal
Na1Zn13_ICSD_105173	metal	metal
Na1_ICSD_159431	metal	metal
Na1_ICSD_182587	metal	metal
Na1_ICSD_189460	metal	metal
Na1_ICSD_44758	metal	metal
Na1_ICSD_644901	metal	metal
Na1_ICSD_644902	metal	metal
Na2Ni1O14P4Zr1_ICSD_65703	insulator	insulator
Na2Ni1O2Rb1_ICSD_73209	metal	insulator
Na2Ni1O2_ICSD_14159	insulator	insulator
Na2O11Si4Ti1_ICSD_16899	insulator	insulator
Na2O11Si4Zr1_ICSD_6267	insulator	insulator
Na2O12P4Sr1_ICSD_37171	insulator	insulator
Na2O13Ti6_ICSD_182965	insulator	insulator
Na2O1S2Ti1_ICSD_67886	insulator	insulator
Na2O1Sb2Ti2_ICSD_91209	metal	insulator
Na2O1_ICSD_644917	insulator	insulator
Na2O2Pb1_ICSD_21058	insulator	insulator
Na2O2Pt1_ICSD_25018	insulator	insulator
Na2O2Zn1_ICSD_404761	insulator	insulator
Na2O2_ICSD_109276	insulator	insulator
Na2O3Pr1_ICSD_64664	insulator	insulator
Na2O3Pt1_ICSD_25019	insulator	insulator
Na2O3Pt1_ICSD_25020	metal	insulator
Na2O3Ru1_ICSD_97584	insulator	insulator
Na2O3S1_ICSD_4432	insulator	insulator
Na2O3S2_ICSD_60026	insulator	insulator
Na2O3Se1_ICSD_89638	insulator	insulator
Na2O3Si1_ICSD_15388	insulator	insulator
Na2O3Tb1_ICSD_64665	metal	insulator
Na2O3Ti1_ICSD_183666	insulator	insulator
Na2O3Zn2_ICSD_25617	insulator	insulator
Na2O3Zn2_ICSD_404913	insulator	insulator
Na2O4Os1_ICSD_261702	insulator	insulator
Na2O4Pd3_ICSD_6157	insulator	insulator
Na2O4S1_ICSD_27955	metal	insulator

Supplementary Table 523. Five-fold cross validated predictions for the metal/insulator classification (515/598).

system	calculated	predicted
Na2O4S1_ICSD_66555	insulator	insulator
Na2O4S1_ICSD_81506	insulator	insulator
Na2O4S2_ICSD_16646	insulator	insulator
Na2O4Se1_ICSD_150706	insulator	insulator
Na2O4Si1Zn1_ICSD_18314	insulator	insulator
Na2O4Si1Zn1_ICSD_83312	insulator	insulator
Na2O4Te1_ICSD_1108	insulator	insulator
Na2O4U1_ICSD_20142	insulator	insulator
Na2O4U1_ICSD_20503	insulator	insulator
Na2O4U1_ICSD_20579	insulator	insulator
Na2O4U1_ICSD_79423	insulator	insulator
Na2O4U1_ICSD_79424	insulator	insulator
Na2O4W1_ICSD_28474	insulator	insulator
Na2O5Si1Ti1_ICSD_1533	insulator	insulator
Na2O5Si1Ti1_ICSD_82153	insulator	insulator
Na2O6Si2Zn1_ICSD_16160	insulator	insulator
Na2O6Si2Zn1_ICSD_20837	insulator	insulator
Na2O6Si2Zn1_ICSD_83427	insulator	insulator
Na2O6V2_ICSD_20556	insulator	insulator
Na2O7P2Pb1_ICSD_92179	insulator	insulator
Na2O7P2Pd1_ICSD_72751	insulator	insulator
Na2O7S2_ICSD_413049	insulator	insulator
Na2O7Sb4_ICSD_914	insulator	insulator
Na2O7Si2Zr1_ICSD_24866	insulator	insulator
Na2O7Si3_ICSD_81134	insulator	insulator
Na2O7Te2_ICSD_391382	insulator	insulator
Na2O7Ti3_ICSD_15463	insulator	insulator
Na2O7V2Zn1_ICSD_153966	insulator	insulator
Na2O7W2_ICSD_1883	insulator	insulator
Na2O8P2V1_ICSD_50376	insulator	insulator
Na2O8S2_ICSD_171374	insulator	insulator
Na2O8Si3Zn1_ICSD_924	insulator	insulator
Na2O9Te3Zn2_ICSD_24434	insulator	insulator
Na2Pd1S2_ICSD_76533	insulator	insulator
Na2Pd1Se2_ICSD_644936	insulator	insulator
Na2Pt1S2_ICSD_87219	insulator	insulator
Na2Pt1Se2_ICSD_40429	insulator	insulator
Na2Pt4Se6_ICSD_78788	insulator	insulator
Na2Rb1Si17_ICSD_91240	metal	metal
Na2S1_ICSD_644959	insulator	insulator
Na2S1_ICSD_92771	insulator	insulator
Na2S1_ICSD_92772	insulator	insulator
Na2S2Zn1_ICSD_33235	insulator	insulator
Na2S2_ICSD_43407	insulator	insulator
Na2S2_ICSD_73180	insulator	insulator
Na2S3U1_ICSD_88097	metal	insulator
Na2S4_ICSD_644957	insulator	insulator
Na2S5Si2_ICSD_166532	insulator	insulator
Na2S5_ICSD_38349	insulator	insulator
Na2Se1_ICSD_645027	insulator	insulator

Supplementary Table 524. Five-fold cross validated predictions for the metal/insulator classification (516/598).

system	calculated	predicted
Na2Se2_ICSD_43408	insulator	insulator
Na2Se3Sn1_ICSD_300237	insulator	insulator
Na2Se3Te1_ICSD_63011	insulator	insulator
Na2Se3Zr1_ICSD_412158	insulator	insulator
Na2Se4_ICSD_402584	insulator	insulator
Na2Sn4Sr1_ICSD_261117	metal	metal
Na2Te1_ICSD_76553	insulator	insulator
Na2Tl1_ICSD_105171	metal	metal
Na2Tl2_ICSD_261793	metal	metal
Na3Nb1O4_ICSD_6116	insulator	insulator
Na3O12P3Sc2_ICSD_20740	insulator	insulator
Na3O1P1S3_ICSD_98651	insulator	insulator
Na3O1S3V1_ICSD_281593	insulator	insulator
Na3O2Tl1_ICSD_202028	insulator	insulator
Na3O3P1S1_ICSD_412368	insulator	insulator
Na3O3Sb1_ICSD_23346	insulator	insulator
Na3O4Sb1_ICSD_10320	insulator	insulator
Na3O5Os1_ICSD_416999	insulator	insulator
Na3O5Re1_ICSD_36637	insulator	insulator
Na3P1S4_ICSD_72860	insulator	insulator
Na3P1_ICSD_26884	insulator	insulator
Na3S3Sb1_ICSD_644966	insulator	insulator
Na3S4Sb1_ICSD_44707	insulator	insulator
Na3Sb1Se3_ICSD_425125	insulator	insulator
Na3Sb1Se4_ICSD_65141	insulator	insulator
Na3Sb1Te3_ICSD_75513	insulator	insulator
Na3Sb1_ICSD_26882	insulator	metal
Na4O12P3Sc2_ICSD_202833	insulator	insulator
Na4O12Si3Zr2_ICSD_38056	insulator	insulator
Na4O12Ti5_ICSD_170677	insulator	insulator
Na4O3Sn1_ICSD_49624	insulator	insulator
Na4O4Pb1_ICSD_74859	insulator	insulator
Na4O4Si1_ICSD_15500	insulator	insulator
Na4O4Sn1_ICSD_202818	insulator	insulator
Na4O4Ti1_ICSD_69621	insulator	insulator
Na4O5Te1_ICSD_202325	insulator	insulator
Na4O5U1_ICSD_68360	insulator	insulator
Na4O5W1_ICSD_85063	insulator	insulator
Na4O7P2_ICSD_10370	insulator	insulator
Na4O9Si3Sr1_ICSD_33943	insulator	insulator
Na4P2Se6_ICSD_415240	insulator	insulator
Na4S4Sn1_ICSD_42035	insulator	insulator
Na4Se4Si1_ICSD_409726	insulator	insulator
Na4Se4Sn1_ICSD_72384	insulator	insulator
Na4Si23_ICSD_248168	metal	insulator
Na4Si4_ICSD_409953	insulator	metal
Na4Sn1Te4_ICSD_37184	insulator	insulator
Na5Nb1O5_ICSD_24819	insulator	insulator
Na5Ni1O2S1_ICSD_412972	metal	insulator
Na5O10P3_ICSD_25837	insulator	insulator

Supplementary Table 525. Five-fold cross validated predictions for the metal/insulator classification (517/598).

system	calculated	predicted
Na5O5Ta1_ICSD_72297	insulator	insulator
Na5O6Os1_ICSD_49746	insulator	insulator
Na5O6Re1_ICSD_38382	insulator	insulator
Na6O4Pb1_ICSD_21059	insulator	insulator
Na6O4Zn1_ICSD_6161	insulator	insulator
Na6O5Pb1_ICSD_15102	insulator	insulator
Na6O5Pb1_ICSD_21036	insulator	insulator
Na6O9S2_ICSD_411442	insulator	insulator
Na6P4Pb3S16_ICSD_280724	insulator	insulator
Na6P4W1_ICSD_71647	insulator	insulator
Na6S4Zn1_ICSD_33236	insulator	insulator
Na6S7Sn2_ICSD_25389	insulator	insulator
Na6Si2Te6_ICSD_15579	insulator	insulator
Na6Sn2Zn1_ICSD_260159	metal	metal
Na8O18Si6Sn1_ICSD_20768	insulator	insulator
Na8Sb4Sn1_ICSD_76496	metal	insulator
Na9Sn4_ICSD_105166	metal	metal
Nb11O30Rb4_ICSD_200367	insulator	insulator
Nb1Nd1O4_ICSD_246956	insulator	insulator
Nb1Nd3O7_ICSD_109060	insulator	insulator
Nb1Ni1P1_ICSD_49728	metal	metal
Nb1Ni1P2_ICSD_645092	metal	metal
Nb1Ni1Si1_ICSD_645107	metal	metal
Nb1Ni1Te5_ICSD_73316	metal	metal
Nb1Ni2Sn1_ICSD_105182	metal	metal
Nb1Ni2_ICSD_188262	metal	metal
Nb1Ni2_ICSD_188271	metal	metal
Nb1Ni3_ICSD_188229	metal	metal
Nb1Ni3_ICSD_188241	metal	metal
Nb1Ni3_ICSD_645068	metal	metal
Nb1O1_ICSD_40318	metal	metal
Nb1O1_ICSD_61634	metal	metal
Nb1O1_ICSD_95729	metal	metal
Nb1O2_ICSD_35181	insulator	insulator
Nb1O2_ICSD_75198	metal	metal
Nb1O3Rb1_ICSD_16380	insulator	insulator
Nb1O3Sr1_ICSD_42004	metal	metal
Nb1O3Sr1_ICSD_88723	metal	insulator
Nb1O4Sb1_ICSD_40011	insulator	insulator
Nb1O4Y1_ICSD_20335	insulator	insulator
Nb1O5P1_ICSD_36626	insulator	insulator
Nb1O5P1_ICSD_51144	insulator	insulator
Nb1O5P1_ICSD_51150	insulator	insulator
Nb1O5P1_ICSD_93769	insulator	insulator
Nb1O7Pr3_ICSD_78007	insulator	insulator
Nb1O8Rb3_ICSD_407327	insulator	insulator
Nb1O9Rb1S2_ICSD_28085	insulator	insulator
Nb1Os1Te4_ICSD_656450	metal	metal
Nb1P1Rh1_ICSD_645173	metal	metal
Nb1P1Ru1_ICSD_645175	metal	metal

Supplementary Table 526. Five-fold cross validated predictions for the metal/insulator classification (518/598).

system	calculated	predicted
Nb1P1S1_ICSD_16075	metal	metal
Nb1P1Se1_ICSD_16076	metal	metal
Nb1P1V1_ICSD_645178	metal	metal
Nb1P1Zr1_ICSD_75008	metal	metal
Nb1P1_ICSD_645167	metal	metal
Nb1P1_ICSD_76027	metal	metal
Nb1P2S8_ICSD_38376	insulator	insulator
Nb1P2_ICSD_645169	metal	metal
Nb1Pb0.33S2_ICSD_74694	metal	metal
Nb1Pb1S2_ICSD_74698	metal	metal
Nb1Pd1Si1_ICSD_416177	metal	metal
Nb1Pd1Te5_ICSD_68103	metal	metal
Nb1Pd2_ICSD_645190	metal	metal
Nb1Pd3_ICSD_105192	metal	metal
Nb1Pt1Si1_ICSD_90433	metal	metal
Nb1Pt1_ICSD_645211	metal	metal
Nb1Pt2_ICSD_645233	metal	metal
Nb1Pt3_ICSD_645210	metal	metal
Nb1Pt3_ICSD_645212	metal	metal
Nb1Re1Si1_ICSD_645257	metal	metal
Nb1Rh1Sb1_ICSD_107122	metal	metal
Nb1Rh1Si1_ICSD_90434	metal	metal
Nb1Rh1Sn1_ICSD_105220	metal	metal
Nb1Rh1_ICSD_645263	metal	metal
Nb1Rh1_ICSD_645264	metal	metal
Nb1Rh1_ICSD_645280	metal	metal
Nb1Rh3_ICSD_645261	metal	metal
Nb1Rh3_ICSD_645275	metal	metal
Nb1Ru1Sb1_ICSD_83929	insulator	metal
Nb1Ru1Si1_ICSD_600057	metal	metal
Nb1Ru1_ICSD_105221	metal	metal
Nb1Ru1_ICSD_105222	metal	metal
Nb1Ru3_ICSD_105224	metal	metal
Nb1S1_ICSD_44992	metal	metal
Nb1S1_ICSD_76564	metal	metal
Nb1S2Sn0.333_ICSD_83038	metal	metal
Nb1S2Sn0.33_ICSD_74695	metal	metal
Nb1S2Sn1_ICSD_100389	metal	metal
Nb1S2_ICSD_24755	metal	metal
Nb1S2_ICSD_43697	metal	metal
Nb1S2_ICSD_50648	metal	metal
Nb1S2_ICSD_645321	metal	metal
Nb1S2_ICSD_66358	metal	metal
Nb1S2_ICSD_68968	metal	metal
Nb1S2_ICSD_72725	metal	metal
Nb1S2_ICSD_79514	metal	metal
Nb1S2_ICSD_85578	metal	metal
Nb1S3_ICSD_2380	insulator	metal
Nb1S3_ICSD_645316	metal	metal
Nb1S4Tl3_ICSD_600246	insulator	insulator

Supplementary Table 527. Five-fold cross validated predictions for the metal/insulator classification (519/598).

system	calculated	predicted
Nb1Sb2_ICSD_18144	metal	metal
Nb1Se2_ICSD_18129	metal	metal
Nb1Se2_ICSD_18132	metal	metal
Nb1Se2_ICSD_645379	metal	metal
Nb1Se2_ICSD_645391	metal	metal
Nb1Se2_ICSD_73093	metal	metal
Nb1Se2_ICSD_76576	metal	metal
Nb1Se3_ICSD_30002	metal	metal
Nb1Se4Tl3_ICSD_600249	insulator	insulator
Nb1Se4_ICSD_645375	metal	metal
Nb1Si2_ICSD_645422	metal	metal
Nb1Si2_ICSD_645450	metal	metal
Nb1Sn2_ICSD_105229	metal	metal
Nb1Sn2_ICSD_645495	metal	metal
Nb1Te2_ICSD_14389	metal	metal
Nb1Te2_ICSD_645529	metal	metal
Nb1Te4_ICSD_60603	metal	metal
Nb1Te4_ICSD_645526	metal	metal
Nb1Zn2_ICSD_105257	metal	metal
Nb1Zn3_ICSD_105258	metal	metal
Nb1_ICSD_0	metal	metal
Nb1_ICSD_41512	metal	metal
Nb1_ICSD_645059	metal	metal
Nb21S8_ICSD_76568	metal	metal
Nb2Ni1O6_ICSD_15853	insulator	insulator
Nb2Ni1S4_ICSD_645093	metal	metal
Nb2Ni1_ICSD_188268	metal	metal
Nb2Ni2Te4_ICSD_84209	metal	metal
Nb2O10Ti1U1_ICSD_23404	insulator	insulator
Nb2O11Te3_ICSD_10034	insulator	insulator
Nb2O13Se4_ICSD_85668	insulator	insulator
Nb2O3_ICSD_263119	metal	metal
Nb2O5_ICSD_188460	insulator	insulator
Nb2O5_ICSD_25765	insulator	insulator
Nb2O5_ICSD_51176	insulator	insulator
Nb2O6Pb1_ICSD_166552	insulator	insulator
Nb2O6Sn1_ICSD_202827	insulator	insulator
Nb2O6Sr1_ICSD_20348	insulator	insulator
Nb2O6Zn1_ICSD_36290	insulator	insulator
Nb2O7Sn2_ICSD_163817	insulator	insulator
Nb2O8Zn3_ICSD_66147	insulator	insulator
Nb2O9Sr4_ICSD_79217	insulator	insulator
Nb2O9Zn4_ICSD_98563	insulator	insulator
Nb2P1S10Tl1_ICSD_417943	insulator	insulator
Nb2P1S10_ICSD_33233	insulator	insulator
Nb2P2Rb2S11_ICSD_412277	insulator	insulator
Nb2P5_ICSD_23281	metal	metal
Nb2Pd3Se8_ICSD_49523	insulator	insulator
Nb2S11Tl4_ICSD_411602	insulator	insulator
Nb2Se11Tl4_ICSD_171148	insulator	insulator

Supplementary Table 528. Five-fold cross validated predictions for the metal/insulator classification (520/598).

system	calculated	predicted
Nb2Se1_ICSD_26183	metal	metal
Nb2Se3_ICSD_42981	metal	metal
Nb2Se4Ti1_ICSD_645395	metal	metal
Nb2Se4V1_ICSD_645402	metal	metal
Nb2Se9_ICSD_8179	insulator	metal
Nb2Si5V4_ICSD_76587	metal	metal
Nb2Te3_ICSD_280016	metal	metal
Nb3.33Pt1.67_ICSD_645232	metal	metal
Nb3Ni1_ICSD_188226	metal	metal
Nb3Ni1_ICSD_188238	metal	metal
Nb3Ni1_ICSD_188250	metal	metal
Nb3Ni2Si1_ICSD_20443	metal	metal
Nb3O10Rb1Sr2_ICSD_93674	insulator	insulator
Nb3O10U1_ICSD_26997	insulator	insulator
Nb3Os1_ICSD_603783	metal	metal
Nb3P1_ICSD_43206	metal	metal
Nb3Pb1_ICSD_105189	metal	metal
Nb3Pt1_ICSD_645218	metal	metal
Nb3Rh1Se6_ICSD_645284	metal	metal
Nb3Rh1_ICSD_645269	metal	metal
Nb3S4_ICSD_645317	metal	metal
Nb3S6V1_ICSD_645340	metal	metal
Nb3Sb1_ICSD_645347	metal	metal
Nb3Sb2Te5_ICSD_417101	insulator	metal
Nb3Sc2Si4_ICSD_600920	metal	metal
Nb3Se4_ICSD_43281	metal	metal
Nb3Si1Te6_ICSD_71681	metal	metal
Nb3Si1_ICSD_108629	metal	metal
Nb3Si1_ICSD_1997	metal	metal
Nb3Si1_ICSD_645413	metal	metal
Nb3Si1_ICSD_645416	metal	metal
Nb3Si2_ICSD_645431	metal	metal
Nb3Sn1_ICSD_105231	metal	metal
Nb3Te1_ICSD_105244	metal	metal
Nb3Te4_ICSD_16606	metal	metal
Nb3Tl1_ICSD_105251	metal	metal
Nb4Ni1P1_ICSD_645089	metal	metal
Nb4Ni1S8_ICSD_645094	metal	metal
Nb4Ni1Se8_ICSD_645099	metal	metal
Nb4Ni1Si1_ICSD_76559	metal	metal
Nb4O5_ICSD_39595	metal	metal
Nb4O6Sr1_ICSD_79355	metal	metal
Nb4S8Ti1_ICSD_645332	metal	metal
Nb4S8V1_ICSD_645339	metal	metal
Nb4Si5V2_ICSD_645460	metal	metal
Nb5Ni1_ICSD_105179	metal	metal
Nb5Ni4P4_ICSD_645087	metal	metal
Nb5O17Sr5_ICSD_79699	metal	insulator
Nb5O1Pt3_ICSD_36277	metal	metal
Nb5O9Sr2_ICSD_71859	metal	metal

Supplementary Table 529. Five-fold cross validated predictions for the metal/insulator classification (521/598).

system	calculated	predicted
Nb5P1Si3_ICSD_165098	metal	metal
Nb5P4Pd4_ICSD_280273	metal	metal
Nb5Sb4_ICSD_154596	metal	metal
Nb5Sb4_ICSD_76573	metal	metal
Nb5Se4_ICSD_76582	metal	metal
Nb5Se8Sn1_ICSD_657512	metal	metal
Nb5Se8Sr1_ICSD_657513	metal	metal
Nb5Si1Sn2_ICSD_16499	metal	metal
Nb5Si3_ICSD_189717	metal	metal
Nb5Si3_ICSD_645428	metal	metal
Nb5Te4_ICSD_105245	metal	metal
Nb6Ni16Si7_ICSD_109220	metal	metal
Nb6Ni6O1_ICSD_74998	metal	metal
Nb6Ni7_ICSD_188280	metal	metal
Nb6O16Sr1_ICSD_60783	insulator	insulator
Nb6Sn5_ICSD_645476	metal	metal
Nb6Sn6Tb1_ICSD_261123	metal	metal
Nb6Zn7_ICSD_645562	metal	metal
Nb7Ni6_ICSD_645077	metal	metal
Nb7P4_ICSD_41238	metal	metal
Nb8O14Sr1_ICSD_202673	insulator	insulator
Nb9O25P1_ICSD_18163	insulator	insulator
Nd1Ni1O2_ICSD_98585	metal	metal
Nd1Ni1P1_ICSD_645618	metal	metal
Nd1Ni1Sb2_ICSD_645624	metal	metal
Nd1Ni1Si2_ICSD_658589	metal	metal
Nd1Ni1Sn1_ICSD_105161	metal	metal
Nd1Ni1Sn2_ICSD_645652	metal	metal
Nd1Ni1_ICSD_645586	metal	metal
Nd1Ni2P2_ICSD_659935	metal	metal
Nd1Ni2Sb2_ICSD_645622	metal	metal
Nd1Ni2Sb2_ICSD_645623	metal	metal
Nd1Ni2Si2_ICSD_105271	metal	metal
Nd1Ni2Sn2_ICSD_160053	metal	metal
Nd1Ni2Sn2_ICSD_645651	metal	metal
Nd1Ni2_ICSD_105265	metal	metal
Nd1Ni4Sn2_ICSD_645647	metal	metal
Nd1Ni5_ICSD_105267	metal	metal
Nd1Ni5_ICSD_645598	metal	metal
Nd1Ni5_ICSD_645614	metal	metal
Nd1O1P1Zn1_ICSD_85776	insulator	insulator
Nd1O1Sb1Zn1_ICSD_182896	metal	metal
Nd1O1Sb1Zn1_ICSD_419354	metal	metal
Nd1O2Rb1_ICSD_27332	insulator	insulator
Nd1O3Rh1_ICSD_172349	insulator	insulator
Nd1O3V1_ICSD_28928	metal	metal
Nd1O4P1_ICSD_184552	insulator	insulator
Nd1O4P1_ICSD_184558	insulator	insulator
Nd1O4P1_ICSD_31565	insulator	insulator
Nd1O4P1_ICSD_62311	insulator	insulator

Supplementary Table 530. Five-fold cross validated predictions for the metal/insulator classification (522/598).

system	calculated	predicted
Nd1O4Sb1_ICSD_245048	insulator	insulator
Nd1O4Ta1_ICSD_415430	insulator	insulator
Nd1O4Ta1_ICSD_79498	insulator	insulator
Nd1O4Ta1_ICSD_90451	insulator	insulator
Nd1O4V1_ICSD_78077	metal	insulator
Nd1O8Rb1W2_ICSD_155378	insulator	insulator
Nd1O9P3_ICSD_2034	insulator	insulator
Nd1O9Ta3_ICSD_66284	insulator	insulator
Nd1Os1Si3_ICSD_645674	metal	metal
Nd1Os2Si2_ICSD_645676	metal	metal
Nd1Os2_ICSD_150520	metal	metal
Nd1Os4P12_ICSD_645670	metal	metal
Nd1Os4Sb12_ICSD_645672	metal	metal
Nd1P12Ru4_ICSD_645686	metal	metal
Nd1P1Pd1_ICSD_710050	metal	metal
Nd1P1_ICSD_645680	metal	metal
Nd1P2Rh2_ICSD_645685	metal	metal
Nd1P2Ru2_ICSD_602103	metal	metal
Nd1P5_ICSD_358	metal	metal
Nd1Pb3_ICSD_645697	metal	metal
Nd1Pd1Sb1_ICSD_645716	metal	metal
Nd1Pd1Sb2_ICSD_658220	metal	metal
Nd1Pd1Sn1_ICSD_419191	metal	metal
Nd1Pd1Sn1_ICSD_54460	metal	metal
Nd1Pd1Tl1_ICSD_105275	metal	metal
Nd1Pd1Zn1_ICSD_183337	metal	metal
Nd1Pd1_ICSD_645704	metal	metal
Nd1Pd2Si1_ICSD_645717	metal	metal
Nd1Pd2Si2_ICSD_601628	metal	metal
Nd1Pd3S4_ICSD_84666	metal	metal
Nd1Pd3_ICSD_656117	metal	metal
Nd1Pt1Sb1_ICSD_76600	metal	metal
Nd1Pt1Si1_ICSD_645745	metal	metal
Nd1Pt1Sn1_ICSD_415952	metal	metal
Nd1Pt1Sn1_ICSD_415953	metal	metal
Nd1Pt1_ICSD_105278	metal	metal
Nd1Pt1_ICSD_645730	metal	metal
Nd1Pt2Si2_ICSD_601629	metal	metal
Nd1Pt2Si2_ICSD_645746	metal	metal
Nd1Pt2Si2_ICSD_645749	metal	metal
Nd1Pt2_ICSD_105279	metal	metal
Nd1Pt3_ICSD_105280	metal	metal
Nd1Pt5_ICSD_658356	metal	metal
Nd1Rb1S2_ICSD_81397	insulator	insulator
Nd1Rb1Se2_ICSD_281070	insulator	insulator
Nd1Rb1Te2_ICSD_413330	insulator	metal
Nd1Rb1Te2_ICSD_98659	metal	insulator
Nd1Rb1Te4_ICSD_412793	insulator	insulator
Nd1Re2_ICSD_645751	metal	metal
Nd1Rh1Si2_ICSD_603359	metal	metal

Supplementary Table 531. Five-fold cross validated predictions for the metal/insulator classification (523/598).

system	calculated	predicted
Nd1Rh1Si3_ICSD_645778	metal	metal
Nd1Rh1Sn1_ICSD_154856	metal	metal
Nd1Rh1Sn1_ICSD_657267	metal	metal
Nd1Rh1_ICSD_645758	metal	metal
Nd1Rh2Si2_ICSD_105284	metal	metal
Nd1Rh2Sn4_ICSD_164992	metal	metal
Nd1Rh2_ICSD_105283	metal	metal
Nd1Rh3Si2_ICSD_645774	metal	metal
Nd1Rh3Si2_ICSD_76602	metal	metal
Nd1Rh3_ICSD_645759	metal	metal
Nd1Ru1Si1_ICSD_41251	metal	metal
Nd1Ru1Si2_ICSD_66316	metal	metal
Nd1Ru1Si3_ICSD_645815	metal	metal
Nd1Ru1Sn3_ICSD_601202	metal	metal
Nd1Ru1Sn3_ICSD_657114	metal	metal
Nd1Ru2Si2_ICSD_645811	metal	metal
Nd1Ru2Si2_ICSD_90298	metal	metal
Nd1Ru2_ICSD_645792	metal	metal
Nd1Ru2_ICSD_645798	metal	metal
Nd1Ru2_ICSD_645805	metal	metal
Nd1Ru4Sb12_ICSD_645809	metal	metal
Nd1S1_ICSD_645849	metal	metal
Nd1S2Tl1_ICSD_57403	insulator	insulator
Nd1S2_ICSD_645846	metal	metal
Nd1S2_ICSD_656242	metal	metal
Nd1Sb1Te1_ICSD_601643	metal	metal
Nd1Sb1_ICSD_76738	metal	metal
Nd1Sb1_ICSD_92168	metal	metal
Nd1Sb2_ICSD_645892	metal	metal
Nd1Sc1Si1_ICSD_645909	metal	metal
Nd1Se1.9_ICSD_65143	insulator	insulator
Nd1Se1_ICSD_645915	metal	metal
Nd1Se2Tl1_ICSD_106988	insulator	metal
Nd1Se2_ICSD_645919	metal	metal
Nd1Si1_ICSD_42944	metal	metal
Nd1Si2_ICSD_645972	metal	metal
Nd1Si2_ICSD_645987	metal	metal
Nd1Si2_ICSD_76665	metal	metal
Nd1Sn1Zn1_ICSD_426467	metal	metal
Nd1Sn2_ICSD_54292	metal	metal
Nd1Sn3_ICSD_657378	metal	metal
Nd1Te1_ICSD_646023	metal	metal
Nd1Te2Tl1_ICSD_646038	insulator	metal
Nd1Te2_ICSD_646009	metal	metal
Nd1Tl1Zn1_ICSD_646055	metal	metal
Nd1Tl1_ICSD_105292	metal	metal
Nd1Tl3_ICSD_105293	metal	metal
Nd1Zn11_ICSD_105303	metal	metal
Nd1Zn1_ICSD_105299	metal	metal
Nd1Zn2_ICSD_105301	metal	metal

Supplementary Table 532. Five-fold cross validated predictions for the metal/insulator classification (524/598).

system	calculated	predicted
Nd1Zn2_ICSD_646064	metal	metal
Nd1Zn5_ICSD_105302	metal	metal
Nd1_ICSD_43571	metal	metal
Nd1_ICSD_43577	metal	metal
Nd1_ICSD_645578	metal	metal
Nd2Ni12P7_ICSD_602470	metal	metal
Nd2Ni1O4_ICSD_71137	metal	insulator
Nd2Ni7P4_ICSD_39360	metal	metal
Nd2O13Te2W2_ICSD_249540	insulator	insulator
Nd2O2S1_ICSD_32515	insulator	insulator
Nd2O2Se1_ICSD_25806	insulator	insulator
Nd2O2Te1_ICSD_22250	insulator	insulator
Nd2O3_ICSD_160208	insulator	insulator
Nd2O3_ICSD_160223	insulator	insulator
Nd2O3_ICSD_184534	insulator	insulator
Nd2O3_ICSD_26867	insulator	insulator
Nd2O4_ICSD_62228	insulator	insulator
Nd2O7Pt2_ICSD_402456	insulator	insulator
Nd2O7Ru2_ICSD_161572	insulator	metal
Nd2O7Sn2_ICSD_82958	metal	insulator
Nd2O7Ti2_ICSD_164026	insulator	insulator
Nd2O7Zr2_ICSD_160164	metal	insulator
Nd2O7Zr2_ICSD_62793	insulator	metal
Nd2Re3Si5_ICSD_10085	metal	metal
Nd2Rh1Si3_ICSD_57432	metal	metal
Nd2S2Te1_ICSD_94478	insulator	metal
Nd2S3_ICSD_645834	insulator	insulator
Nd2S5Sn1_ICSD_645866	metal	metal
Nd2Sb1_ICSD_409859	metal	metal
Nd2Sc1Si2_ICSD_85942	metal	metal
Nd2Te3_ICSD_280889	metal	metal
Nd2Tl1_ICSD_105294	metal	metal
Nd2Zn17_ICSD_601787	metal	metal
Nd2Zn17_ICSD_646068	metal	metal
Nd2Zn17_ICSD_646072	metal	metal
Nd3Ni6Si2_ICSD_645644	metal	metal
Nd3O12Sb5_ICSD_67021	insulator	insulator
Nd3O7Re1_ICSD_96411	metal	insulator
Nd3O7Sb1_ICSD_188502	insulator	insulator
Nd3O7Ta1_ICSD_55659	insulator	insulator
Nd3Pt23Si11_ICSD_187876	metal	metal
Nd3Pt4_ICSD_645736	metal	metal
Nd3Pt7Sb4_ICSD_95852	metal	metal
Nd3Rh4Sn13_ICSD_645789	metal	metal
Nd3Ru4Sn13_ICSD_423136	metal	metal
Nd3S4_ICSD_645833	metal	metal
Nd3Se4_ICSD_645910	metal	metal
Nd3Te4_ICSD_646006	metal	metal
Nd3Tl1_ICSD_646054	metal	metal
Nd3Tl5_ICSD_646052	metal	metal

Supplementary Table 533. Five-fold cross validated predictions for the metal/insulator classification (525/598).

system	calculated	predicted
Nd4O19Os6_ICSD_200870	metal	metal
Nd4O4Se3_ICSD_419129	insulator	insulator
Nd4Sb3_ICSD_645890	metal	metal
Nd4Sb3_ICSD_645901	metal	metal
Ne1_ICSD_43427	insulator	insulator
Ne1_ICSD_65884	insulator	insulator
Ne1_ICSD_65885	insulator	insulator
Ne1_ICSD_65886	insulator	insulator
Ne1_ICSD_65887	insulator	insulator
Ne1_ICSD_65888	insulator	insulator
Ne1_ICSD_65889	insulator	insulator
Ne1_ICSD_65890	insulator	insulator
Ne1_ICSD_65891	insulator	insulator
Ne1_ICSD_65892	insulator	insulator
Ne1_ICSD_65893	insulator	insulator
Ne1_ICSD_65894	insulator	insulator
Ne1_ICSD_65895	insulator	insulator
Ne1_ICSD_65896	insulator	insulator
Ne1_ICSD_65897	insulator	insulator
Ne1_ICSD_65898	insulator	insulator
Ne1_ICSD_65899	insulator	insulator
Ne1_ICSD_65900	insulator	insulator
Ne1_ICSD_65901	insulator	insulator
Ne1_ICSD_65902	insulator	insulator
Ne1_ICSD_65903	insulator	insulator
Ne1_ICSD_65904	insulator	insulator
Ni0.33S2Ti1_ICSD_26313	metal	metal
Ni10Si2Tb1_ICSD_97035	metal	metal
Ni10Zr7_ICSD_240191	metal	metal
Ni11Sc3Si4_ICSD_20659	metal	metal
Ni11Zr9_ICSD_105487	metal	metal
Ni12P5_ICSD_108640	metal	metal
Ni12P7Sc2_ICSD_409431	metal	metal
Ni12P7Tb2_ICSD_602494	metal	metal
Ni12P7U2_ICSD_602731	metal	metal
Ni12P7Y2_ICSD_189494	metal	metal
Ni12P7Yb2_ICSD_602514	metal	metal
Ni12P7Zr2_ICSD_646190	metal	metal
Ni16Sc6Si7_ICSD_109117	metal	metal
Ni16Si7Ta6_ICSD_109219	metal	metal
Ni16Si7Ti6_ICSD_109221	metal	metal
Ni16Si7V6_ICSD_109218	metal	metal
Ni16Si7Zr6_ICSD_109222	metal	metal
Ni1O10Te2V2_ICSD_260257	insulator	insulator
Ni1O12P4Zn1_ICSD_37137	insulator	insulator
Ni1O12Re2Sr4_ICSD_16419	insulator	insulator
Ni1O1Zr3_ICSD_71964	metal	metal
Ni1O1_ICSD_76670	insulator	insulator
Ni1O1_ICSD_92128	insulator	insulator
Ni1O2Rb2_ICSD_66277	insulator	insulator

Supplementary Table 534. Five-fold cross validated predictions for the metal/insulator classification (526/598).

system	calculated	predicted
Ni1O2Rb3_ICSD_424579	insulator	insulator
Ni1O2Rb3_ICSD_424580	insulator	insulator
Ni1O2Sr1_ICSD_36123	insulator	metal
Ni1O2_ICSD_78698	insulator	insulator
Ni1O2_ICSD_88720	insulator	insulator
Ni1O3Pb1_ICSD_187685	metal	insulator
Ni1O3Pr1_ICSD_69186	metal	metal
Ni1O3Se1_ICSD_497	insulator	insulator
Ni1O3Ti1_ICSD_15988	insulator	insulator
Ni1O3Yb1_ICSD_151949	metal	metal
Ni1O4Pr2_ICSD_81577	insulator	metal
Ni1O4Rh2_ICSD_23487	metal	metal
Ni1O4S1_ICSD_16741	insulator	insulator
Ni1O4S1_ICSD_33737	insulator	insulator
Ni1O4Sb2_ICSD_86489	insulator	insulator
Ni1O4Se1_ICSD_25700	insulator	insulator
Ni1O4W1_ICSD_15852	insulator	insulator
Ni1O6Os1Sr2_ICSD_152443	metal	metal
Ni1O6Pb1Sr3_ICSD_152588	insulator	insulator
Ni1O6Pt1Sr3_ICSD_75335	insulator	insulator
Ni1O6Re1Sr2_ICSD_173487	insulator	metal
Ni1O6Rh1Sr3_ICSD_51799	insulator	insulator
Ni1O6Ru1Sr2_ICSD_181753	metal	insulator
Ni1O6Sb2_ICSD_80802	insulator	insulator
Ni1O6Sc1Sr3_ICSD_81659	metal	insulator
Ni1O6Sr2Te1_ICSD_16225	insulator	insulator
Ni1O6Sr2Te1_ICSD_91792	insulator	insulator
Ni1O6Sr2W1_ICSD_109165	insulator	insulator
Ni1O6Sr2W1_ICSD_9294	insulator	insulator
Ni1O6Ta2_ICSD_61198	insulator	insulator
Ni1O6U2_ICSD_166251	insulator	insulator
Ni1O6U2_ICSD_28126	insulator	insulator
Ni1O6V2_ICSD_33881	insulator	insulator
Ni1O8P2Sr1Zn1_ICSD_157945	insulator	insulator
Ni1O8Re2_ICSD_51016	insulator	insulator
Ni1O9Se3Sr2_ICSD_419618	insulator	insulator
Ni1P1Pd1_ICSD_646124	metal	metal
Ni1P1Pr1_ICSD_646128	metal	metal
Ni1P1Rh1_ICSD_646131	metal	metal
Ni1P1Rh1_ICSD_646132	metal	metal
Ni1P1S1_ICSD_93898	metal	metal
Ni1P1S3_ICSD_646133	metal	insulator
Ni1P1Sc1_ICSD_50990	metal	metal
Ni1P1Se3_ICSD_646145	metal	insulator
Ni1P1Ta1_ICSD_646155	metal	metal
Ni1P1Ta4_ICSD_646156	metal	metal
Ni1P1Ti1_ICSD_646165	metal	metal
Ni1P1V1_ICSD_646176	metal	metal
Ni1P1W1_ICSD_646181	metal	metal
Ni1P1Y1_ICSD_646184	metal	metal

Supplementary Table 535. Five-fold cross validated predictions for the metal/insulator classification (527/598).

system	calculated	predicted
Ni1P1Zr1_ICSD_76678	metal	metal
Ni1P1Zr2_ICSD_84826	metal	metal
Ni1P1Zr4_ICSD_646191	metal	metal
Ni1P1_ICSD_188064	metal	metal
Ni1P1_ICSD_261827	metal	metal
Ni1P2Ta1_ICSD_646158	metal	metal
Ni1P2W1_ICSD_646178	metal	metal
Ni1P2Zr1_ICSD_646193	metal	metal
Ni1P2Zr2_ICSD_646195	metal	metal
Ni1P2_ICSD_22221	metal	metal
Ni1P2_ICSD_91560	insulator	metal
Ni1P3Si2_ICSD_79376	metal	insulator
Ni1P3W2_ICSD_41818	metal	metal
Ni1P3_ICSD_92394	metal	metal
Ni1P4Si3_ICSD_39452	insulator	metal
Ni1Pb1_ICSD_108642	metal	metal
Ni1Pb3Tb5_ICSD_152603	metal	metal
Ni1Pd1Si1_ICSD_646219	metal	metal
Ni1Pd1Te1_ICSD_419759	metal	metal
Ni1Pd2Te2_ICSD_416512	metal	metal
Ni1Pr1Sb2_ICSD_646263	metal	metal
Ni1Pr1Sb2_ICSD_658210	metal	metal
Ni1Pr1Si2_ICSD_658590	metal	metal
Ni1Pr1Si2_ICSD_71693	metal	metal
Ni1Pr1Si2_ICSD_71694	metal	metal
Ni1Pr1Sn1_ICSD_419192	metal	metal
Ni1Pr1Sn1_ICSD_646288	metal	metal
Ni1Pr1Sn2_ICSD_646289	metal	metal
Ni1Pr1Zn1_ICSD_54295	metal	metal
Ni1Pr1_ICSD_646227	metal	metal
Ni1Pr1_ICSD_646247	metal	metal
Ni1Pr1_ICSD_99823	metal	metal
Ni1Pt1_ICSD_105316	metal	metal
Ni1Pu1_ICSD_150528	metal	metal
Ni1Rh2S4_ICSD_646317	metal	metal
Ni1Rh2Se4_ICSD_15214	metal	metal
Ni1Rh2Sn1_ICSD_105327	metal	metal
Ni1S17U8_ICSD_646400	insulator	insulator
Ni1S1Sb1_ICSD_646369	metal	metal
Ni1S1_ICSD_151603	insulator	insulator
Ni1S1_ICSD_40054	metal	metal
Ni1S1_ICSD_42493	insulator	insulator
Ni1S1_ICSD_654938	metal	metal
Ni1S2_ICSD_166474	insulator	metal
Ni1S2_ICSD_169570	insulator	metal
Ni1S2_ICSD_169571	insulator	metal
Ni1S2_ICSD_169572	metal	metal
Ni1S2_ICSD_169573	metal	metal
Ni1S2_ICSD_169574	metal	metal
Ni1S2_ICSD_169575	metal	insulator

Supplementary Table 536. Five-fold cross validated predictions for the metal/insulator classification (528/598).

system	calculated	predicted
Ni1S2_ICSD_169577	metal	insulator
Ni1S2_ICSD_169578	metal	metal
Ni1S2_ICSD_659263	metal	insulator
Ni1S4V2_ICSD_35140	metal	metal
Ni1S4V2_ICSD_646403	metal	metal
Ni1S5Ta2_ICSD_61147	metal	metal
Ni1S6Ti3_ICSD_26312	metal	metal
Ni1S6V3_ICSD_35142	metal	metal
Ni1S8Ti4_ICSD_646389	metal	metal
Ni1Sb1Sc1_ICSD_40296	metal	metal
Ni1Sb1Se1_ICSD_646435	metal	metal
Ni1Sb1Si1_ICSD_646436	metal	metal
Ni1Sb1Tb1_ICSD_44951	insulator	insulator
Ni1Sb1Ti1_ICSD_182482	metal	metal
Ni1Sb1Ti1_ICSD_646450	metal	metal
Ni1Sb1Tm1_ICSD_105304	metal	metal
Ni1Sb1Tm1_ICSD_44952	metal	metal
Ni1Sb1V1_ICSD_76702	metal	metal
Ni1Sb1Y1_ICSD_105331	insulator	metal
Ni1Sb1Yb1_ICSD_105332	metal	metal
Ni1Sb1Zn1_ICSD_240198	metal	metal
Ni1Sb1Zr1_ICSD_408195	metal	metal
Ni1Sb1_ICSD_646431	metal	metal
Ni1Sb2Tb1_ICSD_658214	metal	metal
Ni1Sb2U1_ICSD_656841	metal	metal
Ni1Sb2_ICSD_646411	metal	metal
Ni1Sb7Zr3_ICSD_162589	metal	metal
Ni1Sc1Si1_ICSD_105337	metal	metal
Ni1Sc1Si1_ICSD_41800	metal	metal
Ni1Sc1Sn1_ICSD_105338	metal	metal
Ni1Sc1_ICSD_105333	metal	metal
Ni1Sc2_ICSD_646475	metal	metal
Ni1Sc3Si3_ICSD_48004	metal	metal
Ni1Sc6Te2_ICSD_90082	metal	metal
Ni1Se17U8_ICSD_601638	metal	metal
Ni1Se1_ICSD_42596	metal	metal
Ni1Se1_ICSD_646507	metal	metal
Ni1Se1_ICSD_646520	metal	metal
Ni1Se2_ICSD_646532	metal	metal
Ni1Se3U1_ICSD_646553	metal	insulator
Ni1Se4Ti2_ICSD_646544	metal	metal
Ni1Se4V2_ICSD_23970	metal	metal
Ni1Se4V2_ICSD_646555	metal	metal
Ni1Se7Ta2_ICSD_41619	metal	metal
Ni1Si1Th1_ICSD_646618	metal	metal
Ni1Si1Ti1_ICSD_165253	metal	metal
Ni1Si1U1_ICSD_50082	metal	metal
Ni1Si1V1_ICSD_646660	metal	metal
Ni1Si1Y1_ICSD_79598	metal	metal
Ni1Si1Zr1_ICSD_646693	metal	metal

Supplementary Table 537. Five-fold cross validated predictions for the metal/insulator classification (529/598).

system	calculated	predicted
Ni1Si1_ICSD_164764	metal	metal
Ni1Si1_ICSD_187621	metal	metal
Ni1Si1_ICSD_187622	metal	metal
Ni1Si1_ICSD_30626	metal	metal
Ni1Si2Tb1_ICSD_658586	metal	metal
Ni1Si2Tb3_ICSD_646606	metal	metal
Ni1Si2Tm1_ICSD_646632	metal	metal
Ni1Si2Yb1_ICSD_646675	metal	metal
Ni1Si2_ICSD_646567	metal	metal
Ni1Si3Sr1_ICSD_174268	metal	metal
Ni1Sn1Tb1_ICSD_54301	metal	metal
Ni1Sn1Th1_ICSD_105367	insulator	metal
Ni1Sn1Th1_ICSD_646771	metal	metal
Ni1Sn1Ti1_ICSD_105368	metal	insulator
Ni1Sn1Ti1_ICSD_174568	insulator	metal
Ni1Sn1U1_ICSD_105372	metal	metal
Ni1Sn1U1_ICSD_646795	metal	metal
Ni1Sn1U1_ICSD_646797	metal	metal
Ni1Sn1Y1_ICSD_105379	metal	metal
Ni1Sn1Yb1_ICSD_54360	metal	metal
Ni1Sn1Zr1_ICSD_105382	insulator	metal
Ni1Sn1Zr1_ICSD_646828	metal	insulator
Ni1Sn3Sr1_ICSD_105366	metal	metal
Ni1Ta1Te5_ICSD_73314	metal	metal
Ni1Ta2_ICSD_105387	metal	metal
Ni1Ta5_ICSD_646845	metal	metal
Ni1Tb1Zn1_ICSD_416821	metal	metal
Ni1Tb1_ICSD_105394	metal	metal
Ni1Tb1_ICSD_105395	metal	metal
Ni1Tb1_ICSD_646875	metal	metal
Ni1Tb3_ICSD_646861	metal	metal
Ni1Te1_ICSD_42557	metal	metal
Ni1Te1_ICSD_76730	metal	metal
Ni1Te2_ICSD_646892	metal	metal
Ni1Te2_ICSD_646897	metal	metal
Ni1Th1_ICSD_105403	metal	metal
Ni1Ti1_ICSD_150943	metal	metal
Ni1Ti1_ICSD_156442	metal	metal
Ni1Ti1_ICSD_157607	metal	metal
Ni1Ti1_ICSD_161458	metal	metal
Ni1Ti1_ICSD_164154	metal	metal
Ni1Ti1_ICSD_166372	metal	metal
Ni1Ti1_ICSD_187467	metal	metal
Ni1Ti1_ICSD_187469	metal	metal
Ni1Ti2_ICSD_604523	metal	metal
Ni1Tl1_ICSD_105427	metal	metal
Ni1U6_ICSD_105435	metal	metal
Ni1V3_ICSD_647028	metal	metal
Ni1Y1_ICSD_105457	metal	metal
Ni1Y1_ICSD_647049	metal	metal

Supplementary Table 538. Five-fold cross validated predictions for the metal/insulator classification (530/598).

system	calculated	predicted
Ni1Y3_ICSD_658309	metal	metal
Ni1Yb1_ICSD_647130	metal	metal
Ni1Yb3_ICSD_659942	metal	metal
Ni1Zn1_ICSD_105469	metal	metal
Ni1Zn1_ICSD_105472	metal	metal
Ni1Zn1_ICSD_647135	metal	metal
Ni1Zr1_ICSD_647164	metal	metal
Ni1Zr2_ICSD_102805	metal	metal
Ni1_ICSD_163354	metal	metal
Ni1_ICSD_183264	metal	metal
Ni1_ICSD_76668	metal	metal
Ni21P6Sn2_ICSD_419774	metal	metal
Ni21Zr8_ICSD_402864	metal	metal
Ni23Zr6_ICSD_600782	metal	metal
Ni2O12P4_ICSD_35730	insulator	insulator
Ni2O1Ti4_ICSD_15809	metal	metal
Ni2O1Zr4_ICSD_74996	metal	metal
Ni2O4Si1_ICSD_40994	insulator	insulator
Ni2O6P2Sc2Sr4_ICSD_180269	metal	metal
Ni2O7P2_ICSD_100194	insulator	insulator
Ni2O7P2_ICSD_30433	insulator	insulator
Ni2O7Sb2_ICSD_247303	metal	insulator
Ni2O7V2_ICSD_2358	insulator	insulator
Ni2O8Te3_ICSD_50703	insulator	insulator
Ni2P1Sn1_ICSD_61097	metal	metal
Ni2P1_ICSD_105306	metal	metal
Ni2P2Pr1_ICSD_646127	metal	metal
Ni2P2S6_ICSD_657314	insulator	metal
Ni2P2Sr1_ICSD_85406	metal	metal
Ni2P2Tb1_ICSD_646159	metal	metal
Ni2P2Th1_ICSD_74785	metal	metal
Ni2P2Th1_ICSD_74786	metal	metal
Ni2P2Tm1_ICSD_646167	metal	metal
Ni2P2U1_ICSD_76674	metal	metal
Ni2P2Y1_ICSD_646183	metal	metal
Ni2P2Yb1_ICSD_646187	metal	metal
Ni2P2Zr1_ICSD_76679	metal	metal
Ni2Pr1Sb2_ICSD_646261	metal	metal
Ni2Pr1Sb2_ICSD_646262	metal	metal
Ni2Pr1Si2_ICSD_169860	metal	metal
Ni2Pr1Sn2_ICSD_646290	metal	metal
Ni2Pr1_ICSD_105310	metal	metal
Ni2Pr1_ICSD_646237	metal	metal
Ni2Pr1_ICSD_646252	metal	metal
Ni2Pr1_ICSD_646256	metal	metal
Ni2Pr2Sn1_ICSD_425429	metal	metal
Ni2Pu1Si2_ICSD_604292	metal	metal
Ni2Pu1_ICSD_105320	metal	metal
Ni2Pu2Sn1_ICSD_150164	metal	metal
Ni2S2Tl1_ICSD_646396	metal	metal

Supplementary Table 539. Five-fold cross validated predictions for the metal/insulator classification (531/598).

system	calculated	predicted
Ni2S6Ta9_ICSD_63591	metal	metal
Ni2Sb1Te2_ICSD_99117	metal	metal
Ni2Sb1Ti1_ICSD_76700	metal	metal
Ni2Sb1Zr1_ICSD_162585	metal	metal
Ni2Sb1Zr1_ICSD_76703	metal	metal
Ni2Sb2Sr1_ICSD_646442	metal	metal
Ni2Sb2Tb1_ICSD_646443	metal	metal
Ni2Sc1Si2_ICSD_646482	metal	metal
Ni2Sc1Sn1_ICSD_105339	metal	metal
Ni2Sc1_ICSD_105335	metal	metal
Ni2Sc2Sn1_ICSD_54348	metal	metal
Ni2Sc2Sn1_ICSD_658917	metal	metal
Ni2Sc3Si3_ICSD_65760	metal	metal
Ni2Se1Ta1Te1_ICSD_86174	metal	metal
Ni2Se2Tl1_ICSD_646550	metal	metal
Ni2Se2Tl1_ICSD_646551	metal	metal
Ni2Se8Ta11_ICSD_40378	metal	metal
Ni2Si1Ta3_ICSD_76717	metal	metal
Ni2Si1V3_ICSD_646652	metal	metal
Ni2Si1Zn3_ICSD_42980	metal	metal
Ni2Si1_ICSD_165257	metal	metal
Ni2Si1_ICSD_24642	metal	metal
Ni2Si2Tb1_ICSD_76718	metal	metal
Ni2Si2Th1_ICSD_18159	metal	metal
Ni2Si2U1_ICSD_25684	metal	metal
Ni2Si2Y1_ICSD_646667	metal	metal
Ni2Si2Yb1_ICSD_646672	metal	metal
Ni2Si2Zr1_ICSD_20317	metal	metal
Ni2Sn1Tb6_ICSD_646766	metal	metal
Ni2Sn1Ti1_ICSD_105370	metal	metal
Ni2Sn1Tm2_ICSD_425432	metal	metal
Ni2Sn1U1_ICSD_646802	metal	metal
Ni2Sn1U2_ICSD_602807	metal	metal
Ni2Sn1V1_ICSD_105376	metal	metal
Ni2Sn1Y2_ICSD_186294	metal	metal
Ni2Sn1Yb1_ICSD_105381	metal	metal
Ni2Sn1Zr1_ICSD_105383	metal	metal
Ni2Sn1Zr2_ICSD_405564	metal	metal
Ni2Sn1Zr2_ICSD_54303	metal	metal
Ni2Sn2Th1_ICSD_602652	metal	metal
Ni2Sn2U1_ICSD_602762	metal	metal
Ni2Ta1Te3_ICSD_82605	metal	metal
Ni2Ta1_ICSD_105388	metal	metal
Ni2Ta2Te4_ICSD_71063	metal	metal
Ni2Tb1_ICSD_154459	metal	metal
Ni2Tb2Zn1_ICSD_416820	metal	metal
Ni2Tb3_ICSD_646858	metal	metal
Ni2Th1_ICSD_646934	metal	metal
Ni2Tm1_ICSD_646992	metal	metal
Ni2Tm1_ICSD_646993	metal	metal

Supplementary Table 540. Five-fold cross validated predictions for the metal/insulator classification (532/598).

system	calculated	predicted
Ni2U1_ICSD_105436	metal	metal
Ni2U1_ICSD_260985	metal	metal
Ni2U1_ICSD_647006	metal	metal
Ni2V1_ICSD_647027	metal	metal
Ni2Y1_ICSD_183094	metal	metal
Ni2Yb1_ICSD_105466	metal	metal
Ni2Zn11_ICSD_647140	metal	metal
Ni2Zr1_ICSD_105481	metal	metal
Ni31Si12_ICSD_9106	metal	metal
Ni3O1Ti3_ICSD_29055	metal	metal
Ni3O6Te1_ICSD_27076	insulator	insulator
Ni3O8P2_ICSD_153159	insulator	insulator
Ni3O8P2_ICSD_158523	insulator	insulator
Ni3O8V2_ICSD_2646	insulator	insulator
Ni3P1_ICSD_98373	metal	metal
Ni3Pb2S2_ICSD_159363	insulator	metal
Ni3Pb2S2_ICSD_417632	metal	metal
Ni3Pb2Se2_ICSD_646211	metal	metal
Ni3Pr1_ICSD_646231	metal	metal
Ni3Pt1_ICSD_151193	metal	metal
Ni3Rb2S4_ICSD_646311	insulator	insulator
Ni3Rb2Se4_ICSD_78976	insulator	insulator
Ni3S2Sn2_ICSD_646380	metal	metal
Ni3S2Tl2_ICSD_108893	metal	metal
Ni3S2_ICSD_10424	metal	metal
Ni3S2_ICSD_180766	metal	metal
Ni3S2_ICSD_180767	metal	metal
Ni3S4_ICSD_57435	metal	metal
Ni3S8Ta2_ICSD_201972	insulator	insulator
Ni3Sb1_ICSD_646421	metal	metal
Ni3Sb1_ICSD_76693	metal	metal
Ni3Sb2Zn1_ICSD_240197	metal	metal
Ni3Sb4U3_ICSD_657094	insulator	metal
Ni3Sb4Zr3_ICSD_87995	insulator	metal
Ni3Sc2Si1_ICSD_646484	metal	metal
Ni3Se2_ICSD_646514	metal	metal
Ni3Se4_ICSD_42558	metal	metal
Ni3Se4_ICSD_76710	metal	metal
Ni3Se8Ta2_ICSD_80881	insulator	insulator
Ni3Si1_ICSD_646584	metal	metal
Ni3Si1_ICSD_646585	metal	metal
Ni3Si5Tb2_ICSD_658648	metal	metal
Ni3Si5Y2_ICSD_84206	metal	metal
Ni3Sn1_ICSD_105353	metal	metal
Ni3Sn1_ICSD_105354	metal	metal
Ni3Sn1_ICSD_646747	metal	metal
Ni3Sn1_ICSD_646751	metal	metal
Ni3Sn2_ICSD_105358	metal	metal
Ni3Sn4Th3_ICSD_657422	metal	metal
Ni3Sn4U3_ICSD_646805	metal	metal

Supplementary Table 541. Five-fold cross validated predictions for the metal/insulator classification (533/598).

system	calculated	predicted
Ni3Sn4_ICSD_646757	metal	metal
Ni3Ta1_ICSD_105390	metal	metal
Ni3Ta1_ICSD_105391	metal	metal
Ni3Ta1_ICSD_54304	metal	metal
Ni3Tb1_ICSD_646874	metal	metal
Ni3Te2_ICSD_99271	metal	metal
Ni3Th7_ICSD_105406	metal	metal
Ni3Ti0.67Zr0.33_ICSD_646978	metal	metal
Ni3Ti1_ICSD_646944	metal	metal
Ni3V1_ICSD_647034	metal	metal
Ni3Y1_ICSD_105461	metal	metal
Ni3Y1_ICSD_647061	metal	metal
Ni3Y1_ICSD_647095	metal	metal
Ni3Zr1_ICSD_647149	metal	metal
Ni4O8Sr1_ICSD_40469	insulator	insulator
Ni4P2Tb1_ICSD_76673	metal	metal
Ni4P2U1_ICSD_85896	metal	metal
Ni4P2Y1_ICSD_601110	metal	metal
Ni4P2Yb1_ICSD_90270	metal	metal
Ni4P2Zr1_ICSD_40435	metal	metal
Ni4P4Ta5_ICSD_646154	metal	metal
Ni4Sc1Sn1_ICSD_105340	metal	metal
Ni4Sc3Si4_ICSD_646480	metal	metal
Ni4Sn13Yb7_ICSD_420766	metal	metal
Ni4Sn1U1_ICSD_105375	metal	metal
Ni4Sn1Zr1_ICSD_105385	metal	metal
Ni4Sn2Sr1_ICSD_418131	metal	metal
Ni4Ti3_ICSD_105422	metal	metal
Ni4U1Zn1_ICSD_105438	metal	metal
Ni4W1_ICSD_105452	metal	metal
Ni4Zn1Zr1_ICSD_105477	metal	metal
Ni5P2_ICSD_646116	metal	metal
Ni5P3Sr1_ICSD_33914	metal	metal
Ni5P4_ICSD_249340	metal	metal
Ni5Pr1_ICSD_105312	metal	metal
Ni5Pr1_ICSD_646226	metal	metal
Ni5Pu1_ICSD_105323	metal	metal
Ni5Sc1_ICSD_105336	metal	metal
Ni5Sc1_ICSD_646468	metal	metal
Ni5Si3U1_ICSD_23037	metal	metal
Ni5Si3Y1_ICSD_23036	metal	metal
Ni5Tb1_ICSD_186005	metal	metal
Ni5Th1_ICSD_105408	metal	metal
Ni5Th1_ICSD_646923	metal	metal
Ni5Th1_ICSD_646926	metal	metal
Ni5Tm1_ICSD_105433	metal	metal
Ni5U1_ICSD_105437	metal	metal
Ni5Y1_ICSD_54422	metal	metal
Ni5Yb1_ICSD_105468	metal	metal
Ni5Yb1_ICSD_647109	metal	metal

Supplementary Table 542. Five-fold cross validated predictions for the metal/insulator classification (534/598).

system	calculated	predicted
Ni5Zr1_ICSD_150648	metal	metal
Ni6P17Pr6_ICSD_646125	metal	metal
Ni6Pb1Y12_ICSD_54614	metal	metal
Ni6Pr3Si2_ICSD_646283	metal	metal
Ni6Si2Tb3_ICSD_646615	metal	metal
Ni6Si2Yb3_ICSD_646677	metal	metal
Ni7Sc2_ICSD_646467	metal	metal
Ni7Tb2_ICSD_646862	metal	metal
Ni7Th2_ICSD_646927	metal	metal
Ni7Y2_ICSD_108650	metal	metal
Ni7Y2_ICSD_647066	metal	metal
Ni9P5Sr1_ICSD_67898	metal	metal
O1.03Sc1_ICSD_61627	metal	metal
O10P2Re2_ICSD_420988	metal	insulator
O10P2Sn5_ICSD_418458	insulator	insulator
O10P2Sr1V2_ICSD_280073	insulator	insulator
O10P2U2_ICSD_75358	insulator	insulator
O10Ru3Sr2_ICSD_50707	metal	insulator
O10Sb2Tl10_ICSD_4124	insulator	insulator
O10U1V3_ICSD_73610	metal	insulator
O11P2W2_ICSD_24072	insulator	metal
O11Pb1Pt4Sr4_ICSD_249144	insulator	insulator
O11Pr2Te4_ICSD_413655	insulator	insulator
O11Re2V2_ICSD_23423	metal	insulator
O11Sr1Ta4_ICSD_79704	insulator	insulator
O11Sr3Te4_ICSD_56798	insulator	insulator
O11Te4Yb2_ICSD_413663	metal	insulator
O11Ti6_ICSD_90958	metal	insulator
O12P4Tl4_ICSD_2139	insulator	insulator
O12P4U1_ICSD_20736	insulator	insulator
O12Pr3Sb5_ICSD_67020	metal	insulator
O12Pr7_ICSD_655511	metal	insulator
O12Re1Sc6_ICSD_245004	insulator	insulator
O12S1Si3Yb5_ICSD_391123	metal	insulator
O12S3Sc2_ICSD_411221	insulator	insulator
O12Sb5Yb3_ICSD_20945	metal	insulator
O12Se4_ICSD_18180	insulator	insulator
O12Si4Sr4_ICSD_32678	insulator	insulator
O12Tb7_ICSD_73822	metal	insulator
O12Te1Tl6_ICSD_37134	metal	insulator
O12Te1Y6_ICSD_247820	insulator	insulator
O12U1Y6_ICSD_23966	insulator	insulator
O12W1Y6_ICSD_100196	insulator	insulator
O13P4Y2_ICSD_95880	insulator	insulator
O13Rb2Ti6_ICSD_23878	insulator	insulator
O13Sc2Te5_ICSD_417712	insulator	insulator
O13Sr2Ti6_ICSD_10455	insulator	insulator
O13Te5Yb2_ICSD_413668	metal	insulator
O13Te6Zn1_ICSD_249328	insulator	insulator
O13V6_ICSD_50409	metal	insulator

Supplementary Table 543. Five-fold cross validated predictions for the metal/insulator classification (535/598).

system	calculated	predicted
O13V6_ICSD_62117	metal	metal
O14P4Pb1V2_ICSD_78511	insulator	insulator
O14P4Rb2Zn3_ICSD_30965	insulator	insulator
O14P4Sb2_ICSD_202685	insulator	insulator
O14P4Sr1V2_ICSD_86433	metal	insulator
O14Pt1Si2Sr8_ICSD_63003	insulator	insulator
O14Rb2Te2U3_ICSD_414063	insulator	insulator
O14Rb3V5_ICSD_420851	insulator	insulator
O14Ru3Sn15_ICSD_401827	insulator	insulator
O14Si4Sr2Ti2_ICSD_83362	insulator	insulator
O14Tl3V5_ICSD_248229	insulator	insulator
O15Rh5Sr6_ICSD_93117	insulator	metal
O15S2Sb6_ICSD_341	insulator	insulator
O18Ti10_ICSD_31399	insulator	insulator
O1Os1P1Pr1_ICSD_419464	metal	metal
O1P1Pr1Zn1_ICSD_416476	insulator	insulator
O1P1Y1Zn1_ICSD_418523	insulator	insulator
O1P2Sr4_ICSD_33903	insulator	insulator
O1Pa1_ICSD_647256	metal	metal
O1Pb1Sr3_ICSD_100790	metal	metal
O1Pb1Yb3_ICSD_413389	metal	metal
O1Pb1_ICSD_15402	insulator	insulator
O1Pb1_ICSD_36250	insulator	insulator
O1Pb1_ICSD_62846	insulator	insulator
O1Pb2_ICSD_28838	metal	metal
O1Pd1_ICSD_185482	insulator	metal
O1Pd1_ICSD_28837	metal	metal
O1Pd1_ICSD_41617	metal	metal
O1Pd1_ICSD_77650	metal	metal
O1Pd2Zr4_ICSD_97386	metal	metal
O1Pd2_ICSD_77651	metal	metal
O1Pr1Sb1Zn1_ICSD_182895	metal	insulator
O1Pr1Sb1Zn1_ICSD_380342	insulator	metal
O1Pr1_ICSD_77652	metal	metal
O1Pt1_ICSD_105543	metal	metal
O1Pt1_ICSD_164290	insulator	metal
O1Pu1_ICSD_647327	metal	metal
O1Rb2_ICSD_77676	insulator	insulator
O1Rb6_ICSD_29336	metal	metal
O1S1U1_ICSD_421031	insulator	metal
O1S1Zr1_ICSD_31721	insulator	insulator
O1S1Zr1_ICSD_36111	insulator	insulator
O1S2Sb2_ICSD_189831	insulator	insulator
O1S2Tb2_ICSD_167087	insulator	insulator
O1S2Y2_ICSD_67503	insulator	insulator
O1S2Yb2_ICSD_67381	metal	metal
O1S8_ICSD_15998	insulator	insulator
O1Se1Th1_ICSD_26654	insulator	insulator
O1Se1U1_ICSD_73408	metal	insulator
O1Si1Zr1_ICSD_25731	metal	metal

Supplementary Table 544. Five-fold cross validated predictions for the metal/insulator classification (536/598).

system	calculated	predicted
O1Sn1Sr3_ICSD_201036	metal	metal
O1Sn1Yb3_ICSD_413388	metal	metal
O1Sn1_ICSD_15516	insulator	insulator
O1Sn1_ICSD_16481	insulator	insulator
O1Sn1_ICSD_20624	insulator	insulator
O1Sn1_ICSD_424729	insulator	insulator
O1Sn1_ICSD_60619	insulator	insulator
O1Sr1_ICSD_181275	insulator	insulator
O1Sr1_ICSD_26960	insulator	insulator
O1Ta1_ICSD_647482	metal	metal
O1Ta2_ICSD_28387	metal	metal
O1Ta4_ICSD_76022	metal	metal
O1Te1Th1_ICSD_65950	insulator	insulator
O1Ti1_ICSD_647558	metal	metal
O1Ti2Zr1_ICSD_9389	metal	metal
O1Ti2_ICSD_23574	metal	metal
O1Ti3_ICSD_20041	metal	metal
O1Ti3_ICSD_23575	metal	metal
O1Ti3_ICSD_36055	metal	metal
O1Ti6_ICSD_17009	metal	metal
O1Ti6_ICSD_23576	metal	metal
O1Tl2_ICSD_16220	insulator	metal
O1Tl2_ICSD_77699	insulator	insulator
O1U1_ICSD_24223	metal	metal
O1V1_ICSD_28681	insulator	metal
O1V1_ICSD_77705	metal	insulator
O1V3Zr3_ICSD_38407	metal	metal
O1V8_ICSD_166600	metal	metal
O1W3_ICSD_150496	metal	metal
O1Yb1_ICSD_77710	insulator	metal
O1Zn1_ICSD_163382	metal	metal
O1Zn1_ICSD_166357	insulator	insulator
O1Zn1_ICSD_180052	insulator	insulator
O1Zn1_ICSD_181039	insulator	insulator
O1Zn1_ICSD_182357	insulator	insulator
O1Zr1_ICSD_76019	metal	metal
O1Zr2_ICSD_77714	metal	metal
O1Zr3_ICSD_23402	metal	metal
O1Zr3_ICSD_27023	metal	metal
O1Zr3_ICSD_88320	metal	metal
O1_ICSD_92775	insulator	insulator
O23Rb6Si10_ICSD_250379	insulator	insulator
O23Rb6U5V2_ICSD_96479	insulator	insulator
O24P6S1Sr10_ICSD_410783	insulator	insulator
O25P6Si5_ICSD_6197	insulator	insulator
O26Si4Sr3Ta6_ICSD_15936	insulator	insulator
O2Os1_ICSD_30400	metal	metal
O2P1Ti3_ICSD_76020	metal	metal
O2P1Zr3_ICSD_76021	metal	metal
O2Pa1_ICSD_647247	metal	metal

Supplementary Table 545. Five-fold cross validated predictions for the metal/insulator classification (537/598).

system	calculated	predicted
O2Pb1Pd1_ICSD_2277	insulator	metal
O2Pb1_ICSD_23292	insulator	metal
O2Pb1_ICSD_415269	metal	insulator
O2Pb1_ICSD_77648	metal	insulator
O2Pd1_ICSD_647283	metal	insulator
O2Pr1_ICSD_105541	metal	insulator
O2Pr1_ICSD_380398	insulator	insulator
O2Pr2S1_ICSD_109329	insulator	insulator
O2Pr2Se1_ICSD_94415	insulator	insulator
O2Pr2Te1_ICSD_89559	metal	insulator
O2Pt1_ICSD_24923	insulator	insulator
O2Pt1_ICSD_647316	metal	insulator
O2Pt1_ICSD_647320	insulator	insulator
O2Pt1_ICSD_76431	insulator	insulator
O2Pt1_ICSD_77654	metal	metal
O2Pu1_ICSD_186183	metal	metal
O2Pu2S1_ICSD_647333	metal	metal
O2Rb1Sb1_ICSD_411216	insulator	insulator
O2Rb1Sc1_ICSD_1270	insulator	insulator
O2Rb1Sc1_ICSD_31959	insulator	insulator
O2Rb1Tl1_ICSD_33554	insulator	insulator
O2Rb1Tm1_ICSD_15162	metal	insulator
O2Rb1Y1_ICSD_49651	insulator	insulator
O2Rb1Yb1_ICSD_15163	insulator	insulator
O2Rb1_ICSD_180564	metal	metal
O2Rb2Sn1_ICSD_24805	insulator	insulator
O2Rb2Zn1_ICSD_1119	insulator	insulator
O2Rb2_ICSD_180560	insulator	insulator
O2Rb9_ICSD_1239	metal	metal
O2Re1_ICSD_154021	metal	metal
O2Re1_ICSD_24060	metal	metal
O2Re1_ICSD_647349	metal	metal
O2Rh1_ICSD_647367	metal	metal
O2Ru1_ICSD_290498	insulator	metal
O2Ru1_ICSD_66939	metal	metal
O2Ru1_ICSD_84618	metal	metal
O2Ru1_ICSD_84619	metal	metal
O2S1Sc2_ICSD_2450	insulator	insulator
O2S1Tb2_ICSD_109331	insulator	insulator
O2S1Tm2_ICSD_400048	insulator	insulator
O2S1Y2_ICSD_154582	insulator	insulator
O2S1Yb2_ICSD_109335	metal	metal
O2S1Yb2_ICSD_23583	metal	metal
O2S1_ICSD_24645	insulator	insulator
O2Sb1_ICSD_647389	insulator	insulator
O2Se1Yb2_ICSD_25811	metal	metal
O2Se1_ICSD_412234	insulator	insulator
O2Se1_ICSD_72367	insulator	insulator
O2Se1_ICSD_99464	insulator	insulator
O2Se1_ICSD_99465	insulator	insulator

Supplementary Table 546. Five-fold cross validated predictions for the metal/insulator classification (538/598).

system	calculated	predicted
O2Si1_ICSD_154321	insulator	insulator
O2Si1_ICSD_158532	insulator	insulator
O2Si1_ICSD_161310	insulator	insulator
O2Si1_ICSD_162616	insulator	insulator
O2Si1_ICSD_162621	insulator	insulator
O2Si1_ICSD_162625	insulator	insulator
O2Si1_ICSD_162626	insulator	insulator
O2Si1_ICSD_162660	insulator	insulator
O2Si1_ICSD_170476	insulator	insulator
O2Si1_ICSD_170481	insulator	insulator
O2Si1_ICSD_170486	insulator	insulator
O2Si1_ICSD_170489	insulator	insulator
O2Si1_ICSD_170490	insulator	insulator
O2Si1_ICSD_170492	insulator	insulator
O2Si1_ICSD_170496	insulator	insulator
O2Si1_ICSD_170497	insulator	insulator
O2Si1_ICSD_170498	insulator	insulator
O2Si1_ICSD_170500	insulator	insulator
O2Si1_ICSD_170501	insulator	metal
O2Si1_ICSD_170502	insulator	insulator
O2Si1_ICSD_170503	insulator	insulator
O2Si1_ICSD_170504	metal	insulator
O2Si1_ICSD_170506	metal	insulator
O2Si1_ICSD_170507	insulator	insulator
O2Si1_ICSD_170508	insulator	insulator
O2Si1_ICSD_170510	insulator	insulator
O2Si1_ICSD_170512	insulator	insulator
O2Si1_ICSD_170513	insulator	insulator
O2Si1_ICSD_170514	insulator	insulator
O2Si1_ICSD_170515	insulator	insulator
O2Si1_ICSD_170518	insulator	insulator
O2Si1_ICSD_170523	insulator	insulator
O2Si1_ICSD_170524	insulator	insulator
O2Si1_ICSD_170527	insulator	insulator
O2Si1_ICSD_170530	insulator	insulator
O2Si1_ICSD_170531	metal	insulator
O2Si1_ICSD_170532	insulator	insulator
O2Si1_ICSD_170533	insulator	insulator
O2Si1_ICSD_170534	insulator	insulator
O2Si1_ICSD_170535	insulator	insulator
O2Si1_ICSD_170536	insulator	insulator
O2Si1_ICSD_170537	insulator	insulator
O2Si1_ICSD_170538	metal	insulator
O2Si1_ICSD_170539	insulator	insulator
O2Si1_ICSD_170542	insulator	insulator
O2Si1_ICSD_170543	insulator	insulator
O2Si1_ICSD_170545	insulator	insulator
O2Si1_ICSD_170546	insulator	insulator
O2Si1_ICSD_170547	metal	insulator
O2Si1_ICSD_170548	insulator	insulator

Supplementary Table 547. Five-fold cross validated predictions for the metal/insulator classification (539/598).

system	calculated	predicted
O2Si1_ICSD_170549	insulator	insulator
O2Si1_ICSD_170550	insulator	insulator
O2Si1_ICSD_170551	insulator	insulator
O2Si1_ICSD_170552	metal	insulator
O2Si1_ICSD_170553	insulator	insulator
O2Si1_ICSD_170554	insulator	insulator
O2Si1_ICSD_171738	insulator	insulator
O2Si1_ICSD_171739	insulator	insulator
O2Si1_ICSD_172288	insulator	insulator
O2Si1_ICSD_181307	insulator	insulator
O2Si1_ICSD_183701	insulator	insulator
O2Si1_ICSD_200478	insulator	insulator
O2Si1_ICSD_25632	metal	insulator
O2Si1_ICSD_29343	insulator	insulator
O2Si1_ICSD_34889	insulator	insulator
O2Si1_ICSD_41447	insulator	insulator
O2Si1_ICSD_44271	insulator	insulator
O2Si1_ICSD_52371	insulator	insulator
O2Si1_ICSD_56473	insulator	insulator
O2Si1_ICSD_56608	insulator	insulator
O2Si1_ICSD_62581	insulator	insulator
O2Si1_ICSD_75300	insulator	insulator
O2Si1_ICSD_75647	insulator	insulator
O2Si1_ICSD_75649	insulator	insulator
O2Si1_ICSD_75651	insulator	insulator
O2Si1_ICSD_75652	insulator	insulator
O2Si1_ICSD_75654	insulator	insulator
O2Si1_ICSD_75659	insulator	insulator
O2Si1_ICSD_75662	insulator	insulator
O2Si1_ICSD_75664	insulator	insulator
O2Si1_ICSD_75665	insulator	insulator
O2Si1_ICSD_75668	insulator	insulator
O2Si1_ICSD_75669	insulator	insulator
O2Si1_ICSD_77458	insulator	insulator
O2Si1_ICSD_85586	insulator	insulator
O2Si1_ICSD_89289	insulator	insulator
O2Si1_ICSD_89661	insulator	insulator
O2Si1_ICSD_91736	insulator	insulator
O2Si1_ICSD_93975	insulator	insulator
O2Sn1_ICSD_157450	insulator	metal
O2Sn1_ICSD_157451	metal	insulator
O2Sn1_ICSD_157452	insulator	insulator
O2Sn1_ICSD_157453	metal	metal
O2Sn1_ICSD_181282	metal	insulator
O2Sn1_ICSD_39178	metal	insulator
O2Sn1_ICSD_56675	insulator	metal
O2Sn1_ICSD_62199	insulator	insulator
O2Sr1_ICSD_647474	insulator	insulator
O2Ta1_ICSD_76024	metal	metal
O2Tb1_ICSD_28846	metal	insulator

Supplementary Table 548. Five-fold cross validated predictions for the metal/insulator classification (540/598).

system	calculated	predicted
O2Tb2Te1_ICSD_89564	insulator	insulator
O2Tc1_ICSD_173152	metal	insulator
O2Te1U2_ICSD_108986	insulator	metal
O2Te1_ICSD_166846	insulator	insulator
O2Te1_ICSD_166847	insulator	insulator
O2Te1_ICSD_166848	insulator	insulator
O2Te1_ICSD_166849	insulator	insulator
O2Te1_ICSD_27515	insulator	insulator
O2Te1_ICSD_30222	insulator	insulator
O2Te1_ICSD_34423	insulator	insulator
O2Te1_ICSD_56004	metal	insulator
O2Te1_ICSD_655884	insulator	insulator
O2Th1_ICSD_246705	insulator	insulator
O2Th1_ICSD_647523	insulator	insulator
O2Ti1_ICSD_15328	insulator	insulator
O2Ti1_ICSD_154036	insulator	insulator
O2Ti1_ICSD_154609	insulator	insulator
O2Ti1_ICSD_167957	insulator	insulator
O2Ti1_ICSD_189323	insulator	insulator
O2Ti1_ICSD_189324	insulator	insulator
O2Ti1_ICSD_189325	insulator	insulator
O2Ti1_ICSD_36410	insulator	insulator
O2Ti1_ICSD_41056	insulator	insulator
O2Ti1_ICSD_41493	insulator	insulator
O2Ti1_ICSD_657748	insulator	insulator
O2Ti1_ICSD_93098	insulator	insulator
O2Ti1_ICSD_97008	insulator	insulator
O2U1_ICSD_160813	metal	metal
O2U1_ICSD_160815	metal	metal
O2U1_ICSD_647590	metal	metal
O2V1_ICSD_1503	insulator	insulator
O2V1_ICSD_34033	insulator	insulator
O2V1_ICSD_34416	metal	insulator
O2V1_ICSD_34419	insulator	insulator
O2V1_ICSD_51213	metal	insulator
O2V1_ICSD_51214	insulator	insulator
O2V1_ICSD_57155	insulator	metal
O2V1_ICSD_66665	insulator	insulator
O2V1_ICSD_77655	insulator	insulator
O2V1_ICSD_89470	insulator	insulator
O2W1_ICSD_647643	metal	metal
O2W1_ICSD_647647	metal	metal
O2W1_ICSD_74774	insulator	metal
O2Zn1_ICSD_647668	insulator	insulator
O2Zr1_ICSD_164736	insulator	insulator
O2Zr1_ICSD_164861	insulator	insulator
O2Zr1_ICSD_173960	insulator	insulator
O2Zr1_ICSD_56696	insulator	insulator
O2Zr1_ICSD_67004	insulator	insulator
O2Zr1_ICSD_80047	insulator	insulator

Supplementary Table 549. Five-fold cross validated predictions for the metal/insulator classification (541/598).

system	calculated	predicted
O2Zr1_ICSD_92091	metal	insulator
O2_ICSD_164724	metal	insulator
O2_ICSD_173932	insulator	metal
O2_ICSD_173934	insulator	insulator
O2_ICSD_37059	metal	metal
O2_ICSD_43074	metal	insulator
O32P4W8_ICSD_32506	metal	insulator
O36P12Sc4_ICSD_1719	insulator	insulator
O38P4W10_ICSD_35262	metal	metal
O3P4S6_ICSD_86494	insulator	insulator
O3Pb1Rb2_ICSD_1413	insulator	insulator
O3Pb1S1_ICSD_30993	insulator	insulator
O3Pb1S1_ICSD_35360	insulator	insulator
O3Pb1Se1_ICSD_22371	insulator	insulator
O3Pb1Se1_ICSD_94763	insulator	insulator
O3Pb1Sr1_ICSD_4121	insulator	insulator
O3Pb1Tc1_ICSD_109078	metal	metal
O3Pb1Te1_ICSD_61343	insulator	insulator
O3Pb1Ti1_ICSD_182930	insulator	insulator
O3Pb1Ti1_ICSD_27949	insulator	insulator
O3Pb1Ti1_ICSD_51840	insulator	insulator
O3Pb1V1_ICSD_152277	insulator	insulator
O3Pb1V1_ICSD_187637	metal	metal
O3Pb1Zr1_ICSD_262105	insulator	insulator
O3Pb1Zr1_ICSD_51574	insulator	insulator
O3Pb2_ICSD_23760	insulator	insulator
O3Pb2_ICSD_36243	insulator	insulator
O3Pd1Se1_ICSD_249504	insulator	insulator
O3Pd1Se1_ICSD_415955	insulator	insulator
O3Pd1Sr2_ICSD_95214	insulator	metal
O3Pm2_ICSD_160224	insulator	insulator
O3Pm2_ICSD_647284	metal	insulator
O3Pm2_ICSD_96200	metal	insulator
O3Pr1Rh1_ICSD_172348	insulator	insulator
O3Pr1Ru1_ICSD_75570	metal	metal
O3Pr1Sc1_ICSD_710019	insulator	insulator
O3Pr1V1_ICSD_28927	metal	metal
O3Pr2_ICSD_154588	insulator	insulator
O3Pr2_ICSD_160222	insulator	insulator
O3Pr2_ICSD_184533	insulator	insulator
O3Pr2_ICSD_600743	insulator	insulator
O3Pu2_ICSD_109363	metal	metal
O3Pu2_ICSD_647332	metal	metal
O3Rb0.333W1_ICSD_96419	metal	metal
O3Rb0.333W1_ICSD_96420	metal	metal
O3Rb0.333W1_ICSD_96421	metal	metal
O3Rb1Re1S1_ICSD_15088	insulator	insulator
O3Rb1Ta1_ICSD_1633	insulator	insulator
O3Rb1U1_ICSD_99548	insulator	insulator
O3Rb1V1_ICSD_1488	insulator	insulator

Supplementary Table 550. Five-fold cross validated predictions for the metal/insulator classification (542/598).

system	calculated	predicted
O3Rb1_ICSD_59100	insulator	insulator
O3Rb2Sn2_ICSD_24816	insulator	insulator
O3Rb2Tb1_ICSD_1182	metal	insulator
O3Rb2Te1_ICSD_38223	insulator	insulator
O3Rb2Ti1_ICSD_6101	insulator	insulator
O3Re1_ICSD_202338	metal	metal
O3Re1_ICSD_55465	metal	metal
O3Re1_ICSD_647352	metal	metal
O3Re1_ICSD_77680	metal	metal
O3Rh1Sr1_ICSD_187483	metal	metal
O3Rh1Tb1_ICSD_172352	insulator	insulator
O3Rh2_ICSD_181832	metal	insulator
O3Rh2_ICSD_647369	insulator	metal
O3Rh2_ICSD_9206	insulator	metal
O3Ru1Sr1_ICSD_180907	metal	metal
O3Ru1Sr1_ICSD_180910	metal	metal
O3Ru1Sr1_ICSD_50344	metal	metal
O3Ru1Sr1_ICSD_82983	metal	metal
O3S1_ICSD_24723	insulator	insulator
O3Sb1Tl1_ICSD_10142	insulator	insulator
O3Sb1Tl1_ICSD_4123	insulator	insulator
O3Sb1Tl1_ICSD_77329	insulator	insulator
O3Sb2_ICSD_105547	insulator	insulator
O3Sb2_ICSD_27595	insulator	insulator
O3Sc2_ICSD_160203	insulator	insulator
O3Sc2_ICSD_160218	insulator	insulator
O3Sc2_ICSD_27948	insulator	insulator
O3Se1Sr1_ICSD_240888	insulator	insulator
O3Se1Sr1_ICSD_419386	insulator	insulator
O3Se1Zn1_ICSD_163221	insulator	insulator
O3Se1Zn1_ICSD_29500	insulator	insulator
O3Si1Sr1_ICSD_59308	insulator	insulator
O3Si1Zn1_ICSD_158516	insulator	insulator
O3Si1Zn1_ICSD_158517	insulator	insulator
O3Si1Zn1_ICSD_167186	insulator	insulator
O3Sn1Sr1_ICSD_153532	insulator	insulator
O3Sn1Sr1_ICSD_161783	insulator	insulator
O3Sn1Sr1_ICSD_27047	insulator	insulator
O3Sn1Ta1_ICSD_27118	metal	insulator
O3Sn1Ti1_ICSD_186724	insulator	insulator
O3Sn1Tl2_ICSD_6325	metal	insulator
O3Sn1Zn1_ICSD_245943	insulator	insulator
O3Sn1Zn1_ICSD_50404	insulator	insulator
O3Sr1Tb1_ICSD_86734	metal	insulator
O3Sr1Tc1_ICSD_109076	metal	metal
O3Sr1Tc1_ICSD_183451	insulator	insulator
O3Sr1Tc1_ICSD_183452	insulator	insulator
O3Sr1Ti1_ICSD_182247	insulator	insulator
O3Sr1Ti1_ICSD_80874	insulator	insulator
O3Sr1V1_ICSD_88982	metal	metal

Supplementary Table 551. Five-fold cross validated predictions for the metal/insulator classification (543/598).

system	calculated	predicted
O3Sr1Zr1_ICSD_188453	insulator	insulator
O3Sr1Zr1_ICSD_290618	insulator	insulator
O3Sr1Zr1_ICSD_33666	metal	metal
O3Sr1Zr1_ICSD_41083	insulator	insulator
O3Sr1Zr1_ICSD_89361	insulator	insulator
O3Ta2_ICSD_263120	metal	metal
O3Tb2_ICSD_160212	insulator	insulator
O3Tb2_ICSD_184537	insulator	insulator
O3Tb2_ICSD_28172	insulator	insulator
O3Te1Zn1_ICSD_16937	insulator	insulator
O3Te1_ICSD_68372	insulator	insulator
O3Ti1Zn1_ICSD_22382	insulator	insulator
O3Ti2_ICSD_77696	insulator	insulator
O3Tl1V1_ICSD_6108	insulator	insulator
O3Tl2_ICSD_26813	metal	insulator
O3Tl4_ICSD_23478	insulator	insulator
O3Tm2_ICSD_160216	insulator	insulator
O3U1_ICSD_14366	insulator	insulator
O3U1_ICSD_26673	insulator	metal
O3U1_ICSD_31628	insulator	insulator
O3V1Y1_ICSD_95578	metal	insulator
O3V2_ICSD_260212	metal	metal
O3V2_ICSD_647629	metal	metal
O3W1_ICSD_108651	insulator	metal
O3W1_ICSD_188393	insulator	insulator
O3W1_ICSD_188394	insulator	insulator
O3W1_ICSD_32001	insulator	insulator
O3W1_ICSD_50732	insulator	insulator
O3W1_ICSD_654048	insulator	insulator
O3W1_ICSD_84848	insulator	insulator
O3W1_ICSD_86144	insulator	insulator
O3W1_ICSD_88366	insulator	insulator
O3W1_ICSD_89092	insulator	insulator
O3Xe1_ICSD_26627	insulator	insulator
O3Y2_ICSD_160219	insulator	insulator
O3Y2_ICSD_181827	insulator	insulator
O3Y2_ICSD_66730	insulator	insulator
O3Yb2_ICSD_420428	insulator	metal
O3_ICSD_51500	insulator	insulator
O4Os1_ICSD_24672	insulator	insulator
O4Os1_ICSD_63	insulator	insulator
O4P1Pr1_ICSD_184551	insulator	insulator
O4P1Pr1_ICSD_62161	insulator	insulator
O4P1Rb1Zn1_ICSD_71778	insulator	insulator
O4P1Rb3_ICSD_161677	insulator	insulator
O4P1Sb1_ICSD_62977	insulator	insulator
O4P1Sc1_ICSD_16648	insulator	insulator
O4P1Tb1_ICSD_168752	insulator	insulator
O4P1Tb1_ICSD_168754	insulator	insulator
O4P1Tb1_ICSD_168755	insulator	insulator

Supplementary Table 552. Five-fold cross validated predictions for the metal/insulator classification (544/598).

system	calculated	predicted
O4P1Tb1_ICSD_168756	insulator	insulator
O4P1Tb1_ICSD_184561	insulator	insulator
O4P1Tb1_ICSD_79755	insulator	insulator
O4P1Ti1_ICSD_82282	insulator	insulator
O4P1Tl1Zn1_ICSD_74811	insulator	insulator
O4P1Tl1_ICSD_16619	insulator	insulator
O4P1Tl3_ICSD_23474	insulator	insulator
O4P1Tm1_ICSD_184548	insulator	insulator
O4P1V1_ICSD_36521	insulator	insulator
O4P1Y1_ICSD_79754	insulator	insulator
O4P1Yb1_ICSD_36054	metal	insulator
O4Pb1Pt2_ICSD_59657	metal	insulator
O4Pb1S1_ICSD_154273	insulator	insulator
O4Pb1Se1_ICSD_40921	insulator	insulator
O4Pb1Si1Zn1_ICSD_26840	insulator	insulator
O4Pb1Sr2_ICSD_16806	insulator	insulator
O4Pb1U1_ICSD_61344	insulator	insulator
O4Pb1W1_ICSD_155520	insulator	insulator
O4Pb1W1_ICSD_155522	insulator	insulator
O4Pb1W1_ICSD_16189	insulator	insulator
O4Pb1W1_ICSD_164726	insulator	insulator
O4Pb1W1_ICSD_811	metal	insulator
O4Pb2Pt1_ICSD_202214	insulator	insulator
O4Pb2Sn1_ICSD_31482	insulator	insulator
O4Pb3_ICSD_22325	insulator	insulator
O4Pb3_ICSD_647269	insulator	insulator
O4Pb3_ICSD_97282	insulator	insulator
O4Pd1S1_ICSD_79559	insulator	insulator
O4Pd1Se1_ICSD_416646	insulator	insulator
O4Pd1Zn2_ICSD_30076	metal	insulator
O4Pd2Pr1_ICSD_78843	metal	metal
O4Pd2Y1_ICSD_78846	metal	insulator
O4Pd3Sr1_ICSD_16537	metal	metal
O4Pd3Tl1_ICSD_2275	insulator	metal
O4Pr1Sb1_ICSD_245046	insulator	insulator
O4Pr1Sr2_ICSD_90366	insulator	insulator
O4Pr1Ta1_ICSD_415428	insulator	insulator
O4Pr1V1_ICSD_78076	insulator	insulator
O4Pr2Si1Te1_ICSD_89579	insulator	insulator
O4Pt3_ICSD_27836	metal	metal
O4Pt3_ICSD_30444	metal	metal
O4Pu1Sr1_ICSD_31974	metal	insulator
O4Rb1Re1_ICSD_73297	insulator	insulator
O4Rb1Tc1_ICSD_423223	insulator	insulator
O4Rb2Ru1_ICSD_415748	insulator	insulator
O4Rb2U1_ICSD_20582	insulator	insulator
O4Rb2W1_ICSD_24905	insulator	insulator
O4Rb2Zn3_ICSD_40754	insulator	insulator
O4Rb4Sn1_ICSD_280293	insulator	insulator
O4Rh2Zn1_ICSD_647372	insulator	insulator

Supplementary Table 553. Five-fold cross validated predictions for the metal/insulator classification (545/598).

system	calculated	predicted
O4Ru1Sr2_ICSD_75152	metal	metal
O4Ru1_ICSD_415303	insulator	insulator
O4Ru1_ICSD_415306	insulator	insulator
O4S1Sn1_ICSD_245904	insulator	insulator
O4S1Sn1_ICSD_245905	insulator	insulator
O4S1Sn1_ICSD_245907	insulator	insulator
O4S1Sr1_ICSD_23744	insulator	insulator
O4S1Sr1_ICSD_85808	insulator	insulator
O4S1Tl2_ICSD_59944	insulator	insulator
O4S1Zn1_ICSD_2456	insulator	insulator
O4S1Zn1_ICSD_71018	insulator	insulator
O4Sb1Ta1_ICSD_25548	insulator	insulator
O4Sb1V1_ICSD_84567	insulator	insulator
O4Sb2Zn1_ICSD_41222	insulator	insulator
O4Sb2_ICSD_153154	insulator	insulator
O4Sb2_ICSD_24244	insulator	metal
O4Sb2_ICSD_31103	metal	insulator
O4Sb2_ICSD_63272	insulator	insulator
O4Sc1V1_ICSD_164837	insulator	insulator
O4Se1Sr1_ICSD_40923	metal	insulator
O4Se1Sr1_ICSD_47004	insulator	insulator
O4Se1Tl2_ICSD_73411	insulator	insulator
O4Se1Tl2_ICSD_99384	insulator	insulator
O4Se1V1_ICSD_68333	insulator	insulator
O4Si1Sr2_ICSD_36041	insulator	insulator
O4Si1Th1_ICSD_15485	insulator	insulator
O4Si1Ti1_ICSD_166436	insulator	insulator
O4Si1Ti1_ICSD_166437	insulator	insulator
O4Si1Ti1_ICSD_166438	insulator	insulator
O4Si1U1_ICSD_15484	insulator	insulator
O4Si1Zn2_ICSD_167188	insulator	insulator
O4Si1Zn2_ICSD_167189	insulator	insulator
O4Si1Zn2_ICSD_167191	insulator	insulator
O4Si1Zn2_ICSD_167192	insulator	insulator
O4Si1Zn2_ICSD_167193	insulator	insulator
O4Si1Zn2_ICSD_187838	insulator	insulator
O4Si1Zn2_ICSD_2425	insulator	insulator
O4Si1Zr1_ICSD_186167	insulator	insulator
O4Si1Zr1_ICSD_186168	insulator	insulator
O4Si1Zr1_ICSD_187589	insulator	insulator
O4Sn1Sr2_ICSD_150388	insulator	insulator
O4Sn1Sr2_ICSD_59757	insulator	insulator
O4Sn1Sr2_ICSD_59758	insulator	insulator
O4Sn1Sr2_ICSD_81851	insulator	insulator
O4Sn1W1_ICSD_2147	insulator	insulator
O4Sn1W1_ICSD_2840	insulator	insulator
O4Sn1Zn2_ICSD_187039	insulator	insulator
O4Sn2Ti1_ICSD_163230	insulator	insulator
O4Sn3_ICSD_174299	insulator	insulator
O4Sr1Te1_ICSD_8098	insulator	insulator

Supplementary Table 554. Five-fold cross validated predictions for the metal/insulator classification (546/598).

system	calculated	predicted
O4Sr1Tl1V1_ICSD_36654	insulator	insulator
O4Sr1U1_ICSD_23196	insulator	insulator
O4Sr1U1_ICSD_31632	insulator	insulator
O4Sr1W1_ICSD_155745	insulator	insulator
O4Sr2Ti1_ICSD_157402	insulator	insulator
O4Sr2U1_ICSD_647477	insulator	insulator
O4Sr2V1_ICSD_71450	metal	metal
O4Ta1Tb1_ICSD_415434	insulator	insulator
O4Ta1Tm1_ICSD_415459	insulator	insulator
O4Ta1Yb1_ICSD_415460	insulator	insulator
O4Tb1V1_ICSD_78078	insulator	insulator
O4Tb1V1_ICSD_88369	insulator	insulator
O4Te1V1_ICSD_9402	insulator	insulator
O4Ti1Zn2_ICSD_166486	insulator	insulator
O4Tl2W1_ICSD_8212	insulator	insulator
O4Tm1V1_ICSD_15602	insulator	insulator
O4Tm1V1_ICSD_78081	insulator	insulator
O4U1Y1_ICSD_16492	metal	insulator
O4V1Y1_ICSD_246709	insulator	insulator
O4V1Y1_ICSD_246710	insulator	insulator
O4V1Yb1_ICSD_9398	metal	metal
O4V2Zn1_ICSD_55443	metal	metal
O4W1Zn1_ICSD_156483	insulator	insulator
O4W1Zn1_ICSD_162238	insulator	insulator
O5P1Sb1_ICSD_201743	insulator	insulator
O5P1Sn1V1_ICSD_415445	insulator	insulator
O5P1Ta1_ICSD_202041	insulator	insulator
O5P1Ta1_ICSD_87281	insulator	insulator
O5P1V1_ICSD_108983	insulator	insulator
O5P1V1_ICSD_415924	insulator	insulator
O5P1V1_ICSD_425552	insulator	insulator
O5P1V1_ICSD_77598	insulator	insulator
O5P1V1_ICSD_9413	insulator	insulator
O5P1V2_ICSD_79681	metal	insulator
O5P1W1_ICSD_203048	metal	insulator
O5P2_ICSD_655005	insulator	insulator
O5P2_ICSD_77377	metal	insulator
O5P2_ICSD_79698	insulator	insulator
O5P2_ICSD_82688	insulator	metal
O5Pb1Se2_ICSD_79688	insulator	insulator
O5Pb2S1_ICSD_14246	insulator	insulator
O5Pb2Te1_ICSD_407955	insulator	insulator
O5Pb2Te1_ICSD_425090	insulator	insulator
O5Pb2W1_ICSD_61399	insulator	insulator
O5Pb3Se1_ICSD_55076	insulator	insulator
O5S1Sn2_ICSD_35101	insulator	insulator
O5S1Ti1_ICSD_80627	insulator	insulator
O5S1Ti1_ICSD_81348	insulator	insulator
O5S1V1_ICSD_18307	insulator	insulator
O5S1V1_ICSD_80820	insulator	insulator

Supplementary Table 555. Five-fold cross validated predictions for the metal/insulator classification (547/598).

system	calculated	predicted
O5Sb1U1_ICSD_72771	insulator	insulator
O5Sb2V1_ICSD_2292	insulator	insulator
O5Sb2_ICSD_31104	metal	insulator
O5Sb2_ICSD_8050	insulator	insulator
O5Se1U1_ICSD_1623	insulator	insulator
O5Se2Zn1_ICSD_2355	insulator	insulator
O5Se2_ICSD_10471	insulator	insulator
O5Si1Sr3_ICSD_18151	insulator	insulator
O5Si1Y2_ICSD_28021	insulator	insulator
O5Si1Y2_ICSD_51591	insulator	insulator
O5Si2Sr1_ICSD_171567	insulator	insulator
O5Sr2U1_ICSD_23199	insulator	insulator
O5Ta1V1_ICSD_261430	insulator	insulator
O5Ta2_ICSD_188462	insulator	insulator
O5Ta2_ICSD_280397	insulator	insulator
O5Ta2_ICSD_95462	insulator	insulator
O5Te1Tl1V1_ICSD_173863	insulator	insulator
O5Te1U1_ICSD_160677	insulator	insulator
O5Te1U1_ICSD_1624	insulator	insulator
O5Te2Tl2_ICSD_86782	insulator	insulator
O5Te2_ICSD_2523	insulator	insulator
O5Ti1Y2_ICSD_34692	insulator	insulator
O5Ti3_ICSD_20361	insulator	insulator
O5Ti3_ICSD_75193	insulator	insulator
O5Ti4_ICSD_77697	metal	insulator
O5Ti5_ICSD_56694	metal	metal
O5U1V1_ICSD_66388	insulator	insulator
O5V2_ICSD_156051	insulator	insulator
O5V2_ICSD_156053	insulator	insulator
O5V2_ICSD_157988	insulator	insulator
O5V2_ICSD_80594	insulator	insulator
O5V2_ICSD_82152	insulator	insulator
O5V3_ICSD_15899	metal	insulator
O5V3_ICSD_16445	insulator	insulator
O6Os2Pb2_ICSD_23444	metal	metal
O6Os2Rb1_ICSD_157439	metal	metal
O6P2Pd1_ICSD_200677	insulator	insulator
O6P2Sr1_ICSD_280041	insulator	insulator
O6P2Zn1_ICSD_36040	insulator	insulator
O6P4S4_ICSD_27058	insulator	insulator
O6P4_ICSD_24407	insulator	insulator
O6Pb1Re2_ICSD_48205	metal	metal
O6Pb1Sb2_ICSD_81387	insulator	insulator
O6Pb1Se2_ICSD_154718	insulator	insulator
O6Pb1V2_ICSD_36464	insulator	insulator
O6Pb1V2_ICSD_36465	insulator	insulator
O6Pb1V2_ICSD_6109	insulator	insulator
O6Pb2Re1Tc1_ICSD_188745	metal	metal
O6Pb2Sc1Ta1_ICSD_77739	insulator	insulator
O6Pb2Sn2_ICSD_15308	metal	insulator

Supplementary Table 556. Five-fold cross validated predictions for the metal/insulator classification (548/598).

system	calculated	predicted
O6Pb3S1_ICSD_30711	insulator	insulator
O6Pb3S1_ICSD_30712	insulator	insulator
O6Pb3S1_ICSD_61429	insulator	insulator
O6Pb3S1_ICSD_63639	insulator	insulator
O6Pd1Sr4_ICSD_88135	insulator	insulator
O6Pt1Sr3Zn1_ICSD_280519	insulator	insulator
O6Pt1Sr4_ICSD_89329	insulator	insulator
O6Pt3Zn1_ICSD_35339	insulator	insulator
O6Rb2S2_ICSD_27581	insulator	insulator
O6Rb4_ICSD_33961	metal	insulator
O6Rb6Tl2_ICSD_62643	insulator	insulator
O6Re1Sn2Tc1_ICSD_188744	metal	metal
O6Re1Sr2Tb1_ICSD_25401	metal	metal
O6Re1Sr2Tm1_ICSD_25406	metal	metal
O6Re1Sr2Y1_ICSD_25404	metal	metal
O6Re1Sr2Yb1_ICSD_25407	metal	metal
O6Re1Sr2Zn1_ICSD_173489	metal	insulator
O6Re2Sb1_ICSD_51023	metal	metal
O6Rh1Sc1Sr3_ICSD_51137	insulator	insulator
O6Rh1Sr3Tb1_ICSD_51054	insulator	insulator
O6Rh1Sr3Y1_ICSD_51136	insulator	insulator
O6Rh1Sr3Yb1_ICSD_51058	insulator	metal
O6Rh1Sr3Zn1_ICSD_90285	insulator	insulator
O6Rh1Sr4_ICSD_109297	metal	insulator
O6Rh2U1_ICSD_23463	insulator	insulator
O6Ru1Sr2Y1_ICSD_49500	insulator	insulator
O6S1U1_ICSD_2579	insulator	insulator
O6Sb1Sr2Y1_ICSD_157887	insulator	insulator
O6Sb2Sr1_ICSD_74540	insulator	insulator
O6Sb2U1_ICSD_413442	insulator	insulator
O6Sb2W1_ICSD_75595	insulator	insulator
O6Sb2Zn1_ICSD_96612	insulator	insulator
O6Sb4_ICSD_36145	insulator	insulator
O6Sc2Te1_ICSD_417711	insulator	insulator
O6Se1U1_ICSD_2580	insulator	insulator
O6Se2Sn1_ICSD_154716	insulator	insulator
O6Se2Sn1_ICSD_154717	insulator	insulator
O6Se2Sr1Zn1_ICSD_280801	insulator	insulator
O6Se2Ti1_ICSD_200203	insulator	insulator
O6Sn1Ta2_ICSD_54078	insulator	insulator
O6Sr1Ta2_ICSD_39706	insulator	insulator
O6Sr2Ta1Y1_ICSD_247457	insulator	insulator
O6Sr2W1Zn1_ICSD_72811	insulator	insulator
O6Sr3U1_ICSD_23201	insulator	insulator
O6Ta2V1_ICSD_28490	insulator	insulator
O6Ta2Zn1_ICSD_36289	insulator	insulator
O6Te1Tl2_ICSD_4321	metal	insulator
O6Te1Tl6_ICSD_37135	insulator	insulator
O6Te1Y2_ICSD_240875	insulator	insulator
O6Te1Yb2_ICSD_62135	metal	metal

Supplementary Table 557. Five-fold cross validated predictions for the metal/insulator classification (549/598).

system	calculated	predicted
O6Th1Ti2_ICSD_201890	insulator	insulator
O6Th1Ti2_ICSD_89068	insulator	insulator
O6Ti2U1_ICSD_201342	insulator	insulator
O6U1V2_ICSD_28086	metal	insulator
O6V14_ICSD_200243	metal	metal
O6V2W1_ICSD_2575	metal	insulator
O6V2Zn1_ICSD_26998	insulator	insulator
O6V2Zn1_ICSD_30880	insulator	insulator
O6W1Y2_ICSD_20955	insulator	insulator
O6W1Y2_ICSD_65811	insulator	insulator
O6W1Yb2_ICSD_20956	insulator	insulator
O7Os1Tb3_ICSD_187410	insulator	insulator
O7P2Pd2_ICSD_166875	insulator	insulator
O7P2Rb1Ti1_ICSD_69848	insulator	insulator
O7P2Rb2Sr1_ICSD_39506	insulator	insulator
O7P2Si1_ICSD_14250	insulator	insulator
O7P2Si1_ICSD_19047	insulator	insulator
O7P2Si1_ICSD_75116	insulator	insulator
O7P2Sn1_ICSD_30583	insulator	insulator
O7P2Sr2_ICSD_16947	insulator	insulator
O7P2Sr2_ICSD_31004	insulator	insulator
O7P2Th1_ICSD_246234	insulator	insulator
O7P2Ti1_ICSD_189807	insulator	insulator
O7P2V1_ICSD_160942	insulator	insulator
O7P2V1_ICSD_200818	insulator	insulator
O7P2V1_ICSD_93022	insulator	insulator
O7P2W1_ICSD_90439	insulator	insulator
O7P2Zn2_ICSD_18315	insulator	insulator
O7P2Zn2_ICSD_24153	insulator	insulator
O7P2Zr1_ICSD_246397	insulator	insulator
O7P2Zr1_ICSD_280395	insulator	insulator
O7P4S1_ICSD_82358	insulator	insulator
O7P4Se1_ICSD_405209	insulator	insulator
O7Pb1Ti3_ICSD_2104	insulator	insulator
O7Pb2Sb2_ICSD_24246	metal	metal
O7Pb2Sb2_ICSD_27120	metal	insulator
O7Pb2Sb2_ICSD_39274	insulator	insulator
O7Pb3Si2_ICSD_15371	insulator	insulator
O7Pr2Ru2_ICSD_163397	metal	insulator
O7Pr2Ru2_ICSD_78123	insulator	metal
O7Pr2Sn2_ICSD_84744	insulator	insulator
O7Pr2Te2_ICSD_92444	metal	insulator
O7Pr2Zr2_ICSD_249972	insulator	insulator
O7Pr3Re1_ICSD_96410	insulator	insulator
O7Pr3Sb1_ICSD_78010	insulator	insulator
O7Pr3Ta1_ICSD_78008	insulator	insulator
O7Pt2Sb2_ICSD_108965	metal	insulator
O7Pt2Tl2_ICSD_22215	insulator	insulator
O7Rb2U2_ICSD_171465	insulator	insulator
O7Rb2W2_ICSD_300230	insulator	insulator

Supplementary Table 558. Five-fold cross validated predictions for the metal/insulator classification (550/598).

system	calculated	predicted
O7Re1Tb3_ICSD_99253	insulator	insulator
O7Ru2Tb2_ICSD_82305	insulator	insulator
O7Ru2Tl2_ICSD_51158	metal	metal
O7Ru2Y2_ICSD_73799	insulator	insulator
O7Ru2Yb2_ICSD_82306	metal	metal
O7S1Te2_ICSD_90837	insulator	insulator
O7S3Si2Y4_ICSD_261248	insulator	insulator
O7Sb2Sr2_ICSD_62960	insulator	insulator
O7Sc2Si2_ICSD_1457	insulator	insulator
O7Sc2Si2_ICSD_26682	insulator	insulator
O7Sc2Sr1Tb2_ICSD_167053	insulator	insulator
O7Si2Sr1V1_ICSD_30451	insulator	insulator
O7Si2Sr2Zn1_ICSD_247476	insulator	insulator
O7Si2Tl6_ICSD_4230	insulator	insulator
O7Si2Y2_ICSD_164147	insulator	insulator
O7Si2Y2_ICSD_164148	insulator	insulator
O7Si2Y2_ICSD_281312	insulator	insulator
O7Si2Y2_ICSD_28212	insulator	insulator
O7Si2Y2_ICSD_416573	insulator	insulator
O7Si2Yb2_ICSD_16048	insulator	insulator
O7Si2Yb2_ICSD_28097	insulator	insulator
O7Sn1Ta2_ICSD_15206	insulator	insulator
O7Sn2Ta2_ICSD_27119	insulator	insulator
O7Sn2Tb2_ICSD_159787	insulator	insulator
O7Sn2Tm2_ICSD_82963	insulator	insulator
O7Sn2Y2_ICSD_160115	insulator	insulator
O7Sn2Yb2_ICSD_82964	metal	insulator
O7Sr1V3_ICSD_241183	insulator	insulator
O7Sr3V2_ICSD_71320	insulator	metal
O7Sr3Zr2_ICSD_23731	insulator	insulator
O7Ta1Y3_ICSD_10059	insulator	insulator
O7Tb2Ti2_ICSD_159785	insulator	insulator
O7Te3Tl2_ICSD_150779	insulator	insulator
O7Ti2Y2_ICSD_66874	insulator	insulator
O7Ti2Yb2_ICSD_173750	metal	metal
O7Ti4_ICSD_10148	metal	insulator
O7Ti4_ICSD_19016	insulator	insulator
O7Ti4_ICSD_6098	insulator	insulator
O7Tl4V2_ICSD_72810	insulator	insulator
O7V2Y2_ICSD_160600	metal	insulator
O7V2Y2_ICSD_160865	insulator	metal
O7V2Yb2_ICSD_32689	metal	metal
O7V2Zn2_ICSD_250002	insulator	insulator
O7V2Zn2_ICSD_2886	insulator	insulator
O7V4_ICSD_2211	insulator	insulator
O7V4_ICSD_653554	metal	insulator
O7Y2Zr2_ICSD_153818	insulator	insulator
O8P2Pb3_ICSD_14247	insulator	insulator
O8P2Pb3_ICSD_38011	insulator	insulator
O8P2Pb3_ICSD_8095	insulator	insulator

Supplementary Table 559. Five-fold cross validated predictions for the metal/insulator classification (551/598).

system	calculated	predicted
O8P2Rb1Ta1_ICSD_54098	insulator	insulator
O8P2Sn1Sr1_ICSD_420128	insulator	insulator
O8P2Sr1Ti1_ICSD_420127	insulator	insulator
O8P2Sr1Zr1_ICSD_150336	insulator	insulator
O8P2Sr3_ICSD_150869	insulator	insulator
O8P2W1_ICSD_24067	insulator	insulator
O8P2Zn3_ICSD_27554	insulator	insulator
O8P2Zn3_ICSD_34303	insulator	insulator
O8P4_ICSD_406625	insulator	insulator
O8Pb2Te1U1_ICSD_380500	insulator	insulator
O8Pb3V2_ICSD_27651	insulator	insulator
O8Pb3V2_ICSD_29359	insulator	insulator
O8Pb3V2_ICSD_69799	insulator	insulator
O8Rb1S2Tl1_ICSD_21087	insulator	insulator
O8Rb1V3_ICSD_50009	insulator	insulator
O8Rb2V3_ICSD_79378	insulator	insulator
O8Rb3Ta1_ICSD_647346	insulator	insulator
O8Re2Zn1_ICSD_51017	insulator	insulator
O8S4Sn2_ICSD_32684	insulator	insulator
O8Se1Te3_ICSD_201784	insulator	insulator
O8Se2Sr1U1_ICSD_171869	insulator	insulator
O8Se2Te2_ICSD_201413	insulator	insulator
O8Si1Sn6_ICSD_156236	insulator	insulator
O8Si2Sr2Ti1_ICSD_290329	insulator	insulator
O8Sn1Te3_ICSD_9077	insulator	insulator
O8Sr1Te3_ICSD_416448	insulator	insulator
O8Sr3V2_ICSD_73258	insulator	insulator
O8Ta2U1_ICSD_27779	insulator	insulator
O8Ta2Zn3_ICSD_46001	insulator	insulator
O8Te2Tl2U1_ICSD_412560	insulator	insulator
O8Te3Ti1_ICSD_98902	insulator	insulator
O8Te3Zn2_ICSD_50705	insulator	insulator
O8Te3Zr1_ICSD_9079	insulator	insulator
O8Ti3Zn2_ICSD_83525	insulator	insulator
O8Tl1V3_ICSD_20688	insulator	insulator
O8U3_ICSD_28136	metal	metal
O8U3_ICSD_28138	metal	metal
O8U3_ICSD_36315	metal	insulator
O8U3_ICSD_38146	metal	metal
O8V2Zn3_ICSD_23776	insulator	insulator
O8W2Zr1_ICSD_262061	insulator	insulator
O8W3_ICSD_73719	metal	insulator
O8_ICSD_156481	insulator	insulator
O9P2Si1V1_ICSD_628	insulator	insulator
O9P2Te2_ICSD_248226	insulator	insulator
O9P2Th2_ICSD_417156	insulator	insulator
O9P2U2_ICSD_402120	insulator	insulator
O9P2V1Zn2_ICSD_69768	insulator	insulator
O9P2Zr2_ICSD_1922	insulator	insulator
O9P2Zr2_ICSD_416014	insulator	insulator

Supplementary Table 560. Five-fold cross validated predictions for the metal/insulator classification (552/598).

system	calculated	predicted
O9P3Pr1_ICSD_97950	insulator	insulator
O9P3Ru1_ICSD_80363	insulator	insulator
O9P3Sc1_ICSD_20430	insulator	insulator
O9P3V1_ICSD_20764	insulator	insulator
O9P3Y1_ICSD_421262	insulator	insulator
O9Pr1Ta3_ICSD_66285	insulator	insulator
O9Rb2Si3Sn1_ICSD_19028	insulator	insulator
O9Rb2Si3Ti1_ICSD_19026	insulator	insulator
O9Re2V1_ICSD_92317	insulator	insulator
O9S1Zr3_ICSD_68335	insulator	insulator
O9S2Sb2_ICSD_1948	insulator	insulator
O9S2U1_ICSD_423493	insulator	insulator
O9S2W1_ICSD_421958	insulator	insulator
O9S2Zn3_ICSD_15280	insulator	insulator
O9Sb3Tl2_ICSD_77330	metal	insulator
O9Sc2Se3_ICSD_98624	insulator	insulator
O9Sr1V4_ICSD_90926	insulator	insulator
O9Te2V2_ICSD_9255	metal	insulator
O9Te3U1_ICSD_9080	insulator	insulator
O9Te4_ICSD_1885	insulator	insulator
O9Ti3V2_ICSD_108830	metal	insulator
O9Ti4Tl2_ICSD_9239	insulator	insulator
O9Ti5_ICSD_653560	insulator	insulator
O9V4_ICSD_174441	insulator	insulator
O9V5_ICSD_6097	insulator	insulator
Os0.67W1.33_ICSD_647862	metal	metal
Os1P1S1_ICSD_647714	insulator	insulator
Os1P1Se1_ICSD_647716	insulator	insulator
Os1P1Ti1_ICSD_647718	metal	metal
Os1P1Zr1_ICSD_647719	metal	metal
Os1P1Zr1_ICSD_647720	metal	metal
Os1P2_ICSD_993	insulator	insulator
Os1P4_ICSD_647708	insulator	insulator
Os1S1Sb1_ICSD_647751	insulator	insulator
Os1S2_ICSD_300224	insulator	insulator
Os1Sb1Se1_ICSD_647759	insulator	insulator
Os1Sb1Te1_ICSD_647762	insulator	insulator
Os1Sb2_ICSD_647754	insulator	metal
Os1Sc6Te2_ICSD_98972	metal	metal
Os1Se2_ICSD_647769	insulator	insulator
Os1Si1Ti1_ICSD_647795	metal	metal
Os1Si1Zr1_ICSD_647807	metal	metal
Os1Si1_ICSD_15692	insulator	metal
Os1Si1_ICSD_43417	metal	metal
Os1Si2_ICSD_42730	insulator	insulator
Os1Si2_ICSD_647776	metal	insulator
Os1Si3Sm1_ICSD_647784	metal	metal
Os1Si3Th1_ICSD_647791	metal	metal
Os1Ta1Te4_ICSD_656454	metal	metal
Os1Tb3_ICSD_647820	metal	metal

Supplementary Table 561. Five-fold cross validated predictions for the metal/insulator classification (553/598).

system	calculated	predicted
Os1Te2_ICSD_647826	metal	metal
Os1Ti1_ICSD_647842	metal	metal
Os1V1_ICSD_150935	metal	metal
Os1Y3_ICSD_647869	metal	metal
Os1Zr1_ICSD_105580	metal	metal
Os1_ICSD_186792	metal	metal
Os1_ICSD_41523	metal	metal
Os2P2Sr1_ICSD_602096	metal	metal
Os2Pr1Si2_ICSD_647733	metal	metal
Os2Pr1Si2_ICSD_99059	metal	metal
Os2Pr1_ICSD_150519	metal	metal
Os2Pr1_ICSD_647722	metal	metal
Os2Pu1Si2_ICSD_73038	metal	metal
Os2Pu1_ICSD_105559	metal	metal
Os2Pu1_ICSD_647738	metal	metal
Os2Sc1_ICSD_647766	metal	metal
Os2Si2Sm1_ICSD_647786	metal	metal
Os2Si2Tb1_ICSD_90339	metal	metal
Os2Si2Th1_ICSD_603982	metal	metal
Os2Si2U1_ICSD_604008	metal	metal
Os2Si2Y1_ICSD_647804	metal	metal
Os2Si2Yb1_ICSD_647806	metal	metal
Os2Si3_ICSD_647782	insulator	insulator
Os2Si3_ICSD_95591	insulator	insulator
Os2Sm1_ICSD_150521	metal	metal
Os2Tb1_ICSD_647822	metal	metal
Os2Th1_ICSD_150741	metal	metal
Os2U1_ICSD_657117	metal	metal
Os2U1_ICSD_659012	metal	metal
Os2Y1_ICSD_150518	metal	metal
Os2Yb1_ICSD_647872	metal	metal
Os2Zr1_ICSD_647878	metal	metal
Os3Sn7_ICSD_105567	metal	metal
Os3Th7_ICSD_150669	metal	metal
Os3Ti1_ICSD_185638	metal	metal
Os3Zr1_ICSD_185644	metal	metal
Os4P12Pr1_ICSD_647712	metal	metal
Os4Pr1Sb12_ICSD_155181	metal	metal
Os4Pr1Sb12_ICSD_155186	metal	metal
Os4Sb12Sr1_ICSD_658734	metal	metal
Os4Sc11_ICSD_10439	metal	metal
Os4Zr11_ICSD_647874	metal	metal
Os7Sc44_ICSD_105565	metal	metal
P12Pr1Ru4_ICSD_155822	metal	metal
P12Ru4Tb1_ICSD_245294	metal	metal
P12Ru4Th1_ICSD_648033	metal	metal
P13Re6_ICSD_23357	metal	insulator
P14Sn1Zn1_ICSD_601339	insulator	insulator
P14Sr3_ICSD_42461	metal	insulator
P1Pb1Rb1S4_ICSD_249870	insulator	insulator

Supplementary Table 562. Five-fold cross validated predictions for the metal/insulator classification (554/598).

system	calculated	predicted
P1Pb1S4Tl1_ICSD_249170	insulator	insulator
P1Pd1Pr1_ICSD_57159	metal	metal
P1Pd1S1_ICSD_2331	insulator	insulator
P1Pd1Se1_ICSD_77772	insulator	insulator
P1Pd1Sm1_ICSD_57160	metal	metal
P1Pd3_ICSD_85525	metal	metal
P1Pd5Sn1_ICSD_647931	metal	metal
P1Pd5Tl1_ICSD_647937	metal	metal
P1Pd5Zn1_ICSD_647941	metal	metal
P1Pd6_ICSD_26898	metal	metal
P1Pr1_ICSD_77773	metal	metal
P1Pt1Sc1_ICSD_417911	metal	metal
P1Pt1Sm1_ICSD_77776	metal	metal
P1Pt1Tb1_ICSD_77777	metal	metal
P1Pt1Tm1_ICSD_77778	metal	metal
P1Pt1Y1_ICSD_77779	metal	metal
P1Pt1Yb1_ICSD_44967	metal	metal
P1Pt5Sn1_ICSD_647972	metal	metal
P1Pt5Tl1_ICSD_647975	metal	metal
P1Pt5Zn1_ICSD_647976	metal	metal
P1Pu1_ICSD_77781	metal	metal
P1Re2_ICSD_43564	metal	metal
P1Re4Zr9_ICSD_647990	metal	metal
P1Rh1Se1_ICSD_648002	insulator	insulator
P1Rh1Ta1_ICSD_648003	metal	metal
P1Rh1Zr1_ICSD_648009	insulator	metal
P1Rh2_ICSD_648000	metal	metal
P1Ru1S1_ICSD_648023	insulator	insulator
P1Ru1Sc1_ICSD_77790	metal	metal
P1Ru1Se1_ICSD_648028	insulator	insulator
P1Ru1Ta1_ICSD_648031	metal	metal
P1Ru1Ti1_ICSD_77792	metal	metal
P1Ru1Zr1_ICSD_648037	metal	metal
P1Ru1Zr1_ICSD_648038	metal	metal
P1Ru1_ICSD_648015	metal	metal
P1Ru2_ICSD_43686	metal	metal
P1S1Ta1_ICSD_648183	metal	metal
P1S1Tb1_ICSD_648063	insulator	insulator
P1S1Th1_ICSD_154031	metal	metal
P1S1Y1_ICSD_648080	insulator	insulator
P1S3Sn1_ICSD_657344	insulator	insulator
P1S3Zn1_ICSD_79557	insulator	insulator
P1S4Sc1_ICSD_67559	insulator	insulator
P1S4Sn1Tl1_ICSD_68294	insulator	insulator
P1S4Sr1Tl1_ICSD_249346	insulator	insulator
P1S4Tl3_ICSD_201062	insulator	insulator
P1S5Ti1Tl1_ICSD_171214	insulator	insulator
P1Sb1Ti2_ICSD_77796	metal	metal
P1Sb1Zr2_ICSD_77797	metal	metal
P1Sc1_ICSD_157504	metal	metal

Supplementary Table 563. Five-fold cross validated predictions for the metal/insulator classification (555/598).

system	calculated	predicted
P1Sc1_ICSD_180831	metal	metal
P1Sc1_ICSD_188693	insulator	metal
P1Sc3_ICSD_648101	metal	metal
P1Se1Th1_ICSD_648115	metal	metal
P1Se1U1_ICSD_42169	metal	metal
P1Se3Sn1_ICSD_655564	insulator	insulator
P1Se4Tl3_ICSD_41703	insulator	insulator
P1Si1_ICSD_30334	metal	metal
P1Sm1_ICSD_44127	metal	metal
P1Sm1_ICSD_648152	metal	metal
P1Sn1Sr1_ICSD_63594	metal	insulator
P1Sn1_ICSD_16077	metal	metal
P1Sn1_ICSD_77786	metal	metal
P1Sn3Zr5_ICSD_656295	metal	metal
P1Sr1_ICSD_26262	insulator	insulator
P1Ta1_ICSD_108656	metal	metal
P1Ta1_ICSD_648185	metal	metal
P1Ta2_ICSD_43067	metal	metal
P1Ta3_ICSD_10125	metal	metal
P1Ta3_ICSD_648178	metal	metal
P1Tb1_ICSD_648194	metal	metal
P1Tc3_ICSD_35116	metal	metal
P1Te1U1_ICSD_77846	metal	metal
P1Te2Ti2_ICSD_418978	metal	metal
P1Te2Zr2_ICSD_420650	metal	metal
P1Th1_ICSD_648205	metal	metal
P1Ti1_ICSD_76025	metal	metal
P1Ti3_ICSD_43062	metal	metal
P1Tl1_ICSD_184576	metal	metal
P1Tm1_ICSD_77850	metal	metal
P1U1_ICSD_648248	metal	metal
P1U1_ICSD_77852	metal	metal
P1V1Zr1_ICSD_39562	metal	metal
P1V1_ICSD_648267	metal	metal
P1V2_ICSD_648278	metal	metal
P1W1_ICSD_648280	metal	metal
P1W3_ICSD_648282	metal	metal
P1Y1_ICSD_185495	metal	metal
P1Y1_ICSD_77857	metal	metal
P1Yb1_ICSD_656116	metal	metal
P1Zr1_ICSD_77860	metal	metal
P1Zr1_ICSD_77861	metal	metal
P1Zr3_ICSD_43207	metal	metal
P1_ICSD_150873	insulator	metal
P1_ICSD_162244	metal	metal
P1_ICSD_169539	metal	metal
P1_ICSD_27847	metal	metal
P1_ICSD_53301	metal	metal
P1_ICSD_98122	metal	metal
P2Pa1_ICSD_647903	metal	metal

Supplementary Table 564. Five-fold cross validated predictions for the metal/insulator classification (556/598).

system	calculated	predicted
P2Pa1_ICSD_647905	metal	metal
P2Pb2S6_ICSD_40756	insulator	insulator
P2Pb2S6_ICSD_647906	insulator	insulator
P2Pb2Se6_ICSD_40757	metal	insulator
P2Pb2Se6_ICSD_647911	insulator	insulator
P2Pb3S8_ICSD_36473	insulator	insulator
P2Pd15_ICSD_1096	metal	metal
P2Pd2S6_ICSD_647926	metal	insulator
P2Pd2Sr1_ICSD_647932	metal	metal
P2Pd3S8_ICSD_35361	insulator	insulator
P2Pr1Rh2_ICSD_647955	metal	metal
P2Pr1Ru2_ICSD_602105	metal	metal
P2Pt1Si3_ICSD_84944	insulator	insulator
P2Pt1_ICSD_647970	insulator	insulator
P2Pt5_ICSD_24327	metal	metal
P2Rb1S7V1_ICSD_73782	insulator	insulator
P2Rb2S6_ICSD_416176	insulator	insulator
P2Rb2Se6_ICSD_173419	insulator	insulator
P2Rb3S8Sm1_ICSD_419350	insulator	insulator
P2Rh1_ICSD_174228	insulator	insulator
P2Rh2Sr1_ICSD_50186	metal	metal
P2Rh3_ICSD_35626	metal	metal
P2Ru1_ICSD_42737	insulator	insulator
P2Ru2Sm1_ICSD_602121	metal	metal
P2Ru2Sr1_ICSD_648030	metal	metal
P2Ru2Tb1_ICSD_602100	metal	metal
P2Ru2Y1_ICSD_602128	metal	metal
P2Ru2Yb1_ICSD_602104	metal	metal
P2S6Sn2_ICSD_25357	insulator	insulator
P2S6Sn2_ICSD_39232	insulator	insulator
P2S6Sn2_ICSD_648056	insulator	insulator
P2S6Sr2_ICSD_405191	insulator	insulator
P2S6Th1_ICSD_35299	insulator	insulator
P2S6Ti1_ICSD_16403	insulator	insulator
P2S6Tl2_ICSD_38255	insulator	insulator
P2S6U1_ICSD_413318	metal	insulator
P2S6V2_ICSD_648076	insulator	insulator
P2S6Zr1_ICSD_50700	insulator	insulator
P2S7Zr1_ICSD_50701	insulator	insulator
P2S7_ICSD_423061	insulator	insulator
P2S7_ICSD_423062	insulator	insulator
P2S8Zn3_ICSD_95784	insulator	insulator
P2Sc3_ICSD_41679	metal	metal
P2Se5_ICSD_74546	insulator	insulator
P2Se6Sn2_ICSD_403097	insulator	insulator
P2Se6Sr2_ICSD_412766	metal	insulator
P2Si1Zn1_ICSD_648145	insulator	insulator
P2Si1_ICSD_24333	metal	insulator
P2Si1_ICSD_43098	insulator	insulator
P2Sn1Zn1_ICSD_77803	insulator	insulator

Supplementary Table 565. Five-fold cross validated predictions for the metal/insulator classification (557/598).

system	calculated	predicted
P2Sr1Zn2_ICSD_30911	insulator	insulator
P2Ta1_ICSD_648187	metal	metal
P2Th1_ICSD_648206	metal	metal
P2Ti1_ICSD_24351	metal	metal
P2U1_ICSD_76255	metal	metal
P2U1_ICSD_87138	metal	metal
P2V1_ICSD_648276	metal	metal
P2W1_ICSD_37223	metal	metal
P2W1_ICSD_648286	metal	metal
P2Yb1Zn2_ICSD_648298	insulator	insulator
P2Zn1_ICSD_18137	insulator	insulator
P2Zn1_ICSD_250014	insulator	insulator
P2Zn1_ICSD_43334	metal	insulator
P2Zn1_ICSD_648308	insulator	insulator
P2Zn3_ICSD_24487	metal	metal
P2Zn3_ICSD_648299	insulator	insulator
P2Zn3_ICSD_648310	insulator	insulator
P2Zr1_ICSD_24352	metal	metal
P3Pd1_ICSD_647923	metal	metal
P3Pd3Sr2_ICSD_36631	metal	metal
P3Pd7_ICSD_200055	metal	metal
P3Pr1Zn3_ICSD_88675	metal	metal
P3Rb1Zn4_ICSD_262039	insulator	insulator
P3Rb2_ICSD_654296	insulator	insulator
P3Re1_ICSD_647985	insulator	insulator
P3Rh1_ICSD_23712	metal	metal
P3Rh4_ICSD_43704	metal	metal
P3Ru1_ICSD_62420	insulator	insulator
P3Sc7_ICSD_648099	metal	metal
P3Sm1Zn3_ICSD_81030	metal	metal
P3Sn1_ICSD_16293	metal	metal
P3Sn4_ICSD_15014	metal	metal
P3Sr1_ICSD_23628	insulator	insulator
P3Sr1_ICSD_96543	metal	metal
P3Tc1_ICSD_35200	insulator	insulator
P3Tc2_ICSD_41016	metal	metal
P3Tc2_ICSD_41017	metal	metal
P3Ti4_ICSD_648219	metal	metal
P3Ti5_ICSD_77848	metal	metal
P3V4_ICSD_648275	metal	metal
P3V5_ICSD_648274	metal	metal
P4Pa3_ICSD_647904	metal	metal
P4Pd7Tb3_ICSD_409874	metal	metal
P4Re1_ICSD_8197	insulator	insulator
P4Re3_ICSD_48124	metal	metal
P4Rh6Sc1_ICSD_182778	metal	metal
P4Rh6Yb1_ICSD_182779	metal	metal
P4Ru1Si4_ICSD_79006	insulator	insulator
P4Ru1_ICSD_2492	insulator	insulator
P4Ru1_ICSD_648018	insulator	insulator

Supplementary Table 566. Five-fold cross validated predictions for the metal/insulator classification (558/598).

system	calculated	predicted
P4S10_ICSD_174009	insulator	insulator
P4S13V2_ICSD_201569	insulator	insulator
P4S3_ICSD_417155	insulator	insulator
P4S4_ICSD_1703	insulator	insulator
P4S5_ICSD_16681	insulator	insulator
P4S5_ICSD_1995	insulator	insulator
P4S6_ICSD_423037	insulator	insulator
P4Se3_ICSD_40204	insulator	insulator
P4Se4_ICSD_74878	insulator	insulator
P4Se5_ICSD_16140	insulator	insulator
P4Sr3_ICSD_38321	insulator	insulator
P4Sr4Ti1_ICSD_380112	insulator	insulator
P4Tc1_ICSD_35117	insulator	insulator
P4Th3_ICSD_25724	metal	metal
P4Ti7_ICSD_648209	metal	metal
P4U3_ICSD_648243	metal	metal
P4V1_ICSD_38315	metal	metal
P4Zn1_ICSD_40428	insulator	insulator
P4Zr7_ICSD_40281	metal	metal
P4_ICSD_154318	insulator	insulator
P4_ICSD_68326	insulator	insulator
P5Pr1_ICSD_409182	metal	metal
P5Re2_ICSD_24808	insulator	metal
P5Sm1_ICSD_409183	metal	metal
P5Tl1_ICSD_15021	insulator	insulator
P5Tm1_ICSD_409186	metal	metal
P5Y1_ICSD_409188	insulator	metal
P5Yb1_ICSD_409179	metal	metal
P5Yb1_ICSD_409180	insulator	metal
P6Pt4Sr1_ICSD_62517	insulator	insulator
P6S18Zn4_ICSD_1434	insulator	insulator
P6Sn2Sr5_ICSD_63593	insulator	insulator
P7Ru12Sc2_ICSD_77791	metal	metal
P9Zr14_ICSD_31785	metal	metal
Pa1Pt3_ICSD_648336	metal	metal
Pa1Pt5_ICSD_648334	metal	metal
Pa1Rh3_ICSD_105586	metal	metal
Pa1Sb2_ICSD_648339	metal	metal
Pa1_ICSD_648331	metal	metal
Pa1_ICSD_649179	metal	metal
Pa3Sb4_ICSD_601258	metal	metal
Pb0.33S2Ta1_ICSD_74693	metal	metal
Pb13Pr3Rh4_ICSD_648389	metal	metal
Pb13Rh4Sr3_ICSD_105610	metal	metal
Pb1Pd1Pr1_ICSD_657942	metal	metal
Pb1Pd1Y1_ICSD_54315	metal	metal
Pb1Pd1Yb1_ICSD_657947	metal	metal
Pb1Pd2Sm2_ICSD_99193	metal	metal
Pb1Pd2Tb2_ICSD_99195	metal	metal
Pb1Pd2Tm2_ICSD_99199	metal	metal

Supplementary Table 567. Five-fold cross validated predictions for the metal/insulator classification (559/598).

system	calculated	predicted
Pb1Pd2Y1_ICSD_105597	metal	metal
Pb1Pd2Y2_ICSD_99189	metal	metal
Pb1Pd3_ICSD_648358	metal	metal
Pb1Pr3_ICSD_648387	metal	metal
Pb1Pt1_ICSD_105602	metal	metal
Pb1Pt1_ICSD_648398	metal	metal
Pb1Pt3_ICSD_648399	metal	metal
Pb1Pt5Si1_ICSD_648402	metal	metal
Pb1Pu3_ICSD_157519	metal	metal
Pb1Rh1_ICSD_648421	metal	metal
Pb1S1_ICSD_183240	insulator	insulator
Pb1S1_ICSD_183241	insulator	insulator
Pb1S1_ICSD_183243	insulator	insulator
Pb1S1_ICSD_183244	metal	insulator
Pb1S1_ICSD_183245	metal	metal
Pb1S1_ICSD_183248	insulator	insulator
Pb1S1_ICSD_183251	insulator	insulator
Pb1S1_ICSD_183254	insulator	insulator
Pb1S1_ICSD_183255	insulator	metal
Pb1S1_ICSD_648435	insulator	insulator
Pb1S1_ICSD_648438	insulator	insulator
Pb1S1_ICSD_68712	insulator	insulator
Pb1S1_ICSD_68969	insulator	insulator
Pb1S1_ICSD_77865	metal	metal
Pb1S2Ta1_ICSD_648482	metal	metal
Pb1S3Sn1_ICSD_23462	insulator	insulator
Pb1S3Zr1_ICSD_2439	metal	insulator
Pb1S4Sc2_ICSD_154527	insulator	insulator
Pb1Sb2Te4_ICSD_250250	insulator	insulator
Pb1Sc2Se4_ICSD_154528	insulator	insulator
Pb1Se1_ICSD_648514	insulator	insulator
Pb1Se1_ICSD_648522	insulator	metal
Pb1Se1_ICSD_77870	metal	metal
Pb1Se2Ta1_ICSD_648539	metal	metal
Pb1Se2_ICSD_174577	metal	insulator
Pb1Se3Tl4_ICSD_261016	insulator	insulator
Pb1Se3Tl4_ICSD_648545	metal	metal
Pb1Sr1Zn1_ICSD_54319	metal	metal
Pb1Sr1_ICSD_105623	metal	metal
Pb1Sr2_ICSD_105624	metal	insulator
Pb1Te1_ICSD_648582	insulator	metal
Pb1Te1_ICSD_648606	insulator	metal
Pb1Te1_ICSD_77882	metal	metal
Pb1Te3Tl4_ICSD_648620	metal	metal
Pb1Th1_ICSD_105631	metal	metal
Pb1U1_ICSD_105636	metal	metal
Pb1V3_ICSD_105639	metal	metal
Pb1Yb1Zn1_ICSD_648669	metal	metal
Pb1Yb1_ICSD_648664	metal	metal
Pb1Yb2_ICSD_105645	metal	metal

Supplementary Table 568. Five-fold cross validated predictions for the metal/insulator classification (560/598).

system	calculated	predicted
Pb1_ICSD_54314	metal	metal
Pb1_ICSD_77864	metal	metal
Pb1_ICSD_96501	metal	metal
Pb2Pd1_ICSD_105590	metal	metal
Pb2Pd3S2_ICSD_159361	metal	metal
Pb2Pd3S2_ICSD_159365	metal	metal
Pb2Pd3Se2_ICSD_648366	metal	metal
Pb2Pd3Te2_ICSD_163134	metal	metal
Pb2Pt1_ICSD_54316	metal	metal
Pb2Rh1_ICSD_105608	metal	metal
Pb2Rh3S2_ICSD_420727	metal	metal
Pb2Rh3Se2_ICSD_648427	metal	metal
Pb2S4Si1_ICSD_16317	insulator	insulator
Pb2S5Sb2_ICSD_35640	insulator	insulator
Pb2Se4Si1_ICSD_16318	insulator	insulator
Pb2Y1_ICSD_105640	metal	metal
Pb3Pd5_ICSD_648361	metal	metal
Pb3Pr1_ICSD_105600	metal	metal
Pb3Pr1_ICSD_648380	metal	metal
Pb3S12Sn3Y2_ICSD_159889	insulator	insulator
Pb3S15Sb8_ICSD_41849	insulator	insulator
Pb3Sc5_ICSD_105614	metal	metal
Pb3Sr1_ICSD_105625	metal	metal
Pb3Sr2_ICSD_648570	metal	metal
Pb3Tb1_ICSD_105629	metal	metal
Pb3Tb5_ICSD_648576	metal	metal
Pb3Th1_ICSD_648629	metal	metal
Pb3Th5_ICSD_648630	metal	metal
Pb3Tm1_ICSD_105635	metal	metal
Pb3U1_ICSD_648650	metal	metal
Pb3Y1_ICSD_648658	metal	metal
Pb3Y5_ICSD_648656	metal	metal
Pb3Yb1_ICSD_105647	metal	metal
Pb3Yb5_ICSD_105648	metal	metal
Pb3Zr5_ICSD_648672	metal	metal
Pb4Pt1_ICSD_648397	metal	metal
Pb4S11Sb4_ICSD_200601	metal	insulator
Pb5Rh4_ICSD_105609	metal	metal
Pb5Sc6_ICSD_170063	metal	metal
Pd10Te3_ICSD_77903	metal	metal
Pd13Te3_ICSD_172382	metal	metal
Pd13Tl9_ICSD_105731	metal	metal
Pd16S7_ICSD_32053	metal	metal
Pd17Se15_ICSD_108785	metal	metal
Pd17Te4_ICSD_649007	metal	metal
Pd1Pr1Sb1_ICSD_648694	metal	metal
Pd1Pr1Sb2_ICSD_93154	metal	metal
Pd1Pr1Sn1_ICSD_419193	metal	metal
Pd1Pr1Sn1_ICSD_54459	metal	metal
Pd1Pr1Zn1_ICSD_54325	metal	metal

Supplementary Table 569. Five-fold cross validated predictions for the metal/insulator classification (561/598).

system	calculated	predicted
Pd1Pr1_ICSD_648691	metal	metal
Pd1Pr1_ICSD_656730	metal	metal
Pd1Pu1_ICSD_105658	metal	metal
Pd1Rb2Se16_ICSD_410443	insulator	insulator
Pd1Rb2Se2_ICSD_648724	insulator	insulator
Pd1Rb2Te2_ICSD_648727	insulator	insulator
Pd1S1Sb1_ICSD_648757	metal	metal
Pd1S1_ICSD_648756	insulator	metal
Pd1S2_ICSD_648753	insulator	insulator
Pd1S4U2_ICSD_63590	insulator	metal
Pd1S6Ta2_ICSD_655261	metal	metal
Pd1Sb1Sc1_ICSD_415944	insulator	metal
Pd1Sb1Se1_ICSD_648784	metal	metal
Pd1Sb1Sm1_ICSD_648787	metal	metal
Pd1Sb1Tb1_ICSD_648792	metal	metal
Pd1Sb1Te1_ICSD_93906	metal	metal
Pd1Sb1Tm1_ICSD_54328	metal	metal
Pd1Sb1U1_ICSD_648798	metal	metal
Pd1Sb1Yb1_ICSD_391182	metal	metal
Pd1Sb1Yb1_ICSD_391183	metal	metal
Pd1Sb1Yb1_ICSD_648804	metal	metal
Pd1Sb1Zr1_ICSD_92441	metal	metal
Pd1Sb1_ICSD_42598	metal	metal
Pd1Sb2Sm1_ICSD_658221	metal	metal
Pd1Sb2Tb1_ICSD_658223	metal	metal
Pd1Sb2U1_ICSD_50349	metal	metal
Pd1Sb2_ICSD_648773	metal	metal
Pd1Sc1Si1_ICSD_648814	metal	metal
Pd1Sc1Sn1_ICSD_648815	metal	metal
Pd1Sc1Zn1_ICSD_424601	metal	metal
Pd1Sc1_ICSD_648811	metal	metal
Pd1Sc2_ICSD_648813	metal	metal
Pd1Se1_ICSD_409568	insulator	metal
Pd1Se1_ICSD_648823	metal	insulator
Pd1Se2Tl2_ICSD_79601	metal	metal
Pd1Se2_ICSD_16693	insulator	metal
Pd1Se6Ta2_ICSD_600717	metal	metal
Pd1Si1Sr1_ICSD_69791	metal	metal
Pd1Si1Ti1_ICSD_648877	metal	metal
Pd1Si1U1_ICSD_86952	metal	metal
Pd1Si1Y1_ICSD_408080	metal	metal
Pd1Si1Zr1_ICSD_648895	metal	metal
Pd1Si1_ICSD_659957	metal	metal
Pd1Sm1Sn1_ICSD_648908	metal	metal
Pd1Sm1Zn1_ICSD_656590	metal	metal
Pd1Sm1_ICSD_648907	metal	metal
Pd1Sn1Tb1_ICSD_648933	metal	metal
Pd1Sn1Te1_ICSD_162404	metal	metal
Pd1Sn1U1_ICSD_54332	metal	metal
Pd1Sn1U1_ICSD_54384	metal	metal

Supplementary Table 570. Five-fold cross validated predictions for the metal/insulator classification (562/598).

system	calculated	predicted
Pd1Sn1U1_ICSD_648945	metal	metal
Pd1Sn1Y1_ICSD_424012	metal	metal
Pd1Sn1Yb1_ICSD_151166	metal	metal
Pd1Sn1Yb1_ICSD_151167	metal	metal
Pd1Sn1Zr1_ICSD_105705	insulator	insulator
Pd1Sn1_ICSD_105683	metal	metal
Pd1Sn1_ICSD_648928	metal	metal
Pd1Sn2Yb1_ICSD_411798	metal	metal
Pd1Sn3Sr1_ICSD_105692	metal	metal
Pd1Sn3_ICSD_105685	metal	metal
Pd1Sn4_ICSD_105686	metal	metal
Pd1Sr1Tl2_ICSD_165577	metal	metal
Pd1Sr1_ICSD_648963	metal	metal
Pd1Ta1_ICSD_648973	metal	metal
Pd1Tb1Tl1_ICSD_105713	metal	metal
Pd1Tb1Zn1_ICSD_183340	metal	metal
Pd1Tb1_ICSD_648983	metal	metal
Pd1Tb1_ICSD_648985	metal	metal
Pd1Te1_ICSD_659960	metal	metal
Pd1Te2_ICSD_41387	metal	metal
Pd1Th1_ICSD_105716	metal	metal
Pd1Th2_ICSD_102813	metal	metal
Pd1Ti1_ICSD_167647	metal	metal
Pd1Ti1_ICSD_167649	metal	metal
Pd1Ti1_ICSD_184673	metal	metal
Pd1Ti1_ICSD_649035	metal	metal
Pd1Ti2_ICSD_105722	metal	metal
Pd1Ti3_ICSD_167644	metal	metal
Pd1Tl1Y1_ICSD_105732	metal	metal
Pd1Tl1Yb1_ICSD_105733	metal	metal
Pd1Tl2_ICSD_102796	metal	metal
Pd1Tm1_ICSD_105734	metal	metal
Pd1Tm1_ICSD_649076	metal	metal
Pd1V3_ICSD_649092	metal	metal
Pd1Y1Zn1_ICSD_183333	metal	metal
Pd1Y1Zn1_ICSD_183334	metal	metal
Pd1Y1_ICSD_169021	metal	metal
Pd1Y3_ICSD_649111	metal	metal
Pd1Yb1Zn1_ICSD_159304	metal	metal
Pd1Yb1_ICSD_105748	metal	metal
Pd1Yb1_ICSD_649124	insulator	metal
Pd1Yb3_ICSD_649132	metal	metal
Pd1Zn1_ICSD_649135	metal	metal
Pd1Zr1_ICSD_186410	metal	metal
Pd1Zr1_ICSD_55546	metal	metal
Pd1Zr2_ICSD_105758	metal	metal
Pd1_ICSD_64918	metal	metal
Pd20Sb7_ICSD_648771	metal	metal
Pd20Si6U3_ICSD_93930	metal	metal
Pd20Te7_ICSD_42551	metal	metal

Supplementary Table 571. Five-fold cross validated predictions for the metal/insulator classification (563/598).

system	calculated	predicted
Pd2Pr1Si2_ICSD_601261	metal	metal
Pd2Pr1Si2_ICSD_648697	metal	metal
Pd2Pu1Si2_ICSD_73033	metal	metal
Pd2Pu2Sn1_ICSD_150165	metal	metal
Pd2Rh15S15_ICSD_648731	metal	metal
Pd2S4U1_ICSD_62121	insulator	metal
Pd2Sb1_ICSD_77889	metal	metal
Pd2Sb2Sr1_ICSD_61194	metal	metal
Pd2Sb2Sr1_ICSD_61195	metal	metal
Pd2Sc1Sn1_ICSD_105676	metal	metal
Pd2Si1Sm1_ICSD_648860	metal	metal
Pd2Si1Tb1_ICSD_648866	metal	metal
Pd2Si1Y1_ICSD_35087	metal	metal
Pd2Si1Yb1_ICSD_247422	metal	metal
Pd2Si1_ICSD_43209	metal	metal
Pd2Si1_ICSD_659210	metal	metal
Pd2Si2Sm1_ICSD_601262	metal	metal
Pd2Si2Sm1_ICSD_648862	metal	metal
Pd2Si2Sr1_ICSD_648864	metal	metal
Pd2Si2Tb1_ICSD_648868	metal	metal
Pd2Si2Th1_ICSD_77899	metal	metal
Pd2Si2Tm1_ICSD_648879	metal	metal
Pd2Si2U1_ICSD_108867	metal	metal
Pd2Si2Y1_ICSD_648889	metal	metal
Pd2Si2Yb1_ICSD_648891	metal	metal
Pd2Si3Y3_ICSD_35086	metal	metal
Pd2Sn1Tb1_ICSD_105693	metal	metal
Pd2Sn1Tm1_ICSD_105695	metal	metal
Pd2Sn1U2_ICSD_107370	metal	metal
Pd2Sn1Y1_ICSD_648950	metal	metal
Pd2Sn1Yb1_ICSD_105704	metal	metal
Pd2Sn1_ICSD_158364	metal	metal
Pd2Sr1_ICSD_648962	metal	metal
Pd2Ta1_ICSD_648976	metal	metal
Pd2Ti1_ICSD_167652	metal	metal
Pd2Ti1_ICSD_184671	metal	metal
Pd2Tl1_ICSD_649055	metal	metal
Pd2V1_ICSD_105741	metal	metal
Pd2Y3_ICSD_649100	metal	metal
Pd2Yb5_ICSD_649131	metal	metal
Pd2Zn1_ICSD_649139	metal	metal
Pd2Zr1_ICSD_186413	metal	metal
Pd3Pr1_ICSD_648684	metal	metal
Pd3Pu1_ICSD_105659	metal	metal
Pd3Rb2S4_ICSD_41886	insulator	insulator
Pd3Rb2S4_ICSD_648723	insulator	insulator
Pd3Rb2S6U1_ICSD_262562	insulator	insulator
Pd3Rb2Se4_ICSD_33895	insulator	insulator
Pd3Rb2Se6U1_ICSD_262561	insulator	insulator
Pd3S1_ICSD_24347	metal	metal

Supplementary Table 572. Five-fold cross validated predictions for the metal/insulator classification (564/598).

system	calculated	predicted
Pd3S1_ICSD_648754	metal	metal
Pd3S2Tl2_ICSD_648762	metal	metal
Pd3S4Sm1_ICSD_648760	metal	metal
Pd3S4Y1_ICSD_648764	metal	metal
Pd3Sb4U3_ICSD_648799	metal	metal
Pd3Sc1_ICSD_659956	metal	metal
Pd3Se2Tl2_ICSD_648844	metal	metal
Pd3Se8Ta2_ICSD_73318	insulator	insulator
Pd3Si1_ICSD_648855	metal	metal
Pd3Sm1_ICSD_105679	metal	metal
Pd3Sn1_ICSD_648912	metal	metal
Pd3Ta1_ICSD_105709	metal	metal
Pd3Ta1_ICSD_648968	metal	metal
Pd3Tb1_ICSD_648982	metal	metal
Pd3Te2_ICSD_77902	metal	metal
Pd3Th1_ICSD_150532	metal	metal
Pd3Ti1_ICSD_105724	metal	metal
Pd3Ti1_ICSD_649037	metal	metal
Pd3Ti2_ICSD_105725	metal	metal
Pd3Tl1_ICSD_108675	metal	metal
Pd3Tl1_ICSD_247272	metal	metal
Pd3Tl1_ICSD_649062	metal	metal
Pd3Tm1_ICSD_105736	metal	metal
Pd3Tm1_ICSD_54335	metal	metal
Pd3Tm1_ICSD_649074	metal	metal
Pd3Tm1_ICSD_656121	metal	metal
Pd3U1_ICSD_649080	metal	metal
Pd3U1_ICSD_656100	metal	metal
Pd3V1_ICSD_105742	metal	metal
Pd3Y1_ICSD_105745	metal	metal
Pd3Yb1_ICSD_649116	metal	metal
Pd3Zr1_ICSD_105761	metal	metal
Pd3Zr1_ICSD_185643	metal	metal
Pd4Pu3_ICSD_2516	metal	metal
Pd4S1_ICSD_648748	metal	metal
Pd4Se1_ICSD_23864	metal	metal
Pd4Se6Tl2_ICSD_78786	insulator	metal
Pd4Sn16_ICSD_413280	metal	metal
Pd4Tb3_ICSD_648980	metal	metal
Pd4Te1_ICSD_648990	metal	metal
Pd4Th3_ICSD_649031	metal	metal
Pd4Y3_ICSD_649109	metal	metal
Pd4Yb3_ICSD_649127	metal	metal
Pd4Zr3_ICSD_186415	metal	metal
Pd5Sb2_ICSD_648767	metal	metal
Pd5Se1Tl1_ICSD_648842	metal	metal
Pd5Se1Zn1_ICSD_648846	metal	metal
Pd5Sr1_ICSD_105707	metal	metal
Pd5Th3_ICSD_105719	metal	metal
Pd5Ti3_ICSD_105726	metal	metal

Supplementary Table 573. Five-fold cross validated predictions for the metal/insulator classification (565/598).

system	calculated	predicted
Pd5Ti3_ICSD_167651	metal	metal
Pd7Se2_ICSD_65052	metal	metal
Pd7Se4_ICSD_648822	metal	metal
Pd8Sn24_ICSD_413279	metal	metal
Pd9Te4_ICSD_649011	metal	metal
Pr1Pt1Sb1_ICSD_649208	metal	metal
Pr1Pt1Sn1_ICSD_416898	metal	metal
Pr1Pt1Sn1_ICSD_416900	metal	metal
Pr1Pt1_ICSD_108760	metal	metal
Pr1Pt1_ICSD_649190	metal	metal
Pr1Pt1_ICSD_649192	metal	metal
Pr1Pt2Si2_ICSD_649210	metal	metal
Pr1Pt2_ICSD_108683	metal	metal
Pr1Pt2_ICSD_649199	metal	metal
Pr1Pt3_ICSD_108684	metal	metal
Pr1Pt5_ICSD_108685	metal	metal
Pr1Rb1S2_ICSD_81396	insulator	insulator
Pr1Rb1Se2_ICSD_281069	insulator	insulator
Pr1Rb1Te4_ICSD_412794	metal	insulator
Pr1Re2_ICSD_108688	metal	metal
Pr1Re2_ICSD_649213	metal	metal
Pr1Re4Si2_ICSD_649216	metal	metal
Pr1Rh1Si2_ICSD_603334	metal	metal
Pr1Rh1Sn1_ICSD_54339	metal	metal
Pr1Rh1_ICSD_649221	metal	metal
Pr1Rh2Si2_ICSD_162012	metal	metal
Pr1Rh2Si2_ICSD_162015	metal	metal
Pr1Rh2_ICSD_108689	metal	metal
Pr1Rh2_ICSD_649223	metal	metal
Pr1Ru1Si1_ICSD_41253	metal	metal
Pr1Ru1Sn1_ICSD_419477	metal	metal
Pr1Ru1Sn3_ICSD_601227	metal	metal
Pr1Ru2Si2_ICSD_55777	metal	metal
Pr1Ru2_ICSD_649234	metal	metal
Pr1Ru4Sb12_ICSD_649242	metal	metal
Pr1S1_ICSD_649270	metal	metal
Pr1S2_ICSD_418406	insulator	insulator
Pr1S2_ICSD_656241	metal	metal
Pr1S6Y3_ICSD_649298	insulator	insulator
Pr1Sb1Te1_ICSD_601232	metal	metal
Pr1Sb1_ICSD_649306	metal	metal
Pr1Sb1_ICSD_92166	metal	metal
Pr1Sc1Si1_ICSD_649322	metal	metal
Pr1Se1_ICSD_77934	metal	metal
Pr1Se2Tl1_ICSD_649354	metal	insulator
Pr1Se2_ICSD_649341	metal	metal
Pr1Se3Yb1_ICSD_99667	metal	metal
Pr1Si1_ICSD_42945	metal	metal
Pr1Si2_ICSD_649376	metal	metal
Pr1Si2_ICSD_658012	metal	metal

Supplementary Table 574. Five-fold cross validated predictions for the metal/insulator classification (566/598).

system	calculated	predicted
Pr1Sn1Zn1_ICSD_152625	metal	metal
Pr1Sn3_ICSD_649396	metal	metal
Pr1Te1_ICSD_649417	metal	metal
Pr1Te1_ICSD_77936	metal	metal
Pr1Te2Tl1_ICSD_649434	metal	insulator
Pr1Te2_ICSD_649409	metal	metal
Pr1Te2_ICSD_649420	metal	metal
Pr1Tl1_ICSD_108699	metal	metal
Pr1Tl3_ICSD_108700	metal	metal
Pr1Zn11_ICSD_649470	metal	metal
Pr1Zn11_ICSD_649486	insulator	metal
Pr1Zn1_ICSD_108707	metal	metal
Pr1Zn1_ICSD_649465	metal	metal
Pr1Zn2_ICSD_649462	metal	metal
Pr1Zn2_ICSD_649482	metal	metal
Pr1Zn3_ICSD_649473	metal	metal
Pr1Zn5_ICSD_108708	metal	metal
Pr1_ICSD_164283	metal	metal
Pr1_ICSD_43570	metal	metal
Pr1_ICSD_43576	metal	metal
Pr1_ICSD_54338	metal	metal
Pr1_ICSD_649178	metal	metal
Pr1_ICSD_649185	metal	metal
Pr1_ICSD_74984	metal	metal
Pr1_ICSD_77904	metal	metal
Pr2Pt3Si5_ICSD_184982	metal	metal
Pr2S3_ICSD_83383	insulator	insulator
Pr2S5Sn1_ICSD_249665	metal	metal
Pr2Tl1_ICSD_108701	metal	metal
Pr2Zn17_ICSD_104241	metal	metal
Pr2Zn17_ICSD_649469	metal	metal
Pr3Pt23Si11_ICSD_187875	metal	metal
Pr3Pt3Sb4_ICSD_649207	metal	metal
Pr3Pt4_ICSD_108686	metal	metal
Pr3Rh4Sn13_ICSD_102352	metal	metal
Pr3S4_ICSD_649249	metal	metal
Pr3S4_ICSD_649275	metal	metal
Pr3Se4_ICSD_649347	metal	metal
Pr3Sn1_ICSD_649395	metal	metal
Pr3Te4_ICSD_649411	metal	metal
Pr3Te4_ICSD_649426	metal	metal
Pr3Tl1_ICSD_649452	metal	metal
Pr3Tl5_ICSD_649450	metal	metal
Pr4Sb3_ICSD_649308	insulator	metal
Pr5Sb3_ICSD_649307	metal	metal
Pt11Zr9_ICSD_105859	metal	metal
Pt12Si5_ICSD_649606	metal	metal
Pt12Si5_ICSD_77977	metal	metal
Pt1Pu1_ICSD_649512	metal	metal
Pt1Pu2_ICSD_2256	metal	metal

Supplementary Table 575. Five-fold cross validated predictions for the metal/insulator classification (567/598).

system	calculated	predicted
Pt1Rb2S2_ICSD_26259	insulator	insulator
Pt1Rb2Se2_ICSD_40431	insulator	insulator
Pt1Rb2Te2_ICSD_649525	insulator	insulator
Pt1S1Sb1_ICSD_649543	metal	insulator
Pt1S1_ICSD_31131	insulator	insulator
Pt1S1_ICSD_649539	insulator	insulator
Pt1S2_ICSD_603737	insulator	insulator
Pt1S2_ICSD_659963	insulator	insulator
Pt1Sb1Sc1_ICSD_77948	insulator	metal
Pt1Sb1Se1_ICSD_649563	metal	metal
Pt1Sb1Si1_ICSD_413194	metal	metal
Pt1Sb1Sr1_ICSD_59185	metal	metal
Pt1Sb1Tb1_ICSD_77950	insulator	metal
Pt1Sb1Tm1_ICSD_77951	metal	metal
Pt1Sb1Y1_ICSD_44970	insulator	metal
Pt1Sb1Yb1_ICSD_77953	metal	metal
Pt1Sb1_ICSD_649561	metal	metal
Pt1Sb2Zr6_ICSD_157366	metal	metal
Pt1Sb2_ICSD_77946	metal	metal
Pt1Sc1Si1_ICSD_79597	metal	metal
Pt1Sc1Sn1_ICSD_108710	metal	metal
Pt1Sc1Sn1_ICSD_411822	metal	metal
Pt1Sc1Sn1_ICSD_649587	metal	metal
Pt1Sc1Zn1_ICSD_424600	metal	metal
Pt1Sc1_ICSD_649585	metal	metal
Pt1Sc2_ICSD_105785	metal	metal
Pt1Sc3Si3_ICSD_421257	metal	metal
Pt1Se1Si1_ICSD_649595	insulator	insulator
Pt1Se2_ICSD_649589	metal	metal
Pt1Si1Sm1_ICSD_649618	metal	metal
Pt1Si1Sr1_ICSD_604437	metal	metal
Pt1Si1Tb1_ICSD_90291	metal	metal
Pt1Si1Te1_ICSD_400487	insulator	metal
Pt1Si1Ti1_ICSD_260794	metal	metal
Pt1Si1Y1_ICSD_649647	metal	metal
Pt1Si1Yb1_ICSD_649653	metal	metal
Pt1Si1Zr1_ICSD_649654	metal	metal
Pt1Si1_ICSD_2623	metal	metal
Pt1Si1_ICSD_649603	metal	metal
Pt1Si1_ICSD_76627	insulator	metal
Pt1Sm1Sn1_ICSD_416897	metal	metal
Pt1Sm1Sn1_ICSD_416899	metal	metal
Pt1Sm1_ICSD_649657	metal	metal
Pt1Sn1Sr1_ICSD_412012	metal	metal
Pt1Sn1Tb1_ICSD_417533	metal	metal
Pt1Sn1Tb1_ICSD_649683	metal	metal
Pt1Sn1Th1_ICSD_108712	insulator	metal
Pt1Sn1Ti1_ICSD_105799	insulator	metal
Pt1Sn1U1_ICSD_105800	metal	metal
Pt1Sn1U1_ICSD_649699	metal	metal

Supplementary Table 576. Five-fold cross validated predictions for the metal/insulator classification (568/598).

system	calculated	predicted
Pt1Sn1Y1_ICSD_417531	metal	metal
Pt1Sn1Y1_ICSD_649700	metal	metal
Pt1Sn1Yb1_ICSD_410418	metal	metal
Pt1Sn1_ICSD_42593	metal	metal
Pt1Sn2_ICSD_649679	metal	metal
Pt1Sn4_ICSD_105793	metal	metal
Pt1Ta1Te5_ICSD_71292	metal	metal
Pt1Tb1_ICSD_10511	metal	metal
Pt1Tb1_ICSD_649729	metal	metal
Pt1Tb2_ICSD_105806	metal	metal
Pt1Tb3_ICSD_649727	metal	metal
Pt1Te1_ICSD_105812	metal	metal
Pt1Te1_ICSD_41383	metal	metal
Pt1Te2_ICSD_41385	metal	metal
Pt1Th1_ICSD_649759	metal	metal
Pt1Ti1_ICSD_105814	metal	metal
Pt1Ti1_ICSD_649765	metal	metal
Pt1Ti3_ICSD_105816	metal	metal
Pt1Tl1_ICSD_105819	metal	metal
Pt1Tl2_ICSD_649780	metal	metal
Pt1Tl3_ICSD_186644	metal	metal
Pt1Tm2_ICSD_649787	metal	metal
Pt1U1_ICSD_649811	metal	metal
Pt1U1_ICSD_649818	metal	metal
Pt1V1_ICSD_649822	metal	metal
Pt1V1_ICSD_649839	metal	metal
Pt1V3_ICSD_105836	metal	metal
Pt1V3_ICSD_649821	metal	metal
Pt1Y1_ICSD_649850	metal	metal
Pt1Y2_ICSD_649844	metal	metal
Pt1Y3_ICSD_8016	metal	metal
Pt1Yb1Zn1_ICSD_159305	metal	metal
Pt1Yb1_ICSD_649871	insulator	metal
Pt1Yb2_ICSD_649873	metal	metal
Pt1Zn1_ICSD_649882	metal	metal
Pt1Zr1_ICSD_105857	metal	metal
Pt1Zr1_ICSD_649884	metal	metal
Pt1Zr2_ICSD_649886	metal	metal
Pt1_ICSD_41525	metal	metal
Pt1_ICSD_76153	metal	metal
Pt23Si11U3_ICSD_154517	metal	metal
Pt23Si11Yb3_ICSD_182155	metal	metal
Pt2Pu1Si2_ICSD_604296	metal	metal
Pt2Pu1_ICSD_105773	insulator	metal
Pt2Pu2Sn1_ICSD_107351	metal	metal
Pt2Sc1Sn1_ICSD_105788	metal	metal
Pt2Si1Tb1_ICSD_649624	metal	metal
Pt2Si1Y1_ICSD_649645	metal	metal
Pt2Si1Yb1_ICSD_247423	metal	metal
Pt2Si1_ICSD_649608	metal	metal

Supplementary Table 577. Five-fold cross validated predictions for the metal/insulator classification (569/598).

system	calculated	predicted
Pt2Si1_ICSD_649616	metal	metal
Pt2Si2Sm1_ICSD_601234	metal	metal
Pt2Si2Sm1_ICSD_649619	metal	metal
Pt2Si2Tb1_ICSD_649626	metal	metal
Pt2Si2Th1_ICSD_649630	metal	metal
Pt2Si2Th1_ICSD_77979	metal	metal
Pt2Si2U1_ICSD_167712	metal	metal
Pt2Si2U1_ICSD_77980	metal	metal
Pt2Si2Y1_ICSD_601235	metal	metal
Pt2Si2Y1_ICSD_649646	metal	metal
Pt2Si2Yb1_ICSD_77981	metal	metal
Pt2Si3_ICSD_77974	metal	metal
Pt2Sm1_ICSD_649661	metal	metal
Pt2Sn1Tb1_ICSD_649684	metal	metal
Pt2Sn1U1_ICSD_649696	metal	metal
Pt2Sn1U2_ICSD_602785	metal	metal
Pt2Sn1Y1_ICSD_649701	metal	metal
Pt2Sn3_ICSD_649674	metal	metal
Pt2Sr1_ICSD_108148	metal	metal
Pt2Sr3_ICSD_649707	metal	metal
Pt2Ta1_ICSD_105802	metal	metal
Pt2Tb1_ICSD_105807	metal	metal
Pt2Te3_ICSD_41371	metal	metal
Pt2Tm1_ICSD_105823	metal	metal
Pt2U1_ICSD_105832	metal	metal
Pt2V1_ICSD_649828	metal	metal
Pt2W1_ICSD_649841	metal	metal
Pt2Y1_ICSD_105844	metal	metal
Pt2Yb1_ICSD_649863	metal	metal
Pt2Yb5_ICSD_649870	metal	metal
Pt3Pu1_ICSD_649503	metal	metal
Pt3Pu5_ICSD_105776	metal	metal
Pt3Pu5_ICSD_1917	metal	metal
Pt3Rb2S4_ICSD_26267	insulator	insulator
Pt3Rb2S6U1_ICSD_262560	insulator	insulator
Pt3Rb2Se6U1_ICSD_262559	insulator	insulator
Pt3S8Ta2_ICSD_108849	insulator	insulator
Pt3Sb1_ICSD_649557	metal	metal
Pt3Sb2_ICSD_42752	metal	metal
Pt3Sb4U3_ICSD_649575	insulator	metal
Pt3Sc1_ICSD_105787	metal	metal
Pt3Sc2Si2_ICSD_247425	metal	metal
Pt3Se8Ta2_ICSD_77955	insulator	insulator
Pt3Si1_ICSD_246170	metal	metal
Pt3Si1_ICSD_649613	metal	metal
Pt3Si1_ICSD_77975	metal	metal
Pt3Sm1_ICSD_105790	metal	metal
Pt3Sn1_ICSD_105795	metal	metal
Pt3Sn4U3_ICSD_649697	metal	metal
Pt3Ta1_ICSD_105803	metal	metal

Supplementary Table 578. Five-fold cross validated predictions for the metal/insulator classification (570/598).

system	calculated	predicted
Pt3Tb1_ICSD_649732	metal	metal
Pt3Tb5_ICSD_649721	metal	metal
Pt3Te4_ICSD_41372	metal	metal
Pt3Te4_ICSD_77983	metal	metal
Pt3Te4_ICSD_87397	metal	metal
Pt3Th7_ICSD_150671	metal	metal
Pt3Ti1_ICSD_185640	metal	metal
Pt3Ti1_ICSD_649772	metal	metal
Pt3Tl2_ICSD_105821	metal	metal
Pt3Tm1_ICSD_105824	metal	metal
Pt3Tm1_ICSD_105825	metal	metal
Pt3Tm1_ICSD_649796	metal	metal
Pt3Tm5_ICSD_649785	metal	metal
Pt3U1_ICSD_649804	metal	metal
Pt3V1_ICSD_105840	metal	metal
Pt3V1_ICSD_105841	metal	metal
Pt3Y1_ICSD_649857	metal	metal
Pt3Y5_ICSD_649842	metal	metal
Pt3Y7_ICSD_649860	metal	metal
Pt3Yb1_ICSD_649864	metal	metal
Pt3Yb5_ICSD_649868	metal	metal
Pt3Zn1_ICSD_105853	metal	metal
Pt3Zr1_ICSD_185646	metal	metal
Pt3Zr1_ICSD_649888	metal	metal
Pt3Zr5_ICSD_391476	metal	metal
Pt4Rb2Se6_ICSD_69439	insulator	insulator
Pt4S6Tl2_ICSD_78784	insulator	insulator
Pt4Se6Tl2_ICSD_78785	insulator	metal
Pt4Tb3_ICSD_649735	metal	metal
Pt4Te6Tl2_ICSD_78787	metal	metal
Pt4Y5_ICSD_2422	metal	metal
Pt4Yb3_ICSD_105851	metal	metal
Pt5Pu1_ICSD_23043	metal	metal
Pt5Pu1_ICSD_649510	insulator	metal
Pt5S6Tl2_ICSD_73026	metal	insulator
Pt5Sb1Si1_ICSD_649564	metal	metal
Pt5Se4_ICSD_87926	metal	metal
Pt5Si1Tl1_ICSD_649632	metal	metal
Pt5Sr1_ICSD_105801	metal	metal
Pt5Th3_ICSD_649755	metal	metal
Pt5Ti3_ICSD_105817	metal	metal
Pt5U1_ICSD_649806	metal	metal
Pt6Si5_ICSD_649605	metal	metal
Pt7Sb1_ICSD_57471	metal	metal
Pt7Sc4Si2_ICSD_420438	metal	metal
Pt8Sc4Si4_ICSD_420355	metal	metal
Pt8Ti1_ICSD_105818	metal	metal
Pt8V1_ICSD_180978	metal	metal
Pt9Sc5Si7_ICSD_420356	metal	metal
Pu1Re2_ICSD_649899	metal	metal

Supplementary Table 579. Five-fold cross validated predictions for the metal/insulator classification (571/598).

system	calculated	predicted
Pu1Rh2Si2_ICSD_73032	metal	metal
Pu1Rh2_ICSD_649904	metal	metal
Pu1Rh3_ICSD_105861	metal	metal
Pu1Ru1_ICSD_105865	metal	metal
Pu1Ru2Si2_ICSD_73031	metal	metal
Pu1Ru2_ICSD_105866	metal	metal
Pu1S1_ICSD_31709	metal	metal
Pu1S2_ICSD_649932	metal	metal
Pu1Sb1Te1_ICSD_601246	metal	metal
Pu1Sb1_ICSD_57473	metal	metal
Pu1Sb1_ICSD_57474	metal	metal
Pu1Sb1_ICSD_649944	metal	metal
Pu1Sb2_ICSD_649946	metal	metal
Pu1Se1_ICSD_649958	metal	metal
Pu1Se2_ICSD_649957	metal	metal
Pu1Si1_ICSD_109364	metal	metal
Pu1Si1_ICSD_649972	metal	metal
Pu1Si2_ICSD_44867	metal	metal
Pu1Si2_ICSD_649969	metal	metal
Pu1Sn3_ICSD_105874	metal	metal
Pu1Te1_ICSD_649980	metal	metal
Pu1Te1_ICSD_77986	metal	metal
Pu1Te2_ICSD_649988	metal	metal
Pu1Zn2_ICSD_105878	metal	metal
Pu1_ICSD_43709	metal	metal
Pu1_ICSD_44768	metal	metal
Pu1_ICSD_44769	metal	metal
Pu1_ICSD_44866	metal	metal
Pu3Si2_ICSD_649970	metal	metal
Pu3Sn1_ICSD_157518	metal	metal
Pu5Si3_ICSD_16582	metal	metal
Rb1S2Sb1_ICSD_200263	insulator	insulator
Rb1S2Sb1_ICSD_56788	insulator	insulator
Rb1S2Sm1_ICSD_81398	metal	metal
Rb1S2Sn1_ICSD_23449	metal	metal
Rb1S2Sn1_ICSD_650031	metal	metal
Rb1S2Tb1_ICSD_81401	insulator	insulator
Rb1S2Ti1_ICSD_77990	insulator	insulator
Rb1S2Tm1_ICSD_81405	insulator	metal
Rb1S2Yb1_ICSD_81406	metal	metal
Rb1S8Sb1U2_ICSD_87804	insulator	insulator
Rb1S8V5_ICSD_650040	insulator	insulator
Rb1Sb1Se2_ICSD_602244	insulator	insulator
Rb1Sb1_ICSD_14030	insulator	insulator
Rb1Sb2_ICSD_419402	metal	insulator
Rb1Sc5Te8_ICSD_245998	insulator	insulator
Rb1Se2Sm1_ICSD_281071	insulator	metal
Rb1Se2Tb1_ICSD_281073	insulator	insulator
Rb1Se2V1_ICSD_415479	insulator	insulator
Rb1Se8Ti5_ICSD_602323	insulator	insulator

Supplementary Table 580. Five-fold cross validated predictions for the metal/insulator classification (572/598).

system	calculated	predicted
Rb1Se8V5_ICSD_602326	metal	insulator
Rb1Si1_ICSD_43519	insulator	insulator
Rb1Si1_ICSD_650058	insulator	insulator
Rb1Sm1Te2_ICSD_602830	metal	metal
Rb1Sn1_ICSD_650061	insulator	metal
Rb1Te2Y1_ICSD_419996	insulator	insulator
Rb1Te6_ICSD_74837	metal	insulator
Rb1Zn13_ICSD_150476	metal	metal
Rb1_ICSD_109013	metal	metal
Rb1_ICSD_109016	metal	metal
Rb1_ICSD_109017	metal	metal
Rb1_ICSD_109018	metal	metal
Rb1_ICSD_109019	metal	metal
Rb1_ICSD_161381	metal	metal
Rb1_ICSD_248070	metal	metal
Rb1_ICSD_44869	metal	metal
Rb2S1_ICSD_261443	insulator	insulator
Rb2S1_ICSD_261444	insulator	insulator
Rb2S1_ICSD_29208	insulator	insulator
Rb2S2_ICSD_73175	insulator	insulator
Rb2S2_ICSD_73176	insulator	insulator
Rb2S3_ICSD_14092	insulator	insulator
Rb2S4W1_ICSD_281586	insulator	insulator
Rb2S4Zn3_ICSD_602243	insulator	insulator
Rb2S5_ICSD_100321	insulator	insulator
Rb2Sb4Se8_ICSD_402887	insulator	insulator
Rb2Sb4Zn5_ICSD_290263	metal	insulator
Rb2Se1_ICSD_168449	insulator	insulator
Rb2Se2_ICSD_73177	insulator	insulator
Rb2Se3_ICSD_14093	insulator	insulator
Rb2Se4Te1_ICSD_650055	insulator	insulator
Rb2Se4W1_ICSD_650057	insulator	insulator
Rb2Se5Sn2_ICSD_80766	insulator	insulator
Rb2Se5_ICSD_100322	insulator	insulator
Rb2Sn1Te5_ICSD_40556	insulator	insulator
Rb2Te1_ICSD_55116	insulator	insulator
Rb2Te1_ICSD_55128	insulator	insulator
Rb2Te1_ICSD_55153	insulator	insulator
Rb2Te2_ICSD_73179	insulator	insulator
Rb2Te2_ICSD_83350	insulator	insulator
Rb2Te3Zr1_ICSD_410735	insulator	insulator
Rb2Te3_ICSD_77994	insulator	insulator
Rb2Te5_ICSD_30734	insulator	insulator
Rb3Sb1Se3_ICSD_89608	insulator	insulator
Rb3Sb1_ICSD_650044	metal	insulator
Rb4S14Ti3_ICSD_411847	insulator	insulator
Rb4S4Sn1_ICSD_409647	insulator	insulator
Rb4S6Si2_ICSD_409804	insulator	insulator
Rb4Sb4_ICSD_280591	insulator	insulator
Rb4Se6Sn2_ICSD_74844	insulator	insulator

Supplementary Table 581. Five-fold cross validated predictions for the metal/insulator classification (573/598).

system	calculated	predicted
Rb4Te16Zr3_ICSD_280298	insulator	insulator
Rb8Tl11_ICSD_370035	metal	metal
Re0.67Zr1.33_ICSD_650215	metal	metal
Re0.83Zr0.17_ICSD_650213	metal	metal
Re1S2_ICSD_75459	insulator	insulator
Re1S2_ICSD_81814	insulator	insulator
Re1Se2_ICSD_26256	insulator	insulator
Re1Se2_ICSD_650091	metal	insulator
Re1Si1Ta1_ICSD_600060	metal	metal
Re1Si1Ta1_ICSD_650119	metal	metal
Re1Si1Ti1_ICSD_650125	metal	metal
Re1Si1Zr1_ICSD_650139	metal	metal
Re1Si1_ICSD_26616	metal	metal
Re1Si2_ICSD_108721	metal	metal
Re1Ti1_ICSD_105896	metal	metal
Re1Ti2_ICSD_168953	metal	metal
Re1_ICSD_41522	metal	metal
Re1_ICSD_64719	metal	metal
Re24Sc5_ICSD_105891	metal	metal
Re24Ti5_ICSD_650177	metal	metal
Re2Sc1Si3_ICSD_41742	metal	metal
Re2Sc1_ICSD_650085	metal	metal
Re2Si1_ICSD_57480	metal	metal
Re2Sm1_ICSD_650141	metal	metal
Re2Tb1_ICSD_650153	metal	metal
Re2Th1_ICSD_650166	metal	metal
Re2U1_ICSD_650184	metal	metal
Re2W1_ICSD_650203	metal	metal
Re2Y1_ICSD_650202	metal	metal
Re2Yb1_ICSD_650204	metal	metal
Re2Zr1_ICSD_650212	metal	metal
Re3Ru1_ICSD_168956	metal	metal
Re3Sc2Si4_ICSD_10043	metal	metal
Re3Si5U2_ICSD_650129	metal	metal
Re4S4Te4_ICSD_82721	insulator	insulator
Re4Si2Th1_ICSD_402015	metal	metal
Re4Si2Y1_ICSD_41760	metal	metal
Re4Si7_ICSD_151529	insulator	metal
Re5Si3_ICSD_650114	metal	metal
Re7Si6U4_ICSD_2471	metal	metal
Rh17S15_ICSD_410838	metal	metal
Rh1S1Sb1_ICSD_650233	insulator	insulator
Rh1S2_ICSD_105914	metal	metal
Rh1S2_ICSD_650232	metal	metal
Rh1S6Ta3_ICSD_650235	metal	metal
Rh1Sb1Se1_ICSD_650254	insulator	insulator
Rh1Sb1Tb1_ICSD_51842	metal	metal
Rh1Sb1Th1_ICSD_52067	insulator	metal
Rh1Sb1U1_ICSD_52068	metal	metal
Rh1Sb1_ICSD_76621	metal	metal

Supplementary Table 582. Five-fold cross validated predictions for the metal/insulator classification (574/598).

system	calculated	predicted
Rh1Sb1_ICSD_991	metal	metal
Rh1Sb2Ti5_ICSD_95842	metal	metal
Rh1Sb2_ICSD_650241	metal	metal
Rh1Sb3_ICSD_34049	metal	metal
Rh1Sc1Si1_ICSD_79596	metal	metal
Rh1Sc1Si2_ICSD_15248	metal	metal
Rh1Sc1_ICSD_105919	metal	metal
Rh1Sc3Si3_ICSD_421255	metal	metal
Rh1Sc6Te2_ICSD_98971	metal	metal
Rh1Se1_ICSD_52069	metal	metal
Rh1Se2_ICSD_650276	metal	metal
Rh1Se2_ICSD_650286	metal	metal
Rh1Si1Ta1_ICSD_90435	metal	metal
Rh1Si1Tb1_ICSD_52075	metal	metal
Rh1Si1Ti1_ICSD_108723	metal	metal
Rh1Si1U1_ICSD_50083	metal	metal
Rh1Si1Y1_ICSD_32562	metal	metal
Rh1Si1Zr1_ICSD_650356	metal	metal
Rh1Si1_ICSD_182505	metal	metal
Rh1Si1_ICSD_44385	metal	metal
Rh1Si1_ICSD_650297	metal	metal
Rh1Si1_ICSD_653588	metal	metal
Rh1Si2Y2_ICSD_48176	metal	metal
Rh1Si3Sm1_ICSD_650313	metal	metal
Rh1Si3Tb1_ICSD_650325	metal	metal
Rh1Si3Tb2_ICSD_650328	metal	metal
Rh1Si3Th1_ICSD_650335	metal	metal
Rh1Si3Y2_ICSD_650353	metal	metal
Rh1Si3Y3_ICSD_52079	metal	metal
Rh1Sm1Sn1_ICSD_650372	metal	metal
Rh1Sm1Zn1_ICSD_419894	metal	metal
Rh1Sm1_ICSD_650367	metal	metal
Rh1Sn1Tb1_ICSD_650391	metal	metal
Rh1Sn1Th1_ICSD_650394	metal	metal
Rh1Sn1Tm1_ICSD_411824	metal	metal
Rh1Sn1U1_ICSD_650402	metal	metal
Rh1Sn1Y1_ICSD_650406	metal	metal
Rh1Sn1Yb1_ICSD_650407	metal	metal
Rh1Sn1Zr1_ICSD_152133	metal	metal
Rh1Sn1Zr1_ICSD_650412	metal	metal
Rh1Sn1_ICSD_650381	metal	metal
Rh1Sn2_ICSD_650384	metal	metal
Rh1Sn2_ICSD_650385	metal	metal
Rh1Ta1Te4_ICSD_656453	metal	metal
Rh1Tb1Zn1_ICSD_419896	metal	metal
Rh1Tb1_ICSD_105942	metal	metal
Rh1Tb3_ICSD_650435	metal	metal
Rh1Te1_ICSD_26617	metal	metal
Rh1Te2_ICSD_650448	metal	metal
Rh1Te2_ICSD_650452	metal	metal

Supplementary Table 583. Five-fold cross validated predictions for the metal/insulator classification (575/598).

system	calculated	predicted
Rh1Th1_ICSD_650471	metal	metal
Rh1Ti1_ICSD_105952	metal	metal
Rh1Ti1_ICSD_105953	metal	metal
Rh1Ti2_ICSD_650481	metal	metal
Rh1Tm1_ICSD_105959	metal	metal
Rh1V1_ICSD_169385	metal	metal
Rh1V1_ICSD_650512	metal	metal
Rh1V3_ICSD_650522	metal	metal
Rh1Y1_ICSD_650541	metal	metal
Rh1Y3_ICSD_650529	metal	metal
Rh1Yb1_ICSD_108726	metal	metal
Rh1Zn13_ICSD_107575	metal	metal
Rh1Zn1_ICSD_107574	metal	metal
Rh1Zr1_ICSD_105979	metal	metal
Rh1Zr1_ICSD_105981	metal	metal
Rh1Zr1_ICSD_657955	metal	metal
Rh1Zr2_ICSD_650562	metal	metal
Rh1Zr3_ICSD_650551	metal	metal
Rh1_ICSD_41516	metal	metal
Rh1_ICSD_53811	metal	metal
Rh2S3_ICSD_15344	insulator	insulator
Rh2Sb1_ICSD_42960	metal	metal
Rh2Si1Y1_ICSD_61381	metal	metal
Rh2Si1_ICSD_650300	metal	metal
Rh2Si2Sm1_ICSD_602245	metal	metal
Rh2Si2Sm1_ICSD_650312	metal	metal
Rh2Si2Tb1_ICSD_650331	metal	metal
Rh2Si2Th1_ICSD_650337	metal	metal
Rh2Si2Tm1_ICSD_62725	metal	metal
Rh2Si2U1_ICSD_657191	metal	metal
Rh2Si2Y1_ICSD_602257	metal	metal
Rh2Si2Y3_ICSD_30725	metal	metal
Rh2Si2Yb1_ICSD_52083	metal	metal
Rh2Si3Y3_ICSD_650344	metal	metal
Rh2Sm1_ICSD_105927	metal	metal
Rh2Sn1U2_ICSD_246630	metal	metal
Rh2Sn1V1_ICSD_105933	metal	metal
Rh2Sn1V1_ICSD_105934	metal	metal
Rh2Sn1_ICSD_105931	metal	metal
Rh2Sr1_ICSD_105937	metal	metal
Rh2Ta1_ICSD_650415	metal	metal
Rh2Tb1_ICSD_650430	metal	metal
Rh2Th1_ICSD_105948	metal	metal
Rh2U1Zn20_ICSD_183635	metal	metal
Rh2Y1_ICSD_650538	metal	metal
Rh2Yb1_ICSD_650546	metal	metal
Rh2Zn11_ICSD_107576	metal	metal
Rh3S2Sn2_ICSD_420728	metal	metal
Rh3S2Tl2_ICSD_650237	metal	metal
Rh3S4_ICSD_410813	metal	insulator

Supplementary Table 584. Five-fold cross validated predictions for the metal/insulator classification (576/598).

system	calculated	predicted
Rh3Sb4U3_ICSD_164407	metal	metal
Rh3Sc1Si7_ICSD_15243	metal	metal
Rh3Sc1_ICSD_650268	metal	metal
Rh3Se8_ICSD_650285	metal	metal
Rh3Si2Sm1_ICSD_52074	metal	metal
Rh3Si2Y1_ICSD_52080	metal	metal
Rh3Si2Y1_ICSD_650345	metal	metal
Rh3Si4_ICSD_24354	metal	metal
Rh3Si5Tb2_ICSD_154034	metal	metal
Rh3Si5Y2_ICSD_40760	metal	metal
Rh3Ta1_ICSD_105939	metal	metal
Rh3Tb5_ICSD_650433	metal	metal
Rh3Tb7_ICSD_650429	metal	metal
Rh3Te2_ICSD_650451	metal	metal
Rh3Te8_ICSD_2186	metal	metal
Rh3Th1_ICSD_650470	metal	metal
Rh3Th7_ICSD_650458	metal	metal
Rh3Ti1_ICSD_650482	metal	metal
Rh3U1_ICSD_650501	metal	metal
Rh3V1_ICSD_105966	metal	metal
Rh3W1_ICSD_105970	metal	metal
Rh3Y1_ICSD_650532	metal	metal
Rh3Zr1_ICSD_650565	metal	metal
Rh4Sc5Si10_ICSD_650272	metal	metal
Rh4Si5_ICSD_24355	metal	metal
Rh4Sn13Sr3_ICSD_650389	metal	metal
Rh4Sn13Th3_ICSD_102356	metal	metal
Rh4Sn13U3_ICSD_54383	metal	metal
Rh4Sn13Yb3_ICSD_105936	metal	metal
Rh5Si3_ICSD_43338	metal	metal
Rh5Tb1_ICSD_105944	metal	metal
Rh5Ti3_ICSD_105957	metal	metal
Rh5Y1_ICSD_105977	metal	metal
Rh5Zr3_ICSD_62932	metal	metal
Rh7Sb6Yb4_ICSD_409885	metal	metal
Ru1S1Sb1_ICSD_650583	insulator	insulator
Ru1S2_ICSD_56019	insulator	metal
Ru1Sb1Se1_ICSD_650594	insulator	insulator
Ru1Sb1Ta1_ICSD_107123	insulator	metal
Ru1Sb1Te1_ICSD_650595	insulator	insulator
Ru1Sb1Ti1_ICSD_107127	metal	insulator
Ru1Sb1U1_ICSD_650597	metal	metal
Ru1Sb1V1_ICSD_107124	insulator	metal
Ru1Sb1Zr1_ICSD_107126	metal	metal
Ru1Sb1_ICSD_990	metal	metal
Ru1Sb2U1_ICSD_50351	metal	metal
Ru1Sb2_ICSD_42608	insulator	metal
Ru1Sc1Si1_ICSD_420417	metal	metal
Ru1Sc1Si1_ICSD_650603	metal	metal
Ru1Sc1_ICSD_105988	metal	metal

Supplementary Table 585. Five-fold cross validated predictions for the metal/insulator classification (577/598).

system	calculated	predicted
Ru1Sc2Si2_ICSD_420133	metal	metal
Ru1Sc3Si3_ICSD_421254	metal	metal
Ru1Se2_ICSD_68473	insulator	insulator
Ru1Si1Sm1_ICSD_41254	metal	metal
Ru1Si1Ta1_ICSD_600058	metal	metal
Ru1Si1Tb1_ICSD_88064	metal	metal
Ru1Si1Ti1_ICSD_52090	metal	metal
Ru1Si1Ti1_ICSD_57484	metal	metal
Ru1Si1U1_ICSD_658289	metal	metal
Ru1Si1Y1_ICSD_88063	metal	metal
Ru1Si1Zr1_ICSD_16306	metal	metal
Ru1Si1_ICSD_650618	insulator	metal
Ru1Si1_ICSD_650627	metal	metal
Ru1Si2_ICSD_154012	insulator	insulator
Ru1Si2_ICSD_154013	metal	metal
Ru1Si2_ICSD_154014	metal	metal
Ru1Si3Th1_ICSD_650644	metal	metal
Ru1Sm1Sn3_ICSD_657115	metal	metal
Ru1Sn1U1_ICSD_158284	metal	metal
Ru1Sn2_ICSD_105993	metal	metal
Ru1Ta1Te4_ICSD_656452	metal	metal
Ru1Ta1_ICSD_650690	metal	metal
Ru1Ta1_ICSD_650692	metal	metal
Ru1Tb3_ICSD_650703	metal	metal
Ru1Te2_ICSD_650713	insulator	insulator
Ru1Te2_ICSD_659137	insulator	metal
Ru1Th1_ICSD_150820	metal	metal
Ru1Ti1_ICSD_106005	metal	metal
Ru1Tm3_ICSD_650744	metal	metal
Ru1V1_ICSD_106011	metal	metal
Ru1V3Zr2_ICSD_650765	metal	metal
Ru1Y3_ICSD_650772	metal	metal
Ru1Yb1_ICSD_108728	metal	metal
Ru1Zn3_ICSD_416747	metal	metal
Ru1Zr1_ICSD_181289	metal	metal
Ru1_ICSD_41515	metal	metal
Ru1_ICSD_44615	metal	metal
Ru2Sb2Sr1_ICSD_188979	metal	metal
Ru2Sc1_ICSD_650601	metal	metal
Ru2Si1_ICSD_650630	metal	metal
Ru2Si2Sm1_ICSD_55353	metal	metal
Ru2Si2Tb1_ICSD_52089	metal	metal
Ru2Si2Th1_ICSD_603984	metal	metal
Ru2Si2U1_ICSD_657192	metal	metal
Ru2Si2Y1_ICSD_650661	metal	metal
Ru2Si2Yb1_ICSD_55357	metal	metal
Ru2Si3_ICSD_2344	insulator	insulator
Ru2Si3_ICSD_95587	insulator	insulator
Ru2Sm1_ICSD_105991	metal	metal
Ru2Sm1_ICSD_650679	metal	metal

Supplementary Table 586. Five-fold cross validated predictions for the metal/insulator classification (578/598).

system	calculated	predicted
Ru2Sn1U2_ICSD_107368	metal	metal
Ru2Sn2Zn3_ICSD_421663	metal	metal
Ru2Sn3_ICSD_105994	metal	metal
Ru2Tb1_ICSD_650706	metal	metal
Ru2Tb5_ICSD_650702	metal	metal
Ru2Th1_ICSD_106002	metal	metal
Ru2U1Zn20_ICSD_183634	metal	metal
Ru2Y1Zn20_ICSD_152110	metal	metal
Ru2Y1_ICSD_650775	metal	metal
Ru2Y5_ICSD_650770	metal	metal
Ru2Yb1_ICSD_650783	metal	metal
Ru2Zr1_ICSD_650790	metal	metal
Ru3Si2Th1_ICSD_650643	metal	metal
Ru3Si2Y1_ICSD_650662	metal	metal
Ru3Si5Tb2_ICSD_57487	metal	metal
Ru3Sn7_ICSD_150763	metal	metal
Ru3Th7_ICSD_150668	metal	metal
Ru3Ti1_ICSD_185635	metal	metal
Ru3U1_ICSD_650750	metal	metal
Ru3Zr1_ICSD_185641	metal	metal
Ru4Sb12Sr1_ICSD_42962	metal	metal
Ru4Sc11_ICSD_650599	metal	metal
Ru4Si3_ICSD_650617	metal	metal
Ru4Si3_ICSD_650632	metal	metal
Ru4Sn6Y1_ICSD_54354	metal	metal
Ru5Si3_ICSD_650615	metal	metal
Ru9Sb8Zn7_ICSD_261057	metal	metal
S10_ICSD_38263	insulator	insulator
S11Ta2Tl4_ICSD_411603	insulator	insulator
S12_ICSD_26463	insulator	insulator
S14_ICSD_408504	insulator	insulator
S16_ICSD_38264	insulator	insulator
S17Sc1U8_ICSD_650847	metal	insulator
S18_ICSD_6002	insulator	insulator
S1Sb1U1_ICSD_650831	metal	metal
S1Sc1_ICSD_44971	metal	metal
S1Se1U1_ICSD_78584	metal	insulator
S1Se1_ICSD_62195	metal	metal
S1Si1Th1_ICSD_650891	metal	metal
S1Si1Zr1_ICSD_15569	metal	metal
S1Sm1_ICSD_650946	metal	metal
S1Sm1_ICSD_650950	metal	metal
S1Sn1_ICSD_30271	metal	metal
S1Sn1_ICSD_43409	insulator	insulator
S1Sn1_ICSD_52109	insulator	insulator
S1Sn1_ICSD_650998	metal	metal
S1Sn1_ICSD_651004	insulator	insulator
S1Sn1_ICSD_651015	insulator	insulator
S1Sn1_ICSD_67442	metal	metal
S1Sr1_ICSD_28900	insulator	insulator

Supplementary Table 587. Five-fold cross validated predictions for the metal/insulator classification (579/598).

system	calculated	predicted
S1Sr1_ICSD_52111	insulator	insulator
S1Ta1_ICSD_52114	metal	metal
S1Ta2_ICSD_26184	metal	metal
S1Ta6_ICSD_16041	metal	metal
S1Ta6_ICSD_202564	metal	metal
S1Tb1_ICSD_52435	metal	metal
S1Te1U1_ICSD_78585	metal	metal
S1Te1_ICSD_62197	metal	metal
S1Th1_ICSD_651158	metal	metal
S1Ti1_ICSD_25561	metal	metal
S1Ti1_ICSD_52121	metal	metal
S1Ti1_ICSD_52193	metal	metal
S1Ti1_ICSD_651179	metal	metal
S1Ti1_ICSD_651197	metal	metal
S1Ti2_ICSD_15319	metal	metal
S1Tl1_ICSD_52201	metal	metal
S1Tl1_ICSD_651241	metal	insulator
S1Tl2_ICSD_42550	insulator	insulator
S1Tm1_ICSD_106032	metal	metal
S1Tm1_ICSD_52204	metal	metal
S1Tm1_ICSD_603120	metal	metal
S1Tm1_ICSD_651281	metal	metal
S1U1_ICSD_60274	metal	metal
S1V1_ICSD_52211	insulator	metal
S1V1_ICSD_602567	insulator	metal
S1V1_ICSD_651373	metal	insulator
S1V3_ICSD_26516	metal	metal
S1V3_ICSD_651364	metal	metal
S1V4Zr9_ICSD_81823	metal	metal
S1Y1_ICSD_44973	metal	metal
S1Yb1_ICSD_651425	insulator	metal
S1Zn1_ICSD_107132	insulator	insulator
S1Zn1_ICSD_107134	insulator	insulator
S1Zn1_ICSD_107137	metal	metal
S1Zn1_ICSD_107150	insulator	insulator
S1Zn1_ICSD_107185	insulator	insulator
S1Zn1_ICSD_107611	insulator	insulator
S1Zn1_ICSD_15477	insulator	insulator
S1Zn1_ICSD_15478	insulator	insulator
S1Zn1_ICSD_15736	insulator	insulator
S1Zn1_ICSD_15737	insulator	insulator
S1Zn1_ICSD_15740	insulator	insulator
S1Zn1_ICSD_15741	insulator	insulator
S1Zn1_ICSD_162754	insulator	insulator
S1Zn1_ICSD_31076	insulator	insulator
S1Zn1_ICSD_37373	insulator	insulator
S1Zn1_ICSD_37374	insulator	insulator
S1Zn1_ICSD_37375	insulator	insulator
S1Zn1_ICSD_37376	insulator	insulator
S1Zn1_ICSD_37391	insulator	insulator

Supplementary Table 588. Five-fold cross validated predictions for the metal/insulator classification (580/598).

system	calculated	predicted
S1Zn1_ICSD_42192	insulator	insulator
S1Zn1_ICSD_42787	insulator	insulator
S1Zn1_ICSD_42792	insulator	insulator
S1Zn1_ICSD_42799	metal	metal
S1Zn1_ICSD_42806	insulator	insulator
S1Zn1_ICSD_42833	insulator	insulator
S1Zn1_ICSD_42854	insulator	insulator
S1Zn1_ICSD_43070	insulator	insulator
S1Zn1_ICSD_76954	insulator	insulator
S1Zr1_ICSD_24754	metal	metal
S1Zr1_ICSD_52224	metal	metal
S1_ICSD_281124	insulator	insulator
S1_ICSD_57164	metal	metal
S1_ICSD_650792	insulator	insulator
S2Sb1Tl1_ICSD_35498	insulator	insulator
S2Sc1Tl1_ICSD_418474	insulator	insulator
S2Si1_ICSD_16952	insulator	insulator
S2Si1_ICSD_26858	metal	insulator
S2Si1_ICSD_27205	insulator	insulator
S2Sm1Tl1_ICSD_650962	insulator	metal
S2Sn0.33Ta1_ICSD_74692	metal	metal
S2Sn1Ta1_ICSD_651033	metal	metal
S2Sn1_ICSD_42566	insulator	insulator
S2Sn1_ICSD_43003	insulator	insulator
S2Sr1_ICSD_642	insulator	insulator
S2Ta1_ICSD_169771	metal	metal
S2Ta1_ICSD_24756	metal	metal
S2Ta1_ICSD_24757	metal	metal
S2Ta1_ICSD_52117	metal	metal
S2Ta1_ICSD_651083	metal	metal
S2Ta1_ICSD_651086	metal	metal
S2Ta1_ICSD_67651	metal	metal
S2Ta3_ICSD_71143	metal	metal
S2Tb1Tl1_ICSD_651135	insulator	insulator
S2Tb1_ICSD_52119	metal	metal
S2Tb1_ICSD_651124	metal	metal
S2Tc1_ICSD_81816	insulator	metal
S2Th1_ICSD_651152	insulator	insulator
S2Ti1_ICSD_174490	insulator	insulator
S2Ti1_ICSD_181501	metal	metal
S2Ti1_ICSD_181502	metal	insulator
S2Ti1_ICSD_181503	insulator	insulator
S2Ti1_ICSD_181504	metal	insulator
S2Ti1_ICSD_181505	insulator	insulator
S2Ti1_ICSD_181506	metal	metal
S2Ti1_ICSD_601182	insulator	insulator
S2Ti1_ICSD_72042	metal	metal
S2Ti1_ICSD_79803	insulator	insulator
S2Tl1Y1_ICSD_651267	insulator	insulator
S2Tl1Yb1_ICSD_57119	metal	insulator

Supplementary Table 589. Five-fold cross validated predictions for the metal/insulator classification (581/598).

system	calculated	predicted
S2Tl2_ICSD_76752	insulator	insulator
S2Tl2_ICSD_78162	insulator	insulator
S2U1_ICSD_50238	metal	metal
S2U1_ICSD_52208	insulator	insulator
S2U1_ICSD_651311	insulator	insulator
S2V1_ICSD_68713	metal	metal
S2V1_ICSD_77832	metal	insulator
S2V1_ICSD_86519	insulator	metal
S2W1_ICSD_202367	insulator	metal
S2W1_ICSD_651387	insulator	insulator
S2Y1_ICSD_52216	metal	metal
S2Y1_ICSD_651404	metal	metal
S2Yb1_ICSD_57493	metal	metal
S2Yb1_ICSD_651423	metal	metal
S2Yb1_ICSD_651433	insulator	metal
S2Zn1_ICSD_651447	insulator	insulator
S2Zr1_ICSD_601166	insulator	insulator
S2Zr9_ICSD_2774	metal	metal
S3Sb1Tl3_ICSD_603664	insulator	insulator
S3Sb2_ICSD_171852	insulator	insulator
S3Sc1U1_ICSD_2239	metal	metal
S3Sc1Y1_ICSD_23422	insulator	insulator
S3Sc2_ICSD_22236	metal	metal
S3Sm2_ICSD_72821	insulator	insulator
S3Sn1Sr1_ICSD_651032	insulator	insulator
S3Sn1Tl2_ICSD_48152	insulator	insulator
S3Sn1Zr1_ICSD_73711	insulator	insulator
S3Sn2Tl2_ICSD_33531	insulator	insulator
S3Sn2_ICSD_31995	insulator	insulator
S3Sr1Ti1_ICSD_651070	metal	metal
S3Sr1Zr1_ICSD_154104	insulator	insulator
S3Sr1_ICSD_23638	insulator	insulator
S3Sr2Zn1_ICSD_421354	insulator	insulator
S3Sr2Zn1_ICSD_422028	insulator	insulator
S3Ta1Tl1_ICSD_412385	insulator	insulator
S3Ta1_ICSD_15251	metal	metal
S3Tb2_ICSD_80767	insulator	insulator
S3Te1Tl2_ICSD_391285	insulator	insulator
S3Th2_ICSD_651154	metal	metal
S3Ti1_ICSD_604398	insulator	insulator
S3Ti1_ICSD_81124	metal	insulator
S3Tl2Zr1_ICSD_186072	insulator	insulator
S3Tl4_ICSD_2647	metal	insulator
S3Tl4_ICSD_651232	insulator	insulator
S3Tm2_ICSD_39240	insulator	insulator
S3U1_ICSD_651344	insulator	insulator
S3U2_ICSD_246850	metal	insulator
S3U4_ICSD_38354	metal	metal
S3Y2_ICSD_651407	insulator	insulator
S3Y2_ICSD_67502	insulator	insulator

Supplementary Table 590. Five-fold cross validated predictions for the metal/insulator classification (582/598).

system	calculated	predicted
S3Yb2_ICSD_26973	metal	metal
S3Yb2_ICSD_71100	metal	metal
S3Yb2_ICSD_72010	metal	metal
S3Yb2_ICSD_87356	metal	metal
S3Yb2_ICSD_87357	metal	metal
S3Zr1_ICSD_651485	insulator	insulator
S4Sb1Tl3_ICSD_100849	insulator	insulator
S4Sb1Tl3_ICSD_56789	insulator	insulator
S4Sb2Yb1_ICSD_600800	insulator	insulator
S4Sc2Zn1_ICSD_650850	insulator	insulator
S4Si1Sr2_ICSD_650888	insulator	insulator
S4Si1Tl4_ICSD_59170	insulator	insulator
S4Sm3_ICSD_650921	metal	metal
S4Sn1Sr2_ICSD_413024	insulator	insulator
S4Sn1Tl4_ICSD_36369	insulator	insulator
S4Sr1Tb2_ICSD_651069	insulator	insulator
S4Sr1Y2_ICSD_651073	insulator	insulator
S4Sr1Yb2_ICSD_651076	metal	metal
S4Ta1Tl3_ICSD_651107	insulator	insulator
S4Ti1Tl4_ICSD_36368	insulator	insulator
S4Tl3V1_ICSD_16572	insulator	insulator
S4Tl4Zr1_ICSD_261208	insulator	insulator
S4V3_ICSD_41780	metal	metal
S4V3_ICSD_72920	metal	metal
S4V3_ICSD_79969	insulator	metal
S4V5_ICSD_24563	insulator	metal
S4Y2Zn1_ICSD_651411	insulator	insulator
S4Y2Zn1_ICSD_651412	insulator	insulator
S4Yb3_ICSD_15726	metal	metal
S4Zr3_ICSD_651475	metal	metal
S5Sb2Sn2_ICSD_35641	insulator	insulator
S5Sb3Tl1_ICSD_17058	insulator	insulator
S5Sm2Sn1_ICSD_8233	metal	metal
S5Sn1Tb2_ICSD_249668	metal	insulator
S5Sn2Tl2_ICSD_15494	insulator	insulator
S5Th2_ICSD_651159	insulator	insulator
S5Tl2_ICSD_651233	insulator	insulator
S5U2_ICSD_651315	insulator	insulator
S5U3_ICSD_23466	insulator	insulator
S6Si2Tl4_ICSD_35041	insulator	insulator
S6_ICSD_20710	insulator	insulator
S7Sb4Sn1_ICSD_169941	insulator	insulator
S7Tb5_ICSD_651120	metal	metal
S7Y5_ICSD_651397	metal	metal
S7_ICSD_16469	insulator	insulator
S8Ti1Tl1V4_ICSD_651224	metal	insulator
S8Tl1V5_ICSD_100374	insulator	metal
S8Tl1V5_ICSD_603658	insulator	metal
S8_ICSD_200453	metal	metal
S8_ICSD_412326	metal	insulator

Supplementary Table 591. Five-fold cross validated predictions for the metal/insulator classification (583/598).

system	calculated	predicted
S8_ICSD_43251	metal	metal
Sb10Sr11_ICSD_57166	metal	metal
Sb10Yb11_ICSD_26237	metal	metal
Sb1Sc1_ICSD_157507	metal	metal
Sb1Sc1_ICSD_164630	metal	metal
Sb1Sc2_ICSD_409586	metal	metal
Sb1Se1Th1_ICSD_651533	metal	metal
Sb1Se1U1_ICSD_42486	metal	metal
Sb1Se2Tl1_ICSD_36537	insulator	insulator
Sb1Se3Tl3_ICSD_60962	insulator	insulator
Sb1Sm1Te1_ICSD_602285	metal	metal
Sb1Sm1_ICSD_651557	metal	metal
Sb1Sm1_ICSD_651560	metal	metal
Sb1Sn1_ICSD_52294	metal	metal
Sb1Sn1_ICSD_52303	metal	metal
Sb1Sn1_ICSD_53968	metal	metal
Sb1Sn1_ICSD_651575	metal	metal
Sb1Sr2_ICSD_42119	metal	metal
Sb1Ta3_ICSD_52310	metal	metal
Sb1Tb1Zr1_ICSD_152637	metal	metal
Sb1Tb1_ICSD_52437	metal	metal
Sb1Te1Th1_ICSD_602304	metal	metal
Sb1Te1U1_ICSD_651647	metal	metal
Sb1Te2Tl1_ICSD_651642	metal	insulator
Sb1Th1_ICSD_44718	metal	metal
Sb1Th1_ICSD_52316	metal	metal
Sb1Ti1_ICSD_96138	metal	metal
Sb1Ti3_ICSD_43355	metal	metal
Sb1Ti3_ICSD_651683	metal	metal
Sb1Tl1_ICSD_184577	metal	metal
Sb1Tl1_ICSD_60381	metal	metal
Sb1Tm1Zr1_ICSD_107073	metal	metal
Sb1Tm1_ICSD_52325	metal	metal
Sb1Tm1_ICSD_651692	metal	metal
Sb1Tm1_ICSD_651696	metal	metal
Sb1Tm1_ICSD_658026	metal	metal
Sb1U1_ICSD_52326	metal	metal
Sb1U1_ICSD_651704	metal	metal
Sb1V1_ICSD_23910	metal	metal
Sb1V3_ICSD_106037	metal	metal
Sb1Y1Zr1_ICSD_107067	metal	metal
Sb1Y1_ICSD_185499	metal	metal
Sb1Y1_ICSD_43632	metal	metal
Sb1Yb1_ICSD_651761	metal	metal
Sb1Zn1_ICSD_651770	insulator	metal
Sb1Zr1_ICSD_107692	metal	metal
Sb1Zr1_ICSD_52404	metal	metal
Sb1Zr2_ICSD_651781	metal	metal
Sb1Zr3_ICSD_651786	metal	metal
Sb1_ICSD_108182	metal	metal

Supplementary Table 592. Five-fold cross validated predictions for the metal/insulator classification (584/598).

system	calculated	predicted
Sb1_ICSD_109037	metal	metal
Sb1_ICSD_109038	metal	metal
Sb1_ICSD_42678	metal	metal
Sb1_ICSD_52198	metal	metal
Sb1_ICSD_52226	metal	metal
Sb1_ICSD_651488	metal	metal
Sb1_ICSD_651504	metal	metal
Sb2Se1Te2_ICSD_2085	metal	insulator
Sb2Se2Te1_ICSD_60963	insulator	metal
Sb2Se2Te1_ICSD_651529	insulator	metal
Sb2Se3_ICSD_30973	insulator	insulator
Sb2Se4Tl2_ICSD_20374	metal	insulator
Sb2Se4Yb1_ICSD_600795	insulator	insulator
Sb2Si1V4_ICSD_82564	metal	metal
Sb2Sm1_ICSD_43029	metal	metal
Sb2Sn1Te4_ICSD_657316	insulator	insulator
Sb2Sn1Zn1_ICSD_651586	metal	metal
Sb2Sr1Zn1_ICSD_10001	metal	metal
Sb2Sr1Zn2_ICSD_12152	metal	metal
Sb2Sr1_ICSD_52307	metal	metal
Sb2Ta1_ICSD_52311	metal	metal
Sb2Tb1_ICSD_651605	metal	metal
Sb2Tb1_ICSD_651606	metal	metal
Sb2Te1_ICSD_69557	metal	metal
Sb2Te2_ICSD_20459	metal	insulator
Sb2Te3_ICSD_185953	metal	insulator
Sb2Te3_ICSD_185954	metal	metal
Sb2Te3_ICSD_187496	metal	insulator
Sb2Te3_ICSD_651638	metal	metal
Sb2Te3_ICSD_87415	metal	metal
Sb2Th1_ICSD_651663	metal	metal
Sb2Ti1_ICSD_52322	metal	metal
Sb2Tl1Zn2_ICSD_76499	metal	metal
Sb2Tl7_ICSD_52288	metal	metal
Sb2Tm1_ICSD_651689	metal	metal
Sb2U1_ICSD_43030	metal	metal
Sb2V1_ICSD_76407	metal	metal
Sb2V3_ICSD_41814	metal	metal
Sb2Y1_ICSD_651733	metal	metal
Sb2Yb1Zn2_ICSD_419689	metal	metal
Sb2Yb1_ICSD_22235	metal	metal
Sb2Zr1_ICSD_42878	metal	metal
Sb3Si1Zr5_ICSD_57144	metal	metal
Sb3Sm4_ICSD_656382	metal	metal
Sb3Sr2_ICSD_32033	insulator	metal
Sb3Sr5_ICSD_42120	metal	metal
Sb3Sr5_ICSD_77751	metal	metal
Sb3Tb4_ICSD_601429	metal	metal
Sb3Tb5_ICSD_182208	metal	metal
Sb3Ti5_ICSD_1345	metal	metal

Supplementary Table 593. Five-fold cross validated predictions for the metal/insulator classification (585/598).

system	calculated	predicted
Sb3Y4_ICSD_651736	metal	metal
Sb3Y5_ICSD_651739	metal	metal
Sb3Yb4_ICSD_651762	metal	metal
Sb3Yb5_ICSD_26221	metal	metal
Sb3Yb5_ICSD_43032	metal	metal
Sb3Zn1Zr5_ICSD_57167	metal	metal
Sb3Zn3.6_ICSD_247761	metal	metal
Sb3Zr5_ICSD_52408	metal	metal
Sb3Zr5_ICSD_651788	metal	metal
Sb4Sn3Sr1_ICSD_165617	metal	metal
Sb4Ta5_ICSD_280152	metal	metal
Sb4Ta5_ICSD_52312	metal	metal
Sb4Th3_ICSD_16655	metal	metal
Sb4U3_ICSD_167392	metal	metal
Sb4V5_ICSD_164410	metal	metal
Sb4Zr5_ICSD_153609	metal	metal
Sb5Tb2_ICSD_159134	metal	metal
Sb5Ti1U3_ICSD_43019	metal	metal
Sb8Te3_ICSD_152188	metal	metal
Sc1Se1_ICSD_651805	metal	metal
Sc1Se2Tl1_ICSD_418475	insulator	insulator
Sc1Si1Sm1_ICSD_39494	metal	metal
Sc1Si1_ICSD_169408	metal	metal
Sc1Si1_ICSD_42946	metal	metal
Sc1Si2_ICSD_651822	metal	metal
Sc1Sn1_ICSD_169410	metal	metal
Sc1Tc2_ICSD_651831	metal	metal
Sc1Te1_ICSD_262042	metal	metal
Sc1Te1_ICSD_43593	metal	metal
Sc1Te2Tl1_ICSD_418476	insulator	insulator
Sc1Zn12_ICSD_106042	metal	metal
Sc1Zn1_ICSD_106041	metal	metal
Sc1Zn2_ICSD_55576	metal	metal
Sc1_ICSD_153837	metal	metal
Sc1_ICSD_164088	metal	metal
Sc1_ICSD_246445	metal	metal
Sc1_ICSD_52409	metal	metal
Sc1_ICSD_52410	metal	metal
Sc1_ICSD_52412	metal	metal
Sc1_ICSD_651799	metal	metal
Sc2Sr1Te4_ICSD_416325	insulator	insulator
Sc2Te1_ICSD_406579	metal	metal
Sc2Zr1_ICSD_168950	metal	metal
Sc3Zn17_ICSD_106043	metal	metal
Sc5Si3_ICSD_23932	metal	metal
Sc5Si3_ICSD_651814	metal	metal
Sc5Sn3_ICSD_402546	metal	metal
Sc8.2488_ICSD_164200	metal	metal
Se11Ta2Tl4_ICSD_412581	insulator	insulator
Se17U8V1_ICSD_602307	metal	metal

Supplementary Table 594. Five-fold cross validated predictions for the metal/insulator classification (586/598).

system	calculated	predicted
Se1Si1Th1_ICSD_651864	metal	metal
Se1Si1U1_ICSD_651868	metal	metal
Se1Si1Zr1_ICSD_15571	metal	metal
Se1Sm1_ICSD_52421	metal	metal
Se1Sm1_ICSD_651876	metal	metal
Se1Sm1_ICSD_651894	metal	metal
Se1Sn1_ICSD_100673	insulator	insulator
Se1Sn1_ICSD_41740	metal	insulator
Se1Sn1_ICSD_50551	insulator	metal
Se1Sn1_ICSD_52424	insulator	insulator
Se1Sr1_ICSD_28901	insulator	insulator
Se1Ta2_ICSD_657372	metal	metal
Se1Tb1_ICSD_52438	metal	metal
Se1Tb1_ICSD_651983	metal	metal
Se1Te1U1_ICSD_652008	metal	metal
Se1Th1_ICSD_26653	metal	metal
Se1Th1_ICSD_52443	metal	metal
Se1Ti1_ICSD_652036	metal	metal
Se1Ti1_ICSD_652041	metal	metal
Se1Ti1_ICSD_652055	insulator	metal
Se1Ti2_ICSD_96774	metal	metal
Se1Ti2_ICSD_96775	metal	metal
Se1Tl1_ICSD_52446	metal	metal
Se1Tl1_ICSD_652064	insulator	insulator
Se1Tm1_ICSD_52447	metal	metal
Se1Tm1_ICSD_652082	metal	metal
Se1Tm1_ICSD_652083	metal	metal
Se1U1_ICSD_652103	metal	metal
Se1V1_ICSD_83868	insulator	insulator
Se1Y1_ICSD_652182	metal	metal
Se1Yb1_ICSD_52453	insulator	metal
Se1Yb1_ICSD_652207	insulator	insulator
Se1Zn1_ICSD_181672	insulator	insulator
Se1Zn1_ICSD_67778	insulator	insulator
Se1Zr2_ICSD_42988	metal	metal
Se1_ICSD_104187	metal	metal
Se1_ICSD_150731	metal	insulator
Se1_ICSD_164267	insulator	metal
Se1_ICSD_24670	insulator	insulator
Se1_ICSD_52418	metal	metal
Se1_ICSD_57181	metal	metal
Se1_ICSD_659254	metal	insulator
Se2Si1_ICSD_24592	insulator	insulator
Se2Si1_ICSD_651863	insulator	insulator
Se2Sm1Tl1_ICSD_651896	metal	insulator
Se2Sm1_ICSD_651891	metal	metal
Se2Sn1_ICSD_656780	insulator	insulator
Se2Ta1_ICSD_18130	metal	metal
Se2Ta1_ICSD_24316	metal	metal
Se2Ta1_ICSD_24318	metal	metal

Supplementary Table 595. Five-fold cross validated predictions for the metal/insulator classification (587/598).

system	calculated	predicted
Se2Ta1_ICSD_26249	metal	metal
Se2Ta1_ICSD_651948	metal	metal
Se2Ta1_ICSD_651952	metal	metal
Se2Ta1_ICSD_651954	metal	metal
Se2Ta1_ICSD_651958	metal	metal
Se2Tb1Tl1_ICSD_651989	insulator	insulator
Se2Th1_ICSD_652016	insulator	metal
Se2Ti1_ICSD_652052	insulator	metal
Se2Ti9_ICSD_404193	metal	metal
Se2Tl1Tm1_ICSD_652071	metal	insulator
Se2Tl1Y1_ICSD_652075	insulator	insulator
Se2Tl1Yb1_ICSD_652076	metal	insulator
Se2Tm1_ICSD_652081	metal	metal
Se2U1_ICSD_50239	metal	metal
Se2U1_ICSD_652123	insulator	metal
Se2V1_ICSD_652160	metal	metal
Se2V1_ICSD_652163	metal	metal
Se2W1_ICSD_652167	insulator	insulator
Se2Yb1_ICSD_652201	metal	metal
Se2Zn1_ICSD_652213	insulator	insulator
Se2Zr1_ICSD_109291	insulator	insulator
Se3Sm2_ICSD_78780	metal	metal
Se3Sn1Tl4_ICSD_651938	metal	metal
Se3Sr1Zr1_ICSD_84723	metal	insulator
Se3Ta1Tl1_ICSD_415528	insulator	metal
Se3Ta1_ICSD_651959	metal	metal
Se3Ta1_ICSD_651965	metal	metal
Se3Ta2_ICSD_42982	metal	metal
Se3Tb2_ICSD_85417	insulator	insulator
Se3Th1_ICSD_652028	insulator	insulator
Se3Th2_ICSD_652026	metal	metal
Se3Tl2_ICSD_652069	insulator	insulator
Se3Tl5_ICSD_20826	metal	metal
Se3Tl5_ICSD_30376	metal	metal
Se3U1_ICSD_652104	insulator	insulator
Se3U2_ICSD_652100	metal	metal
Se3Yb2_ICSD_281340	metal	metal
Se3Zr1_ICSD_25621	insulator	insulator
Se4Sm3_ICSD_651890	metal	metal
Se4Sr1Tb2_ICSD_651942	insulator	insulator
Se4Sr1Y2_ICSD_651944	insulator	insulator
Se4Sr1Yb2_ICSD_651945	metal	insulator
Se4Ta1Tl3_ICSD_600250	insulator	insulator
Se4Tb3_ICSD_651982	metal	metal
Se4Ti1Tl4_ICSD_36370	insulator	insulator
Se4Ti1Zr1_ICSD_104188	insulator	metal
Se4Ti3_ICSD_601347	metal	metal
Se4Ti3_ICSD_79629	metal	metal
Se4Ti5_ICSD_55075	metal	metal
Se4Tl3V1_ICSD_652072	insulator	insulator

Supplementary Table 596. Five-fold cross validated predictions for the metal/insulator classification (588/598).

system	calculated	predicted
Se4U3_ICSD_60537	metal	metal
Se4V3_ICSD_84195	insulator	metal
Se4V5_ICSD_652166	insulator	metal
Se4Y2Yb1_ICSD_652186	insulator	metal
Se4Y2Zn1_ICSD_652188	insulator	insulator
Se4Yb2Zn1_ICSD_652208	metal	metal
Se4Yb3_ICSD_652193	metal	metal
Se5Sm2Zr1_ICSD_425081	metal	insulator
Se5U3_ICSD_2787	insulator	metal
Se6Si2Tl4_ICSD_35042	insulator	insulator
Se6_ICSD_86496	insulator	metal
Se8Ti5Tl1_ICSD_37121	metal	metal
Se8Tl1V5_ICSD_37122	insulator	metal
Se8_ICSD_2718	insulator	insulator
Se8_ICSD_36333	insulator	insulator
Se9V2_ICSD_48145	insulator	insulator
Si136_ICSD_56721	insulator	insulator
Si1Sm1_ICSD_652266	metal	metal
Si1Sm1_ICSD_652276	metal	metal
Si1Sn1_ICSD_184676	metal	metal
Si1Sn3Zr5_ICSD_656294	metal	metal
Si1Sr1Zn1_ICSD_86388	metal	metal
Si1Sr1_ICSD_160107	metal	metal
Si1Sr1_ICSD_160108	metal	metal
Si1Sr1_ICSD_42140	metal	metal
Si1Sr1_ICSD_78997	metal	metal
Si1Sr2_ICSD_160103	metal	metal
Si1Sr2_ICSD_90771	insulator	metal
Si1Ta2V3_ICSD_652343	metal	metal
Si1Ta2_ICSD_652302	metal	metal
Si1Ta3Te6_ICSD_75617	metal	metal
Si1Ta3_ICSD_108741	metal	metal
Si1Ta4Te4_ICSD_40207	insulator	metal
Si1Tb1Ti1_ICSD_95185	metal	metal
Si1Tb1_ICSD_23585	metal	metal
Si1Tc1_ICSD_652380	metal	metal
Si1Te1Th1_ICSD_652386	metal	metal
Si1Te1Zr1_ICSD_74522	metal	metal
Si1Te2_ICSD_80205	metal	insulator
Si1Th1_ICSD_26570	metal	metal
Si1Th1_ICSD_652406	metal	metal
Si1Ti1Y1_ICSD_88113	metal	metal
Si1Ti1_ICSD_168416	metal	metal
Si1Ti1_ICSD_20375	metal	metal
Si1Ti1_ICSD_652436	metal	metal
Si1Ti3_ICSD_168414	metal	metal
Si1Ti3_ICSD_189704	metal	metal
Si1Tm1_ICSD_652456	metal	metal
Si1U1_ICSD_652481	metal	metal
Si1U3_ICSD_1890	metal	metal

Supplementary Table 597. Five-fold cross validated predictions for the metal/insulator classification (589/598).

system	calculated	predicted
Si1U3_ICSD_31627	metal	metal
Si1U3_ICSD_652468	metal	metal
Si1U3_ICSD_69199	metal	metal
Si1V3_ICSD_52472	metal	metal
Si1V3_ICSD_652512	metal	metal
Si1W3_ICSD_52476	metal	metal
Si1Y1_ICSD_262363	metal	metal
Si1Y1_ICSD_658905	metal	metal
Si1Yb1_ICSD_652599	metal	metal
Si1Zr1_ICSD_43245	metal	metal
Si1Zr1_ICSD_652617	metal	metal
Si1Zr2_ICSD_24717	metal	metal
Si1_ICSD_109036	metal	insulator
Si1_ICSD_181908	insulator	insulator
Si1_ICSD_181909	insulator	insulator
Si1_ICSD_189392	insulator	insulator
Si1_ICSD_189393	insulator	insulator
Si1_ICSD_189394	insulator	insulator
Si1_ICSD_189395	insulator	insulator
Si1_ICSD_189396	insulator	insulator
Si1_ICSD_189401	insulator	insulator
Si1_ICSD_41392	metal	metal
Si1_ICSD_41991	metal	metal
Si1_ICSD_52456	metal	metal
Si1_ICSD_52459	metal	metal
Si1_ICSD_52460	metal	metal
Si1_ICSD_57187	metal	metal
Si1_ICSD_67775	insulator	insulator
Si1_ICSD_67788	insulator	metal
Si1_ICSD_89414	metal	insulator
Si2Sm1_ICSD_652274	metal	metal
Si2Sr1_ICSD_2860	metal	metal
Si2Sr1_ICSD_30373	metal	metal
Si2Sr1_ICSD_37185	metal	metal
Si2Ta1_ICSD_652333	metal	metal
Si2Ta1_ICSD_96028	metal	metal
Si2Tb1_ICSD_20247	metal	metal
Si2Tb1_ICSD_652370	metal	metal
Si2Tc2Th1_ICSD_73028	metal	metal
Si2Te6Tl6_ICSD_416310	insulator	insulator
Si2Th1_ICSD_26569	metal	metal
Si2Th1_ICSD_652390	metal	metal
Si2Th1_ICSD_660234	metal	metal
Si2Th3_ICSD_26571	metal	metal
Si2Th3_ICSD_652398	metal	metal
Si2Ti1_ICSD_1089	metal	metal
Si2Ti1_ICSD_168419	metal	metal
Si2Ti1_ICSD_30217	metal	metal
Si2Ti1_ICSD_652419	metal	metal
Si2Ti1_ICSD_86810	metal	metal

Supplementary Table 598. Five-fold cross validated predictions for the metal/insulator classification (590/598).

system	calculated	predicted
Si2Tm1_ICSD_20251	metal	metal
Si2Tm1_ICSD_604540	metal	metal
Si2Tm1_ICSD_652451	metal	metal
Si2Tm1_ICSD_652455	metal	metal
Si2U1_ICSD_203	metal	metal
Si2U1_ICSD_652472	metal	metal
Si2U3_ICSD_69200	metal	metal
Si2V1_ICSD_652528	metal	metal
Si2V1_ICSD_96025	metal	metal
Si2W1_ICSD_652549	insulator	metal
Si2W1_ICSD_652560	metal	metal
Si2W3_ICSD_652552	metal	metal
Si2Y1_ICSD_150662	metal	metal
Si2Y1_ICSD_652584	metal	metal
Si2Yb1_ICSD_20252	metal	metal
Si2Zr1_ICSD_26758	metal	metal
Si3Sr5_ICSD_160105	metal	metal
Si3Sr5_ICSD_93701	metal	metal
Si3Ta5_ICSD_16775	metal	metal
Si3Ta5_ICSD_652324	metal	metal
Si3Tb5_ICSD_652353	metal	metal
Si3Tc5_ICSD_652383	metal	metal
Si3Ti1V4_ICSD_652443	metal	metal
Si3Ti5_ICSD_652413	metal	metal
Si3U1_ICSD_652466	metal	metal
Si3V5_ICSD_652485	metal	metal
Si3V5_ICSD_652505	metal	metal
Si3W5_ICSD_652550	metal	metal
Si3Y1_ICSD_263005	metal	metal
Si3Y5_ICSD_652577	metal	metal
Si3Yb1_ICSD_710066	metal	metal
Si3Yb5_ICSD_652594	metal	metal
Si3Zr5_ICSD_652612	metal	metal
Si4Ti5_ICSD_168417	metal	metal
Si4Yb5_ICSD_153764	metal	metal
Si4Zr5_ICSD_20357	metal	metal
Si5Ta2V4_ICSD_52463	metal	metal
Si5V6_ICSD_652486	metal	metal
Si5V6_ICSD_652489	metal	metal
Si5Yb3_ICSD_415599	metal	metal
Si6Sr1_ICSD_240779	metal	metal
Si6Tc7U4_ICSD_80520	metal	metal
Si7Tc4_ICSD_652382	insulator	metal
Sm1Sn1Zn1_ICSD_152627	metal	metal
Sm1Sn2_ICSD_657392	metal	metal
Sm1Sn3_ICSD_108747	metal	metal
Sm1Te1_ICSD_52484	metal	metal
Sm1Te1_ICSD_52485	metal	metal
Sm1Te2Tl1_ICSD_652677	metal	metal
Sm1Te2_ICSD_652659	metal	metal

Supplementary Table 599. Five-fold cross validated predictions for the metal/insulator classification (591/598).

system	calculated	predicted
Sm1Tl1_ICSD_106062	metal	metal
Sm1Tl1_ICSD_106063	metal	metal
Sm1Tl3_ICSD_106064	metal	metal
Sm1Zn1_ICSD_106066	metal	metal
Sm1Zn2_ICSD_106067	metal	metal
Sm1Zn5_ICSD_106068	metal	metal
Sm1_ICSD_52482	metal	metal
Sm1_ICSD_652640	metal	metal
Sm2Tl1_ICSD_106065	metal	metal
Sm2Zn17_ICSD_104243	metal	metal
Sm2Zn17_ICSD_652702	metal	metal
Sm3Te4_ICSD_652658	metal	metal
Sm4Sn3_ICSD_652647	metal	metal
Sn10Tb11_ICSD_652733	metal	metal
Sn10Y11_ICSD_652838	metal	metal
Sn1Sr1Zn1_ICSD_54356	metal	metal
Sn1Sr1_ICSD_106073	metal	metal
Sn1Sr2_ICSD_652722	insulator	metal
Sn1Ta3_ICSD_106076	metal	metal
Sn1Tb1Zn1_ICSD_163426	metal	metal
Sn1Te1Zr1_ICSD_80190	metal	metal
Sn1Te1_ICSD_52488	metal	metal
Sn1Te1_ICSD_53956	insulator	insulator
Sn1Te1_ICSD_652741	insulator	insulator
Sn1Te1_ICSD_652743	metal	metal
Sn1Te3Tl4_ICSD_73087	metal	metal
Sn1Te5Tl2_ICSD_73666	metal	insulator
Sn1Ti2_ICSD_169007	metal	metal
Sn1Ti2_ICSD_182428	metal	metal
Sn1Ti2_ICSD_189711	metal	metal
Sn1Ti3_ICSD_106087	metal	metal
Sn1Ti3_ICSD_189710	metal	metal
Sn1Ti3_ICSD_652780	metal	metal
Sn1Tl1_ICSD_652804	metal	metal
Sn1V3_ICSD_106099	metal	metal
Sn1V3_ICSD_652830	metal	metal
Sn1Y1Zn1_ICSD_152622	metal	metal
Sn1Yb1Zn1_ICSD_106444	metal	metal
Sn1Yb1_ICSD_652848	metal	metal
Sn1Yb2_ICSD_106105	metal	metal
Sn1Zr3_ICSD_106108	metal	metal
Sn1_ICSD_108748	metal	metal
Sn1_ICSD_426976	metal	metal
Sn1_ICSD_52487	metal	metal
Sn1_ICSD_652714	metal	metal
Sn1_ICSD_654522	metal	metal
Sn2Sr1Zn2_ICSD_424108	metal	metal
Sn2Ta1_ICSD_106077	metal	metal
Sn2Tb1Zn1_ICSD_163425	metal	metal
Sn2Tb1_ICSD_168671	metal	metal

Supplementary Table 600. Five-fold cross validated predictions for the metal/insulator classification (592/598).

system	calculated	predicted
Sn2Th1_ICSD_106081	metal	metal
Sn2Tm1_ICSD_652806	metal	metal
Sn2V1_ICSD_108752	metal	metal
Sn2Y1Zn1_ICSD_183309	metal	metal
Sn2Y1_ICSD_416771	metal	metal
Sn3Sr1_ICSD_410956	metal	metal
Sn3Sr5_ICSD_652723	metal	metal
Sn3Tb1_ICSD_106079	metal	metal
Sn3Tb5_ICSD_652737	metal	metal
Sn3Th1_ICSD_106082	metal	metal
Sn3Th5_ICSD_106084	metal	metal
Sn3Ti5_ICSD_169008	metal	metal
Sn3Ti5_ICSD_652783	metal	metal
Sn3Ti5_ICSD_652785	metal	metal
Sn3U1_ICSD_652817	metal	metal
Sn3Y1_ICSD_106102	metal	metal
Sn3Y5_ICSD_106103	metal	metal
Sn3Yb1_ICSD_652844	metal	metal
Sn3Yb5_ICSD_652846	metal	metal
Sn3Zr5_ICSD_652854	metal	metal
Sn4Sr1_ICSD_54756	metal	metal
Sn4Th5_ICSD_106085	metal	metal
Sn4Zr5_ICSD_107311	metal	metal
Sn5Sr3_ICSD_411981	metal	metal
Sn5Ti6_ICSD_169009	metal	metal
Sn5Ti6_ICSD_169010	metal	metal
Sn5Yb3_ICSD_710073	metal	metal
Sn6V6Y1_ICSD_182148	metal	metal
Sn7Tb3_ICSD_174437	metal	metal
Sr1Te1_ICSD_52491	insulator	insulator
Sr1Te1_ICSD_53950	insulator	insulator
Sr1Tl1_ICSD_652886	metal	metal
Sr1Tl2_ICSD_106111	metal	metal
Sr1Zn11_ICSD_240990	metal	metal
Sr1Zn13_ICSD_652897	metal	metal
Sr1Zn1_ICSD_652893	metal	metal
Sr1Zn2_ICSD_106113	metal	metal
Sr1Zn2_ICSD_246194	metal	metal
Sr1Zn5_ICSD_418615	metal	metal
Sr1Zn5_ICSD_652894	metal	metal
Sr1_ICSD_109024	metal	metal
Sr1_ICSD_109026	metal	metal
Sr1_ICSD_109027	metal	metal
Sr1_ICSD_652874	metal	metal
Sr1_ICSD_652875	metal	metal
Sr1_ICSD_652876	metal	metal
Sr1_ICSD_81123	metal	metal
Sr3Tl5_ICSD_380325	metal	metal
Sr5Tl3_ICSD_652883	metal	metal
Ta1Tc1_ICSD_106117	metal	metal

Supplementary Table 601. Five-fold cross validated predictions for the metal/insulator classification (593/598).

system	calculated	predicted
Ta1Te2_ICSD_155325	metal	metal
Ta1Te2_ICSD_155326	metal	metal
Ta1Te4_ICSD_62049	metal	metal
Ta1V2_ICSD_106121	metal	metal
Ta1Zn2_ICSD_652936	metal	metal
Ta1Zn2_ICSD_652937	metal	metal
Ta1_ICSD_41520	metal	metal
Ta1_ICSD_652898	metal	metal
Ta1_ICSD_652900	metal	metal
Ta22_ICSD_54203	metal	metal
Ta22_ICSD_54206	metal	metal
Ta2Te3_ICSD_41047	metal	metal
Ta30_ICSD_280872	metal	metal
Ta3Te12_ICSD_74414	metal	metal
Ta6Zn7_ICSD_652935	metal	metal
Ta8_ICSD_54207	metal	metal
Tb1Tc2_ICSD_652949	metal	metal
Tb1Te1_ICSD_106127	metal	metal
Tb1Te2Tl1_ICSD_652962	insulator	insulator
Tb1Te2_ICSD_652955	metal	metal
Tb1Tl1_ICSD_106129	metal	metal
Tb1Tl3_ICSD_652975	metal	metal
Tb1Zn12_ICSD_652988	metal	metal
Tb1Zn1_ICSD_653006	metal	metal
Tb1Zn2_ICSD_106138	metal	metal
Tb1Zn3_ICSD_652985	metal	metal
Tb1_ICSD_104193	metal	metal
Tb1_ICSD_52494	metal	metal
Tb1_ICSD_52496	metal	metal
Tb1_ICSD_652944	metal	metal
Tb1_ICSD_652948	metal	metal
Tb2Tl1_ICSD_106132	metal	metal
Tb2Zn17_ICSD_104244	metal	metal
Tb2Zn17_ICSD_652992	metal	metal
Tb3Tl5_ICSD_652974	metal	metal
Tb3Zn11_ICSD_652984	metal	metal
Tb5Tl3_ICSD_106133	metal	metal
Tb5Tl3_ICSD_652978	metal	metal
Tb6Zn23_ICSD_652986	metal	metal
Tc1Ti1_ICSD_106142	metal	metal
Tc1V1_ICSD_653028	metal	metal
Tc1_ICSD_41514	metal	metal
Tc1_ICSD_52498	metal	metal
Tc2Th1_ICSD_653016	metal	metal
Tc2Y1_ICSD_653038	metal	metal
Tc2Zr1_ICSD_423221	metal	metal
Te1Th1_ICSD_653065	metal	metal
Te1Ti1_ICSD_52503	metal	metal
Te1Ti1_ICSD_653081	insulator	metal
Te1Tl1_ICSD_52050	metal	metal

Supplementary Table 602. Five-fold cross validated predictions for the metal/insulator classification (594/598).

system	calculated	predicted
Te1Tl1_ICSD_90895	metal	metal
Te1Tl1_ICSD_90900	metal	insulator
Te1Tl2_ICSD_280923	insulator	insulator
Te1Tm1_ICSD_107010	metal	metal
Te1Tm1_ICSD_43649	metal	metal
Te1Tm1_ICSD_52505	metal	metal
Te1Tm1_ICSD_52506	metal	metal
Te1U1_ICSD_104195	metal	metal
Te1U1_ICSD_603009	metal	metal
Te1V1_ICSD_52509	insulator	metal
Te1Y1_ICSD_106153	metal	metal
Te1Yb1_ICSD_33674	insulator	metal
Te1Zn1_ICSD_184486	insulator	insulator
Te1Zn1_ICSD_184488	insulator	insulator
Te1Zn1_ICSD_184491	metal	insulator
Te1Zn1_ICSD_31840	metal	insulator
Te1Zn1_ICSD_67779	insulator	insulator
Te1Zn1_ICSD_67792	insulator	metal
Te1Zn1_ICSD_80076	insulator	insulator
Te1Zr1_ICSD_104197	metal	metal
Te1Zr1_ICSD_280620	metal	metal
Te1Zr1_ICSD_52514	metal	metal
Te1Zr1_ICSD_653209	metal	metal
Te1Zr2_ICSD_87994	metal	metal
Te1Zr3_ICSD_51048	metal	metal
Te1Zr3_ICSD_653216	metal	metal
Te1_ICSD_23060	insulator	insulator
Te1_ICSD_42106	metal	metal
Te1_ICSD_52499	metal	metal
Te1_ICSD_52501	metal	metal
Te1_ICSD_653048	insulator	insulator
Te1_ICSD_87272	metal	metal
Te1_ICSD_97742	metal	insulator
Te2Ti1_ICSD_80092	metal	metal
Te2Tl1Y1_ICSD_653098	insulator	insulator
Te2Tm1_ICSD_653106	metal	metal
Te2U1_ICSD_82648	metal	metal
Te2V1.04_ICSD_38369	metal	metal
Te2V1_ICSD_603582	metal	metal
Te2W1_ICSD_653168	metal	metal
Te2W1_ICSD_653170	insulator	metal
Te2Y1_ICSD_653179	metal	metal
Te2Y7_ICSD_160182	metal	metal
Te2Yb1_ICSD_653189	metal	metal
Te2Zr1_ICSD_653213	metal	metal
Te3Tl2_ICSD_26282	insulator	insulator
Te3Tl2_ICSD_410895	insulator	insulator
Te3Tl5_ICSD_40260	metal	metal
Te3Tl5_ICSD_42674	metal	metal
Te3Tl5_ICSD_604388	metal	metal

Supplementary Table 603. Five-fold cross validated predictions for the metal/insulator classification (595/598).

system	calculated	predicted
Te3U1_ICSD_40695	insulator	metal
Te3Zr1_ICSD_42076	metal	metal
Te4Ti1V2_ICSD_653087	metal	metal
Te4Ti1Zr1_ICSD_40496	metal	metal
Te4Ti3_ICSD_43412	metal	metal
Te4Ti5_ICSD_657485	metal	metal
Te4U3_ICSD_653155	metal	metal
Te4V3_ICSD_52510	insulator	metal
Te4V5_ICSD_42881	metal	metal
Te4Zr5_ICSD_653210	metal	metal
Te5U1_ICSD_653137	insulator	metal
Te5Zr1_ICSD_657473	metal	metal
Th1Tl1_ICSD_106158	metal	metal
Th1Tl3_ICSD_106159	metal	metal
Th1Zn2_ICSD_15445	metal	metal
Th1Zn4_ICSD_653259	metal	metal
Th1_ICSD_104198	metal	metal
Th1_ICSD_53787	metal	metal
Th1_ICSD_76038	metal	metal
Th2Tl1_ICSD_653245	metal	metal
Th2Zn17_ICSD_20238	metal	metal
Th2Zn17_ICSD_653255	metal	metal
Th2Zn1_ICSD_653254	metal	metal
Th3Tl5_ICSD_653244	metal	metal
Ti1U2_ICSD_106173	metal	metal
Ti1U2_ICSD_96155	metal	metal
Ti1Y1_ICSD_188184	metal	metal
Ti1Zn2_ICSD_106184	metal	metal
Ti1Zn3_ICSD_106185	metal	metal
Ti1_ICSD_168322	metal	metal
Ti1_ICSD_43733	metal	metal
Ti1_ICSD_44391	metal	metal
Ti1_ICSD_52521	metal	metal
Ti2Zn1_ICSD_653304	metal	metal
Tl1Tm1_ICSD_106191	metal	metal
Tl1Tm1_ICSD_106192	metal	metal
Tl1Tm1_ICSD_653326	metal	metal
Tl1Y1_ICSD_106195	metal	metal
Tl1Yb1_ICSD_106197	metal	metal
Tl1Yb2_ICSD_104201	metal	metal
Tl1_ICSD_43414	metal	metal
Tl1_ICSD_53778	metal	metal
Tl1_ICSD_653316	metal	metal
Tl1_ICSD_653322	metal	metal
Tl3Tm1_ICSD_106193	metal	metal
Tl3Tm1_ICSD_653325	metal	metal
Tl3U1_ICSD_653330	metal	metal
Tl3Y1_ICSD_653336	metal	metal
Tl3Y5_ICSD_653334	metal	metal
Tl3Yb1_ICSD_106199	metal	metal

Supplementary Table 604. Five-fold cross validated predictions for the metal/insulator classification (596/598).

system	calculated	predicted
Tl3Yb8_ICSD_104202	metal	metal
Tl5Y3_ICSD_653335	metal	metal
Tm1Zn1_ICSD_106203	metal	metal
Tm1Zn1_ICSD_653355	metal	metal
Tm1Zn1_ICSD_653366	metal	metal
Tm1Zn2_ICSD_653350	metal	metal
Tm1_ICSD_43584	metal	metal
Tm1_ICSD_52526	metal	metal
Tm1_ICSD_52528	metal	metal
Tm1_ICSD_653345	metal	metal
Tm1_ICSD_653347	metal	metal
U1_ICSD_16057	metal	metal
U1_ICSD_181306	metal	metal
U1_ICSD_43339	metal	metal
U1_ICSD_653380	metal	metal
U2Zn17_ICSD_106208	metal	metal
U2Zn17_ICSD_106209	metal	metal
V1Zn3_ICSD_106240	metal	metal
V1_ICSD_41504	metal	metal
V1_ICSD_44322	metal	metal
V2Zr1_ICSD_653407	metal	metal
V2Zr1_ICSD_653414	metal	metal
V4Zn5_ICSD_106213	metal	metal
W1_ICSD_41521	metal	metal
W1_ICSD_52268	metal	metal
W1_ICSD_52344	metal	metal
W2Zr1_ICSD_653438	metal	metal
W5Zr3_ICSD_653434	metal	metal
Xe1_ICSD_43428	insulator	insulator
Xe1_ICSD_9786	insulator	insulator
Y1Zn12_ICSD_106229	metal	metal
Y1Zn1_ICSD_106226	metal	metal
Y1Zn1_ICSD_653464	metal	metal
Y1Zn3_ICSD_106227	metal	metal
Y1Zn5_ICSD_106228	metal	metal
Y1Zn5_ICSD_653462	metal	metal
Y1_ICSD_187638	metal	metal
Y1_ICSD_41510	metal	metal
Y1_ICSD_653450	metal	metal
Y2Zn17_ICSD_653458	metal	metal
Y2Zn17_ICSD_653467	metal	metal
Y3Zn11_ICSD_653456	metal	metal
Y6Zn23_ICSD_653457	metal	metal
Yb1Zn11_ICSD_653497	metal	metal
Yb1Zn13_ICSD_653482	metal	metal
Yb1Zn1_ICSD_106232	insulator	metal
Yb1Zn1_ICSD_653485	metal	metal
Yb1Zn2_ICSD_653489	metal	metal
Yb1_ICSD_43572	metal	metal
Yb1_ICSD_653477	metal	metal

Supplementary Table 605. Five-fold cross validated predictions for the metal/insulator classification (597/598).

system	calculated	predicted
Yb1_ICSD_653478	metal	metal
Yb2Zn17_ICSD_653483	metal	metal
Yb2Zn17_ICSD_653496	metal	metal
Yb3Zn11_ICSD_653480	metal	metal
Yb3Zn17_ICSD_653490	metal	metal
Zn1Zr1_ICSD_106235	metal	metal
Zn1Zr1_ICSD_181290	metal	metal
Zn1Zr2_ICSD_653511	metal	metal
Zn1_ICSD_52543	metal	metal
Zn22Zr1_ICSD_106238	metal	metal
Zn2Zr1_ICSD_653517	metal	metal
Zr1_ICSD_41511	metal	metal
Zr1_ICSD_52544	metal	metal
Zr1_ICSD_653528	metal	metal

Supplementary Table 606. Five-fold cross validated predictions for the metal/insulator classification (598/598).

Band gap energy

system	calculated	predicted
Ac1Br3_ICSD_31578	4.11 eV	3.80 eV
Ac1Cl3_ICSD_31569	5.08 eV	4.26 eV
Ac2O3_ICSD_31750	3.52 eV	3.45 eV
Ag10C2F8_ICSD_407646	2.26 eV	2.01 eV
Ag15Cl3P4S16_ICSD_416586	1.75 eV	1.56 eV
Ag18O21Si6_ICSD_33907	0.71 eV	0.71 eV
Ag1Al1O2_ICSD_95662	1.69 eV	1.69 eV
Ag1Al1O2_ICSD_99688	1.47 eV	2.04 eV
Ag1Al1S2_ICSD_25356	1.84 eV	1.99 eV
Ag1Al1S2_ICSD_28744	2.35 eV	1.94 eV
Ag1Al1Se2_ICSD_28745	1.56 eV	1.22 eV
Ag1Al1Te2_ICSD_28746	1.36 eV	0.99 eV
Ag1As1C4F6N4S8_ICSD_61131	2.80 eV	2.69 eV
Ag1As1F6Se3_ICSD_418700	1.86 eV	2.22 eV
Ag1As1F7_ICSD_62510	1.45 eV	1.97 eV
Ag1As1Hg1S3_ICSD_31194	1.74 eV	1.23 eV
Ag1As1Hg2O4_ICSD_413087	0.88 eV	1.05 eV
Ag1As1K2_ICSD_1154	1.03 eV	1.08 eV
Ag1As1Na2_ICSD_49007	0.79 eV	0.91 eV
Ag1As1Pb1S3_ICSD_26835	0.33 eV	0.99 eV
Ag1As1S2_ICSD_18101	1.74 eV	1.43 eV
Ag1As1Se2_ICSD_61708	0.17 eV	0.56 eV
Ag1Au1Ba4O6_ICSD_72329	2.04 eV	1.69 eV
Ag1Au1Cl4_ICSD_1152	1.25 eV	1.46 eV
Ag1Au1Cl6Cs2_ICSD_26162	0.98 eV	1.38 eV
Ag1Au1F4_ICSD_90071	2.18 eV	1.55 eV
Ag1Au2F8_ICSD_85416	0.58 eV	1.58 eV
Ag1Au3I8Rb2_ICSD_32031	0.68 eV	1.34 eV
Ag1B1C4N4_ICSD_411179	5.39 eV	4.20 eV
Ag1B1F4_ICSD_415320	4.84 eV	4.50 eV
Ag1B1F5_ICSD_80645	0.80 eV	2.54 eV
Ag1B7O12Sr1_ICSD_406544	3.72 eV	3.50 eV
Ag1Ba1Er1S3_ICSD_75074	1.31 eV	1.45 eV
Ag1Ba1Er1Se3_ICSD_659171	1.42 eV	1.32 eV
Ag1Ba1F1S1_ICSD_183713	1.83 eV	1.83 eV
Ag1Ba1F1Se1_ICSD_183714	1.60 eV	1.61 eV
Ag1Ba1F1Te1_ICSD_419382	1.78 eV	1.50 eV
Ag1Ba1La1Se3_ICSD_659172	1.53 eV	1.21 eV
Ag1Ba1Nd1S3_ICSD_659170	1.76 eV	1.40 eV
Ag1Ba1O9P3_ICSD_50672	4.32 eV	3.84 eV
Ag1Ba1P1_ICSD_41706	0.40 eV	0.50 eV
Ag1Ba1Se3Y1_ICSD_104237	1.42 eV	1.21 eV
Ag1Ba1Te3Y1_ICSD_88717	0.84 eV	0.73 eV
Ag1Bi1Cl2S1_ICSD_413290	1.39 eV	1.32 eV
Ag1Bi1Cr2O8_ICSD_8224	1.87 eV	1.82 eV
Ag1Bi1Cr4O14_ICSD_14234	2.42 eV	1.67 eV
Ag1Bi1K2_ICSD_1156	0.66 eV	0.64 eV
Ag1Bi1P2S6_ICSD_170639	1.38 eV	1.65 eV
Ag1Bi1S2_ICSD_44340	0.78 eV	0.72 eV
Ag1Bi1Se2_ICSD_26518	0.43 eV	0.38 eV

Supplementary Table 607. Five-fold cross validated predictions for the band gap energy (1/277).

system	calculated	predicted
Ag1Bi1Se2_ICSD_26519	0.44 eV	0.44 eV
Ag1Bi1Se2_ICSD_604856	0.44 eV	0.44 eV
Ag1Bi1Te2_ICSD_43266	0.24 eV	0.33 eV
Ag1Bi1Te2_ICSD_604866	0.24 eV	0.30 eV
Ag1Bi2Cl1S3_ICSD_412372	1.13 eV	1.15 eV
Ag1Bi2Cl1Se3_ICSD_412371	0.80 eV	1.01 eV
Ag1Bi3S5_ICSD_171229	0.81 eV	0.90 eV
Ag1Br13Mo6_ICSD_412903	2.37 eV	1.79 eV
Ag1Br14W6_ICSD_410958	2.77 eV	2.18 eV
Ag1Br1H3N1_ICSD_169137	1.97 eV	2.06 eV
Ag1Br1Hg1S1_ICSD_411773	1.72 eV	1.42 eV
Ag1Br1O1Pb1_ICSD_33913	0.36 eV	1.40 eV
Ag1Br1_ICSD_157536	1.57 eV	1.49 eV
Ag1Br1_ICSD_56550	1.56 eV	1.50 eV
Ag1Br3Cs2_ICSD_150288	3.20 eV	2.88 eV
Ag1Br3Rb2_ICSD_150287	3.13 eV	2.86 eV
Ag1Br5Pb2_ICSD_300100	2.58 eV	2.17 eV
Ag1C1H3N2_ICSD_169135	3.15 eV	2.99 eV
Ag1C1N1O1_ICSD_18149	3.30 eV	2.92 eV
Ag1C1N1O1_ICSD_23832	2.68 eV	2.71 eV
Ag1C1N1O1_ICSD_23833	3.22 eV	2.57 eV
Ag1C1N1O1_ICSD_260378	1.99 eV	2.50 eV
Ag1C1N1_ICSD_85783	4.18 eV	2.80 eV
Ag1C1N3O2_ICSD_408288	3.17 eV	2.98 eV
Ag1C2Cl2F6N2Sb1_ICSD_64638	3.84 eV	3.02 eV
Ag1C2Cs1_ICSD_410873	2.97 eV	2.42 eV
Ag1C2F6H2N2Sb1_ICSD_63287	3.85 eV	3.60 eV
Ag1C2H2N3O1_ICSD_63100	3.50 eV	3.28 eV
Ag1C2K1N2S2_ICSD_280587	3.21 eV	2.82 eV
Ag1C2K1N2_ICSD_30275	3.73 eV	3.56 eV
Ag1C2K1_ICSD_410874	2.75 eV	2.47 eV
Ag1C2Li1_ICSD_410868	1.89 eV	2.14 eV
Ag1C2N2Na1_ICSD_65697	3.52 eV	3.16 eV
Ag1C2N3_ICSD_68453	3.89 eV	3.78 eV
Ag1C2N3_ICSD_843	3.80 eV	3.59 eV
Ag1C4H12N1Se5_ICSD_159458	1.19 eV	1.88 eV
Ag1C4N3_ICSD_43823	3.57 eV	3.51 eV
Ag1Ca1Sb1_ICSD_56982	0.14 eV	0.29 eV
Ag1Cd1O4V1_ICSD_401350	2.59 eV	2.14 eV
Ag1Cd2Ga1S4_ICSD_90459	1.39 eV	1.28 eV
Ag1Ce3Ge1S7_ICSD_604947	1.83 eV	1.81 eV
Ag1Ce3S7Si1_ICSD_604959	1.79 eV	1.85 eV
Ag1Ce3Se7Si1_ICSD_604962	1.48 eV	1.44 eV
Ag1Cl1O2_ICSD_15407	1.35 eV	1.74 eV
Ag1Cl1O2_ICSD_16717	0.98 eV	1.39 eV
Ag1Cl1O3_ICSD_30227	3.30 eV	2.51 eV
Ag1Cl1O4Pb4_ICSD_68309	2.43 eV	1.84 eV
Ag1Cl1O4_ICSD_185363	3.64 eV	2.60 eV
Ag1Cl1O4_ICSD_185366	3.46 eV	3.18 eV
Ag1Cl1O4_ICSD_185367	3.38 eV	2.90 eV

Supplementary Table 608. Five-fold cross validated predictions for the band gap energy (2/277).

system	calculated	predicted
Ag1Cl1O4_ICSD_33568	2.57 eV	2.93 eV
Ag1Cl1_ICSD_56539	1.97 eV	1.57 eV
Ag1Cl1_ICSD_56543	1.96 eV	1.38 eV
Ag1Cl1_ICSD_56545	1.38 eV	1.75 eV
Ag1Cl2Cs1_ICSD_150300	2.91 eV	2.47 eV
Ag1Cl3Cs2_ICSD_150286	3.63 eV	3.21 eV
Ag1Cl3Rb2_ICSD_280031	3.59 eV	2.95 eV
Ag1Co1O2_ICSD_180888	0.43 eV	0.85 eV
Ag1Co1O2_ICSD_187261	0.94 eV	0.56 eV
Ag1Co1O2_ICSD_261608	0.94 eV	0.57 eV
Ag1Co1O4P1_ICSD_100520	2.38 eV	2.21 eV
Ag1Co3H2O12P3_ICSD_280774	0.10 eV	2.24 eV
Ag1Cr1O2_ICSD_4149	1.63 eV	1.53 eV
Ag1Cr1S2_ICSD_42395	0.95 eV	0.81 eV
Ag1Cr1Se2_ICSD_68423	0.35 eV	0.55 eV
Ag1Cr2H4O10Sc1_ICSD_156676	2.48 eV	2.16 eV
Ag1Cr3O8_ICSD_155507	1.68 eV	1.35 eV
Ag1Cs1F3_ICSD_23154	0.28 eV	0.93 eV
Ag1Cs1N2O6_ICSD_280069	2.88 eV	2.33 eV
Ag1Cs1O1_ICSD_25745	1.45 eV	1.51 eV
Ag1Cs1O1_ICSD_40160	1.46 eV	1.45 eV
Ag1Cs1O1_ICSD_49754	1.48 eV	1.51 eV
Ag1Cs1Se4_ICSD_87464	1.28 eV	1.29 eV
Ag1Cs2F4_ICSD_16254	0.48 eV	1.64 eV
Ag1Cs2F6K1_ICSD_16783	1.54 eV	1.72 eV
Ag1Cs2I3_ICSD_150291	2.96 eV	2.78 eV
Ag1Cs2S4V1_ICSD_50460	1.30 eV	1.48 eV
Ag1Cs5O9Si3_ICSD_51508	2.54 eV	2.54 eV
Ag1Cu1O2_ICSD_95089	0.27 eV	0.56 eV
Ag1Cu1O4P1_ICSD_165596	1.24 eV	1.08 eV
Ag1Cu1O4P1_ICSD_35590	1.25 eV	1.04 eV
Ag1Cu1O4V1_ICSD_419201	1.13 eV	1.37 eV
Ag1Cu1S1_ICSD_30233	0.47 eV	0.63 eV
Ag1Cu1S1_ICSD_66580	0.63 eV	0.66 eV
Ag1Cu1S1_ICSD_66582	0.58 eV	0.57 eV
Ag1Dy1Se2_ICSD_605083	1.55 eV	1.32 eV
Ag1Dy1Se2_ICSD_605084	1.03 eV	1.09 eV
Ag1Dy1Te2_ICSD_154794	0.88 eV	0.85 eV
Ag1Er1O8W2_ICSD_27743	3.45 eV	2.75 eV
Ag1Er1P2Se6_ICSD_420303	1.92 eV	1.28 eV
Ag1Er1S2_ICSD_423921	1.44 eV	1.35 eV
Ag1Er1Se2_ICSD_605115	1.57 eV	1.43 eV
Ag1Er1Te2_ICSD_154791	0.87 eV	0.85 eV
Ag1F10P1Xe2_ICSD_412662	2.51 eV	2.47 eV
Ag1F11Na1Zr2_ICSD_65179	2.40 eV	3.32 eV
Ag1F11Pd1Zr2_ICSD_65181	2.77 eV	2.41 eV
Ag1F12Sb2_ICSD_65186	1.70 eV	1.82 eV
Ag1F12Ta2_ICSD_62543	2.41 eV	3.00 eV
Ag1F1S1Sr1_ICSD_183709	1.63 eV	1.70 eV
Ag1F1Se1Sr1_ICSD_183710	1.40 eV	1.55 eV

Supplementary Table 609. Five-fold cross validated predictions for the band gap energy (3/277).

system	calculated	predicted
Ag1F1Sr1Te1_ICSD_183711	1.56 eV	1.58 eV
Ag1F1_ICSD_18008	1.08 eV	1.08 eV
Ag1F2O6S2_ICSD_422414	0.80 eV	2.29 eV
Ag1F2_ICSD_6277	1.03 eV	1.31 eV
Ag1F30O6S16Sb1Te6_ICSD_412325	0.95 eV	2.05 eV
Ag1F3K1_ICSD_189150	0.76 eV	1.17 eV
Ag1F3Rb1_ICSD_23153	0.24 eV	0.76 eV
Ag1F3Zn1_ICSD_28950	2.99 eV	2.20 eV
Ag1F3_ICSD_80477	1.22 eV	1.19 eV
Ag1F4K1_ICSD_72715	2.15 eV	1.53 eV
Ag1F4K2_ICSD_421461	2.10 eV	1.87 eV
Ag1F4Na1_ICSD_9903	1.93 eV	1.98 eV
Ag1F4Na2_ICSD_425149	2.05 eV	1.96 eV
Ag1F6Pd1_ICSD_51507	1.34 eV	1.49 eV
Ag1F6Sb1_ICSD_28676	3.37 eV	2.25 eV
Ag1F6Sn1_ICSD_51505	2.07 eV	2.07 eV
Ag1F6Ta1_ICSD_411796	5.27 eV	4.30 eV
Ag1F6Ti1_ICSD_51506	1.97 eV	2.26 eV
Ag1Fe1O6Se2_ICSD_90414	2.17 eV	2.11 eV
Ag1Fe1O7P2_ICSD_421413	2.15 eV	2.24 eV
Ag1Fe1O7V2_ICSD_166891	2.08 eV	1.94 eV
Ag1Fe2S3_ICSD_165661	0.29 eV	0.63 eV
Ag1Ga1O2_ICSD_95665	0.99 eV	0.83 eV
Ag1Ga1P2Se6_ICSD_71971	0.83 eV	1.11 eV
Ag1Ga1S2_ICSD_156785	1.51 eV	1.46 eV
Ag1Ga1S2_ICSD_92052	1.51 eV	1.40 eV
Ag1Ga1Se2_ICSD_605211	0.70 eV	0.92 eV
Ag1Ga1Te2_ICSD_156128	0.52 eV	0.55 eV
Ag1Gd1Se2_ICSD_602138	0.99 eV	0.81 eV
Ag1Ge1La3S7_ICSD_80174	1.96 eV	1.88 eV
Ag1Ge1Pr3S7_ICSD_605303	1.80 eV	1.75 eV
Ag1H2Ni3O12P3_ICSD_280922	3.23 eV	2.37 eV
Ag1H2O4V1_ICSD_75941	2.71 eV	2.05 eV
Ag1H4Mo1N1S4_ICSD_236249	1.46 eV	2.00 eV
Ag1H4N1S4W1_ICSD_84370	1.91 eV	2.02 eV
Ag1Hf1Rb1Te3_ICSD_402632	0.36 eV	0.69 eV
Ag1Hg1I1S1_ICSD_411772	1.54 eV	1.45 eV
Ag1Hg1I1S1_ICSD_54796	1.74 eV	1.48 eV
Ag1Hg1O4V1_ICSD_414429	1.80 eV	1.91 eV
Ag1Hg2N1O5_ICSD_89685	1.20 eV	1.46 eV
Ag1Hg2O4P1_ICSD_2208	1.30 eV	1.18 eV
Ag1Hg3O6Sb1_ICSD_170764	1.04 eV	0.97 eV
Ag1Ho1S2_ICSD_40960	1.35 eV	1.68 eV
Ag1Ho1Se2_ICSD_156419	1.55 eV	1.39 eV
Ag1Ho1Se2_ICSD_605365	1.02 eV	1.13 eV
Ag1Ho1Te2_ICSD_154795	0.88 eV	0.89 eV
Ag1I1O4_ICSD_52380	1.05 eV	1.89 eV
Ag1I1Se3_ICSD_414116	1.51 eV	1.17 eV
Ag1I1Te3_ICSD_414117	1.02 eV	0.84 eV
Ag1I1_ICSD_161581	1.47 eV	1.71 eV

Supplementary Table 610. Five-fold cross validated predictions for the band gap energy (4/277).

system	calculated	predicted
Ag1I1_ICSD_161582	0.40 eV	1.22 eV
Ag1I1_ICSD_164959	1.98 eV	1.70 eV
Ag1I1_ICSD_1899	2.09 eV	1.87 eV
Ag1I1_ICSD_28230	1.98 eV	1.96 eV
Ag1I1_ICSD_52361	1.98 eV	1.72 eV
Ag1I1_ICSD_56557	1.47 eV	1.75 eV
Ag1I1_ICSD_79678	2.00 eV	1.97 eV
Ag1I2Tl1_ICSD_26318	1.63 eV	1.91 eV
Ag1I3K2_ICSD_150289	2.89 eV	2.55 eV
Ag1I3O9Pd1_ICSD_174519	2.20 eV	1.92 eV
Ag1I3Rb2_ICSD_150290	2.89 eV	2.72 eV
Ag1I3Tl2_ICSD_78929	2.61 eV	2.04 eV
Ag1In1O2_ICSD_202429	0.60 eV	1.08 eV
Ag1In1O2_ICSD_95671	0.62 eV	0.73 eV
Ag1In1P2Se6_ICSD_71968	0.82 eV	1.01 eV
Ag1In1S2_ICSD_600585	0.92 eV	0.94 eV
Ag1In1S2_ICSD_605408	1.04 eV	0.93 eV
Ag1In1S2_ICSD_659385	0.56 eV	0.75 eV
Ag1In1Se2_ICSD_605449	0.40 eV	0.67 eV
Ag1In1Te2_ICSD_104476	0.40 eV	0.45 eV
Ag1In5Se8_ICSD_35597	0.40 eV	0.79 eV
Ag1In5Te8_ICSD_151871	0.34 eV	0.55 eV
Ag1K1O1_ICSD_24818	1.50 eV	1.50 eV
Ag1K1O1_ICSD_25744	1.50 eV	1.50 eV
Ag1K2Nb1S4_ICSD_84292	2.23 eV	1.79 eV
Ag1K2P1_ICSD_402572	1.32 eV	1.17 eV
Ag1K2S4Sb1_ICSD_82144	1.44 eV	1.63 eV
Ag1K2S4Ta1_ICSD_84294	2.34 eV	2.07 eV
Ag1K2S4V1_ICSD_66840	1.21 eV	1.51 eV
Ag1K2Sb1_ICSD_1155	1.18 eV	1.04 eV
Ag1K2Se4V1_ICSD_50461	0.67 eV	1.14 eV
Ag1K3Se8Sn3_ICSD_416330	1.15 eV	1.13 eV
Ag1La1O1S1_ICSD_89023	1.67 eV	1.70 eV
Ag1La3S7Si1_ICSD_409845	1.93 eV	1.99 eV
Ag1La3S7Sn1_ICSD_417316	1.88 eV	1.74 eV
Ag1La3Se7Si1_ICSD_605509	1.58 eV	1.50 eV
Ag1La3Se7Sn1_ICSD_417803	1.15 eV	1.18 eV
Ag1Li2Sb1_ICSD_52589	0.41 eV	0.42 eV
Ag1Lu1Se2_ICSD_605535	1.58 eV	1.32 eV
Ag1Mn1O4V1_ICSD_246202	2.08 eV	1.73 eV
Ag1Mn1O4_ICSD_30200	1.39 eV	1.55 eV
Ag1N1O2_ICSD_26750	1.99 eV	2.10 eV
Ag1N1O3_ICSD_1685	2.60 eV	2.58 eV
Ag1N1O3_ICSD_28103	2.59 eV	2.59 eV
Ag1N1O3_ICSD_35157	2.19 eV	2.10 eV
Ag1N1O3_ICSD_374	2.35 eV	2.27 eV
Ag1N2Na1O4_ICSD_20926	2.27 eV	1.98 eV
Ag1N3O4_ICSD_419628	2.65 eV	2.61 eV
Ag1N3_ICSD_183201	2.22 eV	2.19 eV
Ag1N3_ICSD_27135	2.23 eV	2.22 eV

Supplementary Table 611. Five-fold cross validated predictions for the band gap energy (5/277).

system	calculated	predicted
Ag1Na1O1_ICSD_188524	1.31 eV	1.24 eV
Ag1Na1O3S2_ICSD_418297	2.89 eV	2.24 eV
Ag1Na2Sb1_ICSD_10010	0.79 eV	0.84 eV
Ag1Na3O2_ICSD_24817	1.32 eV	1.45 eV
Ag1Na3S2_ICSD_201800	2.23 eV	1.87 eV
Ag1Nb1O3_ICSD_164198	2.13 eV	2.17 eV
Ag1Nb1O3_ICSD_55647	1.84 eV	1.98 eV
Ag1Nb1O3_ICSD_55649	1.77 eV	1.74 eV
Ag1Nb1Rb2S4_ICSD_50463	1.93 eV	1.94 eV
Ag1Nb1Rb2Se4_ICSD_402423	1.86 eV	1.67 eV
Ag1Nb3O8_ICSD_67244	2.38 eV	2.36 eV
Ag1O13P4Ta1_ICSD_86892	3.37 eV	3.21 eV
Ag1O1P1Th1_ICSD_419463	0.17 eV	0.97 eV
Ag1O1Rb1_ICSD_188526	1.45 eV	1.42 eV
Ag1O1Rb1_ICSD_40155	1.45 eV	1.43 eV
Ag1O1_ICSD_202055	0.33 eV	0.42 eV
Ag1O1_ICSD_43741	0.34 eV	0.40 eV
Ag1O2Rh1_ICSD_261561	1.65 eV	1.25 eV
Ag1O2Sc1_ICSD_422442	2.44 eV	1.97 eV
Ag1O2Sc1_ICSD_95668	2.46 eV	2.29 eV
Ag1O2Y1_ICSD_95674	2.54 eV	1.98 eV
Ag1O2Yb1_ICSD_163472	0.50 eV	0.75 eV
Ag1O3Sb1_ICSD_245292	1.09 eV	1.30 eV
Ag1O3Sb1_ICSD_25541	0.61 eV	1.11 eV
Ag1O3Ta1_ICSD_40830	2.58 eV	2.55 eV
Ag1O3Ta1_ICSD_40831	2.57 eV	2.46 eV
Ag1O3Te1Tl1_ICSD_169995	2.67 eV	1.48 eV
Ag1O3V1_ICSD_50645	2.76 eV	1.98 eV
Ag1O3V1_ICSD_82079	2.05 eV	1.66 eV
Ag1O4P1Zn1_ICSD_260974	2.47 eV	2.48 eV
Ag1O4Re1_ICSD_280086	3.09 eV	2.44 eV
Ag1O4Tc1_ICSD_281321	2.34 eV	2.17 eV
Ag1O5Se1V1_ICSD_417773	1.23 eV	1.98 eV
Ag1O5Te1V1_ICSD_417774	2.92 eV	2.36 eV
Ag1O6Tl1V2_ICSD_201934	2.76 eV	2.35 eV
Ag1P1S3_ICSD_653748	1.52 eV	1.68 eV
Ag1P1S4Zn1_ICSD_48197	2.48 eV	2.03 eV
Ag1P1Sr1_ICSD_52596	0.36 eV	0.44 eV
Ag1P2S6Sc1_ICSD_63273	2.23 eV	1.91 eV
Ag1P2S6V1_ICSD_655222	0.89 eV	1.19 eV
Ag1P2Sc1Se6_ICSD_420302	1.94 eV	1.27 eV
Ag1P2Se6V1_ICSD_68143	0.73 eV	0.97 eV
Ag1P2_ICSD_35283	0.75 eV	0.81 eV
Ag1Pb1Pr1_ICSD_107101	0.10 eV	0.18 eV
Ag1Pb1S3Sb1_ICSD_24257	0.78 eV	0.94 eV
Ag1Pb1S3Sb1_ICSD_8166	0.36 eV	0.93 eV
Ag1Pr3S7Si1_ICSD_605682	1.78 eV	1.82 eV
Ag1Pr3Se7Si1_ICSD_605685	1.49 eV	1.35 eV
Ag1Rb1S3U1_ICSD_249702	1.67 eV	1.65 eV
Ag1Rb1Se3U1_ICSD_249703	1.35 eV	1.18 eV

Supplementary Table 612. Five-fold cross validated predictions for the band gap energy (6/277).

system	calculated	predicted
Ag1Rb1Se4_ICSD_87463	1.19 eV	1.14 eV
Ag1Rb2S4Ta1_ICSD_84295	2.30 eV	2.24 eV
Ag1Rb2S4V1_ICSD_66841	1.59 eV	1.59 eV
Ag1Rb2Se4V1_ICSD_50462	1.23 eV	1.13 eV
Ag1Rb3Se8Sn3_ICSD_416294	1.21 eV	1.16 eV
Ag1S1Tl1_ICSD_605754	0.97 eV	1.03 eV
Ag1S2Sb1_ICSD_16578	1.40 eV	1.37 eV
Ag1S2Sb1_ICSD_85130	1.38 eV	1.33 eV
Ag1S2Sb1_ICSD_94647	1.37 eV	1.35 eV
Ag1Sb1Te2_ICSD_704040	0.12 eV	0.37 eV
Ag1Sc1Se2_ICSD_155115	1.25 eV	0.95 eV
Ag1Sc1Se2_ICSD_605797	1.35 eV	1.19 eV
Ag1Se1Tl1_ICSD_100710	0.65 eV	0.69 eV
Ag1Se2Tb1_ICSD_605827	1.02 eV	0.96 eV
Ag1Se2Y1_ICSD_605836	1.52 eV	1.30 eV
Ag1Tb1Te2_ICSD_154793	0.88 eV	0.85 eV
Ag1Te1Tl1_ICSD_52609	0.76 eV	0.56 eV
Ag1Te2Tl3_ICSD_61680	0.59 eV	0.58 eV
Ag1Te2Y1_ICSD_154792	0.87 eV	0.73 eV
Ag2.67Cr1I0.67O4_ICSD_419833	2.08 eV	1.79 eV
Ag2As1Cs1S3_ICSD_421091	1.98 eV	1.80 eV
Ag2As1K1O4_ICSD_409793	1.01 eV	1.43 eV
Ag2As4Hg7I6_ICSD_391132	0.74 eV	0.92 eV
Ag2Ba1Ge1S4_ICSD_10040	1.01 eV	1.15 eV
Ag2Ba1Ge1Se4_ICSD_411405	0.37 eV	0.77 eV
Ag2Ba1Hg2O4_ICSD_40835	1.78 eV	1.35 eV
Ag2Ba1Mn1O8V2_ICSD_408193	2.63 eV	1.99 eV
Ag2Ba1S2_ICSD_50183	1.35 eV	1.31 eV
Ag2Ba1S4Sn1_ICSD_41898	0.61 eV	0.92 eV
Ag2Ba1Se4Sn1_ICSD_170856	0.24 eV	0.61 eV
Ag2Ba1Te2_ICSD_246048	1.02 eV	0.83 eV
Ag2Bi1O3_ICSD_415959	0.66 eV	0.78 eV
Ag2Br14W6_ICSD_410959	2.80 eV	2.24 eV
Ag2Br1N1O3_ICSD_1311	2.40 eV	2.25 eV
Ag2Br6Hg7P8_ICSD_171256	1.01 eV	1.21 eV
Ag2C1Cl1N1O4S1_ICSD_410623	3.18 eV	2.52 eV
Ag2C1N2_ICSD_411091	1.23 eV	1.82 eV
Ag2C1O3_ICSD_281040	0.92 eV	1.45 eV
Ag2C1O3_ICSD_93988	1.13 eV	1.67 eV
Ag2C2O4_ICSD_109603	3.11 eV	2.23 eV
Ag2C4Cs1Cu1N4_ICSD_415572	2.69 eV	2.72 eV
Ag2C4Cu1K1N4_ICSD_415570	2.62 eV	2.76 eV
Ag2C4Cu1N4Rb1_ICSD_415571	2.67 eV	2.77 eV
Ag2C4N4S4Zn1_ICSD_71563	3.28 eV	2.71 eV
Ag2C4O4_ICSD_109770	0.92 eV	1.93 eV
Ag2Ca1O12V4_ICSD_161369	2.62 eV	2.47 eV
Ag2Cd1Ge1S4_ICSD_152753	0.98 eV	1.05 eV
Ag2Cd1Ge1S4_ICSD_423404	1.27 eV	1.16 eV
Ag2Cl1N1O3_ICSD_8013	2.49 eV	2.41 eV
Ag2Cl4Pd1_ICSD_65239	1.88 eV	1.60 eV

Supplementary Table 613. Five-fold cross validated predictions for the band gap energy (7/277).

system	calculated	predicted
Ag2Cl6Re1_ICSD_156662	2.04 eV	1.89 eV
Ag2Cr1O4_ICSD_16298	2.17 eV	1.95 eV
Ag2Cr2O7_ICSD_2433	2.09 eV	1.87 eV
Ag2Cs2S4Ti1_ICSD_280645	1.99 eV	1.76 eV
Ag2F14Zn1Zr2_ICSD_422839	1.35 eV	2.78 eV
Ag2F1H2I1O1_ICSD_32660	2.16 eV	1.90 eV
Ag2F5_ICSD_95832	0.88 eV	0.96 eV
Ag2Fe1S4Sn1_ICSD_42534	0.56 eV	0.61 eV
Ag2Ge1In2S6_ICSD_159759	1.38 eV	1.27 eV
Ag2Ge1In2Se6_ICSD_88168	0.78 eV	0.75 eV
Ag2Ge1K2S4_ICSD_170843	1.89 eV	1.92 eV
Ag2Ge1O3_ICSD_167332	0.58 eV	0.78 eV
Ag2Ge1Pb1S4_ICSD_180802	1.59 eV	1.20 eV
Ag2Ge1Rb2S4_ICSD_170844	2.15 eV	1.80 eV
Ag2Ge1S3_ICSD_41711	0.99 eV	1.13 eV
Ag2H12N4O4S1_ICSD_36585	3.37 eV	2.94 eV
Ag2H1O4P1_ICSD_30503	1.97 eV	2.04 eV
Ag2H3I1O6_ICSD_155415	1.31 eV	1.65 eV
Ag2H4O12S3_ICSD_408949	3.95 eV	3.28 eV
Ag2H6N2O6Pd1S2_ICSD_280075	2.31 eV	2.16 eV
Ag2Hf1S3_ICSD_79251	1.40 eV	1.50 eV
Ag2Hg1I2S1_ICSD_413300	1.69 eV	1.52 eV
Ag2Hg1I4_ICSD_30264	1.36 eV	1.27 eV
Ag2Hg1I4_ICSD_6069	1.51 eV	1.36 eV
Ag2Hg1O2_ICSD_280333	0.78 eV	0.89 eV
Ag2Hg1S2_ICSD_201713	0.70 eV	0.93 eV
Ag2Hg2O12Te3_ICSD_171006	0.74 eV	1.52 eV
Ag2Hg7I6P8_ICSD_171257	0.89 eV	1.08 eV
Ag2I10Tl6_ICSD_35389	1.29 eV	1.73 eV
Ag2I1N1O3_ICSD_8075	2.19 eV	2.14 eV
Ag2I6O18Ti1_ICSD_420852	2.99 eV	2.28 eV
Ag2In2S6Si1_ICSD_189391	1.42 eV	1.31 eV
Ag2In2Se6Si1_ICSD_154635	0.81 eV	0.85 eV
Ag2K1Nb1Se4_ICSD_412479	1.66 eV	1.31 eV
Ag2K1P1S4_ICSD_420033	1.60 eV	1.76 eV
Ag2K1S3Sb1_ICSD_420015	2.03 eV	1.50 eV
Ag2K1S4Sb1_ICSD_82143	0.81 eV	1.19 eV
Ag2K1Se4Ta1_ICSD_412477	1.90 eV	1.52 eV
Ag2K2Se4Sn1_ICSD_90119	0.89 eV	1.08 eV
Ag2Mn1O8Sr1V2_ICSD_408052	2.25 eV	2.10 eV
Ag2Mo1O12Te4_ICSD_420406	2.40 eV	2.26 eV
Ag2Mo1O4_ICSD_36187	2.73 eV	2.27 eV
Ag2Mo2O7_ICSD_31027	2.59 eV	2.33 eV
Ag2Mo3O16Te3_ICSD_420405	2.15 eV	2.41 eV
Ag2Nb4O11_ICSD_180731	2.83 eV	2.78 eV
Ag2Nb4O11_ICSD_180733	2.86 eV	2.75 eV
Ag2O10U1W2_ICSD_98550	2.29 eV	2.46 eV
Ag2O11Ta4_ICSD_180734	3.16 eV	2.61 eV
Ag2O11V4_ICSD_93453	1.45 eV	1.92 eV
Ag2O12Sr1V4_ICSD_161371	2.79 eV	2.44 eV

Supplementary Table 614. Five-fold cross validated predictions for the band gap energy (8/277).

system	calculated	predicted
Ag2O12U2V2_ICSD_81576	2.60 eV	2.25 eV
Ag2O2Pd1_ICSD_51499	0.33 eV	0.68 eV
Ag2O3S1_ICSD_4433	2.91 eV	1.63 eV
Ag2O3Se1_ICSD_78388	2.55 eV	1.73 eV
Ag2O3Si1_ICSD_36589	1.29 eV	1.42 eV
Ag2O3Ti1_ICSD_84970	1.71 eV	1.49 eV
Ag2O3_ICSD_59193	0.40 eV	0.66 eV
Ag2O4S1_ICSD_69096	2.21 eV	2.06 eV
Ag2O4Se1_ICSD_413089	1.38 eV	1.51 eV
Ag2O4Te1_ICSD_414464	0.48 eV	1.08 eV
Ag2O6P1V1_ICSD_73580	2.18 eV	2.09 eV
Ag2O6Te2_ICSD_415472	1.52 eV	1.53 eV
Ag2O6Te2_ICSD_417354	1.83 eV	1.61 eV
Ag2O7P2Pb1_ICSD_93967	3.08 eV	2.27 eV
Ag2O7P2Zn1_ICSD_90917	2.97 eV	2.69 eV
Ag2O7S2_ICSD_423166	3.23 eV	2.53 eV
Ag2O7W2_ICSD_31028	2.73 eV	2.35 eV
Ag2P2S11Ti2_ICSD_84606	1.22 eV	1.47 eV
Ag2Rb1S4Sb1_ICSD_82145	1.11 eV	1.37 eV
Ag2S1_ICSD_262637	0.39 eV	0.81 eV
Ag2S1_ICSD_98452	1.35 eV	0.96 eV
Ag2S1_ICSD_98453	0.92 eV	1.07 eV
Ag2S3Si1_ICSD_180764	1.82 eV	1.57 eV
Ag2S3Te1_ICSD_85135	1.55 eV	1.17 eV
Ag2S4Sn1Zn1_ICSD_605734	0.47 eV	0.71 eV
Ag3As1O4_ICSD_35545	0.43 eV	1.12 eV
Ag3As1S3_ICSD_36352	2.04 eV	1.32 eV
Ag3As1S3_ICSD_419203	1.41 eV	1.38 eV
Ag3As1S4_ICSD_86227	0.87 eV	1.29 eV
Ag3As1Se3_ICSD_76519	0.92 eV	0.84 eV
Ag3As1Se3_ICSD_82636	1.24 eV	1.01 eV
Ag3As2K3_ICSD_32016	1.36 eV	0.90 eV
Ag3Au1S2_ICSD_15732	0.64 eV	0.83 eV
Ag3Au1Se2_ICSD_171959	0.42 eV	0.63 eV
Ag3Au1Te2_ICSD_15733	0.58 eV	0.47 eV
Ag3B1O3_ICSD_32721	0.81 eV	0.94 eV
Ag3Br1S1_ICSD_174097	0.82 eV	1.00 eV
Ag3C1N3O6S1_ICSD_23513	2.61 eV	2.53 eV
Ag3C6Cd1N6Rb1_ICSD_75503	3.41 eV	3.32 eV
Ag3C6Co1N6_ICSD_16959	4.23 eV	3.41 eV
Ag3C6H6La1N6O3_ICSD_170986	3.50 eV	3.07 eV
Ag3C6H6N6O3Tb1_ICSD_249343	3.37 eV	3.56 eV
Ag3C6K1Mn1N6_ICSD_262507	1.86 eV	2.55 eV
Ag3C6K2N6Na1_ICSD_59725	3.43 eV	3.55 eV
Ag3C6K2N6Na1_ICSD_65699	3.39 eV	3.53 eV
Ag3Ce1K2Te4_ICSD_86678	0.98 eV	0.88 eV
Ag3Co2O6Sb1_ICSD_174288	0.50 eV	0.77 eV
Ag3Cs1S2_ICSD_1033	0.76 eV	0.90 eV
Ag3Cs1Se5Tb2_ICSD_93684	1.41 eV	1.26 eV
Ag3Cu1S2_ICSD_163982	0.57 eV	0.73 eV

Supplementary Table 615. Five-fold cross validated predictions for the band gap energy (9/277).

system	calculated	predicted
Ag3Cu1S2_ICSD_163983	0.75 eV	0.75 eV
Ag3F14Hf2_ICSD_65178	1.28 eV	2.83 eV
Ag3Fe1O8V2_ICSD_166890	2.01 eV	1.90 eV
Ag3Ge3P6Sn2_ICSD_52575	0.31 eV	0.45 eV
Ag3I1S1_ICSD_201004	0.99 eV	0.92 eV
Ag3In1O8P2_ICSD_245001	1.99 eV	1.90 eV
Ag3In2O12P3_ICSD_245002	2.01 eV	2.26 eV
Ag3K1Se2_ICSD_402643	0.31 eV	0.40 eV
Ag3K1Te2_ICSD_402230	0.55 eV	0.54 eV
Ag3Li1O2_ICSD_4204	0.87 eV	1.01 eV
Ag3N1O3S1_ICSD_25523	1.52 eV	1.58 eV
Ag3N1O3Se1_ICSD_33581	0.55 eV	1.07 eV
Ag3Na1O2_ICSD_9627	0.82 eV	0.94 eV
Ag3Na1S2_ICSD_73198	1.10 eV	0.88 eV
Ag3O4P1_ICSD_76968	0.87 eV	1.27 eV
Ag3O4Ru1_ICSD_59924	0.89 eV	1.08 eV
Ag3O4Sb1_ICSD_417675	0.24 eV	0.64 eV
Ag3O4V1_ICSD_249417	1.37 eV	1.34 eV
Ag3O4V1_ICSD_417470	1.47 eV	1.45 eV
Ag3P11_ICSD_26563	0.91 eV	0.88 eV
Ag3P1S4_ICSD_416585	1.44 eV	1.52 eV
Ag3P1Se4_ICSD_97760	0.55 eV	0.85 eV
Ag3P2S8Y1_ICSD_417658	2.26 eV	1.94 eV
Ag3P6Si3Sn2_ICSD_52595	0.38 eV	0.65 eV
Ag3P7Sn1_ICSD_411041	0.73 eV	0.68 eV
Ag3Rb1S2_ICSD_1034	0.60 eV	0.76 eV
Ag3Rb1Se2_ICSD_90795	0.38 eV	0.46 eV
Ag3Rb1Se5Sm2_ICSD_91095	0.23 eV	0.87 eV
Ag3Rb1Te2_ICSD_90872	0.62 eV	0.56 eV
Ag3S2Tl1_ICSD_75976	1.11 eV	0.95 eV
Ag3S3Sb1_ICSD_181518	1.31 eV	1.41 eV
Ag3S3Sb1_ICSD_33714	1.84 eV	1.36 eV
Ag3S6Sb2Tl3_ICSD_160100	1.61 eV	1.26 eV
Ag4C4N4S4_ICSD_159147	2.60 eV	2.52 eV
Ag4Cd1Ge2S7_ICSD_95121	1.06 eV	1.11 eV
Ag4I1O4P1_ICSD_245791	1.28 eV	1.37 eV
Ag4I2O4Se1_ICSD_418902	1.50 eV	1.56 eV
Ag4K2S3_ICSD_863	0.55 eV	0.91 eV
Ag4K2Se3_ICSD_54102	0.38 eV	0.51 eV
Ag4Mn1S6Sb2_ICSD_156764	1.59 eV	1.30 eV
Ag4Mn1S6Sb2_ICSD_38360	0.20 eV	0.95 eV
Ag4Mn3O8_ICSD_414178	0.85 eV	1.14 eV
Ag4N2O2S1_ICSD_23111	0.78 eV	1.06 eV
Ag4O4S1Te1_ICSD_421880	1.07 eV	1.18 eV
Ag4O4Si1_ICSD_418314	0.72 eV	0.70 eV
Ag4P2S6_ICSD_24782	2.02 eV	1.71 eV
Ag4P2Se6_ICSD_1727	1.07 eV	0.94 eV
Ag4Rb2S3_ICSD_409727	0.59 eV	0.81 eV
Ag4S8Sn3_ICSD_164435	0.42 eV	0.91 eV
Ag5Ba2La1S6_ICSD_67894	1.66 eV	1.27 eV

Supplementary Table 616. Five-fold cross validated predictions for the band gap energy (10/277).

system	calculated	predicted
Ag5Ba2S6Y1_ICSD_659302	1.68 eV	1.35 eV
Ag5Cl2P1S4_ICSD_416587	1.70 eV	1.58 eV
Ag5Cl5Hg4P8_ICSD_416360	1.58 eV	1.25 eV
Ag5Cs1Se3_ICSD_90871	0.22 eV	0.71 eV
Ag5O4P1Se1_ICSD_420342	0.81 eV	0.95 eV
Ag5O4P1Te1_ICSD_420343	0.97 eV	0.94 eV
Ag5O4Si1_ICSD_165377	0.92 eV	0.86 eV
Ag5S4Sb1_ICSD_36347	1.05 eV	1.09 eV
Ag6Ba1O4_ICSD_9288	1.06 eV	0.96 eV
Ag6Cl1F3Mo2O7_ICSD_413082	2.51 eV	2.29 eV
Ag6Ge10P12_ICSD_70055	0.59 eV	0.53 eV
Ag6Ge2O7_ICSD_404356	0.25 eV	0.55 eV
Ag6K2S4_ICSD_73581	1.53 eV	1.09 eV
Ag6O4Sr1_ICSD_10359	0.95 eV	1.04 eV
Ag6O7Si2_ICSD_404355	0.55 eV	0.71 eV
Ag7As1S6_ICSD_604743	0.80 eV	1.14 eV
Ag7F31Zr6_ICSD_92446	4.26 eV	2.88 eV
Ag7P1S6_ICSD_601451	1.23 eV	1.20 eV
Ag7P1Se6_ICSD_54055	0.66 eV	0.70 eV
Ag7P3S11_ICSD_414335	1.70 eV	1.74 eV
Ag8Ge1S6_ICSD_100079	0.82 eV	0.84 eV
Ag8S6Si1_ICSD_1054	0.97 eV	1.00 eV
Ag8S6Sn1_ICSD_42533	0.59 eV	0.64 eV
Ag8S6Ti1_ICSD_95648	0.87 eV	1.12 eV
Ag9Ge2I1O8_ICSD_380303	0.47 eV	0.74 eV
Al10H2O16_ICSD_23651	4.28 eV	4.72 eV
Al12Ca1O19_ICSD_34394	4.33 eV	4.55 eV
Al12Ca1O27Si4_ICSD_91233	5.24 eV	4.95 eV
Al12Ca8O24S2_ICSD_67589	3.72 eV	3.84 eV
Al12Ca8O24Te2_ICSD_86156	3.13 eV	3.11 eV
Al12Cd8O24S2_ICSD_78368	2.79 eV	2.77 eV
Al12Cd8O24Te2_ICSD_86155	2.65 eV	2.69 eV
Al12Nd4O32Pb8_ICSD_406531	3.00 eV	3.00 eV
Al12O19Sr1_ICSD_2006	4.45 eV	4.49 eV
Al12O24S2Sr8_ICSD_67590	3.34 eV	3.62 eV
Al12O24Sr8Te2_ICSD_82609	2.83 eV	3.07 eV
Al14O25Sr4_ICSD_88527	3.95 eV	4.00 eV
Al1As1Cu1O5_ICSD_91551	1.69 eV	1.62 eV
Al1As1F1Na1O4_ICSD_30205	3.46 eV	3.90 eV
Al1As1H4O6_ICSD_170740	3.66 eV	4.01 eV
Al1As1O4_ICSD_24512	4.26 eV	3.70 eV
Al1As1O4_ICSD_67228	3.87 eV	3.82 eV
Al1As1_ICSD_606008	1.50 eV	1.19 eV
Al1As1_ICSD_67771	1.66 eV	1.18 eV
Al1As2K1O7_ICSD_79711	3.03 eV	3.30 eV
Al1As2K2Na1_ICSD_73280	1.05 eV	0.99 eV
Al1As2Li1O7_ICSD_161498	3.04 eV	3.37 eV
Al1As2Li3Mo2O14_ICSD_260199	2.82 eV	2.49 eV
Al1As2Na1O7_ICSD_75142	2.74 eV	3.19 eV
Al1As2Na3_ICSD_63149	0.90 eV	0.95 eV

Supplementary Table 617. Five-fold cross validated predictions for the band gap energy (11/277).

system	calculated	predicted
Al1As3Ca3_ICSD_32727	0.72 eV	0.72 eV
Al1As3Cl4S5_ICSD_415503	2.26 eV	2.19 eV
Al1As4Ca3Na3_ICSD_402309	0.93 eV	0.82 eV
Al1Au1O2_ICSD_95663	0.96 eV	1.18 eV
Al1B1Ba1F2O3_ICSD_409663	6.00 eV	5.42 eV
Al1B1Ba1F2O3_ICSD_91316	6.04 eV	5.45 eV
Al1B1Ca1O4_ICSD_27647	4.71 eV	4.74 eV
Al1B1H3Na1O10P2_ICSD_409427	5.78 eV	5.26 eV
Al1B1Li2O4_ICSD_50612	4.84 eV	4.87 eV
Al1B1Mg1O4_ICSD_34349	6.01 eV	5.42 eV
Al1B1O3_ICSD_30538	5.83 eV	5.68 eV
Al1B1O4Pb1_ICSD_98572	3.09 eV	3.31 eV
Al1B2Li1O5_ICSD_51314	5.41 eV	5.36 eV
Al1B2Li3O6_ICSD_51754	4.82 eV	5.03 eV
Al1B3Ca1O7_ICSD_161813	5.39 eV	5.55 eV
Al1Ba1F5_ICSD_37033	7.32 eV	7.05 eV
Al1Ba1F5_ICSD_80565	7.19 eV	7.13 eV
Al1Ba1H1Si1_ICSD_162869	0.69 eV	0.70 eV
Al1Ba1La1O4_ICSD_62490	4.39 eV	3.91 eV
Al1Ba2Cu2F11_ICSD_50483	2.51 eV	2.83 eV
Al1Ba2In1O5_ICSD_33805	2.06 eV	2.42 eV
Al1Ba3F9_ICSD_72718	6.58 eV	6.44 eV
Al1Ba3H1O4_ICSD_280520	4.06 eV	3.50 eV
Al1Be1H5_ICSD_156311	3.27 eV	4.19 eV
Al1Be1H5_ICSD_156312	3.59 eV	4.22 eV
Al1Be1La3S7_ICSD_606164	2.04 eV	1.69 eV
Al1Be1Na3O8Si2_ICSD_4334	4.33 eV	4.67 eV
Al1Bi1O3_ICSD_158756	1.53 eV	1.80 eV
Al1Bi1O3_ICSD_185508	2.96 eV	2.31 eV
Al1Bi2Br1Cl4Te2_ICSD_174525	0.94 eV	1.39 eV
Al1Br1Cl4Sb2Te2_ICSD_174524	0.60 eV	1.51 eV
Al1Br4Cs1_ICSD_83435	4.25 eV	3.98 eV
Al1Br4Cu1_ICSD_165608	2.73 eV	2.66 eV
Al1Br4Tl1_ICSD_419829	4.14 eV	3.32 eV
Al1Br7Se1_ICSD_401396	2.17 eV	2.22 eV
Al1C1F5O3Sr2_ICSD_201803	5.06 eV	5.66 eV
Al1C1H2K1O5_ICSD_153303	5.08 eV	4.75 eV
Al1C1H2Na1O5_ICSD_100140	5.01 eV	4.75 eV
Al1C1O1Sc1_ICSD_419683	0.65 eV	1.52 eV
Al1C8H24P1Sn2_ICSD_163054	3.26 eV	3.21 eV
Al1Ca1F14Mg3Na3_ICSD_168054	6.84 eV	6.90 eV
Al1Ca1F5_ICSD_171399	7.13 eV	7.24 eV
Al1Ca1F5_ICSD_69563	7.15 eV	7.06 eV
Al1Ca1F6Li1_ICSD_150332	7.65 eV	7.35 eV
Al1Ca1F6Na1_ICSD_80542	7.12 eV	7.03 eV
Al1Ca1H1O5Si1_ICSD_12127	5.25 eV	4.88 eV
Al1Ca1H1Si1_ICSD_162867	0.37 eV	1.08 eV
Al1Ca1H5_ICSD_156313	0.73 eV	2.58 eV
Al1Ca1H5_ICSD_156314	3.28 eV	3.32 eV
Al1Ca1H5_ICSD_172034	2.93 eV	3.26 eV

Supplementary Table 618. Five-fold cross validated predictions for the band gap energy (12/277).

system	calculated	predicted
Al1Ca1O5Ta1_ICSD_50718	4.07 eV	3.23 eV
Al1Ca1O5Ta1_ICSD_99001	2.68 eV	3.55 eV
Al1Ca2Cl1F2H8O12S2_ICSD_80437	5.52 eV	5.20 eV
Al1Ca2F7H2O1_ICSD_182268	5.44 eV	5.77 eV
Al1Ca2F7_ICSD_100308	7.13 eV	7.21 eV
Al1Ca2F9Pb1_ICSD_180336	5.78 eV	4.95 eV
Al1Ca2Na5O16P4_ICSD_400310	4.66 eV	4.28 eV
Al1Ca3N3_ICSD_410579	2.45 eV	1.75 eV
Al1Ca3Sb3_ICSD_36363	0.29 eV	0.35 eV
Al1Cd1Ce3S7_ICSD_606335	2.38 eV	2.29 eV
Al1Cd1F6Na1_ICSD_80559	4.71 eV	4.71 eV
Al1Cd1La3S7_ICSD_606339	2.32 eV	2.32 eV
Al1Ce1H6_ICSD_247040	2.22 eV	2.17 eV
Al1Ce1O3_ICSD_245264	0.10 eV	2.49 eV
Al1Ce1O3_ICSD_245267	3.95 eV	3.15 eV
Al1Ce1O3_ICSD_245562	2.97 eV	2.25 eV
Al1Ce3Cr1S7_ICSD_606421	0.09 eV	1.14 eV
Al1Ce3Mg1S7_ICSD_606475	2.37 eV	2.28 eV
Al1Ce3S7V1_ICSD_606506	1.22 eV	1.07 eV
Al1Ce3S7Zn1_ICSD_606507	2.56 eV	2.46 eV
Al1Cl1H6O4_ICSD_425880	5.13 eV	4.76 eV
Al1Cl1O1_ICSD_27812	2.40 eV	3.99 eV
Al1Cl3H12O6_ICSD_22071	5.14 eV	4.81 eV
Al1Cl3_ICSD_39566	5.27 eV	4.38 eV
Al1Cl4Cs1_ICSD_8118	5.65 eV	5.16 eV
Al1Cl4Cu1_ICSD_165607	3.27 eV	2.91 eV
Al1Cl4Cu1_ICSD_35050	3.16 eV	3.10 eV
Al1Cl4Ga1_ICSD_62232	4.01 eV	4.34 eV
Al1Cl4In1_ICSD_170790	4.11 eV	4.01 eV
Al1Cl4Li1_ICSD_35277	5.55 eV	5.21 eV
Al1Cl4N1S2_ICSD_27210	2.11 eV	2.62 eV
Al1Cl4Na1_ICSD_71158	5.15 eV	4.90 eV
Al1Cl4Tl1_ICSD_419828	4.65 eV	4.06 eV
Al1Cl6I1_ICSD_26510	2.26 eV	2.56 eV
Al1Cl6I3_ICSD_26403	1.75 eV	1.67 eV
Al1Cl6N1S2_ICSD_14181	1.67 eV	2.29 eV
Al1Cl7S1_ICSD_35685	2.63 eV	2.47 eV
Al1Cl7Se1_ICSD_9064	0.43 eV	2.14 eV
Al1Cl7Te1_ICSD_26043	3.29 eV	2.75 eV
Al1Cl7Te1_ICSD_59133	3.18 eV	2.75 eV
Al1Cl8Nb1_ICSD_62029	2.28 eV	2.49 eV
Al1Co1Dy3S7_ICSD_606550	1.39 eV	1.58 eV
Al1Co1La3S7_ICSD_606601	1.55 eV	1.49 eV
Al1Co1Pr3S7_ICSD_606645	1.51 eV	1.05 eV
Al1Co1S7Y3_ICSD_606649	1.43 eV	1.42 eV
Al1Cr1La3S7_ICSD_606794	0.09 eV	1.17 eV
Al1Cs1Cu1F6_ICSD_240292	1.94 eV	2.48 eV
Al1Cs1F4_ICSD_10012	6.95 eV	6.81 eV
Al1Cs1H8N4_ICSD_2537	2.79 eV	3.21 eV
Al1Cs1O2_ICSD_28372	4.49 eV	3.54 eV

Supplementary Table 619. Five-fold cross validated predictions for the band gap energy (13/277).

system	calculated	predicted
Al1Cs1O4Si1_ICSD_160822	1.05 eV	3.46 eV
Al1Cs1O4Si1_ICSD_186607	2.73 eV	3.11 eV
Al1Cs1O4Si1_ICSD_186610	3.98 eV	3.41 eV
Al1Cs1Te3_ICSD_300180	1.33 eV	1.35 eV
Al1Cs2F6Na1_ICSD_41801	6.67 eV	6.55 eV
Al1Cs3Ge2O7_ICSD_412140	3.39 eV	3.18 eV
Al1Cs6K3Sb4_ICSD_300152	0.65 eV	0.65 eV
Al1Cs6Sb3_ICSD_300128	0.61 eV	0.61 eV
Al1Cu1F6K1_ICSD_59003	2.16 eV	2.48 eV
Al1Cu1O2_ICSD_32630	2.00 eV	1.74 eV
Al1Cu1O2_ICSD_95661	2.03 eV	1.81 eV
Al1Cu1S2_ICSD_187058	2.07 eV	1.89 eV
Al1Cu1Se2_ICSD_603539	1.23 eV	1.18 eV
Al1Cu1Te2_ICSD_28735	1.29 eV	0.98 eV
Al1Dy3Fe1S7_ICSD_607296	1.64 eV	1.54 eV
Al1Dy3Ni1S7_ICSD_607332	1.57 eV	1.48 eV
Al1F1Na1O4P1_ICSD_40522	5.44 eV	5.27 eV
Al1F1O4Sr3_ICSD_50736	4.05 eV	3.83 eV
Al1F2Na5O8P2_ICSD_62645	4.68 eV	4.66 eV
Al1F3H2O2Pb1_ICSD_79740	4.49 eV	4.01 eV
Al1F3K2O4S1_ICSD_161272	5.78 eV	5.43 eV
Al1F3_ICSD_130021	7.42 eV	7.55 eV
Al1F3_ICSD_202681	7.51 eV	7.40 eV
Al1F3_ICSD_29131	7.43 eV	7.49 eV
Al1F3_ICSD_36034	7.64 eV	7.28 eV
Al1F3_ICSD_72174	7.31 eV	7.37 eV
Al1F3_ICSD_79816	7.42 eV	6.93 eV
Al1F4H4N1_ICSD_33539	6.79 eV	6.66 eV
Al1F4K1_ICSD_16413	6.93 eV	6.98 eV
Al1F4K1_ICSD_166825	6.92 eV	6.92 eV
Al1F4K1_ICSD_285	6.78 eV	6.66 eV
Al1F4K1_ICSD_60525	6.89 eV	6.82 eV
Al1F4Na1_ICSD_166361	6.66 eV	6.65 eV
Al1F4Na1_ICSD_20572	5.78 eV	6.41 eV
Al1F4Rb1_ICSD_54120	6.91 eV	6.98 eV
Al1F4Rb1_ICSD_54123	6.95 eV	6.91 eV
Al1F4Rb1_ICSD_77914	6.85 eV	6.81 eV
Al1F4Tl1_ICSD_200637	4.26 eV	4.24 eV
Al1F4Tl1_ICSD_202455	4.24 eV	4.12 eV
Al1F5Fe1_ICSD_78012	3.94 eV	3.21 eV
Al1F5H10N2O1_ICSD_201652	6.12 eV	5.97 eV
Al1F5H2K2O1_ICSD_81863	5.98 eV	6.27 eV
Al1F5H4Mg1O2_ICSD_411650	5.81 eV	5.70 eV
Al1F5K2_ICSD_81864	6.29 eV	5.87 eV
Al1F5Mn1_ICSD_73812	4.50 eV	4.49 eV
Al1F5Mn1_ICSD_9912	4.50 eV	4.49 eV
Al1F5Tl2_ICSD_25616	4.09 eV	3.69 eV
Al1F6H12N3_ICSD_96591	5.73 eV	5.68 eV
Al1F6H18N6Ru1_ICSD_91763	4.38 eV	3.49 eV
Al1F6H6K1O2_ICSD_69434	6.93 eV	6.51 eV

Supplementary Table 620. Five-fold cross validated predictions for the band gap energy (14/277).

system	calculated	predicted
Al1F6H8N2Na1_ICSD_249157	6.49 eV	6.37 eV
Al1F6K2Li1_ICSD_27672	7.22 eV	7.04 eV
Al1F6K2Li1_ICSD_408553	7.45 eV	6.97 eV
Al1F6K2Li1_ICSD_48149	7.22 eV	7.06 eV
Al1F6K2Na1_ICSD_164216	6.83 eV	6.64 eV
Al1F6K2Na1_ICSD_34201	6.84 eV	6.37 eV
Al1F6K3_ICSD_262078	6.31 eV	6.73 eV
Al1F6Li1Na2_ICSD_280906	7.07 eV	6.83 eV
Al1F6Li1Pd1_ICSD_73132	2.96 eV	3.13 eV
Al1F6Li1Sr1_ICSD_164563	7.33 eV	7.31 eV
Al1F6Li1Sr1_ICSD_68905	7.31 eV	7.36 eV
Al1F6Li1Yb1_ICSD_411131	2.63 eV	3.73 eV
Al1F6Li3_ICSD_34672	7.90 eV	7.58 eV
Al1F6Li3_ICSD_85171	7.60 eV	7.65 eV
Al1F6Na1Rb2_ICSD_290318	6.80 eV	6.68 eV
Al1F6Na3_ICSD_30201	5.74 eV	6.38 eV
Al1F6Na3_ICSD_74210	5.91 eV	6.49 eV
Al1F6Pd1Rb1_ICSD_78749	2.43 eV	3.42 eV
Al1F7Mg1Na2_ICSD_33509	6.66 eV	6.69 eV
Al1F7Mg1Na2_ICSD_33510	6.64 eV	6.62 eV
Al1F7Mg1Na2_ICSD_33511	6.66 eV	6.67 eV
Al1F7Na2Ni1_ICSD_72289	4.60 eV	3.84 eV
Al1F7Na2Zn1_ICSD_400729	5.89 eV	6.11 eV
Al1Fe1La3S7_ICSD_607565	1.99 eV	1.28 eV
Al1Fe1O3_ICSD_203203	1.75 eV	2.28 eV
Al1Fe1O5P1_ICSD_74760	3.32 eV	3.14 eV
Al1Fe1S7Tb3_ICSD_607622	1.66 eV	1.52 eV
Al1Fe2V1_ICSD_57832	0.77 eV	0.58 eV
Al1Gd1O3_ICSD_59848	4.96 eV	4.28 eV
Al1Gd3Mg1S7_ICSD_607881	2.34 eV	2.12 eV
Al1Gd3Mn1S7_ICSD_607887	0.80 eV	1.26 eV
Al1Gd3S7V1_ICSD_607921	0.88 eV	1.16 eV
Al1Gd3S7Zn1_ICSD_607922	2.21 eV	2.18 eV
Al1Ge1Li1O4_ICSD_67238	3.11 eV	3.69 eV
Al1Ge1Li1_ICSD_152087	0.15 eV	0.58 eV
Al1Ge1Li3O5_ICSD_72098	2.14 eV	3.24 eV
Al1Ge1O5Y1_ICSD_32744	3.77 eV	3.95 eV
Al1Ge2Nd1O7_ICSD_35144	3.14 eV	3.33 eV
Al1H12N3O15_ICSD_96764	3.53 eV	3.35 eV
Al1H12N3O15_ICSD_96765	3.40 eV	3.07 eV
Al1H1O10Si4_ICSD_33924	5.39 eV	4.24 eV
Al1H1O2_ICSD_166340	5.90 eV	4.91 eV
Al1H1O2_ICSD_173074	3.91 eV	4.06 eV
Al1H1O2_ICSD_173076	3.93 eV	4.04 eV
Al1H1O2_ICSD_59609	5.56 eV	4.82 eV
Al1H1O4Si1_ICSD_85555	0.63 eV	3.56 eV
Al1H1Si1Sr1_ICSD_162868	0.61 eV	0.81 eV
Al1H24Li1Mg10_ICSD_158274	3.59 eV	3.30 eV
Al1H2Li1O5Si1_ICSD_161497	4.88 eV	4.76 eV
Al1H2Na3O9P2_ICSD_84643	4.51 eV	4.55 eV

Supplementary Table 621. Five-fold cross validated predictions for the band gap energy (15/277).

system	calculated	predicted
Al1H3O3_ICSD_164050	5.09 eV	4.98 eV
Al1H3O3_ICSD_181006	5.73 eV	5.09 eV
Al1H3O3_ICSD_181008	5.80 eV	5.23 eV
Al1H3O3_ICSD_240781	4.68 eV	4.89 eV
Al1H3O3_ICSD_26830	3.63 eV	4.41 eV
Al1H3_ICSD_15225	2.46 eV	3.08 eV
Al1H3_ICSD_182534	3.10 eV	3.12 eV
Al1H3_ICSD_182535	3.17 eV	3.50 eV
Al1H3_ICSD_182536	3.23 eV	3.18 eV
Al1H4K1O14P4_ICSD_63033	5.63 eV	5.42 eV
Al1H4K1_ICSD_99082	5.04 eV	4.04 eV
Al1H4K1_ICSD_99083	4.76 eV	3.88 eV
Al1H4Li1_ICSD_22247	4.58 eV	4.18 eV
Al1H4Na1_ICSD_165838	1.88 eV	3.70 eV
Al1H4Na1_ICSD_99257	4.67 eV	3.41 eV
Al1H5Mg1_ICSD_165987	3.28 eV	3.58 eV
Al1H5O9P2_ICSD_34613	5.53 eV	5.39 eV
Al1H5Sr1_ICSD_156315	3.55 eV	3.37 eV
Al1H6I1O4_ICSD_424556	4.31 eV	3.76 eV
Al1H6K2Li1_ICSD_152890	2.48 eV	2.56 eV
Al1H6K2Li1_ICSD_245318	2.61 eV	2.92 eV
Al1H6K3_ICSD_153683	1.84 eV	3.07 eV
Al1H6K3_ICSD_153684	2.17 eV	2.32 eV
Al1H6La1_ICSD_247038	2.18 eV	2.39 eV
Al1H6Li1Mg1_ICSD_165986	3.37 eV	3.55 eV
Al1H6Li1Na2_ICSD_152893	2.83 eV	2.54 eV
Al1H6Li3_ICSD_99217	3.61 eV	4.10 eV
Al1H6Nd1_ICSD_247044	2.01 eV	2.09 eV
Al1H6Pr1_ICSD_247042	2.10 eV	2.08 eV
Al1H8K1N4_ICSD_2538	2.92 eV	2.48 eV
Al1H8N4Rb1_ICSD_40168	0.20 eV	2.26 eV
Al1I3O9_ICSD_152758	3.33 eV	3.29 eV
Al1I3_ICSD_391247	3.26 eV	2.57 eV
Al1I4In1_ICSD_418802	3.00 eV	2.91 eV
Al1I4Na1_ICSD_400521	3.42 eV	3.19 eV
Al1I4Tl1_ICSD_419827	3.44 eV	3.00 eV
Al1I7Te1_ICSD_401395	1.87 eV	1.85 eV
Al1I8P1_ICSD_35403	1.47 eV	2.19 eV
Al1In1S3_ICSD_8257	2.43 eV	2.24 eV
Al1K1Mo2O8_ICSD_28018	3.78 eV	3.46 eV
Al1K1O2_ICSD_151883	3.76 eV	3.89 eV
Al1K1O2_ICSD_169481	4.00 eV	3.62 eV
Al1K1O2_ICSD_262975	2.93 eV	3.64 eV
Al1K1O4Si1_ICSD_34350	4.51 eV	4.48 eV
Al1K1O4Si1_ICSD_83449	4.52 eV	4.49 eV
Al1K1O7P2_ICSD_2888	5.31 eV	5.14 eV
Al1K1O8S2_ICSD_6305	5.49 eV	4.94 eV
Al1K1O8Si3_ICSD_83536	0.18 eV	3.19 eV
Al1K1Sb4_ICSD_300157	0.25 eV	0.45 eV
Al1K1Te2_ICSD_411171	1.89 eV	1.59 eV

Supplementary Table 622. Five-fold cross validated predictions for the band gap energy (16/277).

system	calculated	predicted
Al1K1Te2_ICSD_44703	1.70 eV	1.75 eV
Al1K2Li1P2_ICSD_77275	1.90 eV	1.54 eV
Al1K2Na1P2_ICSD_73279	1.51 eV	1.33 eV
Al1K6Na3Sb4_ICSD_401209	0.75 eV	0.75 eV
Al1La1O3_ICSD_153821	3.66 eV	3.54 eV
Al1La1O3_ICSD_153830	3.24 eV	3.23 eV
Al1La1O3_ICSD_153836	3.13 eV	3.17 eV
Al1La1O3_ICSD_180416	3.29 eV	2.87 eV
Al1La1O3_ICSD_180417	3.50 eV	3.48 eV
Al1La1O3_ICSD_90549	3.15 eV	3.26 eV
Al1La3Mg1S7_ICSD_608298	2.31 eV	2.36 eV
Al1La3Mn1S7_ICSD_608303	1.87 eV	1.70 eV
Al1La3Ni1S7_ICSD_608317	0.09 eV	1.07 eV
Al1La3S7Ti1_ICSD_608322	1.23 eV	1.62 eV
Al1La3S7V1_ICSD_608323	1.16 eV	1.13 eV
Al1La3S7Zn1_ICSD_608324	2.63 eV	2.38 eV
Al1Li1Mo2O8_ICSD_16175	3.54 eV	3.36 eV
Al1Li1Na2P2_ICSD_402083	1.89 eV	1.54 eV
Al1Li1O12P4_ICSD_74860	5.74 eV	5.62 eV
Al1Li1O2_ICSD_23815	4.59 eV	5.00 eV
Al1Li1O2_ICSD_28288	6.11 eV	5.41 eV
Al1Li1O4Si1_ICSD_22011	4.85 eV	4.98 eV
Al1Li1O4Si1_ICSD_22014	4.80 eV	4.88 eV
Al1Li1O4Si1_ICSD_32595	4.85 eV	4.98 eV
Al1Li1O4Si1_ICSD_38167	4.70 eV	4.81 eV
Al1Li1O4Si1_ICSD_97909	5.05 eV	4.94 eV
Al1Li1O6Si2_ICSD_158512	5.33 eV	5.13 eV
Al1Li1O6Si2_ICSD_30521	5.40 eV	4.99 eV
Al1Li1S2_ICSD_608360	4.11 eV	3.28 eV
Al1Li1Se2_ICSD_280225	3.02 eV	2.49 eV
Al1Li1Si1_ICSD_413257	0.17 eV	0.61 eV
Al1Li1Te2_ICSD_162672	2.44 eV	1.92 eV
Al1Li3N2_ICSD_25565	2.94 eV	2.81 eV
Al1Li5O4_ICSD_1037	4.80 eV	4.64 eV
Al1Li5O4_ICSD_42697	4.67 eV	4.68 eV
Al1Mg1O5P1_ICSD_156822	5.04 eV	4.99 eV
Al1Mn1Pr3S7_ICSD_608498	1.74 eV	1.49 eV
Al1Mn1Ti2_ICSD_185877	0.23 eV	0.38 eV
Al1Mo1O7V1_ICSD_280775	2.29 eV	2.77 eV
Al1Mo2Na1O8_ICSD_281210	3.58 eV	3.01 eV
Al1Mo2O8Tl1_ICSD_250339	3.78 eV	3.09 eV
Al1Mo4S8_ICSD_36564	0.52 eV	0.53 eV
Al1N1Na3O9P3_ICSD_75366	5.00 eV	4.88 eV
Al1N1_ICSD_105522	4.43 eV	3.43 eV
Al1N1_ICSD_163950	3.27 eV	3.10 eV
Al1N1_ICSD_163951	3.26 eV	3.81 eV
Al1N1_ICSD_188512	3.31 eV	4.02 eV
Al1N1_ICSD_31169	4.06 eV	3.37 eV
Al1Na1O2_ICSD_22216	4.73 eV	3.92 eV
Al1Na1O2_ICSD_79404	3.82 eV	3.88 eV

Supplementary Table 623. Five-fold cross validated predictions for the band gap energy (17/277).

system	calculated	predicted
Al1Na1O4Si1_ICSD_34884	4.48 eV	4.51 eV
Al1Na1O4Si1_ICSD_36324	4.37 eV	4.42 eV
Al1Na1O6Si2_ICSD_162546	5.24 eV	4.83 eV
Al1Na1O7P2_ICSD_400462	5.29 eV	5.23 eV
Al1Na1Se2_ICSD_300173	2.25 eV	2.13 eV
Al1Na1Se2_ICSD_44704	2.17 eV	2.02 eV
Al1Na1Te2_ICSD_44701	1.28 eV	1.39 eV
Al1Na3P2_ICSD_402081	1.40 eV	1.41 eV
Al1Nb1O4_ICSD_24078	3.61 eV	3.12 eV
Al1Nd1O3_ICSD_90573	3.70 eV	3.67 eV
Al1Ni1Pr3S7_ICSD_608833	0.10 eV	1.00 eV
Al1Ni1S7Y3_ICSD_608836	1.59 eV	1.50 eV
Al1O2Rb1_ICSD_28373	3.38 eV	3.77 eV
Al1O2Tl1_ICSD_29010	2.21 eV	1.94 eV
Al1O3Pr1_ICSD_90566	3.96 eV	3.48 eV
Al1O3Y1_ICSD_27100	3.50 eV	4.16 eV
Al1O3Y1_ICSD_4115	5.55 eV	4.43 eV
Al1O4P1_ICSD_158619	5.64 eV	5.57 eV
Al1O4P1_ICSD_159272	4.96 eV	4.60 eV
Al1O4P1_ICSD_159273	2.92 eV	4.76 eV
Al1O4P1_ICSD_162670	4.92 eV	5.21 eV
Al1O4P1_ICSD_24511	5.41 eV	5.04 eV
Al1O4P1_ICSD_261306	5.30 eV	4.70 eV
Al1O4P1_ICSD_279582	5.33 eV	5.07 eV
Al1O4P1_ICSD_417475	5.37 eV	4.80 eV
Al1O4P1_ICSD_50101	5.63 eV	5.60 eV
Al1O4P1_ICSD_66996	4.67 eV	4.86 eV
Al1O4P1_ICSD_66998	3.19 eV	4.59 eV
Al1O4P1_ICSD_66999	4.67 eV	3.71 eV
Al1O4P1_ICSD_9643	5.48 eV	5.17 eV
Al1O4P1_ICSD_98384	5.48 eV	5.22 eV
Al1O4Rb1Si1_ICSD_160823	3.81 eV	3.75 eV
Al1O4Rb1Si1_ICSD_4335	3.49 eV	3.64 eV
Al1O4Si1Tl1_ICSD_89722	3.52 eV	3.36 eV
Al1O4Ta1_ICSD_33885	4.17 eV	3.23 eV
Al1O4Ta1_ICSD_67676	3.46 eV	3.26 eV
Al1O4W1_ICSD_4164	1.44 eV	2.71 eV
Al1O9P3_ICSD_26759	5.62 eV	5.63 eV
Al1P1S4_ICSD_15910	2.58 eV	2.48 eV
Al1P1_ICSD_67770	1.96 eV	1.57 eV
Al1P1_ICSD_67783	1.63 eV	1.55 eV
Al1Rb6Sb3_ICSD_300217	0.28 eV	0.56 eV
Al1Sb1_ICSD_609290	1.23 eV	0.85 eV
Al1Sb9Yb11_ICSD_186084	0.61 eV	0.46 eV
Al1Se2Tl1_ICSD_100130	0.57 eV	1.10 eV
Al1Si1Te3_ICSD_75001	1.23 eV	1.22 eV
Al22Na2O34_ICSD_34905	4.51 eV	4.60 eV
Al2As2Cs2O7_ICSD_154363	3.66 eV	3.54 eV
Al2As3K3_ICSD_60950	1.19 eV	1.03 eV
Al2As4Ca3_ICSD_60161	1.12 eV	1.03 eV

Supplementary Table 624. Five-fold cross validated predictions for the band gap energy (18/277).

system	calculated	predicted
Al2As4Sr3_ICSD_423787	1.15 eV	0.97 eV
Al2B2Ba1O7_ICSD_409171	4.67 eV	4.63 eV
Al2B2Ca1O7_ICSD_86785	4.83 eV	4.62 eV
Al2B2Cs2O7_ICSD_423471	4.22 eV	4.12 eV
Al2B2K2O7_ICSD_409420	4.57 eV	4.45 eV
Al2B2Na2O7_ICSD_93389	4.35 eV	4.59 eV
Al2B2O7Sr1_ICSD_89423	4.64 eV	4.65 eV
Al2B2O7Sr1_ICSD_91031	4.70 eV	4.62 eV
Al2Ba1Ge2O8_ICSD_1282	3.47 eV	3.37 eV
Al2Ba1O4_ICSD_21080	3.95 eV	4.08 eV
Al2Ba1O4_ICSD_246027	4.09 eV	3.96 eV
Al2Ba1O7Sb2_ICSD_154362	3.42 eV	3.06 eV
Al2Ba1S4_ICSD_35136	3.65 eV	3.22 eV
Al2Ba1Te4_ICSD_41165	1.54 eV	1.45 eV
Al2Ba2Ca1F14Mg1_ICSD_20393	7.19 eV	6.82 eV
Al2Ba3F12_ICSD_413546	6.72 eV	6.94 eV
Al2Ba3N4_ICSD_410578	1.57 eV	1.65 eV
Al2Ba3O12Si3_ICSD_27386	3.29 eV	4.04 eV
Al2Ba7O19Sc6_ICSD_39442	2.51 eV	3.20 eV
Al2Be1O4_ICSD_34806	6.13 eV	5.60 eV
Al2Be2Cl2Na8O24Si8_ICSD_34665	4.64 eV	4.70 eV
Al2Be3O18Si6_ICSD_202091	5.32 eV	5.52 eV
Al2Bi2Br12_ICSD_414262	2.59 eV	2.45 eV
Al2Bi2Cl12_ICSD_414261	3.83 eV	3.20 eV
Al2Br6N2S2_ICSD_38378	1.64 eV	1.87 eV
Al2Br6N2Se2_ICSD_82802	0.28 eV	1.68 eV
Al2Br6_ICSD_83433	3.14 eV	3.67 eV
Al2Br6_ICSD_83434	3.90 eV	3.44 eV
Al2Br7K1_ICSD_2592	4.11 eV	3.78 eV
Al2Br8Ti1_ICSD_39243	2.97 eV	2.75 eV
Al2Br8Ti1_ICSD_40904	2.92 eV	2.73 eV
Al2C2Mg1_ICSD_85739	1.75 eV	1.05 eV
Al2C6H22O2Si2_ICSD_172440	5.03 eV	4.42 eV
Al2Ca1Cl8_ICSD_56730	5.42 eV	4.56 eV
Al2Ca1H4O10Si2_ICSD_80837	5.12 eV	4.77 eV
Al2Ca1H6O13Si3_ICSD_30967	5.03 eV	4.84 eV
Al2Ca1H8_ICSD_246482	4.52 eV	3.12 eV
Al2Ca1O4_ICSD_172780	4.98 eV	4.38 eV
Al2Ca1O4_ICSD_41661	4.38 eV	4.39 eV
Al2Ca1S4_ICSD_46016	3.53 eV	3.07 eV
Al2Ca2Fe1H1O13Si3_ICSD_63661	2.31 eV	2.11 eV
Al2Ca2H1Mn1O13Si3_ICSD_26354	1.09 eV	2.32 eV
Al2Ca2O7Si1_ICSD_24588	4.29 eV	4.24 eV
Al2Ca2O9Sn2_ICSD_260890	2.44 eV	2.92 eV
Al2Ca3F14Na2_ICSD_202657	7.11 eV	7.03 eV
Al2Ca3F8H6O8S1_ICSD_31248	5.73 eV	5.70 eV
Al2Ca3H12O12_ICSD_34227	4.11 eV	4.32 eV
Al2Ca3N4_ICSD_280347	2.24 eV	2.25 eV
Al2Ca3N4_ICSD_280348	2.19 eV	2.19 eV
Al2Ca3N4_ICSD_280349	2.54 eV	2.20 eV

Supplementary Table 625. Five-fold cross validated predictions for the band gap energy (19/277).

system	calculated	predicted
Al2Ca3O12Si3_ICSD_94614	5.00 eV	4.64 eV
Al2Ca5Sb6_ICSD_183853	0.18 eV	0.28 eV
Al2Cd1O4_ICSD_183382	2.80 eV	2.95 eV
Al2Cd1S4_ICSD_25634	2.78 eV	2.26 eV
Al2Cd1S4_ICSD_43025	2.54 eV	2.55 eV
Al2Cd1Se4_ICSD_174192	2.05 eV	1.66 eV
Al2Cd1Se4_ICSD_51423	1.32 eV	1.50 eV
Al2Cd1Te4_ICSD_25640	1.66 eV	1.43 eV
Al2Cd3O12Si3_ICSD_27384	3.06 eV	3.05 eV
Al2Cl10N2P2_ICSD_391165	3.83 eV	3.43 eV
Al2Cl1F25Sr10_ICSD_202936	6.42 eV	6.22 eV
Al2Cl1H6Li1O6_ICSD_83509	5.13 eV	4.89 eV
Al2Cl2O5Sr3_ICSD_68365	4.21 eV	4.20 eV
Al2Cl6O1Sn1_ICSD_411777	3.90 eV	3.26 eV
Al2Cl8Co1_ICSD_22143	3.26 eV	3.02 eV
Al2Cl8Cu1_ICSD_80107	1.27 eV	2.59 eV
Al2Cl8Mg1_ICSD_62046	5.25 eV	4.85 eV
Al2Cl8Ni1_ICSD_417872	2.88 eV	2.93 eV
Al2Cl8Pd1_ICSD_15595	2.16 eV	2.79 eV
Al2Cl8Te4_ICSD_10322	1.34 eV	1.89 eV
Al2Cl8Ti1_ICSD_39565	3.16 eV	3.39 eV
Al2Cl8V1_ICSD_415951	3.15 eV	2.85 eV
Al2Co1O4_ICSD_290133	3.96 eV	3.17 eV
Al2Co3O12Si3_ICSD_27383	3.84 eV	3.38 eV
Al2Cs2O7Sb2_ICSD_154361	3.64 eV	3.47 eV
Al2Cs2O9P2_ICSD_280275	4.91 eV	4.49 eV
Al2Cs6Te6_ICSD_300181	2.16 eV	1.81 eV
Al2Eu1S4_ICSD_607464	3.06 eV	2.63 eV
Al2Eu1Se4_ICSD_607466	2.65 eV	1.90 eV
Al2F12Li3Na3_ICSD_31110	7.09 eV	7.13 eV
Al2F16Sr5_ICSD_411613	6.93 eV	6.90 eV
Al2F1K4Nb11O20_ICSD_65738	0.65 eV	1.49 eV
Al2F2Ge1O4_ICSD_409714	3.98 eV	4.46 eV
Al2F2K2O3_ICSD_421736	4.00 eV	4.54 eV
Al2Fe1S4_ICSD_607619	1.57 eV	1.27 eV
Al2Fe3O12Si3_ICSD_80671	3.33 eV	2.83 eV
Al2Gd2O7Sr1_ICSD_33580	3.59 eV	3.49 eV
Al2Ge1H2O6_ICSD_78792	3.83 eV	3.73 eV
Al2Ge2O7_ICSD_201750	3.35 eV	3.50 eV
Al2H12O12Sr3_ICSD_20529	3.88 eV	4.15 eV
Al2H12O15Se3_ICSD_72871	4.77 eV	4.00 eV
Al2H3O9P3_ICSD_74527	6.27 eV	4.25 eV
Al2H4K1Ni1O14P3_ICSD_75347	4.06 eV	3.74 eV
Al2H4Li2O14Si4_ICSD_88917	4.85 eV	4.61 eV
Al2H4Na2O12Si3_ICSD_31309	4.54 eV	4.32 eV
Al2H4O9Si2_ICSD_30996	3.85 eV	3.61 eV
Al2H4O9Si2_ICSD_63192	2.64 eV	3.35 eV
Al2H4O9Si2_ICSD_80083	3.91 eV	3.44 eV
Al2H4O9Si2_ICSD_87771	1.75 eV	3.79 eV
Al2H4O9Si2_ICSD_98133	4.93 eV	4.14 eV

Supplementary Table 626. Five-fold cross validated predictions for the band gap energy (20/277).

system	calculated	predicted
Al2H8Mg1_ICSD_152538	4.41 eV	3.89 eV
Al2Hg1S4_ICSD_25635	2.02 eV	1.95 eV
Al2Hg1S4_ICSD_608160	1.30 eV	1.82 eV
Al2Hg1Se4_ICSD_25638	1.43 eV	1.07 eV
Al2Hg1Se4_ICSD_608163	0.39 eV	1.07 eV
Al2Hg1Te4_ICSD_25641	1.10 eV	1.23 eV
Al2I12Sb2_ICSD_38253	2.26 eV	1.97 eV
Al2K2O24P8_ICSD_260827	5.19 eV	5.20 eV
Al2K2O7Sb2_ICSD_280310	3.50 eV	3.12 eV
Al2K6O6_ICSD_74968	2.90 eV	2.68 eV
Al2K6Se6_ICSD_300172	2.38 eV	2.01 eV
Al2K6Te6_ICSD_300168	2.15 eV	1.71 eV
Al2Mg1O4_ICSD_161057	4.85 eV	4.33 eV
Al2Mg1O4_ICSD_182859	5.11 eV	5.01 eV
Al2Mg1S4_ICSD_107308	2.01 eV	2.49 eV
Al2Mg1S4_ICSD_79672	3.06 eV	3.06 eV
Al2Mg1Se4_ICSD_83363	1.06 eV	1.55 eV
Al2Mg2Se5_ICSD_41928	1.07 eV	1.69 eV
Al2Mg3O12Si3_ICSD_24941	4.94 eV	5.04 eV
Al2Mn1S4_ICSD_608509	1.77 eV	1.72 eV
Al2Mn1Te4_ICSD_608538	1.03 eV	1.18 eV
Al2Mn3O12Si3_ICSD_52396	3.57 eV	2.95 eV
Al2N2O3Si1Sr1_ICSD_408170	3.71 eV	3.90 eV
Al2N4Sr3_ICSD_74824	1.91 eV	1.89 eV
Al2Na6Se6_ICSD_300166	0.55 eV	1.38 eV
Al2Na6Te6_ICSD_300163	0.16 eV	1.14 eV
Al2Na7Sb5_ICSD_48168	0.28 eV	0.58 eV
Al2O10Rb2Si3_ICSD_180324	4.38 eV	3.96 eV
Al2O12P3Rb3_ICSD_280211	4.42 eV	4.56 eV
Al2O12P3Tl3_ICSD_280212	3.45 eV	3.50 eV
Al2O12S3_ICSD_73249	6.04 eV	5.44 eV
Al2O12Si3Sr3_ICSD_27385	4.44 eV	4.19 eV
Al2O12W3_ICSD_73878	4.39 eV	3.58 eV
Al2O12W3_ICSD_73879	4.38 eV	3.58 eV
Al2O3_ICSD_161061	5.31 eV	4.96 eV
Al2O3_ICSD_161062	4.25 eV	3.87 eV
Al2O3_ICSD_169722	4.23 eV	4.83 eV
Al2O3_ICSD_173014	2.62 eV	4.12 eV
Al2O3_ICSD_30026	5.85 eV	4.77 eV
Al2O3_ICSD_82504	4.46 eV	4.52 eV
Al2O3_ICSD_84375	4.83 eV	4.72 eV
Al2O4Pb1_ICSD_33532	3.95 eV	3.36 eV
Al2O4Sr1_ICSD_160296	4.15 eV	4.06 eV
Al2O4Sr1_ICSD_160298	4.06 eV	4.00 eV
Al2O4Zn1_ICSD_94159	4.49 eV	4.14 eV
Al2O5Si1_ICSD_76936	4.84 eV	5.18 eV
Al2O5Si1_ICSD_85742	5.44 eV	4.90 eV
Al2O5Si1_ICSD_85745	5.55 eV	5.22 eV
Al2O5Si1_ICSD_85746	5.46 eV	4.52 eV
Al2O5Ti1_ICSD_27681	3.43 eV	3.35 eV

Supplementary Table 627. Five-fold cross validated predictions for the band gap energy (21/277).

system	calculated	predicted
Al2O6Rb6_ICSD_74969	2.66 eV	2.66 eV
Al2O7Rb2Sb2_ICSD_154360	3.59 eV	3.44 eV
Al2O9Pb2Si2_ICSD_159977	2.77 eV	3.14 eV
Al2O9Tb4_ICSD_164882	4.23 eV	4.04 eV
Al2O9Y4_ICSD_51076	4.32 eV	4.06 eV
Al2Os1_ICSD_58108	0.51 eV	0.55 eV
Al2P4Sr3_ICSD_409134	1.33 eV	1.04 eV
Al2Pb1S4_ICSD_609026	2.60 eV	2.57 eV
Al2Ru1_ICSD_58156	0.15 eV	0.17 eV
Al2Ru1_ICSD_609234	0.10 eV	0.21 eV
Al2S3_ICSD_609250	2.43 eV	2.95 eV
Al2S3_ICSD_73220	3.09 eV	2.93 eV
Al2S4Sr1_ICSD_609259	3.90 eV	3.38 eV
Al2S4Zn1_ICSD_609280	0.24 eV	1.90 eV
Al2S4Zn1_ICSD_76278	2.47 eV	2.52 eV
Al2Sb6Sr5_ICSD_62304	0.76 eV	0.50 eV
Al2Se3_ICSD_14373	1.76 eV	1.84 eV
Al2Se4Sr1_ICSD_49732	2.97 eV	2.38 eV
Al2Se4Zn1_ICSD_25636	2.28 eV	1.84 eV
Al2Se4Zn1_ICSD_609325	1.58 eV	1.60 eV
Al2Te3_ICSD_406353	1.86 eV	1.57 eV
Al2Te4Zn1_ICSD_25639	1.71 eV	1.57 eV
Al2Te5_ICSD_78941	0.97 eV	1.12 eV
Al3B4Gd1O12_ICSD_100831	5.23 eV	4.65 eV
Al3B4Nd1O12_ICSD_20800	3.93 eV	3.90 eV
Al3B4Nd1O12_ICSD_6175	3.71 eV	4.25 eV
Al3B4O12Pr1_ICSD_160892	4.28 eV	4.07 eV
Al3B4O12Y1_ICSD_20223	5.35 eV	4.88 eV
Al3Bi5Cl12_ICSD_201993	1.82 eV	2.17 eV
Al3Br12La1_ICSD_72281	3.46 eV	3.29 eV
Al3Br12Pr1_ICSD_72282	2.34 eV	2.98 eV
Al3C2O5Sc3_ICSD_420953	0.67 eV	1.70 eV
Al3C3Sc1_ICSD_43477	0.30 eV	0.31 eV
Al3C3Sc1_ICSD_62308	0.29 eV	0.33 eV
Al3C3Tm1_ICSD_606281	0.13 eV	0.63 eV
Al3Ca2H1O13Si3_ICSD_9245	5.04 eV	4.81 eV
Al3Cl12Tb1_ICSD_410939	4.22 eV	3.92 eV
Al3Cs2F12Na1_ICSD_646	6.90 eV	6.82 eV
Al3Cu2Ni3Zr12_ICSD_166652	0.10 eV	0.38 eV
Al3F12K2Na1_ICSD_40178	6.79 eV	6.89 eV
Al3F12Na1Rb2_ICSD_40177	6.79 eV	6.84 eV
Al3F14Na5_ICSD_26419	6.56 eV	6.72 eV
Al3F19Pb5_ICSD_203224	5.17 eV	4.30 eV
Al3Gd1O8Pb2_ICSD_404479	2.98 eV	3.02 eV
Al3H14Na5_ICSD_246195	2.60 eV	3.41 eV
Al3H6K1O14S2_ICSD_18141	5.49 eV	5.31 eV
Al3Ho1O8Pb2_ICSD_67819	2.99 eV	2.99 eV
Al3I1Te3_ICSD_66030	1.97 eV	1.76 eV
Al3Lu1O8Pb2_ICSD_67820	2.99 eV	2.93 eV
Al3Mo1_ICSD_105517	0.11 eV	0.18 eV

Supplementary Table 628. Five-fold cross validated predictions for the band gap energy (22/277).

system	calculated	predicted
Al3O12Sc2Y3_ICSD_67055	4.27 eV	4.02 eV
Al3O27P9_ICSD_409479	5.52 eV	5.31 eV
Al4As8K12_ICSD_300121	0.47 eV	0.87 eV
Al4B2Co1O10_ICSD_1975	3.73 eV	3.47 eV
Al4Ba1S7_ICSD_33237	3.13 eV	3.08 eV
Al4Be1Mg1O8_ICSD_36361	5.53 eV	5.40 eV
Al4Bi2O9_ICSD_26807	2.80 eV	3.05 eV
Al4Bi2S8_ICSD_408439	2.63 eV	2.40 eV
Al4Bi2Se8_ICSD_408440	2.25 eV	1.83 eV
Al4Bi4Cl16S4_ICSD_414154	2.84 eV	2.46 eV
Al4Bi4Cl16Se4_ICSD_414155	2.60 eV	2.23 eV
Al4Bi4Cl16Te4_ICSD_411714	2.15 eV	2.06 eV
Al4C1N3O1_ICSD_409682	0.16 eV	2.78 eV
Al4C1O4_ICSD_18204	3.82 eV	3.99 eV
Al4C3_ICSD_606173	1.32 eV	1.16 eV
Al4Ca1H2O12Si2_ICSD_34855	4.53 eV	4.79 eV
Al4Ca1O7_ICSD_16191	4.00 eV	4.17 eV
Al4Cl14Te4_ICSD_10323	1.44 eV	2.48 eV
Al4F9K3O8P2_ICSD_79700	5.46 eV	5.35 eV
Al4H1O14Ta3_ICSD_67673	3.51 eV	3.33 eV
Al4H3O15P3_ICSD_6193	5.52 eV	4.82 eV
Al4H6O18P6_ICSD_415615	1.89 eV	4.69 eV
Al4K12P8_ICSD_300130	0.70 eV	1.23 eV
Al4La17N33Si9_ICSD_416358	1.41 eV	1.91 eV
Al4Mg2O18Si5_ICSD_86347	4.82 eV	5.07 eV
Al4Mn13O28Sb2Si2_ICSD_12137	1.10 eV	1.43 eV
Al4Na4P12Sr8_ICSD_409319	0.31 eV	0.90 eV
Al4O15Sr6Y2_ICSD_262993	4.01 eV	3.85 eV
Al4O7Sr1_ICSD_16751	3.73 eV	3.76 eV
Al4O7Sr1_ICSD_34803	3.24 eV	3.81 eV
Al5B1O9_ICSD_167310	4.94 eV	5.18 eV
Al5C3N1_ICSD_14398	0.82 eV	1.30 eV
Al5C3N1_ICSD_36303	1.44 eV	0.96 eV
Al5Er3O12_ICSD_170147	4.76 eV	4.52 eV
Al5Eu3O12_ICSD_245326	0.74 eV	1.54 eV
Al5Gd3O12_ICSD_23849	4.11 eV	4.37 eV
Al5Ho3O12_ICSD_33603	4.67 eV	4.65 eV
Al5Lu3O12_ICSD_182354	5.08 eV	4.65 eV
Al5Lu3O12_ICSD_23846	4.59 eV	5.26 eV
Al5Na1O12Ti2_ICSD_15346	3.09 eV	3.31 eV
Al5O12Tb3_ICSD_33602	4.47 eV	4.47 eV
Al5O12Y3_ICSD_31496	4.50 eV	4.48 eV
Al5O12Yb3_ICSD_170159	0.62 eV	0.43 eV
Al6Br2Ge6Li8O24_ICSD_87991	3.65 eV	3.68 eV
Al6Br2Ge6Na8O24_ICSD_65665	3.82 eV	3.77 eV
Al6Br2Na8O24Si6_ICSD_417676	4.60 eV	4.42 eV
Al6Ca4O13_ICSD_16177	3.64 eV	3.96 eV
Al6Ca4O13_ICSD_245370	3.95 eV	3.74 eV
Al6Ca4O16S1_ICSD_28480	4.67 eV	4.43 eV
Al6Ca4O16W1_ICSD_28481	3.97 eV	3.47 eV

Supplementary Table 629. Five-fold cross validated predictions for the band gap energy (23/277).

system	calculated	predicted
Al6Ca5O14_ICSD_1714	4.22 eV	4.04 eV
Al6Cl2Ge6Li8O24_ICSD_87990	4.14 eV	3.85 eV
Al6Cl2Ge6Na8O24_ICSD_65664	4.05 eV	3.92 eV
Al6Cl2K8O24Si6_ICSD_41191	4.57 eV	4.27 eV
Al6Cl2Li8O24Si6_ICSD_41186	5.25 eV	5.04 eV
Al6Cl2Na8O24Si6_ICSD_29443	4.69 eV	4.67 eV
Al6F21Na1Rb2_ICSD_68555	7.00 eV	7.01 eV
Al6Ge6I2Li8O24_ICSD_87992	3.14 eV	3.38 eV
Al6Ge6I2Na8O24_ICSD_65666	3.35 eV	3.32 eV
Al6I2Na8O24Si6_ICSD_68960	4.17 eV	3.80 eV
Al6O11Sr2_ICSD_97713	4.14 eV	4.06 eV
Al6O16S1Sr4_ICSD_28482	4.38 eV	4.23 eV
Al6O16Sr4W1_ICSD_28483	3.61 eV	3.49 eV
Al7S12Tl3_ICSD_72324	2.80 eV	2.49 eV
Al7Te10_ICSD_62659	1.39 eV	1.68 eV
Al8Be1Mg3O16_ICSD_31227	5.24 eV	5.24 eV
Al8Mo3_ICSD_58002	0.10 eV	0.12 eV
Al9Fe2H1O24Si4_ICSD_16769	3.23 eV	3.19 eV
Ar1_ICSD_426923	8.46 eV	7.93 eV
Ar1_ICSD_77918	8.45 eV	7.52 eV
As1.01Ce1Se0.99_ICSD_249802	0.17 eV	0.32 eV
As11Rb3_ICSD_412872	1.48 eV	0.87 eV
As12Ce1Fe4_ICSD_610003	0.16 eV	0.29 eV
As12Ce1Ru4_ICSD_610013	0.15 eV	0.21 eV
As12Os4Th1_ICSD_611145	0.13 eV	0.37 eV
As14Ba3_ICSD_1404	0.10 eV	0.44 eV
As1Au1K2S4_ICSD_85681	1.13 eV	1.57 eV
As1Au1Na2_ICSD_23254	0.65 eV	0.64 eV
As1B1Ba1O5_ICSD_404439	3.44 eV	3.90 eV
As1B1Ba3O3_ICSD_402682	1.52 eV	1.54 eV
As1B1O4_ICSD_413436	3.86 eV	4.05 eV
As1B1O4_ICSD_413438	4.28 eV	4.01 eV
As1B1O5Pb1_ICSD_404328	3.38 eV	3.36 eV
As1B1_ICSD_181292	1.20 eV	1.00 eV
As1B2P1_ICSD_181293	1.06 eV	1.30 eV
As1B6_ICSD_68151	2.66 eV	1.76 eV
As1Ba1Li1_ICSD_56445	0.59 eV	0.67 eV
As1Ba1O6V1_ICSD_83629	2.67 eV	2.72 eV
As1Be1Cs1O4_ICSD_74027	3.88 eV	3.73 eV
As1Be1Li1_ICSD_100004	1.00 eV	0.78 eV
As1Be1Na1_ICSD_100091	1.28 eV	0.90 eV
As1Bi1Ca2O6_ICSD_91475	3.23 eV	2.75 eV
As1Bi1Mg2O6_ICSD_73895	3.37 eV	3.10 eV
As1Bi1Mn1O5_ICSD_59721	2.44 eV	1.85 eV
As1Bi1Ni1O5_ICSD_92916	2.84 eV	2.13 eV
As1Bi1O4_ICSD_27199	1.76 eV	2.61 eV
As1Bi1O4_ICSD_30636	2.74 eV	1.95 eV
As1Bi1O5Pb1_ICSD_419124	2.30 eV	2.46 eV
As1Br1Hg3O4_ICSD_411758	1.86 eV	1.88 eV
As1Br1Hg3S4_ICSD_280330	1.52 eV	1.54 eV

Supplementary Table 630. Five-fold cross validated predictions for the band gap energy (24/277).

system	calculated	predicted
As1Br1Hg3Se4_ICSD_280331	1.27 eV	1.21 eV
As1Br3Ca3_ICSD_426	1.67 eV	1.84 eV
As1Br3F6S1_ICSD_202502	2.51 eV	2.35 eV
As1Br3F6Te1_ICSD_200689	3.08 eV	2.34 eV
As1Br3F6_ICSD_33811	1.61 eV	2.13 eV
As1Br3_ICSD_26774	3.07 eV	2.74 eV
As1C1Cl2F9S1_ICSD_60141	3.81 eV	3.75 eV
As1C1F11N2S3_ICSD_81839	3.26 eV	3.62 eV
As1C1F5N2O1S2_ICSD_16422	2.83 eV	3.24 eV
As1C1F6H5O1_ICSD_408996	5.02 eV	4.50 eV
As1C1F9H3N1O1_ICSD_41317	4.74 eV	3.91 eV
As1C2Cs1H6Se2_ICSD_171201	2.23 eV	2.60 eV
As1C2F6H5O2_ICSD_407344	3.06 eV	4.12 eV
As1C2F6N1O2_ICSD_410737	4.64 eV	4.08 eV
As1C3H10I1_ICSD_171203	3.58 eV	3.18 eV
As1C3H6N1_ICSD_170721	4.37 eV	3.93 eV
As1C3N3_ICSD_35330	4.23 eV	3.99 eV
As1Ca1Co1H1O5_ICSD_240725	2.38 eV	2.72 eV
As1Ca1Cu1_ICSD_49741	0.15 eV	0.32 eV
As1Ca1F1Mg1O4_ICSD_26000	3.91 eV	3.85 eV
As1Ca1F1Mg1O4_ICSD_56862	3.93 eV	3.95 eV
As1Ca1H1Ni1O5_ICSD_202422	2.33 eV	2.43 eV
As1Ca1H1O5Zn1_ICSD_63285	3.10 eV	3.38 eV
As1Ca1Na1O4_ICSD_262123	3.59 eV	3.51 eV
As1Ca1Rb1_ICSD_409177	1.31 eV	0.99 eV
As1Ca2Cl1O4_ICSD_26234	3.79 eV	3.85 eV
As1Ca2F13H4_ICSD_415156	4.80 eV	5.57 eV
As1Ca2I1_ICSD_65218	1.55 eV	1.66 eV
As1Ca2_ICSD_166865	0.21 eV	0.53 eV
As1Ca3Cl3_ICSD_36002	1.84 eV	2.04 eV
As1Ca3N1_ICSD_56968	0.72 eV	0.73 eV
As1Ca3N1_ICSD_657356	0.86 eV	0.83 eV
As1Cd1Ce1O1_ICSD_88268	1.12 eV	0.71 eV
As1Cd1K1_ICSD_609963	0.26 eV	0.59 eV
As1Cd1_ICSD_432	0.17 eV	0.13 eV
As1Cd2Cl2_ICSD_26013	1.02 eV	1.30 eV
As1Cd2F1O4_ICSD_202983	1.83 eV	2.30 eV
As1Cd3Cl3_ICSD_23306	1.48 eV	1.41 eV
As1Ce1F1O4_ICSD_166934	2.02 eV	2.98 eV
As1Ce1O4_ICSD_280982	0.82 eV	2.14 eV
As1Cl13Nb1P1_ICSD_25110	2.55 eV	2.45 eV
As1Cl13P1Sb1_ICSD_25109	2.12 eV	1.75 eV
As1Cl13P1Ta1_ICSD_25111	2.58 eV	2.33 eV
As1Cl1Co2O4_ICSD_902	2.91 eV	2.39 eV
As1Cl1Cu2O4_ICSD_901	1.09 eV	1.13 eV
As1Cl1Hg3O4_ICSD_411757	2.06 eV	1.93 eV
As1Cl1Hg3S4_ICSD_280329	1.56 eV	1.57 eV
As1Cl1O2Pb1_ICSD_66246	3.33 eV	3.09 eV
As1Cl2Hg2_ICSD_39930	1.30 eV	1.41 eV
As1Cl3F5O1P1_ICSD_82761	4.48 eV	3.47 eV

Supplementary Table 631. Five-fold cross validated predictions for the band gap energy (25/277).

system	calculated	predicted
As1Cl3F6S1_ICSD_60076	3.77 eV	3.44 eV
As1Cl3F6Se1_ICSD_66843	3.72 eV	2.99 eV
As1Cl3_ICSD_35133	4.01 eV	2.97 eV
As1Cl5_ICSD_412103	1.44 eV	2.19 eV
As1Co1Li1O4_ICSD_155305	2.74 eV	2.52 eV
As1Co1S1_ICSD_36395	0.13 eV	0.59 eV
As1Co1S1_ICSD_610107	0.87 eV	0.95 eV
As1Co1S1_ICSD_69129	1.09 eV	0.85 eV
As1Co1Se1_ICSD_41731	0.46 eV	0.69 eV
As1Cr1O4_ICSD_62132	1.95 eV	1.63 eV
As1Cs1F4_ICSD_413041	4.60 eV	4.80 eV
As1Cs1F6_ICSD_408070	5.13 eV	5.12 eV
As1Cs1H12Mg1O10_ICSD_260150	4.17 eV	3.98 eV
As1Cs1O5Ti1_ICSD_280315	3.19 eV	3.25 eV
As1Cs1Se2_ICSD_65299	1.33 eV	1.28 eV
As1Cs2Li1O4_ICSD_36645	3.71 eV	3.52 eV
As1Cs2Na1O4_ICSD_36533	3.52 eV	3.39 eV
As1Cs3O4_ICSD_412392	3.57 eV	3.30 eV
As1Cs3Se4_ICSD_404082	1.46 eV	1.56 eV
As1Cs5P4S12_ICSD_260946	2.41 eV	2.12 eV
As1Cu1F7_ICSD_413972	2.19 eV	1.95 eV
As1Cu1H1O5Zn1_ICSD_160894	1.80 eV	1.55 eV
As1Cu1K2_ICSD_43936	1.12 eV	1.06 eV
As1Cu1Na2_ICSD_43937	0.87 eV	0.82 eV
As1Cu1O4Pb1_ICSD_61677	1.96 eV	1.54 eV
As1Cu1O4Tl1_ICSD_50458	1.19 eV	1.25 eV
As1Cu1O4Tl2_ICSD_407563	1.78 eV	1.46 eV
As1Cu1O4Tl2_ICSD_50456	1.59 eV	1.39 eV
As1Cu1S1_ICSD_23826	0.13 eV	0.18 eV
As1Cu1S1_ICSD_240925	0.14 eV	0.21 eV
As1Cu1Se2_ICSD_42884	0.33 eV	0.60 eV
As1Cu1Sr1_ICSD_107943	0.18 eV	0.33 eV
As1Cu3S4_ICSD_95547	0.22 eV	0.81 eV
As1Cu4K1S4_ICSD_75430	1.57 eV	1.23 eV
As1Dy1O4_ICSD_16512	3.63 eV	3.57 eV
As1Dy1Pd1_ICSD_656672	0.10 eV	0.12 eV
As1Er1O1Zn1_ICSD_420203	1.12 eV	1.08 eV
As1Eu1Na1S4_ICSD_262583	1.23 eV	1.54 eV
As1F11H1N1S1Xe1_ICSD_249654	2.52 eV	2.97 eV
As1F11H1N1Te1Xe1_ICSD_171447	2.36 eV	2.40 eV
As1F12I1_ICSD_249129	0.60 eV	2.62 eV
As1F3_ICSD_35132	5.16 eV	4.23 eV
As1F5H6N2_ICSD_412507	2.68 eV	3.41 eV
As1F5N4S4_ICSD_14083	1.75 eV	1.64 eV
As1F5_ICSD_65477	4.57 eV	4.49 eV
As1F6H2Li1O1_ICSD_59367	4.18 eV	4.47 eV
As1F6H3O1_ICSD_61236	4.82 eV	4.35 eV
As1F6H6Li1O3_ICSD_416608	3.80 eV	4.20 eV
As1F6I1Se6_ICSD_35351	1.56 eV	1.92 eV
As1F6I3_ICSD_15527	1.33 eV	1.70 eV

Supplementary Table 632. Five-fold cross validated predictions for the band gap energy (26/277).

system	calculated	predicted
As1F6I5_ICSD_59115	1.38 eV	1.46 eV
As1F6In1_ICSD_417952	3.35 eV	4.18 eV
As1F6K1_ICSD_16663	4.79 eV	4.09 eV
As1F6K1_ICSD_2362	4.83 eV	4.96 eV
As1F6Li1_ICSD_74831	5.04 eV	4.60 eV
As1F6N1O2_ICSD_68906	3.19 eV	2.96 eV
As1F6N1S2_ICSD_62120	2.60 eV	2.81 eV
As1F6N2S3_ICSD_31787	0.59 eV	1.54 eV
As1F6N2S3_ICSD_4043	1.51 eV	1.59 eV
As1F6Na1_ICSD_184563	5.08 eV	4.93 eV
As1F6Na1_ICSD_184564	4.92 eV	4.96 eV
As1F6Rb1_ICSD_408069	4.94 eV	4.96 eV
As1F6Tl1_ICSD_417954	4.61 eV	3.57 eV
As1F7Kr1_ICSD_279624	2.45 eV	2.70 eV
As1F7Pb1_ICSD_411788	4.21 eV	3.53 eV
As1F7Sn1_ICSD_816	3.42 eV	3.46 eV
As1F8Sb1_ICSD_9920	3.87 eV	3.33 eV
As1F9O1S1_ICSD_10193	3.91 eV	3.82 eV
As1Fe1Li1O4_ICSD_245182	1.79 eV	2.15 eV
As1Fe1Li1_ICSD_187132	0.21 eV	0.37 eV
As1Fe1S1_ICSD_15987	0.13 eV	0.61 eV
As1Fe1S1_ICSD_185809	0.63 eV	0.68 eV
As1Fe1Te1_ICSD_610529	0.99 eV	0.68 eV
As1Ga1O4_ICSD_33256	3.10 eV	3.06 eV
As1Ga1O4_ICSD_423937	3.15 eV	3.04 eV
As1Ga1_ICSD_610543	0.29 eV	0.37 eV
As1Ga1_ICSD_67773	0.31 eV	0.51 eV
As1Gd1O1Zn1_ICSD_420206	1.07 eV	0.98 eV
As1Ge1Se1_ICSD_100828	1.45 eV	1.04 eV
As1Ge1_ICSD_610598	0.20 eV	0.36 eV
As1H1Hg1O5Zn1_ICSD_281591	1.57 eV	1.90 eV
As1H1O4Pb1_ICSD_29552	2.99 eV	3.07 eV
As1H1O5Pb1Zn1_ICSD_98385	3.37 eV	2.87 eV
As1H1O5Zn2_ICSD_34868	2.64 eV	2.89 eV
As1H2Li1O4_ICSD_62024	3.99 eV	3.70 eV
As1H2Li1O5Zn1_ICSD_409396	3.43 eV	3.53 eV
As1H2Mn1O5_ICSD_71164	1.39 eV	1.96 eV
As1H36Li3N12Se4_ICSD_409539	1.70 eV	2.73 eV
As1H4Na1O5_ICSD_4284	3.85 eV	3.76 eV
As1H4Na1O6Zn1_ICSD_407316	3.10 eV	3.41 eV
As1H6N1O4_ICSD_66206	4.13 eV	3.67 eV
As1H6N1O4_ICSD_66208	3.39 eV	3.59 eV
As1H6N1O4_ICSD_66210	3.67 eV	3.59 eV
As1Hg3I1Se4_ICSD_280332	1.25 eV	1.15 eV
As1Ho1O4_ICSD_155919	3.60 eV	3.66 eV
As1Ho1Pd1_ICSD_71619	0.11 eV	0.12 eV
As1I1Se1_ICSD_200799	1.31 eV	1.30 eV
As1I3_ICSD_56571	2.21 eV	2.07 eV
As1K1Li2_ICSD_78938	0.70 eV	0.74 eV
As1K1Mg1_ICSD_610753	1.15 eV	1.10 eV

Supplementary Table 633. Five-fold cross validated predictions for the band gap energy (27/277).

system	calculated	predicted
As1K1Mo1O6_ICSD_203218	2.53 eV	2.65 eV
As1K1Ni1O4_ICSD_63544	2.67 eV	2.52 eV
As1K1O2_ICSD_413149	3.84 eV	3.53 eV
As1K1O5Ti1_ICSD_75322	3.26 eV	3.09 eV
As1K1S5Sn1_ICSD_281038	1.49 eV	1.41 eV
As1K1Se2_ICSD_65297	1.57 eV	1.28 eV
As1K1Sn1_ICSD_610765	0.28 eV	0.38 eV
As1K1Zn1_ICSD_10459	0.46 eV	0.55 eV
As1K1Zn1_ICSD_43985	0.39 eV	0.65 eV
As1K1_ICSD_409653	0.68 eV	0.67 eV
As1K3S3_ICSD_610764	2.60 eV	2.48 eV
As1K3Se11Ta2_ICSD_413600	1.19 eV	1.23 eV
As1K3Se3_ICSD_50492	2.11 eV	1.84 eV
As1La1O1Zn1_ICSD_420204	0.80 eV	0.89 eV
As1La1O3_ICSD_423416	2.75 eV	3.21 eV
As1La1O4_ICSD_415338	2.50 eV	2.82 eV
As1La1S1_ICSD_610779	0.18 eV	0.51 eV
As1La1Te1_ICSD_280231	0.14 eV	0.33 eV
As1Li1Mg1O4_ICSD_67523	3.46 eV	3.64 eV
As1Li1Mg1_ICSD_107954	1.37 eV	0.73 eV
As1Li1Mn1O4_ICSD_245181	1.73 eV	1.97 eV
As1Li1Mo1O6_ICSD_15035	2.38 eV	2.59 eV
As1Li1Mo1O6_ICSD_59822	2.33 eV	2.32 eV
As1Li1Mo2O9_ICSD_170039	2.49 eV	2.55 eV
As1Li1Ni1O4_ICSD_245184	2.71 eV	2.46 eV
As1Li1O3_ICSD_16617	2.95 eV	3.06 eV
As1Li1O3_ICSD_202862	3.38 eV	3.22 eV
As1Li1O4Rb2_ICSD_36644	3.55 eV	3.54 eV
As1Li1O5Ti1_ICSD_172582	3.28 eV	3.15 eV
As1Li1O5V1_ICSD_90991	1.40 eV	2.11 eV
As1Li1S2_ICSD_419061	1.05 eV	1.60 eV
As1Li1Se2_ICSD_248116	0.73 eV	0.59 eV
As1Li1Se2_ICSD_248118	0.35 eV	0.85 eV
As1Li1Zn1_ICSD_74504	0.94 eV	0.51 eV
As1Li1_ICSD_26472	0.38 eV	0.58 eV
As1Li2Na1O4_ICSD_73200	3.89 eV	3.76 eV
As1Li3O4_ICSD_75927	4.13 eV	3.86 eV
As1Li3S3_ICSD_59381	2.28 eV	2.57 eV
As1Li3_ICSD_26878	0.64 eV	0.79 eV
As1Li3_ICSD_610785	0.40 eV	0.68 eV
As1Lu1O4_ICSD_2506	3.43 eV	4.15 eV
As1Mg1Na1_ICSD_610829	1.00 eV	1.07 eV
As1Mn1Na1O4_ICSD_95087	1.81 eV	2.22 eV
As1Mn1O4_ICSD_165271	0.42 eV	1.31 eV
As1Mo1O6Rb1_ICSD_280174	2.43 eV	2.73 eV
As1Na1Ni1O4_ICSD_63353	2.63 eV	2.49 eV
As1Na1O2_ICSD_413148	3.60 eV	3.29 eV
As1Na1O3_ICSD_16654	2.84 eV	2.91 eV
As1Na1O5Ti1_ICSD_421302	2.82 eV	3.03 eV
As1Na1S2_ICSD_854	1.38 eV	1.78 eV

Supplementary Table 634. Five-fold cross validated predictions for the band gap energy (28/277).

system	calculated	predicted
As1Na1Sr1_ICSD_402448	0.82 eV	0.79 eV
As1Na1Zn1_ICSD_610979	0.66 eV	0.64 eV
As1Na1_ICSD_182158	0.56 eV	0.64 eV
As1Na3S3_ICSD_645	2.52 eV	2.39 eV
As1Na3Se3_ICSD_50491	2.01 eV	1.86 eV
As1Nb2Rb3Se11_ICSD_413598	1.17 eV	1.21 eV
As1Nd1O1Zn1_ICSD_85778	0.82 eV	0.87 eV
As1Nd1O4_ICSD_155918	3.40 eV	2.79 eV
As1O1Y1Zn1_ICSD_420205	1.19 eV	0.93 eV
As1O2Rb1_ICSD_413150	3.88 eV	3.54 eV
As1O3Sb1_ICSD_37187	0.94 eV	2.21 eV
As1O4P1_ICSD_31879	4.56 eV	3.59 eV
As1O4Sb1_ICSD_23316	3.06 eV	2.31 eV
As1O4Sc1_ICSD_155920	3.52 eV	3.32 eV
As1O4Tb1_ICSD_16329	3.65 eV	3.69 eV
As1O4Tb1_ICSD_200231	3.66 eV	3.51 eV
As1O4Tl1Zn1_ICSD_74812	3.34 eV	2.87 eV
As1O4Tl3_ICSD_407561	2.56 eV	2.06 eV
As1O4Y1_ICSD_24513	3.66 eV	3.83 eV
As1O5P1_ICSD_36649	2.01 eV	3.42 eV
As1O5Rb1Sn1_ICSD_80977	1.82 eV	2.48 eV
As1O5Rb1Ti1_ICSD_280131	3.25 eV	3.15 eV
As1O5Sb1_ICSD_36650	1.78 eV	2.37 eV
As1Pd1Tb1_ICSD_656671	0.11 eV	0.11 eV
As1Rb1_ICSD_412594	0.59 eV	0.67 eV
As1Rb3Se16_ICSD_405959	0.90 eV	1.03 eV
As1Rb3Se4_ICSD_404080	1.19 eV	1.54 eV
As1Ru1Te1_ICSD_611299	1.06 eV	0.66 eV
As1S1_ICSD_180621	1.95 eV	1.97 eV
As1S2Tl1_ICSD_658371	1.71 eV	1.54 eV
As1S2_ICSD_424590	1.32 eV	1.76 eV
As1S3Tl3_ICSD_611332	1.23 eV	1.19 eV
As1S3Tl3_ICSD_79580	1.54 eV	1.41 eV
As1S4Tl3_ICSD_61057	1.68 eV	1.66 eV
As1Se3Tl3_ICSD_603666	0.76 eV	0.68 eV
As1Si1_ICSD_611404	1.31 eV	1.04 eV
As1Zn1_ICSD_431	0.50 eV	0.27 eV
As2Au1K5_ICSD_40698	0.37 eV	0.56 eV
As2B1K3_ICSD_300105	1.31 eV	1.20 eV
As2B1Rb3_ICSD_402082	1.29 eV	1.17 eV
As2B4Cl4_ICSD_80153	3.39 eV	2.68 eV
As2Ba1Cd2_ICSD_30917	0.24 eV	0.28 eV
As2Ba1Co2O8_ICSD_260062	2.63 eV	2.27 eV
As2Ba1Ga2_ICSD_380478	1.24 eV	0.65 eV
As2Ba1Mg2_ICSD_30916	1.07 eV	1.02 eV
As2Ba1Ni2O8_ICSD_27014	2.71 eV	2.21 eV
As2Ba1Zn2_ICSD_12146	0.33 eV	0.31 eV
As2Ba1_ICSD_414139	0.42 eV	0.42 eV
As2Ba2Cd1_ICSD_422941	0.41 eV	0.51 eV
As2Ba2Ge1_ICSD_35151	0.53 eV	0.53 eV

Supplementary Table 635. Five-fold cross validated predictions for the band gap energy (29/277).

system	calculated	predicted
As2Ba2Mn2O1_ICSD_75454	0.27 eV	0.68 eV
As2Ba2Se5_ICSD_60954	1.02 eV	0.98 eV
As2Ba2Zn1_ICSD_421423	0.67 eV	0.55 eV
As2Ba3O8_ICSD_404438	3.92 eV	3.41 eV
As2Ba4O1_ICSD_33905	0.67 eV	0.81 eV
As2Be1K4_ICSD_300111	0.88 eV	0.96 eV
As2Be2Ca1_ICSD_609867	0.66 eV	0.77 eV
As2Be2Mg1_ICSD_609872	0.41 eV	0.86 eV
As2Br1La3O7_ICSD_421948	3.13 eV	3.20 eV
As2Br2Cd2Hg2_ICSD_240354	1.02 eV	1.00 eV
As2Br3Hg4_ICSD_82312	0.73 eV	0.93 eV
As2Ca1Cd2_ICSD_100065	0.31 eV	0.40 eV
As2Ca1Cu1O7_ICSD_82623	1.61 eV	1.50 eV
As2Ca1F20Xe4_ICSD_412759	2.57 eV	2.85 eV
As2Ca1H4O8_ICSD_2809	3.77 eV	3.59 eV
As2Ca1H6Mn1O10_ICSD_200702	2.55 eV	2.83 eV
As2Ca1Mg2_ICSD_100041	1.26 eV	1.13 eV
As2Ca1O6_ICSD_77379	3.32 eV	3.22 eV
As2Ca1Zn2_ICSD_609920	0.72 eV	0.53 eV
As2Ca2Cd1_ICSD_422578	0.72 eV	0.58 eV
As2Ca2Cd1_ICSD_422579	0.92 eV	0.71 eV
As2Ca2H4Mn1O10_ICSD_156223	2.55 eV	2.43 eV
As2Ca2O7_ICSD_32602	3.30 eV	3.23 eV
As2Ca4O1_ICSD_68203	1.20 eV	1.22 eV
As2Cd1Hg4I4_ICSD_416973	0.49 eV	1.00 eV
As2Cd1K4_ICSD_300190	0.61 eV	0.64 eV
As2Cd1O6_ICSD_280576	1.99 eV	2.04 eV
As2Cd1Si1_ICSD_603895	0.46 eV	0.68 eV
As2Cd1Sr2_ICSD_422940	0.58 eV	0.55 eV
As2Cd1_ICSD_16037	0.22 eV	0.19 eV
As2Cd2O7_ICSD_280579	1.72 eV	2.15 eV
As2Cd2Sr1_ICSD_23249	0.22 eV	0.32 eV
As2Cd4I3_ICSD_1964	1.20 eV	1.06 eV
As2Ce1Li2_ICSD_32042	0.50 eV	0.51 eV
As2Ce1_ICSD_609997	0.10 eV	0.13 eV
As2Cl13Sb1_ICSD_26088	1.19 eV	2.03 eV
As2Cl2In2O5_ICSD_422701	3.27 eV	2.93 eV
As2Cl3F5O1_ICSD_82760	3.29 eV	3.53 eV
As2Cl3Hg3Tl1_ICSD_411520	0.83 eV	1.11 eV
As2Cl4F6_ICSD_33884	2.84 eV	3.30 eV
As2Cl6O2_ICSD_411196	2.49 eV	3.01 eV
As2Co1O6_ICSD_80350	2.23 eV	2.20 eV
As2Co2O7_ICSD_69001	2.12 eV	2.04 eV
As2Co2O8Sr1_ICSD_400764	2.64 eV	2.55 eV
As2Co7O12_ICSD_403080	1.07 eV	1.40 eV
As2Cs2F8O2_ICSD_6070	3.74 eV	4.06 eV
As2Cs2Pd1_ICSD_69647	0.96 eV	0.83 eV
As2Cs2Se9Sn1_ICSD_281267	0.66 eV	0.90 eV
As2Cs2Si1_ICSD_71225	1.02 eV	0.89 eV
As2Cs2Sn1_ICSD_71226	0.72 eV	0.67 eV

Supplementary Table 636. Five-fold cross validated predictions for the band gap energy (30/277).

system	calculated	predicted
As2Cs4Te6_ICSD_405235	0.80 eV	0.98 eV
As2Cu1K5_ICSD_40699	0.31 eV	0.55 eV
As2Cu1Na4O8_ICSD_63058	1.87 eV	1.65 eV
As2Cu1O8Zn2_ICSD_18115	1.76 eV	1.67 eV
As2Cu2O7_ICSD_162061	0.98 eV	1.03 eV
As2Cu2O7_ICSD_162062	1.05 eV	1.04 eV
As2Cu3K3_ICSD_32015	1.48 eV	1.01 eV
As2Cu3O8_ICSD_201733	0.33 eV	0.91 eV
As2Cu3O8_ICSD_24198	1.06 eV	0.98 eV
As2Cu3O8_ICSD_63057	1.12 eV	0.95 eV
As2Dy1K3S8_ICSD_420737	1.71 eV	1.81 eV
As2Eu3S8_ICSD_249612	1.49 eV	1.42 eV
As2Eu4O1_ICSD_1222	0.16 eV	0.39 eV
As2F12Hg3_ICSD_9323	2.63 eV	2.01 eV
As2F12Hg4_ICSD_35412	0.74 eV	2.22 eV
As2F12I4S2_ICSD_14074	1.22 eV	2.18 eV
As2F12Mn1O4S2_ICSD_411791	3.02 eV	3.10 eV
As2F12Mn1_ICSD_83635	4.02 eV	2.65 eV
As2F12N2S1Se2_ICSD_71357	2.26 eV	2.55 eV
As2F12N2S3_ICSD_54159	2.19 eV	2.42 eV
As2F12N2Se3_ICSD_73584	2.32 eV	2.65 eV
As2F16Mg1Xe2_ICSD_281694	2.97 eV	3.14 eV
As2F18Pb1Xe3_ICSD_391093	2.54 eV	2.52 eV
As2F7K1_ICSD_36332	4.77 eV	4.50 eV
As2F8K2O2_ICSD_9027	3.67 eV	4.17 eV
As2F8O2Rb2_ICSD_9028	3.64 eV	3.90 eV
As2Fe1Li1O7_ICSD_75180	2.40 eV	2.24 eV
As2Fe4O11_ICSD_100442	1.38 eV	1.62 eV
As2Ga1K1O7_ICSD_82391	2.71 eV	2.98 eV
As2Ga1K2Li1_ICSD_401208	0.80 eV	0.84 eV
As2Ga1K2Na1_ICSD_300129	0.84 eV	0.83 eV
As2Ga1Li1Na2_ICSD_402111	0.73 eV	0.75 eV
As2Ga1Li1O7_ICSD_161500	2.57 eV	2.98 eV
As2Ge1K2_ICSD_71222	0.82 eV	0.79 eV
As2Ge1Mg1_ICSD_182368	0.55 eV	0.82 eV
As2Ge1Te4_ICSD_68111	0.24 eV	0.33 eV
As2Ge1_ICSD_23872	0.51 eV	0.36 eV
As2Ge2Te5_ICSD_68112	0.34 eV	0.31 eV
As2Ge3Te6_ICSD_68113	0.34 eV	0.31 eV
As2Ge4Te7_ICSD_68114	0.29 eV	0.32 eV
As2Ge5Te8_ICSD_63174	0.24 eV	0.30 eV
As2H16O16Zn3_ICSD_100492	3.62 eV	3.26 eV
As2H2K1O8Sc1_ICSD_59820	3.84 eV	3.71 eV
As2H3Li1O7_ICSD_172988	1.96 eV	2.79 eV
As2H4O9Ti1_ICSD_86771	3.28 eV	3.32 eV
As2H4O9V1_ICSD_69585	2.29 eV	2.11 eV
As2H6O11U1_ICSD_411217	3.42 eV	2.88 eV
As2H8O12Zn3_ICSD_290615	3.26 eV	3.36 eV
As2Hg1K4_ICSD_402573	0.71 eV	0.75 eV
As2Hg1O6_ICSD_409490	0.74 eV	1.18 eV

Supplementary Table 637. Five-fold cross validated predictions for the band gap energy (31/277).

system	calculated	predicted
As2Hg2O6_ICSD_411230	1.24 eV	1.50 eV
As2Hg3O8_ICSD_72527	1.00 eV	1.19 eV
As2Hg4I3_ICSD_67227	0.69 eV	0.68 eV
As2Hg4O7_ICSD_391228	1.15 eV	1.33 eV
As2Hg6O8_ICSD_2604	0.49 eV	1.24 eV
As2In1K2Li1_ICSD_402147	0.65 eV	0.79 eV
As2Ir1_ICSD_610739	0.74 eV	0.57 eV
As2K2Ni1_ICSD_300120	0.78 eV	0.92 eV
As2K2Pd1_ICSD_32009	0.93 eV	0.87 eV
As2K2Pt1_ICSD_610762	0.66 eV	0.76 eV
As2K2S6Sn1_ICSD_281039	0.78 eV	1.43 eV
As2K2Si1_ICSD_40426	1.00 eV	1.01 eV
As2K3La1O8_ICSD_421979	3.75 eV	3.49 eV
As2K3Nb1O9_ICSD_202980	3.49 eV	3.16 eV
As2K3Nd1S8_ICSD_240365	1.68 eV	1.77 eV
As2K4Zn1_ICSD_409919	0.71 eV	0.70 eV
As2La1_ICSD_280294	0.50 eV	0.23 eV
As2La1_ICSD_610769	0.12 eV	0.23 eV
As2Li1O7Sc1_ICSD_161499	3.42 eV	3.46 eV
As2Li3Nd1_ICSD_49626	0.15 eV	0.34 eV
As2Mg1Si1_ICSD_182367	1.26 eV	1.07 eV
As2Mg2O7_ICSD_16885	3.05 eV	3.31 eV
As2Mg2Sr1_ICSD_610831	1.34 eV	1.16 eV
As2Mg3_ICSD_24485	0.54 eV	1.06 eV
As2Mg3_ICSD_25504	0.90 eV	1.13 eV
As2Mg3_ICSD_610824	1.44 eV	1.01 eV
As2Mn1O6_ICSD_80351	1.75 eV	1.72 eV
As2Mn1S5Tl2_ICSD_17035	1.46 eV	1.47 eV
As2Mn2O7_ICSD_69003	1.27 eV	1.33 eV
As2Mo2O12Sr1_ICSD_87794	2.57 eV	2.47 eV
As2Na1O7Sc1_ICSD_161501	3.40 eV	3.36 eV
As2Na2_ICSD_421420	0.71 eV	0.55 eV
As2Na4O7_ICSD_2887	3.21 eV	3.13 eV
As2Na4O9Ti1_ICSD_59321	3.54 eV	3.34 eV
As2Na4Te4_ICSD_300185	1.05 eV	1.15 eV
As2Nd1_ICSD_1730	0.10 eV	0.12 eV
As2Ni1O6_ICSD_80349	2.18 eV	1.96 eV
As2Ni2O7_ICSD_69002	1.51 eV	1.84 eV
As2Ni3O8_ICSD_63708	2.03 eV	2.15 eV
As2Ni3O8_ICSD_63709	2.23 eV	2.15 eV
As2O11W2_ICSD_15020	2.86 eV	2.67 eV
As2O12S3_ICSD_32586	4.57 eV	3.80 eV
As2O13Pb8_ICSD_99432	2.12 eV	2.04 eV
As2O13V4_ICSD_60781	1.28 eV	1.73 eV
As2O14Pb3Te1Zn3_ICSD_85574	3.00 eV	2.89 eV
As2O1Sr4_ICSD_33904	1.19 eV	0.93 eV
As2O1Yb4_ICSD_402951	0.65 eV	0.60 eV
As2O3_ICSD_100434	2.90 eV	2.99 eV
As2O3_ICSD_27588	2.74 eV	2.80 eV
As2O3_ICSD_409611	4.02 eV	3.07 eV

Supplementary Table 638. Five-fold cross validated predictions for the band gap energy (32/277).

system	calculated	predicted
As2O4Pb1_ICSD_65027	2.85 eV	2.54 eV
As2O4Zn1_ICSD_202249	3.53 eV	3.11 eV
As2O4_ICSD_10436	3.34 eV	2.82 eV
As2O5_ICSD_10015	1.35 eV	1.67 eV
As2O5_ICSD_987	1.45 eV	1.96 eV
As2O6Pb1_ICSD_81063	2.13 eV	2.08 eV
As2O6Pd1_ICSD_187098	1.72 eV	1.63 eV
As2O6S1_ICSD_4297	4.37 eV	3.67 eV
As2O6Sr1_ICSD_420296	3.26 eV	3.09 eV
As2O6Sr1_ICSD_420297	3.24 eV	2.96 eV
As2O6Zn3_ICSD_10400	3.71 eV	3.56 eV
As2O6Zn3_ICSD_417004	3.99 eV	3.43 eV
As2O7Ti1_ICSD_73476	2.95 eV	2.82 eV
As2O7Ti1_ICSD_73477	2.83 eV	2.73 eV
As2O8Pb3_ICSD_200414	3.30 eV	2.59 eV
As2O8Sr3_ICSD_420295	3.80 eV	3.56 eV
As2O8Zn3_ICSD_404199	2.42 eV	2.80 eV
As2O8Zn3_ICSD_404229	2.38 eV	2.60 eV
As2O9S2_ICSD_63530	4.04 eV	3.87 eV
As2O9V3_ICSD_424067	2.39 eV	1.96 eV
As2Os1_ICSD_611138	0.89 eV	0.58 eV
As2P2S7_ICSD_30706	2.64 eV	2.22 eV
As2Pr1_ICSD_611219	0.12 eV	0.10 eV
As2Pt1Rb2_ICSD_107529	0.80 eV	0.73 eV
As2Pt1_ICSD_38428	0.17 eV	0.48 eV
As2Rb2Si1_ICSD_60617	0.95 eV	1.01 eV
As2Rb2Sn1_ICSD_71223	0.63 eV	0.67 eV
As2Rh1_ICSD_42616	0.21 eV	0.36 eV
As2Ru1_ICSD_611289	0.48 eV	0.45 eV
As2S3_ICSD_185819	2.00 eV	1.80 eV
As2S6Sn1Tl2_ICSD_72907	0.72 eV	1.28 eV
As2Se3_ICSD_2600	1.34 eV	1.04 eV
As2Se3_ICSD_611373	0.60 eV	1.05 eV
As2Si1Zn1_ICSD_23707	1.12 eV	0.86 eV
As2Si1_ICSD_611405	1.09 eV	0.96 eV
As2Sr1Zn2_ICSD_23248	0.60 eV	0.51 eV
As2Te3_ICSD_30981	0.44 eV	0.44 eV
As2Te3_ICSD_41040	0.41 eV	0.35 eV
As2Te3_ICSD_68110	0.40 eV	0.44 eV
As2Yb1Zn2_ICSD_88231	0.54 eV	0.34 eV
As2Zn1_ICSD_2021	0.28 eV	0.38 eV
As2Zn3_ICSD_611608	0.17 eV	0.41 eV
As3Ba3In1_ICSD_402338	0.52 eV	0.39 eV
As3Ba3Nb1O1_ICSD_408853	1.10 eV	1.04 eV
As3Ba3O1Ta1_ICSD_280155	1.10 eV	1.14 eV
As3Ba5Cl1O12_ICSD_260065	4.00 eV	3.86 eV
As3Be3Cl1Li4O12_ICSD_74526	4.44 eV	4.33 eV
As3Bi2K3O12_ICSD_59887	3.32 eV	2.59 eV
As3Br1Cd2_ICSD_100815	0.79 eV	0.82 eV
As3Br1Cd2_ICSD_75170	0.79 eV	0.80 eV

Supplementary Table 639. Five-fold cross validated predictions for the band gap energy (33/277).

system	calculated	predicted
As3Br1Hg2_ICSD_75169	0.90 eV	0.79 eV
As3Ca2_ICSD_43876	0.16 eV	0.54 eV
As3Ca3Ga1_ICSD_60126	0.37 eV	0.45 eV
As3Ca5F1O12_ICSD_172996	3.96 eV	3.81 eV
As3Cd2I1_ICSD_40449	0.78 eV	0.77 eV
As3Cd2I1_ICSD_8216	0.78 eV	0.81 eV
As3Cl1O12Pb5_ICSD_69960	3.06 eV	2.90 eV
As3Cl1O12Sr5_ICSD_260196	3.83 eV	3.90 eV
As3Cl1O9Pb5_ICSD_100168	2.66 eV	2.55 eV
As3Cl1O9Pb5_ICSD_31863	2.02 eV	2.49 eV
As3Cs5Si1_ICSD_65716	0.59 eV	0.69 eV
As3Cs6In1_ICSD_300145	0.51 eV	0.57 eV
As3Cu4K1O12_ICSD_63059	0.84 eV	1.02 eV
As3F1O12Sr5_ICSD_260044	3.91 eV	3.84 eV
As3F6Sb1Se4_ICSD_24413	1.89 eV	1.73 eV
As3H1Sr5_ICSD_173027	0.68 eV	0.92 eV
As3H2Na1O9_ICSD_65011	2.61 eV	2.96 eV
As3H5O10_ICSD_14327	3.01 eV	3.10 eV
As3Hf1Na5_ICSD_66026	0.71 eV	0.92 eV
As3Hg1S6Tl1_ICSD_38363	1.24 eV	1.53 eV
As3K5O10_ICSD_23302	3.04 eV	3.24 eV
As3K6Nb1O1_ICSD_409630	1.01 eV	1.34 eV
As3La1Zn3_ICSD_261981	0.10 eV	0.25 eV
As3Mg4Na1O12_ICSD_59888	3.19 eV	3.31 eV
As3Na1O12Ti2_ICSD_421531	2.76 eV	2.97 eV
As3Na1O12Zr2_ICSD_97956	3.77 eV	3.59 eV
As3Na1Zn4_ICSD_262036	0.32 eV	0.45 eV
As3Na5Sn1_ICSD_656058	0.33 eV	0.34 eV
As3O1Sr3Ta1_ICSD_409567	1.32 eV	1.16 eV
As3Rb1Zn4_ICSD_262038	0.24 eV	0.29 eV
As3Rb5Si1_ICSD_300191	0.38 eV	0.59 eV
As3Sr2Zn2_ICSD_262413	0.18 eV	0.41 eV
As3Sr4_ICSD_402110	0.59 eV	0.73 eV
As4Ba3Si2_ICSD_41183	0.82 eV	0.77 eV
As4Ba3Sn2_ICSD_30702	0.72 eV	0.54 eV
As4Ba4Si1_ICSD_26467	0.99 eV	0.95 eV
As4Ba4Ti1_ICSD_380115	1.11 eV	0.99 eV
As4Bi1Cl7Hg6_ICSD_411204	1.28 eV	1.22 eV
As4Br16Ga4Hg11_ICSD_411676	0.87 eV	1.22 eV
As4Br1K1O6_ICSD_65206	2.73 eV	2.83 eV
As4Br6Cd1Hg6_ICSD_417039	1.36 eV	1.10 eV
As4Br7Cr1Hg6_ICSD_411481	1.30 eV	1.10 eV
As4Br7Hg6Ti1_ICSD_411239	1.24 eV	1.17 eV
As4C4F12_ICSD_9071	3.31 eV	3.28 eV
As4Ca1Na2O12_ICSD_409335	2.62 eV	3.06 eV
As4Ca3Ge2_ICSD_16455	0.60 eV	0.66 eV
As4Ca3In2_ICSD_61336	0.22 eV	0.37 eV
As4Ca3Si2_ICSD_16456	0.82 eV	0.81 eV
As4Ca5H10O20_ICSD_21068	3.83 eV	3.81 eV
As4Cd2Ge1_ICSD_42132	0.15 eV	0.27 eV

Supplementary Table 640. Five-fold cross validated predictions for the band gap energy (34/277).

system	calculated	predicted
As4Cl1Cu1S3_ICSD_419754	1.76 eV	1.73 eV
As4Cl1K1O6_ICSD_65205	3.04 eV	2.85 eV
As4Cl2Cu2S3_ICSD_419755	1.72 eV	1.62 eV
As4Cl7Hg6In1_ICSD_411820	1.39 eV	1.21 eV
As4Cl7Hg6Mo1_ICSD_411240	0.80 eV	1.16 eV
As4Cl7Hg6Ti1_ICSD_411238	1.20 eV	1.33 eV
As4Cs1F13_ICSD_281641	5.27 eV	4.57 eV
As4Cs4Se8_ICSD_171373	1.30 eV	1.33 eV
As4Cu3K2O12_ICSD_65416	1.32 eV	1.37 eV
As4Cu6Hg3S12_ICSD_169803	0.49 eV	0.77 eV
As4Eu3_ICSD_8211	0.28 eV	0.25 eV
As4Ga1Na3Sr3_ICSD_402286	0.44 eV	0.72 eV
As4Ge1Sr4_ICSD_610619	0.75 eV	0.92 eV
As4Ge1Te7_ICSD_41107	0.36 eV	0.32 eV
As4Ge2Sr3_ICSD_16454	0.77 eV	0.66 eV
As4I1K1O6_ICSD_16889	2.36 eV	2.67 eV
As4K7Nb1_ICSD_380109	1.13 eV	0.87 eV
As4K7Ta1_ICSD_380110	1.06 eV	0.94 eV
As4Mg1_ICSD_1079	0.81 eV	0.62 eV
As4Na2O11_ICSD_62963	2.07 eV	2.56 eV
As4Na8Ti1_ICSD_73310	1.05 eV	0.97 eV
As4Nb1Rb7_ICSD_380111	0.78 eV	0.98 eV
As4Pb9S15_ICSD_18097	1.57 eV	1.28 eV
As4Rb5Ta1Tl2_ICSD_85784	1.46 eV	0.93 eV
As4S3_ICSD_188058	2.29 eV	1.79 eV
As4S4_ICSD_187210	2.06 eV	1.97 eV
As4S5_ICSD_16107	1.98 eV	1.88 eV
As4Se3_ICSD_611376	1.67 eV	1.02 eV
As4Se4_ICSD_2056	0.16 eV	1.18 eV
As4Se4_ICSD_2599	1.40 eV	0.72 eV
As4Si1Sr4_ICSD_611409	1.11 eV	0.98 eV
As4Si2Sr3_ICSD_16453	0.91 eV	0.89 eV
As4Sn3_ICSD_163834	0.26 eV	0.31 eV
As4Sr3_ICSD_100110	0.63 eV	0.58 eV
As4Sr4Ti1_ICSD_380113	1.46 eV	1.09 eV
As5B1O20Pb6_ICSD_404329	2.93 eV	2.92 eV
As5Cs3O9_ICSD_413151	3.13 eV	3.35 eV
As5I1Te7_ICSD_31877	0.25 eV	0.62 eV
As5K6Sn3_ICSD_71009	0.54 eV	0.65 eV
As6Ba1Pt4_ICSD_62519	0.19 eV	0.18 eV
As6Bi1K3Se12_ICSD_180763	1.41 eV	1.11 eV
As6Ca5Sn2_ICSD_61037	0.25 eV	0.33 eV
As6Cs4_ICSD_409382	0.42 eV	0.70 eV
As6Na10Sn2_ICSD_40559	0.13 eV	0.46 eV
As6Pt4Sr1_ICSD_62518	0.17 eV	0.20 eV
As6Rb4_ICSD_409381	0.39 eV	0.61 eV
As8Ge38I8_ICSD_22033	1.01 eV	0.88 eV
As8S9_ICSD_98792	1.73 eV	1.96 eV
Au1Ba1Gd1Se3_ICSD_88718	0.18 eV	0.93 eV
Au1Ba4C4K1O4_ICSD_40854	2.70 eV	2.31 eV

Supplementary Table 641. Five-fold cross validated predictions for the band gap energy (35/277).

system	calculated	predicted
Au1Ba4Na1O8_ICSD_73189	1.37 eV	1.93 eV
Au1Bi1K2_ICSD_380341	0.89 eV	0.88 eV
Au1Bi1Li2_ICSD_261786	0.09 eV	0.62 eV
Au1Bi1Na2_ICSD_261788	0.49 eV	0.53 eV
Au1Bi5Na2O11_ICSD_164986	0.91 eV	1.22 eV
Au1Br1C2H8N4S2_ICSD_72684	3.38 eV	3.15 eV
Au1Br1F6_ICSD_93481	2.04 eV	1.91 eV
Au1Br1Se1_ICSD_2897	0.38 eV	1.23 eV
Au1Br1_ICSD_200286	2.29 eV	2.06 eV
Au1Br1_ICSD_200287	2.05 eV	2.05 eV
Au1Br4K1_ICSD_280033	0.73 eV	1.20 eV
Au1Br8Te1_ICSD_63129	0.80 eV	1.32 eV
Au1C1Cl1O1_ICSD_33526	3.39 eV	2.38 eV
Au1C1Cl3S2_ICSD_63379	1.96 eV	2.26 eV
Au1C1N1_ICSD_165175	2.01 eV	2.34 eV
Au1C2Cl1H3N1_ICSD_152108	4.00 eV	2.95 eV
Au1C2Cl1H8N4S2_ICSD_170718	3.51 eV	3.20 eV
Au1C2Cs1_ICSD_411251	2.39 eV	2.41 eV
Au1C2K1N2S2_ICSD_159001	2.77 eV	2.73 eV
Au1C2K1N2_ICSD_26498	3.45 eV	3.13 eV
Au1C2K1_ICSD_411255	2.42 eV	2.44 eV
Au1C2Li1_ICSD_411253	1.86 eV	2.09 eV
Au1C2N2Rb1S2_ICSD_159002	3.00 eV	2.56 eV
Au1C2N2Rb1_ICSD_66037	3.45 eV	3.04 eV
Au1C2Na1_ICSD_411254	1.55 eV	2.10 eV
Au1C2Rb1_ICSD_411252	2.58 eV	2.40 eV
Au1C4H12N1_ICSD_110302	2.93 eV	2.99 eV
Au1C4H2K1N4O1_ICSD_16043	4.25 eV	3.48 eV
Au1Cl1F3P1_ICSD_415842	3.78 eV	2.91 eV
Au1Cl1O1_ICSD_8190	1.10 eV	1.36 eV
Au1Cl1_ICSD_6052	2.28 eV	1.61 eV
Au1Cl3_ICSD_22146	1.24 eV	1.20 eV
Au1Cl4Cs1_ICSD_423233	1.87 eV	1.45 eV
Au1Cl4K1_ICSD_73080	1.49 eV	1.47 eV
Au1Cl4N5S5_ICSD_61254	1.68 eV	1.64 eV
Au1Cl4P1_ICSD_15565	2.80 eV	2.20 eV
Au1Cl4Rb1_ICSD_26021	1.69 eV	1.41 eV
Au1Cl4Tl1_ICSD_62107	1.82 eV	1.64 eV
Au1Cl5S1_ICSD_39532	1.68 eV	1.73 eV
Au1Cl7S1_ICSD_62908	1.78 eV	1.69 eV
Au1Cl7Se1_ICSD_62511	1.74 eV	1.65 eV
Au1Cl7Te1_ICSD_61350	1.58 eV	1.48 eV
Au1Cl8P1_ICSD_62236	1.78 eV	2.10 eV
Au1Co1Na4O5_ICSD_36661	2.00 eV	1.65 eV
Au1Cr1S2_ICSD_88852	0.98 eV	0.85 eV
Au1Cr3O8_ICSD_155508	1.17 eV	1.41 eV
Au1Cs1F4_ICSD_152056	2.98 eV	2.63 eV
Au1Cs1K2O2_ICSD_62064	2.29 eV	1.80 eV
Au1Cs1N12_ICSD_416487	1.52 eV	1.70 eV
Au1Cs1O1_ICSD_409553	1.66 eV	1.67 eV

Supplementary Table 642. Five-fold cross validated predictions for the band gap energy (36/277).

system	calculated	predicted
Au1Cs1Se3_ICSD_84002	1.06 eV	1.41 eV
Au1Cs1Te1_ICSD_71653	1.48 eV	1.06 eV
Au1Cs1_ICSD_150971	0.75 eV	0.57 eV
Au1Cs3Ge4_ICSD_413725	0.92 eV	0.90 eV
Au1Cs3Pb4_ICSD_107448	0.55 eV	0.47 eV
Au1Eu3O6_ICSD_411503	0.24 eV	1.05 eV
Au1F11Th2_ICSD_89619	2.59 eV	3.07 eV
Au1F11U2_ICSD_152058	1.09 eV	1.94 eV
Au1F12Sb2Xe2_ICSD_412106	1.45 eV	2.15 eV
Au1F3_ICSD_16876	2.07 eV	1.83 eV
Au1F4K1_ICSD_9906	2.88 eV	2.69 eV
Au1F4Li1_ICSD_33953	2.60 eV	2.59 eV
Au1F4Li1_ICSD_9908	2.60 eV	2.40 eV
Au1F4Na1_ICSD_9905	2.62 eV	2.45 eV
Au1F4Rb1_ICSD_9907	2.96 eV	2.70 eV
Au1F6K1_ICSD_415874	2.20 eV	1.96 eV
Au1F6Li1_ICSD_165209	2.10 eV	1.75 eV
Au1F6O2_ICSD_171655	0.09 eV	1.53 eV
Au1F6Tl1_ICSD_95770	1.78 eV	1.83 eV
Au1Ga1O2_ICSD_95666	0.20 eV	0.94 eV
Au1Gd3O6_ICSD_411500	1.80 eV	1.69 eV
Au1Ge4K3_ICSD_413728	0.82 eV	0.78 eV
Au1Ge4Rb3_ICSD_413724	0.89 eV	0.81 eV
Au1I1Te1_ICSD_1661	0.93 eV	1.12 eV
Au1I1_ICSD_24619	2.29 eV	1.66 eV
Au1I2K5O2_ICSD_40376	1.98 eV	1.80 eV
Au1I4K1O12_ICSD_417267	2.12 eV	1.83 eV
Au1K1N12_ICSD_416489	1.47 eV	1.69 eV
Au1K1N4O12_ICSD_16141	2.01 eV	2.24 eV
Au1K1Na2O2_ICSD_61226	2.18 eV	1.95 eV
Au1K1O2_ICSD_15115	1.36 eV	1.37 eV
Au1K1O8S2_ICSD_412094	1.54 eV	2.11 eV
Au1K1P2S7_ICSD_165325	1.50 eV	1.68 eV
Au1K1S1_ICSD_202178	2.24 eV	2.08 eV
Au1K1S5_ICSD_402875	1.78 eV	1.66 eV
Au1K1Se1_ICSD_656713	1.90 eV	1.70 eV
Au1K1Se2_ICSD_84003	0.92 eV	1.11 eV
Au1K1Se5_ICSD_67372	1.01 eV	1.07 eV
Au1K2P1S4_ICSD_85679	1.78 eV	1.80 eV
Au1K2P1_ICSD_300201	1.21 eV	1.03 eV
Au1K2Sb1_ICSD_380340	1.20 eV	1.09 eV
Au1K3O1_ICSD_79086	0.64 eV	0.87 eV
Au1K3P2Se8_ICSD_165321	1.28 eV	1.32 eV
Au1K3Se13_ICSD_67373	0.66 eV	0.99 eV
Au1K3Se2_ICSD_402000	2.10 eV	1.62 eV
Au1K3Sn4_ICSD_107444	0.42 eV	0.56 eV
Au1K5P2_ICSD_40700	0.52 eV	0.67 eV
Au1La1O3_ICSD_73873	1.97 eV	1.74 eV
Au1Li1S1_ICSD_165259	2.12 eV	1.94 eV
Au1Li1S1_ICSD_280534	2.12 eV	1.80 eV

Supplementary Table 643. Five-fold cross validated predictions for the band gap energy (37/277).

system	calculated	predicted
Au1Li3O3_ICSD_15113	2.04 eV	1.75 eV
Au1Li3S2_ICSD_280535	2.47 eV	2.13 eV
Au1N12Rb1_ICSD_416488	1.50 eV	1.53 eV
Au1Na1O2_ICSD_409547	1.32 eV	1.59 eV
Au1Na1Se2_ICSD_84004	0.86 eV	1.00 eV
Au1Na2O2Rb1_ICSD_411460	2.17 eV	1.99 eV
Au1Na2Sb1_ICSD_23255	0.58 eV	0.66 eV
Au1Na3O2_ICSD_62066	2.11 eV	1.90 eV
Au1Na3S2_ICSD_202329	2.37 eV	1.95 eV
Au1O1Rb1_ICSD_409552	1.68 eV	1.64 eV
Au1O1Rb3_ICSD_75499	0.27 eV	0.70 eV
Au1O2Rb1_ICSD_15116	1.76 eV	1.66 eV
Au1O2Sc1_ICSD_95669	2.24 eV	2.12 eV
Au1O2Y1_ICSD_95675	2.81 eV	2.16 eV
Au1O8Rb1S2_ICSD_412095	1.77 eV	1.88 eV
Au1O8Rb1Se2_ICSD_413989	1.57 eV	1.78 eV
Au1P1S4Tl2_ICSD_85680	1.41 eV	1.63 eV
Au1P1S4_ICSD_413009	1.21 eV	1.35 eV
Au1Pb4Rb3_ICSD_107447	0.34 eV	0.49 eV
Au1Rb1S1_ICSD_71654	2.13 eV	2.13 eV
Au1Rb1Se1_ICSD_656714	1.83 eV	1.72 eV
Au1Rb1Se3U1_ICSD_420483	1.12 eV	1.07 eV
Au1Rb1Te1_ICSD_71652	1.28 eV	0.94 eV
Au1Rb1Te3U1_ICSD_420485	0.54 eV	0.79 eV
Au1Rb1_ICSD_58428	0.16 eV	0.68 eV
Au1Rb2S4Sb1_ICSD_54507	1.63 eV	1.50 eV
Au1Rb3Sn4_ICSD_107445	0.57 eV	0.58 eV
Au2Ba1F12_ICSD_39316	1.77 eV	2.04 eV
Au2Ba1F8_ICSD_65289	2.96 eV	2.52 eV
Au2Ba1O4_ICSD_80327	1.69 eV	1.61 eV
Au2Br2H12N4_ICSD_80216	3.65 eV	2.68 eV
Au2Br6Cs2_ICSD_170696	0.66 eV	0.96 eV
Au2Br6Rb2_ICSD_9577	1.11 eV	1.04 eV
Au2Br6_ICSD_4069	0.65 eV	1.05 eV
Au2C10Cl8S8_ICSD_65114	0.21 eV	1.82 eV
Au2Ca1F12_ICSD_39315	1.71 eV	1.89 eV
Au2Ca1O4_ICSD_79801	1.84 eV	1.57 eV
Au2Cd1F12_ICSD_415873	1.99 eV	1.70 eV
Au2Cd1F8_ICSD_85413	2.31 eV	2.25 eV
Au2Cd2Rb2S4_ICSD_85582	2.07 eV	1.70 eV
Au2Cl6Cs2_ICSD_417370	0.91 eV	1.27 eV
Au2Cl6Cs2_ICSD_56470	0.93 eV	1.36 eV
Au2Cs1F7_ICSD_152057	2.28 eV	2.37 eV
Au2Cs2I6_ICSD_186066	0.64 eV	0.90 eV
Au2Cs2Se3_ICSD_85708	1.51 eV	1.36 eV
Au2F11H1Mg1_ICSD_415872	2.07 eV	2.06 eV
Au2F8Hg1_ICSD_85414	2.08 eV	2.09 eV
Au2F8Mg1_ICSD_65287	2.44 eV	2.42 eV
Au2F8Ni1_ICSD_65288	2.23 eV	2.00 eV
Au2F8Zn1_ICSD_65286	2.24 eV	2.19 eV

Supplementary Table 644. Five-fold cross validated predictions for the band gap energy (38/277).

system	calculated	predicted
Au2K2P2Se6_ICSD_165322	1.09 eV	1.19 eV
Au2K2S4Sn1_ICSD_74022	1.56 eV	1.72 eV
Au2La4O9_ICSD_74989	2.12 eV	1.95 eV
Au2O10Se3_ICSD_170260	1.09 eV	1.41 eV
Au2O11Se4_ICSD_15495	1.41 eV	1.57 eV
Au2O3_ICSD_8014	1.03 eV	0.99 eV
Au2O4Sr1_ICSD_80328	1.77 eV	1.61 eV
Au2O7Se2_ICSD_37009	1.21 eV	1.31 eV
Au2P2Se6Tl2_ICSD_171216	1.01 eV	0.93 eV
Au2P3_ICSD_8058	0.17 eV	0.59 eV
Au2S1_ICSD_612282	1.96 eV	1.32 eV
Au3C6Co1K1N6_ICSD_201056	2.69 eV	2.78 eV
Au3C6H6La1N6O3_ICSD_170989	2.72 eV	3.13 eV
Au3C6K1N6Ni1_ICSD_249724	2.82 eV	2.90 eV
Au3Cl8Rb3_ICSD_9578	0.92 eV	1.71 eV
Au3Dy3Sb4_ICSD_611802	0.46 eV	0.44 eV
Au3Er3Sb4_ICSD_611833	0.47 eV	0.46 eV
Au3F12La1_ICSD_78915	2.57 eV	2.32 eV
Au3Gd3Sb4_ICSD_611925	0.27 eV	0.36 eV
Au3Ge1Na1_ICSD_16463	0.15 eV	0.77 eV
Au3Ho3Sb4_ICSD_612001	0.46 eV	0.45 eV
Au3K3Sb2_ICSD_78977	1.24 eV	0.89 eV
Au3La3Sb4_ICSD_612105	0.39 eV	0.42 eV
Au3Lu3Sb4_ICSD_612134	0.48 eV	0.45 eV
Au3Na1Si1_ICSD_16462	0.15 eV	0.79 eV
Au3O2Rb5_ICSD_91308	0.97 eV	1.14 eV
Au3P7Sn1_ICSD_416407	0.21 eV	0.56 eV
Au3Rb1Se2_ICSD_82541	2.01 eV	1.16 eV
Au3Rb3Sb2_ICSD_78978	1.35 eV	0.86 eV
Au3Sb4Tb3_ICSD_612293	0.46 eV	0.41 eV
Au3Sb4Y3_ICSD_957	0.48 eV	0.46 eV
Au4Cd1K2S4_ICSD_85583	2.18 eV	1.64 eV
Au4Cl8_ICSD_201436	0.97 eV	1.37 eV
Au4S3Tl2_ICSD_51235	1.24 eV	1.31 eV
Au5Cs7O2_ICSD_411334	1.03 eV	1.01 eV
Au5Cs7O2_ICSD_95821	1.04 eV	1.07 eV
Au5O2Rb7_ICSD_95825	0.89 eV	0.98 eV
Au6Rb4S5_ICSD_82556	1.94 eV	1.90 eV
Au7I1P10_ICSD_12162	0.75 eV	0.80 eV
Au7I1P10_ICSD_8059	0.84 eV	0.76 eV
B0.667N0.667_ICSD_162872	4.46 eV	4.24 eV
B10Ba2H2Na2O19_ICSD_95447	4.76 eV	5.08 eV
B10Ca1La2O19_ICSD_92866	5.81 eV	5.50 eV
B10O21Pb6_ICSD_2641	3.25 eV	3.35 eV
B12Br1Cs3H12_ICSD_414584	4.75 eV	4.54 eV
B12Br1H12K3_ICSD_414581	4.81 eV	4.65 eV
B12Br1H12Rb3_ICSD_414583	4.74 eV	4.44 eV
B12C2Li2_ICSD_415556	1.39 eV	1.68 eV
B12C2Mg1_ICSD_416801	2.14 eV	1.65 eV
B12C3_ICSD_612562	1.52 eV	1.21 eV

Supplementary Table 645. Five-fold cross validated predictions for the band gap energy (39/277).

system	calculated	predicted
B12Cl1Cs3H12_ICSD_414586	4.94 eV	4.77 eV
B12Cl1H12Rb3_ICSD_414585	4.94 eV	4.23 eV
B12Cs2H12_ICSD_92501	5.37 eV	4.73 eV
B12Cs3H12I1_ICSD_98622	4.54 eV	4.19 eV
B12H12I1K3_ICSD_98619	4.60 eV	4.48 eV
B12H12I1Rb3_ICSD_98620	4.61 eV	4.41 eV
B12H12K2_ICSD_36148	5.52 eV	4.93 eV
B12H12Rb2_ICSD_20015	5.47 eV	5.02 eV
B12H12Tl2_ICSD_422433	4.53 eV	3.89 eV
B12H20Li2O4_ICSD_163690	5.26 eV	4.78 eV
B12H20N2_ICSD_98618	5.65 eV	5.19 eV
B12Ni1_ICSD_614986	0.25 eV	1.19 eV
B12O24Se2Zn8_ICSD_74057	4.94 eV	3.83 eV
B12P2_ICSD_62748	2.49 eV	1.73 eV
B13C2Li1_ICSD_415557	2.54 eV	1.86 eV
B18Hg2Rb4Se18_ICSD_410758	1.87 eV	1.68 eV
B1Ba1Bi1O4_ICSD_154105	3.60 eV	2.95 eV
B1Ba1Bi1S4_ICSD_248222	1.98 eV	2.12 eV
B1Ba1Cl1F4_ICSD_188654	5.99 eV	5.87 eV
B1Ba1F10P1_ICSD_420597	7.25 eV	6.41 eV
B1Ba1F1O3Zn1_ICSD_248042	3.94 eV	3.91 eV
B1Ba1F2Ga1O3_ICSD_91315	5.04 eV	4.86 eV
B1Ba1F3O1_ICSD_16684	5.88 eV	6.11 eV
B1Ba1Li1O3_ICSD_73218	4.39 eV	3.40 eV
B1Ba1Li1O3_ICSD_92843	2.29 eV	3.23 eV
B1Ba1Li1S3_ICSD_82352	2.16 eV	2.40 eV
B1Ba1Na1O3_ICSD_250086	3.66 eV	3.78 eV
B1Ba1O5P1_ICSD_50875	5.49 eV	5.22 eV
B1Ba1O5P1_ICSD_99842	5.49 eV	5.23 eV
B1Ba1S4Sb1_ICSD_248221	2.36 eV	2.15 eV
B1Ba2Cl17Zr6_ICSD_203141	1.08 eV	1.55 eV
B1Ba2Cl1N2_ICSD_418947	2.75 eV	2.49 eV
B1Ba3O3P1_ICSD_402017	1.46 eV	1.73 eV
B1Ba3O7P1_ICSD_150328	3.33 eV	3.93 eV
B1Be2Cs1F2O3_ICSD_20000	5.92 eV	5.91 eV
B1Be2F1O3_ICSD_200264	6.36 eV	6.18 eV
B1Be2F1O3_ICSD_56847	6.35 eV	6.24 eV
B1Be2F2K1O3_ICSD_16178	6.04 eV	5.88 eV
B1Be2F2K1O3_ICSD_77277	6.05 eV	5.73 eV
B1Be2F2Na1O3_ICSD_75594	5.89 eV	5.87 eV
B1Be2F2O3Rb1_ICSD_260439	6.04 eV	5.94 eV
B1Be2F2O3Rb1_ICSD_4223	5.92 eV	6.24 eV
B1Be2H1O4_ICSD_34650	5.86 eV	5.94 eV
B1Bi1O3_ICSD_413621	3.10 eV	2.93 eV
B1Bi1O4Pb1_ICSD_183393	2.84 eV	2.74 eV
B1Bi1O4Pb1_ICSD_416639	1.51 eV	2.57 eV
B1Bi1_ICSD_184569	0.22 eV	0.59 eV
B1Br1C4F4H11N1_ICSD_249155	5.09 eV	5.03 eV
B1Br1Eu2N2_ICSD_409982	1.45 eV	1.49 eV
B1Br1Mg2N2_ICSD_261620	3.32 eV	3.02 eV

Supplementary Table 646. Five-fold cross validated predictions for the band gap energy (40/277).

system	calculated	predicted
B1Br1N2Sr2_ICSD_261795	2.40 eV	2.39 eV
B1Br20K8Zr6_ICSD_165310	0.97 eV	1.71 eV
B1Br3I3P1_ICSD_411438	1.94 eV	2.25 eV
B1Br3P4S3_ICSD_165186	2.70 eV	2.25 eV
B1Br3_ICSD_173374	3.78 eV	3.58 eV
B1C1Ca3Cl2N1_ICSD_33850	2.17 eV	2.11 eV
B1C1Cl2N1Sr3_ICSD_74914	1.87 eV	2.39 eV
B1C1F4H6N3_ICSD_202434	5.68 eV	5.71 eV
B1C1F6K1_ICSD_1194	7.00 eV	6.81 eV
B1C1H5N2_ICSD_2165	5.45 eV	5.36 eV
B1C1Li1_ICSD_245288	0.85 eV	1.97 eV
B1C2Cs1F8_ICSD_200869	6.40 eV	6.43 eV
B1C2N1_ICSD_93040	2.11 eV	2.65 eV
B1C2N1_ICSD_93041	1.67 eV	2.24 eV
B1C3.33N1_ICSD_161278	0.49 eV	2.08 eV
B1C3Cs1F9N1O2_ICSD_410079	3.39 eV	4.02 eV
B1C3F4H10N1_ICSD_171202	7.40 eV	6.55 eV
B1C3H12N1_ICSD_249799	6.10 eV	5.70 eV
B1C3K2N3_ICSD_262588	2.45 eV	4.17 eV
B1C4Cl1F4H11N1_ICSD_320194	6.36 eV	6.04 eV
B1C4Cs1N4_ICSD_414556	6.31 eV	5.20 eV
B1C4Cu1N4_ICSD_414557	3.52 eV	3.92 eV
B1C4H4N5_ICSD_414561	6.28 eV	5.04 eV
B1C4K1N4_ICSD_411180	6.22 eV	5.01 eV
B1C4K1O8_ICSD_281621	3.50 eV	3.98 eV
B1C4Li1N4_ICSD_414558	6.35 eV	4.81 eV
B1C4N4Na1_ICSD_414559	6.52 eV	4.43 eV
B1C4N4Rb1_ICSD_414563	6.33 eV	5.93 eV
B1C4N4Tl1_ICSD_414564	5.18 eV	4.55 eV
B1C4Na1O8_ICSD_281622	3.53 eV	3.94 eV
B1Ca1Ga1O4_ICSD_93390	4.01 eV	4.14 eV
B1Ca1H1O3_ICSD_181633	4.94 eV	4.94 eV
B1Ca1H1O5Si1_ICSD_168627	5.41 eV	5.15 eV
B1Ca1H3Ni1O10P2_ICSD_409898	3.84 eV	3.51 eV
B1Ca1Li1O3_ICSD_99386	5.03 eV	4.36 eV
B1Ca1O5P1_ICSD_87893	5.31 eV	5.49 eV
B1Ca2Cl1N2_ICSD_406361	3.33 eV	2.80 eV
B1Ca2Cl1O3_ICSD_342	4.57 eV	4.13 eV
B1Ca2F1N2_ICSD_50842	2.91 eV	3.34 eV
B1Ca2H1N2_ICSD_414006	3.01 eV	2.73 eV
B1Ca3N3_ICSD_95814	0.59 eV	1.70 eV
B1Cd1Cs1O3_ICSD_189199	1.81 eV	2.60 eV
B1Cd1Li1O3_ICSD_200615	2.11 eV	2.62 eV
B1Cd1Li1O3_ICSD_20191	2.21 eV	2.39 eV
B1Ce1N2_ICSD_410417	2.07 eV	2.14 eV
B1Ce1O3_ICSD_99689	4.72 eV	4.36 eV
B1Ce1O3_ICSD_99690	4.39 eV	4.18 eV
B1Ce1O5Si1_ICSD_28026	5.24 eV	4.60 eV
B1Ce1S3_ICSD_421071	2.44 eV	2.32 eV
B1Cl1F4O2_ICSD_166598	1.91 eV	4.10 eV

Supplementary Table 647. Five-fold cross validated predictions for the band gap energy (41/277).

system	calculated	predicted
B1Cl1F6_ICSD_68277	3.33 eV	4.55 eV
B1Cl1H4Na2O4_ICSD_32518	4.63 eV	4.71 eV
B1Cl1Mg2N2_ICSD_413908	3.66 eV	3.52 eV
B1Cl1N2Sr2_ICSD_50845	2.76 eV	2.61 eV
B1Cl2H4K1Zn1_ICSD_174294	5.00 eV	4.29 eV
B1Cl3_ICSD_27869	4.69 eV	4.59 eV
B1Co3O5_ICSD_93484	0.63 eV	1.57 eV
B1Co3O7P1_ICSD_51317	2.98 eV	2.56 eV
B1Cr1O3_ICSD_43311	2.26 eV	2.01 eV
B1Cr1O4Pb1_ICSD_97663	2.24 eV	2.30 eV
B1Cr2O12P3_ICSD_409459	3.01 eV	2.65 eV
B1Cs1F4_ICSD_95828	7.55 eV	7.27 eV
B1Cs1Li2S3_ICSD_411530	3.10 eV	2.83 eV
B1Cs1Na2O3_ICSD_67524	3.29 eV	3.10 eV
B1Cs1Se3_ICSD_411342	1.64 eV	1.73 eV
B1Cs3P2_ICSD_300123	1.69 eV	1.42 eV
B1Cs3S3_ICSD_391170	2.48 eV	2.48 eV
B1Cu1S2_ICSD_156413	1.94 eV	1.71 eV
B1Cu1Se2_ICSD_613591	1.67 eV	1.22 eV
B1Cu2H5O6_ICSD_54883	0.11 eV	1.33 eV
B1Dy1O3_ICSD_59849	5.37 eV	4.66 eV
B1Dy3O9W1_ICSD_250419	3.42 eV	3.47 eV
B1F1H4N1O4P1_ICSD_170949	5.74 eV	5.59 eV
B1F1N2Sr2_ICSD_50843	2.57 eV	2.82 eV
B1F3H4O2_ICSD_26551	7.16 eV	6.46 eV
B1F3Mg3O3_ICSD_4226	5.40 eV	5.26 eV
B1F3Na2O4S1_ICSD_424150	5.76 eV	5.40 eV
B1F3_ICSD_24783	8.04 eV	7.16 eV
B1F4H2N3S3_ICSD_201705	2.27 eV	3.51 eV
B1F4H3O1_ICSD_15591	7.54 eV	7.24 eV
B1F4H4N1_ICSD_93978	7.48 eV	7.13 eV
B1F4H5N2_ICSD_245055	6.31 eV	6.27 eV
B1F4In1_ICSD_50218	4.55 eV	5.26 eV
B1F4K1_ICSD_22260	7.83 eV	7.60 eV
B1F4Li1_ICSD_171375	8.22 eV	7.73 eV
B1F4N1O2_ICSD_240721	2.90 eV	2.72 eV
B1F4Na1_ICSD_30349	7.83 eV	7.00 eV
B1F4Rb1_ICSD_24016	7.68 eV	7.27 eV
B1F4Tl1_ICSD_300222	5.78 eV	4.77 eV
B1F5Li2_ICSD_426821	7.53 eV	7.66 eV
B1F7N2O1S2_ICSD_414492	4.90 eV	4.84 eV
B1F7S1_ICSD_10337	4.96 eV	5.43 eV
B1F7Sn2_ICSD_15263	4.38 eV	4.30 eV
B1F8N1_ICSD_165425	2.91 eV	3.32 eV
B1F8N1_ICSD_63311	2.90 eV	2.97 eV
B1F9Sn3_ICSD_15264	4.37 eV	4.22 eV
B1Fe1Ni2O5_ICSD_69615	1.39 eV	1.41 eV
B1Fe1O3_ICSD_34474	1.47 eV	1.99 eV
B1Fe1O4Pb1_ICSD_97668	1.87 eV	1.71 eV
B1Fe2O12P3_ICSD_260895	2.30 eV	2.85 eV

Supplementary Table 648. Five-fold cross validated predictions for the band gap energy (42/277).

system	calculated	predicted
B1Fe2O4_ICSD_88450	0.35 eV	1.15 eV
B1Fe3O5_ICSD_164815	0.52 eV	0.81 eV
B1Fe3O6_ICSD_23863	1.54 eV	1.62 eV
B1Ga1O4Pb1_ICSD_279600	3.37 eV	3.06 eV
B1Gd1N2_ICSD_657369	2.17 eV	2.08 eV
B1Gd1O3_ICSD_87779	2.41 eV	4.17 eV
B1Gd3O9W1_ICSD_250417	3.38 eV	3.31 eV
B1Ge1La1O5_ICSD_39262	3.92 eV	4.06 eV
B1Ge1Li1O4_ICSD_28106	2.67 eV	4.10 eV
B1Ge1Li1O4_ICSD_67535	4.43 eV	4.48 eV
B1Ge2K1O6_ICSD_281258	3.39 eV	3.67 eV
B1H10Li4N3_ICSD_161018	3.61 eV	3.79 eV
B1H1Na2O3_ICSD_27211	3.82 eV	3.84 eV
B1H1O2_ICSD_34639	7.06 eV	6.02 eV
B1H2Li1_ICSD_153289	0.86 eV	3.64 eV
B1H2Na1O7Si2_ICSD_12134	4.06 eV	4.61 eV
B1H3In1Na1O10P2_ICSD_409585	3.17 eV	3.63 eV
B1H3Mg3O6_ICSD_250420	3.48 eV	3.95 eV
B1H3Na1O10P2V1_ICSD_409651	1.12 eV	2.27 eV
B1H3O3_ICSD_52290	5.98 eV	5.47 eV
B1H4K1_ICSD_160985	6.11 eV	5.63 eV
B1H4K1_ICSD_160986	1.40 eV	3.42 eV
B1H4Li1_ICSD_173101	5.51 eV	5.64 eV
B1H4Li1_ICSD_186262	6.83 eV	6.72 eV
B1H4Li1_ICSD_245569	6.77 eV	6.48 eV
B1H4Li1_ICSD_95207	6.77 eV	6.50 eV
B1H4N1O8P2Zn1_ICSD_410872	4.81 eV	4.63 eV
B1H4Na1O4_ICSD_167112	5.11 eV	4.95 eV
B1H4Na1_ICSD_159243	6.25 eV	6.38 eV
B1H4Na1_ICSD_181024	6.32 eV	6.40 eV
B1H4Na1_ICSD_182733	6.63 eV	5.80 eV
B1H4Na1_ICSD_182734	6.54 eV	6.33 eV
B1H4Na1_ICSD_261750	6.31 eV	6.34 eV
B1H4Na1_ICSD_261751	5.46 eV	6.17 eV
B1H5In1N1O9P2_ICSD_409584	4.05 eV	4.11 eV
B1H5Li1N1_ICSD_180548	4.12 eV	4.97 eV
B1H6N1_ICSD_165975	5.95 eV	5.62 eV
B1H6N1_ICSD_180547	6.04 eV	5.99 eV
B1H6N1_ICSD_181423	6.06 eV	5.97 eV
B1H7Li1N1_ICSD_169560	5.63 eV	5.41 eV
B1I1N2Sr2_ICSD_50598	2.53 eV	2.41 eV
B1I3P4S3_ICSD_165187	2.27 eV	2.14 eV
B1I3_ICSD_28328	2.67 eV	2.57 eV
B1In1O3_ICSD_75254	2.83 eV	2.91 eV
B1In2O12P3_ICSD_420643	4.01 eV	3.81 eV
B1K1Li2O3_ICSD_48177	4.02 eV	4.01 eV
B1K1Mg1O3_ICSD_174336	4.37 eV	4.08 eV
B1K1Na2O3_ICSD_33261	2.82 eV	3.06 eV
B1K1O6Si2_ICSD_380488	5.31 eV	5.12 eV
B1K2Li1O3_ICSD_60949	3.60 eV	3.51 eV

Supplementary Table 649. Five-fold cross validated predictions for the band gap energy (43/277).

system	calculated	predicted
B1K2Na1P2_ICSD_300133	1.79 eV	1.62 eV
B1K3O13Sb4_ICSD_411501	1.85 eV	2.46 eV
B1K3P2_ICSD_300104	1.65 eV	1.55 eV
B1K3S3_ICSD_411607	2.71 eV	2.59 eV
B1La1O3_ICSD_15383	4.40 eV	4.29 eV
B1La1O5Si1_ICSD_83397	5.15 eV	5.06 eV
B1La3O9W1_ICSD_39809	3.43 eV	3.32 eV
B1Li1Mn1O3_ICSD_94318	2.58 eV	2.45 eV
B1Li1O2_ICSD_200891	5.37 eV	5.43 eV
B1Li1O2_ICSD_34256	7.29 eV	5.43 eV
B1Li1O3Sr1_ICSD_92842	2.81 eV	3.69 eV
B1Li1O4Si1_ICSD_67536	6.41 eV	5.47 eV
B1Li1S3Sr1_ICSD_380105	2.39 eV	2.67 eV
B1Li1Si2_ICSD_425643	1.15 eV	1.00 eV
B1Li3N2_ICSD_155126	3.31 eV	3.18 eV
B1Li3N2_ICSD_155129	3.36 eV	3.18 eV
B1Li3N2_ICSD_655673	3.44 eV	3.22 eV
B1Li3O3_ICSD_9105	5.14 eV	4.65 eV
B1Li3S3_ICSD_75223	3.05 eV	3.26 eV
B1Mg1Na1O3_ICSD_249567	3.62 eV	4.20 eV
B1Mg3N3_ICSD_79623	1.37 eV	2.20 eV
B1Mn1O4Pb1_ICSD_97664	0.68 eV	1.64 eV
B1N1_ICSD_162871	4.46 eV	4.32 eV
B1N1_ICSD_162875	3.57 eV	4.12 eV
B1N1_ICSD_162876	5.75 eV	5.13 eV
B1N1_ICSD_162877	1.57 eV	3.39 eV
B1N1_ICSD_162880	3.49 eV	3.76 eV
B1N1_ICSD_162882	3.54 eV	3.98 eV
B1N1_ICSD_162883	5.13 eV	4.32 eV
B1N1_ICSD_186246	4.46 eV	4.52 eV
B1N1_ICSD_42002	4.46 eV	4.76 eV
B1N1_ICSD_614873	5.21 eV	4.62 eV
B1N2Na3_ICSD_68619	1.69 eV	1.96 eV
B1N2Nd1_ICSD_657367	1.99 eV	2.18 eV
B1Na1O2_ICSD_34645	4.14 eV	4.22 eV
B1Na1O3Sr1_ICSD_172420	3.87 eV	3.85 eV
B1Na1O4Si1_ICSD_39459	5.42 eV	5.33 eV
B1Na2O3Rb1_ICSD_67525	2.96 eV	3.03 eV
B1Na3O3_ICSD_1351	2.99 eV	3.09 eV
B1Na3P2_ICSD_300124	1.89 eV	1.56 eV
B1Na3S3_ICSD_411608	2.58 eV	2.54 eV
B1Nb1Ni2O6_ICSD_32583	2.76 eV	2.50 eV
B1Nb1O4_ICSD_63202	2.80 eV	2.97 eV
B1Nd1O3_ICSD_240380	3.87 eV	3.90 eV
B1Nd1O3_ICSD_412407	3.71 eV	4.11 eV
B1Nd1S3_ICSD_421073	2.02 eV	2.26 eV
B1O2Tl1_ICSD_36404	2.85 eV	2.89 eV
B1O3P1Sr3_ICSD_401207	2.02 eV	1.95 eV
B1O3Pr1_ICSD_421745	4.36 eV	3.99 eV
B1O3Sc1_ICSD_65010	4.62 eV	4.79 eV

Supplementary Table 650. Five-fold cross validated predictions for the band gap energy (44/277).

system	calculated	predicted
B1O3Tl3_ICSD_8084	1.37 eV	1.26 eV
B1O3V1_ICSD_45060	1.43 eV	1.95 eV
B1O3Y1_ICSD_44162	5.17 eV	4.66 eV
B1O4P1_ICSD_26890	7.25 eV	6.11 eV
B1O4P1_ICSD_413435	7.01 eV	6.14 eV
B1O4Ta1_ICSD_402404	3.64 eV	3.07 eV
B1O5P1Pb1_ICSD_93598	4.28 eV	3.88 eV
B1O5P1Sr1_ICSD_77519	5.39 eV	5.39 eV
B1O5P1Sr1_ICSD_97675	5.37 eV	5.40 eV
B1O7P1Zn3_ICSD_406386	4.00 eV	3.66 eV
B1O8P2Rb1Zn1_ICSD_410870	5.14 eV	4.55 eV
B1O9Tb3W1_ICSD_250418	3.43 eV	3.43 eV
B1P1S4_ICSD_24618	2.23 eV	2.45 eV
B1P1_ICSD_184570	1.25 eV	1.15 eV
B1P1_ICSD_615155	1.08 eV	1.46 eV
B1P2Rb3_ICSD_402084	1.58 eV	1.46 eV
B1Pr1S3_ICSD_421072	2.33 eV	2.18 eV
B1Rb1S3_ICSD_73084	2.28 eV	2.64 eV
B1Rb1Se3_ICSD_411343	1.52 eV	1.68 eV
B1S2Tl1_ICSD_71593	1.75 eV	1.63 eV
B1S3Tb1_ICSD_710042	2.31 eV	2.15 eV
B1S3Tl1_ICSD_73085	1.68 eV	1.77 eV
B1S3Tl3_ICSD_202528	1.40 eV	1.54 eV
B1Sb1_ICSD_184571	0.75 eV	0.76 eV
B1Se3Tl3_ICSD_40375	1.24 eV	1.22 eV
B1_ICSD_659240	1.45 eV	1.23 eV
B28_ICSD_164659	1.54 eV	1.65 eV
B2Ba1Be2O6_ICSD_67975	5.39 eV	4.65 eV
B2Ba1Cu1O5_ICSD_84683	1.68 eV	1.49 eV
B2Ba1F8_ICSD_240991	7.99 eV	7.04 eV
B2Ba1Ga2O7_ICSD_91280	4.05 eV	3.88 eV
B2Ba1O6Ti1_ICSD_183931	2.89 eV	3.38 eV
B2Ba1O6Zr1_ICSD_95527	4.14 eV	3.73 eV
B2Ba1S4_ICSD_412516	2.72 eV	2.47 eV
B2Ba1Se6_ICSD_411967	1.70 eV	1.62 eV
B2Ba2Ca1O6_ICSD_80429	4.08 eV	3.93 eV
B2Ba2Cd1O6_ICSD_180954	3.12 eV	2.83 eV
B2Ba2Cl1Gd1O6_ICSD_262073	4.00 eV	3.77 eV
B2Ba2Cl1Ho1O6_ICSD_65933	4.09 eV	3.79 eV
B2Ba2Cl1O6Y1_ICSD_262072	4.04 eV	3.89 eV
B2Ba2Cl1O6Yb1_ICSD_65934	0.16 eV	1.56 eV
B2Ba2Mg1O6_ICSD_75986	4.53 eV	4.02 eV
B2Ba3N4_ICSD_412663	2.51 eV	2.07 eV
B2Ba3O12Ti3_ICSD_99460	2.55 eV	2.74 eV
B2Ba4N4O1_ICSD_400338	2.81 eV	2.18 eV
B2Be1C2_ICSD_418618	0.22 eV	1.42 eV
B2Be1Ca1O5_ICSD_72520	5.59 eV	5.38 eV
B2Be2Ca1F1K1O6_ICSD_183886	5.25 eV	5.19 eV
B2Be2Ca1F1Na1O6_ICSD_183885	5.01 eV	5.15 eV
B2Be2F1K1O6Sr1_ICSD_183887	4.91 eV	4.98 eV

Supplementary Table 651. Five-fold cross validated predictions for the band gap energy (45/277).

system	calculated	predicted
B2Be2O7Sr2_ICSD_79025	4.09 eV	4.44 eV
B2Bi1F1O4_ICSD_172481	4.22 eV	3.89 eV
B2Bi2Ca1O7_ICSD_245016	3.23 eV	2.96 eV
B2Bi2O7Zn1_ICSD_152281	2.59 eV	2.88 eV
B2Br4Cl6N2P2_ICSD_412556	2.89 eV	2.88 eV
B2C2Ca1_ICSD_88019	0.48 eV	1.35 eV
B2C2Mg1_ICSD_421839	1.31 eV	1.30 eV
B2C2Mg1_ICSD_79587	1.02 eV	1.00 eV
B2C6F8Fe1O6_ICSD_152380	4.38 eV	3.63 eV
B2C8Cu1N8_ICSD_415546	2.52 eV	3.65 eV
B2C8Hg1N8_ICSD_412297	4.59 eV	4.16 eV
B2C8N8Zn1_ICSD_415547	5.69 eV	4.63 eV
B2Ca1H10N2_ICSD_246139	3.84 eV	4.81 eV
B2Ca1H12O10_ICSD_23016	5.43 eV	4.81 eV
B2Ca1H2_ICSD_183133	0.13 eV	2.27 eV
B2Ca1H8_ICSD_163262	4.96 eV	5.00 eV
B2Ca1H8_ICSD_163263	5.22 eV	4.75 eV
B2Ca1H8_ICSD_163480	5.15 eV	5.16 eV
B2Ca1H8_ICSD_164182	4.94 eV	4.86 eV
B2Ca1H8_ICSD_168224	5.09 eV	5.15 eV
B2Ca1H8_ICSD_168225	5.09 eV	5.08 eV
B2Ca1Li4O6_ICSD_99503	4.74 eV	4.43 eV
B2Ca1O4_ICSD_20097	6.02 eV	5.45 eV
B2Ca1O4_ICSD_23241	6.06 eV	5.80 eV
B2Ca1O6Sn1_ICSD_30998	3.17 eV	3.27 eV
B2Ca1O8Si2_ICSD_26491	5.66 eV	5.59 eV
B2Ca2O5_ICSD_66516	4.75 eV	4.91 eV
B2Ca3O6_ICSD_23664	4.81 eV	4.48 eV
B2Cd1F1K1O6Zn2_ICSD_248025	3.06 eV	3.12 eV
B2Cd1H8_ICSD_262600	3.69 eV	3.63 eV
B2Cd2O5_ICSD_200621	2.59 eV	2.67 eV
B2Cd2O5_ICSD_281357	2.60 eV	2.57 eV
B2Cd3O6_ICSD_66170	1.62 eV	2.30 eV
B2Ce1Cl1O4_ICSD_413236	5.02 eV	4.44 eV
B2Ce3Cl3O6_ICSD_413237	4.32 eV	3.87 eV
B2Cl1La1O4_ICSD_413209	5.18 eV	4.62 eV
B2Cl1O4Pr1_ICSD_95851	3.90 eV	4.07 eV
B2Cl4_ICSD_31693	2.92 eV	3.76 eV
B2Co1O4_ICSD_422063	3.17 eV	3.23 eV
B2Co2O5_ICSD_24284	3.53 eV	2.49 eV
B2Co3O6_ICSD_24035	2.85 eV	2.24 eV
B2Cs1Li5O6_ICSD_61203	3.48 eV	3.99 eV
B2Cs1Nb1O6_ICSD_79703	3.30 eV	3.19 eV
B2Cs1O6Ta1_ICSD_80423	3.43 eV	3.52 eV
B2Cs3Li2Na1O6_ICSD_36531	3.41 eV	3.35 eV
B2Cu1O6Pb2_ICSD_155317	1.64 eV	1.66 eV
B2Cu1O6Sr2_ICSD_202934	1.59 eV	1.59 eV
B2Cu2O6Sr1_ICSD_247206	1.18 eV	1.20 eV
B2Cu2O6Sr1_ICSD_247207	1.18 eV	1.21 eV
B2Cu2O6Sr1_ICSD_80592	1.18 eV	1.24 eV

Supplementary Table 652. Five-fold cross validated predictions for the band gap energy (46/277).

system	calculated	predicted
B2Eu3O6_ICSD_86479	1.93 eV	1.56 eV
B2F10H1Nd1_ICSD_420467	4.85 eV	4.99 eV
B2F10H1Pr1_ICSD_420466	5.11 eV	5.03 eV
B2F3Gd3O6_ICSD_51140	5.35 eV	4.85 eV
B2F4_ICSD_27867	4.88 eV	6.58 eV
B2F8H6N2_ICSD_49614	7.44 eV	6.94 eV
B2Fe1O4_ICSD_420403	2.93 eV	2.74 eV
B2Fe1_ICSD_425310	0.68 eV	0.82 eV
B2Fe2H2K2O18P4_ICSD_407797	2.44 eV	3.05 eV
B2Ga2K2O7_ICSD_50039	3.85 eV	4.01 eV
B2Ga2O7Sr1_ICSD_91279	4.02 eV	3.84 eV
B2Ga6Ge6H8Na8O24_ICSD_188639	3.54 eV	3.70 eV
B2H16Na2O14_ICSD_30532	3.87 eV	4.96 eV
B2H4Li2O8_ICSD_100854	3.61 eV	5.29 eV
B2H6_ICSD_1312	5.15 eV	5.46 eV
B2H6_ICSD_413919	5.00 eV	5.51 eV
B2H8K1Li1_ICSD_173236	6.09 eV	5.71 eV
B2H8K1Na1_ICSD_163376	6.19 eV	6.07 eV
B2H8K1Na1_ICSD_163377	6.18 eV	4.66 eV
B2H8K1Na1_ICSD_163378	6.36 eV	5.68 eV
B2H8K1Na1_ICSD_163379	6.40 eV	6.41 eV
B2H8Mg1_ICSD_155717	6.07 eV	5.87 eV
B2H8Mg1_ICSD_161140	6.55 eV	5.81 eV
B2H8Mg1_ICSD_187436	6.60 eV	4.96 eV
B2H8Mg1_ICSD_187440	3.81 eV	5.60 eV
B2H8Mg1_ICSD_187459	5.99 eV	5.64 eV
B2H8Mg1_ICSD_262083	5.48 eV	5.94 eV
B2H8Zn1_ICSD_161376	4.89 eV	4.87 eV
B2Hg3O6_ICSD_409688	2.64 eV	2.12 eV
B2K1O6Ta1_ICSD_162214	3.65 eV	3.49 eV
B2K2La2O7_ICSD_174543	4.09 eV	4.01 eV
B2K2O6Zr1_ICSD_67982	3.40 eV	3.61 eV
B2K3Nb3O12_ICSD_85091	2.24 eV	2.31 eV
B2K3Nb3O12_ICSD_968	2.37 eV	2.42 eV
B2K3O12Ta3_ICSD_201143	2.88 eV	2.89 eV
B2K3O6Y1_ICSD_245925	3.19 eV	3.79 eV
B2Li2S5_ICSD_401723	2.45 eV	2.79 eV
B2Li2Se5_ICSD_411410	1.63 eV	1.70 eV
B2Li3O6Sc1_ICSD_241234	2.55 eV	3.84 eV
B2Li3O6Sc1_ICSD_261256	4.90 eV	4.60 eV
B2Mg1O6Sn1_ICSD_28266	3.10 eV	3.31 eV
B2Mg1O6Sr2_ICSD_240897	4.60 eV	4.48 eV
B2Mg2O5_ICSD_24789	5.08 eV	4.89 eV
B2Mg2O5_ICSD_81229	5.16 eV	5.15 eV
B2Mg3O6_ICSD_24036	5.14 eV	5.15 eV
B2Mn1O6Sn1_ICSD_79165	2.02 eV	2.36 eV
B2Na1O5Sc1_ICSD_409522	5.01 eV	4.66 eV
B2Na2S5_ICSD_401724	2.61 eV	2.59 eV
B2Na3O6Sc1_ICSD_262733	3.59 eV	3.76 eV
B2Na4O5_ICSD_10061	3.75 eV	3.44 eV

Supplementary Table 653. Five-fold cross validated predictions for the band gap energy (47/277).

system	calculated	predicted
B2Ni1O4_ICSD_418385	3.24 eV	2.93 eV
B2Ni3O6_ICSD_2016	2.55 eV	2.37 eV
B2O3_ICSD_24649	6.25 eV	6.25 eV
B2O3_ICSD_36066	6.26 eV	6.26 eV
B2O4Sr1_ICSD_203226	4.94 eV	5.23 eV
B2O4Sr1_ICSD_69102	6.37 eV	5.44 eV
B2O5Sr2_ICSD_50739	4.27 eV	4.48 eV
B2O5Zr1_ICSD_418931	3.79 eV	3.93 eV
B2O6Pb1Zn2_ICSD_171139	3.76 eV	3.11 eV
B2O6Sn1Sr1_ICSD_28267	3.11 eV	3.14 eV
B2O6Sr3_ICSD_93395	4.39 eV	4.34 eV
B2O6U1_ICSD_248127	3.79 eV	3.69 eV
B2O6U1_ICSD_49908	3.84 eV	3.72 eV
B2O7Se2_ICSD_172383	4.01 eV	3.78 eV
B2O8Si2Sr1_ICSD_83368	5.49 eV	5.44 eV
B2O9S2_ICSD_426544	6.68 eV	5.83 eV
B2S4Sr1_ICSD_71594	2.83 eV	2.63 eV
B2S6Sr3_ICSD_412879	2.32 eV	2.39 eV
B3Ba1Li1S6_ICSD_82353	2.84 eV	2.61 eV
B3Ba4N6Na1_ICSD_401210	2.05 eV	2.00 eV
B3Ba5Br1O9_ICSD_422557	3.76 eV	3.62 eV
B3Ba5C1N1O9_ICSD_414486	3.31 eV	3.03 eV
B3Ba5Cl1O9_ICSD_422556	3.71 eV	3.83 eV
B3Be2K1O7_ICSD_248202	5.42 eV	5.50 eV
B3Be2K1O7_ICSD_248203	4.91 eV	5.39 eV
B3Be2O7Rb1_ICSD_248205	4.79 eV	5.22 eV
B3Be3F4Na1O9Sr3_ICSD_423143	4.79 eV	5.33 eV
B3Bi1O6_ICSD_245893	3.94 eV	3.73 eV
B3Bi1O6_ICSD_416822	3.86 eV	3.74 eV
B3Ca1H7O9_ICSD_75922	5.41 eV	5.21 eV
B3Ca4Gd1O10_ICSD_86172	4.37 eV	4.11 eV
B3Ca4K1O9_ICSD_171422	4.60 eV	4.52 eV
B3Ca4La1O10_ICSD_180596	4.28 eV	4.19 eV
B3Ca4Li1N6_ICSD_83419	2.20 eV	2.30 eV
B3Ca4Na1O9_ICSD_171421	4.08 eV	4.38 eV
B3Ca5F1O9_ICSD_65763	4.25 eV	4.42 eV
B3Ce1O6_ICSD_413903	5.22 eV	4.94 eV
B3Ce1O6_ICSD_99691	5.15 eV	4.60 eV
B3Cl1Eu5O9_ICSD_422555	2.04 eV	1.98 eV
B3Cl1O9Sr5_ICSD_71871	3.94 eV	4.03 eV
B3Cl6N3_ICSD_14276	3.13 eV	3.06 eV
B3Cs1H8_ICSD_65947	5.97 eV	4.74 eV
B3Eu4Li1N6_ICSD_400465	0.55 eV	1.32 eV
B3Gd1O6_ICSD_8083	5.06 eV	4.81 eV
B3H12Y1_ICSD_169076	4.49 eV	4.79 eV
B3H2Na3O7_ICSD_1939	4.54 eV	4.58 eV
B3H3O14Zn8_ICSD_416894	3.15 eV	3.49 eV
B3H6N3_ICSD_401085	5.35 eV	4.89 eV
B3K1O6Zn1_ICSD_174357	4.75 eV	4.62 eV
B3K3O6_ICSD_16005	4.13 eV	3.95 eV

Supplementary Table 654. Five-fold cross validated predictions for the band gap energy (48/277).

system	calculated	predicted
B3K3S6_ICSD_79614	2.76 eV	2.61 eV
B3La1O6_ICSD_20355	5.07 eV	5.34 eV
B3La2Na3O9_ICSD_151884	3.74 eV	4.03 eV
B3La3N6_ICSD_410598	2.12 eV	2.17 eV
B3Li1N6Sr4_ICSD_402173	2.08 eV	2.06 eV
B3Li1O5_ICSD_415200	6.37 eV	6.00 eV
B3Li1S6Sr1_ICSD_79616	2.91 eV	2.61 eV
B3N6Na1Sr4_ICSD_92577	1.97 eV	2.08 eV
B3Na3O9Sc2_ICSD_245063	3.69 eV	4.00 eV
B3Na3S6_ICSD_79613	2.52 eV	2.67 eV
B3Nd1O6_ICSD_20075	3.59 eV	4.38 eV
B3O5Rb1_ICSD_91545	5.29 eV	5.42 eV
B3O5Tl1_ICSD_84855	4.22 eV	3.91 eV
B3O6Pr1_ICSD_95850	4.28 eV	4.39 eV
B3O6Rb3_ICSD_59826	3.93 eV	3.90 eV
B3O9Rb1Zn4_ICSD_92616	3.39 eV	3.40 eV
B3O9Sc1Sr3_ICSD_75339	4.85 eV	4.59 eV
B3Rb3S6_ICSD_79615	2.82 eV	2.67 eV
B3Si1_ICSD_412621	1.41 eV	1.18 eV
B4Ba2O11Sc2_ICSD_86436	3.71 eV	4.45 eV
B4Ba5F2O10_ICSD_73905	4.51 eV	4.35 eV
B4C1Cl6O1_ICSD_280617	4.05 eV	3.58 eV
B4C2Ca4H6Mg1O18_ICSD_80438	4.22 eV	4.12 eV
B4Ca1O7_ICSD_412710	6.31 eV	6.56 eV
B4Cl4_ICSD_27872	3.33 eV	3.16 eV
B4Co1O7_ICSD_420402	3.86 eV	3.49 eV
B4Cu1Er2O10_ICSD_401710	1.71 eV	1.84 eV
B4Cu1Ho2O10_ICSD_408029	1.72 eV	1.81 eV
B4Cu1Li6O10_ICSD_249215	3.23 eV	2.41 eV
B4Cu1O7_ICSD_391408	1.54 eV	1.91 eV
B4Dy1Mo1_ICSD_613645	0.31 eV	0.33 eV
B4Dy1W1_ICSD_613694	0.26 eV	0.28 eV
B4Er1Mo1_ICSD_613775	0.33 eV	0.33 eV
B4Er1W1_ICSD_613826	0.28 eV	0.29 eV
B4Fe1O7_ICSD_420401	3.46 eV	3.28 eV
B4Fe3La1O12_ICSD_83506	2.07 eV	2.02 eV
B4Fe3Nd1O12_ICSD_83507	1.57 eV	1.98 eV
B4Fe3O12Tb1_ICSD_96455	2.14 eV	2.20 eV
B4Ga2Li6O12_ICSD_9987	4.16 eV	4.15 eV
B4Ga3Nd1O12_ICSD_200321	3.56 eV	3.76 eV
B4Gd1Mo1_ICSD_614347	0.22 eV	0.27 eV
B4Gd1W1_ICSD_614393	0.20 eV	0.27 eV
B4H16Hf1_ICSD_35379	5.72 eV	3.95 eV
B4H16Na1Sc1_ICSD_166748	5.11 eV	4.99 eV
B4H16U1_ICSD_63132	2.85 eV	3.76 eV
B4Hg1O7_ICSD_415347	1.79 eV	3.07 eV
B4Ho1Mo1_ICSD_614475	0.32 eV	0.31 eV
B4Ho1W1_ICSD_614514	0.27 eV	0.29 eV
B4Ho2Ni1O10_ICSD_404814	3.48 eV	3.06 eV
B4La1O12Sc3_ICSD_83405	4.72 eV	4.59 eV

Supplementary Table 655. Five-fold cross validated predictions for the band gap energy (49/277).

system	calculated	predicted
B4La1O12Sc3_ICSD_89012	4.72 eV	4.43 eV
B4La1O12Sc3_ICSD_89013	4.24 eV	4.57 eV
B4Li2O7_ICSD_163177	5.62 eV	5.83 eV
B4Mn1O7_ICSD_391409	5.10 eV	3.75 eV
B4Mo1Tb1_ICSD_614843	0.30 eV	0.28 eV
B4Mo1Y1_ICSD_20081	0.31 eV	0.32 eV
B4Ni1O7_ICSD_391407	2.93 eV	3.39 eV
B4O11Sc2Sr2_ICSD_86435	4.64 eV	4.50 eV
B4O7Pb1_ICSD_185672	4.20 eV	3.44 eV
B4O7Sn1_ICSD_249206	3.55 eV	4.04 eV
B4O7Sr1_ICSD_27404	7.11 eV	6.17 eV
B4O7Zn1_ICSD_412688	5.88 eV	5.55 eV
B4Tb1W1_ICSD_615552	0.26 eV	0.25 eV
B4W1Y1_ICSD_615702	0.27 eV	0.26 eV
B5Ba2Li1O10_ICSD_71875	4.92 eV	4.72 eV
B5Ca1Cl1H2O10Sr1_ICSD_91540	4.95 eV	4.70 eV
B5Ca1Na3O10_ICSD_61165	4.43 eV	4.63 eV
B5Ca2Cl1H2O10_ICSD_100261	3.54 eV	4.73 eV
B5Ca2Cl1H2O10_ICSD_74548	5.01 eV	4.77 eV
B5Ca3La3O15_ICSD_93394	4.68 eV	4.40 eV
B5Cs2Li3O10_ICSD_180730	4.48 eV	4.29 eV
B5Cu1O10Tb1_ICSD_401453	2.08 eV	2.19 eV
B5H12N1O12_ICSD_90001	5.56 eV	5.59 eV
B5H2Li3O10_ICSD_20155	5.89 eV	5.28 eV
B5H8K1O12_ICSD_6292	5.60 eV	5.03 eV
B5H9_ICSD_24636	5.80 eV	4.62 eV
B5Na3O10Sr1_ICSD_260005	4.58 eV	4.63 eV
B6Ba2Ca1O12_ICSD_30890	4.96 eV	5.15 eV
B6Ba2Cd1O12_ICSD_425704	4.42 eV	3.97 eV
B6Ba2Co1O12_ICSD_391014	2.92 eV	3.52 eV
B6Ba2Mg1O12_ICSD_290356	5.19 eV	5.17 eV
B6Ba2Mn1O12_ICSD_391013	4.01 eV	3.40 eV
B6Ba2O12Zn1_ICSD_404486	4.31 eV	4.73 eV
B6Ba3Ge2O16_ICSD_261403	4.08 eV	4.09 eV
B6Br1K3O10_ICSD_172400	5.21 eV	4.54 eV
B6Co4O13_ICSD_96561	3.73 eV	3.14 eV
B6Cr1H16_ICSD_170564	2.33 eV	2.79 eV
B6Cs1Li1O10_ICSD_75995	5.07 eV	5.12 eV
B6Cs2H6_ICSD_65508	4.15 eV	4.24 eV
B6Cs2O10_ICSD_25815	5.27 eV	5.26 eV
B6Dy4O15_ICSD_412041	5.44 eV	5.30 eV
B6H10_ICSD_43253	4.00 eV	4.97 eV
B6H5K2N1O2_ICSD_67890	1.78 eV	3.33 eV
B6H6K2_ICSD_65507	5.02 eV	4.80 eV
B6Ho4O15_ICSD_412991	5.50 eV	5.28 eV
B6Na2O11Zn1_ICSD_167333	5.02 eV	4.77 eV
B6O13Zn4_ICSD_100290	4.04 eV	4.59 eV
B6O18Sc1Sr6Y1_ICSD_67648	4.68 eV	4.52 eV
B6O1_ICSD_71065	1.86 eV	2.75 eV
B6Si1_ICSD_20240	0.14 eV	0.95 eV

Supplementary Table 656. Five-fold cross validated predictions for the band gap energy (50/277).

system	calculated	predicted
B7Br1Cu3O13_ICSD_201347	0.80 eV	1.03 eV
B7Br1Mn3O13_ICSD_80420	3.81 eV	3.04 eV
B7Cl1Co3O13_ICSD_158297	3.02 eV	3.19 eV
B7Cl1Cr3O13_ICSD_4231	2.59 eV	2.69 eV
B7Cl1Mg3O13_ICSD_30351	5.92 eV	5.48 eV
B7Cl1O13Zn3_ICSD_55444	4.95 eV	4.75 eV
B7Cr3I1O13_ICSD_62178	1.85 eV	2.55 eV
B7Cu3I1O13_ICSD_61058	0.33 eV	0.94 eV
B7Fe3I1O13_ICSD_78420	2.70 eV	2.43 eV
B7I1Ni3O13_ICSD_27946	1.98 eV	2.33 eV
B8Ca1H4O15_ICSD_250323	5.51 eV	5.53 eV
B8La9Na3O27_ICSD_95753	3.92 eV	4.10 eV
B8S16_ICSD_15267	2.55 eV	2.72 eV
B9Ba1Li1O15_ICSD_93013	5.66 eV	5.56 eV
B9Ba1Na1O15_ICSD_93014	5.48 eV	5.40 eV
B9Mg1N1_ICSD_280938	1.68 eV	1.43 eV
Ba10N12Ti4_ICSD_79102	1.56 eV	1.09 eV
Ba10O24P6S1_ICSD_410785	3.13 eV	3.96 eV
Ba1Be1F1O4P1_ICSD_200922	3.74 eV	4.63 eV
Ba1Be1F4_ICSD_414412	7.55 eV	7.09 eV
Ba1Be1La2O5_ICSD_65292	3.82 eV	3.56 eV
Ba1Be1O4Si1_ICSD_86792	4.60 eV	4.39 eV
Ba1Be2N2_ICSD_415304	1.17 eV	1.92 eV
Ba1Be2O7Si2_ICSD_100030	5.54 eV	5.51 eV
Ba1Be2O7Si2_ICSD_263133	5.57 eV	5.21 eV
Ba1Bi1Cl1O2_ICSD_79532	3.18 eV	3.12 eV
Ba1Bi1I1O2_ICSD_97511	2.96 eV	2.81 eV
Ba1Bi1Na1_ICSD_413810	0.30 eV	0.44 eV
Ba1Bi1O3_ICSD_151895	0.13 eV	0.77 eV
Ba1Bi1O3_ICSD_172756	0.69 eV	0.71 eV
Ba1Bi1Se3_ICSD_10505	0.72 eV	0.81 eV
Ba1Bi2La1S6_ICSD_85461	0.67 eV	1.04 eV
Ba1Bi2Mg2_ICSD_100049	0.47 eV	0.39 eV
Ba1Bi2Mo4O16_ICSD_416023	2.96 eV	2.34 eV
Ba1Br1Cl1_ICSD_35458	4.93 eV	4.60 eV
Ba1Br1F1_ICSD_155005	4.87 eV	4.81 eV
Ba1Br1H1_ICSD_25544	3.21 eV	3.37 eV
Ba1Br2H4O2_ICSD_33949	4.37 eV	4.12 eV
Ba1Br2O6_ICSD_40287	4.33 eV	3.99 eV
Ba1Br2O6_ICSD_66035	4.18 eV	4.02 eV
Ba1Br2_ICSD_262675	4.38 eV	4.26 eV
Ba1Br2_ICSD_79892	3.92 eV	4.05 eV
Ba1Br6Th1_ICSD_78770	2.93 eV	2.57 eV
Ba1C1Cl1H2N1O1S1_ICSD_82878	4.85 eV	4.21 eV
Ba1C1Cl1N1S1_ICSD_94400	4.11 eV	3.61 eV
Ba1C1Cu1F2O3_ICSD_79864	1.72 eV	2.02 eV
Ba1C1F2Mn1O3_ICSD_95740	4.11 eV	3.15 eV
Ba1C1F2O3Zn1_ICSD_95739	5.36 eV	4.47 eV
Ba1C1N2_ICSD_75041	2.30 eV	2.97 eV
Ba1C1O3_ICSD_158389	4.44 eV	4.05 eV

Supplementary Table 657. Five-fold cross validated predictions for the band gap energy (51/277).

system	calculated	predicted
Ba1C1O3_ICSD_91897	3.97 eV	4.39 eV
Ba1C1Si1_ICSD_168411	0.97 eV	0.91 eV
Ba1C1Si1_ICSD_168413	0.30 eV	0.79 eV
Ba1C2Ca1O6_ICSD_100477	4.12 eV	4.46 eV
Ba1C2Ca1O6_ICSD_157982	4.68 eV	4.44 eV
Ba1C2Ca2F2O6_ICSD_245746	4.73 eV	4.60 eV
Ba1C2Ce1F1O6_ICSD_37195	3.28 eV	3.43 eV
Ba1C2Ce1F1O6_ICSD_74178	4.29 eV	3.54 eV
Ba1C2F2O6Pb2_ICSD_280899	4.06 eV	3.13 eV
Ba1C2H2O4_ICSD_151335	4.68 eV	4.26 eV
Ba1C2H2O5_ICSD_151115	3.16 eV	3.52 eV
Ba1C2H4O6_ICSD_162707	3.42 eV	3.63 eV
Ba1C2Mg1O6_ICSD_24435	4.17 eV	4.16 eV
Ba1C2Mg1O6_ICSD_89038	4.08 eV	4.27 eV
Ba1C2N2S2_ICSD_94428	3.85 eV	3.63 eV
Ba1C2O4_ICSD_261703	3.25 eV	3.43 eV
Ba1C2_ICSD_168408	2.88 eV	2.05 eV
Ba1C2_ICSD_186576	1.38 eV	1.47 eV
Ba1C2_ICSD_88098	1.61 eV	1.46 eV
Ba1C2_ICSD_88102	1.91 eV	2.38 eV
Ba1C4Ce2F1Na1O12_ICSD_77499	4.20 eV	4.23 eV
Ba1C4H6O7_ICSD_62387	2.68 eV	3.34 eV
Ba1C4H8N4O4Pt1_ICSD_851	2.21 eV	2.61 eV
Ba1C4O4_ICSD_412830	2.56 eV	2.95 eV
Ba1C8N8Pt2Rb2_ICSD_409861	2.49 eV	3.14 eV
Ba1Ca1Fe4O7_ICSD_161791	1.19 eV	1.23 eV
Ba1Ca1Fe4O8_ICSD_15174	1.59 eV	1.51 eV
Ba1Ca1Ga4O8_ICSD_280042	3.19 eV	3.05 eV
Ba1Ca1O4Si1_ICSD_67092	4.38 eV	4.26 eV
Ba1Ca1O7V2_ICSD_80810	3.22 eV	2.76 eV
Ba1Ca1Pb1_ICSD_615799	0.22 eV	0.26 eV
Ba1Ca1Sn1_ICSD_58641	0.28 eV	0.32 eV
Ba1Ca2Mg1O8Si2_ICSD_422406	4.99 eV	4.46 eV
Ba1Ca2N12P6_ICSD_415714	4.07 eV	3.89 eV
Ba1Ca2O9Si3_ICSD_24426	4.57 eV	4.46 eV
Ba1Ca4Co2N4_ICSD_409921	0.52 eV	0.65 eV
Ba1Ca4Cu2N4_ICSD_86066	0.35 eV	0.93 eV
Ba1Cd0.333O3Ta0.667_ICSD_156335	3.60 eV	3.41 eV
Ba1Cd1F1Sb1_ICSD_421815	0.38 eV	0.98 eV
Ba1Cd1Ge1S4_ICSD_23343	2.68 eV	2.12 eV
Ba1Cd1O7P2_ICSD_72673	3.67 eV	3.49 eV
Ba1Cd1O7V2_ICSD_80811	3.09 eV	2.77 eV
Ba1Cd1S2_ICSD_66655	1.69 eV	1.75 eV
Ba1Cd1Sb2Yb1_ICSD_422280	0.18 eV	0.31 eV
Ba1Cd2P2_ICSD_615814	0.78 eV	0.68 eV
Ba1Ce1Cu1S3_ICSD_659174	1.21 eV	1.20 eV
Ba1Ce1Cu1Se3_ICSD_659176	0.94 eV	0.99 eV
Ba1Ce1N2_ICSD_74791	0.52 eV	1.05 eV
Ba1Ce1O3_ICSD_29109	2.96 eV	2.24 eV
Ba1Ce1O3_ICSD_79627	2.89 eV	3.03 eV

Supplementary Table 658. Five-fold cross validated predictions for the band gap energy (52/277).

system	calculated	predicted
Ba1Ce1O3_ICSD_88590	2.89 eV	2.74 eV
Ba1Ce1O3_ICSD_94347	2.87 eV	2.84 eV
Ba1Ce2Co1S5_ICSD_93708	1.14 eV	0.98 eV
Ba1Ce2Mn1S5_ICSD_91228	0.37 eV	1.02 eV
Ba1Ce2S5Zn1_ICSD_93712	1.36 eV	1.35 eV
Ba1Cl1Cu1O4P1_ICSD_79883	1.40 eV	1.68 eV
Ba1Cl1F1_ICSD_35491	5.44 eV	5.38 eV
Ba1Cl1H1O1_ICSD_74721	4.86 eV	4.53 eV
Ba1Cl1H1_ICSD_37201	3.48 eV	3.26 eV
Ba1Cl1O2Sb1_ICSD_200962	2.88 eV	2.85 eV
Ba1Cl1O3V1_ICSD_50786	2.34 eV	2.16 eV
Ba1Cl2H2O1_ICSD_60886	5.12 eV	4.81 eV
Ba1Cl2H4O2_ICSD_2254	4.54 eV	4.76 eV
Ba1Cl2H6O11_ICSD_65020	5.30 eV	4.74 eV
Ba1Cl2Hg2O2_ICSD_77509	1.60 eV	1.80 eV
Ba1Cl2O6_ICSD_40285	5.27 eV	4.36 eV
Ba1Cl2_ICSD_183924	5.13 eV	4.70 eV
Ba1Cl2_ICSD_2190	4.57 eV	4.88 eV
Ba1Cl2_ICSD_2191	5.44 eV	4.13 eV
Ba1Cl2_ICSD_79891	3.54 eV	4.52 eV
Ba1Cl4Pd1_ICSD_411837	2.12 eV	2.25 eV
Ba1Cl4Zn1_ICSD_410193	4.96 eV	4.89 eV
Ba1Cl4Zn1_ICSD_411951	4.90 eV	4.71 eV
Ba1Cl5Gd1_ICSD_407832	3.88 eV	3.90 eV
Ba1Co1Dy2O5_ICSD_74974	2.35 eV	2.13 eV
Ba1Co1Dy2O5_ICSD_85060	1.90 eV	1.74 eV
Ba1Co1Er2O5_ICSD_65469	1.94 eV	1.86 eV
Ba1Co1Er2O5_ICSD_73168	2.37 eV	2.20 eV
Ba1Co1F4_ICSD_261189	4.36 eV	3.74 eV
Ba1Co1F6Li1_ICSD_41178	2.98 eV	3.75 eV
Ba1Co1Ho2O5_ICSD_65467	2.36 eV	2.18 eV
Ba1Co1La2S5_ICSD_95267	1.18 eV	1.06 eV
Ba1Co1Lu2O5_ICSD_74977	2.29 eV	1.81 eV
Ba1Co1Nd2O5_ICSD_202973	1.73 eV	1.71 eV
Ba1Co1Nd2S5_ICSD_96554	1.17 eV	1.22 eV
Ba1Co1O2_ICSD_25813	1.63 eV	1.42 eV
Ba1Co1O3_ICSD_88670	0.60 eV	1.24 eV
Ba1Co1O4Si1_ICSD_73778	3.46 eV	2.79 eV
Ba1Co1O5Y2_ICSD_85061	2.36 eV	1.72 eV
Ba1Co1O7P2_ICSD_202853	3.92 eV	3.48 eV
Ba1Co2H2O9P2_ICSD_84830	3.70 eV	3.19 eV
Ba1Co2O7Si2_ICSD_74160	3.60 eV	2.96 eV
Ba1Co3H2O10V2_ICSD_236321	2.64 eV	2.11 eV
Ba1Co4Ho1O7_ICSD_420423	0.35 eV	0.85 eV
Ba1Co4Ho1O7_ICSD_420426	0.96 eV	0.74 eV
Ba1Co4Ho1O7_ICSD_420427	0.99 eV	0.91 eV
Ba1Co4O7Y1_ICSD_188853	0.82 eV	0.91 eV
Ba1Co4O7Y1_ICSD_95745	0.95 eV	1.02 eV
Ba1Cr1F5_ICSD_31705	3.49 eV	2.87 eV
Ba1Cr1O4_ICSD_188534	2.92 eV	2.78 eV

Supplementary Table 659. Five-fold cross validated predictions for the band gap energy (53/277).

system	calculated	predicted
Ba1Cr1O4_ICSD_62560	2.93 eV	2.76 eV
Ba1Cr1S2_ICSD_165626	0.33 eV	0.84 eV
Ba1Cs2H6N2O12_ICSD_411040	0.70 eV	2.82 eV
Ba1Cu1Dy1Te3_ICSD_85601	0.61 eV	0.89 eV
Ba1Cu1Dy2O5_ICSD_93463	1.06 eV	1.17 eV
Ba1Cu1Er1S3_ICSD_78176	1.32 eV	1.31 eV
Ba1Cu1Er1Se3_ICSD_659168	1.10 eV	1.18 eV
Ba1Cu1Er2O5_ICSD_72165	1.04 eV	1.13 eV
Ba1Cu1F1S1_ICSD_183712	1.93 eV	1.82 eV
Ba1Cu1F1Se1_ICSD_75585	1.74 eV	1.60 eV
Ba1Cu1F1Te1_ICSD_245624	1.29 eV	1.53 eV
Ba1Cu1F4_ICSD_9930	1.66 eV	2.37 eV
Ba1Cu1F7Fe1_ICSD_60952	2.15 eV	2.07 eV
Ba1Cu1Gd1Se3_ICSD_280698	1.01 eV	0.97 eV
Ba1Cu1Ho2O5_ICSD_72163	1.05 eV	1.12 eV
Ba1Cu1La1S3_ICSD_659175	1.16 eV	1.28 eV
Ba1Cu1La1Se3_ICSD_659178	1.01 eV	1.10 eV
Ba1Cu1La1Te3_ICSD_88715	0.75 eV	0.92 eV
Ba1Cu1N1_ICSD_86064	0.15 eV	0.75 eV
Ba1Cu1Na2O8V2_ICSD_72364	1.75 eV	1.63 eV
Ba1Cu1Nd1S3_ICSD_659173	1.10 eV	1.30 eV
Ba1Cu1Nd2O5_ICSD_154576	1.64 eV	1.61 eV
Ba1Cu1O10Si4_ICSD_71864	2.36 eV	2.10 eV
Ba1Cu1O5Pr2_ICSD_85952	1.67 eV	1.37 eV
Ba1Cu1O5Y2_ICSD_72572	1.09 eV	1.43 eV
Ba1Cu1O6Se2_ICSD_202387	1.60 eV	1.74 eV
Ba1Cu1O6Se2_ICSD_202388	1.55 eV	1.50 eV
Ba1Cu1O7V2_ICSD_40839	1.71 eV	1.68 eV
Ba1Cu1S3Sc1_ICSD_659165	1.17 eV	1.19 eV
Ba1Cu1S3Y1_ICSD_659166	1.34 eV	1.20 eV
Ba1Cu1Se3Y1_ICSD_659169	1.11 eV	1.17 eV
Ba1Cu1Te3Y1_ICSD_88716	0.62 eV	0.69 eV
Ba1Cu2Ge1S4_ICSD_10006	1.32 eV	1.05 eV
Ba1Cu2Ge1Se4_ICSD_411404	0.68 eV	0.82 eV
Ba1Cu2Ge2O7_ICSD_77133	0.99 eV	1.20 eV
Ba1Cu2O2_ICSD_9456	1.76 eV	1.46 eV
Ba1Cu2O7Si2_ICSD_51281	1.27 eV	1.43 eV
Ba1Cu2S2_ICSD_89573	1.24 eV	1.20 eV
Ba1Cu2S2_ICSD_89575	0.70 eV	0.99 eV
Ba1Cu2S4Sn1_ICSD_52685	0.53 eV	1.03 eV
Ba1Cu2Se2_ICSD_89574	0.82 eV	0.82 eV
Ba1Cu2Se2_ICSD_89576	0.55 eV	0.65 eV
Ba1Cu2Se4Sn1_ICSD_170857	0.58 eV	0.67 eV
Ba1Cu2Te2_ICSD_51444	0.78 eV	0.82 eV
Ba1Cu3H2O10V2_ICSD_67726	1.20 eV	1.45 eV
Ba1Cu3O4_ICSD_83079	0.67 eV	1.14 eV
Ba1Cu4O17P4V1_ICSD_406667	1.04 eV	1.65 eV
Ba1Cu4S3_ICSD_15138	0.84 eV	0.93 eV
Ba1Cu4S3_ICSD_15139	0.52 eV	0.71 eV
Ba1Cu5La4O12_ICSD_79398	0.34 eV	0.94 eV

Supplementary Table 660. Five-fold cross validated predictions for the band gap energy (54/277).

system	calculated	predicted
Ba1Dy1Fe2O4.996_ICSD_99611	0.45 eV	1.02 eV
Ba1Dy2Ni1O5_ICSD_72627	1.92 eV	1.92 eV
Ba1Dy2O5Pd1_ICSD_404496	2.36 eV	2.37 eV
Ba1Dy2S4_ICSD_615841	1.75 eV	1.70 eV
Ba1Dy2Se4_ICSD_615842	1.37 eV	1.22 eV
Ba1Dy2Te4_ICSD_90334	0.78 eV	0.83 eV
Ba1Er2F8_ICSD_151699	7.23 eV	6.94 eV
Ba1Er2Ni1O5_ICSD_69323	1.98 eV	1.84 eV
Ba1Er2O10Si3_ICSD_167615	4.79 eV	4.40 eV
Ba1Er2S4_ICSD_188656	1.74 eV	1.73 eV
Ba1Er2Se4_ICSD_615844	1.37 eV	1.32 eV
Ba1Er2Te4_ICSD_90337	0.78 eV	0.83 eV
Ba1F10Te2_ICSD_81862	4.88 eV	4.05 eV
Ba1F10Zr2_ICSD_202530	5.76 eV	5.33 eV
Ba1F11Li1Zr2_ICSD_67512	5.73 eV	5.95 eV
Ba1F12Sb2_ICSD_39346	4.08 eV	4.37 eV
Ba1F1I1_ICSD_1128	4.02 eV	3.70 eV
Ba1F1O6P1U1_ICSD_249983	3.10 eV	3.56 eV
Ba1F2Fe2O7P2_ICSD_88824	3.61 eV	2.95 eV
Ba1F2_ICSD_181246	6.61 eV	5.78 eV
Ba1F2_ICSD_183923	6.81 eV	6.12 eV
Ba1F2_ICSD_41650	5.34 eV	6.00 eV
Ba1F3H1_ICSD_35409	5.50 eV	5.92 eV
Ba1F3Li1_ICSD_45310	6.57 eV	6.17 eV
Ba1F4Fe1_ICSD_82765	3.07 eV	3.21 eV
Ba1F4Mg1_ICSD_182596	6.78 eV	6.75 eV
Ba1F4Mg1_ICSD_182599	6.81 eV	6.73 eV
Ba1F4Mn1_ICSD_182603	3.31 eV	2.91 eV
Ba1F4Ni1_ICSD_261190	3.99 eV	3.73 eV
Ba1F4O1Ti1_ICSD_72740	3.72 eV	4.04 eV
Ba1F4Pd1_ICSD_108991	2.55 eV	2.50 eV
Ba1F4Sn1_ICSD_166207	3.74 eV	3.95 eV
Ba1F4Zn1_ICSD_402925	5.72 eV	5.50 eV
Ba1F5Ga1_ICSD_200316	5.86 eV	5.62 eV
Ba1F5Mn1_ICSD_38317	1.83 eV	2.93 eV
Ba1F5Sb1_ICSD_68455	4.74 eV	4.42 eV
Ba1F6Ge1_ICSD_26614	5.55 eV	5.20 eV
Ba1F6Ni1_ICSD_35396	1.72 eV	2.97 eV
Ba1F6Pb1_ICSD_25521	2.86 eV	3.45 eV
Ba1F6Si1_ICSD_26613	7.38 eV	6.55 eV
Ba1F6Sn1_ICSD_33788	4.45 eV	3.97 eV
Ba1F6Te1_ICSD_88416	4.79 eV	4.52 eV
Ba1F6Ti1_ICSD_33789	4.80 eV	4.44 eV
Ba1F6Zr1_ICSD_1697	6.32 eV	6.16 eV
Ba1F6Zr1_ICSD_36122	6.26 eV	5.95 eV
Ba1F7Fe1Zn1_ICSD_36001	3.58 eV	3.27 eV
Ba1F7Ga1Mn1_ICSD_201525	3.71 eV	3.56 eV
Ba1F7Na1Zr1_ICSD_67515	6.07 eV	6.26 eV
Ba1F7Ta1_ICSD_417251	5.82 eV	5.20 eV
Ba1F8Tm2_ICSD_20103	1.97 eV	4.34 eV

Supplementary Table 661. Five-fold cross validated predictions for the band gap energy (55/277).

system	calculated	predicted
Ba1Fe1H1O5P1_ICSD_174376	3.18 eV	2.86 eV
Ba1Fe1K1O3_ICSD_412877	2.14 eV	1.77 eV
Ba1Fe1La2S5_ICSD_51602	0.95 eV	0.97 eV
Ba1Fe1Nd2S5_ICSD_51605	1.11 eV	1.11 eV
Ba1Fe1O10Si4_ICSD_156832	3.28 eV	3.00 eV
Ba1Fe1O10Si4_ICSD_31205	3.31 eV	2.95 eV
Ba1Fe1Pr2S5_ICSD_51604	1.27 eV	1.05 eV
Ba1Fe2O4_ICSD_171001	1.54 eV	1.32 eV
Ba1Fe2O4_ICSD_2769	1.54 eV	1.50 eV
Ba1Fe2O5Y1_ICSD_281203	0.48 eV	0.99 eV
Ba1Fe2S3_ICSD_16307	0.93 eV	0.71 eV
Ba1Fe2S4_ICSD_23081	0.14 eV	0.54 eV
Ba1Fe2Se3_ICSD_290594	0.61 eV	0.71 eV
Ba1Fe2Se3_ICSD_290595	0.92 eV	0.78 eV
Ba1Fe2Se3_ICSD_424315	0.62 eV	0.71 eV
Ba1Fe4O8Sr1_ICSD_1943	1.62 eV	1.42 eV
Ba1Fe4O8Sr1_ICSD_37011	1.63 eV	1.57 eV
Ba1Ga1Ge1H1_ICSD_173573	0.21 eV	0.41 eV
Ba1Ga1H1Si1_ICSD_173572	0.54 eV	0.48 eV
Ba1Ga1H1Sn1_ICSD_173574	0.15 eV	0.40 eV
Ba1Ga1H5_ICSD_240693	3.11 eV	3.22 eV
Ba1Ga1La1O4_ICSD_180536	3.93 eV	3.70 eV
Ba1Ga1Nd1O4_ICSD_69641	3.71 eV	3.43 eV
Ba1Ga2P2_ICSD_380479	1.42 eV	0.84 eV
Ba1Ga2S4_ICSD_615871	2.96 eV	2.73 eV
Ba1Ga2Sb2_ICSD_280662	0.10 eV	0.30 eV
Ba1Ga2Se4_ICSD_24386	1.86 eV	1.62 eV
Ba1Ga4S7_ICSD_162960	2.57 eV	2.50 eV
Ba1Gd2O5Pd1_ICSD_83233	2.41 eV	2.43 eV
Ba1Gd2O5Pt1_ICSD_202171	2.92 eV	2.39 eV
Ba1Gd2O7Sc2_ICSD_167604	3.61 eV	3.44 eV
Ba1Gd2S4_ICSD_615879	1.62 eV	1.53 eV
Ba1Ge1O3_ICSD_23925	3.49 eV	2.96 eV
Ba1Ge1O8P2_ICSD_423040	4.07 eV	4.12 eV
Ba1Ge2O5_ICSD_60061	1.74 eV	2.32 eV
Ba1Ge2P2_ICSD_26416	0.49 eV	0.71 eV
Ba1Ge2_ICSD_615881	0.47 eV	0.39 eV
Ba1Ge3O9Sn1_ICSD_10384	2.70 eV	2.58 eV
Ba1H1I1_ICSD_37203	2.80 eV	3.20 eV
Ba1H2N2O5_ICSD_200864	2.43 eV	2.63 eV
Ba1H2N2O5_ICSD_65641	2.42 eV	2.63 eV
Ba1H2Ni3O10V2_ICSD_424330	2.22 eV	2.00 eV
Ba1H2O14P4Si1_ICSD_41357	5.71 eV	5.18 eV
Ba1H2O14Se4V2_ICSD_79518	2.11 eV	2.19 eV
Ba1H2O4Sn2_ICSD_37115	2.15 eV	2.77 eV
Ba1H2O8S2_ICSD_62494	5.58 eV	5.22 eV
Ba1H2_ICSD_615909	2.87 eV	2.44 eV
Ba1H3Li1_ICSD_23977	1.15 eV	1.88 eV
Ba1H3Li1_ICSD_416463	2.21 eV	1.65 eV
Ba1H3Na1Pd1_ICSD_165609	1.55 eV	1.93 eV

Supplementary Table 662. Five-fold cross validated predictions for the band gap energy (56/277).

system	calculated	predicted
Ba1H4I2O2_ICSD_407360	3.67 eV	3.66 eV
Ba1H4O3_ICSD_63017	4.25 eV	4.16 eV
Ba1H4O4P2_ICSD_59934	5.31 eV	5.04 eV
Ba1H4O8P2_ICSD_1297	5.57 eV	5.10 eV
Ba1H6O8Si2_ICSD_26971	5.06 eV	4.62 eV
Ba1H7Re1_ICSD_247116	1.00 eV	2.48 eV
Ba1H8O5_ICSD_67109	4.80 eV	4.45 eV
Ba1H9Re1_ICSD_247104	3.85 eV	3.48 eV
Ba1H9Re1_ICSD_247107	3.67 eV	2.65 eV
Ba1H9Re1_ICSD_247109	2.89 eV	2.52 eV
Ba1H9Re1_ICSD_247110	2.89 eV	3.60 eV
Ba1H9Re1_ICSD_247111	0.77 eV	2.74 eV
Ba1H9Re1_ICSD_75460	3.79 eV	3.42 eV
Ba1Hf1N2_ICSD_184055	1.26 eV	1.12 eV
Ba1Hf1O8P2_ICSD_245690	4.92 eV	4.57 eV
Ba1Hf1O9Si3_ICSD_183835	4.70 eV	4.45 eV
Ba1Hg1O2_ICSD_68616	2.17 eV	1.90 eV
Ba1Hg1O2_ICSD_83411	2.26 eV	1.98 eV
Ba1Hg1O5Ru1_ICSD_81070	1.03 eV	1.32 eV
Ba1Hg1S2_ICSD_32648	1.15 eV	1.31 eV
Ba1Hg1S4Sn1_ICSD_10456	1.70 eV	1.51 eV
Ba1Ho2Ni1O5_ICSD_67930	1.97 eV	1.87 eV
Ba1Ho2O4_ICSD_154812	3.23 eV	3.26 eV
Ba1Ho2O5Pd1_ICSD_404497	2.31 eV	2.35 eV
Ba1Ho2S4_ICSD_615930	1.74 eV	1.72 eV
Ba1Ho2Te4_ICSD_90336	0.78 eV	0.79 eV
Ba1I2O6_ICSD_23276	3.54 eV	3.06 eV
Ba1I2_ICSD_15707	3.47 eV	2.85 eV
Ba1I2_ICSD_36210	3.06 eV	2.71 eV
Ba1I2_ICSD_79893	1.73 eV	2.92 eV
Ba1I6U1_ICSD_78772	2.18 eV	1.95 eV
Ba1In2La2O7_ICSD_95984	2.64 eV	2.53 eV
Ba1In2Nd2O7_ICSD_168283	2.57 eV	2.39 eV
Ba1In2O14P4_ICSD_180003	4.09 eV	3.81 eV
Ba1In2P2_ICSD_260564	0.34 eV	0.56 eV
Ba1In2Te4_ICSD_41168	0.98 eV	0.79 eV
Ba1K1Nb1S4_ICSD_415335	2.21 eV	1.81 eV
Ba1K1O4P1_ICSD_202430	4.96 eV	4.83 eV
Ba1K1O4V1_ICSD_418461	3.97 eV	3.26 eV
Ba1K1P1S4_ICSD_414639	2.32 eV	2.27 eV
Ba1K1P1Se4_ICSD_414637	1.21 eV	1.56 eV
Ba1K4O9Si3_ICSD_246254	4.22 eV	3.81 eV
Ba1K4S8V2_ICSD_240378	1.76 eV	1.73 eV
Ba1La1Sb2Se6_ICSD_421269	0.52 eV	0.68 eV
Ba1La2Mn1S5_ICSD_90640	1.20 eV	0.98 eV
Ba1La2O14Te5_ICSD_417616	3.23 eV	2.89 eV
Ba1La2O5Pt1_ICSD_68794	2.84 eV	2.47 eV
Ba1La2O5Zn1_ICSD_172768	3.26 eV	3.09 eV
Ba1La2O7Sc2_ICSD_167599	3.80 eV	3.56 eV
Ba1La2S5Zn1_ICSD_93711	1.40 eV	1.51 eV

Supplementary Table 663. Five-fold cross validated predictions for the band gap energy (57/277).

system	calculated	predicted
Ba1Li1P1_ICSD_416890	0.97 eV	0.80 eV
Ba1Li1P1_ICSD_56444	0.70 eV	0.90 eV
Ba1Li1Sb1_ICSD_280574	0.70 eV	0.64 eV
Ba1Li2Mg1O8P2_ICSD_236294	5.56 eV	4.88 eV
Ba1Li2O4Si1_ICSD_180289	4.38 eV	4.21 eV
Ba1Lu2S4_ICSD_422891	1.73 eV	1.69 eV
Ba1Mg0.333Nb0.667O3_ICSD_95497	2.91 eV	3.01 eV
Ba1Mg0.333O3Ta0.667_ICSD_95495	3.40 eV	3.47 eV
Ba1Mg1Na2O8P2_ICSD_262716	5.02 eV	4.74 eV
Ba1Mg1O4Si1_ICSD_73776	4.12 eV	4.34 eV
Ba1Mg1O7Te2_ICSD_262408	1.84 eV	2.63 eV
Ba1Mg2O8V2_ICSD_20429	3.54 eV	2.96 eV
Ba1Mg2P2_ICSD_30914	1.14 eV	1.12 eV
Ba1Mg2Sb2_ICSD_100047	0.85 eV	0.71 eV
Ba1Mn1O3_ICSD_10250	0.73 eV	0.84 eV
Ba1Mn1O3_ICSD_10331	1.94 eV	1.34 eV
Ba1Mn1O3_ICSD_23874	0.67 eV	0.96 eV
Ba1Mn1O3_ICSD_66822	0.85 eV	1.21 eV
Ba1Mn1O3_ICSD_89995	1.96 eV	1.22 eV
Ba1Mn1O4Rb1_ICSD_80640	1.71 eV	1.61 eV
Ba1Mn1O7P2_ICSD_78658	3.89 eV	3.16 eV
Ba1Mn1Pr2S5_ICSD_91229	1.12 eV	1.06 eV
Ba1Mn1S2_ICSD_31453	1.54 eV	1.33 eV
Ba1Mn2O14Si4Sr2_ICSD_39593	1.61 eV	2.49 eV
Ba1Mn2O3_ICSD_10038	0.24 eV	1.13 eV
Ba1Mn2O8_ICSD_23443	1.85 eV	1.14 eV
Ba1Mn2O8_ICSD_24128	0.20 eV	1.30 eV
Ba1Mo1O4_ICSD_16166	3.95 eV	3.13 eV
Ba1Mo1O6Se1_ICSD_82255	2.99 eV	2.78 eV
Ba1Mo1O8P2_ICSD_79507	0.82 eV	2.20 eV
Ba1Mo2O11Se2_ICSD_82256	2.43 eV	2.44 eV
Ba1Mo2O12P2_ICSD_62833	2.81 eV	2.63 eV
Ba1Mo2O14P3_ICSD_153067	1.01 eV	2.08 eV
Ba1Mo2O14P4_ICSD_72339	2.80 eV	1.90 eV
Ba1Mo2O9Te1_ICSD_159460	2.74 eV	2.75 eV
Ba1Mo4O16P2_ICSD_75147	2.09 eV	1.75 eV
Ba1Mo6S8_ICSD_65775	0.15 eV	0.53 eV
Ba1N10Si7_ICSD_405772	3.92 eV	3.71 eV
Ba1N12P6Sr2_ICSD_415716	4.15 eV	3.68 eV
Ba1N2O2Si2_ICSD_419450	2.99 eV	3.75 eV
Ba1N2O4_ICSD_29510	2.37 eV	2.95 eV
Ba1N2O6_ICSD_56087	3.56 eV	2.97 eV
Ba1N2Si1_ICSD_170265	2.92 eV	2.56 eV
Ba1N2Zr1_ICSD_74904	0.99 eV	1.11 eV
Ba1N4P2_ICSD_414350	3.91 eV	3.42 eV
Ba1N6_ICSD_412253	3.81 eV	3.23 eV
Ba1N7Si4Y1_ICSD_98276	2.73 eV	3.01 eV
Ba1N7Si4Yb1_ICSD_160649	0.09 eV	0.61 eV
Ba1N8O1Si6_ICSD_415272	4.22 eV	3.80 eV
Ba1N8Si6_ICSD_417444	3.26 eV	3.42 eV

Supplementary Table 664. Five-fold cross validated predictions for the band gap energy (58/277).

system	calculated	predicted
Ba1Na1O7Sc1Si2_ICSD_166998	4.53 eV	4.57 eV
Ba1Na1P1_ICSD_402227	0.84 eV	0.87 eV
Ba1Na2O6Si2_ICSD_10217	4.16 eV	4.23 eV
Ba1Nb0.67O3Sr0.33_ICSD_186191	3.03 eV	3.29 eV
Ba1Nb2O11V2_ICSD_165097	2.33 eV	2.26 eV
Ba1Nb2O6_ICSD_39272	2.64 eV	2.46 eV
Ba1Nb2O6_ICSD_39320	3.00 eV	3.02 eV
Ba1Nb8O14_ICSD_79976	0.31 eV	1.02 eV
Ba1Nd2O5Pd1_ICSD_62609	2.64 eV	2.47 eV
Ba1Nd2O5Pt1_ICSD_47178	2.73 eV	2.45 eV
Ba1Nd2O5Zn1_ICSD_68808	3.21 eV	2.76 eV
Ba1Nd2S4_ICSD_615994	1.78 eV	1.45 eV
Ba1Nd2S5Zn1_ICSD_93714	1.13 eV	1.32 eV
Ba1Ni1O2_ICSD_15760	1.98 eV	1.50 eV
Ba1Ni1O3_ICSD_15761	1.46 eV	1.42 eV
Ba1Ni1O3_ICSD_175	1.45 eV	1.44 eV
Ba1Ni1O5Tb2_ICSD_66078	0.45 eV	1.72 eV
Ba1Ni1O5Y2_ICSD_71327	1.95 eV	1.95 eV
Ba1Ni2O8P2_ICSD_280167	3.29 eV	2.73 eV
Ba1Ni2O8V2_ICSD_201621	2.36 eV	1.91 eV
Ba1Ni4O8_ICSD_20898	0.20 eV	0.90 eV
Ba1O13Ti6_ICSD_2922	2.92 eV	2.50 eV
Ba1O14P4Ti2_ICSD_69103	2.86 eV	3.42 eV
Ba1O14P4V2_ICSD_66538	1.51 eV	2.29 eV
Ba1O14Sr4U3_ICSD_50812	3.05 eV	3.03 eV
Ba1O1S1Zn1_ICSD_171239	2.42 eV	2.04 eV
Ba1O1_ICSD_15301	2.75 eV	2.30 eV
Ba1O1_ICSD_173921	2.36 eV	2.55 eV
Ba1O1_ICSD_58663	2.09 eV	2.24 eV
Ba1O2.8V1_ICSD_78164	0.55 eV	1.50 eV
Ba1O2Zn1_ICSD_25812	2.78 eV	2.48 eV
Ba1O2_ICSD_180398	2.22 eV	2.69 eV
Ba1O2_ICSD_80750	2.23 eV	2.71 eV
Ba1O3Pb1_ICSD_94312	0.21 eV	0.90 eV
Ba1O3Pb1_ICSD_94313	0.19 eV	0.99 eV
Ba1O3Pr1_ICSD_163752	1.64 eV	1.84 eV
Ba1O3Pr1_ICSD_163753	0.32 eV	1.66 eV
Ba1O3Se1_ICSD_54156	3.88 eV	3.52 eV
Ba1O3Si1_ICSD_156705	3.04 eV	3.47 eV
Ba1O3Si1_ICSD_156706	2.13 eV	3.38 eV
Ba1O3Si1_ICSD_6245	4.53 eV	3.91 eV
Ba1O3Tc1_ICSD_109077	0.31 eV	0.80 eV
Ba1O3Te1_ICSD_10107	3.17 eV	3.10 eV
Ba1O3Te1_ICSD_4320	2.76 eV	2.56 eV
Ba1O3Th1_ICSD_29110	2.92 eV	3.04 eV
Ba1O3Ti1_ICSD_100802	2.13 eV	2.10 eV
Ba1O3Ti1_ICSD_154346	0.36 eV	1.92 eV
Ba1O3Ti1_ICSD_186461	2.14 eV	2.12 eV
Ba1O3Ti1_ICSD_31155	2.14 eV	2.08 eV
Ba1O3Ti1_ICSD_34619	2.18 eV	2.20 eV

Supplementary Table 665. Five-fold cross validated predictions for the band gap energy (59/277).

system	calculated	predicted
Ba1O3Ti1_ICSD_55221	2.19 eV	2.22 eV
Ba1O3Ti1_ICSD_73628	2.14 eV	2.14 eV
Ba1O3Ti1_ICSD_73640	2.14 eV	2.13 eV
Ba1O3Zr1_ICSD_90049	3.04 eV	2.81 eV
Ba1O4P1Rb1_ICSD_72001	5.02 eV	4.67 eV
Ba1O4S1_ICSD_186427	5.91 eV	5.76 eV
Ba1O4S1_ICSD_23743	4.51 eV	4.64 eV
Ba1O4S1_ICSD_33732	5.95 eV	5.73 eV
Ba1O4Se1_ICSD_409810	3.69 eV	3.62 eV
Ba1O4Si1Zn1_ICSD_73777	4.00 eV	3.83 eV
Ba1O4Tb2_ICSD_78661	3.20 eV	3.27 eV
Ba1O4U1_ICSD_36239	3.01 eV	2.94 eV
Ba1O4W1_ICSD_155512	4.69 eV	3.34 eV
Ba1O4W1_ICSD_155516	2.45 eV	3.55 eV
Ba1O4Y2_ICSD_89640	3.24 eV	3.43 eV
Ba1O5Pd1Pr2_ICSD_202172	2.52 eV	2.23 eV
Ba1O5Pd1Tb2_ICSD_202173	2.41 eV	2.32 eV
Ba1O5Pd1Y2_ICSD_202819	2.37 eV	2.26 eV
Ba1O5Se2_ICSD_54157	3.38 eV	3.09 eV
Ba1O5Si2_ICSD_10162	4.40 eV	4.48 eV
Ba1O5Tb2Zn1_ICSD_69721	3.43 eV	3.15 eV
Ba1O5Ti2_ICSD_157775	2.17 eV	2.09 eV
Ba1O5Ti2_ICSD_162369	3.19 eV	2.71 eV
Ba1O5Ti2_ICSD_2356	0.63 eV	1.67 eV
Ba1O5Ti2_ICSD_281548	2.21 eV	2.24 eV
Ba1O6P1V1_ICSD_66699	2.47 eV	2.72 eV
Ba1O6P2_ICSD_15714	5.27 eV	5.15 eV
Ba1O6Sb2_ICSD_74541	2.89 eV	2.34 eV
Ba1O6Te2_ICSD_8017	1.74 eV	2.27 eV
Ba1O6Ti1U1_ICSD_156646	2.74 eV	2.73 eV
Ba1O6V2_ICSD_80938	2.76 eV	2.21 eV
Ba1O7P2Zn1_ICSD_39396	4.75 eV	4.70 eV
Ba1O7Si2V1_ICSD_78029	2.17 eV	2.39 eV
Ba1O7Te2Zn1_ICSD_262409	1.91 eV	2.39 eV
Ba1O7U2_ICSD_22206	2.91 eV	2.56 eV
Ba1O8P2Sn1_ICSD_420129	3.18 eV	3.49 eV
Ba1O8P2Th1_ICSD_421965	5.21 eV	4.59 eV
Ba1O8P2Zn2_ICSD_300159	4.50 eV	4.46 eV
Ba1O8P2Zr1_ICSD_153124	4.15 eV	4.23 eV
Ba1O8P2Zr1_ICSD_173842	3.83 eV	4.08 eV
Ba1O8Si2U1_ICSD_79817	3.55 eV	3.02 eV
Ba1O8V3_ICSD_80299	1.58 eV	2.03 eV
Ba1O8V3_ICSD_82063	1.59 eV	2.02 eV
Ba1O9Si3Sn1_ICSD_10385	3.36 eV	3.37 eV
Ba1O9Si3Ti1_ICSD_290229	3.55 eV	3.85 eV
Ba1O9Si4_ICSD_80067	4.35 eV	4.31 eV
Ba1O9Te1W2_ICSD_281502	3.25 eV	2.92 eV
Ba1O9Ti4_ICSD_49575	3.20 eV	2.50 eV
Ba1O9V4_ICSD_173002	0.96 eV	1.41 eV
Ba1P10_ICSD_35295	1.29 eV	1.09 eV

Supplementary Table 666. Five-fold cross validated predictions for the band gap energy (60/277).

system	calculated	predicted
Ba1P3Pt2_ICSD_62520	0.26 eV	0.47 eV
Ba1P3_ICSD_23618	0.56 eV	0.53 eV
Ba1P4Te2_ICSD_412643	1.17 eV	0.93 eV
Ba1P8_ICSD_96544	0.89 eV	0.96 eV
Ba1Pd1S2_ICSD_63588	0.88 eV	1.08 eV
Ba1Pd2S4_ICSD_79930	1.08 eV	1.08 eV
Ba1Pr2S5Zn1_ICSD_93713	1.37 eV	1.29 eV
Ba1Pt2S3_ICSD_201146	1.28 eV	1.39 eV
Ba1Pt2S3_ICSD_55077	1.61 eV	1.39 eV
Ba1Rb1S4Ta1_ICSD_421845	2.59 eV	2.10 eV
Ba1S1_ICSD_52690	2.15 eV	1.66 eV
Ba1S2Sn1_ICSD_2587	1.68 eV	1.38 eV
Ba1S2_ICSD_2004	1.58 eV	1.48 eV
Ba1S3Sn2_ICSD_26333	1.26 eV	1.41 eV
Ba1S3Te1_ICSD_8	1.91 eV	1.67 eV
Ba1S3U1_ICSD_23289	1.93 eV	1.30 eV
Ba1S3V1_ICSD_154183	0.30 eV	0.63 eV
Ba1S3V1_ICSD_154184	0.34 eV	0.60 eV
Ba1S3V1_ICSD_616099	0.24 eV	0.52 eV
Ba1S3Zr1_ICSD_165977	1.02 eV	0.97 eV
Ba1S3_ICSD_23637	1.37 eV	1.23 eV
Ba1S3_ICSD_26765	1.02 eV	1.54 eV
Ba1S4Sb2_ICSD_38372	1.45 eV	1.55 eV
Ba1S4Tb2_ICSD_616078	1.75 eV	1.64 eV
Ba1S4Y2_ICSD_616101	1.76 eV	1.53 eV
Ba1Sb1Te3_ICSD_10506	0.39 eV	0.56 eV
Ba1Sc2Te4_ICSD_416326	0.67 eV	0.77 eV
Ba1Se1_ICSD_43655	1.95 eV	1.63 eV
Ba1Se1_ICSD_52695	1.21 eV	1.50 eV
Ba1Se2_ICSD_16358	0.62 eV	1.05 eV
Ba1Se3_ICSD_16359	0.93 eV	0.99 eV
Ba1Se4Y2_ICSD_422980	1.39 eV	1.28 eV
Ba1Se4Yb2_ICSD_616135	0.53 eV	1.07 eV
Ba1Si2_ICSD_168407	0.77 eV	0.64 eV
Ba1Tb2Te4_ICSD_90333	0.78 eV	0.78 eV
Ba1Te1_ICSD_43656	1.59 eV	1.23 eV
Ba1Te1_ICSD_616164	0.78 eV	1.28 eV
Ba1Te2_ICSD_80280	0.34 eV	0.67 eV
Ba1Te3_ICSD_36366	0.74 eV	0.50 eV
Ba1Te4Y2_ICSD_90335	0.80 eV	0.78 eV
Ba2Bi1Dy1O6_ICSD_68612	1.87 eV	2.12 eV
Ba2Bi1Fe1S5_ICSD_261419	1.22 eV	1.44 eV
Ba2Bi1Ir1O6_ICSD_174290	0.44 eV	0.70 eV
Ba2Bi1O6Sb1_ICSD_172761	1.78 eV	1.80 eV
Ba2Bi1O6Sb1_ICSD_172762	1.64 eV	1.44 eV
Ba2Bi1O6Ta1_ICSD_153120	2.69 eV	3.00 eV
Ba2Bi1O6Ta1_ICSD_154150	2.78 eV	2.74 eV
Ba2Bi1O6Y1_ICSD_65555	1.93 eV	2.54 eV
Ba2Bi1O6Yb1_ICSD_80902	0.30 eV	0.79 eV
Ba2Bi2O6_ICSD_28164	0.10 eV	0.84 eV

Supplementary Table 667. Five-fold cross validated predictions for the band gap energy (61/277).

system	calculated	predicted
Ba2Bi2Zn1_ICSD_421424	0.18 eV	0.29 eV
Ba2Br1Cu1O2_ICSD_67395	2.62 eV	2.38 eV
Ba2Br1H3_ICSD_415127	2.13 eV	2.38 eV
Ba2Br1In1O3_ICSD_81878	1.99 eV	2.10 eV
Ba2Br1N1_ICSD_262056	1.14 eV	1.35 eV
Ba2Br2F10Pb4_ICSD_411087	3.28 eV	3.06 eV
Ba2Br2O1_ICSD_423479	3.31 eV	3.89 eV
Ba2Br5Cs1_ICSD_402191	4.44 eV	4.02 eV
Ba2C2Co1F2O6_ICSD_95721	3.92 eV	3.79 eV
Ba2C2F3O6Y1_ICSD_72733	5.27 eV	4.94 eV
Ba2C3Ce1F1O9_ICSD_72446	4.62 eV	4.29 eV
Ba2C3Cs2O9_ICSD_73170	4.35 eV	4.12 eV
Ba2C3F1La1O9_ICSD_250059	4.60 eV	4.66 eV
Ba2C4H12N2O10S2_ICSD_59807	3.60 eV	4.04 eV
Ba2Ca1Cr2Cu1F14_ICSD_419545	2.29 eV	2.73 eV
Ba2Ca1Cu1F14Fe2_ICSD_202761	2.97 eV	2.41 eV
Ba2Ca1Ir1O6_ICSD_74029	0.42 eV	0.59 eV
Ba2Ca1Mo1O6_ICSD_45317	2.12 eV	2.29 eV
Ba2Ca1N4W1_ICSD_409472	1.84 eV	1.75 eV
Ba2Ca1O6Pd3_ICSD_73082	2.07 eV	1.48 eV
Ba2Ca1O6Te1_ICSD_246112	3.00 eV	2.75 eV
Ba2Ca1O6W1_ICSD_246111	2.86 eV	3.03 eV
Ba2Ca1O6W1_ICSD_246117	2.95 eV	2.95 eV
Ba2Cd1O6U1_ICSD_167503	2.88 eV	2.51 eV
Ba2Cd1S3_ICSD_66654	2.14 eV	1.80 eV
Ba2Cd1Se3_ICSD_66653	1.62 eV	1.46 eV
Ba2Cd1Te3_ICSD_88849	1.44 eV	1.26 eV
Ba2Cd2K1Sb3_ICSD_420620	0.25 eV	0.34 eV
Ba2Ce1Cl7_ICSD_000	4.74 eV	4.39 eV
Ba2Ce1O6Pt1_ICSD_66408	2.19 eV	2.12 eV
Ba2Cl1Co2F7_ICSD_79479	3.94 eV	3.70 eV
Ba2Cl1Co4O7_ICSD_245991	1.09 eV	1.24 eV
Ba2Cl1Cu1O2_ICSD_1038	2.65 eV	2.19 eV
Ba2Cl1F3_ICSD_183926	5.73 eV	5.34 eV
Ba2Cl1F7Mn1Ni1_ICSD_79480	2.20 eV	3.01 eV
Ba2Cl1F7Ni2_ICSD_84782	3.61 eV	3.35 eV
Ba2Cl1F7Zn2_ICSD_79478	4.76 eV	4.93 eV
Ba2Cl1H3_ICSD_416893	2.09 eV	2.75 eV
Ba2Cl1In1O3_ICSD_81877	2.08 eV	2.19 eV
Ba2Cl1Mn1O7Si2_ICSD_281430	1.33 eV	2.58 eV
Ba2Cl1N1_ICSD_262135	1.11 eV	1.71 eV
Ba2Cl1P1_ICSD_28134	1.25 eV	1.18 eV
Ba2Cl1P7_ICSD_24398	1.57 eV	1.33 eV
Ba2Cl2Co1O6Se2_ICSD_280965	3.81 eV	2.81 eV
Ba2Cl7Er1_ICSD_82494	4.95 eV	4.60 eV
Ba2Cl7Gd1_ICSD_82495	4.59 eV	4.66 eV
Ba2Cl7La1_ICSD_000	4.81 eV	4.39 eV
Ba2Cl7Sc1_ICSD_408056	4.11 eV	4.18 eV
Ba2Cl7Sc1_ICSD_56832	4.16 eV	4.15 eV
Ba2Cl7Y1_ICSD_000	4.71 eV	4.82 eV

Supplementary Table 668. Five-fold cross validated predictions for the band gap energy (62/277).

system	calculated	predicted
Ba2Co1F6_ICSD_21057	3.77 eV	3.38 eV
Ba2Co1Ge2O7_ICSD_290483	2.70 eV	2.13 eV
Ba2Co1O4_ICSD_16234	1.61 eV	1.72 eV
Ba2Co1O4_ICSD_92321	1.84 eV	1.62 eV
Ba2Co1O6Re1_ICSD_109254	0.29 eV	1.28 eV
Ba2Co1O7Si2_ICSD_281293	3.79 eV	3.24 eV
Ba2Co1O7Si2_ICSD_81472	3.70 eV	3.07 eV
Ba2Cr1O4_ICSD_73892	1.75 eV	1.62 eV
Ba2Cr1O6Ta1_ICSD_74410	2.23 eV	2.24 eV
Ba2Cr7O14_ICSD_2766	0.19 eV	1.33 eV
Ba2Cs1Nb3O10_ICSD_93676	1.64 eV	2.05 eV
Ba2Cu1F6_ICSD_100028	2.69 eV	2.39 eV
Ba2Cu1Ge2O7_ICSD_77132	1.53 eV	1.45 eV
Ba2Cu1I1O2_ICSD_67394	2.58 eV	2.02 eV
Ba2Cu1O6Te1_ICSD_88703	0.27 eV	0.93 eV
Ba2Cu1O6U1_ICSD_157853	1.38 eV	1.34 eV
Ba2Cu1O6U1_ICSD_169534	1.37 eV	1.37 eV
Ba2Cu1O6W1_ICSD_33569	0.91 eV	1.07 eV
Ba2Cu1O6W1_ICSD_72813	0.91 eV	1.11 eV
Ba2Cu1O7Si2_ICSD_20905	1.43 eV	1.53 eV
Ba2Cu1O7Si2_ICSD_97762	1.43 eV	1.64 eV
Ba2Cu1O8P2_ICSD_81457	1.47 eV	1.71 eV
Ba2Cu2O12Si4_ICSD_71535	1.56 eV	1.49 eV
Ba2Cu2S5U1_ICSD_418464	0.92 eV	1.09 eV
Ba2Cu3Li1O6_ICSD_401239	0.37 eV	0.95 eV
Ba2Cu3Na1O6_ICSD_72328	0.37 eV	0.87 eV
Ba2Cu3S6V1_ICSD_83969	0.92 eV	0.97 eV
Ba2Cu5F14_ICSD_202138	1.93 eV	2.09 eV
Ba2Dy1Ga1Se5_ICSD_262886	1.68 eV	1.57 eV
Ba2Dy1Nb1O6_ICSD_109156	3.09 eV	2.91 eV
Ba2Dy1O6Sb1_ICSD_150863	3.46 eV	3.29 eV
Ba2Er1Ga1Se5_ICSD_262887	1.69 eV	1.66 eV
Ba2Er1Nb1O6_ICSD_245456	3.10 eV	3.12 eV
Ba2Er1O6Ru1_ICSD_59743	1.09 eV	1.13 eV
Ba2Er1O6Sb1_ICSD_245459	3.37 eV	3.35 eV
Ba2F10I2Pb4_ICSD_411088	2.84 eV	3.09 eV
Ba2F10Mg3_ICSD_50228	6.89 eV	6.74 eV
Ba2F10Ni3_ICSD_23364	4.21 eV	3.68 eV
Ba2F18Ni7_ICSD_65610	4.21 eV	3.40 eV
Ba2F18Zn7_ICSD_40925	5.40 eV	5.20 eV
Ba2F1In1O3_ICSD_79261	2.21 eV	2.20 eV
Ba2F1N1_ICSD_262049	1.09 eV	1.24 eV
Ba2F2Fe2O1S2_ICSD_249689	1.58 eV	1.47 eV
Ba2F2Fe2O1Se2_ICSD_249687	1.57 eV	1.48 eV
Ba2F2Mn2O1Se2_ICSD_183149	2.05 eV	1.58 eV
Ba2F2S3Sn1_ICSD_171343	2.05 eV	1.93 eV
Ba2F2Se3Sn1_ICSD_171344	1.55 eV	1.43 eV
Ba2F4O3W1_ICSD_33271	3.70 eV	3.45 eV
Ba2F6Ni1_ICSD_21056	3.87 eV	3.36 eV
Ba2F6O1Ti1_ICSD_72150	3.85 eV	4.00 eV

Supplementary Table 669. Five-fold cross validated predictions for the band gap energy (63/277).

system	calculated	predicted
Ba2F6Pd1_ICSD_88802	2.84 eV	2.89 eV
Ba2F6Zn1_ICSD_21054	5.87 eV	5.32 eV
Ba2F8Zr1_ICSD_85720	5.94 eV	5.90 eV
Ba2Fe1Ge2O7_ICSD_22358	1.27 eV	2.18 eV
Ba2Fe1Mo1O6_ICSD_246543	0.41 eV	1.09 eV
Ba2Fe1O6U1_ICSD_27018	1.92 eV	1.76 eV
Ba2Fe1O6W1_ICSD_95518	1.61 eV	1.91 eV
Ba2Fe1O6W1_ICSD_99061	2.54 eV	1.94 eV
Ba2Fe1S3_ICSD_615850	1.74 eV	1.09 eV
Ba2Fe1S5Sb1_ICSD_261418	1.14 eV	1.39 eV
Ba2Fe1Se3_ICSD_615861	1.60 eV	1.29 eV
Ba2Ga1H1O14P4_ICSD_280043	5.10 eV	4.64 eV
Ba2Ga2S5_ICSD_38256	2.80 eV	2.67 eV
Ba2Gd1Nb1O6_ICSD_109155	3.02 eV	2.86 eV
Ba2Gd1Nb1O6_ICSD_172405	3.15 eV	2.97 eV
Ba2Ge1P2_ICSD_35150	0.59 eV	0.73 eV
Ba2Ge1S4_ICSD_615898	2.24 eV	2.37 eV
Ba2Ge1Se2Te2_ICSD_414165	0.81 eV	1.03 eV
Ba2Ge1Se4_ICSD_414166	1.38 eV	1.45 eV
Ba2Ge2Mg1O7_ICSD_419312	3.56 eV	3.40 eV
Ba2Ge2Mn1O7_ICSD_184630	2.15 eV	2.18 eV
Ba2Ge2O1S6Zn1_ICSD_14174	2.08 eV	2.30 eV
Ba2Ge2O7Zn1_ICSD_420550	3.37 eV	3.19 eV
Ba2Ge2O8Ti1_ICSD_281271	3.66 eV	3.62 eV
Ba2Ge2O8Ti1_ICSD_39133	3.66 eV	3.61 eV
Ba2Ge2Se5_ICSD_410791	1.27 eV	1.36 eV
Ba2Ge2Te5_ICSD_59001	0.61 eV	0.79 eV
Ba2Ge4S10_ICSD_66868	2.16 eV	1.98 eV
Ba2H1N1_ICSD_67510	1.09 eV	1.20 eV
Ba2H3I1_ICSD_423520	2.19 eV	2.46 eV
Ba2H4Pd1_ICSD_165182	1.59 eV	1.96 eV
Ba2H7O7Tl1_ICSD_411058	2.50 eV	2.86 eV
Ba2Hf1S4_ICSD_80652	0.87 eV	1.38 eV
Ba2Hg1S3_ICSD_32647	1.35 eV	1.59 eV
Ba2Ho1Nb1O6_ICSD_109157	3.09 eV	2.98 eV
Ba2Ho1O6Ru1_ICSD_99638	1.10 eV	1.10 eV
Ba2Ho1O6Sb1_ICSD_150862	3.44 eV	3.39 eV
Ba2Ho1O6Ta1_ICSD_158358	3.56 eV	3.47 eV
Ba2Ho1O6Ta1_ICSD_158359	3.50 eV	3.43 eV
Ba2I1Na1O6_ICSD_425446	2.21 eV	3.07 eV
Ba2I2O1_ICSD_391434	3.03 eV	2.86 eV
Ba2In1Nb1O6_ICSD_172166	3.65 eV	3.10 eV
Ba2In1O6Ru1_ICSD_15260	1.31 eV	1.04 eV
Ba2In1O6Ta1_ICSD_261481	4.10 eV	3.57 eV
Ba2In1Sb1Se5_ICSD_425179	0.80 eV	0.97 eV
Ba2In1Se5Y1_ICSD_262888	1.19 eV	1.21 eV
Ba2In2O5_ICSD_89438	1.06 eV	1.68 eV
Ba2In2Se5_ICSD_67187	1.44 eV	1.20 eV
Ba2Ir1La1O6_ICSD_152678	0.35 eV	0.31 eV
Ba2Ir1La1O6_ICSD_174140	0.19 eV	0.47 eV

Supplementary Table 670. Five-fold cross validated predictions for the band gap energy (64/277).

system	calculated	predicted
Ba2Ir1O6Pr1_ICSD_150323	0.19 eV	0.27 eV
Ba2Ir1O6Pr1_ICSD_155828	0.20 eV	0.14 eV
Ba2Ir1O6Sr1_ICSD_74030	0.52 eV	0.48 eV
Ba2K8O24U6_ICSD_91785	2.87 eV	2.90 eV
Ba2La1O6Ru1_ICSD_100793	1.27 eV	1.19 eV
Ba2La1O6Ru1_ICSD_155549	1.30 eV	1.01 eV
Ba2La1O6Ru1_ICSD_37028	0.79 eV	1.06 eV
Ba2La1O6Sb1_ICSD_153136	3.74 eV	3.26 eV
Ba2La1O6Ta1_ICSD_160168	2.86 eV	3.52 eV
Ba2La1O6Ta1_ICSD_160169	2.86 eV	3.32 eV
Ba2La1O6Ta1_ICSD_160170	3.68 eV	3.66 eV
Ba2Li1N4Re1_ICSD_411453	1.82 eV	1.67 eV
Ba2Li1O6Re1_ICSD_109259	1.91 eV	1.92 eV
Ba2Li3N4Nb1_ICSD_75516	2.41 eV	2.13 eV
Ba2Li3N4Ta1_ICSD_75031	2.39 eV	2.18 eV
Ba2Lu1Nb1O6_ICSD_109161	0.98 eV	2.76 eV
Ba2Lu1O6Ru1_ICSD_202869	1.05 eV	0.75 eV
Ba2Mg1O6U1_ICSD_23099	3.07 eV	2.92 eV
Ba2Mg1O6W1_ICSD_183771	2.91 eV	2.97 eV
Ba2Mg1O7Si2_ICSD_183983	4.44 eV	4.39 eV
Ba2Mg1O7Si2_ICSD_81117	4.46 eV	4.47 eV
Ba2Mn1O3_ICSD_15508	1.65 eV	1.32 eV
Ba2Mn1S3_ICSD_26231	1.86 eV	1.71 eV
Ba2Mn1Se3_ICSD_26230	1.66 eV	1.54 eV
Ba2Mn1Te3_ICSD_1151	1.29 eV	0.95 eV
Ba2Mo1Nd1O6_ICSD_172390	0.12 eV	2.00 eV
Ba2Mo1Ni1O6_ICSD_98192	2.20 eV	1.86 eV
Ba2N2Zn1_ICSD_80377	0.61 eV	0.77 eV
Ba2N3Nb1_ICSD_74906	1.99 eV	1.68 eV
Ba2N3Ta1_ICSD_74503	2.03 eV	1.74 eV
Ba2N3V1_ICSD_80177	1.46 eV	1.37 eV
Ba2N8Si5_ICSD_401501	2.88 eV	2.93 eV
Ba2Na1Nb5O15_ICSD_53267	2.49 eV	2.52 eV
Ba2Na1Ni3O6_ICSD_59588	1.06 eV	1.48 eV
Ba2Na1O6Re1_ICSD_200876	1.97 eV	1.72 eV
Ba2Nb1O6Tb1_ICSD_245455	3.15 eV	2.94 eV
Ba2Nb1O6Y1_ICSD_172407	3.08 eV	3.15 eV
Ba2Nb2O10Te1_ICSD_405153	3.02 eV	3.03 eV
Ba2Nb6O21Te2_ICSD_405107	2.41 eV	2.71 eV
Ba2Nd1O6Ru1_ICSD_155551	0.33 eV	0.92 eV
Ba2Ni1O6Te1_ICSD_25005	2.27 eV	2.17 eV
Ba2Ni1O6U1_ICSD_169535	2.61 eV	2.11 eV
Ba2Ni1O6W1_ICSD_24984	3.06 eV	2.66 eV
Ba2O10Si4_ICSD_100314	4.74 eV	4.35 eV
Ba2O11Te2V2_ICSD_261183	3.27 eV	2.85 eV
Ba2O13Ti6_ICSD_300030	1.01 eV	1.58 eV
Ba2O21Ta6Te2_ICSD_405108	3.03 eV	2.88 eV
Ba2O3Pd1_ICSD_202812	0.61 eV	1.31 eV
Ba2O3Zn1_ICSD_36659	3.10 eV	2.53 eV
Ba2O4Pb1_ICSD_66544	1.23 eV	1.55 eV

Supplementary Table 671. Five-fold cross validated predictions for the band gap energy (65/277).

system	calculated	predicted
Ba2O4Sn1_ICSD_81849	2.08 eV	2.00 eV
Ba2O4Ti1_ICSD_2625	3.62 eV	2.93 eV
Ba2O4V1_ICSD_72625	1.24 eV	2.42 eV
Ba2O4Zr1_ICSD_39707	2.98 eV	3.00 eV
Ba2O5Re1_ICSD_202393	0.44 eV	1.31 eV
Ba2O5Tl2_ICSD_6322	0.59 eV	1.28 eV
Ba2O5W1_ICSD_62489	2.87 eV	3.13 eV
Ba2O6Pb1U1_ICSD_167504	2.15 eV	2.17 eV
Ba2O6Pr1Sb1_ICSD_153137	3.11 eV	2.82 eV
Ba2O6Ru1Y1_ICSD_202864	1.11 eV	0.96 eV
Ba2O6Sb1Tb1_ICSD_38332	3.51 eV	3.11 eV
Ba2O6Sb1Y1_ICSD_84650	3.54 eV	3.26 eV
Ba2O6Sr1Te1_ICSD_246109	3.12 eV	2.98 eV
Ba2O6Sr1W1_ICSD_246114	3.06 eV	3.19 eV
Ba2O6Ta1Y1_ICSD_171176	3.54 eV	3.32 eV
Ba2O6Ta1Y1_ICSD_261453	3.48 eV	3.54 eV
Ba2O6U1Zn1_ICSD_167502	2.89 eV	2.75 eV
Ba2O6W1Zn1_ICSD_423034	2.99 eV	2.87 eV
Ba2O7P2_ICSD_261125	5.14 eV	4.82 eV
Ba2O7Si2Zn1_ICSD_409588	4.27 eV	4.12 eV
Ba2O7U2_ICSD_63076	3.15 eV	2.82 eV
Ba2O8Si2Ti1_ICSD_201844	4.33 eV	3.72 eV
Ba2O8Si2V1_ICSD_51479	2.31 eV	2.76 eV
Ba2O9P2Te1_ICSD_416032	3.42 eV	3.59 eV
Ba2O9V3_ICSD_404713	1.29 eV	1.78 eV
Ba2Re6S11_ICSD_201072	1.31 eV	1.15 eV
Ba2S3Zn1_ICSD_653999	2.08 eV	2.01 eV
Ba2S3_ICSD_70058	1.59 eV	1.66 eV
Ba2S4Si1_ICSD_42661	3.00 eV	2.70 eV
Ba2S4Sn1_ICSD_16273	2.05 eV	1.92 eV
Ba2S4Sn1_ICSD_42036	1.68 eV	1.90 eV
Ba2S4Ti1_ICSD_616084	2.11 eV	1.64 eV
Ba2S4Zr1_ICSD_80651	0.61 eV	1.23 eV
Ba2Sb2Zn1_ICSD_421425	0.38 eV	0.29 eV
Ba2Sb3_ICSD_61089	0.26 eV	0.25 eV
Ba2Se4Si1_ICSD_49750	2.27 eV	1.67 eV
Ba2Se5Sn1_ICSD_248033	1.17 eV	1.12 eV
Ba2Si1Te4_ICSD_49751	1.10 eV	0.92 eV
Ba3Be1Cl18Zr6_ICSD_33993	1.01 eV	1.74 eV
Ba3Bi1Cl3O3_ICSD_69618	3.98 eV	3.72 eV
Ba3Bi1Ir2O9_ICSD_174292	0.43 eV	0.61 eV
Ba3Bi1N1_ICSD_152055	0.67 eV	0.63 eV
Ba3Bi1Na1O6_ICSD_72839	1.77 eV	2.03 eV
Ba3Bi1O9Ru2_ICSD_72448	0.88 eV	0.87 eV
Ba3Bi2O16P4_ICSD_61061	3.82 eV	3.43 eV
Ba3Bi2O9Te1_ICSD_90842	2.13 eV	1.68 eV
Ba3Br2Fe2O5_ICSD_48179	2.20 eV	1.77 eV
Ba3C1Cl4O3_ICSD_174065	4.76 eV	4.21 eV
Ba3C1F7O3Sc1_ICSD_75255	5.21 eV	5.35 eV
Ba3C5F2La2O15_ICSD_72445	4.43 eV	4.35 eV

Supplementary Table 672. Five-fold cross validated predictions for the band gap energy (66/277).

system	calculated	predicted
Ba3Ca1O9Ru2_ICSD_152482	0.86 eV	0.99 eV
Ba3Ca1O9Ru2_ICSD_71209	0.98 eV	0.94 eV
Ba3Ca1O9Ru2_ICSD_73183	0.99 eV	0.97 eV
Ba3Ca1O9Sb2_ICSD_249664	3.17 eV	2.41 eV
Ba3Ca2N6Si2_ICSD_187336	1.57 eV	1.69 eV
Ba3Cl2Cu2O4_ICSD_115	1.11 eV	1.41 eV
Ba3Cl2Fe2O5_ICSD_48178	2.22 eV	1.72 eV
Ba3Cl2O5W1_ICSD_63518	3.60 eV	2.96 eV
Ba3Co1Nb2O9_ICSD_150431	2.58 eV	1.67 eV
Ba3Co1O9Ru2_ICSD_69092	0.79 eV	0.78 eV
Ba3Co1O9Sb2_ICSD_151442	2.33 eV	1.70 eV
Ba3Cr1O5_ICSD_73893	1.47 eV	1.71 eV
Ba3Cr2Mo1O9_ICSD_81071	1.12 eV	1.25 eV
Ba3Cr2O9W1_ICSD_81072	1.75 eV	1.65 eV
Ba3Cr2S6_ICSD_97540	0.92 eV	0.91 eV
Ba3Cu2Er2O10Pt1_ICSD_69569	1.30 eV	1.34 eV
Ba3Cu2Ho2O10Pt1_ICSD_62391	1.31 eV	1.30 eV
Ba3Cu2O10Pt1Y2_ICSD_62390	1.33 eV	1.35 eV
Ba3Dy1O9Ru2_ICSD_401914	0.60 eV	0.60 eV
Ba3Dy4O9_ICSD_72480	3.24 eV	3.29 eV
Ba3Er1O9Ru2_ICSD_401915	0.57 eV	0.66 eV
Ba3Er4O9_ICSD_72481	3.28 eV	3.49 eV
Ba3F12In2_ICSD_48182	4.63 eV	4.53 eV
Ba3Fe1S5_ICSD_280	0.29 eV	1.29 eV
Ba3Fe3Nb1O14Si2_ICSD_162894	2.32 eV	2.26 eV
Ba3Fe3Se7_ICSD_16310	0.40 eV	0.82 eV
Ba3Ga2Ge4O14_ICSD_250124	2.23 eV	2.80 eV
Ba3Ga2N4_ICSD_82736	1.52 eV	1.38 eV
Ba3Ga2S6_ICSD_201421	2.68 eV	2.60 eV
Ba3Ga3Nb1O14Si2_ICSD_154215	3.55 eV	3.37 eV
Ba3Ge1O1_ICSD_50512	0.31 eV	0.57 eV
Ba3Ho4O9_ICSD_33807	3.26 eV	3.38 eV
Ba3In1O9Ru2_ICSD_15261	0.73 eV	0.90 eV
Ba3In2O11Zn5_ICSD_73192	2.29 eV	1.96 eV
Ba3In2P4_ICSD_402812	1.13 eV	0.84 eV
Ba3Ir2Na1O9_ICSD_413452	0.21 eV	0.54 eV
Ba3Ir2O9Sr1_ICSD_245254	0.19 eV	0.37 eV
Ba3Ir2O9Y1_ICSD_16367	0.17 eV	0.31 eV
Ba3La1O9Ru2_ICSD_51926	0.66 eV	0.84 eV
Ba3Lu1O9Ru2_ICSD_51929	0.54 eV	0.67 eV
Ba3Lu4O9_ICSD_38383	3.48 eV	3.52 eV
Ba3Mg1O8Si2_ICSD_419862	4.70 eV	3.88 eV
Ba3Mg1O9Ru2_ICSD_33529	0.88 eV	0.99 eV
Ba3Mg1O9Sb2_ICSD_33527	2.65 eV	2.58 eV
Ba3Mn2O8_ICSD_280045	1.59 eV	1.62 eV
Ba3N1Sb1_ICSD_152054	0.71 eV	0.62 eV
Ba3N2O12Si6_ICSD_421322	4.62 eV	4.47 eV
Ba3N2O1Zn1_ICSD_55536	0.52 eV	1.08 eV
Ba3N4O9Si6_ICSD_415918	4.37 eV	4.02 eV
Ba3N8O6P6_ICSD_710077	4.54 eV	4.31 eV

Supplementary Table 673. Five-fold cross validated predictions for the band gap energy (67/277).

system	calculated	predicted
Ba3Na1Nb1O6_ICSD_72330	3.23 eV	3.29 eV
Ba3Na1O6Ru1_ICSD_405133	1.45 eV	1.45 eV
Ba3Na1O6Ta1_ICSD_72331	3.47 eV	3.49 eV
Ba3Nb2O18P4_ICSD_90878	3.46 eV	3.15 eV
Ba3Nb2O8_ICSD_95193	4.46 eV	3.25 eV
Ba3Nb2O9Sr1_ICSD_24390	3.48 eV	3.53 eV
Ba3Nb2O9Sr1_ICSD_37161	3.69 eV	3.44 eV
Ba3Nb2O9Zn1_ICSD_157044	2.95 eV	2.99 eV
Ba3Nb6O26Si4_ICSD_16029	2.57 eV	3.10 eV
Ba3Ni1O4_ICSD_30662	2.05 eV	1.75 eV
Ba3Ni1O9Ru2_ICSD_50831	1.00 eV	0.98 eV
Ba3Ni1O9Sb2_ICSD_1177	0.72 eV	1.56 eV
Ba3Ni1O9Ta2_ICSD_240281	3.50 eV	2.71 eV
Ba3O11Te4_ICSD_37069	3.23 eV	3.01 eV
Ba3O13P4_ICSD_280908	5.11 eV	4.74 eV
Ba3O1Sb2_ICSD_280592	0.33 eV	0.48 eV
Ba3O1Sb4_ICSD_415032	0.34 eV	0.70 eV
Ba3O20Si4Te1Zn6_ICSD_416231	3.24 eV	3.08 eV
Ba3O23Si4Ta6_ICSD_18316	0.17 eV	2.59 eV
Ba3O26Si4Ta6_ICSD_18317	3.26 eV	2.91 eV
Ba3O3S4V2_ICSD_279607	0.90 eV	1.28 eV
Ba3O4P2Se4_ICSD_414638	1.31 eV	1.88 eV
Ba3O5Si1_ICSD_1449	3.53 eV	3.24 eV
Ba3O6Sb2_ICSD_413764	2.93 eV	2.52 eV
Ba3O6W1_ICSD_76437	2.72 eV	3.03 eV
Ba3O8P2_ICSD_69450	5.15 eV	4.60 eV
Ba3O8V2_ICSD_78169	3.72 eV	3.16 eV
Ba3O9Ru2Sr1_ICSD_48102	1.23 eV	0.86 eV
Ba3O9Ru2Tb1_ICSD_95165	0.60 eV	0.58 eV
Ba3O9Ru2Y1_ICSD_51924	0.60 eV	0.69 eV
Ba3O9Ru2Zn1_ICSD_69090	0.86 eV	0.97 eV
Ba3O9Sb2Sr1_ICSD_249663	3.13 eV	2.74 eV
Ba3O9Sr1Ta2_ICSD_24391	3.92 eV	3.81 eV
Ba3O9Sr1Ta2_ICSD_27496	3.45 eV	3.68 eV
Ba3O9Sr1Ta2_ICSD_37162	4.15 eV	3.97 eV
Ba3O9Te2_ICSD_100797	2.36 eV	2.62 eV
Ba3O9Y4_ICSD_87118	3.30 eV	3.55 eV
Ba3P2S8_ICSD_417422	1.98 eV	2.07 eV
Ba3P4Sn2_ICSD_80132	0.81 eV	0.83 eV
Ba3P4_ICSD_38322	0.48 eV	0.65 eV
Ba3P6Si4_ICSD_29261	0.40 eV	0.94 eV
Ba3S5Si1_ICSD_26377	2.81 eV	2.57 eV
Ba3S5Ti1_ICSD_203087	1.38 eV	1.51 eV
Ba3S7Sn2_ICSD_166524	1.95 eV	1.84 eV
Ba3S7Zr2_ICSD_59271	0.82 eV	1.02 eV
Ba3S8Ta2_ICSD_202878	0.81 eV	1.17 eV
Ba4Br2Si3_ICSD_411640	0.33 eV	1.09 eV
Ba4Br6O1_ICSD_391435	4.16 eV	4.19 eV
Ba4C2Cu1Li1O10_ICSD_401122	0.87 eV	1.33 eV
Ba4C2Cu1Na1O10_ICSD_66776	1.00 eV	1.28 eV

Supplementary Table 674. Five-fold cross validated predictions for the band gap energy (68/277).

system	calculated	predicted
Ba4Cd3S10Tb2_ICSD_411167	1.92 eV	1.35 eV
Ba4Ce1Mn3O12_ICSD_99661	1.87 eV	1.57 eV
Ba4Cl12Co2_ICSD_280274	3.27 eV	3.09 eV
Ba4Cl2N4W1_ICSD_50003	2.22 eV	2.16 eV
Ba4Cl6O1_ICSD_16026	4.38 eV	4.39 eV
Ba4Er1O12Ru3_ICSD_174186	0.29 eV	0.41 eV
Ba4F24H1P3_ICSD_419143	6.02 eV	6.50 eV
Ba4Ga2S7_ICSD_201420	2.66 eV	2.44 eV
Ba4Ge1P4_ICSD_32560	1.01 eV	0.94 eV
Ba4H0.666Nb2O9.333_ICSD_165531	3.45 eV	3.16 eV
Ba4Hf3S10_ICSD_602359	0.19 eV	0.41 eV
Ba4Ho1O12Ru3_ICSD_160868	0.28 eV	0.42 eV
Ba4I6O1_ICSD_280584	3.98 eV	3.42 eV
Ba4In2S8_ICSD_261680	1.95 eV	1.66 eV
Ba4K1O1Sb3_ICSD_410747	1.02 eV	0.84 eV
Ba4Li1Nb3O12_ICSD_19009	2.79 eV	2.96 eV
Ba4Li1O12Ta3_ICSD_19010	3.21 eV	3.12 eV
Ba4Mg1N6Si2_ICSD_187335	1.14 eV	1.53 eV
Ba4Mn3O10_ICSD_51881	1.04 eV	1.35 eV
Ba4Mn3O12Pr1_ICSD_99662	1.52 eV	1.68 eV
Ba4Na1O12Sb3_ICSD_160173	1.52 eV	2.20 eV
Ba4O16Si6_ICSD_100310	4.52 eV	4.25 eV
Ba4O1Rb1Sb3_ICSD_415036	0.94 eV	0.84 eV
Ba4O1Sb2_ICSD_402284	0.64 eV	0.53 eV
Ba4O6Pt1_ICSD_65706	2.00 eV	2.20 eV
Ba4O9Ta2_ICSD_166900	4.09 eV	3.77 eV
Ba4P3_ICSD_73206	0.74 eV	0.84 eV
Ba4P4Si1_ICSD_32559	1.09 eV	0.91 eV
Ba4P4Ti1_ICSD_380114	1.14 eV	1.14 eV
Ba4S10Zr3_ICSD_72656	0.47 eV	0.84 eV
Ba4Sb4Se11_ICSD_31500	1.10 eV	0.94 eV
Ba5Br1O12P3_ICSD_409482	4.97 eV	4.59 eV
Ba5Br2O9Ru2_ICSD_245668	1.42 eV	1.23 eV
Ba5Br6O4Si1_ICSD_73365	4.26 eV	3.95 eV
Ba5Cd2O2Sb4_ICSD_423458	0.10 eV	0.49 eV
Ba5Cl1Mn3O12_ICSD_61356	1.73 eV	2.09 eV
Ba5Cl1O12P3_ICSD_8191	5.06 eV	4.72 eV
Ba5Cl1O12V3_ICSD_170769	3.95 eV	2.83 eV
Ba5Cl1O15Os3_ICSD_80447	1.20 eV	1.65 eV
Ba5Cl1O15Re3_ICSD_73928	2.64 eV	1.69 eV
Ba5Cl2O9Ta2_ICSD_240872	3.60 eV	3.27 eV
Ba5Co5O14_ICSD_153698	1.27 eV	1.27 eV
Ba5Cr1N5_ICSD_82360	0.17 eV	0.79 eV
Ba5Er8O21Zn4_ICSD_400436	3.54 eV	3.33 eV
Ba5Fe4S11_ICSD_16372	0.49 eV	0.83 eV
Ba5Gd8O21Zn4_ICSD_94223	3.25 eV	3.32 eV
Ba5Hf4S13_ICSD_71936	0.25 eV	0.45 eV
Ba5Ho8O21Zn4_ICSD_73186	3.51 eV	3.33 eV
Ba5Nb4O15_ICSD_157477	2.79 eV	2.80 eV
Ba5O10Ru2_ICSD_75386	0.77 eV	0.92 eV

Supplementary Table 675. Five-fold cross validated predictions for the band gap energy (69/277).

system	calculated	predicted
Ba5O15Ta4_ICSD_16028	3.10 eV	3.19 eV
Ba5P4_ICSD_413273	0.93 eV	0.69 eV
Ba5Sb4_ICSD_280022	0.25 eV	0.30 eV
Ba5Sb4_ICSD_52693	0.27 eV	0.30 eV
Ba6C60_ICSD_70062	0.17 eV	1.02 eV
Ba6Cl2Eu1F12_ICSD_411225	3.13 eV	4.22 eV
Ba6Cl2O12Pt1Ru2_ICSD_72280	1.50 eV	1.22 eV
Ba6Cl2O12Ru3_ICSD_99681	0.92 eV	1.13 eV
Ba6F26Mg7_ICSD_50217	6.81 eV	6.72 eV
Ba6Ga2P6_ICSD_402177	0.60 eV	0.83 eV
Ba6Hf5S16_ICSD_71935	0.30 eV	0.37 eV
Ba6Mn5O16_ICSD_97017	1.30 eV	1.07 eV
Ba6Na2Nb2O17P2_ICSD_249742	3.26 eV	3.32 eV
Ba6Nb8O30Ti2_ICSD_36081	2.17 eV	2.74 eV
Ba6O17Ti4Y2_ICSD_96630	3.03 eV	2.99 eV
Ba6O18W4_ICSD_9725	3.45 eV	3.14 eV
Ba6P6Sn2_ICSD_35342	0.77 eV	0.72 eV
Ba7Cl2F12_ICSD_87084	6.06 eV	5.16 eV
Ba7Cu1F34Fe6_ICSD_203173	2.51 eV	2.59 eV
Ba7Ir6O19_ICSD_74686	0.27 eV	0.29 eV
Ba8Si6Sn1_ICSD_169999	0.27 eV	0.54 eV
Ba9C1N10Nb2O1_ICSD_412664	1.80 eV	1.72 eV
Ba9N12Nb2_ICSD_402743	1.27 eV	1.77 eV
Ba9O24Sc2Si6_ICSD_75175	4.51 eV	3.92 eV
Be17Ca12O29_ICSD_14359	3.66 eV	4.33 eV
Be1Bi1La3S7_ICSD_616183	1.73 eV	1.53 eV
Be1Br2_ICSD_92584	5.19 eV	4.56 eV
Be1C1N2_ICSD_181041	3.85 eV	4.65 eV
Be1C1N2_ICSD_181042	5.48 eV	4.96 eV
Be1C4Cl2H16O4S2_ICSD_249588	4.47 eV	4.54 eV
Be1C4K2O8_ICSD_1446	3.39 eV	4.09 eV
Be1Ca1F1Na1O6Si2_ICSD_15314	5.29 eV	5.19 eV
Be1Ca2O7Si2_ICSD_31234	4.99 eV	4.82 eV
Be1Cl2_ICSD_173559	6.65 eV	6.35 eV
Be1Cl2_ICSD_173560	6.35 eV	6.40 eV
Be1Cl2_ICSD_173561	6.59 eV	6.52 eV
Be1Cl2_ICSD_31696	6.28 eV	5.68 eV
Be1Cl2_ICSD_78774	6.52 eV	6.46 eV
Be1Cl4Cs2_ICSD_49622	5.54 eV	5.23 eV
Be1Cr1Dy3S7_ICSD_616230	0.88 eV	0.99 eV
Be1Cr1Er3S7_ICSD_616231	0.86 eV	1.10 eV
Be1Cr1La3S7_ICSD_616233	0.97 eV	0.73 eV
Be1Cr1Nd3S7_ICSD_616236	0.95 eV	0.93 eV
Be1Cr1S7Y3_ICSD_616240	0.90 eV	1.06 eV
Be1Cs1F3_ICSD_290358	5.35 eV	6.14 eV
Be1Cs1F3_ICSD_9870	6.65 eV	6.80 eV
Be1Cs1F4Li1_ICSD_42463	6.89 eV	6.98 eV
Be1Cs1F4Li1_ICSD_9434	0.13 eV	0.64 eV
Be1Cs1H3_ICSD_173446	3.19 eV	3.61 eV
Be1Cs2F4_ICSD_153084	6.87 eV	6.61 eV

Supplementary Table 676. Five-fold cross validated predictions for the band gap energy (70/277).

system	calculated	predicted
Be1Cs2F4_ICSD_23152	6.71 eV	6.68 eV
Be1F2_ICSD_173557	8.04 eV	7.61 eV
Be1F2_ICSD_173558	8.08 eV	7.57 eV
Be1F2_ICSD_261194	8.34 eV	7.71 eV
Be1F2_ICSD_9481	8.09 eV	7.62 eV
Be1F3H4N1_ICSD_61060	6.48 eV	6.59 eV
Be1F4H4Li1N1_ICSD_9433	6.56 eV	6.49 eV
Be1F4H5Li1N2_ICSD_1903	5.62 eV	6.28 eV
Be1F4H8N2_ICSD_200916	6.62 eV	6.14 eV
Be1F4K1Li1_ICSD_2773	6.85 eV	7.05 eV
Be1F4K2_ICSD_153081	6.87 eV	6.84 eV
Be1F4K2_ICSD_50337	6.84 eV	6.83 eV
Be1F4Li2_ICSD_14360	7.59 eV	7.53 eV
Be1F4Na2_ICSD_20366	6.39 eV	6.56 eV
Be1F4Na2_ICSD_28105	6.75 eV	6.62 eV
Be1F4Pb1_ICSD_24568	5.19 eV	4.46 eV
Be1F4Rb2_ICSD_153082	6.65 eV	6.63 eV
Be1F4Rb2_ICSD_61800	6.65 eV	6.75 eV
Be1F4Tl2_ICSD_171183	4.44 eV	4.40 eV
Be1F4Yb1_ICSD_411132	1.98 eV	1.99 eV
Be1F5K3_ICSD_14114	6.21 eV	6.32 eV
Be1F6Ho1K1_ICSD_2143	7.39 eV	5.15 eV
Be1Ga1La3S7_ICSD_616274	1.13 eV	1.71 eV
Be1Ge1Na2O4_ICSD_80357	3.66 eV	3.58 eV
Be1H2O2_ICSD_50445	5.72 eV	5.77 eV
Be1H2O4Se1_ICSD_410094	4.79 eV	4.35 eV
Be1H2_ICSD_161367	5.42 eV	4.83 eV
Be1H3K1_ICSD_173445	3.21 eV	3.61 eV
Be1H3Li1_ICSD_162770	4.81 eV	4.37 eV
Be1H3Li1_ICSD_162772	4.41 eV	4.76 eV
Be1H3Li1_ICSD_162773	4.40 eV	4.72 eV
Be1H3Li1_ICSD_162774	4.56 eV	4.44 eV
Be1H3Li1_ICSD_162775	4.89 eV	4.38 eV
Be1H3Li1_ICSD_173447	2.39 eV	3.61 eV
Be1H3Na1_ICSD_158250	0.91 eV	2.47 eV
Be1H3Rb1_ICSD_173449	3.29 eV	3.20 eV
Be1H4N1O4P1_ICSD_85445	5.24 eV	5.41 eV
Be1H4Na2_ICSD_159452	4.58 eV	3.84 eV
Be1H4O4Sr1_ICSD_180022	4.16 eV	4.31 eV
Be1H8I2O10_ICSD_83330	4.01 eV	3.65 eV
Be1H8O8S1_ICSD_74572	5.46 eV	5.18 eV
Be1H8O8Se1_ICSD_150083	3.99 eV	4.27 eV
Be1I2_ICSD_92585	4.12 eV	3.13 eV
Be1In1La3S7_ICSD_616297	1.66 eV	1.48 eV
Be1K1O4P1_ICSD_4255	5.24 eV	5.26 eV
Be1K1O9P3_ICSD_40866	5.54 eV	5.40 eV
Be1K4P2_ICSD_300110	1.14 eV	1.09 eV
Be1La3S7Sb1_ICSD_616308	1.50 eV	1.43 eV
Be1La3S7Sc1_ICSD_616309	1.32 eV	1.49 eV
Be1La3S7Ti1_ICSD_616310	1.25 eV	1.24 eV

Supplementary Table 677. Five-fold cross validated predictions for the band gap energy (71/277).

system	calculated	predicted
Be1La3S7V1_ICSD_616311	0.45 eV	0.95 eV
Be1Li1N1_ICSD_402341	2.69 eV	3.01 eV
Be1Li1P1_ICSD_616316	1.16 eV	1.07 eV
Be1Li1Sb1_ICSD_616318	0.85 eV	0.95 eV
Be1Li2O4Si1_ICSD_2319	5.58 eV	5.39 eV
Be1N2Si1_ICSD_25704	5.14 eV	4.34 eV
Be1N2Si1_ICSD_44112	3.56 eV	4.08 eV
Be1N4P2_ICSD_421385	4.04 eV	3.95 eV
Be1Na1Sb1_ICSD_100092	0.89 eV	0.89 eV
Be1Na2O2_ICSD_67154	2.84 eV	2.95 eV
Be1O1_ICSD_163467	6.85 eV	6.44 eV
Be1O1_ICSD_163826	8.17 eV	6.71 eV
Be1O1_ICSD_18147	7.09 eV	6.39 eV
Be1O1_ICSD_34237	7.45 eV	6.86 eV
Be1O4S1_ICSD_44801	6.94 eV	5.44 eV
Be1O6P2_ICSD_100404	5.83 eV	5.72 eV
Be1P2_ICSD_2262	0.83 eV	1.19 eV
Be1S1_ICSD_186889	3.14 eV	1.58 eV
Be1S1_ICSD_52719	0.99 eV	2.51 eV
Be1Se1_ICSD_52720	0.11 eV	1.85 eV
Be1Se1_ICSD_616419	2.67 eV	0.87 eV
Be1Te1_ICSD_290008	2.02 eV	0.96 eV
Be2C1_ICSD_183009	1.17 eV	0.90 eV
Be2Ca1P2_ICSD_616191	0.73 eV	0.77 eV
Be2Ca3H10O20P4_ICSD_40671	5.33 eV	5.36 eV
Be2Cs1F5_ICSD_2801	7.16 eV	6.98 eV
Be2Dy2Ge1O7_ICSD_39121	4.42 eV	4.42 eV
Be2Er2Ge1O7_ICSD_39123	4.38 eV	4.46 eV
Be2F5Rb1_ICSD_28541	6.85 eV	7.07 eV
Be2F7H4Li2N1_ICSD_240273	6.93 eV	6.87 eV
Be2F7Li1Na2_ICSD_9430	6.92 eV	6.92 eV
Be2F7Li2Rb1_ICSD_72	7.19 eV	7.20 eV
Be2F8K2Pb1_ICSD_9902	5.55 eV	4.62 eV
Be2F8K2Sr1_ICSD_109005	7.17 eV	7.12 eV
Be2Gd2Ge1O7_ICSD_39120	4.28 eV	4.17 eV
Be2Ge1La2O7_ICSD_39117	4.00 eV	3.90 eV
Be2Ge1O7Pr2_ICSD_39118	3.85 eV	3.53 eV
Be2Ge1O7Y2_ICSD_39122	4.49 eV	4.36 eV
Be2Ho2O7Si1_ICSD_73521	5.09 eV	4.64 eV
Be2K2Na4O5_ICSD_33849	2.46 eV	2.37 eV
Be2K4O4_ICSD_23633	1.14 eV	2.24 eV
Be2La2O5_ICSD_36063	4.04 eV	3.83 eV
Be2Mg1N2_ICSD_413358	4.06 eV	3.01 eV
Be2Mg1P2_ICSD_616328	0.66 eV	0.77 eV
Be2O10P3Rb1_ICSD_72985	5.62 eV	5.24 eV
Be2O4Si1_ICSD_202275	5.82 eV	5.88 eV
Be2O7Si1Y2_ICSD_23233	5.16 eV	4.63 eV
Be3Ca3F2Li2O12Si3_ICSD_39389	5.74 eV	5.12 eV
Be3Cd2F12Rb2_ICSD_15155	5.39 eV	4.80 eV
Be3Cl1Li4O12P3_ICSD_74525	6.22 eV	6.05 eV

Supplementary Table 678. Five-fold cross validated predictions for the band gap energy (72/277).

system	calculated	predicted
Be3F12K2Mg2_ICSD_15152	7.01 eV	7.11 eV
Be3F12K2Mn2_ICSD_83658	4.51 eV	3.78 eV
Be3F12K2Zn2_ICSD_24962	6.56 eV	6.61 eV
Be3F9K1Zn1_ICSD_18022	6.95 eV	6.85 eV
Be3F9Rb1Zn1_ICSD_23133	6.90 eV	6.92 eV
Be3H4O10P2_ICSD_88664	5.85 eV	5.61 eV
Be3K4O5_ICSD_33808	3.21 eV	2.50 eV
Be3Mn4O12S1Si3_ICSD_2709	3.13 eV	2.96 eV
Be3N2_ICSD_185490	2.76 eV	3.76 eV
Be3N2_ICSD_185491	4.27 eV	3.53 eV
Be3N2_ICSD_25656	3.33 eV	3.57 eV
Be3N2_ICSD_412667	3.32 eV	3.34 eV
Be3O4Sr1_ICSD_26179	4.13 eV	4.34 eV
Be3P2_ICSD_187677	0.22 eV	0.97 eV
Be3P2_ICSD_616384	0.89 eV	0.84 eV
Be4C6K6O19_ICSD_412642	4.78 eV	4.32 eV
Be4Ca2N4_ICSD_413357	2.40 eV	2.68 eV
Be4H2O9Si2_ICSD_202360	5.76 eV	5.39 eV
Be4N4Sr2_ICSD_413356	1.87 eV	2.36 eV
Be4Na10O17Si4_ICSD_68750	3.98 eV	3.90 eV
Be4Na1O7Sb1_ICSD_27599	3.39 eV	3.90 eV
Be4O7Te1_ICSD_1322	1.29 eV	3.54 eV
Be6Br2Li8O24P6_ICSD_80472	5.93 eV	5.20 eV
Be6Cd8O24S2Si6_ICSD_81485	2.98 eV	3.08 eV
Be6Cd8O24Se2Si6_ICSD_81486	2.93 eV	3.06 eV
Be6Cd8O24Si6Te2_ICSD_81487	2.95 eV	2.99 eV
Be6Ge6Mn8O24S2_ICSD_83841	2.40 eV	2.28 eV
Be6Ge6Mn8O24Se2_ICSD_83842	2.25 eV	2.24 eV
Be6Mn8O24Se2Si6_ICSD_83839	2.96 eV	2.97 eV
Be6Mn8O24Si6Te2_ICSD_83840	2.94 eV	2.83 eV
Be6O24S2Si6Zn8_ICSD_201641	4.51 eV	4.22 eV
Bi12Cl12Pt1_ICSD_415739	0.87 eV	1.18 eV
Bi12O20Si1_ICSD_422389	2.41 eV	2.39 eV
Bi14Br16Pd1_ICSD_412895	1.18 eV	1.55 eV
Bi1Br1Cd1S2_ICSD_171725	1.80 eV	1.63 eV
Bi1Br1Cd1Se2_ICSD_171726	1.44 eV	1.31 eV
Bi1Br1Cu3O8Se2_ICSD_280759	1.12 eV	1.03 eV
Bi1Br1Mn1S2_ICSD_415307	1.55 eV	1.38 eV
Bi1Br1O1_ICSD_61225	2.31 eV	1.92 eV
Bi1Br1O2Sr1_ICSD_97509	3.16 eV	2.93 eV
Bi1Br1_ICSD_1560	0.68 eV	1.26 eV
Bi1Br3_ICSD_100293	2.62 eV	2.29 eV
Bi1Br3_ICSD_100294	2.86 eV	2.20 eV
Bi1Br7Hg6Sb4_ICSD_411218	1.02 eV	1.10 eV
Bi1C1Ca1F1O4_ICSD_87759	1.07 eV	2.39 eV
Bi1C3H6N3S6_ICSD_422349	2.49 eV	2.59 eV
Bi1C4N4Rb1S4_ICSD_164	2.47 eV	2.77 eV
Bi1C5H5K2O10_ICSD_109956	4.12 eV	3.65 eV
Bi1Ca1Cl1O2_ICSD_84635	3.63 eV	2.93 eV
Bi1Ca1Li1_ICSD_616539	0.58 eV	0.50 eV

Supplementary Table 679. Five-fold cross validated predictions for the band gap energy (73/277).

system	calculated	predicted
Bi1Ca1O5V1_ICSD_73184	3.23 eV	2.99 eV
Bi1Ca2O6V1_ICSD_50939	3.02 eV	2.87 eV
Bi1Ca2_ICSD_42136	0.10 eV	0.26 eV
Bi1Ca3N1_ICSD_106320	0.38 eV	0.58 eV
Bi1Cd1Cl1O2_ICSD_280770	2.88 eV	2.35 eV
Bi1Cd1Cl1S2_ICSD_171724	1.98 eV	1.77 eV
Bi1Cd1I1Se2_ICSD_171727	1.32 eV	1.27 eV
Bi1Ce1O1S2_ICSD_80	0.55 eV	1.18 eV
Bi1Ce1Pd1_ICSD_616563	0.10 eV	0.10 eV
Bi1Cl1Cu3O8Se2_ICSD_54190	1.28 eV	1.16 eV
Bi1Cl1F8_ICSD_39555	2.22 eV	2.71 eV
Bi1Cl1Mn1S2_ICSD_172156	1.70 eV	1.52 eV
Bi1Cl1O1_ICSD_24608	2.57 eV	2.23 eV
Bi1Cl1O2Sr1_ICSD_84636	3.33 eV	3.19 eV
Bi1Cl1O3Se1_ICSD_411169	3.56 eV	3.49 eV
Bi1Cl1O3Se1_ICSD_98000	3.57 eV	3.26 eV
Bi1Cl1S1_ICSD_100173	1.95 eV	1.56 eV
Bi1Cl1Te1_ICSD_79362	1.34 eV	1.09 eV
Bi1Cl2Cu1S1_ICSD_413289	0.87 eV	1.17 eV
Bi1Cl2K1O4S1_ICSD_155203	4.19 eV	3.51 eV
Bi1Cl3O3Sr3_ICSD_80637	4.06 eV	3.68 eV
Bi1Cl3_ICSD_41179	3.55 eV	2.56 eV
Bi1Cl4N3S4_ICSD_9460	2.03 eV	1.99 eV
Bi1Cl6Cs1K2_ICSD_201983	3.92 eV	3.32 eV
Bi1Cl6Cs2Na1_ICSD_59195	3.71 eV	3.72 eV
Bi1Cl6Tl3_ICSD_421317	2.66 eV	2.65 eV
Bi1Cl8F4H3K6_ICSD_39524	4.18 eV	3.63 eV
Bi1Co1O3_ICSD_157833	0.68 eV	1.17 eV
Bi1Co1Zr1_ICSD_107120	0.87 eV	0.57 eV
Bi1Cr1O3_ICSD_160454	1.55 eV	1.48 eV
Bi1Cr1O3_ICSD_160456	1.31 eV	1.40 eV
Bi1Cr2O8Rb1_ICSD_201624	2.42 eV	2.38 eV
Bi1Cs1F6_ICSD_15122	2.91 eV	3.19 eV
Bi1Cs1Ge1S4_ICSD_281168	2.02 eV	2.00 eV
Bi1Cs1O2_ICSD_406564	2.34 eV	1.99 eV
Bi1Cs1S2_ICSD_72975	1.68 eV	1.67 eV
Bi1Cs1S4Si1_ICSD_281169	2.50 eV	2.31 eV
Bi1Cs2F6K1_ICSD_9383	4.93 eV	4.75 eV
Bi1Cs2F6Na1_ICSD_9382	5.02 eV	4.83 eV
Bi1Cs2F6Rb1_ICSD_9384	4.76 eV	4.35 eV
Bi1Cs2F6Tl1_ICSD_9385	3.09 eV	3.27 eV
Bi1Cs3O3_ICSD_406563	3.06 eV	2.67 eV
Bi1Cs3Se3_ICSD_85410	2.09 eV	1.82 eV
Bi1Cs3_ICSD_58769	0.22 eV	0.31 eV
Bi1Cs3_ICSD_659568	0.21 eV	0.33 eV
Bi1Cu1O1Se1_ICSD_74475	0.52 eV	0.62 eV
Bi1Cu1O8W2_ICSD_67569	1.43 eV	1.68 eV
Bi1Cu1P2Se6_ICSD_170643	0.94 eV	1.07 eV
Bi1Cu1Pb1S3_ICSD_9120	0.83 eV	0.83 eV
Bi1Cu1Pt1S3_ICSD_180450	0.76 eV	0.99 eV

Supplementary Table 680. Five-fold cross validated predictions for the band gap energy (74/277).

system	calculated	predicted
Bi1Cu1S2_ICSD_38779	0.72 eV	0.84 eV
Bi1Cu2O6P1_ICSD_75387	0.71 eV	1.09 eV
Bi1Cu3I1O8Se2_ICSD_54191	0.81 eV	1.09 eV
Bi1Cu3S3_ICSD_616615	1.01 eV	0.97 eV
Bi1Dy1Ni1_ICSD_58779	0.11 eV	0.12 eV
Bi1Er1Ge1O5_ICSD_96409	3.32 eV	2.66 eV
Bi1F1O1_ICSD_24096	3.13 eV	2.52 eV
Bi1F3_ICSD_1269	4.69 eV	3.73 eV
Bi1F3_ICSD_25567	3.96 eV	3.38 eV
Bi1F3_ICSD_655136	3.95 eV	3.24 eV
Bi1F4K1_ICSD_63166	3.26 eV	3.55 eV
Bi1F4Li1_ICSD_65404	4.84 eV	3.16 eV
Bi1F4Rb1_ICSD_63167	2.90 eV	3.13 eV
Bi1F5K2_ICSD_418777	4.77 eV	4.26 eV
Bi1F5_ICSD_25023	1.97 eV	2.69 eV
Bi1F6K1Rb2_ICSD_9387	4.77 eV	4.72 eV
Bi1F6K1_ICSD_25024	2.91 eV	3.32 eV
Bi1F6K1_ICSD_25025	2.85 eV	3.33 eV
Bi1F6Li1_ICSD_15119	2.75 eV	3.37 eV
Bi1F6Na1Rb2_ICSD_9386	4.92 eV	4.59 eV
Bi1F6Na1_ICSD_15120	2.88 eV	3.22 eV
Bi1F6Rb1_ICSD_15121	2.78 eV	2.92 eV
Bi1F7Kr1_ICSD_279626	2.45 eV	2.45 eV
Bi1Fe1O3_ICSD_162264	1.66 eV	1.71 eV
Bi1Fe1O3_ICSD_168320	1.47 eV	1.34 eV
Bi1Fe1O3_ICSD_168321	1.34 eV	1.27 eV
Bi1Fe1O3_ICSD_180498	1.67 eV	1.53 eV
Bi1Fe1O3_ICSD_188467	1.34 eV	1.10 eV
Bi1Fe1O3_ICSD_22342	1.57 eV	1.50 eV
Bi1Fe2H1O9Si2_ICSD_200069	2.28 eV	2.20 eV
Bi1Ga1O3_ICSD_157550	1.30 eV	1.18 eV
Bi1Ga1O3_ICSD_171709	1.91 eV	2.41 eV
Bi1Ge1K1S4_ICSD_421486	1.94 eV	1.99 eV
Bi1Ge1Rb1S4_ICSD_281167	1.98 eV	2.00 eV
Bi1H1O12P4_ICSD_1967	5.01 eV	3.96 eV
Bi1H1O7Sr4_ICSD_419369	1.26 eV	2.41 eV
Bi1I1Mn1Se2_ICSD_415138	1.23 eV	1.00 eV
Bi1I1O1_ICSD_391354	1.52 eV	1.96 eV
Bi1I1O2Sr1_ICSD_97510	2.89 eV	2.76 eV
Bi1I1O3Te1_ICSD_56218	2.34 eV	2.07 eV
Bi1I1O4_ICSD_262019	2.40 eV	2.43 eV
Bi1I1S1_ICSD_25575	1.00 eV	0.98 eV
Bi1I1Se1_ICSD_280311	1.56 eV	1.19 eV
Bi1I1Te1_ICSD_263109	1.20 eV	0.83 eV
Bi1I1_ICSD_1558	0.71 eV	0.89 eV
Bi1I1_ICSD_1559	0.70 eV	1.05 eV
Bi1I3_ICSD_187608	2.27 eV	2.38 eV
Bi1I3_ICSD_187611	2.28 eV	2.36 eV
Bi1I3_ICSD_20676	2.41 eV	2.30 eV
Bi1I3_ICSD_56570	2.51 eV	2.36 eV

Supplementary Table 681. Five-fold cross validated predictions for the band gap energy (75/277).

system	calculated	predicted
Bi1I6Tl3_ICSD_417537	2.48 eV	2.19 eV
Bi1In1O3_ICSD_158758	0.22 eV	1.10 eV
Bi1In1O3_ICSD_171756	2.91 eV	2.45 eV
Bi1In1O3_ICSD_171757	2.49 eV	2.37 eV
Bi1In1S3_ICSD_290195	1.35 eV	1.41 eV
Bi1Ir1S1_ICSD_616740	0.74 eV	0.88 eV
Bi1Ir1Se1_ICSD_616741	0.58 eV	0.73 eV
Bi1K1Li6O6_ICSD_71035	1.52 eV	1.99 eV
Bi1K1Mg1_ICSD_616748	0.43 eV	0.61 eV
Bi1K1O2_ICSD_407209	1.85 eV	1.81 eV
Bi1K1O3_ICSD_76976	0.57 eV	2.11 eV
Bi1K1O8W2_ICSD_391361	2.76 eV	2.86 eV
Bi1K1P2S6_ICSD_409686	2.23 eV	2.06 eV
Bi1K1P2Se6_ICSD_90153	1.76 eV	1.62 eV
Bi1K1S4Si1_ICSD_421485	2.04 eV	2.18 eV
Bi1K1_ICSD_55065	0.42 eV	0.40 eV
Bi1K2Rb1Se3_ICSD_85412	2.15 eV	1.98 eV
Bi1K3O3_ICSD_407293	3.00 eV	2.50 eV
Bi1K3P2S8_ICSD_81772	2.11 eV	2.03 eV
Bi1K3Se3_ICSD_78841	2.07 eV	1.87 eV
Bi1K3Te3_ICSD_300183	1.51 eV	1.15 eV
Bi1K9O24U6_ICSD_66528	2.58 eV	2.72 eV
Bi1Li1Mg1_ICSD_108112	0.46 eV	0.56 eV
Bi1Li1O2_ICSD_25385	2.17 eV	1.99 eV
Bi1Li1O4Pd2_ICSD_202930	1.13 eV	1.18 eV
Bi1Li1Sr1_ICSD_58800	0.55 eV	0.48 eV
Bi1Li1Yb1_ICSD_602201	0.25 eV	0.38 eV
Bi1Li3O4_ICSD_109087	1.16 eV	1.96 eV
Bi1Li3_ICSD_58797	0.37 eV	1.00 eV
Bi1Li5O5_ICSD_203031	1.45 eV	1.79 eV
Bi1Mg2O6P1_ICSD_73894	3.63 eV	3.40 eV
Bi1Mg2O6V1_ICSD_72175	3.18 eV	2.69 eV
Bi1Mn1O3_ICSD_245299	0.28 eV	0.32 eV
Bi1Mn1O3_ICSD_245300	0.26 eV	0.30 eV
Bi1Mn1O3_ICSD_50795	0.28 eV	0.31 eV
Bi1Mn1O5V1_ICSD_59720	2.12 eV	1.92 eV
Bi1Mn2O5_ICSD_169736	0.30 eV	0.84 eV
Bi1Mn2O6P1_ICSD_59673	1.99 eV	1.94 eV
Bi1N1Sr3_ICSD_152053	0.26 eV	0.31 eV
Bi1Na1O2_ICSD_10317	1.20 eV	1.39 eV
Bi1Na1O3_ICSD_27553	1.16 eV	1.98 eV
Bi1Na1O6Sr3_ICSD_419367	1.79 eV	2.17 eV
Bi1Na3O3_ICSD_23347	2.91 eV	2.46 eV
Bi1Na3O4_ICSD_10319	1.05 eV	1.77 eV
Bi1Nb1O4_ICSD_415850	2.64 eV	2.64 eV
Bi1Nb1O4_ICSD_74338	2.96 eV	2.63 eV
Bi1O2Rb1_ICSD_407208	2.12 eV	2.00 eV
Bi1O3Rb3_ICSD_407294	3.11 eV	2.49 eV
Bi1O3Rh1_ICSD_188150	0.91 eV	1.29 eV
Bi1O3Sc1_ICSD_171384	2.79 eV	2.63 eV

Supplementary Table 682. Five-fold cross validated predictions for the band gap energy (76/277).

system	calculated	predicted
Bi1O3Sc1_ICSD_171385	2.78 eV	2.63 eV
Bi1O3Sc1_ICSD_181115	0.66 eV	1.93 eV
Bi1O3Sr1_ICSD_85173	0.19 eV	1.27 eV
Bi1O4P1_ICSD_189659	3.84 eV	3.55 eV
Bi1O4P1_ICSD_27469	3.69 eV	3.77 eV
Bi1O4Sb1_ICSD_80821	0.50 eV	1.33 eV
Bi1O4Ta1_ICSD_415849	2.99 eV	2.59 eV
Bi1O4Ta1_ICSD_97423	3.00 eV	2.68 eV
Bi1O4V1_ICSD_100601	2.28 eV	2.11 eV
Bi1O4V1_ICSD_100733	2.76 eV	2.27 eV
Bi1O5P1Pb1_ICSD_419125	3.36 eV	3.12 eV
Bi1O5Pb1V1_ICSD_419123	2.20 eV	2.39 eV
Bi1O5Pb1V1_ICSD_419126	2.91 eV	2.54 eV
Bi1O6P1Zn2_ICSD_91234	3.25 eV	2.96 eV
Bi1O8P1Pb4_ICSD_50649	2.56 eV	2.30 eV
Bi1Os1Se1_ICSD_616892	0.74 eV	0.87 eV
Bi1P2S6Tl1_ICSD_249461	2.01 eV	2.04 eV
Bi1Rb1S2_ICSD_52735	1.35 eV	1.64 eV
Bi1Rb1S4Si1_ICSD_281166	2.07 eV	2.24 eV
Bi1Rb1_ICSD_55066	0.39 eV	0.40 eV
Bi1Rb3Se3_ICSD_85411	2.08 eV	1.83 eV
Bi1Rh1S1_ICSD_617013	0.45 eV	0.64 eV
Bi1Rh1Se1_ICSD_617014	0.28 eV	0.50 eV
Bi1S2Tl1_ICSD_172572	0.46 eV	0.88 eV
Bi1Se2Tl1_ICSD_43314	0.23 eV	0.48 eV
Bi1Te2Tl1_ICSD_15412	0.43 eV	0.38 eV
Bi24Ge2O40_ICSD_68431	2.44 eV	2.28 eV
Bi24O40Pb2_ICSD_75392	1.74 eV	2.03 eV
Bi24O40Ti2_ICSD_75389	2.39 eV	2.15 eV
Bi2Br1Dy1O4_ICSD_92417	1.34 eV	1.39 eV
Bi2Br1Er1O4_ICSD_92420	1.37 eV	1.41 eV
Bi2Br1Eu1O4_ICSD_92414	0.18 eV	0.33 eV
Bi2Br1Gd1O4_ICSD_92415	1.30 eV	1.24 eV
Bi2Br1Ho1O4_ICSD_92419	1.39 eV	1.37 eV
Bi2Br1In1Se4_ICSD_159465	1.29 eV	1.12 eV
Bi2Br1Lu1O4_ICSD_92423	1.41 eV	1.46 eV
Bi2Br1Nd1O4_ICSD_92412	1.25 eV	1.29 eV
Bi2Br1O4Pr1_ICSD_92411	1.12 eV	1.15 eV
Bi2Br1O4Tb1_ICSD_92416	1.36 eV	1.36 eV
Bi2Br1O4Y1_ICSD_92418	1.35 eV	1.39 eV
Bi2Br8Te4_ICSD_83806	1.04 eV	2.02 eV
Bi2Br9Cs3_ICSD_1142	2.58 eV	2.20 eV
Bi2Br9Cs3_ICSD_96723	2.58 eV	2.21 eV
Bi2C1O5_ICSD_94740	1.14 eV	2.33 eV
Bi2C2Ca1O8_ICSD_94741	1.06 eV	1.99 eV
Bi2Ca1Mg2_ICSD_261988	0.46 eV	0.46 eV
Bi2Ca4O1_ICSD_416137	0.82 eV	0.75 eV
Bi2Cd1Cs2S5_ICSD_97427	1.74 eV	1.65 eV
Bi2Cd1Ge1O6_ICSD_82157	1.91 eV	2.34 eV
Bi2Cl1Dy1O4_ICSD_92404	1.44 eV	1.41 eV

Supplementary Table 683. Five-fold cross validated predictions for the band gap energy (77/277).

system	calculated	predicted
Bi2Cl1Er1O4_ICSD_92407	1.44 eV	1.47 eV
Bi2Cl1Eu1O4_ICSD_92401	0.41 eV	0.37 eV
Bi2Cl1Gd1O4_ICSD_92402	1.27 eV	1.25 eV
Bi2Cl1Ho1O4_ICSD_92406	1.41 eV	1.42 eV
Bi2Cl1In1S4_ICSD_484	1.88 eV	1.58 eV
Bi2Cl1Lu1O4_ICSD_92410	1.47 eV	1.59 eV
Bi2Cl1Nd1O4_ICSD_92399	1.31 eV	1.37 eV
Bi2Cl1O4Pr1_ICSD_92398	1.09 eV	1.25 eV
Bi2Cl1O4Tb1_ICSD_92403	1.37 eV	1.38 eV
Bi2Cl1O4Y1_ICSD_92405	1.35 eV	1.61 eV
Bi2Cl1O4Yb1_ICSD_92409	0.17 eV	0.29 eV
Bi2Co1Mn1O6_ICSD_153856	0.76 eV	0.89 eV
Bi2Co1O7S1_ICSD_65135	2.55 eV	2.36 eV
Bi2Cr1Fe1O6_ICSD_246426	1.81 eV	1.57 eV
Bi2Cs1Cu1S4_ICSD_93370	0.75 eV	1.04 eV
Bi2Cs2Pd1_ICSD_658703	0.54 eV	0.48 eV
Bi2Cs2Pt1_ICSD_658701	0.49 eV	0.60 eV
Bi2Cs3I9_ICSD_1448	2.07 eV	2.20 eV
Bi2Cs3I9_ICSD_411633	2.37 eV	2.19 eV
Bi2Cu1K1S4_ICSD_91297	0.89 eV	0.95 eV
Bi2Cu1O12Se4_ICSD_66826	1.75 eV	1.61 eV
Bi2Cu1O4_ICSD_12104	0.43 eV	0.62 eV
Bi2Cu1O4_ICSD_68812	0.83 eV	0.84 eV
Bi2Dy1I1O4_ICSD_92431	1.37 eV	1.40 eV
Bi2Er1I1O4_ICSD_89618	1.42 eV	1.37 eV
Bi2Eu1I1O4_ICSD_92428	0.13 eV	0.41 eV
Bi2Eu1Se4_ICSD_600805	1.02 eV	0.73 eV
Bi2Fe4O9_ICSD_26808	1.34 eV	1.27 eV
Bi2Ga2In4S12_ICSD_410032	1.84 eV	1.44 eV
Bi2Ga4O9_ICSD_248245	2.92 eV	2.62 eV
Bi2Ga4S8_ICSD_408441	2.55 eV	1.99 eV
Bi2Ga4Se8_ICSD_408442	1.93 eV	1.73 eV
Bi2Ge1O5_ICSD_62488	2.31 eV	2.27 eV
Bi2Ge1O5_ICSD_65522	2.34 eV	2.43 eV
Bi2Ge1O5_ICSD_94334	2.32 eV	2.37 eV
Bi2Ge1Te4_ICSD_658633	0.59 eV	0.45 eV
Bi2Ge3O9_ICSD_100277	2.98 eV	2.89 eV
Bi2H16Na6O14_ICSD_412583	3.94 eV	3.11 eV
Bi2Hg1S4_ICSD_14189	1.22 eV	1.16 eV
Bi2Ho1I1O4_ICSD_92433	1.40 eV	1.37 eV
Bi2I1La1O4_ICSD_92424	1.21 eV	1.32 eV
Bi2I1Lu1O4_ICSD_92437	1.48 eV	1.41 eV
Bi2I1Nd1O4_ICSD_92426	1.28 eV	1.23 eV
Bi2I1O4Pr1_ICSD_92425	1.28 eV	1.19 eV
Bi2I1O4Tb1_ICSD_92430	1.34 eV	1.37 eV
Bi2I1O4Y1_ICSD_92432	1.40 eV	1.40 eV
Bi2In4S9_ICSD_2839	1.66 eV	1.39 eV
Bi2K3O12P3_ICSD_409582	3.77 eV	3.61 eV
Bi2K4O5_ICSD_408000	2.64 eV	2.45 eV
Bi2Li8O10Pd1_ICSD_73000	1.25 eV	1.53 eV

Supplementary Table 684. Five-fold cross validated predictions for the band gap energy (78/277).

system	calculated	predicted
Bi2Mg2Sr1_ICSD_616807	0.46 eV	0.43 eV
Bi2Mn1Ni1O6_ICSD_159285	0.86 eV	0.98 eV
Bi2Mo1O6_ICSD_14266	2.11 eV	1.29 eV
Bi2Mo1O6_ICSD_17070	0.44 eV	1.52 eV
Bi2Mo1O6_ICSD_37251	0.36 eV	1.46 eV
Bi2Na1O11Sb3_ICSD_79859	2.39 eV	2.14 eV
Bi2Nb2O9Pb1_ICSD_20668	0.88 eV	1.76 eV
Bi2O10Pb2V2_ICSD_60577	2.93 eV	2.65 eV
Bi2O10Te2W1_ICSD_78917	2.87 eV	2.32 eV
Bi2O11Ti4_ICSD_79769	2.36 eV	2.48 eV
Bi2O13P4_ICSD_65133	4.54 eV	3.79 eV
Bi2O2S1_ICSD_29451	0.84 eV	0.69 eV
Bi2O2Se1_ICSD_411143	0.40 eV	1.01 eV
Bi2O3_ICSD_168808	0.16 eV	0.98 eV
Bi2O3_ICSD_168810	2.39 eV	1.85 eV
Bi2O3_ICSD_168811	2.59 eV	2.23 eV
Bi2O3_ICSD_168813	1.86 eV	1.96 eV
Bi2O3_ICSD_168814	2.61 eV	2.04 eV
Bi2O3_ICSD_168815	1.82 eV	2.08 eV
Bi2O3_ICSD_169686	2.35 eV	2.18 eV
Bi2O3_ICSD_186365	1.12 eV	1.07 eV
Bi2O3_ICSD_261777	2.31 eV	2.05 eV
Bi2O3_ICSD_27152	1.78 eV	1.95 eV
Bi2O3_ICSD_421855	1.59 eV	1.76 eV
Bi2O3_ICSD_616890	2.30 eV	2.18 eV
Bi2O3_ICSD_62979	1.67 eV	1.83 eV
Bi2O4Pd1_ICSD_200145	0.95 eV	1.00 eV
Bi2O4Pd1_ICSD_9622	0.26 eV	0.84 eV
Bi2O4Sr1_ICSD_80668	2.69 eV	1.40 eV
Bi2O4_ICSD_79500	1.22 eV	1.40 eV
Bi2O5Se1_ICSD_409518	2.88 eV	2.50 eV
Bi2O5Si1_ICSD_30995	2.73 eV	2.77 eV
Bi2O5Sr2_ICSD_86415	2.27 eV	2.03 eV
Bi2O5Te1_ICSD_36446	2.44 eV	2.25 eV
Bi2O6Te1_ICSD_6239	1.24 eV	1.59 eV
Bi2O6Ti1Zn1_ICSD_162767	1.51 eV	2.07 eV
Bi2O6Ti1Zn1_ICSD_162768	1.66 eV	2.00 eV
Bi2O6Ti1Zn1_ICSD_186802	2.83 eV	2.06 eV
Bi2O6Ti1Zn1_ICSD_186803	1.63 eV	1.91 eV
Bi2O6U1_ICSD_1805	2.21 eV	1.70 eV
Bi2O6U1_ICSD_1806	2.02 eV	1.68 eV
Bi2O6W1_ICSD_23584	2.07 eV	2.18 eV
Bi2O6W1_ICSD_66579	2.09 eV	2.36 eV
Bi2O6W1_ICSD_67647	2.15 eV	1.92 eV
Bi2O7Pt2_ICSD_161104	1.07 eV	1.20 eV
Bi2O7Sn2_ICSD_50311	2.63 eV	2.24 eV
Bi2O7Ti2_ICSD_180394	2.89 eV	2.45 eV
Bi2O9Si3_ICSD_155234	3.61 eV	3.29 eV
Bi2O9W2_ICSD_88428	2.31 eV	2.02 eV
Bi2Pb1S4_ICSD_31859	1.01 eV	1.08 eV

Supplementary Table 685. Five-fold cross validated predictions for the band gap energy (79/277).

system	calculated	predicted
Bi2Pb1Te4_ICSD_616936	0.49 eV	0.43 eV
Bi2Pb2Se5_ICSD_30372	0.49 eV	0.57 eV
Bi2Pb3S6_ICSD_92981	0.61 eV	0.93 eV
Bi2S1Te2_ICSD_26720	0.41 eV	0.72 eV
Bi2S3_ICSD_617021	1.36 eV	1.23 eV
Bi2S4Yb1_ICSD_600812	1.13 eV	1.18 eV
Bi2Se2Te1_ICSD_54838	0.76 eV	0.59 eV
Bi2Se3_ICSD_171571	0.99 eV	0.74 eV
Bi2Se3_ICSD_617083	0.49 eV	0.70 eV
Bi2Se3_ICSD_617096	0.56 eV	1.06 eV
Bi2Te3_ICSD_617187	0.31 eV	0.36 eV
Bi3Br1Se4_ICSD_411096	1.04 eV	1.12 eV
Bi3Cl5O10Te4_ICSD_168978	2.37 eV	2.80 eV
Bi3Cs1Se5_ICSD_171610	1.00 eV	0.81 eV
Bi3F6N1_ICSD_79395	2.79 eV	3.28 eV
Bi3Fe1Mo2O12_ICSD_45	2.44 eV	1.88 eV
Bi3Mn4N1O15_ICSD_260393	0.87 eV	1.45 eV
Bi3Na1O10V2_ICSD_88455	2.72 eV	2.41 eV
Bi3O7Sb1_ICSD_51779	2.19 eV	1.87 eV
Bi3O8Re1_ICSD_185903	2.82 eV	2.61 eV
Bi3O8Re1_ICSD_185904	2.72 eV	2.38 eV
Bi4Br2O5_ICSD_412591	2.55 eV	2.42 eV
Bi4Br2O9Te2_ICSD_79508	0.88 eV	1.98 eV
Bi4Ce3Pd3_ICSD_419162	0.28 eV	0.13 eV
Bi4Ce3Pt3_ICSD_616565	0.33 eV	0.37 eV
Bi4Cl14Se4_ICSD_410910	1.35 eV	2.44 eV
Bi4Cl16Te14_ICSD_391157	0.59 eV	1.05 eV
Bi4Cl1O8Ta1_ICSD_59601	1.13 eV	1.71 eV
Bi4Cu3La3_ICSD_167250	0.18 eV	0.25 eV
Bi4Ge1Te7_ICSD_42891	0.64 eV	0.43 eV
Bi4Ge3O12_ICSD_108872	3.46 eV	2.91 eV
Bi4I2O5_ICSD_412590	2.25 eV	2.33 eV
Bi4I2Ru1_ICSD_406949	0.53 eV	0.64 eV
Bi4Li1Nb3O14_ICSD_391261	2.90 eV	2.66 eV
Bi4O11Ta2_ICSD_50601	2.85 eV	2.58 eV
Bi4O12Si3_ICSD_69430	3.91 eV	3.21 eV
Bi4O7_ICSD_51778	1.05 eV	1.76 eV
Bi4Pb1Te7_ICSD_250249	0.38 eV	0.40 eV
Bi4Sn1Te7_ICSD_236253	0.39 eV	0.45 eV
Bi4Te3_ICSD_30526	0.13 eV	0.34 eV
Bi5Cl12Ga3_ICSD_414089	1.80 eV	2.33 eV
Bi5I1O7_ICSD_30978	2.53 eV	2.07 eV
Bi6Cu2Pb2S12_ICSD_160417	0.82 eV	1.00 eV
Bi6Se13Sr4_ICSD_62782	0.49 eV	0.84 eV
Bi7Cs3Se12_ICSD_61785	0.62 eV	0.79 eV
Bi7F11O5_ICSD_167074	3.92 eV	3.19 eV
Bi7O18Ta3_ICSD_280093	2.37 eV	2.46 eV
Bi8K2Se13_ICSD_72976	0.80 eV	0.89 eV
Bi9I3Rh2_ICSD_411136	0.46 eV	0.64 eV
Br1.6Cl0.4Sr1_ICSD_35052	4.44 eV	4.44 eV

Supplementary Table 686. Five-fold cross validated predictions for the band gap energy (80/277).

system	calculated	predicted
Br10Co1Er6_ICSD_424427	0.17 eV	0.50 eV
Br10Co1Y6_ICSD_424465	0.18 eV	0.43 eV
Br10Gd6Ir1_ICSD_424456	0.10 eV	0.15 eV
Br10Ir1Tb6_ICSD_424434	0.09 eV	0.21 eV
Br10Ni1Tb6_ICSD_424435	0.36 eV	1.11 eV
Br10Ni1Y6_ICSD_424466	0.42 eV	0.50 eV
Br10O1Ta2Te4_ICSD_401906	1.00 eV	1.42 eV
Br10O4Pb9_ICSD_35381	2.92 eV	2.73 eV
Br10Pd1Y6_ICSD_424467	0.33 eV	0.52 eV
Br10Rb1Re3_ICSD_33911	0.90 eV	1.19 eV
Br10Re2Te6_ICSD_410190	1.41 eV	1.45 eV
Br11Cs1Nb4_ICSD_26077	0.32 eV	0.83 eV
Br12Co1Ho7_ICSD_424451	0.45 eV	0.60 eV
Br12Co1Sc7_ICSD_424474	0.37 eV	0.98 eV
Br12Os1Se2_ICSD_182315	1.03 eV	1.22 eV
Br12Ta2Te4_ICSD_401905	1.16 eV	1.37 eV
Br14Cd1W6_ICSD_80887	2.26 eV	2.35 eV
Br14Mo6Pb1_ICSD_36573	2.49 eV	2.04 eV
Br14Rb2W6_ICSD_390028	2.91 eV	1.70 eV
Br14Ta6_ICSD_402031	1.11 eV	1.13 eV
Br15Co1Th6_ICSD_33926	0.70 eV	0.99 eV
Br16Re4Te8_ICSD_78924	2.05 eV	1.64 eV
Br17Nb6Rb3S1_ICSD_400496	0.55 eV	0.95 eV
Br18Cs1Er1Ta6_ICSD_77666	1.22 eV	1.19 eV
Br18K4Nb6_ICSD_49687	1.07 eV	0.86 eV
Br18Nb6Tl4_ICSD_402033	1.04 eV	1.27 eV
Br1C1Cl1F1H3_ICSD_424851	2.69 eV	3.51 eV
Br1C1Cu1H2N2_ICSD_420807	2.55 eV	2.21 eV
Br1C1K1N2O4_ICSD_42826	2.10 eV	2.75 eV
Br1C2H8N1_ICSD_110459	4.70 eV	4.58 eV
Br1C2H8N1_ICSD_400651	4.71 eV	4.28 eV
Br1C2N2Ni1Rb1_ICSD_380470	2.30 eV	2.21 eV
Br1C3H10N1_ICSD_171150	4.75 eV	4.75 eV
Br1C4N3_ICSD_246789	3.30 eV	3.18 eV
Br1C5Mn1O5_ICSD_281349	2.82 eV	2.88 eV
Br1C5O5Re1_ICSD_66697	2.49 eV	2.54 eV
Br1C5O5Tc1_ICSD_167773	2.60 eV	2.52 eV
Br1C8H20Li1O4_ICSD_151064	4.61 eV	4.44 eV
Br1Ca1H1_ICSD_25542	4.14 eV	3.08 eV
Br1Ca2H3_ICSD_420928	1.57 eV	3.09 eV
Br1Ca2N1_ICSD_153105	2.42 eV	2.05 eV
Br1Cd1S2Sb1_ICSD_171723	1.58 eV	1.55 eV
Br1Cd2P3_ICSD_100817	1.17 eV	1.16 eV
Br1Cd2P3_ICSD_100880	1.17 eV	1.26 eV
Br1Ce2S5Sb1_ICSD_51484	1.46 eV	1.58 eV
Br1Ce3S8Si2_ICSD_39052	2.69 eV	2.53 eV
Br1Cl14P3_ICSD_80214	1.56 eV	2.02 eV
Br1Cl1_ICSD_424850	1.75 eV	2.06 eV
Br1Cr1O1_ICSD_27092	1.31 eV	1.45 eV
Br1Cr1S1_ICSD_69659	0.63 eV	0.80 eV

Supplementary Table 687. Five-fold cross validated predictions for the band gap energy (81/277).

system	calculated	predicted
Br1Cr2O4P1_ICSD_410823	2.03 eV	2.18 eV
Br1Cs1F6_ICSD_65712	3.99 eV	4.30 eV
Br1Cs1O3_ICSD_74769	4.29 eV	3.93 eV
Br1Cs1O4_ICSD_201158	3.68 eV	3.68 eV
Br1Cs1_ICSD_53834	4.27 eV	4.12 eV
Br1Cs1_ICSD_61516	4.27 eV	4.22 eV
Br1Cu1H6N2_ICSD_170947	2.41 eV	2.03 eV
Br1Cu1Hg1S1_ICSD_412308	0.73 eV	0.92 eV
Br1Cu1Hg1Se1_ICSD_411771	0.78 eV	0.91 eV
Br1Cu1N4S4_ICSD_33515	1.09 eV	1.64 eV
Br1Cu1O2Sr2_ICSD_65470	2.74 eV	2.24 eV
Br1Cu1Se3_ICSD_71309	1.15 eV	1.03 eV
Br1Cu1Te2_ICSD_67252	0.91 eV	0.92 eV
Br1Cu1_ICSD_30091	0.96 eV	1.13 eV
Br1Cu1_ICSD_78275	1.14 eV	1.06 eV
Br1Cu1_ICSD_78278	0.76 eV	1.06 eV
Br1Cu1_ICSD_78280	0.89 eV	1.00 eV
Br1Cu6P1S5_ICSD_89450	1.10 eV	1.09 eV
Br1Dy1Mo1O4_ICSD_425268	1.82 eV	1.93 eV
Br1Dy1S1_ICSD_79106	2.87 eV	2.55 eV
Br1Er1Mo1O4_ICSD_425270	1.91 eV	2.15 eV
Br1Eu1I1_ICSD_59885	2.99 eV	3.16 eV
Br1Eu2P1_ICSD_202067	0.84 eV	0.88 eV
Br1Eu3O4_ICSD_34605	0.23 eV	1.09 eV
Br1F1Pb1_ICSD_155011	2.58 eV	2.75 eV
Br1F1Sr1_ICSD_159279	5.00 eV	4.79 eV
Br1F3Rb1Sb1_ICSD_200109	3.77 eV	3.55 eV
Br1F3_ICSD_39441	2.17 eV	2.74 eV
Br1F4K1_ICSD_16633	0.87 eV	3.72 eV
Br1F4K1_ICSD_16700	3.12 eV	3.92 eV
Br1F4N1O2_ICSD_412427	2.33 eV	2.52 eV
Br1F4Rb1_ICSD_65713	3.32 eV	3.28 eV
Br1F5O3Te1_ICSD_50200	2.77 eV	2.69 eV
Br1F5Sn3_ICSD_1383	3.19 eV	2.96 eV
Br1F5_ICSD_31690	3.47 eV	2.77 eV
Br1F6O2Sb1_ICSD_173608	1.98 eV	2.40 eV
Br1F8Sb1_ICSD_9886	2.74 eV	2.17 eV
Br1Fe1O3Sr2_ICSD_93509	0.85 eV	0.91 eV
Br1Gd1Mo1O4_ICSD_425266	1.84 eV	1.89 eV
Br1Gd1O1_ICSD_41071	4.34 eV	3.83 eV
Br1Gd3S8Si2_ICSD_411995	2.37 eV	2.25 eV
Br1H1Hg1O4_ICSD_29039	1.99 eV	2.20 eV
Br1H1K2O1_ICSD_78734	3.50 eV	3.35 eV
Br1H1O1Pb1_ICSD_404573	3.11 eV	2.79 eV
Br1H1O1Rb2_ICSD_78735	3.35 eV	3.37 eV
Br1H1O1Sr1_ICSD_407720	4.31 eV	3.93 eV
Br1H1Sr1_ICSD_25543	3.80 eV	3.53 eV
Br1H1_ICSD_28842	1.32 eV	1.78 eV
Br1H2Li1O1_ICSD_391154	4.40 eV	4.05 eV
Br1H2O6Rb1Se2Zn1_ICSD_409916	3.67 eV	3.64 eV

Supplementary Table 688. Five-fold cross validated predictions for the band gap energy (82/277).

system	calculated	predicted
Br1H3Li4O3_ICSD_412730	4.45 eV	4.33 eV
Br1H4N1O4_ICSD_165085	3.05 eV	3.14 eV
Br1H4N1_ICSD_27724	4.27 eV	4.00 eV
Br1H4N1_ICSD_43300	3.96 eV	3.94 eV
Br1H4Na1O2_ICSD_8125	4.26 eV	3.97 eV
Br1H4P1_ICSD_23691	4.03 eV	3.62 eV
Br1H5O2_ICSD_34105	4.36 eV	3.73 eV
Br1H6Li1O7_ICSD_73706	3.13 eV	3.67 eV
Br1H6Na1O5_ICSD_74654	2.17 eV	3.56 eV
Br1Hf1N1_ICSD_51773	1.89 eV	1.81 eV
Br1Hf1N1_ICSD_95720	2.12 eV	1.90 eV
Br1Hg1I1_ICSD_109010	2.26 eV	2.14 eV
Br1Hg2N1_ICSD_24706	0.70 eV	1.45 eV
Br1Hg2P3_ICSD_74770	0.93 eV	1.19 eV
Br1Hg3I1Te2_ICSD_99125	1.28 eV	1.31 eV
Br1Ho1Mo1O4_ICSD_425269	1.84 eV	2.06 eV
Br1In1O1_ICSD_24059	2.12 eV	2.22 eV
Br1In1O3Te1_ICSD_420301	3.03 eV	2.86 eV
Br1In1S4Sb2_ICSD_159467	1.54 eV	1.56 eV
Br1In1Sb2Se4_ICSD_159466	1.11 eV	1.12 eV
Br1In1Te1_ICSD_100705	1.81 eV	1.63 eV
Br1In1_ICSD_62239	1.26 eV	1.56 eV
Br1In5S5_ICSD_414219	1.15 eV	1.14 eV
Br1In5Se5_ICSD_414218	0.75 eV	0.84 eV
Br1K1O3_ICSD_74767	3.81 eV	3.71 eV
Br1K1O4_ICSD_15222	3.07 eV	3.37 eV
Br1K1_ICSD_290558	3.92 eV	3.96 eV
Br1K1_ICSD_52243	4.32 eV	4.20 eV
Br1K3Mo2O7_ICSD_2163	2.99 eV	3.03 eV
Br1K3O1_ICSD_33920	0.88 eV	1.05 eV
Br1La1O1_ICSD_24612	4.17 eV	4.06 eV
Br1La1O7Pb6_ICSD_249390	1.92 eV	1.97 eV
Br1La3S8Si2_ICSD_411996	2.77 eV	2.76 eV
Br1Li10N3_ICSD_78819	1.37 eV	1.72 eV
Br1Li1_ICSD_44274	4.93 eV	4.68 eV
Br1Li3O1_ICSD_67265	4.28 eV	3.09 eV
Br1Li6O5P1_ICSD_421480	5.10 eV	4.27 eV
Br1Lu1O1_ICSD_249338	4.33 eV	4.23 eV
Br1Lu1S1_ICSD_6082	2.91 eV	2.36 eV
Br1Mn1S2Sb1_ICSD_172782	1.37 eV	1.32 eV
Br1Mn1Sb1Se2_ICSD_172784	1.09 eV	1.08 eV
Br1Mo1Nd1O4_ICSD_425264	2.06 eV	2.02 eV
Br1Mo1O4Pr1_ICSD_425263	2.07 eV	1.96 eV
Br1Mo1O4Tb1_ICSD_425267	1.81 eV	2.00 eV
Br1Mo1S1_ICSD_163308	1.12 eV	0.99 eV
Br1Mo1Te4_ICSD_82245	0.85 eV	0.70 eV
Br1N1O3_ICSD_407765	2.24 eV	1.91 eV
Br1N1Sr2_ICSD_172600	1.85 eV	1.79 eV
Br1N1Ti1_ICSD_27395	1.20 eV	1.19 eV
Br1N1Zn2_ICSD_425736	2.12 eV	1.93 eV

Supplementary Table 689. Five-fold cross validated predictions for the band gap energy (83/277).

system	calculated	predicted
Br1N1Zr1_ICSD_25507	2.76 eV	1.57 eV
Br1N1Zr1_ICSD_27393	1.69 eV	1.97 eV
Br1N1Zr1_ICSD_87797	1.46 eV	1.50 eV
Br1Na1O3_ICSD_1302	4.47 eV	3.77 eV
Br1Na1_ICSD_53822	4.11 eV	3.90 eV
Br1Nd1O3Te1_ICSD_92319	0.12 eV	1.65 eV
Br1O12P3Sr5_ICSD_87102	4.95 eV	4.56 eV
Br1O1Pr1_ICSD_262128	3.68 eV	3.17 eV
Br1O1Rb3_ICSD_77196	0.41 eV	1.16 eV
Br1O1Sc1_ICSD_170774	3.77 eV	3.02 eV
Br1O1Ti1_ICSD_155650	2.05 eV	1.95 eV
Br1O1V1_ICSD_27010	1.15 eV	1.22 eV
Br1O1Y1_ICSD_0	4.41 eV	4.35 eV
Br1O3Rb1_ICSD_74768	3.97 eV	3.98 eV
Br1O3Tl1_ICSD_76966	3.54 eV	3.24 eV
Br1O4Tl1_ICSD_65660	3.02 eV	3.30 eV
Br1O4Y3_ICSD_419384	4.21 eV	3.87 eV
Br1Rb1_ICSD_18017	4.22 eV	4.06 eV
Br1Rb1_ICSD_61522	3.95 eV	4.12 eV
Br1S1Sb1_ICSD_40918	1.77 eV	1.27 eV
Br1S1Sb1_ICSD_88585	1.43 eV	1.67 eV
Br1S1Sb1_ICSD_88586	1.51 eV	1.18 eV
Br1S1Sb1_ICSD_88587	1.77 eV	1.81 eV
Br1Se2Tl5_ICSD_75960	0.32 eV	0.76 eV
Br1Te2_ICSD_426519	0.60 eV	0.93 eV
Br1Tl1_ICSD_109144	2.15 eV	2.24 eV
Br1Tl1_ICSD_61519	2.27 eV	2.21 eV
Br1Tl1_ICSD_61532	1.95 eV	1.85 eV
Br2C1Gd2_ICSD_47226	0.39 eV	0.68 eV
Br2C1O3Pb2_ICSD_29114	2.81 eV	2.91 eV
Br2C1Se4_ICSD_38387	1.50 eV	1.47 eV
Br2C2H6N2S1_ICSD_408001	2.58 eV	2.69 eV
Br2C2H6S1_ICSD_407035	2.48 eV	3.04 eV
Br2C4Cd1H8N8_ICSD_405833	3.38 eV	3.06 eV
Br2C4H11N1_ICSD_240878	3.94 eV	4.06 eV
Br2C4K2N4Pt1_ICSD_413705	2.00 eV	2.12 eV
Br2C4Mg2Na6O12_ICSD_27791	3.89 eV	3.77 eV
Br2C4N4Pt1Rb2_ICSD_852	2.08 eV	2.00 eV
Br2C6Ca1H24O6_ICSD_174396	4.90 eV	4.33 eV
Br2C6H18N4S4_ICSD_151023	3.38 eV	3.09 eV
Br2Ca1H12O6_ICSD_1141	4.13 eV	4.15 eV
Br2Ca1_ICSD_246715	4.45 eV	4.40 eV
Br2Ca1_ICSD_56766	4.49 eV	4.45 eV
Br2Ca3Si1_ICSD_89538	0.32 eV	1.58 eV
Br2Cd1Sb2Se3_ICSD_159464	1.13 eV	1.19 eV
Br2Cd1_ICSD_25782	2.89 eV	2.85 eV
Br2Cd1_ICSD_31536	2.93 eV	2.83 eV
Br2Cd1_ICSD_52367	2.86 eV	2.79 eV
Br2Cl2Cr1Cs1H18N6O8_ICSD_280499	1.42 eV	2.57 eV
Br2Cl2Cu1Rb2_ICSD_15147	1.01 eV	1.55 eV

Supplementary Table 690. Five-fold cross validated predictions for the band gap energy (84/277).

system	calculated	predicted
Br2Cl6I3Sb1_ICSD_26404	1.10 eV	1.33 eV
Br2Co1H12O14_ICSD_73397	2.48 eV	2.12 eV
Br2Co1H12O6_ICSD_40634	3.63 eV	3.36 eV
Br2Co1H4O2_ICSD_22085	2.89 eV	3.03 eV
Br2Co1O3Sb2_ICSD_418858	2.63 eV	2.09 eV
Br2Co1_ICSD_52364	2.76 eV	2.43 eV
Br2Co5O12Se4_ICSD_416966	3.18 eV	2.84 eV
Br2Cr1_ICSD_23903	1.67 eV	1.85 eV
Br2Cs1F1_ICSD_69124	1.74 eV	2.92 eV
Br2Cs1Li1_ICSD_40479	4.12 eV	4.33 eV
Br2Cu2O5Te2_ICSD_152960	1.00 eV	1.20 eV
Br2Cu2O6Sr2Te1_ICSD_172071	1.12 eV	1.24 eV
Br2Cu3O6Te2_ICSD_414443	0.25 eV	1.02 eV
Br2Eu1_ICSD_60316	3.15 eV	2.89 eV
Br2F10Ge1_ICSD_321	2.84 eV	2.84 eV
Br2F12Pb7_ICSD_92293	4.04 eV	3.85 eV
Br2Ga6Li8O24Si6_ICSD_87988	4.02 eV	3.95 eV
Br2Ga6Na8O24Si6_ICSD_417688	4.09 eV	3.97 eV
Br2Ge1_ICSD_100088	2.48 eV	2.60 eV
Br2H10N2O2_ICSD_61783	1.93 eV	2.88 eV
Br2H12Mg1O6_ICSD_189844	4.18 eV	4.14 eV
Br2H12Ni1O14_ICSD_65047	0.89 eV	2.12 eV
Br2H12Sr7_ICSD_418949	3.71 eV	3.29 eV
Br2H2O1Sr1_ICSD_76502	4.17 eV	4.31 eV
Br2H4Mn1O2_ICSD_22084	3.38 eV	3.13 eV
Br2H6O5U1_ICSD_170208	2.26 eV	2.78 eV
Br2Hg1K2N2O12_ICSD_24405	1.96 eV	2.61 eV
Br2Hg1_ICSD_151889	1.65 eV	1.83 eV
Br2Hg1_ICSD_39319	2.48 eV	2.30 eV
Br2Hg2O6_ICSD_31925	3.19 eV	2.24 eV
Br2Hg2Pb1S2_ICSD_85585	2.17 eV	1.83 eV
Br2Hg2S2Sn1_ICSD_82329	1.76 eV	1.85 eV
Br2Hg2S2Sn1_ICSD_85584	2.32 eV	1.81 eV
Br2Hg2_ICSD_23721	2.09 eV	1.84 eV
Br2Hg3S2_ICSD_82787	1.53 eV	1.51 eV
Br2Hg3Se2_ICSD_99092	1.32 eV	1.30 eV
Br2Hg3Te2_ICSD_27402	1.73 eV	1.34 eV
Br2K4O1_ICSD_68505	1.00 eV	1.61 eV
Br2La1S2Sb1_ICSD_93666	2.16 eV	1.87 eV
Br2La1_ICSD_65481	0.32 eV	0.80 eV
Br2Li5N1_ICSD_78836	2.09 eV	1.95 eV
Br2Mg1_ICSD_165973	4.48 eV	4.09 eV
Br2Mn1_ICSD_60250	3.38 eV	2.75 eV
Br2Mn1_ICSD_67500	3.39 eV	2.68 eV
Br2Mn1_ICSD_67517	3.41 eV	2.54 eV
Br2Mn2Sb2Se4_ICSD_172785	0.92 eV	0.95 eV
Br2Mo1O2_ICSD_422483	1.40 eV	1.44 eV
Br2N3S2V1_ICSD_61088	0.91 eV	1.35 eV
Br2Na4O1_ICSD_67283	2.04 eV	2.03 eV
Br2Nb1O1_ICSD_416669	0.91 eV	1.15 eV

Supplementary Table 691. Five-fold cross validated predictions for the band gap energy (85/277).

system	calculated	predicted
Br2Nb1Se2_ICSD_202821	1.11 eV	1.03 eV
Br2Nb1Se2_ICSD_202822	1.09 eV	1.11 eV
Br2Ni1_ICSD_22106	2.15 eV	1.78 eV
Br2Ni5O12Se4_ICSD_240325	2.72 eV	2.43 eV
Br2Ni5O12Te4_ICSD_96912	2.50 eV	2.17 eV
Br2O1Rb4_ICSD_411954	0.58 eV	1.46 eV
Br2O1S1_ICSD_62972	2.72 eV	2.16 eV
Br2O1V1_ICSD_24381	0.40 eV	1.64 eV
Br2O2Pb3_ICSD_82923	2.40 eV	2.54 eV
Br2O3_ICSD_78369	1.77 eV	2.31 eV
Br2O5Sb4_ICSD_24030	2.37 eV	2.53 eV
Br2O6Pb3Se2_ICSD_422640	2.91 eV	2.84 eV
Br2O6Pb3Te2_ICSD_248131	2.51 eV	2.51 eV
Br2O6Sr1_ICSD_61158	4.22 eV	3.79 eV
Br2Pd1_ICSD_27443	1.28 eV	1.38 eV
Br2Re6S8_ICSD_40701	1.09 eV	1.14 eV
Br2Re6Se8_ICSD_202824	0.83 eV	0.98 eV
Br2S2_ICSD_37020	2.13 eV	2.17 eV
Br2Se2_ICSD_37017	1.72 eV	1.51 eV
Br2Se2_ICSD_37019	1.52 eV	1.61 eV
Br2Sr1_ICSD_262673	4.31 eV	4.29 eV
Br2Ti1_ICSD_26078	2.52 eV	2.01 eV
Br2V1_ICSD_246906	2.63 eV	2.08 eV
Br2Yb1_ICSD_26045	3.05 eV	2.87 eV
Br2Zn1_ICSD_26080	3.53 eV	3.13 eV
Br2_ICSD_201693	1.32 eV	1.71 eV
Br3C1Cd1H6N3_ICSD_71850	3.32 eV	2.80 eV
Br3C1Eu2Li1N2_ICSD_415050	2.76 eV	2.51 eV
Br3C1F1_ICSD_72370	3.31 eV	3.54 eV
Br3C1Li1N2Sr2_ICSD_415051	3.98 eV	3.13 eV
Br3C2H8Hg1N1_ICSD_110527	2.53 eV	2.72 eV
Br3Ca1Cs1_ICSD_77242	4.40 eV	4.23 eV
Br3Ca1In1_ICSD_54138	3.12 eV	2.48 eV
Br3Cd1Cs1_ICSD_24483	1.07 eV	2.45 eV
Br3Cd1Cs1_ICSD_281176	3.00 eV	2.94 eV
Br3Cd1Cs1_ICSD_41453	3.00 eV	2.85 eV
Br3Cd1H4N1_ICSD_71849	2.62 eV	2.69 eV
Br3Cd1H5N2_ICSD_71851	2.92 eV	2.48 eV
Br3Cd1In1_ICSD_79512	2.19 eV	2.50 eV
Br3Cd1Rb1_ICSD_808	2.79 eV	2.85 eV
Br3Cd1Tl1_ICSD_39808	2.75 eV	2.66 eV
Br3Cd4P2_ICSD_75508	1.39 eV	1.46 eV
Br3Cr1In1_ICSD_54136	1.56 eV	1.66 eV
Br3Cr1_ICSD_24768	1.80 eV	1.76 eV
Br3Cs1Cu1_ICSD_10184	0.34 eV	1.39 eV
Br3Cs1Cu2_ICSD_49613	2.14 eV	2.01 eV
Br3Cs1Ge1_ICSD_80317	1.22 eV	1.61 eV
Br3Cs1Ge1_ICSD_80319	1.35 eV	1.93 eV
Br3Cs1Ge1_ICSD_80320	0.75 eV	1.31 eV
Br3Cs1Li2_ICSD_245981	4.30 eV	4.28 eV

Supplementary Table 692. Five-fold cross validated predictions for the band gap energy (86/277).

system	calculated	predicted
Br3Cs1Mg1_ICSD_87260	4.05 eV	3.81 eV
Br3Cs1Mn1_ICSD_2782	3.20 eV	3.03 eV
Br3Cs1Pb1_ICSD_109295	1.75 eV	1.79 eV
Br3Cs1Pb1_ICSD_29073	1.74 eV	1.70 eV
Br3Cs1Sn1_ICSD_4071	0.72 eV	1.22 eV
Br3Cs1V1_ICSD_201833	2.31 eV	2.11 eV
Br3Cs1Yb1_ICSD_77243	2.96 eV	3.03 eV
Br3Cu1K2_ICSD_150293	2.48 eV	2.53 eV
Br3Cu1Rb2_ICSD_150295	2.42 eV	2.44 eV
Br3Cu2Rb1_ICSD_150306	2.17 eV	1.93 eV
Br3F3N3P3_ICSD_16099	4.23 eV	3.60 eV
Br3F6S1Sb1_ICSD_66842	2.54 eV	2.24 eV
Br3Fe1In1_ICSD_75469	1.91 eV	2.04 eV
Br3Fe1K1_ICSD_4064	3.25 eV	2.55 eV
Br3Fe1Tl1_ICSD_36217	2.58 eV	2.23 eV
Br3Fe1_ICSD_410924	1.55 eV	2.04 eV
Br3Ga1Li1_ICSD_61338	2.99 eV	3.27 eV
Br3Ga1_ICSD_413456	3.27 eV	3.09 eV
Br3Gd1_ICSD_2610	3.78 eV	2.05 eV
Br3Gd2N1_ICSD_402278	3.03 eV	2.35 eV
Br3Gd2_ICSD_9581	0.17 eV	1.27 eV
Br3Gd5O10Se2_ICSD_419349	3.48 eV	3.49 eV
Br3Ge1Rb1_ICSD_65244	3.18 eV	2.71 eV
Br3Hg2Te1_ICSD_417407	1.89 eV	1.83 eV
Br3Hg3Sb2Tl1_ICSD_411521	0.64 eV	0.88 eV
Br3In1K1_ICSD_50510	2.32 eV	2.74 eV
Br3In1Mg1_ICSD_402533	2.02 eV	2.72 eV
Br3In1Mn1_ICSD_75470	1.95 eV	2.24 eV
Br3In1_ICSD_65198	2.57 eV	2.41 eV
Br3Ir1_ICSD_14212	2.00 eV	1.50 eV
Br3K1Mn1_ICSD_42441	3.34 eV	2.83 eV
Br3La1_ICSD_31581	1.35 eV	2.89 eV
Br3La1_ICSD_65479	3.57 eV	2.45 eV
Br3Li6N1_ICSD_16798	2.09 eV	2.29 eV
Br3Li6N1_ICSD_84092	1.34 eV	2.30 eV
Br3Mn1Rb1_ICSD_14077	3.08 eV	3.08 eV
Br3Mo1Te6_ICSD_82246	1.28 eV	0.97 eV
Br3N1Se2_ICSD_83696	1.24 eV	1.62 eV
Br3N3S4_ICSD_14049	1.72 eV	1.67 eV
Br3Nb1O1_ICSD_418089	2.03 eV	1.58 eV
Br3Ni1Rb1_ICSD_15011	2.16 eV	1.98 eV
Br3O1P1_ICSD_23243	3.06 eV	3.08 eV
Br3O1P1_ICSD_9137	3.08 eV	3.07 eV
Br3O1Pa1_ICSD_1892	1.75 eV	2.51 eV
Br3P1_ICSD_8052	3.28 eV	2.37 eV
Br3Pr1_ICSD_31583	1.61 eV	2.44 eV
Br3Pr1_ICSD_65077	2.80 eV	2.05 eV
Br3Rb1Ti1_ICSD_154257	1.51 eV	2.13 eV
Br3Rb1V1_ICSD_201831	2.26 eV	2.06 eV
Br3Rh1_ICSD_28245	1.80 eV	1.73 eV

Supplementary Table 693. Five-fold cross validated predictions for the band gap energy (87/277).

system	calculated	predicted
Br3Sb1_ICSD_14217	3.27 eV	2.48 eV
Br3Tc1_ICSD_260162	0.11 eV	0.89 eV
Br3Ti1_ICSD_39242	2.27 eV	2.55 eV
Br3U1_ICSD_4070	2.54 eV	2.27 eV
Br4C1N2S3_ICSD_32746	1.26 eV	1.97 eV
Br4C2H12Hg1N6_ICSD_260202	2.77 eV	2.90 eV
Br4C4H8Si1_ICSD_171154	4.18 eV	3.60 eV
Br4C6Cd1Hg4N6S6_ICSD_412498	1.74 eV	2.26 eV
Br4C6O6Ru2_ICSD_48158	2.29 eV	2.18 eV
Br4C8O8W2_ICSD_49689	1.90 eV	2.31 eV
Br4Cd1Cs2_ICSD_40416	3.39 eV	3.16 eV
Br4Cs1Ga1_ICSD_4037	3.36 eV	3.50 eV
Br4Cs1Tl1_ICSD_61180	1.95 eV	1.96 eV
Br4Cs2Hg1_ICSD_200910	2.70 eV	2.77 eV
Br4Cs2I2Pd1_ICSD_240482	0.27 eV	0.89 eV
Br4Cs2I2Pd1_ICSD_412833	0.83 eV	0.70 eV
Br4Cs2Mn1_ICSD_27251	3.60 eV	2.83 eV
Br4Cs2O2U1_ICSD_20130	2.31 eV	2.73 eV
Br4Cs2Re6Se8_ICSD_88611	1.48 eV	1.39 eV
Br4Cs2Zn1_ICSD_41537	4.00 eV	3.81 eV
Br4Cs3Li1_ICSD_245976	3.87 eV	3.90 eV
Br4Fe1Li2_ICSD_82200	3.00 eV	2.73 eV
Br4Fe1Tl1_ICSD_402064	1.86 eV	2.16 eV
Br4Ga1Li1_ICSD_61337	3.34 eV	3.32 eV
Br4Ga1Na1_ICSD_69650	3.49 eV	3.39 eV
Br4Ga2_ICSD_62665	2.95 eV	2.96 eV
Br4Ge1_ICSD_409856	2.95 eV	2.89 eV
Br4Ge1_ICSD_409857	3.00 eV	2.90 eV
Br4Ge4S6_ICSD_24370	2.44 eV	2.21 eV
Br4Hg3Te1_ICSD_82788	1.91 eV	1.70 eV
Br4I2Pd1Rb2_ICSD_412835	0.65 eV	0.79 eV
Br4In1K1_ICSD_409063	2.88 eV	2.64 eV
Br4In1Na1_ICSD_65462	2.83 eV	2.74 eV
Br4In2_ICSD_60851	2.34 eV	2.12 eV
Br4K1Tl1_ICSD_35418	1.78 eV	2.14 eV
Br4K2Pd1_ICSD_1982	1.61 eV	1.67 eV
Br4K2Pt1_ICSD_6063	1.98 eV	1.85 eV
Br4K2Zn1_ICSD_78449	3.91 eV	3.84 eV
Br4K2Zn1_ICSD_99702	3.89 eV	3.85 eV
Br4Li2Mg1_ICSD_73276	4.51 eV	3.98 eV
Br4Li2Mn1_ICSD_33865	3.67 eV	3.08 eV
Br4Li2Zn1_ICSD_73223	4.07 eV	3.82 eV
Br4Mn1Rb2_ICSD_8174	2.72 eV	2.53 eV
Br4Na2Zn1_ICSD_73224	3.97 eV	3.74 eV
Br4Nb1O1Tl1_ICSD_415203	1.41 eV	1.38 eV
Br4O1Rb6_ICSD_411955	1.53 eV	1.50 eV
Br4O1W1_ICSD_49547	1.23 eV	1.26 eV
Br4Os1Y4_ICSD_71513	0.67 eV	0.86 eV
Br4Os1_ICSD_61042	0.29 eV	0.93 eV
Br4Pd4Te3_ICSD_418003	0.94 eV	0.88 eV

Supplementary Table 694. Five-fold cross validated predictions for the band gap energy (88/277).

system	calculated	predicted
Br4Rb2Zn1_ICSD_63000	3.73 eV	3.84 eV
Br4Rb2Zn1_ICSD_77077	3.78 eV	3.79 eV
Br4Rb2Zn1_ICSD_77078	3.88 eV	3.69 eV
Br4Rb2Zn1_ICSD_97854	3.83 eV	3.85 eV
Br4S1Tl6_ICSD_40521	1.81 eV	1.61 eV
Br4S1W1_ICSD_16126	1.45 eV	1.42 eV
Br4Si1_ICSD_710060	4.10 eV	3.82 eV
Br4Si1_ICSD_710061	4.07 eV	3.87 eV
Br4Sn1_ICSD_26033	2.41 eV	2.35 eV
Br4Th1_ICSD_130013	3.01 eV	2.96 eV
Br4Th1_ICSD_6056	3.05 eV	2.88 eV
Br4Ti1_ICSD_22103	3.02 eV	2.72 eV
Br4Ti1_ICSD_39241	3.06 eV	2.77 eV
Br4Tl2_ICSD_14216	1.71 eV	2.07 eV
Br4U1_ICSD_2339	2.20 eV	2.06 eV
Br5Cd1Cs3_ICSD_39599	3.02 eV	3.02 eV
Br5Ce2_ICSD_167088	0.29 eV	0.53 eV
Br5Cs1Hg2_ICSD_200751	2.01 eV	2.15 eV
Br5Cs1Sn2_ICSD_151987	2.44 eV	2.31 eV
Br5Cs3Fe1_ICSD_4065	2.95 eV	2.50 eV
Br5Cs3Hg1_ICSD_200538	2.77 eV	2.81 eV
Br5In1Sn2_ICSD_152014	2.27 eV	2.24 eV
Br5K1Sn2_ICSD_152005	2.36 eV	2.39 eV
Br5K2Pr1_ICSD_48191	3.30 eV	3.17 eV
Br5K2U1_ICSD_72616	2.78 eV	2.97 eV
Br5La2_ICSD_65480	0.23 eV	0.87 eV
Br5Nb1_ICSD_409917	1.80 eV	1.38 eV
Br5Nb1_ICSD_67298	1.70 eV	1.64 eV
Br5Nd3S2_ICSD_60134	2.10 eV	2.19 eV
Br5P1_ICSD_22140	1.62 eV	2.23 eV
Br5Pa1_ICSD_22132	1.91 eV	2.92 eV
Br5Pb1Tl3_ICSD_15594	3.11 eV	2.90 eV
Br5Pb1Tl3_ICSD_200875	2.89 eV	2.99 eV
Br5Pb2Rb1_ICSD_151990	3.04 eV	2.50 eV
Br5Pr2_ICSD_62231	0.14 eV	1.35 eV
Br5Rb1Sn2_ICSD_151989	2.39 eV	2.35 eV
Br5Rb3Zn1_ICSD_41536	3.78 eV	3.78 eV
Br5Ru1Y4_ICSD_165336	0.24 eV	0.78 eV
Br5Sn2Tl1_ICSD_151988	2.36 eV	2.34 eV
Br5Ta1_ICSD_109324	2.18 eV	1.68 eV
Br5W1_ICSD_409227	0.62 eV	1.28 eV
Br6Ca1Th1_ICSD_78768	1.85 eV	2.29 eV
Br6Cd1Rb4_ICSD_60625	3.27 eV	3.25 eV
Br6Cs2Na1Y1_ICSD_65733	4.20 eV	3.54 eV
Br6Cs2Pt1_ICSD_77381	1.36 eV	1.17 eV
Br6Cs2Sn1_ICSD_158957	1.24 eV	1.59 eV
Br6Cs2Te1_ICSD_24151	2.16 eV	1.99 eV
Br6Cs2Te1_ICSD_65058	2.16 eV	1.99 eV
Br6Cs4Pb1_ICSD_25124	3.88 eV	3.53 eV
Br6Dy1Li1Rb2_ICSD_402536	4.19 eV	3.33 eV

Supplementary Table 695. Five-fold cross validated predictions for the band gap energy (89/277).

system	calculated	predicted
Br6Eu4O1_ICSD_65172	2.64 eV	2.57 eV
Br6Gd1Na3_ICSD_82354	1.11 eV	2.95 eV
Br6H12Mg1O6Zn2_ICSD_49914	3.91 eV	3.55 eV
Br6H12N4Sc2_ICSD_281573	3.47 eV	3.30 eV
Br6H12O6Zn3_ICSD_64614	3.90 eV	3.64 eV
Br6H16Hg1N4_ICSD_391363	1.99 eV	2.09 eV
Br6H16Hg1N4_ICSD_391364	2.10 eV	2.01 eV
Br6H6N2Sc2_ICSD_281574	3.55 eV	3.33 eV
Br6H8N2Re1_ICSD_412159	2.07 eV	2.22 eV
Br6Hg1Tl4_ICSD_9325	1.76 eV	1.92 eV
Br6Hg3Se2Zr1_ICSD_412467	1.37 eV	1.54 eV
Br6Hg7P4Sn1_ICSD_411860	1.66 eV	1.37 eV
Br6Hg7P4_ICSD_73818	1.32 eV	1.35 eV
Br6In2Th1_ICSD_80180	2.86 eV	2.04 eV
Br6In2Zr1_ICSD_54137	2.17 eV	2.18 eV
Br6K2Pt1_ICSD_23771	1.10 eV	1.30 eV
Br6K2Re1_ICSD_26623	2.12 eV	1.59 eV
Br6K2Se1_ICSD_36228	1.69 eV	2.30 eV
Br6K2Sn1_ICSD_158955	1.04 eV	1.75 eV
Br6K2Te1_ICSD_37270	2.24 eV	2.09 eV
Br6K2Te1_ICSD_65118	1.97 eV	2.03 eV
Br6Mo6S3_ICSD_35356	1.29 eV	1.05 eV
Br6N3P3_ICSD_27271	3.04 eV	2.86 eV
Br6Na3Sc1_ICSD_401335	3.79 eV	3.89 eV
Br6Na3Y1_ICSD_82355	4.26 eV	3.57 eV
Br6Nb2Se2_ICSD_35375	2.36 eV	1.78 eV
Br6Nb2Te2_ICSD_35376	1.87 eV	1.81 eV
Br6O1Sr4_ICSD_418452	4.23 eV	4.21 eV
Br6Pb1Rb4_ICSD_65300	3.90 eV	3.43 eV
Br6Pb2Pd1_ICSD_78874	1.73 eV	1.73 eV
Br6Pb4Se1_ICSD_21039	1.88 eV	2.28 eV
Br6Pd1Rb2_ICSD_36305	0.54 eV	0.92 eV
Br6Pd1Se2_ICSD_405774	1.46 eV	1.41 eV
Br6Rb2Sn1_ICSD_158956	1.14 eV	1.40 eV
Br6Rb2Te1_ICSD_49520	2.09 eV	1.98 eV
Br6Rb2Te1_ICSD_49521	2.06 eV	1.96 eV
Br6Rb2U1_ICSD_82949	3.02 eV	2.98 eV
Br6Sr1Th1_ICSD_78769	2.02 eV	2.46 eV
Br6Te1Tl2_ICSD_99127	2.16 eV	2.18 eV
Br6W1_ICSD_62048	1.14 eV	1.48 eV
Br7Fe1Se1_ICSD_39528	0.32 eV	1.48 eV
Br7Fe1Te1_ICSD_39531	1.93 eV	1.61 eV
Br7Ga1Se1_ICSD_78913	2.23 eV	2.13 eV
Br7Hg6In1Sb4_ICSD_420139	1.08 eV	1.19 eV
Br7Hg6Sb5_ICSD_411219	0.96 eV	0.92 eV
Br7In4_ICSD_401451	2.27 eV	2.28 eV
Br7Nb3S1_ICSD_81078	0.92 eV	1.08 eV
Br7P1_ICSD_26025	1.25 eV	1.88 eV
Br7S1Ta3_ICSD_51101	0.95 eV	1.13 eV
Br8Cs2Re2_ICSD_26060	0.48 eV	1.51 eV

Supplementary Table 696. Five-fold cross validated predictions for the band gap energy (90/277).

system	calculated	predicted
Br8Ga2Pd1_ICSD_413229	1.82 eV	1.95 eV
Br8Ge38P8_ICSD_22031	1.41 eV	1.10 eV
Br8Hg2Pd1Rb2_ICSD_203188	1.96 eV	1.49 eV
Br8Li6Mg1_ICSD_73275	4.68 eV	4.32 eV
Br8N4P4_ICSD_2716	2.49 eV	2.32 eV
Br8Nb3_ICSD_25766	0.39 eV	0.77 eV
Br8S9Ta4_ICSD_171236	1.35 eV	1.33 eV
Br9Cs3Er2_ICSD_203114	4.23 eV	4.01 eV
Br9Cs3Ho2_ICSD_72798	4.22 eV	4.17 eV
Br9Cs3Mo2_ICSD_26213	1.27 eV	1.35 eV
Br9Cs3Tb2_ICSD_203109	4.13 eV	3.65 eV
Br9Cs3Y2_ICSD_37221	4.03 eV	4.06 eV
Br9Cs5Nb2S4_ICSD_410590	1.89 eV	1.86 eV
Br9In7_ICSD_78804	1.52 eV	2.00 eV
Br9K3Mo2_ICSD_202299	1.08 eV	1.39 eV
Br9Mo2Rb3_ICSD_202293	1.23 eV	1.19 eV
Br9Nb2Rb3_ICSD_402032	0.51 eV	1.08 eV
Br9Nb2S4Tl5_ICSD_418796	1.84 eV	1.63 eV
Br9O4Pb8Tl1_ICSD_36007	2.89 eV	2.69 eV
Br9Os2Rb3_ICSD_56896	1.08 eV	1.11 eV
Br9Rb3Sb2_ICSD_39823	1.91 eV	2.04 eV
Br9Rb3V2_ICSD_61235	1.15 eV	1.77 eV
Br9Ta1Te1_ICSD_410949	2.28 eV	1.58 eV
Br9Te1W1_ICSD_410948	0.51 eV	1.29 eV
C10Cl1Co3O9_ICSD_200825	2.17 eV	2.65 eV
C10Cs1_ICSD_657337	0.75 eV	1.04 eV
C10Cu2H18N2O10_ICSD_171405	0.50 eV	1.49 eV
C10H14N8Ni1O2_ICSD_109784	1.31 eV	2.43 eV
C10H18N2O10Rh2_ICSD_172424	2.93 eV	2.42 eV
C10Hg1Mn2O10_ICSD_71879	2.70 eV	2.72 eV
C10O10Os3S1_ICSD_201861	2.79 eV	2.57 eV
C11N4_ICSD_184896	2.42 eV	2.85 eV
C12Cl12N12O4P4S12Sb4_ICSD_80097	3.31 eV	2.69 eV
C12Co2N12Zn3_ICSD_157849	5.19 eV	4.13 eV
C12F10Se1_ICSD_410720	2.77 eV	3.43 eV
C12H36I4Ni1O6S6_ICSD_170024	0.66 eV	1.91 eV
C12Mn2O12Pt1_ICSD_41117	1.75 eV	2.25 eV
C12N6_ICSD_30815	3.12 eV	2.96 eV
C12Ni4O18P4_ICSD_14337	3.43 eV	3.14 eV
C12O12Pt1Re2_ICSD_60074	1.80 eV	2.39 eV
C12O12Ru4Se4_ICSD_92913	2.19 eV	2.18 eV
C14Cl12H24Mn1N8Nb6_ICSD_110055	1.04 eV	2.49 eV
C14H24K1Mn1N8_ICSD_107665	1.11 eV	2.52 eV
C16Co4O16Sn1_ICSD_67320	2.12 eV	2.70 eV
C16Cr2H24O8Sb4_ICSD_241130	2.26 eV	2.35 eV
C1Ca1F1K1O3_ICSD_154682	4.35 eV	4.53 eV
C1Ca1F1O3Rb1_ICSD_262232	4.32 eV	4.21 eV
C1Ca1H12O9_ICSD_151488	4.91 eV	4.75 eV
C1Ca1H2O4_ICSD_100847	5.23 eV	4.58 eV
C1Ca1N2_ICSD_26677	3.65 eV	3.32 eV

Supplementary Table 697. Five-fold cross validated predictions for the band gap energy (91/277).

system	calculated	predicted
C1Ca1O3_ICSD_150	5.00 eV	4.79 eV
C1Ca1O3_ICSD_161820	5.73 eV	4.86 eV
C1Ca1O3_ICSD_169933	5.00 eV	4.50 eV
C1Ca1O3_ICSD_181959	4.63 eV	4.73 eV
C1Ca1O3_ICSD_280991	4.23 eV	4.69 eV
C1Ca1O3_ICSD_52152	4.17 eV	4.58 eV
C1Ca1O3_ICSD_83607	4.96 eV	4.87 eV
C1Ca2F2O3_ICSD_100607	5.17 eV	5.16 eV
C1Ca4N4_ICSD_167787	1.81 eV	1.71 eV
C1Ca7H4O23Si6_ICSD_73333	4.55 eV	4.61 eV
C1Cd1Cl2H4N2O1_ICSD_39960	3.60 eV	3.26 eV
C1Cd1N2_ICSD_95264	2.40 eV	1.90 eV
C1Cd1N2_ICSD_95265	0.73 eV	2.06 eV
C1Cd1O3_ICSD_20181	2.78 eV	2.54 eV
C1Cd3H2O8Se1_ICSD_95816	2.89 eV	2.89 eV
C1Ce1F1O3_ICSD_81673	4.52 eV	4.29 eV
C1Ce1H2O6P2_ICSD_262898	1.87 eV	3.27 eV
C1Cl14Ti6_ICSD_75502	1.20 eV	1.88 eV
C1Cl14Zr6_ICSD_202915	1.28 eV	1.09 eV
C1Cl18Rb4Zr6_ICSD_165401	1.27 eV	1.62 eV
C1Cl18W6_ICSD_413026	1.09 eV	0.97 eV
C1Cl1Co1H18N6O3_ICSD_412059	2.43 eV	3.14 eV
C1Cl1Cu1H2N2_ICSD_247128	2.12 eV	2.35 eV
C1Cl1Cu1O1_ICSD_63490	2.58 eV	2.20 eV
C1Cl1F3O3S1_ICSD_96950	4.60 eV	4.60 eV
C1Cl1F3_ICSD_49696	6.57 eV	6.01 eV
C1Cl1F4I1_ICSD_280343	2.70 eV	3.28 eV
C1Cl1H3O1S1_ICSD_107497	2.64 eV	3.43 eV
C1Cl1H4N5_ICSD_2762	3.38 eV	3.94 eV
C1Cl1H5N2O4S1_ICSD_39575	3.03 eV	3.61 eV
C1Cl1H5N2S1_ICSD_250292	4.34 eV	3.35 eV
C1Cl1H6N1O1_ICSD_241238	5.33 eV	4.39 eV
C1Cl1H6N3O1_ICSD_62660	5.20 eV	4.66 eV
C1Cl1Hg1N1S1_ICSD_409579	2.44 eV	2.43 eV
C1Cl1La1N2_ICSD_413904	3.39 eV	3.18 eV
C1Cl1La2N3_ICSD_412412	1.67 eV	2.44 eV
C1Cl1N1O2S2_ICSD_91453	2.71 eV	2.65 eV
C1Cl1N1Pb1S1_ICSD_409835	3.11 eV	2.63 eV
C1Cl1N1_ICSD_16660	5.79 eV	4.47 eV
C1Cl1N5S3_ICSD_62270	2.29 eV	2.17 eV
C1Cl2Eu2N2_ICSD_391256	2.79 eV	2.21 eV
C1Cl2F2_ICSD_33947	4.73 eV	4.79 eV
C1Cl2F2_ICSD_33948	5.52 eV	4.69 eV
C1Cl2Gd2_ICSD_400348	0.44 eV	0.86 eV
C1Cl2H3N1O2S1_ICSD_151036	2.64 eV	3.37 eV
C1Cl2Hg2N2_ICSD_412608	1.85 eV	2.03 eV
C1Cl2Lu2_ICSD_62227	0.13 eV	1.41 eV
C1Cl2N2Sr2_ICSD_391257	4.19 eV	3.30 eV
C1Cl2O3Pb2_ICSD_36241	3.22 eV	3.10 eV
C1Cl2Sc2_ICSD_59124	1.10 eV	1.77 eV

Supplementary Table 698. Five-fold cross validated predictions for the band gap energy (92/277).

system	calculated	predicted
C1Cl3F1_ICSD_80891	4.93 eV	4.54 eV
C1Cl3F3Si1_ICSD_173735	6.16 eV	5.10 eV
C1Cl3H3Te1_ICSD_281531	2.54 eV	2.31 eV
C1Cl4H5N1Si2_ICSD_410261	4.97 eV	4.06 eV
C1Cl5H6Hg2N3_ICSD_59241	2.56 eV	2.48 eV
C1Cl6H2O4Pb4_ICSD_88936	3.58 eV	2.88 eV
C1Cl6N1Sb1_ICSD_279639	1.94 eV	2.06 eV
C1Co1O3_ICSD_61066	3.10 eV	2.68 eV
C1Cs1F3O1_ICSD_69971	6.10 eV	5.53 eV
C1Cs1F3O3S1_ICSD_415056	5.61 eV	5.20 eV
C1Cs1H1N2O2_ICSD_170588	1.03 eV	2.25 eV
C1Cs1H1O2_ICSD_172202	4.41 eV	4.16 eV
C1Cs1H1O3_ICSD_300259	4.32 eV	3.67 eV
C1Cs1N1S1_ICSD_60875	4.33 eV	3.35 eV
C1Cs2H6O6_ICSD_411684	4.07 eV	3.81 eV
C1Cs2I18Zr7_ICSD_72606	0.16 eV	0.42 eV
C1Cs2O3_ICSD_14156	3.34 eV	3.42 eV
C1Cs4O4_ICSD_245445	1.64 eV	1.30 eV
C1Cs4O4_ICSD_245446	1.20 eV	1.30 eV
C1Cs4O4_ICSD_245447	0.82 eV	1.33 eV
C1Cs4O4_ICSD_245449	0.41 eV	0.97 eV
C1Cs4O4_ICSD_245450	1.32 eV	1.35 eV
C1Cs4O4_ICSD_245451	1.55 eV	1.04 eV
C1Cs4O4_ICSD_245453	0.51 eV	1.20 eV
C1Cu1F1K1O3_ICSD_75401	0.52 eV	1.37 eV
C1Cu1H1O4Tl1_ICSD_74875	1.58 eV	1.71 eV
C1Cu1H1O5S1_ICSD_281184	3.63 eV	2.30 eV
C1Cu1H8N2O5P2_ICSD_279591	1.87 eV	1.57 eV
C1Cu1N1S1_ICSD_124	2.75 eV	2.56 eV
C1Cu1N1S1_ICSD_24372	2.59 eV	2.31 eV
C1Cu1N1S1_ICSD_32578	2.43 eV	2.38 eV
C1Cu1O3_ICSD_6179	0.73 eV	1.03 eV
C1Dy1H1O4_ICSD_409351	5.22 eV	4.70 eV
C1Dy2N2O2_ICSD_416881	3.98 eV	3.79 eV
C1Er2N2O2_ICSD_416880	4.01 eV	3.79 eV
C1Eu2I3Li1N2_ICSD_170261	3.03 eV	2.39 eV
C1F1H3O2_ICSD_170925	5.45 eV	5.28 eV
C1F1H6N1_ICSD_110656	6.04 eV	5.84 eV
C1F1H7N3O3P1_ICSD_151288	5.08 eV	5.10 eV
C1F1K1O3Sr1_ICSD_262231	4.07 eV	4.24 eV
C1F1K3O3_ICSD_66028	3.86 eV	3.88 eV
C1F1La1O3_ICSD_26678	4.68 eV	4.59 eV
C1F1N1S1Sn1_ICSD_2418	2.88 eV	2.74 eV
C1F1O3Rb1Sr1_ICSD_262233	4.14 eV	4.29 eV
C1F1O3Rb3_ICSD_66029	3.47 eV	3.52 eV
C1F1O3Tl3_ICSD_2515	2.40 eV	2.57 eV
C1F2Gd2_ICSD_33912	0.77 eV	1.65 eV
C1F2Ho2_ICSD_71626	1.13 eV	2.01 eV
C1F2K2O6S2_ICSD_92475	5.65 eV	5.25 eV
C1F2N1O2P1_ICSD_248121	5.90 eV	4.55 eV

Supplementary Table 699. Five-fold cross validated predictions for the band gap energy (93/277).

system	calculated	predicted
C1F2Na1O3Yb1_ICSD_172520	0.43 eV	1.97 eV
C1F2O3Pb2_ICSD_35413	3.47 eV	3.47 eV
C1F3H1O3S1_ICSD_65782	6.50 eV	6.01 eV
C1F3H2Na1O4S1_ICSD_151032	5.75 eV	5.54 eV
C1F3H3O4S1_ICSD_2615	6.38 eV	5.87 eV
C1F3H5O5S1_ICSD_2007	6.19 eV	5.64 eV
C1F3Hg1O3S1_ICSD_98942	3.29 eV	3.15 eV
C1F3I1N2O6_ICSD_78758	3.33 eV	2.78 eV
C1F3I1_ICSD_73268	3.71 eV	3.55 eV
C1F3Li1O3S1_ICSD_110018	6.53 eV	5.91 eV
C1F3O3Rb1S1_ICSD_415817	5.65 eV	5.21 eV
C1F4H6N3Sb1_ICSD_39318	4.61 eV	3.95 eV
C1F4_ICSD_66659	8.91 eV	6.83 eV
C1F5H7N4Zr1_ICSD_281003	4.30 eV	4.74 eV
C1F5N1S1_ICSD_413757	4.27 eV	4.87 eV
C1F5N1S2_ICSD_413754	2.95 eV	3.92 eV
C1F6H6N3P1_ICSD_203170	5.18 eV	5.34 eV
C1F7I1_ICSD_401706	4.65 eV	3.30 eV
C1Fe1H5O4P1_ICSD_153033	3.38 eV	2.81 eV
C1Fe1Na3O7P1_ICSD_77053	3.24 eV	3.02 eV
C1Fe1O3_ICSD_169791	0.17 eV	2.47 eV
C1Fe1O3_ICSD_169796	3.16 eV	1.37 eV
C1Ga1N1Si1_ICSD_183047	0.58 eV	1.87 eV
C1Ga1N5Sr4_ICSD_171166	1.45 eV	1.70 eV
C1Ga3N3Si1_ICSD_183050	2.57 eV	2.11 eV
C1Gd1H5O7P1_ICSD_161193	3.69 eV	4.31 eV
C1Ge1H3N1O1_ICSD_201197	4.99 eV	4.05 eV
C1Ge1_ICSD_182363	1.65 eV	1.22 eV
C1H1K1N2O2_ICSD_170586	0.83 eV	2.21 eV
C1H1K1N2_ICSD_401784	3.88 eV	3.88 eV
C1H1K1O2_ICSD_151294	4.32 eV	4.27 eV
C1H1K1O3_ICSD_157166	5.02 eV	3.75 eV
C1H1K1O3_ICSD_43015	0.15 eV	2.67 eV
C1H1Li1O2_ICSD_151274	5.03 eV	4.50 eV
C1H1N1_ICSD_187644	3.64 eV	4.82 eV
C1H1N1_ICSD_76418	6.78 eV	4.78 eV
C1H1N1_ICSD_76419	6.60 eV	4.03 eV
C1H1N2Na1_ICSD_2495	4.04 eV	3.56 eV
C1H1Na1O2_ICSD_109643	2.35 eV	4.22 eV
C1H1Na1O2_ICSD_151256	4.83 eV	3.45 eV
C1H1Na1O3_ICSD_18183	5.24 eV	4.36 eV
C1H1Na1O3_ICSD_26933	4.87 eV	4.42 eV
C1H1O2Rb1_ICSD_172203	4.43 eV	4.31 eV
C1H1O2Tl1_ICSD_110364	3.52 eV	3.48 eV
C1H1O2Tl1_ICSD_151295	3.50 eV	3.54 eV
C1H1O4Y1_ICSD_644	5.09 eV	4.59 eV
C1H1_ICSD_187642	4.95 eV	4.53 eV
C1H2N2_ICSD_40446	4.54 eV	4.71 eV
C1H2Na2O4_ICSD_15959	4.18 eV	4.00 eV
C1H2O2_ICSD_151248	4.84 eV	4.79 eV

Supplementary Table 700. Five-fold cross validated predictions for the band gap energy (94/277).

system	calculated	predicted
C1H3Hg1N3_ICSD_761194	3.64 eV	3.05 eV
C1H3K1N2O1S1_ICSD_16412	1.10 eV	2.93 eV
C1H3Li1O3_ICSD_151231	5.25 eV	4.83 eV
C1H3N1O1Si1_ICSD_201196	5.63 eV	5.14 eV
C1H3N1O3Zn1_ICSD_41113	0.15 eV	2.59 eV
C1H3N5O4_ICSD_281338	2.91 eV	2.72 eV
C1H4Ho2O7_ICSD_62030	5.00 eV	4.56 eV
C1H4N1Na1O2_ICSD_830	5.84 eV	4.00 eV
C1H4N2O1_ICSD_151523	4.72 eV	4.97 eV
C1H4N2O1_ICSD_245371	4.95 eV	4.56 eV
C1H4N2O2S1_ICSD_165279	2.95 eV	4.11 eV
C1H4N2S1_ICSD_69145	3.39 eV	4.06 eV
C1H4N2S1_ICSD_956	4.33 eV	3.43 eV
C1H4N3Rb1_ICSD_422591	3.63 eV	3.64 eV
C1H4Na4O9P2_ICSD_19	2.13 eV	3.95 eV
C1H5N1O2_ICSD_151222	5.11 eV	5.20 eV
C1H5N1O3_ICSD_158914	5.16 eV	4.80 eV
C1H5N3O4_ICSD_34697	3.52 eV	3.16 eV
C1H6N2O2_ICSD_172855	4.81 eV	4.79 eV
C1H6N2S2_ICSD_421191	2.71 eV	2.99 eV
C1H6N2S2_ICSD_421192	2.53 eV	2.67 eV
C1H6N4O1_ICSD_2812	4.97 eV	4.24 eV
C1H6N4S1_ICSD_42827	3.42 eV	3.64 eV
C1H7N3O3_ICSD_163085	3.38 eV	3.27 eV
C1H9N4O4P1_ICSD_163005	5.00 eV	4.97 eV
C1H9N7O3_ICSD_8100	3.16 eV	3.15 eV
C1Hf1_ICSD_185992	0.30 eV	0.58 eV
C1Hg1N2_ICSD_411067	2.45 eV	2.48 eV
C1Hg1N2_ICSD_412278	1.92 eV	2.68 eV
C1Ho2N2O2_ICSD_416882	4.00 eV	3.96 eV
C1Ho2N6Si4_ICSD_94032	3.21 eV	2.84 eV
C1I12Zr6_ICSD_60918	0.28 eV	0.27 eV
C1I1N1_ICSD_77911	4.33 eV	3.33 eV
C1I4_ICSD_30789	1.06 eV	1.80 eV
C1K1Li1O3_ICSD_84638	4.59 eV	4.06 eV
C1K1N1S1_ICSD_65075	4.11 eV	3.45 eV
C1K1N1_ICSD_173942	5.22 eV	4.02 eV
C1K1N1_ICSD_27351	4.93 eV	4.61 eV
C1K1N3O2_ICSD_408287	3.33 eV	2.43 eV
C1K1N3O6_ICSD_20483	2.12 eV	2.40 eV
C1K1O5Pu1_ICSD_15685	0.24 eV	2.55 eV
C1K2N2_ICSD_423580	3.22 eV	3.24 eV
C1K2O3_ICSD_10191	3.79 eV	3.28 eV
C1K2O3_ICSD_52535	3.27 eV	3.54 eV
C1K2O3_ICSD_662	3.62 eV	3.50 eV
C1K3O8V1_ICSD_60813	2.37 eV	2.73 eV
C1K4O4_ICSD_245417	2.30 eV	2.20 eV
C1K4O4_ICSD_245418	1.95 eV	2.00 eV
C1K4O4_ICSD_245419	1.44 eV	1.99 eV
C1K4O4_ICSD_245422	1.57 eV	1.87 eV

Supplementary Table 701. Five-fold cross validated predictions for the band gap energy (95/277).

system	calculated	predicted
C1K4O4_ICSD_245424	2.34 eV	1.98 eV
C1K4O4_ICSD_245425	1.78 eV	1.88 eV
C1K4O4_ICSD_245427	2.09 eV	2.14 eV
C1K4O4_ICSD_245428	3.01 eV	2.20 eV
C1La2N2O4Si1_ICSD_420943	4.20 eV	3.58 eV
C1La2O5_ICSD_51468	3.95 eV	3.82 eV
C1Li1N1_ICSD_77321	5.49 eV	4.11 eV
C1Li1Na1O3_ICSD_89650	4.44 eV	3.75 eV
C1Li2N2_ICSD_200369	3.72 eV	4.09 eV
C1Li2O3_ICSD_66941	5.04 eV	4.25 eV
C1Li2O3_ICSD_96486	3.75 eV	4.52 eV
C1Li4O4_ICSD_245388	4.17 eV	4.70 eV
C1Li4O4_ICSD_245389	3.89 eV	4.18 eV
C1Li4O4_ICSD_245390	4.69 eV	4.29 eV
C1Li4O4_ICSD_245395	2.99 eV	3.99 eV
C1Li4O4_ICSD_245396	3.98 eV	4.47 eV
C1Li4O4_ICSD_245398	4.00 eV	4.31 eV
C1Li4O4_ICSD_245401	6.11 eV	5.13 eV
C1Li4O4_ICSD_245402	5.30 eV	5.18 eV
C1Li4O4_ICSD_245403	5.40 eV	5.16 eV
C1Li4O4_ICSD_245405	4.93 eV	4.71 eV
C1Mg1N2_ICSD_44110	3.85 eV	3.80 eV
C1Mg1N2_ICSD_75039	3.76 eV	3.89 eV
C1Mg1O3_ICSD_161821	5.13 eV	4.64 eV
C1Mg1O3_ICSD_52150	4.96 eV	5.00 eV
C1Mg1O3_ICSD_63663	4.96 eV	4.95 eV
C1Mn1N2_ICSD_170135	2.01 eV	1.92 eV
C1Mn1Na3O7P1_ICSD_200789	3.32 eV	3.08 eV
C1Mn1O3_ICSD_28556	3.46 eV	2.06 eV
C1N1Na1O1_ICSD_27138	4.46 eV	3.85 eV
C1N1Na1S1_ICSD_22273	4.06 eV	3.33 eV
C1N1Na1_ICSD_77172	5.04 eV	3.68 eV
C1N1Th1_ICSD_2785	1.23 eV	1.26 eV
C1N2Na2_ICSD_411341	3.05 eV	3.39 eV
C1N2Nd2O2_ICSD_82265	3.36 eV	3.23 eV
C1N2Ni1_ICSD_249388	1.19 eV	1.92 eV
C1N2O2Pr2_ICSD_82264	3.44 eV	3.04 eV
C1N2O2Tm2_ICSD_416883	1.04 eV	2.32 eV
C1N2O2Y2_ICSD_245332	3.97 eV	3.90 eV
C1N2O2Y2_ICSD_245333	3.94 eV	3.82 eV
C1N2Pb1_ICSD_16600	1.73 eV	1.72 eV
C1N2Pb1_ICSD_410915	1.70 eV	1.82 eV
C1N2Sr1_ICSD_182046	3.62 eV	3.37 eV
C1N2Sr1_ICSD_59860	3.20 eV	3.34 eV
C1N2Tl2_ICSD_417297	1.89 eV	2.07 eV
C1N2Zn1_ICSD_280523	3.85 eV	4.16 eV
C1N2_ICSD_247676	4.63 eV	2.55 eV
C1N2_ICSD_247677	3.57 eV	3.65 eV
C1N2_ICSD_247678	0.17 eV	3.17 eV
C1N2_ICSD_247679	2.33 eV	3.83 eV

Supplementary Table 702. Five-fold cross validated predictions for the band gap energy (96/277).

system	calculated	predicted
C1N2_ICSD_247680	1.07 eV	3.51 eV
C1N4S3_ICSD_62269	1.92 eV	1.90 eV
C1N4Si2_ICSD_93544	4.36 eV	3.97 eV
C1N4Sr4_ICSD_170798	1.22 eV	1.50 eV
C1N6O1_ICSD_174516	3.60 eV	3.78 eV
C1N6Si4Y2_ICSD_155158	3.11 eV	2.68 eV
C1Na2O3_ICSD_81013	3.22 eV	3.67 eV
C1Na2O3_ICSD_95549	3.87 eV	3.52 eV
C1Na2S3_ICSD_73094	1.18 eV	2.31 eV
C1Na3O7P1Sr1_ICSD_39559	3.87 eV	4.29 eV
C1Na4O4_ICSD_245406	2.03 eV	2.49 eV
C1Na4O4_ICSD_245407	1.59 eV	2.30 eV
C1Na4O4_ICSD_245408	2.46 eV	2.25 eV
C1Na4O4_ICSD_245410	1.69 eV	2.21 eV
C1Na4O4_ICSD_245411	2.19 eV	2.47 eV
C1Na4O4_ICSD_245412	1.97 eV	2.19 eV
C1Na4O4_ICSD_245414	3.19 eV	2.25 eV
C1Na4O4_ICSD_245416	2.23 eV	2.34 eV
C1Ni1O3_ICSD_61067	3.02 eV	2.60 eV
C1O1S1_ICSD_33540	4.82 eV	4.05 eV
C1O1_ICSD_26962	5.89 eV	5.51 eV
C1O1_ICSD_40937	6.02 eV	5.51 eV
C1O2_ICSD_110775	6.46 eV	6.46 eV
C1O2_ICSD_110776	6.43 eV	6.51 eV
C1O2_ICSD_166852	6.47 eV	5.33 eV
C1O2_ICSD_188892	6.70 eV	6.33 eV
C1O2_ICSD_188893	6.46 eV	6.50 eV
C1O2_ICSD_290503	7.61 eV	6.01 eV
C1O2_ICSD_59378	6.63 eV	6.46 eV
C1O2_ICSD_98406	6.43 eV	6.50 eV
C1O2_ICSD_98407	6.60 eV	6.44 eV
C1O3Pb1_ICSD_247487	3.38 eV	3.22 eV
C1O3Rb2_ICSD_414122	3.27 eV	3.39 eV
C1O3Rb2_ICSD_414123	3.44 eV	3.32 eV
C1O3Sr1_ICSD_27293	4.44 eV	4.36 eV
C1O3Tl2_ICSD_4239	2.95 eV	2.51 eV
C1O3Zn1_ICSD_100679	4.09 eV	3.88 eV
C1O4Pb2_ICSD_91714	3.43 eV	2.99 eV
C1O4Rb4_ICSD_245429	1.79 eV	1.12 eV
C1O4Rb4_ICSD_245430	0.48 eV	1.19 eV
C1O4Rb4_ICSD_245431	0.94 eV	1.25 eV
C1O4Rb4_ICSD_245433	0.47 eV	1.13 eV
C1O4Rb4_ICSD_245437	1.78 eV	1.16 eV
C1O4Rb4_ICSD_245439	1.33 eV	1.26 eV
C1O4Rb4_ICSD_245440	0.69 eV	1.41 eV
C1O4Rb4_ICSD_245442	1.26 eV	1.34 eV
C1O4Rb4_ICSD_245444	1.28 eV	0.92 eV
C1O5U1_ICSD_27053	3.79 eV	2.77 eV
C1O5U1_ICSD_87760	3.78 eV	2.91 eV
C1O6P1Sn2_ICSD_50969	1.92 eV	2.81 eV

Supplementary Table 703. Five-fold cross validated predictions for the band gap energy (97/277).

system	calculated	predicted
C1O7Si1Y2_ICSD_88878	4.78 eV	4.55 eV
C1Os1_ICSD_168277	0.39 eV	0.37 eV
C1Os1_ICSD_185996	0.41 eV	0.47 eV
C1Ru1_ICSD_181771	0.96 eV	0.65 eV
C1S14_ICSD_26464	2.50 eV	2.17 eV
C1S2_ICSD_33547	3.46 eV	3.04 eV
C1S3Tl2_ICSD_420223	1.88 eV	1.78 eV
C1Se2_ICSD_60374	2.57 eV	1.89 eV
C1Si1_ICSD_107204	1.75 eV	2.02 eV
C1Si1_ICSD_161173	1.85 eV	1.88 eV
C1Si1_ICSD_164974	1.37 eV	1.81 eV
C1Si1_ICSD_18136	1.74 eV	1.90 eV
C1Si1_ICSD_24168	1.96 eV	1.96 eV
C1Si1_ICSD_24169	2.02 eV	1.91 eV
C1Si1_ICSD_24170	2.22 eV	2.03 eV
C1Si1_ICSD_24261	2.29 eV	2.02 eV
C1Si1_ICSD_24630	1.78 eV	1.89 eV
C1Si1_ICSD_24631	2.02 eV	1.88 eV
C1Si1_ICSD_27635	1.97 eV	1.91 eV
C1Si1_ICSD_28303	1.81 eV	1.85 eV
C1Si1_ICSD_38150	2.00 eV	1.96 eV
C1Si1_ICSD_42858	1.97 eV	1.93 eV
C1Si1_ICSD_42859	1.99 eV	1.88 eV
C1Si1_ICSD_42862	1.99 eV	1.87 eV
C1Si1_ICSD_43827	1.63 eV	1.83 eV
C1Zr1_ICSD_183162	0.48 eV	0.54 eV
C1_ICSD_182269	4.28 eV	4.25 eV
C1_ICSD_182760	3.49 eV	2.95 eV
C1_ICSD_182761	2.48 eV	2.29 eV
C1_ICSD_186577	3.33 eV	3.11 eV
C1_ICSD_28417	0.26 eV	1.44 eV
C1_ICSD_53780	0.23 eV	0.43 eV
C1_ICSD_655131	4.12 eV	2.67 eV
C1_ICSD_66466	4.53 eV	4.36 eV
C1_ICSD_66467	4.40 eV	4.56 eV
C1_ICSD_66468	4.37 eV	4.38 eV
C1_ICSD_66469	4.57 eV	2.78 eV
C1_ICSD_66470	4.44 eV	2.79 eV
C1_ICSD_88814	0.52 eV	1.91 eV
C1_ICSD_88821	2.45 eV	3.60 eV
C22F14_ICSD_411879	2.22 eV	3.31 eV
C2Ca11N10_ICSD_410862	0.78 eV	1.32 eV
C2Ca1H2O4_ICSD_151251	4.65 eV	4.56 eV
C2Ca1H2O5_ICSD_153499	3.54 eV	4.18 eV
C2Ca1H4N2O2S2_ICSD_412784	4.06 eV	3.91 eV
C2Ca1H6N4O4_ICSD_9201	4.68 eV	4.31 eV
C2Ca1H6O7_ICSD_77096	3.66 eV	4.51 eV
C2Ca1H8O6P2_ICSD_261905	5.43 eV	4.68 eV
C2Ca1K2O6_ICSD_29442	4.39 eV	3.91 eV
C2Ca1Mg1O6_ICSD_66335	5.01 eV	4.77 eV

Supplementary Table 704. Five-fold cross validated predictions for the band gap energy (98/277).

system	calculated	predicted
C2Ca1N2S2_ICSD_412783	3.44 eV	3.31 eV
C2Ca1O4_ICSD_246004	3.13 eV	4.02 eV
C2Ca1O6Sr1_ICSD_201461	4.79 eV	4.56 eV
C2Ca1_ICSD_24074	1.58 eV	1.87 eV
C2Ca1_ICSD_31092	1.75 eV	1.80 eV
C2Ca1_ICSD_410313	1.32 eV	1.90 eV
C2Ca1_ICSD_54184	1.93 eV	1.96 eV
C2Ca1_ICSD_54188	2.23 eV	1.84 eV
C2Ca1_ICSD_66663	1.99 eV	1.92 eV
C2Ca2Cl2H4O6_ICSD_95291	3.52 eV	4.24 eV
C2Ca2O10Te2_ICSD_4319	3.85 eV	3.35 eV
C2Cd1Cl2H8N4S2_ICSD_83430	3.55 eV	3.61 eV
C2Cd1Cl3H8N1_ICSD_110600	4.17 eV	3.40 eV
C2Cd1Cl4H12N2_ICSD_110649	3.58 eV	3.26 eV
C2Cd1H2O4_ICSD_151254	4.33 eV	3.18 eV
C2Cd1N2S2_ICSD_32597	3.34 eV	3.03 eV
C2Cd1N2_ICSD_20748	6.14 eV	4.24 eV
C2Cd1O4_ICSD_170029	0.26 eV	2.75 eV
C2Ce6I10_ICSD_415091	0.30 eV	0.57 eV
C2Cl10N2Sb2_ICSD_279638	1.93 eV	2.13 eV
C2Cl1F2H1O1S1_ICSD_417530	3.68 eV	3.47 eV
C2Cl1H5O1_ICSD_151284	5.21 eV	5.13 eV
C2Cl1H8N1O1Si1_ICSD_110528	5.02 eV	4.42 eV
C2Cl1H8N1_ICSD_110601	5.39 eV	5.27 eV
C2Cl1Mg1Na3O6_ICSD_4237	4.22 eV	4.39 eV
C2Cl1N2Ni1Rb1_ICSD_380471	2.56 eV	2.63 eV
C2Cl2F1H13O8_ICSD_404343	1.12 eV	3.57 eV
C2Cl2H10N2Pt1_ICSD_240919	3.10 eV	2.73 eV
C2Cl2H12N4O4S2Zn1_ICSD_240218	5.38 eV	4.59 eV
C2Cl2H4Hg1N4_ICSD_405834	2.30 eV	2.91 eV
C2Cl2Hg3N4_ICSD_412609	1.75 eV	2.05 eV
C2Cl2O2Pt1_ICSD_82803	3.25 eV	2.68 eV
C2Cl2O4Pb2_ICSD_99805	2.93 eV	2.99 eV
C2Cl3Cu1H8N1_ICSD_400123	1.22 eV	1.73 eV
C2Cl3H8N1Pd1_ICSD_170106	2.21 eV	2.44 eV
C2Cl3N3S1_ICSD_86483	3.18 eV	3.30 eV
C2Cl4Cu1H10N2_ICSD_408537	1.17 eV	2.00 eV
C2Cl4N3P1_ICSD_81344	3.78 eV	3.82 eV
C2Cl4S2_ICSD_300291	2.93 eV	3.21 eV
C2Cl8N1Nb1_ICSD_402856	2.51 eV	2.17 eV
C2Cl8O4Sb2_ICSD_109900	2.11 eV	2.38 eV
C2Co1H2N4_ICSD_236414	2.35 eV	3.02 eV
C2Co1H4O6_ICSD_59927	2.96 eV	3.08 eV
C2Co1H6N2O3S2_ICSD_87	2.88 eV	2.96 eV
C2Co1La1_ICSD_617416	0.10 eV	0.22 eV
C2Co1N7Sr6_ICSD_162927	0.76 eV	1.68 eV
C2Co1Pr1_ICSD_78551	0.09 eV	0.28 eV
C2Co3O10Se2_ICSD_56465	2.52 eV	2.50 eV
C2Cs1F6N1O4S2_ICSD_281183	6.15 eV	5.42 eV
C2Cs1H1O4_ICSD_39364	2.34 eV	3.36 eV

Supplementary Table 705. Five-fold cross validated predictions for the band gap energy (99/277).

system	calculated	predicted
C2Cs1H1_ICSD_107491	4.59 eV	3.49 eV
C2Cs1I3N2_ICSD_32521	3.46 eV	3.19 eV
C2Cs1K1_ICSD_189822	3.46 eV	3.23 eV
C2Cs1Na1_ICSD_189824	3.30 eV	3.04 eV
C2Cs1Rb1_ICSD_189823	3.37 eV	3.23 eV
C2Cs2F4O4Pt1_ICSD_109890	2.79 eV	2.92 eV
C2Cs2H2O5_ICSD_249314	3.41 eV	3.43 eV
C2Cs2H4O6_ICSD_39365	3.40 eV	3.76 eV
C2Cs2O4_ICSD_163582	4.17 eV	3.69 eV
C2Cs2Pd1_ICSD_94396	2.09 eV	1.58 eV
C2Cs2Pt1_ICSD_94397	1.56 eV	1.43 eV
C2Cs2_ICSD_51533	2.87 eV	2.98 eV
C2Cs2_ICSD_51534	3.07 eV	2.97 eV
C2Cu1H6I1Se1_ICSD_174213	2.80 eV	2.05 eV
C2Cu1H6O7_ICSD_240734	1.27 eV	1.48 eV
C2Cu1K1N2_ICSD_27923	3.02 eV	3.33 eV
C2Cu1K1_ICSD_412037	2.34 eV	2.30 eV
C2Cu1K2O6_ICSD_200779	1.61 eV	1.41 eV
C2Cu1Na2O6_ICSD_10355	0.40 eV	1.47 eV
C2Cu1Rb1_ICSD_391118	2.22 eV	2.29 eV
C2Cu1Rb1_ICSD_412039	2.33 eV	2.37 eV
C2Cu3H2O8_ICSD_2934	1.23 eV	1.12 eV
C2Cu4H3N5_ICSD_414512	2.24 eV	2.53 eV
C2Cu5H8I4N2O4_ICSD_170205	2.92 eV	2.15 eV
C2Dy1K1O6_ICSD_407227	4.30 eV	4.31 eV
C2Er7I12N1_ICSD_401810	1.23 eV	1.22 eV
C2Eu1N2S2_ICSD_94426	2.68 eV	2.25 eV
C2Eu1_ICSD_380495	0.45 eV	1.02 eV
C2Eu1_ICSD_380496	0.18 eV	0.90 eV
C2F1H12N6O3P1_ICSD_151292	4.91 eV	4.82 eV
C2F1N1O1S1_ICSD_261212	4.38 eV	3.85 eV
C2F1N1O1S1_ICSD_261213	3.75 eV	3.62 eV
C2F2O3_ICSD_414408	5.26 eV	5.40 eV
C2F3Li1N4Pr2_ICSD_419925	3.19 eV	3.38 eV
C2F4O4S1_ICSD_170271	6.17 eV	5.56 eV
C2F4Te2_ICSD_73583	1.66 eV	2.89 eV
C2F5H12N6Sb1_ICSD_79544	4.95 eV	4.08 eV
C2F5O4Rb1Sb2_ICSD_109634	3.07 eV	3.56 eV
C2F6Fe1H12O12S2_ICSD_109429	3.86 eV	2.78 eV
C2F6H12N2Si1_ICSD_110673	6.78 eV	6.68 eV
C2F6H12N2Si1_ICSD_240237	6.79 eV	6.66 eV
C2F6H12N6Si1_ICSD_59237	5.20 eV	4.97 eV
C2F6H12N6Ti1_ICSD_36529	3.21 eV	3.98 eV
C2F6H12O12S2V1_ICSD_68779	2.37 eV	2.98 eV
C2F6H18N8O2Si1_ICSD_280103	5.01 eV	4.97 eV
C2F6H1N1O4S2_ICSD_81464	6.29 eV	5.94 eV
C2F6H2N2O6S3_ICSD_59804	5.92 eV	5.64 eV
C2F6Na2O4Sb2_ICSD_109946	3.08 eV	3.45 eV
C2F6Na2O4Sb2_ICSD_200225	3.08 eV	3.29 eV
C2F6O3_ICSD_401780	4.36 eV	5.45 eV

Supplementary Table 706. Five-fold cross validated predictions for the band gap energy (100/277).

system	calculated	predicted
C2F6Te2_ICSD_401990	2.40 eV	3.00 eV
C2Fe1H2N4_ICSD_419222	3.02 eV	2.22 eV
C2Fe1H4O6_ICSD_161344	2.21 eV	2.60 eV
C2Fe1H6N2O4_ICSD_260020	2.07 eV	2.64 eV
C2Ga2Ge4H10N2O12_ICSD_158855	3.48 eV	3.45 eV
C2Gd1H7O8S1_ICSD_163610	4.81 eV	4.26 eV
C2Gd1K1O6_ICSD_407226	4.08 eV	3.84 eV
C2H10N2O5_ICSD_64934	3.39 eV	4.40 eV
C2H10N4Ni1O6_ICSD_48155	3.90 eV	2.71 eV
C2H10N8O6S2Zn1_ICSD_170757	3.45 eV	3.55 eV
C2H12N14O1_ICSD_280660	1.96 eV	3.41 eV
C2H12N6Ni1S2_ICSD_72515	2.90 eV	2.52 eV
C2H12N6O8S2Zn1_ICSD_34708	4.84 eV	4.47 eV
C2H14N4O8Te1_ICSD_203185	2.79 eV	3.38 eV
C2H18N18_ICSD_280661	1.99 eV	3.63 eV
C2H1K1N4Ni1S2_ICSD_1732	1.76 eV	2.33 eV
C2H1K1O4_ICSD_246788	3.13 eV	3.41 eV
C2H1K5N4_ICSD_409559	3.78 eV	3.45 eV
C2H1N3_ICSD_240778	4.41 eV	4.26 eV
C2H2K2O5_ICSD_246782	3.41 eV	3.68 eV
C2H2K2O6_ICSD_246300	3.39 eV	3.78 eV
C2H2N2O1_ICSD_30774	3.89 eV	3.89 eV
C2H2N4Ni1_ICSD_172908	2.40 eV	2.51 eV
C2H2Na2O6_ICSD_246301	3.17 eV	4.04 eV
C2H2O4Sr1_ICSD_260441	4.69 eV	4.73 eV
C2H2O4Sr1_ICSD_260442	4.75 eV	4.60 eV
C2H2O5Rb2_ICSD_240494	3.42 eV	3.72 eV
C2H3In1O5_ICSD_249482	4.40 eV	3.66 eV
C2H3K1O3_ICSD_77115	5.19 eV	4.63 eV
C2H3K1O4_ICSD_151266	4.80 eV	4.48 eV
C2H3Na1O5_ICSD_1296	3.23 eV	3.86 eV
C2H3O4Rb1_ICSD_151357	4.82 eV	4.39 eV
C2H4I1N2Na1O2_ICSD_67245	4.37 eV	3.67 eV
C2H4Mn1O6_ICSD_240902	2.37 eV	2.76 eV
C2H4Mn2O12P2_ICSD_281672	2.93 eV	2.86 eV
C2H4N2Ni1S4_ICSD_110002	2.35 eV	2.34 eV
C2H4N4O4_ICSD_172534	2.35 eV	2.99 eV
C2H4N4_ICSD_281051	4.72 eV	3.90 eV
C2H4O6V1_ICSD_151270	2.64 eV	2.53 eV
C2H4O6Zn1_ICSD_56466	3.49 eV	3.56 eV
C2H5N1O1_ICSD_56913	4.95 eV	4.94 eV
C2H5N3S2_ICSD_37081	1.49 eV	2.74 eV
C2H5Na3O8_ICSD_35191	4.62 eV	4.07 eV
C2H6K1N1O4S2_ICSD_281526	5.31 eV	4.96 eV
C2H6Mn1O6_ICSD_173098	3.56 eV	2.98 eV
C2H6N1O4Rb1S2_ICSD_281527	5.18 eV	4.86 eV
C2H6N2Ni1O4_ICSD_260019	2.17 eV	2.64 eV
C2H6N2Rb2_ICSD_425117	3.75 eV	2.84 eV
C2H6N4O1_ICSD_414279	4.63 eV	4.38 eV
C2H6N4O4Zn1_ICSD_9202	4.92 eV	3.70 eV

Supplementary Table 707. Five-fold cross validated predictions for the band gap energy (101/277).

system	calculated	predicted
C2H6Ni1O6_ICSD_151342	3.72 eV	3.14 eV
C2H6O12U2_ICSD_172777	3.16 eV	2.93 eV
C2H6O1Se1_ICSD_171011	3.85 eV	3.97 eV
C2H6O4S1_ICSD_171689	6.21 eV	4.82 eV
C2H6O6S2Zn1_ICSD_109792	3.96 eV	4.33 eV
C2H6O6S2_ICSD_261156	4.00 eV	4.69 eV
C2H6O6Zn1_ICSD_151355	4.71 eV	4.40 eV
C2H6O6_ICSD_246777	3.27 eV	4.10 eV
C2H6S2_ICSD_249027	3.77 eV	3.10 eV
C2H6Se2_ICSD_249029	1.99 eV	2.30 eV
C2H6Te2_ICSD_249030	1.59 eV	2.22 eV
C2H8I1N1_ICSD_110536	4.33 eV	4.01 eV
C2H8I3N1Sn1_ICSD_402164	2.75 eV	2.60 eV
C2H8I3N1_ICSD_110538	1.97 eV	2.84 eV
C2H8In2O14Se2_ICSD_249780	3.42 eV	3.27 eV
C2H8N1O2P1S1_ICSD_110565	4.48 eV	4.50 eV
C2H8N1O4P1_ICSD_110400	5.52 eV	5.17 eV
C2H8N1O7V3_ICSD_110550	1.58 eV	2.11 eV
C2H8N2O3_ICSD_240961	3.45 eV	3.72 eV
C2H8N6O6S2_ICSD_32752	3.44 eV	3.22 eV
C2H8N6S2Zn1_ICSD_18146	3.92 eV	3.37 eV
C2H9K1Mg1O10_ICSD_63138	5.03 eV	4.65 eV
C2H9K1Ni1O10_ICSD_68710	3.98 eV	3.14 eV
C2H9N3O3_ICSD_163084	3.44 eV	3.43 eV
C2Hg1I1K1N2_ICSD_18198	2.64 eV	2.38 eV
C2Hg1N2O2_ICSD_245330	3.63 eV	2.92 eV
C2Hg1N2S2_ICSD_10304	2.12 eV	2.46 eV
C2Hg1N2Se2_ICSD_85760	2.17 eV	2.26 eV
C2Hg1N2_ICSD_30343	4.82 eV	3.25 eV
C2Hg1O4_ICSD_56459	2.63 eV	2.71 eV
C2Hg2N2O1_ICSD_29037	3.17 eV	2.67 eV
C2Ho1K1O6_ICSD_407223	4.33 eV	4.26 eV
C2I10La6_ICSD_415092	0.40 eV	0.85 eV
C2I12Pr6Rb1_ICSD_417636	0.45 eV	1.10 eV
C2I1K1N2_ICSD_40370	4.51 eV	3.56 eV
C2I2_ICSD_246785	3.32 eV	2.56 eV
C2I3N2Rb1_ICSD_35411	3.20 eV	3.31 eV
C2I4O2Pt2_ICSD_68098	1.77 eV	1.68 eV
C2K1N3_ICSD_411930	4.67 eV	4.39 eV
C2K1N3_ICSD_411931	3.16 eV	3.61 eV
C2K1N3_ICSD_411932	4.32 eV	4.13 eV
C2K1Nd1O6_ICSD_407225	3.09 eV	3.54 eV
C2K1O6Yb1_ICSD_407224	0.11 eV	1.75 eV
C2K2Mg1O6_ICSD_31295	4.04 eV	4.32 eV
C2K2N4O8_ICSD_109049	1.99 eV	2.59 eV
C2K2O4_ICSD_165561	2.74 eV	3.06 eV
C2K2O6_ICSD_412335	4.18 eV	3.59 eV
C2K2Pd1_ICSD_421489	1.81 eV	1.65 eV
C2K2Pt1_ICSD_421491	1.30 eV	1.24 eV
C2K2_ICSD_36142	3.26 eV	3.03 eV

Supplementary Table 708. Five-fold cross validated predictions for the band gap energy (102/277).

system	calculated	predicted
C2K2_ICSD_89528	2.72 eV	3.03 eV
C2La1Li1N4_ICSD_420440	3.04 eV	3.39 eV
C2La2O2_ICSD_462	2.99 eV	2.13 eV
C2Li2O4_ICSD_173993	3.24 eV	3.77 eV
C2Li2_ICSD_89535	3.31 eV	2.88 eV
C2Mg1Na2O6_ICSD_100482	4.40 eV	4.53 eV
C2Mg1_ICSD_88057	2.56 eV	2.15 eV
C2N2Ni1S2_ICSD_31320	1.77 eV	2.31 eV
C2N2O6S2_ICSD_23342	0.10 eV	2.25 eV
C2N2Pb1S2_ICSD_143	2.64 eV	2.69 eV
C2N2S1_ICSD_25520	3.85 eV	3.59 eV
C2N2S2Se1_ICSD_26610	2.25 eV	2.53 eV
C2N2S2Sn1_ICSD_201193	2.52 eV	2.68 eV
C2N2S2Sr1_ICSD_94427	3.82 eV	3.77 eV
C2N2S2Zn1_ICSD_22359	3.90 eV	3.41 eV
C2N2S3_ICSD_25505	2.61 eV	2.63 eV
C2N2S4_ICSD_77184	2.95 eV	2.50 eV
C2N2S9_ICSD_77442	2.58 eV	2.38 eV
C2N2Se1_ICSD_173995	3.64 eV	3.13 eV
C2N2Se1_ICSD_27441	3.47 eV	3.37 eV
C2N2Se2_ICSD_171332	2.26 eV	2.64 eV
C2N2Se3_ICSD_171333	1.99 eV	2.29 eV
C2N2Zn1_ICSD_22392	6.07 eV	5.39 eV
C2N2_ICSD_15870	4.79 eV	5.09 eV
C2N3Na1_ICSD_280187	1.40 eV	3.74 eV
C2N3Rb1_ICSD_411934	4.52 eV	4.47 eV
C2N3Rb1_ICSD_411935	4.16 eV	4.08 eV
C2N4S4_ICSD_170226	0.60 eV	1.91 eV
C2N4Si1_ICSD_93543	4.25 eV	4.26 eV
C2Na2O4_ICSD_171459	3.39 eV	3.20 eV
C2Na2Pd1_ICSD_411388	0.94 eV	1.54 eV
C2Na2Pt1_ICSD_50173	0.69 eV	1.11 eV
C2Na2_ICSD_28066	3.37 eV	2.85 eV
C2Na2_ICSD_95835	3.47 eV	3.02 eV
C2O4Pb1_ICSD_109830	2.79 eV	3.11 eV
C2O4Rb2_ICSD_163587	2.94 eV	3.26 eV
C2O4Rb2_ICSD_165562	4.24 eV	3.72 eV
C2O4Sn1_ICSD_150101	2.65 eV	2.50 eV
C2O4Tl2_ICSD_170127	2.86 eV	3.08 eV
C2O6Rb2_ICSD_412971	3.91 eV	3.88 eV
C2Os1_ICSD_168280	0.12 eV	0.78 eV
C2Pd1Rb2_ICSD_94394	1.93 eV	1.63 eV
C2Pt1Rb2_ICSD_94395	1.44 eV	1.39 eV
C2Rb2_ICSD_51529	3.58 eV	3.19 eV
C2Sr1_ICSD_410317	1.62 eV	1.48 eV
C2Sr1_ICSD_91051	2.27 eV	2.03 eV
C2U1_ICSD_619006	0.37 eV	0.77 eV
C2Yb1_ICSD_619131	0.18 eV	0.07 eV
C3Ca2H1Li1_ICSD_188224	1.25 eV	1.61 eV
C3Ca2K2O9_ICSD_83431	4.70 eV	4.44 eV

Supplementary Table 709. Five-fold cross validated predictions for the band gap energy (103/277).

system	calculated	predicted
C3Ca2Na2O9_ICSD_36237	4.82 eV	4.60 eV
C3Ca3Cl2_ICSD_33818	1.28 eV	2.39 eV
C3Cd1K1N3O3_ICSD_4097	4.21 eV	3.55 eV
C3Cd1N3Rb1S3_ICSD_14147	1.88 eV	2.63 eV
C3Ce1O13P1Sr3_ICSD_76608	1.83 eV	3.63 eV
C3Ce2F1Na1O9_ICSD_183233	4.07 eV	3.94 eV
C3Cl1H10N1O1_ICSD_110267	5.32 eV	4.77 eV
C3Cl1H10N1O4_ICSD_110284	5.61 eV	4.91 eV
C3Cl1H10N1O4_ICSD_110285	5.58 eV	4.92 eV
C3Cl1H9Si1_ICSD_171369	5.62 eV	5.28 eV
C3Cl3Cs1O3Os1_ICSD_39588	3.71 eV	2.80 eV
C3Cl3Ga1H9O1P1_ICSD_173260	4.88 eV	3.85 eV
C3Cl3H10N1Sn1_ICSD_170096	3.39 eV	3.04 eV
C3Cl3N3_ICSD_86484	3.74 eV	3.29 eV
C3Cl6H5O5Ru1Sb1_ICSD_24785	3.21 eV	2.60 eV
C3Cl7Mo1O1S2_ICSD_413426	1.40 eV	1.85 eV
C3Co1H3K1O6_ICSD_181922	3.71 eV	3.55 eV
C3Co1H3K1O6_ICSD_181923	3.66 eV	3.44 eV
C3Cr2N6_ICSD_421391	1.72 eV	2.28 eV
C3Cs1N3Se3_ICSD_4101	1.75 eV	2.73 eV
C3Cs2O9Sr2_ICSD_169231	4.58 eV	4.40 eV
C3Cu2H2N3O1Rb1_ICSD_281775	2.71 eV	2.93 eV
C3Eu4I3Li1N6_ICSD_170262	2.14 eV	2.08 eV
C3F1H7N1O5Sn1_ICSD_250206	3.13 eV	3.48 eV
C3F1La2Na1O9_ICSD_183234	4.03 eV	4.32 eV
C3F3H12N1_ICSD_110221	7.27 eV	6.44 eV
C3F3H6N3O2S1_ICSD_152106	2.20 eV	4.35 eV
C3F6_ICSD_151184	4.91 eV	5.41 eV
C3Fe1N1O4Tl1_ICSD_66317	2.62 eV	2.68 eV
C3Ga1N1Si3_ICSD_183049	2.60 eV	1.83 eV
C3H10I1N1_ICSD_249166	4.33 eV	4.19 eV
C3H20N4O10_ICSD_281534	4.79 eV	4.59 eV
C3H2O4_ICSD_109620	3.49 eV	4.59 eV
C3H3O6Sc1_ICSD_109661	3.72 eV	4.54 eV
C3H3O6Sc1_ICSD_281595	3.98 eV	4.02 eV
C3H3O6Y1_ICSD_109739	3.99 eV	4.20 eV
C3H5K1N2O5_ICSD_260200	3.05 eV	3.79 eV
C3H6N1Sb1_ICSD_170722	3.69 eV	3.38 eV
C3H6N4O3_ICSD_30562	4.36 eV	3.98 eV
C3H6O3_ICSD_151224	6.22 eV	5.02 eV
C3H7N3O5_ICSD_80262	4.77 eV	3.76 eV
C3H9N3O2_ICSD_109968	4.87 eV	4.87 eV
C3Hg1K1N3O3_ICSD_100116	3.25 eV	3.48 eV
C3Hg1K1N3S3_ICSD_20752	2.01 eV	2.04 eV
C3Hg1K1N3S3_ICSD_85761	2.12 eV	2.29 eV
C3Hg1N3Rb1S3_ICSD_101	1.98 eV	2.16 eV
C3K4O11U1_ICSD_69130	3.15 eV	3.06 eV
C3Lu2N6_ICSD_240311	3.78 eV	3.08 eV
C3Mg2_ICSD_71941	1.67 eV	2.12 eV
C3N4_ICSD_246659	2.48 eV	2.36 eV

Supplementary Table 710. Five-fold cross validated predictions for the band gap energy (104/277).

system	calculated	predicted
C3N4_ICSD_41950	3.17 eV	2.49 eV
C3N4_ICSD_41952	1.34 eV	2.81 eV
C3N4_ICSD_83261	3.77 eV	2.51 eV
C3N4_ICSD_83263	2.89 eV	2.53 eV
C3N4_ICSD_83265	1.45 eV	2.99 eV
C3N4_ICSD_97564	0.87 eV	2.80 eV
C3O11Tl4U1_ICSD_61345	2.92 eV	2.93 eV
C3O1S7_ICSD_165374	2.16 eV	2.52 eV
C3O2_ICSD_411461	3.92 eV	3.87 eV
C3O9Rb2Sr2_ICSD_169232	4.47 eV	4.35 eV
C3Os2_ICSD_168275	0.19 eV	0.53 eV
C3S8_ICSD_53591	1.70 eV	2.37 eV
C3Sc4_ICSD_42760	0.67 eV	0.68 eV
C4Ca1Cu1H4O8_ICSD_151315	1.95 eV	1.73 eV
C4Ca1Mg3O12_ICSD_201729	4.81 eV	4.71 eV
C4Ca1N6_ICSD_411362	4.55 eV	3.95 eV
C4Cd1Cl2H8N2_ICSD_162987	2.16 eV	3.40 eV
C4Cd1H20N2O12S4_ICSD_406374	4.91 eV	3.98 eV
C4Cd1H4O6_ICSD_249685	2.57 eV	2.87 eV
C4Cd1H6O2S4_ICSD_110011	2.41 eV	2.83 eV
C4Cd1H8N6S4_ICSD_170723	3.31 eV	3.27 eV
C4Cd1Hg1N4S4_ICSD_4102	2.48 eV	2.49 eV
C4Cd1Hg1N4Se4_ICSD_249203	2.20 eV	2.32 eV
C4Cd1K2N4_ICSD_23994	6.00 eV	5.27 eV
C4Cd1N4S4Zn1_ICSD_280039	3.52 eV	2.92 eV
C4Cd1N4Se4Zn1_ICSD_249202	3.17 eV	2.58 eV
C4Cd1N6_ICSD_413333	3.04 eV	4.18 eV
C4Cd1N6_ICSD_413334	4.55 eV	3.47 eV
C4Cl1N3_ICSD_246790	4.32 eV	4.22 eV
C4Cl2Cs2N4Pt1_ICSD_421674	2.78 eV	2.85 eV
C4Cl2F12N3P2V1_ICSD_68388	3.01 eV	2.98 eV
C4Cl2F6Te1_ICSD_260283	3.04 eV	3.28 eV
C4Cl2H12O6V2_ICSD_168381	3.18 eV	2.78 eV
C4Cl2H16N8Ni1S4_ICSD_61123	2.31 eV	2.62 eV
C4Cl2K2N4Pt1_ICSD_413704	2.63 eV	2.71 eV
C4Cl2Mg2Na6O12_ICSD_27790	3.78 eV	4.27 eV
C4Cl2N4Pt1Rb2_ICSD_419370	2.72 eV	2.67 eV
C4Cl2O4Os1_ICSD_171560	2.92 eV	2.53 eV
C4Cl2O4Os1_ICSD_171561	2.38 eV	2.70 eV
C4Cl3Co1Ga1K1O4_ICSD_410883	3.84 eV	3.24 eV
C4Cl3Co1O4Sn1_ICSD_69685	2.94 eV	3.09 eV
C4Cl4Fe1H12N1_ICSD_159487	2.60 eV	2.80 eV
C4Cl4H12S2Sn1_ICSD_170801	2.43 eV	2.72 eV
C4Cl4H14N2Sn1_ICSD_170799	3.01 eV	3.23 eV
C4Cl4N4S4_ICSD_48164	2.78 eV	2.35 eV
C4Cl6H16N2Sn1_ICSD_110603	2.54 eV	3.31 eV
C4Cl6H16N2Ti1_ICSD_281532	2.73 eV	3.64 eV
C4Co1Cs1O4_ICSD_31360	3.86 eV	3.40 eV
C4Co1F3O4Si1_ICSD_16069	4.10 eV	3.69 eV
C4Co1Hg1N4S4_ICSD_36062	2.34 eV	2.24 eV

Supplementary Table 711. Five-fold cross validated predictions for the band gap energy (105/277).

system	calculated	predicted
C4Co1Hg1N4Se4_ICSD_172058	2.17 eV	2.23 eV
C4Co1Li1O4_ICSD_30854	4.09 eV	3.48 eV
C4Co1N6_ICSD_85618	3.21 eV	3.23 eV
C4Co1Na1O4_ICSD_30855	3.83 eV	3.66 eV
C4Co1O4Tl1_ICSD_6011	3.11 eV	3.06 eV
C4Cr1H4K1O10_ICSD_172524	3.08 eV	2.60 eV
C4Cr1H4O10Rb1_ICSD_281565	3.03 eV	2.48 eV
C4Cr1H8N6S4_ICSD_69067	2.33 eV	2.43 eV
C4Cs1H12O14V1_ICSD_109663	2.24 eV	2.61 eV
C4Cs2H2N4O2Pt1_ICSD_418911	2.60 eV	2.58 eV
C4Cs2O4_ICSD_154357	2.69 eV	2.83 eV
C4Cu1H4O8Sr1_ICSD_150120	1.96 eV	1.87 eV
C4Cu1K3N4_ICSD_76933	4.25 eV	3.42 eV
C4F12Ge4S6_ICSD_62223	3.34 eV	3.17 eV
C4F12Ge4Se6_ICSD_62224	2.41 eV	2.88 eV
C4F12H17O20S4V1_ICSD_201678	0.98 eV	2.73 eV
C4F12N2O8S4Sr1_ICSD_163023	6.21 eV	5.60 eV
C4F12P4_ICSD_23899	1.08 eV	4.66 eV
C4F1H12N1O4Os1_ICSD_110743	2.56 eV	2.73 eV
C4F1H12O5Re1_ICSD_415419	3.04 eV	3.08 eV
C4F2H13N1_ICSD_155703	6.54 eV	6.38 eV
C4F3H9Si1_ICSD_162079	6.36 eV	6.06 eV
C4F6Hg1_ICSD_165635	3.61 eV	3.28 eV
C4F6Hg2O4_ICSD_2022	3.24 eV	2.79 eV
C4Fe1H12N10S4_ICSD_170705	3.29 eV	2.61 eV
C4Fe1H12O6S4_ICSD_203234	1.58 eV	2.43 eV
C4Fe1Hg1N4S4_ICSD_87888	1.91 eV	2.23 eV
C4Fe1K2O4_ICSD_200025	3.28 eV	2.96 eV
C4Fe2H2O6_ICSD_109774	1.85 eV	1.88 eV
C4H10N8Ni1S2_ICSD_110013	3.05 eV	2.80 eV
C4H11N1O10_ICSD_249174	3.37 eV	3.96 eV
C4H12I1N1O3_ICSD_416527	3.96 eV	3.68 eV
C4H12I1N1_ICSD_55080	4.20 eV	4.16 eV
C4H12I3O2S2Tl1_ICSD_170738	1.70 eV	2.23 eV
C4H12Mn1N10_ICSD_110766	2.95 eV	2.36 eV
C4H12N2O2Se1_ICSD_402259	3.31 eV	3.78 eV
C4H12N2O2_ICSD_150985	2.59 eV	4.05 eV
C4H12O3P2_ICSD_170836	5.36 eV	5.14 eV
C4H12O4Si1_ICSD_171152	4.77 eV	4.74 eV
C4H1O4Rb1_ICSD_172323	2.70 eV	2.69 eV
C4H20N4Ni1O12Pd1_ICSD_417562	1.43 eV	2.68 eV
C4H20N4O12Pd1Zn1_ICSD_417563	3.56 eV	2.65 eV
C4H20N4O12Pt1Zn1_ICSD_417565	3.42 eV	3.23 eV
C4H2O4_ICSD_151146	2.87 eV	3.92 eV
C4H2O6Sn2_ICSD_96547	2.13 eV	2.47 eV
C4H4Mo2O8_ICSD_151313	2.07 eV	2.25 eV
C4H4N4O2Pt1_ICSD_409975	3.42 eV	2.91 eV
C4H4N4_ICSD_170078	3.59 eV	4.12 eV
C4H4Na5O14Sc1_ICSD_411988	4.96 eV	4.49 eV
C4H5Na3O8_ICSD_109935	4.46 eV	4.31 eV

Supplementary Table 712. Five-fold cross validated predictions for the band gap energy (106/277).

system	calculated	predicted
C4H6Hg1N4O2_ICSD_418607	3.87 eV	3.43 eV
C4H6N2S2Sn1_ICSD_171668	3.64 eV	3.37 eV
C4H6O7Sr1_ICSD_109771	2.85 eV	3.49 eV
C4H7O11Y1_ICSD_109808	3.54 eV	4.32 eV
C4H8I4Sn1_ICSD_110024	2.98 eV	2.79 eV
C4H8Mg1N6O4_ICSD_414648	4.78 eV	4.38 eV
C4H8N2O4_ICSD_172054	2.80 eV	3.92 eV
C4H8O4Sn1_ICSD_151276	4.33 eV	3.64 eV
C4H8O8Zn1_ICSD_109772	2.69 eV	3.63 eV
C4H9N1O6Zn1_ICSD_240733	4.64 eV	4.45 eV
C4H9N1Se2_ICSD_163646	3.54 eV	3.35 eV
C4Hg1K2N4_ICSD_62084	5.84 eV	5.31 eV
C4Hg1Mn1N4S4_ICSD_87889	2.28 eV	2.13 eV
C4Hg1Mn1N4Se4_ICSD_240722	2.13 eV	2.08 eV
C4Hg1N4Rb2_ICSD_100685	5.82 eV	4.66 eV
C4Hg1N4S4Zn1_ICSD_280028	2.49 eV	2.73 eV
C4Hg1N4Se4Zn1_ICSD_188764	2.32 eV	2.43 eV
C4I2K2N4Pt1_ICSD_413706	1.35 eV	1.85 eV
C4I2N4Pt1Rb2_ICSD_421673	1.45 eV	1.80 eV
C4I2O4Ru1_ICSD_23919	2.18 eV	2.25 eV
C4K1N3_ICSD_77046	3.74 eV	3.01 eV
C4K1N4Tl1_ICSD_170125	5.42 eV	4.62 eV
C4K2N4Ni1_ICSD_24099	4.02 eV	4.22 eV
C4K2N4Pd1S4_ICSD_9473	1.64 eV	2.29 eV
C4K2N4Pd1_ICSD_413899	4.43 eV	3.93 eV
C4K2N4Pt1_ICSD_413898	2.90 eV	3.24 eV
C4K2N4Zn1_ICSD_23993	6.09 eV	5.23 eV
C4La1N8Rb1Si1_ICSD_419337	4.08 eV	3.75 eV
C4Li2O4_ICSD_154354	2.22 eV	3.29 eV
C4Mg1N6_ICSD_411361	4.64 eV	4.75 eV
C4Mg2Na6O16S1_ICSD_158962	4.54 eV	4.19 eV
C4Mg2Na6O16S1_ICSD_27792	3.72 eV	4.37 eV
C4Mn1N1O5_ICSD_15221	2.32 eV	2.49 eV
C4Mn1N6_ICSD_92075	2.16 eV	2.85 eV
C4N3Na1_ICSD_31926	3.55 eV	4.12 eV
C4N4Ni1Tl2_ICSD_249564	3.38 eV	3.12 eV
C4N4Ni1Zn1_ICSD_169760	4.14 eV	4.51 eV
C4N4Pd1Tl2_ICSD_249565	3.69 eV	3.19 eV
C4N4Pd1Zn1_ICSD_169759	4.72 eV	4.22 eV
C4N4Pt1Rb2_ICSD_171492	2.97 eV	3.08 eV
C4N4Pt1Tl2_ICSD_202570	3.46 eV	3.04 eV
C4N4Pt1Zn1_ICSD_169758	4.43 eV	3.35 eV
C4N6Ni1_ICSD_85617	3.03 eV	3.05 eV
C4N6Pb1_ICSD_412288	3.64 eV	3.23 eV
C4N6Sr1_ICSD_411363	4.68 eV	4.00 eV
C4Na2O4_ICSD_154355	2.50 eV	2.45 eV
C4Na2O8Sn1_ICSD_388	2.82 eV	3.17 eV
C4Ni1O4_ICSD_74196	3.95 eV	3.14 eV
C4O4Pb1_ICSD_248144	3.01 eV	2.72 eV
C4O4Rb2_ICSD_154356	2.67 eV	2.91 eV

Supplementary Table 713. Five-fold cross validated predictions for the band gap energy (107/277).

system	calculated	predicted
C4O4Ru1_ICSD_72577	2.13 eV	2.28 eV
C4O4Tl2_ICSD_260404	3.36 eV	3.03 eV
C5Cd1Fe1N6O1_ICSD_109420	2.68 eV	2.96 eV
C5Cl1Mn1O5_ICSD_16135	2.99 eV	2.92 eV
C5Cl1O5Re1_ICSD_35625	2.64 eV	2.68 eV
C5Cl1O5Tc1_ICSD_167772	2.77 eV	2.64 eV
C5Cu1Fe1N6O1_ICSD_414021	2.24 eV	2.13 eV
C5Cu2H8N4_ICSD_281777	2.76 eV	2.89 eV
C5Dy1H9N2O8_ICSD_151327	4.50 eV	4.40 eV
C5Er1H9N2O8_ICSD_151326	4.48 eV	4.30 eV
C5Fe1H8N6O4_ICSD_83731	2.78 eV	2.66 eV
C5Fe1N6O1Tl2_ICSD_14184	2.54 eV	2.79 eV
C5Fe1O5_ICSD_23918	3.64 eV	3.24 eV
C5Fe1O5_ICSD_300118	3.61 eV	3.51 eV
C5H10O5_ICSD_151281	6.42 eV	5.16 eV
C5H12N2O1_ICSD_150997	4.63 eV	4.90 eV
C5I1Mn1O5_ICSD_71878	2.63 eV	2.74 eV
C5I1O5Re1_ICSD_71219	2.29 eV	2.40 eV
C5I1O5Tc1_ICSD_85335	2.38 eV	2.16 eV
C5Ir4_ICSD_181486	0.49 eV	0.40 eV
C5N4_ICSD_2130	5.93 eV	3.99 eV
C60K6_ICSD_66879	0.59 eV	0.82 eV
C60Rb6_ICSD_66880	0.69 eV	0.86 eV
C60Sr3_ICSD_75354	0.31 eV	1.30 eV
C60_ICSD_74523	1.35 eV	1.12 eV
C60_ICSD_95370	0.80 eV	0.97 eV
C60_ICSD_96620	0.95 eV	0.94 eV
C68Co2O8_ICSD_166882	1.35 eV	2.09 eV
C6Cd1Cs2Fe1N6_ICSD_151696	4.69 eV	3.79 eV
C6Cd1N6Pd1_ICSD_6093	4.12 eV	4.42 eV
C6Cd2Fe1N6_ICSD_417826	3.02 eV	3.75 eV
C6Cd2N6Os1_ICSD_417821	2.61 eV	2.93 eV
C6Cd2N6Ru1_ICSD_417822	2.87 eV	3.39 eV
C6Cl2H18N4S2Se2_ICSD_151024	3.45 eV	3.52 eV
C6Cl3H21N2_ICSD_402690	5.31 eV	5.19 eV
C6Cl6H16O14Ru3_ICSD_413219	1.51 eV	2.32 eV
C6Co1Cs2Li1N6_ICSD_60539	5.12 eV	4.35 eV
C6Co1Cs2N6Rb1_ICSD_411128	5.10 eV	4.44 eV
C6Co1Er1H8N6O4_ICSD_171622	4.94 eV	4.15 eV
C6Co1Er1N6_ICSD_171616	4.78 eV	4.14 eV
C6Co1Fe1H18N12_ICSD_411630	3.20 eV	2.91 eV
C6Co1H3N6_ICSD_28502	4.94 eV	4.03 eV
C6Co1K3N6_ICSD_23048	5.16 eV	3.83 eV
C6Co1Li1N6Rb2_ICSD_83393	5.03 eV	4.43 eV
C6Co2H18N12_ICSD_2561	4.05 eV	3.30 eV
C6Cr1Cs2Li1N6_ICSD_68068	3.70 eV	3.94 eV
C6Cr1O6_ICSD_8286	3.63 eV	2.93 eV
C6Cs2Fe1Li1N6_ICSD_60542	2.81 eV	3.56 eV
C6Cs2Fe1N6Ni1_ICSD_151706	3.64 eV	3.62 eV
C6Cs2Ir1Li1N6_ICSD_9032	5.66 eV	3.86 eV

Supplementary Table 714. Five-fold cross validated predictions for the band gap energy (108/277).

system	calculated	predicted
C6Cs2N9Na1_ICSD_407809	4.41 eV	4.19 eV
C6Cu1Fe1K2N6_ICSD_28654	3.37 eV	2.81 eV
C6Cu1Fe1K2N6_ICSD_99499	1.59 eV	2.89 eV
C6Cu1Fe1Li2N6_ICSD_28656	2.10 eV	2.58 eV
C6Cu1Fe1N6Na2_ICSD_28657	2.16 eV	2.70 eV
C6Cu1Fe1N6Rb2_ICSD_28659	3.73 eV	3.45 eV
C6Cu2F6O6_ICSD_260351	3.08 eV	1.74 eV
C6Eu1N9_ICSD_391267	0.91 eV	2.14 eV
C6F12Fe1O6Sb2_ICSD_280005	4.44 eV	4.06 eV
C6F12O6Os1Sb2_ICSD_152388	4.65 eV	3.73 eV
C6F12O6Ru1Sb2_ICSD_152386	4.87 eV	4.28 eV
C6F5H3O3Se1_ICSD_410719	3.47 eV	3.59 eV
C6F6H24N2O2Sn1_ICSD_110229	4.24 eV	3.90 eV
C6F6Na4O12Sn4_ICSD_109824	2.46 eV	3.22 eV
C6Fe1H4N6_ICSD_30559	4.51 eV	3.23 eV
C6Fe1K2N6Ni1_ICSD_28667	3.73 eV	3.42 eV
C6Fe1K3N6_ICSD_31883	2.60 eV	2.86 eV
C6Fe1Mn2N6_ICSD_417824	3.66 eV	3.17 eV
C6Fe1N6Pb2_ICSD_51493	3.70 eV	3.31 eV
C6Fe1N6Sn2_ICSD_51494	3.82 eV	3.52 eV
C6Gd1N9_ICSD_416002	3.56 eV	3.12 eV
C6H12N10O2S2Zn1_ICSD_405377	3.79 eV	3.72 eV
C6H12N6O8Pt1_ICSD_281359	2.67 eV	2.90 eV
C6H18I2Pb2_ICSD_405865	3.13 eV	3.05 eV
C6H18N3O1P1_ICSD_409222	3.91 eV	4.38 eV
C6H20N2S6_ICSD_110278	1.75 eV	3.01 eV
C6H8Ho1K1N6O4Ru1_ICSD_281332	3.89 eV	3.87 eV
C6I4O6Os2_ICSD_68901	1.90 eV	2.15 eV
C6K3Mn1N6_ICSD_24933	1.38 eV	2.81 eV
C6La1N9_ICSD_391272	3.69 eV	3.38 eV
C6Mn2N6Os1_ICSD_417825	3.11 eV	2.77 eV
C6Mn2N6Ru1_ICSD_417823	3.40 eV	2.89 eV
C6Mo1O6_ICSD_30809	3.23 eV	2.48 eV
C6N9Nd1_ICSD_391269	1.86 eV	2.85 eV
C6N9Pr1_ICSD_391270	3.48 eV	3.06 eV
C6Na2O6_ICSD_170805	1.03 eV	2.64 eV
C6O6W1_ICSD_300206	3.10 eV	2.77 eV
C6O8Tl4_ICSD_260372	2.92 eV	3.09 eV
C7Ho4_ICSD_74817	0.58 eV	1.05 eV
C8Ca1H16K2O24Zr1_ICSD_245276	3.08 eV	3.56 eV
C8Cd1H10O12_ICSD_249683	2.79 eV	2.87 eV
C8Cl1H20Li1O4_ICSD_151063	4.89 eV	4.91 eV
C8Cl4H24N2Pd1_ICSD_59900	2.44 eV	2.59 eV
C8Co1H24Li1N8O12_ICSD_110738	2.72 eV	2.89 eV
C8Co2O8_ICSD_16567	2.83 eV	3.01 eV
C8Cu3H8Na2O16_ICSD_171669	1.03 eV	1.44 eV
C8F12O8Ru2_ICSD_171336	1.38 eV	2.79 eV
C8F12Pt1S4_ICSD_165606	1.07 eV	2.49 eV
C8Ga2H26N4_ICSD_163002	3.45 eV	4.28 eV
C8Ge2H24In1P1_ICSD_163057	3.51 eV	3.44 eV

Supplementary Table 715. Five-fold cross validated predictions for the band gap energy (109/277).

system	calculated	predicted
C8H16Hg1N6S2_ICSD_260899	3.57 eV	3.38 eV
C8H24Mn1N2Se10Sn4_ICSD_249783	1.22 eV	2.07 eV
C8H24N2O4S1_ICSD_52027	3.89 eV	4.77 eV
C8H4K2Ni1O6S4_ICSD_165299	1.71 eV	2.44 eV
C8I2Mo2O8_ICSD_36	0.73 eV	1.98 eV
C8K1O8Y1_ICSD_170216	2.09 eV	2.94 eV
C8K5N8Nb1_ICSD_16458	1.78 eV	3.23 eV
C8O4Se2_ICSD_172229	1.49 eV	2.28 eV
C8_ICSD_20351	3.01 eV	3.52 eV
C9Fe2O9_ICSD_31030	2.67 eV	3.05 eV
Ca10Li1Mg1Sb9_ICSD_171161	0.92 eV	0.64 eV
Ca10O24P6Se1_ICSD_410781	3.77 eV	4.00 eV
Ca10O25P6_ICSD_87727	3.18 eV	4.54 eV
Ca12Li6Mo4N16O3_ICSD_83865	2.38 eV	2.12 eV
Ca12Li6N16O3W4_ICSD_83866	2.66 eV	2.35 eV
Ca14Si19_ICSD_78963	0.11 eV	0.71 eV
Ca1Cd2P2_ICSD_100063	0.92 eV	0.83 eV
Ca1Cd2Sb2_ICSD_12151	0.19 eV	0.21 eV
Ca1Cl1F1_ICSD_1130	5.66 eV	5.78 eV
Ca1Cl1Fe1O2_ICSD_69869	0.56 eV	1.25 eV
Ca1Cl1Fe1O2_ICSD_96556	1.65 eV	1.52 eV
Ca1Cl1H1O1_ICSD_24403	5.01 eV	4.63 eV
Ca1Cl1H1_ICSD_25601	3.92 eV	3.93 eV
Ca1Cl2H12O6_ICSD_59142	4.98 eV	4.84 eV
Ca1Cl2H4O2_ICSD_960	5.35 eV	4.96 eV
Ca1Cl2H8O4_ICSD_16484	5.31 eV	4.94 eV
Ca1Cl2H8O4_ICSD_8061	5.10 eV	5.31 eV
Ca1Cl2O4_ICSD_171020	2.60 eV	3.85 eV
Ca1Cl2_ICSD_51238	5.44 eV	5.17 eV
Ca1Cl2_ICSD_56421	5.34 eV	5.23 eV
Ca1Cl2_ICSD_56769	5.59 eV	5.26 eV
Ca1Cl2_ICSD_86209	2.14 eV	4.06 eV
Ca1Cl6H24Mg2O12_ICSD_16326	5.11 eV	4.82 eV
Ca1Co1F6Li1_ICSD_32730	2.79 eV	2.94 eV
Ca1Co1O6Si2_ICSD_159546	4.03 eV	3.47 eV
Ca1Co1O7P2_ICSD_202852	3.90 eV	3.82 eV
Ca1Co3O14P4_ICSD_74047	3.86 eV	3.43 eV
Ca1Cr1F5_ICSD_10286	2.74 eV	2.31 eV
Ca1Cr1F5_ICSD_23174	2.74 eV	2.29 eV
Ca1Cr1F6Li1_ICSD_73985	4.01 eV	3.12 eV
Ca1Cr1F6_ICSD_10343	1.59 eV	1.87 eV
Ca1Cr1O10Si4_ICSD_83465	3.29 eV	2.94 eV
Ca1Cr1O4_ICSD_30283	2.36 eV	2.10 eV
Ca1Cr1O7P2_ICSD_411743	2.77 eV	2.66 eV
Ca1Cs1F3_ICSD_290448	6.90 eV	6.43 eV
Ca1Cs1H3_ICSD_168718	3.13 eV	3.06 eV
Ca1Cs1H3_ICSD_619213	3.13 eV	2.87 eV
Ca1Cs1N3O6_ICSD_56433	2.65 eV	2.58 eV
Ca1Cs2F4_ICSD_82616	6.07 eV	6.00 eV
Ca1Cs2H4_ICSD_261358	3.00 eV	2.93 eV

Supplementary Table 716. Five-fold cross validated predictions for the band gap energy (110/277).

system	calculated	predicted
Ca1Cu1F4_ICSD_9928	2.67 eV	2.15 eV
Ca1Cu1Ge2O6_ICSD_170819	1.71 eV	1.48 eV
Ca1Cu1Ge2O6_ICSD_170823	1.20 eV	1.38 eV
Ca1Cu1H2O5Si1_ICSD_30926	0.10 eV	1.26 eV
Ca1Cu1O10Si4_ICSD_402012	2.37 eV	2.33 eV
Ca1Cu1O2_ICSD_84868	0.29 eV	1.27 eV
Ca1Cu1P1_ICSD_49740	0.14 eV	0.43 eV
Ca1Cu3Ge4O12_ICSD_184279	1.71 eV	1.29 eV
Ca1Cu3O12Pt4_ICSD_248230	0.62 eV	0.96 eV
Ca1Cu3O12Sn4_ICSD_184281	0.71 eV	0.78 eV
Ca1Cu3O12Ti4_ICSD_161826	1.22 eV	1.38 eV
Ca1Cu3O12Ti4_ICSD_246526	1.22 eV	1.37 eV
Ca1Er2S4_ICSD_619253	1.56 eV	1.71 eV
Ca1F1H4O5P1_ICSD_2802	5.08 eV	5.43 eV
Ca1F2_ICSD_51237	5.14 eV	6.50 eV
Ca1F2_ICSD_51239	6.24 eV	6.78 eV
Ca1F2_ICSD_51283	7.45 eV	7.20 eV
Ca1F2_ICSD_60368	7.13 eV	6.28 eV
Ca1F3K1_ICSD_153629	6.62 eV	6.50 eV
Ca1F3K1_ICSD_154073	6.14 eV	6.28 eV
Ca1F3Rb1_ICSD_201252	6.40 eV	6.34 eV
Ca1F4H2_ICSD_419144	7.59 eV	6.79 eV
Ca1F4Pd1_ICSD_32674	2.22 eV	2.44 eV
Ca1F4Zn1_ICSD_31366	5.93 eV	5.91 eV
Ca1F5Fe1_ICSD_412862	3.34 eV	2.70 eV
Ca1F5Mn1_ICSD_69632	1.33 eV	2.20 eV
Ca1F5Ti1_ICSD_49529	3.26 eV	3.69 eV
Ca1F6Ga1Li1_ICSD_152300	6.51 eV	6.55 eV
Ca1F6Li1Ni1_ICSD_32732	1.96 eV	3.11 eV
Ca1F6Pb1_ICSD_25522	3.44 eV	3.62 eV
Ca1F6Pd1_ICSD_26164	2.61 eV	2.31 eV
Ca1F6Pt1_ICSD_37443	3.16 eV	2.75 eV
Ca1F6Rh1_ICSD_42160	1.30 eV	1.98 eV
Ca1F6Si1_ICSD_183914	7.52 eV	6.92 eV
Ca1F6Sn1_ICSD_35713	4.83 eV	4.67 eV
Ca1F6Sn1_ICSD_35723	4.86 eV	4.76 eV
Ca1F6Sn2_ICSD_92907	3.56 eV	3.95 eV
Ca1F8Hf1Li2_ICSD_95248	6.99 eV	5.63 eV
Ca1Fe1Ge2O6_ICSD_290257	1.21 eV	1.98 eV
Ca1Fe1O2_ICSD_246245	1.24 eV	1.18 eV
Ca1Fe1O3_ICSD_92340	0.55 eV	1.18 eV
Ca1Fe1O3_ICSD_92342	0.16 eV	1.13 eV
Ca1Fe1O6Si2_ICSD_83438	2.82 eV	2.47 eV
Ca1Fe1O6Ti2_ICSD_79353	1.66 eV	1.53 eV
Ca1Fe2O4_ICSD_16695	1.37 eV	1.04 eV
Ca1Fe3O12Ti4_ICSD_79277	2.01 eV	1.69 eV
Ca1Fe3O5_ICSD_16354	0.72 eV	0.57 eV
Ca1Fe4O6_ICSD_16355	0.46 eV	0.54 eV
Ca1Fe5O7_ICSD_16356	0.41 eV	0.49 eV
Ca1Ga1Ge1H1_ICSD_173567	0.47 eV	0.45 eV

Supplementary Table 717. Five-fold cross validated predictions for the band gap energy (111/277).

system	calculated	predicted
Ca1Ga1H1Si1_ICSD_173566	0.25 eV	0.84 eV
Ca1Ga1H1Sn1_ICSD_173568	0.27 eV	0.47 eV
Ca1Ga1La1O4_ICSD_96463	3.44 eV	3.46 eV
Ca1Ga1Li1N2_ICSD_415411	2.22 eV	1.89 eV
Ca1Ga2P2_ICSD_422525	0.21 eV	0.59 eV
Ca1Ga2S4_ICSD_619292	2.64 eV	2.62 eV
Ca1Ga4O7_ICSD_10351	2.67 eV	2.64 eV
Ca1Ge1Li2O4_ICSD_19024	4.06 eV	3.74 eV
Ca1Ge1N2_ICSD_23523	2.62 eV	2.22 eV
Ca1Ge1O3_ICSD_403086	3.41 eV	2.93 eV
Ca1Ge1O5Zr1_ICSD_154327	1.64 eV	3.01 eV
Ca1Ge1O8P2_ICSD_423043	3.84 eV	4.30 eV
Ca1Ge1Sr1_ICSD_172006	0.43 eV	0.40 eV
Ca1Ge2O5_ICSD_151581	2.42 eV	2.50 eV
Ca1Ge2O5_ICSD_156194	2.65 eV	2.74 eV
Ca1Ge2O5_ICSD_200614	2.40 eV	2.51 eV
Ca1Ge2O6Zn1_ICSD_59938	2.70 eV	2.65 eV
Ca1H10O8Zn2_ICSD_260592	3.99 eV	3.87 eV
Ca1H12N2O10S2_ICSD_410199	4.76 eV	5.24 eV
Ca1H1I1_ICSD_25545	3.45 eV	3.04 eV
Ca1H1K3O8P2_ICSD_35344	4.96 eV	4.87 eV
Ca1H1Mn1O5Si1_ICSD_83774	1.20 eV	2.25 eV
Ca1H1Na1O4Si1_ICSD_97911	4.50 eV	4.66 eV
Ca1H1O4P1_ICSD_87196	5.43 eV	5.33 eV
Ca1H2I2O7_ICSD_36635	3.59 eV	3.27 eV
Ca1H2K2O9S2_ICSD_157072	5.37 eV	5.33 eV
Ca1H2O12P4_ICSD_82359	5.68 eV	5.57 eV
Ca1H2O2_ICSD_15471	4.03 eV	3.94 eV
Ca1H2O5Si1Zn1_ICSD_34944	4.09 eV	4.06 eV
Ca1H2O7P2_ICSD_91818	5.71 eV	5.64 eV
Ca1H2_ICSD_157943	0.63 eV	2.09 eV
Ca1H2_ICSD_261635	3.01 eV	1.92 eV
Ca1H3K1_ICSD_168716	3.32 eV	3.31 eV
Ca1H3Li1_ICSD_168713	2.55 eV	2.91 eV
Ca1H3Na1_ICSD_168715	3.31 eV	3.26 eV
Ca1H3O4P1_ICSD_411737	6.08 eV	5.77 eV
Ca1H3Rb1_ICSD_65195	3.26 eV	3.15 eV
Ca1H4Mg1Ni1_ICSD_400003	1.95 eV	2.43 eV
Ca1H4O4P2_ICSD_44413	5.60 eV	5.43 eV
Ca1H4O6S1_ICSD_2059	5.28 eV	4.96 eV
Ca1H4O7Se2_ICSD_84441	4.23 eV	4.09 eV
Ca1H4Rb2_ICSD_65196	3.30 eV	3.06 eV
Ca1H5O6P1_ICSD_98804	5.34 eV	5.36 eV
Ca1H6K1N3_ICSD_1359	2.15 eV	2.18 eV
Ca1H6N3Rb1_ICSD_14014	1.96 eV	2.45 eV
Ca1H6O7P2_ICSD_36285	6.41 eV	6.09 eV
Ca1H6O9P2_ICSD_133	5.46 eV	5.55 eV
Ca1H8K2N12O4_ICSD_34953	4.17 eV	3.33 eV
Ca1H8O14P2V2_ICSD_67660	1.98 eV	1.95 eV
Ca1Hg1O2_ICSD_80717	2.11 eV	2.00 eV

Supplementary Table 718. Five-fold cross validated predictions for the band gap energy (112/277).

system	calculated	predicted
Ca1Ho2S4_ICSD_619369	1.54 eV	1.41 eV
Ca1I2O6_ICSD_1391	1.28 eV	2.92 eV
Ca1I2_ICSD_52280	3.80 eV	3.09 eV
Ca1In2O4_ICSD_52390	2.13 eV	1.98 eV
Ca1In2P2_ICSD_260562	0.48 eV	0.39 eV
Ca1Ir2P2_ICSD_95756	0.48 eV	0.52 eV
Ca1K1O9P3_ICSD_281588	5.05 eV	5.17 eV
Ca1K2N4O8_ICSD_85505	1.24 eV	2.14 eV
Ca1Li1N1_ICSD_107304	1.38 eV	1.46 eV
Ca1Li1Sb1_ICSD_52775	0.63 eV	0.70 eV
Ca1Li2N4Si2_ICSD_421548	3.48 eV	3.32 eV
Ca1Li2O4Si1_ICSD_19023	5.20 eV	4.77 eV
Ca1Li2O7Ta2_ICSD_245841	3.25 eV	2.95 eV
Ca1Li4O8Si2Sr1_ICSD_79867	4.83 eV	4.63 eV
Ca1Lu2S4_ICSD_200013	1.33 eV	1.47 eV
Ca1Mg1O6Si2_ICSD_159522	4.94 eV	4.83 eV
Ca1Mg2N2_ICSD_411175	2.00 eV	1.71 eV
Ca1Mg2O12S3_ICSD_418454	5.63 eV	5.33 eV
Ca1Mg2Sb2_ICSD_100045	0.81 eV	0.80 eV
Ca1Mn1O3_ICSD_168906	0.89 eV	1.03 eV
Ca1Mn1O3_ICSD_86645	0.39 eV	0.59 eV
Ca1Mn1O6Si2_ICSD_168042	3.31 eV	2.39 eV
Ca1Mn1O7P2_ICSD_78659	4.09 eV	3.33 eV
Ca1Mn2O4_ICSD_93749	1.10 eV	0.87 eV
Ca1Mo1O4_ICSD_60553	3.35 eV	2.93 eV
Ca1Mo1O6Sr2_ICSD_72815	2.11 eV	2.23 eV
Ca1Mo1O6Sr2_ICSD_72816	1.98 eV	2.55 eV
Ca1Mo4O16U1_ICSD_202234	2.63 eV	2.48 eV
Ca1N1O2Ta1_ICSD_161824	1.66 eV	1.97 eV
Ca1N2O6_ICSD_52351	3.67 eV	3.31 eV
Ca1N2Si1_ICSD_170267	3.43 eV	3.03 eV
Ca1N6_ICSD_35227	3.51 eV	3.23 eV
Ca1Na10Sn12_ICSD_240006	0.66 eV	0.64 eV
Ca1Na1O4V1_ICSD_32573	3.68 eV	3.20 eV
Ca1Na2O12V4_ICSD_161368	2.91 eV	2.70 eV
Ca1Na2O4Si1_ICSD_24235	3.38 eV	3.63 eV
Ca1Na2O7P2_ICSD_89468	4.95 eV	4.84 eV
Ca1Na2O8S2_ICSD_28429	5.45 eV	4.97 eV
Ca1Na4O18P6_ICSD_281508	5.18 eV	5.34 eV
Ca1Nb2O11P2_ICSD_72115	2.76 eV	2.96 eV
Ca1Nb2O4_ICSD_88779	1.32 eV	1.20 eV
Ca1Nb2O6_ICSD_15208	3.86 eV	3.09 eV
Ca1Ni1O6Si2_ICSD_159545	1.21 eV	3.21 eV
Ca1Ni1O6Si2_ICSD_246208	3.98 eV	2.31 eV
Ca1Ni3O14P4_ICSD_74046	3.57 eV	2.95 eV
Ca1Ni4O8_ICSD_40470	0.38 eV	1.13 eV
Ca1O11P4_ICSD_74953	5.30 eV	5.51 eV
Ca1O11Ta4_ICSD_1854	3.79 eV	3.35 eV
Ca1O1S1Zn1_ICSD_245309	3.16 eV	2.26 eV
Ca1O1_ICSD_161831	3.11 eV	3.12 eV

Supplementary Table 719. Five-fold cross validated predictions for the band gap energy (113/277).

system	calculated	predicted
Ca1O1_ICSD_90486	3.64 eV	3.16 eV
Ca1O24P6Sn4_ICSD_261116	2.98 eV	3.16 eV
Ca1O24P6Zr4_ICSD_151683	4.17 eV	4.04 eV
Ca1O2_ICSD_619462	2.72 eV	3.24 eV
Ca1O3Pt1_ICSD_173278	1.62 eV	1.61 eV
Ca1O3Rh1_ICSD_164775	0.24 eV	1.16 eV
Ca1O3Si1_ICSD_20571	4.83 eV	4.41 eV
Ca1O3Si1_ICSD_240432	3.56 eV	3.68 eV
Ca1O3Si1_ICSD_240442	3.55 eV	3.85 eV
Ca1O3Si1_ICSD_240450	3.56 eV	3.89 eV
Ca1O3Si1_ICSD_240456	3.68 eV	4.15 eV
Ca1O3Si1_ICSD_240461	3.67 eV	3.80 eV
Ca1O3Si1_ICSD_240465	3.70 eV	4.01 eV
Ca1O3Si1_ICSD_240468	3.67 eV	3.74 eV
Ca1O3Si1_ICSD_40658	3.47 eV	3.52 eV
Ca1O3Si1_ICSD_87694	4.72 eV	4.44 eV
Ca1O3Sn1_ICSD_2373	2.95 eV	2.07 eV
Ca1O3Sn1_ICSD_56095	0.69 eV	1.40 eV
Ca1O3Tc1_ICSD_261341	0.79 eV	0.96 eV
Ca1O3Te1_ICSD_260238	3.28 eV	2.81 eV
Ca1O3Ti1_ICSD_162919	2.79 eV	2.82 eV
Ca1O3Ti1_ICSD_165801	2.90 eV	2.69 eV
Ca1O3Ti1_ICSD_187294	2.36 eV	2.54 eV
Ca1O3Ti1_ICSD_29116	2.62 eV	2.86 eV
Ca1O3Ti1_ICSD_31864	2.36 eV	2.29 eV
Ca1O3Ti1_ICSD_50364	2.69 eV	2.66 eV
Ca1O3V1_ICSD_88978	0.21 eV	1.81 eV
Ca1O3Zr1_ICSD_56094	3.21 eV	3.08 eV
Ca1O3Zr1_ICSD_97466	3.82 eV	3.16 eV
Ca1O4Rh2_ICSD_170597	1.76 eV	1.08 eV
Ca1O4S1_ICSD_171053	3.13 eV	4.92 eV
Ca1O4S1_ICSD_79527	5.68 eV	5.54 eV
Ca1O4S1_ICSD_86316	5.68 eV	5.53 eV
Ca1O4Te1_ICSD_8097	2.26 eV	2.49 eV
Ca1O4U1_ICSD_31631	2.80 eV	2.83 eV
Ca1O4W1_ICSD_60548	4.10 eV	3.61 eV
Ca1O5Se2_ICSD_27209	3.49 eV	3.41 eV
Ca1O5Si1Sn1_ICSD_96819	2.16 eV	2.41 eV
Ca1O5Si1Sn1_ICSD_96820	2.26 eV	2.48 eV
Ca1O5Si1Ti1_ICSD_159343	3.38 eV	3.36 eV
Ca1O5Si2_ICSD_87498	4.78 eV	4.73 eV
Ca1O5Te2_ICSD_260041	3.36 eV	2.66 eV
Ca1O5V2_ICSD_82689	1.58 eV	1.36 eV
Ca1O6P2_ICSD_417710	5.53 eV	5.29 eV
Ca1O6P2_ICSD_60117	5.51 eV	5.40 eV
Ca1O6Sb2_ICSD_74539	2.77 eV	2.42 eV
Ca1O6Si2Zn1_ICSD_158143	4.49 eV	4.29 eV
Ca1O6Sr2W1_ICSD_36459	2.88 eV	2.96 eV
Ca1O6Sr2W1_ICSD_36460	2.91 eV	3.02 eV
Ca1O6Ta2_ICSD_24091	3.59 eV	3.19 eV

Supplementary Table 720. Five-fold cross validated predictions for the band gap energy (114/277).

system	calculated	predicted
Ca1O6Ta2_ICSD_47121	2.67 eV	3.18 eV
Ca1O6V2_ICSD_21064	2.84 eV	2.17 eV
Ca1O7Si2Zr1_ICSD_203131	4.58 eV	4.38 eV
Ca1O7V3_ICSD_241204	2.13 eV	2.08 eV
Ca1O8P2U1_ICSD_81388	3.27 eV	3.40 eV
Ca1O8P2Zn2_ICSD_202571	4.32 eV	4.42 eV
Ca1O8Te3_ICSD_100661	2.00 eV	2.32 eV
Ca1O9P2V2_ICSD_79735	1.46 eV	2.32 eV
Ca1O9V4_ICSD_2556	2.17 eV	1.97 eV
Ca1P1Pt1_ICSD_60826	0.10 eV	0.35 eV
Ca1P2Zn2_ICSD_619477	0.91 eV	0.94 eV
Ca1Pb1Sr1_ICSD_172008	0.28 eV	0.28 eV
Ca1Rb1Sb1_ICSD_409178	1.51 eV	0.82 eV
Ca1S1_ICSD_186785	2.38 eV	2.29 eV
Ca1S3Zr1_ICSD_23286	1.19 eV	1.40 eV
Ca1S4Y2_ICSD_619557	1.53 eV	1.66 eV
Ca1S4Yb2_ICSD_619559	0.56 eV	1.21 eV
Ca1Se1_ICSD_167834	3.17 eV	2.30 eV
Ca1Se1_ICSD_53948	2.08 eV	2.63 eV
Ca1Si1Sr1_ICSD_172005	0.42 eV	0.56 eV
Ca1Sn1Sr1_ICSD_172007	0.34 eV	0.35 eV
Ca1Te1_ICSD_619616	1.55 eV	1.24 eV
Ca22O36S2Si8_ICSD_26407	3.51 eV	3.64 eV
Ca2Cd1P2_ICSD_422580	1.31 eV	0.99 eV
Ca2Cd1Sb2_ICSD_173172	0.31 eV	0.28 eV
Ca2Cl1Cr1O4_ICSD_15315	0.99 eV	2.29 eV
Ca2Cl1Fe1O3_ICSD_56880	0.66 eV	1.00 eV
Ca2Cl1N1_ICSD_153101	2.38 eV	2.23 eV
Ca2Cl1O4P1_ICSD_15316	5.38 eV	4.90 eV
Ca2Cl2Cu1O10Te4_ICSD_171007	1.77 eV	1.77 eV
Ca2Cl2O3Si1_ICSD_200221	5.11 eV	4.45 eV
Ca2Co1O3_ICSD_95439	1.62 eV	1.25 eV
Ca2Co1O7Si2_ICSD_94137	3.69 eV	3.46 eV
Ca2Cr4O16P4_ICSD_412383	2.05 eV	2.64 eV
Ca2Cs1O10Ta3_ICSD_89011	2.42 eV	2.33 eV
Ca2Cu1Fe1O3S1_ICSD_169992	0.60 eV	0.89 eV
Ca2Cu1Fe1O3Se1_ICSD_169993	0.51 eV	0.73 eV
Ca2Cu1P3Zn2_ICSD_89517	0.30 eV	0.34 eV
Ca2F2Mg5O22Si8_ICSD_22020	5.01 eV	4.89 eV
Ca2F4_ICSD_246961	7.14 eV	5.92 eV
Ca2Fe1H8O12P2_ICSD_200477	3.97 eV	2.92 eV
Ca2Fe1N2_ICSD_72389	0.25 eV	0.66 eV
Ca2Fe1O6W1_ICSD_81203	2.01 eV	1.91 eV
Ca2Fe1O6W1_ICSD_81204	2.00 eV	1.80 eV
Ca2Fe2O5_ICSD_161513	0.62 eV	0.84 eV
Ca2Fe7O11_ICSD_100827	0.39 eV	0.58 eV
Ca2Fe9O13_ICSD_100826	0.57 eV	0.42 eV
Ca2Ge1O4_ICSD_16258	2.85 eV	2.96 eV
Ca2Ge1O4_ICSD_173468	3.57 eV	3.08 eV
Ca2Ge1O4_ICSD_182051	3.53 eV	3.15 eV

Supplementary Table 721. Five-fold cross validated predictions for the band gap energy (115/277).

system	calculated	predicted
Ca2Ge1S4_ICSD_619332	2.49 eV	2.44 eV
Ca2Ge1_ICSD_42455	0.32 eV	0.35 eV
Ca2Ge2O7Zn1_ICSD_69387	3.27 eV	3.12 eV
Ca2Ge7O16_ICSD_200603	2.80 eV	2.79 eV
Ca2Ge7O16_ICSD_249322	2.80 eV	2.75 eV
Ca2H18O12Re6S8_ICSD_415689	2.54 eV	2.34 eV
Ca2H1N1_ICSD_292	1.64 eV	1.81 eV
Ca2H1Na1O9Si3_ICSD_26820	4.85 eV	4.88 eV
Ca2H2Mg5O24Si8_ICSD_9659	4.86 eV	4.63 eV
Ca2H4Mg1O10P2_ICSD_4258	5.39 eV	5.11 eV
Ca2H4Mn1O10P2_ICSD_156225	4.17 eV	3.03 eV
Ca2H4O13P3V1_ICSD_165406	1.82 eV	2.48 eV
Ca2H4O9P2_ICSD_1912	5.15 eV	5.37 eV
Ca2H6Ru1_ICSD_656082	3.05 eV	2.70 eV
Ca2Hf7O16_ICSD_4136	4.32 eV	3.74 eV
Ca2I1N1_ICSD_65216	2.35 eV	1.80 eV
Ca2I1P1_ICSD_6068	1.70 eV	1.77 eV
Ca2Ir1O4_ICSD_25500	0.18 eV	0.48 eV
Ca2K2O12S3_ICSD_40988	5.31 eV	5.48 eV
Ca2K8O24U6_ICSD_91783	2.93 eV	2.95 eV
Ca2Li6Mn2N6_ICSD_408324	0.69 eV	1.56 eV
Ca2Mg1O6W1_ICSD_281564	3.43 eV	3.13 eV
Ca2Mg1O7Si2_ICSD_158177	4.47 eV	4.42 eV
Ca2Mn1O6Sb1_ICSD_246090	0.74 eV	1.53 eV
Ca2Mn1O6Ta1_ICSD_246091	0.43 eV	1.57 eV
Ca2Mn3O8_ICSD_24847	1.35 eV	0.98 eV
Ca2N2Zn1_ICSD_69049	0.69 eV	1.11 eV
Ca2N3P1_ICSD_72532	2.59 eV	2.83 eV
Ca2N3V1_ICSD_409644	1.78 eV	1.73 eV
Ca2N4Sr1W1_ICSD_401331	2.65 eV	2.14 eV
Ca2N8Si5_ICSD_79070	3.35 eV	3.51 eV
Ca2Na3O6Ta1_ICSD_280284	3.97 eV	3.32 eV
Ca2Nb2O7_ICSD_22411	1.77 eV	2.45 eV
Ca2Nb3O10Rb1_ICSD_260289	1.85 eV	1.90 eV
Ca2O10Rb1Ta3_ICSD_89010	2.45 eV	2.33 eV
Ca2O12Rb2S3_ICSD_249339	5.40 eV	5.17 eV
Ca2O12Si4Zr1_ICSD_73801	4.85 eV	4.60 eV
Ca2O20Si8Th1_ICSD_64745	4.92 eV	3.99 eV
Ca2O4Pb1_ICSD_36629	1.49 eV	1.79 eV
Ca2O4Ru1_ICSD_94244	0.97 eV	1.03 eV
Ca2O4Si1_ICSD_182052	4.16 eV	3.81 eV
Ca2O4Si1_ICSD_182054	3.30 eV	4.03 eV
Ca2O4Si1_ICSD_245074	4.26 eV	4.22 eV
Ca2O4Si1_ICSD_280995	4.41 eV	4.19 eV
Ca2O4Si1_ICSD_40657	4.32 eV	3.79 eV
Ca2O4Sn1_ICSD_9011	2.45 eV	2.22 eV
Ca2O5U1_ICSD_23198	3.13 eV	3.08 eV
Ca2O6Pd1W1_ICSD_83258	1.31 eV	1.90 eV
Ca2O6Pd1W1_ICSD_83259	1.25 eV	1.95 eV
Ca2O6Si1Ti1_ICSD_83455	2.76 eV	3.25 eV

Supplementary Table 722. Five-fold cross validated predictions for the band gap energy (116/277).

system	calculated	predicted
Ca2O7Sb2_ICSD_166282	1.74 eV	2.21 eV
Ca2O7Si2Zn1_ICSD_30262	4.33 eV	4.23 eV
Ca2O7Ta2_ICSD_27121	2.46 eV	2.69 eV
Ca2O7V2_ICSD_421266	2.90 eV	2.50 eV
Ca2O8Pt3_ICSD_65412	1.61 eV	1.52 eV
Ca2O8Se3_ICSD_54155	3.43 eV	3.48 eV
Ca2O9Pb1Si3_ICSD_18098	4.36 eV	3.61 eV
Ca2P2S6_ICSD_405192	3.08 eV	2.69 eV
Ca2P2_ICSD_83352	0.34 eV	0.94 eV
Ca2S4Si1_ICSD_619542	3.10 eV	2.96 eV
Ca2S4Sn1_ICSD_619548	2.05 eV	2.07 eV
Ca2S5Sb2_ICSD_201044	1.45 eV	1.66 eV
Ca2Sb1_ICSD_42135	0.13 eV	0.31 eV
Ca2Se4Si1_ICSD_619574	2.40 eV	2.00 eV
Ca2Si1_ICSD_187352	0.55 eV	0.42 eV
Ca2Si1_ICSD_42453	0.30 eV	0.41 eV
Ca3Cl2Ge1O4_ICSD_249329	4.16 eV	4.11 eV
Ca3Cl2O4Si1_ICSD_9088	4.41 eV	4.53 eV
Ca3Cl3P1_ICSD_202075	1.84 eV	2.58 eV
Ca3Co1O6Rh1_ICSD_50809	0.73 eV	1.27 eV
Ca3Co1O6Sc1_ICSD_245503	2.99 eV	2.44 eV
Ca3Cr1N3_ICSD_40205	0.44 eV	0.79 eV
Ca3Cr2O12Si3_ICSD_82743	3.16 eV	2.63 eV
Ca3Cr3O16P4_ICSD_412381	3.10 eV	3.05 eV
Ca3Cu2P4Zn2_ICSD_89515	0.19 eV	0.28 eV
Ca3Fe1O6Rh1_ICSD_96484	1.85 eV	1.15 eV
Ca3Fe2Ge3O12_ICSD_28176	2.17 eV	1.92 eV
Ca3Fe2O12Si3_ICSD_202964	2.39 eV	2.79 eV
Ca3Ga2N4_ICSD_170442	1.99 eV	1.91 eV
Ca3Ga2O12Si3_ICSD_27387	4.33 eV	4.30 eV
Ca3Ga3O14Si2Ta1_ICSD_245381	4.03 eV	3.51 eV
Ca3Ga4O9_ICSD_100356	2.70 eV	2.97 eV
Ca3Ge2P4_ICSD_41181	0.72 eV	0.88 eV
Ca3Ge3O12Sc2_ICSD_20215	3.44 eV	3.14 eV
Ca3Ge3O12Y2_ICSD_280048	3.22 eV	3.37 eV
Ca3Ge4_ICSD_185654	0.33 eV	0.45 eV
Ca3Hf1O9Si2_ICSD_79452	4.68 eV	4.37 eV
Ca3I3P1_ICSD_9026	2.16 eV	1.93 eV
Ca3In1P3_ICSD_60125	1.00 eV	0.77 eV
Ca3In2O12Si3_ICSD_27390	3.78 eV	3.29 eV
Ca3Li1O6Os1_ICSD_248206	1.26 eV	1.64 eV
Ca3Li1O6Ru1_ICSD_96219	1.26 eV	1.72 eV
Ca3Li4N6Si2_ICSD_420675	2.29 eV	2.48 eV
Ca3Mn1N3_ICSD_67888	0.32 eV	0.63 eV
Ca3Mn1Ni1O6_ICSD_50805	2.16 eV	1.59 eV
Ca3Mn1O6Zn1_ICSD_50808	2.35 eV	2.01 eV
Ca3Mn2O7_ICSD_55666	0.28 eV	0.38 eV
Ca3Mn2O7_ICSD_96697	0.22 eV	0.58 eV
Ca3N1P1_ICSD_106350	0.81 eV	0.94 eV
Ca3N2_ICSD_162797	1.38 eV	1.12 eV

Supplementary Table 723. Five-fold cross validated predictions for the band gap energy (117/277).

system	calculated	predicted
Ca3N2_ICSD_169726	1.36 eV	1.26 eV
Ca3N2_ICSD_169727	1.52 eV	1.28 eV
Ca3N2_ICSD_419865	0.77 eV	1.16 eV
Ca3N2_ICSD_50991	1.11 eV	1.09 eV
Ca3N3V1_ICSD_72118	1.11 eV	0.79 eV
Ca3Na1O6Ru1_ICSD_50020	1.36 eV	1.67 eV
Ca3Na2O9Ta2_ICSD_280154	3.66 eV	3.51 eV
Ca3O12Sc2Si3_ICSD_156537	4.23 eV	4.20 eV
Ca3O12Te2Zn3_ICSD_64791	2.15 eV	2.39 eV
Ca3O12Te2Zn3_ICSD_67045	2.46 eV	2.38 eV
Ca3O5Si1_ICSD_22501	3.69 eV	3.51 eV
Ca3O5Si1_ICSD_81100	3.31 eV	3.68 eV
Ca3O6Te1_ICSD_35085	1.74 eV	2.44 eV
Ca3O6Tl2_ICSD_413876	0.73 eV	1.57 eV
Ca3O6U1_ICSD_23200	3.15 eV	2.90 eV
Ca3O8P2_ICSD_158736	5.47 eV	4.90 eV
Ca3O8V2_ICSD_412273	3.43 eV	2.77 eV
Ca3O9Si2Zr1_ICSD_79453	4.46 eV	4.40 eV
Ca3O9Si3_ICSD_87716	4.71 eV	4.39 eV
Ca4Cl6O1_ICSD_33883	5.23 eV	4.86 eV
Ca4Cu3P5Zn2_ICSD_89518	0.16 eV	0.33 eV
Ca4F1Na5O16P4_ICSD_20906	4.73 eV	4.44 eV
Ca4F2O7Si2_ICSD_64710	4.67 eV	4.62 eV
Ca4Ge1N4_ICSD_280251	1.42 eV	1.67 eV
Ca4Mn3O10_ICSD_85669	0.28 eV	0.44 eV
Ca4N4Ti1_ICSD_172879	1.47 eV	1.32 eV
Ca4O1P2_ICSD_68202	1.27 eV	1.28 eV
Ca4O1Sb2_ICSD_16353	0.91 eV	1.04 eV
Ca4O6Pd1_ICSD_88134	2.03 eV	1.94 eV
Ca4O6Pt1_ICSD_59252	2.67 eV	2.20 eV
Ca5Cl1O12P3_ICSD_24237	5.30 eV	4.67 eV
Ca5Co2N4_ICSD_409920	0.63 eV	0.61 eV
Ca5F1O12P3_ICSD_52385	5.52 eV	4.55 eV
Ca5F1O12V3_ICSD_172997	3.84 eV	3.23 eV
Ca5Ga6O14_ICSD_63052	3.16 eV	3.07 eV
Ca5Ge3O11_ICSD_403085	1.87 eV	3.02 eV
Ca5H1O13P3_ICSD_180315	5.20 eV	4.56 eV
Ca5In2Sb6_ICSD_36467	0.19 eV	0.22 eV
Ca5N4O2W1_ICSD_409475	2.33 eV	2.15 eV
Ca5N6Si2_ICSD_414462	2.17 eV	2.15 eV
Ca5O14Te3_ICSD_245873	1.68 eV	2.36 eV
Ca5P8_ICSD_74854	1.14 eV	0.87 eV
Ca5Sb3_ICSD_2065	0.24 eV	0.41 eV
Ca6H6O13Si2_ICSD_39725	3.68 eV	4.16 eV
Ca6Mn1N5_ICSD_80184	0.84 eV	0.86 eV
Ca7Cl2H12_ICSD_420927	3.71 eV	3.11 eV
Ca7N9Nb1Si2_ICSD_414461	2.06 eV	1.80 eV
Cd1Cl1H1O1_ICSD_26752	1.41 eV	2.40 eV
Cd1Cl1H1O1_ICSD_91087	2.61 eV	1.90 eV
Cd1Cl1K4O15V5_ICSD_404097	2.31 eV	2.49 eV

Supplementary Table 724. Five-fold cross validated predictions for the band gap energy (118/277).

system	calculated	predicted
Cd1Cl1O15Rb4V5_ICSD_406783	2.30 eV	2.47 eV
Cd1Cl1O15Tl4V5_ICSD_406784	2.14 eV	2.46 eV
Cd1Cl1O3V1_ICSD_50788	2.66 eV	2.36 eV
Cd1Cl1S2Sb1_ICSD_171722	1.66 eV	1.65 eV
Cd1Cl2O2Pb2_ICSD_168248	2.55 eV	2.45 eV
Cd1Cl2_ICSD_62202	3.59 eV	3.10 eV
Cd1Cl3Cs1_ICSD_24484	1.93 eV	2.74 eV
Cd1Cl3Cs1_ICSD_4039	2.62 eV	3.03 eV
Cd1Cl3K1_ICSD_31357	3.58 eV	3.63 eV
Cd1Cl3Rb1_ICSD_200206	3.62 eV	2.98 eV
Cd1Cl3Rb1_ICSD_33552	2.35 eV	3.04 eV
Cd1Cl3Tl1_ICSD_39807	3.28 eV	2.85 eV
Cd1Cl4Cs2_ICSD_16576	3.32 eV	3.13 eV
Cd1Cl4Cu1H8O4_ICSD_280299	1.77 eV	2.12 eV
Cd1Cl4Na2_ICSD_69344	3.73 eV	3.70 eV
Cd1Cl4Rb2_ICSD_51168	3.33 eV	3.23 eV
Cd1Cl6K4_ICSD_60753	4.09 eV	3.91 eV
Cd1Cl6Rb4_ICSD_39622	4.04 eV	3.88 eV
Cd1Cl8Na6_ICSD_35070	4.23 eV	4.10 eV
Cd1Co1F6Li1_ICSD_32731	2.52 eV	2.44 eV
Cd1Cr1F6_ICSD_10346	1.56 eV	2.12 eV
Cd1Cr1O4_ICSD_18118	2.32 eV	2.17 eV
Cd1Cr1O4_ICSD_18119	2.43 eV	2.26 eV
Cd1Cr2H14N4O8_ICSD_80187	2.87 eV	2.70 eV
Cd1Cr2O4_ICSD_37428	2.44 eV	2.11 eV
Cd1Cr2S4_ICSD_619692	1.06 eV	0.79 eV
Cd1Cr2Se4_ICSD_42022	0.35 eV	0.47 eV
Cd1Cr4Cs2H4O16_ICSD_154326	2.70 eV	2.40 eV
Cd1Cs1Dy1Te3_ICSD_173312	1.56 eV	1.50 eV
Cd1Cs1Er1Te3_ICSD_173313	1.57 eV	1.44 eV
Cd1Cs1F3_ICSD_290344	3.56 eV	2.75 eV
Cd1Cs1Ho1Te3_ICSD_173315	1.57 eV	1.50 eV
Cd1Cs1La1Te3_ICSD_173316	1.60 eV	1.37 eV
Cd1Cs1N3O6_ICSD_95536	2.41 eV	2.63 eV
Cd1Cs1Nd1Te3_ICSD_173318	1.45 eV	1.43 eV
Cd1Cs1Se3Y1_ICSD_281433	2.13 eV	1.54 eV
Cd1Cs1Tb1Te3_ICSD_173320	1.56 eV	1.44 eV
Cd1Cs2I4_ICSD_65654	2.90 eV	2.53 eV
Cd1Cs3I5_ICSD_403103	2.88 eV	2.84 eV
Cd1Cu1F4_ICSD_73478	2.62 eV	2.19 eV
Cd1Cu1O4V1_ICSD_401454	1.43 eV	1.56 eV
Cd1Cu2Ge1S4_ICSD_26150	0.69 eV	0.76 eV
Cd1Cu2S4Si1_ICSD_619770	1.35 eV	1.37 eV
Cd1Cu2Se4Si1_ICSD_619783	0.63 eV	0.66 eV
Cd1Cu2Si1Te4_ICSD_656151	0.47 eV	0.49 eV
Cd1Cu3O12Ti4_ICSD_39467	1.22 eV	1.47 eV
Cd1Dy2S4_ICSD_52798	1.12 eV	1.26 eV
Cd1Dy2Se4_ICSD_246499	0.62 eV	0.79 eV
Cd1Er2S4_ICSD_100518	1.18 eV	1.31 eV
Cd1Er2Se4_ICSD_619834	0.67 eV	1.00 eV

Supplementary Table 725. Five-fold cross validated predictions for the band gap energy (119/277).

system	calculated	predicted
Cd1F1H1O1_ICSD_27754	2.05 eV	2.41 eV
Cd1F2_ICSD_183501	2.90 eV	2.42 eV
Cd1F2_ICSD_28864	3.05 eV	2.83 eV
Cd1F3K1_ICSD_44788	3.40 eV	3.33 eV
Cd1F3Rb1_ICSD_49587	3.47 eV	3.34 eV
Cd1F5Mn1_ICSD_69633	0.80 eV	2.07 eV
Cd1F6H12O6Sn2_ICSD_166590	4.53 eV	3.86 eV
Cd1F6Pd1_ICSD_26166	2.27 eV	2.02 eV
Cd1F6Pt1_ICSD_37445	2.69 eV	2.56 eV
Cd1F6Rh1_ICSD_42162	1.30 eV	1.62 eV
Cd1F6Sn1_ICSD_25017	3.58 eV	3.41 eV
Cd1F6Ti1_ICSD_16233	4.90 eV	3.52 eV
Cd1F7Rb1Zr1_ICSD_245783	5.20 eV	4.74 eV
Cd1F7Tl1Zr1_ICSD_245784	5.01 eV	4.76 eV
Cd1Fe2O4_ICSD_619857	1.54 eV	1.34 eV
Cd1Ga1In1S4_ICSD_20785	0.42 eV	0.98 eV
Cd1Ga2O4_ICSD_159739	1.77 eV	1.83 eV
Cd1Ga2S4_ICSD_619878	2.19 eV	1.97 eV
Cd1Ga2Se4_ICSD_30908	1.41 eV	1.30 eV
Cd1Ga2Te4_ICSD_656264	1.10 eV	0.93 eV
Cd1Ge1Li2O4_ICSD_20031	2.52 eV	2.54 eV
Cd1Ge1Li2S4_ICSD_249872	2.27 eV	2.12 eV
Cd1Ge1O3_ICSD_23531	1.36 eV	1.59 eV
Cd1Ge1O3_ICSD_30971	1.54 eV	1.46 eV
Cd1Ge1O3_ICSD_77134	1.52 eV	1.73 eV
Cd1Ge1P2_ICSD_600509	0.69 eV	0.76 eV
Cd1Ge1Te4Tl2_ICSD_172502	0.37 eV	0.37 eV
Cd1Ge2O5_ICSD_23039	1.48 eV	1.77 eV
Cd1H10N4O8S2_ICSD_240376	4.39 eV	3.81 eV
Cd1H1N1O4_ICSD_35355	3.13 eV	2.62 eV
Cd1H1O3P1_ICSD_240864	3.50 eV	3.39 eV
Cd1H2I3O1Rb1_ICSD_809	2.55 eV	2.33 eV
Cd1H2O2_ICSD_165225	1.83 eV	2.23 eV
Cd1H2O4Se1_ICSD_59347	3.93 eV	3.05 eV
Cd1H2O5Se1_ICSD_26897	2.59 eV	3.12 eV
Cd1H3N1O5_ICSD_36557	3.30 eV	3.02 eV
Cd1H4Mg1N4O10_ICSD_64618	2.57 eV	3.09 eV
Cd1H4Na2O10S2_ICSD_98681	4.08 eV	3.65 eV
Cd1H4Na2O10Se2_ICSD_98682	2.95 eV	3.22 eV
Cd1H4O4P2_ICSD_171022	4.47 eV	3.95 eV
Cd1H6N1O5P1_ICSD_200085	3.40 eV	3.80 eV
Cd1H6O7P2_ICSD_1382	4.93 eV	4.10 eV
Cd1Hg1O2_ICSD_74848	0.50 eV	1.01 eV
Cd1Hg2O6S1_ICSD_413287	1.37 eV	1.34 eV
Cd1Hg2O6Se1_ICSD_413288	0.95 eV	1.38 eV
Cd1Ho2S4_ICSD_620002	1.15 eV	1.24 eV
Cd1Ho2Se4_ICSD_620011	0.64 eV	0.76 eV
Cd1I2O6_ICSD_1397	3.28 eV	3.04 eV
Cd1I2O6_ICSD_415011	3.28 eV	3.22 eV
Cd1I2_ICSD_108921	2.39 eV	2.37 eV

Supplementary Table 726. Five-fold cross validated predictions for the band gap energy (120/277).

system	calculated	predicted
Cd1I2_ICSD_108924	2.40 eV	2.38 eV
Cd1I2_ICSD_108926	2.38 eV	2.38 eV
Cd1I2_ICSD_20745	2.36 eV	2.37 eV
Cd1I2_ICSD_28384	2.27 eV	2.32 eV
Cd1I2_ICSD_37377	2.40 eV	2.39 eV
Cd1I2_ICSD_37378	2.39 eV	2.39 eV
Cd1I2_ICSD_38116	2.37 eV	2.36 eV
Cd1I2_ICSD_42173	2.43 eV	2.30 eV
Cd1I2_ICSD_42198	2.39 eV	2.39 eV
Cd1I2_ICSD_42205	2.26 eV	2.32 eV
Cd1I2_ICSD_42207	2.38 eV	2.35 eV
Cd1I2_ICSD_42210	2.38 eV	2.39 eV
Cd1I2_ICSD_42227	2.41 eV	2.37 eV
Cd1I2_ICSD_42233	2.39 eV	2.37 eV
Cd1I2_ICSD_42258	2.39 eV	2.39 eV
Cd1I2_ICSD_42262	2.37 eV	2.38 eV
Cd1I2_ICSD_42268	2.40 eV	2.37 eV
Cd1I2_ICSD_42269	2.43 eV	2.27 eV
Cd1I2_ICSD_43443	2.40 eV	2.37 eV
Cd1I2_ICSD_43852	2.46 eV	2.45 eV
Cd1I2_ICSD_44792	2.39 eV	2.39 eV
Cd1I2_ICSD_44793	2.38 eV	2.38 eV
Cd1I2_ICSD_9025	2.38 eV	2.38 eV
Cd1I2_ICSD_9190	2.37 eV	2.37 eV
Cd1I4S8Sb6_ICSD_27733	1.55 eV	1.41 eV
Cd1I6In4_ICSD_66290	1.51 eV	1.69 eV
Cd1I6Tl4_ICSD_66292	2.18 eV	1.88 eV
Cd1In2O4_ICSD_159740	1.08 eV	1.20 eV
Cd1In2O4_ICSD_52389	1.08 eV	1.08 eV
Cd1In2S4_ICSD_601181	1.20 eV	1.02 eV
Cd1In2Se4_ICSD_151954	1.04 eV	0.93 eV
Cd1In2Se4_ICSD_151955	0.82 eV	0.84 eV
Cd1In2Se4_ICSD_620049	0.94 eV	0.93 eV
Cd1In2Te4_ICSD_25651	0.90 eV	0.72 eV
Cd1Ir1O6Sr3_ICSD_81904	0.37 eV	0.61 eV
Cd1K1N3O6_ICSD_28647	0.86 eV	1.62 eV
Cd1K1N3O6_ICSD_95538	2.31 eV	2.31 eV
Cd1K2N12_ICSD_659621	2.85 eV	3.00 eV
Cd1K2O2_ICSD_25004	1.41 eV	1.44 eV
Cd1K2O7P2_ICSD_12117	3.86 eV	3.50 eV
Cd1K2Sb2Sr1_ICSD_422273	0.36 eV	0.48 eV
Cd1K2Se4Sn1_ICSD_185468	1.57 eV	1.36 eV
Cd1K4P2_ICSD_61084	0.83 eV	0.92 eV
Cd1K6O4_ICSD_62053	1.62 eV	1.62 eV
Cd1K6Te4_ICSD_420087	1.68 eV	1.54 eV
Cd1Li1O4P1_ICSD_65050	3.36 eV	3.36 eV
Cd1Li1O4P1_ICSD_71862	3.35 eV	3.36 eV
Cd1Li2S4Sn1_ICSD_249873	1.77 eV	1.96 eV
Cd1Lu2S4_ICSD_620127	1.27 eV	1.33 eV
Cd1Lu2Se4_ICSD_620129	0.75 eV	0.98 eV

Supplementary Table 727. Five-fold cross validated predictions for the band gap energy (121/277).

system	calculated	predicted
Cd1Mn2O4_ICSD_24258	0.70 eV	0.89 eV
Cd1Mo1O4_ICSD_30371	2.39 eV	2.37 eV
Cd1Mo1O6P1_ICSD_82090	1.80 eV	2.30 eV
Cd1Mo1O6Te1_ICSD_93794	3.12 eV	2.91 eV
Cd1Mo3O12Th1_ICSD_26012	3.59 eV	2.91 eV
Cd1N2O6_ICSD_297	3.45 eV	2.96 eV
Cd1N3O6Rb1_ICSD_95537	2.35 eV	2.36 eV
Cd1N3O6Tl1_ICSD_28650	0.66 eV	1.60 eV
Cd1N3O6Tl1_ICSD_95539	2.15 eV	2.17 eV
Cd1Na1O4P1_ICSD_10153	2.72 eV	3.13 eV
Cd1Na1O4P1_ICSD_6210	3.04 eV	2.99 eV
Cd1Na1O4V1_ICSD_151411	3.11 eV	2.63 eV
Cd1Na2O8S2_ICSD_173033	3.91 eV	3.37 eV
Cd1Na2S4Sn1_ICSD_281233	1.79 eV	1.64 eV
Cd1Na2Sb2Yb1_ICSD_422274	0.32 eV	0.32 eV
Cd1Na4P2_ICSD_67262	0.73 eV	0.88 eV
Cd1O14P4V2_ICSD_74554	1.61 eV	1.94 eV
Cd1O2Rb2_ICSD_62054	1.36 eV	1.43 eV
Cd1O2_ICSD_36151	1.42 eV	1.15 eV
Cd1O3S1_ICSD_62640	3.43 eV	3.12 eV
Cd1O3S1_ICSD_62641	3.31 eV	3.23 eV
Cd1O3Se1_ICSD_75273	3.16 eV	2.92 eV
Cd1O3Se1_ICSD_75274	3.49 eV	3.19 eV
Cd1O3Sn1_ICSD_180401	0.45 eV	0.84 eV
Cd1O3Te1_ICSD_60067	3.10 eV	2.35 eV
Cd1O3Ti1_ICSD_15989	3.14 eV	2.70 eV
Cd1O3Ti1_ICSD_16686	2.84 eV	2.71 eV
Cd1O3Ti1_ICSD_62151	2.62 eV	2.46 eV
Cd1O3V1_ICSD_88387	1.42 eV	2.07 eV
Cd1O4Rh2_ICSD_28954	1.89 eV	1.80 eV
Cd1O4S1_ICSD_130014	2.22 eV	2.60 eV
Cd1O4S1_ICSD_60571	3.00 eV	2.23 eV
Cd1O4S1_ICSD_9723	1.61 eV	2.63 eV
Cd1O4U1_ICSD_20504	2.68 eV	2.43 eV
Cd1O4U1_ICSD_26427	2.64 eV	2.63 eV
Cd1O4U1_ICSD_26428	2.68 eV	2.46 eV
Cd1O4W1_ICSD_186159	3.07 eV	2.65 eV
Cd1O5Se2_ICSD_75230	2.98 eV	2.62 eV
Cd1O6P2_ICSD_15861	4.02 eV	3.81 eV
Cd1O6P2_ICSD_8070	3.80 eV	3.78 eV
Cd1O6Pt1Sr3_ICSD_280518	2.41 eV	2.47 eV
Cd1O6Pt3_ICSD_35407	0.58 eV	0.66 eV
Cd1O6Sb2_ICSD_181929	1.46 eV	1.34 eV
Cd1O6Sr2W1_ICSD_245684	3.22 eV	3.03 eV
Cd1O6Sr2W1_ICSD_71839	3.24 eV	3.08 eV
Cd1O6Sr2W1_ICSD_71840	3.07 eV	3.03 eV
Cd1O6V2_ICSD_15926	2.28 eV	2.18 eV
Cd1O7S2_ICSD_63067	2.94 eV	3.03 eV
Cd1P1S3_ICSD_61393	1.79 eV	1.69 eV
Cd1P1S3_ICSD_80875	1.83 eV	1.69 eV

Supplementary Table 728. Five-fold cross validated predictions for the band gap energy (122/277).

system	calculated	predicted
Cd1P2Rb2Se6_ICSD_50959	0.28 eV	1.33 eV
Cd1P2Si1_ICSD_44260	1.41 eV	1.13 eV
Cd1P2_ICSD_26199	1.48 eV	1.37 eV
Cd1P2_ICSD_620210	1.45 eV	1.45 eV
Cd1P2_ICSD_62336	1.45 eV	1.46 eV
Cd1P4_ICSD_25605	0.63 eV	0.93 eV
Cd1S1_ICSD_31075	1.19 eV	0.99 eV
Cd1S1_ICSD_600773	0.42 eV	0.83 eV
Cd1S1_ICSD_60629	1.26 eV	0.99 eV
Cd1S1_ICSD_620315	0.41 eV	0.72 eV
Cd1S2_ICSD_620305	1.26 eV	1.46 eV
Cd1S4Sc2_ICSD_94994	1.28 eV	1.37 eV
Cd1S4Y2_ICSD_620371	1.15 eV	1.08 eV
Cd1Sb1_ICSD_620395	0.15 eV	0.14 eV
Cd1Sc2Se4_ICSD_620411	0.77 eV	0.88 eV
Cd1Se1_ICSD_180931	0.64 eV	0.83 eV
Cd1Se1_ICSD_415785	0.69 eV	0.81 eV
Cd1Se2_ICSD_620416	0.69 eV	0.74 eV
Cd1Se4Y2_ICSD_620457	0.65 eV	0.69 eV
Cd1Sn1Te4Tl2_ICSD_172503	0.24 eV	0.42 eV
Cd1Te1_ICSD_150941	0.74 eV	0.66 eV
Cd1Te1_ICSD_620514	0.71 eV	0.74 eV
Cd1Te1_ICSD_67863	0.31 eV	0.56 eV
Cd1Te2Zn1_ICSD_181019	1.03 eV	0.95 eV
Cd1Tm1_ICSD_620576	0.19 eV	0.56 eV
Cd2Cl1H3O3_ICSD_39285	2.24 eV	2.66 eV
Cd2Cl1P3_ICSD_100818	1.23 eV	1.31 eV
Cd2Cl1P3_ICSD_100879	1.24 eV	1.28 eV
Cd2Cl2P1_ICSD_412647	1.14 eV	1.64 eV
Cd2Cu1O8P2_ICSD_249777	1.89 eV	1.63 eV
Cd2Eu1P2_ICSD_78987	0.49 eV	0.45 eV
Cd2F1O4P1_ICSD_2361	2.51 eV	2.46 eV
Cd2H2O6S1_ICSD_652	2.81 eV	2.91 eV
Cd2H4O4_ICSD_40186	1.85 eV	1.97 eV
Cd2I1P3_ICSD_100816	1.14 eV	1.16 eV
Cd2I1P3_ICSD_100881	1.14 eV	1.14 eV
Cd2I7Tl3_ICSD_63340	1.78 eV	2.15 eV
Cd2Ir1Na3O6_ICSD_404507	0.47 eV	1.08 eV
Cd2K2O12S3_ICSD_200291	3.83 eV	3.72 eV
Cd2K2O12S3_ICSD_40279	3.80 eV	3.89 eV
Cd2K2O3_ICSD_16223	0.78 eV	1.44 eV
Cd2Mn3O8_ICSD_16957	1.15 eV	1.09 eV
Cd2Na14O9_ICSD_2195	1.43 eV	1.61 eV
Cd2Na4O10Si3_ICSD_20185	2.86 eV	3.04 eV
Cd2Nb2O7_ICSD_169000	2.08 eV	2.18 eV
Cd2Nb2O7_ICSD_33672	1.96 eV	2.24 eV
Cd2Nb2O7_ICSD_75601	2.08 eV	2.01 eV
Cd2O12Rb2S3_ICSD_281315	3.80 eV	3.75 eV
Cd2O12S3Tl2_ICSD_66714	4.13 eV	3.45 eV
Cd2O4Si1_ICSD_161025	1.16 eV	1.53 eV

Supplementary Table 729. Five-fold cross validated predictions for the band gap energy (123/277).

system	calculated	predicted
Cd2O4Si1_ICSD_50531	1.58 eV	1.47 eV
Cd2O4Sn1_ICSD_69297	0.35 eV	0.76 eV
Cd2O5U1_ICSD_166737	2.79 eV	2.42 eV
Cd2O7P2_ICSD_23542	2.82 eV	2.82 eV
Cd2O7Sb2_ICSD_165168	0.48 eV	0.86 eV
Cd2O7Sb2_ICSD_77064	0.65 eV	0.72 eV
Cd2O7Ta2_ICSD_77061	2.12 eV	2.53 eV
Cd2O7V2_ICSD_24191	2.62 eV	2.58 eV
Cd2P2Se6_ICSD_620234	1.13 eV	1.14 eV
Cd2P2Sr1_ICSD_30912	0.81 eV	0.74 eV
Cd2Sb2Sr1_ICSD_12153	0.12 eV	0.19 eV
Cd2Sb2Yb1_ICSD_78989	0.14 eV	0.23 eV
Cd3Cl2O2_ICSD_300028	1.31 eV	1.86 eV
Cd3Cl3P1_ICSD_201025	1.82 eV	1.48 eV
Cd3Cs2K2O5_ICSD_60895	1.33 eV	1.66 eV
Cd3Ge3O12Sc2_ICSD_20216	2.29 eV	2.45 eV
Cd3K2Se4_ICSD_710063	1.48 eV	1.36 eV
Cd3Na2O10Si3_ICSD_28416	2.57 eV	2.65 eV
Cd3Na2O14P4_ICSD_93593	3.14 eV	3.43 eV
Cd3O10Se3_ICSD_280951	3.18 eV	3.08 eV
Cd3O5Si1_ICSD_23170	1.11 eV	1.46 eV
Cd3O6Te1_ICSD_35084	0.28 eV	1.59 eV
Cd3O8P2_ICSD_20202	2.32 eV	2.73 eV
Cd3Sb3Tl2_ICSD_76500	0.13 eV	0.17 eV
Cd4Cl3P2_ICSD_50938	1.37 eV	1.39 eV
Cd4Cl6O13Te6_ICSD_171334	3.29 eV	2.91 eV
Cd4F6O1_ICSD_74031	2.06 eV	2.18 eV
Cd4Ge1S6_ICSD_26214	1.41 eV	1.20 eV
Cd4Ge1Se6_ICSD_600752	0.79 eV	0.83 eV
Cd4I3P2_ICSD_23307	1.42 eV	1.32 eV
Cd4I3Sb2_ICSD_80589	1.15 eV	1.09 eV
Cd4K1O12V3_ICSD_807	2.46 eV	2.53 eV
Cd4K6Se13Sn3_ICSD_413739	1.21 eV	1.26 eV
Cd4N12P6S1_ICSD_71019	3.07 eV	2.99 eV
Cd4S6Si1_ICSD_620339	1.51 eV	1.70 eV
Cd4Se6Si1_ICSD_96265	0.87 eV	1.08 eV
Cd5Cl1O12P3_ICSD_130	2.23 eV	2.43 eV
Cd6Sb12Sr11_ICSD_413701	0.33 eV	0.25 eV
Cd7Cl6P4_ICSD_38044	1.78 eV	1.49 eV
Cd7P10_ICSD_200596	1.12 eV	0.87 eV
Ce1Cl1O1_ICSD_72154	3.96 eV	3.49 eV
Ce1Cl1Te1_ICSD_426281	0.83 eV	0.83 eV
Ce1Cl3_ICSD_31575	4.25 eV	3.76 eV
Ce1Cl6Cs2_ICSD_14339	2.27 eV	3.60 eV
Ce1Cr1O3_ICSD_28931	1.77 eV	1.64 eV
Ce1Cr1S3_ICSD_88975	0.44 eV	0.53 eV
Ce1Cs1Cu1S3_ICSD_156245	1.15 eV	1.37 eV
Ce1Cs1Hg1Se3_ICSD_281442	0.09 eV	1.09 eV
Ce1Cs1S2_ICSD_73533	2.28 eV	2.19 eV
Ce1Cu1O1S1_ICSD_96344	2.00 eV	1.72 eV

Supplementary Table 730. Five-fold cross validated predictions for the band gap energy (124/277).

system	calculated	predicted
Ce1Cu1O1Te1_ICSD_416521	1.56 eV	1.24 eV
Ce1Cu1S2_ICSD_415076	1.36 eV	1.23 eV
Ce1Cu1Se2_ICSD_99676	0.95 eV	0.87 eV
Ce1Dy1S3_ICSD_200015	0.80 eV	0.86 eV
Ce1Er3S6_ICSD_620979	1.01 eV	1.10 eV
Ce1F3_ICSD_4	4.76 eV	4.89 eV
Ce1F3_ICSD_42470	5.73 eV	5.52 eV
Ce1F3_ICSD_64720	4.88 eV	4.64 eV
Ce1F4K1_ICSD_23229	5.35 eV	4.99 eV
Ce1F7Zr1_ICSD_39776	4.22 eV	5.13 eV
Ce1Fe4P12_ICSD_621050	0.59 eV	0.46 eV
Ce1H8O12S2_ICSD_417358	1.59 eV	2.70 eV
Ce1H8O7P3_ICSD_88339	4.94 eV	3.93 eV
Ce1I1S1_ICSD_23490	0.93 eV	1.60 eV
Ce1I4O12_ICSD_20033	2.00 eV	2.09 eV
Ce1K1S2_ICSD_621439	2.40 eV	2.32 eV
Ce1K1S4Si1_ICSD_87946	2.66 eV	2.59 eV
Ce1K1S4Si1_ICSD_95774	2.51 eV	2.54 eV
Ce1K2O8P2_ICSD_173150	2.02 eV	3.31 eV
Ce1K3O8P2_ICSD_65193	4.92 eV	3.99 eV
Ce1Li1O2_ICSD_47116	2.88 eV	2.81 eV
Ce1Li2N2_ICSD_34003	1.83 eV	1.63 eV
Ce1Li2P2_ICSD_42016	0.77 eV	0.74 eV
Ce1Li8O6_ICSD_61219	3.44 eV	3.15 eV
Ce1Mo6Se8_ICSD_621544	0.21 eV	0.54 eV
Ce1N1O2Si1_ICSD_9751	2.51 eV	2.22 eV
Ce1N2Sr1_ICSD_95805	1.02 eV	1.14 eV
Ce1Na1Se2_ICSD_621570	1.91 eV	1.70 eV
Ce1Nb1O4_ICSD_414675	0.10 eV	2.48 eV
Ce1O16Zr7_ICSD_164737	2.72 eV	2.80 eV
Ce1O1P1Zn1_ICSD_416475	1.37 eV	1.24 eV
Ce1O1Sb1Zn1_ICSD_85449	0.21 eV	0.52 eV
Ce1O2_ICSD_169031	2.50 eV	1.69 eV
Ce1O2_ICSD_189287	1.13 eV	1.90 eV
Ce1O2_ICSD_28753	2.42 eV	2.32 eV
Ce1O3Sr1_ICSD_154927	2.93 eV	3.00 eV
Ce1O3V1_ICSD_162747	0.81 eV	1.15 eV
Ce1O4P1_ICSD_182582	1.97 eV	2.78 eV
Ce1O4P1_ICSD_182586	2.73 eV	3.39 eV
Ce1O4P1_ICSD_184550	5.02 eV	4.23 eV
Ce1O4P1_ICSD_22265	2.30 eV	3.25 eV
Ce1O4P1_ICSD_27859	4.59 eV	4.80 eV
Ce1O4P1_ICSD_31563	4.95 eV	4.63 eV
Ce1O4P1_ICSD_39135	4.70 eV	3.89 eV
Ce1O4Si1_ICSD_92040	2.03 eV	3.29 eV
Ce1O4Sr2_ICSD_86768	2.88 eV	2.23 eV
Ce1O4Ta1_ICSD_415427	2.87 eV	3.25 eV
Ce1O4V1_ICSD_183203	0.92 eV	1.83 eV
Ce1O4V1_ICSD_78075	2.40 eV	2.10 eV
Ce1O4Zr1_ICSD_164740	2.40 eV	2.66 eV

Supplementary Table 731. Five-fold cross validated predictions for the band gap energy (125/277).

system	calculated	predicted
Ce1O6Se2_ICSD_60778	1.99 eV	2.31 eV
Ce1O7P2_ICSD_160452	2.24 eV	3.72 eV
Ce1O8Zr3_ICSD_164738	2.43 eV	2.76 eV
Ce1O9P3_ICSD_417805	5.35 eV	4.73 eV
Ce1O9Ta3_ICSD_66281	2.81 eV	3.02 eV
Ce1Os4P12_ICSD_621731	0.33 eV	0.47 eV
Ce1P12Ru4_ICSD_621765	0.34 eV	0.33 eV
Ce1P1S1_ICSD_621769	0.21 eV	0.39 eV
Ce1P2_ICSD_621753	0.35 eV	0.37 eV
Ce1P5_ICSD_621759	0.12 eV	0.26 eV
Ce1Rb1S2_ICSD_81395	2.39 eV	2.31 eV
Ce1Rb1Se2_ICSD_281068	2.03 eV	1.73 eV
Ce1S2_ICSD_418404	0.35 eV	0.37 eV
Ce1S2_ICSD_653996	0.35 eV	0.38 eV
Ce1S3Sc1_ICSD_400014	2.13 eV	1.66 eV
Ce1S3Yb1_ICSD_622073	0.18 eV	0.60 eV
Ce1Se2Tl1_ICSD_622131	0.46 eV	0.52 eV
Ce1Se2Tl1_ICSD_622133	0.47 eV	0.53 eV
Ce1Se3Yb1_ICSD_99666	0.09 eV	0.47 eV
Ce1Te2Tl1_ICSD_622280	0.15 eV	0.41 eV
Ce2Cl3N1_ICSD_261615	3.38 eV	2.88 eV
Ce2F4Se1_ICSD_21011	3.33 eV	3.55 eV
Ce2Fe2O3S2_ICSD_181167	1.15 eV	1.20 eV
Ce2Ge3O9_ICSD_83342	2.46 eV	2.29 eV
Ce2H1Li1O3_ICSD_56745	1.64 eV	1.77 eV
Ce2Mn2O3Se2_ICSD_181935	1.74 eV	1.53 eV
Ce2N2Se1_ICSD_421995	1.06 eV	1.25 eV
Ce2O2S1_ICSD_154584	2.47 eV	2.47 eV
Ce2O2Te1_ICSD_9101	1.87 eV	1.51 eV
Ce2O3_ICSD_154587	2.82 eV	2.63 eV
Ce2O3_ICSD_160221	2.67 eV	2.58 eV
Ce2O3_ICSD_26865	0.29 eV	1.48 eV
Ce2O3_ICSD_96202	2.88 eV	2.54 eV
Ce2O4Se1Si1_ICSD_59941	2.61 eV	2.45 eV
Ce2O7Zr2_ICSD_168595	2.98 eV	2.97 eV
Ce2O9Si1Ti2_ICSD_410201	2.61 eV	2.73 eV
Ce2S3_ICSD_89499	0.77 eV	0.59 eV
Ce2S5Si1_ICSD_97318	2.41 eV	2.19 eV
Ce3Cl1S8Si2_ICSD_412222	2.75 eV	2.73 eV
Ce3Cl3O6Ta1_ICSD_202554	0.92 eV	2.53 eV
Ce3Cu1Ge1S7_ICSD_620858	1.72 eV	1.66 eV
Ce3Cu1Ge1Se7_ICSD_620859	1.23 eV	1.18 eV
Ce3Cu1S7Si1_ICSD_418242	1.65 eV	1.70 eV
Ce3Cu1S7Sn1_ICSD_155937	1.64 eV	1.50 eV
Ce3Cu1Se7Si1_ICSD_156282	1.40 eV	1.38 eV
Ce3Cu1Se7Sn1_ICSD_152824	1.10 eV	0.90 eV
Ce3I1S8Si2_ICSD_86173	2.58 eV	2.50 eV
Ce3N1S3_ICSD_416218	1.02 eV	1.39 eV
Ce3O16Zr5_ICSD_164739	2.38 eV	2.73 eV
Ce3O8Zr1_ICSD_164742	2.30 eV	2.43 eV

Supplementary Table 732. Five-fold cross validated predictions for the band gap energy (126/277).

system	calculated	predicted
Ce3Pt3Sb4_ICSD_621896	0.44 eV	0.35 eV
Ce4Cl3N1S3_ICSD_420115	3.04 eV	2.26 eV
Ce4Cr1S7_ICSD_603696	0.38 eV	0.61 eV
Ce4Cu1S7_ICSD_620904	0.94 eV	1.38 eV
Ce4O4S3_ICSD_2578	1.39 eV	1.81 eV
Ce4O4Se3_ICSD_419127	1.24 eV	1.35 eV
Ce5O16Zr3_ICSD_164741	2.39 eV	2.41 eV
Ce7O12_ICSD_88754	2.12 eV	2.37 eV
Ce7O16Zr1_ICSD_164743	2.33 eV	2.39 eV
Cl10Co1Tb6_ICSD_424437	0.11 eV	1.06 eV
Cl10Cs3O1Re2_ICSD_31	0.35 eV	1.52 eV
Cl10Fe1W2_ICSD_422521	0.64 eV	1.28 eV
Cl10Gd6O12Si4_ICSD_416000	4.41 eV	3.98 eV
Cl10K4O1Re2_ICSD_108904	0.82 eV	1.79 eV
Cl10K4O1Ru2_ICSD_8054	0.92 eV	1.87 eV
Cl10K4O1W2_ICSD_1923	1.22 eV	2.14 eV
Cl10Mo2N4S4_ICSD_47102	0.79 eV	1.33 eV
Cl10Mo2S4Se2_ICSD_84359	2.03 eV	1.68 eV
Cl10Nb1P1_ICSD_26096	2.32 eV	2.35 eV
Cl10Nb2O1Te4_ICSD_49920	1.25 eV	1.80 eV
Cl10Nb2P8S20_ICSD_416095	1.39 eV	1.74 eV
Cl10Nb2_ICSD_16757	2.43 eV	2.11 eV
Cl10O12Si4Y6_ICSD_94464	4.55 eV	4.25 eV
Cl10O7Si6_ICSD_409628	5.52 eV	4.76 eV
Cl10P1Ta1_ICSD_26097	2.32 eV	2.34 eV
Cl10P1U1_ICSD_35464	2.55 eV	2.45 eV
Cl10S4Se2W2_ICSD_84360	2.39 eV	1.44 eV
Cl10U2_ICSD_26089	2.23 eV	2.51 eV
Cl10W2_ICSD_2398	0.76 eV	0.87 eV
Cl11Cs1Hg5_ICSD_39767	2.73 eV	2.55 eV
Cl11Hg5Tl1_ICSD_14108	2.67 eV	2.57 eV
Cl11K1Nb2_ICSD_420871	2.41 eV	2.10 eV
Cl11N1Re2S2_ICSD_38379	0.86 eV	1.70 eV
Cl11N3P4_ICSD_71913	2.56 eV	3.31 eV
Cl12Hf1Se2_ICSD_404545	2.79 eV	2.68 eV
Cl12I2Nb6_ICSD_65968	1.09 eV	0.80 eV
Cl12Mo1Se2_ICSD_404535	0.99 eV	1.34 eV
Cl12Mo1Te2_ICSD_404224	0.73 eV	1.07 eV
Cl12N1P2Ta1_ICSD_280896	2.91 eV	2.32 eV
Cl12O2S2Ti2_ICSD_410940	2.85 eV	2.81 eV
Cl12O2S2Ti2_ICSD_410941	2.80 eV	2.55 eV
Cl12O2S2Zr2_ICSD_16128	3.24 eV	2.92 eV
Cl12O4P2Sb2_ICSD_49749	2.27 eV	2.46 eV
Cl12Os1S2_ICSD_74680	1.02 eV	1.72 eV
Cl12Os1Te2_ICSD_74682	0.94 eV	1.49 eV
Cl12Re1Te2_ICSD_68656	1.82 eV	1.58 eV
Cl12Re2S1_ICSD_411053	1.44 eV	1.69 eV
Cl12Sc7_ICSD_36424	0.60 eV	2.66 eV
Cl12Se2Zr1_ICSD_404533	2.82 eV	2.43 eV
Cl12Ta2Te4_ICSD_401907	1.16 eV	1.31 eV

Supplementary Table 733. Five-fold cross validated predictions for the band gap energy (127/277).

system	calculated	predicted
Cl12Te4W2_ICSD_66023	0.17 eV	0.79 eV
Cl12Te4W2_ICSD_74789	0.32 eV	0.83 eV
Cl13Li1Mo6_ICSD_410368	2.76 eV	1.63 eV
Cl14Hg1Mo6_ICSD_26046	1.94 eV	2.28 eV
Cl14Mo4O4Tl2_ICSD_408774	1.94 eV	1.77 eV
Cl14Mo6Pb1_ICSD_36572	2.90 eV	2.10 eV
Cl14Nb6O4Ti2_ICSD_406839	1.07 eV	1.19 eV
Cl14O8P4V2_ICSD_420125	2.04 eV	2.26 eV
Cl15Co1Zr6_ICSD_71148	1.03 eV	1.18 eV
Cl16Ge6_ICSD_86673	3.15 eV	3.05 eV
Cl16Mo1Te3_ICSD_404954	0.92 eV	1.18 eV
Cl16O6P4Sn2_ICSD_26182	2.80 eV	2.66 eV
Cl16Re4S4Te4_ICSD_165333	2.40 eV	2.11 eV
Cl16Re4Se4Te4_ICSD_165334	2.34 eV	2.18 eV
Cl16Re4Te8_ICSD_165335	2.26 eV	2.07 eV
Cl16Ru2S6_ICSD_410112	2.03 eV	1.82 eV
Cl16Si6_ICSD_2767	4.76 eV	3.57 eV
Cl17P1W6_ICSD_422270	0.33 eV	0.75 eV
Cl18Cs2Pb1Ta6_ICSD_88711	1.41 eV	1.28 eV
Cl18Cs2W6_ICSD_109513	0.79 eV	0.98 eV
Cl18Cu2Nb6Rb2_ICSD_402275	1.12 eV	1.03 eV
Cl18In2Li2Nb6_ICSD_75071	0.97 eV	1.65 eV
Cl18In4Ta6_ICSD_78835	1.24 eV	1.28 eV
Cl18K2Mn1Nb6_ICSD_82102	1.08 eV	1.04 eV
Cl18K2Nb6Sr1_ICSD_280933	1.10 eV	1.29 eV
Cl18K2W6_ICSD_260305	0.71 eV	0.81 eV
Cl18K4Nb6_ICSD_41118	1.11 eV	1.29 eV
Cl18K4Ta6_ICSD_59851	1.36 eV	1.37 eV
Cl18Mo2P2_ICSD_410649	0.90 eV	1.36 eV
Cl18Nb6Rb4_ICSD_402272	1.13 eV	1.04 eV
Cl18Nb6Tl2V1_ICSD_96347	0.18 eV	1.09 eV
Cl18P2Re2_ICSD_410188	1.55 eV	1.66 eV
Cl18P2Sn2_ICSD_60093	2.29 eV	2.36 eV
Cl18P2Ti2_ICSD_23137	2.24 eV	2.61 eV
Cl18Rb2W6_ICSD_260304	0.75 eV	0.86 eV
Cl18W6_ICSD_51513	0.14 eV	0.61 eV
Cl1Co1O3Sr2_ICSD_91750	0.26 eV	0.97 eV
Cl1Cr1Cs1O3_ICSD_6076	2.78 eV	2.67 eV
Cl1Cr1K1O3_ICSD_26591	2.77 eV	2.74 eV
Cl1Cr1O1_ICSD_4086	1.71 eV	1.81 eV
Cl1Cr1O3Rb1_ICSD_9030	2.79 eV	2.73 eV
Cl1Cr3O12Sr5_ICSD_37096	0.99 eV	2.10 eV
Cl1Cs1F3Sb1_ICSD_200296	3.65 eV	3.83 eV
Cl1Cs1Mn2O7V2_ICSD_418764	1.95 eV	1.89 eV
Cl1Cs1N1O4Tc1_ICSD_65802	2.30 eV	2.54 eV
Cl1Cs1O4_ICSD_33566	5.33 eV	4.20 eV
Cl1Cs1O4_ICSD_63364	5.48 eV	4.89 eV
Cl1Cs1_ICSD_52274	4.84 eV	4.92 eV
Cl1Cs1_ICSD_655032	5.01 eV	4.61 eV
Cl1Cs3O1_ICSD_401664	1.60 eV	1.67 eV

Supplementary Table 734. Five-fold cross validated predictions for the band gap energy (128/277).

system	calculated	predicted
Cl1Cu1F10_ICSD_35387	0.29 eV	1.62 eV
Cl1Cu1H1O1_ICSD_91088	1.02 eV	1.40 eV
Cl1Cu1H3N1_ICSD_170946	2.36 eV	1.93 eV
Cl1Cu1Hg1S1_ICSD_412310	0.83 eV	0.96 eV
Cl1Cu1Hg1Se1_ICSD_16450	0.93 eV	1.02 eV
Cl1Cu1K4O15V5_ICSD_401042	1.08 eV	1.45 eV
Cl1Cu1La1Nb2O7_ICSD_88032	0.22 eV	0.95 eV
Cl1Cu1La1O7Ta2_ICSD_51261	0.25 eV	1.00 eV
Cl1Cu1Se2_ICSD_68292	1.03 eV	1.05 eV
Cl1Cu1Te2_ICSD_641	0.73 eV	0.98 eV
Cl1Cu1_ICSD_60711	1.28 eV	1.23 eV
Cl1Cu1_ICSD_78272	1.02 eV	1.26 eV
Cl1Cu1_ICSD_78273	1.14 eV	1.26 eV
Cl1Cu3S3Te1_ICSD_85789	1.01 eV	1.12 eV
Cl1Dy1H2O2_ICSD_260833	4.65 eV	4.63 eV
Cl1Dy1Mo1O4_ICSD_420052	2.64 eV	2.65 eV
Cl1Dy1O3Se1_ICSD_418537	4.37 eV	4.34 eV
Cl1Dy1O3Ti1_ICSD_73096	3.60 eV	2.70 eV
Cl1Dy1S1_ICSD_417434	2.76 eV	2.55 eV
Cl1Er1H2O2_ICSD_260835	4.71 eV	4.45 eV
Cl1Er1Mo1O4_ICSD_420053	2.61 eV	2.33 eV
Cl1Er1O3Se1_ICSD_412861	4.36 eV	4.34 eV
Cl1Er1S1_ICSD_21009	2.93 eV	2.50 eV
Cl1Eu1F1_ICSD_108938	3.03 eV	2.50 eV
Cl1Eu1O3Ti1_ICSD_73095	0.18 eV	1.91 eV
Cl1F10Sb1_ICSD_280731	2.32 eV	2.76 eV
Cl1F1Na6O8S2_ICSD_29330	4.81 eV	4.41 eV
Cl1F1O1Pb2_ICSD_200213	2.56 eV	2.48 eV
Cl1F1O7Pb2Se2Ti1_ICSD_189152	2.89 eV	2.76 eV
Cl1F1Pb1_ICSD_155010	3.47 eV	3.20 eV
Cl1F1Sn1_ICSD_647	3.46 eV	2.80 eV
Cl1F1Sr1_ICSD_159278	5.94 eV	5.56 eV
Cl1F1Yb1_ICSD_35390	3.16 eV	2.04 eV
Cl1F1_ICSD_406442	2.07 eV	2.82 eV
Cl1F3K1Sb1_ICSD_20656	4.42 eV	4.02 eV
Cl1F3Sn2_ICSD_200032	3.54 eV	3.28 eV
Cl1F3_ICSD_19079	2.57 eV	3.08 eV
Cl1F4N1O1_ICSD_50216	1.60 eV	2.54 eV
Cl1F5Ge1O2_ICSD_60108	1.74 eV	3.31 eV
Cl1F5N1Re1_ICSD_33542	2.08 eV	2.51 eV
Cl1F6K3Re1_ICSD_80170	3.31 eV	2.61 eV
Cl1F6O2Sb1_ICSD_173609	1.93 eV	2.25 eV
Cl1F8Nb1_ICSD_39501	3.39 eV	4.07 eV
Cl1F8Sb1_ICSD_9899	3.16 eV	2.59 eV
Cl1F8Ta1_ICSD_39502	3.40 eV	4.23 eV
Cl1Fe1Li1Mo1O4_ICSD_65346	2.23 eV	2.00 eV
Cl1Fe1Mo1O4_ICSD_202187	1.93 eV	2.06 eV
Cl1Fe1N3O3_ICSD_281495	1.47 eV	2.08 eV
Cl1Fe1O3Sr2_ICSD_93508	0.82 eV	0.68 eV
Cl1Fe1O4W1_ICSD_80798	2.13 eV	2.22 eV

Supplementary Table 735. Five-fold cross validated predictions for the band gap energy (129/277).

system	calculated	predicted
Cl1Fe2H3O12Te4_ICSD_50706	1.90 eV	2.13 eV
Cl1Fe2O4P1_ICSD_171	3.27 eV	2.32 eV
Cl1Fe3O8Pb4_ICSD_15521	0.69 eV	0.75 eV
Cl1Fe3O8Pb4_ICSD_9205	0.69 eV	0.71 eV
Cl1Ga1Te1_ICSD_15582	2.16 eV	1.64 eV
Cl1Gd1H2O2_ICSD_260831	4.40 eV	4.17 eV
Cl1Gd1Mo1O4_ICSD_420055	2.69 eV	2.73 eV
Cl1Gd1O1_ICSD_59232	4.74 eV	4.28 eV
Cl1Gd1O3Se1_ICSD_418535	4.33 eV	4.09 eV
Cl1Gd1O4S1_ICSD_410323	4.39 eV	4.22 eV
Cl1Gd1O4W1_ICSD_35292	3.26 eV	2.81 eV
Cl1Gd1O5Te2_ICSD_88674	0.33 eV	1.61 eV
Cl1Ge1H3_ICSD_62109	4.69 eV	3.43 eV
Cl1H12N7P2_ICSD_405490	4.65 eV	4.15 eV
Cl1H13O6_ICSD_2235	5.23 eV	5.28 eV
Cl1H1Hg1O4_ICSD_29038	2.05 eV	2.26 eV
Cl1H1Hg1O4_ICSD_59874	2.37 eV	2.29 eV
Cl1H1O1Pb1_ICSD_28035	3.39 eV	3.12 eV
Cl1H1O1Pb1_ICSD_74291	2.93 eV	2.88 eV
Cl1H1O1Pb1_ICSD_76932	2.78 eV	3.03 eV
Cl1H1O1Sr1_ICSD_407719	4.97 eV	4.71 eV
Cl1H1O3Pb3_ICSD_94064	2.16 eV	2.55 eV
Cl1H1Sr1_ICSD_37200	4.01 eV	3.83 eV
Cl1H1_ICSD_28844	2.83 eV	2.37 eV
Cl1H2Hg1N1_ICSD_92480	2.38 eV	2.27 eV
Cl1H2Ho1O2_ICSD_260834	4.67 eV	4.66 eV
Cl1H2Li1O1_ICSD_281198	5.37 eV	4.77 eV
Cl1H2Lu1O2_ICSD_260841	4.88 eV	4.47 eV
Cl1H2Na1O5_ICSD_173	5.21 eV	4.91 eV
Cl1H2Nd1O2_ICSD_20047	3.88 eV	3.56 eV
Cl1H2O2Tb1_ICSD_260832	3.96 eV	3.86 eV
Cl1H2O6Rb1Se2Zn1_ICSD_409915	4.39 eV	3.92 eV
Cl1H3Li4O3_ICSD_74929	4.58 eV	4.70 eV
Cl1H3O5_ICSD_23866	5.30 eV	4.66 eV
Cl1H4N1O1_ICSD_14204	5.32 eV	3.82 eV
Cl1H4N1O1_ICSD_85479	2.23 eV	2.88 eV
Cl1H4N1O2_ICSD_59921	2.94 eV	4.44 eV
Cl1H4N1O4_ICSD_6241	5.27 eV	4.94 eV
Cl1H4Na1O2_ICSD_2313	4.95 eV	4.49 eV
Cl1H4Na1O6_ICSD_425697	5.18 eV	4.71 eV
Cl1H5N2O4_ICSD_9894	5.39 eV	4.55 eV
Cl1H5O2_ICSD_15353	5.20 eV	4.95 eV
Cl1H6Li1O7_ICSD_32534	5.29 eV	4.83 eV
Cl1H6Na1O5_ICSD_1954	3.02 eV	4.30 eV
Cl1Hf1N1_ICSD_261539	2.21 eV	1.79 eV
Cl1Hg2O12Rb3S3_ICSD_436	2.37 eV	2.92 eV
Cl1Hg2O12S3Tl3_ICSD_435	2.74 eV	2.55 eV
Cl1Hg2P3_ICSD_74771	1.12 eV	1.28 eV
Cl1Hg3I1S2_ICSD_250174	1.38 eV	1.46 eV
Cl1Hg3O4P1_ICSD_411756	2.37 eV	1.88 eV

Supplementary Table 736. Five-fold cross validated predictions for the band gap energy (130/277).

system	calculated	predicted
Cl1Ho1Mo1O4_ICSD_420056	2.63 eV	2.58 eV
Cl1Ho1O3Se1_ICSD_418538	4.37 eV	4.39 eV
Cl1Ho1O3Te1_ICSD_95844	3.86 eV	3.22 eV
Cl1I1Sn1_ICSD_23262	2.51 eV	1.92 eV
Cl1I1_ICSD_23886	1.49 eV	1.20 eV
Cl1I1_ICSD_26032	1.06 eV	1.39 eV
Cl1In1K6Te4_ICSD_79543	1.98 eV	1.73 eV
Cl1In1O1_ICSD_24058	2.56 eV	2.38 eV
Cl1In1O3Te1_ICSD_279576	3.15 eV	3.06 eV
Cl1In1S4Sb2_ICSD_159468	1.58 eV	1.60 eV
Cl1In1Te1_ICSD_21031	1.72 eV	1.55 eV
Cl1In1_ICSD_2429	2.16 eV	2.03 eV
Cl1In1_ICSD_2432	1.27 eV	1.50 eV
Cl1In1_ICSD_425449	1.32 eV	1.40 eV
Cl1In5S5_ICSD_414221	1.25 eV	1.18 eV
Cl1In5Se5_ICSD_414220	0.92 eV	0.90 eV
Cl1K1Mo1O5P1_ICSD_50988	1.35 eV	1.77 eV
Cl1K1O2_ICSD_59935	2.32 eV	3.24 eV
Cl1K1O3_ICSD_16690	5.51 eV	4.72 eV
Cl1K1O3_ICSD_26685	5.49 eV	4.65 eV
Cl1K1O3_ICSD_9483	5.17 eV	4.77 eV
Cl1K1O4_ICSD_33562	5.14 eV	4.62 eV
Cl1K1O4_ICSD_413444	5.53 eV	5.04 eV
Cl1K1_ICSD_240512	5.04 eV	4.83 eV
Cl1K1_ICSD_290514	4.89 eV	4.49 eV
Cl1K2Na1O6S2_ICSD_24677	5.07 eV	4.88 eV
Cl1K3O1S3W1_ICSD_10318	2.58 eV	2.23 eV
Cl1La1Nb2O6_ICSD_35428	2.96 eV	2.76 eV
Cl1La1O1_ICSD_24611	4.47 eV	4.45 eV
Cl1La1O4S1_ICSD_408944	1.56 eV	3.74 eV
Cl1La1Se1_ICSD_188001	1.29 eV	1.33 eV
Cl1La1Te1_ICSD_426279	0.89 eV	1.08 eV
Cl1La3O8Si2_ICSD_65023	5.14 eV	3.99 eV
Cl1La3S8Si2_ICSD_412221	2.84 eV	2.75 eV
Cl1Li1O2_ICSD_59936	2.63 eV	4.02 eV
Cl1Li1O4_ICSD_165579	5.75 eV	4.87 eV
Cl1Li1_ICSD_52235	6.27 eV	4.99 eV
Cl1Li4N1_ICSD_84649	1.77 eV	2.20 eV
Cl1Li6O5P1_ICSD_421479	5.24 eV	4.99 eV
Cl1Lu1O3Se1_ICSD_241239	4.60 eV	4.24 eV
Cl1Mg2O4P1_ICSD_2844	5.04 eV	4.95 eV
Cl1Mn1O3_ICSD_416749	2.06 eV	2.20 eV
Cl1Mn1S2Sb1_ICSD_151925	1.49 eV	1.44 eV
Cl1Mn2O4P1_ICSD_2813	3.14 eV	2.53 eV
Cl1Mo1O4Tb1_ICSD_420059	2.66 eV	2.69 eV
Cl1Mo1O4Tm1_ICSD_420060	0.34 eV	1.92 eV
Cl1Mo1O4Y1_ICSD_249647	2.68 eV	2.27 eV
Cl1Mo1O5P1Rb1_ICSD_50989	1.33 eV	1.77 eV
Cl1N1O1_ICSD_411511	1.93 eV	2.62 eV
Cl1N1O4S1_ICSD_27059	1.32 eV	1.97 eV

Supplementary Table 737. Five-fold cross validated predictions for the band gap energy (131/277).

system	calculated	predicted
Cl1N1O6_ICSD_25817	1.38 eV	2.05 eV
Cl1N1Sr2_ICSD_410769	1.93 eV	1.94 eV
Cl1N1Ti1_ICSD_27396	1.22 eV	1.55 eV
Cl1N1Zn2_ICSD_425734	2.33 eV	2.27 eV
Cl1N1Zr1_ICSD_25506	1.35 eV	1.53 eV
Cl1N1Zr1_ICSD_93740	1.72 eV	1.48 eV
Cl1N3O2S3_ICSD_36378	2.32 eV	2.10 eV
Cl1N3_ICSD_424502	2.27 eV	2.35 eV
Cl1N5S4_ICSD_14085	1.98 eV	2.30 eV
Cl1N5S5_ICSD_62528	1.50 eV	1.96 eV
Cl1Na1O3_ICSD_26684	5.66 eV	4.83 eV
Cl1Na1O4_ICSD_172567	5.25 eV	4.93 eV
Cl1Na1O4_ICSD_33567	4.49 eV	5.05 eV
Cl1Na1O4_ICSD_51730	5.43 eV	4.92 eV
Cl1Na1_ICSD_100633	5.04 eV	4.73 eV
Cl1Na1_ICSD_43434	4.03 eV	4.01 eV
Cl1Na3O1_ICSD_67319	2.10 eV	2.25 eV
Cl1Nb1O8Pb2Se2_ICSD_189153	2.64 eV	2.45 eV
Cl1Nd1O5Te2_ICSD_88673	0.28 eV	1.15 eV
Cl1Nd1Te1_ICSD_426777	0.41 eV	0.97 eV
Cl1Nd3O8Si2_ICSD_92490	3.83 eV	3.39 eV
Cl1O12P3Pb5_ICSD_24238	3.38 eV	3.36 eV
Cl1O12P3Sr5_ICSD_2089	5.08 eV	4.85 eV
Cl1O12Sr5V3_ICSD_171381	3.93 eV	2.75 eV
Cl1O1Ti1_ICSD_39314	2.20 eV	2.07 eV
Cl1O1V1_ICSD_27011	1.17 eV	1.36 eV
Cl1O1Y1_ICSD_31667	5.10 eV	4.54 eV
Cl1O1Y1_ICSD_60585	4.29 eV	4.13 eV
Cl1O1Yb1_ICSD_6077	0.13 eV	1.10 eV
Cl1O2Pb1Sb1_ICSD_86229	1.92 eV	2.35 eV
Cl1O2Rb1_ICSD_162802	2.38 eV	3.11 eV
Cl1O2_ICSD_67665	0.96 eV	1.97 eV
Cl1O3Pb1V1_ICSD_420548	2.31 eV	2.60 eV
Cl1O3Rb1_ICSD_10283	5.25 eV	4.54 eV
Cl1O3Sb1Te1_ICSD_86095	3.14 eV	2.84 eV
Cl1O3Se1Tb1_ICSD_418536	4.38 eV	4.26 eV
Cl1O3Sr1V1_ICSD_50787	2.13 eV	2.39 eV
Cl1O3Tl1_ICSD_76434	4.33 eV	3.10 eV
Cl1O4P1Sn2_ICSD_16519	2.58 eV	2.85 eV
Cl1O4Pb1Re1_ICSD_171147	3.44 eV	2.19 eV
Cl1O4Rb1S1Zn1_ICSD_386	4.95 eV	4.10 eV
Cl1O4Rb1_ICSD_33565	5.22 eV	4.91 eV
Cl1O4Rb1_ICSD_51728	5.45 eV	5.11 eV
Cl1O4S1Tl1Zn1_ICSD_387	4.58 eV	3.61 eV
Cl1O4Sb3_ICSD_410039	2.68 eV	2.74 eV
Cl1O4Tl1_ICSD_33564	4.57 eV	2.95 eV
Cl1O9Re1S2_ICSD_419178	2.43 eV	2.25 eV
Cl1Rb1_ICSD_18016	4.83 eV	4.84 eV
Cl1Rb1_ICSD_61521	4.77 eV	4.87 eV
Cl1Rh1Te1_ICSD_56853	1.27 eV	0.95 eV

Supplementary Table 738. Five-fold cross validated predictions for the band gap energy (132/277).

system	calculated	predicted
Cl1Tl1_ICSD_109143	2.58 eV	2.09 eV
Cl1Tl1_ICSD_29107	2.17 eV	2.11 eV
Cl1Tl1_ICSD_61518	2.52 eV	2.13 eV
Cl2Co1H12O6_ICSD_34429	4.00 eV	3.55 eV
Cl2Co1H4O2_ICSD_34651	3.38 eV	3.17 eV
Cl2Co1O8_ICSD_33288	3.79 eV	3.23 eV
Cl2Co1_ICSD_15939	3.35 eV	2.77 eV
Cl2Co2O3Te1_ICSD_415798	2.94 eV	2.49 eV
Cl2Co5O12Se4_ICSD_416965	3.25 eV	3.03 eV
Cl2Cr1H12I1N4_ICSD_48186	2.48 eV	2.40 eV
Cl2Cr1O2_ICSD_416750	2.40 eV	2.17 eV
Cl2Cr1_ICSD_27490	2.09 eV	2.06 eV
Cl2Cr3F6K2_ICSD_60897	2.24 eV	2.46 eV
Cl2Cs1Cu2I1_ICSD_60960	2.48 eV	2.08 eV
Cl2Cs1I1_ICSD_14260	2.20 eV	2.23 eV
Cl2Cs1K1O8_ICSD_22504	5.50 eV	5.01 eV
Cl2Cs1Li1_ICSD_35397	5.04 eV	4.97 eV
Cl2Cs1Li1_ICSD_423634	5.08 eV	5.12 eV
Cl2Cu1H4O2_ICSD_15087	1.58 eV	1.59 eV
Cl2Cu1H4O4Pb2_ICSD_81589	1.56 eV	1.55 eV
Cl2Cu1H6N2_ICSD_180189	1.18 eV	2.26 eV
Cl2Cu1K2O4S1_ICSD_22364	1.60 eV	1.86 eV
Cl2Cu1O6Se2Sr2_ICSD_174067	1.67 eV	1.82 eV
Cl2Cu1_ICSD_66645	0.82 eV	1.25 eV
Cl2Cu2O1_ICSD_96610	0.63 eV	0.96 eV
Cl2Cu2O5Te2_ICSD_89978	1.27 eV	1.02 eV
Cl2Cu2O6Pb1Se2_ICSD_189154	0.89 eV	1.18 eV
Cl2Cu2O6Pb1Te2_ICSD_189155	1.07 eV	1.25 eV
Cl2Cu2O6Se2Sr1_ICSD_174068	1.39 eV	1.46 eV
Cl2Cu2O6Sr1Te2_ICSD_416653	1.25 eV	1.36 eV
Cl2Cu3H6Mg1O6_ICSD_240663	1.55 eV	1.64 eV
Cl2Cu3H6O6Zn1_ICSD_425834	1.19 eV	1.53 eV
Cl2Cu3O6Se2_ICSD_240496	0.60 eV	0.84 eV
Cl2Cu3O6Se2_ICSD_92165	0.79 eV	0.85 eV
Cl2Cu5O8Se2_ICSD_8163	0.88 eV	0.81 eV
Cl2F12Pb7_ICSD_10402	4.21 eV	3.89 eV
Cl2F1Tb1_ICSD_418279	4.86 eV	4.52 eV
Cl2F2I2O2_ICSD_411237	2.07 eV	2.18 eV
Cl2F2N3O2P1S2_ICSD_2352	4.79 eV	4.06 eV
Cl2F6I1Sb1_ICSD_15526	1.88 eV	1.86 eV
Cl2Fe1H4O2_ICSD_15597	3.19 eV	2.83 eV
Cl2Fe1H8O4_ICSD_9488	3.64 eV	2.56 eV
Cl2Fe1_ICSD_64831	2.82 eV	2.66 eV
Cl2Fe5O18Te6_ICSD_245637	1.15 eV	2.13 eV
Cl2Ga6Li8O24Si6_ICSD_87987	4.48 eV	4.29 eV
Cl2Ga6Na8O24Si6_ICSD_417705	4.16 eV	4.20 eV
Cl2H12Mg1O6_ICSD_34694	4.82 eV	5.19 eV
Cl2H12N4O8Zn1_ICSD_74790	4.45 eV	3.58 eV
Cl2H12Ni1O6_ICSD_22284	3.61 eV	3.15 eV
Cl2H12O6Sr1_ICSD_48110	5.02 eV	4.65 eV

Supplementary Table 739. Five-fold cross validated predictions for the band gap energy (133/277).

system	calculated	predicted
Cl2H12Sr7_ICSD_418948	3.86 eV	3.40 eV
Cl2H1K3O6Pt1S2_ICSD_16439	3.28 eV	2.69 eV
Cl2H1N1O4S2_ICSD_165233	4.05 eV	3.08 eV
Cl2H2Mg1O1_ICSD_172993	5.35 eV	4.82 eV
Cl2H2Mo1O3_ICSD_25031	2.41 eV	2.17 eV
Cl2H2O1Sr1_ICSD_60883	5.04 eV	5.03 eV
Cl2H4Mg1O10_ICSD_261132	5.09 eV	4.73 eV
Cl2H4Mg1O2_ICSD_172992	5.34 eV	4.68 eV
Cl2H4Mn1O2_ICSD_15596	3.94 eV	3.38 eV
Cl2H4O6Zn1_ICSD_8231	2.52 eV	3.37 eV
Cl2H6N2_ICSD_23145	0.09 eV	3.88 eV
Cl2H6N2_ICSD_240903	5.46 eV	2.27 eV
Cl2H8Mg1O12_ICSD_261131	5.32 eV	5.16 eV
Cl2H8Mg1O12_ICSD_262271	5.24 eV	5.32 eV
Cl2H8Mn1N4_ICSD_25784	3.84 eV	3.06 eV
Cl2H8N2O2Pt1_ICSD_35759	1.97 eV	2.52 eV
Cl2H8N2O2Pt1_ICSD_35760	2.64 eV	2.41 eV
Cl2H8N4Zn1_ICSD_15875	4.88 eV	3.74 eV
Cl2Hg1O2Pb2_ICSD_74973	2.26 eV	2.28 eV
Cl2Hg1_ICSD_23277	2.86 eV	2.53 eV
Cl2Hg2_ICSD_31173	2.58 eV	2.21 eV
Cl2Hg3O2_ICSD_28399	0.27 eV	1.40 eV
Cl2Hg3S2_ICSD_27399	1.92 eV	1.69 eV
Cl2Hg3Se2_ICSD_27400	1.78 eV	1.57 eV
Cl2Hg3Te2_ICSD_27401	1.88 eV	1.54 eV
Cl2Hg4O2_ICSD_16662	1.87 eV	1.49 eV
Cl2I1N3S4_ICSD_412782	1.62 eV	1.82 eV
Cl2I2Ta1_ICSD_69688	1.41 eV	1.30 eV
Cl2K5Na1O12S4_ICSD_24676	5.24 eV	4.88 eV
Cl2Li5N1_ICSD_84763	1.95 eV	2.51 eV
Cl2Mg1O8_ICSD_261619	5.25 eV	5.10 eV
Cl2Mg1_ICSD_17063	5.51 eV	4.56 eV
Cl2Mg1_ICSD_51244	4.26 eV	4.97 eV
Cl2Mg1_ICSD_51245	4.95 eV	5.35 eV
Cl2Mg1_ICSD_51246	3.46 eV	4.87 eV
Cl2Mg1_ICSD_51247	5.14 eV	4.45 eV
Cl2Mg1_ICSD_56147	5.56 eV	4.83 eV
Cl2Mn1_ICSD_33752	3.88 eV	3.07 eV
Cl2N2O4Pd1Rb2_ICSD_93914	2.13 eV	2.18 eV
Cl2N2S2Te1_ICSD_170631	2.01 eV	1.80 eV
Cl2N2S3_ICSD_23974	1.70 eV	2.20 eV
Cl2N3S2V1_ICSD_30742	0.97 eV	1.41 eV
Cl2N4O12S10_ICSD_72781	1.33 eV	2.50 eV
Cl2N4S4_ICSD_26373	2.56 eV	2.25 eV
Cl2Nb1S2_ICSD_10484	1.66 eV	1.46 eV
Cl2Nb1Se2_ICSD_10483	1.07 eV	1.22 eV
Cl2Nd2O7Ta2_ICSD_108807	3.25 eV	3.17 eV
Cl2Ni1O8_ICSD_33289	3.41 eV	3.25 eV
Cl2Ni1_ICSD_14208	2.83 eV	2.50 eV
Cl2Ni2O8Sr1Te3_ICSD_418683	3.05 eV	2.48 eV

Supplementary Table 740. Five-fold cross validated predictions for the band gap energy (134/277).

system	calculated	predicted
Cl2Ni7O18Te6_ICSD_240326	2.72 eV	2.34 eV
Cl2O1Pd2_ICSD_61333	1.47 eV	1.65 eV
Cl2O1S1_ICSD_62971	3.60 eV	2.82 eV
Cl2O1V1_ICSD_24380	0.82 eV	1.81 eV
Cl2O1_ICSD_407768	1.06 eV	1.66 eV
Cl2O2Pb3_ICSD_23521	2.46 eV	2.46 eV
Cl2O2U1_ICSD_36056	2.64 eV	2.59 eV
Cl2O4Pb1_ICSD_68484	1.27 eV	2.85 eV
Cl2O4Sr1_ICSD_171021	2.52 eV	3.79 eV
Cl2O5Sb4_ICSD_2233	2.65 eV	2.69 eV
Cl2O6Pb1_ICSD_40286	4.34 eV	3.43 eV
Cl2O6Pb2Pd1Se2_ICSD_423573	2.02 eV	2.15 eV
Cl2O6Pb2Pd1Te2_ICSD_423574	1.94 eV	1.97 eV
Cl2O6Pb3Te2_ICSD_281171	2.56 eV	2.58 eV
Cl2O6Sr1_ICSD_61157	5.22 eV	4.59 eV
Cl2O6_ICSD_62063	1.19 eV	1.84 eV
Cl2O7Pr2Ta2_ICSD_108806	3.19 eV	3.05 eV
Cl2O7_ICSD_63680	3.68 eV	2.65 eV
Cl2Pb1_ICSD_43344	3.81 eV	3.29 eV
Cl2Pb1_ICSD_81978	3.17 eV	3.04 eV
Cl2Pd1Se6_ICSD_405207	1.34 eV	1.14 eV
Cl2Pd1_ICSD_404624	2.04 eV	1.78 eV
Cl2Pd1_ICSD_421219	1.62 eV	1.57 eV
Cl2Pd1_ICSD_421220	1.57 eV	1.60 eV
Cl2Pd1_ICSD_421221	1.49 eV	1.71 eV
Cl2Pt1_ICSD_28527	2.40 eV	1.67 eV
Cl2Re6S8_ICSD_67658	1.20 eV	1.21 eV
Cl2Re6Se8_ICSD_35623	1.24 eV	1.03 eV
Cl2Ru1Te8_ICSD_422366	0.20 eV	0.78 eV
Cl2S1_ICSD_38351	2.10 eV	2.35 eV
Cl2Se2_ICSD_37018	2.15 eV	2.05 eV
Cl2Si1_ICSD_85526	2.01 eV	3.88 eV
Cl2Sn1_ICSD_15452	1.92 eV	1.28 eV
Cl2Sn1_ICSD_81977	0.38 eV	1.80 eV
Cl2Sr1_ICSD_18011	5.22 eV	4.32 eV
Cl2Ti1_ICSD_23177	2.66 eV	2.60 eV
Cl2Ti1_ICSD_38219	2.65 eV	2.42 eV
Cl2Tm1_ICSD_447	2.63 eV	3.42 eV
Cl2V1_ICSD_15901	2.80 eV	2.50 eV
Cl2V1_ICSD_246905	2.80 eV	2.44 eV
Cl2Yb1_ICSD_6054	3.40 eV	3.15 eV
Cl2Zn1_ICSD_1692	4.60 eV	4.07 eV
Cl2Zn1_ICSD_26152	4.31 eV	3.75 eV
Cl2Zn1_ICSD_26154	4.63 eV	3.88 eV
Cl2Zr1_ICSD_20144	0.84 eV	1.42 eV
Cl2_ICSD_201696	2.39 eV	2.29 eV
Cl2_ICSD_22406	2.48 eV	2.95 eV
Cl3Co1Cs1_ICSD_27511	3.34 eV	2.89 eV
Cl3Co1Rb1_ICSD_24305	2.50 eV	2.78 eV
Cl3Co1Tl1_ICSD_155189	2.45 eV	2.52 eV

Supplementary Table 741. Five-fold cross validated predictions for the band gap energy (135/277).

system	calculated	predicted
Cl3Co1Tl1_ICSD_155191	2.49 eV	2.51 eV
Cl3Cr1Cs1_ICSD_41802	1.86 eV	2.16 eV
Cl3Cr1Rb1_ICSD_2225	1.86 eV	2.01 eV
Cl3Cr1Rb1_ICSD_56837	2.10 eV	2.04 eV
Cl3Cr1Rb1_ICSD_8184	2.06 eV	2.02 eV
Cl3Cr1_ICSD_22080	2.41 eV	2.20 eV
Cl3Cr1_ICSD_22081	2.45 eV	2.22 eV
Cl3Cr1_ICSD_33578	2.45 eV	2.17 eV
Cl3Cs1Cu1_ICSD_26666	1.24 eV	1.44 eV
Cl3Cs1Cu2_ICSD_14201	2.31 eV	1.91 eV
Cl3Cs1Fe1_ICSD_300249	3.02 eV	2.83 eV
Cl3Cs1Ge1_ICSD_23121	2.20 eV	2.81 eV
Cl3Cs1Ge1_ICSD_75215	1.98 eV	2.76 eV
Cl3Cs1Li2_ICSD_245974	5.28 eV	5.03 eV
Cl3Cs1Li2_ICSD_423635	5.25 eV	5.03 eV
Cl3Cs1Mg1_ICSD_54167	5.12 eV	4.86 eV
Cl3Cs1Mn1_ICSD_2555	3.64 eV	3.44 eV
Cl3Cs1Ni1_ICSD_60262	2.87 eV	2.80 eV
Cl3Cs1Pb1_ICSD_109294	2.18 eV	2.23 eV
Cl3Cs1Pb1_ICSD_201250	2.19 eV	2.08 eV
Cl3Cs1Sn1_ICSD_14199	2.89 eV	3.35 eV
Cl3Cs1Sn1_ICSD_28082	0.99 eV	1.92 eV
Cl3Cs1V1_ICSD_201832	2.36 eV	2.04 eV
Cl3Cs1Yb1_ICSD_31461	3.07 eV	3.23 eV
Cl3Cu1K1_ICSD_15590	1.13 eV	1.53 eV
Cl3Cu1K2_ICSD_150292	2.69 eV	2.61 eV
Cl3Cu1Rb1_ICSD_15523	1.06 eV	1.06 eV
Cl3Cu1Rb1_ICSD_40944	1.06 eV	1.08 eV
Cl3Cu1Rb1_ICSD_56838	1.06 eV	1.07 eV
Cl3Cu1Rb2_ICSD_150294	2.62 eV	2.34 eV
Cl3Dy1_ICSD_40064	3.83 eV	3.28 eV
Cl3Er1H2O13_ICSD_410764	5.28 eV	4.38 eV
Cl3F2Rb2Sb1_ICSD_200497	3.06 eV	3.43 eV
Cl3F2Sb1_ICSD_200039	1.76 eV	2.01 eV
Cl3F2Sb1_ICSD_380014	1.80 eV	2.05 eV
Cl3F3N3P3_ICSD_16098	5.02 eV	4.57 eV
Cl3Fe1K1_ICSD_10134	3.55 eV	2.99 eV
Cl3Fe1Mn7O10_ICSD_69062	0.95 eV	1.51 eV
Cl3Fe1_ICSD_27500	1.99 eV	2.30 eV
Cl3Fe1_ICSD_39765	1.97 eV	2.17 eV
Cl3Ga1_ICSD_30336	4.15 eV	3.73 eV
Cl3Ga1_ICSD_413455	4.14 eV	4.09 eV
Cl3Gd1_ICSD_22270	3.94 eV	4.10 eV
Cl3Gd2N1_ICSD_47224	3.25 eV	3.17 eV
Cl3Gd2_ICSD_9580	0.22 eV	2.01 eV
Cl3Ge1Rb1_ICSD_12149	3.77 eV	2.99 eV
Cl3H12N5O1Os1_ICSD_39306	2.75 eV	2.70 eV
Cl3H12O6V1_ICSD_418528	1.94 eV	2.60 eV
Cl3H1La3O6Ta1_ICSD_62189	1.02 eV	2.91 eV
Cl3H4K1Mn1O2_ICSD_14170	3.98 eV	2.91 eV

Supplementary Table 742. Five-fold cross validated predictions for the band gap energy (136/277).

system	calculated	predicted
Cl3H6La1O9_ICSD_29439	2.58 eV	3.20 eV
Cl3Hg1Na1_ICSD_38302	2.53 eV	2.53 eV
Cl3I1N1V1_ICSD_10470	1.37 eV	1.67 eV
Cl3Ir1Te6_ICSD_423672	1.24 eV	1.01 eV
Cl3Ir1_ICSD_23171	2.46 eV	1.79 eV
Cl3K1Mn1_ICSD_32710	2.98 eV	3.36 eV
Cl3K2N3O5Os1_ICSD_39424	2.46 eV	2.46 eV
Cl3K2N3O5Ru1_ICSD_97229	2.20 eV	2.61 eV
Cl3K2N3O6Pt1_ICSD_20709	2.66 eV	2.83 eV
Cl3La1_ICSD_31574	4.47 eV	4.20 eV
Cl3La2N1_ICSD_165789	3.24 eV	2.99 eV
Cl3La3O6U1_ICSD_73201	1.95 eV	2.48 eV
Cl3La3O6W1_ICSD_35595	3.17 eV	3.21 eV
Cl3La3O7Si2_ICSD_82386	4.88 eV	4.44 eV
Cl3La4N1S3_ICSD_74902	3.00 eV	2.25 eV
Cl3Mg1Rb1_ICSD_15279	4.59 eV	4.66 eV
Cl3Mg1Rb1_ICSD_4036	4.75 eV	4.89 eV
Cl3Mn1Na1_ICSD_2552	4.08 eV	3.52 eV
Cl3Mn1Rb1_ICSD_36125	3.21 eV	3.05 eV
Cl3Mn1Tl1_ICSD_23167	2.61 eV	2.77 eV
Cl3Mn1Tl1_ICSD_32712	2.90 eV	2.82 eV
Cl3Mo1N1_ICSD_15117	1.87 eV	1.67 eV
Cl3Mo1O1_ICSD_4411	1.98 eV	1.89 eV
Cl3Mo1S2_ICSD_28062	1.54 eV	1.40 eV
Cl3Mo1_ICSD_26109	0.74 eV	0.87 eV
Cl3Mo1_ICSD_83878	0.71 eV	1.05 eV
Cl3N1Nd2_ICSD_79944	2.77 eV	2.91 eV
Cl3N1Nd4S3_ICSD_92443	2.60 eV	2.54 eV
Cl3N1Pr2_ICSD_79943	3.10 eV	3.10 eV
Cl3N1Se2_ICSD_84030	1.41 eV	1.86 eV
Cl3N1W1_ICSD_165376	1.97 eV	1.55 eV
Cl3N1Y2_ICSD_65829	2.06 eV	2.54 eV
Cl3N1_ICSD_4034	2.18 eV	2.51 eV
Cl3N3S3_ICSD_30558	2.67 eV	2.19 eV
Cl3N3Ti1_ICSD_15996	2.68 eV	2.56 eV
Cl3Nb1O1_ICSD_26471	2.65 eV	2.75 eV
Cl3Nb1O1_ICSD_412071	2.95 eV	2.69 eV
Cl3Nd1_ICSD_31577	2.92 eV	3.02 eV
Cl3Nd3O6U1_ICSD_73203	1.86 eV	2.23 eV
Cl3Ni1Rb1_ICSD_15010	2.77 eV	2.52 eV
Cl3O10Se2Tb5_ICSD_154235	3.73 eV	3.91 eV
Cl3O1P1_ICSD_9128	4.56 eV	3.64 eV
Cl3O1V1_ICSD_250365	2.87 eV	2.20 eV
Cl3O2Re1_ICSD_416056	1.95 eV	1.84 eV
Cl3O6Pr3U1_ICSD_73202	1.90 eV	2.02 eV
Cl3O6Pr3W1_ICSD_20626	3.12 eV	2.46 eV
Cl3P1_ICSD_32027	4.04 eV	3.09 eV
Cl3Pr1_ICSD_202925	3.02 eV	3.29 eV
Cl3Pr1_ICSD_65079	3.02 eV	3.29 eV
Cl3Rb1Ti1_ICSD_49747	1.50 eV	2.36 eV

Supplementary Table 743. Five-fold cross validated predictions for the band gap energy (137/277).

system	calculated	predicted
Cl3Rb1V1_ICSD_201830	2.32 eV	1.89 eV
Cl3Rh1_ICSD_25764	2.20 eV	1.84 eV
Cl3Ru1_ICSD_20717	1.09 eV	0.99 eV
Cl3Ru1_ICSD_414041	0.54 eV	1.12 eV
Cl3S8Sb1_ICSD_35741	2.83 eV	2.01 eV
Cl3Sb1_ICSD_8258	3.76 eV	2.72 eV
Cl3Sc1_ICSD_74517	4.26 eV	3.99 eV
Cl3Tb1_ICSD_23938	3.80 eV	2.98 eV
Cl3Tb1_ICSD_63541	4.11 eV	4.06 eV
Cl3Tb1_ICSD_63543	4.51 eV	3.77 eV
Cl3Tc1_ICSD_261105	0.11 eV	0.95 eV
Cl3Ti1_ICSD_26070	2.54 eV	2.71 eV
Cl3Ti1_ICSD_29035	2.48 eV	2.57 eV
Cl3Ti1_ICSD_39426	2.51 eV	2.42 eV
Cl3Ti1_ICSD_39427	2.46 eV	2.31 eV
Cl3Ti1_ICSD_39429	2.51 eV	2.30 eV
Cl3Ti1_ICSD_39430	2.53 eV	2.61 eV
Cl3U1_ICSD_2353	2.71 eV	2.70 eV
Cl3V1_ICSD_38237	1.28 eV	2.05 eV
Cl3Y1_ICSD_15684	4.86 eV	4.28 eV
Cl3Y2_ICSD_23337	0.76 eV	2.10 eV
Cl4Co1K2_ICSD_661	3.74 eV	3.03 eV
Cl4Co1Li2_ICSD_73227	2.82 eV	2.68 eV
Cl4Co1Rb2_ICSD_87515	3.62 eV	3.05 eV
Cl4Co1Rb2_ICSD_87517	3.76 eV	2.91 eV
Cl4Cr1Cs2_ICSD_41571	0.35 eV	1.76 eV
Cl4Cr1Li2_ICSD_202627	2.08 eV	2.25 eV
Cl4Cr1Li2_ICSD_403035	1.96 eV	2.12 eV
Cl4Cr1Na2_ICSD_66201	2.19 eV	2.31 eV
Cl4Cr1Rb2_ICSD_41570	0.24 eV	1.25 eV
Cl4Cr1Rb2_ICSD_9858	1.07 eV	1.11 eV
Cl4Cs1Ga1_ICSD_201670	4.55 eV	4.41 eV
Cl4Cs2Hg1_ICSD_39568	3.03 eV	2.95 eV
Cl4Cs2I2Pd1_ICSD_240484	0.98 eV	1.23 eV
Cl4Cs2Mg1_ICSD_9005	5.02 eV	4.96 eV
Cl4Cs2O2U1_ICSD_56859	3.23 eV	3.04 eV
Cl4Cs2O2U1_ICSD_66542	3.21 eV	3.16 eV
Cl4Cs2Pd1_ICSD_95812	2.39 eV	2.15 eV
Cl4Cs2Yb1_ICSD_49623	2.84 eV	3.02 eV
Cl4Cs3Li1_ICSD_245969	4.74 eV	4.60 eV
Cl4Cs6O1_ICSD_411634	2.00 eV	1.81 eV
Cl4Cu1Ga1_ICSD_300103	2.49 eV	2.36 eV
Cl4Cu1H4K2O2_ICSD_16052	1.64 eV	1.67 eV
Cl4Cu1H4O2Rb2_ICSD_71867	1.65 eV	1.96 eV
Cl4Cu1Rb2_ICSD_15145	1.12 eV	1.53 eV
Cl4Er1Na1_ICSD_82364	4.98 eV	4.49 eV
Cl4F1Sb1_ICSD_74783	1.43 eV	1.96 eV
Cl4F1Ta1_ICSD_27413	3.24 eV	2.15 eV
Cl4F5N1Te1W1_ICSD_37149	2.13 eV	2.46 eV
Cl4F6P1Sb1_ICSD_71876	4.50 eV	3.05 eV

Supplementary Table 744. Five-fold cross validated predictions for the band gap energy (138/277).

system	calculated	predicted
Cl4Fe1Na1_ICSD_16994	2.43 eV	2.73 eV
Cl4Fe1Rb1_ICSD_63470	2.52 eV	2.67 eV
Cl4Ga1K1_ICSD_281297	4.60 eV	4.54 eV
Cl4Ga1Li1_ICSD_60849	4.16 eV	4.47 eV
Cl4Ga1Rb1_ICSD_409650	4.44 eV	4.48 eV
Cl4Ga1Tl1_ICSD_419825	4.15 eV	3.63 eV
Cl4Ga2H2_ICSD_165558	4.82 eV	3.74 eV
Cl4Ga2_ICSD_14218	3.81 eV	3.90 eV
Cl4Gd1Li1_ICSD_38326	4.32 eV	3.87 eV
Cl4Gd1Na1_ICSD_33784	4.38 eV	4.41 eV
Cl4Gd2S1_ICSD_56742	2.18 eV	3.04 eV
Cl4Ge1_ICSD_280880	4.01 eV	3.46 eV
Cl4H10O5Sn1_ICSD_413631	2.85 eV	2.62 eV
Cl4H18K1N6O8Ru1_ICSD_280489	4.40 eV	2.74 eV
Cl4H26O14Sc2_ICSD_39838	4.94 eV	4.91 eV
Cl4H2Hg1K2O1_ICSD_30211	2.70 eV	3.18 eV
Cl4H4N1Tl1_ICSD_14020	2.42 eV	2.32 eV
Cl4H8Si5_ICSD_262931	3.76 eV	4.18 eV
Cl4Hf1_ICSD_402054	4.17 eV	3.66 eV
Cl4Hg2K2O3S1_ICSD_419256	2.22 eV	2.38 eV
Cl4Hg3O1_ICSD_35649	1.48 eV	1.89 eV
Cl4Hg3S2Zn1_ICSD_420783	1.31 eV	1.66 eV
Cl4Hg4I2S1_ICSD_422546	0.55 eV	1.74 eV
Cl4I1K1_ICSD_30203	2.30 eV	2.24 eV
Cl4I2O6Pb3_ICSD_247144	3.33 eV	2.68 eV
Cl4K1Tl1_ICSD_14105	2.30 eV	2.47 eV
Cl4K2Mg1_ICSD_4035	4.76 eV	4.88 eV
Cl4K2O2Os1_ICSD_36231	2.31 eV	1.92 eV
Cl4K2Pd1_ICSD_2723	2.18 eV	1.94 eV
Cl4K2Pt1_ICSD_68768	2.48 eV	2.10 eV
Cl4K2Zn1_ICSD_68824	4.72 eV	4.72 eV
Cl4K2Zn1_ICSD_80861	4.69 eV	4.70 eV
Cl4Li2Zn1_ICSD_202743	4.55 eV	4.59 eV
Cl4Li2Zn1_ICSD_73222	5.09 eV	4.63 eV
Cl4Lu1Na1_ICSD_78994	5.19 eV	4.02 eV
Cl4Mg1Na2_ICSD_69343	4.93 eV	4.62 eV
Cl4Mn1Na2_ICSD_9136	3.94 eV	3.22 eV
Cl4Mn1Rb2_ICSD_1139	3.39 eV	2.41 eV
Cl4Mo1O1_ICSD_41418	1.49 eV	1.63 eV
Cl4N1Re1_ICSD_419181	1.28 eV	1.59 eV
Cl4N1V1_ICSD_28128	1.58 eV	1.56 eV
Cl4N4S4Te1_ICSD_416253	1.91 eV	1.89 eV
Cl4Na1Sc1_ICSD_402273	4.11 eV	4.52 eV
Cl4Na2Pd1_ICSD_202971	2.17 eV	2.42 eV
Cl4Na2Ti1_ICSD_400264	2.57 eV	3.19 eV
Cl4Na2Zn1_ICSD_402063	4.88 eV	4.58 eV
Cl4O1Os1_ICSD_417247	1.54 eV	1.48 eV
Cl4O1Rb6_ICSD_405193	1.66 eV	1.93 eV
Cl4O1W1_ICSD_426524	2.02 eV	1.69 eV
Cl4Os1Sc4_ICSD_421532	0.81 eV	0.79 eV

Supplementary Table 745. Five-fold cross validated predictions for the band gap energy (139/277).

system	calculated	predicted
Cl4Os1_ICSD_1165	1.28 eV	1.33 eV
Cl4Pd1Tl2_ICSD_89601	1.43 eV	1.80 eV
Cl4Pt1_ICSD_22073	0.33 eV	1.56 eV
Cl4Rb2Zn1_ICSD_35657	4.71 eV	4.68 eV
Cl4Rb2Zn1_ICSD_63188	4.22 eV	4.50 eV
Cl4Rb2Zn1_ICSD_68569	4.71 eV	4.68 eV
Cl4Re6Se8Tl2_ICSD_162300	1.59 eV	1.36 eV
Cl4S1Sm2_ICSD_89534	0.96 eV	2.27 eV
Cl4S1Tl6_ICSD_35289	1.79 eV	1.67 eV
Cl4S1W1_ICSD_16125	2.15 eV	1.51 eV
Cl4Se1_ICSD_15578	3.09 eV	2.19 eV
Cl4Si1_ICSD_62279	5.55 eV	4.33 eV
Cl4Sn1_ICSD_411242	3.14 eV	2.69 eV
Cl4Sr1Zn1_ICSD_410192	4.62 eV	4.38 eV
Cl4Ta1_ICSD_402406	1.46 eV	1.71 eV
Cl4Tc1_ICSD_26055	1.81 eV	1.52 eV
Cl4Th1_ICSD_26197	3.90 eV	3.58 eV
Cl4Th1_ICSD_6055	4.02 eV	2.84 eV
Cl4Ti1_ICSD_280981	3.69 eV	2.98 eV
Cl4Tl2_ICSD_4031	2.21 eV	2.52 eV
Cl4U1_ICSD_202331	2.27 eV	2.55 eV
Cl4V1_ICSD_250364	0.99 eV	1.77 eV
Cl4Zr1_ICSD_26049	3.63 eV	2.97 eV
Cl5Co1Cs3_ICSD_14087	3.42 eV	3.05 eV
Cl5Co1Cs3_ICSD_15245	3.42 eV	3.00 eV
Cl5Co1Tl3_ICSD_10137	2.71 eV	2.58 eV
Cl5Cs1Pb2_ICSD_249888	3.83 eV	2.91 eV
Cl5Cs1Pd2_ICSD_411722	1.95 eV	1.87 eV
Cl5Cs1Sn2_ICSD_93791	3.07 eV	2.57 eV
Cl5Cs3Hg1_ICSD_630	2.94 eV	3.08 eV
Cl5Cs3Li2_ICSD_245971	4.98 eV	4.93 eV
Cl5Cs3Zn1_ICSD_240876	4.50 eV	4.37 eV
Cl5Fe1N4S4_ICSD_47103	1.53 eV	1.86 eV
Cl5Fe1Tl3_ICSD_10138	2.72 eV	2.42 eV
Cl5K1Pb2_ICSD_416431	3.63 eV	3.24 eV
Cl5K1Sn2_ICSD_152003	2.98 eV	2.55 eV
Cl5K2Mo1O1_ICSD_28215	2.16 eV	2.09 eV
Cl5K2N1O1Ru1_ICSD_20713	2.17 eV	1.79 eV
Cl5K2N1Os1_ICSD_27560	2.23 eV	2.02 eV
Cl5K2N1Os1_ICSD_43729	2.00 eV	2.18 eV
Cl5K2Sb1_ICSD_6059	3.24 eV	2.68 eV
Cl5K2U1_ICSD_41551	3.01 eV	3.26 eV
Cl5Li1Tm2_ICSD_201996	2.90 eV	3.61 eV
Cl5Li1Yb2_ICSD_77273	3.59 eV	2.86 eV
Cl5Mo1_ICSD_26500	0.76 eV	1.09 eV
Cl5Mo1_ICSD_84620	0.71 eV	1.00 eV
Cl5N3O1P2S1_ICSD_43	4.01 eV	3.44 eV
Cl5Nb1_ICSD_66537	2.48 eV	2.46 eV
Cl5Nb3O2_ICSD_91781	1.06 eV	1.42 eV
Cl5O4Re2_ICSD_12111	1.39 eV	1.53 eV

Supplementary Table 746. Five-fold cross validated predictions for the band gap energy (140/277).

system	calculated	predicted
Cl5O4Re2_ICSD_416429	1.58 eV	1.73 eV
Cl5P1_ICSD_26661	1.77 eV	2.41 eV
Cl5P4S4Ta1_ICSD_419157	2.00 eV	1.78 eV
Cl5Pa1_ICSD_15391	2.41 eV	2.94 eV
Cl5Pb1Tl3_ICSD_1262	3.27 eV	3.14 eV
Cl5Pb2Tl1_ICSD_417	3.44 eV	3.44 eV
Cl5Sb1_ICSD_250363	1.86 eV	2.16 eV
Cl6Cr1Na3_ICSD_62035	2.74 eV	3.13 eV
Cl6Cs1Sb1_ICSD_155691	1.97 eV	2.46 eV
Cl6Cs2Er1Na1_ICSD_50361	5.35 eV	4.92 eV
Cl6Cs2Ge1_ICSD_28892	2.20 eV	3.02 eV
Cl6Cs2Hg1Pd1_ICSD_36616	1.82 eV	1.87 eV
Cl6Cs2Ho1Na1_ICSD_245366	5.30 eV	5.08 eV
Cl6Cs2In1Li1_ICSD_65735	3.32 eV	3.41 eV
Cl6Cs2Li1Sc1_ICSD_65734	3.88 eV	4.17 eV
Cl6Cs2Li1Y1_ICSD_65731	4.88 eV	4.70 eV
Cl6Cs2Mo1_ICSD_409808	2.14 eV	1.90 eV
Cl6Cs2Na1Tb1_ICSD_96062	5.20 eV	4.78 eV
Cl6Cs2Pb1_ICSD_26713	1.44 eV	2.17 eV
Cl6Cs2Pb1_ICSD_29033	1.43 eV	2.01 eV
Cl6Cs2Pt1_ICSD_26709	2.22 eV	2.13 eV
Cl6Cs2Pt1_ICSD_29032	2.21 eV	1.94 eV
Cl6Cs2Re1_ICSD_64613	2.45 eV	1.83 eV
Cl6Cs2Se1_ICSD_26693	2.74 eV	2.76 eV
Cl6Cs2Sn1_ICSD_26699	2.40 eV	2.33 eV
Cl6Cs2Te1_ICSD_26704	2.79 eV	2.69 eV
Cl6Cs2Th1_ICSD_16658	4.75 eV	3.70 eV
Cl6Cs2Ti1_ICSD_26690	2.75 eV	2.67 eV
Cl6Cs2U1_ICSD_202332	3.01 eV	2.63 eV
Cl6Cs2Zr1_ICSD_26695	3.64 eV	3.39 eV
Cl6Cs4Pb1_ICSD_35703	4.26 eV	3.69 eV
Cl6Er1Na3_ICSD_65145	4.81 eV	4.49 eV
Cl6Eu4O1_ICSD_65171	2.78 eV	2.79 eV
Cl6Fe1H12O6Pt1_ICSD_163595	1.18 eV	2.16 eV
Cl6Fe1Hf1_ICSD_39817	3.26 eV	1.77 eV
Cl6Fe1K3Na1_ICSD_170747	3.37 eV	3.27 eV
Cl6Fe1Zr1_ICSD_39666	2.66 eV	1.82 eV
Cl6Ga1N1Se2_ICSD_71903	1.64 eV	2.26 eV
Cl6Ga1Sb1_ICSD_24786	3.90 eV	3.28 eV
Cl6Gd3N1_ICSD_63582	3.47 eV	3.54 eV
Cl6H16N5O3Rh1_ICSD_411835	2.58 eV	2.43 eV
Cl6H18In2N6_ICSD_281343	3.90 eV	3.25 eV
Cl6H22O10Sn1_ICSD_27180	2.73 eV	3.31 eV
Cl6H4N1Sb1_ICSD_412875	1.95 eV	2.20 eV
Cl6H4N1W1_ICSD_412876	0.77 eV	2.12 eV
Cl6H5O2Sb1_ICSD_280168	1.81 eV	2.36 eV
Cl6H8N2Pt1_ICSD_97927	1.91 eV	2.16 eV
Cl6H8N2Re1_ICSD_8044	2.27 eV	2.28 eV
Cl6H8N2Sn1_ICSD_605	2.03 eV	2.69 eV
Cl6Hf1Hg3Se2_ICSD_412466	1.62 eV	1.69 eV

Supplementary Table 747. Five-fold cross validated predictions for the band gap energy (141/277).

system	calculated	predicted
Cl6Hf1Te4_ICSD_401589	1.27 eV	1.56 eV
Cl6Hf1Te8_ICSD_410306	0.88 eV	1.07 eV
Cl6Hg3S2Zr1_ICSD_412469	1.79 eV	1.62 eV
Cl6Hg3Se2Zr1_ICSD_412468	1.44 eV	1.63 eV
Cl6Hg3Te2U1_ICSD_419437	2.80 eV	2.05 eV
Cl6Hg6P4Sn1_ICSD_411863	2.11 eV	1.57 eV
Cl6I1P1_ICSD_26594	1.76 eV	1.72 eV
Cl6I1Se6W1_ICSD_78789	0.65 eV	0.90 eV
Cl6I2_ICSD_24714	1.73 eV	1.65 eV
Cl6In1Na3_ICSD_154092	3.88 eV	3.87 eV
Cl6K2Mn1_ICSD_9679	0.50 eV	2.53 eV
Cl6K2Pd1_ICSD_65037	1.12 eV	1.48 eV
Cl6K2Pt1_ICSD_52033	1.96 eV	1.96 eV
Cl6K2Pu1_ICSD_202526	0.27 eV	2.37 eV
Cl6K2Re1_ICSD_23769	2.30 eV	2.37 eV
Cl6K2Sn1_ICSD_1669	2.36 eV	2.45 eV
Cl6K2Sn1_ICSD_1670	2.60 eV	3.22 eV
Cl6K2Sn1_ICSD_6058	2.18 eV	2.26 eV
Cl6K2Tc1_ICSD_22096	2.41 eV	2.10 eV
Cl6K2Tc2_ICSD_63512	2.26 eV	1.66 eV
Cl6K2Ti1_ICSD_26934	2.62 eV	2.62 eV
Cl6K3Mn1Na1_ICSD_290109	4.14 eV	3.84 eV
Cl6K4Mn1_ICSD_24475	3.72 eV	3.28 eV
Cl6Mn1Rb2_ICSD_9347	0.61 eV	2.34 eV
Cl6Mo1Tl2_ICSD_408777	1.91 eV	2.03 eV
Cl6N1S2Sb1_ICSD_2441	1.71 eV	1.75 eV
Cl6N2O2V1_ICSD_413012	1.31 eV	1.88 eV
Cl6N3P3_ICSD_16136	4.12 eV	3.88 eV
Cl6N6S4W2_ICSD_405886	0.81 eV	1.63 eV
Cl6Na1Nb1_ICSD_36518	2.48 eV	2.49 eV
Cl6Na1Sb1_ICSD_36517	2.04 eV	2.61 eV
Cl6Na3Y1_ICSD_59886	4.93 eV	4.33 eV
Cl6Nb1Rb2_ICSD_245748	0.62 eV	1.97 eV
Cl6O1S8Ti4_ICSD_64624	2.09 eV	1.81 eV
Cl6O1Sr4_ICSD_402497	4.57 eV	4.52 eV
Cl6O1Yb4_ICSD_202169	2.81 eV	2.42 eV
Cl6O3Si3_ICSD_71444	5.57 eV	4.56 eV
Cl6Pb1Rb2_ICSD_26712	1.32 eV	1.96 eV
Cl6Pd1Rb2_ICSD_33710	1.22 eV	1.42 eV
Cl6Pd1S2_ICSD_39434	2.04 eV	1.92 eV
Cl6Pt1Rb2_ICSD_26708	2.09 eV	1.93 eV
Cl6Pt1S2_ICSD_66013	2.16 eV	1.88 eV
Cl6Pt1Tl2_ICSD_26710	1.92 eV	2.10 eV
Cl6Rb2Se1_ICSD_26692	2.61 eV	2.77 eV
Cl6Rb2Sn1_ICSD_9022	2.25 eV	2.19 eV
Cl6Rb2Te1_ICSD_26703	2.68 eV	2.59 eV
Cl6Rb2Ti1_ICSD_26689	2.67 eV	2.51 eV
Cl6Rb2Zr1_ICSD_26694	3.57 eV	3.16 eV
Cl6Re6Te16_ICSD_405813	0.89 eV	0.94 eV
Cl6S8W1_ICSD_65685	0.70 eV	1.17 eV

Supplementary Table 748. Five-fold cross validated predictions for the band gap energy (142/277).

system	calculated	predicted
Cl6Se4Zr1_ICSD_412285	1.32 eV	1.95 eV
Cl6Sn1Tl2_ICSD_26700	2.14 eV	2.41 eV
Cl6Te1Tl2_ICSD_26705	2.55 eV	2.32 eV
Cl6W1_ICSD_425147	1.91 eV	1.29 eV
Cl6W1_ICSD_425148	1.86 eV	1.58 eV
Cl7Fe1Se1_ICSD_39529	2.38 eV	2.11 eV
Cl7Fe1Te1_ICSD_39530	2.37 eV	2.14 eV
Cl7Ga2In1_ICSD_418803	3.79 eV	3.76 eV
Cl7Ga3_ICSD_67279	3.96 eV	3.96 eV
Cl7Hg6P4Ti1_ICSD_411415	1.80 eV	1.63 eV
Cl7I1S1_ICSD_200374	1.67 eV	1.79 eV
Cl7In1Y2_ICSD_201429	3.99 eV	3.39 eV
Cl7K1Mo2O2_ICSD_410954	2.07 eV	1.99 eV
Cl7Mo1O1Se1_ICSD_410101	1.84 eV	1.54 eV
Cl7Mo2O2Tl1_ICSD_408775	1.84 eV	2.13 eV
Cl7N2Sb1Se3_ICSD_406352	1.68 eV	1.59 eV
Cl7Nb3Se5_ICSD_10066	1.27 eV	1.33 eV
Cl7Nb3Te1_ICSD_79213	0.90 eV	0.81 eV
Cl8Co1Li6_ICSD_202401	3.71 eV	2.94 eV
Cl8Cr1Li5_ICSD_67743	2.62 eV	2.67 eV
Cl8Fe1Li6_ICSD_73217	2.03 eV	2.99 eV
Cl8Fe1P1_ICSD_22232	2.43 eV	2.06 eV
Cl8Ga2Ni1_ICSD_417870	2.92 eV	2.73 eV
Cl8Hg2Pd1Rb2_ICSD_203187	2.42 eV	1.88 eV
Cl8Hg2Pd1Tl2_ICSD_203186	2.17 eV	1.84 eV
Cl8I1P1_ICSD_60420	1.68 eV	1.84 eV
Cl8I1Sb1_ICSD_26509	1.60 eV	1.61 eV
Cl8I3Sb1_ICSD_26402	1.18 eV	1.26 eV
Cl8Ir1K3Sn1_ICSD_69143	2.01 eV	1.95 eV
Cl8Li6V1_ICSD_100834	3.06 eV	3.03 eV
Cl8Mn1Na6_ICSD_1845	4.23 eV	3.63 eV
Cl8Mn3Na2_ICSD_1846	3.96 eV	3.54 eV
Cl8N4P4_ICSD_23897	2.16 eV	3.24 eV
Cl8N4P4_ICSD_33711	3.85 eV	3.32 eV
Cl8N6Sb2_ICSD_10353	1.93 eV	2.05 eV
Cl8Na2Ti3_ICSD_401026	2.63 eV	2.52 eV
Cl8Nb3_ICSD_408645	0.43 eV	0.81 eV
Cl8O12Si8_ICSD_79706	5.67 eV	4.47 eV
Cl8O2Se2Sn1_ICSD_35477	2.63 eV	2.24 eV
Cl8O2Se4Te3W2_ICSD_380129	0.74 eV	1.40 eV
Cl8O4Si4_ICSD_71445	5.54 eV	5.61 eV
Cl8O9Re4_ICSD_10302	0.98 eV	1.71 eV
Cl8Pt1S2_ICSD_66012	1.89 eV	1.93 eV
Cl9Cr2Cs3_ICSD_94384	2.44 eV	1.61 eV
Cl9Cs1Mn4_ICSD_34750	4.01 eV	3.09 eV
Cl9Cs3Fe2_ICSD_22074	1.66 eV	2.67 eV
Cl9Cs3In2_ICSD_2491	3.54 eV	3.37 eV
Cl9Cs3Ru2_ICSD_201057	0.94 eV	1.71 eV
Cl9Cs3Sb2_ICSD_26039	2.44 eV	2.39 eV
Cl9Cs3Sc2_ICSD_24215	4.26 eV	4.27 eV

Supplementary Table 749. Five-fold cross validated predictions for the band gap energy (143/277).

system	calculated	predicted
Cl9Cs3Ti2_ICSD_402407	2.59 eV	2.48 eV
Cl9Cs3Tl2_ICSD_26657	2.20 eV	2.55 eV
Cl9Cs3V2_ICSD_61234	1.35 eV	2.06 eV
Cl9Cs3W2_ICSD_202296	1.29 eV	1.78 eV
Cl9Cs3Y2_ICSD_409540	4.88 eV	4.47 eV
Cl9In5_ICSD_37217	3.01 eV	2.80 eV
Cl9In7_ICSD_71062	2.23 eV	2.20 eV
Cl9K3Mo2_ICSD_202298	1.17 eV	1.59 eV
Cl9K3W2_ICSD_16760	1.38 eV	1.28 eV
Cl9Mo2Rb3_ICSD_202292	1.49 eV	1.35 eV
Cl9Nb1Te1_ICSD_410946	2.39 eV	2.11 eV
Cl9Nb2S4Tl5_ICSD_417942	1.70 eV	1.67 eV
Cl9P1Sn1_ICSD_60094	2.42 eV	2.18 eV
Cl9P1Te1_ICSD_21060	1.80 eV	1.86 eV
Cl9P1V1_ICSD_1047	1.48 eV	1.82 eV
Cl9Re3_ICSD_14209	0.92 eV	0.94 eV
Cl9S1Sb1_ICSD_59130	1.77 eV	1.83 eV
Cl9S1U1_ICSD_37116	2.28 eV	2.43 eV
Cl9Ta1Te1_ICSD_410947	2.78 eV	1.97 eV
Cl9Te1W1_ICSD_410945	0.47 eV	1.10 eV
Co1Cr1O4_ICSD_23492	1.90 eV	1.75 eV
Co1Cr2Se4_ICSD_601038	0.15 eV	0.21 eV
Co1Cr4H4K2O16_ICSD_154325	2.43 eV	2.55 eV
Co1Cs1F3_ICSD_15091	0.18 eV	2.83 eV
Co1Cs1K2O2_ICSD_74889	1.86 eV	1.69 eV
Co1Cs2I4_ICSD_87897	2.35 eV	2.10 eV
Co1Cs2O3_ICSD_6154	0.94 eV	1.72 eV
Co1Cs2O4Si1_ICSD_93878	3.29 eV	2.38 eV
Co1Cs2S2_ICSD_67389	2.12 eV	1.93 eV
Co1Cu1O2_ICSD_27803	1.51 eV	1.26 eV
Co1Cu1O7P2_ICSD_75379	0.93 eV	1.36 eV
Co1Cu2Ge1S4_ICSD_622541	0.70 eV	0.76 eV
Co1Cu2S4Si1_ICSD_99292	1.08 eV	1.00 eV
Co1Cu2S4Sn1_ICSD_99294	0.20 eV	0.47 eV
Co1Dy1Si1_ICSD_88272	0.11 eV	0.32 eV
Co1Dy1Sn1_ICSD_54584	0.17 eV	0.19 eV
Co1Er1Sn1_ICSD_106463	0.18 eV	0.21 eV
Co1Er7I12_ICSD_424430	0.43 eV	0.47 eV
Co1F11Na1Zr2_ICSD_81221	4.55 eV	3.52 eV
Co1F12O4S2Sb2_ICSD_411789	3.32 eV	3.00 eV
Co1F1Na1O4S1_ICSD_290052	3.67 eV	3.46 eV
Co1F2_ICSD_26604	4.21 eV	2.73 eV
Co1F3Na1_ICSD_4404	4.16 eV	3.28 eV
Co1F3_ICSD_16672	1.48 eV	2.47 eV
Co1F4K2_ICSD_33522	2.48 eV	2.77 eV
Co1F4Li1_ICSD_202919	2.48 eV	2.40 eV
Co1F4Rb2_ICSD_69683	2.49 eV	2.66 eV
Co1F6H12O6Si1_ICSD_2900	2.60 eV	2.66 eV
Co1F6H12O6Sn1_ICSD_75984	0.39 eV	2.22 eV
Co1F6H12O6Sn2_ICSD_27799	4.52 eV	3.03 eV

Supplementary Table 750. Five-fold cross validated predictions for the band gap energy (144/277).

system	calculated	predicted
Co1F6K1Rb2_ICSD_42147	2.41 eV	2.68 eV
Co1F6Li1Sr1_ICSD_41177	2.88 eV	3.25 eV
Co1F6Na1Rb2_ICSD_42148	2.52 eV	2.98 eV
Co1F6Pt1_ICSD_37447	2.57 eV	2.25 eV
Co1F6Rb2_ICSD_9701	2.27 eV	2.61 eV
Co1F6Sn1_ICSD_25014	2.70 eV	2.52 eV
Co1F6Zr1_ICSD_83724	3.54 eV	3.05 eV
Co1Fe1La3S7_ICSD_603954	0.91 eV	0.79 eV
Co1Ga2S4_ICSD_623159	2.08 eV	1.42 eV
Co1Gd1O3_ICSD_45153	0.98 eV	1.09 eV
Co1Ge1Na2O4_ICSD_23425	1.90 eV	2.01 eV
Co1Ge1O3_ICSD_26814	1.89 eV	1.78 eV
Co1Ge1Te1_ICSD_160511	0.27 eV	0.53 eV
Co1Ge2O7Sr2_ICSD_262399	2.56 eV	2.45 eV
Co1H1O2_ICSD_22285	2.70 eV	2.48 eV
Co1H1O2_ICSD_56288	2.51 eV	2.22 eV
Co1H1O5P1Zn1_ICSD_416823	3.70 eV	3.01 eV
Co1H2Mo1O7Se1_ICSD_249955	2.56 eV	2.36 eV
Co1H2O2_ICSD_88940	2.78 eV	2.49 eV
Co1H2O5S1_ICSD_71346	3.82 eV	3.58 eV
Co1H2O5Se1_ICSD_66747	2.86 eV	2.81 eV
Co1H4K2O10Se2_ICSD_98686	3.15 eV	3.15 eV
Co1H6K2O8P2_ICSD_59884	4.03 eV	3.46 eV
Co1H6N1O5P1_ICSD_280044	3.80 eV	3.58 eV
Co1H8I2O10_ICSD_408060	3.67 eV	2.94 eV
Co1H8O14P2V2_ICSD_281336	0.11 eV	1.80 eV
Co1Ho1Sn1_ICSD_108173	0.18 eV	0.19 eV
Co1I10Y6_ICSD_424841	0.17 eV	0.46 eV
Co1I12Sc7_ICSD_63303	0.25 eV	0.86 eV
Co1I12Tb7_ICSD_424442	0.25 eV	0.48 eV
Co1I2_ICSD_52368	2.04 eV	2.11 eV
Co1I4Rb2_ICSD_1029	2.32 eV	1.93 eV
Co1In2S4_ICSD_623937	1.31 eV	0.91 eV
Co1In2Se4_ICSD_658918	0.64 eV	0.66 eV
Co1K1O2_ICSD_15770	1.09 eV	0.99 eV
Co1K1O2_ICSD_4199	0.69 eV	1.26 eV
Co1K2O12P4_ICSD_2069	4.41 eV	3.65 eV
Co1K2O2_ICSD_74940	0.77 eV	1.33 eV
Co1K2S2_ICSD_623958	1.96 eV	1.65 eV
Co1K2Se2_ICSD_67390	1.76 eV	1.61 eV
Co1K3N6O12_ICSD_26746	2.67 eV	2.74 eV
Co1K3O2_ICSD_73212	1.71 eV	1.40 eV
Co1K6S4_ICSD_68603	2.01 eV	1.86 eV
Co1K6Se4_ICSD_68605	1.74 eV	1.64 eV
Co1La3O6S3W1_ICSD_380405	1.17 eV	0.81 eV
Co1La4S7_ICSD_624013	1.78 eV	1.57 eV
Co1Li1N2Sr2_ICSD_72387	0.74 eV	0.76 eV
Co1Li1O2_ICSD_161384	0.40 eV	1.77 eV
Co1Li1O2_ICSD_182442	2.74 eV	1.76 eV
Co1Li1O4P1_ICSD_247497	3.58 eV	3.53 eV

Supplementary Table 751. Five-fold cross validated predictions for the band gap energy (145/277).

system	calculated	predicted
Co1Li2O8W2_ICSD_92852	3.03 eV	2.58 eV
Co1Li6O4_ICSD_62688	3.19 eV	2.99 eV
Co1Li8O6_ICSD_21026	1.44 eV	2.56 eV
Co1Mn1O3_ICSD_31854	1.29 eV	1.34 eV
Co1Mo1O4_ICSD_23808	2.66 eV	2.10 eV
Co1Mo1O4_ICSD_281235	1.97 eV	2.13 eV
Co1Mo1O6Sr2_ICSD_153544	1.59 eV	1.52 eV
Co1Mo1O6Te1_ICSD_93795	2.89 eV	2.48 eV
Co1Mo2S4_ICSD_2018	0.36 eV	0.38 eV
Co1N2O6_ICSD_412004	2.79 eV	2.91 eV
Co1N6Na3O12_ICSD_280730	2.51 eV	2.79 eV
Co1N6Na3O12_ICSD_39573	2.48 eV	2.77 eV
Co1Na1O2_ICSD_6152	2.69 eV	2.00 eV
Co1Na1O4P1_ICSD_280175	2.71 eV	3.26 eV
Co1Na1O4P1_ICSD_82752	3.59 eV	3.38 eV
Co1Na2O14P4Zr1_ICSD_65704	3.67 eV	3.62 eV
Co1Na2S2_ICSD_67386	1.51 eV	1.55 eV
Co1Na3O2_ICSD_73211	1.30 eV	1.30 eV
Co1Na4O3_ICSD_10473	1.46 eV	1.25 eV
Co1Na4O3_ICSD_10501	1.46 eV	1.23 eV
Co1Na5O2S1_ICSD_412978	1.08 eV	1.68 eV
Co1Na6S4_ICSD_624260	2.20 eV	1.91 eV
Co1Na6Se4_ICSD_68604	1.70 eV	1.65 eV
Co1Nb1O4_ICSD_16377	1.09 eV	1.97 eV
Co1Ni1O12P4_ICSD_37136	3.96 eV	3.43 eV
Co1O10P2V2_ICSD_419532	1.32 eV	1.94 eV
Co1O1_ICSD_29082	0.87 eV	0.98 eV
Co1O1_ICSD_43458	1.01 eV	0.76 eV
Co1O2Rb3_ICSD_94437	1.49 eV	1.51 eV
Co1O2_ICSD_88722	0.97 eV	1.36 eV
Co1O2_ICSD_95440	1.32 eV	1.06 eV
Co1O3Se1_ICSD_496	3.15 eV	2.83 eV
Co1O3Si1_ICSD_17054	3.61 eV	3.16 eV
Co1O3Te1_ICSD_500	2.57 eV	2.37 eV
Co1O3Ti1_ICSD_16548	3.11 eV	2.19 eV
Co1O4P1_ICSD_246224	1.38 eV	2.88 eV
Co1O4Re1_ICSD_72872	0.71 eV	1.63 eV
Co1O4Rh2_ICSD_109301	1.93 eV	1.38 eV
Co1O4S1_ICSD_16985	3.65 eV	3.52 eV
Co1O4S1_ICSD_18176	2.70 eV	2.55 eV
Co1O4S1_ICSD_33736	3.44 eV	3.19 eV
Co1O4Sb2_ICSD_262626	2.06 eV	1.70 eV
Co1O4Se1_ICSD_109072	2.25 eV	2.49 eV
Co1O4U1_ICSD_26939	2.35 eV	1.95 eV
Co1O4W1_ICSD_15851	2.77 eV	2.17 eV
Co1O6Pb2Te1_ICSD_405782	1.61 eV	1.82 eV
Co1O6Pb2W1_ICSD_77912	1.96 eV	2.08 eV
Co1O6Pt3_ICSD_35338	0.57 eV	0.75 eV
Co1O6Sb2_ICSD_203094	0.79 eV	1.18 eV
Co1O6Sr2W1_ICSD_245470	2.26 eV	1.83 eV

Supplementary Table 752. Five-fold cross validated predictions for the band gap energy (146/277).

system	calculated	predicted
Co1O6Sr3Zn1_ICSD_189230	1.42 eV	1.54 eV
Co1O6Ta2_ICSD_166673	3.06 eV	2.49 eV
Co1O6U2_ICSD_166250	2.64 eV	2.64 eV
Co1O6U2_ICSD_28127	2.65 eV	2.62 eV
Co1O6V2_ICSD_188911	1.38 eV	2.02 eV
Co1O6V2_ICSD_263002	2.18 eV	2.00 eV
Co1O6V2_ICSD_46003	2.21 eV	2.01 eV
Co1O8Re2_ICSD_51015	3.02 eV	2.97 eV
Co1P1Se1_ICSD_624624	0.74 eV	0.79 eV
Co1P3Si3_ICSD_82221	1.17 eV	1.15 eV
Co1Rb2S2_ICSD_67388	2.07 eV	1.74 eV
Co1Rb2Se2_ICSD_67391	1.88 eV	1.68 eV
Co1Rh2S4_ICSD_624812	0.91 eV	0.86 eV
Co1S17U8_ICSD_624880	0.33 eV	0.91 eV
Co1S1Sb1_ICSD_624862	0.65 eV	0.64 eV
Co1S1Sb1_ICSD_624864	0.58 eV	0.62 eV
Co1S1_ICSD_624831	0.28 eV	0.56 eV
Co1S5U2_ICSD_624883	0.97 eV	0.72 eV
Co1Sb1Ti1_ICSD_624919	0.82 eV	0.62 eV
Co1U1_ICSD_625521	0.12 eV	0.41 eV
Co2H1O5P1_ICSD_79333	3.30 eV	3.04 eV
Co2K2Na4O5_ICSD_64678	1.45 eV	1.39 eV
Co2K2O12S3_ICSD_81082	3.81 eV	3.67 eV
Co2K4O5Rb2_ICSD_33797	1.45 eV	1.52 eV
Co2K6O7_ICSD_6153	1.47 eV	1.69 eV
Co2La2O3Se2_ICSD_261191	0.96 eV	1.21 eV
Co2La3S7_ICSD_624016	0.09 eV	0.79 eV
Co2Mo3O8_ICSD_10143	1.92 eV	1.83 eV
Co2Na3O6Sb1_ICSD_245538	2.11 eV	1.66 eV
Co2Na4O5Rb2_ICSD_64680	1.52 eV	1.43 eV
Co2Na6O6_ICSD_99580	2.00 eV	1.59 eV
Co2Na7O6Rb1_ICSD_66006	0.42 eV	1.28 eV
Co2O12P4_ICSD_300027	4.14 eV	3.89 eV
Co2O14P4Si1_ICSD_82403	4.20 eV	3.46 eV
Co2O3Rb2_ICSD_95833	1.46 eV	1.29 eV
Co2O4Rb5_ICSD_73190	1.31 eV	1.37 eV
Co2O4Si1_ICSD_260092	3.24 eV	2.71 eV
Co2O4Si1_ICSD_8132	1.99 eV	2.18 eV
Co2O4Si1_ICSD_859	1.44 eV	2.17 eV
Co2O7P2_ICSD_59291	3.76 eV	2.42 eV
Co2O7P2_ICSD_74542	0.49 eV	2.56 eV
Co2O7Sb2_ICSD_247302	0.90 eV	1.48 eV
Co2O7V2_ICSD_2357	2.57 eV	2.26 eV
Co2O8P2Sr1_ICSD_73960	3.81 eV	3.19 eV
Co2O8Te3_ICSD_50702	3.15 eV	2.58 eV
Co2P2S6_ICSD_624616	1.26 eV	1.40 eV
Co2S2Tl1_ICSD_100438	0.12 eV	0.50 eV
Co3Cs2S4_ICSD_65256	1.40 eV	1.15 eV
Co3O4_ICSD_69373	0.31 eV	1.10 eV
Co3O8P2_ICSD_38260	1.90 eV	2.68 eV

Supplementary Table 753. Five-fold cross validated predictions for the band gap energy (147/277).

system	calculated	predicted
Co3O8P2_ICSD_6208	2.98 eV	2.44 eV
Co3O8U2_ICSD_86237	2.62 eV	1.77 eV
Co3O8V2_ICSD_2645	2.49 eV	2.13 eV
Co3S4_ICSD_106489	0.42 eV	0.76 eV
Co3Sb4U3_ICSD_624924	0.34 eV	0.40 eV
Co5O15Sr6_ICSD_155311	0.37 eV	0.86 eV
Co6F18Rb6_ICSD_410388	4.09 eV	2.71 eV
Co9S8_ICSD_31753	0.70 eV	0.63 eV
Co9Se8_ICSD_624990	0.84 eV	0.58 eV
Cr1Cs1F4_ICSD_2278	2.51 eV	2.10 eV
Cr1Cs1H8N2O8_ICSD_261393	1.79 eV	2.35 eV
Cr1Cs1I3_ICSD_23382	1.34 eV	1.23 eV
Cr1Cs1Li1O4_ICSD_63417	3.16 eV	2.84 eV
Cr1Cs1P2S7_ICSD_260893	1.59 eV	1.48 eV
Cr1Cs2F6K1_ICSD_9705	3.96 eV	3.85 eV
Cr1Cu1O2_ICSD_163253	1.82 eV	1.45 eV
Cr1Cu1O2_ICSD_82065	1.64 eV	1.26 eV
Cr1Cu1O3S1Sr2_ICSD_84962	1.38 eV	1.02 eV
Cr1Cu1O4_ICSD_60825	1.08 eV	1.02 eV
Cr1Cu1S2_ICSD_42393	0.31 eV	0.75 eV
Cr1Dy1O4_ICSD_167718	0.19 eV	0.68 eV
Cr1Dy1O4_ICSD_93788	0.19 eV	0.45 eV
Cr1Dy1O6Te1_ICSD_164943	2.57 eV	2.53 eV
Cr1Er1O4_ICSD_247773	0.61 eV	0.81 eV
Cr1Er1O4_ICSD_96406	0.18 eV	0.64 eV
Cr1Er1O6Te1_ICSD_164945	2.55 eV	2.40 eV
Cr1Eu1O4_ICSD_51696	0.30 eV	0.58 eV
Cr1Eu1O6Te1_ICSD_164940	0.09 eV	0.97 eV
Cr1F2_ICSD_31827	2.56 eV	2.18 eV
Cr1F3H18O9_ICSD_2379	3.89 eV	2.79 eV
Cr1F3K1_ICSD_172844	1.78 eV	1.97 eV
Cr1F3K1_ICSD_27690	0.56 eV	1.58 eV
Cr1F3O1_ICSD_59123	2.03 eV	2.31 eV
Cr1F3_ICSD_59972	2.74 eV	1.91 eV
Cr1F4Na1_ICSD_37108	2.87 eV	2.66 eV
Cr1F4Na2_ICSD_67250	2.55 eV	2.51 eV
Cr1F4Sr1_ICSD_9929	3.51 eV	2.85 eV
Cr1F4_ICSD_78778	1.50 eV	1.68 eV
Cr1F5Mn1_ICSD_1617	2.28 eV	1.95 eV
Cr1F5Rb1_ICSD_418674	1.55 eV	1.83 eV
Cr1F5Rb2_ICSD_2343	3.32 eV	2.60 eV
Cr1F5_ICSD_419661	1.35 eV	2.12 eV
Cr1F6H18Mn1N6_ICSD_2728	0.09 eV	2.29 eV
Cr1F6H8N2Na1_ICSD_418735	3.90 eV	2.73 eV
Cr1F6Hg1_ICSD_10347	1.41 eV	1.79 eV
Cr1F6K1Rb2_ICSD_9707	4.01 eV	3.55 eV
Cr1F6K1_ICSD_418672	1.33 eV	1.66 eV
Cr1F6K2Na1_ICSD_40965	3.89 eV	3.35 eV
Cr1F6Li2_ICSD_10350	1.45 eV	2.31 eV
Cr1F6Mg1_ICSD_10344	1.46 eV	1.89 eV

Supplementary Table 754. Five-fold cross validated predictions for the band gap energy (148/277).

system	calculated	predicted
Cr1F6Mn1Na1_ICSD_938	3.29 eV	2.43 eV
Cr1F6Mo1_ICSD_50507	0.53 eV	1.67 eV
Cr1F6Na1Rb2_ICSD_9706	3.84 eV	3.49 eV
Cr1F6Na1_ICSD_418671	1.31 eV	2.03 eV
Cr1F6Na3_ICSD_27070	3.97 eV	2.83 eV
Cr1F6Nb1_ICSD_75384	1.32 eV	1.53 eV
Cr1F6Rb1_ICSD_418673	1.34 eV	1.43 eV
Cr1F6Rb2_ICSD_29006	3.64 eV	3.03 eV
Cr1F7Pb2_ICSD_174348	3.37 eV	2.77 eV
Cr1F7Rb3_ICSD_9597	0.78 eV	1.74 eV
Cr1F8H2K2_ICSD_418678	1.84 eV	2.20 eV
Cr1F8H2Na2_ICSD_418677	1.76 eV	2.10 eV
Cr1Gd1O4_ICSD_51903	0.18 eV	0.83 eV
Cr1Gd1O6Te1_ICSD_164941	2.59 eV	2.34 eV
Cr1Ge2Li1O6_ICSD_174432	2.70 eV	2.37 eV
Cr1Ge2Na1O6_ICSD_260079	2.56 eV	2.45 eV
Cr1H10N4O8S2_ICSD_279585	2.79 eV	2.62 eV
Cr1H1O2_ICSD_64754	2.90 eV	2.61 eV
Cr1H1O2_ICSD_88574	2.90 eV	2.54 eV
Cr1H8N2O4_ICSD_16047	2.88 eV	2.42 eV
Cr1Hg1O4_ICSD_2224	1.18 eV	1.38 eV
Cr1Hg1O4_ICSD_416147	1.34 eV	1.28 eV
Cr1Hg1O4_ICSD_416148	1.03 eV	1.34 eV
Cr1Hg1O6Pb2_ICSD_280778	2.07 eV	1.90 eV
Cr1Hg5O5S2_ICSD_76860	1.74 eV	1.32 eV
Cr1Hg5O6_ICSD_81605	0.85 eV	1.25 eV
Cr1Ho1O4_ICSD_169873	0.63 eV	0.50 eV
Cr1Ho1O4_ICSD_173263	0.19 eV	0.51 eV
Cr1Ho1O6Te1_ICSD_164944	2.56 eV	2.48 eV
Cr1I1K1O6_ICSD_24341	2.63 eV	2.49 eV
Cr1I2_ICSD_2596	0.69 eV	1.45 eV
Cr1I2_ICSD_4073	1.19 eV	1.67 eV
Cr1I3Rb1_ICSD_15504	1.34 eV	1.31 eV
Cr1I6Tl4_ICSD_15502	1.42 eV	1.34 eV
Cr1I6Tl4_ICSD_38143	1.15 eV	1.59 eV
Cr1K1O2_ICSD_40267	2.87 eV	2.14 eV
Cr1K1O2_ICSD_425293	2.77 eV	1.91 eV
Cr1K1O7V2_ICSD_245133	2.03 eV	2.03 eV
Cr1K1P2S7_ICSD_91754	1.58 eV	1.57 eV
Cr1K1S2_ICSD_25723	1.47 eV	1.33 eV
Cr1K2O4_ICSD_2402	3.05 eV	2.55 eV
Cr1K3Mn1O8_ICSD_26803	0.83 eV	1.67 eV
Cr1K3O4_ICSD_108934	1.17 eV	2.30 eV
Cr1K3O8_ICSD_30404	1.82 eV	2.38 eV
Cr1La1O3_ICSD_29120	1.79 eV	1.81 eV
Cr1La1O3_ICSD_91270	2.07 eV	1.73 eV
Cr1La1O3_ICSD_91271	2.08 eV	1.78 eV
Cr1La1O6Te1_ICSD_164936	2.58 eV	2.32 eV
Cr1La1S3_ICSD_51123	0.26 eV	0.45 eV
Cr1La1Se3_ICSD_626229	0.15 eV	0.48 eV

Supplementary Table 755. Five-fold cross validated predictions for the band gap energy (149/277).

system	calculated	predicted
Cr1La3O6S3W1_ICSD_380404	1.38 eV	1.08 eV
Cr1La4S7_ICSD_603686	0.23 eV	0.75 eV
Cr1Li1Mo2O8_ICSD_247056	2.55 eV	2.26 eV
Cr1Li1O2_ICSD_167479	2.95 eV	2.22 eV
Cr1Li1O4Rb1_ICSD_72548	2.98 eV	2.95 eV
Cr1Li1O4Rb1_ICSD_72552	2.95 eV	2.97 eV
Cr1Li1O6Si2_ICSD_55163	2.98 eV	2.62 eV
Cr1Li1O7P2_ICSD_240965	2.69 eV	2.51 eV
Cr1Li1O8W2_ICSD_165780	2.21 eV	2.35 eV
Cr1Li1S2_ICSD_626233	1.16 eV	0.98 eV
Cr1Li2O4_ICSD_1972	2.94 eV	2.78 eV
Cr1Mg1O4_ICSD_18117	2.41 eV	2.58 eV
Cr1Mg1O4_ICSD_18120	2.35 eV	2.52 eV
Cr1Mn2O9Pb2_ICSD_185692	1.20 eV	1.25 eV
Cr1Mo1O7V1_ICSD_408563	2.36 eV	1.98 eV
Cr1Na1O2_ICSD_24595	2.86 eV	2.27 eV
Cr1Na1O6Si2_ICSD_9670	2.83 eV	2.71 eV
Cr1Na1O8W2_ICSD_161849	1.95 eV	2.35 eV
Cr1Na1S2_ICSD_660322	1.28 eV	1.17 eV
Cr1Na1Se2_ICSD_42391	0.62 eV	0.83 eV
Cr1Na2O4_ICSD_26330	2.86 eV	2.62 eV
Cr1Na4O4_ICSD_62676	1.86 eV	1.95 eV
Cr1Nd1O6Te1_ICSD_164938	1.83 eV	2.20 eV
Cr1Ni1O4_ICSD_18116	1.80 eV	1.72 eV
Cr1O2_ICSD_186838	0.16 eV	0.65 eV
Cr1O3Y1_ICSD_28909	1.59 eV	1.20 eV
Cr1O3_ICSD_16031	0.55 eV	1.16 eV
Cr1O4P1_ICSD_62159	1.80 eV	1.75 eV
Cr1O4Pb1_ICSD_91735	1.93 eV	2.00 eV
Cr1O4Sr1_ICSD_159266	1.37 eV	2.14 eV
Cr1O4Sr1_ICSD_160793	2.79 eV	1.95 eV
Cr1O4Tb1_ICSD_169015	0.65 eV	0.77 eV
Cr1O4Tb1_ICSD_26983	0.19 eV	0.40 eV
Cr1O4Tl2_ICSD_247477	2.38 eV	2.03 eV
Cr1O4U1_ICSD_15859	2.23 eV	1.61 eV
Cr1O4V1_ICSD_36244	1.86 eV	1.84 eV
Cr1O4V1_ICSD_79865	2.13 eV	1.77 eV
Cr1O4W1_ICSD_8269	0.75 eV	1.32 eV
Cr1O4Y1_ICSD_157933	0.21 eV	1.08 eV
Cr1O4Y1_ICSD_157934	0.64 eV	0.97 eV
Cr1O4Yb1_ICSD_26987	0.51 eV	0.16 eV
Cr1O5Pb2_ICSD_34831	1.90 eV	1.97 eV
Cr1O6Sr2Ta1_ICSD_157544	2.78 eV	2.33 eV
Cr1O6Ta2_ICSD_15306	1.19 eV	1.55 eV
Cr1O6Ta2_ICSD_9390	2.32 eV	1.84 eV
Cr1O6Tb1Te1_ICSD_164942	2.58 eV	2.52 eV
Cr1O6Te1Y1_ICSD_164948	2.60 eV	2.48 eV
Cr1O6Te1Yb1_ICSD_164947	0.54 eV	0.85 eV
Cr1O7Rb1V2_ICSD_245134	2.04 eV	1.96 eV
Cr1P1S4_ICSD_937	1.00 eV	1.13 eV

Supplementary Table 756. Five-fold cross validated predictions for the band gap energy (150/277).

system	calculated	predicted
Cr1S1_ICSD_16723	0.19 eV	0.47 eV
Cr1S3Sb1_ICSD_74601	0.98 eV	0.92 eV
Cr1Sb1Se3_ICSD_84866	0.62 eV	0.70 eV
Cr1Si1Te3_ICSD_626809	0.27 eV	0.51 eV
Cr1Si2_ICSD_71502	0.93 eV	0.93 eV
Cr1Si2_ICSD_96026	0.93 eV	0.91 eV
Cr2Cs3I9_ICSD_201965	0.80 eV	1.17 eV
Cr2Cs3I9_ICSD_201966	0.89 eV	0.92 eV
Cr2Cs6O7_ICSD_174316	1.41 eV	1.68 eV
Cr2Cu1O4_ICSD_16708	0.57 eV	1.38 eV
Cr2F5_ICSD_80639	1.29 eV	2.00 eV
Cr2Fe1K1O8_ICSD_34472	1.20 eV	1.71 eV
Cr2Fe1Se4_ICSD_43042	0.16 eV	0.28 eV
Cr2Fe3O12Si3_ICSD_27375	3.01 eV	2.49 eV
Cr2Ge2Te6_ICSD_79270	0.20 eV	0.35 eV
Cr2Ge3Mn3O12_ICSD_262732	2.08 eV	1.69 eV
Cr2H4In1Na1O10_ICSD_172070	2.67 eV	2.56 eV
Cr2H4K2Mg1O10_ICSD_96781	2.84 eV	2.80 eV
Cr2H4Li2O9_ICSD_20207	2.61 eV	2.60 eV
Cr2Hg1O4_ICSD_245275	1.20 eV	1.60 eV
Cr2Hg1S4_ICSD_626162	0.43 eV	0.69 eV
Cr2K2O7_ICSD_15824	2.50 eV	2.41 eV
Cr2K2O7_ICSD_280204	2.72 eV	2.63 eV
Cr2K3Na1O8_ICSD_74557	3.04 eV	2.84 eV
Cr2K3Na1O8_ICSD_74559	3.01 eV	2.64 eV
Cr2Li14N8O1_ICSD_151435	2.40 eV	2.70 eV
Cr2Li2N8O1Sr6_ICSD_411983	1.87 eV	1.60 eV
Cr2Mg1O4_ICSD_160954	2.76 eV	2.43 eV
Cr2Mg1O4_ICSD_290589	2.77 eV	2.55 eV
Cr2Mg3O12Si3_ICSD_27371	3.40 eV	2.29 eV
Cr2Mn1O4_ICSD_167400	2.34 eV	1.56 eV
Cr2Mn1S4_ICSD_164401	0.91 eV	0.68 eV
Cr2Mn3O12Si3_ICSD_77436	2.87 eV	2.29 eV
Cr2Ni1O4_ICSD_280061	1.58 eV	1.51 eV
Cr2Ni1O4_ICSD_280062	1.89 eV	1.62 eV
Cr2O11Te4_ICSD_1	2.29 eV	2.29 eV
Cr2O3_ICSD_167280	2.37 eV	1.31 eV
Cr2O4Si1_ICSD_75639	0.40 eV	1.73 eV
Cr2O4Si1_ICSD_89073	1.90 eV	1.26 eV
Cr2O4Zn1_ICSD_290591	2.63 eV	2.43 eV
Cr2O4Zn1_ICSD_290592	2.63 eV	2.28 eV
Cr2O6Re1_ICSD_173874	0.69 eV	1.08 eV
Cr2O6Te1_ICSD_24794	1.28 eV	1.39 eV
Cr2O6U1_ICSD_274	1.17 eV	1.43 eV
Cr2O6W1_ICSD_20314	1.14 eV	1.50 eV
Cr2O7Rb2_ICSD_15034	2.77 eV	2.69 eV
Cr2O7Rb2_ICSD_409745	2.69 eV	2.59 eV
Cr2O7Tl2_ICSD_413950	2.31 eV	2.06 eV
Cr2S4Ti1_ICSD_626640	0.15 eV	0.47 eV
Cr2S4Zn1_ICSD_164168	1.17 eV	0.84 eV

Supplementary Table 757. Five-fold cross validated predictions for the band gap energy (151/277).

system	calculated	predicted
Cr2S8Tl1V3_ICSD_74296	0.63 eV	0.62 eV
Cr2Se4Zn1_ICSD_626751	0.39 eV	0.48 eV
Cr3Cu1O8_ICSD_155506	0.47 eV	1.20 eV
Cr3H6K1O14S2_ICSD_75977	2.44 eV	2.10 eV
Cr3In1O8_ICSD_155504	0.22 eV	1.34 eV
Cr3K1O8_ICSD_15921	1.76 eV	1.60 eV
Cr3Li1O8_ICSD_155851	1.79 eV	1.96 eV
Cr3Na1O8_ICSD_82620	1.75 eV	1.52 eV
Cr3O12Tl2_ICSD_201793	1.72 eV	1.81 eV
Cr3O8Rb1_ICSD_82622	1.77 eV	1.62 eV
Cr3O8Tl1_ICSD_155505	1.75 eV	1.49 eV
Cr3O8_ICSD_155847	0.59 eV	1.19 eV
Cr3Rb1S5_ICSD_73143	0.71 eV	0.73 eV
Cr3S5Tl1_ICSD_23632	0.54 eV	0.59 eV
Cr4Cs2Cu1H4O16_ICSD_156678	1.65 eV	1.75 eV
Cr4Cu1Fe1S8_ICSD_53108	0.12 eV	0.64 eV
Cr4H4K2O16Zn1_ICSD_96785	2.59 eV	2.61 eV
Cr5Cs1S8_ICSD_2566	0.71 eV	0.65 eV
Cr5In1Se8_ICSD_601719	0.17 eV	0.34 eV
Cr5K1S8_ICSD_2568	0.74 eV	0.75 eV
Cr5K1Se8_ICSD_602350	0.18 eV	0.26 eV
Cr5Na1Se8_ICSD_602328	0.15 eV	0.21 eV
Cr5Rb1S8_ICSD_2567	0.72 eV	0.64 eV
Cr5Rb1Se8_ICSD_602314	0.18 eV	0.30 eV
Cr5S8Tl1_ICSD_40821	0.70 eV	0.58 eV
Cr5Se8Tl1_ICSD_88229	0.18 eV	0.28 eV
Cr8O21_ICSD_155849	1.55 eV	1.42 eV
Cs0.33Mo1O3_ICSD_65418	0.77 eV	1.79 eV
Cs1Cu1F4_ICSD_35264	1.82 eV	1.61 eV
Cs1Cu1La2Se4_ICSD_93680	1.07 eV	1.30 eV
Cs1Cu1O1_ICSD_40161	1.42 eV	1.51 eV
Cs1Cu1O2_ICSD_15097	1.28 eV	1.28 eV
Cs1Cu1S4_ICSD_402075	1.87 eV	1.63 eV
Cs1Cu1Se4_ICSD_75195	1.17 eV	1.17 eV
Cs1Cu2I3_ICSD_150305	2.37 eV	2.11 eV
Cs1Cu3Er2Se5_ICSD_413675	1.42 eV	1.37 eV
Cs1Cu3O2_ICSD_413342	1.70 eV	1.31 eV
Cs1Cu3S2_ICSD_23326	2.09 eV	1.32 eV
Cs1Dy1S2_ICSD_73540	2.14 eV	2.16 eV
Cs1Dy1Te3Zn1_ICSD_170189	1.45 eV	1.42 eV
Cs1Er1S2_ICSD_602875	2.23 eV	2.04 eV
Cs1Er1S2_ICSD_73542	2.15 eV	1.76 eV
Cs1Er1Te3Zn1_ICSD_170192	1.47 eV	1.41 eV
Cs1Eu1F3_ICSD_49577	1.56 eV	2.88 eV
Cs1Eu2I5_ICSD_41099	3.26 eV	3.00 eV
Cs1F1O2S1_ICSD_93076	4.39 eV	4.53 eV
Cs1F1O3S1_ICSD_82520	5.16 eV	5.24 eV
Cs1F1_ICSD_44288	5.28 eV	5.42 eV
Cs1F1_ICSD_53832	5.28 eV	5.21 eV
Cs1F1_ICSD_61563	5.96 eV	5.61 eV

Supplementary Table 758. Five-fold cross validated predictions for the band gap energy (152/277).

system	calculated	predicted
Cs1F2H1O6S2_ICSD_82521	5.61 eV	4.83 eV
Cs1F2H1_ICSD_45858	6.52 eV	6.28 eV
Cs1F2Li1_ICSD_18020	6.33 eV	6.13 eV
Cs1F2Li1_ICSD_245965	6.24 eV	6.02 eV
Cs1F2N1O4S2_ICSD_81866	5.33 eV	4.97 eV
Cs1F2O2P1_ICSD_16875	5.22 eV	5.16 eV
Cs1F3Hg1_ICSD_15168	0.72 eV	1.21 eV
Cs1F3Mg1_ICSD_49584	6.70 eV	6.57 eV
Cs1F3Mn1_ICSD_31765	3.48 eV	2.59 eV
Cs1F3Mo1O2_ICSD_9710	3.33 eV	3.26 eV
Cs1F3Ni1_ICSD_15092	4.15 eV	4.24 eV
Cs1F3Ni1_ICSD_61278	4.77 eV	4.15 eV
Cs1F3O1V1_ICSD_249402	2.30 eV	2.35 eV
Cs1F3Pb1_ICSD_93438	3.31 eV	3.38 eV
Cs1F3Pb1_ICSD_93439	2.93 eV	2.75 eV
Cs1F3Sr1_ICSD_49578	6.22 eV	6.00 eV
Cs1F3Yb1_ICSD_49579	1.78 eV	1.24 eV
Cs1F4Fe1_ICSD_6043	2.52 eV	2.58 eV
Cs1F4Li3_ICSD_245968	6.66 eV	6.04 eV
Cs1F4Mn1_ICSD_72396	1.98 eV	2.06 eV
Cs1F4Ti1_ICSD_27230	2.83 eV	2.97 eV
Cs1F4V1_ICSD_167354	0.17 eV	1.93 eV
Cs1F5Pd1Rb2_ICSD_35286	2.95 eV	2.72 eV
Cs1F5Pd2_ICSD_35284	1.81 eV	1.42 eV
Cs1F5Te1_ICSD_200252	4.64 eV	4.59 eV
Cs1F6Nb1_ICSD_183851	5.68 eV	4.05 eV
Cs1F6Re1_ICSD_201511	1.89 eV	2.88 eV
Cs1F6Ru1_ICSD_28694	2.37 eV	2.17 eV
Cs1F6Sb1_ICSD_201886	4.99 eV	4.64 eV
Cs1F7Mo1_ICSD_78390	3.34 eV	3.20 eV
Cs1F7Rb2Si1_ICSD_9592	5.49 eV	5.74 eV
Cs1F7Sb2_ICSD_14119	4.21 eV	4.12 eV
Cs1F7W1_ICSD_78391	4.33 eV	4.12 eV
Cs1F7Xe1_ICSD_404986	3.25 eV	3.48 eV
Cs1F8Re1_ICSD_410829	1.84 eV	2.75 eV
Cs1Fe1I4_ICSD_66811	1.22 eV	1.63 eV
Cs1Fe1O2_ICSD_180532	1.77 eV	1.61 eV
Cs1Fe1O2_ICSD_421173	1.75 eV	1.55 eV
Cs1Fe1O8S2_ICSD_245667	2.13 eV	2.22 eV
Cs1Ga1H1O10P3_ICSD_409692	4.69 eV	4.74 eV
Cs1Ga1H4_ICSD_169711	4.97 eV	4.75 eV
Cs1Ga1H4_ICSD_169712	4.65 eV	4.82 eV
Cs1Ga1S2_ICSD_41888	3.10 eV	2.30 eV
Cs1Ga1S3_ICSD_281723	2.53 eV	2.49 eV
Cs1Gd1S2_ICSD_602861	2.08 eV	2.05 eV
Cs1Gd1S2_ICSD_73538	2.01 eV	2.09 eV
Cs1Ge1I3_ICSD_62559	0.95 eV	1.51 eV
Cs1Ge1S5Ta1_ICSD_417875	0.99 eV	1.26 eV
Cs1Ge1_ICSD_43517	1.38 eV	0.99 eV
Cs1H12Mg1O10P1_ICSD_281563	4.76 eV	4.81 eV

Supplementary Table 759. Five-fold cross validated predictions for the band gap energy (153/277).

system	calculated	predicted
Cs1H1Mn1O10P3_ICSD_51089	0.82 eV	2.56 eV
Cs1H1O3S1_ICSD_56615	5.85 eV	4.80 eV
Cs1H1O3Se1_ICSD_202718	4.10 eV	3.76 eV
Cs1H1O4S1_ICSD_91808	5.38 eV	5.23 eV
Cs1H1O4Se1_ICSD_290645	3.64 eV	3.46 eV
Cs1H1_ICSD_53235	2.59 eV	2.44 eV
Cs1H1_ICSD_53236	2.32 eV	2.59 eV
Cs1H2O4P1_ICSD_200895	5.15 eV	5.16 eV
Cs1H3Mg1_ICSD_162260	2.65 eV	2.69 eV
Cs1H3O2_ICSD_280909	4.71 eV	3.77 eV
Cs1H4Li1N2_ICSD_62654	2.05 eV	1.66 eV
Cs1H4Li1O5S1_ICSD_200780	4.50 eV	4.82 eV
Cs1H6Li2N3_ICSD_65151	0.59 eV	1.88 eV
Cs1Hg1La1Se3_ICSD_281441	1.35 eV	0.89 eV
Cs1Hg1Nd1Se3_ICSD_281444	1.40 eV	1.34 eV
Cs1Hg1Se3Y1_ICSD_281440	1.40 eV	1.43 eV
Cs1Ho1K2O8P2_ICSD_64760	4.88 eV	4.14 eV
Cs1Ho1S2_ICSD_73541	2.14 eV	2.16 eV
Cs1Ho1Te3Zn1_ICSD_170191	1.46 eV	1.45 eV
Cs1I1O3_ICSD_61009	3.16 eV	2.96 eV
Cs1I1O4_ICSD_83375	2.51 eV	2.81 eV
Cs1I1_ICSD_53835	3.68 eV	3.69 eV
Cs1I1_ICSD_61517	3.86 eV	3.59 eV
Cs1I1_ICSD_9204	3.67 eV	3.66 eV
Cs1I3Mg1_ICSD_87262	3.19 eV	3.16 eV
Cs1I3Mn1_ICSD_15950	2.55 eV	2.42 eV
Cs1I3Pb1_ICSD_161481	1.45 eV	1.37 eV
Cs1I3Sn1_ICSD_14070	2.31 eV	1.71 eV
Cs1I3Sn1_ICSD_69995	0.62 eV	0.96 eV
Cs1I3Sn1_ICSD_69997	0.46 eV	0.74 eV
Cs1I3Ti1_ICSD_154258	1.52 eV	2.00 eV
Cs1I3V1_ICSD_26454	2.23 eV	1.71 eV
Cs1I4Li3_ICSD_245989	3.71 eV	3.90 eV
Cs1I4Tl1_ICSD_59110	1.25 eV	1.74 eV
Cs1In3O5_ICSD_23630	1.60 eV	1.59 eV
Cs1K2O8P2Sc1_ICSD_61787	4.51 eV	4.09 eV
Cs1K2O8P2Yb1_ICSD_280936	0.22 eV	1.91 eV
Cs1K2Sb1_ICSD_53237	0.88 eV	0.78 eV
Cs1La1Nb2O7_ICSD_82378	1.82 eV	2.25 eV
Cs1La1Te3Zn1_ICSD_170184	1.49 eV	1.32 eV
Cs1Li1Mo1O4_ICSD_20805	4.32 eV	3.01 eV
Cs1Li1O4S1_ICSD_63181	5.50 eV	4.65 eV
Cs1Li1O4S1_ICSD_63182	5.16 eV	5.10 eV
Cs1Li1O4S1_ICSD_88900	4.90 eV	5.21 eV
Cs1Lu1S2_ICSD_73545	2.09 eV	1.98 eV
Cs1Mg1O4P1_ICSD_260423	4.41 eV	4.46 eV
Cs1Mn1O4_ICSD_16819	2.23 eV	2.17 eV
Cs1Mo3Te3_ICSD_603673	0.26 eV	0.53 eV
Cs1N1O2_ICSD_50328	2.33 eV	2.72 eV
Cs1N1O3_ICSD_16339	3.43 eV	3.06 eV

Supplementary Table 760. Five-fold cross validated predictions for the band gap energy (154/277).

system	calculated	predicted
Cs1N1O3_ICSD_35555	3.60 eV	3.14 eV
Cs1N2Nb1_ICSD_72546	1.80 eV	1.78 eV
Cs1N3O4S2_ICSD_281172	2.64 eV	3.16 eV
Cs1N3_ICSD_627047	4.18 eV	3.79 eV
Cs1Na1O3Ti1_ICSD_78752	3.73 eV	3.58 eV
Cs1Na1S1_ICSD_41323	2.45 eV	2.30 eV
Cs1Na1Se1_ICSD_41322	2.04 eV	1.89 eV
Cs1Na1Te1_ICSD_405538	1.99 eV	1.82 eV
Cs1Nb1O3_ICSD_1266	3.99 eV	2.96 eV
Cs1Nb3O10Sr2_ICSD_93675	1.75 eV	1.93 eV
Cs1Nd1O2_ICSD_27336	2.82 eV	2.55 eV
Cs1O10Sr2Ta3_ICSD_93677	2.26 eV	2.40 eV
Cs1O2Sb1_ICSD_59329	2.36 eV	2.41 eV
Cs1O2Y1_ICSD_49652	2.84 eV	3.10 eV
Cs1O3V1_ICSD_1489	3.10 eV	2.79 eV
Cs1O3_ICSD_33847	0.83 eV	1.77 eV
Cs1O4P1Zn1_ICSD_85456	2.12 eV	3.63 eV
Cs1O4P1Zn1_ICSD_85457	4.28 eV	3.28 eV
Cs1O4Re1_ICSD_72817	4.22 eV	3.61 eV
Cs1O4Re1_ICSD_74505	4.33 eV	3.60 eV
Cs1O5V2_ICSD_850	1.73 eV	2.27 eV
Cs1O8S2V1_ICSD_73736	1.72 eV	1.91 eV
Cs1O8V3_ICSD_50010	2.05 eV	2.12 eV
Cs1P1Pb1S4_ICSD_409821	2.36 eV	2.19 eV
Cs1P1Pb1Se4_ICSD_165348	1.60 eV	1.35 eV
Cs1P2S7V1_ICSD_59910	1.27 eV	1.37 eV
Cs1P3Zn4_ICSD_262031	0.38 eV	0.57 eV
Cs1Pr1S2_ICSD_73548	1.93 eV	1.81 eV
Cs1S2Sb1_ICSD_200798	0.93 eV	1.65 eV
Cs1S2Tb1_ICSD_602889	2.21 eV	2.01 eV
Cs1S2Tb1_ICSD_73539	2.13 eV	2.11 eV
Cs1S6Sb1_ICSD_67977	2.08 eV	1.73 eV
Cs1Sb1_ICSD_14031	0.55 eV	0.55 eV
Cs1Sb1_ICSD_412154	0.54 eV	0.54 eV
Cs1Sb2Se4_ICSD_61220	1.11 eV	1.01 eV
Cs1Si1_ICSD_43520	1.57 eV	1.33 eV
Cs1Si1_ICSD_627104	1.20 eV	1.27 eV
Cs1Te4_ICSD_47182	0.43 eV	0.60 eV
Cs2Cu1F6K1_ICSD_59102	1.82 eV	1.65 eV
Cs2Cu1F6_ICSD_65259	0.46 eV	1.31 eV
Cs2Cu2Sb2Se5_ICSD_88681	0.99 eV	1.08 eV
Cs2Er1F6Na1_ICSD_23143	6.85 eV	5.67 eV
Cs2F10O1Sb2_ICSD_21016	3.87 eV	4.15 eV
Cs2F1O3P1_ICSD_172345	4.41 eV	4.36 eV
Cs2F4Hg1_ICSD_72353	2.03 eV	1.95 eV
Cs2F5Mn1_ICSD_69675	1.42 eV	1.82 eV
Cs2F6Fe1K1_ICSD_42144	2.21 eV	3.02 eV
Cs2F6Fe1Na1_ICSD_65503	3.80 eV	2.87 eV
Cs2F6Fe1Tl1_ICSD_6036	2.64 eV	2.57 eV
Cs2F6Ga1Li1_ICSD_9004	6.21 eV	6.20 eV

Supplementary Table 761. Five-fold cross validated predictions for the band gap energy (155/277).

system	calculated	predicted
Cs2F6Ga1Na1_ICSD_55698	6.03 eV	5.90 eV
Cs2F6Ge1_ICSD_35547	6.01 eV	5.72 eV
Cs2F6Hf1_ICSD_25600	7.22 eV	7.12 eV
Cs2F6In1Na1_ICSD_24921	5.41 eV	4.65 eV
Cs2F6K1Mn1_ICSD_91036	1.62 eV	2.88 eV
Cs2F6K1Mn1_ICSD_91037	3.91 eV	2.65 eV
Cs2F6K1Mo1_ICSD_4053	3.28 eV	2.76 eV
Cs2F6K1Rh1_ICSD_4056	3.40 eV	3.16 eV
Cs2F6K1Ti1_ICSD_67146	1.47 eV	2.48 eV
Cs2F6K1V1_ICSD_27344	3.18 eV	2.79 eV
Cs2F6K1Y1_ICSD_25367	6.47 eV	6.06 eV
Cs2F6Mn1_ICSD_47201	3.23 eV	2.72 eV
Cs2F6Mn1_ICSD_76272	3.21 eV	2.89 eV
Cs2F6Mo1Tl1_ICSD_15772	2.97 eV	2.76 eV
Cs2F6Na1Sc1_ICSD_22116	7.06 eV	6.44 eV
Cs2F6Na1Tl1_ICSD_22118	3.62 eV	4.02 eV
Cs2F6Na1Y1_ICSD_25368	6.84 eV	6.51 eV
Cs2F6Nb1_ICSD_72832	1.18 eV	2.57 eV
Cs2F6Rb1Y1_ICSD_25366	6.19 eV	5.79 eV
Cs2F6Si1_ICSD_26871	6.89 eV	6.66 eV
Cs2F6Si1_ICSD_38548	6.90 eV	6.63 eV
Cs2F6Sn1_ICSD_281	4.82 eV	4.40 eV
Cs2F6Tl1V1_ICSD_27343	2.56 eV	2.66 eV
Cs2F6Zr1_ICSD_25598	6.71 eV	5.32 eV
Cs2Fe2K4O5_ICSD_65942	1.53 eV	1.66 eV
Cs2Ga1Li3O4_ICSD_69491	3.69 eV	3.40 eV
Cs2Ge1Li2O4_ICSD_36532	3.66 eV	3.38 eV
Cs2Ge1Te4_ICSD_78830	0.49 eV	0.72 eV
Cs2Ge2Se5_ICSD_411030	1.59 eV	1.57 eV
Cs2Ge3O9Sn1_ICSD_19031	2.39 eV	2.72 eV
Cs2Ge3O9Ti1_ICSD_19030	3.46 eV	3.15 eV
Cs2H12N6Sn1_ICSD_79987	2.37 eV	2.71 eV
Cs2H4Mg1_ICSD_162261	3.00 eV	2.97 eV
Cs2H6N2P4_ICSD_413072	1.69 eV	2.23 eV
Cs2H8O10Sb2_ICSD_422768	3.23 eV	3.34 eV
Cs2Hf1I6_ICSD_201308	2.20 eV	1.54 eV
Cs2Hg1I4_ICSD_63110	2.01 eV	1.86 eV
Cs2Hg1I4_ICSD_9361	1.43 eV	1.87 eV
Cs2Hg1O2_ICSD_25513	2.05 eV	1.74 eV
Cs2Hg3I8_ICSD_4074	1.72 eV	1.80 eV
Cs2Hg3S8Sn2_ICSD_85598	1.53 eV	1.58 eV
Cs2Hg6S7_ICSD_260060	0.61 eV	1.22 eV
Cs2I4O11_ICSD_413942	2.88 eV	2.62 eV
Cs2I4Zn1_ICSD_82932	3.35 eV	2.96 eV
Cs2I5Li3_ICSD_35655	3.84 eV	3.61 eV
Cs2I6O18Zr1_ICSD_413881	3.41 eV	3.38 eV
Cs2I6Pd1_ICSD_280189	0.71 eV	0.82 eV
Cs2I6Pd1_ICSD_280190	0.15 eV	0.51 eV
Cs2I6Pt1_ICSD_37193	0.59 eV	0.58 eV
Cs2I6Te1_ICSD_38105	1.41 eV	1.59 eV

Supplementary Table 762. Five-fold cross validated predictions for the band gap energy (156/277).

system	calculated	predicted
Cs2I8_ICSD_44621	0.15 eV	1.55 eV
Cs2K1O14Si4U2_ICSD_249936	0.18 eV	2.53 eV
Cs2Li1Mn1O4_ICSD_72361	1.84 eV	2.29 eV
Cs2Li1O4V1_ICSD_40219	3.97 eV	3.69 eV
Cs2Li1S4V1_ICSD_414186	1.81 eV	1.79 eV
Cs2Li2O4Si1_ICSD_33809	3.77 eV	3.81 eV
Cs2Li2O4Ti1_ICSD_33810	3.90 eV	3.46 eV
Cs2Mn1O4_ICSD_39503	0.60 eV	1.60 eV
Cs2Mn1S2_ICSD_65455	2.18 eV	1.81 eV
Cs2Mn1S6Te2_ICSD_165378	1.64 eV	1.71 eV
Cs2Mn1Se2_ICSD_65458	1.92 eV	1.78 eV
Cs2Mo1O2S2_ICSD_423982	2.41 eV	2.22 eV
Cs2N2Tb6Te7_ICSD_419405	0.78 eV	1.01 eV
Cs2Na1O4V1_ICSD_65963	3.92 eV	3.46 eV
Cs2Ni1O2_ICSD_423923	2.36 eV	1.98 eV
Cs2Ni1P2S6_ICSD_93485	2.10 eV	1.93 eV
Cs2O12Sr1V4_ICSD_250105	2.67 eV	2.65 eV
Cs2O12Te4_ICSD_59168	0.29 eV	1.93 eV
Cs2O1_ICSD_27919	0.64 eV	1.39 eV
Cs2O2Pb1_ICSD_2268	1.83 eV	1.68 eV
Cs2O2Zn1_ICSD_2378	1.94 eV	1.78 eV
Cs2O2_ICSD_25529	1.64 eV	1.92 eV
Cs2O3Pb1_ICSD_2908	1.42 eV	1.48 eV
Cs2O3Pb1_ICSD_62140	1.39 eV	1.42 eV
Cs2O3Pr1_ICSD_1181	1.60 eV	2.17 eV
Cs2O3Sn2_ICSD_24392	2.55 eV	2.02 eV
Cs2O3Te1_ICSD_59164	3.54 eV	3.25 eV
Cs2O3Zr1_ICSD_67345	3.85 eV	3.10 eV
Cs2O4Ru1_ICSD_33799	1.34 eV	1.51 eV
Cs2O4U1_ICSD_20581	2.64 eV	2.54 eV
Cs2O6S2_ICSD_200865	5.64 eV	4.49 eV
Cs2O7P2Sr1_ICSD_39507	4.84 eV	4.46 eV
Cs2O7U2_ICSD_72588	2.56 eV	2.62 eV
Cs2O8S2_ICSD_26726	3.50 eV	4.35 eV
Cs2O9Si3Zr1_ICSD_93133	4.79 eV	4.10 eV
Cs2P2Pt1_ICSD_658699	1.11 eV	0.97 eV
Cs2P2S6_ICSD_33277	1.93 eV	2.10 eV
Cs2P2Si1_ICSD_71224	1.27 eV	1.22 eV
Cs2Pd3S4_ICSD_26250	1.70 eV	1.40 eV
Cs2Pd3Se4_ICSD_33892	1.21 eV	1.15 eV
Cs2Pt1Te2_ICSD_627076	1.23 eV	1.25 eV
Cs2Pt1_ICSD_413241	1.99 eV	0.94 eV
Cs2S1_ICSD_183207	2.10 eV	1.95 eV
Cs2S2_ICSD_200474	1.71 eV	1.74 eV
Cs2S3Ti1_ICSD_49739	1.70 eV	1.81 eV
Cs2S3_ICSD_14094	1.36 eV	1.56 eV
Cs2S5_ICSD_201073	1.84 eV	1.71 eV
Cs2S6Sn2_ICSD_73008	1.46 eV	1.52 eV
Cs2S6_ICSD_24678	1.54 eV	1.61 eV
Cs2S7Sb4_ICSD_2193	1.59 eV	1.61 eV

Supplementary Table 763. Five-fold cross validated predictions for the band gap energy (157/277).

system	calculated	predicted
Cs2S8Sb4_ICSD_67976	1.40 eV	1.55 eV
Cs2Se1_ICSD_41687	1.81 eV	1.65 eV
Cs2Se3Zr1_ICSD_409294	1.36 eV	1.62 eV
Cs2Se3_ICSD_14095	0.73 eV	1.14 eV
Cs2Se4W1_ICSD_627102	1.67 eV	1.55 eV
Cs2Se5_ICSD_60087	1.12 eV	1.02 eV
Cs2Se6Sn2_ICSD_402842	0.98 eV	1.16 eV
Cs2Se6Te2_ICSD_84993	0.72 eV	1.03 eV
Cs2Si2Te6_ICSD_61397	1.20 eV	1.09 eV
Cs2Sn1Te4_ICSD_74826	0.60 eV	0.64 eV
Cs2Te1_ICSD_36501	1.76 eV	1.12 eV
Cs2Te2_ICSD_83351	0.98 eV	0.85 eV
Cs2Te3_ICSD_53245	0.52 eV	0.57 eV
Cs2Te5_ICSD_36017	0.32 eV	0.51 eV
Cs3Er2I9_ICSD_300232	2.86 eV	2.46 eV
Cs3F3H12N4_ICSD_421716	4.85 eV	3.93 eV
Cs3F3Mo2O6_ICSD_200691	3.94 eV	3.31 eV
Cs3F4Li1_ICSD_245962	5.63 eV	5.47 eV
Cs3F5Li2_ICSD_245964	4.62 eV	5.36 eV
Cs3F6Tl1_ICSD_19076	3.33 eV	2.99 eV
Cs3F6Y1_ICSD_19078	5.33 eV	5.72 eV
Cs3F7Ge1_ICSD_202917	4.34 eV	4.84 eV
Cs3F7Mn1_ICSD_9598	1.53 eV	1.63 eV
Cs3F7Ni1_ICSD_9600	0.74 eV	2.37 eV
Cs3F7Si1_ICSD_9588	5.63 eV	5.78 eV
Cs3F7Ti1_ICSD_9594	3.29 eV	3.49 eV
Cs3Fe1S3_ICSD_71991	1.33 eV	1.46 eV
Cs3H1O8Se2_ICSD_40439	3.72 eV	3.49 eV
Cs3I9Nb2_ICSD_421295	0.41 eV	1.17 eV
Cs3I9Sb2_ICSD_84989	1.87 eV	1.69 eV
Cs3I9Sb2_ICSD_89695	1.54 eV	1.69 eV
Cs3I9Y2_ICSD_26564	2.59 eV	2.62 eV
Cs3I9Zr2_ICSD_26565	0.40 eV	1.48 eV
Cs3N11P6_ICSD_51397	4.11 eV	3.97 eV
Cs3Ni1O2_ICSD_424578	1.31 eV	1.49 eV
Cs3O3Sb1_ICSD_279580	3.08 eV	2.60 eV
Cs3O4P1_ICSD_161678	3.56 eV	3.59 eV
Cs3O4Sb1_ICSD_411211	3.12 eV	2.87 eV
Cs3O8Ta1_ICSD_30408	3.01 eV	3.18 eV
Cs3P1Se4_ICSD_415021	1.81 eV	1.74 eV
Cs3P7_ICSD_62259	2.36 eV	1.35 eV
Cs3S4Sb1_ICSD_409619	2.13 eV	1.98 eV
Cs3Sb1Se3_ICSD_89609	2.32 eV	1.96 eV
Cs3Sb1Se4_ICSD_404083	1.44 eV	1.61 eV
Cs3Sb1_ICSD_53243	0.76 eV	0.74 eV
Cs4F10Mg3_ICSD_16084	6.27 eV	6.34 eV
Cs4Fe1O3_ICSD_423336	1.33 eV	1.16 eV
Cs4Fe1O4_ICSD_174309	1.41 eV	1.51 eV
Cs4Ge2S8_ICSD_281352	2.45 eV	2.30 eV
Cs4Ge2Se6_ICSD_415215	1.61 eV	1.59 eV

Supplementary Table 764. Five-fold cross validated predictions for the band gap energy (158/277).

system	calculated	predicted
Cs4Ge2Se8_ICSD_74832	1.62 eV	1.46 eV
Cs4Ge4S10_ICSD_409455	2.60 eV	2.42 eV
Cs4H8O4P2Se6_ICSD_260478	1.65 eV	2.38 eV
Cs4Ir1O4_ICSD_72287	1.05 eV	1.45 eV
Cs4O3Pb1_ICSD_65246	1.22 eV	1.60 eV
Cs4O4Pb1_ICSD_16363	1.53 eV	1.83 eV
Cs4O4Sn1_ICSD_65970	2.22 eV	2.23 eV
Cs4O4Zr1_ICSD_65160	2.74 eV	2.24 eV
Cs4O8P4S4_ICSD_20543	4.28 eV	3.31 eV
Cs4P2Pd1Se8_ICSD_165313	1.53 eV	1.47 eV
Cs4P2Se10_ICSD_418434	1.48 eV	1.45 eV
Cs4P2Se9_ICSD_81298	1.50 eV	1.41 eV
Cs4P6_ICSD_65185	0.84 eV	0.85 eV
Cs4S6Si2_ICSD_409176	1.76 eV	2.12 eV
Cs4Se10Si4_ICSD_409816	2.59 eV	1.88 eV
Cs4Se4Si1_ICSD_409480	2.22 eV	1.97 eV
Cs4Se6Sn2_ICSD_67251	1.58 eV	1.40 eV
Cs4Se8Si2_ICSD_409815	1.94 eV	1.60 eV
Cs5Te3_ICSD_34000	0.32 eV	1.20 eV
Cs6Fe2O5_ICSD_174313	1.46 eV	1.40 eV
Cs6Fe2O5_ICSD_73134	1.46 eV	1.50 eV
Cs6Ga1K3Sb4_ICSD_300150	0.64 eV	0.61 eV
Cs6Ga2Se6_ICSD_35293	2.11 eV	2.10 eV
Cs6Ge2Se6_ICSD_409512	1.58 eV	1.80 eV
Cs6I8Re6S8_ICSD_88610	2.42 eV	1.76 eV
Cs6Ni1O8Si2_ICSD_409506	1.79 eV	2.21 eV
Cs6O7Si2_ICSD_411665	3.18 eV	2.42 eV
Cs6P2Se14Sn2_ICSD_87578	1.57 eV	1.48 eV
Cs8Fe2O7_ICSD_174315	0.27 eV	1.24 eV
Cs8Ga4Se10_ICSD_30810	2.12 eV	1.97 eV
Cs8O19Ta6_ICSD_411856	3.48 eV	3.55 eV
Cs9F27Ni9_ICSD_410393	4.31 eV	3.56 eV
Cu11O26V6_ICSD_201626	0.95 eV	0.92 eV
Cu1Dy1O1Se1_ICSD_80359	1.46 eV	1.41 eV
Cu1Dy1O8W2_ICSD_73749	1.45 eV	1.68 eV
Cu1Dy1Pb1Se3_ICSD_152517	1.39 eV	1.39 eV
Cu1Dy1S2_ICSD_172847	1.87 eV	1.76 eV
Cu1Dy1S2_ICSD_627161	1.87 eV	1.77 eV
Cu1Dy2Ge4O12_ICSD_95168	2.03 eV	2.00 eV
Cu1Dy2Rb1Se4_ICSD_91092	1.07 eV	1.13 eV
Cu1Dy3Ge1S7_ICSD_627147	1.88 eV	1.74 eV
Cu1Dy3Ge1Se7_ICSD_280032	1.17 eV	1.25 eV
Cu1Dy3S7Si1_ICSD_156279	1.88 eV	1.86 eV
Cu1Dy3S7Sn1_ICSD_155943	1.36 eV	1.44 eV
Cu1Dy3Se7Si1_ICSD_627179	1.55 eV	1.49 eV
Cu1Dy3Se7Sn1_ICSD_152552	0.90 eV	1.06 eV
Cu1Er1O8W2_ICSD_73747	1.44 eV	1.61 eV
Cu1Er1Pb1Se3_ICSD_152519	1.34 eV	1.41 eV
Cu1Er1S2_ICSD_156291	1.92 eV	1.88 eV
Cu1Er1S2_ICSD_172849	1.91 eV	1.89 eV

Supplementary Table 765. Five-fold cross validated predictions for the band gap energy (159/277).

system	calculated	predicted
Cu1Er2Ge4O12_ICSD_89987	2.05 eV	2.08 eV
Cu1Er2K1Se4_ICSD_95029	1.08 eV	1.13 eV
Cu1Er3Ge1S7_ICSD_155080	1.80 eV	1.75 eV
Cu1Er3S7Si1_ICSD_627245	1.90 eV	1.80 eV
Cu1Er3S7Sn1_ICSD_627247	1.33 eV	1.55 eV
Cu1Eu1F1S1_ICSD_172797	1.53 eV	1.47 eV
Cu1Eu1F1Se1_ICSD_172799	1.27 eV	1.39 eV
Cu1Eu1F1Te1_ICSD_419381	1.61 eV	1.38 eV
Cu1F11Na1Zr2_ICSD_78869	2.76 eV	3.11 eV
Cu1F16Sb2Xe2_ICSD_249655	1.81 eV	1.77 eV
Cu1F1H4O5P1_ICSD_33923	1.72 eV	1.33 eV
Cu1F1Na1O4S1_ICSD_262273	1.59 eV	1.87 eV
Cu1F1Nb1O3_ICSD_200533	1.69 eV	1.78 eV
Cu1F1S1Sr1_ICSD_157433	1.83 eV	1.75 eV
Cu1F1Se1Sr1_ICSD_172798	1.51 eV	1.49 eV
Cu1F1Sr1Te1_ICSD_183708	1.80 eV	1.56 eV
Cu1F24Sb2Xe6_ICSD_416303	1.64 eV	1.84 eV
Cu1F2H4O2_ICSD_27866	2.26 eV	1.69 eV
Cu1F2O3W1_ICSD_60759	1.95 eV	1.75 eV
Cu1F2_ICSD_9790	1.85 eV	1.65 eV
Cu1F3K1_ICSD_21111	1.28 eV	1.26 eV
Cu1F3K1_ICSD_8000	1.29 eV	1.31 eV
Cu1F3K1_ICSD_9856	1.42 eV	1.33 eV
Cu1F3Na1_ICSD_69655	1.82 eV	1.70 eV
Cu1F3Rb1_ICSD_109293	0.22 eV	1.11 eV
Cu1F3Rb1_ICSD_69656	1.35 eV	0.84 eV
Cu1F4K2_ICSD_31688	0.85 eV	1.72 eV
Cu1F4Na2_ICSD_34602	2.65 eV	1.99 eV
Cu1F4Sr1_ICSD_9927	2.71 eV	2.34 eV
Cu1F6H8O4Si1_ICSD_39564	1.87 eV	1.93 eV
Cu1F6K2Na1_ICSD_22111	1.70 eV	1.68 eV
Cu1F6Pt1_ICSD_64660	1.61 eV	1.83 eV
Cu1F6Sn1_ICSD_36514	2.14 eV	1.89 eV
Cu1F6Sr2_ICSD_9926	2.83 eV	2.32 eV
Cu1F6Zr1_ICSD_30117	2.14 eV	2.68 eV
Cu1Fe1O2_ICSD_246913	0.84 eV	1.22 eV
Cu1Fe1O3S1Sr2_ICSD_84963	0.75 eV	1.03 eV
Cu1Fe2In1Se4_ICSD_160047	0.55 eV	0.50 eV
Cu1Ga1I4_ICSD_400817	2.04 eV	1.82 eV
Cu1Ga1O2_ICSD_60846	1.03 eV	1.21 eV
Cu1Ga1O2_ICSD_95664	1.04 eV	0.99 eV
Cu1Ga1O3S1Sr2_ICSD_83630	0.92 eV	1.52 eV
Cu1Ga1S2_ICSD_187021	1.14 eV	1.19 eV
Cu1Ga1Se2_ICSD_603766	0.41 eV	0.67 eV
Cu1Ga1Te2_ICSD_627551	0.43 eV	0.48 eV
Cu1Ga2O10Se3_ICSD_249926	1.43 eV	1.68 eV
Cu1Gd1O1Se1_ICSD_80358	1.22 eV	1.37 eV
Cu1Gd1S3Sr1_ICSD_250435	1.30 eV	1.33 eV
Cu1Gd2K1Mo4O16_ICSD_404016	1.40 eV	1.60 eV
Cu1Gd2K1S4_ICSD_408854	1.00 eV	1.16 eV

Supplementary Table 766. Five-fold cross validated predictions for the band gap energy (160/277).

system	calculated	predicted
Cu1Gd2Rb1Se4_ICSD_91091	0.87 eV	0.96 eV
Cu1Gd3Ge1S7_ICSD_627608	1.66 eV	1.62 eV
Cu1Gd3S7Si1_ICSD_627636	1.60 eV	1.64 eV
Cu1Gd3S7Sn1_ICSD_627638	1.35 eV	1.42 eV
Cu1Ge1Ho3S7_ICSD_159888	1.81 eV	1.78 eV
Cu1Ge1La3S7_ICSD_627711	1.82 eV	1.77 eV
Cu1Ge1La3Se7_ICSD_95026	1.29 eV	1.25 eV
Cu1Ge1Nd3S7_ICSD_627749	1.61 eV	1.63 eV
Cu1Ge1Nd3Se7_ICSD_627750	1.15 eV	1.17 eV
Cu1Ge1Pr3S7_ICSD_627770	1.69 eV	1.66 eV
Cu1Ge1Pr3Se7_ICSD_154744	1.13 eV	1.20 eV
Cu1Ge1S7Tb3_ICSD_155078	1.87 eV	1.72 eV
Cu1Ge1S7Y3_ICSD_154626	1.87 eV	1.64 eV
Cu1Ge1Se7Tb3_ICSD_154748	1.17 eV	1.14 eV
Cu1Ge1Se7Y3_ICSD_157111	1.17 eV	1.20 eV
Cu1Ge2Nd2O8_ICSD_81073	1.55 eV	1.40 eV
Cu1Ge2O7Sr2_ICSD_86843	1.45 eV	1.56 eV
Cu1Ge4Ho2O12_ICSD_89986	2.05 eV	2.03 eV
Cu1Ge4O12Tb2_ICSD_95167	2.05 eV	1.87 eV
Cu1H12N2O12_ICSD_171146	2.29 eV	1.55 eV
Cu1H1I1O4_ICSD_41804	1.46 eV	1.56 eV
Cu1H1O4P1_ICSD_260593	1.76 eV	1.52 eV
Cu1H1O5Se1Tl1_ICSD_36582	1.57 eV	1.54 eV
Cu1H1_ICSD_44859	0.78 eV	1.09 eV
Cu1H2K1O5P1_ICSD_9	1.32 eV	1.62 eV
Cu1H2O2_ICSD_68459	1.33 eV	1.29 eV
Cu1H2O4Si1_ICSD_20199	1.54 eV	1.35 eV
Cu1H2O5Se1_ICSD_39887	1.79 eV	1.48 eV
Cu1H2O6Pb1S1_ICSD_164673	1.61 eV	1.79 eV
Cu1H2O6Se2_ICSD_51676	1.60 eV	1.73 eV
Cu1H3O7V2_ICSD_281323	0.27 eV	1.12 eV
Cu1H4O4P2_ICSD_280918	2.06 eV	1.62 eV
Cu1H4O4P2_ICSD_280921	1.42 eV	1.70 eV
Cu1H5O5P1_ICSD_260208	0.12 eV	1.40 eV
Cu1H6N1O5P1_ICSD_250189	1.00 eV	1.47 eV
Cu1H6N4O4_ICSD_72461	1.05 eV	1.20 eV
Cu1H6O5Sr1_ICSD_154808	1.97 eV	1.57 eV
Cu1H6O7S1_ICSD_34679	2.05 eV	1.60 eV
Cu1Hf1K1S3_ICSD_409293	0.91 eV	1.29 eV
Cu1Hf1Na1Se3_ICSD_402578	0.53 eV	0.86 eV
Cu1Hf1S3Tl1_ICSD_82562	0.98 eV	1.23 eV
Cu1Hf1Se3Tl1_ICSD_82563	0.56 eV	0.67 eV
Cu1Hf2Na1Se5_ICSD_98476	0.56 eV	0.76 eV
Cu1Hg1I1S1_ICSD_413429	0.75 eV	1.12 eV
Cu1Ho1Pb1Se3_ICSD_154669	1.37 eV	1.34 eV
Cu1Ho1S2_ICSD_172848	1.90 eV	1.87 eV
Cu1Ho1S2_ICSD_627960	1.89 eV	1.86 eV
Cu1Ho2K1Mo4O16_ICSD_380015	1.41 eV	1.56 eV
Cu1Ho2K1S4_ICSD_97561	1.22 eV	1.32 eV
Cu1Ho2K1Se4_ICSD_95028	1.07 eV	1.14 eV

Supplementary Table 767. Five-fold cross validated predictions for the band gap energy (161/277).

system	calculated	predicted
Cu1Ho2O5Sr1_ICSD_172226	1.10 eV	1.25 eV
Cu1Ho3S7Si1_ICSD_159886	1.94 eV	1.87 eV
Cu1Ho3S7Sn1_ICSD_155944	1.34 eV	1.43 eV
Cu1Ho3Se7Si1_ICSD_159887	1.66 eV	1.49 eV
Cu1I1P4Se4_ICSD_410913	1.75 eV	1.54 eV
Cu1I1Te2_ICSD_67253	1.03 eV	0.96 eV
Cu1I1_ICSD_23990	1.65 eV	1.51 eV
Cu1I1_ICSD_246688	1.97 eV	1.76 eV
Cu1I1_ICSD_246690	1.35 eV	1.61 eV
Cu1I1_ICSD_24772	1.67 eV	1.75 eV
Cu1I1_ICSD_30363	1.99 eV	1.80 eV
Cu1I1_ICSD_84217	1.99 eV	1.80 eV
Cu1I2O6_ICSD_2232	1.65 eV	1.46 eV
Cu1I2O6_ICSD_4327	1.53 eV	1.63 eV
Cu1In1Mo2O8_ICSD_73097	0.72 eV	1.28 eV
Cu1In1O2_ICSD_91058	0.44 eV	0.96 eV
Cu1In1O2_ICSD_95670	0.45 eV	0.55 eV
Cu1In1O8W2_ICSD_74944	1.11 eV	1.48 eV
Cu1In1P2Se6_ICSD_71969	0.55 eV	0.91 eV
Cu1In1S2_ICSD_600239	0.41 eV	0.54 eV
Cu1In1Te2_ICSD_628160	0.26 eV	0.43 eV
Cu1Ir1O6Sr3_ICSD_36628	0.54 eV	0.72 eV
Cu1K1Na2O2_ICSD_47105	1.78 eV	1.52 eV
Cu1K1Nd2S4_ICSD_97558	1.13 eV	1.29 eV
Cu1K1O1_ICSD_37325	1.55 eV	1.52 eV
Cu1K1O1_ICSD_40158	1.57 eV	1.50 eV
Cu1K1O2_ICSD_203081	1.19 eV	1.17 eV
Cu1K1O4P1_ICSD_100172	0.27 eV	1.16 eV
Cu1K1Pd1Se5_ICSD_281423	1.16 eV	1.01 eV
Cu1K1S1_ICSD_49008	2.26 eV	1.82 eV
Cu1K1S3Th1_ICSD_170864	1.79 eV	1.42 eV
Cu1K1S3U1_ICSD_249701	1.58 eV	1.38 eV
Cu1K1S3Zr1_ICSD_80624	0.74 eV	1.10 eV
Cu1K1S4Tb2_ICSD_97560	1.18 eV	1.19 eV
Cu1K1S4Y2_ICSD_97557	1.18 eV	1.53 eV
Cu1K1Se1_ICSD_12157	0.46 eV	1.04 eV
Cu1K1Se3U1_ICSD_156246	1.47 eV	1.13 eV
Cu1K1Se3Zr1_ICSD_80625	0.34 eV	0.77 eV
Cu1K1Te1_ICSD_12158	0.82 eV	0.87 eV
Cu1K2N6O12Pb1_ICSD_36135	1.43 eV	1.83 eV
Cu1K2Nb1S4_ICSD_402422	2.06 eV	1.89 eV
Cu1K2Nb1Se4_ICSD_73324	1.52 eV	1.37 eV
Cu1K2O12P4_ICSD_23754	2.29 eV	2.45 eV
Cu1K2P1_ICSD_61082	1.35 eV	1.23 eV
Cu1K2S3Sb1_ICSD_415483	1.45 eV	1.66 eV
Cu1K2S4V1_ICSD_81414	1.40 eV	1.42 eV
Cu1K2Sb1_ICSD_53298	1.26 eV	1.09 eV
Cu1K2Se4Ta1_ICSD_72427	2.25 eV	1.79 eV
Cu1K2Se4V1_ICSD_84303	1.11 eV	0.97 eV
Cu1K3O2_ICSD_48180	1.80 eV	1.46 eV

Supplementary Table 768. Five-fold cross validated predictions for the band gap energy (162/277).

system	calculated	predicted
Cu1K5Sb2_ICSD_32032	0.76 eV	0.76 eV
Cu1K6O8Si2_ICSD_85404	1.52 eV	2.03 eV
Cu1La1O1S1_ICSD_201046	2.03 eV	1.76 eV
Cu1La1O1Se1_ICSD_96758	1.73 eV	1.60 eV
Cu1La1O1Te1_ICSD_154591	1.67 eV	1.50 eV
Cu1La1O2_ICSD_18102	2.81 eV	2.34 eV
Cu1La1O8W2_ICSD_68614	1.76 eV	1.88 eV
Cu1La1S1Te1_ICSD_88012	0.50 eV	0.94 eV
Cu1La1S2_ICSD_24375	0.19 eV	1.19 eV
Cu1La1S2_ICSD_415078	1.43 eV	0.90 eV
Cu1La1Se2_ICSD_99675	0.98 eV	0.92 eV
Cu1La2S4_ICSD_628243	1.70 eV	1.23 eV
Cu1La3S7Sn1_ICSD_628248	1.71 eV	1.59 eV
Cu1La3Se7Si1_ICSD_628256	1.50 eV	1.50 eV
Cu1La3Se7Sn1_ICSD_152545	1.16 eV	1.16 eV
Cu1Li1O1_ICSD_49755	1.61 eV	1.27 eV
Cu1Li1O2_ICSD_74978	0.63 eV	0.96 eV
Cu1Li1O4V1_ICSD_65677	1.33 eV	1.30 eV
Cu1Li1O9P3_ICSD_2808	2.17 eV	1.89 eV
Cu1Li2O2_ICSD_174134	0.86 eV	0.99 eV
Cu1Li2O2_ICSD_67150	0.91 eV	1.21 eV
Cu1Li2O7P2_ICSD_160321	1.84 eV	2.09 eV
Cu1Li2O8W2_ICSD_92854	1.49 eV	1.88 eV
Cu1Li2Sb1_ICSD_181259	0.50 eV	0.58 eV
Cu1Li3O3_ICSD_4201	1.31 eV	1.36 eV
Cu1Lu1S2_ICSD_628310	1.78 eV	1.62 eV
Cu1Mg1P1_ICSD_8221	0.18 eV	0.59 eV
Cu1Mn1O2_ICSD_158960	0.26 eV	0.84 eV
Cu1Mn1O4V1_ICSD_170136	1.38 eV	1.53 eV
Cu1N2O6_ICSD_28477	1.08 eV	1.31 eV
Cu1N2Ta1_ICSD_71136	0.90 eV	1.08 eV
Cu1N3_ICSD_30633	1.74 eV	2.06 eV
Cu1Na1O1_ICSD_15099	1.56 eV	1.24 eV
Cu1Na1O2_ICSD_80561	0.79 eV	0.78 eV
Cu1Na1O4P1_ICSD_35451	1.53 eV	1.30 eV
Cu1Na1S3Ti1_ICSD_73886	0.50 eV	1.11 eV
Cu1Na1S3Zr1_ICSD_73887	0.67 eV	0.92 eV
Cu1Na1Se1_ICSD_12155	0.37 eV	0.88 eV
Cu1Na1Se3Zr1_ICSD_73888	0.42 eV	0.68 eV
Cu1Na1Te1_ICSD_12156	0.84 eV	0.70 eV
Cu1Na2O2_ICSD_422751	0.68 eV	0.95 eV
Cu1Na2O7P2_ICSD_80418	1.75 eV	1.91 eV
Cu1Na2P1_ICSD_1153	0.88 eV	1.05 eV
Cu1Na5O2S1_ICSD_412977	1.82 eV	1.55 eV
Cu1Nb1O3_ICSD_201899	1.50 eV	1.29 eV
Cu1Nb1Rb2S4_ICSD_84304	2.03 eV	1.97 eV
Cu1Nb1Rb2Se4_ICSD_84305	1.73 eV	1.72 eV
Cu1Nd1O1S1_ICSD_96346	1.77 eV	1.63 eV
Cu1Nd1O8W2_ICSD_40381	1.57 eV	1.64 eV
Cu1Nd1S2_ICSD_51405	1.30 eV	1.32 eV

Supplementary Table 769. Five-fold cross validated predictions for the band gap energy (163/277).

system	calculated	predicted
Cu1Nd1Se2_ICSD_95827	0.87 eV	0.96 eV
Cu1Nd2Rb1S4_ICSD_93678	1.12 eV	1.24 eV
Cu1Nd3S7Si1_ICSD_156275	1.63 eV	1.70 eV
Cu1Nd3S7Sn1_ICSD_155938	1.55 eV	1.48 eV
Cu1Nd3Se7Si1_ICSD_628529	1.32 eV	1.37 eV
Cu1Nd3Se7Sn1_ICSD_152548	1.05 eV	1.00 eV
Cu1Ni1S3Sb1_ICSD_628553	0.88 eV	0.83 eV
Cu1O1Rb1_ICSD_15100	1.45 eV	1.47 eV
Cu1O1Rb1_ICSD_188523	1.45 eV	1.48 eV
Cu1O2Pr1_ICSD_246822	2.83 eV	2.03 eV
Cu1O2Rb1_ICSD_15096	1.27 eV	1.21 eV
Cu1O2Rh1_ICSD_29214	1.56 eV	1.48 eV
Cu1O2Sc1_ICSD_151929	2.64 eV	1.61 eV
Cu1O2Sc1_ICSD_55689	2.64 eV	2.00 eV
Cu1O2Y1_ICSD_60848	2.76 eV	2.41 eV
Cu1O2Y1_ICSD_95673	2.76 eV	2.03 eV
Cu1O3Se1_ICSD_29506	0.81 eV	1.11 eV
Cu1O3Se1_ICSD_498	1.18 eV	0.96 eV
Cu1O3Ta1_ICSD_164934	1.40 eV	1.54 eV
Cu1O3V1_ICSD_19046	0.91 eV	1.33 eV
Cu1O3V1_ICSD_9414	1.14 eV	1.04 eV
Cu1O4P1Tl1_ICSD_50457	1.18 eV	1.51 eV
Cu1O4S1_ICSD_71017	1.19 eV	1.35 eV
Cu1O4Se1_ICSD_109073	0.30 eV	0.99 eV
Cu1O4U1_ICSD_36071	0.86 eV	1.35 eV
Cu1O5Se2_ICSD_603	1.11 eV	1.34 eV
Cu1O5Te2_ICSD_2542	1.48 eV	1.36 eV
Cu1O6Pt1Sr3_ICSD_66347	1.25 eV	1.57 eV
Cu1O6Rh1Sr3_ICSD_51800	0.58 eV	1.03 eV
Cu1O6S2Tl2_ICSD_2896	0.78 eV	1.49 eV
Cu1O6Sb2_ICSD_84789	0.25 eV	0.61 eV
Cu1O6Se2Sr1_ICSD_202389	1.60 eV	1.52 eV
Cu1O6Sr2Te1_ICSD_88702	1.27 eV	1.08 eV
Cu1O6Sr2W1_ICSD_33571	1.01 eV	1.49 eV
Cu1O6Ta2_ICSD_83367	0.67 eV	1.55 eV
Cu1O6Te1Tl4_ICSD_187561	1.41 eV	1.43 eV
Cu1O6V2_ICSD_21067	1.15 eV	1.46 eV
Cu1O6V2_ICSD_28151	1.63 eV	1.32 eV
Cu1O7Rb4Si2_ICSD_85405	1.62 eV	1.92 eV
Cu1O7Si2Sr2_ICSD_86842	1.55 eV	1.55 eV
Cu1O7Sr1V2_ICSD_39620	1.69 eV	1.62 eV
Cu1O8P2Sr2_ICSD_51714	1.48 eV	1.77 eV
Cu1O8Tb1W2_ICSD_74943	1.74 eV	1.65 eV
Cu1O8W2Y1_ICSD_36622	1.45 eV	1.65 eV
Cu1P1Sr1_ICSD_53323	0.11 eV	0.36 eV
Cu1P2_ICSD_35282	0.88 eV	0.88 eV
Cu1Pb1S3Y1_ICSD_152555	1.78 eV	1.34 eV
Cu1Pb1Se3Tb1_ICSD_152516	1.42 eV	1.15 eV
Cu1Pd1Rb1Se5_ICSD_281424	1.13 eV	1.02 eV
Cu1Pr1S2_ICSD_95829	1.36 eV	1.27 eV

Supplementary Table 770. Five-fold cross validated predictions for the band gap energy (164/277).

system	calculated	predicted
Cu1Pr1Se2_ICSD_99677	0.97 eV	0.92 eV
Cu1Pr1Se3Sr1_ICSD_414181	1.06 eV	1.15 eV
Cu1Pr3S7Si1_ICSD_628722	1.63 eV	1.60 eV
Cu1Pr3S7Sn1_ICSD_155939	1.56 eV	1.52 eV
Cu1Pr3Se7Si1_ICSD_156283	1.35 eV	1.40 eV
Cu1Rb1S3U1_ICSD_249704	1.61 eV	1.50 eV
Cu1Rb1Se4_ICSD_404225	1.08 eV	1.09 eV
Cu1Rb2S4Ta1_ICSD_414271	2.75 eV	2.18 eV
Cu1Rb2S4V1_ICSD_84302	1.43 eV	1.44 eV
Cu1Rb2Se4Ta1_ICSD_414272	2.18 eV	1.88 eV
Cu1S2Sb1_ICSD_628829	1.02 eV	0.81 eV
Cu1S2Sc1_ICSD_15298	1.42 eV	1.28 eV
Cu1S2Tb1_ICSD_415490	1.43 eV	1.51 eV
Cu1S2Y1_ICSD_152553	1.85 eV	1.72 eV
Cu1S2Y1_ICSD_92458	1.85 eV	1.76 eV
Cu1S3Ta1_ICSD_187747	0.62 eV	1.09 eV
Cu1S3Tl1Zr1_ICSD_82560	0.78 eV	0.94 eV
Cu1S7Si1Tb3_ICSD_156278	1.82 eV	1.77 eV
Cu1S7Si1Y3_ICSD_152792	1.85 eV	1.81 eV
Cu1S7Sn1Tb3_ICSD_155942	1.40 eV	1.47 eV
Cu1S7Sn1Y3_ICSD_152554	1.36 eV	1.61 eV
Cu1Sb1Se2_ICSD_30358	0.73 eV	0.64 eV
Cu1Se2Tb1_ICSD_157555	1.03 eV	1.05 eV
Cu1Se3Th1Tl1_ICSD_262810	1.45 eV	0.96 eV
Cu1Se3Tl1Zr1_ICSD_82561	0.39 eV	0.65 eV
Cu1Se7Si1Tb3_ICSD_629074	1.51 eV	1.40 eV
Cu1Se7Si1Y3_ICSD_152793	1.59 eV	1.27 eV
Cu1Se7Sn1Tb3_ICSD_152551	0.93 eV	0.97 eV
Cu1Te3Ti1Tl1_ICSD_81571	0.27 eV	0.38 eV
Cu2Eu1S4Sn1_ICSD_419600	0.69 eV	1.00 eV
Cu2Fe1Ge1S4_ICSD_627304	0.87 eV	0.66 eV
Cu2Fe1Ge1Se4_ICSD_627313	0.19 eV	0.48 eV
Cu2Fe1S2_ICSD_171909	0.18 eV	0.70 eV
Cu2Fe1S4Si1_ICSD_627355	1.47 eV	1.20 eV
Cu2Fe1S4Sn1_ICSD_627358	0.24 eV	0.43 eV
Cu2Fe1Se4Si1_ICSD_627368	0.92 eV	0.54 eV
Cu2Fe4S7_ICSD_15973	0.61 eV	0.74 eV
Cu2Ge1Hg1S4_ICSD_152762	0.30 eV	0.45 eV
Cu2Ge1Hg1S4_ICSD_187020	0.25 eV	0.51 eV
Cu2Ge1Mg1S4_ICSD_425555	1.09 eV	1.23 eV
Cu2Ge1Mn1S4_ICSD_627735	0.89 eV	0.94 eV
Cu2Ge1Mn1Se4_ICSD_627741	0.20 eV	0.55 eV
Cu2Ge1S3_ICSD_85138	0.36 eV	0.76 eV
Cu2Ge1S4Sr1_ICSD_10005	1.38 eV	1.12 eV
Cu2Ge1S4Zn1_ICSD_152752	0.66 eV	0.85 eV
Cu2Ge1S4Zn1_ICSD_627793	0.78 eV	0.84 eV
Cu2Ge1Se4Sr1_ICSD_411406	0.85 eV	0.87 eV
Cu2H12N8O10_ICSD_281342	0.51 eV	1.51 eV
Cu2H1K3O14P4_ICSD_62742	1.79 eV	1.70 eV
Cu2H1O5P1_ICSD_39679	1.06 eV	1.33 eV

Supplementary Table 771. Five-fold cross validated predictions for the band gap energy (165/277).

system	calculated	predicted
Cu2H4Na2O13Si4_ICSD_414048	0.18 eV	1.35 eV
Cu2Hf1Te3_ICSD_41072	0.17 eV	0.43 eV
Cu2Hf3Se8Tl2_ICSD_81554	0.61 eV	0.62 eV
Cu2Hg1I4_ICSD_30265	0.62 eV	1.11 eV
Cu2Hg1I4_ICSD_79679	0.94 eV	1.06 eV
Cu2Hg1S4Si1_ICSD_627928	0.72 eV	0.99 eV
Cu2Hg1Se4Si1_ICSD_627935	0.20 eV	0.54 eV
Cu2I3Rb1_ICSD_65250	2.11 eV	1.92 eV
Cu2I6Tl4_ICSD_65962	1.82 eV	1.84 eV
Cu2In2S6Si1_ICSD_157375	0.83 eV	1.30 eV
Cu2K1S4V1_ICSD_402924	1.34 eV	1.16 eV
Cu2K1Se4Ta1_ICSD_73957	1.82 eV	1.50 eV
Cu2K1Se4V1_ICSD_280059	1.06 eV	0.94 eV
Cu2K2S4Th1_ICSD_170865	0.92 eV	1.34 eV
Cu2K3O4_ICSD_380487	0.18 eV	1.09 eV
Cu2La6S14Si2_ICSD_23519	1.78 eV	1.70 eV
Cu2Li2O6Te1_ICSD_189655	1.17 eV	1.30 eV
Cu2Mg1Se4Si1_ICSD_425556	1.12 eV	0.96 eV
Cu2Mn1S4Si1_ICSD_628375	1.76 eV	1.42 eV
Cu2Mn1Se4Si1_ICSD_628396	0.89 eV	0.88 eV
Cu2Mo1O7Se1_ICSD_249957	1.30 eV	1.46 eV
Cu2Na1O2_ICSD_169713	0.82 eV	0.86 eV
Cu2Na2O11Si4_ICSD_240930	1.85 eV	1.57 eV
Cu2Na2O6Te1_ICSD_170637	1.21 eV	1.11 eV
Cu2Na3O6Sb1_ICSD_153037	1.26 eV	1.26 eV
Cu2O10P2U1_ICSD_59598	1.26 eV	1.58 eV
Cu2O1_ICSD_180846	0.65 eV	0.84 eV
Cu2O2Pb1_ICSD_400657	0.82 eV	0.59 eV
Cu2O2S2Sr2Zn1_ICSD_84735	1.43 eV	1.50 eV
Cu2O2Sr1_ICSD_25002	2.01 eV	1.32 eV
Cu2O2Tl1_ICSD_36535	0.50 eV	0.69 eV
Cu2O4P1_ICSD_80181	0.09 eV	0.78 eV
Cu2O4S1_ICSD_40452	1.31 eV	1.02 eV
Cu2O4Se1_ICSD_60652	0.80 eV	0.75 eV
Cu2O4Se1_ICSD_60653	0.77 eV	0.90 eV
Cu2O4W1_ICSD_202669	1.60 eV	1.29 eV
Cu2O4W1_ICSD_62058	1.17 eV	1.45 eV
Cu2O5S1_ICSD_34649	0.77 eV	1.13 eV
Cu2O7P2_ICSD_157107	1.12 eV	1.15 eV
Cu2O7P2_ICSD_27436	1.08 eV	1.17 eV
Cu2O7V2_ICSD_164189	1.26 eV	0.96 eV
Cu2O7V2_ICSD_171028	1.32 eV	1.03 eV
Cu2O7V2_ICSD_1831	0.15 eV	0.29 eV
Cu2O7V2_ICSD_34756	0.20 eV	0.26 eV
Cu2O8Te3_ICSD_50704	1.13 eV	1.20 eV
Cu2P7_ICSD_35281	0.80 eV	0.98 eV
Cu2Pb1S4Si1_ICSD_152763	1.54 eV	1.37 eV
Cu2Rb1S4V1_ICSD_280516	1.37 eV	1.06 eV
Cu2Rb2S4Sn1_ICSD_74020	1.00 eV	1.33 eV
Cu2Rb2S4Ti1_ICSD_280644	1.89 eV	1.48 eV

Supplementary Table 772. Five-fold cross validated predictions for the band gap energy (166/277).

system	calculated	predicted
Cu2S13U6_ICSD_61417	1.11 eV	0.98 eV
Cu2S1_ICSD_16550	0.37 eV	0.58 eV
Cu2S2Yb1_ICSD_628970	1.11 eV	1.21 eV
Cu2S3Si1_ICSD_70057	1.60 eV	1.43 eV
Cu2S3Si1_ICSD_88235	1.52 eV	1.32 eV
Cu2S4Si1Zn1_ICSD_628869	1.70 eV	1.48 eV
Cu2S4Sn1Sr1_ICSD_356	0.51 eV	1.06 eV
Cu2S4Sn1Sr1_ICSD_43131	0.53 eV	1.08 eV
Cu2S4Sn1Zn1_ICSD_628895	0.32 eV	0.42 eV
Cu2S4W1_ICSD_72529	1.57 eV	1.75 eV
Cu2S4W1_ICSD_98909	1.74 eV	1.42 eV
Cu2S7U3_ICSD_82155	0.88 eV	0.92 eV
Cu2Se3Si1_ICSD_88236	0.55 eV	0.59 eV
Cu2Se4Si1Zn1_ICSD_629079	0.75 eV	0.73 eV
Cu2Se7U3_ICSD_82156	0.50 eV	0.71 eV
Cu2Si1Te4Zn1_ICSD_656150	0.36 eV	0.57 eV
Cu2Te3Ti1_ICSD_402631	0.18 eV	0.35 eV
Cu2Te3Zr1_ICSD_56463	0.09 eV	0.33 eV
Cu3Dy3Sb4_ICSD_658643	0.26 eV	0.26 eV
Cu3Er1Te3_ICSD_154741	0.51 eV	0.71 eV
Cu3Er2K1Se5_ICSD_174372	1.31 eV	1.23 eV
Cu3Er2Rb1S5_ICSD_93682	1.71 eV	1.60 eV
Cu3Er2Rb1Se5_ICSD_96686	1.36 eV	1.34 eV
Cu3Er3Sb4_ICSD_658645	0.30 eV	0.28 eV
Cu3F4H2O2_ICSD_98699	1.50 eV	1.45 eV
Cu3F7Na1_ICSD_202587	1.79 eV	1.62 eV
Cu3Gd1Mn4O12_ICSD_153870	0.08 eV	0.69 eV
Cu3Gd3Sb4_ICSD_658641	0.11 eV	0.21 eV
Cu3Ge5K2O14_ICSD_410828	1.44 eV	1.44 eV
Cu3Ho2K1Se5_ICSD_174371	1.27 eV	1.22 eV
Cu3Ho2Rb1S5_ICSD_59858	1.69 eV	1.59 eV
Cu3Ho3Sb4_ICSD_658644	0.29 eV	0.27 eV
Cu3K1S2_ICSD_100001	0.70 eV	0.70 eV
Cu3K1Te2_ICSD_100830	0.68 eV	0.52 eV
Cu3K2O12Se4_ICSD_50541	1.27 eV	1.29 eV
Cu3K3P2_ICSD_12163	1.50 eV	1.29 eV
Cu3N1_ICSD_167838	0.52 eV	0.64 eV
Cu3N5Sr6_ICSD_83962	0.62 eV	0.63 eV
Cu3Nb1S4_ICSD_183994	1.89 eV	1.54 eV
Cu3Nb1Se4_ICSD_628485	1.56 eV	1.30 eV
Cu3Nb1Te4_ICSD_628495	1.07 eV	0.89 eV
Cu3O12Pb1Sn4_ICSD_162102	0.96 eV	0.91 eV
Cu3O12Sn4Sr1_ICSD_162101	0.66 eV	0.80 eV
Cu3O4V1_ICSD_418372	0.72 eV	1.02 eV
Cu3O6Te1_ICSD_189131	0.61 eV	0.71 eV
Cu3O6W1_ICSD_15001	0.94 eV	1.34 eV
Cu3O8V2_ICSD_27184	0.95 eV	1.06 eV
Cu3O8V2_ICSD_27310	0.92 eV	0.88 eV
Cu3P1S4_ICSD_412240	1.38 eV	1.32 eV
Cu3P1Se4_ICSD_95412	0.30 eV	0.62 eV

Supplementary Table 773. Five-fold cross validated predictions for the band gap energy (167/277).

system	calculated	predicted
Cu3Rb1S2_ICSD_409646	0.72 eV	0.76 eV
Cu3S2Tl1_ICSD_23290	0.63 eV	0.51 eV
Cu3S3Sb1_ICSD_403113	1.15 eV	0.97 eV
Cu3S4Ta1_ICSD_183995	2.17 eV	1.85 eV
Cu3S4V1_ICSD_15490	1.28 eV	1.01 eV
Cu3S8Ta2Tl3_ICSD_406210	2.10 eV	1.42 eV
Cu3Sb1Se3_ICSD_401095	0.75 eV	0.65 eV
Cu3Sb4Tb3_ICSD_658642	0.24 eV	0.26 eV
Cu3Sb4Y3_ICSD_658636	0.27 eV	0.28 eV
Cu3Se2Tl1_ICSD_629126	0.38 eV	0.41 eV
Cu3Se4Ta1_ICSD_108406	1.80 eV	1.46 eV
Cu3Se4V1_ICSD_629148	1.06 eV	0.86 eV
Cu3Ta1Te4_ICSD_629308	1.27 eV	0.92 eV
Cu3Te4V1_ICSD_629351	0.70 eV	0.69 eV
Cu4Dy4K2S9_ICSD_97562	1.09 eV	1.14 eV
Cu4Dy4K2Se9_ICSD_174374	0.92 eV	1.06 eV
Cu4Ho4K2S9_ICSD_97563	1.12 eV	1.18 eV
Cu4K2Se9Y4_ICSD_174375	0.90 eV	0.98 eV
Cu4Na1O12Te2Tl3_ICSD_421466	1.01 eV	1.10 eV
Cu4Na2S3_ICSD_100829	0.30 eV	0.76 eV
Cu4Ni1S7Si2_ICSD_100778	0.51 eV	1.15 eV
Cu4P8Si1_ICSD_78967	1.24 eV	0.95 eV
Cu4S4Ti1_ICSD_82558	1.69 eV	1.15 eV
Cu5H4O12V2_ICSD_54831	1.24 eV	1.24 eV
Cu5K1O13V3_ICSD_400802	0.88 eV	1.12 eV
Cu5Li2O14Si4_ICSD_2409	1.52 eV	1.43 eV
Cu5Na2O14Si4_ICSD_416179	1.39 eV	1.24 eV
Cu5Na8O10_ICSD_415349	0.10 eV	0.70 eV
Cu5O10P2_ICSD_1292	0.81 eV	0.93 eV
Cu5O4Rb3_ICSD_35246	1.74 eV	1.41 eV
Cu6La4Mn1S10_ICSD_96415	1.66 eV	1.14 eV
Cu6O8Pb1_ICSD_280596	0.20 eV	0.56 eV
Cu7P1S6_ICSD_628639	1.10 eV	1.03 eV
Cu7P1Se6_ICSD_628648	0.21 eV	0.59 eV
Cu8S6Si1_ICSD_24374	0.96 eV	0.88 eV
Dy1F1O1_ICSD_184010	5.01 eV	5.09 eV
Dy1Fe1O3_ICSD_280091	1.26 eV	0.99 eV
Dy1H3O3_ICSD_200095	3.71 eV	3.87 eV
Dy1H4K1O9P2_ICSD_167522	4.67 eV	4.72 eV
Dy1I1S1_ICSD_79107	2.71 eV	2.64 eV
Dy1K1S2_ICSD_44946	2.34 eV	2.32 eV
Dy1Li1O12P4_ICSD_260001	5.93 eV	5.68 eV
Dy1Li1O2_ICSD_422562	4.58 eV	3.80 eV
Dy1Li1O2_ICSD_45512	3.96 eV	3.98 eV
Dy1Li1S2_ICSD_44958	1.92 eV	1.99 eV
Dy1Li1Se2_ICSD_44964	1.54 eV	1.52 eV
Dy1Mn1O3_ICSD_91711	0.45 eV	0.62 eV
Dy1Mn2O5_ICSD_18308	1.07 eV	1.01 eV
Dy1N1_ICSD_629962	0.14 eV	0.21 eV
Dy1Na1S2_ICSD_629966	2.27 eV	2.28 eV

Supplementary Table 774. Five-fold cross validated predictions for the band gap energy (168/277).

system	calculated	predicted
Dy1Na1Se2_ICSD_629969	1.85 eV	1.76 eV
Dy1Ni1Sb1_ICSD_103339	0.15 eV	0.14 eV
Dy1O14P5_ICSD_240973	5.24 eV	5.17 eV
Dy1O19Ta7_ICSD_203232	3.38 eV	3.23 eV
Dy1O1P1Zn1_ICSD_418526	1.42 eV	1.29 eV
Dy1O2Rb1_ICSD_15159	3.60 eV	3.34 eV
Dy1O4P1_ICSD_79756	5.90 eV	6.05 eV
Dy1O4Ta1_ICSD_415435	4.18 eV	4.10 eV
Dy1O4V1_ICSD_247716	2.78 eV	2.78 eV
Dy1O4V1_ICSD_37365	2.96 eV	2.76 eV
Dy1O6Rh1Sr3_ICSD_51055	2.83 eV	2.77 eV
Dy1O8Rb1S2_ICSD_20896	5.68 eV	4.81 eV
Dy1O8Rb1W2_ICSD_22361	3.56 eV	3.51 eV
Dy1O9P3_ICSD_416844	5.59 eV	5.06 eV
Dy1P1S1_ICSD_630062	0.30 eV	0.32 eV
Dy1P5_ICSD_409184	0.14 eV	0.21 eV
Dy1Pt1Sb1_ICSD_53374	0.23 eV	0.35 eV
Dy1Rb1S2_ICSD_81402	2.31 eV	2.29 eV
Dy1S2Tl1_ICSD_57244	1.52 eV	1.48 eV
Dy1Se2Tl1_ICSD_106991	1.26 eV	1.10 eV
Dy1Te2Tl1_ICSD_630345	0.63 eV	0.64 eV
Dy2Hf2O7_ICSD_245138	3.23 eV	3.44 eV
Dy2Mo4O18Sb2_ICSD_417674	3.11 eV	2.85 eV
Dy2O13Te5_ICSD_413664	2.99 eV	3.07 eV
Dy2O1S2_ICSD_36603	2.17 eV	2.29 eV
Dy2O1S2_ICSD_36604	2.56 eV	2.48 eV
Dy2O2S1_ICSD_109332	3.00 eV	2.85 eV
Dy2O2Te1_ICSD_89565	1.09 eV	1.57 eV
Dy2O3_ICSD_160213	4.20 eV	4.17 eV
Dy2O3_ICSD_160228	3.99 eV	4.00 eV
Dy2O3_ICSD_185606	3.93 eV	3.92 eV
Dy2O7Ru2_ICSD_78151	0.55 eV	0.66 eV
Dy2O7Sn2_ICSD_82960	2.21 eV	2.16 eV
Dy2O7Tc2_ICSD_109080	1.40 eV	0.99 eV
Dy2O7Ti2_ICSD_188553	3.24 eV	3.09 eV
Dy2O7V2_ICSD_160863	0.81 eV	1.67 eV
Dy2S3_ICSD_66532	0.73 eV	0.91 eV
Dy2S4Sr1_ICSD_630212	1.79 eV	1.64 eV
Dy2S4Zn1_ICSD_630225	2.40 eV	2.18 eV
Dy2Se3_ICSD_419	0.36 eV	0.80 eV
Dy2Se4Sr1_ICSD_630270	1.41 eV	1.37 eV
Dy3F1O12Se4_ICSD_411748	3.76 eV	3.75 eV
Dy3Fe1Ga1S7_ICSD_236399	1.75 eV	1.68 eV
Dy3Fe1Ga1Se7_ICSD_236400	1.40 eV	1.23 eV
Dy3Fe5O12_ICSD_23856	1.68 eV	1.41 eV
Dy3Ga1O6_ICSD_99495	3.63 eV	3.60 eV
Dy3Ga5O12_ICSD_409391	3.41 eV	3.39 eV
Dy3N1S3_ICSD_416219	1.43 eV	1.41 eV
Dy3N1Se3_ICSD_420156	1.19 eV	1.36 eV
Dy3O7Re1_ICSD_99254	0.35 eV	0.77 eV

Supplementary Table 775. Five-fold cross validated predictions for the band gap energy (169/277).

system	calculated	predicted
Er1F1Mo1O4_ICSD_419253	3.63 eV	3.49 eV
Er1F1Na1O4P1_ICSD_94521	5.52 eV	4.90 eV
Er1F1O1_ICSD_184012	5.18 eV	5.01 eV
Er1F1O3Se1_ICSD_419954	4.30 eV	4.34 eV
Er1F1S1_ICSD_94476	1.33 eV	1.58 eV
Er1F3_ICSD_81411	7.70 eV	7.30 eV
Er1F5K2_ICSD_20404	5.78 eV	5.90 eV
Er1F6Na1Rb2_ICSD_23142	6.49 eV	6.39 eV
Er1Ge1Na1O4_ICSD_54199	3.58 eV	3.47 eV
Er1H1O2_ICSD_34473	3.99 eV	4.15 eV
Er1H3O3_ICSD_200097	3.78 eV	3.74 eV
Er1H3_ICSD_187369	0.18 eV	0.74 eV
Er1I1Se1_ICSD_50194	2.30 eV	1.77 eV
Er1K1O12P4_ICSD_200795	2.59 eV	4.73 eV
Er1K1O2_ICSD_27002	3.96 eV	3.55 eV
Er1K1O8W2_ICSD_157832	3.46 eV	3.36 eV
Er1K1S2_ICSD_44948	2.37 eV	2.36 eV
Er1K1Te2_ICSD_71560	1.33 eV	1.20 eV
Er1Li1O12P4_ICSD_33242	5.71 eV	5.69 eV
Er1Li1O2_ICSD_95380	4.13 eV	4.31 eV
Er1Li1O2_ICSD_95381	4.24 eV	3.57 eV
Er1Li1S2_ICSD_630736	2.01 eV	2.02 eV
Er1Li1Se2_ICSD_44966	1.62 eV	1.57 eV
Er1N1_ICSD_169223	0.16 eV	0.39 eV
Er1Na1O2_ICSD_2739	4.10 eV	3.59 eV
Er1Na1O2_ICSD_97544	4.11 eV	3.49 eV
Er1Na1O8S2_ICSD_90775	5.75 eV	4.95 eV
Er1Na1P2S6_ICSD_415672	2.55 eV	2.25 eV
Er1Na1S2_ICSD_73481	2.31 eV	2.23 eV
Er1Na1Se2_ICSD_50195	1.88 eV	1.78 eV
Er1Ni1Sb1_ICSD_103273	0.14 eV	0.17 eV
Er1O14P5_ICSD_23304	5.24 eV	5.11 eV
Er1O2Rb1_ICSD_15161	3.65 eV	3.46 eV
Er1O3V1_ICSD_161781	0.79 eV	1.00 eV
Er1O4P1_ICSD_184547	5.92 eV	5.45 eV
Er1O4Ta1_ICSD_415437	4.20 eV	3.73 eV
Er1O4V1_ICSD_69118	2.71 eV	2.72 eV
Er1O4V1_ICSD_78080	2.96 eV	2.83 eV
Er1O6Rh1Sr3_ICSD_51057	2.86 eV	2.71 eV
Er1O9P3_ICSD_20935	5.74 eV	5.39 eV
Er1P1Pd1_ICSD_69673	0.10 eV	0.25 eV
Er1P1S1_ICSD_630913	0.19 eV	0.53 eV
Er1Pd1Sb1_ICSD_57259	0.12 eV	0.28 eV
Er1Rb1S2_ICSD_81404	2.36 eV	2.25 eV
Er1Rb1Se2_ICSD_281075	1.98 eV	1.91 eV
Er1S2Tl1_ICSD_106620	1.41 eV	1.41 eV
Er1Se2Tl1_ICSD_106993	1.22 eV	1.25 eV
Er1Te2Tl1_ICSD_631187	0.62 eV	0.64 eV
Er2Eu1S4_ICSD_630396	1.35 eV	1.37 eV
Er2Eu1Se4_ICSD_630398	1.20 eV	1.04 eV

Supplementary Table 776. Five-fold cross validated predictions for the band gap energy (170/277).

system	calculated	predicted
Er2Ge2O7_ICSD_16164	3.65 eV	3.66 eV
Er2Hf2O7_ICSD_162006	3.01 eV	3.41 eV
Er2Mg1Se4_ICSD_630754	1.37 eV	1.27 eV
Er2Mn2O7_ICSD_202517	0.91 eV	0.90 eV
Er2O11Te4_ICSD_413661	3.23 eV	2.92 eV
Er2O13Te5_ICSD_413666	3.00 eV	2.96 eV
Er2O1S2_ICSD_67379	2.64 eV	2.49 eV
Er2O2S1_ICSD_109334	3.07 eV	2.92 eV
Er2O2Se1_ICSD_25810	2.19 eV	2.02 eV
Er2O3_ICSD_160215	4.20 eV	4.29 eV
Er2O3_ICSD_160230	4.11 eV	4.12 eV
Er2O3_ICSD_39189	3.96 eV	3.93 eV
Er2O5Si1_ICSD_419309	4.70 eV	4.34 eV
Er2O7Ru2_ICSD_97533	0.56 eV	0.64 eV
Er2O7Si2_ICSD_74779	4.79 eV	4.44 eV
Er2O7Sn2_ICSD_84749	2.17 eV	2.37 eV
Er2O7Tc2_ICSD_109081	1.29 eV	0.93 eV
Er2O7Ti2_ICSD_24209	3.11 eV	3.04 eV
Er2O7V2_ICSD_160866	0.85 eV	1.72 eV
Er2O9Se3_ICSD_411749	3.93 eV	3.95 eV
Er2Pb1S4_ICSD_630919	2.25 eV	1.75 eV
Er2Pb1Se4_ICSD_630921	1.81 eV	1.35 eV
Er2S3_ICSD_631065	0.63 eV	0.96 eV
Er2S3_ICSD_72292	1.46 eV	1.20 eV
Er2S3_ICSD_73558	1.16 eV	0.84 eV
Er2S4Sr1_ICSD_631075	1.81 eV	1.67 eV
Er2S4Yb1_ICSD_631087	1.18 eV	1.24 eV
Er2S4Zn1_ICSD_631088	2.43 eV	2.23 eV
Er2Se4Sr1_ICSD_631123	1.43 eV	1.46 eV
Er2Se4Yb1_ICSD_631130	1.11 eV	1.19 eV
Er3F10K1_ICSD_418210	7.06 eV	6.65 eV
Er3Fe5O12_ICSD_71464	0.09 eV	1.13 eV
Er3Ga1O6_ICSD_99497	3.57 eV	3.66 eV
Er3Ga1S6_ICSD_630576	2.12 eV	1.86 eV
Er3Ga5O12_ICSD_9238	3.45 eV	3.51 eV
Er3La1S6_ICSD_630730	1.02 eV	1.10 eV
Er3Nd1S6_ICSD_630810	0.97 eV	1.09 eV
Er6I10Ir1_ICSD_424428	0.10 eV	0.69 eV
Er6I10Ni1_ICSD_424429	0.53 eV	0.91 eV
Eu1F1Mo1O4_ICSD_419248	0.26 eV	1.81 eV
Eu1F1O1_ICSD_184006	0.15 eV	1.21 eV
Eu1F2_ICSD_260882	2.11 eV	1.65 eV
Eu1F3_ICSD_95244	0.80 eV	2.10 eV
Eu1F4Mg1_ICSD_86246	2.67 eV	2.95 eV
Eu1F7Sn1_ICSD_71585	0.90 eV	2.08 eV
Eu1Ga2Se4_ICSD_631269	1.49 eV	1.51 eV
Eu1Ge1Li2S4_ICSD_281012	1.85 eV	1.91 eV
Eu1Ge1Na1O4_ICSD_186847	0.28 eV	1.61 eV
Eu1H1O12P4_ICSD_250030	0.39 eV	2.43 eV
Eu1H2_ICSD_631320	1.57 eV	1.59 eV

Supplementary Table 777. Five-fold cross validated predictions for the band gap energy (171/277).

system	calculated	predicted
Eu1H3Li1_ICSD_416461	0.46 eV	1.49 eV
Eu1H4Mg1Ni1_ICSD_657978	1.50 eV	1.92 eV
Eu1Ho2S4_ICSD_631336	1.41 eV	1.33 eV
Eu1Ho2Se4_ICSD_631338	1.21 eV	0.97 eV
Eu1I2_ICSD_22131	3.26 eV	3.00 eV
Eu1I2_ICSD_260561	3.09 eV	2.86 eV
Eu1I2_ICSD_56816	3.34 eV	3.03 eV
Eu1In2Se4_ICSD_631352	0.57 eV	0.93 eV
Eu1Ir2P2_ICSD_73530	0.46 eV	0.52 eV
Eu1K1Na1Nb1O5_ICSD_420036	2.08 eV	2.31 eV
Eu1K1O4P1_ICSD_36647	2.95 eV	2.48 eV
Eu1K1P1S4_ICSD_279622	0.79 eV	1.81 eV
Eu1K1P1Se4_ICSD_280200	1.18 eV	1.23 eV
Eu1Li1O12P4_ICSD_416878	0.32 eV	2.51 eV
Eu1Li1O2_ICSD_422560	0.47 eV	1.51 eV
Eu1Li1O4Ti1_ICSD_81858	0.17 eV	1.69 eV
Eu1Li1O8S2_ICSD_200222	0.30 eV	1.61 eV
Eu1Li1P1Se4_ICSD_280199	1.06 eV	1.37 eV
Eu1Li2O4Si1_ICSD_50442	2.09 eV	2.01 eV
Eu1Mg2Sb2_ICSD_412653	0.17 eV	0.48 eV
Eu1N2O2Si2_ICSD_416046	2.31 eV	1.69 eV
Eu1N7Si4Y1_ICSD_150460	2.20 eV	1.87 eV
Eu1Na10Sn12_ICSD_172209	0.65 eV	0.61 eV
Eu1Nb2O6_ICSD_247276	0.70 eV	1.84 eV
Eu1O1_ICSD_53437	0.17 eV	0.84 eV
Eu1O2Rb1_ICSD_27334	0.49 eV	1.64 eV
Eu1O3Ti1_ICSD_187102	0.72 eV	1.00 eV
Eu1O3Ti1_ICSD_187108	0.77 eV	0.66 eV
Eu1O3Ti1_ICSD_187204	0.78 eV	0.74 eV
Eu1O4P1Rb1_ICSD_36648	3.11 eV	2.74 eV
Eu1O4S1_ICSD_68244	3.58 eV	1.91 eV
Eu1O4Sb1_ICSD_245019	0.43 eV	0.63 eV
Eu1O4Ta1_ICSD_415432	0.26 eV	1.91 eV
Eu1O4W1_ICSD_185361	1.86 eV	2.05 eV
Eu1O6Rh1Sr3_ICSD_51053	2.02 eV	1.69 eV
Eu1O8Rb1S2_ICSD_48002	0.12 eV	1.73 eV
Eu1P2Zn2_ICSD_631503	0.09 eV	0.48 eV
Eu1S1_ICSD_183957	0.59 eV	0.49 eV
Eu1S1_ICSD_631595	0.65 eV	0.81 eV
Eu1Se1_ICSD_183958	0.38 eV	0.54 eV
Eu1Se1_ICSD_631657	0.85 eV	0.67 eV
Eu1Se4Y2_ICSD_631671	1.23 eV	1.13 eV
Eu1Te1_ICSD_33612	0.84 eV	0.88 eV
Eu2F1K5O13Si4_ICSD_249458	1.98 eV	2.23 eV
Eu2Ga2Ge1S7_ICSD_262242	1.37 eV	1.95 eV
Eu2Ge1S4_ICSD_8242	1.85 eV	1.39 eV
Eu2Ge1Se4_ICSD_412227	1.31 eV	1.15 eV
Eu2Ge1Se4_ICSD_412228	1.15 eV	1.21 eV
Eu2H6Ru1_ICSD_656083	1.78 eV	1.78 eV
Eu2I1P1_ICSD_202068	0.84 eV	0.92 eV

Supplementary Table 778. Five-fold cross validated predictions for the band gap energy (172/277).

system	calculated	predicted
Eu2O3_ICSD_659185	0.14 eV	0.54 eV
Eu2O3_ICSD_8056	0.18 eV	0.22 eV
Eu2O4Si1_ICSD_1510	1.72 eV	1.85 eV
Eu2O4Si1_ICSD_23615	1.99 eV	1.46 eV
Eu2O7Si2_ICSD_34586	0.27 eV	1.64 eV
Eu2O7Ti2_ICSD_92767	0.26 eV	1.47 eV
Eu2S4Si1_ICSD_95775	1.93 eV	1.98 eV
Eu2S4Sn1_ICSD_413022	0.98 eV	1.34 eV
Eu3F10Rb1_ICSD_14027	1.06 eV	2.08 eV
Eu3Ga1O6_ICSD_99492	0.40 eV	1.05 eV
Eu3O12Sb5_ICSD_67023	0.31 eV	1.39 eV
Eu4I6O1_ICSD_281554	2.88 eV	2.56 eV
Eu4O1Sb2_ICSD_402953	0.15 eV	0.34 eV
Eu5O15Ta4_ICSD_166562	1.40 eV	1.82 eV
F10H1P1Pb2_ICSD_419141	5.45 eV	3.95 eV
F10Hg3Nb2O4S1_ICSD_60920	2.40 eV	2.56 eV
F10Hg3O4S1Ta2_ICSD_60922	2.43 eV	2.97 eV
F10In3Rb1_ICSD_200052	3.65 eV	3.64 eV
F10K1Tb3_ICSD_28214	6.96 eV	6.15 eV
F10K1Y3_ICSD_155137	6.95 eV	6.48 eV
F10K1Yb3_ICSD_28258	0.97 eV	0.89 eV
F10Na1Sb3_ICSD_1968	4.96 eV	4.40 eV
F10Nb1Sb1_ICSD_16095	4.73 eV	4.21 eV
F10Pb3Zr1_ICSD_100600	4.67 eV	4.23 eV
F11Fe1Na1Zr2_ICSD_81220	3.11 eV	3.60 eV
F11Hf2Na1V1_ICSD_94460	2.89 eV	3.57 eV
F11Kr3Sb1_ICSD_279628	1.23 eV	2.19 eV
F11La1Zr2_ICSD_424909	5.77 eV	5.01 eV
F11Mn1Na1Zr2_ICSD_81219	4.27 eV	3.53 eV
F11Na1Ni1Zr2_ICSD_81222	4.97 eV	3.60 eV
F11Na1Pd1Zr2_ICSD_77241	3.12 eV	3.87 eV
F11Na1V1Zr2_ICSD_78868	1.89 eV	3.51 eV
F11Na1Zn1Zr2_ICSD_81223	5.90 eV	4.66 eV
F11Pr1Zr2_ICSD_72156	3.95 eV	4.81 eV
F12Fe2Li3Na3_ICSD_17056	3.78 eV	2.93 eV
F12Ge5_ICSD_10295	3.89 eV	4.22 eV
F12I4Sb2_ICSD_63301	0.55 eV	1.98 eV
F12K2O1Zr3_ICSD_281350	5.42 eV	5.13 eV
F12Mn1Sb2_ICSD_411522	0.50 eV	2.26 eV
F12O1Tl2Zr3_ICSD_48003	4.25 eV	4.47 eV
F12O8Os2Xe2_ICSD_260293	2.61 eV	2.22 eV
F12P4Pt1_ICSD_418726	4.99 eV	3.21 eV
F12Sb2Te4_ICSD_201222	1.16 eV	2.18 eV
F13H4P1Sr2_ICSD_415154	7.24 eV	6.34 eV
F13K1Sb4_ICSD_24740	4.75 eV	4.62 eV
F13K1Sb4_ICSD_4049	4.68 eV	4.46 eV
F13N1O2Xe2_ICSD_404987	2.89 eV	2.48 eV
F13Na5Zr2_ICSD_14133	5.56 eV	5.14 eV
F14H2Mg1Sb2_ICSD_412893	4.40 eV	4.35 eV
F14K4Sb2Sn1_ICSD_241228	3.57 eV	4.28 eV

Supplementary Table 779. Five-fold cross validated predictions for the band gap energy (173/277).

system	calculated	predicted
F14N2Ni1_ICSD_26397	2.29 eV	2.74 eV
F14Sb4_ICSD_35709	3.84 eV	3.23 eV
F14Sr4Zn3_ICSD_72991	5.85 eV	5.67 eV
F15Pr1Zr3_ICSD_72838	4.88 eV	4.20 eV
F16Ge7_ICSD_405946	4.36 eV	3.79 eV
F18Mg6Rb6_ICSD_410385	6.68 eV	6.73 eV
F18Ni6Rb6_ICSD_410390	4.06 eV	3.58 eV
F18P2Pb1Xe3_ICSD_249570	2.50 eV	2.86 eV
F18P2Sr1Xe3_ICSD_249568	2.60 eV	3.85 eV
F1Fe1H4Na1O6S1_ICSD_169110	3.72 eV	2.89 eV
F1Fe1Li1O4S1_ICSD_180389	2.61 eV	2.64 eV
F1Fe1Na1O4S1_ICSD_262276	3.53 eV	3.09 eV
F1Fe1O3Sr2_ICSD_93510	0.69 eV	1.14 eV
F1Fe1O3Te1_ICSD_241141	2.18 eV	1.96 eV
F1Fe1O4S1_ICSD_182945	1.89 eV	2.57 eV
F1Ga1O3Te1_ICSD_241140	3.68 eV	3.22 eV
F1Ga1O4Sr3_ICSD_50734	3.70 eV	3.49 eV
F1Gd1Mo1O4_ICSD_419249	3.66 eV	3.26 eV
F1Gd1O1_ICSD_184007	4.57 eV	4.42 eV
F1Gd1O4S1_ICSD_410324	5.73 eV	4.81 eV
F1Gd1S1_ICSD_93355	1.07 eV	1.26 eV
F1Gd3O12Se4_ICSD_411086	3.80 eV	3.65 eV
F1H10N2O4P1_ICSD_2803	4.91 eV	5.04 eV
F1H1Hg1O1_ICSD_200744	1.46 eV	1.57 eV
F1H1Hg1O3Te1_ICSD_413078	2.92 eV	2.13 eV
F1H1Mg1O1_ICSD_186501	4.72 eV	4.97 eV
F1H1Na1O4P1Sn1_ICSD_96768	3.56 eV	3.41 eV
F1H1O1_ICSD_63681	3.22 eV	4.44 eV
F1H1O3S1_ICSD_65781	3.06 eV	3.72 eV
F1H2K1Li1O4P1_ICSD_2044	4.97 eV	4.93 eV
F1H2O1Rb1_ICSD_250327	5.32 eV	5.27 eV
F1H3K1O3P1_ICSD_9151	6.54 eV	5.47 eV
F1H3O1_ICSD_24382	6.73 eV	5.92 eV
F1H3O4S1_ICSD_33866	5.49 eV	5.50 eV
F1H3Si1_ICSD_60065	6.03 eV	6.01 eV
F1H4K1O2_ICSD_165373	5.15 eV	4.79 eV
F1H4K1O4_ICSD_22366	4.22 eV	4.61 eV
F1H4Li1N1O3P1_ICSD_1439	5.23 eV	5.13 eV
F1H4Mg1Na1O6S1_ICSD_201712	5.02 eV	5.31 eV
F1H4N1_ICSD_14294	5.96 eV	5.49 eV
F1H4Na1Ni1O6S1_ICSD_169112	1.52 eV	2.77 eV
F1H5N2_ICSD_6019	5.45 eV	5.15 eV
F1H6N1Na1O4P1_ICSD_240974	3.47 eV	4.41 eV
F1H6N1O2_ICSD_28552	3.47 eV	5.13 eV
F1H6Na1O6Te1_ICSD_42	3.57 eV	3.67 eV
F1H7N2_ICSD_419918	4.88 eV	5.01 eV
F1Hg2O3P1_ICSD_414292	1.45 eV	1.99 eV
F1Ho1Mo1O4_ICSD_419252	3.65 eV	3.52 eV
F1Ho1O1_ICSD_184011	5.10 eV	5.02 eV
F1Ho1O3Se1_ICSD_419000	4.30 eV	4.34 eV

Supplementary Table 780. Five-fold cross validated predictions for the band gap energy (174/277).

system	calculated	predicted
F1Ho1S1_ICSD_94475	1.30 eV	1.58 eV
F1Ho3O12Se4_ICSD_419001	3.71 eV	3.95 eV
F1I1O2_ICSD_280804	2.89 eV	2.45 eV
F1I1Pb1_ICSD_155012	2.17 eV	2.34 eV
F1I1Sr1_ICSD_155009	4.30 eV	4.01 eV
F1In1O1_ICSD_2521	1.50 eV	2.55 eV
F1In1O3Te1_ICSD_260009	3.04 eV	2.97 eV
F1K1O2S1_ICSD_89602	3.99 eV	4.54 eV
F1K1O2Se1_ICSD_78398	3.32 eV	3.67 eV
F1K1O3Xe1_ICSD_15219	2.14 eV	2.93 eV
F1K1O4S1Sn1_ICSD_94532	3.84 eV	3.65 eV
F1K1O6Te3_ICSD_95727	3.09 eV	2.97 eV
F1K1_ICSD_61558	6.22 eV	5.93 eV
F1K1_ICSD_64686	5.96 eV	6.13 eV
F1K2O3P1_ICSD_200441	4.45 eV	4.44 eV
F1K3O4S1_ICSD_81835	5.22 eV	4.58 eV
F1K3O4Se1_ICSD_82750	3.84 eV	3.66 eV
F1La1O1_ICSD_30622	4.33 eV	4.47 eV
F1La1O1_ICSD_76427	4.74 eV	4.49 eV
F1La1S1_ICSD_93350	1.41 eV	1.68 eV
F1La1Se1_ICSD_21010	1.75 eV	1.39 eV
F1Li1Ni1O4S1_ICSD_180391	3.18 eV	2.77 eV
F1Li1O3S1_ICSD_1384	5.79 eV	5.19 eV
F1Li1O4P1V1_ICSD_184601	1.60 eV	2.14 eV
F1Li1O4Si1Sr2_ICSD_79805	4.79 eV	4.62 eV
F1Li1_ICSD_18012	8.74 eV	6.74 eV
F1Li1_ICSD_184904	7.49 eV	5.01 eV
F1Lu1O3Se1_ICSD_417449	4.13 eV	4.52 eV
F1Lu1S1_ICSD_89549	2.26 eV	1.85 eV
F1Mg1Na1O4S1_ICSD_262272	5.59 eV	5.25 eV
F1Mg2N1_ICSD_17021	2.13 eV	3.54 eV
F1Mn2O4P1_ICSD_2814	2.84 eV	2.45 eV
F1Mo1O4Tb1_ICSD_419250	3.67 eV	3.57 eV
F1Mo1O4Y1_ICSD_249646	3.64 eV	3.29 eV
F1N1O1_ICSD_411510	3.18 eV	2.18 eV
F1N1O3Xe1_ICSD_174511	2.37 eV	2.37 eV
F1N1Th1_ICSD_236315	2.14 eV	2.80 eV
F1N3O2S4_ICSD_14028	2.15 eV	2.46 eV
F1Na1Nb6O15_ICSD_24109	1.83 eV	2.05 eV
F1Na1O4P1Y1_ICSD_51463	5.49 eV	4.99 eV
F1Na1O4S1Zn1_ICSD_262274	4.81 eV	4.52 eV
F1Na1_ICSD_44276	6.13 eV	5.77 eV
F1Na3O4S1_ICSD_9519	2.22 eV	4.05 eV
F1Na3O4W1_ICSD_417289	4.50 eV	3.98 eV
F1Na5O16Si4Y4_ICSD_20795	4.77 eV	4.10 eV
F1Nd1O1_ICSD_95655	3.85 eV	3.36 eV
F1Nd1S1_ICSD_94471	1.23 eV	1.32 eV
F1Nd1Se1_ICSD_108968	0.87 eV	1.10 eV
F1Nd1Te1_ICSD_108969	0.33 eV	0.85 eV
F1O1Sb1_ICSD_19019	3.30 eV	2.99 eV

Supplementary Table 781. Five-fold cross validated predictions for the band gap energy (175/277).

system	calculated	predicted
F1O1Sc1_ICSD_100564	4.79 eV	4.75 eV
F1O1Tb1_ICSD_184009	4.94 eV	4.58 eV
F1O1Y1_ICSD_184004	4.91 eV	4.66 eV
F1O1Y1_ICSD_76426	4.95 eV	4.81 eV
F1O1Yb1_ICSD_75557	0.28 eV	0.50 eV
F1O2Rb1S1_ICSD_93064	4.10 eV	4.15 eV
F1O3P1Rb2_ICSD_172343	4.31 eV	4.29 eV
F1O3P1Sn1_ICSD_2039	3.60 eV	3.54 eV
F1O3S1Tl1_ICSD_424114	4.55 eV	3.78 eV
F1O3Se1Y1_ICSD_418898	4.27 eV	4.16 eV
F1O3Se1Yb1_ICSD_419953	0.08 eV	0.97 eV
F1O3Tc1_ICSD_249509	2.49 eV	1.98 eV
F1O4P1V1_ICSD_183878	1.32 eV	1.63 eV
F1O4Rb1S1_ICSD_8275	2.42 eV	4.11 eV
F1O4S1Y1_ICSD_92288	5.84 eV	5.20 eV
F1Pr1S1_ICSD_94470	1.25 eV	1.35 eV
F1Rb1_ICSD_53828	5.54 eV	5.64 eV
F1Rb1_ICSD_61562	5.93 eV	5.68 eV
F1S1Tb1_ICSD_94474	1.26 eV	1.37 eV
F1S1Y1_ICSD_2597	2.56 eV	1.88 eV
F1S1Y1_ICSD_87130	1.31 eV	2.24 eV
F1Se1Y1_ICSD_1827	2.17 eV	1.70 eV
F1Tl1_ICSD_16112	3.05 eV	2.53 eV
F1Tl1_ICSD_16113	3.07 eV	3.04 eV
F1Tl1_ICSD_90992	3.18 eV	3.07 eV
F1Tl1_ICSD_90993	3.00 eV	2.76 eV
F1Tl1_ICSD_9893	2.19 eV	2.68 eV
F1_ICSD_426939	0.18 eV	2.25 eV
F20O2Re2Sb2_ICSD_201546	2.12 eV	2.38 eV
F24Sb2Xe6Zn1_ICSD_416302	2.61 eV	2.42 eV
F25K1Th6_ICSD_2711	6.87 eV	5.53 eV
F2Fe1_ICSD_73729	2.63 eV	2.64 eV
F2Fe2H1O3P1_ICSD_391478	3.32 eV	2.45 eV
F2Fe2O1S2Sr2_ICSD_249688	1.43 eV	1.34 eV
F2Fe2O1Se2Sr2_ICSD_249690	1.45 eV	1.49 eV
F2Ge1_ICSD_18030	4.10 eV	3.99 eV
F2H1K1_ICSD_9345	6.91 eV	6.78 eV
F2H1Li1_ICSD_23883	8.03 eV	7.33 eV
F2H1N1O4S2_ICSD_50523	5.73 eV	4.70 eV
F2H1N1_ICSD_404200	4.42 eV	4.62 eV
F2H1Na1_ICSD_26870	6.83 eV	6.15 eV
F2H1P1S1_ICSD_63001	4.66 eV	4.06 eV
F2H1P1_ICSD_406360	3.80 eV	5.52 eV
F2H1Rb1_ICSD_45859	6.68 eV	6.61 eV
F2H2N1P1_ICSD_201528	4.81 eV	4.84 eV
F2H2O5S1Sn2_ICSD_39455	3.82 eV	3.46 eV
F2H4N1O2P1_ICSD_16152	5.20 eV	5.45 eV
F2H4O1_ICSD_24383	7.03 eV	6.55 eV
F2H5In1N2_ICSD_87965	2.44 eV	3.70 eV
F2H8O4Zn1_ICSD_14064	5.29 eV	4.41 eV

Supplementary Table 782. Five-fold cross validated predictions for the band gap energy (176/277).

system	calculated	predicted
F2Hg1_ICSD_33614	0.97 eV	1.65 eV
F2Hg2_ICSD_27700	1.78 eV	1.71 eV
F2Hg3S2_ICSD_16927	1.83 eV	1.64 eV
F2I1K1O2_ICSD_26600	3.91 eV	3.79 eV
F2I1Na1O2_ICSD_260003	4.28 eV	3.69 eV
F2I2O6S2_ICSD_74647	1.96 eV	2.98 eV
F2K1Mg2Na1O10Si4_ICSD_98196	5.21 eV	4.87 eV
F2K1O2P1_ICSD_26640	5.23 eV	5.26 eV
F2K2O5P2_ICSD_1659	5.03 eV	5.00 eV
F2K3Na1O6P2_ICSD_1904	4.51 eV	4.66 eV
F2Kr1_ICSD_23534	2.79 eV	3.09 eV
F2Kr1_ICSD_279623	2.91 eV	3.25 eV
F2Li1Rb1_ICSD_18019	6.12 eV	6.16 eV
F2Mg1_ICSD_51242	5.64 eV	6.01 eV
F2Mg1_ICSD_51243	6.37 eV	5.61 eV
F2Mg1_ICSD_94279	6.83 eV	6.82 eV
F2Mg1_ICSD_94280	6.84 eV	6.83 eV
F2Mg1_ICSD_94283	6.73 eV	6.75 eV
F2Mn1_ICSD_12167	3.35 eV	2.71 eV
F2Mn1_ICSD_20365	2.80 eV	3.14 eV
F2Mn1_ICSD_53985	2.57 eV	2.62 eV
F2N1P1_ICSD_9684	5.62 eV	5.61 eV
F2N3O1P1_ICSD_248120	4.83 eV	4.60 eV
F2Na1Nb1O2_ICSD_23238	3.33 eV	3.50 eV
F2Na1O2V1_ICSD_75418	2.21 eV	2.55 eV
F2Ni1_ICSD_34307	4.02 eV	3.60 eV
F2Ni1_ICSD_73726	4.01 eV	3.47 eV
F2O1Pb2_ICSD_10416	2.84 eV	1.68 eV
F2O1Pb2_ICSD_76964	0.89 eV	2.46 eV
F2O1Se1_ICSD_12110	4.15 eV	3.61 eV
F2O1Te1_ICSD_88415	4.21 eV	3.01 eV
F2O2P1Rb1_ICSD_1980	5.18 eV	5.13 eV
F2O2S1_ICSD_62968	5.88 eV	4.85 eV
F2O2U1_ICSD_31630	4.64 eV	3.35 eV
F2O2Xe1_ICSD_10203	2.46 eV	2.44 eV
F2O3Os1_ICSD_73732	2.36 eV	2.21 eV
F2O3S1Xe1_ICSD_10357	2.46 eV	2.56 eV
F2O3Te2_ICSD_82162	3.36 eV	2.49 eV
F2O4Rb1S1Sb1_ICSD_32709	4.68 eV	4.01 eV
F2O7Te2V2_ICSD_173748	2.18 eV	1.97 eV
F2Pb1_ICSD_154995	4.37 eV	3.64 eV
F2Pb1_ICSD_86738	4.40 eV	3.25 eV
F2Pd1_ICSD_100567	1.77 eV	1.54 eV
F2Pd1_ICSD_16763	1.34 eV	1.62 eV
F2S3Sn1Sr2_ICSD_171346	1.83 eV	1.96 eV
F2Sb2Se4Sr2_ICSD_171430	0.22 eV	1.06 eV
F2Se3Sn1Sr2_ICSD_171345	1.28 eV	1.47 eV
F2Sn1_ICSD_10485	3.36 eV	2.92 eV
F2Sn1_ICSD_14194	3.30 eV	3.31 eV
F2Sn1_ICSD_14195	3.31 eV	3.25 eV

Supplementary Table 783. Five-fold cross validated predictions for the band gap energy (177/277).

system	calculated	predicted
F2Sr1_ICSD_260881	6.78 eV	6.70 eV
F2Sr1_ICSD_262350	5.86 eV	5.94 eV
F2Ti1_ICSD_65410	2.03 eV	2.58 eV
F2V1_ICSD_201245	1.71 eV	1.70 eV
F2Xe1_ICSD_26625	2.66 eV	3.05 eV
F2Zn1_ICSD_20364	4.97 eV	4.90 eV
F2Zn1_ICSD_280605	4.95 eV	4.76 eV
F2_ICSD_16262	2.60 eV	2.19 eV
F2_ICSD_22271	2.50 eV	2.59 eV
F3Fe1Na1_ICSD_68981	2.68 eV	3.02 eV
F3Fe1_ICSD_202047	2.80 eV	1.91 eV
F3Fe1_ICSD_240399	2.68 eV	1.98 eV
F3Fe1_ICSD_29132	2.51 eV	2.09 eV
F3Ga1H3N1_ICSD_89503	1.84 eV	4.42 eV
F3Ga1_ICSD_409507	4.92 eV	4.72 eV
F3H1Sr1_ICSD_35408	5.95 eV	6.15 eV
F3H2P1_ICSD_406359	6.43 eV	5.85 eV
F3Hg1Rb1_ICSD_15169	0.65 eV	1.21 eV
F3Ho1_ICSD_200955	7.74 eV	7.23 eV
F3I1O1_ICSD_4076	3.64 eV	2.91 eV
F3I1Sn2_ICSD_2419	2.45 eV	2.76 eV
F3I1_ICSD_411036	1.73 eV	2.49 eV
F3In1_ICSD_18028	4.20 eV	3.30 eV
F3Ir1_ICSD_77619	2.05 eV	1.74 eV
F3K1Mg1_ICSD_56096	6.93 eV	6.56 eV
F3K1Mn1_ICSD_246921	2.75 eV	2.53 eV
F3K1Mn1_ICSD_35507	2.81 eV	2.67 eV
F3K1Mn1_ICSD_37254	2.70 eV	2.53 eV
F3K1Mn1_ICSD_4054	2.72 eV	2.68 eV
F3K1Mn1_ICSD_89343	2.72 eV	2.75 eV
F3K1Ni1_ICSD_44786	3.65 eV	2.74 eV
F3K1O1Te1_ICSD_201155	4.01 eV	4.29 eV
F3K1Pd1_ICSD_73167	0.92 eV	2.05 eV
F3K1V1_ICSD_28145	1.81 eV	1.23 eV
F3K1Zn1_ICSD_63157	5.17 eV	5.43 eV
F3K2O2V1_ICSD_9122	2.99 eV	2.55 eV
F3K3N3O9Sb1_ICSD_35677	3.08 eV	3.21 eV
F3K3O4Ti1_ICSD_39273	3.07 eV	3.12 eV
F3K3O4Ti1_ICSD_39811	3.07 eV	3.07 eV
F3La1_ICSD_164054	6.19 eV	5.27 eV
F3La1_ICSD_164055	5.81 eV	6.28 eV
F3La1_ICSD_167553	6.99 eV	6.82 eV
F3La1_ICSD_16964	5.54 eV	6.93 eV
F3La1_ICSD_246323	7.71 eV	6.38 eV
F3La1_ICSD_28538	7.66 eV	6.40 eV
F3La1_ICSD_34108	7.29 eV	7.25 eV
F3La1_ICSD_96134	5.12 eV	6.03 eV
F3La3O9Si3_ICSD_89522	5.33 eV	4.85 eV
F3Mg1Na1_ICSD_156157	6.61 eV	6.59 eV
F3Mg1Na1_ICSD_156158	6.61 eV	6.61 eV

Supplementary Table 784. Five-fold cross validated predictions for the band gap energy (178/277).

system	calculated	predicted
F3Mg1Na1_ICSD_171813	5.93 eV	6.35 eV
F3Mg1Na1_ICSD_90283	6.75 eV	6.60 eV
F3Mg1Rb1_ICSD_49585	6.93 eV	6.63 eV
F3Mg3N1_ICSD_262326	3.58 eV	4.91 eV
F3Mn1Na1_ICSD_65770	2.71 eV	2.82 eV
F3Mn1Rb1_ICSD_43722	2.97 eV	2.62 eV
F3Mn1_ICSD_19080	0.72 eV	1.43 eV
F3Mn1_ICSD_73113	0.66 eV	1.46 eV
F3Mo1Na3O3_ICSD_97452	3.87 eV	3.02 eV
F3Mo1_ICSD_30612	1.74 eV	2.16 eV
F3Mo1_ICSD_68527	2.50 eV	1.75 eV
F3N3S3_ICSD_21015	4.07 eV	3.28 eV
F3Na1Ni1_ICSD_423598	4.38 eV	3.40 eV
F3Na1Ni1_ICSD_9008	3.96 eV	3.63 eV
F3Na1V1_ICSD_60611	0.43 eV	1.60 eV
F3Na1Zn1_ICSD_69349	5.15 eV	5.40 eV
F3Na7O12S3Sn1_ICSD_250242	4.91 eV	4.08 eV
F3Nd1_ICSD_155557	5.26 eV	5.03 eV
F3Nd1_ICSD_16967	4.28 eV	4.82 eV
F3Nd1_ICSD_63049	5.21 eV	4.97 eV
F3Ni1_ICSD_87943	1.22 eV	1.95 eV
F3O1Rb1V1_ICSD_249403	2.30 eV	2.18 eV
F3O1V1_ICSD_249507	3.62 eV	2.34 eV
F3O2Re1_ICSD_415421	3.50 eV	2.59 eV
F3O4P1Sn3_ICSD_37133	3.77 eV	3.30 eV
F3Pb1Rb1_ICSD_49591	3.05 eV	2.84 eV
F3Pd1Rb1_ICSD_73166	0.96 eV	1.92 eV
F3Pr1_ICSD_16966	4.15 eV	4.65 eV
F3Pr1_ICSD_77741	5.16 eV	4.26 eV
F3Rb1V1_ICSD_28146	2.19 eV	1.58 eV
F3Rb1Yb1_ICSD_49590	1.09 eV	1.70 eV
F3Rb1Zn1_ICSD_41618	5.26 eV	5.11 eV
F3Rh1_ICSD_29134	1.26 eV	1.84 eV
F3Rh1_ICSD_62262	2.08 eV	1.44 eV
F3Ru1_ICSD_16673	0.53 eV	1.26 eV
F3Sb1_ICSD_30411	4.49 eV	3.60 eV
F3Sc1_ICSD_261083	6.57 eV	6.43 eV
F3Sc1_ICSD_30215	6.08 eV	6.48 eV
F3Sc1_ICSD_36011	6.61 eV	6.19 eV
F3Tb1_ICSD_167473	7.74 eV	6.79 eV
F3Ti1_ICSD_52159	1.91 eV	2.57 eV
F3Tl1_ICSD_10365	1.25 eV	2.32 eV
F3U1_ICSD_24966	2.86 eV	3.71 eV
F3V1_ICSD_30624	1.13 eV	1.67 eV
F3Y1_ICSD_15961	5.60 eV	6.16 eV
F3Y1_ICSD_6023	7.60 eV	6.69 eV
F3Yb1_ICSD_9844	0.81 eV	1.40 eV
F4Fe1K1_ICSD_60615	2.72 eV	2.39 eV
F4Fe1Na1_ICSD_15501	3.00 eV	2.79 eV
F4Fe1Rb1_ICSD_21071	2.64 eV	2.43 eV

Supplementary Table 785. Five-fold cross validated predictions for the band gap energy (179/277).

system	calculated	predicted
F4Fe1Rb1_ICSD_21072	2.38 eV	2.19 eV
F4Fe1Rb1_ICSD_85499	2.49 eV	2.31 eV
F4Fe1Rb1_ICSD_85500	2.54 eV	2.23 eV
F4Ga1H4N1_ICSD_410849	5.26 eV	4.58 eV
F4Ge1_ICSD_202558	5.27 eV	4.13 eV
F4H1K1O1Te1_ICSD_155199	5.03 eV	4.58 eV
F4H1Mn1Na2O3P1_ICSD_424942	3.25 eV	3.06 eV
F4H1O1Rb1Te1_ICSD_155198	4.97 eV	4.24 eV
F4H1P1_ICSD_406358	7.45 eV	6.10 eV
F4H2O3Te2_ICSD_9653	1.86 eV	2.90 eV
F4H3K1_ICSD_202107	7.32 eV	6.35 eV
F4H3N1Zr1_ICSD_50233	4.74 eV	4.90 eV
F4H6N2Si1_ICSD_78902	6.69 eV	6.17 eV
F4H6O1_ICSD_32569	7.37 eV	6.60 eV
F4H7In1N2O1_ICSD_35	4.15 eV	3.72 eV
F4H7N1_ICSD_38337	7.23 eV	6.76 eV
F4H8N2O1V1_ICSD_23317	2.46 eV	2.46 eV
F4H8N2_ICSD_87361	5.30 eV	4.95 eV
F4Hf1_ICSD_66008	5.40 eV	5.67 eV
F4Hg1Rb2_ICSD_72352	1.94 eV	2.25 eV
F4In1Li1_ICSD_66693	4.16 eV	4.02 eV
F4Ir1_ICSD_23483	0.86 eV	1.47 eV
F4K1Mn1_ICSD_72394	2.21 eV	2.31 eV
F4K1Mo1Na1O2_ICSD_422707	1.84 eV	2.82 eV
F4K1O1V1_ICSD_9295	3.60 eV	2.71 eV
F4K1Pr1_ICSD_281289	4.26 eV	4.88 eV
F4K1Sc1_ICSD_72736	6.54 eV	6.66 eV
F4K2Mg1_ICSD_33519	6.42 eV	6.38 eV
F4K2Mn1_ICSD_23183	2.85 eV	2.33 eV
F4K2Ni1_ICSD_15576	3.82 eV	3.38 eV
F4K2O1Te1_ICSD_412955	4.89 eV	4.37 eV
F4K2Pd1_ICSD_33888	3.09 eV	2.74 eV
F4K2Zn1_ICSD_100298	5.56 eV	5.55 eV
F4La1Rb1_ICSD_262425	6.84 eV	6.13 eV
F4Li1Lu1_ICSD_152948	7.73 eV	6.24 eV
F4Li1Mn1_ICSD_62655	1.87 eV	2.08 eV
F4Li1O2Re1_ICSD_280011	3.23 eV	3.08 eV
F4Li1Sc1_ICSD_413966	6.56 eV	6.82 eV
F4Li1Y1_ICSD_27896	7.83 eV	7.02 eV
F4Li1Y1_ICSD_55692	7.32 eV	7.33 eV
F4Li1Yb1_ICSD_9914	1.31 eV	1.66 eV
F4Mg1Rb2_ICSD_69681	6.23 eV	6.14 eV
F4Mg1Sr1_ICSD_86248	6.22 eV	6.72 eV
F4Mn1Na1_ICSD_71456	2.11 eV	2.25 eV
F4Mn1Rb1_ICSD_73587	0.81 eV	1.90 eV
F4N4S4_ICSD_18202	3.46 eV	3.35 eV
F4Na1Sb1_ICSD_24750	4.33 eV	4.50 eV
F4Na1Ti1_ICSD_389	3.07 eV	3.76 eV
F4Na1V1_ICSD_407013	1.61 eV	2.23 eV
F4Na2O2W1_ICSD_201173	4.05 eV	3.91 eV

Supplementary Table 786. Five-fold cross validated predictions for the band gap energy (180/277).

system	calculated	predicted
F4Na2Pd1_ICSD_71101	2.60 eV	2.50 eV
F4Nb1_ICSD_25768	0.71 eV	2.33 eV
F4Ni1Rb2_ICSD_69682	3.87 eV	2.71 eV
F4O1Os1_ICSD_417246	1.40 eV	1.91 eV
F4O1Ru1_ICSD_417249	0.95 eV	1.50 eV
F4O1Tc1_ICSD_16143	1.93 eV	1.66 eV
F4O1U1_ICSD_200058	3.59 eV	3.26 eV
F4O1W1_ICSD_10393	2.79 eV	3.13 eV
F4O2Sn4_ICSD_948	2.67 eV	2.64 eV
F4Pb1Pd1_ICSD_108992	1.92 eV	2.08 eV
F4Pb1Sn1_ICSD_152954	3.56 eV	3.36 eV
F4Pb1_ICSD_78895	1.90 eV	2.88 eV
F4Pd1Sr1_ICSD_108990	2.38 eV	2.56 eV
F4Pd1_ICSD_1555	1.03 eV	1.34 eV
F4Pt1_ICSD_71579	1.56 eV	1.34 eV
F4Ru1_ICSD_165398	1.60 eV	1.28 eV
F4S1W1_ICSD_37245	3.20 eV	3.26 eV
F4Sb1Tl1_ICSD_201084	3.60 eV	4.00 eV
F4Se1_ICSD_85451	4.05 eV	3.71 eV
F4Si1_ICSD_24500	7.70 eV	5.98 eV
F4Sn1_ICSD_16794	2.70 eV	2.81 eV
F4Sr1Zn1_ICSD_16807	5.84 eV	5.82 eV
F4Tc1_ICSD_249918	0.17 eV	1.37 eV
F4Te1_ICSD_9869	3.99 eV	3.86 eV
F4V1_ICSD_65785	1.91 eV	1.50 eV
F4Xe1_ICSD_26626	2.80 eV	2.69 eV
F4Zr1_ICSD_35100	4.90 eV	4.73 eV
F5Fe1H2K2O1_ICSD_16090	1.84 eV	2.40 eV
F5Fe1H4Mn1O2_ICSD_202193	0.54 eV	1.55 eV
F5Fe1H4Mn1O2_ICSD_62362	0.45 eV	1.17 eV
F5Fe1H4O2Zn1_ICSD_202194	2.80 eV	2.40 eV
F5Fe2H4O2_ICSD_201799	0.90 eV	0.88 eV
F5Ga1H4Mn1O2_ICSD_245172	3.51 eV	3.07 eV
F5H2K2Mn1O1_ICSD_26071	1.42 eV	2.14 eV
F5H2K2O1V1_ICSD_59818	1.99 eV	2.22 eV
F5H2Mn1O1Rb2_ICSD_2435	1.36 eV	1.87 eV
F5H2Mn1O1Tl2_ICSD_71517	1.50 eV	1.64 eV
F5H2Mn1O1Tl2_ICSD_71518	1.21 eV	1.71 eV
F5H2N1Te1_ICSD_171449	2.62 eV	3.14 eV
F5H3N1P1_ICSD_36010	2.61 eV	4.34 eV
F5H8N1_ICSD_38338	7.33 eV	7.07 eV
F5H8N2Nb1O2_ICSD_200233	4.96 eV	4.19 eV
F5Hf1Rb1_ICSD_95846	6.80 eV	6.62 eV
F5I1N2O4_ICSD_406388	1.35 eV	2.09 eV
F5I1_ICSD_6021	4.70 eV	2.53 eV
F5K1Li1Y1_ICSD_39668	7.14 eV	6.80 eV
F5K1Na1Nb1O1_ICSD_422708	4.71 eV	3.63 eV
F5K1Te1_ICSD_26051	5.20 eV	4.85 eV
F5K2Pd1Rb1_ICSD_33887	2.94 eV	2.84 eV
F5K2Sb1_ICSD_24742	4.68 eV	4.44 eV

Supplementary Table 787. Five-fold cross validated predictions for the band gap energy (181/277).

system	calculated	predicted
F5K2Sb1_ICSD_39634	4.59 eV	4.47 eV
F5K2Ti1_ICSD_261469	3.17 eV	3.54 eV
F5Li2Mn1_ICSD_202394	1.89 eV	2.25 eV
F5Lu3O2_ICSD_80365	5.70 eV	5.25 eV
F5Mn1Rb2_ICSD_82218	1.32 eV	2.16 eV
F5N3W1_ICSD_201198	3.13 eV	2.98 eV
F5Na1Te1_ICSD_202879	5.25 eV	4.71 eV
F5Na2Sb1_ICSD_28061	4.83 eV	4.53 eV
F5Nb1_ICSD_26647	4.99 eV	3.61 eV
F5O1Os1_ICSD_16872	1.47 eV	1.56 eV
F5O1Os1_ICSD_240332	1.55 eV	1.57 eV
F5O1Sn2_ICSD_409393	1.52 eV	2.48 eV
F5O2S1Sb1_ICSD_67273	3.46 eV	2.96 eV
F5O2Sb3_ICSD_62476	3.81 eV	3.05 eV
F5O4Tc1Xe1_ICSD_280249	2.11 eV	1.95 eV
F5P1_ICSD_62554	7.08 eV	6.28 eV
F5Pd1Rb3_ICSD_35285	2.88 eV	2.65 eV
F5Pd2Rb1_ICSD_72299	0.26 eV	1.40 eV
F5Sb1Sr1_ICSD_68454	4.77 eV	4.46 eV
F5Ta1_ICSD_171155	5.69 eV	4.55 eV
F5Te1Tl1_ICSD_90619	4.46 eV	3.80 eV
F5Tl1Zr1_ICSD_26558	4.79 eV	4.63 eV
F5U1_ICSD_31657	3.99 eV	3.32 eV
F5U1_ICSD_659	2.94 eV	3.57 eV
F6Fe1H12O6Sn1_ICSD_68452	1.24 eV	1.92 eV
F6Fe1H8N2Na1_ICSD_418736	2.19 eV	3.09 eV
F6Fe1K1Rb2_ICSD_9001	2.25 eV	2.87 eV
F6Fe1K2Na1_ICSD_61277	2.18 eV	2.92 eV
F6Fe1Li1Mn1_ICSD_35231	0.57 eV	1.83 eV
F6Fe1Li1Mn1_ICSD_35233	2.26 eV	2.10 eV
F6Fe1Li1Rb2_ICSD_16400	1.15 eV	2.28 eV
F6Fe1Li1Sr1_ICSD_32729	3.83 eV	3.18 eV
F6Fe1Na1Rb2_ICSD_40966	2.14 eV	2.99 eV
F6Fe1Na1Rb2_ICSD_42146	3.80 eV	2.74 eV
F6Fe1Na3_ICSD_20157	3.03 eV	2.42 eV
F6Fe1Nb1_ICSD_63307	0.88 eV	1.76 eV
F6Fe1Nb1_ICSD_63309	1.08 eV	1.82 eV
F6Fe1Zr1_ICSD_35725	3.11 eV	2.64 eV
F6Fe2Li1_ICSD_202705	1.22 eV	1.51 eV
F6Fe2Rb1_ICSD_186747	0.99 eV	2.29 eV
F6Ga1H8N2Na1_ICSD_418737	5.83 eV	5.64 eV
F6Ga1K1Rb2_ICSD_416605	5.96 eV	5.87 eV
F6Ga1Li1Mn1_ICSD_4045	3.90 eV	3.24 eV
F6Ga1Li1Pd1_ICSD_78748	2.85 eV	3.00 eV
F6Ga1Li1Rb2_ICSD_50468	6.16 eV	6.00 eV
F6Ge1H12Mg1O6_ICSD_401096	4.56 eV	4.68 eV
F6Ge1K2_ICSD_24026	5.72 eV	5.76 eV
F6Ge1K2_ICSD_30310	5.84 eV	5.73 eV
F6Ge1Li2_ICSD_23406	5.68 eV	5.98 eV
F6Ge1Li2_ICSD_23407	5.98 eV	5.66 eV

Supplementary Table 788. Five-fold cross validated predictions for the band gap energy (182/277).

system	calculated	predicted
F6Ge1Na2_ICSD_30344	5.57 eV	5.65 eV
F6Ge1Na2_ICSD_69623	5.72 eV	5.69 eV
F6Ge1Rb2_ICSD_25662	5.82 eV	5.62 eV
F6Ge1Rb2_ICSD_26633	5.76 eV	5.80 eV
F6Ge1Rb2_ICSD_68982	5.94 eV	5.88 eV
F6H10O4Si1_ICSD_40388	6.48 eV	6.26 eV
F6H12Mn1O6Ti1_ICSD_202786	2.43 eV	2.66 eV
F6H12Ni1O6Sn1_ICSD_75985	3.02 eV	2.73 eV
F6H12O6Sn2Zn1_ICSD_166589	3.04 eV	3.51 eV
F6H12O6Ti1Zn1_ICSD_62375	4.06 eV	3.88 eV
F6H2N3Sb1_ICSD_41354	0.48 eV	2.84 eV
F6H3O1Sb1_ICSD_66552	4.66 eV	3.93 eV
F6H6N2Si1_ICSD_35702	7.09 eV	6.65 eV
F6H8In1N2Na1_ICSD_79099	5.13 eV	4.48 eV
F6H8N2Sn1_ICSD_409509	4.65 eV	3.89 eV
F6H8N2Ti1_ICSD_24834	5.26 eV	5.03 eV
F6H8N2Ti1_ICSD_24835	5.23 eV	5.20 eV
F6Hf1K2_ICSD_29514	6.46 eV	6.66 eV
F6Hf1K2_ICSD_47243	6.81 eV	6.62 eV
F6Hf1K2_ICSD_47244	6.91 eV	6.04 eV
F6Hf1K2_ICSD_47246	6.83 eV	6.53 eV
F6Hf1Rb2_ICSD_25599	7.10 eV	6.99 eV
F6Hf1V1_ICSD_94455	2.93 eV	2.95 eV
F6Hg2Ni2O1_ICSD_27329	0.85 eV	1.51 eV
F6Hg2Ni2S1_ICSD_4083	1.79 eV	1.82 eV
F6Hg2O1Zn2_ICSD_27330	1.10 eV	1.81 eV
F6Hg3S2Si1_ICSD_23605	2.80 eV	2.17 eV
F6Ho1Na1Rb2_ICSD_86273	6.45 eV	6.50 eV
F6Ho1Na1Rb2_ICSD_86274	6.49 eV	6.38 eV
F6I5Sb1_ICSD_27557	0.93 eV	1.36 eV
F6In1K2Na1_ICSD_22113	5.35 eV	4.50 eV
F6Ir1K2_ICSD_95779	1.24 eV	2.12 eV
F6Ir1Rb2_ICSD_240955	1.31 eV	2.17 eV
F6Ir1_ICSD_171654	1.54 eV	1.51 eV
F6K1Mo1Rb2_ICSD_15773	2.79 eV	2.63 eV
F6K1Mo1Tl2_ICSD_15775	1.59 eV	2.34 eV
F6K1Na1Sn1_ICSD_39590	4.70 eV	4.85 eV
F6K1Nb1_ICSD_16729	5.59 eV	4.07 eV
F6K1Os1_ICSD_27664	2.46 eV	2.40 eV
F6K1P1_ICSD_25576	7.20 eV	6.42 eV
F6K1P1_ICSD_56255	6.97 eV	6.39 eV
F6K1Rb2Rh1_ICSD_27339	3.46 eV	3.08 eV
F6K1Rb2Sc1_ICSD_81679	5.85 eV	6.11 eV
F6K1Rb2Sc1_ICSD_81681	6.44 eV	6.13 eV
F6K1Rb2Sc1_ICSD_81683	6.29 eV	6.18 eV
F6K1Rb2Ti1_ICSD_42142	0.95 eV	1.86 eV
F6K1Rb2V1_ICSD_27345	2.90 eV	2.72 eV
F6K1Rb2Y1_ICSD_73472	5.77 eV	5.79 eV
F6K1Rh1_ICSD_165215	0.87 eV	1.72 eV
F6K1Ru1_ICSD_28692	2.24 eV	2.07 eV

Supplementary Table 789. Five-fold cross validated predictions for the band gap energy (183/277).

system	calculated	predicted
F6K1Sb1_ICSD_42509	4.89 eV	4.90 eV
F6K1Sb1_ICSD_632	4.92 eV	4.83 eV
F6K1Sb1_ICSD_76422	4.93 eV	4.62 eV
F6K2Mn1Na1_ICSD_61117	1.31 eV	2.12 eV
F6K2Mn1_ICSD_47213	3.22 eV	2.67 eV
F6K2Mn1_ICSD_60417	3.17 eV	2.91 eV
F6K2Mo1Na1_ICSD_15777	3.10 eV	2.87 eV
F6K2Na1Ni1_ICSD_42151	3.75 eV	3.52 eV
F6K2Na1Rh1_ICSD_27342	3.27 eV	3.01 eV
F6K2Na1Sc1_ICSD_22112	6.64 eV	6.28 eV
F6K2Na1Tl1_ICSD_22114	3.64 eV	4.23 eV
F6K2Na1V1_ICSD_186883	3.21 eV	2.86 eV
F6K2Na1Y1_ICSD_22115	6.25 eV	6.35 eV
F6K2Ni1_ICSD_6046	2.23 eV	2.90 eV
F6K2O3Ta2_ICSD_15866	4.09 eV	4.14 eV
F6K2Pd1_ICSD_27486	2.54 eV	2.71 eV
F6K2Pt1_ICSD_87360	3.10 eV	2.98 eV
F6K2Re1_ICSD_1528	3.17 eV	2.70 eV
F6K2Rh1_ICSD_28779	1.25 eV	1.98 eV
F6K2Si1_ICSD_158483	7.18 eV	6.76 eV
F6K2Si1_ICSD_38546	7.24 eV	6.91 eV
F6K2Th1_ICSD_31609	6.35 eV	6.10 eV
F6K2Ti1_ICSD_24659	5.23 eV	3.78 eV
F6K2U1_ICSD_31604	3.43 eV	3.53 eV
F6K2U1_ICSD_31610	3.43 eV	3.20 eV
F6K2Zr1_ICSD_14126	5.69 eV	5.75 eV
F6K2Zr1_ICSD_865	5.55 eV	5.64 eV
F6K3Mo1_ICSD_4403	2.54 eV	2.64 eV
F6K3N1O3Si1_ICSD_159390	3.37 eV	4.11 eV
F6K3W1_ICSD_51264	1.23 eV	3.27 eV
F6K3Y1_ICSD_416296	6.48 eV	6.27 eV
F6Li1Mn1Ti1_ICSD_69047	3.19 eV	2.58 eV
F6Li1Mn1V1_ICSD_167073	1.97 eV	1.58 eV
F6Li1Nb1_ICSD_165202	5.48 eV	3.20 eV
F6Li1Ni1Sr1_ICSD_32733	1.97 eV	2.74 eV
F6Li1Os1_ICSD_165214	2.36 eV	2.08 eV
F6Li1P1_ICSD_74830	7.52 eV	7.16 eV
F6Li1Pt1_ICSD_165208	0.23 eV	2.09 eV
F6Li1Ru1_ICSD_165203	2.16 eV	1.79 eV
F6Li1Sb1_ICSD_23924	4.83 eV	4.47 eV
F6Li1Ta1_ICSD_165205	6.35 eV	5.07 eV
F6Li1V2_ICSD_35382	0.13 eV	1.43 eV
F6Li2Mn1_ICSD_15791	2.61 eV	2.41 eV
F6Li2Mo1_ICSD_23173	1.23 eV	1.72 eV
F6Li2Nb1_ICSD_201755	1.07 eV	3.28 eV
F6Li2O3Ta2_ICSD_405777	3.99 eV	4.30 eV
F6Li2Pd1_ICSD_165212	2.34 eV	1.95 eV
F6Li2Pt1_ICSD_165216	2.99 eV	2.09 eV
F6Li2Rh1_ICSD_165211	1.18 eV	1.20 eV
F6Li2Rh1_ICSD_6044	1.19 eV	1.18 eV

Supplementary Table 790. Five-fold cross validated predictions for the band gap energy (184/277).

system	calculated	predicted
F6Li2Ru1_ICSD_165210	0.36 eV	1.22 eV
F6Li2Ti1_ICSD_18313	4.87 eV	4.04 eV
F6Li2Zr1_ICSD_155020	6.46 eV	6.28 eV
F6Li2Zr1_ICSD_2644	6.47 eV	6.20 eV
F6Li2Zr1_ICSD_409667	6.12 eV	6.16 eV
F6Mg1Pb1_ICSD_15106	3.05 eV	3.24 eV
F6Mg1Pd1_ICSD_26163	2.29 eV	2.16 eV
F6Mg1Rh1_ICSD_42159	1.21 eV	1.73 eV
F6Mn1Na1Rb2_ICSD_9708	1.53 eV	2.48 eV
F6Mn1Na3_ICSD_66315	1.31 eV	2.39 eV
F6Mn1Pt1_ICSD_73114	2.28 eV	2.09 eV
F6Mn1Rb2_ICSD_25578	3.23 eV	3.03 eV
F6Mn1Rb2_ICSD_25579	3.23 eV	2.89 eV
F6Mn1Sn1_ICSD_25010	2.12 eV	2.04 eV
F6Mo1Na1Rb2_ICSD_15774	3.33 eV	3.07 eV
F6Mo1Na1Tl2_ICSD_15776	2.07 eV	2.22 eV
F6Mo1_ICSD_171653	4.00 eV	3.11 eV
F6Mo1_ICSD_1879	4.10 eV	2.63 eV
F6N1Re1_ICSD_33543	2.55 eV	2.35 eV
F6N1S2Sb1_ICSD_170871	2.66 eV	2.80 eV
F6N3P3_ICSD_26649	5.68 eV	5.34 eV
F6Na1Ni1Rb2_ICSD_42150	3.79 eV	3.38 eV
F6Na1P1_ICSD_90615	6.98 eV	6.79 eV
F6Na1Rb2Rh1_ICSD_27341	3.23 eV	3.19 eV
F6Na1Rb2Ti1_ICSD_42143	1.62 eV	2.40 eV
F6Na1Rb2V1_ICSD_27346	3.17 eV	2.89 eV
F6Na1Rb2Y1_ICSD_25369	6.46 eV	6.41 eV
F6Na1Sb1_ICSD_25538	4.88 eV	4.69 eV
F6Na1Sb1_ICSD_56251	4.86 eV	4.56 eV
F6Na2Nb1_ICSD_201756	1.06 eV	3.77 eV
F6Na2Si1_ICSD_30348	6.87 eV	6.69 eV
F6Na2Si1_ICSD_40917	7.00 eV	6.98 eV
F6Na2Si1_ICSD_61274	6.98 eV	6.98 eV
F6Na2Sn1_ICSD_68980	4.96 eV	4.66 eV
F6Na2Th1_ICSD_418145	6.48 eV	5.50 eV
F6Na2Ti1_ICSD_24477	5.14 eV	4.47 eV
F6Na2Ti1_ICSD_40916	5.22 eV	4.73 eV
F6Na2U1_ICSD_31605	3.40 eV	3.66 eV
F6Na2U1_ICSD_31623	3.49 eV	3.46 eV
F6Na2U1_ICSD_8034	3.53 eV	3.46 eV
F6Na2Zr1_ICSD_9877	6.25 eV	5.85 eV
F6Na3Nb1O1_ICSD_35761	5.38 eV	4.38 eV
F6Na3Ni1_ICSD_26073	1.58 eV	2.75 eV
F6Na3Sc1_ICSD_401761	6.37 eV	6.35 eV
F6Na3V1_ICSD_27347	2.06 eV	2.66 eV
F6Nb1Rb2_ICSD_72831	1.22 eV	2.69 eV
F6Ni1Pb1_ICSD_15108	1.95 eV	2.35 eV
F6Ni1Pt1_ICSD_78905	2.64 eV	2.28 eV
F6Ni1Rb2_ICSD_29005	2.31 eV	2.37 eV
F6Ni1Rh1_ICSD_42164	1.34 eV	1.53 eV

Supplementary Table 791. Five-fold cross validated predictions for the band gap energy (185/277).

system	calculated	predicted
F6Ni1Sn1_ICSD_25015	3.29 eV	2.59 eV
F6Ni1Sr1_ICSD_30114	1.62 eV	2.67 eV
F6Ni2Rb1_ICSD_31781	0.16 eV	2.44 eV
F6O1W1Xe1_ICSD_1853	3.13 eV	2.79 eV
F6O2P1Sb1_ICSD_51535	4.42 eV	3.60 eV
F6O2Pt1_ICSD_78851	0.19 eV	1.78 eV
F6O2Sb1_ICSD_78849	1.36 eV	2.36 eV
F6O4Pb1Zr3_ICSD_60959	2.85 eV	3.57 eV
F6O9Si2Ti1Y4_ICSD_20003	3.26 eV	4.02 eV
F6Os1_ICSD_171657	0.56 eV	1.67 eV
F6P1Tl1_ICSD_28899	6.38 eV	4.75 eV
F6Pa1Rb1_ICSD_36078	3.97 eV	4.70 eV
F6Pb1Pt1_ICSD_4057	2.39 eV	2.28 eV
F6Pb1Te1_ICSD_81865	4.59 eV	3.94 eV
F6Pb1Zn1_ICSD_15107	2.54 eV	2.74 eV
F6Pb1Zr1_ICSD_4051	5.01 eV	4.72 eV
F6Pb2_ICSD_23467	1.89 eV	3.02 eV
F6Pd1Pt1_ICSD_64661	2.03 eV	2.11 eV
F6Pd1Rb2_ICSD_28675	2.84 eV	2.38 eV
F6Pd1Zn1_ICSD_26165	2.06 eV	2.03 eV
F6Pd1Zr1_ICSD_73133	3.00 eV	3.16 eV
F6Pt1Rb2_ICSD_35108	3.18 eV	2.92 eV
F6Pt1Zn1_ICSD_37444	2.56 eV	2.44 eV
F6Rb1Ru1_ICSD_28693	2.30 eV	2.11 eV
F6Rb1Sb1_ICSD_408071	4.90 eV	4.69 eV
F6Rb2Rh1_ICSD_28780	1.33 eV	1.98 eV
F6Rb2Si1_ICSD_38547	7.03 eV	6.71 eV
F6Rb2Th1_ICSD_25829	6.23 eV	5.77 eV
F6Rb2U1_ICSD_14116	3.50 eV	3.44 eV
F6Rb2Zr1_ICSD_25597	6.65 eV	5.56 eV
F6Rb3Tl1_ICSD_19075	3.16 eV	3.31 eV
F6Rb3Y1_ICSD_19077	5.06 eV	5.93 eV
F6Rh1Zn1_ICSD_42161	1.24 eV	1.37 eV
F6S1_ICSD_214	5.79 eV	6.16 eV
F6S1_ICSD_41229	5.90 eV	5.82 eV
F6S1_ICSD_63334	6.10 eV	5.59 eV
F6S1_ICSD_71013	5.86 eV	5.80 eV
F6Sb1Tl1_ICSD_36264	4.36 eV	3.38 eV
F6Si1Tl2_ICSD_38549	4.96 eV	3.90 eV
F6Sn1Tl2_ICSD_410801	3.93 eV	3.38 eV
F6Sn1Zn1_ICSD_25012	3.85 eV	3.80 eV
F6Sn2_ICSD_33786	2.24 eV	2.74 eV
F6Te1_ICSD_67609	4.37 eV	3.55 eV
F6Ti1Tl2_ICSD_410802	4.38 eV	3.95 eV
F6Ti1Zr1_ICSD_94456	0.84 eV	3.43 eV
F6U1_ICSD_9000	3.85 eV	3.30 eV
F6V1Zr1_ICSD_94454	2.11 eV	2.63 eV
F6W1_ICSD_36220	5.03 eV	3.25 eV
F6Xe2_ICSD_18128	2.52 eV	2.78 eV
F7Fe1Na2Ni1_ICSD_1511	2.75 eV	2.79 eV

Supplementary Table 792. Five-fold cross validated predictions for the band gap energy (186/277).

system	calculated	predicted
F7Fe1Na2Ni1_ICSD_202870	2.76 eV	2.28 eV
F7Ga1Na2Ni1_ICSD_408111	4.19 eV	3.73 eV
F7Gd1Sn1_ICSD_78898	3.94 eV	3.17 eV
F7H1I1N1O1_ICSD_71582	2.38 eV	2.30 eV
F7H1K2Ti1_ICSD_187321	5.23 eV	3.85 eV
F7H1K3Nb1O1_ICSD_26620	4.58 eV	3.82 eV
F7Ho2K1_ICSD_27196	6.91 eV	6.48 eV
F7I1Xe1_ICSD_26059	3.07 eV	2.33 eV
F7I1_ICSD_18191	1.85 eV	3.20 eV
F7In1Sr2_ICSD_38307	5.04 eV	4.66 eV
F7In2K1_ICSD_1307	3.80 eV	3.89 eV
F7K1Sb2_ICSD_14118	4.17 eV	4.59 eV
F7K1Sc2_ICSD_47227	6.21 eV	6.35 eV
F7K1Ti2_ICSD_174209	2.29 eV	3.25 eV
F7K1Yb2_ICSD_16350	0.44 eV	1.12 eV
F7K2Nb1_ICSD_14132	4.94 eV	4.46 eV
F7K2Pa1_ICSD_24144	4.00 eV	4.65 eV
F7K2Ta1_ICSD_260876	5.69 eV	5.59 eV
F7K3Si1_ICSD_23875	5.92 eV	6.33 eV
F7La1Sn1_ICSD_78896	0.14 eV	3.20 eV
F7Li3Th1_ICSD_1726	5.60 eV	5.78 eV
F7Mn1Rb1Zr1_ICSD_78042	4.47 eV	4.30 eV
F7Mn1Rb3_ICSD_9599	1.60 eV	1.69 eV
F7Mn1Tl1Zr1_ICSD_78041	4.49 eV	3.82 eV
F7Na2Ni1V1_ICSD_407015	0.08 eV	2.03 eV
F7Ni1Rb3_ICSD_9601	0.77 eV	2.42 eV
F7Pb1Ta1_ICSD_417253	5.30 eV	4.38 eV
F7Pb2Rh1_ICSD_37141	2.78 eV	2.56 eV
F7Rb3Si1_ICSD_9589	5.65 eV	5.58 eV
F7Rb3Ti1_ICSD_9595	3.50 eV	3.26 eV
F7Re1_ICSD_78311	2.11 eV	1.91 eV
F7Sb1Xe1_ICSD_411868	2.77 eV	2.65 eV
F7Sc1Sr2_ICSD_74360	6.76 eV	6.54 eV
F7Sn1Tl1_ICSD_78899	1.62 eV	2.57 eV
F7Sn1Y1_ICSD_71586	4.18 eV	4.24 eV
F7Sn1Y1_ICSD_74963	4.35 eV	4.24 eV
F7Sr1Ta1_ICSD_417254	5.65 eV	5.09 eV
F8Fe3H4O2_ICSD_37140	0.52 eV	1.67 eV
F8H10In2N2O2_ICSD_97067	4.87 eV	3.83 eV
F8H12O6Zr2_ICSD_1309	5.29 eV	4.61 eV
F8H1K3Sn1_ICSD_26993	4.65 eV	4.52 eV
F8H1Na3Ti1_ICSD_14131	2.21 eV	4.24 eV
F8I2Pb5_ICSD_36334	2.89 eV	2.93 eV
F8K3Ta1_ICSD_248063	3.98 eV	5.19 eV
F8Li1Na1Y2_ICSD_201623	7.30 eV	6.88 eV
F8Li1Na1Yb2_ICSD_202052	1.16 eV	2.08 eV
F8N2O2Xe1_ICSD_23535	2.18 eV	2.53 eV
F8N4P4_ICSD_31125	5.10 eV	5.05 eV
F8Na2U1_ICSD_165293	2.92 eV	3.24 eV
F8Na3Pa1_ICSD_16153	4.22 eV	4.56 eV

Supplementary Table 793. Five-fold cross validated predictions for the band gap energy (187/277).

system	calculated	predicted
F8Na3Ta1_ICSD_260875	5.11 eV	5.30 eV
F8Sn3_ICSD_32592	2.68 eV	2.72 eV
F8Sn3_ICSD_61162	1.34 eV	2.16 eV
F9K1Te2_ICSD_84364	4.51 eV	4.40 eV
F9K1U2_ICSD_16065	3.36 eV	3.29 eV
F9K5Th1_ICSD_14127	5.71 eV	5.43 eV
F9Na1Th2_ICSD_245846	6.68 eV	5.53 eV
F9Nb1Se1_ICSD_9898	5.49 eV	4.29 eV
F9Sb1Xe2_ICSD_90622	1.95 eV	2.03 eV
Fe1Ga2S4_ICSD_100706	0.72 eV	1.15 eV
Fe1Ga2Se4_ICSD_631817	0.80 eV	1.07 eV
Fe1Ga3_ICSD_631760	0.54 eV	0.45 eV
Fe1Ge1Si1_ICSD_150274	0.51 eV	0.52 eV
Fe1Ge2Li1O6_ICSD_162951	2.21 eV	2.32 eV
Fe1Ge2Na1O6_ICSD_180307	2.54 eV	2.17 eV
Fe1Ge3Pr1_ICSD_658628	0.10 eV	0.13 eV
Fe1H10N4O8S2_ICSD_240720	0.94 eV	2.16 eV
Fe1H1Li1O5P1_ICSD_167610	1.33 eV	2.35 eV
Fe1H1O2_ICSD_1544	0.82 eV	1.47 eV
Fe1H1O2_ICSD_167358	1.70 eV	1.59 eV
Fe1H1O2_ICSD_24885	1.63 eV	1.19 eV
Fe1H2K1O6P2_ICSD_423281	2.06 eV	2.39 eV
Fe1H2O5P1_ICSD_167029	1.92 eV	2.14 eV
Fe1H2O5S1_ICSD_71345	3.78 eV	2.37 eV
Fe1H2O5V1_ICSD_87710	2.13 eV	1.90 eV
Fe1H2O6P2Rb1_ICSD_423278	2.06 eV	2.62 eV
Fe1H4K2O10S2_ICSD_87958	3.62 eV	2.96 eV
Fe1H5N1O10P3_ICSD_20782	2.47 eV	2.77 eV
Fe1H6Mg2_ICSD_107500	3.25 eV	2.61 eV
Fe1H6Sr2_ICSD_632211	3.03 eV	2.67 eV
Fe1H8O12Re2_ICSD_402963	0.62 eV	2.14 eV
Fe1I3O9_ICSD_286	2.33 eV	2.29 eV
Fe1I3Tl1_ICSD_26422	2.18 eV	2.02 eV
Fe1I4Rb2_ICSD_8010	2.59 eV	2.00 eV
Fe1In1O3_ICSD_80469	0.96 eV	0.93 eV
Fe1In2S4_ICSD_632381	0.09 eV	0.99 eV
Fe1K1Mo2O8_ICSD_153864	2.10 eV	2.05 eV
Fe1K1Mo2O8_ICSD_28020	2.28 eV	2.31 eV
Fe1K1O2_ICSD_421186	1.31 eV	1.40 eV
Fe1K1O8S2_ICSD_26004	2.01 eV	2.30 eV
Fe1K1S2_ICSD_68383	0.56 eV	0.98 eV
Fe1K2Li3O4_ICSD_33280	2.95 eV	2.31 eV
Fe1K2Na1O3_ICSD_36380	1.99 eV	1.69 eV
Fe1K2O4_ICSD_2876	1.54 eV	1.58 eV
Fe1K2P2S6_ICSD_300226	1.72 eV	2.14 eV
Fe1K2P2S6_ICSD_657803	2.43 eV	1.79 eV
Fe1K2P2Se6_ICSD_165358	1.95 eV	1.57 eV
Fe1K3O2_ICSD_73215	1.29 eV	1.36 eV
Fe1K3Se3_ICSD_89586	0.96 eV	1.41 eV
Fe1La1O3_ICSD_236176	1.09 eV	0.98 eV

Supplementary Table 794. Five-fold cross validated predictions for the band gap energy (188/277).

system	calculated	predicted
Fe1La1O3_ICSD_29118	0.54 eV	1.17 eV
Fe1La3Mn1S7_ICSD_35581	0.75 eV	0.87 eV
Fe1La3Ni1S7_ICSD_603955	0.99 eV	0.88 eV
Fe1La3O6S3W1_ICSD_380402	0.13 eV	1.08 eV
Fe1La3O6_ICSD_421426	2.81 eV	1.68 eV
Fe1La4O1Se6_ICSD_391306	1.52 eV	1.22 eV
Fe1La4S7_ICSD_632416	1.68 eV	1.43 eV
Fe1La5O16Re3_ICSD_96339	0.30 eV	0.95 eV
Fe1Li1Mo2O8_ICSD_241134	2.33 eV	2.29 eV
Fe1Li1O4P1_ICSD_161479	3.64 eV	2.96 eV
Fe1Li1O4P1_ICSD_97766	3.18 eV	3.08 eV
Fe1Li1O4Si1_ICSD_183763	2.83 eV	2.75 eV
Fe1Li1O4Si1_ICSD_186523	2.55 eV	2.62 eV
Fe1Li1O6Si2_ICSD_9669	2.51 eV	2.32 eV
Fe1Li1O7P2_ICSD_95751	2.46 eV	2.74 eV
Fe1Li1O8W2_ICSD_16170	2.26 eV	2.36 eV
Fe1Li1O9P3_ICSD_20610	3.71 eV	3.23 eV
Fe1Li1P1_ICSD_187135	0.23 eV	0.52 eV
Fe1Li2O4Si1_ICSD_161306	2.83 eV	2.66 eV
Fe1Li2O4Si1_ICSD_185581	2.73 eV	2.69 eV
Fe1Li2O4Si1_ICSD_261336	0.51 eV	2.24 eV
Fe1Li4N2_ICSD_68700	0.94 eV	1.43 eV
Fe1Mo1O4_ICSD_43012	1.47 eV	1.99 eV
Fe1Mo1O6Sr2_ICSD_169377	1.50 eV	1.46 eV
Fe1Mo1O6Sr2_ICSD_172212	1.52 eV	1.21 eV
Fe1Mo1O7V1_ICSD_408562	2.15 eV	2.04 eV
Fe1Mo2Na1O8_ICSD_166610	1.97 eV	2.05 eV
Fe1Mo2S4_ICSD_94749	0.67 eV	0.36 eV
Fe1Mo2S4_ICSD_94751	0.16 eV	0.50 eV
Fe1N2Sr2_ICSD_72390	0.44 eV	0.56 eV
Fe1Na1O2_ICSD_27117	1.88 eV	1.68 eV
Fe1Na1O2_ICSD_33775	1.87 eV	1.70 eV
Fe1Na1O4P1_ICSD_56292	3.50 eV	2.97 eV
Fe1Na1O4Ti1_ICSD_36090	1.73 eV	1.66 eV
Fe1Na1O6Si2_ICSD_10221	2.57 eV	2.54 eV
Fe1Na1O8S2_ICSD_166768	1.88 eV	2.28 eV
Fe1Na1O8Se2_ICSD_74001	1.77 eV	1.97 eV
Fe1Na1O8W2_ICSD_161847	2.27 eV	2.11 eV
Fe1Na1S2_ICSD_37026	1.01 eV	1.02 eV
Fe1Na2O4_ICSD_50478	1.05 eV	1.85 eV
Fe1Na3O8P2_ICSD_93790	2.26 eV	2.80 eV
Fe1Na4O3_ICSD_1410	1.09 eV	1.20 eV
Fe1Na4O4_ICSD_89702	0.48 eV	1.60 eV
Fe1Na6S4_ICSD_72303	1.87 eV	2.06 eV
Fe1Na6Se4_ICSD_72304	1.62 eV	1.52 eV
Fe1Nb1O4_ICSD_14016	1.78 eV	1.75 eV
Fe1Nb1O4_ICSD_429	1.78 eV	1.79 eV
Fe1Nb1O4_ICSD_8128	2.20 eV	2.03 eV
Fe1Nb1Sb1_ICSD_83928	0.75 eV	0.75 eV
Fe1Nb2O6_ICSD_31943	2.10 eV	2.15 eV

Supplementary Table 795. Five-fold cross validated predictions for the band gap energy (189/277).

system	calculated	predicted
Fe1O13Te6_ICSD_417293	2.11 eV	2.12 eV
Fe1O2Rb1_ICSD_421188	1.68 eV	1.58 eV
Fe1O2Tl1_ICSD_29011	0.89 eV	1.08 eV
Fe1O3Si1_ICSD_75929	2.40 eV	2.60 eV
Fe1O3Ti1_ICSD_246515	1.87 eV	1.87 eV
Fe1O3Ti1_ICSD_246516	1.58 eV	1.50 eV
Fe1O3Ti1_ICSD_30665	1.98 eV	2.03 eV
Fe1O3Zr1_ICSD_247122	2.50 eV	1.75 eV
Fe1O4P1_ICSD_155065	1.42 eV	2.20 eV
Fe1O4P1_ICSD_281079	1.85 eV	2.17 eV
Fe1O4P1_ICSD_290337	2.03 eV	2.13 eV
Fe1O4P1_ICSD_38062	2.51 eV	2.16 eV
Fe1O4P1_ICSD_412735	2.38 eV	2.20 eV
Fe1O4S1_ICSD_23507	3.51 eV	2.86 eV
Fe1O4S1_ICSD_240947	3.69 eV	3.01 eV
Fe1O4Sb2_ICSD_155152	1.53 eV	1.40 eV
Fe1O4Sb2_ICSD_4459	1.17 eV	1.52 eV
Fe1O4Si1_ICSD_186521	2.23 eV	2.49 eV
Fe1O4U1_ICSD_15709	1.45 eV	1.76 eV
Fe1O4V1_ICSD_82161	1.62 eV	1.75 eV
Fe1O4W1_ICSD_26811	1.88 eV	2.12 eV
Fe1O5Ti2_ICSD_37231	1.76 eV	1.68 eV
Fe1O6Sr2W1_ICSD_78678	2.28 eV	2.07 eV
Fe1O6Ta2_ICSD_201754	2.26 eV	2.31 eV
Fe1O7Rb1Se2_ICSD_90415	2.02 eV	2.37 eV
Fe1O8Rb1Se2_ICSD_75550	2.06 eV	1.79 eV
Fe1P1S3_ICSD_61392	1.54 eV	0.67 eV
Fe1P2_ICSD_42904	0.91 eV	0.71 eV
Fe1P4Si4_ICSD_79005	1.17 eV	1.14 eV
Fe1P4_ICSD_2413	1.23 eV	1.01 eV
Fe1P4_ICSD_2442	1.19 eV	1.11 eV
Fe1P4_ICSD_65415	1.03 eV	1.08 eV
Fe1Rb1S2_ICSD_633207	0.51 eV	0.98 eV
Fe1Rb1Se2_ICSD_40781	0.53 eV	0.91 eV
Fe1Rh2Se4_ICSD_603354	0.18 eV	0.48 eV
Fe1S17U8_ICSD_633354	1.13 eV	0.71 eV
Fe1S1Sb1_ICSD_24161	0.67 eV	0.63 eV
Fe1S1_ICSD_35006	1.00 eV	0.86 eV
Fe1S1_ICSD_43694	0.78 eV	0.93 eV
Fe1S1_ICSD_633265	0.98 eV	0.61 eV
Fe1S1_ICSD_68845	0.76 eV	0.93 eV
Fe1S1_ICSD_87501	1.19 eV	0.81 eV
Fe1S1_ICSD_87503	0.41 eV	0.90 eV
Fe1S2Tl1_ICSD_63381	0.50 eV	0.74 eV
Fe1S2_ICSD_109374	1.74 eV	1.35 eV
Fe1S2_ICSD_633289	2.01 eV	1.53 eV
Fe1S4Sb2_ICSD_93911	0.90 eV	1.07 eV
Fe1S4V2_ICSD_35634	0.88 eV	0.42 eV
Fe1S5Th2_ICSD_633325	1.58 eV	1.08 eV
Fe1S5U2_ICSD_41899	0.55 eV	0.92 eV

Supplementary Table 796. Five-fold cross validated predictions for the band gap energy (190/277).

system	calculated	predicted
Fe1S6Ti3_ICSD_53533	0.64 eV	0.59 eV
Fe1Sb1V1_ICSD_53539	0.84 eV	0.63 eV
Fe1Sb2_ICSD_186635	0.57 eV	0.41 eV
Fe1Se0.875_ICSD_167196	0.18 eV	0.56 eV
Fe1Se1_ICSD_165957	0.49 eV	0.59 eV
Fe1Se1_ICSD_185465	0.51 eV	0.55 eV
Fe1Se1_ICSD_53542	0.56 eV	0.57 eV
Fe1Se2_ICSD_633475	1.50 eV	1.09 eV
Fe1Se2_ICSD_633489	1.16 eV	0.84 eV
Fe1Se4Ti2_ICSD_633504	0.86 eV	0.47 eV
Fe1Se5Th2_ICSD_633494	1.21 eV	0.97 eV
Fe1Si1_ICSD_81495	0.87 eV	0.66 eV
Fe1Si2_ICSD_23408	0.97 eV	0.84 eV
Fe1Te2_ICSD_633866	0.70 eV	0.57 eV
Fe2Ga1V1_ICSD_631850	0.70 eV	0.66 eV
Fe2Ga2S5_ICSD_631804	0.66 eV	0.79 eV
Fe2Ge1O4_ICSD_93973	1.04 eV	1.25 eV
Fe2Ge1S4_ICSD_23526	1.40 eV	1.18 eV
Fe2Ge1Se4_ICSD_87086	0.80 eV	0.70 eV
Fe2Ge1Ti1_ICSD_186057	1.25 eV	0.62 eV
Fe2Ge3Mn3O12_ICSD_18111	1.90 eV	1.41 eV
Fe2H1Na1O8P2_ICSD_171329	2.90 eV	2.67 eV
Fe2H1O9Sb1Si2_ICSD_200068	1.97 eV	2.09 eV
Fe2K1S2_ICSD_186572	0.14 eV	0.66 eV
Fe2K1S3_ICSD_99507	0.32 eV	0.50 eV
Fe2K2Na4O6_ICSD_36612	1.98 eV	1.65 eV
Fe2K2Nb4O13_ICSD_50038	1.48 eV	1.52 eV
Fe2K3Na1O8_ICSD_159909	1.39 eV	1.84 eV
Fe2K4O5_ICSD_154372	2.00 eV	1.69 eV
Fe2K6O5_ICSD_174312	1.27 eV	1.37 eV
Fe2K6O6_ICSD_6149	1.80 eV	1.75 eV
Fe2K6S6_ICSD_425068	1.25 eV	1.56 eV
Fe2La2S5_ICSD_1478	0.20 eV	0.42 eV
Fe2La3S7_ICSD_603953	0.80 eV	0.81 eV
Fe2Li2O4_ICSD_31149	0.96 eV	1.24 eV
Fe2Mg1O4_ICSD_157694	0.71 eV	1.30 eV
Fe2Mg1O4_ICSD_24493	1.45 eV	1.28 eV
Fe2Mg1O4_ICSD_94080	0.74 eV	1.18 eV
Fe2Mg3O12Si3_ICSD_27373	2.46 eV	2.34 eV
Fe2Mn1O4_ICSD_170910	0.79 eV	0.83 eV
Fe2Mn1O4_ICSD_24497	0.78 eV	0.79 eV
Fe2Mo3Na3O12_ICSD_281400	0.62 eV	1.60 eV
Fe2Mo3O8_ICSD_61069	1.62 eV	1.45 eV
Fe2Na2O1Se2_ICSD_186502	1.34 eV	1.03 eV
Fe2O12P4_ICSD_33740	3.51 eV	3.09 eV
Fe2O12S3_ICSD_21018	2.13 eV	2.36 eV
Fe2O12Se4Sr1_ICSD_81569	2.15 eV	2.33 eV
Fe2O14P4Pb1_ICSD_83648	2.35 eV	2.02 eV
Fe2O14P4Sr1_ICSD_79719	1.33 eV	2.47 eV
Fe2O3_ICSD_161287	1.28 eV	0.88 eV

Supplementary Table 797. Five-fold cross validated predictions for the band gap energy (191/277).

system	calculated	predicted
Fe2O3_ICSD_189439	1.31 eV	0.77 eV
Fe2O3_ICSD_96076	0.26 eV	0.96 eV
Fe2O4Si1_ICSD_158572	2.39 eV	2.36 eV
Fe2O4Si1_ICSD_169083	2.37 eV	2.42 eV
Fe2O4Si1_ICSD_185523	2.90 eV	2.37 eV
Fe2O4Si1_ICSD_87462	2.40 eV	2.40 eV
Fe2O5P1_ICSD_32641	1.29 eV	2.24 eV
Fe2O5Rb4_ICSD_154291	1.71 eV	1.55 eV
Fe2O5Te1_ICSD_8282	1.66 eV	1.76 eV
Fe2O5Ti1_ICSD_24416	1.64 eV	1.67 eV
Fe2O5Ti1_ICSD_36183	1.68 eV	1.63 eV
Fe2O6Rb6_ICSD_154371	1.81 eV	1.66 eV
Fe2O6Te1_ICSD_24795	1.12 eV	1.41 eV
Fe2O6W1_ICSD_1147	1.58 eV	1.92 eV
Fe2O7P2_ICSD_17062	3.38 eV	2.41 eV
Fe2O7P2_ICSD_240243	3.40 eV	2.51 eV
Fe2O7P2_ICSD_36208	0.13 eV	1.78 eV
Fe2O7Sr1Tb2_ICSD_19038	0.85 eV	1.08 eV
Fe2P2S6_ICSD_657319	0.09 eV	1.17 eV
Fe2Rb1S3_ICSD_99506	0.25 eV	0.47 eV
Fe2Rb6S6_ICSD_425088	1.31 eV	1.32 eV
Fe2S4Si1_ICSD_88517	1.53 eV	1.26 eV
Fe2Sn1Ti1_ICSD_186058	1.08 eV	0.63 eV
Fe3H28O30S4_ICSD_15207	0.37 eV	2.30 eV
Fe3H6K1O14S2_ICSD_189302	0.82 eV	1.68 eV
Fe3H6Na1O14S2_ICSD_160409	1.62 eV	1.73 eV
Fe3H6O14P1Pb1S1_ICSD_68352	0.59 eV	2.00 eV
Fe3O12Si3V2_ICSD_27376	1.50 eV	2.14 eV
Fe3O4_ICSD_263010	0.16 eV	0.46 eV
Fe3O4_ICSD_35002	0.78 eV	0.59 eV
Fe3O4_ICSD_65338	0.76 eV	0.68 eV
Fe3O6Rb5_ICSD_174319	1.66 eV	1.51 eV
Fe3O7P1_ICSD_36207	1.93 eV	1.71 eV
Fe3O8P2_ICSD_56293	3.20 eV	2.39 eV
Fe4O5_ICSD_185515	0.19 eV	0.22 eV
Fe4P12Th1_ICSD_200827	0.10 eV	0.43 eV
Fe4Rb8S10_ICSD_425071	0.93 eV	1.41 eV
Fe5H1O8_ICSD_166135	0.56 eV	1.39 eV
Fe5Nd3O12_ICSD_260556	1.74 eV	0.85 eV
Fe5O12Pr3_ICSD_422690	0.10 eV	1.09 eV
Fe5O12Si3_ICSD_27377	1.92 eV	2.29 eV
Fe5O12Tb3_ICSD_22320	1.68 eV	1.14 eV
Fe5O12Y3_ICSD_88504	0.09 eV	1.11 eV
Fe8Rb24S24_ICSD_425089	1.34 eV	1.34 eV
Ga0.5N0.5O0.5Zn0.5_ICSD_160638	0.85 eV	1.74 eV
Ga1Gd3O6_ICSD_420733	3.51 eV	3.49 eV
Ga1Ge1H1Sr1_ICSD_173570	0.45 eV	0.55 eV
Ga1Ge1Li3O5_ICSD_72101	1.49 eV	2.52 eV
Ga1Ge1N1Sr2_ICSD_420413	0.11 eV	0.49 eV
Ga1H1O2_ICSD_409671	2.96 eV	3.32 eV

Supplementary Table 798. Five-fold cross validated predictions for the band gap energy (192/277).

system	calculated	predicted
Ga1H1O4Se1_ICSD_422882	3.95 eV	3.69 eV
Ga1H1Si1Sr1_ICSD_173569	0.44 eV	0.63 eV
Ga1H1Sn1Sr1_ICSD_173571	0.36 eV	0.36 eV
Ga1H2Na3O9P2_ICSD_84644	4.04 eV	4.31 eV
Ga1H4K1_ICSD_169709	4.98 eV	4.93 eV
Ga1H4Li1_ICSD_169706	4.46 eV	4.37 eV
Ga1H4Li1_ICSD_169707	4.61 eV	4.07 eV
Ga1H4Na1_ICSD_67309	4.69 eV	3.78 eV
Ga1H4Rb1_ICSD_169710	4.92 eV	4.92 eV
Ga1H5Sr1_ICSD_240697	3.38 eV	3.56 eV
Ga1Ho1O3_ICSD_420711	3.77 eV	3.64 eV
Ga1Ho3O6_ICSD_99496	3.60 eV	3.65 eV
Ga1I3Li1_ICSD_202642	2.26 eV	2.59 eV
Ga1I3O9_ICSD_171008	3.30 eV	2.98 eV
Ga1I3_ICSD_413457	2.31 eV	2.43 eV
Ga1I4K1_ICSD_400816	2.59 eV	2.56 eV
Ga1I4Tl1_ICSD_419824	2.56 eV	2.32 eV
Ga1I7Te1_ICSD_407982	1.85 eV	1.87 eV
Ga1In1O3_ICSD_30339	1.50 eV	1.54 eV
Ga1In1O5Zn2_ICSD_380305	1.89 eV	1.72 eV
Ga1In1O7Zn4_ICSD_247275	1.76 eV	1.76 eV
Ga1In1S3_ICSD_62929	2.15 eV	1.88 eV
Ga1In1Se3_ICSD_62930	1.37 eV	1.43 eV
Ga1K1O14Te6_ICSD_280793	2.98 eV	2.85 eV
Ga1K1S2_ICSD_107928	2.58 eV	2.48 eV
Ga1K1Sb4_ICSD_300158	0.28 eV	0.29 eV
Ga1K2Li3O4_ICSD_35290	3.49 eV	3.15 eV
Ga1K2Na1P2_ICSD_300112	1.35 eV	1.24 eV
Ga1K6Na3Sb4_ICSD_77274	0.69 eV	0.68 eV
Ga1La1O3_ICSD_161782	3.37 eV	3.38 eV
Ga1La1O3_ICSD_51039	3.39 eV	3.34 eV
Ga1La1O3_ICSD_83349	3.40 eV	3.43 eV
Ga1La2N3_ICSD_160089	1.47 eV	1.40 eV
Ga1La3Mn1S7_ICSD_634493	2.02 eV	1.64 eV
Ga1Li1Mo2O8_ICSD_241135	3.46 eV	2.95 eV
Ga1Li1O2_ICSD_18152	3.31 eV	3.49 eV
Ga1Li1O2_ICSD_28388	3.94 eV	3.27 eV
Ga1Li1O6Si2_ICSD_80108	4.40 eV	4.40 eV
Ga1Li1O6Si2_ICSD_80924	4.38 eV	4.42 eV
Ga1Li1O8W2_ICSD_28008	3.18 eV	3.17 eV
Ga1Li1S2_ICSD_96914	3.03 eV	2.66 eV
Ga1Li1Se2_ICSD_634546	2.12 eV	2.03 eV
Ga1Li1Si1_ICSD_103786	0.19 eV	0.47 eV
Ga1Li1Te2_ICSD_162555	1.63 eV	1.15 eV
Ga1Li3N2_ICSD_419137	2.48 eV	2.61 eV
Ga1Li3Na2O4_ICSD_37071	3.30 eV	3.40 eV
Ga1Li3O5Si1_ICSD_72100	2.58 eV	3.44 eV
Ga1Li5O4_ICSD_9082	3.84 eV	3.82 eV
Ga1Mn1Ti2_ICSD_189697	0.18 eV	0.30 eV
Ga1Mo4S8_ICSD_106732	0.53 eV	0.71 eV

Supplementary Table 799. Five-fold cross validated predictions for the band gap energy (193/277).

system	calculated	predicted
Ga1Mo4Se8_ICSD_36563	0.60 eV	0.77 eV
Ga1Mo4Se8_ICSD_634710	0.91 eV	0.72 eV
Ga1N1_ICSD_159250	1.07 eV	1.59 eV
Ga1N1_ICSD_181358	1.93 eV	1.53 eV
Ga1N1_ICSD_185155	1.75 eV	0.89 eV
Ga1N1_ICSD_41500	0.63 eV	1.46 eV
Ga1N3Sr3_ICSD_281259	0.60 eV	0.96 eV
Ga1N5O14_ICSD_280268	1.14 eV	2.08 eV
Ga1N5Sr6_ICSD_281260	0.30 eV	0.77 eV
Ga1Na1O2_ICSD_36652	2.93 eV	2.93 eV
Ga1Na1O6Si2_ICSD_156699	4.26 eV	4.24 eV
Ga1Na1Te2_ICSD_44702	0.24 eV	0.77 eV
Ga1Na3P4Sr3_ICSD_402285	0.76 eV	0.87 eV
Ga1Nb1O4_ICSD_18187	3.68 eV	3.03 eV
Ga1Nd1O3_ICSD_24856	3.52 eV	3.09 eV
Ga1Nd3O6_ICSD_99490	2.95 eV	3.10 eV
Ga1O2Tl1_ICSD_33579	2.14 eV	1.80 eV
Ga1O3Pr1_ICSD_76049	0.63 eV	2.01 eV
Ga1O3Y1_ICSD_1999	2.95 eV	3.40 eV
Ga1O4P1_ICSD_155446	4.52 eV	4.34 eV
Ga1O4P1_ICSD_30883	4.52 eV	4.50 eV
Ga1O4Rb5_ICSD_420328	1.86 eV	2.09 eV
Ga1O6Sb1Sr2_ICSD_157014	1.31 eV	1.36 eV
Ga1O6Sb1Sr2_ICSD_157017	0.79 eV	1.47 eV
Ga1O6Tb3_ICSD_99494	3.64 eV	3.59 eV
Ga1O6Y3_ICSD_155086	3.71 eV	3.49 eV
Ga1P1S4_ICSD_2613	2.27 eV	2.26 eV
Ga1P1_ICSD_67772	1.35 eV	1.33 eV
Ga1P1_ICSD_77087	1.64 eV	1.19 eV
Ga1S1_ICSD_40824	1.49 eV	1.82 eV
Ga1S1_ICSD_635244	1.68 eV	1.52 eV
Ga1S2Tl1_ICSD_180614	1.77 eV	1.57 eV
Ga1Se1_ICSD_2002	0.95 eV	0.88 eV
Ga1Se1_ICSD_635382	1.06 eV	0.89 eV
Ga1Se1_ICSD_660262	0.79 eV	0.94 eV
Ga1Se1_ICSD_73388	0.74 eV	0.81 eV
Ga1Se2Tl1_ICSD_100131	0.54 eV	0.83 eV
Ga1Se2Tl1_ICSD_1573	1.29 eV	1.15 eV
Ga1Se2Tl1_ICSD_157752	1.26 eV	1.30 eV
Ga1Te1_ICSD_43328	0.27 eV	0.63 eV
Ga1Te2Tl1_ICSD_635523	0.50 eV	0.65 eV
Ga2Ge1La2S8_ICSD_262241	1.72 eV	2.03 eV
Ga2Ge4O14Pb3_ICSD_250123	2.21 eV	2.51 eV
Ga2H3O9P3_ICSD_74528	5.28 eV	4.36 eV
Ga2H6O12Te3_ICSD_240957	3.75 eV	3.35 eV
Ga2Hg1S4_ICSD_189739	1.61 eV	1.38 eV
Ga2Hg1Se4_ICSD_188545	0.95 eV	1.01 eV
Ga2Hg1Te4_ICSD_53575	0.69 eV	0.70 eV
Ga2I3_ICSD_24822	2.21 eV	2.33 eV
Ga2I8Pd1_ICSD_413230	1.38 eV	1.54 eV

Supplementary Table 800. Five-fold cross validated predictions for the band gap energy (194/277).

system	calculated	predicted
Ga2In6O8Pt1_ICSD_411505	0.62 eV	1.04 eV
Ga2K2Na4O6_ICSD_62136	2.56 eV	2.59 eV
Ga2K6O6_ICSD_2269	2.66 eV	2.58 eV
Ga2K6Se6_ICSD_300170	2.12 eV	1.94 eV
Ga2K6Te6_ICSD_300171	1.56 eV	1.71 eV
Ga2La2O1S6Zn1_ICSD_61044	2.58 eV	2.31 eV
Ga2Mg1O4_ICSD_86507	3.29 eV	3.18 eV
Ga2Mg1S4_ICSD_15350	2.02 eV	2.21 eV
Ga2Mn1S4_ICSD_634668	2.31 eV	1.54 eV
Ga2Mn1Se4_ICSD_634676	1.49 eV	1.04 eV
Ga2Mn1Se4_ICSD_659100	0.74 eV	1.13 eV
Ga2Mn2Pr6S14_ICSD_634657	1.74 eV	1.56 eV
Ga2Mn2S5_ICSD_634669	0.24 eV	0.97 eV
Ga2N4Sr3_ICSD_170441	1.63 eV	1.37 eV
Ga2Ni1S4_ICSD_634901	0.98 eV	1.15 eV
Ga2O11Te4_ICSD_94349	3.49 eV	3.17 eV
Ga2O3_ICSD_162252	1.37 eV	1.95 eV
Ga2O3_ICSD_184327	2.14 eV	2.14 eV
Ga2O3_ICSD_635016	2.65 eV	2.29 eV
Ga2O4Pb1_ICSD_33533	3.48 eV	2.64 eV
Ga2O4Pb1_ICSD_80129	3.51 eV	2.78 eV
Ga2O4Sr1_ICSD_15539	3.15 eV	3.00 eV
Ga2O4Zn1_ICSD_81107	2.85 eV	2.59 eV
Ga2O6Rb6_ICSD_2270	2.44 eV	2.31 eV
Ga2O6Te1_ICSD_78346	0.83 eV	2.25 eV
Ga2O9Te3_ICSD_261175	3.76 eV	3.18 eV
Ga2Os1_ICSD_103785	0.72 eV	0.59 eV
Ga2Pb1Se4_ICSD_635052	1.76 eV	1.31 eV
Ga2Ru1_ICSD_635228	0.14 eV	0.29 eV
Ga2S2Te1_ICSD_8028	2.06 eV	1.89 eV
Ga2S3_ICSD_409550	1.75 eV	1.95 eV
Ga2S3_ICSD_488	1.98 eV	2.16 eV
Ga2S4Sr1_ICSD_635274	2.72 eV	2.51 eV
Ga2S4Zn1_ICSD_635300	2.07 eV	2.27 eV
Ga2S4Zn1_ICSD_635305	2.44 eV	2.15 eV
Ga2S5Sn2_ICSD_37173	1.67 eV	1.98 eV
Ga2Se2Te1_ICSD_64617	1.69 eV	1.21 eV
Ga2Se3_ICSD_35028	1.06 eV	1.42 eV
Ga2Se4Sr1_ICSD_635401	1.82 eV	1.69 eV
Ga2Se4Yb1_ICSD_635414	1.53 eV	1.39 eV
Ga2Se4Zn1_ICSD_168594	1.55 eV	1.43 eV
Ga2Sr1Te4_ICSD_41166	0.40 eV	0.91 eV
Ga2Te4Zn1_ICSD_44888	1.11 eV	1.09 eV
Ga2Te5_ICSD_1085	0.89 eV	0.64 eV
Ga3Gd1Ru1_ICSD_634241	0.67 eV	0.33 eV
Ga3I1Te3_ICSD_61678	1.16 eV	1.12 eV
Ga3I1Te3_ICSD_66031	1.17 eV	1.13 eV
Ga3K1_ICSD_20664	0.20 eV	0.32 eV
Ga3N5Sr3_ICSD_170443	1.44 eV	1.41 eV
Ga3Na3O12Te2_ICSD_418645	1.90 eV	2.51 eV

Supplementary Table 801. Five-fold cross validated predictions for the band gap energy (195/277).

system	calculated	predicted
Ga3O14Si2Sr3Ta1_ICSD_421591	3.96 eV	3.40 eV
Ga3O7P1_ICSD_59364	3.26 eV	3.32 eV
Ga3Os1_ICSD_635024	0.65 eV	0.71 eV
Ga3Rb1S5_ICSD_419822	2.60 eV	2.63 eV
Ga3Rb1_ICSD_103943	0.18 eV	0.23 eV
Ga3Ru1_ICSD_412078	0.31 eV	0.30 eV
Ga4Ge1O8_ICSD_202044	2.15 eV	2.26 eV
Ga4O7Sr1_ICSD_10316	2.51 eV	2.77 eV
Ga4O8Ti1_ICSD_155638	2.73 eV	2.57 eV
Ga4O9Sr3_ICSD_51546	2.95 eV	3.07 eV
Ga5Gd3O12_ICSD_184931	3.25 eV	3.42 eV
Ga5H1O8_ICSD_236277	2.29 eV	2.82 eV
Ga5Ho3O12_ICSD_409390	3.43 eV	3.35 eV
Ga5La3O14Sn1_ICSD_412328	3.14 eV	2.92 eV
Ga5Li1O8_ICSD_33716	2.70 eV	2.82 eV
Ga5Na1O12Ti2_ICSD_108816	2.62 eV	2.80 eV
Ga5Nd3O12_ICSD_84872	3.04 eV	3.04 eV
Ga5O12Tb3_ICSD_184934	3.39 eV	3.40 eV
Ga5O12Y3_ICSD_80148	3.46 eV	3.32 eV
Ga5O12Yb3_ICSD_23851	0.52 eV	1.06 eV
Ga6I2Li8O24Si6_ICSD_87989	3.50 eV	3.57 eV
Ga7Te10_ICSD_400668	0.88 eV	0.98 eV
Ga9O2S13Tl3_ICSD_61256	2.24 eV	1.98 eV
Gd1Ge1K1S4_ICSD_603183	2.26 eV	2.27 eV
Gd1Ge1Na1O4_ICSD_85496	3.48 eV	3.32 eV
Gd1H3O3_ICSD_200093	3.58 eV	3.58 eV
Gd1I1S1_ICSD_416462	2.52 eV	2.01 eV
Gd1K1Na1Nb1O5_ICSD_420037	3.40 eV	3.48 eV
Gd1K1Na1O5Ta1_ICSD_419854	3.48 eV	3.28 eV
Gd1K1O12P4_ICSD_171710	5.28 eV	4.72 eV
Gd1K1O3Pd1_ICSD_417105	1.32 eV	1.47 eV
Gd1K1O8W2_ICSD_68249	3.05 eV	3.39 eV
Gd1K1S2_ICSD_44945	2.12 eV	2.23 eV
Gd1K1Te2_ICSD_98657	1.11 eV	1.23 eV
Gd1K2O8Rb1V2_ICSD_39370	3.36 eV	3.36 eV
Gd1K3O8P2_ICSD_260894	4.92 eV	4.66 eV
Gd1Li1O12P4_ICSD_416442	5.91 eV	5.27 eV
Gd1Li1O2_ICSD_27769	1.71 eV	2.86 eV
Gd1Li1O2_ICSD_422561	4.28 eV	2.85 eV
Gd1Li1Se2_ICSD_44961	1.22 eV	1.38 eV
Gd1Na1O2_ICSD_97542	3.64 eV	3.67 eV
Gd1Na1O4Si1_ICSD_16188	4.20 eV	3.94 eV
Gd1Na1O4Ti1_ICSD_81537	3.07 eV	3.23 eV
Gd1Na1O4Ti1_ICSD_82006	2.99 eV	3.07 eV
Gd1Na1S2_ICSD_37332	2.08 eV	2.15 eV
Gd1Na1Se2_ICSD_636002	1.67 eV	1.63 eV
Gd1Ni1Sb1_ICSD_636074	0.14 eV	0.17 eV
Gd1O1P1Zn1_ICSD_418524	1.32 eV	1.27 eV
Gd1O2Rb1_ICSD_27335	3.36 eV	3.15 eV
Gd1O3Sc1_ICSD_65513	4.28 eV	3.77 eV

Supplementary Table 802. Five-fold cross validated predictions for the band gap energy (196/277).

system	calculated	predicted
Gd1O4P1_ICSD_184554	5.57 eV	4.70 eV
Gd1O4P1_ICSD_184560	2.46 eV	3.71 eV
Gd1O4P1_ICSD_79753	2.01 eV	3.31 eV
Gd1O4Ta1_ICSD_415433	4.12 eV	3.89 eV
Gd1O4V1_ICSD_81703	2.94 eV	2.82 eV
Gd1O6Ru1Sr2_ICSD_183381	1.18 eV	1.21 eV
Gd1O8Rb1W2_ICSD_152962	3.54 eV	3.43 eV
Gd1O9P3_ICSD_155301	5.43 eV	5.11 eV
Gd1Rb1S2_ICSD_81400	2.12 eV	2.17 eV
Gd1Rb1Se2_ICSD_281072	1.73 eV	1.65 eV
Gd1S2Tl1_ICSD_57323	1.52 eV	1.39 eV
Gd1S3Sc1_ICSD_173844	1.92 eV	1.83 eV
Gd1Se2Tl1_ICSD_106989	1.04 eV	0.84 eV
Gd1Se2Tl1_ICSD_636407	0.14 eV	0.80 eV
Gd2Ge1O5_ICSD_61372	3.57 eV	3.63 eV
Gd2Hf2O7_ICSD_236213	3.45 eV	3.39 eV
Gd2O1S2_ICSD_409708	2.27 eV	2.05 eV
Gd2O1Se2_ICSD_280201	1.53 eV	1.33 eV
Gd2O2S1_ICSD_636114	2.76 eV	2.56 eV
Gd2O2Se1_ICSD_25808	1.99 eV	1.79 eV
Gd2O2Te1_ICSD_89563	1.10 eV	1.28 eV
Gd2O3_ICSD_160211	3.80 eV	3.79 eV
Gd2O3_ICSD_162247	3.81 eV	3.53 eV
Gd2O3_ICSD_184528	3.59 eV	3.67 eV
Gd2O3_ICSD_40473	3.62 eV	3.66 eV
Gd2O4Si1Te1_ICSD_409569	1.75 eV	2.44 eV
Gd2O4Sr1_ICSD_96232	3.41 eV	3.13 eV
Gd2O5Si1_ICSD_27728	4.64 eV	4.37 eV
Gd2O6Te1_ICSD_412447	2.95 eV	2.99 eV
Gd2O6W1_ICSD_62887	2.63 eV	3.16 eV
Gd2O6W1_ICSD_62888	3.44 eV	3.06 eV
Gd2O7Sc2Sr1_ICSD_167052	3.44 eV	2.88 eV
Gd2O7Si2_ICSD_20316	4.93 eV	4.13 eV
Gd2O7Sn2_ICSD_84753	2.15 eV	2.24 eV
Gd2O7Ti2_ICSD_164024	3.27 eV	3.08 eV
Gd2O7Zr2_ICSD_188035	2.85 eV	3.28 eV
Gd2S3_ICSD_33785	0.42 eV	0.66 eV
Gd2S5Zr1_ICSD_425080	1.00 eV	1.18 eV
Gd2Se3_ICSD_636396	0.18 eV	0.40 eV
Gd3In1Se6_ICSD_280242	0.48 eV	0.57 eV
Gd3N1S3_ICSD_416220	1.17 eV	1.25 eV
Gd3Nb1O7_ICSD_174285	2.98 eV	3.13 eV
Gd3O12Sb5_ICSD_67024	3.10 eV	2.21 eV
Gd3O3Sb1_ICSD_380460	0.25 eV	0.59 eV
Gd3O7Re1_ICSD_99252	0.35 eV	0.77 eV
Gd3O7Ru1_ICSD_92067	1.08 eV	1.11 eV
Gd4O4Se4Ti1_ICSD_93873	0.84 eV	1.24 eV
Ge136_ICSD_245948	0.67 eV	0.57 eV
Ge1H2O4Sr1_ICSD_30320	4.08 eV	3.91 eV
Ge1H3K3O2S3_ICSD_418259	2.96 eV	3.08 eV

Supplementary Table 803. Five-fold cross validated predictions for the band gap energy (197/277).

system	calculated	predicted
Ge1H4_ICSD_183084	1.22 eV	3.50 eV
Ge1Hf1O4_ICSD_202080	4.06 eV	3.60 eV
Ge1Hf1Te4_ICSD_241122	0.38 eV	0.32 eV
Ge1Hg1Te4Tl2_ICSD_172504	0.19 eV	0.33 eV
Ge1Hg2O4_ICSD_26340	0.98 eV	1.12 eV
Ge1Hg2Se4_ICSD_636599	0.40 eV	0.73 eV
Ge1Hg4S6_ICSD_636597	0.37 eV	0.84 eV
Ge1Ho1Na1O4_ICSD_95964	3.57 eV	3.45 eV
Ge1I1O6Rb1_ICSD_73613	2.46 eV	2.68 eV
Ge1I2La2_ICSD_414170	0.18 eV	0.32 eV
Ge1I2La2_ICSD_414171	0.22 eV	0.15 eV
Ge1I2Y2_ICSD_249477	0.18 eV	0.70 eV
Ge1I2_ICSD_23176	1.98 eV	1.78 eV
Ge1I2_ICSD_27674	1.80 eV	1.60 eV
Ge1I3Rb1_ICSD_40216	0.55 eV	1.14 eV
Ge1I3Rb1_ICSD_85041	2.21 eV	1.99 eV
Ge1I3Tl1_ICSD_201144	1.90 eV	2.06 eV
Ge1I4_ICSD_67895	1.97 eV	2.06 eV
Ge1I6K2O18_ICSD_2826	3.15 eV	2.59 eV
Ge1In1Li1O4_ICSD_62229	2.34 eV	2.54 eV
Ge1In2Li2S6_ICSD_262643	2.04 eV	1.92 eV
Ge1In2Li2Se6_ICSD_262645	1.13 eV	1.28 eV
Ge1In4S4_ICSD_400222	1.12 eV	1.13 eV
Ge1In7Ir1O8_ICSD_417829	0.45 eV	0.98 eV
Ge1K1La1S4_ICSD_73970	2.34 eV	2.35 eV
Ge1K1La1Se4_ICSD_73971	1.61 eV	1.60 eV
Ge1K1N1O1_ICSD_60002	2.64 eV	2.59 eV
Ge1K1Nd1S4_ICSD_603182	2.23 eV	2.22 eV
Ge1K1O5P1_ICSD_39735	3.18 eV	3.69 eV
Ge1K1O5Ta1_ICSD_39585	4.03 eV	3.48 eV
Ge1K1Pr1Se4_ICSD_409812	1.54 eV	1.47 eV
Ge1K1S4Tb1_ICSD_409811	2.42 eV	2.24 eV
Ge1K1S4Y1_ICSD_603205	2.40 eV	2.35 eV
Ge1K1_ICSD_43515	0.95 eV	0.90 eV
Ge1K2Se4_ICSD_78828	1.75 eV	1.67 eV
Ge1K2Te4_ICSD_38341	1.03 eV	0.96 eV
Ge1K4O4_ICSD_37271	2.79 eV	2.55 eV
Ge1K4S4_ICSD_418257	2.70 eV	2.51 eV
Ge1K4Se4_ICSD_418258	2.00 eV	1.94 eV
Ge1La2Mg1O6_ICSD_97016	4.29 eV	3.22 eV
Ge1La2O5_ICSD_59727	3.84 eV	3.73 eV
Ge1Li1Nd1O4_ICSD_200774	3.97 eV	3.11 eV
Ge1Li1O4Sc1_ICSD_62481	3.90 eV	3.59 eV
Ge1Li1O5Ta1_ICSD_39211	3.85 eV	3.63 eV
Ge1Li1Te2_ICSD_35676	0.31 eV	0.75 eV
Ge1Li2O3_ICSD_100403	3.51 eV	3.40 eV
Ge1Li2O4Rb2_ICSD_61087	3.35 eV	3.37 eV
Ge1Li2O5Ti1_ICSD_250297	3.82 eV	3.47 eV
Ge1Li2O5V1_ICSD_86777	1.80 eV	2.33 eV
Ge1Li2Pb1S4_ICSD_281011	2.26 eV	1.81 eV

Supplementary Table 804. Five-fold cross validated predictions for the band gap energy (198/277).

system	calculated	predicted
Ge1Li2Zn1_ICSD_171498	0.13 eV	0.58 eV
Ge1Li4S4_ICSD_95649	2.45 eV	2.52 eV
Ge1Li8O6_ICSD_65175	3.43 eV	3.37 eV
Ge1Lu1Na1O4_ICSD_54196	3.84 eV	3.52 eV
Ge1Mg1N2_ICSD_23502	2.59 eV	2.68 eV
Ge1Mg1N4Sr3_ICSD_97382	1.42 eV	1.55 eV
Ge1Mg1O3_ICSD_160840	2.44 eV	2.69 eV
Ge1Mg1O3_ICSD_171787	3.63 eV	3.11 eV
Ge1Mg1O3_ICSD_35533	2.83 eV	3.06 eV
Ge1Mg1O3_ICSD_35534	2.45 eV	3.05 eV
Ge1Mg1P2_ICSD_182369	1.49 eV	1.10 eV
Ge1Mg2O4_ICSD_1086	2.99 eV	2.85 eV
Ge1Mg2O4_ICSD_63533	3.35 eV	3.38 eV
Ge1Mg2O4_ICSD_86505	3.01 eV	3.40 eV
Ge1Mg2S4_ICSD_23525	2.29 eV	2.31 eV
Ge1Mg2_ICSD_52283	0.20 eV	0.53 eV
Ge1Mn1N2_ICSD_23528	0.93 eV	1.09 eV
Ge1Mn1O3_ICSD_69591	1.64 eV	1.44 eV
Ge1Mn1O4Zn1_ICSD_166628	1.02 eV	1.60 eV
Ge1Mn1O5Pr1_ICSD_80421	1.11 eV	1.63 eV
Ge1Mn1Te4Tl2_ICSD_172506	0.36 eV	0.42 eV
Ge1Mn2O4_ICSD_22046	0.83 eV	0.78 eV
Ge1Mn2O4_ICSD_23587	1.06 eV	1.07 eV
Ge1Mn2O4_ICSD_6312	0.29 eV	0.86 eV
Ge1Mn2S4_ICSD_656252	1.25 eV	1.21 eV
Ge1Mn2Se4_ICSD_391296	0.58 eV	0.76 eV
Ge1Mn2_ICSD_184947	0.22 eV	0.47 eV
Ge1N2O1Si1_ICSD_169122	3.59 eV	3.14 eV
Ge1N2O1Si1_ICSD_169123	3.51 eV	2.75 eV
Ge1N2Zn1_ICSD_656277	2.08 eV	1.67 eV
Ge1Na1Nb1O5_ICSD_39209	3.18 eV	3.13 eV
Ge1Na1O4Tb1_ICSD_54197	3.56 eV	3.52 eV
Ge1Na1O4Tm1_ICSD_54198	2.56 eV	2.66 eV
Ge1Na1O4Y1_ICSD_28213	1.56 eV	2.95 eV
Ge1Na1O4Y1_ICSD_85497	3.61 eV	3.12 eV
Ge1Na1O4Yb1_ICSD_54195	0.34 eV	1.15 eV
Ge1Na1O5Sb1_ICSD_39646	1.98 eV	2.40 eV
Ge1Na1O5Ta1_ICSD_39210	3.42 eV	3.45 eV
Ge1Na1_ICSD_43275	0.67 eV	0.71 eV
Ge1Na2O3_ICSD_1622	2.95 eV	2.90 eV
Ge1Na2O5Ti1_ICSD_160149	3.27 eV	3.46 eV
Ge1Na2O5Ti1_ICSD_20129	3.61 eV	3.37 eV
Ge1Na2O6Te1_ICSD_88789	2.65 eV	2.44 eV
Ge1Na2S3_ICSD_23455	2.45 eV	2.28 eV
Ge1Na2Se3_ICSD_49003	1.60 eV	1.51 eV
Ge1Na4O4_ICSD_61496	2.81 eV	2.61 eV
Ge1Ni2O4_ICSD_69508	1.97 eV	1.57 eV
Ge1O2_ICSD_158598	1.23 eV	1.31 eV
Ge1O2_ICSD_158600	1.18 eV	1.30 eV
Ge1O2_ICSD_23783	3.17 eV	2.87 eV

Supplementary Table 805. Five-fold cross validated predictions for the band gap energy (199/277).

system	calculated	predicted
Ge1O2_ICSD_281600	1.46 eV	2.18 eV
Ge1O2_ICSD_53869	0.89 eV	1.93 eV
Ge1O2_ICSD_59626	3.14 eV	3.08 eV
Ge1O2_ICSD_66592	3.14 eV	2.99 eV
Ge1O2_ICSD_94241	0.74 eV	1.95 eV
Ge1O3Pb1_ICSD_172521	2.66 eV	2.68 eV
Ge1O3Sr1_ICSD_28603	2.95 eV	2.79 eV
Ge1O3Zn1_ICSD_183725	1.87 eV	2.41 eV
Ge1O3Zn1_ICSD_33722	2.66 eV	2.22 eV
Ge1O4Sr2_ICSD_56382	3.17 eV	3.21 eV
Ge1O4Th1_ICSD_156945	3.76 eV	3.65 eV
Ge1O4Th1_ICSD_202081	3.71 eV	3.38 eV
Ge1O4U1_ICSD_16639	0.09 eV	1.95 eV
Ge1O4Zr1_ICSD_29262	3.75 eV	3.63 eV
Ge1O5Pb3_ICSD_100275	2.28 eV	1.86 eV
Ge1O5Pb3_ICSD_200517	1.63 eV	1.79 eV
Ge1O5Sr1Ti1_ICSD_158144	2.60 eV	2.79 eV
Ge1O6Se2_ICSD_422884	2.73 eV	2.69 eV
Ge1O6Sr1Te1_ICSD_88790	2.96 eV	2.70 eV
Ge1O6Te2_ICSD_260278	3.12 eV	2.64 eV
Ge1O7P2_ICSD_74876	3.79 eV	3.85 eV
Ge1O8P2Sr1_ICSD_423041	4.04 eV	3.99 eV
Ge1O8P2Sr1_ICSD_423042	4.01 eV	4.14 eV
Ge1O8Zr3_ICSD_29263	3.88 eV	3.47 eV
Ge1P1_ICSD_637492	0.50 eV	0.98 eV
Ge1P2Zn1_ICSD_637513	1.24 eV	1.03 eV
Ge1P4Sr4_ICSD_32558	1.04 eV	1.00 eV
Ge1Pb1S3_ICSD_2090	1.75 eV	1.87 eV
Ge1Pb2S4_ICSD_16272	1.99 eV	1.67 eV
Ge1Pd1S3_ICSD_408505	1.73 eV	1.07 eV
Ge1Pt1S1_ICSD_637646	0.91 eV	0.90 eV
Ge1Pt1Se1_ICSD_637651	0.54 eV	0.60 eV
Ge1Pt1Ti1_ICSD_188965	0.93 eV	0.50 eV
Ge1Rb1S5Ta1_ICSD_417874	0.97 eV	1.22 eV
Ge1Rb1_ICSD_43516	1.07 eV	1.00 eV
Ge1Rh1Te1_ICSD_260373	0.28 eV	0.40 eV
Ge1Ru1_ICSD_637744	0.27 eV	0.45 eV
Ge1S1_ICSD_155418	0.45 eV	1.06 eV
Ge1S1_ICSD_637785	1.20 eV	1.30 eV
Ge1S2_ICSD_167194	2.51 eV	2.01 eV
Ge1S2_ICSD_44	2.23 eV	2.15 eV
Ge1S2_ICSD_85527	2.16 eV	2.14 eV
Ge1S3Sn1_ICSD_637796	1.46 eV	1.62 eV
Ge1S4Sr2_ICSD_637797	2.28 eV	2.32 eV
Ge1S4Tl4_ICSD_100161	1.90 eV	1.51 eV
Ge1S4Yb2_ICSD_637815	1.75 eV	1.48 eV
Ge1Sb4Te7_ICSD_42875	0.23 eV	0.29 eV
Ge1Se1_ICSD_53906	0.28 eV	0.61 eV
Ge1Se1_ICSD_637854	0.82 eV	0.75 eV
Ge1Se2_ICSD_50761	1.43 eV	1.13 eV

Supplementary Table 806. Five-fold cross validated predictions for the band gap energy (200/277).

system	calculated	predicted
Ge1Se4Sr2_ICSD_413023	1.70 eV	1.51 eV
Ge1Se4Yb2_ICSD_637890	0.98 eV	1.07 eV
Ge1Sr2_ICSD_53923	0.38 eV	0.36 eV
Ge1Te1_ICSD_188458	0.69 eV	0.42 eV
Ge1Te1_ICSD_56042	0.34 eV	0.60 eV
Ge1Te1_ICSD_56604	0.51 eV	0.54 eV
Ge1Te1_ICSD_638005	0.48 eV	0.42 eV
Ge1Te1_ICSD_638009	0.36 eV	0.50 eV
Ge1Te4Zr1_ICSD_240906	0.30 eV	0.33 eV
Ge1Te6Ti1_ICSD_73014	0.41 eV	0.41 eV
Ge1_ICSD_173520	0.32 eV	0.33 eV
Ge2Hg3K2S8_ICSD_281506	1.48 eV	1.49 eV
Ge2In1Li1Yb2_ICSD_174355	0.12 eV	0.21 eV
Ge2In2O7_ICSD_20053	1.54 eV	2.18 eV
Ge2In2O7_ICSD_74896	1.87 eV	1.92 eV
Ge2In6O9Pt1_ICSD_170897	1.37 eV	1.21 eV
Ge2K2O6Zn1_ICSD_65740	3.11 eV	3.09 eV
Ge2K2O7Pb2_ICSD_30247	3.15 eV	2.41 eV
Ge2K2O7Zr1_ICSD_88843	3.84 eV	3.49 eV
Ge2K2Pb1S6_ICSD_170601	2.16 eV	2.01 eV
Ge2K4Se6_ICSD_300233	1.64 eV	1.68 eV
Ge2K6S6_ICSD_47111	2.38 eV	2.36 eV
Ge2K6Se6_ICSD_47112	1.78 eV	1.68 eV
Ge2K6Te6_ICSD_10108	1.05 eV	1.42 eV
Ge2La2O7_ICSD_202606	3.57 eV	3.10 eV
Ge2Li1N3_ICSD_636887	2.70 eV	2.44 eV
Ge2Li2O5_ICSD_28178	3.04 eV	3.10 eV
Ge2Li4N6Sr3_ICSD_96422	2.05 eV	1.94 eV
Ge2Lu2O7_ICSD_39929	3.84 eV	3.87 eV
Ge2Mg1O7Sr2_ICSD_420522	3.55 eV	3.38 eV
Ge2Mn1O7Sr2_ICSD_84033	2.08 eV	2.05 eV
Ge2N2O1_ICSD_200842	2.53 eV	2.51 eV
Ge2N3Na1_ICSD_47100	2.26 eV	2.35 eV
Ge2N4Si1_ICSD_183103	1.64 eV	2.49 eV
Ge2N6Sr5_ICSD_419164	1.36 eV	1.36 eV
Ge2Na1O6Sc1_ICSD_22503	3.54 eV	3.26 eV
Ge2Na2O7Zr1_ICSD_20402	3.64 eV	3.43 eV
Ge2Na2Se5_ICSD_40453	1.36 eV	1.50 eV
Ge2Na6S7_ICSD_25388	2.43 eV	2.34 eV
Ge2Na6Se6_ICSD_61400	1.88 eV	1.76 eV
Ge2Na6Te6_ICSD_47113	1.26 eV	1.13 eV
Ge2O7Sr2Zn1_ICSD_420521	3.34 eV	3.09 eV
Ge2O7Yb2_ICSD_65618	0.33 eV	0.55 eV
Ge2Os1_ICSD_43690	0.13 eV	0.47 eV
Ge2P2Rb6Se14_ICSD_261951	1.57 eV	1.49 eV
Ge2P4Sr3_ICSD_41182	0.91 eV	0.82 eV
Ge2Rb4S6_ICSD_409729	2.23 eV	2.22 eV
Ge2Rb4Se6_ICSD_402719	1.44 eV	1.59 eV
Ge2S6Tl4_ICSD_2240	1.70 eV	1.57 eV
Ge2Sb2Te5_ICSD_42876	0.16 eV	0.29 eV

Supplementary Table 807. Five-fold cross validated predictions for the band gap energy (201/277).

system	calculated	predicted
Ge2Se6Tl4_ICSD_35043	1.09 eV	1.03 eV
Ge2Sr1_ICSD_152799	0.31 eV	0.38 eV
Ge2Te6Tl6_ICSD_49658	0.66 eV	0.65 eV
Ge38I8P8_ICSD_16533	1.06 eV	1.11 eV
Ge3K1O9Ta1_ICSD_10380	3.46 eV	3.54 eV
Ge3La2O9_ICSD_83341	2.65 eV	3.02 eV
Ge3La4S12_ICSD_2046	2.04 eV	1.78 eV
Ge3Li2Ni1O8_ICSD_68339	2.89 eV	2.65 eV
Ge3N4_ICSD_156339	1.32 eV	1.69 eV
Ge3N4_ICSD_637162	1.94 eV	1.75 eV
Ge3N4_ICSD_658934	1.81 eV	1.58 eV
Ge3N4_ICSD_87767	1.85 eV	1.56 eV
Ge3N4_ICSD_97569	1.56 eV	1.64 eV
Ge3Na1P3_ICSD_184000	1.09 eV	0.84 eV
Ge3Nb1O9Rb1_ICSD_10382	3.33 eV	3.12 eV
Ge3O12Sb4_ICSD_421470	3.23 eV	2.65 eV
Ge3O12Sr3Y2_ICSD_80582	3.11 eV	3.36 eV
Ge3O25P6Si2_ICSD_202643	4.06 eV	4.00 eV
Ge3O8Zn2_ICSD_27103	2.66 eV	2.50 eV
Ge3O9Rb1Ta1_ICSD_10381	3.43 eV	3.45 eV
Ge3O9Sb2_ICSD_95735	2.94 eV	2.58 eV
Ge3O9Ta1Tl1_ICSD_10383	3.63 eV	3.39 eV
Ge3O9Ti1Tl2_ICSD_19029	2.74 eV	2.76 eV
Ge3Os2_ICSD_95593	0.90 eV	0.51 eV
Ge3Ru2_ICSD_95589	0.38 eV	0.27 eV
Ge4I4S6_ICSD_24371	2.17 eV	2.02 eV
Ge4K4Se10_ICSD_300262	1.78 eV	1.67 eV
Ge4Na1O12P3_ICSD_240922	4.40 eV	3.66 eV
Ge4Na4S10_ICSD_25382	2.46 eV	2.35 eV
Ge4Na4Se10_ICSD_300263	1.54 eV	1.46 eV
Ge4Na8Se10_ICSD_61401	1.66 eV	1.66 eV
Ge4Na8Te10_ICSD_37183	0.97 eV	1.09 eV
Ge4O9Pb1_ICSD_201282	3.43 eV	2.40 eV
Ge4S10Tl4_ICSD_640	2.05 eV	1.83 eV
Ge4Se10Tl4_ICSD_26415	1.43 eV	1.12 eV
Ge5Mg14O24_ICSD_9371	3.39 eV	3.53 eV
Ge5O25P6_ICSD_9212	3.50 eV	3.69 eV
Ge7H8N2O15_ICSD_407222	2.70 eV	3.19 eV
Ge9Li4O20_ICSD_34361	2.86 eV	2.76 eV
H10In1N3O14_ICSD_417341	3.53 eV	3.22 eV
H10In2O17S3_ICSD_391355	4.16 eV	3.83 eV
H10Mn1N1Na1O10P2_ICSD_413407	3.85 eV	3.05 eV
H10N2O8P2_ICSD_14169	5.68 eV	5.37 eV
H10N3Nd1O11S3_ICSD_410898	3.79 eV	3.63 eV
H10N4O4S1_ICSD_24103	5.13 eV	4.88 eV
H10O8S1_ICSD_2783	5.50 eV	5.21 eV
H10O8Se1_ICSD_404659	3.96 eV	4.11 eV
H11N2O4P1_ICSD_32572	5.45 eV	5.01 eV
H11Na2O8P1_ICSD_1602	5.21 eV	5.09 eV
H12I8Mg1O6_ICSD_32507	1.38 eV	2.58 eV

Supplementary Table 808. Five-fold cross validated predictions for the band gap energy (202/277).

system	calculated	predicted
H12K2N6Sn1_ICSD_79985	2.29 eV	2.22 eV
H12Li3O10V1_ICSD_174238	4.06 eV	2.71 eV
H12Li4N4O12P4_ICSD_415548	4.61 eV	5.02 eV
H12Mg1N6O6_ICSD_61681	4.49 eV	4.69 eV
H12Mg1O9S1_ICSD_48112	4.99 eV	5.23 eV
H12Mg1O9S2_ICSD_35664	4.37 eV	4.71 eV
H12Mg1O9Se1_ICSD_48115	5.08 eV	4.66 eV
H12Mg1O9Te1_ICSD_48114	4.42 eV	4.06 eV
H12Mn1N2O10S2_ICSD_410200	4.36 eV	3.22 eV
H12Mn2N4O8Zn1_ICSD_162384	1.56 eV	2.19 eV
H12N10O8Pd1_ICSD_412607	2.58 eV	2.06 eV
H12N16Pd2_ICSD_410384	0.16 eV	1.87 eV
H12N2Ni1O10S2_ICSD_410198	2.41 eV	2.55 eV
H12N2O14U1_ICSD_23825	3.49 eV	2.92 eV
H12N3Nb1O8_ICSD_161455	3.13 eV	3.34 eV
H12N4O4P2_ICSD_6211	4.85 eV	5.23 eV
H12N6Na2Sn1_ICSD_83963	1.12 eV	2.34 eV
H12N6Ni1O4_ICSD_201209	0.63 eV	2.10 eV
H12Na2O9Si1_ICSD_9171	4.68 eV	4.82 eV
H12Ni1O9S1_ICSD_26149	3.76 eV	3.27 eV
H12O12S2Zn1_ICSD_1836	5.18 eV	4.77 eV
H12O18P4Sr2_ICSD_61237	5.35 eV	5.32 eV
H13Mg1O9P1_ICSD_74520	5.60 eV	5.30 eV
H14N4O6Ru1S2_ICSD_40202	3.26 eV	2.78 eV
H14N4O8S2_ICSD_73623	5.18 eV	5.18 eV
H16Mg1N1O10P1_ICSD_29385	4.85 eV	5.04 eV
H16Mg1O10P2_ICSD_250187	5.53 eV	5.30 eV
H16N2O16P4Zn1_ICSD_59919	5.14 eV	4.53 eV
H16N4O12P4_ICSD_16148	5.25 eV	5.40 eV
H16Ni1O10P2_ICSD_281216	2.32 eV	3.04 eV
H16O20S3Y2_ICSD_281358	5.42 eV	4.84 eV
H1Hg1N1O4_ICSD_6186	1.70 eV	2.16 eV
H1Ho1O2_ICSD_2944	4.28 eV	4.07 eV
H1Ho1Se1_ICSD_78957	1.49 eV	1.56 eV
H1I1Li2O1_ICSD_74930	4.33 eV	3.62 eV
H1I1O3_ICSD_415140	3.05 eV	3.02 eV
H1I1O3_ICSD_66643	3.22 eV	2.92 eV
H1I1O4Zn1_ICSD_185598	3.48 eV	3.10 eV
H1I1Sr1_ICSD_25546	3.36 eV	3.17 eV
H1In1O2_ICSD_166255	1.71 eV	2.25 eV
H1In1O2_ICSD_166257	1.72 eV	2.25 eV
H1In1O2_ICSD_24093	2.05 eV	1.77 eV
H1In1O4Se1_ICSD_415267	3.56 eV	3.09 eV
H1K1O1_ICSD_61047	3.48 eV	3.35 eV
H1K1O3Se1_ICSD_20862	4.23 eV	3.69 eV
H1K1O3Si1_ICSD_30892	4.74 eV	4.36 eV
H1K1O7Se2V1_ICSD_82535	1.96 eV	1.81 eV
H1K1O8P2Zn2_ICSD_412588	3.25 eV	4.08 eV
H1K1_ICSD_183287	3.44 eV	3.40 eV
H1K2N1O6S2_ICSD_15880	5.10 eV	4.42 eV

Supplementary Table 809. Five-fold cross validated predictions for the band gap energy (203/277).

system	calculated	predicted
H1K3O8S2_ICSD_249551	5.41 eV	4.97 eV
H1La1O1_ICSD_48122	2.19 eV	3.27 eV
H1La1O2_ICSD_60675	4.51 eV	3.68 eV
H1La2Li1O3_ICSD_56744	1.92 eV	2.44 eV
H1Li1Nd2O3_ICSD_56747	1.62 eV	2.30 eV
H1Li1O3P1Tl1_ICSD_201040	3.99 eV	3.15 eV
H1Li1O3Se1_ICSD_8255	4.31 eV	3.98 eV
H1Li1O4S1_ICSD_82373	5.98 eV	5.49 eV
H1Li1S1_ICSD_98019	3.54 eV	3.37 eV
H1Li1S1_ICSD_98020	3.51 eV	3.27 eV
H1Li1S1_ICSD_98021	3.48 eV	3.16 eV
H1Li1_ICSD_173192	2.96 eV	3.52 eV
H1Li2N1_ICSD_168791	2.14 eV	2.28 eV
H1Li2N1_ICSD_169858	2.59 eV	2.48 eV
H1Li2O8P2Sc1_ICSD_409955	5.02 eV	4.94 eV
H1Li3O4Te1_ICSD_38036	3.60 eV	3.44 eV
H1Li4N1_ICSD_409633	1.92 eV	1.88 eV
H1Mg1Na3O8P2_ICSD_81226	4.88 eV	4.93 eV
H1Mg2O5P1_ICSD_40828	4.96 eV	4.81 eV
H1Mn1O2_ICSD_27457	1.90 eV	1.32 eV
H1Mn1O2_ICSD_84949	0.64 eV	1.68 eV
H1Mn1O7P2_ICSD_415152	1.38 eV	2.37 eV
H1N1O4Zn1_ICSD_415537	1.95 eV	3.12 eV
H1N1Sr1_ICSD_410656	1.73 eV	2.53 eV
H1N2O9P1Pb2_ICSD_260268	3.33 eV	3.17 eV
H1N3Si2_ICSD_202970	3.98 eV	3.97 eV
H1Na1O1_ICSD_41231	2.84 eV	3.13 eV
H1Na1O1_ICSD_61045	2.86 eV	3.15 eV
H1Na1O4S1_ICSD_1436	2.83 eV	3.99 eV
H1Na1O4Se1_ICSD_88893	3.65 eV	3.50 eV
H1Na1O4Si1Zn1_ICSD_88001	3.92 eV	4.05 eV
H1Na1O7P2Zn1_ICSD_39491	4.97 eV	4.67 eV
H1Na1O8P2Zn2_ICSD_73954	4.52 eV	4.39 eV
H1Na1_ICSD_261353	3.80 eV	2.36 eV
H1Na1_ICSD_261354	1.04 eV	2.93 eV
H1Na2O5P1Zn1_ICSD_280153	3.95 eV	3.89 eV
H1Nd1O2_ICSD_162390	3.75 eV	3.13 eV
H1Ni1O2_ICSD_169981	0.24 eV	1.84 eV
H1O10P3Pb2_ICSD_2494	4.30 eV	3.60 eV
H1O1Rb1_ICSD_61048	3.13 eV	2.99 eV
H1O2Tb1_ICSD_6164	3.54 eV	3.49 eV
H1O2Y1_ICSD_28442	4.67 eV	4.28 eV
H1O2Yb1_ICSD_26341	0.29 eV	0.59 eV
H1O3P1Sn1_ICSD_25034	2.53 eV	3.02 eV
H1O4P1Sn1_ICSD_658	3.38 eV	3.44 eV
H1O4Rb1S1_ICSD_36326	5.32 eV	4.78 eV
H1O5P1Zn2_ICSD_62245	4.03 eV	3.86 eV
H1O5P1Zn2_ICSD_79334	3.87 eV	3.89 eV
H1O5P1Zn2_ICSD_81313	3.88 eV	3.85 eV
H1O5Rb5Si1_ICSD_417054	2.60 eV	2.99 eV

Supplementary Table 810. Five-fold cross validated predictions for the band gap energy (204/277).

system	calculated	predicted
H1O8P2Rb1Zn2_ICSD_280530	4.39 eV	4.14 eV
H1O8Rb3S2_ICSD_60050	5.24 eV	4.94 eV
H1Rb1_ICSD_183292	2.91 eV	2.87 eV
H1Se1Y1_ICSD_72008	1.52 eV	1.64 eV
H1Si1_ICSD_41478	2.16 eV	2.47 eV
H1_ICSD_426942	8.61 eV	6.76 eV
H20N4O2P4S8_ICSD_16451	2.57 eV	3.24 eV
H24Li2N8Te2_ICSD_409556	1.34 eV	2.40 eV
H2Hg1O5Se1_ICSD_1712	1.49 eV	2.31 eV
H2Hg2O11S2Zn2_ICSD_413515	2.12 eV	2.55 eV
H2I2O1Sr1_ICSD_76503	3.62 eV	3.54 eV
H2I3K1O1_ICSD_26338	1.94 eV	2.36 eV
H2In1O5P1_ICSD_86674	3.50 eV	3.79 eV
H2K1Mn1O5P1_ICSD_71177	3.19 eV	2.81 eV
H2K1N1O3S1_ICSD_15324	5.16 eV	4.97 eV
H2K1N1_ICSD_25619	2.08 eV	2.41 eV
H2K1O2P1_ICSD_59833	5.13 eV	4.81 eV
H2K1O3P1S1_ICSD_75217	4.53 eV	4.52 eV
H2K1O4P1_ICSD_186817	5.40 eV	4.91 eV
H2K1O4P1_ICSD_29258	4.92 eV	4.87 eV
H2K1O4P1_ICSD_31151	5.45 eV	5.37 eV
H2K1O4P1_ICSD_87895	5.62 eV	5.20 eV
H2K2Mn3O10V2_ICSD_203214	1.98 eV	1.84 eV
H2K2O5Ru1_ICSD_36593	1.59 eV	2.50 eV
H2K2O7P2_ICSD_36043	2.96 eV	4.22 eV
H2K3O10Si3Tb1_ICSD_413425	4.61 eV	4.24 eV
H2La1N1O7S1_ICSD_415675	1.50 eV	2.83 eV
H2Li1N1O3_ICSD_37180	2.79 eV	2.96 eV
H2Li1N1Sr2_ICSD_417994	1.46 eV	1.84 eV
H2Li1N1_ICSD_168792	3.18 eV	2.91 eV
H2Li1O2P1_ICSD_59823	5.31 eV	4.63 eV
H2Li1O3P1_ICSD_15045	4.26 eV	4.25 eV
H2Li1O3P1_ICSD_7	3.37 eV	4.54 eV
H2Li1O5P1Zn1_ICSD_79351	4.46 eV	4.33 eV
H2Li2Mg1N2_ICSD_165890	2.35 eV	3.00 eV
H2Li2O5S1_ICSD_20617	5.56 eV	5.42 eV
H2Li2O5Se1_ICSD_281276	4.00 eV	3.94 eV
H2Mg1Mo1O5_ICSD_202695	3.97 eV	2.93 eV
H2Mg1O2_ICSD_79032	4.35 eV	4.16 eV
H2Mg1O5S1_ICSD_26304	5.50 eV	5.11 eV
H2Mg1O5Se1_ICSD_66745	3.40 eV	3.85 eV
H2Mg1O6Se2_ICSD_59310	4.13 eV	3.64 eV
H2Mg1O8S2_ICSD_62325	0.32 eV	3.99 eV
H2Mg1O8Se2_ICSD_66966	3.24 eV	3.46 eV
H2Mg1_ICSD_155807	3.74 eV	3.12 eV
H2Mg1_ICSD_155808	3.65 eV	2.90 eV
H2Mg1_ICSD_155809	1.93 eV	2.67 eV
H2Mg1_ICSD_155810	2.66 eV	3.33 eV
H2Mg1_ICSD_155811	2.10 eV	2.29 eV
H2Mg2O3_ICSD_95472	3.56 eV	3.72 eV

Supplementary Table 811. Five-fold cross validated predictions for the band gap energy (205/277).

system	calculated	predicted
H2Mg3O4_ICSD_95471	3.78 eV	3.75 eV
H2Mg4O5_ICSD_95470	4.01 eV	3.92 eV
H2Mg5O6_ICSD_95469	3.98 eV	3.88 eV
H2Mn1O2_ICSD_23591	1.66 eV	1.95 eV
H2Mn1O5P1_ICSD_62220	1.45 eV	2.50 eV
H2Mn1O7P2_ICSD_419085	4.41 eV	2.96 eV
H2Mn1O8S2_ICSD_408752	4.22 eV	2.92 eV
H2Mn2O6S1_ICSD_413556	2.83 eV	2.80 eV
H2Mn3O10Se3_ICSD_281509	2.76 eV	2.37 eV
H2Mn6O17P4_ICSD_412152	2.93 eV	2.37 eV
H2N1Na1_ICSD_34290	1.99 eV	2.28 eV
H2N2Na2O4_ICSD_10182	3.01 eV	2.85 eV
H2N2O5Sr1_ICSD_38403	2.27 eV	2.77 eV
H2N2S6_ICSD_2535	2.89 eV	2.23 eV
H2Na3O6Os1_ICSD_172060	0.93 eV	1.64 eV
H2Ni1O2_ICSD_169978	3.09 eV	2.10 eV
H2Ni1O5S1_ICSD_71347	3.51 eV	2.48 eV
H2Ni1O5Se1_ICSD_66748	0.91 eV	2.40 eV
H2O10P2Ti2_ICSD_160167	3.23 eV	2.93 eV
H2O13Ti6_ICSD_186994	3.23 eV	2.84 eV
H2O1_ICSD_27837	5.53 eV	5.42 eV
H2O1_ICSD_31683	5.52 eV	5.42 eV
H2O1_ICSD_64771	5.53 eV	5.51 eV
H2O2P1Rb1_ICSD_250131	5.15 eV	4.83 eV
H2O2Pb1_ICSD_20701	2.26 eV	2.54 eV
H2O2Zn1_ICSD_50447	3.70 eV	3.35 eV
H2O2_ICSD_34253	4.45 eV	4.33 eV
H2O3Se1_ICSD_31929	4.51 eV	3.80 eV
H2O4P1Rb1_ICSD_69317	5.40 eV	5.06 eV
H2O4P1Rb1_ICSD_69318	5.27 eV	5.22 eV
H2O4P1Tl1_ICSD_8051	3.19 eV	3.70 eV
H2O4P1Tl1_ICSD_81068	4.03 eV	3.33 eV
H2O4Pb3_ICSD_60054	2.84 eV	2.47 eV
H2O4S1_ICSD_38429	6.11 eV	4.92 eV
H2O4S1_ICSD_82732	0.55 eV	2.25 eV
H2O4S1_ICSD_95409	6.02 eV	5.00 eV
H2O4Se1_ICSD_60942	3.36 eV	3.72 eV
H2O4U1_ICSD_2867	3.59 eV	3.00 eV
H2O4U1_ICSD_9153	2.91 eV	3.34 eV
H2O5S1Zn1_ICSD_71348	4.86 eV	4.38 eV
H2O5Se1V1_ICSD_69994	1.92 eV	1.77 eV
H2O5Se1Zn1_ICSD_66749	2.95 eV	3.26 eV
H2O6P2Rb1V1_ICSD_423279	1.44 eV	2.08 eV
H2O6P2Zr1_ICSD_201970	4.11 eV	4.15 eV
H2O6P2Zr1_ICSD_74463	4.15 eV	4.11 eV
H2O6S1Ti1_ICSD_72973	2.74 eV	3.28 eV
H2O6Se2Sr1_ICSD_410935	4.20 eV	3.88 eV
H2O8Pb4S1_ICSD_84850	2.79 eV	3.00 eV
H2O8S2Zn1_ICSD_63418	2.92 eV	3.08 eV
H2O8Se2Zn1_ICSD_66967	2.75 eV	2.86 eV

Supplementary Table 812. Five-fold cross validated predictions for the band gap energy (206/277).

system	calculated	predicted
H2S1_ICSD_168207	4.21 eV	3.21 eV
H2S1_ICSD_50333	3.25 eV	3.80 eV
H2Sr1_ICSD_163569	3.10 eV	2.33 eV
H2Sr1_ICSD_163576	1.30 eV	1.99 eV
H2_ICSD_28344	6.42 eV	6.60 eV
H2_ICSD_28465	7.38 eV	7.23 eV
H2_ICSD_28539	6.07 eV	6.90 eV
H2_ICSD_62003	6.83 eV	7.23 eV
H2_ICSD_67425	7.37 eV	6.56 eV
H3Ho1_ICSD_638194	0.17 eV	1.10 eV
H3I1Li1N1_ICSD_55064	0.80 eV	2.38 eV
H3I1Si1_ICSD_65044	4.11 eV	3.33 eV
H3I1Sr2_ICSD_422181	2.07 eV	2.45 eV
H3I3N2_ICSD_27416	0.78 eV	2.00 eV
H3K1Li2O3_ICSD_65148	2.85 eV	3.61 eV
H3K1Mg1_ICSD_638209	2.50 eV	1.67 eV
H3K1N1O3P1_ICSD_23291	4.12 eV	4.83 eV
H3K1O2_ICSD_47114	4.41 eV	4.20 eV
H3K1O6Se2_ICSD_2838	4.57 eV	3.92 eV
H3K1Si1_ICSD_65954	2.50 eV	3.94 eV
H3La1O12Se3_ICSD_411831	0.62 eV	2.17 eV
H3La1O3_ICSD_167480	3.62 eV	3.46 eV
H3Li1Mg1_ICSD_181326	4.09 eV	3.23 eV
H3Li1O2_ICSD_9138	4.74 eV	4.55 eV
H3Li1O6Se2_ICSD_8243	4.30 eV	3.78 eV
H3Li1Sr1_ICSD_23978	1.81 eV	2.10 eV
H3Li4Mg1N3_ICSD_165891	2.62 eV	2.85 eV
H3Mg1Na1_ICSD_159174	3.30 eV	3.49 eV
H3N1Na1O3P1_ICSD_16608	5.18 eV	4.98 eV
H3N1O3S1_ICSD_802	5.32 eV	4.85 eV
H3N1O6Sr2_ICSD_415799	2.89 eV	3.21 eV
H3N1_ICSD_84461	4.33 eV	5.18 eV
H3N3Pb1S2_ICSD_49538	1.89 eV	2.51 eV
H3Na1O2_ICSD_33909	4.36 eV	4.19 eV
H3Na1O5S1_ICSD_28259	5.40 eV	5.08 eV
H3Na1O6Si2_ICSD_39749	5.06 eV	4.65 eV
H3Na1O8S2Zn2_ICSD_61213	4.75 eV	4.35 eV
H3O2Rb1_ICSD_47115	4.23 eV	4.26 eV
H3O3P1_ICSD_33790	7.10 eV	5.88 eV
H3O3Tb1_ICSD_1224	3.55 eV	3.51 eV
H3O3Y1_ICSD_200098	3.68 eV	4.13 eV
H3O4P1Sr1_ICSD_411738	5.77 eV	5.75 eV
H3O4P1_ICSD_15887	5.91 eV	5.72 eV
H3O9P3Sb2_ICSD_26555	3.92 eV	3.64 eV
H3O9P3Sc2_ICSD_409724	4.99 eV	5.24 eV
H3Tb1_ICSD_638485	0.17 eV	1.11 eV
H3Y1_ICSD_154809	0.21 eV	2.26 eV
H4.001N1.667Rb1_ICSD_418413	2.11 eV	2.52 eV
H4Hg2O9P2_ICSD_413085	1.92 eV	2.81 eV
H4I1N1O4_ICSD_280083	2.42 eV	2.63 eV

Supplementary Table 813. Five-fold cross validated predictions for the band gap energy (207/277).

system	calculated	predicted
H4I1Na1O2_ICSD_23134	3.72 eV	3.47 eV
H4I1P1_ICSD_22083	3.68 eV	3.26 eV
H4I2O8Zn1_ICSD_410881	3.38 eV	3.07 eV
H4I3N1Pb1_ICSD_240915	0.92 eV	1.93 eV
H4I3N1_ICSD_48202	1.81 eV	1.72 eV
H4I3O11Yb1_ICSD_413352	0.19 eV	1.60 eV
H4I4K2O14Zn1_ICSD_260726	3.20 eV	3.05 eV
H4K2Mg1_ICSD_68358	3.48 eV	3.08 eV
H4K2Mn3O12P4_ICSD_260451	4.34 eV	2.88 eV
H4K2O6Os1_ICSD_409612	0.79 eV	1.98 eV
H4K2Zn1_ICSD_187277	4.19 eV	3.69 eV
H4La1N1O8S2_ICSD_20895	5.48 eV	4.07 eV
H4Li2Mg1_ICSD_181325	3.53 eV	2.99 eV
H4Mg1Na2O8S2_ICSD_35767	5.05 eV	4.96 eV
H4Mg2Ni1_ICSD_162413	1.66 eV	2.25 eV
H4Mg3O5_ICSD_95473	3.57 eV	3.62 eV
H4Mg3O9Si2_ICSD_75933	4.25 eV	4.64 eV
H4N1O3V1_ICSD_164689	3.11 eV	2.56 eV
H4N1O4Re1_ICSD_1394	4.17 eV	2.93 eV
H4N1O4Tc1_ICSD_10427	3.35 eV	2.55 eV
H4N2Ni1_ICSD_69723	2.16 eV	2.33 eV
H4N2O3_ICSD_27453	3.22 eV	3.24 eV
H4N2O3_ICSD_2772	3.12 eV	3.40 eV
H4N2O3_ICSD_37128	3.28 eV	3.21 eV
H4N2O8Zn1_ICSD_9286	3.43 eV	2.78 eV
H4N4S4_ICSD_15394	3.30 eV	2.91 eV
H4N4_ICSD_2236	4.16 eV	3.52 eV
H4Na2O10Se2Zn1_ICSD_98685	3.20 eV	3.63 eV
H4Na2O5Ti1_ICSD_183668	0.88 eV	2.64 eV
H4Na2O8S4_ICSD_40833	3.76 eV	3.83 eV
H4Na2O8Se2_ICSD_71786	4.09 eV	3.90 eV
H4Na2Pt1_ICSD_638356	2.52 eV	2.67 eV
H4Na6O18S4Zn1_ICSD_61246	5.02 eV	4.74 eV
H4Ni2O10P2Sr1_ICSD_261127	2.04 eV	2.63 eV
H4O10Si2Zn4_ICSD_422820	3.78 eV	3.69 eV
H4O10V4_ICSD_260368	1.02 eV	1.54 eV
H4O11P2Ti2_ICSD_84307	3.09 eV	2.98 eV
H4O12S3Sr1_ICSD_404139	5.85 eV	5.33 eV
H4O3Sr1_ICSD_63016	4.34 eV	4.37 eV
H4O4P2Pb1_ICSD_59933	3.88 eV	3.86 eV
H4O4P2Sr1_ICSD_59932	5.42 eV	5.47 eV
H4O4P2Zn1_ICSD_66911	4.25 eV	4.52 eV
H4O4V2_ICSD_41123	1.20 eV	1.58 eV
H4O6P2Sr1_ICSD_412173	6.48 eV	5.88 eV
H4O8P2Pb1_ICSD_86778	4.33 eV	3.92 eV
H4O9P2V1_ICSD_20182	2.28 eV	1.91 eV
H4S6Si4_ICSD_36379	3.32 eV	3.44 eV
H4Si1_ICSD_159307	5.83 eV	4.38 eV
H5I1O6_ICSD_25790	2.33 eV	2.72 eV
H5K1O8P2_ICSD_23097	5.61 eV	5.26 eV

Supplementary Table 814. Five-fold cross validated predictions for the band gap energy (208/277).

system	calculated	predicted
H5K2O6Sb1_ICSD_422765	3.13 eV	3.12 eV
H5K3O14P4_ICSD_30511	5.30 eV	5.25 eV
H5K3Zn1_ICSD_187417	3.70 eV	3.43 eV
H5Li1N2O4S1_ICSD_2051	5.47 eV	5.16 eV
H5N1O3Se1_ICSD_171372	4.42 eV	3.87 eV
H5Na3O16Se4_ICSD_411720	3.45 eV	3.46 eV
H5O8P2Rb1_ICSD_20915	3.27 eV	4.32 eV
H5O8P2Tl1_ICSD_30509	5.00 eV	4.24 eV
H5Rb3Zn1_ICSD_187418	3.27 eV	3.47 eV
H6Hf1Na2O6_ICSD_88890	4.41 eV	3.93 eV
H6Hf1O10P2_ICSD_94481	5.23 eV	4.27 eV
H6Hg1I3N1O1_ICSD_409701	1.64 eV	2.04 eV
H6Ir1Li1Mg1_ICSD_180122	3.31 eV	2.80 eV
H6Ir1Na3_ICSD_40575	3.71 eV	3.19 eV
H6K2O6Pb1_ICSD_92466	2.14 eV	3.04 eV
H6K2O6Sn1_ICSD_92465	3.31 eV	2.95 eV
H6La1O12P3_ICSD_240963	5.25 eV	4.48 eV
H6Li1Mn1O7_ICSD_33776	2.17 eV	2.88 eV
H6Li1N1O6_ICSD_48040	3.28 eV	3.28 eV
H6Li2N3Rb1_ICSD_56394	0.46 eV	1.76 eV
H6Li2Pt1_ICSD_236283	2.68 eV	2.84 eV
H6Li4Ru1_ICSD_638272	3.04 eV	2.63 eV
H6Mg1O6Si1_ICSD_185677	5.48 eV	4.93 eV
H6Mg2Na2Ni1_ICSD_260394	1.83 eV	2.39 eV
H6N1Nd1O10S1_ICSD_416245	3.20 eV	3.00 eV
H6N1Ni1O5P1_ICSD_424553	3.54 eV	3.04 eV
H6N1O2P1_ICSD_250178	5.07 eV	4.79 eV
H6N1O4P1_ICSD_246299	5.51 eV	5.09 eV
H6N1O4P1_ICSD_29257	5.21 eV	5.43 eV
H6N2O4S1_ICSD_16055	2.14 eV	3.97 eV
H6N2O4S1_ICSD_1977	5.41 eV	5.36 eV
H6N3O12Tl1_ICSD_1723	1.91 eV	2.94 eV
H6N3O1P1_ICSD_16151	4.70 eV	4.77 eV
H6N4O4Pd1_ICSD_420291	2.58 eV	2.26 eV
H6N4O4Pt1_ICSD_420292	2.48 eV	2.55 eV
H6Na1O6P1_ICSD_66	5.22 eV	5.08 eV
H6Na2O6Sn1_ICSD_92464	3.10 eV	3.25 eV
H6Na2Pt1_ICSD_236284	3.47 eV	2.79 eV
H6Na4Ru1_ICSD_638362	2.68 eV	2.46 eV
H6Nd1O12P3_ICSD_426743	3.22 eV	3.28 eV
H6O10P2Zr1_ICSD_94480	4.41 eV	4.12 eV
H6O12P3Pr1_ICSD_155271	2.33 eV	3.37 eV
H6O12P3Sc1_ICSD_20432	5.40 eV	5.17 eV
H6O12Sc2Se3_ICSD_391277	4.66 eV	3.90 eV
H6O1Si2_ICSD_30501	5.26 eV	4.74 eV
H6O5Sr1Zn1_ICSD_50177	4.01 eV	3.93 eV
H6O8Se2Zn1_ICSD_200525	0.42 eV	2.60 eV
H6Os1Sr2_ICSD_638409	2.88 eV	2.30 eV
H6Ru1Sr2_ICSD_655142	2.85 eV	2.89 eV
H7La2O11P3_ICSD_249116	5.50 eV	4.77 eV

Supplementary Table 815. Five-fold cross validated predictions for the band gap energy (209/277).

system	calculated	predicted
H7Mg3Tc1_ICSD_262724	2.98 eV	3.02 eV
H7N1O6_ICSD_1902	3.57 eV	3.48 eV
H7N2O4P1_ICSD_15083	5.42 eV	5.27 eV
H7Na5O18Se4_ICSD_40276	3.31 eV	3.87 eV
H8I1N4P1_ICSD_400461	1.01 eV	2.74 eV
H8I1N4P1_ICSD_74814	4.45 eV	3.55 eV
H8K1N3_ICSD_414496	2.27 eV	3.38 eV
H8K1N4Y1_ICSD_202010	2.68 eV	2.83 eV
H8K2Ni1O16P4_ICSD_281592	4.46 eV	3.66 eV
H8K2O14V2_ICSD_67322	1.53 eV	2.29 eV
H8K2O16P4Zn1_ICSD_281290	5.23 eV	5.25 eV
H8K4O4P2Se6_ICSD_260476	1.85 eV	2.53 eV
H8La1O7P3_ICSD_88015	5.20 eV	5.35 eV
H8Mg1O14V4_ICSD_185911	1.84 eV	1.85 eV
H8Mg5O9_ICSD_95474	3.61 eV	3.80 eV
H8Mn1O16P4Tl2_ICSD_416649	1.36 eV	2.62 eV
H8Mo1N2O2S2_ICSD_41688	2.07 eV	2.51 eV
H8N10Zn1_ICSD_421952	4.11 eV	3.10 eV
H8N2O4S1_ICSD_34258	5.08 eV	4.91 eV
H8N2O4Se1_ICSD_838	3.61 eV	4.05 eV
H8N2S5_ICSD_30984	2.15 eV	2.12 eV
H8N8Na1O12Rh1_ICSD_169810	2.60 eV	2.61 eV
H8Ni1O12Re2_ICSD_402773	2.77 eV	2.69 eV
H8Ni1O16P4Tl2_ICSD_416651	2.51 eV	2.81 eV
H8O10P2Zn1_ICSD_417431	0.18 eV	3.46 eV
H8O12Si8_ICSD_75244	6.37 eV	5.25 eV
H8O16P4Tl2Zn1_ICSD_416652	4.96 eV	4.11 eV
H8O8Sn1Sr2_ICSD_91102	3.03 eV	3.25 eV
H8O8U1_ICSD_167992	3.04 eV	2.99 eV
H8Si1_ICSD_169739	6.81 eV	6.61 eV
H8Si1_ICSD_169740	6.76 eV	6.55 eV
H9K1O5_ICSD_36598	4.71 eV	4.57 eV
H9K2Re1_ICSD_27563	5.23 eV	3.18 eV
H9N1Si3_ICSD_201428	4.20 eV	4.11 eV
H9N2O4P1_ICSD_2799	4.93 eV	5.15 eV
Hf1I1N1_ICSD_51774	0.98 eV	1.32 eV
Hf1I4_ICSD_109275	3.24 eV	2.39 eV
Hf1I4_ICSD_200826	2.35 eV	1.97 eV
Hf1I4_ICSD_62473	2.41 eV	2.36 eV
Hf1Mo2O8_ICSD_98065	2.83 eV	2.37 eV
Hf1N1O3Ta1_ICSD_186407	2.07 eV	1.98 eV
Hf1N1O3Ta1_ICSD_186408	1.46 eV	2.17 eV
Hf1N1O3Ta1_ICSD_186409	2.98 eV	2.20 eV
Hf1N2Sr1_ICSD_82538	0.32 eV	0.73 eV
Hf1N2_ICSD_290428	0.59 eV	1.22 eV
Hf1N2_ICSD_290429	1.18 eV	1.50 eV
Hf1Ni1Sn1_ICSD_638731	0.15 eV	0.29 eV
Hf1O1S1_ICSD_23327	2.87 eV	1.98 eV
Hf1O2_ICSD_173965	4.23 eV	3.80 eV
Hf1O2_ICSD_173966	4.67 eV	3.67 eV

Supplementary Table 816. Five-fold cross validated predictions for the band gap energy (210/277).

system	calculated	predicted
Hf1O2_ICSD_173968	3.38 eV	3.93 eV
Hf1O2_ICSD_180834	3.80 eV	3.92 eV
Hf1O2_ICSD_187015	4.01 eV	3.71 eV
Hf1O2_ICSD_83863	3.42 eV	3.86 eV
Hf1O3Pb1_ICSD_161702	2.42 eV	2.38 eV
Hf1O3Pb1_ICSD_174110	2.78 eV	2.70 eV
Hf1O3Pb1_ICSD_33194	2.83 eV	2.88 eV
Hf1O3Sr1_ICSD_161594	3.73 eV	3.79 eV
Hf1O3Sr1_ICSD_161595	3.68 eV	3.82 eV
Hf1O3Sr1_ICSD_161597	3.99 eV	3.78 eV
Hf1O3Sr1_ICSD_161598	4.08 eV	3.88 eV
Hf1O3Sr1_ICSD_164620	3.73 eV	3.63 eV
Hf1O3Sr1_ICSD_164622	3.96 eV	3.80 eV
Hf1O3Sr1_ICSD_84280	4.17 eV	3.88 eV
Hf1O4Si1_ICSD_187732	5.30 eV	4.62 eV
Hf1O7V2_ICSD_90134	2.64 eV	2.60 eV
Hf1O8Te3_ICSD_9078	3.85 eV	2.94 eV
Hf1O8W2_ICSD_188441	3.82 eV	3.29 eV
Hf1P2S6_ICSD_47228	0.83 eV	1.24 eV
Hf1Pb1S3_ICSD_65668	1.43 eV	1.23 eV
Hf1Pd1Sn1_ICSD_106773	0.18 eV	0.22 eV
Hf1S2_ICSD_182677	1.22 eV	1.16 eV
Hf1S3Sn1_ICSD_65667	1.24 eV	1.17 eV
Hf1S3_ICSD_638846	1.12 eV	1.10 eV
Hf1S4Tl4_ICSD_261210	1.50 eV	1.52 eV
Hf1S5Y2_ICSD_185	1.23 eV	1.26 eV
Hf1Se2_ICSD_182678	0.51 eV	0.55 eV
Hf1Se3Tl2_ICSD_186071	0.65 eV	0.69 eV
Hf1Se3_ICSD_638898	0.26 eV	0.54 eV
Hf1Te4Tl4_ICSD_168651	0.43 eV	0.59 eV
Hf2Ho2O7_ICSD_162005	3.12 eV	3.25 eV
Hf2La2O7_ICSD_153815	4.46 eV	3.90 eV
Hf2Nd2O7_ICSD_236210	3.77 eV	3.62 eV
Hf2O7Y2_ICSD_153819	3.34 eV	3.52 eV
Hf2O9P2_ICSD_416013	3.51 eV	4.63 eV
Hf3N4_ICSD_97997	1.02 eV	1.14 eV
Hf5K4O12_ICSD_180186	3.33 eV	3.64 eV
Hg1Ho2O4_ICSD_69734	2.09 eV	2.10 eV
Hg1I1N1O3_ICSD_32580	1.94 eV	2.31 eV
Hg1I2O6_ICSD_98546	2.69 eV	2.27 eV
Hg1I2_ICSD_281133	1.40 eV	1.64 eV
Hg1I2_ICSD_412988	2.26 eV	1.70 eV
Hg1I2_ICSD_67069	1.29 eV	1.90 eV
Hg1I6Tl4_ICSD_14018	1.13 eV	1.55 eV
Hg1I6Tl4_ICSD_250133	1.14 eV	1.55 eV
Hg1In2S4_ICSD_56081	0.42 eV	1.04 eV
Hg1In2Se4_ICSD_25649	0.67 eV	0.73 eV
Hg1In2Te4_ICSD_639056	0.56 eV	0.58 eV
Hg1K2O2_ICSD_66275	2.07 eV	1.86 eV
Hg1K2S2_ICSD_74500	2.12 eV	1.93 eV

Supplementary Table 817. Five-fold cross validated predictions for the band gap energy (211/277).

system	calculated	predicted
Hg1K2Se4Sn1_ICSD_413308	1.39 eV	1.22 eV
Hg1K4P2_ICSD_67263	0.94 eV	0.86 eV
Hg1K6S4_ICSD_266	1.71 eV	1.85 eV
Hg1K6Se4_ICSD_639060	1.41 eV	1.61 eV
Hg1Mo1O4_ICSD_2533	2.24 eV	2.17 eV
Hg1N3O6Tl1_ICSD_28646	0.61 eV	1.58 eV
Hg1N6_ICSD_21029	2.57 eV	2.39 eV
Hg1Na1O4P1_ICSD_32511	1.43 eV	2.12 eV
Hg1Na2O2_ICSD_27409	1.66 eV	1.74 eV
Hg1Na2S2_ICSD_74499	2.17 eV	1.86 eV
Hg1Na4P2_ICSD_67260	0.91 eV	0.87 eV
Hg1O14P4V2_ICSD_78510	1.04 eV	1.52 eV
Hg1O1_ICSD_24062	1.25 eV	1.24 eV
Hg1O1_ICSD_32561	1.18 eV	1.21 eV
Hg1O1_ICSD_40316	1.20 eV	1.21 eV
Hg1O1_ICSD_639125	1.24 eV	1.24 eV
Hg1O2Rb2_ICSD_25514	2.01 eV	1.82 eV
Hg1O2Sr1_ICSD_165068	2.18 eV	2.10 eV
Hg1O2Sr1_ICSD_69739	2.18 eV	1.59 eV
Hg1O2_ICSD_24774	0.32 eV	0.56 eV
Hg1O2_ICSD_655816	0.27 eV	0.61 eV
Hg1O3Se1_ICSD_412547	2.59 eV	2.34 eV
Hg1O3Se1_ICSD_79694	2.48 eV	2.39 eV
Hg1O3Te1_ICSD_61673	2.25 eV	1.71 eV
Hg1O3Ti1_ICSD_19005	1.37 eV	1.87 eV
Hg1O3V1_ICSD_82242	2.09 eV	1.74 eV
Hg1O4Re1_ICSD_74885	2.76 eV	2.40 eV
Hg1O4S1_ICSD_28402	1.35 eV	1.75 eV
Hg1O4S1_ICSD_31870	1.35 eV	1.63 eV
Hg1O4Se1_ICSD_412403	0.83 eV	1.43 eV
Hg1O4W1_ICSD_169667	2.27 eV	1.69 eV
Hg1O4W1_ICSD_169671	0.82 eV	1.82 eV
Hg1O6V2_ICSD_26996	2.01 eV	1.77 eV
Hg1O7P2Pd1_ICSD_420533	2.12 eV	1.82 eV
Hg1O8P2Pd2_ICSD_420532	1.65 eV	1.81 eV
Hg1Rb1Sb1Se3_ICSD_89018	1.06 eV	1.16 eV
Hg1Rb2Sn1Te4_ICSD_262390	0.53 eV	0.80 eV
Hg1Rb6S4_ICSD_639158	1.60 eV	1.78 eV
Hg1Rb6Se4_ICSD_639159	1.35 eV	1.53 eV
Hg1S1_ICSD_169614	1.52 eV	1.42 eV
Hg1S1_ICSD_81923	1.43 eV	1.44 eV
Hg1Se1_ICSD_639202	0.59 eV	0.75 eV
Hg1Se4Si1Tl2_ICSD_247763	0.92 eV	0.93 eV
Hg1Se4Sn1Tl2_ICSD_247764	0.58 eV	0.64 eV
Hg1Te1_ICSD_162603	0.26 eV	0.24 eV
Hg1Te1_ICSD_639251	0.24 eV	0.25 eV
Hg2I1Na1O2_ICSD_36110	1.38 eV	1.59 eV
Hg2I1O1_ICSD_33275	0.91 eV	1.38 eV
Hg2I2Pb1S2_ICSD_59204	1.66 eV	1.38 eV
Hg2I2_ICSD_36189	1.63 eV	1.57 eV

Supplementary Table 818. Five-fold cross validated predictions for the band gap energy (212/277).

system	calculated	predicted
Hg2Mo1O4_ICSD_90083	1.98 eV	1.89 eV
Hg2Mo1O4_ICSD_90084	1.88 eV	1.93 eV
Hg2Mo2O7_ICSD_51511	2.14 eV	2.03 eV
Hg2Mo2O7_ICSD_83374	2.17 eV	2.10 eV
Hg2N2O4_ICSD_60055	2.04 eV	1.75 eV
Hg2N2O6_ICSD_412933	2.58 eV	2.14 eV
Hg2N6_ICSD_98661	2.16 eV	1.83 eV
Hg2Nb2O7_ICSD_22226	0.53 eV	1.64 eV
Hg2O3Se1_ICSD_412303	2.30 eV	2.10 eV
Hg2O3Se1_ICSD_412304	2.35 eV	2.05 eV
Hg2O4S1_ICSD_15005	1.96 eV	1.49 eV
Hg2O4Se1_ICSD_15006	1.36 eV	1.72 eV
Hg2O4W1_ICSD_90085	1.94 eV	2.12 eV
Hg2O5Te1_ICSD_98671	0.52 eV	1.22 eV
Hg2O7P2_ICSD_59280	1.48 eV	1.95 eV
Hg2O7V2_ICSD_2517	1.33 eV	1.54 eV
Hg2P2S6_ICSD_2564	1.75 eV	1.59 eV
Hg2P2S7_ICSD_2490	1.80 eV	1.61 eV
Hg2P2Se6_ICSD_639132	1.12 eV	1.20 eV
Hg3I2S2_ICSD_411154	1.31 eV	1.43 eV
Hg3I2Se2_ICSD_99093	1.08 eV	1.24 eV
Hg3I2Te2_ICSD_65806	1.35 eV	1.21 eV
Hg3Na2S4_ICSD_71597	1.33 eV	1.54 eV
Hg3O10Se3_ICSD_280914	1.90 eV	2.20 eV
Hg3O5Se4_ICSD_418001	1.89 eV	1.57 eV
Hg3O6S1_ICSD_24147	0.53 eV	0.64 eV
Hg3O6S1_ICSD_96753	0.58 eV	0.60 eV
Hg3O6Te1_ICSD_30325	0.60 eV	1.00 eV
Hg3O6Te1_ICSD_98672	1.14 eV	0.78 eV
Hg3Rb2S8Sn2_ICSD_85599	1.56 eV	1.42 eV
Hg3S4Tl2_ICSD_182745	1.61 eV	1.29 eV
Hg3Se4Tl2_ICSD_182746	1.06 eV	0.81 eV
Hg3Te4Tl2_ICSD_182747	0.47 eV	0.47 eV
Hg4I3Sb2_ICSD_80588	0.73 eV	0.82 eV
Hg4N2O8_ICSD_59156	1.54 eV	1.75 eV
Hg4S6Si1_ICSD_95227	0.43 eV	1.11 eV
Hg6O7Si2_ICSD_69123	1.50 eV	1.22 eV
Hg6O8P2_ICSD_410760	0.71 eV	1.43 eV
Hg7O10P2_ICSD_411300	1.50 eV	1.35 eV
Hg9O16P4_ICSD_411299	1.86 eV	1.33 eV
Ho1I1S1_ICSD_425285	2.71 eV	2.55 eV
Ho1K1O8W2_ICSD_182626	3.50 eV	3.31 eV
Ho1K1S2_ICSD_44947	2.35 eV	2.29 eV
Ho1Li1O2_ICSD_109172	4.02 eV	4.14 eV
Ho1Li1S2_ICSD_639371	1.97 eV	1.96 eV
Ho1Li1Se2_ICSD_44965	1.57 eV	1.52 eV
Ho1Mn1O3_ICSD_182632	0.16 eV	0.74 eV
Ho1Mn2O5_ICSD_84354	1.07 eV	0.97 eV
Ho1N1_ICSD_169207	0.17 eV	0.17 eV
Ho1Na1S2_ICSD_56229	2.32 eV	2.27 eV

Supplementary Table 819. Five-fold cross validated predictions for the band gap energy (213/277).

system	calculated	predicted
Ho1Na1Se2_ICSD_639444	1.87 eV	1.72 eV
Ho1Nb1O4_ICSD_73392	4.19 eV	3.56 eV
Ho1Ni1Sb1_ICSD_639501	0.14 eV	0.19 eV
Ho1O2Rb1_ICSD_15160	3.62 eV	3.50 eV
Ho1O3V1_ICSD_10018	0.89 eV	1.04 eV
Ho1O4P1_ICSD_246677	5.91 eV	5.46 eV
Ho1O4Ta1_ICSD_415436	4.21 eV	4.21 eV
Ho1O4Ta1_ICSD_79499	4.30 eV	3.86 eV
Ho1O4V1_ICSD_78079	2.96 eV	2.87 eV
Ho1O6Rh1Sr3_ICSD_51056	2.84 eV	2.79 eV
Ho1P1S1_ICSD_639545	0.21 eV	0.32 eV
Ho1P5_ICSD_409185	0.14 eV	0.24 eV
Ho1Pd1Sb1_ICSD_108547	0.55 eV	0.24 eV
Ho1Rb1S2_ICSD_81403	2.33 eV	2.29 eV
Ho1Rb1Se2_ICSD_281074	1.96 eV	1.85 eV
Ho1S2Tl1_ICSD_639673	1.47 eV	1.44 eV
Ho1Se2Tl1_ICSD_639712	1.25 eV	1.13 eV
Ho1Te2Tl1_ICSD_639771	0.63 eV	0.64 eV
Ho2Mg1Se4_ICSD_639387	1.32 eV	1.29 eV
Ho2O2S1_ICSD_109333	3.04 eV	2.90 eV
Ho2O2Se1_ICSD_25809	2.14 eV	1.96 eV
Ho2O3_ICSD_160214	4.26 eV	4.27 eV
Ho2O3_ICSD_41268	3.95 eV	4.00 eV
Ho2O3_ICSD_420710	4.06 eV	4.08 eV
Ho2O7Ru2_ICSD_96730	0.55 eV	0.51 eV
Ho2O7Sn2_ICSD_84748	2.20 eV	2.30 eV
Ho2Pb1S4_ICSD_639552	2.25 eV	1.73 eV
Ho2S3_ICSD_22252	1.46 eV	1.27 eV
Ho2S3_ICSD_72291	0.61 eV	0.86 eV
Ho2S4Sr1_ICSD_639669	1.82 eV	1.65 eV
Ho2S4Zn1_ICSD_639680	2.40 eV	2.29 eV
Ho2Se4Yb1_ICSD_639718	1.10 eV	1.02 eV
Ho3O3Sb1_ICSD_380458	0.36 eV	0.76 eV
Ho3O6Sc1_ICSD_23480	4.38 eV	3.35 eV
Ho3O7Re1_ICSD_245106	0.52 eV	0.74 eV
Ho3O7Ta1_ICSD_55656	3.63 eV	3.34 eV
Ho3Pr1S6_ICSD_639588	1.00 eV	1.06 eV
Ho6N2Se6_ICSD_420157	1.53 eV	1.29 eV
Ho6O12W1_ICSD_92038	3.05 eV	3.16 eV
I10Ir1Tb6_ICSD_424438	0.12 eV	0.51 eV
I10Ir2Te16_ICSD_425201	1.00 eV	0.86 eV
I10La6Os1_ICSD_409817	0.20 eV	0.49 eV
I10Pt1Y6_ICSD_424468	0.61 eV	0.46 eV
I10Ru1Y6_ICSD_74645	0.20 eV	0.28 eV
I12K4O1Ti4_ICSD_413504	0.26 eV	1.50 eV
I12Nb2Te8_ICSD_78371	0.88 eV	1.24 eV
I12Ru2Te14_ICSD_425196	0.93 eV	0.94 eV
I14Mo6Pb1_ICSD_36574	1.99 eV	1.75 eV
I15La10Os2_ICSD_83856	0.21 eV	0.32 eV
I1In1Te1_ICSD_100703	1.74 eV	1.52 eV

Supplementary Table 820. Five-fold cross validated predictions for the band gap energy (214/277).

system	calculated	predicted
I1In1_ICSD_55184	1.32 eV	1.32 eV
I1In3Te3_ICSD_66032	1.18 eV	1.04 eV
I1K1Ni1O6_ICSD_60313	1.70 eV	1.94 eV
I1K1O3_ICSD_423584	2.86 eV	2.69 eV
I1K1O3_ICSD_424864	2.87 eV	2.82 eV
I1K1O3_ICSD_9329	3.00 eV	2.87 eV
I1K1O3_ICSD_97995	2.46 eV	2.59 eV
I1K1O4_ICSD_83377	2.31 eV	2.61 eV
I1K1_ICSD_22158	3.86 eV	3.54 eV
I1K1_ICSD_61555	3.29 eV	3.46 eV
I1K3O1_ICSD_36512	1.04 eV	1.08 eV
I1K3O5_ICSD_4325	1.26 eV	2.29 eV
I1La1O1_ICSD_24613	3.75 eV	2.91 eV
I1La3S8Si2_ICSD_280940	2.67 eV	2.60 eV
I1Li1Mo1O6_ICSD_156006	2.18 eV	2.23 eV
I1Li1O3_ICSD_20032	3.59 eV	3.50 eV
I1Li1O3_ICSD_2642	3.70 eV	3.50 eV
I1Li1O3_ICSD_40364	3.65 eV	3.49 eV
I1Li1O3_ICSD_41199	3.67 eV	3.49 eV
I1Li1O4_ICSD_400552	2.19 eV	3.06 eV
I1Li1_ICSD_414242	4.38 eV	3.90 eV
I1Li1_ICSD_44275	4.24 eV	4.01 eV
I1Li1_ICSD_44781	4.22 eV	3.42 eV
I1Li6P1S5_ICSD_421083	2.29 eV	2.51 eV
I1Lu1O1_ICSD_240948	3.25 eV	2.80 eV
I1Mn1Sb1Se2_ICSD_281558	1.01 eV	1.02 eV
I1N1Sr2_ICSD_172602	1.79 eV	1.82 eV
I1N1Ti1_ICSD_27394	0.55 eV	1.14 eV
I1N1Zn2_ICSD_425735	1.86 eV	1.65 eV
I1N1Zr1_ICSD_27392	1.23 eV	1.27 eV
I1N1Zr1_ICSD_51772	0.74 eV	1.17 eV
I1N3Rb2_ICSD_202077	3.94 eV	2.76 eV
I1Na1Ni1O6_ICSD_23543	1.64 eV	2.01 eV
I1Na1O3_ICSD_20168	3.69 eV	3.33 eV
I1Na1O4_ICSD_14287	1.86 eV	2.59 eV
I1Na1_ICSD_52240	3.62 eV	3.46 eV
I1Nb1O2_ICSD_418061	0.76 eV	1.04 eV
I1Nb1Se1_ICSD_201283	0.20 eV	0.76 eV
I1Nb2Te6_ICSD_71516	0.68 eV	0.63 eV
I1O1Sc1_ICSD_419335	2.39 eV	2.54 eV
I1O1Y1_ICSD_0	3.41 eV	3.10 eV
I1O3Rb1_ICSD_2825	2.84 eV	2.72 eV
I1O3Tl1_ICSD_62106	2.80 eV	2.37 eV
I1O4Rb1_ICSD_89510	2.44 eV	2.60 eV
I1O4Tl1_ICSD_52342	2.03 eV	2.53 eV
I1O5Rb3_ICSD_4326	1.88 eV	2.33 eV
I1O6Rb1Sn1_ICSD_73614	2.03 eV	2.21 eV
I1O7Sb5_ICSD_146	2.57 eV	2.51 eV
I1O7Sb5_ICSD_62369	2.44 eV	2.41 eV
I1P3Se4_ICSD_62673	1.67 eV	1.75 eV

Supplementary Table 821. Five-fold cross validated predictions for the band gap energy (215/277).

system	calculated	predicted
I1Rb1_ICSD_53831	3.79 eV	3.45 eV
I1Rb1_ICSD_61523	3.35 eV	2.97 eV
I1S1Sb1_ICSD_25572	0.41 eV	1.15 eV
I1S1Sb1_ICSD_28264	1.63 eV	1.55 eV
I1S1Sb1_ICSD_28265	1.38 eV	1.08 eV
I1S1Sb1_ICSD_28305	1.01 eV	1.26 eV
I1S1Sb1_ICSD_41589	1.63 eV	1.56 eV
I1S1Sb1_ICSD_85298	0.72 eV	1.04 eV
I1S1Sb1_ICSD_85299	1.53 eV	1.54 eV
I1S8Si2Tb3_ICSD_411784	2.45 eV	2.23 eV
I1Sb1Se1_ICSD_35470	1.37 eV	1.25 eV
I1Sb1Te1_ICSD_35471	0.86 eV	0.83 eV
I1Se2Tl5_ICSD_152031	0.66 eV	0.66 eV
I1Ta1Te4_ICSD_67533	0.42 eV	0.79 eV
I1Te1_ICSD_108	0.81 eV	0.87 eV
I1Te1_ICSD_109	0.75 eV	0.92 eV
I1Tl1_ICSD_55194	2.11 eV	1.98 eV
I1Tl1_ICSD_60491	2.15 eV	2.08 eV
I1Tl1_ICSD_61520	1.79 eV	1.69 eV
I2K4O9_ICSD_25694	1.92 eV	2.39 eV
I2La2O4Si1_ICSD_72653	3.51 eV	2.92 eV
I2Mg1_ICSD_281551	3.60 eV	3.36 eV
I2Mn1_ICSD_33673	2.68 eV	2.59 eV
I2N2S1_ICSD_72363	1.50 eV	1.85 eV
I2Na4O1_ICSD_67112	2.28 eV	2.06 eV
I2Nb1O1_ICSD_36255	0.93 eV	0.93 eV
I2Ni1_ICSD_22108	1.35 eV	1.66 eV
I2O1Sr2_ICSD_409896	3.28 eV	3.01 eV
I2O1Ta1_ICSD_80109	1.11 eV	1.23 eV
I2O2Pb3_ICSD_201857	2.27 eV	2.20 eV
I2O4_ICSD_182670	1.48 eV	2.20 eV
I2O5_ICSD_182671	2.63 eV	2.20 eV
I2O6Pb1_ICSD_74493	2.70 eV	2.74 eV
I2O6Pb3Se2_ICSD_422641	2.72 eV	2.55 eV
I2O6S1_ICSD_6220	1.89 eV	2.41 eV
I2O6Zn1_ICSD_54086	3.19 eV	3.29 eV
I2O7Ti1_ICSD_424632	2.63 eV	2.84 eV
I2O8Rb1V1_ICSD_281304	2.71 eV	2.01 eV
I2O8U1_ICSD_92833	2.82 eV	2.60 eV
I2P4S3_ICSD_26219	1.91 eV	2.12 eV
I2P4S3_ICSD_26485	2.22 eV	1.92 eV
I2P4Se3_ICSD_14281	1.71 eV	1.82 eV
I2P4Se3_ICSD_36365	2.08 eV	1.80 eV
I2Pb1_ICSD_108914	2.41 eV	2.29 eV
I2Pb1_ICSD_23762	2.40 eV	2.36 eV
I2Pb1_ICSD_23763	2.38 eV	2.38 eV
I2Pb1_ICSD_23764	2.36 eV	2.37 eV
I2Pb1_ICSD_24263	2.35 eV	2.36 eV
I2Pb1_ICSD_24264	2.40 eV	2.32 eV
I2Pb1_ICSD_24265	2.37 eV	2.43 eV

Supplementary Table 822. Five-fold cross validated predictions for the band gap energy (216/277).

system	calculated	predicted
I2Pb1_ICSD_60186	2.35 eV	2.38 eV
I2Pb1_ICSD_60327	2.38 eV	2.39 eV
I2Pb1_ICSD_77324	2.29 eV	2.25 eV
I2Pd1Te1_ICSD_409062	0.67 eV	1.09 eV
I2Pd1_ICSD_23169	1.19 eV	1.12 eV
I2Pd1_ICSD_25120	1.19 eV	1.17 eV
I2Pt1_ICSD_60760	1.51 eV	1.03 eV
I2Sn1_ICSD_2831	1.70 eV	1.55 eV
I2Sr1_ICSD_203137	3.80 eV	3.69 eV
I2Sr1_ICSD_37050	3.79 eV	3.65 eV
I2Te2Th1_ICSD_94402	1.05 eV	1.07 eV
I2Tm1_ICSD_43731	2.57 eV	2.22 eV
I2V1_ICSD_246907	2.44 eV	1.65 eV
I2Yb1_ICSD_77907	3.25 eV	2.65 eV
I2Zn1_ICSD_77058	1.95 eV	2.70 eV
I2Zr1_ICSD_24807	0.28 eV	0.43 eV
I2Zr1_ICSD_26418	0.27 eV	0.35 eV
I2_ICSD_20329	0.42 eV	0.83 eV
I2_ICSD_67616	0.99 eV	0.68 eV
I3In1O9_ICSD_154654	3.44 eV	2.93 eV
I3In1O9_ICSD_250450	3.14 eV	2.86 eV
I3Mn1Tl1_ICSD_26420	2.30 eV	2.21 eV
I3Na1O8_ICSD_418336	3.15 eV	2.74 eV
I3Nb1O1_ICSD_418088	0.76 eV	0.98 eV
I3O13Rb1V2_ICSD_281306	1.73 eV	2.13 eV
I3O1W1_ICSD_65183	0.56 eV	1.07 eV
I3O9Sc1_ICSD_411945	3.91 eV	3.03 eV
I3O9Tl1_ICSD_247059	2.21 eV	2.40 eV
I3P1_ICSD_311	2.25 eV	2.09 eV
I3Pb1Tl1_ICSD_1199	2.12 eV	2.06 eV
I3Pb2S2Sb1_ICSD_418344	1.77 eV	1.31 eV
I3Rb1Sn1_ICSD_400934	2.01 eV	1.55 eV
I3Rb1Yb1_ICSD_86314	3.12 eV	2.77 eV
I3Rb1_ICSD_61348	1.84 eV	1.47 eV
I3S2Sb1Sn2_ICSD_23344	0.63 eV	1.03 eV
I3Sb1_ICSD_30906	2.14 eV	2.13 eV
I3Sb1_ICSD_56572	2.12 eV	2.15 eV
I3Ti1_ICSD_23172	1.85 eV	1.86 eV
I3Tl1_ICSD_61349	1.65 eV	1.81 eV
I3U1_ICSD_1851	0.64 eV	1.46 eV
I3U1_ICSD_201171	1.61 eV	1.16 eV
I3Y1_ICSD_170773	3.03 eV	2.26 eV
I4In1Li1_ICSD_36599	2.37 eV	2.37 eV
I4In1Na1_ICSD_65463	2.47 eV	2.37 eV
I4In2_ICSD_46034	1.76 eV	1.99 eV
I4Li2Zn1_ICSD_402062	3.40 eV	3.40 eV
I4Mg2O12_ICSD_415820	3.47 eV	3.39 eV
I4Mn2O12_ICSD_415822	2.56 eV	2.41 eV
I4Nb1_ICSD_23916	0.59 eV	1.12 eV
I4O12Zn2_ICSD_415821	3.27 eV	3.07 eV

Supplementary Table 823. Five-fold cross validated predictions for the band gap energy (217/277).

system	calculated	predicted
I4O12Zr1_ICSD_15405	3.17 eV	3.08 eV
I4O12_ICSD_80196	1.91 eV	2.18 eV
I4O1Te9Ti4_ICSD_82142	0.95 eV	0.91 eV
I4O20Tl2U3_ICSD_93862	2.70 eV	2.68 eV
I4P2_ICSD_36293	1.92 eV	1.99 eV
I4Pt1_ICSD_15173	0.81 eV	0.72 eV
I4Rb2Zn1_ICSD_56425	3.27 eV	3.10 eV
I4S1Tl6_ICSD_29265	1.76 eV	1.51 eV
I4Se1Tl6_ICSD_40520	1.62 eV	1.31 eV
I4Si1_ICSD_91745	2.81 eV	2.64 eV
I4Sn1_ICSD_18010	1.49 eV	1.94 eV
I4Sn1_ICSD_38140	1.79 eV	2.03 eV
I4Ta1_ICSD_391406	0.75 eV	0.95 eV
I4Te1_ICSD_37226	1.76 eV	1.49 eV
I4Ti1_ICSD_39820	1.44 eV	1.81 eV
I4Tl2Zn1_ICSD_37099	2.98 eV	2.18 eV
I4U1_ICSD_16464	1.67 eV	1.70 eV
I4Zr1_ICSD_62243	2.06 eV	1.64 eV
I4Zr1_ICSD_8068	2.02 eV	1.14 eV
I5In1Pb2_ICSD_152000	1.84 eV	2.01 eV
I5In1Sn2_ICSD_151995	1.42 eV	1.66 eV
I5In1Sr2_ICSD_152022	2.77 eV	2.58 eV
I5In3Sn1_ICSD_74762	1.99 eV	1.98 eV
I5K1Sn2_ICSD_152010	1.50 eV	1.83 eV
I5K2U1_ICSD_41550	2.55 eV	2.01 eV
I5Nb1_ICSD_10457	1.08 eV	0.91 eV
I5Nb1_ICSD_25503	0.75 eV	1.00 eV
I5Pb1Tl3_ICSD_85309	2.60 eV	2.34 eV
I6In1O18Rb3_ICSD_422058	3.02 eV	2.81 eV
I6In2_ICSD_23136	2.30 eV	2.05 eV
I6K2Pt1_ICSD_37190	0.73 eV	1.16 eV
I6K2Tc1_ICSD_65510	1.25 eV	1.31 eV
I6Mo1O18Rb2_ICSD_413880	1.06 eV	2.41 eV
I6Na2U1_ICSD_78683	2.09 eV	2.29 eV
I6Nb2Se2_ICSD_410743	0.89 eV	0.87 eV
I6Nb2Te2_ICSD_35377	1.03 eV	1.17 eV
I6O18Rb2Zr1_ICSD_414091	3.38 eV	2.93 eV
I6O18Tl4_ICSD_247060	1.93 eV	2.43 eV
I6O1Sr4_ICSD_280585	3.58 eV	3.41 eV
I6Pb1Th1_ICSD_73139	2.20 eV	1.98 eV
I6Pb1Tl4_ICSD_100069	2.29 eV	2.27 eV
I6Pb5S2_ICSD_21040	1.85 eV	1.39 eV
I6Pt1Rb2_ICSD_37191	0.44 eV	0.68 eV
I6Pt2_ICSD_47120	0.35 eV	1.00 eV
I6Rb2Te1_ICSD_36009	1.50 eV	1.65 eV
I6Rb4Yb1_ICSD_72344	3.05 eV	2.89 eV
I6S1Sn4_ICSD_12165	1.39 eV	1.74 eV
I6Si2_ICSD_83643	2.83 eV	2.56 eV
I6Sn1Th1_ICSD_73138	1.80 eV	2.03 eV
I6Te1Tl2_ICSD_99128	1.63 eV	1.84 eV

Supplementary Table 824. Five-fold cross validated predictions for the band gap energy (218/277).

system	calculated	predicted
I7In1Te1_ICSD_407981	1.80 eV	1.88 eV
I7Nb3Te1_ICSD_86723	0.67 eV	0.62 eV
I7Nb3Te1_ICSD_86724	0.65 eV	0.69 eV
I7Se1Ta3_ICSD_82791	0.70 eV	0.75 eV
I7Ta3Te1_ICSD_82792	0.69 eV	0.71 eV
I8Nb3_ICSD_25767	0.23 eV	0.48 eV
I8Pt3_ICSD_60761	0.84 eV	0.85 eV
I9Re3_ICSD_25114	0.87 eV	0.85 eV
In1K1O2_ICSD_380401	2.21 eV	1.79 eV
In1K1O8W2_ICSD_10187	4.47 eV	3.70 eV
In1K1P2S7_ICSD_91755	2.13 eV	2.05 eV
In1K1Te2_ICSD_25347	1.02 eV	0.88 eV
In1K2Na1Sb2_ICSD_77181	0.60 eV	0.67 eV
In1K2P2S7_ICSD_248034	1.93 eV	2.05 eV
In1K3P2_ICSD_73281	0.79 eV	0.84 eV
In1La1O3_ICSD_281549	2.82 eV	2.31 eV
In1Li1Mg1Mo3O12_ICSD_63519	3.14 eV	3.11 eV
In1Li1Mo2O8_ICSD_91269	2.78 eV	2.71 eV
In1Li1Mo3O8_ICSD_30579	1.80 eV	1.71 eV
In1Li1O2_ICSD_639886	2.01 eV	1.92 eV
In1Li1O6Si2_ICSD_55178	3.73 eV	3.47 eV
In1Li1O7P2_ICSD_60935	4.09 eV	3.84 eV
In1Li1O8W2_ICSD_423127	3.17 eV	2.99 eV
In1Li1Se2_ICSD_56531	0.89 eV	1.08 eV
In1Li1Se2_ICSD_56532	1.63 eV	1.51 eV
In1Li1Se2_ICSD_656944	1.68 eV	1.59 eV
In1Li1Te2_ICSD_639906	1.42 eV	0.98 eV
In1Mn1Ti2_ICSD_189700	0.21 eV	0.28 eV
In1Mo2O8Rb1_ICSD_10186	3.95 eV	3.35 eV
In1Na1O2_ICSD_34600	2.07 eV	1.87 eV
In1Na1O6Si2_ICSD_183781	3.62 eV	3.54 eV
In1Na1O8W2_ICSD_16263	3.25 eV	3.27 eV
In1Na1S2_ICSD_640037	1.92 eV	1.82 eV
In1Na1Se2_ICSD_25558	1.10 eV	1.27 eV
In1Na1Te2_ICSD_25346	0.66 eV	0.71 eV
In1Na3O4Rb2_ICSD_33868	1.98 eV	2.05 eV
In1Na3O8P2_ICSD_87833	3.39 eV	3.57 eV
In1Na5O4_ICSD_69630	1.84 eV	1.79 eV
In1Na5S4_ICSD_300175	2.06 eV	2.06 eV
In1O3Y1_ICSD_251	2.16 eV	2.58 eV
In1O4P1_ICSD_16618	2.49 eV	2.71 eV
In1O4P1_ICSD_85579	2.88 eV	2.76 eV
In1O4V1_ICSD_155162	3.03 eV	2.60 eV
In1O4V1_ICSD_80360	2.90 eV	2.58 eV
In1O6Rh1Sr3_ICSD_51138	2.19 eV	1.63 eV
In1O6Sr2Ta1_ICSD_188420	4.09 eV	3.35 eV
In1O8Rb1W2_ICSD_24859	4.46 eV	4.07 eV
In1O8Se2V1_ICSD_261603	2.16 eV	2.13 eV
In1O9P3_ICSD_421261	4.53 eV	3.92 eV
In1P1S4_ICSD_1699	2.42 eV	1.75 eV

Supplementary Table 825. Five-fold cross validated predictions for the band gap energy (219/277).

system	calculated	predicted
In1P1_ICSD_180911	0.63 eV	0.67 eV
In1P1_ICSD_600858	0.58 eV	0.65 eV
In1P2Se6Tl1_ICSD_171212	1.47 eV	1.31 eV
In1P3Sr3_ICSD_65056	0.84 eV	0.68 eV
In1Rb1Te2_ICSD_75346	1.15 eV	1.02 eV
In1S1_ICSD_409645	0.20 eV	0.73 eV
In1S1_ICSD_640349	1.32 eV	0.69 eV
In1S2Tl1_ICSD_108557	0.69 eV	0.73 eV
In1S2Tl1_ICSD_600748	0.93 eV	0.95 eV
In1S2Tl1_ICSD_600863	1.38 eV	1.20 eV
In1S2Tl1_ICSD_640393	0.12 eV	0.38 eV
In1S2Tl1_ICSD_654688	0.46 eV	0.52 eV
In1S3Sb1_ICSD_300207	1.49 eV	1.19 eV
In1Se1_ICSD_640490	0.43 eV	0.32 eV
In1Se1_ICSD_640503	0.61 eV	0.37 eV
In1Se1_ICSD_640505	0.35 eV	0.53 eV
In1Se1_ICSD_71083	0.99 eV	0.44 eV
In1Se2Tl1_ICSD_600818	0.68 eV	0.49 eV
In1Si1Te3_ICSD_657680	0.53 eV	0.75 eV
In1Te2Tl1_ICSD_600697	0.53 eV	0.52 eV
In2Li2S6Si1_ICSD_262642	2.19 eV	2.23 eV
In2Li2Se6Si1_ICSD_262644	1.52 eV	1.39 eV
In2Mg1O4_ICSD_157770	1.99 eV	1.94 eV
In2Mg1S4_ICSD_53096	1.88 eV	1.73 eV
In2Mn1S4_ICSD_639973	1.48 eV	0.93 eV
In2Mn1Te4_ICSD_60053	0.51 eV	0.53 eV
In2Mn1Te4_ICSD_639984	0.46 eV	0.55 eV
In2Mn2O7_ICSD_56517	1.29 eV	1.21 eV
In2Na6S6_ICSD_300165	2.33 eV	2.08 eV
In2O3_ICSD_169429	1.12 eV	1.04 eV
In2O3_ICSD_181834	0.96 eV	0.80 eV
In2O3_ICSD_181836	0.44 eV	0.94 eV
In2O3_ICSD_24325	1.16 eV	1.04 eV
In2O5P1_ICSD_413858	2.11 eV	2.32 eV
In2O6Te1_ICSD_60260	1.13 eV	1.75 eV
In2O7Si2_ICSD_1458	1.94 eV	2.41 eV
In2O7Si2_ICSD_74897	2.82 eV	2.68 eV
In2P2Sr1_ICSD_260563	0.24 eV	0.44 eV
In2P4Sr3_ICSD_61335	0.54 eV	0.62 eV
In2Pb1S4_ICSD_100687	1.43 eV	0.98 eV
In2Rb4S5_ICSD_23253	2.14 eV	1.97 eV
In2Rb6S6_ICSD_23252	2.29 eV	1.96 eV
In2S3_ICSD_12148	0.40 eV	0.90 eV
In2S3_ICSD_23844	0.92 eV	1.01 eV
In2S4Zn1_ICSD_16200	0.40 eV	0.43 eV
In2S4Zn1_ICSD_44637	0.40 eV	0.50 eV
In2S4Zn1_ICSD_603024	0.48 eV	0.72 eV
In2S4Zn1_ICSD_81811	1.40 eV	0.99 eV
In2S7Sn3_ICSD_68366	0.46 eV	0.80 eV
In2Sb6Sr5_ICSD_36468	0.19 eV	0.22 eV

Supplementary Table 826. Five-fold cross validated predictions for the band gap energy (220/277).

system	calculated	predicted
In2Se3_ICSD_108563	0.49 eV	0.91 eV
In2Se3_ICSD_1376	1.07 eV	0.66 eV
In2Se3_ICSD_602266	0.17 eV	0.43 eV
In2Se4Yb1_ICSD_640533	1.33 eV	1.07 eV
In2Se4Zn1_ICSD_25647	1.13 eV	0.97 eV
In2Sr1Te4_ICSD_41167	0.91 eV	0.75 eV
In2Te4Zn1_ICSD_25650	0.93 eV	0.85 eV
In2Te5_ICSD_640615	0.84 eV	0.51 eV
In3Na7Se8_ICSD_300178	1.23 eV	1.58 eV
In3O12Sb5_ICSD_68240	2.41 eV	1.94 eV
In3O8P2_ICSD_66831	3.10 eV	2.95 eV
In3Rb1S5_ICSD_59667	1.28 eV	1.38 eV
In3Ru1_ICSD_59518	0.18 eV	0.18 eV
In3Ru1_ICSD_640343	0.18 eV	0.18 eV
In3S5Tl1_ICSD_402124	1.77 eV	1.00 eV
In4O7Rb2_ICSD_6321	1.62 eV	1.63 eV
In4P8Rb12_ICSD_402811	0.48 eV	0.77 eV
In4S4Sn1_ICSD_36513	1.16 eV	1.08 eV
In4Se3_ICSD_425490	0.19 eV	0.35 eV
In4Se4Sn1_ICSD_56324	0.84 eV	0.82 eV
In4Te3_ICSD_79121	0.17 eV	0.49 eV
In5K1S8_ICSD_40875	1.36 eV	1.42 eV
In5Rb1S8_ICSD_40877	1.36 eV	1.44 eV
In5S6Tl1_ICSD_56388	1.02 eV	0.93 eV
In5S7Tl1_ICSD_402125	0.76 eV	0.88 eV
In5Se7Tl1_ICSD_402126	0.36 eV	0.46 eV
In6O12Re1_ICSD_245005	0.48 eV	1.30 eV
In6O12Te1_ICSD_245526	1.15 eV	1.43 eV
In6O12W1_ICSD_27197	1.96 eV	2.12 eV
In6S7_ICSD_640348	0.33 eV	0.60 eV
In6Se7_ICSD_640500	0.20 eV	0.23 eV
In6Se7_ICSD_9135	0.16 eV	0.27 eV
In7Te10_ICSD_400669	0.90 eV	0.78 eV
Ir1K3Li1O4_ICSD_75045	1.14 eV	1.34 eV
Ir1K4O4_ICSD_47223	1.16 eV	1.45 eV
Ir1La3O7_ICSD_181572	0.33 eV	0.48 eV
Ir1Li8O6_ICSD_61217	0.72 eV	1.71 eV
Ir1Mg1O6Sr2_ICSD_77835	0.20 eV	1.01 eV
Ir1N2_ICSD_240755	0.22 eV	0.96 eV
Ir1N6Na3O12_ICSD_39572	2.58 eV	2.45 eV
Ir1Na4O4_ICSD_67826	1.15 eV	1.24 eV
Ir1Ni1O6Sr3_ICSD_80285	0.64 eV	0.95 eV
Ir1O6Sr3Zn1_ICSD_82660	0.29 eV	0.73 eV
Ir1O7Pr3_ICSD_86473	0.22 eV	0.46 eV
Ir1O9P3_ICSD_419717	3.02 eV	2.26 eV
Ir1P1Se1_ICSD_640901	1.51 eV	0.90 eV
Ir1P1Te1_ICSD_601289	0.90 eV	0.63 eV
Ir1P2_ICSD_174231	0.51 eV	0.74 eV
Ir1S1Sb1_ICSD_41400	1.43 eV	1.25 eV
Ir1S1Sb1_ICSD_74630	1.20 eV	1.33 eV

Supplementary Table 827. Five-fold cross validated predictions for the band gap energy (221/277).

system	calculated	predicted
Ir1S2_ICSD_640950	0.60 eV	0.77 eV
Ir1Sb1Se1_ICSD_640966	1.18 eV	0.89 eV
Ir1Sb1Te1_ICSD_640967	0.66 eV	0.71 eV
Ir1Sb2_ICSD_43502	0.35 eV	0.58 eV
Ir1Se2_ICSD_640985	0.11 eV	0.52 eV
Ir2P2Sr1_ICSD_73531	0.44 eV	0.50 eV
Ir2S3_ICSD_640948	1.10 eV	0.82 eV
K0.33Mo1O3_ICSD_1053	0.73 eV	1.53 eV
K1.33Na0.67O5Si2_ICSD_170786	4.18 eV	4.20 eV
K1La1Na1Nb1O5_ICSD_94743	3.40 eV	3.36 eV
K1La1Na1O5Ta1_ICSD_419855	3.46 eV	3.57 eV
K1La1O12P4_ICSD_161016	4.90 eV	4.70 eV
K1La1O12P4_ICSD_33241	5.37 eV	5.30 eV
K1La1O2_ICSD_27001	3.65 eV	3.31 eV
K1La1O3Pd1_ICSD_417108	1.12 eV	1.34 eV
K1La1O4Ti1_ICSD_261365	3.04 eV	3.13 eV
K1La1S2_ICSD_44942	2.41 eV	2.35 eV
K1La1S4Si1_ICSD_414544	2.59 eV	2.62 eV
K1La1S4Si1_ICSD_603184	2.74 eV	2.65 eV
K1La1Se4Si1_ICSD_603185	2.19 eV	1.77 eV
K1La1Te2_ICSD_98654	1.42 eV	1.31 eV
K1La2Nb1O6_ICSD_160450	3.65 eV	3.51 eV
K1Li1Mn1O2_ICSD_49021	1.13 eV	1.49 eV
K1Li1O1_ICSD_30964	2.34 eV	2.65 eV
K1Li1O2Zn1_ICSD_49022	2.69 eV	2.68 eV
K1Li1O4S1_ICSD_56106	5.34 eV	5.32 eV
K1Li1O4S1_ICSD_71365	5.31 eV	5.27 eV
K1Li1O4S1_ICSD_71366	4.37 eV	4.98 eV
K1Li1O4S1_ICSD_71367	5.32 eV	4.97 eV
K1Li1O4S1_ICSD_88832	4.44 eV	4.56 eV
K1Li1O5Si2_ICSD_82457	4.70 eV	4.71 eV
K1Li1S1_ICSD_290450	3.04 eV	2.63 eV
K1Li1Se1_ICSD_290451	2.51 eV	2.26 eV
K1Li1Te1_ICSD_290452	2.43 eV	2.10 eV
K1Li4Nb1O5_ICSD_73124	4.10 eV	3.53 eV
K1Li6O6Ta1_ICSD_73159	4.55 eV	3.97 eV
K1Lu1O8W2_ICSD_155155	3.40 eV	3.14 eV
K1Mg1O9P3_ICSD_28012	4.95 eV	5.16 eV
K1Mg1P1_ICSD_641231	1.68 eV	1.27 eV
K1Mn1Na1O2_ICSD_61403	0.62 eV	1.04 eV
K1Mn1O4P1_ICSD_78840	3.02 eV	2.97 eV
K1Mn1O4_ICSD_44625	2.06 eV	2.10 eV
K1Mn1O8Se2_ICSD_80430	1.17 eV	2.11 eV
K1Mn1Sb1_ICSD_602269	0.29 eV	0.45 eV
K1Mo2O8Sc1_ICSD_28019	3.69 eV	3.43 eV
K1Mo2O8Y1_ICSD_20478	3.48 eV	3.36 eV
K1N1O2_ICSD_36202	2.50 eV	2.59 eV
K1N1O2_ICSD_86117	2.50 eV	2.38 eV
K1N1O3_ICSD_28077	2.91 eV	2.49 eV
K1N1O3_ICSD_71398	2.98 eV	2.64 eV

Supplementary Table 828. Five-fold cross validated predictions for the band gap energy (222/277).

system	calculated	predicted
K1N3_ICSD_155168	4.17 eV	3.96 eV
K1Na1Nb1Nd1O5_ICSD_420038	3.47 eV	3.19 eV
K1Na1Nb1O5Pr1_ICSD_420039	3.15 eV	2.18 eV
K1Na1Nd1O5Ta1_ICSD_419856	3.55 eV	3.54 eV
K1Na1O1_ICSD_32743	2.16 eV	2.00 eV
K1Na1O2Zn1_ICSD_38327	2.19 eV	2.16 eV
K1Na1O3Ti1_ICSD_47177	4.03 eV	3.66 eV
K1Na1O4S1_ICSD_26014	4.99 eV	5.00 eV
K1Na1O4S1_ICSD_77343	5.01 eV	5.08 eV
K1Na1O5Pr1Ta1_ICSD_419857	0.10 eV	2.66 eV
K1Na1O6V2_ICSD_2199	3.24 eV	2.86 eV
K1Na1O7S2_ICSD_413050	5.21 eV	4.69 eV
K1Na1S1_ICSD_62658	2.51 eV	2.28 eV
K1Na1Se1_ICSD_67278	2.05 eV	1.95 eV
K1Na1Te1_ICSD_67276	1.96 eV	1.70 eV
K1Na2Ni1O2_ICSD_79878	1.39 eV	1.67 eV
K1Na2Sb1_ICSD_44332	0.73 eV	0.87 eV
K1Na3O5W1_ICSD_40249	3.68 eV	3.43 eV
K1Nb1Nd2O6_ICSD_160451	3.43 eV	3.50 eV
K1Nb1O3_ICSD_9532	1.73 eV	2.22 eV
K1Nb1O3_ICSD_9533	2.05 eV	2.05 eV
K1Nb1O3_ICSD_9534	2.23 eV	2.14 eV
K1Nb1S2_ICSD_26286	0.83 eV	1.17 eV
K1Nb1Se2_ICSD_26288	0.71 eV	0.90 eV
K1Nd1O12P4_ICSD_4254	4.11 eV	4.33 eV
K1Nd1O3Pd1_ICSD_417107	1.22 eV	1.29 eV
K1Nd1O8S2_ICSD_200268	3.59 eV	3.69 eV
K1Nd1O8W2_ICSD_9364	3.57 eV	3.58 eV
K1Nd1S2_ICSD_641272	2.28 eV	2.28 eV
K1Nd1Te2_ICSD_98656	1.18 eV	1.26 eV
K1Nd1Te4_ICSD_412792	0.18 eV	0.42 eV
K1Ni1O4P1_ICSD_81271	2.99 eV	2.96 eV
K1Ni1O9P3_ICSD_91527	4.48 eV	3.42 eV
K1Ni1P1S4_ICSD_79482	1.96 eV	1.71 eV
K1O12P3Th2_ICSD_30432	5.04 eV	4.81 eV
K1O12P3Zr2_ICSD_4427	4.29 eV	4.16 eV
K1O12P4Pr1_ICSD_86289	4.70 eV	3.86 eV
K1O12P4Y1_ICSD_241230	5.34 eV	5.25 eV
K1O12Se2V3_ICSD_81231	2.34 eV	2.11 eV
K1O12Th2V3_ICSD_8232	3.38 eV	2.89 eV
K1O13P4Ta1_ICSD_20735	3.84 eV	3.91 eV
K1O1Tl1_ICSD_1570	1.17 eV	1.17 eV
K1O2Pr1_ICSD_40266	2.56 eV	2.56 eV
K1O2Sb1_ICSD_411214	2.00 eV	2.28 eV
K1O2Sc1_ICSD_34958	3.95 eV	3.72 eV
K1O2Tl1_ICSD_33553	0.88 eV	1.23 eV
K1O2Y1_ICSD_49650	3.97 eV	3.66 eV
K1O3Pd2_ICSD_248051	1.00 eV	1.29 eV
K1O3Sb1_ICSD_33546	2.67 eV	2.47 eV
K1O3Sb1_ICSD_41203	1.79 eV	1.98 eV

Supplementary Table 829. Five-fold cross validated predictions for the band gap energy (223/277).

system	calculated	predicted
K1O3Sb1_ICSD_77333	1.13 eV	1.89 eV
K1O3Ta1_ICSD_184922	2.32 eV	2.69 eV
K1O3U1_ICSD_71241	3.01 eV	2.71 eV
K1O3V1_ICSD_1486	3.03 eV	2.63 eV
K1O3_ICSD_47163	0.63 eV	1.67 eV
K1O4P1Sr1_ICSD_83598	5.11 eV	4.70 eV
K1O4P1Zn1_ICSD_88955	4.32 eV	3.84 eV
K1O4Pb1V1_ICSD_80816	3.37 eV	2.83 eV
K1O4Re1_ICSD_72501	4.06 eV	3.63 eV
K1O4Ru1_ICSD_26612	0.20 eV	1.75 eV
K1O4Sr1V1_ICSD_404250	3.84 eV	3.73 eV
K1O4Sr1V1_ICSD_91782	3.83 eV	3.62 eV
K1O4Tc1_ICSD_61	3.26 eV	2.56 eV
K1O5Sb3_ICSD_28493	3.12 eV	2.35 eV
K1O7P2Y1_ICSD_75171	5.21 eV	4.94 eV
K1O7Sb1Zn4_ICSD_421546	1.57 eV	2.06 eV
K1O8P2Sb1_ICSD_61788	2.95 eV	3.16 eV
K1O8Pr1S2_ICSD_200543	3.64 eV	3.60 eV
K1O8S2V1_ICSD_59175	1.79 eV	1.60 eV
K1O8V3_ICSD_421064	2.05 eV	2.09 eV
K1O8W2Y1_ICSD_90378	3.50 eV	3.52 eV
K1O8W2Yb1_ICSD_280877	0.18 eV	1.07 eV
K1P1Pb1S4_ICSD_171379	2.37 eV	2.00 eV
K1P1Pd1S4_ICSD_165315	1.81 eV	1.65 eV
K1P1S5Ti1_ICSD_82262	1.60 eV	1.65 eV
K1P1Se5Ti1_ICSD_81297	1.25 eV	1.39 eV
K1P1Zn1_ICSD_12160	0.99 eV	0.93 eV
K1P1_ICSD_14010	1.03 eV	0.97 eV
K1P2S6Sb1_ICSD_409751	2.26 eV	2.25 eV
K1P2S7V1_ICSD_91756	1.24 eV	1.41 eV
K1P2Sb1Se6_ICSD_90152	1.80 eV	1.55 eV
K1P3Zn4_ICSD_262034	0.66 eV	0.89 eV
K1Pr1Se4Si1_ICSD_416622	2.13 eV	1.58 eV
K1Rb1S1_ICSD_72326	2.21 eV	2.21 eV
K1S2Sb1_ICSD_641324	1.60 eV	1.40 eV
K1S2Tb1_ICSD_641329	2.32 eV	2.27 eV
K1S2Ti1_ICSD_641335	1.93 eV	1.49 eV
K1S2Y1_ICSD_641347	2.33 eV	2.21 eV
K1S4Si1Y1_ICSD_602576	2.82 eV	2.60 eV
K1S8V5_ICSD_602318	0.36 eV	0.57 eV
K1Sb1Se2_ICSD_100125	1.42 eV	1.34 eV
K1Sb1Se2_ICSD_44678	1.39 eV	1.45 eV
K1Sb1Sn1_ICSD_40816	0.23 eV	0.35 eV
K1Sb1Zn1_ICSD_12161	0.62 eV	0.47 eV
K1Sb1Zn1_ICSD_44680	0.52 eV	0.62 eV
K1Sb1_ICSD_56529	0.74 eV	0.57 eV
K1Sb2_ICSD_80945	0.20 eV	0.43 eV
K1Se2Sm1_ICSD_602818	0.32 eV	0.83 eV
K1Se8Ti5_ICSD_602313	0.46 eV	0.55 eV
K1Si1_ICSD_641367	1.18 eV	1.21 eV

Supplementary Table 830. Five-fold cross validated predictions for the band gap energy (224/277).

system	calculated	predicted
K1Te2Y1_ICSD_419995	1.31 eV	1.30 eV
K2Li14O14Pb3_ICSD_35250	1.92 eV	1.96 eV
K2Li1O4V1_ICSD_65945	3.96 eV	3.38 eV
K2Li4O6U1_ICSD_65158	3.05 eV	2.94 eV
K2Li6O8Pb2_ICSD_37084	1.49 eV	2.09 eV
K2Mg1P2Se6_ICSD_413168	1.97 eV	1.75 eV
K2Mg2O12S3_ICSD_40986	5.56 eV	5.47 eV
K2Mg2O7Si2_ICSD_185362	4.96 eV	4.23 eV
K2Mn1O4_ICSD_22245	0.47 eV	1.39 eV
K2Mn1O6Se2_ICSD_71540	3.66 eV	2.51 eV
K2Mn1O7V2_ICSD_417892	2.44 eV	2.07 eV
K2Mn1P2S6_ICSD_658727	1.36 eV	1.99 eV
K2Mn1P2Se6_ICSD_165356	1.67 eV	1.60 eV
K2Mn1S2_ICSD_65453	2.06 eV	1.63 eV
K2Mn1Se2_ICSD_65456	1.75 eV	1.58 eV
K2Mn1Te2_ICSD_65459	1.89 eV	1.57 eV
K2Mn2O12S3_ICSD_158733	4.48 eV	4.01 eV
K2Mn2O15Si4Zn4_ICSD_20121	2.00 eV	2.42 eV
K2Mn2O3_ICSD_61038	0.44 eV	1.02 eV
K2Mn3S4_ICSD_411172	1.80 eV	1.35 eV
K2Mo1O1S3_ICSD_423988	1.79 eV	1.87 eV
K2Mo1O2S2_ICSD_423986	2.13 eV	2.21 eV
K2Mo1O2S2_ICSD_423987	2.25 eV	2.31 eV
K2Mo1O3S1_ICSD_423989	2.97 eV	2.33 eV
K2Mo1O4_ICSD_150842	4.21 eV	3.42 eV
K2Mo1S4_ICSD_409563	1.52 eV	1.64 eV
K2Mo2O7_ICSD_20133	3.34 eV	3.38 eV
K2Mo3O10_ICSD_24118	3.28 eV	3.12 eV
K2N2O5S1_ICSD_31031	3.30 eV	2.82 eV
K2N6Ni1O12Sr1_ICSD_179	2.05 eV	2.29 eV
K2Nb5O15Y1_ICSD_82147	1.90 eV	2.23 eV
K2Ni1O2_ICSD_201891	2.34 eV	1.86 eV
K2Ni1P2_ICSD_300119	1.28 eV	1.17 eV
K2Ni2O12S3_ICSD_40987	3.47 eV	3.25 eV
K2Ni3S4_ICSD_641275	0.95 eV	0.96 eV
K2O11Sb4_ICSD_2061	1.85 eV	2.17 eV
K2O12P3Pr1Zr1_ICSD_250163	4.33 eV	4.05 eV
K2O12P3Ti2_ICSD_202888	1.70 eV	2.98 eV
K2O12P4Sr1_ICSD_15725	5.60 eV	5.13 eV
K2O12S3Zn2_ICSD_82873	4.84 eV	4.65 eV
K2O12Te4_ICSD_1728	1.13 eV	1.91 eV
K2O13Ti6_ICSD_184901	3.21 eV	2.99 eV
K2O14Te2U3_ICSD_414064	2.94 eV	2.75 eV
K2O15Si6Ti1_ICSD_158934	3.75 eV	3.74 eV
K2O1_ICSD_180571	1.71 eV	1.55 eV
K2O2Pd1_ICSD_6158	2.04 eV	1.60 eV
K2O2Zn1_ICSD_34603	2.44 eV	2.12 eV
K2O2_ICSD_36641	2.34 eV	2.10 eV
K2O3Pb1_ICSD_15929	1.14 eV	1.52 eV
K2O3Pb1_ICSD_22063	1.25 eV	1.41 eV

Supplementary Table 831. Five-fold cross validated predictions for the band gap energy (225/277).

system	calculated	predicted
K2O3Pb2_ICSD_1412	1.69 eV	1.61 eV
K2O3S1_ICSD_60762	4.91 eV	3.71 eV
K2O3Sn1_ICSD_16265	1.86 eV	2.20 eV
K2O3Sn2_ICSD_2216	1.32 eV	1.45 eV
K2O3Sn2_ICSD_40463	1.34 eV	1.55 eV
K2O3Te1_ICSD_65640	3.58 eV	3.09 eV
K2O3Ti1_ICSD_162216	3.77 eV	3.71 eV
K2O3Zr1_ICSD_16264	3.62 eV	3.57 eV
K2O4Ru1_ICSD_415749	1.27 eV	1.27 eV
K2O4U1_ICSD_380361	2.86 eV	2.49 eV
K2O4W1_ICSD_26181	4.47 eV	3.94 eV
K2O4Zn3_ICSD_62146	2.09 eV	1.93 eV
K2O5S2_ICSD_16701	3.21 eV	4.30 eV
K2O5Ti2_ICSD_36097	3.73 eV	3.16 eV
K2O5Zr2_ICSD_18301	3.81 eV	3.52 eV
K2O6S2Se1_ICSD_25027	2.99 eV	3.29 eV
K2O6S2_ICSD_36154	4.93 eV	4.31 eV
K2O6Si2Zn1_ICSD_79705	4.29 eV	3.81 eV
K2O7S2_ICSD_249741	5.21 eV	4.92 eV
K2O7U2_ICSD_202984	2.66 eV	2.75 eV
K2O7W2_ICSD_67284	2.31 eV	2.59 eV
K2O7Zn6_ICSD_1120	1.48 eV	1.71 eV
K2O8P2V1_ICSD_200773	2.43 eV	2.35 eV
K2O8Pb1S2_ICSD_27652	4.46 eV	3.52 eV
K2O8Rb1Tb1V2_ICSD_39369	3.40 eV	3.28 eV
K2O8S2_ICSD_16972	3.50 eV	4.31 eV
K2O8V3_ICSD_1925	1.72 eV	2.29 eV
K2O9Si3Sn1_ICSD_19027	3.47 eV	3.38 eV
K2O9Si3Ti1_ICSD_19025	3.68 eV	3.67 eV
K2O9Si3Ti1_ICSD_412920	3.83 eV	3.57 eV
K2O9Si3Zr1_ICSD_56898	4.81 eV	4.46 eV
K2O9Si4_ICSD_31201	4.64 eV	3.96 eV
K2P2Pd1S6_ICSD_165316	1.44 eV	1.81 eV
K2P2Pd1_ICSD_15584	1.25 eV	1.16 eV
K2P2S6_ICSD_33278	1.71 eV	1.94 eV
K2P2Se6_ICSD_173417	1.41 eV	1.48 eV
K2P2Si1_ICSD_36367	1.20 eV	1.19 eV
K2Pd1S2_ICSD_641296	1.47 eV	1.63 eV
K2Pd1Se2_ICSD_641298	1.02 eV	1.15 eV
K2Pd1Te2_ICSD_641302	0.79 eV	0.94 eV
K2Pd3S4_ICSD_41885	1.16 eV	1.09 eV
K2Pd3S6U1_ICSD_262563	1.34 eV	1.28 eV
K2Pt1S15_ICSD_12164	1.91 eV	1.66 eV
K2Pt1S2_ICSD_26258	1.60 eV	1.62 eV
K2Pt1Se2_ICSD_40430	1.28 eV	1.31 eV
K2Pt1Te2_ICSD_40432	0.94 eV	1.04 eV
K2Pt4S6_ICSD_40062	1.46 eV	1.49 eV
K2Pt4Se6_ICSD_69438	1.00 eV	1.11 eV
K2S1_ICSD_183837	2.32 eV	2.20 eV
K2S1_ICSD_412535	2.04 eV	2.05 eV

Supplementary Table 832. Five-fold cross validated predictions for the band gap energy (226/277).

system	calculated	predicted
K2S2_ICSD_43406	1.47 eV	1.56 eV
K2S3Te1_ICSD_280023	2.31 eV	2.02 eV
K2S3Ti1_ICSD_72377	1.53 eV	1.86 eV
K2S3_ICSD_1263	1.26 eV	1.63 eV
K2S5Sn2_ICSD_73007	1.19 eV	1.40 eV
K2S5_ICSD_641320	1.75 eV	1.82 eV
K2S7Sb4_ICSD_25329	1.55 eV	1.35 eV
K2Sb4Se8_ICSD_402886	1.10 eV	1.02 eV
K2Se1_ICSD_168448	2.10 eV	1.85 eV
K2Se2_ICSD_73172	0.71 eV	0.98 eV
K2Se3Te1_ICSD_63012	1.77 eV	1.38 eV
K2Se3_ICSD_1264	0.63 eV	1.00 eV
K2Se4W1_ICSD_59242	1.32 eV	1.42 eV
K2Se5Sn2_ICSD_72379	0.84 eV	1.11 eV
K2Se5_ICSD_72380	0.75 eV	1.08 eV
K2Se8Sn4_ICSD_72386	1.01 eV	0.88 eV
K2Sn1Te5_ICSD_36215	0.45 eV	0.59 eV
K2Te1_ICSD_182742	2.14 eV	1.47 eV
K2Te2Zn1_ICSD_420088	2.22 eV	1.65 eV
K2Te2_ICSD_96740	0.19 eV	0.60 eV
K2Te2_ICSD_96741	0.67 eV	0.96 eV
K2Te3U1_ICSD_89546	0.17 eV	0.92 eV
K2Te3Zr1_ICSD_410734	0.44 eV	0.86 eV
K2Te3_ICSD_2453	0.53 eV	0.78 eV
K3Li1Na2O6Te1_ICSD_65978	2.22 eV	2.59 eV
K3Mn1O4_ICSD_108935	1.87 eV	1.68 eV
K3N11P6_ICSD_50211	4.01 eV	4.12 eV
K3N1O3_ICSD_37204	0.20 eV	1.88 eV
K3N1O4_ICSD_36632	3.64 eV	2.69 eV
K3Na1O6Th2_ICSD_38325	3.55 eV	3.19 eV
K3Na1O8Ru2_ICSD_416039	1.14 eV	1.17 eV
K3Na1O8Ru2_ICSD_416040	1.24 eV	1.17 eV
K3Na1O8S2_ICSD_186839	5.27 eV	4.77 eV
K3Na1O8Se2_ICSD_54168	3.55 eV	3.53 eV
K3Nb1O8_ICSD_30405	3.02 eV	2.99 eV
K3Nd1O8P2_ICSD_8274	3.67 eV	4.06 eV
K3Nd1O8V2_ICSD_84234	3.58 eV	3.31 eV
K3Ni1O2_ICSD_262579	1.46 eV	1.44 eV
K3Ni1O2_ICSD_73216	1.48 eV	1.38 eV
K3Ni2O4_ICSD_14157	1.08 eV	1.03 eV
K3O14V5_ICSD_24068	2.25 eV	2.19 eV
K3O3S1V1_ICSD_419400	3.11 eV	2.42 eV
K3O3Sb1_ICSD_279579	3.24 eV	2.50 eV
K3O4Sb1_ICSD_280867	1.86 eV	2.30 eV
K3O4V1_ICSD_108936	4.05 eV	3.26 eV
K3O4V1_ICSD_4138	3.68 eV	3.34 eV
K3O7Sc1Si2_ICSD_413432	3.65 eV	3.91 eV
K3O8Ta1_ICSD_30406	3.00 eV	3.36 eV
K3O8V3_ICSD_100782	1.44 eV	2.58 eV
K3P1S4_ICSD_24599	2.77 eV	2.34 eV

Supplementary Table 833. Five-fold cross validated predictions for the band gap energy (227/277).

system	calculated	predicted
K3P1Se16_ICSD_280849	1.09 eV	1.21 eV
K3P1_ICSD_25550	0.22 eV	0.83 eV
K3P5Ru1Se10_ICSD_406200	0.98 eV	1.42 eV
K3S3Sb1_ICSD_641323	2.69 eV	2.32 eV
K3S4Sb1_ICSD_402642	2.16 eV	2.17 eV
K3S4Sb1_ICSD_41895	2.12 eV	1.96 eV
K3Sb1Se3_ICSD_89607	2.29 eV	1.98 eV
K3Sb1Se4_ICSD_65142	1.42 eV	1.56 eV
K3Sb1Te3_ICSD_300182	1.70 eV	1.37 eV
K3Sb1_ICSD_641351	0.68 eV	0.62 eV
K3Sb1_ICSD_656327	0.41 eV	0.59 eV
K4Mo8O52P12_ICSD_35463	2.06 eV	2.16 eV
K4N2O14S4_ICSD_16879	2.03 eV	3.73 eV
K4N6Ni1O12_ICSD_24529	1.96 eV	2.21 eV
K4Na2O6Te1_ICSD_202073	2.12 eV	2.21 eV
K4Na2O6Tl2_ICSD_74956	1.27 eV	1.47 eV
K4Ni3O6_ICSD_426450	0.53 eV	1.20 eV
K4O12W3Zn1_ICSD_200518	4.49 eV	3.31 eV
K4O12Zr5_ICSD_14024	3.18 eV	3.29 eV
K4O21Te2U5_ICSD_153175	2.73 eV	2.74 eV
K4O3Sb2_ICSD_280170	1.24 eV	1.82 eV
K4O4Pb1_ICSD_37268	1.68 eV	1.77 eV
K4O4Sn1_ICSD_158	2.17 eV	2.10 eV
K4O7P2_ICSD_187833	3.75 eV	3.59 eV
K4O7V2_ICSD_250388	3.43 eV	3.11 eV
K4O8P2_ICSD_418250	3.20 eV	3.89 eV
K4P2Pd1S8_ICSD_165312	1.92 eV	1.92 eV
K4P2Zn1_ICSD_67261	0.91 eV	0.90 eV
K4P6_ICSD_33259	0.70 eV	0.78 eV
K4P8Te4_ICSD_249937	1.19 eV	1.24 eV
K4S11Ta2_ICSD_410672	1.81 eV	1.94 eV
K4S14Ti3_ICSD_68154	1.63 eV	1.74 eV
K4Se6Sn2_ICSD_300234	1.44 eV	1.42 eV
K4Si4_ICSD_409852	1.27 eV	1.22 eV
K4Sn4_ICSD_409435	0.71 eV	0.65 eV
K5Te3_ICSD_66024	0.21 eV	1.23 eV
K6Mg1O4_ICSD_2340	1.68 eV	1.79 eV
K6Mn1S4_ICSD_65448	1.88 eV	1.97 eV
K6Mn1Se4_ICSD_65450	1.65 eV	1.62 eV
K6Mn1Te4_ICSD_65452	1.68 eV	1.54 eV
K6N4O3W2_ICSD_73549	0.47 eV	1.63 eV
K6O5Pb2_ICSD_74873	2.12 eV	1.78 eV
K6O9Se2_ICSD_240701	1.63 eV	2.69 eV
K6Se6Sn2_ICSD_410863	1.46 eV	1.70 eV
K6Si2Te6_ICSD_1238	1.71 eV	1.59 eV
K6Sn2Te6_ICSD_10109	1.08 eV	1.31 eV
K7Li1Si8_ICSD_61144	1.70 eV	1.36 eV
K8O24Sr2U6_ICSD_91784	2.93 eV	2.85 eV
K8Sb4Sn1_ICSD_44679	0.29 eV	0.52 eV
Kr1_ICSD_43726	7.18 eV	7.22 eV

Supplementary Table 834. Five-fold cross validated predictions for the band gap energy (228/277).

system	calculated	predicted
Kr1_ICSD_9785	7.18 eV	7.15 eV
La1Li1O12P4_ICSD_416877	5.97 eV	5.24 eV
La1Li1O4Ti1_ICSD_91843	2.96 eV	2.93 eV
La1Li3P2_ICSD_49627	0.30 eV	0.84 eV
La1Lu1O3_ICSD_51449	4.87 eV	4.21 eV
La1Mg1Na1O6Te1_ICSD_78532	2.47 eV	2.86 eV
La1Mg1Na1O6W1_ICSD_174386	3.19 eV	3.03 eV
La1Mg1Na1O6W1_ICSD_40497	2.85 eV	3.15 eV
La1Mn1O3_ICSD_54919	1.13 eV	0.93 eV
La1Mo2O8Rb1_ICSD_42700	3.83 eV	3.23 eV
La1N1O2Si1_ICSD_9752	0.83 eV	2.87 eV
La1N1_ICSD_162195	1.45 eV	0.97 eV
La1N2O1Ta1_ICSD_411138	0.95 eV	1.32 eV
La1N3O6_ICSD_281587	2.25 eV	2.65 eV
La1Na1O4Ti1_ICSD_82003	3.16 eV	3.09 eV
La1Na1O6Se2_ICSD_68467	3.94 eV	3.25 eV
La1Na1O8S2_ICSD_20354	5.66 eV	4.51 eV
La1Na1Se2_ICSD_641485	1.90 eV	1.69 eV
La1Nb1O4_ICSD_37139	4.25 eV	3.60 eV
La1Nb1O4_ICSD_73390	4.24 eV	3.97 eV
La1Nb1O6Te1_ICSD_413007	3.45 eV	3.12 eV
La1O1P1Zn1_ICSD_85777	0.87 eV	1.10 eV
La1O2Rb1_ICSD_27331	3.38 eV	3.18 eV
La1O3Ru1_ICSD_75569	0.70 eV	0.65 eV
La1O3Tl1_ICSD_200088	0.32 eV	1.55 eV
La1O3Tm1_ICSD_419665	2.88 eV	3.14 eV
La1O3V1_ICSD_86557	0.88 eV	1.29 eV
La1O3Y1_ICSD_89455	4.56 eV	4.04 eV
La1O3Yb1_ICSD_15093	0.61 eV	1.35 eV
La1O4P1_ICSD_184549	5.39 eV	4.02 eV
La1O4P1_ICSD_21066	1.38 eV	4.37 eV
La1O4P1_ICSD_31564	5.25 eV	4.57 eV
La1O4P1_ICSD_79747	2.95 eV	4.04 eV
La1O4P1_ICSD_92155	5.43 eV	5.10 eV
La1O4Ta1_ICSD_415429	3.69 eV	3.35 eV
La1O4Ta1_ICSD_97688	3.52 eV	3.43 eV
La1O4V1_ICSD_155240	0.99 eV	2.24 eV
La1O4V1_ICSD_400	3.28 eV	2.78 eV
La1O4V1_ICSD_411083	3.06 eV	2.66 eV
La1O7Rb1Ta2_ICSD_81870	2.52 eV	2.40 eV
La1O8Rb1W2_ICSD_27737	3.64 eV	3.56 eV
La1O9P3_ICSD_202640	5.47 eV	5.29 eV
La1O9Sb3_ICSD_55720	1.41 eV	2.13 eV
La1O9V3_ICSD_411084	2.59 eV	2.05 eV
La1P1S1_ICSD_641637	0.23 eV	0.48 eV
La1P2_ICSD_42015	0.34 eV	0.42 eV
La1P5_ICSD_357	0.14 eV	0.25 eV
La1P7_ICSD_41938	0.41 eV	0.46 eV
La1Rb1S2_ICSD_81394	2.40 eV	2.32 eV
La1Rb1S4Si1_ICSD_414545	2.69 eV	2.52 eV

Supplementary Table 835. Five-fold cross validated predictions for the band gap energy (229/277).

system	calculated	predicted
La1Rb1Se2_ICSD_281067	2.05 eV	1.94 eV
La1S2_ICSD_659874	0.47 eV	0.78 eV
La1Se1.9_ICSD_69730	0.46 eV	0.72 eV
La2Mn2O3Se2_ICSD_181387	1.80 eV	1.59 eV
La2Mo1O12Te3_ICSD_171245	3.55 eV	3.05 eV
La2Mo1O6_ICSD_25611	3.52 eV	2.80 eV
La2Ni1O4_ICSD_56319	1.14 eV	1.55 eV
La2Ni1O4_ICSD_98562	0.18 eV	0.94 eV
La2O12W3_ICSD_78180	3.79 eV	3.34 eV
La2O2S1_ICSD_154583	2.97 eV	2.48 eV
La2O2S2_ICSD_2455	1.10 eV	1.83 eV
La2O2S2_ICSD_68498	1.59 eV	1.72 eV
La2O2Se1_ICSD_25804	2.34 eV	2.02 eV
La2O2Te1_ICSD_89557	1.91 eV	1.69 eV
La2O3_ICSD_160205	3.77 eV	3.58 eV
La2O3_ICSD_160220	3.44 eV	3.15 eV
La2O3_ICSD_184531	3.53 eV	3.36 eV
La2O3_ICSD_26864	1.60 eV	2.64 eV
La2O4Se1Si1_ICSD_69552	2.73 eV	2.82 eV
La2O5Pd2_ICSD_65031	1.84 eV	1.62 eV
La2O5Si1_ICSD_109414	4.62 eV	3.67 eV
La2O6S1_ICSD_66823	5.22 eV	4.13 eV
La2O6Te1_ICSD_62134	2.93 eV	2.95 eV
La2O7Sn2_ICSD_153813	2.10 eV	2.25 eV
La2O7Ti2_ICSD_164027	3.27 eV	3.19 eV
La2O7Zr2_ICSD_154752	3.82 eV	3.40 eV
La2O9Si1Ti2_ICSD_75583	3.05 eV	3.08 eV
La2O9W2_ICSD_93721	3.00 eV	3.17 eV
La2S3_ICSD_15151	0.93 eV	0.85 eV
La2S5Si1_ICSD_240952	2.47 eV	2.35 eV
La2S5Zr1_ICSD_425078	1.10 eV	1.10 eV
La3Li5Nb2O12_ICSD_68251	3.54 eV	3.56 eV
La3Li5O12Ta2_ICSD_68252	4.06 eV	3.77 eV
La3N1S3_ICSD_416221	1.21 eV	1.18 eV
La3Nb1O7_ICSD_10058	3.07 eV	3.28 eV
La3O10Re2_ICSD_202001	0.31 eV	1.59 eV
La3O3Sb1_ICSD_380456	0.69 eV	1.05 eV
La3O7Os1_ICSD_59664	1.11 eV	0.80 eV
La3O7Ru1_ICSD_416718	1.17 eV	1.35 eV
La3O7Sb1_ICSD_246732	2.68 eV	2.71 eV
La3O7Ta1_ICSD_168909	3.65 eV	3.49 eV
La3O7Ta1_ICSD_55655	3.64 eV	3.58 eV
La3O8Re1_ICSD_38323	1.96 eV	1.54 eV
La3Rh2_ICSD_656947	0.10 eV	0.41 eV
La4Mn1O1S6_ICSD_391305	1.17 eV	1.58 eV
La4Mn1O1Se6_ICSD_391303	1.12 eV	1.37 eV
La4Mn1S7_ICSD_641433	2.29 eV	1.58 eV
La4N2S3_ICSD_415780	1.08 eV	1.14 eV
La4N2Te3_ICSD_414591	0.84 eV	1.04 eV
La4Ni1S7_ICSD_641558	1.49 eV	1.40 eV

Supplementary Table 836. Five-fold cross validated predictions for the band gap energy (230/277).

system	calculated	predicted
La4O10Re2_ICSD_81	1.21 eV	0.91 eV
La4O12Ti3_ICSD_91952	3.21 eV	3.14 eV
La4O4Se3_ICSD_419128	1.44 eV	1.41 eV
La4O7Pd1_ICSD_65032	1.10 eV	1.33 eV
La5N1O2S4_ICSD_420918	1.33 eV	1.58 eV
La6O12U1_ICSD_202585	2.67 eV	3.06 eV
La6O18Re4_ICSD_30535	0.50 eV	0.77 eV
La8Ni4O17_ICSD_73921	0.34 eV	0.99 eV
Li0.667Mn0.667Ni0.333O2_ICSD_90112	1.51 eV	1.45 eV
Li10O9Zn4_ICSD_23634	3.01 eV	2.98 eV
Li14O14Pb3Rb2_ICSD_35405	1.90 eV	1.90 eV
Li14O14Pr3Rb2_ICSD_203142	1.98 eV	2.54 eV
Li16N8Nb2O1_ICSD_174443	3.39 eV	3.09 eV
Li16N8O1Ta2_ICSD_71696	3.36 eV	3.28 eV
Li1Mg1N1_ICSD_167573	2.19 eV	2.28 eV
Li1Mg1O4P1_ICSD_201138	5.44 eV	5.09 eV
Li1Mg1O4V1_ICSD_63477	3.46 eV	2.96 eV
Li1Mg4O12V3_ICSD_250192	3.28 eV	2.97 eV
Li1Mn1O2_ICSD_154823	0.93 eV	1.06 eV
Li1Mn1O2_ICSD_40486	1.22 eV	1.09 eV
Li1Mn1O2_ICSD_98164	0.46 eV	0.86 eV
Li1Mn1O4P1_ICSD_97763	3.46 eV	3.13 eV
Li1Mn1O4V1_ICSD_247772	2.23 eV	1.58 eV
Li1Mn1O4_ICSD_73115	1.51 eV	2.02 eV
Li1Mn1O7P2_ICSD_415153	1.31 eV	2.62 eV
Li1Mo1O4Rb1_ICSD_20641	0.83 eV	3.06 eV
Li1Mo1O7P2_ICSD_68522	3.02 eV	2.61 eV
Li1Mo1S2_ICSD_150688	1.06 eV	0.97 eV
Li1Mo2O8Sb1_ICSD_80595	1.83 eV	2.14 eV
Li1Mo2O8Tl3_ICSD_84291	3.73 eV	2.92 eV
Li1Mo2O8Y1_ICSD_23313	3.23 eV	2.99 eV
Li1Mo3O8Sc1_ICSD_28525	1.52 eV	1.66 eV
Li1Mo3O8Y1_ICSD_28526	1.62 eV	1.60 eV
Li1N1O1Si1_ICSD_34106	5.26 eV	4.95 eV
Li1N1O3_ICSD_67981	2.98 eV	3.15 eV
Li1N1Sr1_ICSD_87414	0.26 eV	1.09 eV
Li1N1Zn1_ICSD_16790	1.30 eV	1.82 eV
Li1N2P1_ICSD_32713	3.81 eV	4.12 eV
Li1N2U1_ICSD_98663	1.50 eV	1.90 eV
Li1N3Si2_ICSD_89524	5.01 eV	4.33 eV
Li1N3_ICSD_155166	3.58 eV	3.07 eV
Li1N4Re1Sr2_ICSD_411454	1.88 eV	1.88 eV
Li1Na1O4S1_ICSD_14364	5.50 eV	5.23 eV
Li1Na1O6V2_ICSD_20836	3.07 eV	2.72 eV
Li1Na1Se1_ICSD_67359	2.47 eV	2.34 eV
Li1Na1Te1_ICSD_67274	2.30 eV	1.87 eV
Li1Nb1O2_ICSD_300243	1.66 eV	1.75 eV
Li1Nb1O3_ICSD_169693	2.68 eV	3.03 eV
Li1Nb1O3_ICSD_28298	3.42 eV	2.87 eV
Li1Nb1O4Zn1_ICSD_75526	3.25 eV	2.97 eV

Supplementary Table 837. Five-fold cross validated predictions for the band gap energy (231/277).

system	calculated	predicted
Li1Nb1O4Zn1_ICSD_75597	2.89 eV	3.17 eV
Li1Nb1O4Zn1_ICSD_85735	3.25 eV	2.97 eV
Li1Nb1O6Sr3_ICSD_380359	3.51 eV	3.17 eV
Li1Nb1O6W1_ICSD_202779	2.57 eV	2.80 eV
Li1Nb1S2_ICSD_26284	0.72 eV	0.85 eV
Li1Nb3O8_ICSD_2921	3.16 eV	2.94 eV
Li1Nd1O4Ti1_ICSD_91844	3.02 eV	2.67 eV
Li1Ni1O2_ICSD_164853	0.39 eV	1.56 eV
Li1Ni1O4P1_ICSD_184770	3.45 eV	3.06 eV
Li1Ni1O4P1_ICSD_97767	2.98 eV	2.95 eV
Li1Ni1O8Rb5Si2_ICSD_40269	2.99 eV	2.75 eV
Li1O12P3Sn2_ICSD_83832	2.96 eV	3.24 eV
Li1O12P3Zr2_ICSD_201935	4.14 eV	4.15 eV
Li1O12P4Tb1_ICSD_240703	5.94 eV	5.41 eV
Li1O12Te2V3_ICSD_249325	1.84 eV	2.07 eV
Li1O1Rb1_ICSD_65170	2.24 eV	2.53 eV
Li1O2Pr1_ICSD_56752	3.04 eV	2.36 eV
Li1O2Rh1_ICSD_29213	2.43 eV	2.17 eV
Li1O2Rh1_ICSD_59179	2.38 eV	1.63 eV
Li1O2Sc1_ICSD_31316	4.35 eV	4.03 eV
Li1O2Tb1_ICSD_21013	2.04 eV	2.93 eV
Li1O2Tl1_ICSD_33633	0.46 eV	1.29 eV
Li1O2V1_ICSD_202541	1.34 eV	1.49 eV
Li1O2Y1_ICSD_45511	4.11 eV	3.72 eV
Li1O3Rb1Zn2_ICSD_67145	2.59 eV	2.13 eV
Li1O3Sb1_ICSD_24051	2.14 eV	2.19 eV
Li1O3Ta1_ICSD_9537	3.75 eV	3.42 eV
Li1O3U1_ICSD_22310	3.17 eV	2.83 eV
Li1O3V1_ICSD_23477	2.77 eV	2.76 eV
Li1O3V1_ICSD_2899	3.03 eV	2.33 eV
Li1O4P1Pb1_ICSD_39206	3.53 eV	3.25 eV
Li1O4Rb1S1_ICSD_174059	5.08 eV	5.18 eV
Li1O4Rb2V1_ICSD_40218	3.93 eV	3.75 eV
Li1O4Ru2_ICSD_290491	0.23 eV	1.15 eV
Li1O4V2_ICSD_89352	0.15 eV	1.24 eV
Li1O5P1Ti1_ICSD_39534	3.36 eV	3.44 eV
Li1O5P1Ti1_ICSD_39761	3.23 eV	3.21 eV
Li1O5Si1Ta1_ICSD_39648	3.94 eV	3.75 eV
Li1O5V2_ICSD_23817	0.51 eV	0.71 eV
Li1O5V2_ICSD_27245	0.81 eV	1.06 eV
Li1O5V2_ICSD_50982	0.26 eV	0.67 eV
Li1O5V2_ICSD_88639	0.35 eV	0.56 eV
Li1O6Re1Sr2_ICSD_246730	1.97 eV	1.88 eV
Li1O6Re1Sr2_ICSD_418991	2.06 eV	2.11 eV
Li1O6Ru1Sr3_ICSD_50022	1.37 eV	1.51 eV
Li1O6Sb1W1_ICSD_202748	2.24 eV	2.91 eV
Li1O6Sc1Si2_ICSD_152075	5.24 eV	4.95 eV
Li1O6Sc1Si2_ICSD_152079	5.27 eV	4.77 eV
Li1O6Si2Ti1_ICSD_96292	2.96 eV	3.36 eV
Li1O6Si2V1_ICSD_55164	1.75 eV	2.31 eV

Supplementary Table 838. Five-fold cross validated predictions for the band gap energy (232/277).

system	calculated	predicted
Li1O6Sr3Ta1_ICSD_380360	3.95 eV	3.44 eV
Li1O7P2Sc1_ICSD_91496	5.17 eV	5.00 eV
Li1O7P2Ti1_ICSD_166164	3.14 eV	3.26 eV
Li1O7P2V1_ICSD_80551	1.78 eV	1.92 eV
Li1O8Pr1W2_ICSD_200520	3.45 eV	3.11 eV
Li1O8Ta3_ICSD_1318	3.49 eV	3.10 eV
Li1O8V3_ICSD_16689	1.75 eV	1.25 eV
Li1O9P3Pb1_ICSD_1123	4.72 eV	4.22 eV
Li1P1S4Zn1_ICSD_95785	2.70 eV	2.40 eV
Li1P1Sr1_ICSD_416889	1.35 eV	0.92 eV
Li1P1Sr1_ICSD_56443	0.80 eV	1.03 eV
Li1P1Zn1_ICSD_642242	1.37 eV	0.82 eV
Li1P1_ICSD_642222	0.80 eV	1.10 eV
Li1P5_ICSD_88710	1.25 eV	1.49 eV
Li1P7_ICSD_23621	1.65 eV	1.56 eV
Li1Rb2S4V1_ICSD_414185	1.77 eV	1.62 eV
Li1S2Sb1_ICSD_40457	1.20 eV	1.46 eV
Li1S2Sc1_ICSD_642305	1.88 eV	2.01 eV
Li1S2Ti1_ICSD_642322	1.41 eV	1.12 eV
Li1S2Y1_ICSD_44957	1.92 eV	1.89 eV
Li1Sb1Sr1_ICSD_172216	0.67 eV	0.63 eV
Li1Sb1Zn1_ICSD_642350	0.47 eV	0.41 eV
Li1Se2Tb1_ICSD_44962	1.51 eV	1.48 eV
Li1Se2Y1_ICSD_44963	1.52 eV	1.29 eV
Li1Te2Ti1_ICSD_44908	0.86 eV	0.74 eV
Li1Te3_ICSD_935	0.18 eV	0.60 eV
Li2Mg1Mn3O8_ICSD_90648	1.88 eV	1.92 eV
Li2Mg1Si1_ICSD_167141	0.17 eV	0.35 eV
Li2Mg1Si1_ICSD_181255	0.13 eV	0.40 eV
Li2Mn1O3_ICSD_21022	1.80 eV	1.61 eV
Li2Mn1O4Si1_ICSD_161305	3.07 eV	2.67 eV
Li2Mn3O8Zn1_ICSD_26995	1.57 eV	1.78 eV
Li2N1O2P1_ICSD_188494	5.60 eV	5.13 eV
Li2N2Na4_ICSD_92309	0.50 eV	1.72 eV
Li2N2Th1_ICSD_16232	1.89 eV	2.31 eV
Li2N2Zr1_ICSD_78790	1.58 eV	2.03 eV
Li2Na1Sb1_ICSD_57410	0.68 eV	0.82 eV
Li2Na6O10W2_ICSD_49027	3.50 eV	3.47 eV
Li2Nb2O7Sr1_ICSD_88463	1.92 eV	2.20 eV
Li2Nb2O7Sr1_ICSD_88467	1.95 eV	2.41 eV
Li2Ni1O2_ICSD_25000	2.31 eV	2.18 eV
Li2Ni1O2_ICSD_73874	3.26 eV	2.05 eV
Li2Ni1O3_ICSD_153094	1.31 eV	2.37 eV
Li2Ni1O8W2_ICSD_92853	2.87 eV	2.87 eV
Li2O13Ti6_ICSD_182966	3.30 eV	3.02 eV
Li2O1_ICSD_108886	4.90 eV	4.65 eV
Li2O1_ICSD_642219	4.90 eV	4.72 eV
Li2O20P4U3_ICSD_246191	3.42 eV	3.34 eV
Li2O2Pd1_ICSD_19007	1.95 eV	2.00 eV
Li2O2_ICSD_180557	1.98 eV	2.99 eV

Supplementary Table 839. Five-fold cross validated predictions for the band gap energy (233/277).

system	calculated	predicted
Li2O2_ICSD_50658	1.73 eV	2.34 eV
Li2O3Pb1_ICSD_35182	1.04 eV	1.68 eV
Li2O3Pr1_ICSD_154704	1.98 eV	2.33 eV
Li2O3Re1_ICSD_200999	0.71 eV	2.03 eV
Li2O3Ru1_ICSD_202611	0.79 eV	1.21 eV
Li2O3Ru1_ICSD_78721	0.82 eV	1.14 eV
Li2O3Si1_ICSD_853	5.08 eV	5.24 eV
Li2O3Sn1_ICSD_21053	2.67 eV	2.25 eV
Li2O3Te1_ICSD_38416	3.96 eV	3.07 eV
Li2O3Te1_ICSD_4317	3.61 eV	3.22 eV
Li2O3Ti1_ICSD_261239	3.48 eV	3.34 eV
Li2O3Zr1_ICSD_31941	3.74 eV	3.74 eV
Li2O3Zr1_ICSD_94896	3.74 eV	3.80 eV
Li2O4Rb2Si1_ICSD_61086	3.86 eV	3.76 eV
Li2O4S1_ICSD_153807	5.99 eV	5.65 eV
Li2O4S1_ICSD_30276	6.03 eV	5.71 eV
Li2O4Si1Sr1_ICSD_167334	4.66 eV	4.42 eV
Li2O4Te1_ICSD_1485	2.27 eV	2.86 eV
Li2O4U1_ICSD_200297	2.94 eV	2.92 eV
Li2O4U1_ICSD_20578	3.03 eV	2.97 eV
Li2O4W1_ICSD_10479	4.05 eV	4.15 eV
Li2O4W1_ICSD_14196	3.71 eV	3.82 eV
Li2O4W1_ICSD_15395	5.06 eV	4.11 eV
Li2O5Si1Ti1_ICSD_78059	3.77 eV	3.64 eV
Li2O5Si1V1_ICSD_86775	2.07 eV	2.46 eV
Li2O5Si2_ICSD_78562	5.16 eV	4.94 eV
Li2O6Sn1Te1_ICSD_40265	1.86 eV	2.35 eV
Li2O6Te1Zr1_ICSD_71489	2.75 eV	3.35 eV
Li2O7Sr1Ta2_ICSD_246277	2.53 eV	2.83 eV
Li2O7Sr1Ta2_ICSD_88464	2.42 eV	3.02 eV
Li2O7Ti3_ICSD_426263	3.43 eV	3.20 eV
Li2O7W2_ICSD_1897	3.55 eV	3.18 eV
Li2S1_ICSD_657597	3.39 eV	3.06 eV
Li2S1_ICSD_91283	3.56 eV	3.06 eV
Li2S1_ICSD_91284	3.90 eV	3.15 eV
Li2S3Te1_ICSD_415120	0.97 eV	1.83 eV
Li2Se1_ICSD_642355	2.98 eV	2.57 eV
Li2Se3Te1_ICSD_415121	0.53 eV	1.17 eV
Li2Te1_ICSD_642398	2.50 eV	2.04 eV
Li3N1_ICSD_156903	1.22 eV	0.97 eV
Li3N1_ICSD_660252	1.00 eV	1.88 eV
Li3N2Sc1_ICSD_98140	2.17 eV	1.99 eV
Li3N4Nb1Sr2_ICSD_84715	2.14 eV	2.12 eV
Li3N4Sr2Ta1_ICSD_84716	2.16 eV	2.42 eV
Li3Nb1O4_ICSD_30246	4.02 eV	3.66 eV
Li3Nd3O12W2_ICSD_245640	3.23 eV	3.41 eV
Li3Ni2O6Sb1_ICSD_183999	2.69 eV	2.15 eV
Li3O4P1_ICSD_10257	5.80 eV	5.62 eV
Li3O4P1_ICSD_20208	5.43 eV	5.31 eV
Li3O4Sb1_ICSD_82864	3.06 eV	2.89 eV

Supplementary Table 840. Five-fold cross validated predictions for the band gap energy (234/277).

system	calculated	predicted
Li3O4Ta1_ICSD_37126	4.68 eV	3.80 eV
Li3O4U1_ICSD_109090	3.08 eV	2.87 eV
Li3O4V1_ICSD_19002	4.01 eV	2.94 eV
Li3O6Sb1Zn2_ICSD_69189	3.01 eV	2.64 eV
Li3P1_ICSD_26880	0.70 eV	0.88 eV
Li3S3Sb1_ICSD_424834	2.36 eV	2.31 eV
Li3Sb1_ICSD_26879	0.48 eV	0.81 eV
Li3Sb1_ICSD_642341	0.69 eV	0.62 eV
Li4Mo1O5_ICSD_40270	2.71 eV	2.92 eV
Li4N2Na2_ICSD_92305	2.16 eV	2.01 eV
Li4N2Na2_ICSD_92306	2.17 eV	2.02 eV
Li4N3Ta1_ICSD_412585	2.70 eV	2.98 eV
Li4N6Si2Sr3_ICSD_421259	2.08 eV	2.06 eV
Li4O4Pb1_ICSD_38350	1.45 eV	2.18 eV
Li4O4Si1_ICSD_98615	4.78 eV	4.71 eV
Li4O4Ti1_ICSD_75164	4.73 eV	3.71 eV
Li4O5Se1_ICSD_92395	2.45 eV	3.61 eV
Li4O5Te1_ICSD_2403	2.72 eV	3.24 eV
Li4O5U1_ICSD_20452	3.12 eV	3.01 eV
Li4O5W1_ICSD_108819	3.57 eV	3.48 eV
Li4O7P2_ICSD_59243	5.62 eV	5.40 eV
Li4P2Sr1_ICSD_416888	0.97 eV	0.85 eV
Li5N2Na1_ICSD_92313	2.67 eV	1.47 eV
Li5N2Na1_ICSD_92314	0.38 eV	1.61 eV
Li5N4Re1_ICSD_92467	2.95 eV	2.41 eV
Li5O4Tl1_ICSD_16219	1.66 eV	2.18 eV
Li5O5Sb1_ICSD_203030	2.93 eV	2.87 eV
Li5O6Re1_ICSD_30590	2.31 eV	2.40 eV
Li5O6Re1_ICSD_38381	2.26 eV	2.24 eV
Li6Mn2N6Sr2_ICSD_412874	0.50 eV	1.35 eV
Li6Mo1N4_ICSD_66095	2.71 eV	2.45 eV
Li6N4W1_ICSD_153620	3.13 eV	2.79 eV
Li6O4Zn1_ICSD_62137	3.98 eV	3.61 eV
Li6O6Te1_ICSD_40247	2.95 eV	3.02 eV
Li6O6U1_ICSD_25350	3.16 eV	3.13 eV
Li6O6U1_ICSD_48209	3.18 eV	3.14 eV
Li6O7Si2_ICSD_25752	4.80 eV	4.90 eV
Li6O7Zr2_ICSD_41321	3.92 eV	3.74 eV
Li7Mn1N4_ICSD_154076	1.10 eV	2.24 eV
Li7N4Nb1_ICSD_71547	3.33 eV	3.13 eV
Li7N4P1_ICSD_642182	3.47 eV	2.89 eV
Li7N4Ta1_ICSD_67560	3.28 eV	3.19 eV
Li7N4V1_ICSD_409501	3.15 eV	2.93 eV
Li7N4V1_ICSD_44368	2.99 eV	2.95 eV
Li7N4V1_ICSD_96941	2.35 eV	2.75 eV
Li7O6Sb1_ICSD_15631	3.50 eV	3.14 eV
Li7O6Ta1_ICSD_74950	4.55 eV	4.16 eV
Li7O8Rb1Si2_ICSD_33864	4.72 eV	4.30 eV
Li7P3S11_ICSD_157654	2.59 eV	2.54 eV
Li8N2Se1_ICSD_247256	1.78 eV	2.04 eV

Supplementary Table 841. Five-fold cross validated predictions for the band gap energy (235/277).

system	calculated	predicted
Li8N2Te1_ICSD_247257	2.31 eV	1.80 eV
Li8O6Pb1_ICSD_47104	2.34 eV	2.40 eV
Li8O6Pt1_ICSD_61218	2.52 eV	2.25 eV
Li8O6Sn1_ICSD_15104	3.43 eV	3.02 eV
Li9N1S3_ICSD_240749	2.43 eV	2.59 eV
Lu1N1_ICSD_169211	0.20 eV	0.44 eV
Lu1O3Pr1_ICSD_50758	4.35 eV	4.21 eV
Lu1O3V1_ICSD_185830	0.51 eV	0.88 eV
Lu1O4P1_ICSD_162338	7.44 eV	7.46 eV
Lu1O4P1_ICSD_162340	6.00 eV	6.63 eV
Lu1O4P1_ICSD_162341	6.70 eV	7.09 eV
Lu1O4P1_ICSD_162346	7.83 eV	7.40 eV
Lu1O4P1_ICSD_201133	7.09 eV	6.79 eV
Lu1O4V1_ICSD_160959	2.78 eV	2.83 eV
Lu1O4V1_ICSD_162326	2.79 eV	2.88 eV
Lu1O4V1_ICSD_246685	3.36 eV	2.98 eV
Lu1O4V1_ICSD_419281	3.25 eV	3.04 eV
Lu1O4V1_ICSD_419285	2.86 eV	3.08 eV
Lu1O4V1_ICSD_419288	2.92 eV	3.07 eV
Lu1P1S4_ICSD_412857	1.70 eV	1.58 eV
Lu1Rb1S2_ICSD_81407	2.34 eV	2.05 eV
Lu1S2Tl1_ICSD_642581	1.31 eV	1.39 eV
Lu1Se2Tl1_ICSD_642602	1.14 eV	1.13 eV
Lu1Te2Tl1_ICSD_642625	0.47 eV	0.59 eV
Lu2Mg1S4_ICSD_37420	2.01 eV	1.75 eV
Lu2Mg1Se4_ICSD_44912	1.48 eV	1.32 eV
Lu2Mn1S4_ICSD_37421	1.48 eV	1.41 eV
Lu2Mn1Se4_ICSD_642434	1.03 eV	0.90 eV
Lu2Mn2O7_ICSD_56518	0.96 eV	1.26 eV
Lu2O13Te5_ICSD_413669	3.04 eV	3.10 eV
Lu2O2S1_ICSD_109336	3.30 eV	2.32 eV
Lu2O3_ICSD_160232	4.24 eV	4.00 eV
Lu2O3_ICSD_33659	4.01 eV	4.03 eV
Lu2O5Si1_ICSD_89624	4.86 eV	4.68 eV
Lu2O7Si2_ICSD_412249	5.33 eV	4.69 eV
Lu2O7Sn2_ICSD_82965	4.22 eV	2.93 eV
Lu2O7V2_ICSD_32688	0.88 eV	1.29 eV
Lu2S3_ICSD_26974	2.54 eV	1.81 eV
Lu2S4Sr1_ICSD_642578	1.85 eV	1.69 eV
Lu2S4Zn1_ICSD_35089	2.41 eV	2.20 eV
Lu6O12U1_ICSD_23967	2.81 eV	3.12 eV
Mg1Mn2O4_ICSD_16858	0.97 eV	1.40 eV
Mg1Mo1N2_ICSD_185913	0.71 eV	0.79 eV
Mg1Mo1O4_ICSD_20418	3.61 eV	3.11 eV
Mg1Mo1O6Sr2_ICSD_187662	2.21 eV	2.44 eV
Mg1Mo2O7_ICSD_1314	3.51 eV	3.08 eV
Mg1N2Si1_ICSD_186509	4.18 eV	3.65 eV
Mg1N2Si1_ICSD_23501	3.97 eV	3.74 eV
Mg1N2Si1_ICSD_44109	3.47 eV	3.52 eV
Mg1Na1Sb1_ICSD_41797	1.04 eV	0.73 eV

Supplementary Table 842. Five-fold cross validated predictions for the band gap energy (236/277).

system	calculated	predicted
Mg1Nb2O6_ICSD_85008	3.69 eV	3.13 eV
Mg1O1_ICSD_161842	3.38 eV	3.26 eV
Mg1O1_ICSD_162607	3.26 eV	3.30 eV
Mg1O1_ICSD_166273	3.01 eV	4.00 eV
Mg1O1_ICSD_181457	4.03 eV	3.33 eV
Mg1O1_ICSD_181459	3.87 eV	3.44 eV
Mg1O1_ICSD_181462	3.07 eV	3.06 eV
Mg1O1_ICSD_181463	3.01 eV	3.12 eV
Mg1O1_ICSD_181464	2.74 eV	3.04 eV
Mg1O1_ICSD_181465	2.89 eV	3.16 eV
Mg1O1_ICSD_181466	3.30 eV	3.29 eV
Mg1O1_ICSD_26958	4.47 eV	3.34 eV
Mg1O2_ICSD_41732	3.73 eV	4.18 eV
Mg1O3Se1_ICSD_494	4.01 eV	3.62 eV
Mg1O3Si1_ICSD_158959	4.64 eV	4.47 eV
Mg1O3Si1_ICSD_26489	4.61 eV	4.53 eV
Mg1O3Si1_ICSD_30893	4.62 eV	4.96 eV
Mg1O3Si1_ICSD_34074	4.68 eV	4.78 eV
Mg1O3Si1_ICSD_41659	5.41 eV	5.12 eV
Mg1O3Si1_ICSD_80669	4.51 eV	4.75 eV
Mg1O3Si1_ICSD_89805	5.79 eV	5.23 eV
Mg1O3Ti1_ICSD_169657	3.83 eV	3.36 eV
Mg1O3V1_ICSD_284	2.41 eV	2.73 eV
Mg1O4P1Rb1_ICSD_421738	4.11 eV	4.47 eV
Mg1O4P1Rb1_ICSD_421740	4.38 eV	4.54 eV
Mg1O4Rh2_ICSD_109299	2.19 eV	1.84 eV
Mg1O4S1_ICSD_16759	5.48 eV	5.06 eV
Mg1O4S1_ICSD_240893	5.54 eV	5.35 eV
Mg1O4Sb2_ICSD_4122	2.55 eV	2.59 eV
Mg1O4Se1_ICSD_109070	2.85 eV	3.33 eV
Mg1O4Ti2_ICSD_184695	1.32 eV	2.44 eV
Mg1O4W1_ICSD_67905	3.35 eV	3.17 eV
Mg1O5Se2_ICSD_402917	3.02 eV	3.18 eV
Mg1O5Te2_ICSD_4318	2.38 eV	2.90 eV
Mg1O5Ti2_ICSD_37232	3.22 eV	3.15 eV
Mg1O5V2_ICSD_50981	1.97 eV	1.97 eV
Mg1O6Pb2Te1_ICSD_86139	2.24 eV	2.10 eV
Mg1O6Pb2W1_ICSD_67880	2.59 eV	2.63 eV
Mg1O6Pt1Sr3_ICSD_281299	3.02 eV	2.29 eV
Mg1O6Pt3_ICSD_35340	0.65 eV	1.15 eV
Mg1O6Sb2_ICSD_40345	1.04 eV	1.89 eV
Mg1O6Sr2W1_ICSD_152576	2.92 eV	3.08 eV
Mg1O6Sr2W1_ICSD_155308	3.07 eV	3.04 eV
Mg1O6Ta2_ICSD_150419	3.06 eV	3.32 eV
Mg1O6V2_ICSD_10391	2.62 eV	2.17 eV
Mg1O7Si2Sr2_ICSD_183978	4.49 eV	4.48 eV
Mg1P1S3_ICSD_642729	2.81 eV	2.44 eV
Mg1P2Si1_ICSD_22189	1.35 eV	1.20 eV
Mg1P4_ICSD_113	0.68 eV	1.23 eV
Mg1S1_ICSD_159401	3.39 eV	2.92 eV

Supplementary Table 843. Five-fold cross validated predictions for the band gap energy (237/277).

system	calculated	predicted
Mg1S1_ICSD_44930	2.77 eV	3.04 eV
Mg1S4Sc2_ICSD_37423	1.90 eV	1.90 eV
Mg1Sc2Se4_ICSD_642814	1.42 eV	1.46 eV
Mg1Se17U8_ICSD_601593	0.73 eV	0.86 eV
Mg1Se1_ICSD_159398	2.55 eV	2.09 eV
Mg1Se1_ICSD_53946	1.77 eV	2.16 eV
Mg1Se1_ICSD_658986	2.57 eV	2.18 eV
Mg1Se2_ICSD_642815	1.64 eV	1.57 eV
Mg1Se4Y2_ICSD_76052	1.30 eV	1.28 eV
Mg1Te1_ICSD_168346	0.44 eV	1.40 eV
Mg1Te1_ICSD_168347	2.32 eV	1.47 eV
Mg1Te1_ICSD_168349	0.83 eV	1.39 eV
Mg1Te1_ICSD_642882	2.36 eV	1.80 eV
Mg1Te2_ICSD_41733	1.11 eV	0.87 eV
Mg2Mn1O8P2_ICSD_100784	3.44 eV	3.35 eV
Mg2Mo3O12Tl2_ICSD_250400	3.84 eV	3.33 eV
Mg2Mo3O8_ICSD_248080	2.05 eV	2.03 eV
Mg2N1Na2O9P3_ICSD_186877	5.08 eV	5.00 eV
Mg2N2Sr1_ICSD_410826	1.69 eV	1.59 eV
Mg2N3P1_ICSD_27229	3.53 eV	3.66 eV
Mg2O12P4_ICSD_4280	5.53 eV	5.31 eV
Mg2O4Si1_ICSD_171065	4.49 eV	4.63 eV
Mg2O4Si1_ICSD_27533	4.41 eV	4.35 eV
Mg2O4Si1_ICSD_27534	4.59 eV	4.32 eV
Mg2O4Si1_ICSD_27535	4.15 eV	4.55 eV
Mg2O4Si1_ICSD_27536	4.39 eV	4.35 eV
Mg2O4Si1_ICSD_27537	3.55 eV	4.38 eV
Mg2O4Si1_ICSD_83783	4.76 eV	4.55 eV
Mg2O4Si1_ICSD_83792	4.65 eV	4.42 eV
Mg2O4Sn1_ICSD_187038	1.52 eV	2.60 eV
Mg2O7P2_ICSD_22328	5.32 eV	5.05 eV
Mg2O7V2_ICSD_2321	2.68 eV	2.73 eV
Mg2O8Te3_ICSD_424305	3.46 eV	2.69 eV
Mg2P2Se6_ICSD_642731	2.03 eV	1.62 eV
Mg2S4Si1_ICSD_642791	2.97 eV	2.88 eV
Mg2S4Sn1_ICSD_642793	1.85 eV	1.97 eV
Mg2Sb2Sr1_ICSD_100046	0.98 eV	0.82 eV
Mg2Sb2Yb1_ICSD_412655	0.65 eV	0.61 eV
[truncated: 1,328,126 more chars]
